# Characterizing the Google Books Corpus: Strong Limits to Inferences of Socio-Cultural and Linguistic Evolution

**DOI:** 10.1371/journal.pone.0137041

**Published:** 2015-10-07

**Authors:** Eitan Adam Pechenick, Christopher M. Danforth, Peter Sheridan Dodds

**Affiliations:** 1 Department of Mathematics and Statistics, University of Vermont, Burlington, Vermont, United States of America; 2 Center for Complex Systems, University of Vermont, Burlington, Vermont, United States of America; 3 Computational Story Lab, University of Vermont, Burlington, Vermont, United States of America; 4 Vermont Advanced Computing Core, University of Vermont, Burlington, Vermont, United States of America; Centre de Physique Théorique, FRANCE

## Abstract

It is tempting to treat frequency trends from the Google Books data sets as indicators of the “true” popularity of various words and phrases. Doing so allows us to draw quantitatively strong conclusions about the evolution of cultural perception of a given topic, such as time or gender. However, the Google Books corpus suffers from a number of limitations which make it an obscure mask of cultural popularity. A primary issue is that the corpus is in effect a library, containing one of each book. A single, prolific author is thereby able to noticeably insert new phrases into the Google Books lexicon, whether the author is widely read or not. With this understood, the Google Books corpus remains an important data set to be considered more lexicon-like than text-like. Here, we show that a distinct problematic feature arises from the inclusion of scientific texts, which have become an increasingly substantive portion of the corpus throughout the 1900s. The result is a surge of phrases typical to academic articles but less common in general, such as references to time in the form of citations. We use information theoretic methods to highlight these dynamics by examining and comparing major contributions via a divergence measure of English data sets between decades in the period 1800–2000. We find that only the English Fiction data set from the second version of the corpus is not heavily affected by professional texts. Overall, our findings call into question the vast majority of existing claims drawn from the Google Books corpus, and point to the need to fully characterize the dynamics of the corpus before using these data sets to draw broad conclusions about cultural and linguistic evolution.

## Introduction

The Google Books data set is captivating both for its availability and its incredible size. The first version of the data set, published in 2009, incorporates over 5 million books [1]. These are, in turn, a subset selected for quality of optical character recognition and metadata—e.g., dates of publication—from 15 million digitized books, largely provided by university libraries. These 5 million books contain over half a trillion words, 361 billion of which are in English. Along with separate data sets for American English, British English, and English Fiction; the first version also includes Spanish, French, German, Russian, Chinese, and Hebrew data sets. The second version, published in 2012 [2], contains 8 million books with half a trillion words in English alone, and also includes books in Italian. The contents of the sampled books are split into case-sensitive *n*-grams which are typically blocks of text separated into *n* = 1, …, 5 pieces by whitespace—e.g., “I” is a 1-gram, and “I am” is a 2-gram

A central if subtle and deceptive feature of the Google Books corpus, and for others composed in a similar fashion, is that the corpus is a reflection of a library in which only one of each book is available. Ideally, we would be able to apply different popularity filters to the corpus. For example, we could ask to have *n*-gram frequencies adjusted according to book sales in the UK, library usage data in the US, or how often each page in each book is read on Amazon’s Kindle service (all over defined periods of time). Evidently, incorporating popularity in any useful fashion would be an extremely difficult undertaking on the part of Google.

We are left with the fact that the Google Books library has ultimately been furnished by the efforts and choices of authors, editors, and publishing houses, who collectively aim to anticipate or dictate what people will read. This adds a further distancing from “true culture” as the ability to predict cultural success is often rendered fundamentally impossible due to social influence processes [3]—we have one seed for each tree but no view of the real forest that will emerge.

We therefore observe that the Google Books corpus encodes only a small-scale kind of popularity: how often *n*-grams appear in a library with all books given (in principle) equal importance and tied to their year of publication (new editions and reprints allow some books to appear more than once). The corpus is thus more akin to a lexicon for a collection of texts, rather than the collection itself. But problematically, because Google Books *n*-grams do have frequency of usage associated with them based on this small-scale popularity, the data set readily conveys an illusion of large-scale cultural popularity. An *n*-gram which declines in usage frequency over time may in fact become more often read by a particular demographic focused on a specific genre of books. For example, “Frodo” first appears in the second Google Books English Fiction corpus in the mid 1950s and declines thereafter in popularity with a few resurgent spikes [4].

While this limitation to small-scale popularity tempers the kinds of conclusions we can draw, the evolution of *n*-grams within the Google Books corpus—their relative abundance, their growth and decay—still gives us a valuable lens into how language use and culture has changed over time. Our contribution here will be to show:

1. A principled approach for exploring word and phrase evolution;
2. How the Google Books corpus is challenged in other respects orthogonal to the its library-like nature, particularly by the inclusion of scientific and medical journals; and
3. How future analyses of the Google Books corpus should be considered.

**Fig 1:**
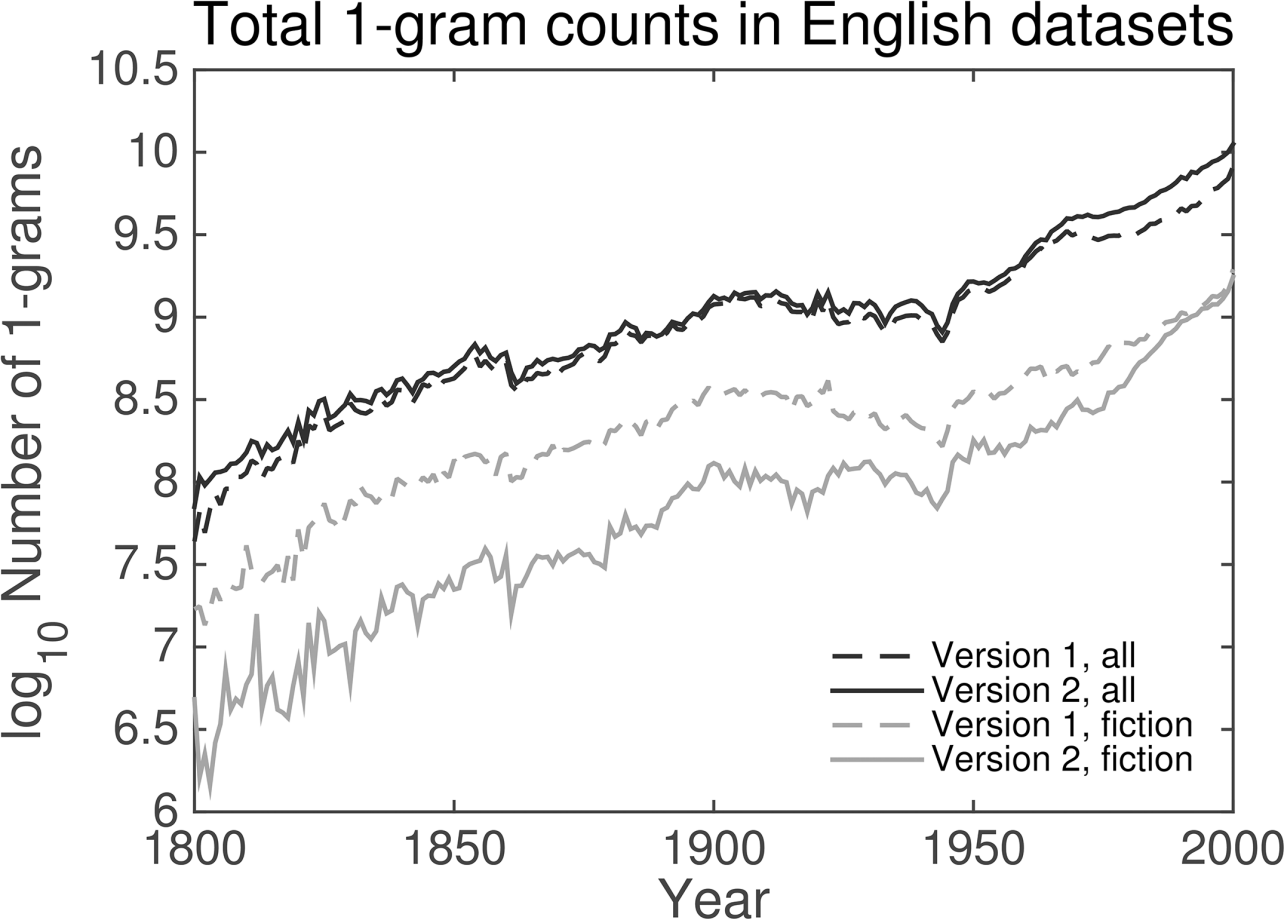
The logarithms of the total 1-gram counts for the Google Books English data sets (dark gray) and English Fiction data sets (light gray). The dashed and solid curves denote the 2009 and 2012 versions of the data sets. In all four examples, an exponential increase in volume is apparent over time with notable exceptions during wartime when the total volume decreases, clearest during the American Civil War and both World Wars. While the total volume for English increases between versions, the volume for English fiction decreases drastically, suggesting a more rigorous filtering process.

For ease of comparison with related work, we focus primarily on 1-grams from selected English data sets between the years 1800 and 2000. In this work, we will use the terms “word” and “1-gram” interchangeably for the sake of convenience. The total volume of (non-unique) English 1-grams grows exponentially between these years, as shown in Fig 1, except during major conflicts—e.g., the American Civil War and both World Wars—when the total volume dips substantially. We also observe a slight increase in volume between the first and second version of the unfiltered English data set. Between the two English Fiction data sets, however, the total volume actually decreases considerably, which indicates insufficient filtering was used in producing the first version, and immediately suggests the initial English Fiction data set may not be appropriate for any kind of analysis.

The simplest possible analysis involving any Google Books data set is to track the relative frequencies of a specific set of words or phrases. Examples of such analyses involve words or phrases surrounding individuality [5], gender [6], urbanization [7], and time [1, 8], all of which are of profound interest. However, the strength of all conclusions drawn from these must take into account both the number of words and phrases in question (anywhere from two [7] to twenty [5] or more at a time) and the sampling methods used to build the Google Books corpus.

Many researchers have carried out broad analyses of the Google Books corpus, examining properties and dynamics of entire languages. These include analyses of Zipf’s and Heaps’ laws as applied to the corpus [9], the rates of verb regularization [1], rates of word introduction and obsolescence and durations of cultural memory [8], as well as an observed decrease in the need for new words in several languages [10]. However, these studies also appear to take for granted that the data sets sample in a consistent manner from works spanning the last two centuries.

Analysis of the emotional content of books suggests a lag of roughly a decade between exogenous events and their effects in literature [11], complicating the use of the Google Books data sets directly as snapshots of cultural identity.

**Fig 2:**
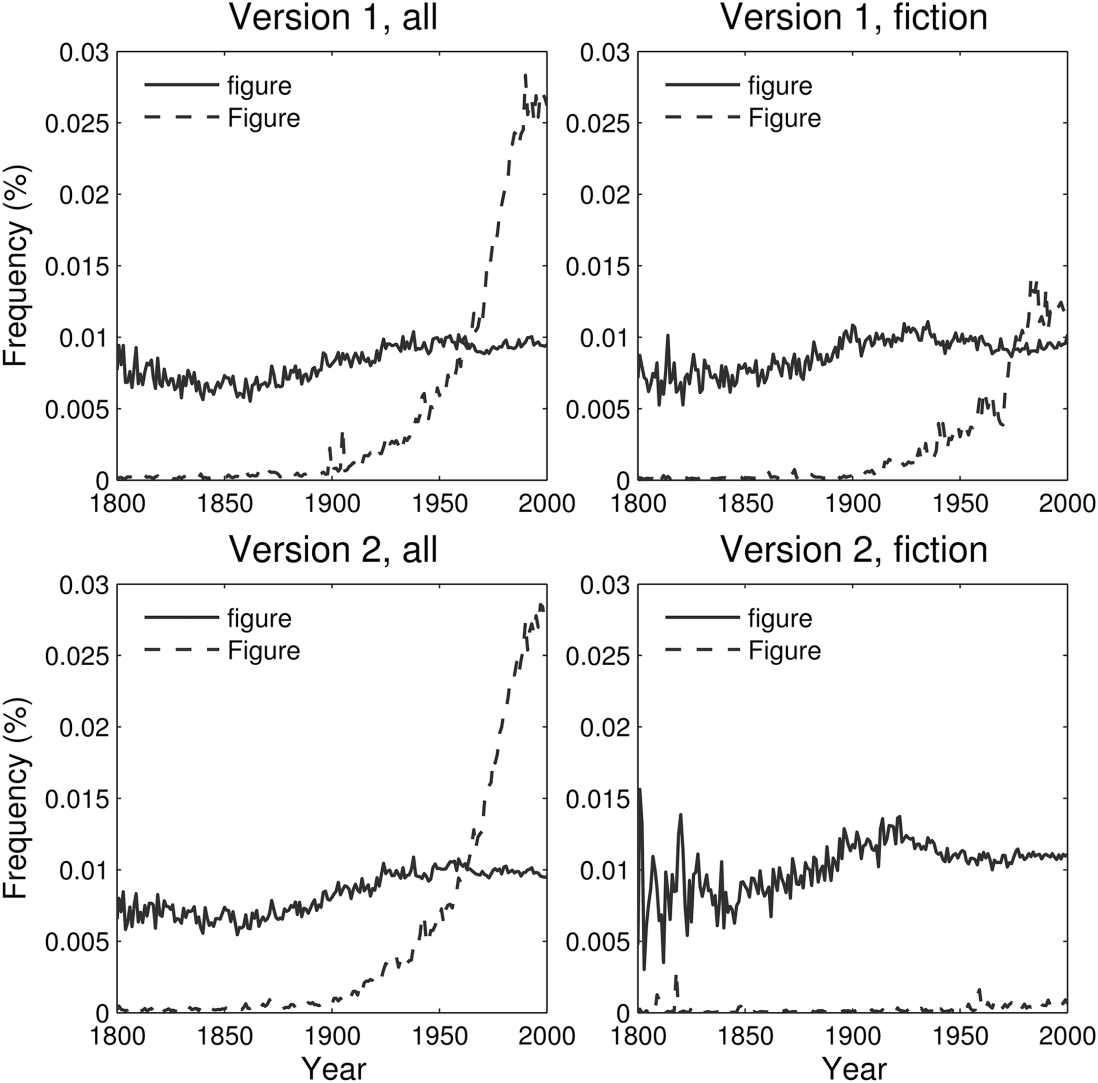
Relative frequencies of “Figure” vs “figure” in both versions of the Google Books corpus for both English (all) and English Fiction. In the English data sets, the capitalized term rapidly surpasses the uncapitalized term in the 1960s. For the first English Fiction data set, this effect is delayed until the 1970s. As shown later, only the second version of the English Fiction data set demonstrates a filtering of scientific terminology. These trends strongly suggest an increase starting around 1900 in the sampling of scientific texts in both English data sets and the first English Fiction data set.

As we will demonstrate, an assumption of unbiased sampling of books is not reasonable during the last century and especially during recent decades, which is of particular importance to all analyses concerned with recent social change. Since parsing in the data sets is case-sensitive, we can give a suggestive illustration of this observation in Fig 2, which displays the relative (normalized) frequencies of “figure” versus “Figure” in both versions of the corpus and for both English and English Fiction. In both versions of the English data set, the capitalized version, “Figure,” surpasses its lowercase counterpart during the 1960s. Since the majority of books in the corpus originated in university libraries [1], a major effect of scientific texts on the dynamics of the data set is quite plausible. This trend is also apparent—albeit delayed—in the first version of the English Fiction data set, which again suggests insufficient filtering during the compilation process for that version.

Because of Google Books library-like nature, authors are not represented equally or by any measure of popularity in any given data set but are instead roughly by their own prolificacy. This leaves room for individual authors to have noteworthy effects on the dynamics of the data sets, as we will demonstrate in Section Results and Discussion.

Lastly, due to copyright laws, the public data sets do not include metadata (see supporting online material [1]), and the data are truncated to avoid inference of authorship, which severely limits any analysis of censorship [1, 12] in the corpus. Under these conditions, we will show that much caution must be used when employing these data sets—with a possible exception of the second version of English Fiction—to draw cultural conclusions from the frequencies of words or phrases in the corpus.

We structure the remainder of the paper as follows. In Sec. Methods, we describe how to use Jensen-Shannon divergence to highlight the dynamics over time of both versions of the English and English Fiction data sets, paying particular attention to key contributing words. In Sec. Results and Discussion, we display and discuss examples of these highlights, exploring the extent of the scientific literature bias and issues with individual authors; we also provide a detailed inspection of some example decade–decade comparisons. We offer concluding remarks in Section Concluding Remarks.

## Methods

### Statistical divergence between years

We examine the dynamics of the Google Books corpus by calculating the statistical divergence between the distributions of 1-grams in two given years. A commonly used measure of statistical divergence is Kullback-Leibler (KL) divergence [13], based on which we use a bounded, symmetric measure. Given a language with *N* unique words and 1-gram distributions *P* in the first year and *Q* in second, the KL divergence between *P* and *Q* can be expressed as

$$D_{KL}\left(P\;\|\;Q\right)=\sum_{i=1}^{N}p_{i}\log_{2}\frac{p_{i}}{q_{i}},$$ (1)

where *p*_*i*_ is the probability of observing the *i* ^th^ 1-gram random chosen from the 1-gram distribution for first year, and *q*_*i*_ is the probability of observing the same word in the second year. The units of KL divergence is bits, and may be interpreted as the average number of bits wasted if a text from the first year is encoded efficiently, but according to the distribution from the latter, incorrect year. To demonstrate this, we may rewrite the previous equation as

$$D_{KL}\left(P\;\|\;Q\right)=-\sum_{i=1}^{N}q_{i}\log_{2}p_{i}-H\left(P\right),$$ (2)

where *H*(*P*) = −∑_*i*_ *p*_*i*_log_2_ *p*_*i*_ is the Shannon entropy [14], also the average number of bits required per word in an efficient encoding for the original distribution; and the remaining term is the average number of bits required per word in an efficient, but mistaken, encoding of a given text. However, if a single (say, the *j* ^th^) 1-gram in the language exists in the first year, but not in the second, then *q*_*j*_ = 0, and the divergence diverges. Since this scenario is not extraordinary for the data sets in question, we instead use Jensen-Shannon divergence (JSD) [15] given by

$$D_{\text{JS}}\left(P\;\|\;Q\right)=\frac{1}{2}(D_{KL}\left(P\|M\right)+D_{KL}\left(Q\|M\right)),$$ (3)

where $M=\frac{1}{2}\left(P+Q\right)$ is a mixed distribution of the two years. This measure of divergence is bounded between 0 when the distributions are the same and 1 bit in the extreme case when there is no overlap between the 1-grams in the two distributions. If we begin with a uniform distribution of *N* species and replace *k* of those species with *k* entirely new ones, the JSD between the original and new distribution is *k*/*N*, the proportion of species replaced. The JSD is also symmetric, which is an added convenience. The JSD may be expressed as

$$D_{\text{JS}}\left(P\;\|\;Q\right)=H\left(M\right)-\frac{1}{2}(H(P)+H(Q)),$$ (4)

from which it is apparent that a similar waste analogy holds as with KL divergence, with the mixed distribution taking the place of the approximation regardless of the year a text was written.

### Key contributions of individual words

The form for Jensen-Shannon divergence given in Eq 4 can be broken down into contributions from individual words, where the contribution from the *i* ^th^ word to the divergence between two years is given by

$$D_{\text{JS},i}\left(P\;\|\;Q\right)=-m_{i}\log_{2}m_{i}+\frac{1}{2}(p_{i}\log_{2}p_{i}+q_{i}\log_{2}q_{i}).$$ (5)

Some rearrangement gives

$$D_{\text{JS},i}\left(P\;\|\;Q\right)=m_{i}\cdot \frac{1}{2}(r_{i}\log_{2}r_{i}+\left(2-r_{i}\right)\log_{2}\left(2-r_{i}\right)),$$ (6)

where *r*_*i*_ = *p*_*i*_/*m*_*i*_, so that contribution from an individual word is proportional to the average probability of the word, and the proportion depends on the ratio between the smaller probability (without loss of generality) and the average. Namely, we may reframe the equation above as

$$D_{\text{JS},i}\left(P\;\|\;Q\right)=m_{i}C\left(r_{i}\right).$$ (7)

**Fig 3:**
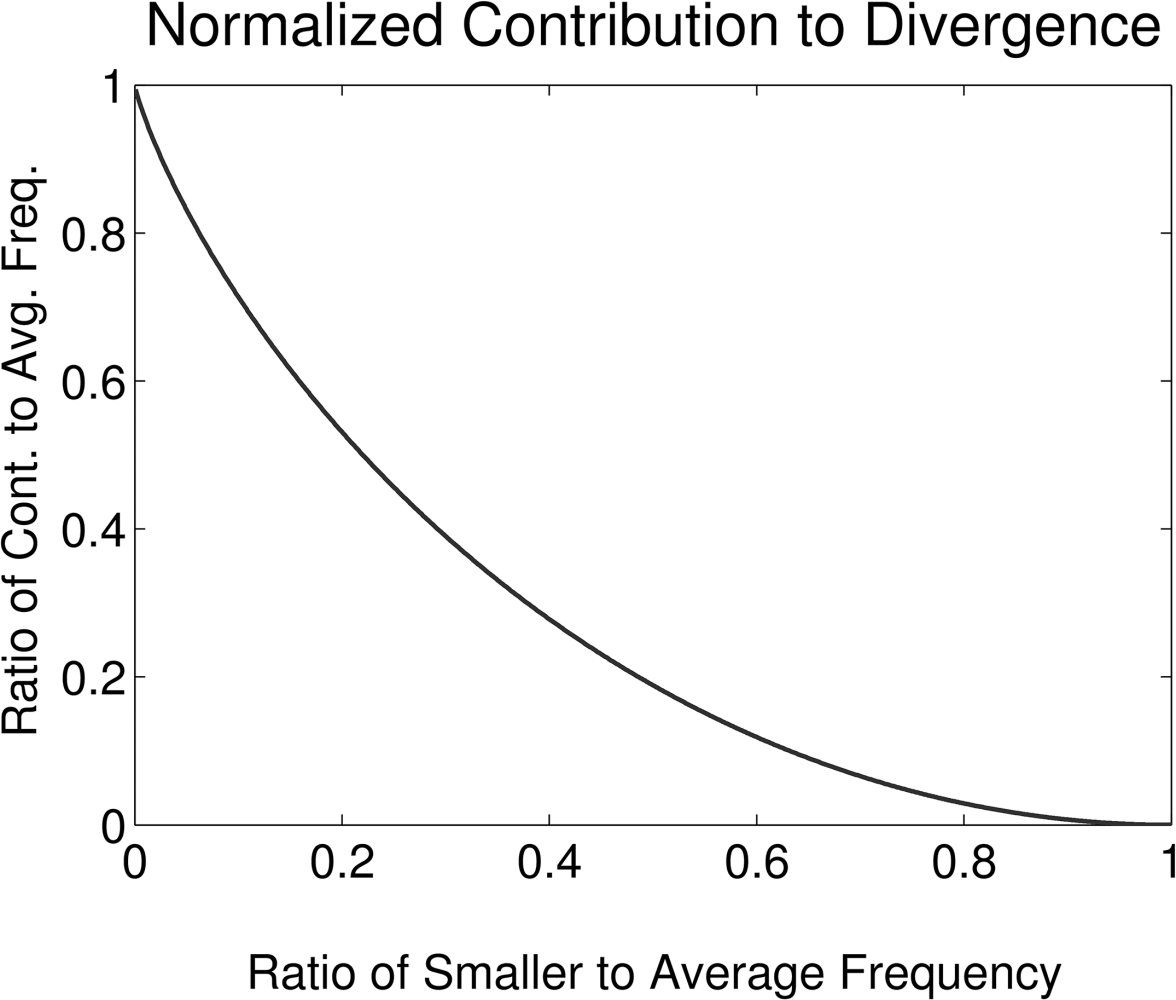
For the ratio *r* between the smaller relative probability of an element and the average, *C*(*r*) is the proportion of the average contributed to the Jensen-Shannon divergence (see Eqs 6 and 7). In particular, if *r* = 1 (no change), then the contribution is zero; if *r* = 0, the contribution is half its probability in the distribution in which it occurs with nonzero probability.

Words with larger average probability yield greater contributions as do those with smaller ratios, *r*, between the smaller and average probability. So while a common 1-gram—such as “the,” “if,” or a period—changing subtly can have a large effect on the divergence, so can an uncommon (or entirely new) word given a sufficient shift from one year to the next. The size of the contribution relative to the average probability is displayed in Fig 3 for ratios ranging from 0 to 1. *C*(*r*_*i*_) is symmetric about *r*_*i*_ = 1 (i.e., no change), so no novel behavior is lost by omitting the case where *r*_*i*_ > 1 (i.e., when *p*_*i*_ is the larger probability). The maximum possible contribution (in bits) is precisely the average probability of the word in question, which occurs if and only if the smaller probability is 0. No contribution is made if and only if the probability remains unchanged.

We coarse-grain the data at the level of decades—e.g., between 1800-to-1809 and 1990-to-1999—by averaging the relative normalized frequency of each unique word in a given decade over all years in that decade. (Each year is weighted equally.) This allows convenient calculation and sorting of contributions to divergence of individual 1-grams between any two time periods.

## Results and Discussion

### Broad view of language evolution within Google Books

**Fig 4:**
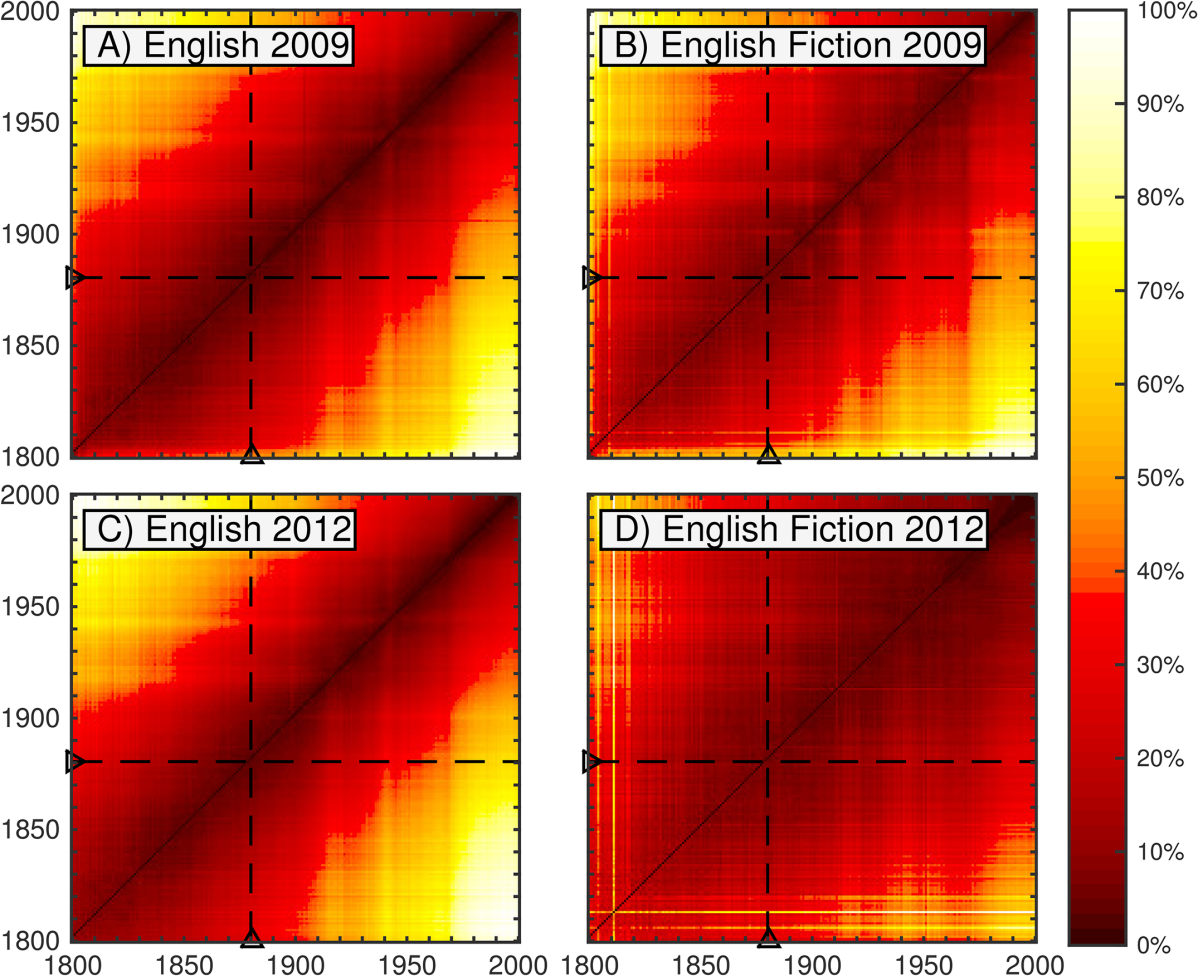
Heatmaps showing the JSD between every pair of years between 1800 and 2000, contributed by words appearing above a normalized frequency threshold of 10^−5^. The dashed lines highlight the divergences to and from the year 1880, which are featured in Fig 5. The off-diagonal elements represent divergences between consecutive years, as in Fig 6. The color represents the percentage of the maximum divergence observed in the given time range for each data set. The divergence between a year and itself is zero. For any given year, the divergence increases with the distance (number of years) from the diagonal—sharply at first, then gradually. Interesting features of the maps are the presence of two cross-hairs in the first half of the 20th century, which strongly suggests a wartime shift in the language, as well as an asymmetry that suggests a particularly high divergence between the first half century and the last quarter century observed.

Fig 4 shows the JSD between the 1-gram distributions for every pair of years between 1800 and 2000 contributed by 1-grams present above a threshold normalized frequency of 10^−5^ for both versions of the English and English Fiction data sets (i.e., words that appear with normalized frequency at least 1 in 10^5^).

**Fig 5:**
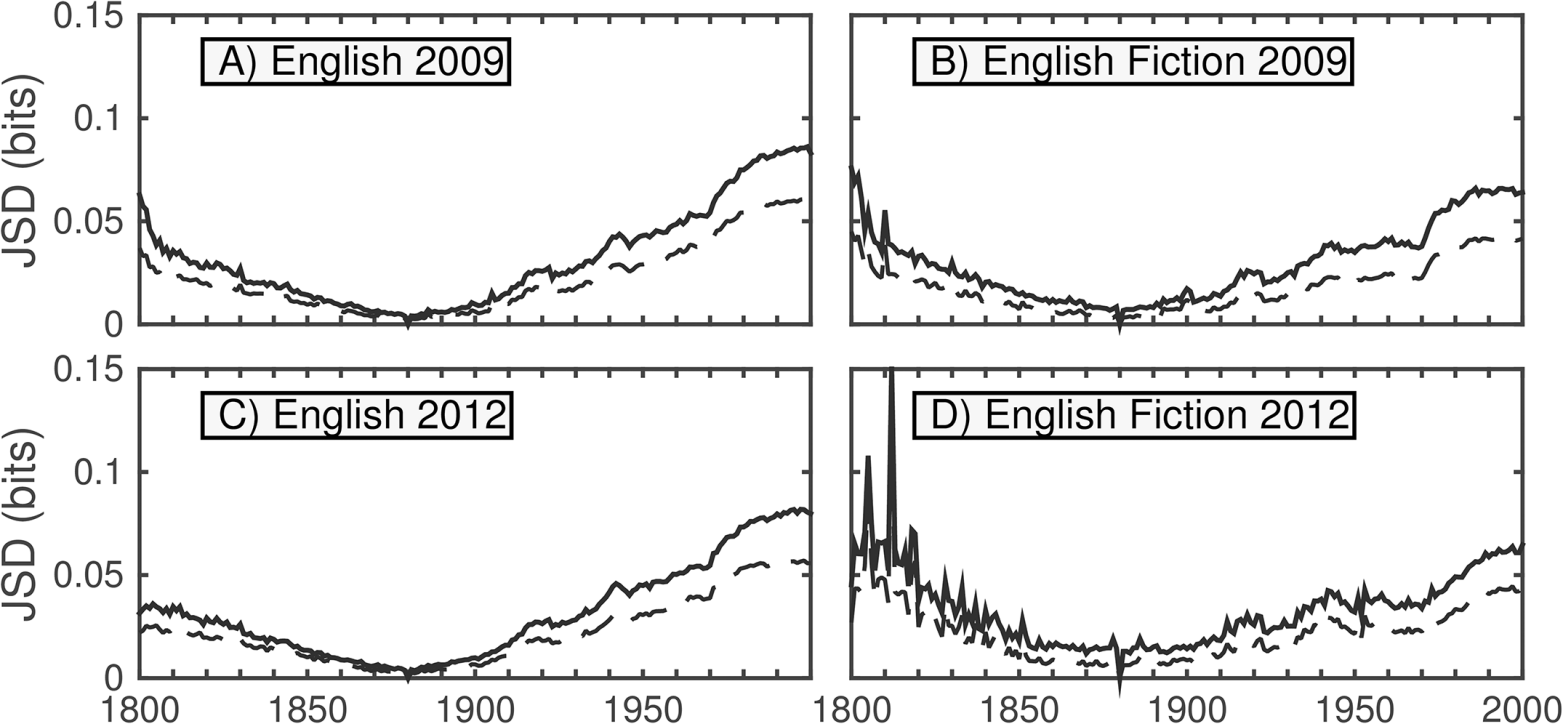
JSD between 1880 and each displayed year for given data set, corresponding to dashed lines from Fig 4. Contributions are counted for all words appearing above a 10^−5^ threshold in a given year; for the dashed curves, the threshold is 10^−4^. Typical behavior in each case consists of a relatively large jump between one year and the next with a more gradual rise afterward (in both directions). Exceptions include wartime, particularly the two World Wars, during which the divergence is greater than usual; however, after the conclusion of these periods, the cumulative divergence settles back to the previous trend. Initial spikiness in (D) is likely due to low volume.

**Fig 6:**
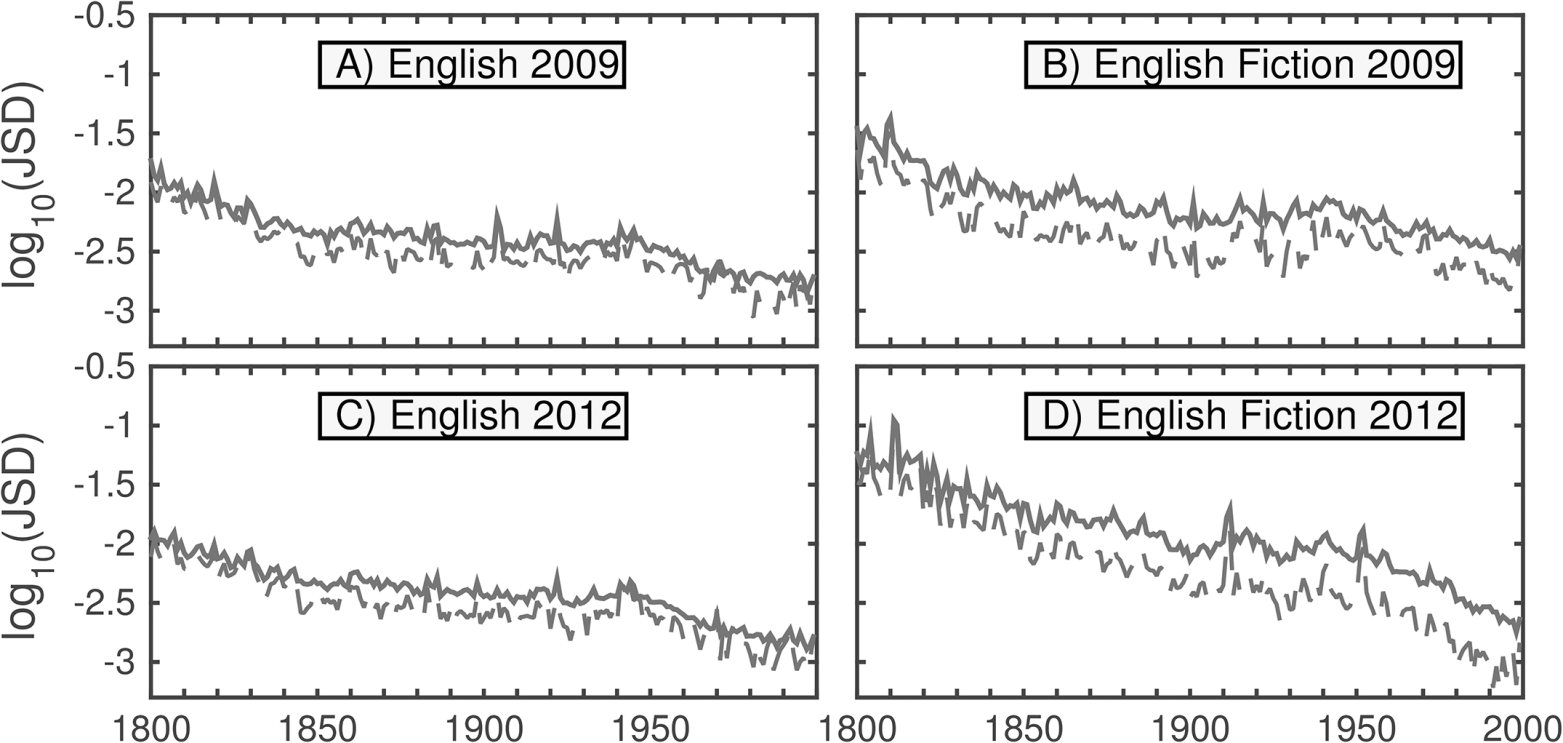
Consecutive year (between each year and the following year) base-10 logarithms of JSD, corresponding to off-diagonals in Fig 4. For the solid curves, contributions are counted for all words appearing above a 10^−5^ threshold in a given year; for the dashed curves, the threshold is 10^−4^. Divergences between consecutive years typically decline through the mid-19th century, remain relatively steady until the mid-20th century, then continue to decline gradually over time.

A major qualitative aspect apparent from the heatmaps is a gradual increase in divergence with differences in time—the lexicon underlying Google steadily evolves—though this is strongly curtailed for the second English Fiction corpus. We see the heatmaps are “pinched” toward the diagonal in the vicinities of the two world wars. Also visible is an asymmetry that suggests a particularly high divergence between the first half century and the last quarter century observed. We examine these effects more closely in Figs 5 and 6 by taking two slices of the heatmaps. We specifically consider the divergences of each year compared with 1880 (dashed lines), and the divergences between consecutive years (off-diagonal). To verify qualitative consistency, we also include analogous contribution curves using the more restrictive threshold of 10^−4^.

While the initial divergence between any two consecutive years is noticeable, the divergence increases (for the most part) steadily with the time difference. The cross-hairs from the heatmap resolve into war-time bumps in divergence, which quickly settle in peacetime. The larger boost to the divergence in recent decades, however, is more persistent suggesting a more fundamental change in the data set, which we will examine in more depth later in this section. Divergences between consecutive years typically decline through the mid-19th century. Divergences then remain relatively steady until the mid-20th century, then continue to decline gradually over time, which may be consistent with previous findings of decreased rates of word introduction and increased rates of word obsolescence in many Google Books data sets over time [8] and a slowing down of linguistic evolution over time as the vocabulary of a language expands [10]. The initial spikes in divergence in the second version of the fiction data set are likely due to the lower initial volume observed in Fig 1.

### Decade-decade comparisons using JSD word shifts

#### General observations

**Fig 7:**
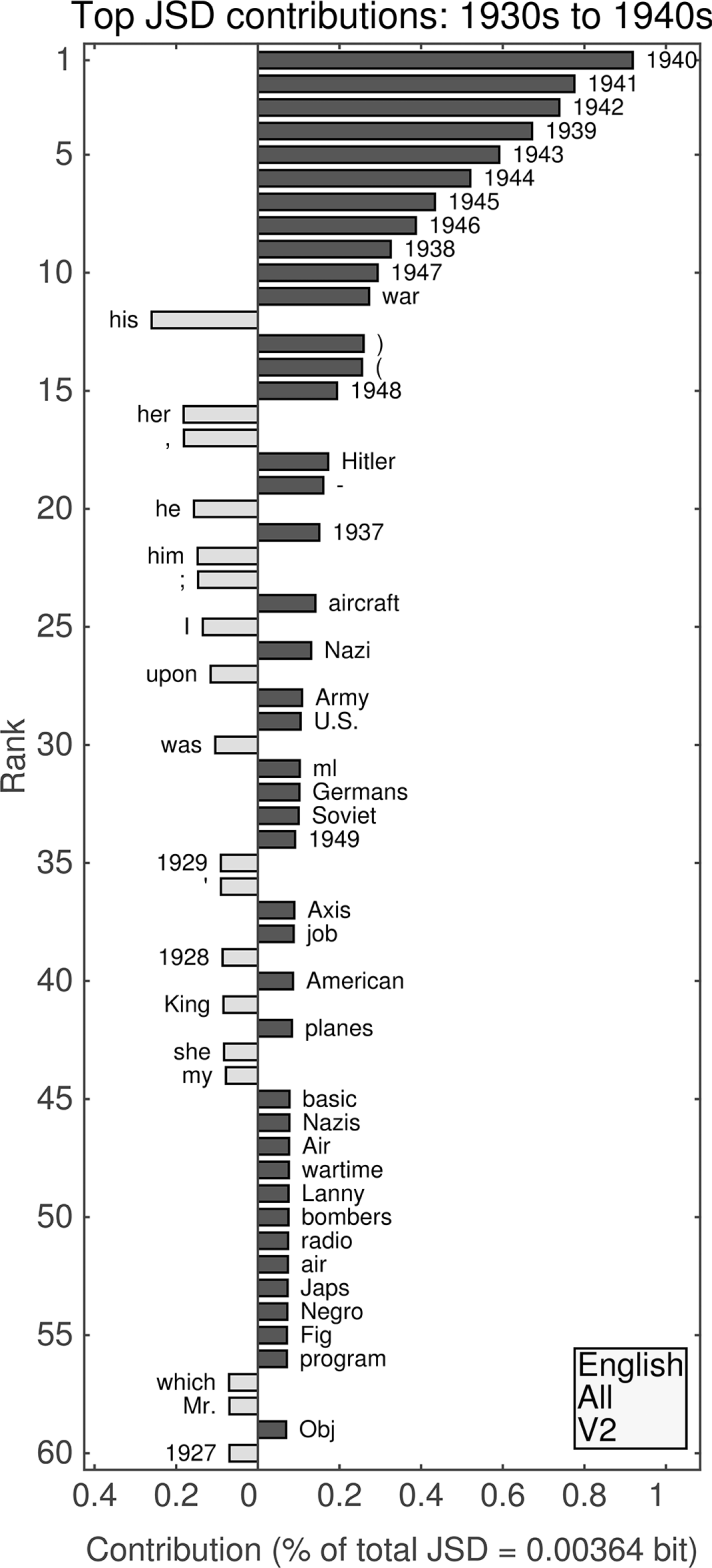
(English, all; Version 2.) Top 60 individual contributions of 1-grams to the JSD between the 1930s and the 1940s. Each contribution is given as a percentage of the total JSD (see horizontal axis label) between the two given decades. All contributions are positive; bars to the left of center represent words that were more common in the earlier decade, whereas bars to the right represent words that became more common in the later decade.

**Fig 8:**
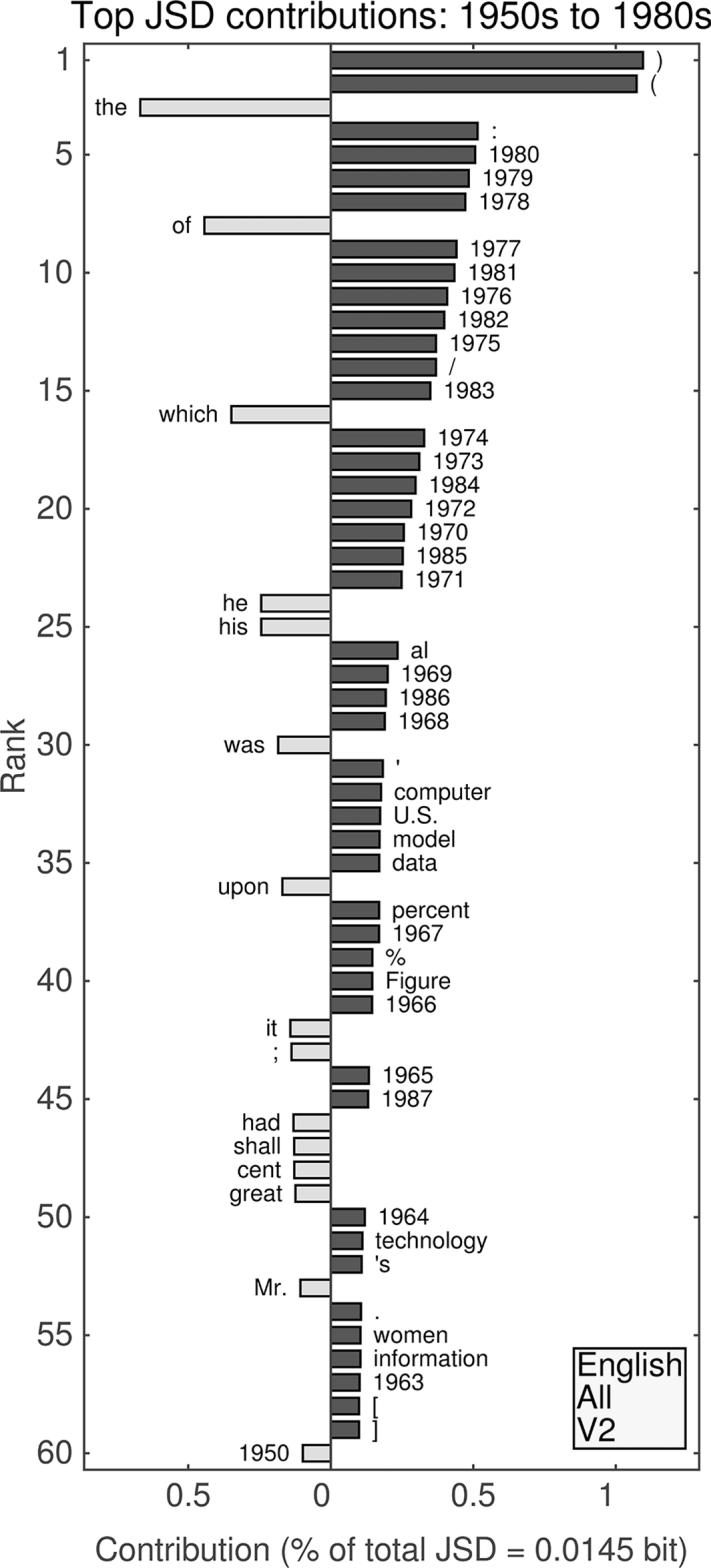
(English, all; Version 2.) Top 60 individual contributions of 1-grams to the JSD between the 1950s and the 1980s. Each contribution is given as a percentage of the total JSD (see horizontal axis label) between the two given decades. All contributions are positive; bars to the left of center represent words that were more common in the earlier decade, whereas bars to the right represent words that became more common in the later decade.

**Fig 9:**
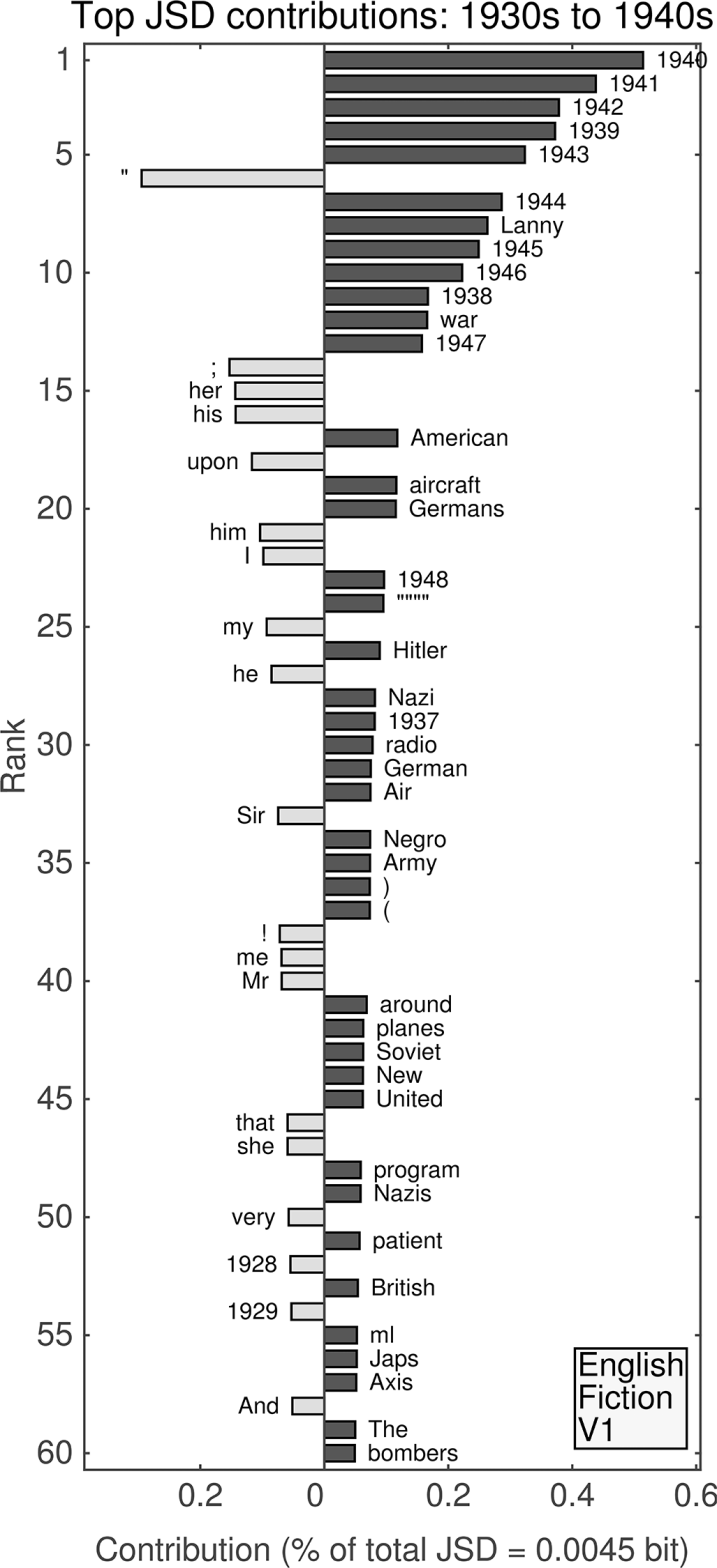
(English Fiction, Version 1.) Top 60 individual contributions of 1-grams to the JSD between the 1930s and the 1940s. Each contribution is given as a percentage of the total JSD (see horizontal axis label) between the two given decades. All contributions are positive; bars to the left of center represent words that were more common in the earlier decade, whereas bars to the right represent words that became more common in the later decade.

**Fig 10:**
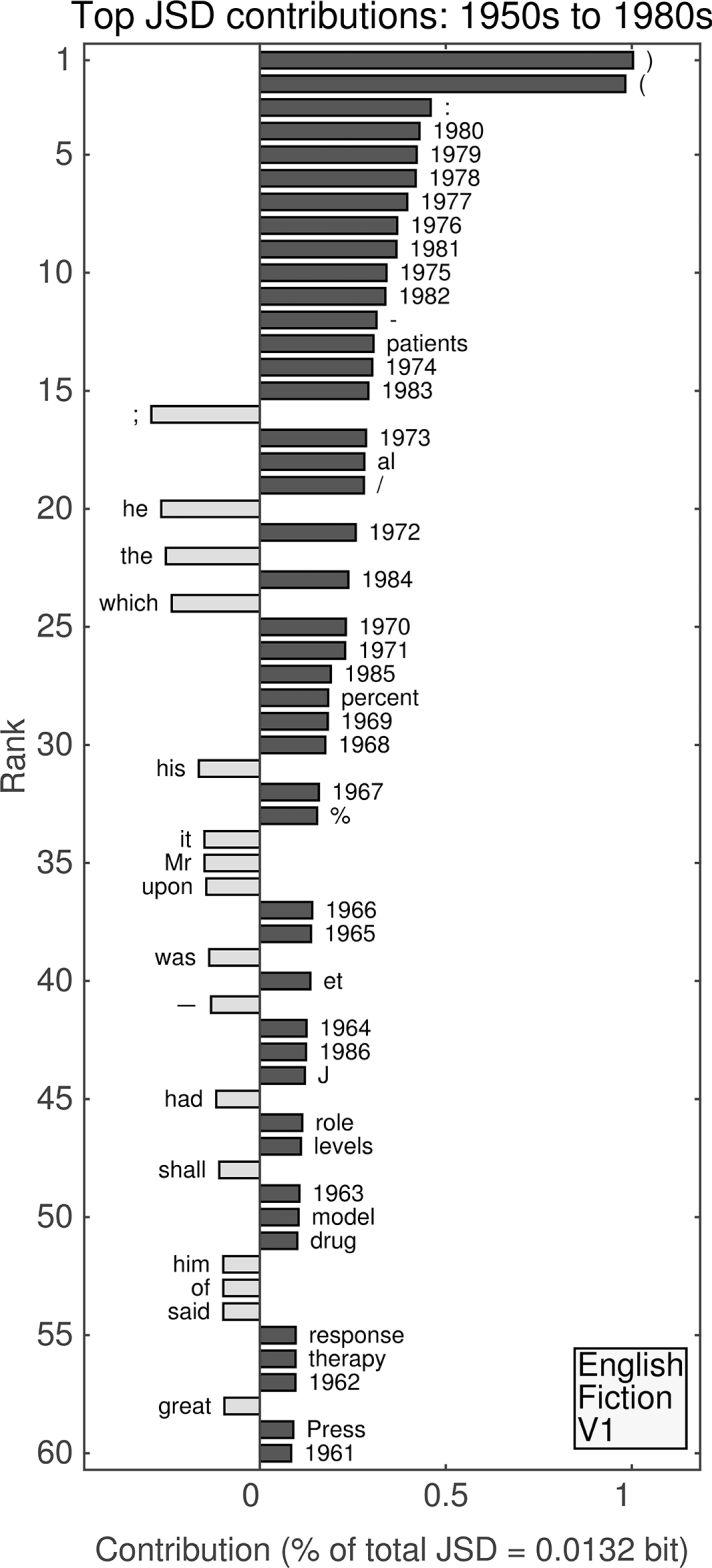
(English Fiction, Version 1.) Top 60 individual contributions of 1-grams to the JSD between the 1950s and the 1980s. Each contribution is given as a percentage of the total JSD (see horizontal axis label) between the two given decades. All contributions are positive; bars to the left of center represent words that were more common in the earlier decade, whereas bars to the right represent words that became more common in the later decade.

**Fig 11:**
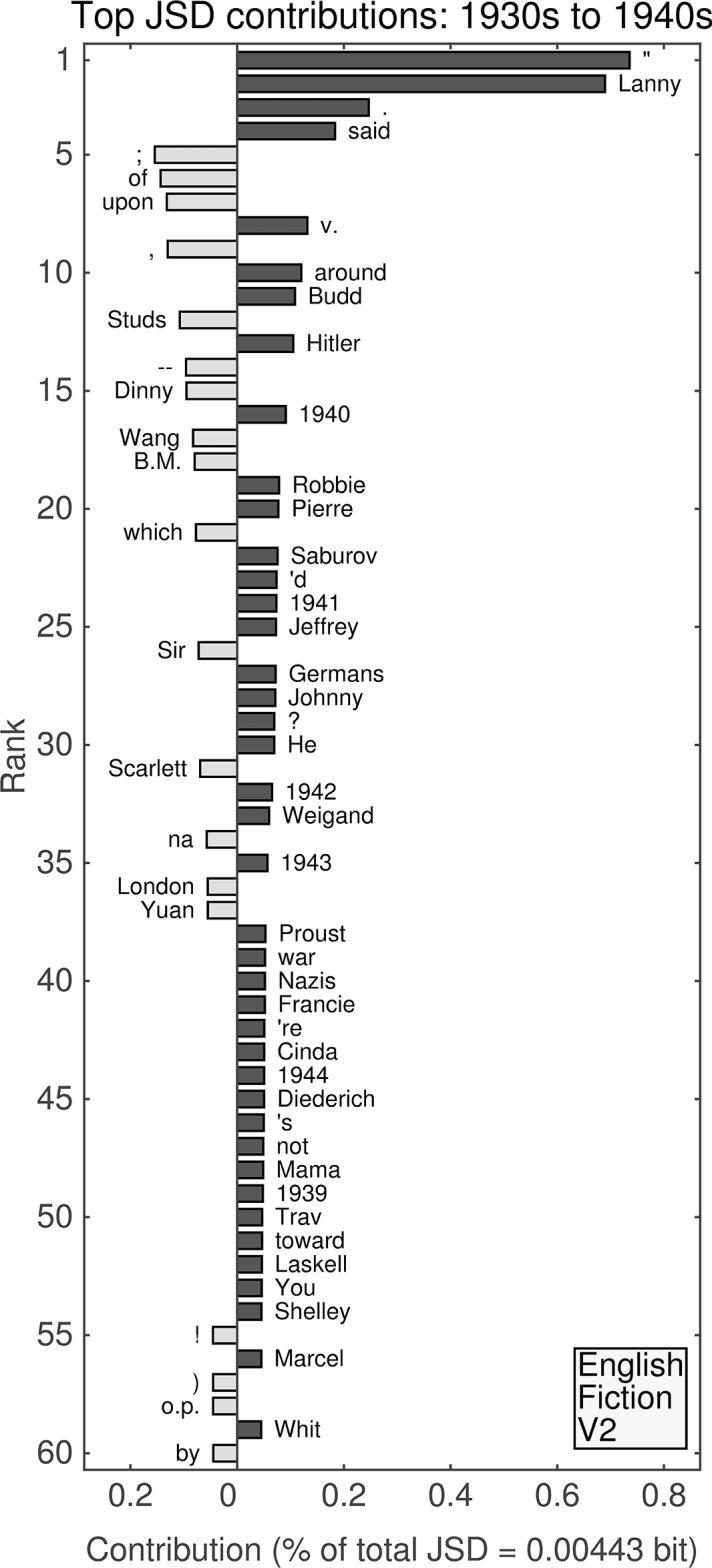
(English Fiction, Version 2.) Top 60 individual contributions of 1-grams to the JSD between the 1930s and the 1940s. Each contribution is given as a percentage of the total JSD (see horizontal axis label) between the two given decades. All contributions are positive; bars to the left of center represent words that were more common in the earlier decade, whereas bars to the right represent words that became more common in the later decade.

**Fig 12:**
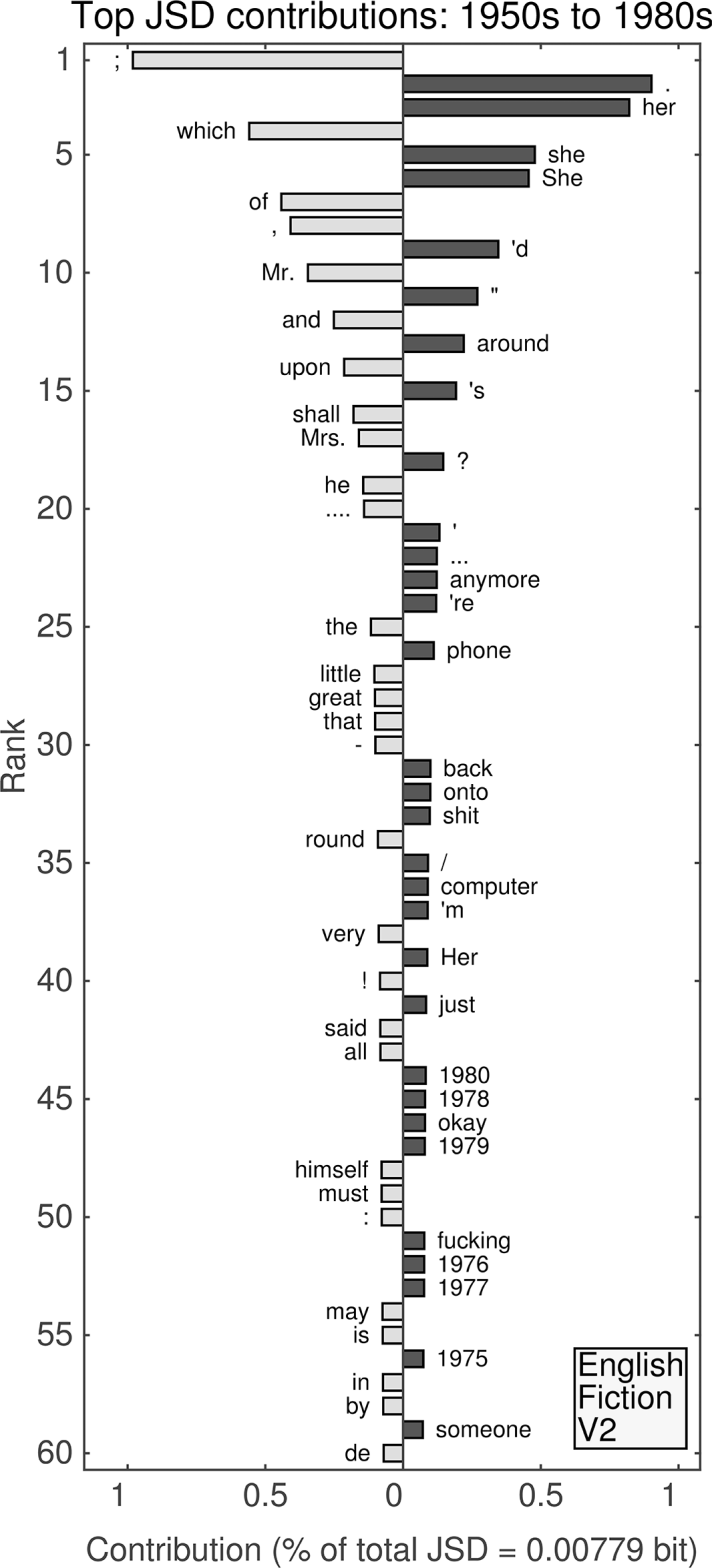
(English Fiction, Version 2.) Top 60 individual contributions of 1-grams to the JSD between the 1950s and the 1980s. Each contribution is given as a percentage of the total JSD (see horizontal axis label) between the two given decades (see title). All contributions are positive; bars to the left of center represent words that were more common in the earlier decade, whereas bars to the right represent words that became more common in the later decade.

We present “word shifts” [16] for a few examples of inter-decade divergences in Figs 7–12, specifically comparing the 1940s to the 1930s and the 1980s to the 1950s for the first unfiltered English data set (Figs 7–8) and both English Fiction data sets (Figs 9–12). We provide a full set of such comparisons in S1–S4 Files. For each of the four data sets, the largest contributions to all divergences generally appear to be from increased relative frequencies of use of words between decades. For the unfiltered data sets, these are in turn heavily influenced by increased mention of years, which is less pronounced for English Fiction.

The 1940s literature, unsurprisingly, features more references to Hitler and war than the 1930s, along with other World War II-related military and political terms. This is seen regardless of the specific data set used and is fairly encouraging. Curiously, regardless of the specific data set, a noticeable contribution is given by an increase in relative use of the words “Lanny” and “Budd,” in reference to one character (Lanny Budd) frequently written about by Upton Sinclair during that decade. In the fiction data sets, this character dominates the charts.

#### Second unfiltered English data set: 1930s versus 1940s

A comparison of the 1930s and 1940s for the second version of the unfiltered English data set (Fig 7) shows dynamics dominated by references to years. (The first version is similar. For analogous figures, see S1–S4 Files.) Eight of the top ten contributions to the divergence between those decades are due to increased relative frequencies of use of each of years between 1940 and 1949, their contribution decreasing chronologically, and the other two top ten words are the last two years of the previous decade (“1948” and “1949” appear at ranks 15 and 34, respectively). The last three years in the 1920s also appear by way of decreased relative frequency of use in the top 60 contributions. Other notable differences include:

- The 11th highest contribution is from “war,” which increased in relative frequency.
- “Hitler” and “Nazi” (increased relative frequencies) are ranked 18th and 26th, respectively.
- Parentheses (13th and 14th) show increased relative frequencies of use.
- Personal pronouns show decreased relative frequencies of use.
- The word “King” (41st) also shows a decreased relative frequency, possibly due to the British line of succession.

#### Second unfiltered English data set: 1950s versus 1980s

- The top two contributions between the 1950s and the 1980s (see Fig 8) in the English data set are both parentheses, which show dramatically increased relative frequencies of use.
- Combined with increased relative frequencies for the colon (4th), solidus/virgule (or forward slash) (14th), “computer” (32nd), and square brackets (58th and 59th), this suggests that the primary changes between the 1950s and the 1980s are due specifically to computational sources.
- Other technical words showing noticeable increases include “model” (34th), “data” (35th), “percent” and the percentage sign (37th and 39th), “Figure” (40th), “technology” (51st), and “information” (56th).
- Similarly to the divergence between the 1930s and 1940s, 19 out of the top 30 places are accounted for by increased relative frequencies of use in years between 1968 and 1980.
- The words “the” (3rd), “of” (8th), and “which” (16th) all decrease noticeably in relative frequency and are the highest ranked alphabetical 1-grams.
- Unlike the divergence between the 1930s and 1940s, only masculine pronouns show decreases in the top 60, while “women” (55th) increases.

#### First English fiction data set: 1930s versus 1940s

The first version of English Fiction shows similar dynamics to the second version of the unfiltered data set between the 1930s and the 1940s (see Fig 9) with yearly mentions dominating the ranks. Some exceptions include:

- “Lanny” rising in rank from 49th to 8th.
- Parentheses falling from 13th and 14th to 36th and 37th. “ml” (increased relative frequency of use in the 1940s) falling from 31st to 55th.
- “radio” (with increased relative frequency) rising from 51st to 30th.
- “King” is no longer in the top 60 contributions.
- “patient” enters the top 60 (ranked 51st).

#### First English fiction data set: 1950s versus 1980s

This similarity between the original English Fiction data set and the unfiltered data set also appears in the divergence between the 1950s and the 1980s (see Fig 10) with parentheses and years dominating. Moreover, “patients” ranks 13th (with increased relative frequency of use) despite not appearing in the top 60 for the unfiltered data set. These observations, combined with increases in “levels” (47th), “drug” (51st), “response” (55th), and “therapy” (56th) demonstrate the original fiction data set is strongly influenced by medical journals. Therefore, this data set cannot be considered as primarily fiction despite the label.

#### Second English fiction data set: 1930s versus 1940s

**Fig 13:**
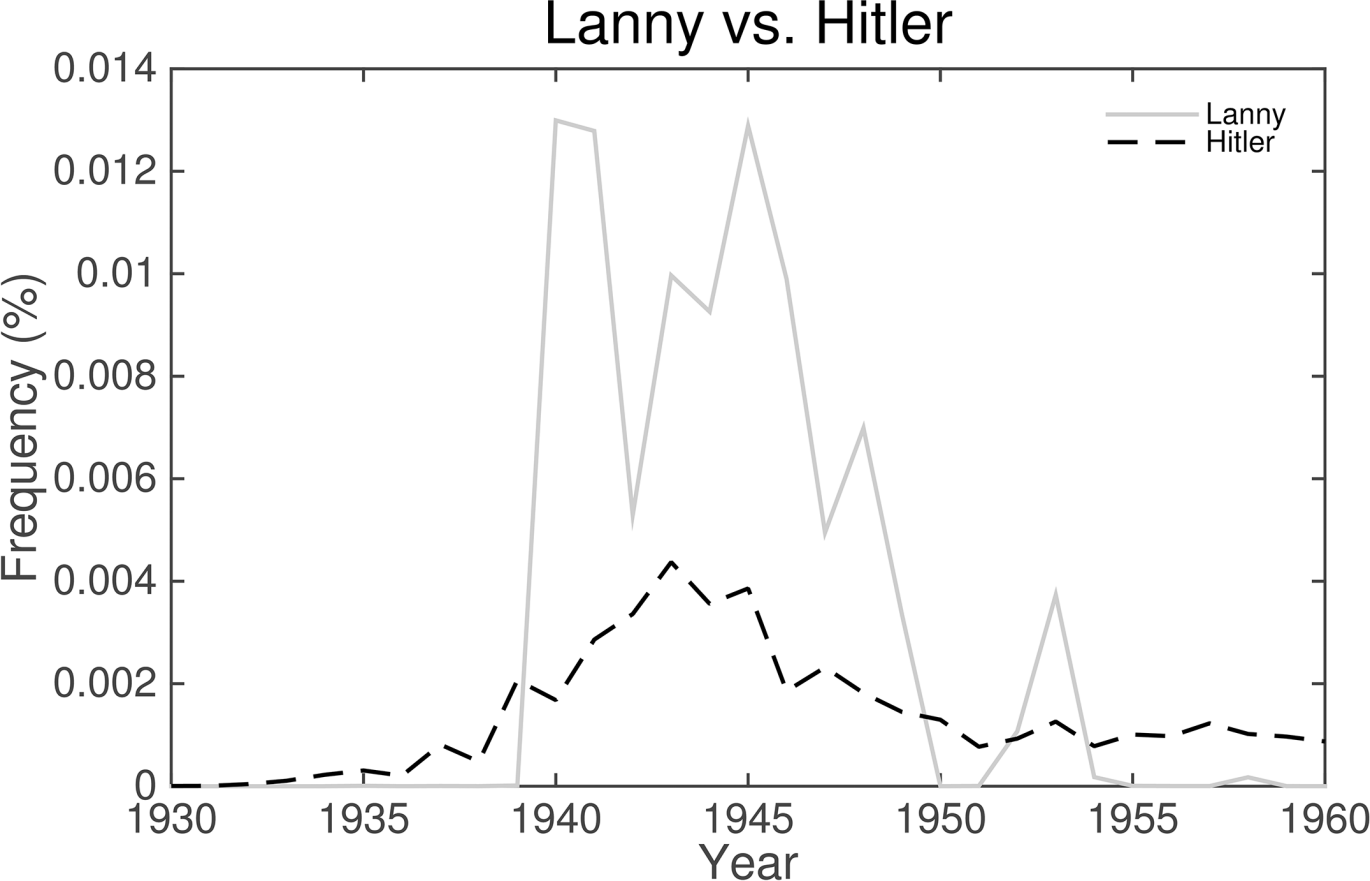
Upton Sinclair wrote 11 Lanny Budd novels set during World War II. The first of these was published in 1940, and the last was published in 1953. The net effect of Sinclair’s efforts is that his character appears much more frequently in the English Fiction (Version 2) data set than Hitler during most of the war. This demonstrates the potential impact of a single prolific author on the corpus.

Fortunately, the same is not true for the second version of the English Fiction data set. This is quickly apparent upon inspection of the two greatest contributions to the divergence between the 1930s and the 1940s (see Fig 11). The first of these is due to a dramatic increase in the relative frequencies of use of quotation marks, which implies increased dialogue. The second is the name “Lanny” in reference to the recurring character Lanny Budd from 11 Upton Sinclair novels published between 1940 and 1953. “Budd” ranks 11th in the chart ahead of “Hitler” (13th). The normalized frequency series for “Lanny” and “Hitler” provided in Fig 13 demonstrate that Lanny received more mention than Hitler during this time period. The chart is littered with the names of fictional characters:

- Studs Lonigan, the 1930s protagonist of a James T. Farrel trilogy, secures the 12th spot. (Naturally, he is mentioned fewer times during the 1940s.)
- Dinny Cherrel from the 1930s *The Forsyte Saga* by John Galsworthy secures rank 15.
- Wang Yuan from the 1930s *The House of Earth* trilogy by Pearl S. Buck ranks 17th and 37th.
- Detective Bill Weigand, a recurring character created by Richard Lockridge in the 1940s, secures rank 33.
- The eponymous, original Asimov robot from the 1940 short story, “Robbie,” ranks 19th.
- “Mama” (ranked 48th) is none other than the subject of *Mama’s Bank Account*, published in 1943 by Kathryn Forbes.
- “Saburov” (ranked 22nd) from *Days and Nights* by Konstantin Simonov and “Diederich” (ranked 45th) from *Der Untertan* by Heinrich Mann are subjects of works translated into English in the 1940s.

We note that while Marcel Proust (56th and 33rd), who died in 1922 may be present in the 1940s due to letters translated by Mina Curtiss in 1949 or other references not technically fiction. Similarly, “B.M.” (18th) may refer to the author B. M. Bower. Thus, the vast majority of prominent words in the word shift may be traced not only to authors of fiction, but to the content of their work. Moreover, the greatest contributions to divergence appear to correspond to the most prolific authors, particularly Upton Sinclair.

#### Second English fiction data set: 1930s versus 1940s

**Fig 14:**
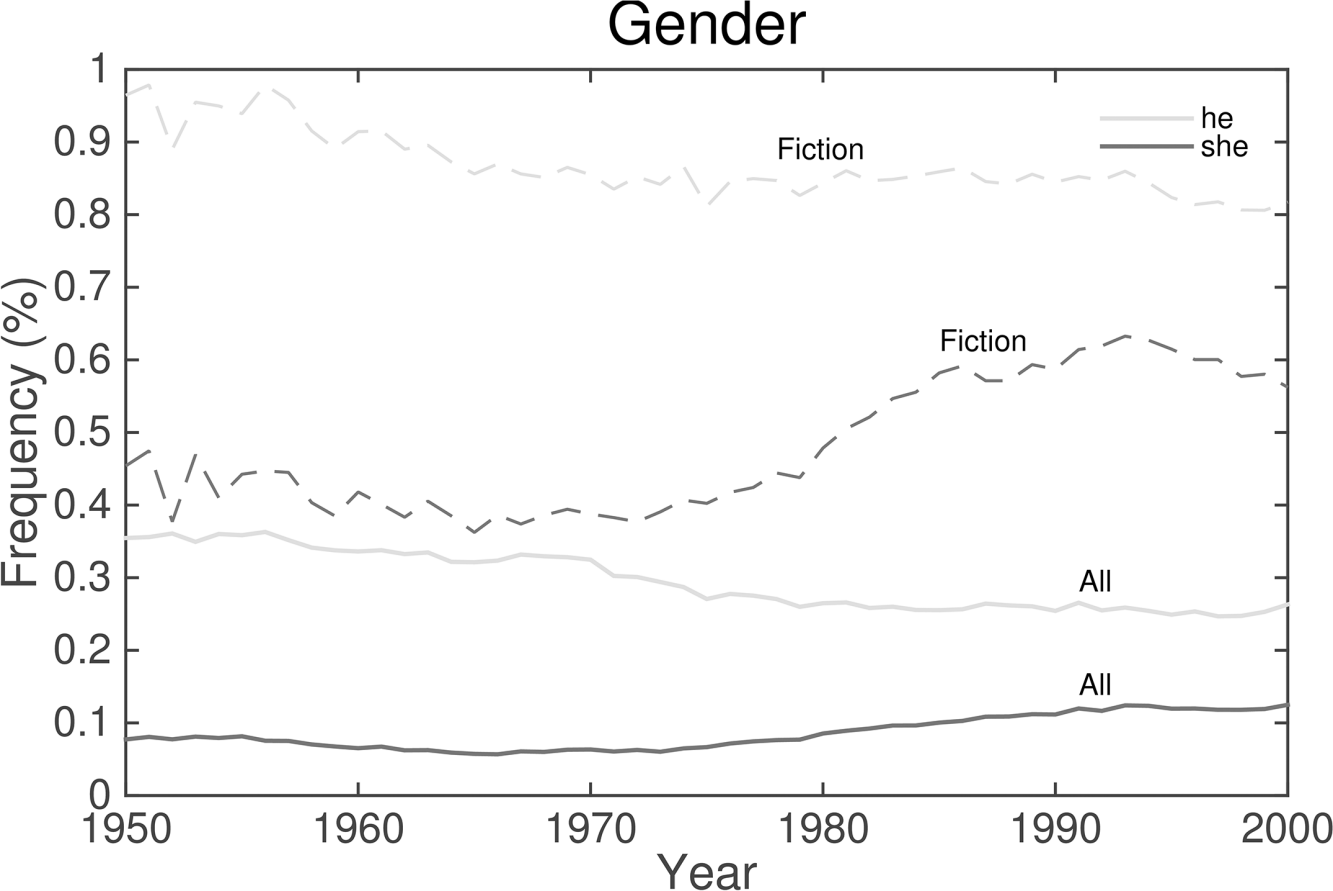
Time series for “he” and “she” for Version 2. The unfiltered normalized frequencies are given by the solid curve. Normalized frequencies in fiction are given by the dashed curve. These personal pronouns are more common in fiction. The pronoun “she” gains popularity through the 1990s in both data sets, with a more pronounced growth in fiction.

While there are no names of characters in the top divergences between the 1950s and the 1980s, the updated fiction data set (Fig 12) displays far more variety than the original version, including:

- Decreases in relative frequencies of masculine pronouns—e.g., “he” (rank 19) and “himself” (rank 48)—and corresponding increases for feminine pronouns—e.g., “her” (3rd), “she” (5th), and “She” (6th). We present times series for “he” and “she” in Fig 14.
- An increase in relative frequencies of contractions (see ranks 9, 15, and 21).
- A decrease in “shall” (16th) and “must” (49th), and a variety of increased profanity (particularly ranks 33 and 51).
- Decreases in “Mr.” (10th) and “Mrs.” (17th).
- Various shifts in punctuation, particularly fewer semicolons (1st) and more periods (2VD). Quotation (11th) and question (18th) marks both see increased relative frequencies of use in the 1980s, and the four-period ellipsis (20th) loses ground to the three-period version (22TD).

### The rise of scientific literature in the Google Books corpus

**Fig 15:**
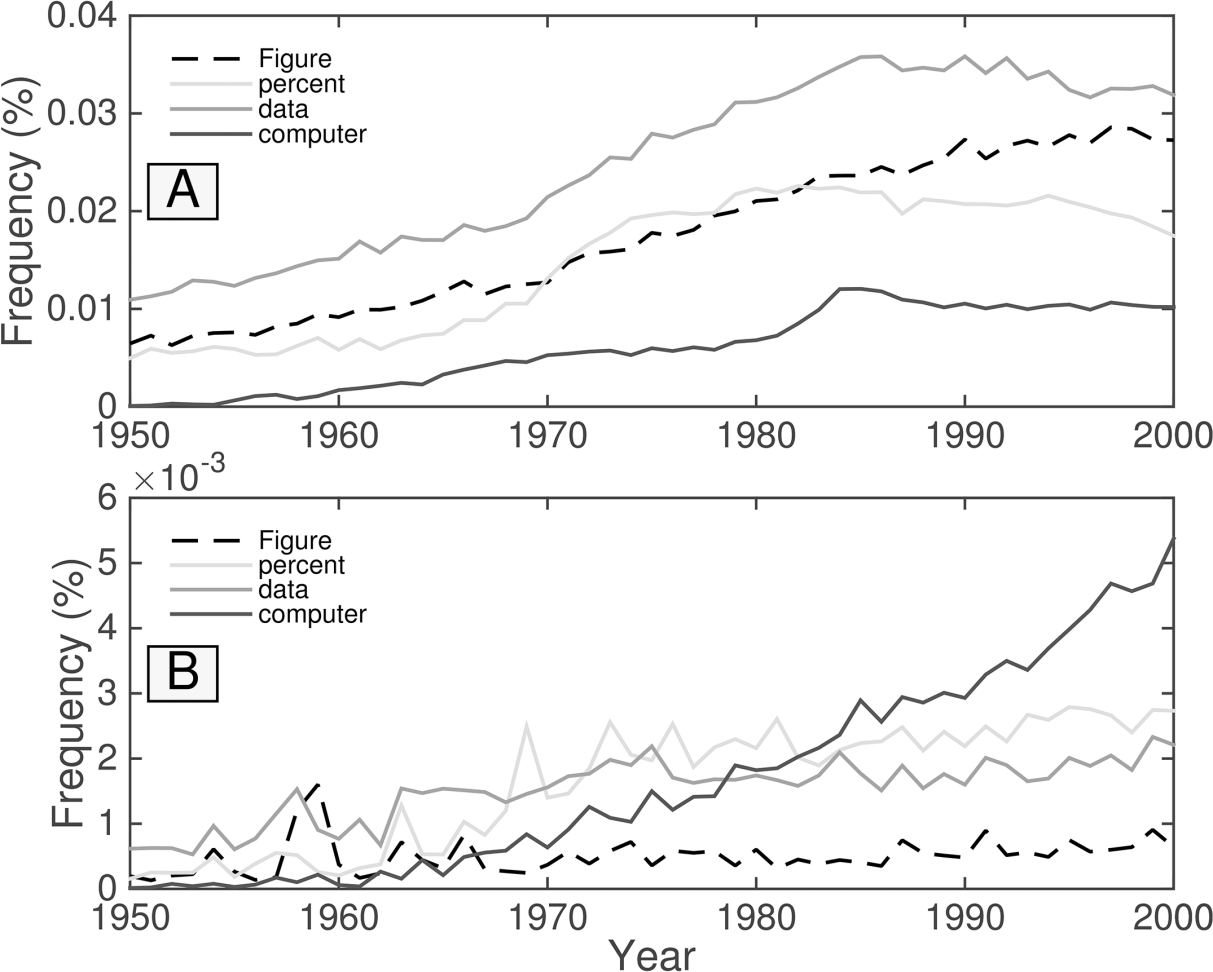
Time series of technical terms from Version 2: (a) English all, (b) English fiction. In the unfiltered data set, these technical terms appear frequently and increase in usage though the 1980s. In fiction, technical terms show up far less frequently and remain relatively stable in usage with the notable exception of “computer,” which has been gradually gaining popularity since the 1960s.

As our JSD analysis has shown above, the unfiltered English data sets feature more general scientific terms and we compare “percent,” “data,” “Figure,” and “model” in Fig 15. The original fiction data set also features these, but also places “patients,” “drug,” “response,” and “therapy” among the top 60 contributions. The primary difference between the unfiltered and original fiction data sets in the 1980s (compared to the 1950s) appears to consist of the nature of journals sampled. The unfiltered components predicted and observed for this particular data set seem to be dominated by medical journals.

**Fig 16:**
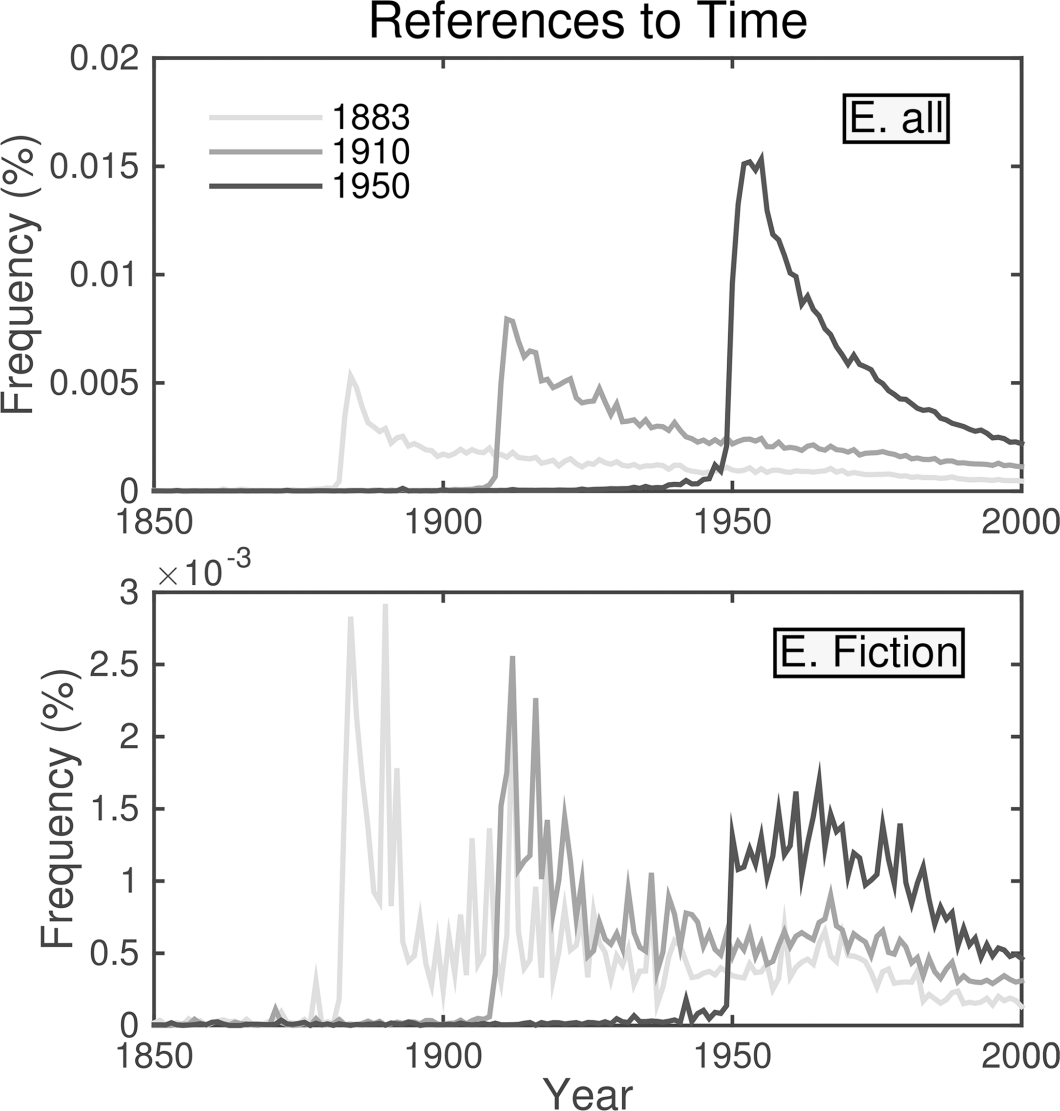
Normalized frequencies of references to years. The top panel resembles a figure from [1] using unfiltered data from English Version 2. (The cited paper uses Version 1.) Note the characteristic rapid rises and gradual declines, as well as the increasing peaks in yearly references. However, while the characteristic shape is still present in fiction (Version 2, bottom)—at much reduced levels—the peaks do not rise. The rising effect is likely due to citations from scientific texts.

As well as having more mentions of time and technical terms (and parentheses) in the 1980s than in the 1950s, both unfiltered versions and the first fiction data set include both “et” and “al” with greater relative frequency in the 1980s. Perhaps more importantly, years do not have a large effect on the dynamics in the second English Fiction data set. We see in Fig 16 that while peaks for years rise in the unfiltered data, they do not in fiction. The absence of rising peaks in fiction strongly suggests the rise in peak relative frequencies of years in the larger data set is due to a citation bias in the unfiltered data set from high sampling of scientific journals. This bias casts strong doubt on conclusions that we as a culture forget things more quickly than we once did based on the observation that half-lives for mentions of a given year decline over time [1].

The exponential rise in scientific literature is not a new phenomenon, and as de Solla Price stated in 1963 [17] (p. 81) when discussing the half-lives for citations of scientific literature, “In fields embarrassed by an inundation of literature there will be a tendency to bury as much of the past as possible and to cite older papers less often than is their statistical due.” It would see that an explanation for declining half-lives in the mentions of years lies in the dynamics of the memory of scientific discoveries rather than that of culture.

For the second fiction data set, we observe in Fig 15B, that “computer” gains popularity in the fiction data set despite other technical words remaining relatively steady in usage, as we might expect. This should be encouraging for anyone attempting to analyze colloquial English, despite the prolificacy bias apparent from the authors such as Upton Sinclair.

In S1–S4 Files, we include the top 60 contributions to divergences between each pair of the 20 decades in each of the four data sets analyzed in this paper. In total, 760 figures are included (190 per data set) for a grand total of 45,600 contributions. We highlight some of these here.

- For divergences to and from the first decade of the 1800s, many of the contributions are due to a reduction of optical character recognition confusion between the letters ‘f’ and ‘s’. For example, in the second unfiltered data set between the 1800s and 1810s, the top two contributions are due to reductions in “fame” and “os,” respectively. The word “same” (ranked 11th) is the first increasing contribution. Decreased relative frequencies of “os,” “ï¿¼sirst,” “thofe,” “fo,” “fay,” “cafe,” “fays,” “fome,” and “faid” (ranks 3 through 10, respectively) and “lise” (12th) all suggest digital misreadings of both ‘f’ and the long ‘s’. (The 13th contribution is “Napoleon,” who is mentioned with greater relative frequently in the 1810s.)
- Contributions between the 1830s and the 1860s in the second unfiltered data set highlight the American Civil War and its aftermath. “State” (11th), “General” (19th), “States” (20th), “Union” (37th), “Confederate” (48th), “Government” (52nd), “Federal” (56th), and “Constitution” (59th) all show increased relative frequency of use. Religious terms tend to decline during this period—e.g., “church” (14th), “God” (24th), and “religion” (58th).
- Between the 1940s and 1960s, the second unfiltered dataset shows increases for “nuclear” (43rd), “Vietnam” (47th), and “Communist” (50th). The relative frequency of “war” (25th) decreases substantially. Meanwhile in fiction, “Lanny” (5th) declines, while “television” (38th) and the Hardy Boys (“Hardy” ranks 51st) appear with greater relative frequencies.
- Between the 1960s and 1970s, the second fiction data set is strongly affected by “Garp” (*The World According to Garp* by John Irving, 1978) at rank 19, increased relative frequencies of profanity (ranks 27, 33, and 38), and increased mentions of “Nixon” (41st) and “Spock” (47th, likely due to “Star Trek” novels).
- Between the 1980s and 1990s, the second fiction set shows increased relative frequencies of use of the words “gay” (15th), “lesbian” (19th), “AIDS” (24th), and “gender” (27th). Female pronouns (2nd, 8th, and 9th) show increased relative frequencies of use in continuance of Fig 12.

## Concluding Remarks

Based on our introductory remarks and ensuing detailed analysis, it should now be clear that the contents of the Google Books corpus do not represent an unbiased sampling of publications. Beyond being library-like, the evolution of the corpus throughout the 1900s is increasingly dominated by scientific publications rather than popular works. We have shown that even the first data set specifically labeled as fiction appears to be saturated with medical literature.

When examining these data sets in the future, it will therefore be necessary to first identify and distinguish the popular and scientific components in order to form a picture of the corpus that is informative about cultural and linguistic evolution. For instance, one should ask how much of any observed gender shift in language reflects word choice in popular works and how much is due to changes in scientific norms, as well as which might precede the other if they are somewhat in balance.

Even if we are able to restrict our focus to popular works by appropriately filtering scientific terms, the library-like nature of the Google Books corpus will mean the resultant normalized frequencies of words cannot be a direct measure of the “true” cultural popularity of those words as they are read (again, Frodo). Secondarily, not only will there be a delay between changes in the public popularity of words and their appearance in print, normalized frequencies will also be affected by the prolificacy of the authors. In the case of Upton Sinclair’s Lanny Budd, a fictional character was vaulted to the upper echelons of words affecting divergence (even surpassing Hitler) by virtue of appearing as the protagonist in 11 novels between 1940 and 1953. Google Books is at best a limited proxy for social information after the fact.

The Google Books corpus’s beguiling power to immediately quantify a vast range of linguistic trends warrants a very cautious approach to any effort to extract scientifically meaningful results. Our analysis provides a possible framework for improvements to previous and future works which, if performed on English data, ought to focus solely on the second version of the English Fiction data set, or otherwise properly account for the biases of the unfiltered corpus.

## Supporting Information

S1 File Decade-decade word comparisons using JSD word shifts for the second (2012) version of the Google Books English data set. (PDF)

S2 File Decade-decade word comparisons using JSD word shifts for the first (2009) version of the Google Books English data set. (PDF)

S3 File Decade-decade word comparisons using JSD word shifts for the second (2012) version of the Google Books English Fiction data set. (PDF)

S4 File Decade-decade word comparisons using JSD word shifts for the first (2009) version of the Google Books English Fiction data set. (PDF)
