# Supplementary material for: Characterizing the Google Books Corpus: Strong Limits to Inferences of Socio-Cultural and Linguistic Evolution
Source: PLoS One. 2015 Oct 7;10(10):e0137041. doi: 10.1371/journal.pone.0137041 (PMC4596490; doi:10.1371/journal.pone.0137041)
Supplement: S1 File — (PDF) [file pone.0137041.s001.pdf]

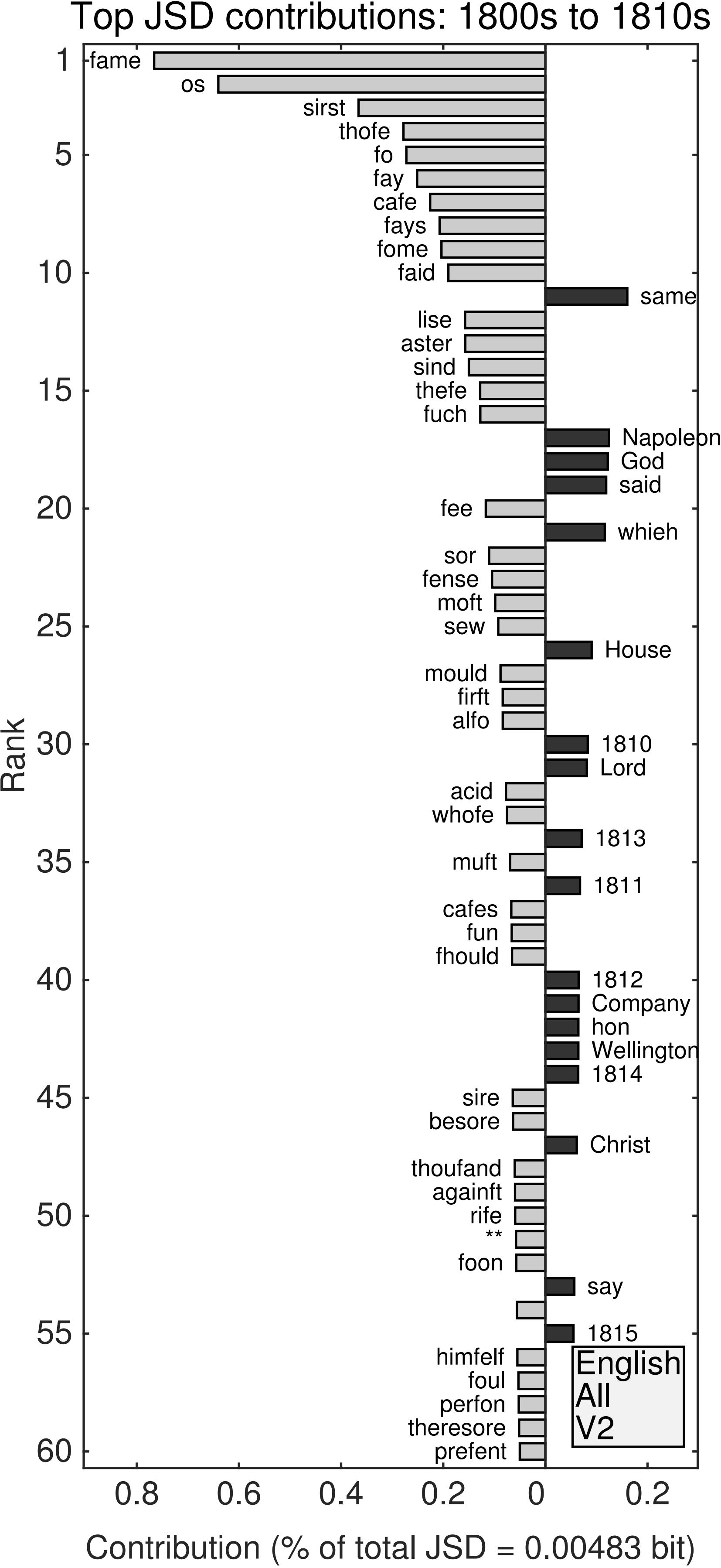
Top JSD contributions: 1800s to 1810s
Rank
fame
os
sirst
thofe
fo
fay
cafe
fays
fome
faid
same
lise
aster
sind
thefe
fuch
Napoleon
God
said
fee
whieh
sor
fense
moft
sew
House
mould
firft
alfo
1810
Lord
acid
whofe
1813
muft
1811
cafes
fun
fhould
1812
Company
hon
Wellington
1814
sire
besore
Christ
thoufand
againft
rife
**
foon
say
1815
himfelf
foul
perfon
theresore
prefent
English
All
V2
1
5
10
15
20
25
30
35
40
45
50
55
60
0.8
0.6
0.4
0.2
0
0.2
Contribution (% of total JSD = 0.00483 bit)

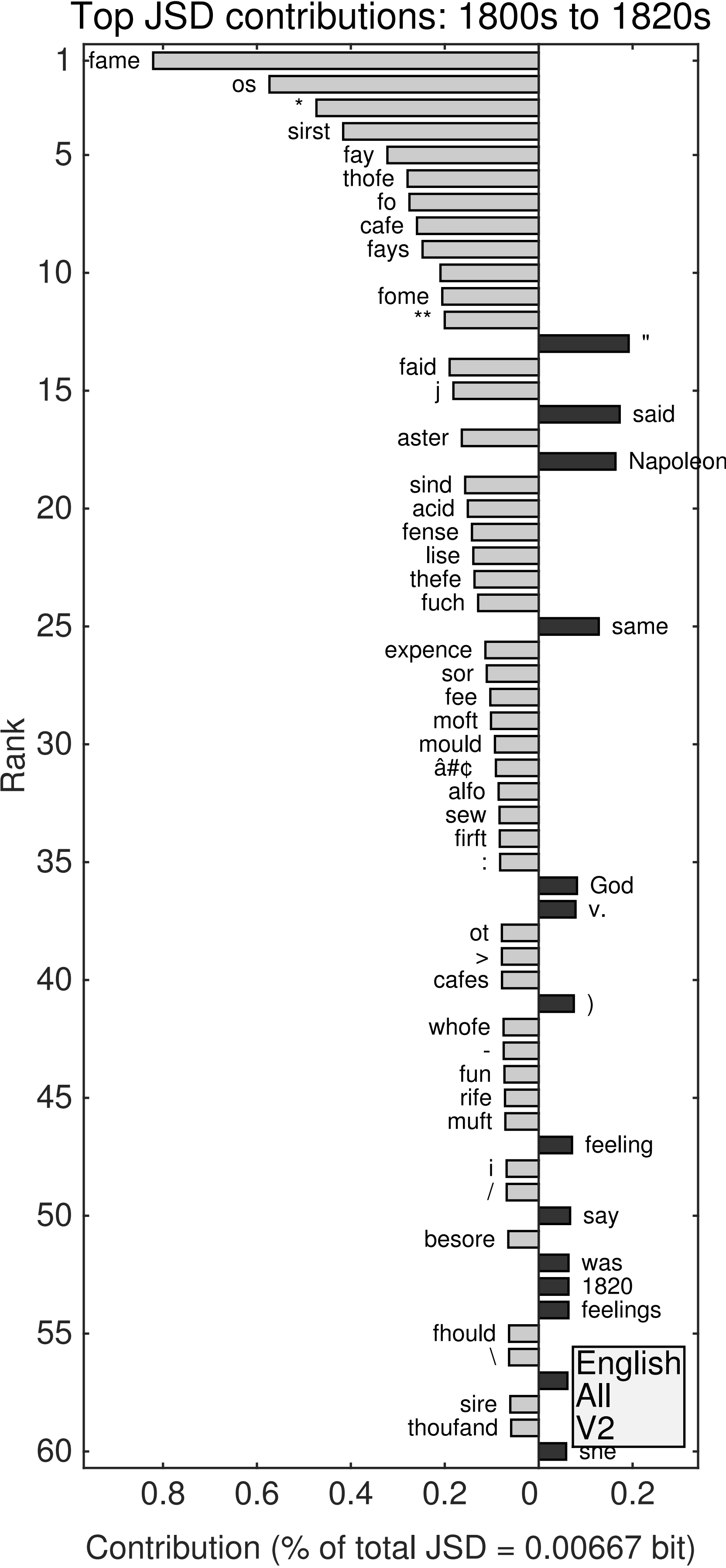

Top JSD contributions: 1800s to 1820s
Rank
Contribution (% of total JSD = 0.00667 bit)
0.8
0.6
0.4
0.2
0
0.2
1
5
10
15
20
25
30
35
40
45
50
55
60
fame
os
*
sirst
fay
thofe
fo
cafe
fays
fome
**
"
faid
j
said
aster
Napoleon
sind
acid
fense
lise
thefe
fuch
same
expence
sor
fee
moft
mould
â#¢
alfo
sew
firft
:
God
v.
ot
>
cafes
)
whofe
-
fun
rife
muft
feeling
i
/
say
besore
was
1820
feelings
fhould
\
sire
thoufand
she
English
All
V2

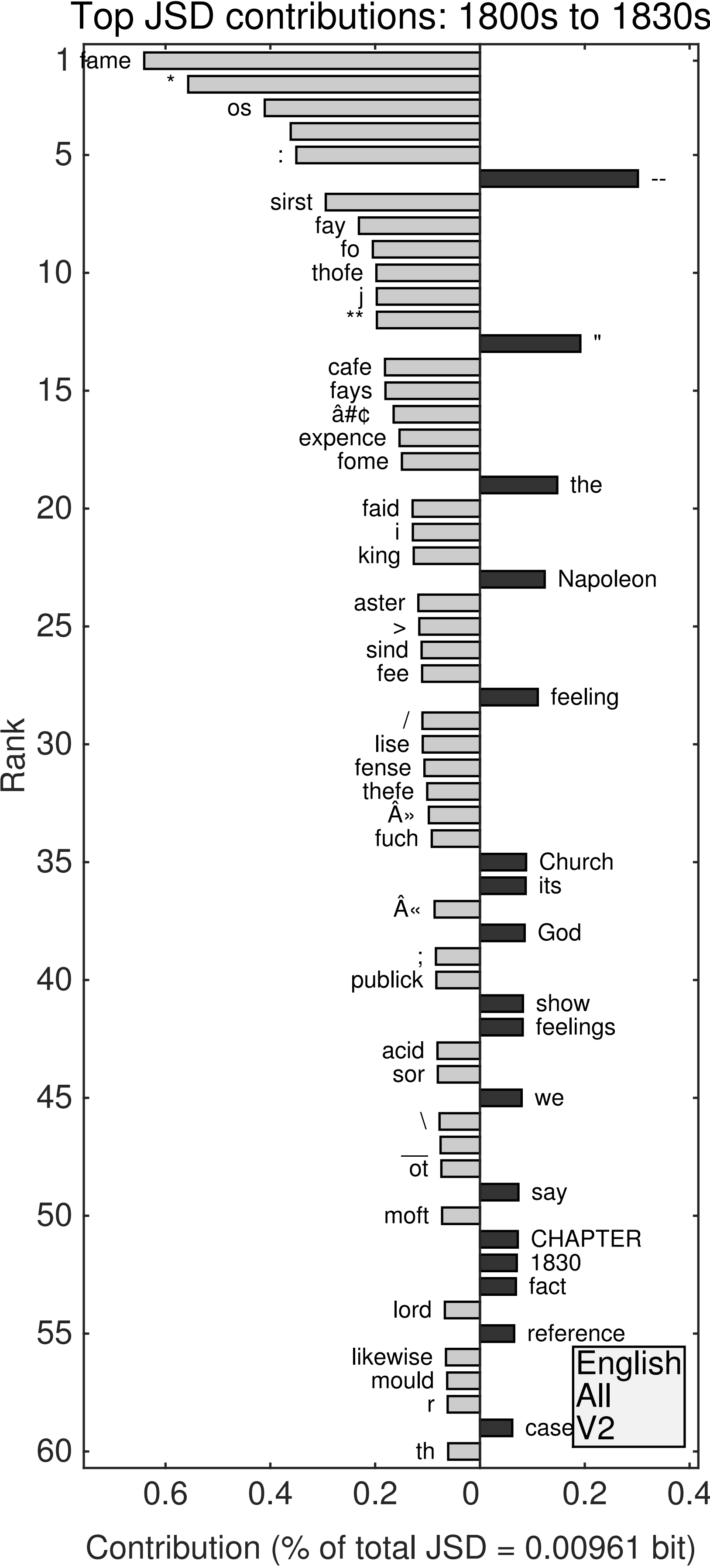

Top JSD contributions: 1800s to 1830s
Rank
Contribution (% of total JSD = 0.00961 bit)
fame
*
os
:
--
sirst
fay
fo
thofe
j
**
"
cafe
fays
â#¢
expence
fome
the
faid
i
king
Napoleon
aster
>
sind
fee
feeling
/
lise
fense
thefe
Â»
fuch
Church
its
Â«
God
;
publick
show
feelings
acid
sor
we
\
___
ot
say
moft
CHAPTER
1830
fact
lord
reference
likewise
mould
r
case
th
English
All
V2

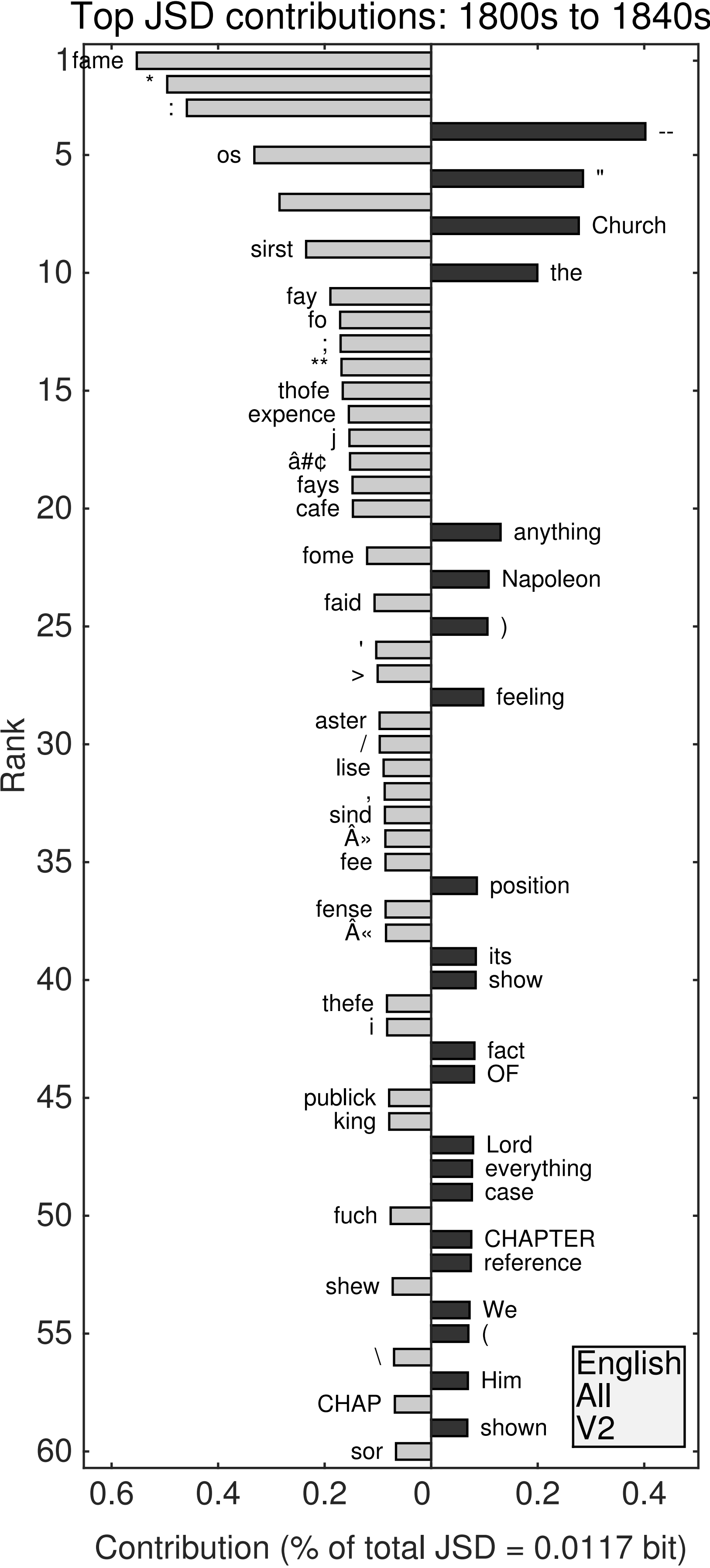

Top JSD contributions: 1800s to 1840s
Rank
Contribution (% of total JSD = 0.0117 bit)
fame
*
:
--
os
"
Church
sirst
the
fay
fo
;
**
thofe
expence
j
â#¢
fays
cafe
anything
fome
Napoleon
faid
)
'
>
feeling
aster
/
lise
,
sind
Â»
fee
position
fense
Â«
its
show
thefe
i
fact
OF
publick
king
Lord
everything
case
fuch
CHAPTER
reference
shew
We
(
\
Him
CHAP
shown
sor
English
All
V2

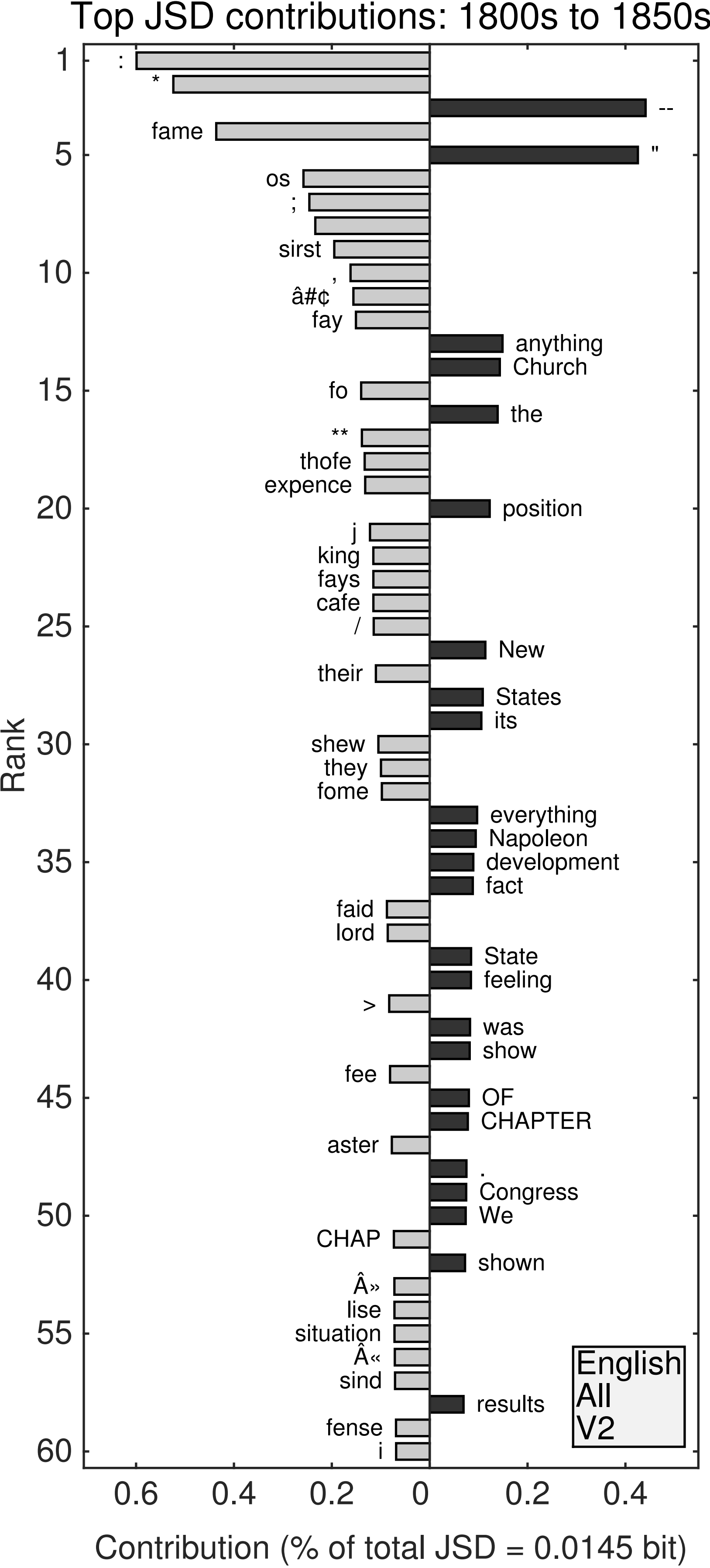

Top JSD contributions: 1800s to 1850s
Rank
Contribution (% of total JSD = 0.0145 bit)
0.6
0.4
0.2
0
0.2
0.4
1
5
10
15
20
25
30
35
40
45
50
55
60
:
*
--
fame
"
os
;
sirst
,
â#¢
fay
anything
Church
fo
the
**
thofe
expence
position
j
king
fays
cafe
/
New
their
States
its
shew
they
fome
everything
Napoleon
development
fact
faid
lord
State
feeling
>
was
show
fee
OF
CHAPTER
aster
.
Congress
We
CHAP
shown
Â»
lise
situation
Â«
sind
results
fense
i
English
All
V2

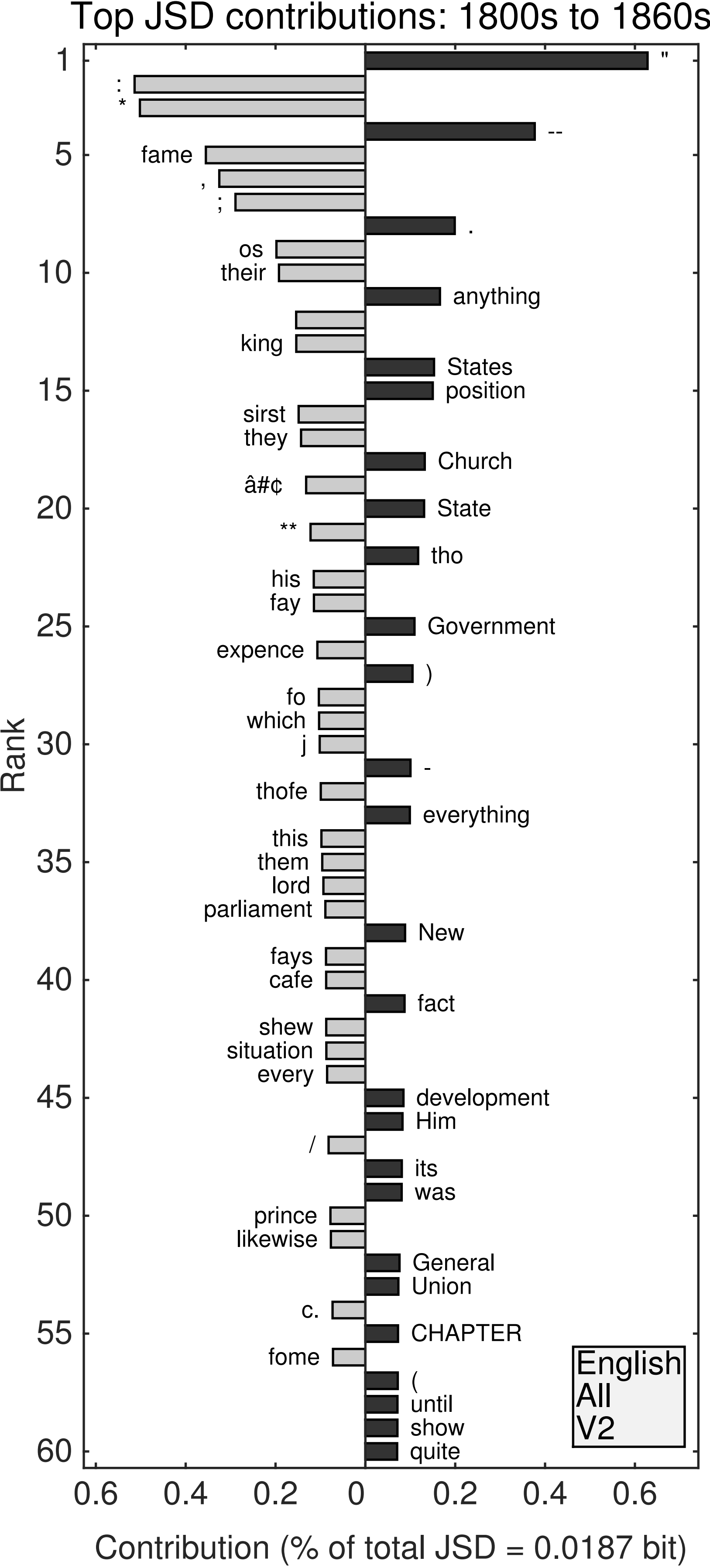

Top JSD contributions: 1800s to 1860s
Rank
Contribution (% of total JSD = 0.0187 bit)
English
All
V2
"
:
*
--
fame
,
;
.
os
their
anything
king
States
position
sirst
they
Church
â#¢
State
**
tho
his
fay
Government
expence
)
fo
which
j
-
thofe
everything
this
them
lord
parliament
New
fays
cafe
fact
shew
situation
every
development
Him
/
its
was
prince
likewise
General
Union
c.
CHAPTER
fome
(
until
show
quite

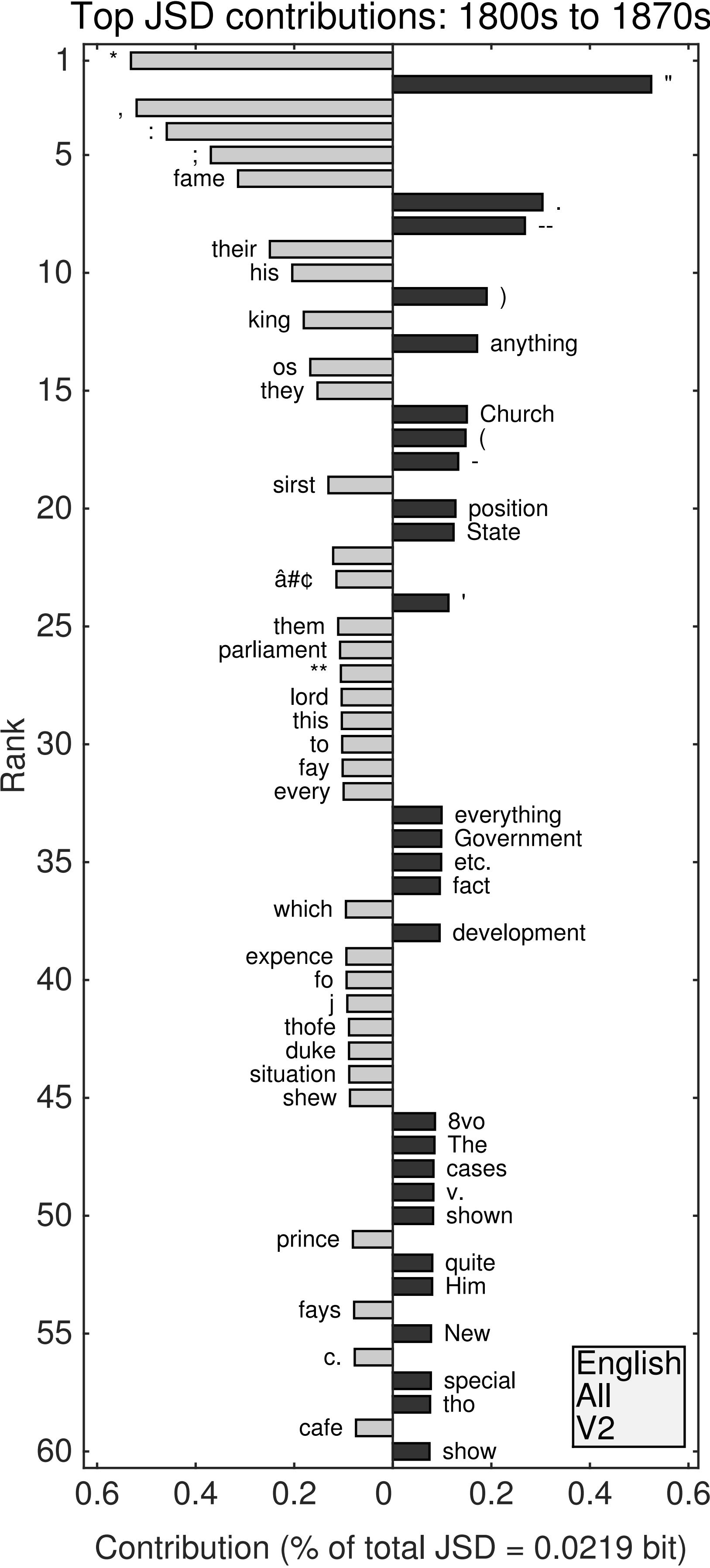

Top JSD contributions: 1800s to 1870s
Rank
Contribution (% of total JSD = 0.0219 bit)
English
All
V2
*
"
,
:
;
fame
.
--
their
his
)
king
anything
os
they
Church
(
-
sirst
position
State
â#¢
'
them
parliament
**
lord
this
to
fay
every
everything
Government
etc.
fact
which
development
expence
fo
j
thofe
duke
situation
shew
8vo
The
cases
v.
shown
prince
quite
Him
fays
New
c.
special
tho
cafe
show

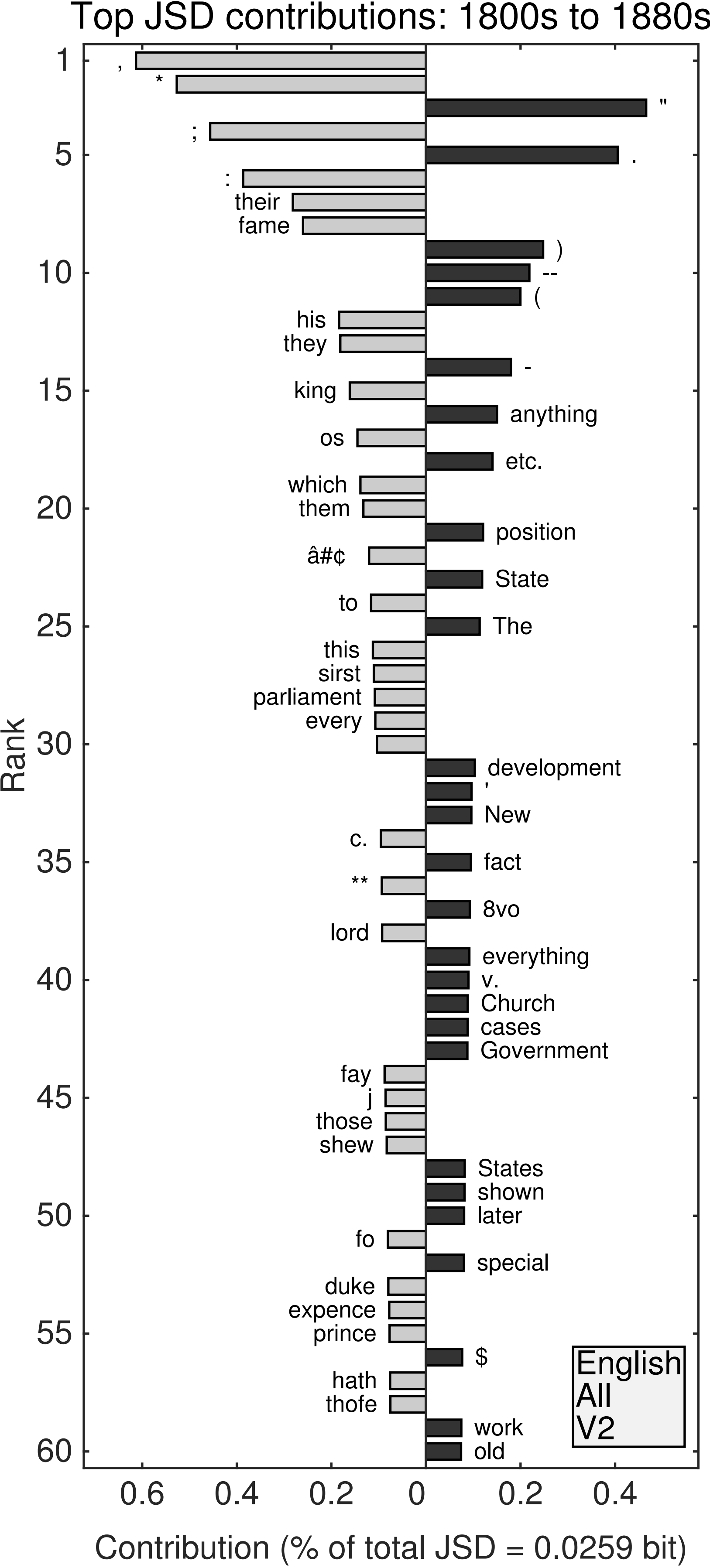
Top JSD contributions: 1800s to 1880s
Rank
Contribution (% of total JSD = 0.0259 bit)
English
All
V2
,
*
"
;
.
:
their
fame
)
--
(
his
they
-
king
anything
os
etc.
which
them
position
â#¢
State
to
The
this
sirst
parliament
every
development
'
New
c.
fact
**
8vo
lord
everything
v.
Church
cases
Government
fay
j
those
shew
States
shown
later
fo
special
duke
expence
prince
$
hath
thofe
work
old

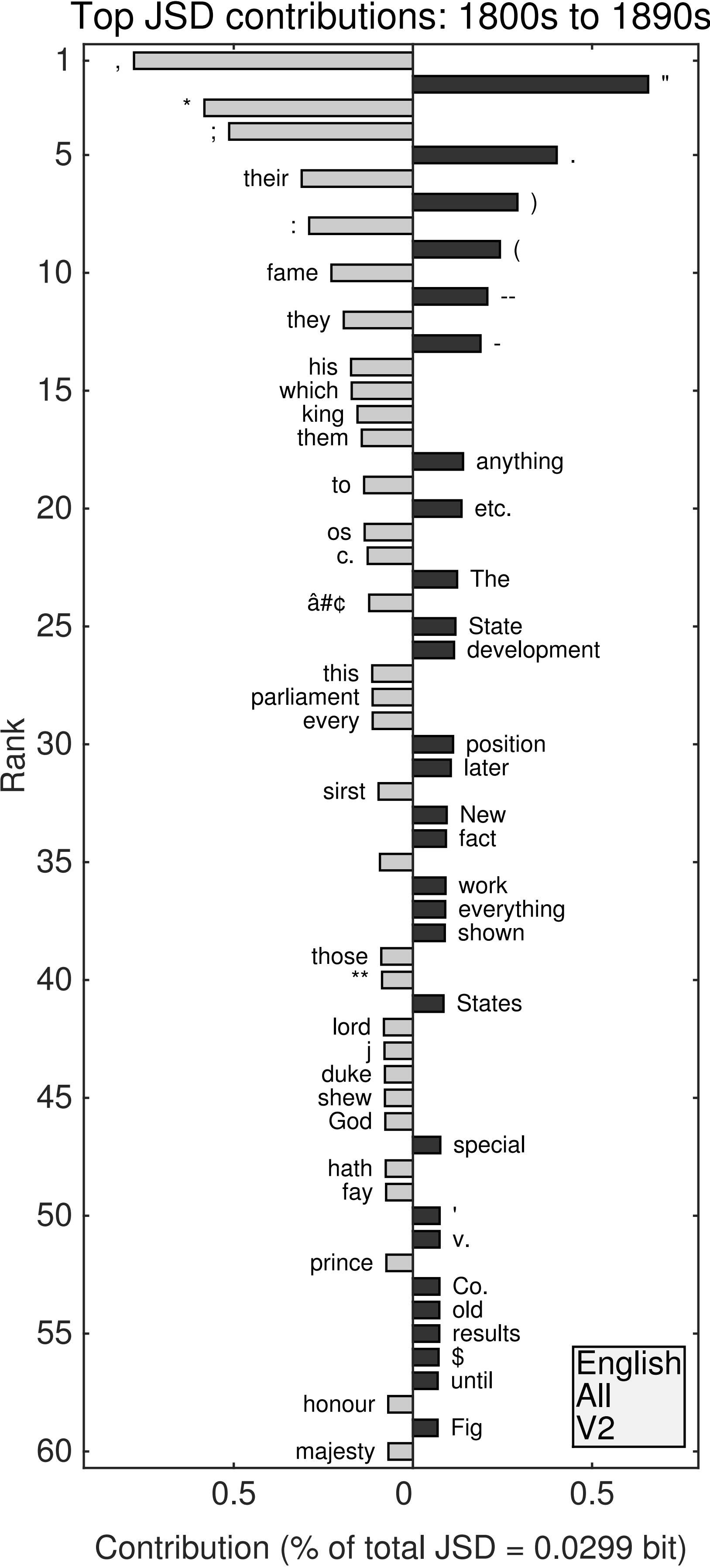

Top JSD contributions: 1800s to 1890s
Rank
Contribution (% of total JSD = 0.0299 bit)
1
5
10
15
20
25
30
35
40
45
50
55
60
0.5
0
0.5
,
"
*
;
.
their
)
:
(
fame
--
they
-
his
which
king
them
anything
to
etc.
os
c.
The
â#¢
State
development
this
parliament
every
position
later
sirst
New
fact
work
everything
shown
those
**
States
lord
j
duke
shew
God
special
hath
fay
'
v.
prince
Co.
old
results
$
until
honour
Fig
majesty
English
All
V2

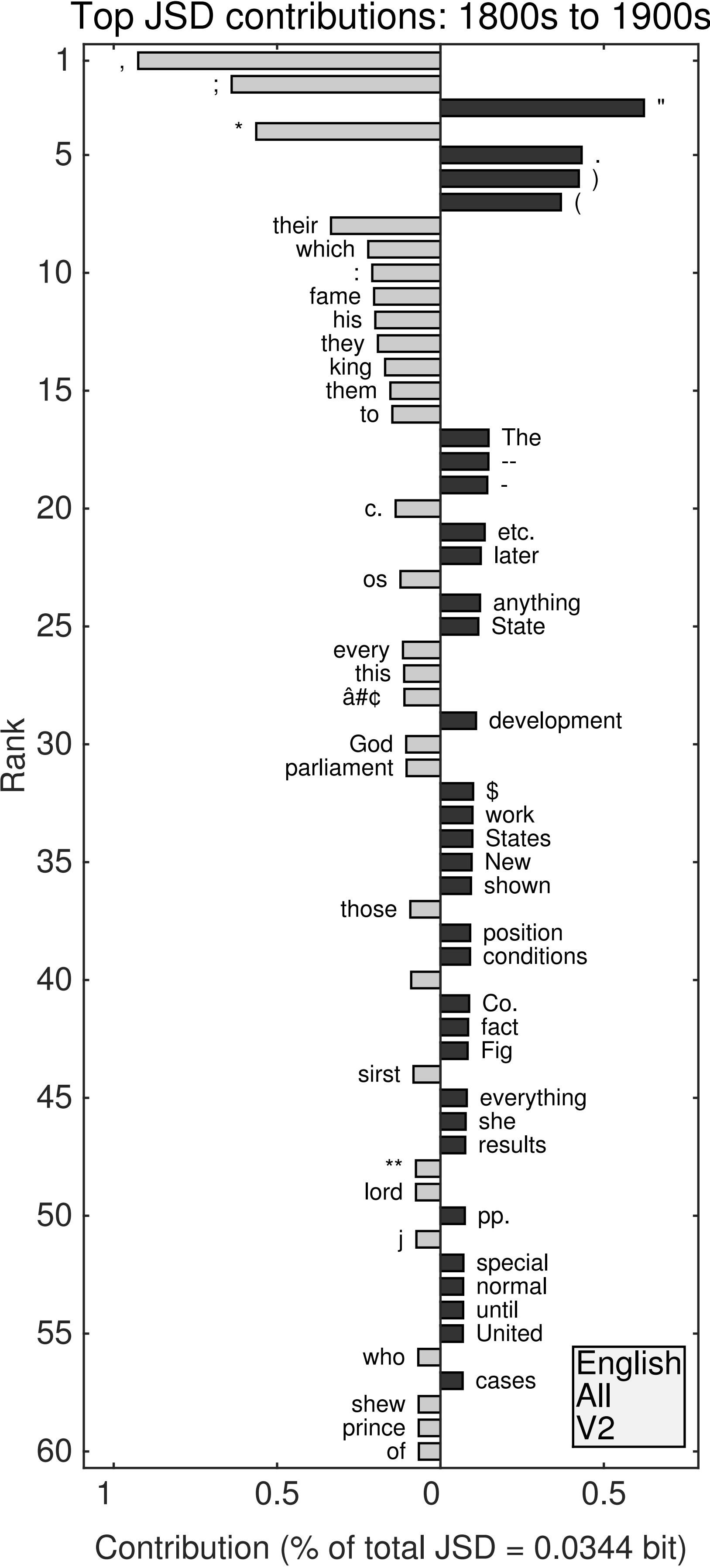

Top JSD contributions: 1800s to 1900s
Rank
Contribution (% of total JSD = 0.0344 bit)
English
All
V2
,
;
"
*
.
)
(
their
which
:
fame
his
they
king
them
to
The
--
-
c.
etc.
later
os
anything
State
every
this
â#¢
development
God
parliament
$
work
States
New
shown
those
position
conditions
Co.
fact
Fig
sirst
everything
she
results
**
lord
pp.
j
special
normal
until
United
who
cases
shew
prince
of

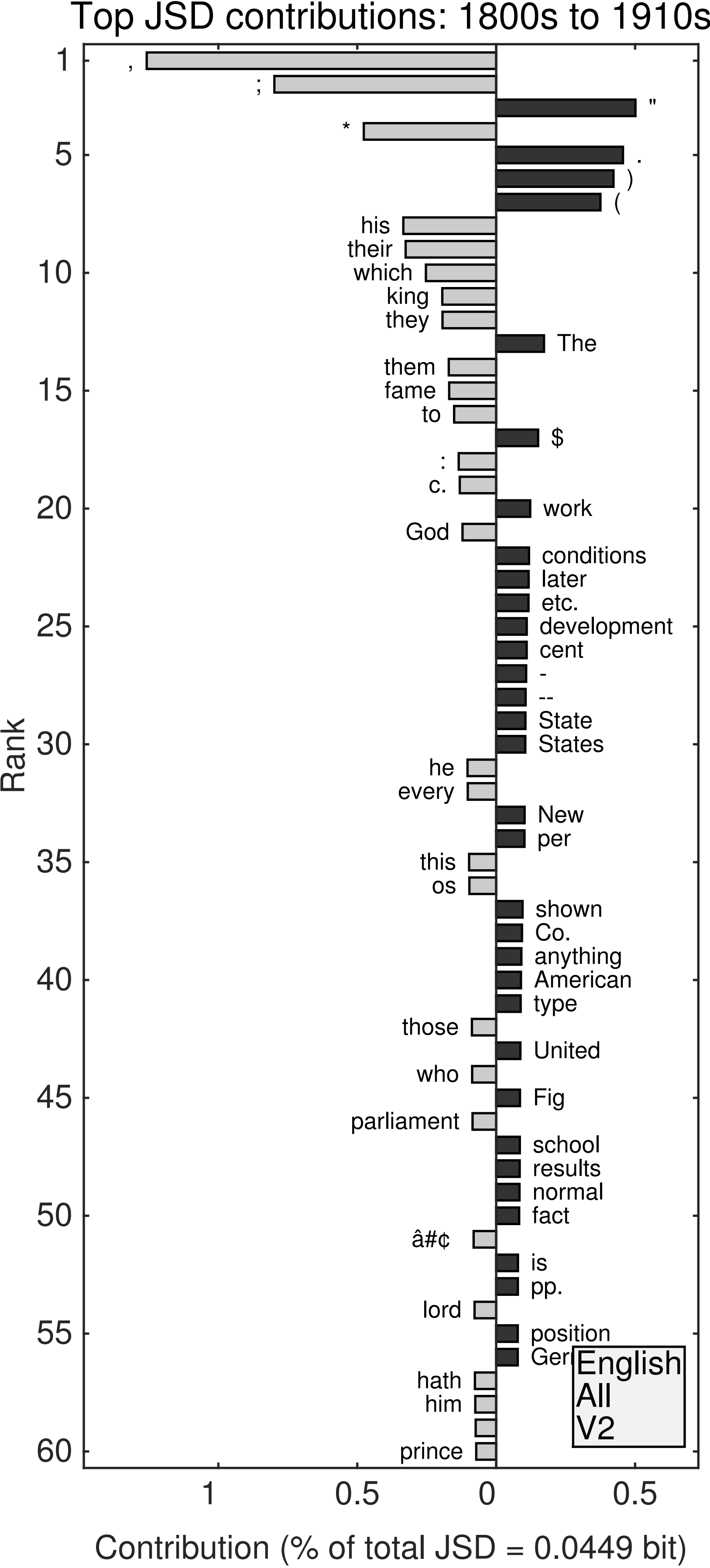
Top JSD contributions: 1800s to 1910s
Rank
Contribution (% of total JSD = 0.0449 bit)
1
5
10
15
20
25
30
35
40
45
50
55
60
1
0.5
0
0.5
,
;
"
*
.
)
(
his
their
which
king
they
The
them
fame
to
$
:
c.
work
God
conditions
later
etc.
development
cent
-
--
State
States
he
every
New
per
this
os
shown
Co.
anything
American
type
those
United
who
Fig
parliament
school
results
normal
fact
â#¢
is
pp.
lord
position
Ger
hath
him
prince
English
All
V2

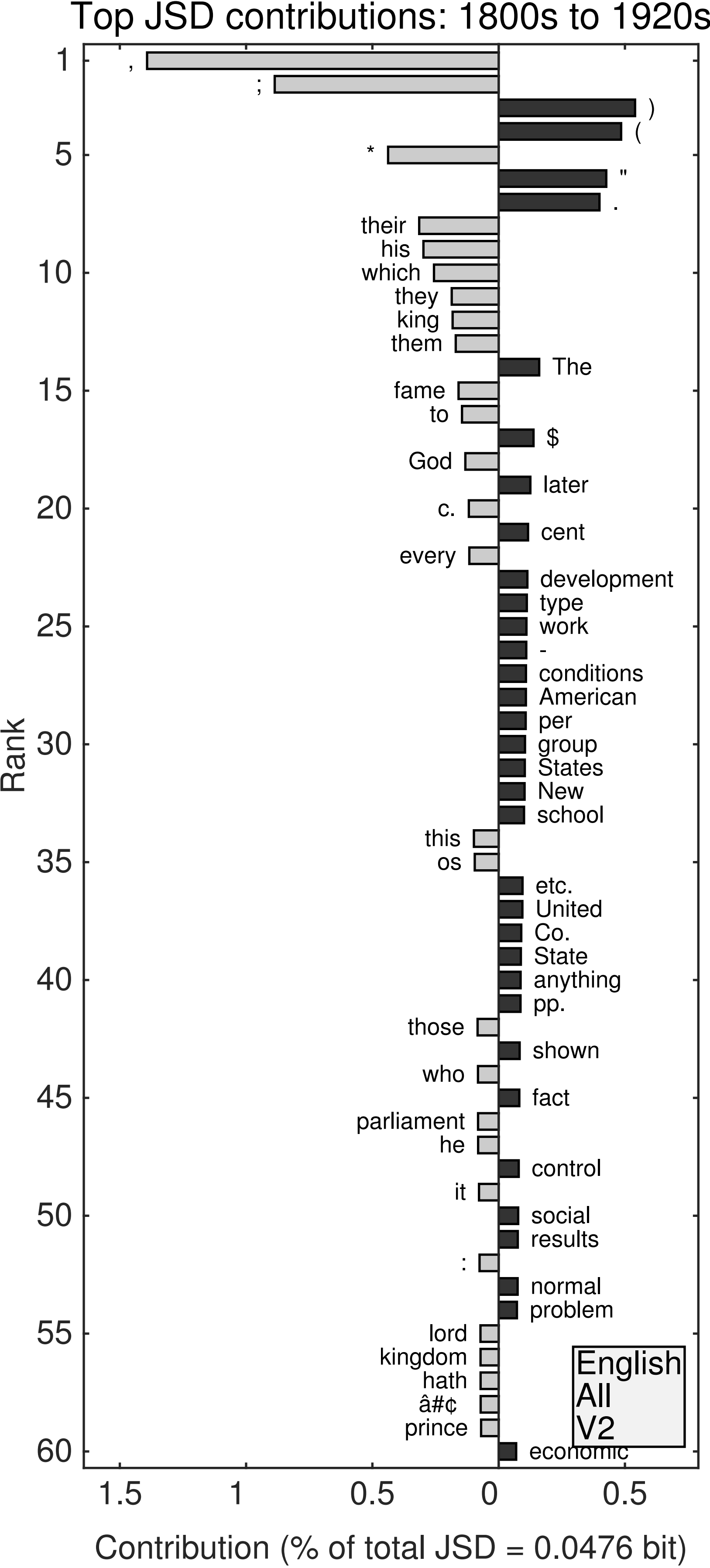

Top JSD contributions: 1800s to 1920s
Rank
Contribution (% of total JSD = 0.0476 bit)
1.5
1
0.5
0
0.5
1
5
10
15
20
25
30
35
40
45
50
55
60
,
;
)
(
*
"
.
their
his
which
they
king
them
The
fame
to
$
God
later
c.
cent
every
development
type
work
-
conditions
American
per
group
States
New
school
this
os
etc.
United
Co.
State
anything
pp.
those
shown
who
fact
parliament
he
control
it
social
results
:
normal
problem
lord
kingdom
hath
â#¢
prince
economic
English
All
V2

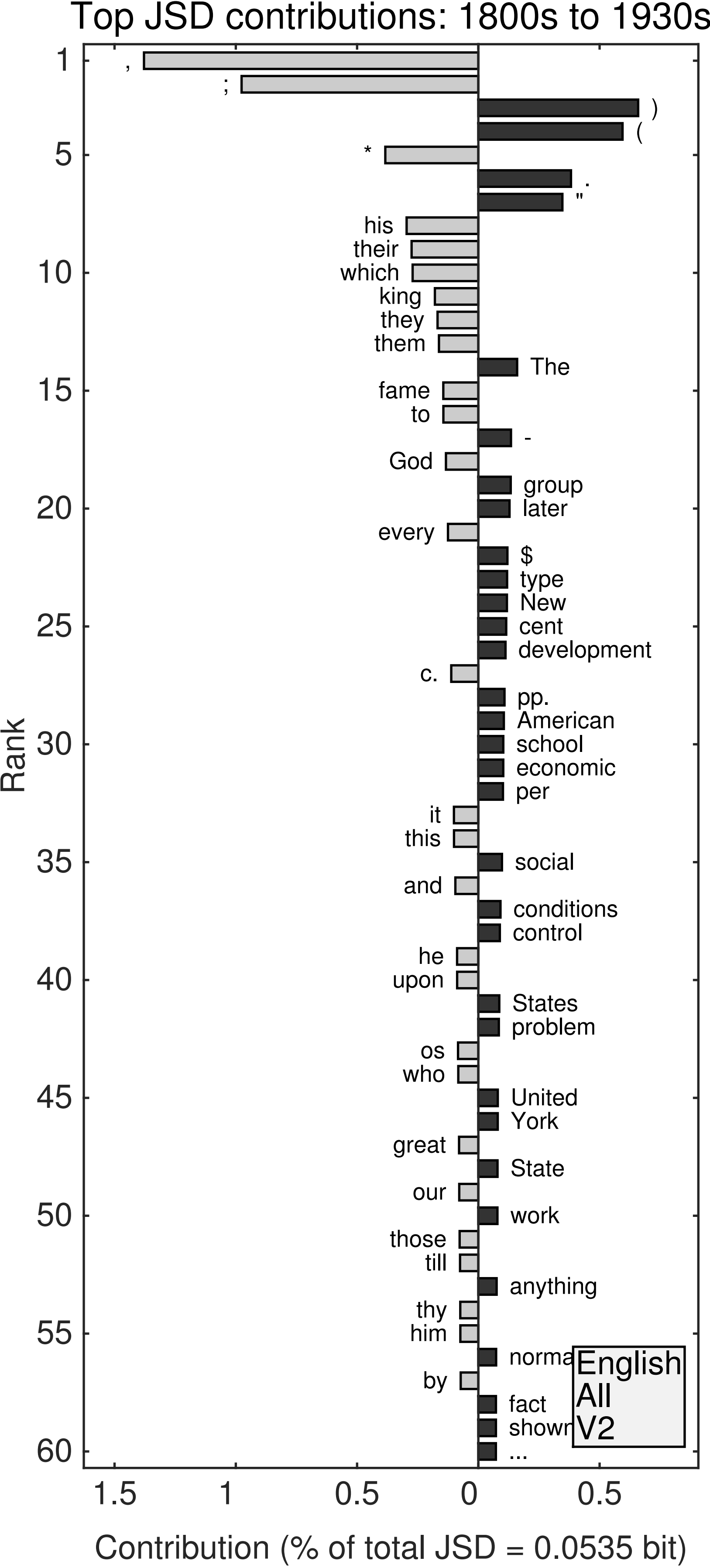
Top JSD contributions: 1800s to 1930s
Rank
Contribution (% of total JSD = 0.0535 bit)
1.5
1
0.5
0
0.5
1
5
10
15
20
25
30
35
40
45
50
55
60
,
;
)
(
*
.
"
his
their
which
king
they
them
The
fame
to
-
God
group
later
every
$
type
New
cent
development
c.
pp.
American
school
economic
per
it
this
social
and
conditions
control
he
upon
States
problem
os
who
United
York
great
State
our
work
those
till
anything
thy
him
by
fact
...
English
All
V2

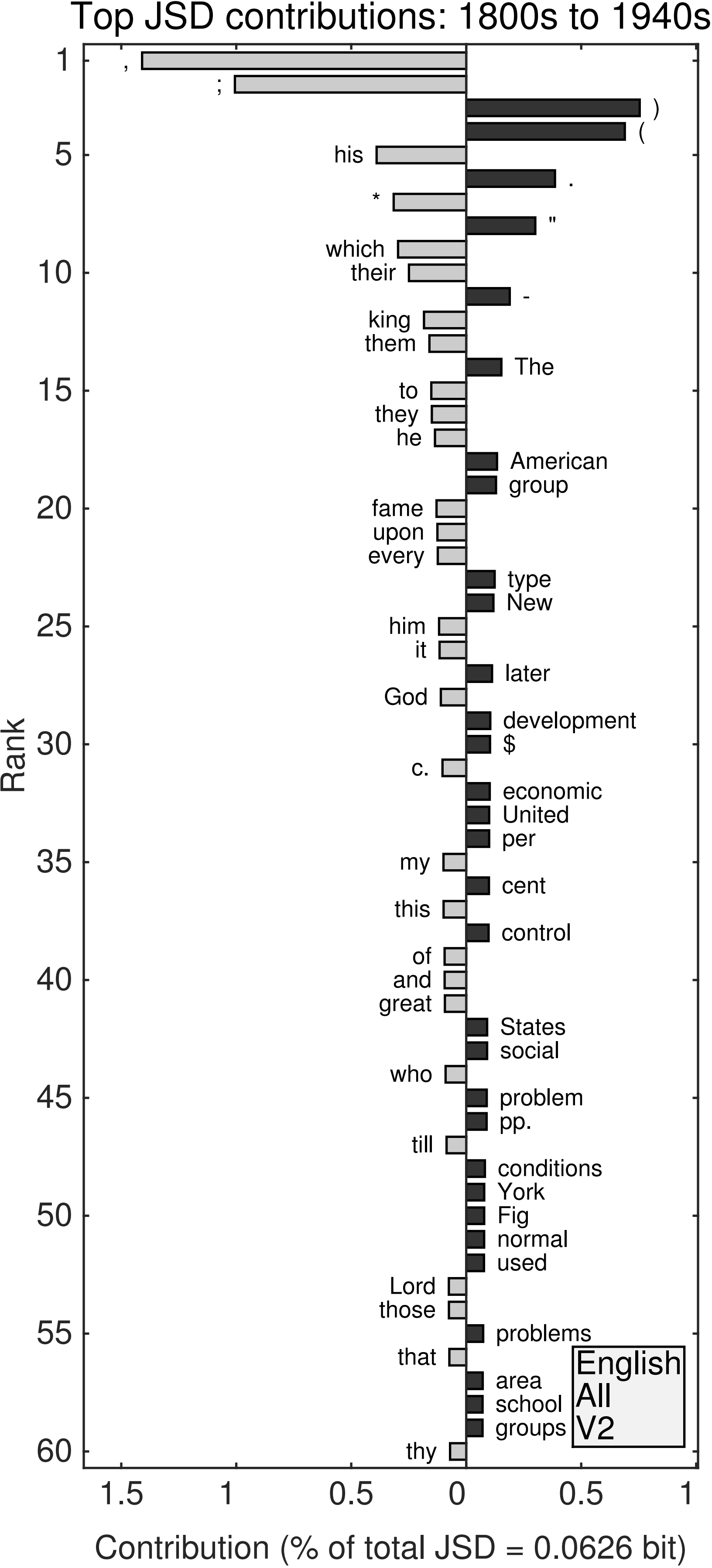
Top JSD contributions: 1800s to 1940s
Rank
Contribution (% of total JSD = 0.0626 bit)
,
;
)
(
his
.
*
"
which
their
-
king
them
The
to
they
he
American
group
fame
upon
every
type
New
him
it
later
God
development
$
c.
economic
United
per
my
cent
this
control
of
and
great
States
social
who
problem
pp.
till
conditions
York
Fig
normal
used
Lord
those
problems
that
area
school
groups
thy
English
All
V2

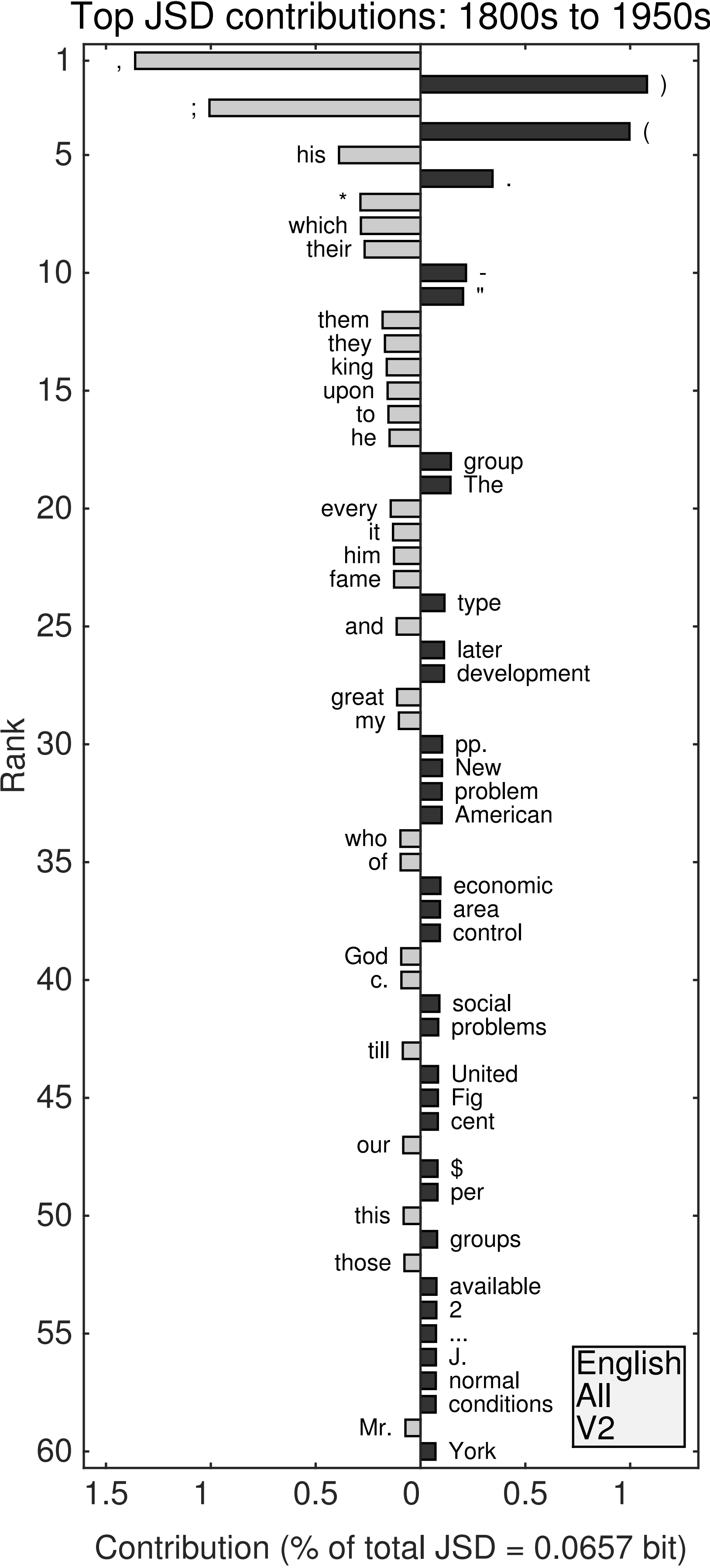

Top JSD contributions: 1800s to 1950s
Rank
Contribution (% of total JSD = 0.0657 bit)
English
All
V2
,) ; (his . * which their - " them they king upon to he group The every it him fame type and later development great my pp. New problem American who of economic area control God c. social problems till United Fig cent our $ per this groups those available 2 ... J. normal conditions Mr. York

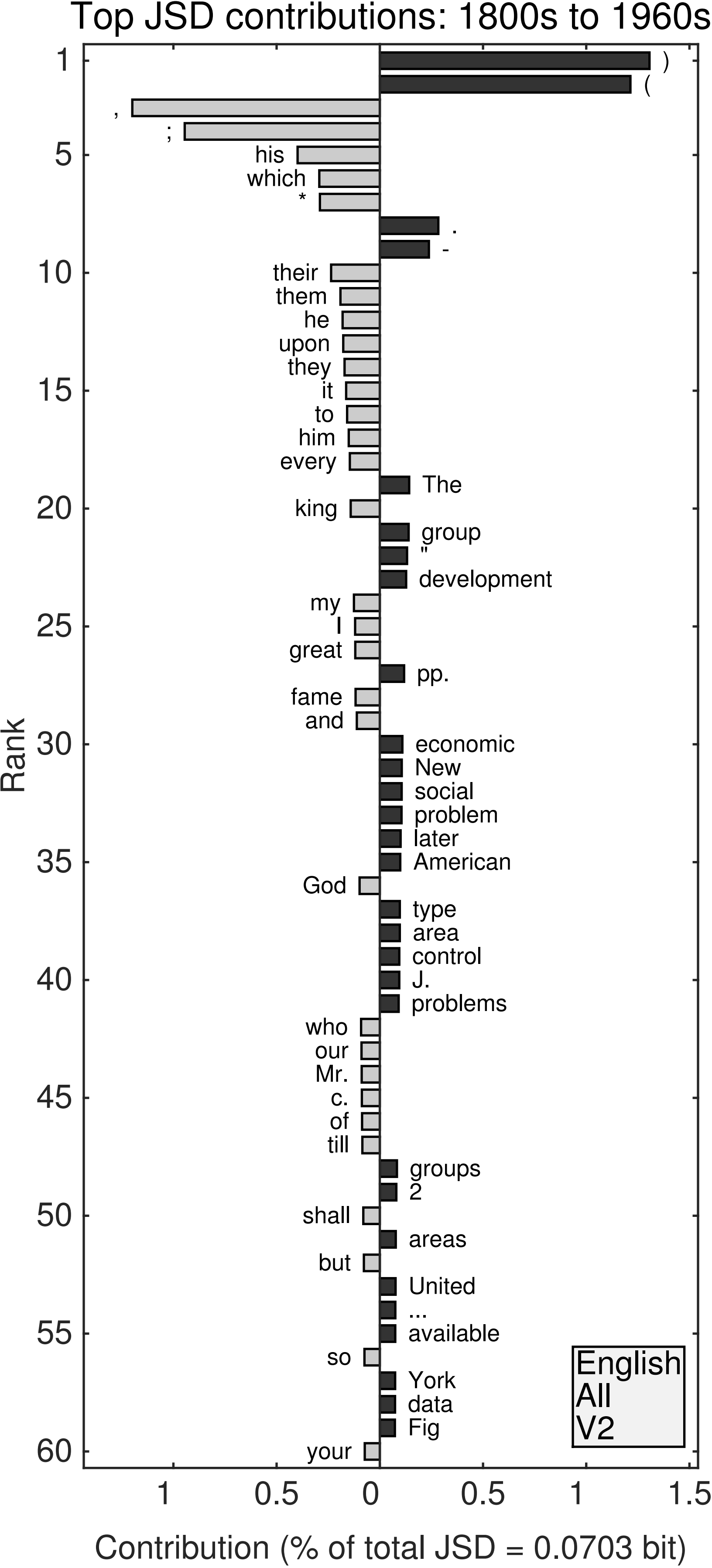

Top JSD contributions: 1800s to 1960s
Rank
Contribution (% of total JSD = 0.0703 bit)
1
5
10
15
20
25
30
35
40
45
50
55
60
1
0.5
0
0.5
1
1.5
)
(
,
;
his
which
*
.
-
their
them
he
upon
they
it
to
him
every
The
king
group
"
development
my
I
great
pp.
fame
and
economic
New
social
problem
later
American
God
type
area
control
J.
problems
who
our
Mr.
c.
of
till
groups
2
shall
areas
but
United
...
available
so
York
data
Fig
your
English
All
V2

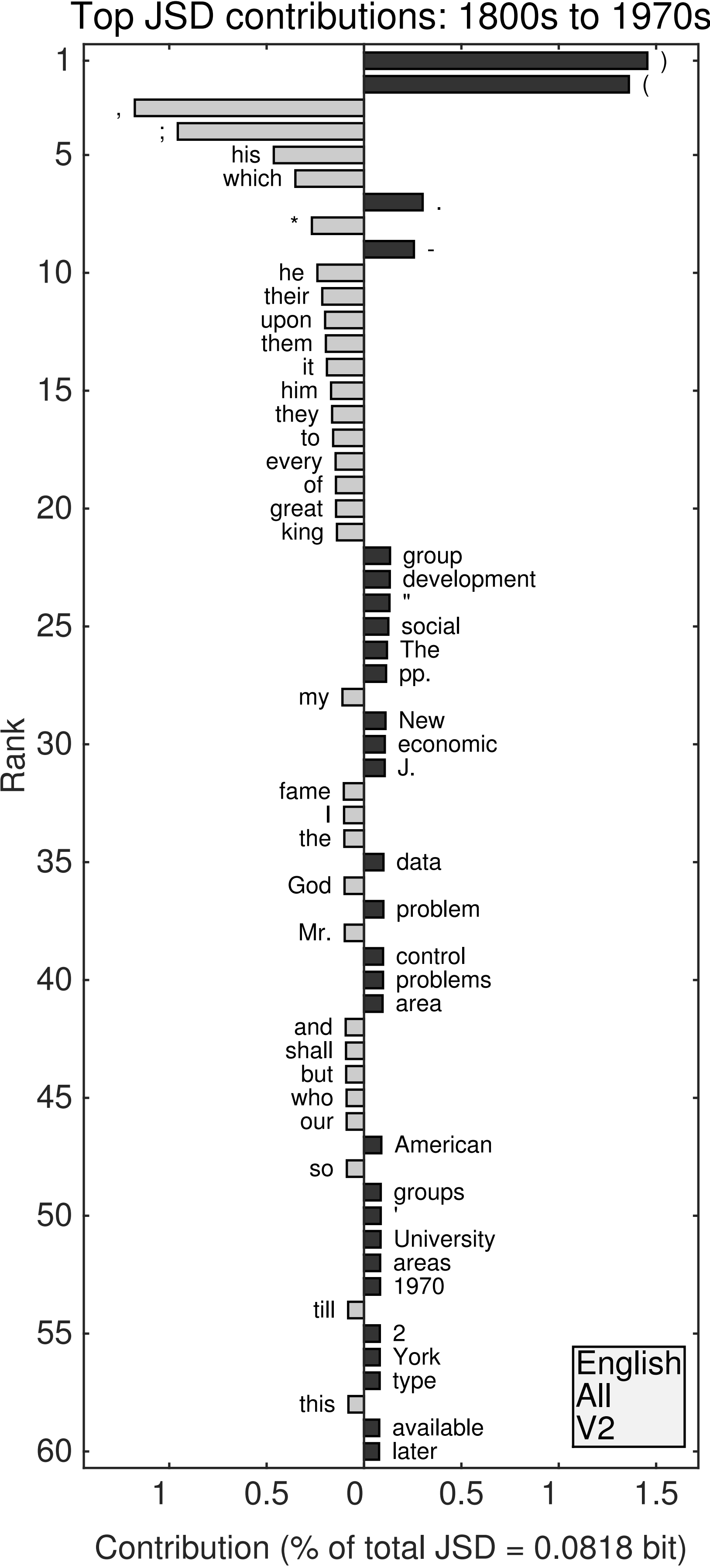

Top JSD contributions: 1800s to 1970s
Rank
Contribution (% of total JSD = 0.0818 bit)
1
5
10
15
20
25
30
35
40
45
50
55
60
1
0.5
0
0.5
1
1.5
)
(
,
;
his
which
.
*
-
he
their
upon
them
it
him
they
to
every
of
great
king
group
development
"
social
The
pp.
my
New
economic
J.
fame
I
the
data
God
problem
Mr.
control
problems
area
and
shall
but
who
our
American
so
groups
'
University
areas
1970
till
2
York
type
this
available
later
English
All
V2

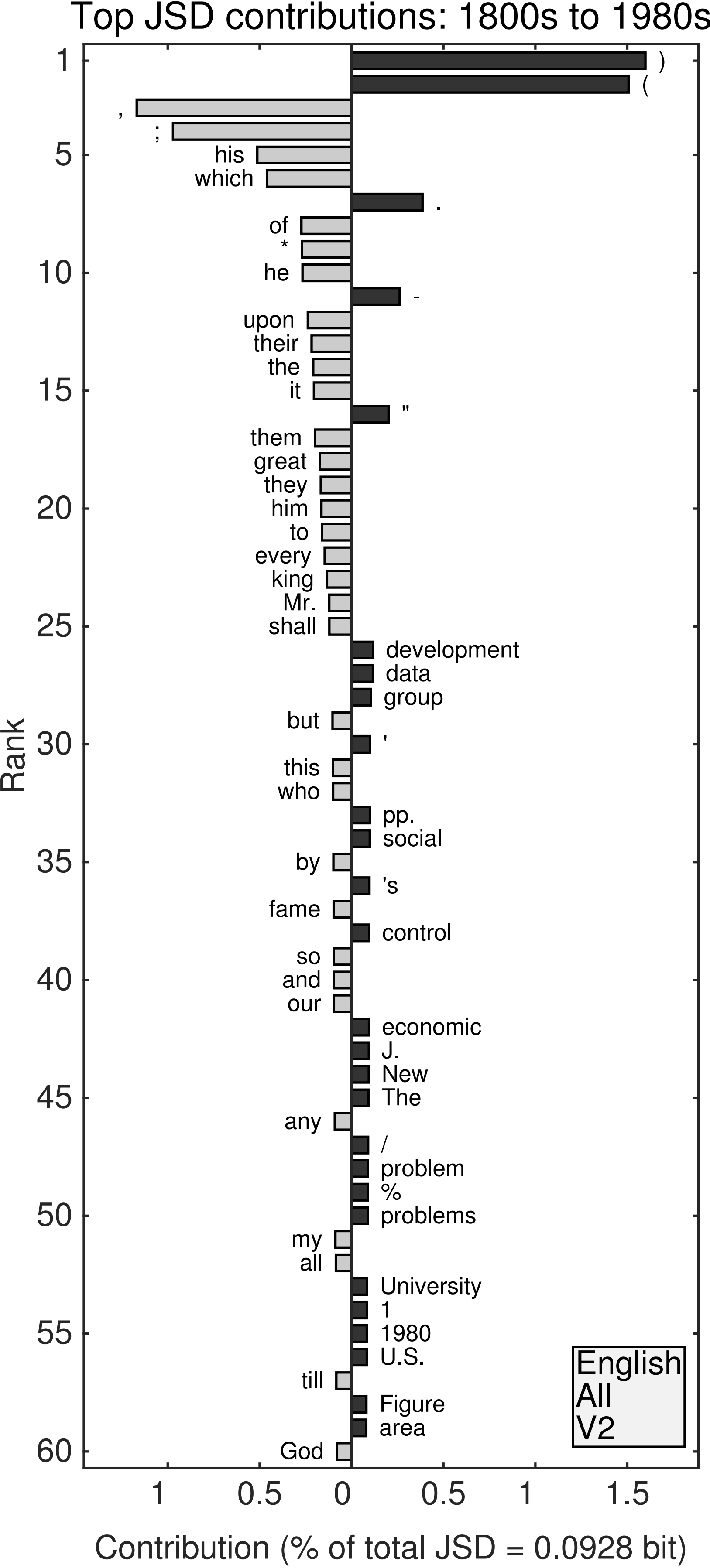

Top JSD contributions: 1800s to 1980s
Rank
Contribution (% of total JSD = 0.0928 bit)
)
(
,
;
his
which
.
of
*
he
-
upon
their
the
it
"
them
great
they
him
to
every
king
Mr.
shall
development
data
group
but
'
this
who
pp.
social
by
's
fame
control
so
and
our
economic
J.
New
The
any
/
problem
%
problems
my
all
University
1
1980
U.S.
till
Figure
area
God
English
All
V2

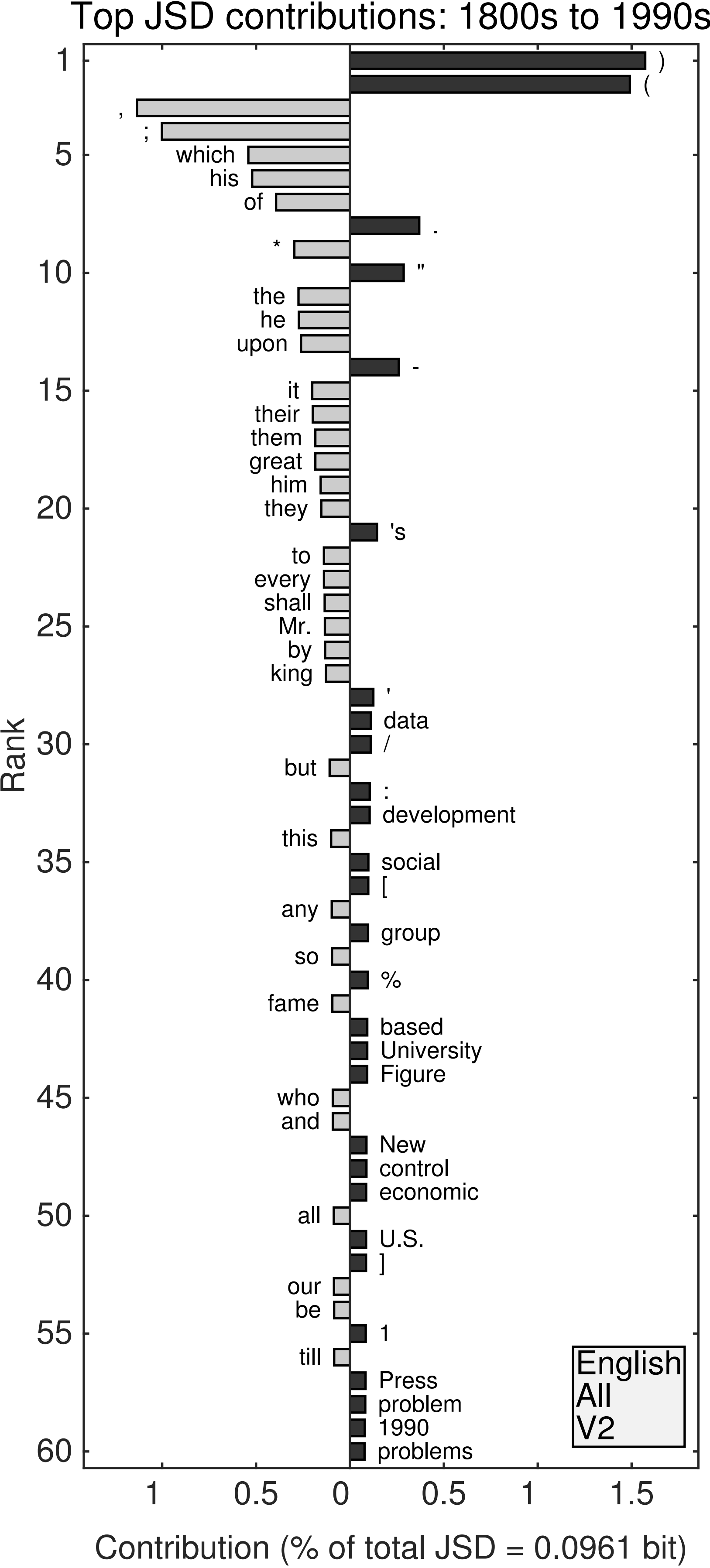
Top JSD contributions: 1800s to 1990s
Rank
Contribution (% of total JSD = 0.0961 bit)
English
All
V2
)
(
,
;
which
his
of
.
*
"
the
he
upon
-
it
their
them
great
him
they
's
to
every
shall
Mr.
by
king
'
data
/
but
:
development
this
social
[
any
group
so
%
fame
based
University
Figure
who
and
New
control
economic
all
U.S.
]
our
be
1
till
Press
problem
1990
problems

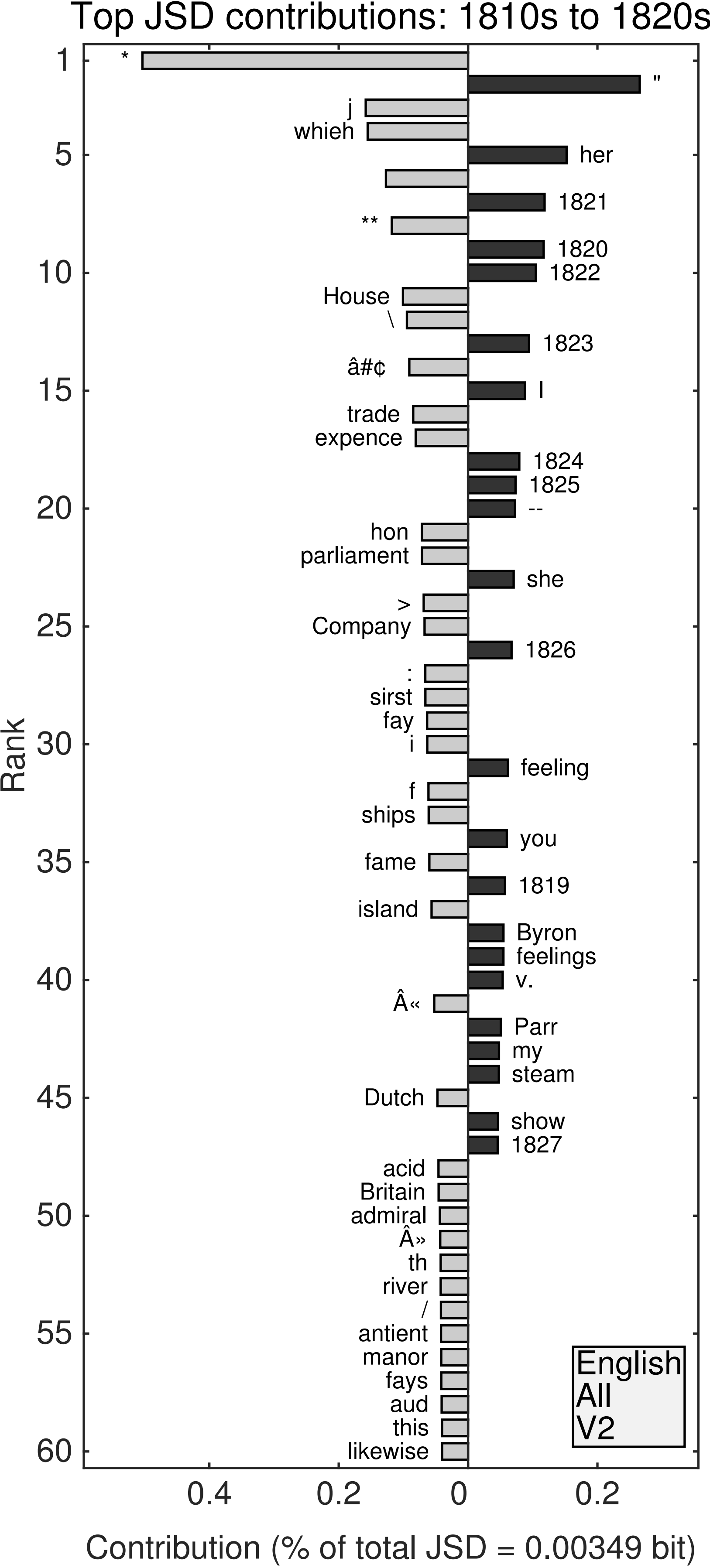

Top JSD contributions: 1810s to 1820s
Rank
1
5
10
15
20
25
30
35
40
45
50
55
60
*
"
j
whieh
her
1821
**
1820
1822
House
\
1823
â#¢
I
trade
expence
1824
1825
--
hon
parliament
she
>
Company
1826
:
sirst
fay
i
feeling
f
ships
you
fame
1819
island
Byron
feelings
v.
Â«
Parr
my
steam
Dutch
show
1827
acid
Britain
admiral
Â»
th
river
/
antient
manor
fays
aud
this
likewise
English
All
V2
0.4
0.2
0
0.2
Contribution (% of total JSD = 0.00349 bit)

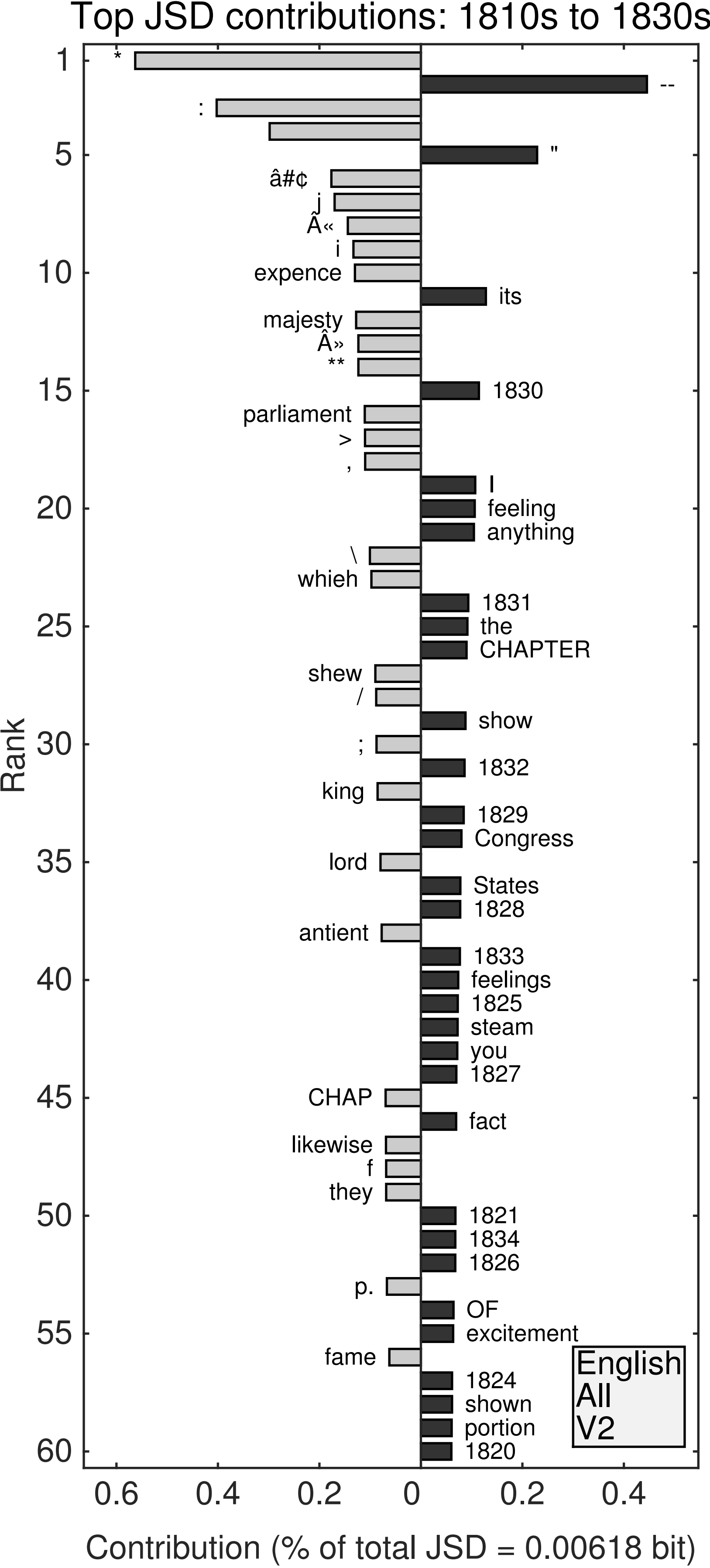

Top JSD contributions: 1810s to 1830s
Rank
Contribution (% of total JSD = 0.00618 bit)
*
--
:
"
â#¢
j
Â«
i
expence
its
majesty
Â»
**
1830
parliament
>
,
I
feeling
anything
\
whieh
1831
the
CHAPTER
shew
/
show
;
1832
king
1829
Congress
lord
States
1828
antient
1833
feelings
1825
steam
you
1827
CHAP
fact
likewise
f
they
1821
1834
1826
p.
OF
excitement
fame
1824
shown
portion
1820
English
All
V2

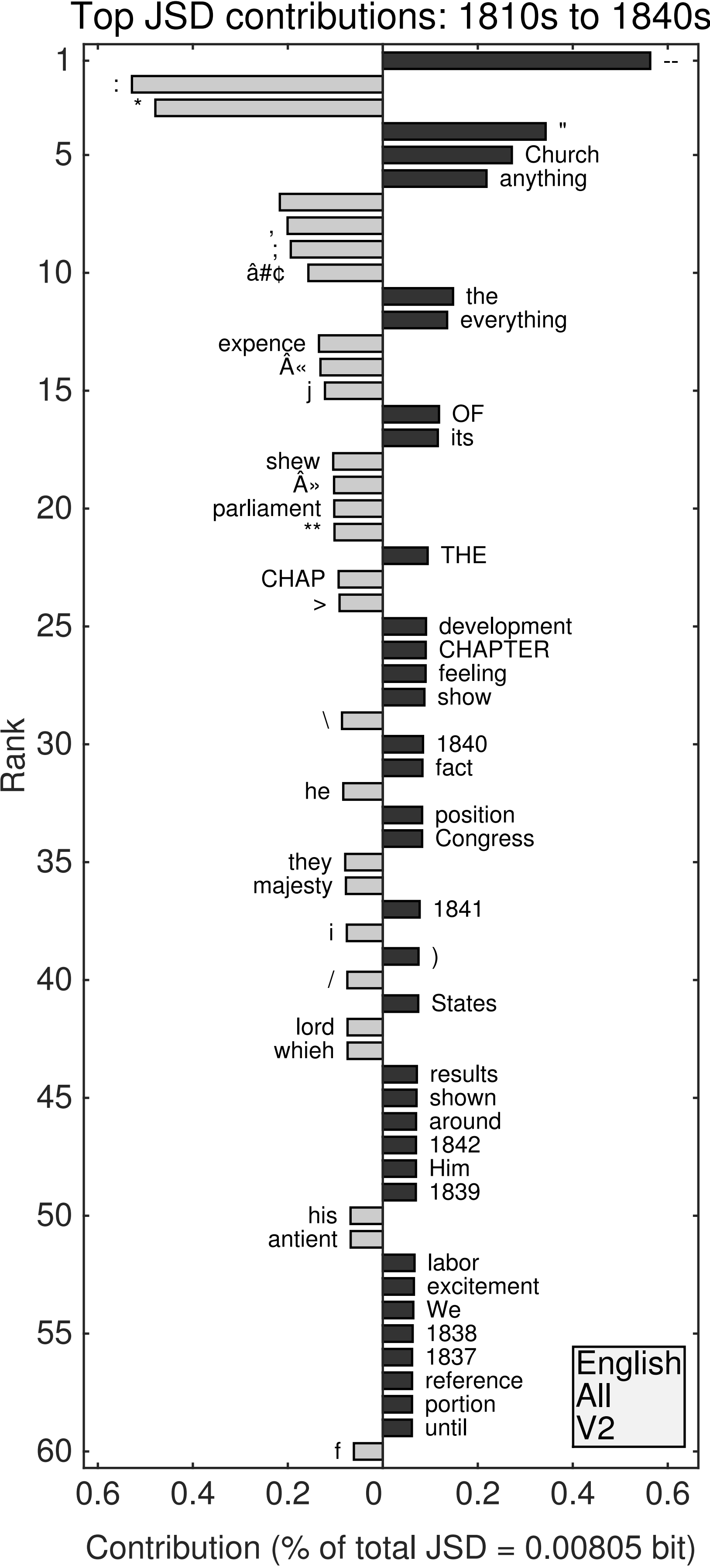

Top JSD contributions: 1810s to 1840s
Rank
Contribution (% of total JSD = 0.00805 bit)
1
5
10
15
20
25
30
35
40
45
50
55
60
0.6
0.4
0.2
0
0.2
0.4
0.6
--
:
*
"
Church
anything
,
;
â#¢
the
everything
expence
Â«
j
OF
its
shew
Â»
parliament
**
THE
CHAP
>
development
CHAPTER
feeling
show
\
1840
fact
he
position
Congress
they
majesty
1841
i
)
/
States
lord
whieh
results
shown
around
1842
Him
1839
his
antient
labor
excitement
We
1838
1837
reference
portion
until
f
English
All
V2

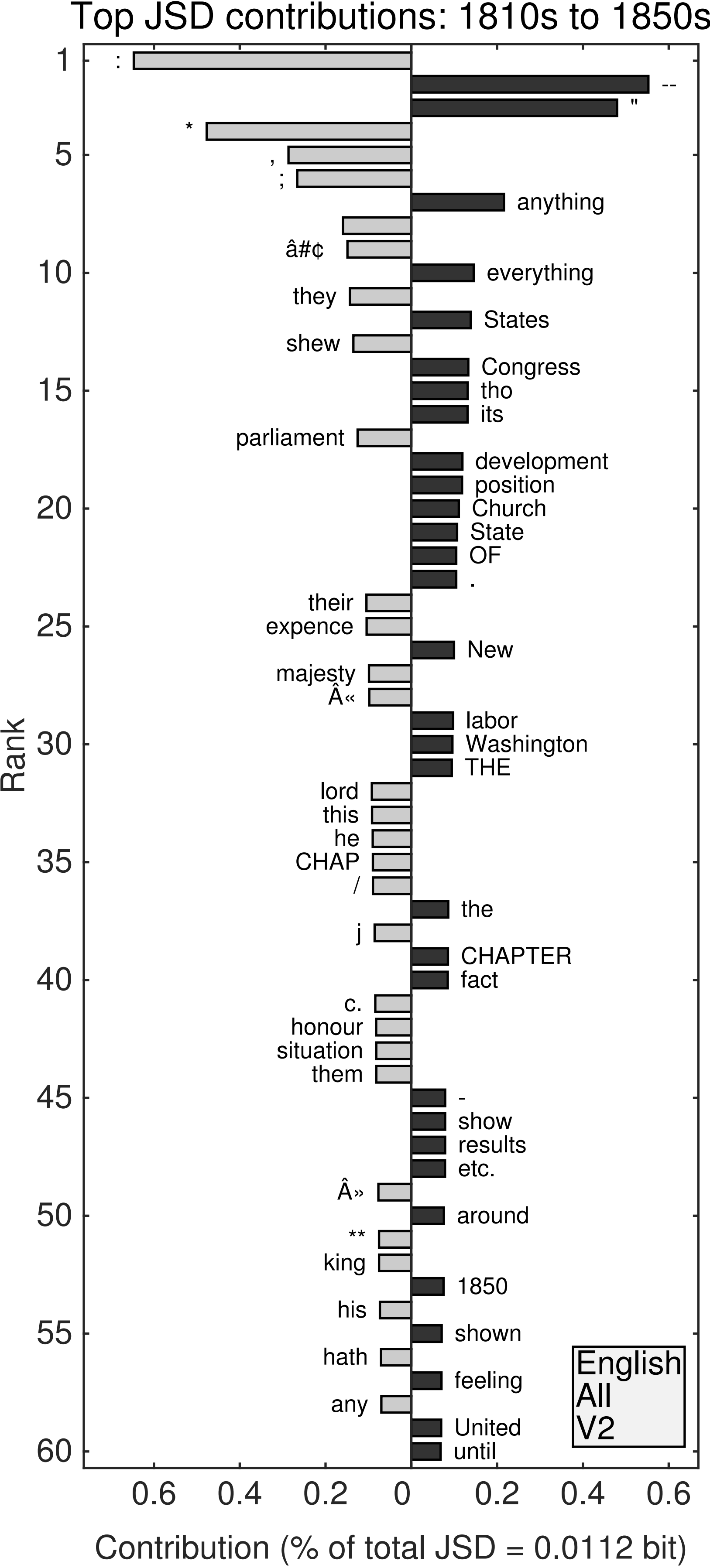

Top JSD contributions: 1810s to 1850s
Rank
:
--
"
*
,
;
anything
â#¢
everything
they
States
shew
Congress
tho
its
parliament
development
position
Church
State
OF
.
their
expence
New
majesty
Â«
labor
Washington
THE
lord
this
he
CHAP
/
the
j
CHAPTER
fact
c.
honour
situation
them
-
show
results
etc.
Â»
around
**
king
1850
his
shown
hath
feeling
any
United
until
English
All
V2
Contribution (% of total JSD = 0.0112 bit)

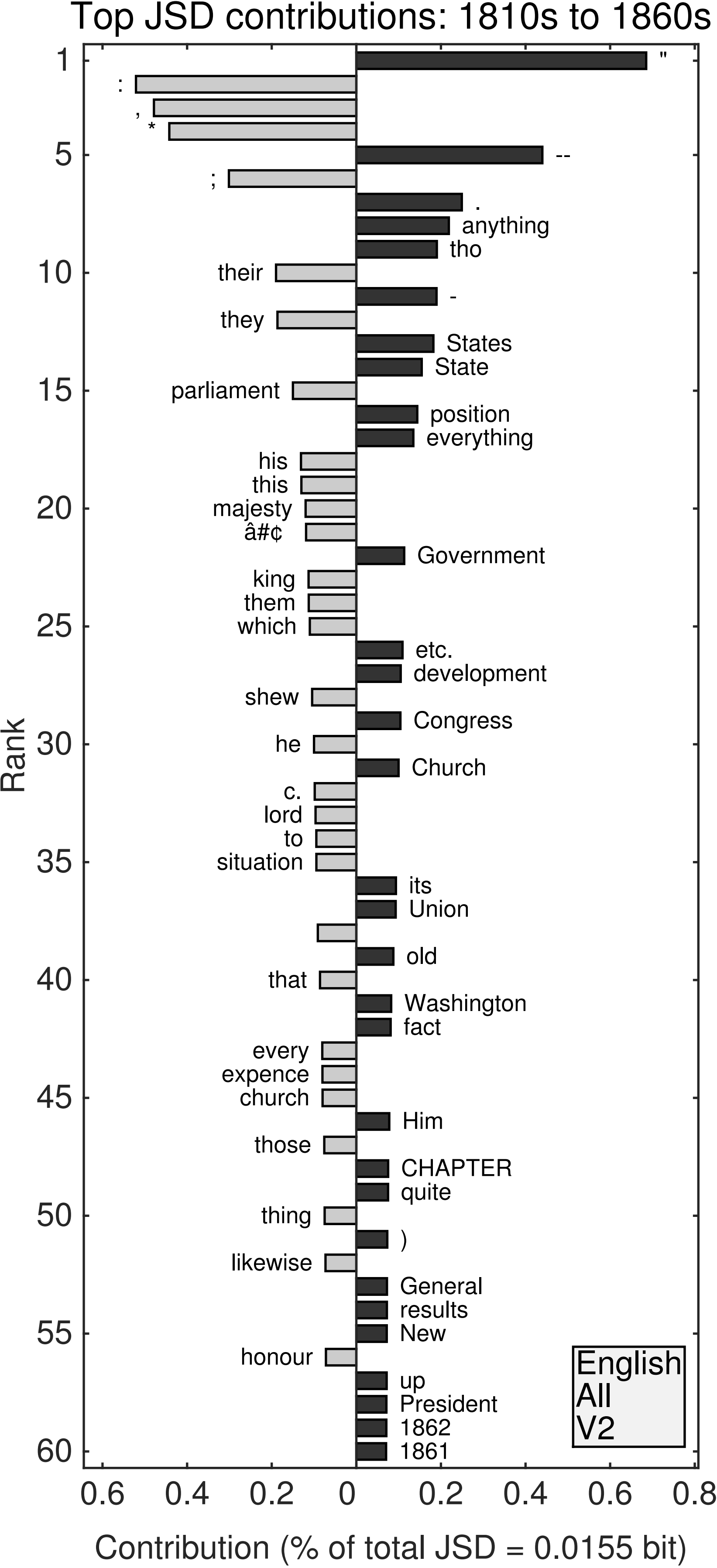

Top JSD contributions: 1810s to 1860s
Rank
Contribution (% of total JSD = 0.0155 bit)
"
:
,
*
--
;
.
anything
tho
their
-
they
States
State
parliament
position
everything
his
this
majesty
â#¢
Government
king
them
which
etc.
development
shew
Congress
he
Church
c.
lord
to
situation
its
Union
old
that
Washington
fact
every
expence
church
Him
those
CHAPTER
quite
thing
)
likewise
General
results
New
honour
up
President
1862
1861
English
All
V2

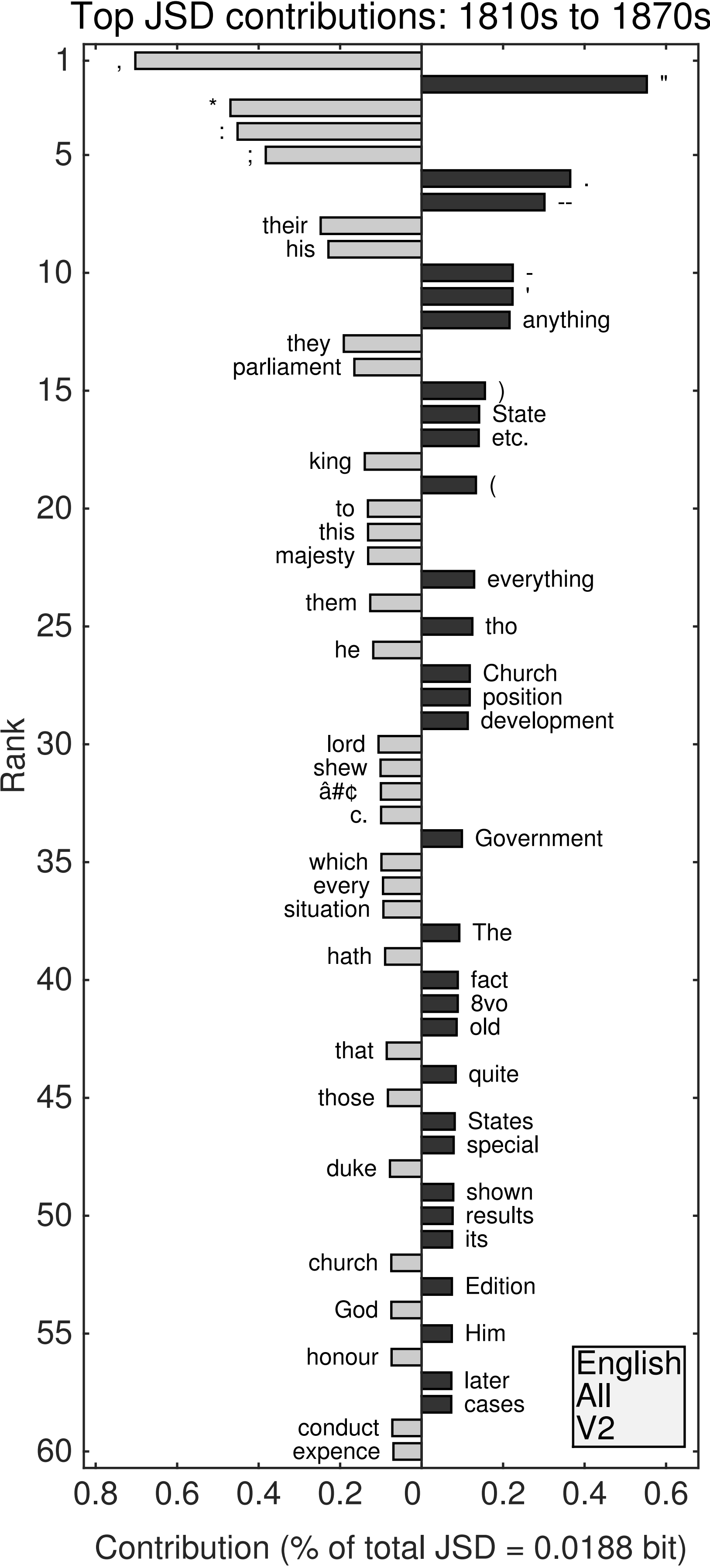

Top JSD contributions: 1810s to 1870s
Rank
Contribution (% of total JSD = 0.0188 bit)
,
"
*
:
;
.
--
their
his
-
'
anything
they
parliament
)
State
etc.
king
(
to
this
majesty
everything
them
tho
he
Church
position
development
lord
shew
â#¢
c.
Government
which
every
situation
The
hath
fact
8vo
old
that
quite
those
States
special
duke
shown
results
its
church
Edition
God
Him
honour
later
cases
conduct
expence
English
All
V2

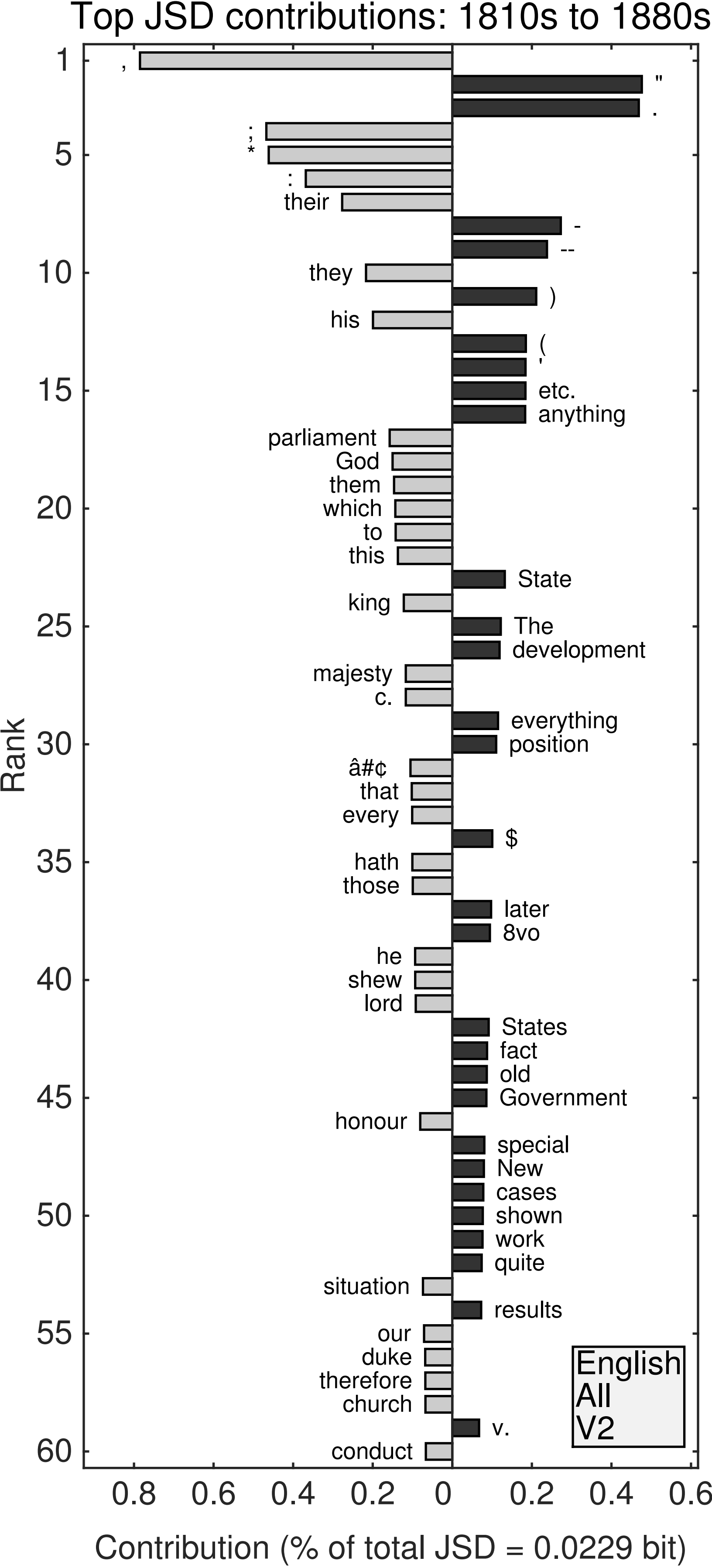

Top JSD contributions: 1810s to 1880s
Rank
Contribution (% of total JSD = 0.0229 bit)
0.8 0.6 0.4 0.2 0 0.2 0.4 0.6
1 5 10 15 20 25 30 35 40 45 50 55 60
,
"
.
;
*
:
their
-
--
they
)
his
(
'
etc.
anything
parliament
God
them
which
to
this
State
king
The
development
majesty
c.
everything
position
â#¢
that
every
$
hath
those
later
8vo
he
shew
lord
States
fact
old
Government
honour
special
New
cases
shown
work
quite
situation
results
our
duke
therefore
church
v.
conduct
English
All
V2

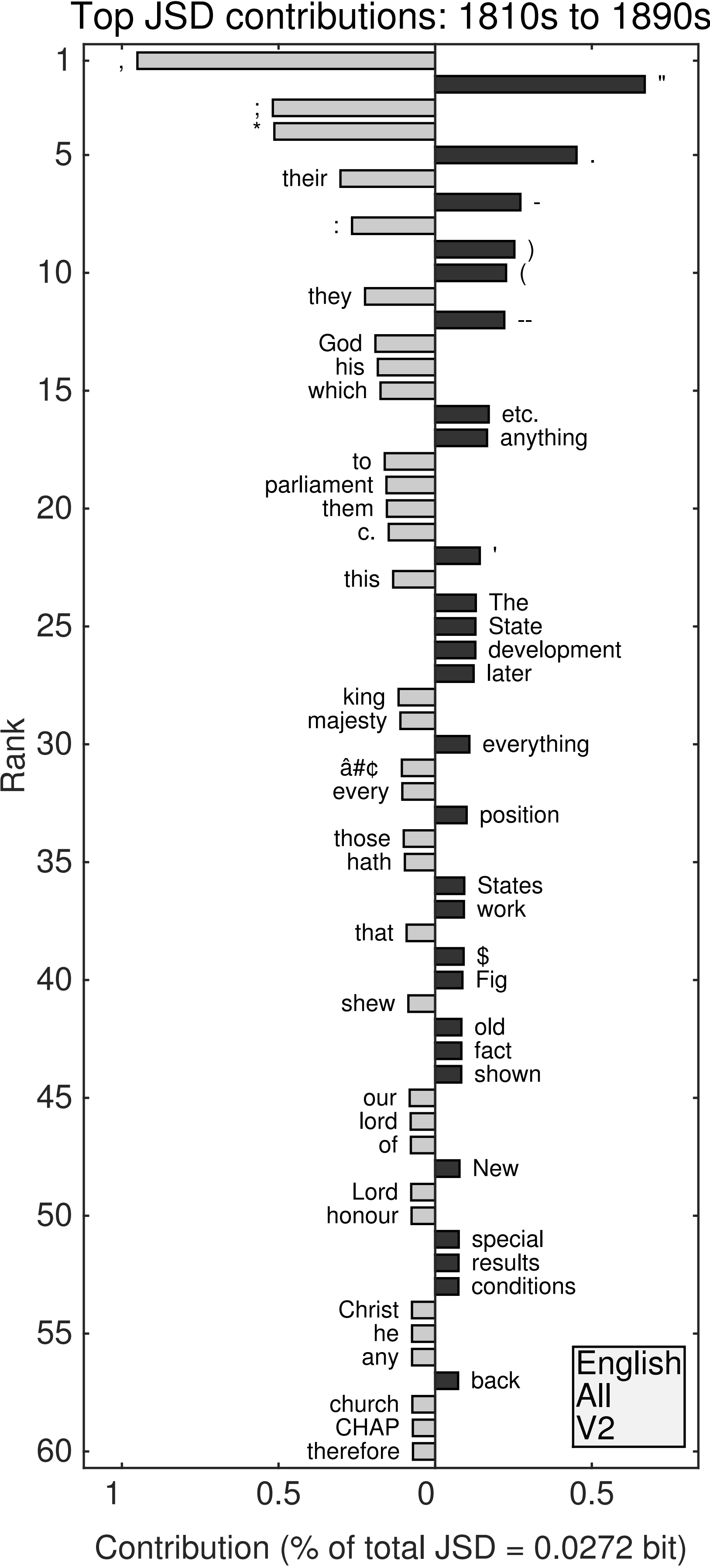

Top JSD contributions: 1810s to 1890s
Rank
,
"
;
*
.
their
-
:
)
(
they
--
God
his
which
etc.
anything
to
parliament
them
c.
'
this
The
State
development
later
king
majesty
everything
â#¢
every
position
those
hath
States
work
that
$
Fig
shew
old
fact
shown
our
lord
of
New
Lord
honour
special
results
conditions
Christ
he
any
back
church
CHAP
therefore
English
All
V2
1 5 10 15 20 25 30 35 40 45 50 55 60
1 0.5 0 0.5
Contribution (% of total JSD = 0.0272 bit)

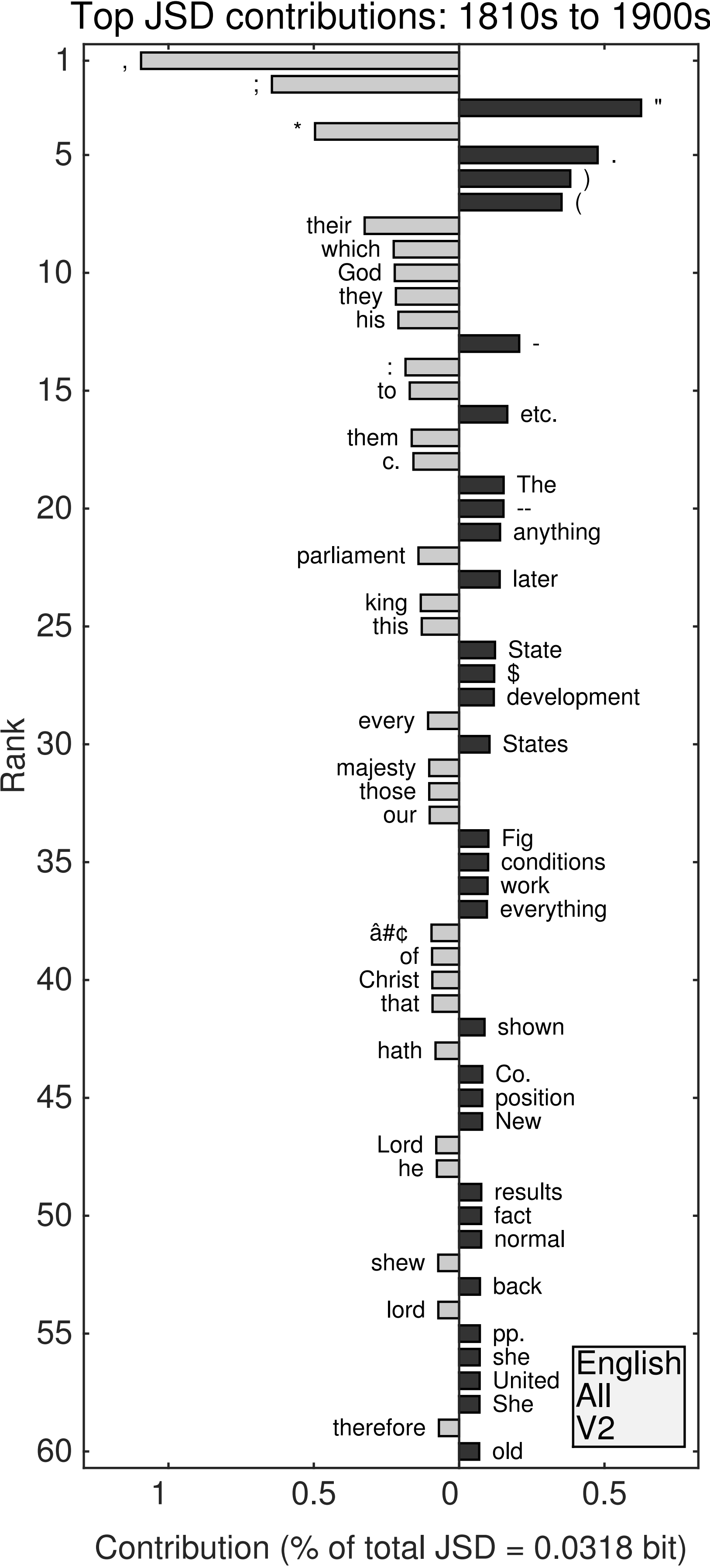

Top JSD contributions: 1810s to 1900s
Rank
Contribution (% of total JSD = 0.0318 bit)
1
5
10
15
20
25
30
35
40
45
50
55
60
1
0.5
0
0.5
,
;
"
*
.
)
(
their
which
God
they
his
-
:
to
etc.
them
c.
The
--
anything
parliament
later
king
this
State
$
development
every
States
majesty
those
our
Fig
conditions
work
everything
â#¢
of
Christ
that
shown
hath
Co.
position
New
Lord
he
results
fact
normal
shew
back
lord
pp.
she
United
She
therefore
old
English
All
V2

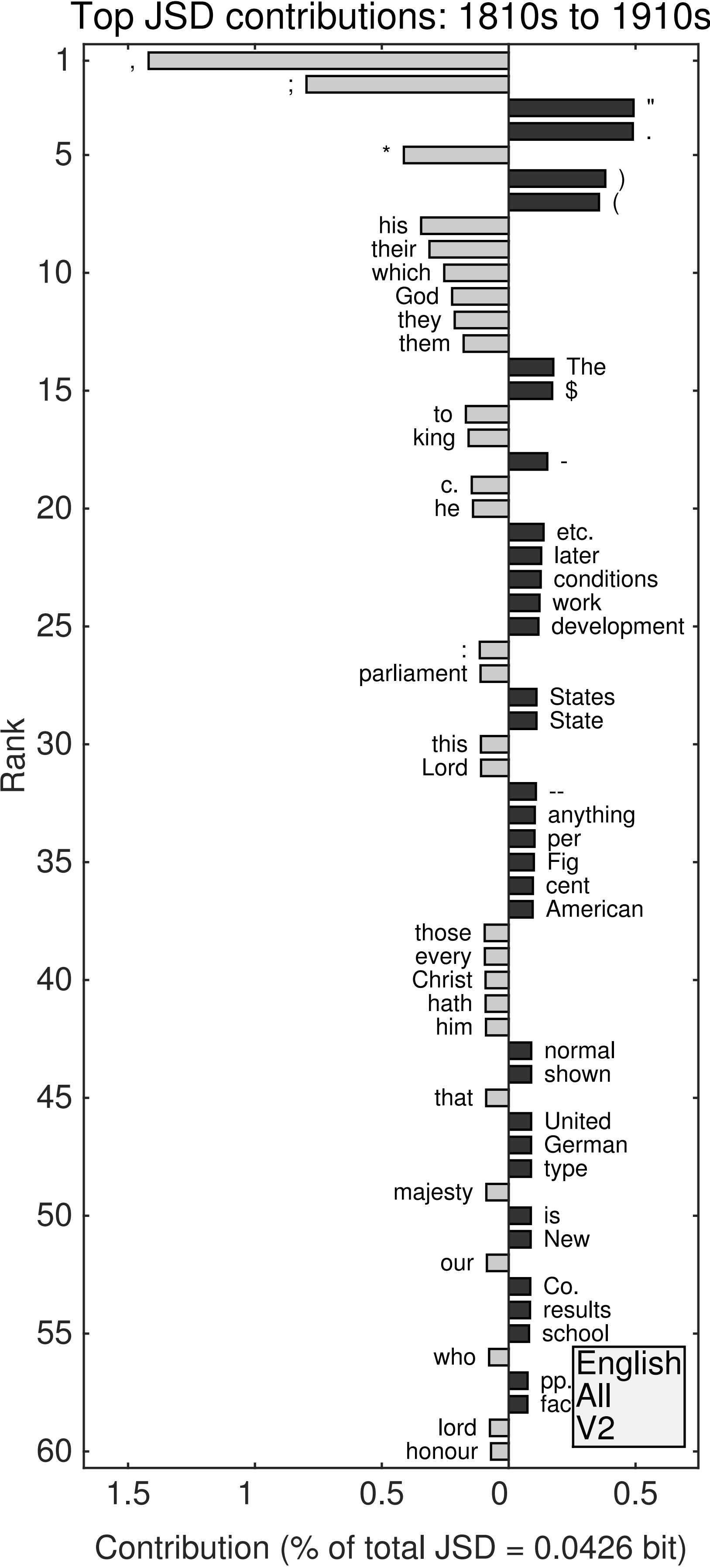

Top JSD contributions: 1810s to 1910s
Rank
1
5
10
15
20
25
30
35
40
45
50
55
60
,
;
"
.
*
)
(
his
their
which
God
they
them
The
$
to
king
-
c.
he
etc.
later
conditions
work
development
:
parliament
States
State
this
Lord
--
anything
per
Fig
cent
American
those
every
Christ
hath
him
normal
shown
that
United
German
type
majesty
is
New
our
Co.
results
school
who
pp.
fac
lord
honour
English
All
V2
1.5
1
0.5
0
0.5
Contribution (% of total JSD = 0.0426 bit)

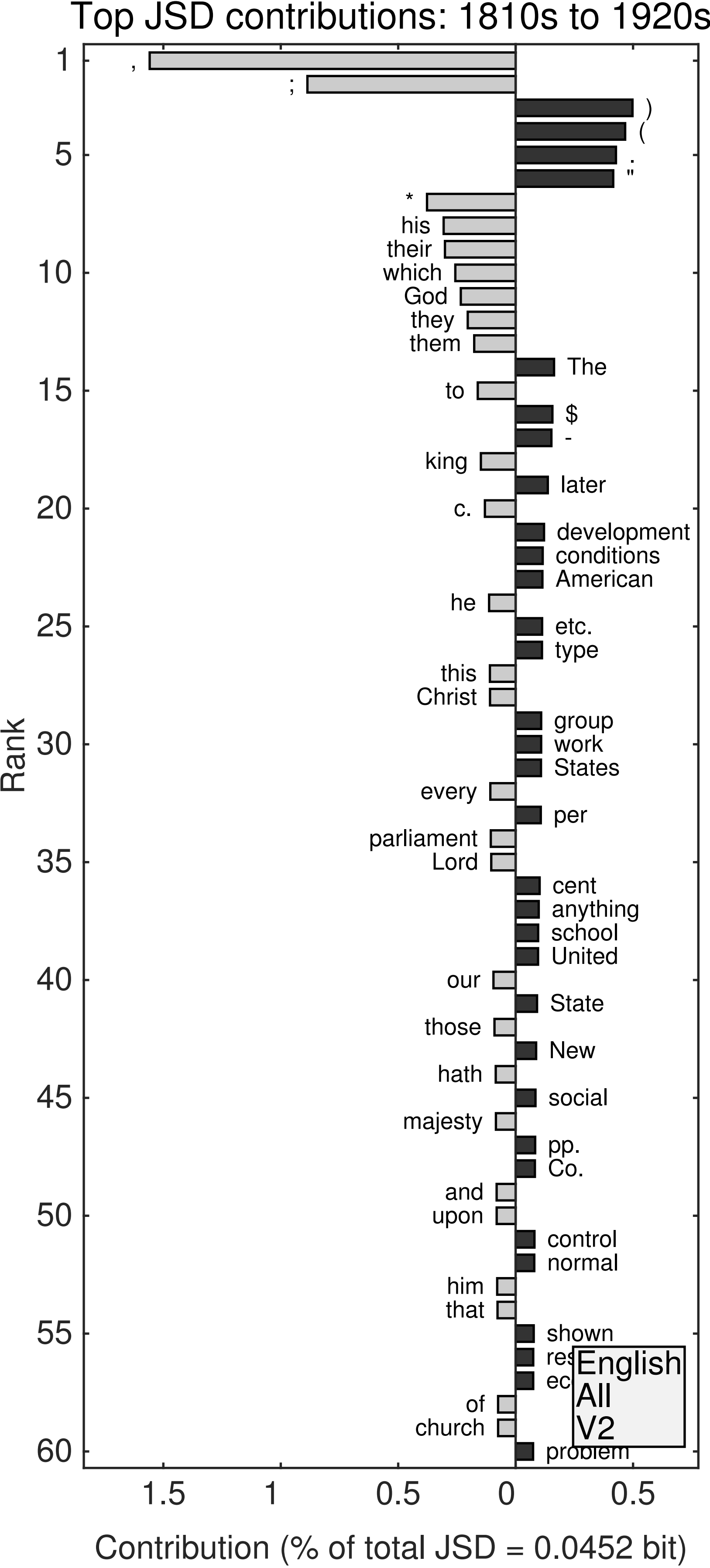

Top JSD contributions: 1810s to 1920s
Rank
Contribution (% of total JSD = 0.0452 bit)
English
All
V2

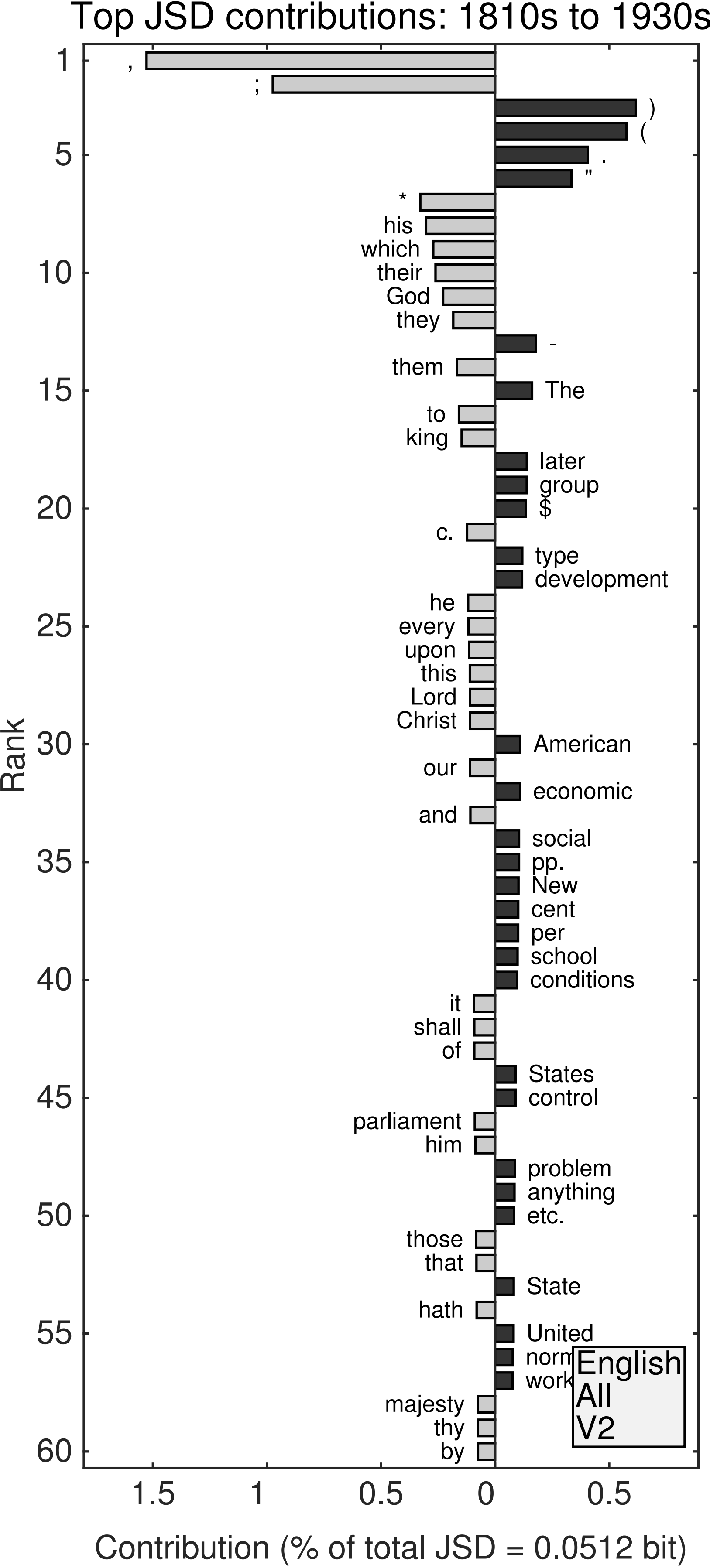
Top JSD contributions: 1810s to 1930s
Rank
Contribution (% of total JSD = 0.0512 bit)
English
All
V2
,
;
)
(
.
"
*
his
which
their
God
they
-
them
The
to
king
later
group
$
c.
type
development
he
every
upon
this
Lord
Christ
American
our
economic
and
social
pp.
New
cent
per
school
conditions
it
shall
of
States
control
parliament
him
problem
anything
etc.
those
that
State
hath
United
majesty
thy
by

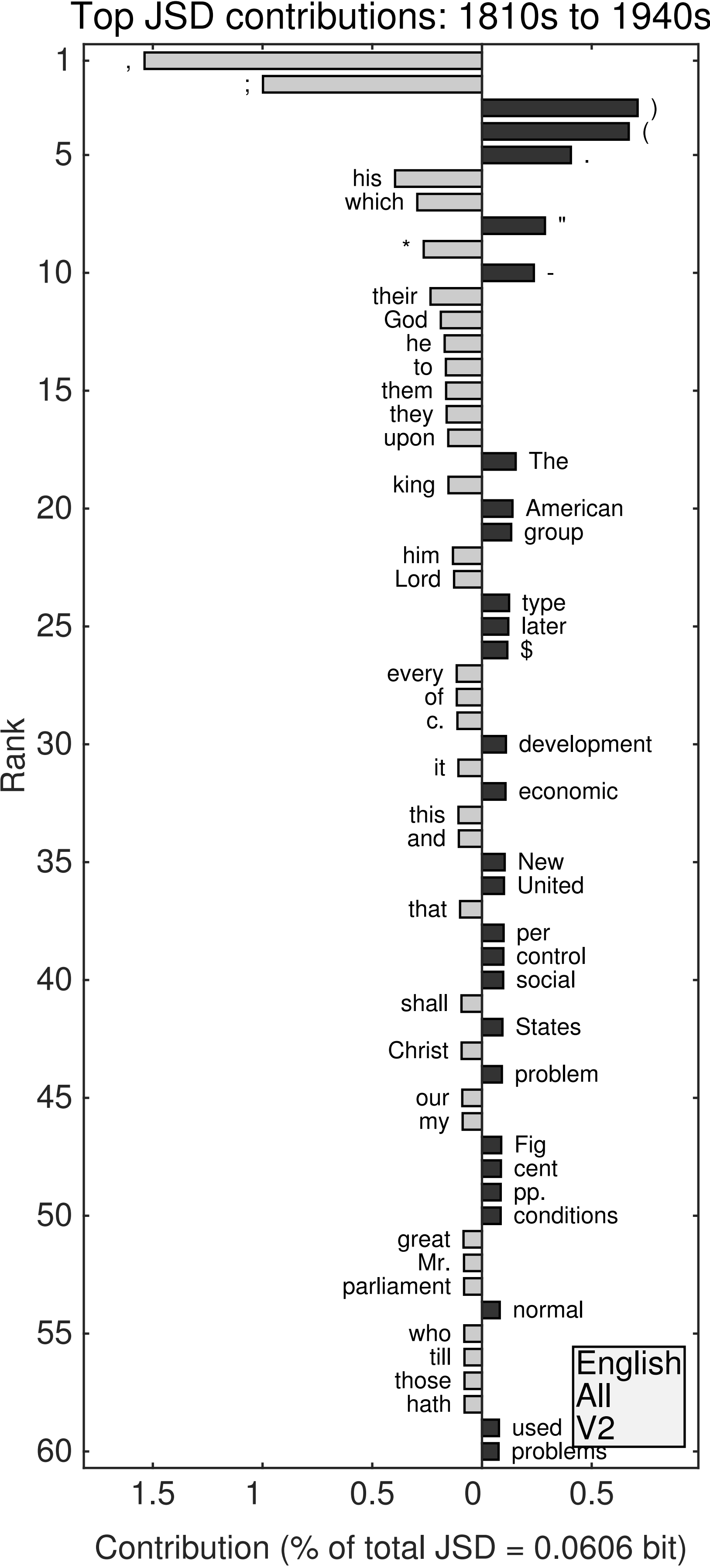
Top JSD contributions: 1810s to 1940s
Rank
1
5
10
15
20
25
30
35
40
45
50
55
60
,
;
)
(
.
his
which
"
*
-
their
God
he
to
them
they
upon
The
king
American
group
him
Lord
type
later
$
every
of
c.
development
it
economic
this
and
New
United
that
per
control
social
shall
States
Christ
problem
our
my
Fig
cent
pp.
conditions
great
Mr.
parliament
normal
who
till
those
hath
used
problems
English
All
V2
1.5
1
0.5
0
0.5
Contribution (% of total JSD = 0.0606 bit)

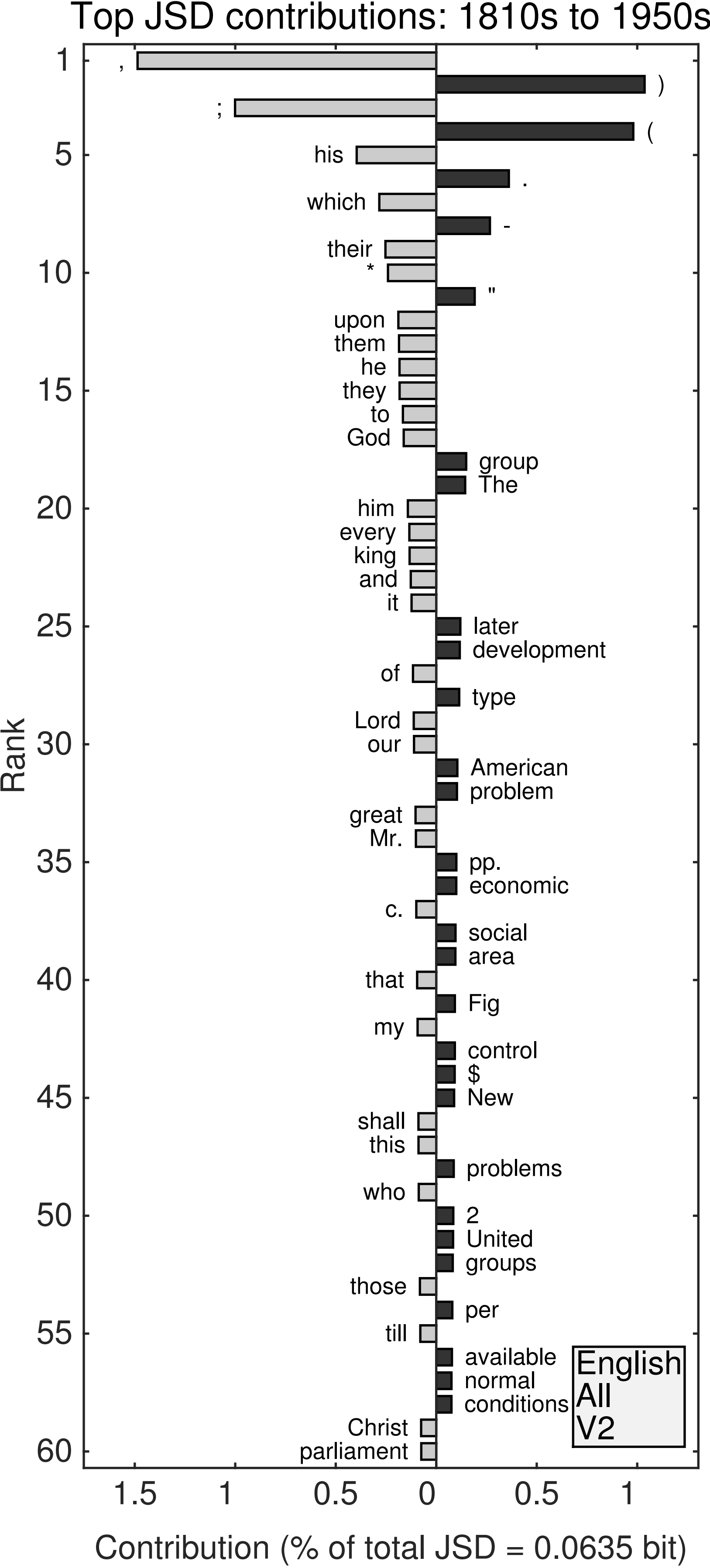
Top JSD contributions: 1810s to 1950s
Rank
1
5
10
15
20
25
30
35
40
45
50
55
60
,
)
;
(
his
.
which
-
their
*
"
upon
them
he
they
to
God
group
The
him
every
king
and
it
later
development
of
type
Lord
our
American
problem
great
Mr.
pp.
economic
c.
social
area
that
Fig
my
control
$
New
shall
this
problems
who
2
United
groups
those
per
till
available
normal
conditions
Christ
parliament
English
All
V2
1.5
1
0.5
0
0.5
1
Contribution (% of total JSD = 0.0635 bit)

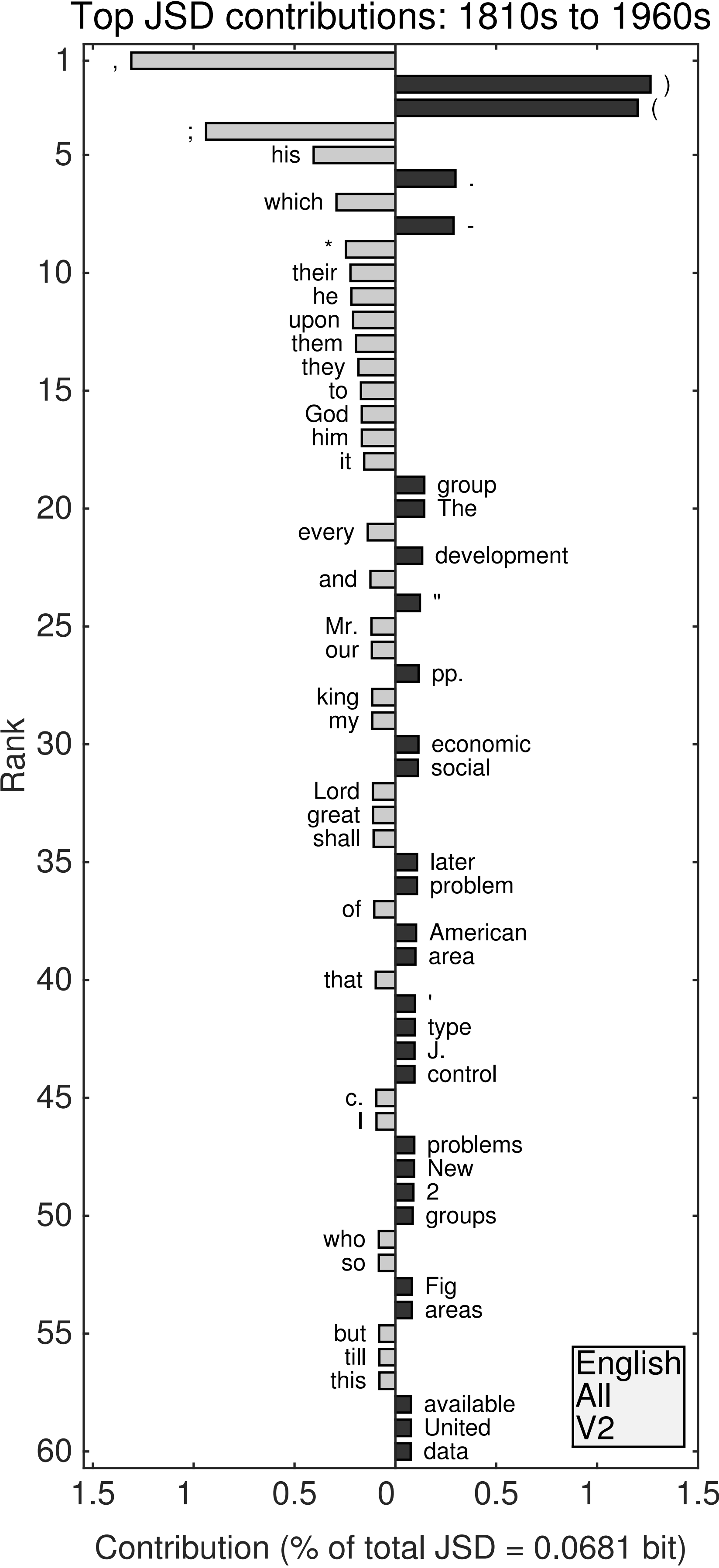
Top JSD contributions: 1810s to 1960s
Rank
Contribution (% of total JSD = 0.0681 bit)
,
)
(
;
his
.
which
-
*
their
he
upon
them
they
to
God
him
it
group
The
every
development
and
"
Mr.
our
pp.
king
my
economic
social
Lord
great
shall
later
problem
of
American
area
that
'
type
J.
control
c.
I
problems
New
2
groups
who
so
Fig
areas
but
till
this
available
United
data
English
All
V2

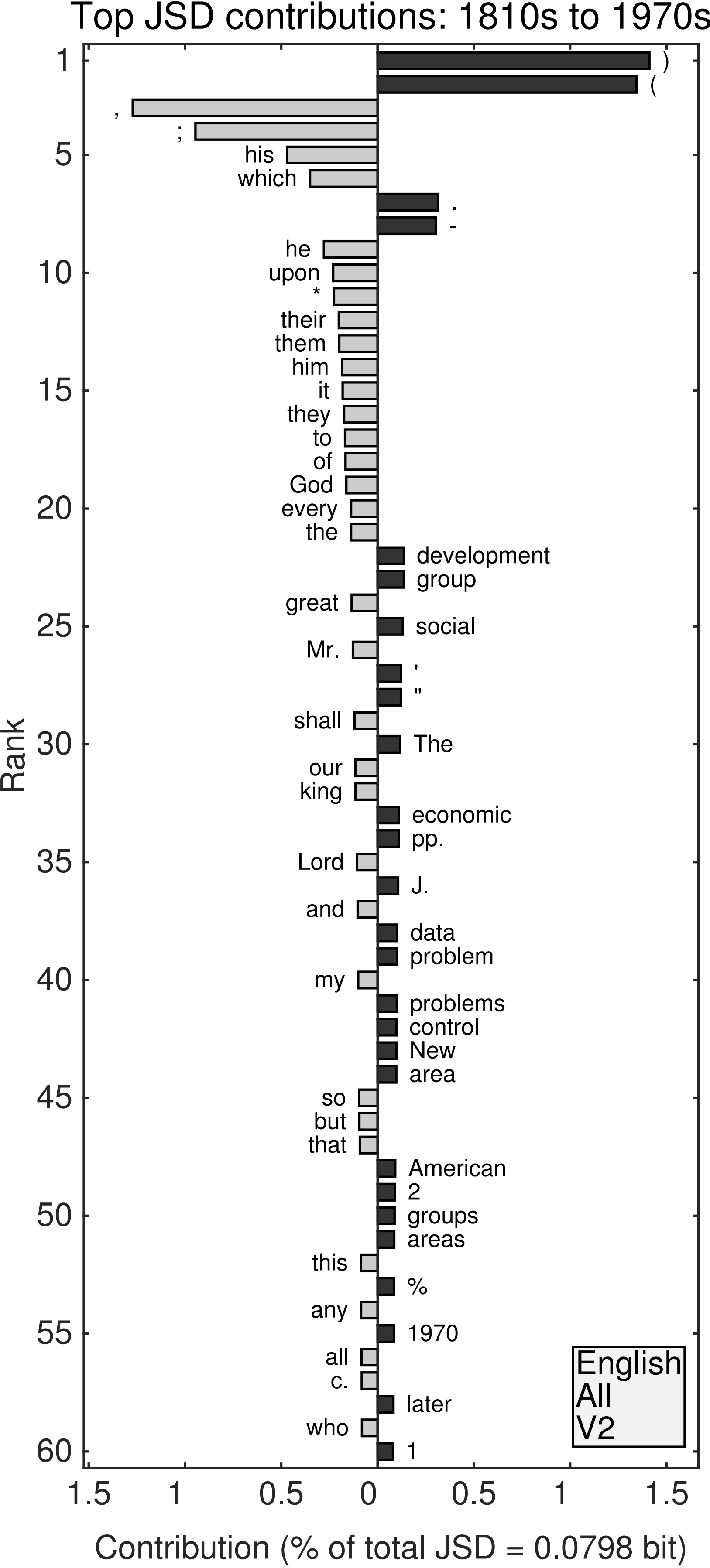

Top JSD contributions: 1810s to 1970s
Rank
1
5
10
15
20
25
30
35
40
45
50
55
60
)
(
,
;
his
which
.
-
he
upon
*
their
them
him
it
they
to
of
God
every
the
development
group
great
social
Mr.
'
"
shall
The
our
king
economic
pp.
Lord
J.
and
data
problem
my
problems
control
New
area
so
but
that
American
2
groups
areas
this
%
any
1970
all
c.
later
who
1
English
All
V2
1.5
1
0.5
0
0.5
1
1.5
Contribution (% of total JSD = 0.0798 bit)

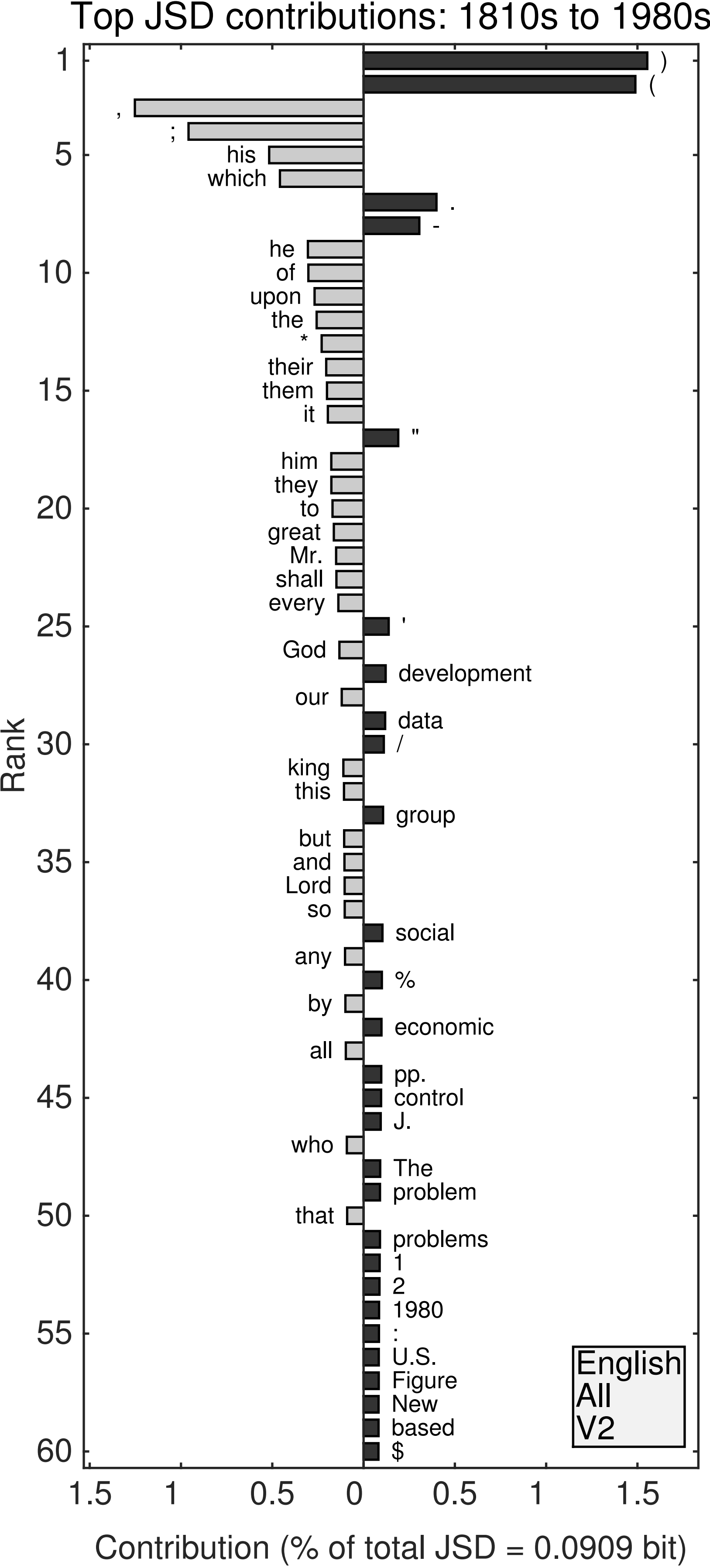

Top JSD contributions: 1810s to 1980s
Rank
)
(
,
;
his
which
.
-
he
of
upon
the
*
their
them
it
"
him
they
to
great
Mr.
shall
every
'
God
development
our
data
/
king
this
group
but
and
Lord
so
social
any
%
by
economic
all
pp.
control
J.
who
The
problem
that
problems
1
2
1980
:
U.S.
Figure
New
based
$
English
All
V2
Contribution (% of total JSD = 0.0909 bit)

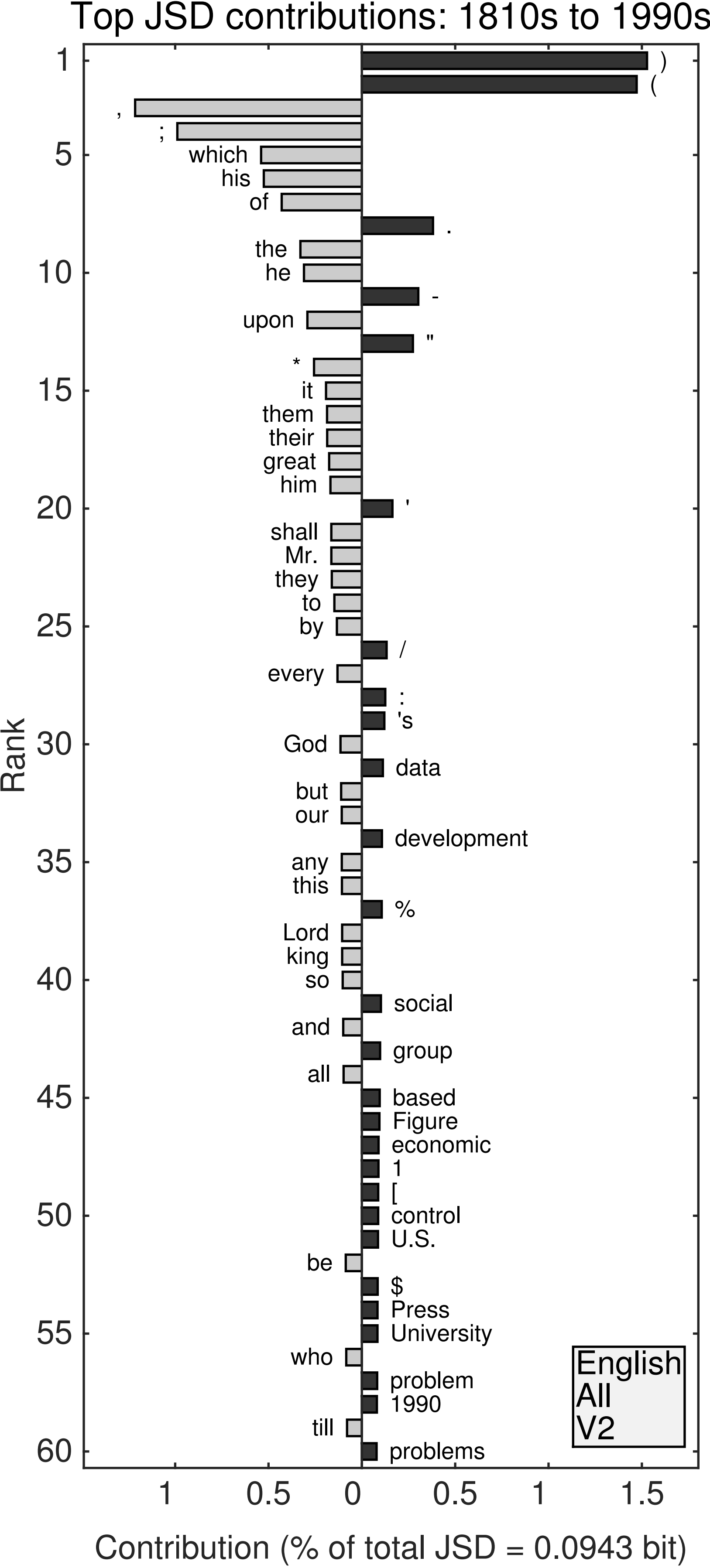

Top JSD contributions: 1810s to 1990s
Rank
Contribution (% of total JSD = 0.0943 bit)
1
5
10
15
20
25
30
35
40
45
50
55
60
1
0.5
0
0.5
1
1.5
)
(
,
;
which
his
of
.
the
he
-
upon
"
*
it
them
their
great
him
'
shall
Mr.
they
to
by
/
every
:
's
God
data
but
our
development
any
this
%
Lord
king
so
social
and
group
all
based
Figure
economic
1
[
control
U.S.
be
$
Press
University
who
problem
1990
till
problems
English
All
V2

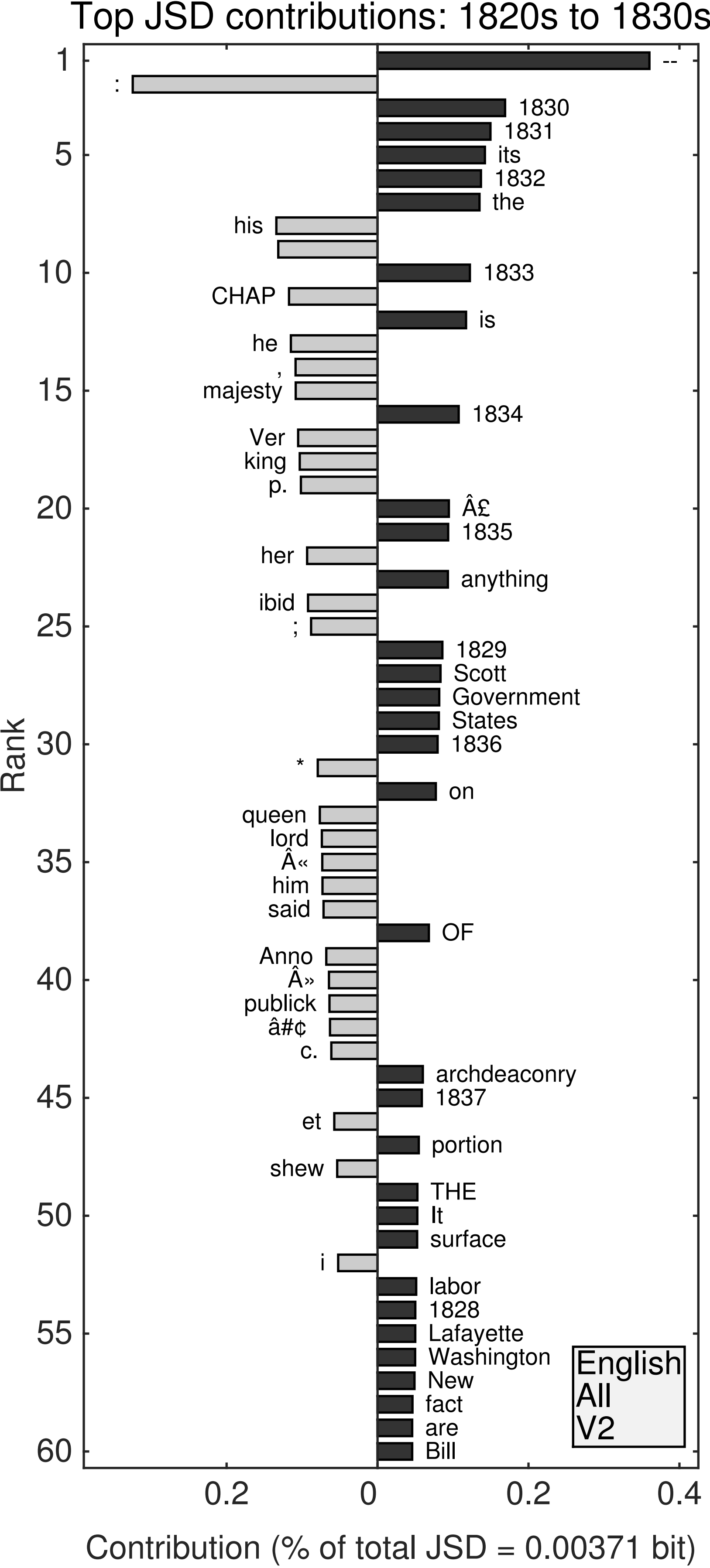
Top JSD contributions: 1820s to 1830s
Rank
Contribution (% of total JSD = 0.00371 bit)
English
All
V2
--
:
1830
1831
its
1832
the
his
1833
CHAP
is
he
,
majesty
1834
Ver
king
p.
Â£
1835
her
anything
ibid
;
1829
Scott
Government
States
1836
*
on
queen
lord
Â«
him
said
OF
Anno
Â»
publick
â#¢
c.
archdeaconry
1837
et
portion
shew
THE
It
surface
i
labor
1828
Lafayette
Washington
New
fact
are
Bill

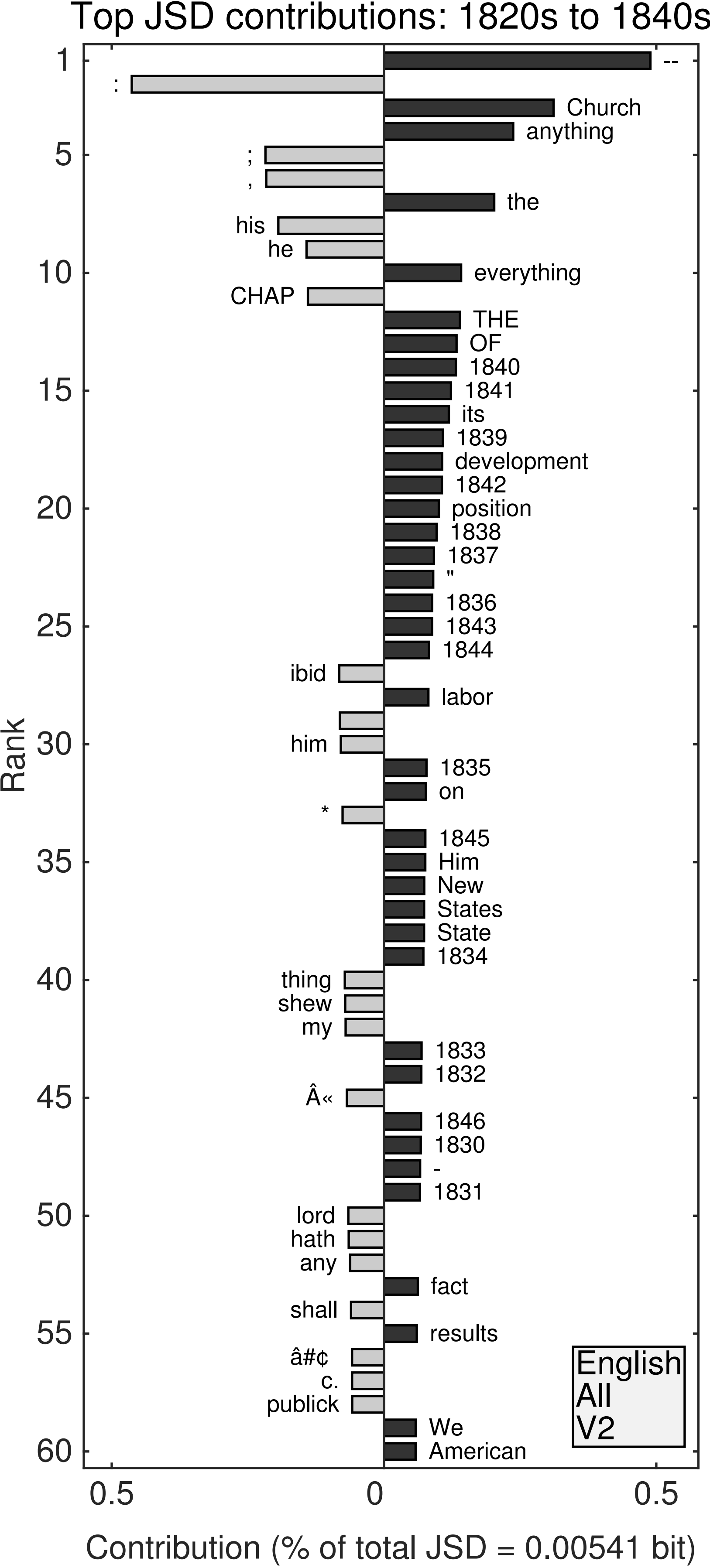

Top JSD contributions: 1820s to 1840s
Rank
Contribution (% of total JSD = 0.00541 bit)
0.5
0
0.5
1
5
10
15
20
25
30
35
40
45
50
55
60
--
:
Church
anything
;
,
the
his
he
everything
CHAP
THE
OF
1840
1841
its
1839
development
1842
position
1838
1837
"
1836
1843
1844
ibid
labor
him
1835
on
*
1845
Him
New
States
State
1834
thing
shew
my
1833
1832
Â«
1846
1830
-
1831
lord
hath
any
fact
shall
results
â#¢
c.
publick
We
American
English
All
V2

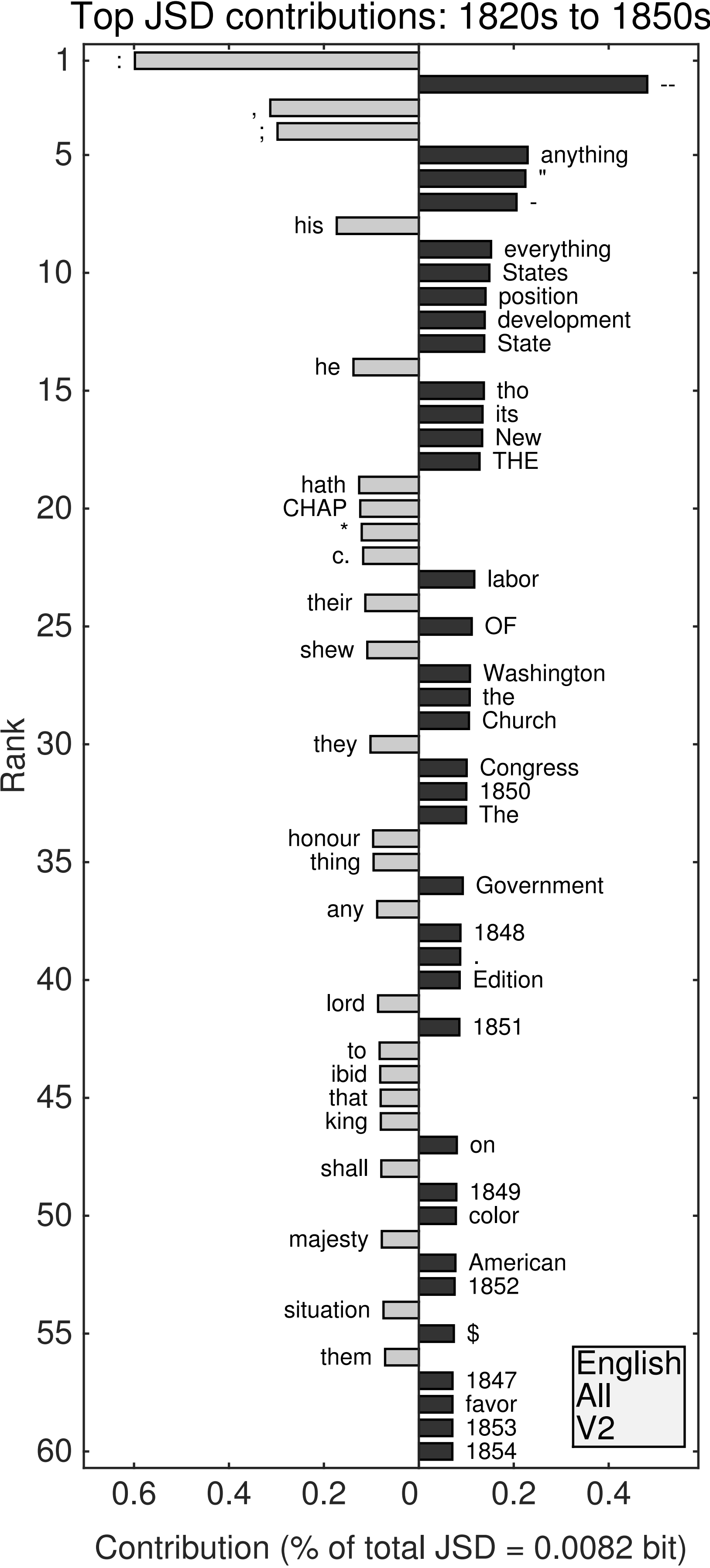

Top JSD contributions: 1820s to 1850s
Rank
Contribution (% of total JSD = 0.0082 bit)
English
All
V2
:
--
,
;
anything
"
-
his
everything
States
position
development
State
he
tho
its
New
THE
hath
CHAP
*
c.
labor
their
OF
shew
Washington
the
Church
they
Congress
1850
The
honour
thing
Government
any
1848
.
Edition
lord
1851
to
ibid
that
king
on
shall
1849
color
majesty
American
1852
situation
$
them
1847
favor
1853
1854

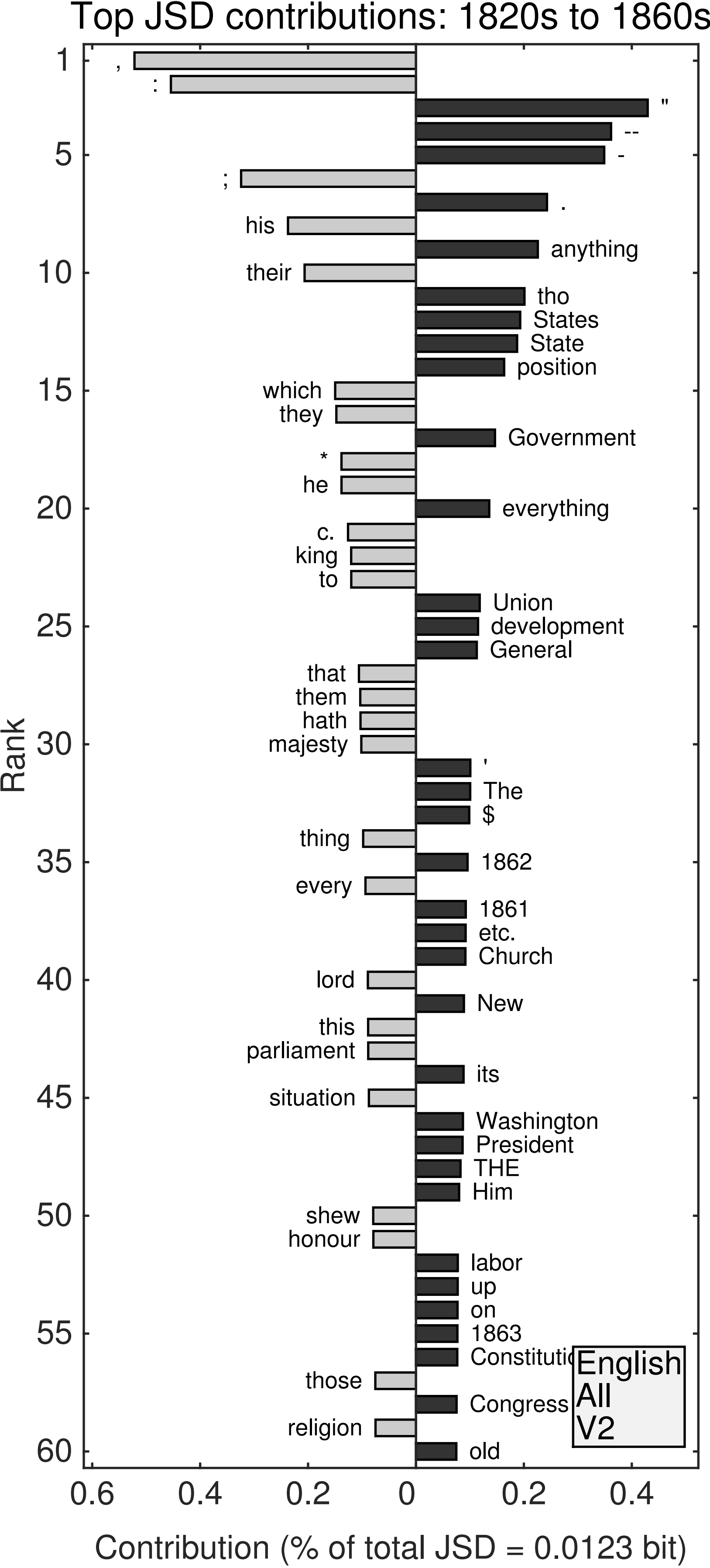

Top JSD contributions: 1820s to 1860s
Rank
Contribution (% of total JSD = 0.0123 bit)
1
5
10
15
20
25
30
35
40
45
50
55
60
0.6
0.4
0.2
0
0.2
0.4
,
:
"
--
-
;
.
his
anything
their
tho
States
State
position
which
they
Government
*
he
everything
c.
king
to
Union
development
General
that
them
hath
majesty
'
The
$
thing
1862
every
1861
etc.
Church
lord
New
this
parliament
its
situation
Washington
President
THE
Him
shew
honour
labor
up
on
1863
Constitutio
those
Congress
religion
old
English
All
V2

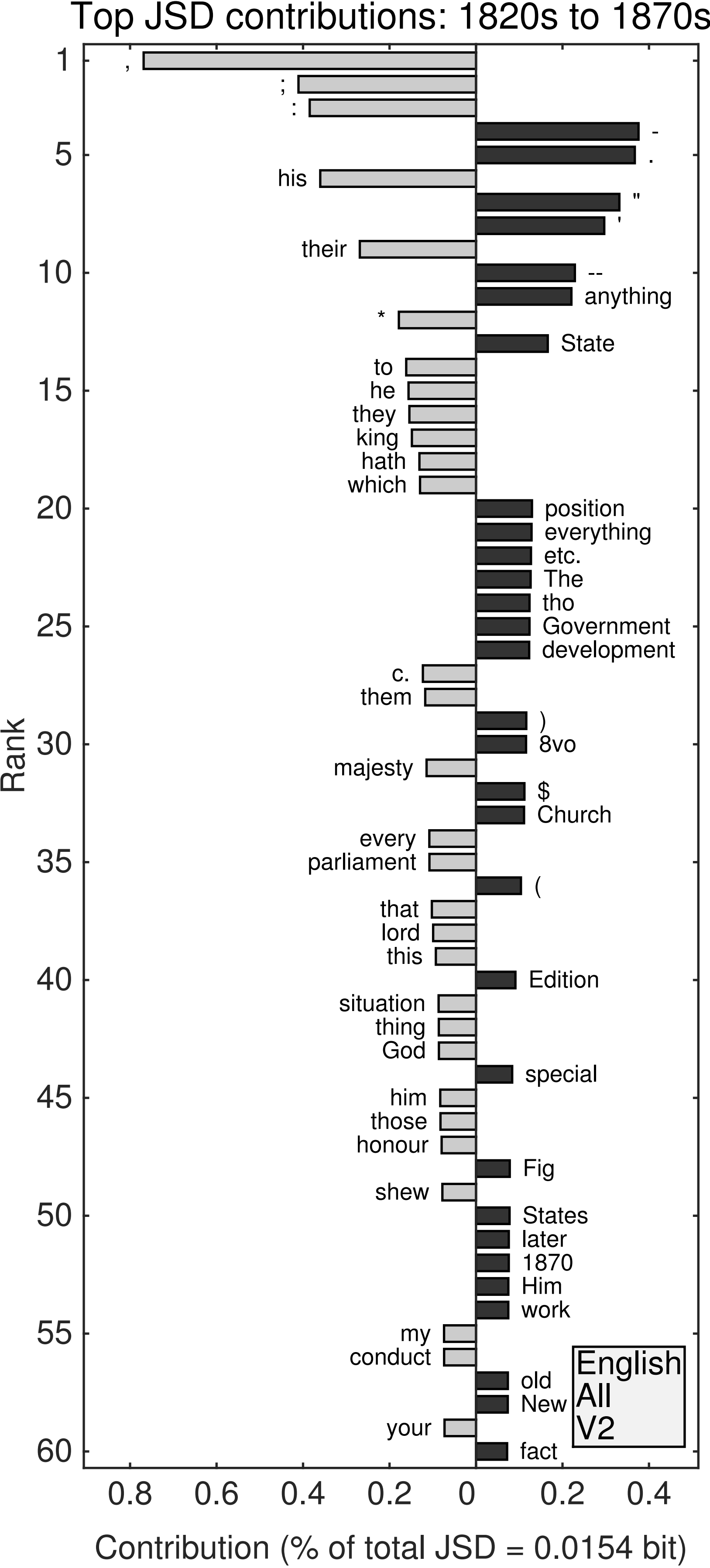

Top JSD contributions: 1820s to 1870s
Rank
Contribution (% of total JSD = 0.0154 bit)
English
All
V2
,
;
:
-
.
his
"
'
their
--
anything
*
State
to
he
they
king
hath
which
position
everything
etc.
The
tho
Government
development
c.
them
)
8vo
majesty
$
Church
every
parliament
(
that
lord
this
Edition
situation
thing
God
special
him
those
honour
Fig
shew
States
later
1870
Him
work
my
conduct
old
New
your
fact

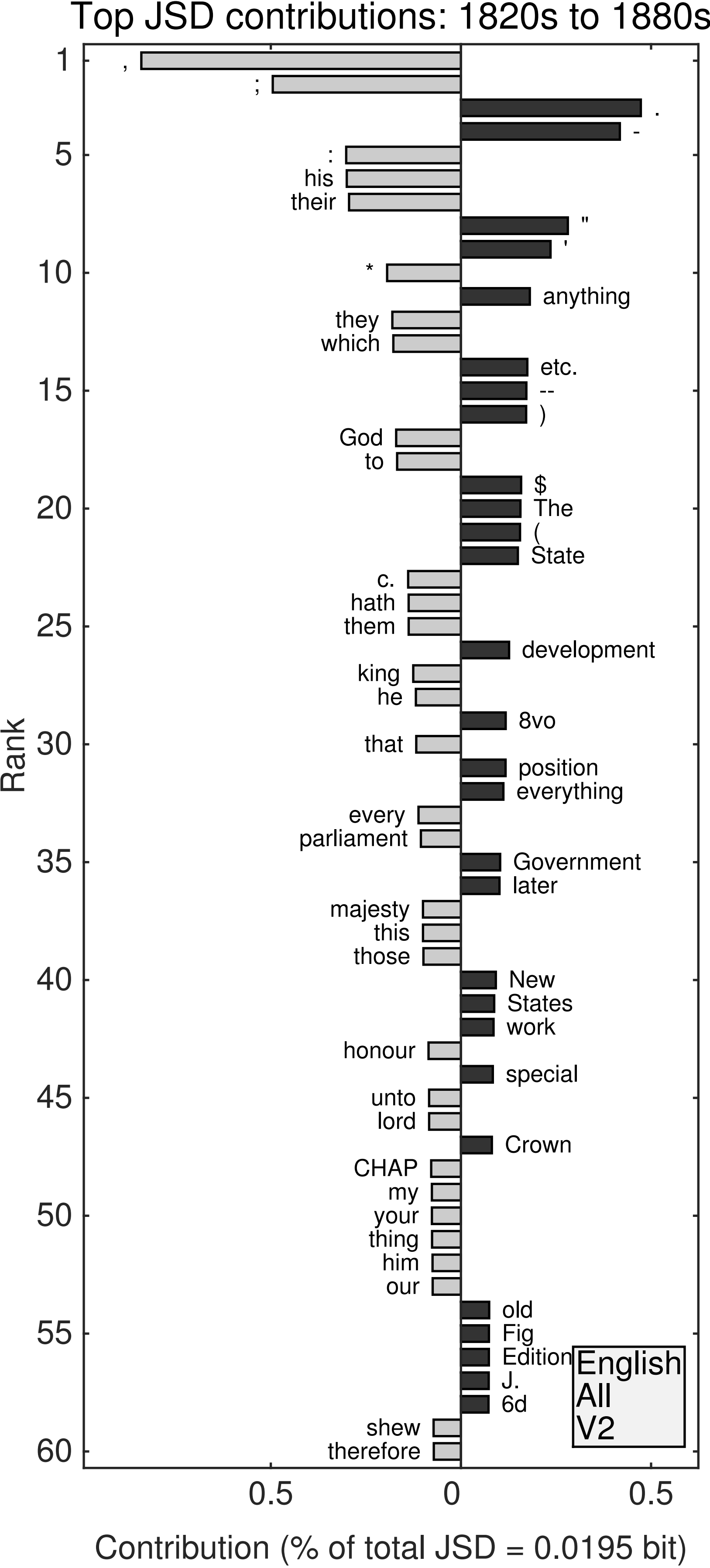

Top JSD contributions: 1820s to 1880s
Rank
Contribution (% of total JSD = 0.0195 bit)
0.5
0
0.5
1
5
10
15
20
25
30
35
40
45
50
55
60
,
;
.
-
:
his
their
"
'
*
anything
they
which
etc.
--
)
God
to
$
The
(
State
c.
hath
them
development
king
he
8vo
that
position
everything
every
parliament
Government
later
majesty
this
those
New
States
work
honour
special
unto
lord
Crown
CHAP
my
your
thing
him
our
old
Fig
Edition
J.
6d
shew
therefore
English
All
V2

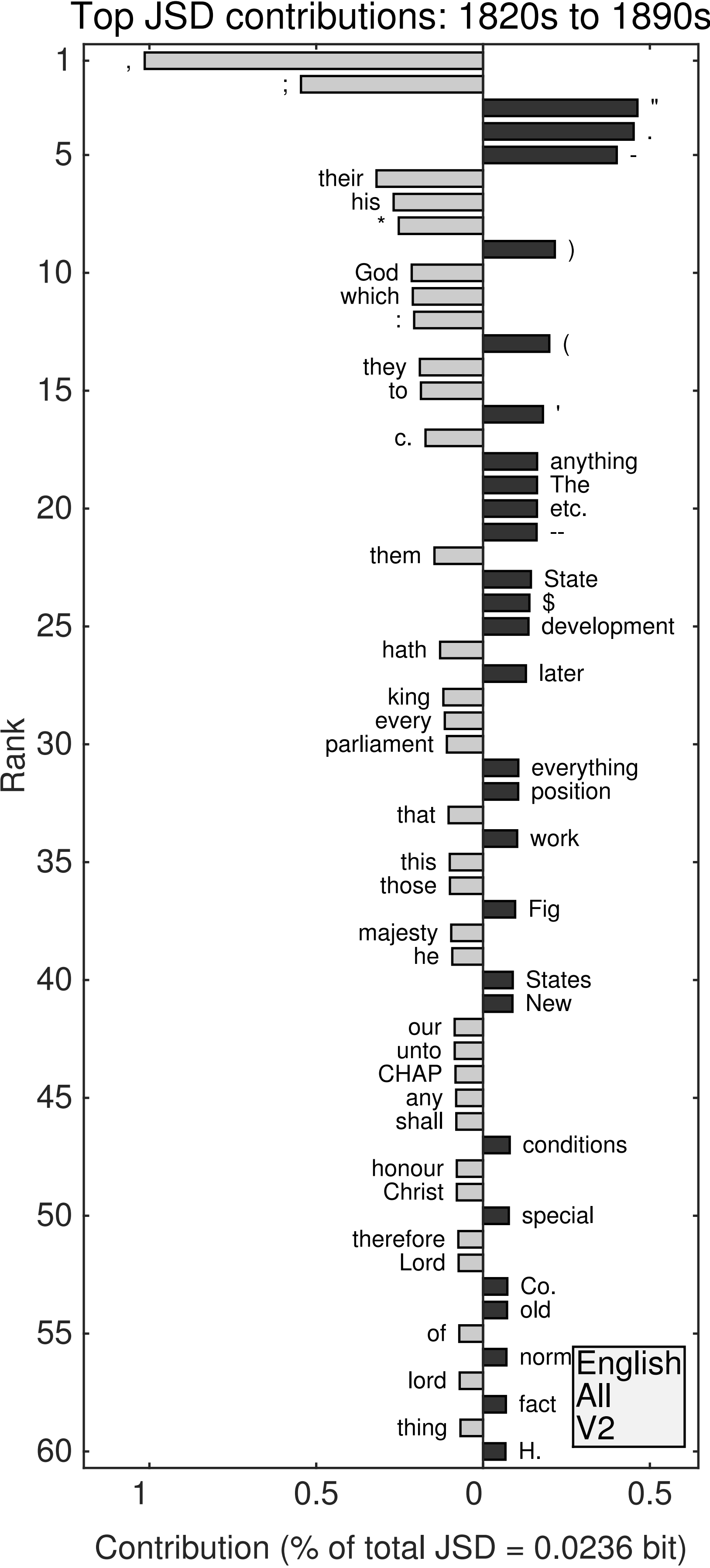

Top JSD contributions: 1820s to 1890s
Rank
Contribution (% of total JSD = 0.0236 bit)
1
5
10
15
20
25
30
35
40
45
50
55
60
1
0.5
0
0.5
,
;
"
.
-
their
his
*
)
God
which
:
(
they
to
'
c.
anything
The
etc.
--
them
State
$
development
hath
later
king
every
parliament
everything
position
that
work
this
those
Fig
majesty
he
States
New
our
unto
CHAP
any
shall
conditions
honour
Christ
special
therefore
Lord
Co.
old
of
norm
lord
fact
thing
H.
English
All
V2

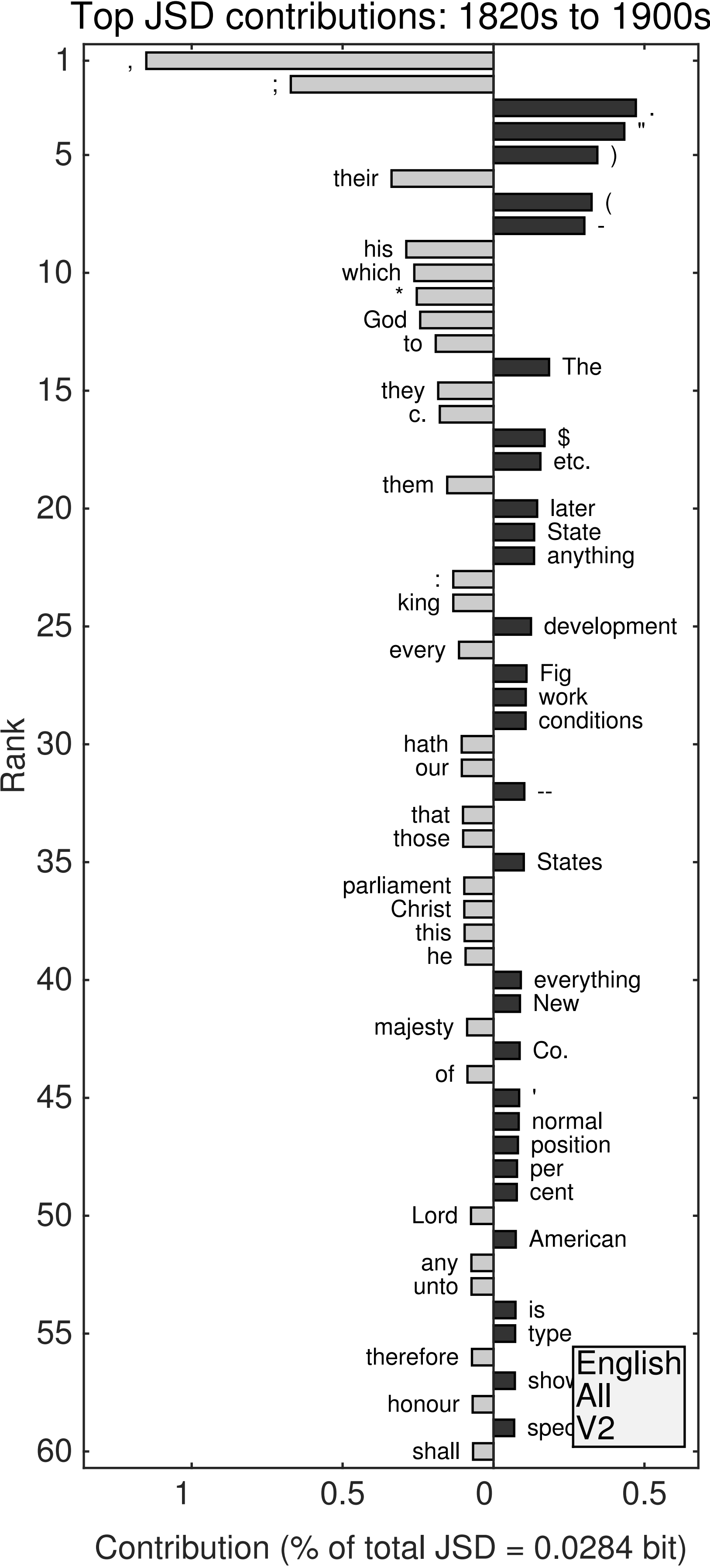
Top JSD contributions: 1820s to 1900s
Rank
1
5
10
15
20
25
30
35
40
45
50
55
60
,
;
.
"
)
their
(
-
his
which
*
God
to
The
they
c.
$
etc.
them
later
State
anything
:
king
development
every
Fig
work
conditions
hath
our
--
that
those
States
parliament
Christ
this
he
everything
New
majesty
Co.
of
'
normal
position
per
cent
Lord
American
any
unto
is
type
therefore
honour
shall
English
All
V2
1
0.5
0
0.5
Contribution (% of total JSD = 0.0284 bit)

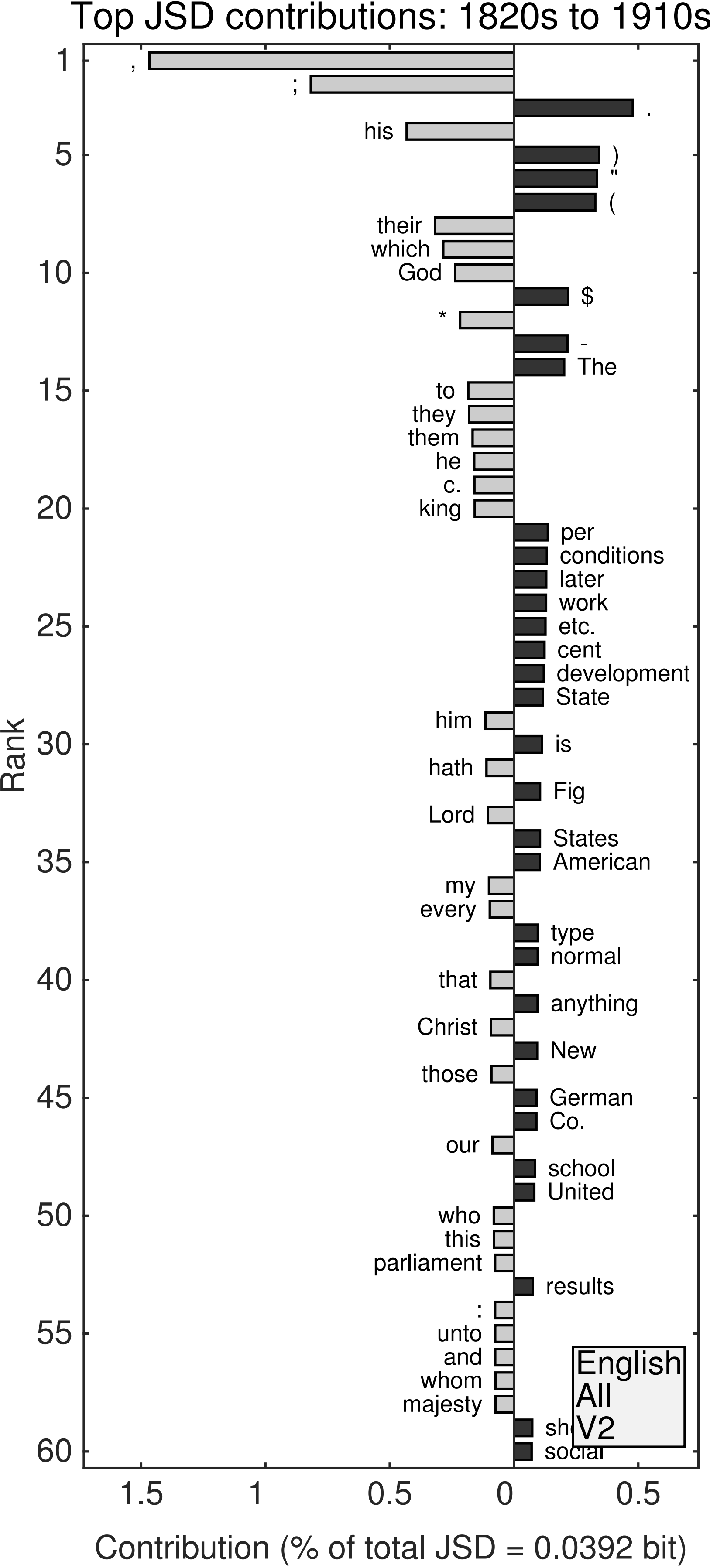

Top JSD contributions: 1820s to 1910s
,
;
.
his
)
"
(
their
which
God
$
*
-
The
to
they
them
he
c.
king
per
conditions
later
work
etc.
cent
development
State
him
is
hath
Fig
Lord
States
American
my
every
type
normal
that
anything
Christ
New
those
German
Co.
our
school
United
who
this
parliament
results
:
unto
and
whom
majesty
English
All
V2
Rank
1
5
10
15
20
25
30
35
40
45
50
55
60
1.5
1
0.5
0
0.5
Contribution (% of total JSD = 0.0392 bit)

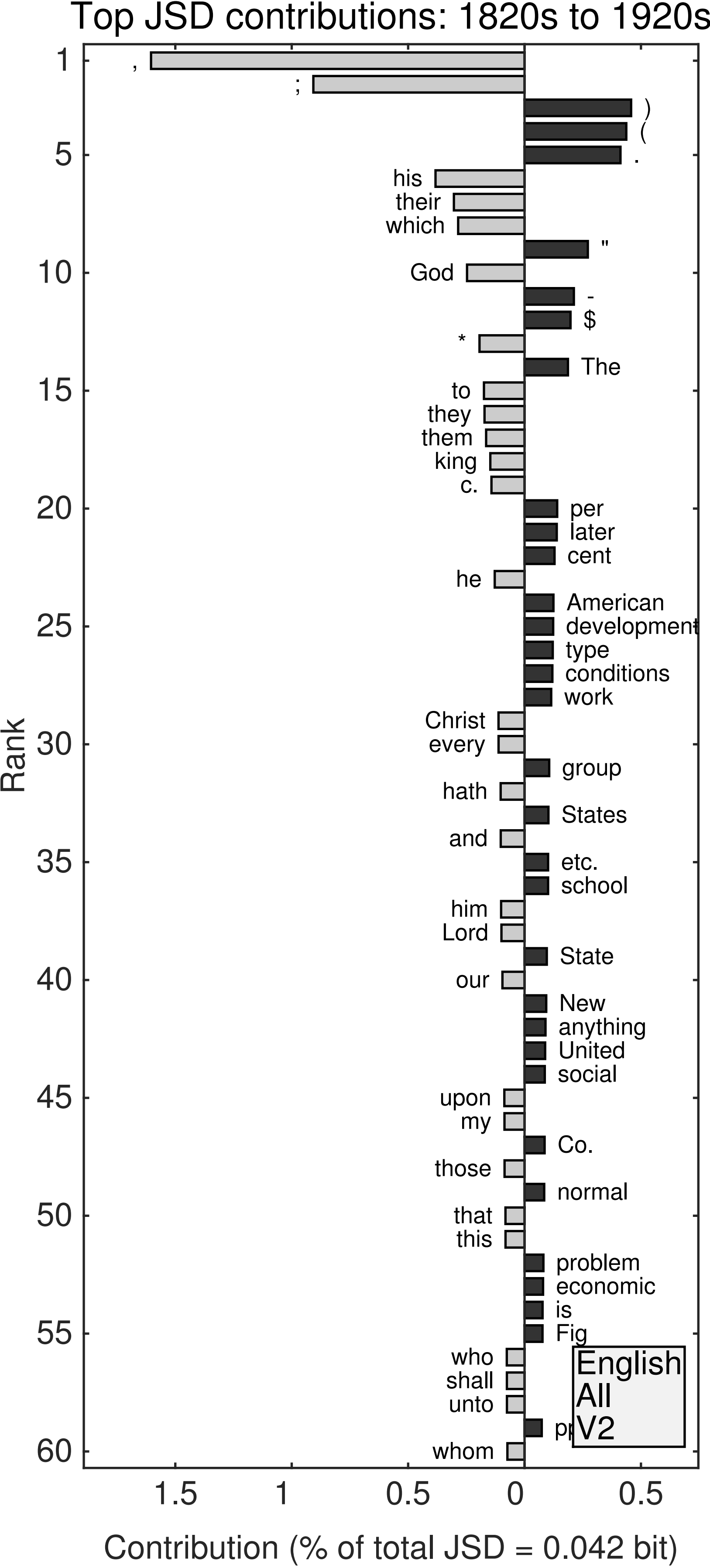

Top JSD contributions: 1820s to 1920s
Rank
Contribution (% of total JSD = 0.042 bit)
1
5
10
15
20
25
30
35
40
45
50
55
60
1.5
1
0.5
0
0.5
,
;
)
(
.
his
their
which
"
God
-
$
*
The
to
they
them
king
c.
per
later
cent
he
American
development
type
conditions
work
Christ
every
group
hath
States
and
etc.
school
him
Lord
State
our
New
anything
United
social
upon
my
Co.
those
normal
that
this
problem
economic
is
Fig
who
shall
unto
whom
English
All
V2

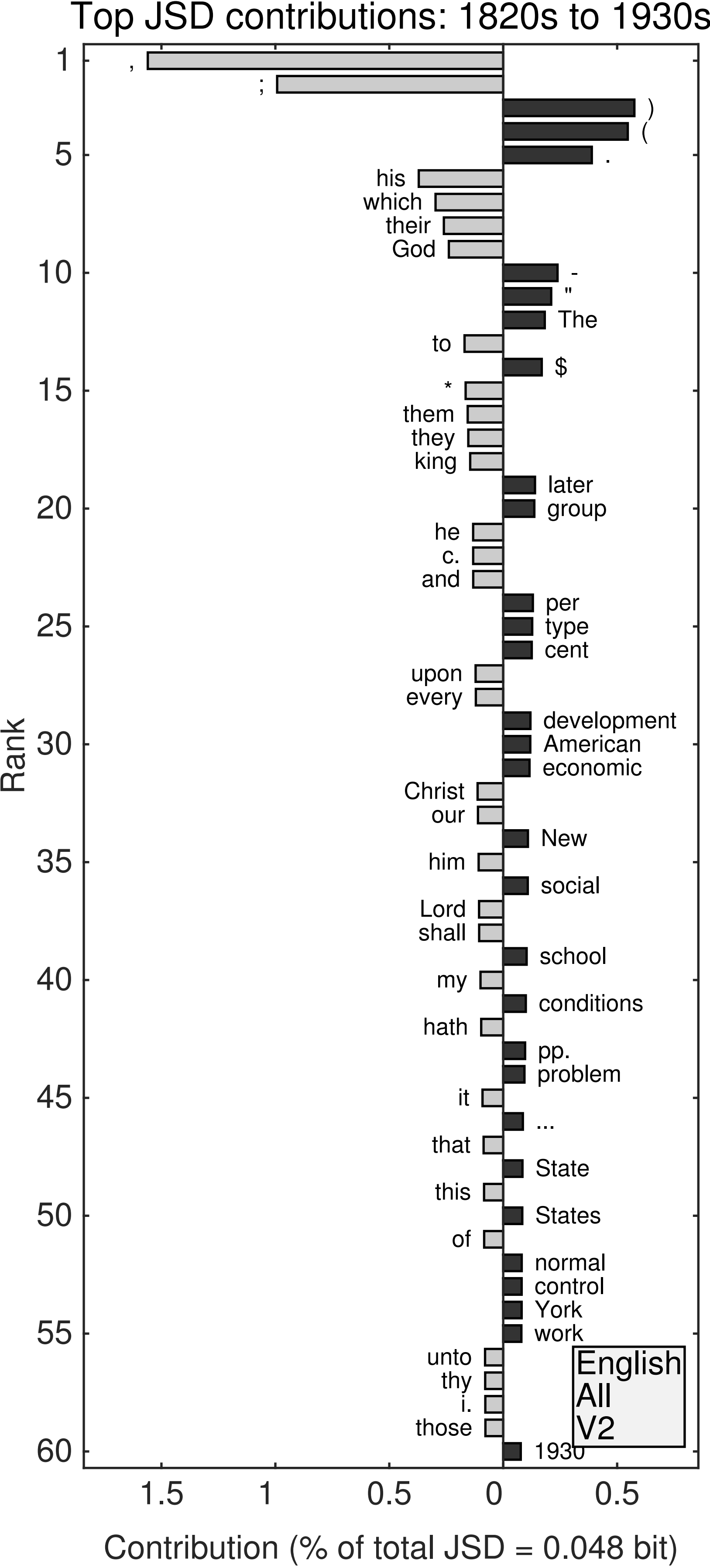

Top JSD contributions: 1820s to 1930s
Rank
Contribution (% of total JSD = 0.048 bit)
1.5
1
0.5
0
0.5
1
5
10
15
20
25
30
35
40
45
50
55
60
,
;
)
(
.
his
which
their
God
-
"
The
to
$
*
them
they
king
later
group
he
c.
and
per
type
cent
upon
every
development
American
economic
Christ
our
New
him
social
Lord
shall
school
my
conditions
hath
pp.
problem
it
...
that
State
this
States
of
normal
control
York
work
unto
thy
i.
those
1930
English
All
V2

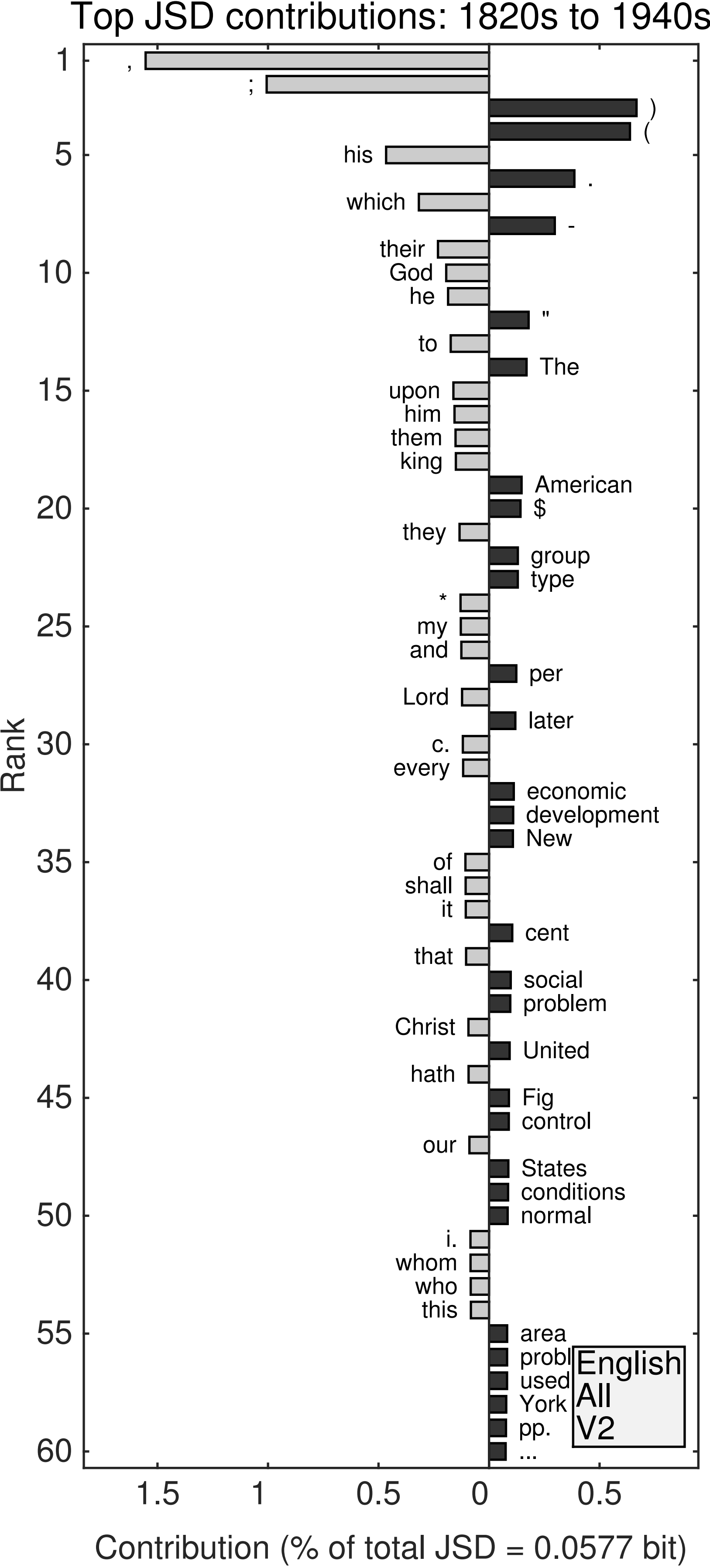

Top JSD contributions: 1820s to 1940s
Rank
Contribution (% of total JSD = 0.0577 bit)
,
;
)
(
his
.
which
-
their
God
he
"
to
The
upon
him
them
king
American
$
they
group
type
*
my
and
per
Lord
later
c.
every
economic
development
New
of
shall
it
cent
that
social
problem
Christ
United
hath
Fig
control
our
States
conditions
normal
i.
whom
who
this
area
probl
used
York
pp.
...
English
All
V2

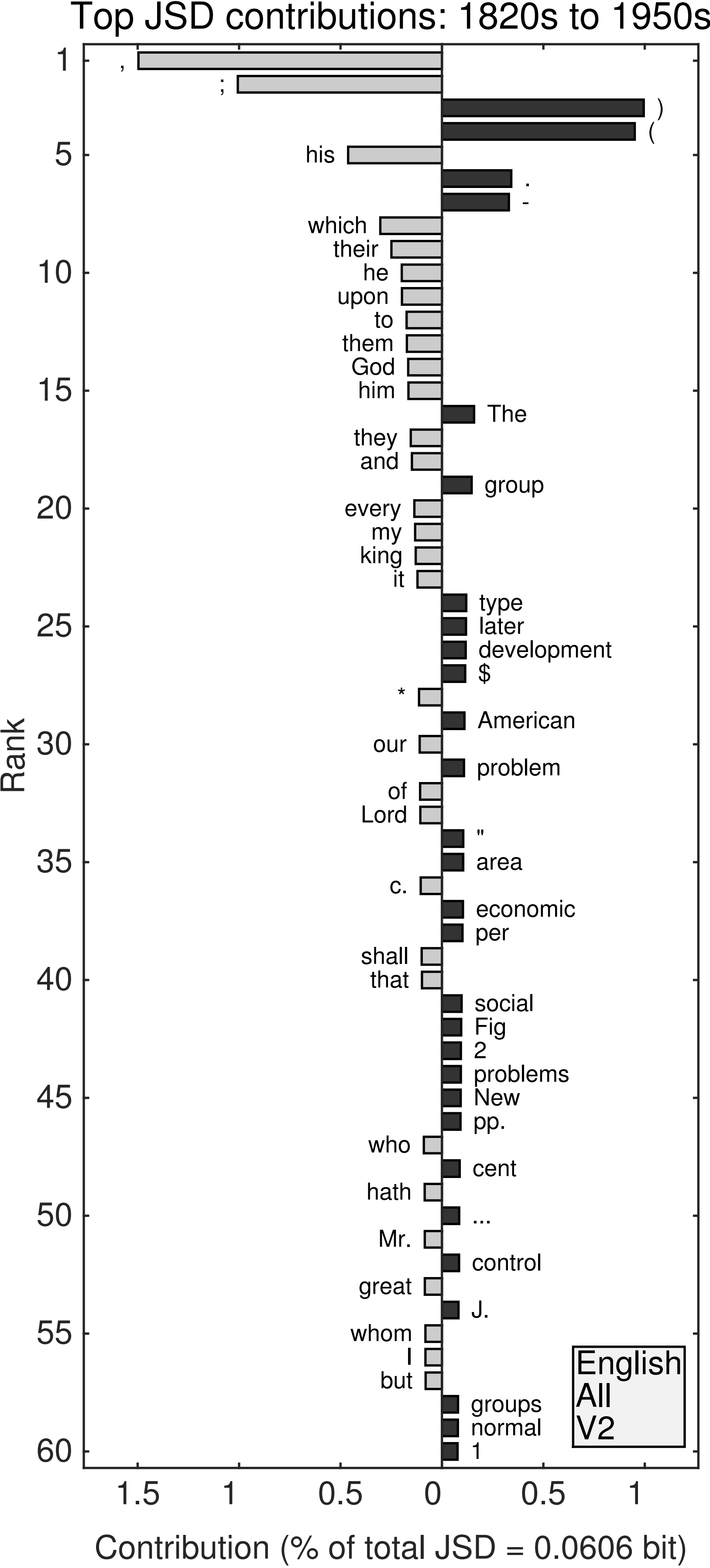

Top JSD contributions: 1820s to 1950s
Rank
Contribution (% of total JSD = 0.0606 bit)
, ;) (his . - which their he upon to them God him The they and group every my king it type later development $ * American our problem of Lord " area c. economic per shall that social Fig 2 problems New pp. who cent hath ... Mr. control great J. whom I but groups normal 1
English
All
V2

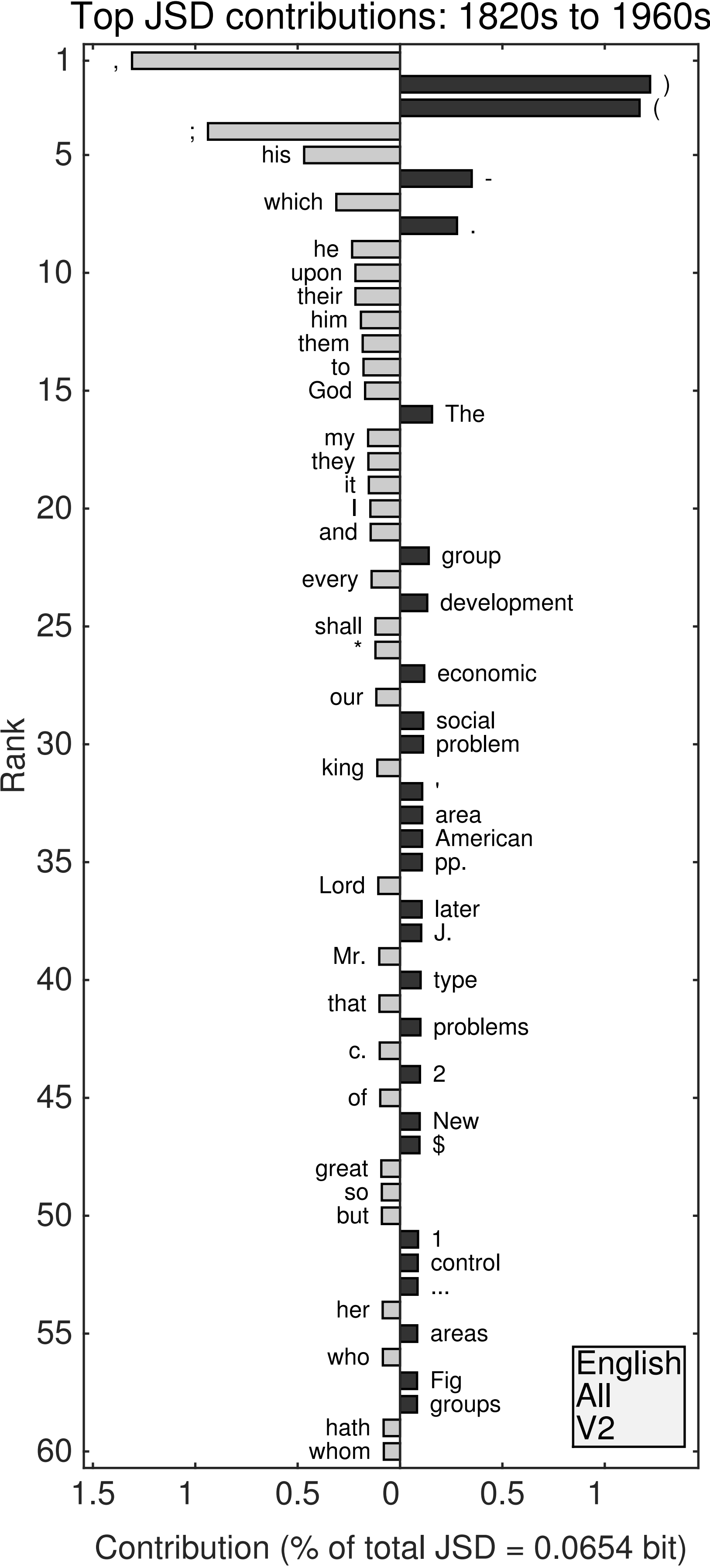

Top JSD contributions: 1820s to 1960s
Rank
Contribution (% of total JSD = 0.0654 bit)
1.5
1
0.5
0
0.5
1
1
5
10
15
20
25
30
35
40
45
50
55
60
,
)
(
;
his
-
which
.
he
upon
their
him
them
to
God
The
my
they
it
I
and
group
every
development
shall
*
economic
our
social
problem
king
'
area
American
pp.
Lord
later
J.
Mr.
type
that
problems
c.
2
of
New
$
great
so
but
1
control
...
her
areas
who
Fig
groups
hath
whom
English
All
V2

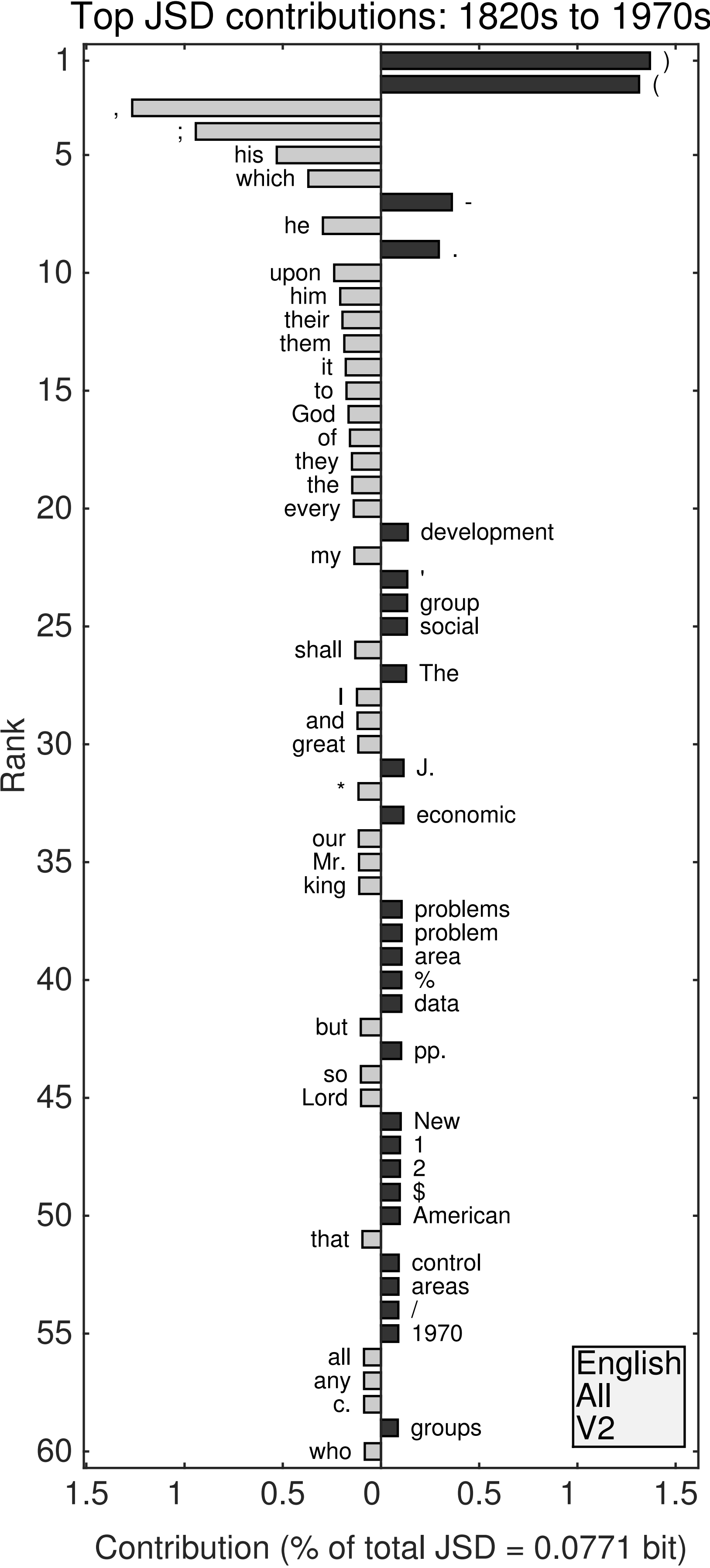
Top JSD contributions: 1820s to 1970s
Rank
Contribution (% of total JSD = 0.0771 bit)
)
(
,
;
his
which
-
he
.
upon
him
their
them
it
to
God
of
they
the
every
development
my
'
group
social
shall
The
I
and
great
J.
*
economic
our
Mr.
king
problems
problem
area
%
data
but
pp.
so
Lord
New
1
2
$
American
that
control
areas
/
1970
all
any
c.
groups
who
English
All
V2

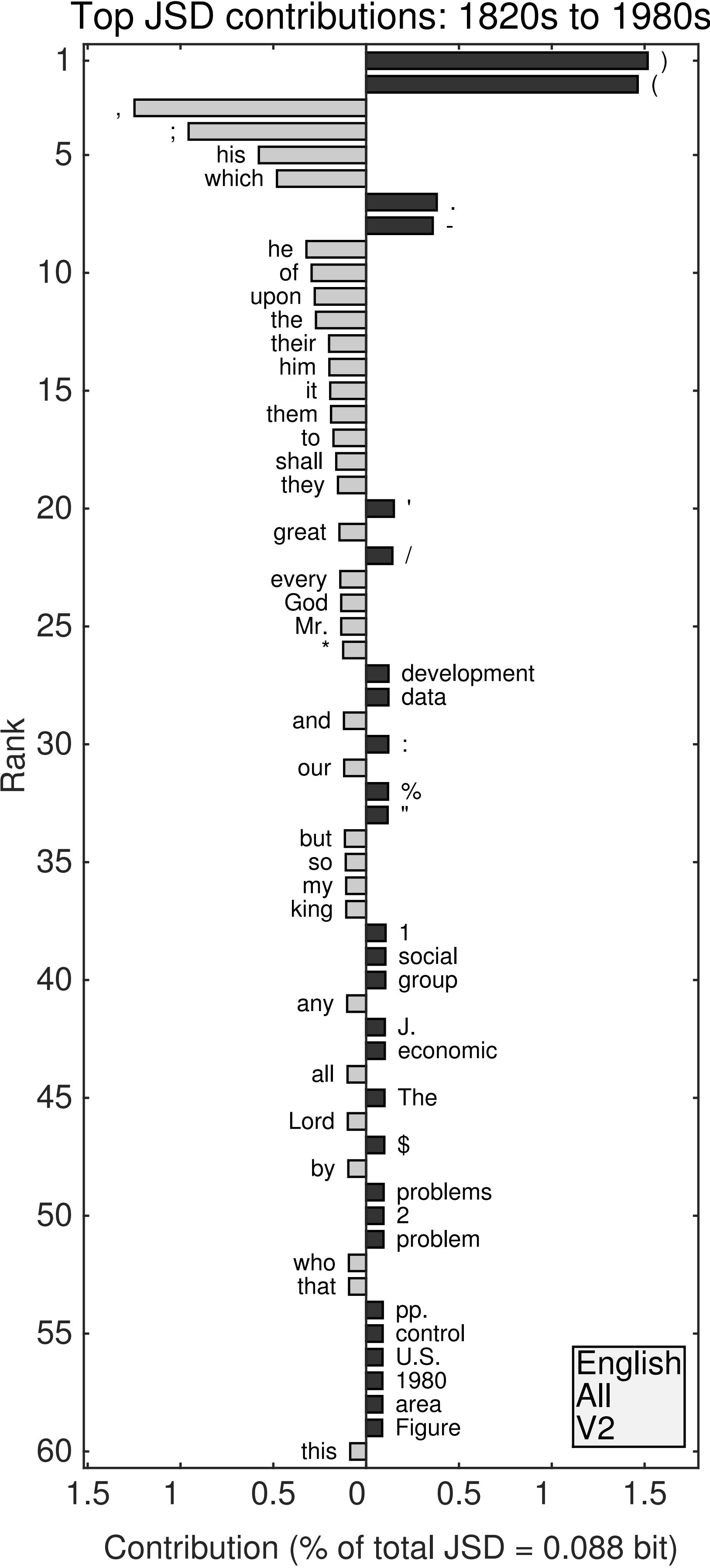

Top JSD contributions: 1820s to 1980s
Rank
Contribution (% of total JSD = 0.088 bit)
)
(
,
;
his
which
.
-
he
of
upon
the
their
him
it
them
to
shall
they
'
great
/
every
God
Mr.
*
development
data
and
:
our
%
"
but
so
my
king
1
social
group
any
J.
economic
all
The
Lord
$
by
problems
2
problem
who
that
pp.
control
U.S.
1980
area
Figure
this
English
All
V2

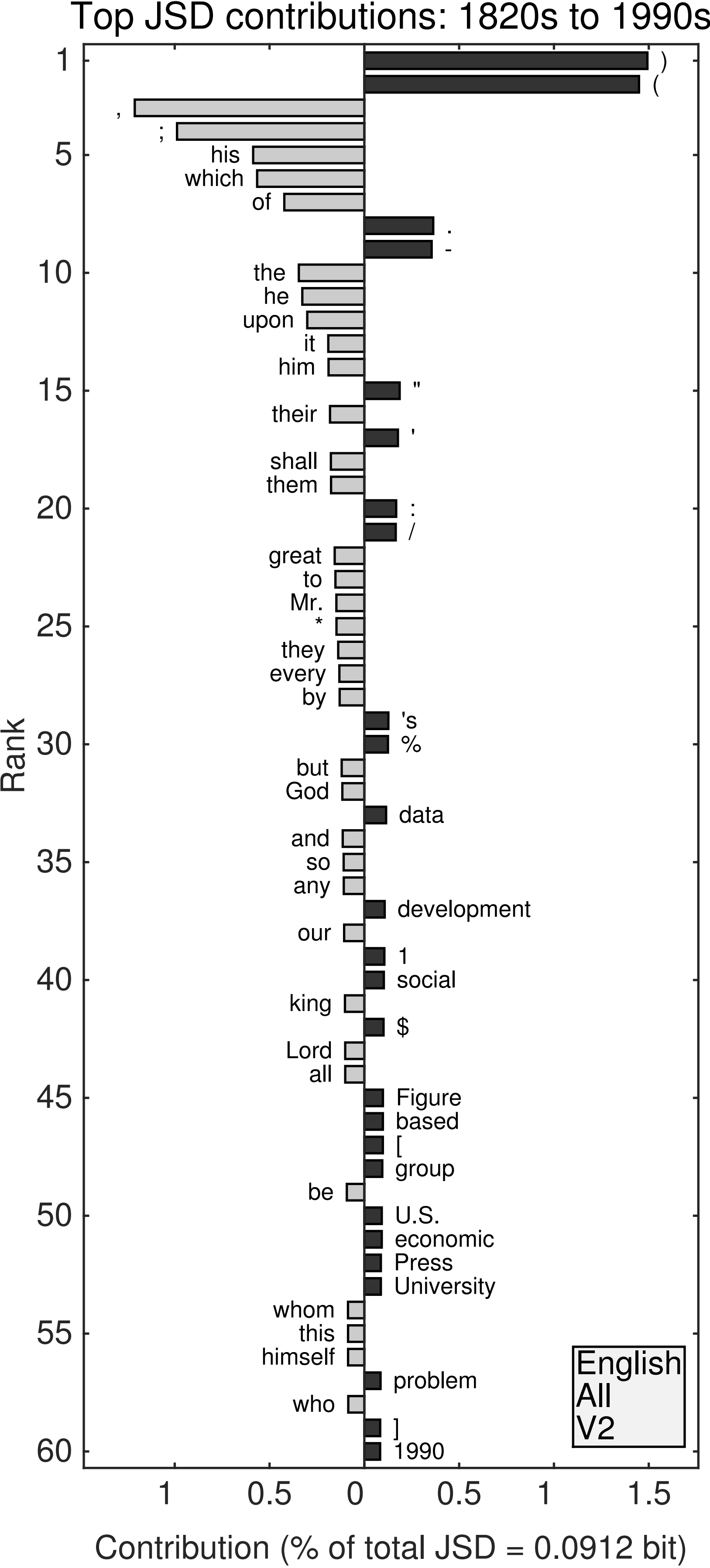

Top JSD contributions: 1820s to 1990s
Rank
Contribution (% of total JSD = 0.0912 bit)
English
All
V2
1
5
10
15
20
25
30
35
40
45
50
55
60
1
0.5
0
0.5
1
1.5
)
(
,
;
his
which
of
.
-
the
he
upon
it
him
"
their
'
shall
them
:
/
great
to
Mr.
*
they
every
by
's
%
but
God
data
and
so
any
development
our
1
social
king
$
Lord
all
Figure
based
[
group
be
U.S.
economic
Press
University
whom
this
himself
problem
who
]
1990

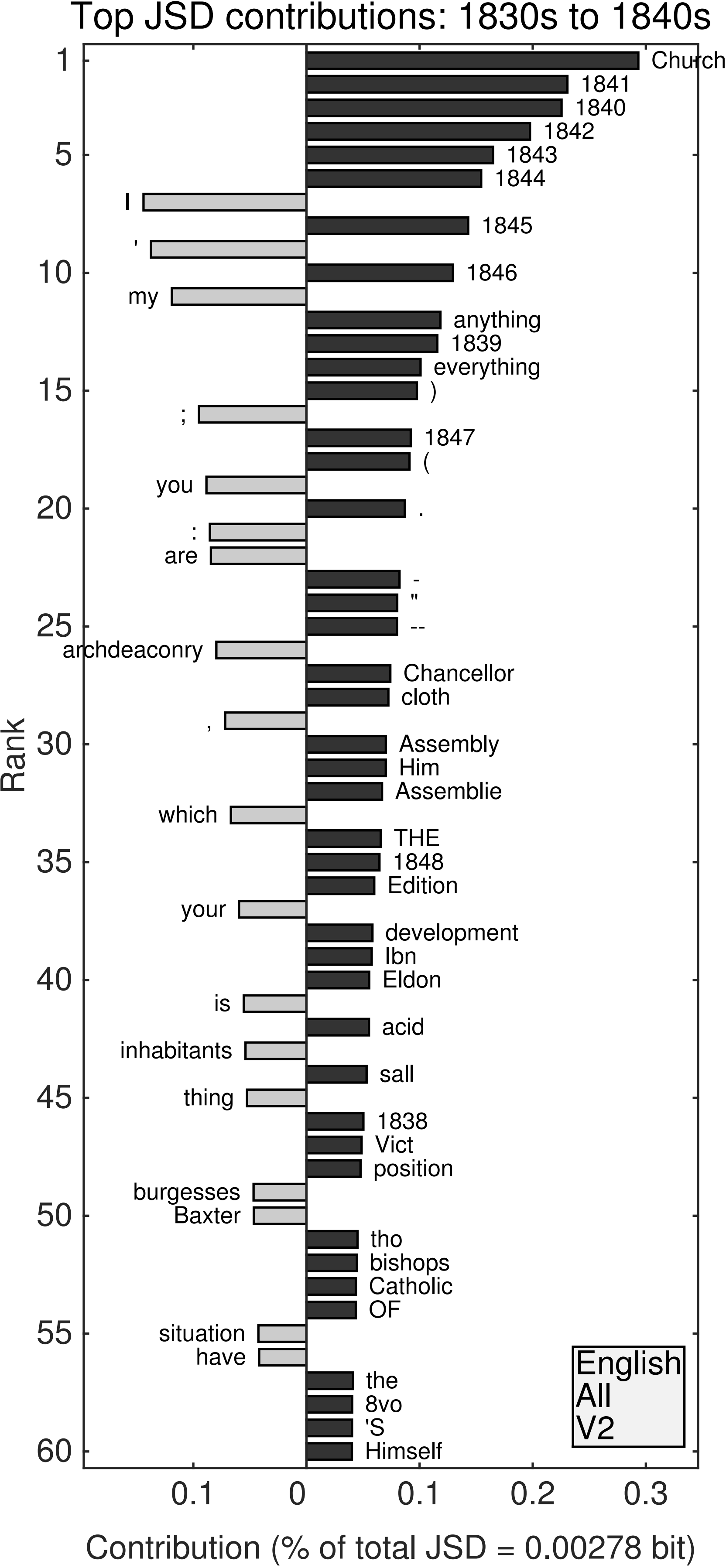

Top JSD contributions: 1830s to 1840s
Rank
Contribution (% of total JSD = 0.00278 bit)
Church
1841
1840
1842
1843
1844
I
1845
'
1846
my
anything
1839
everything
)
;
1847
(
you
.
:
are
-
"
--
archdeaconry
Chancellor
cloth
,
Assembly
Him
Assemblie
which
THE
1848
Edition
your
development
Ibn
Eldon
is
acid
inhabitants
sall
thing
1838
Vict
position
burgesses
Baxter
tho
bishops
Catholic
OF
situation
have
the
8vo
'S
Himself
English
All
V2

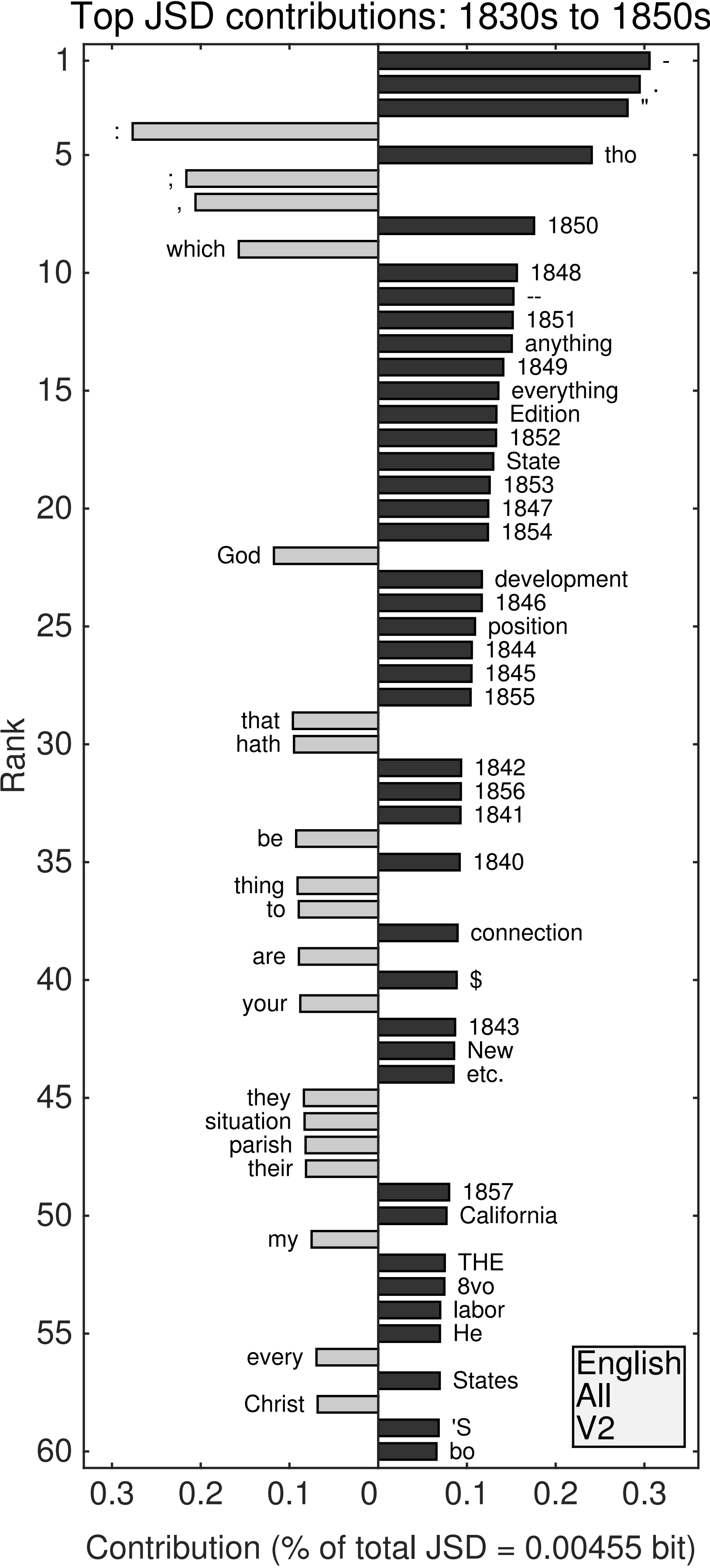

Top JSD contributions: 1830s to 1850s
Rank
Contribution (% of total JSD = 0.00455 bit)
1
5
10
15
20
25
30
35
40
45
50
55
60
0.3
0.2
0.1
0
0.1
0.2
0.3
-
.
"
:
tho
;
,
1850
which
1848
--
1851
anything
1849
everything
Edition
1852
State
1853
1847
1854
God
development
1846
position
1844
1845
1855
that
hath
1842
1856
1841
be
1840
thing
to
connection
are
$
your
1843
New
etc.
they
situation
parish
their
1857
California
my
THE
8vo
labor
He
every
States
Christ
'S
bo
English
All
V2

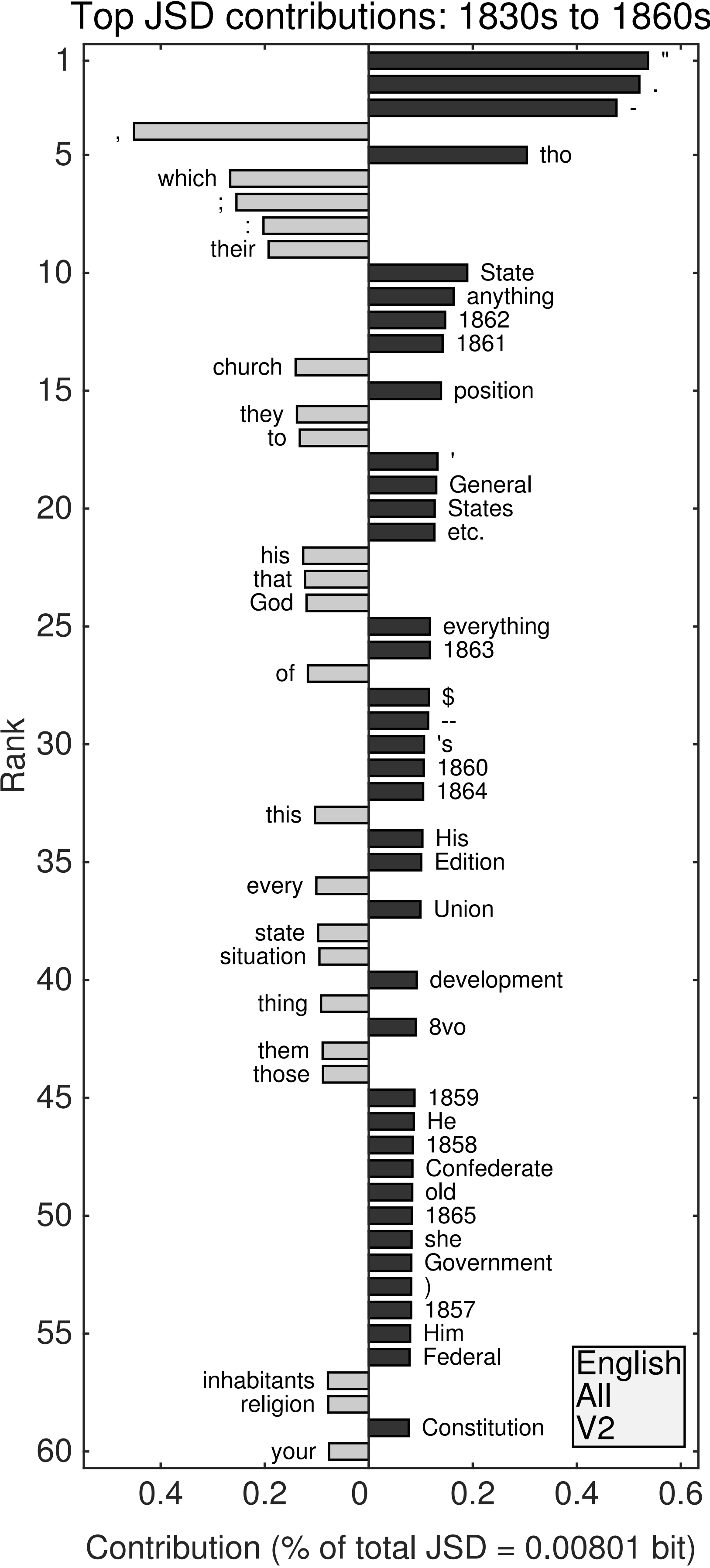

Top JSD contributions: 1830s to 1860s
Rank
Contribution (% of total JSD = 0.00801 bit)
"
.
-
,
tho
which
;
:
their
State
anything
1862
1861
church
position
they
to
'
General
States
etc.
his
that
God
everything
1863
of
$
--
's
1860
1864
this
His
Edition
every
Union
state
situation
development
thing
8vo
them
those
1859
He
1858
Confederate
old
1865
she
Government
)
1857
Him
Federal
inhabitants
religion
Constitution
your
English
All
V2

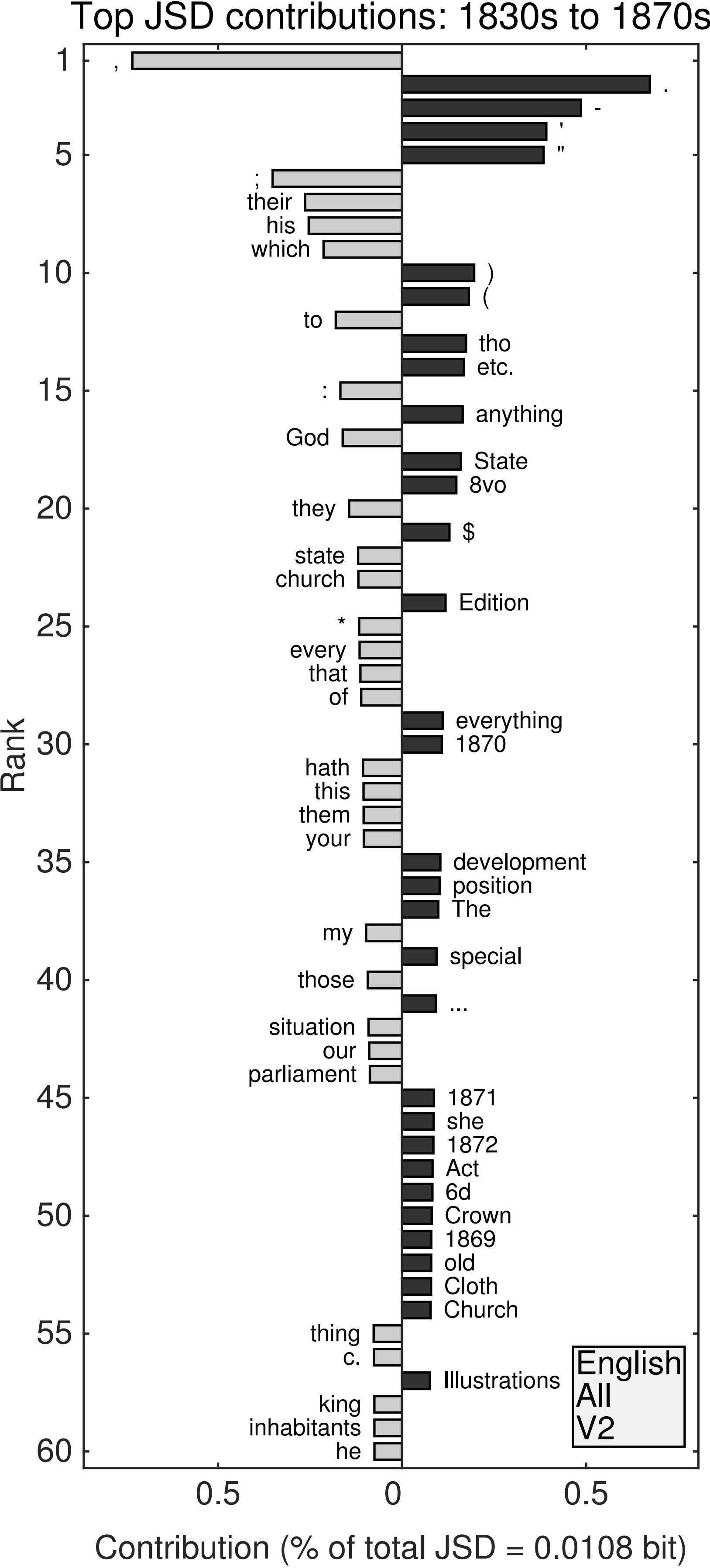
Top JSD contributions: 1830s to 1870s
Rank
Contribution (% of total JSD = 0.0108 bit)
English
All
V2
1
5
10
15
20
25
30
35
40
45
50
55
60
0.5
0
0.5
,
.
-
'
"
;
their
his
which
)
(
to
tho
etc.
:
anything
God
State
8vo
they
$
state
church
Edition
*
every
that
of
everything
1870
hath
this
them
your
development
position
The
my
special
those
...
situation
our
parliament
1871
she
1872
Act
6d
Crown
1869
old
Cloth
Church
thing
c.
Illustrations
king
inhabitants
he

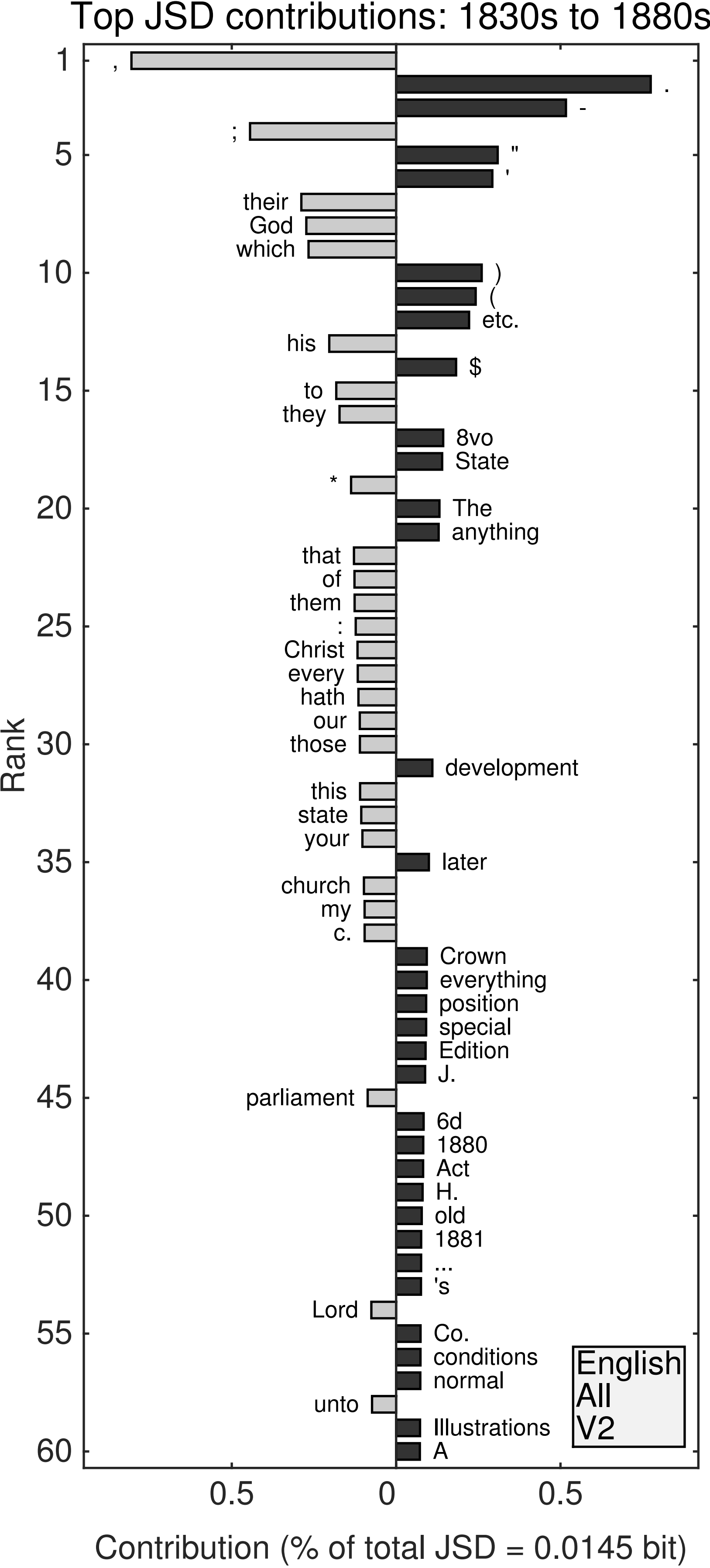

Top JSD contributions: 1830s to 1880s
Rank
Contribution (% of total JSD = 0.0145 bit)
,
.
-
;
"
'
their
God
which
)
(
etc.
his
$
to
they
8vo
State
*
The
anything
that
of
them
:
Christ
every
hath
our
those
development
this
state
your
later
church
my
c.
Crown
everything
position
special
Edition
J.
parliament
6d
1880
Act
H.
old
1881
...
's
Lord
Co.
conditions
normal
unto
Illustrations
A
English
All
V2

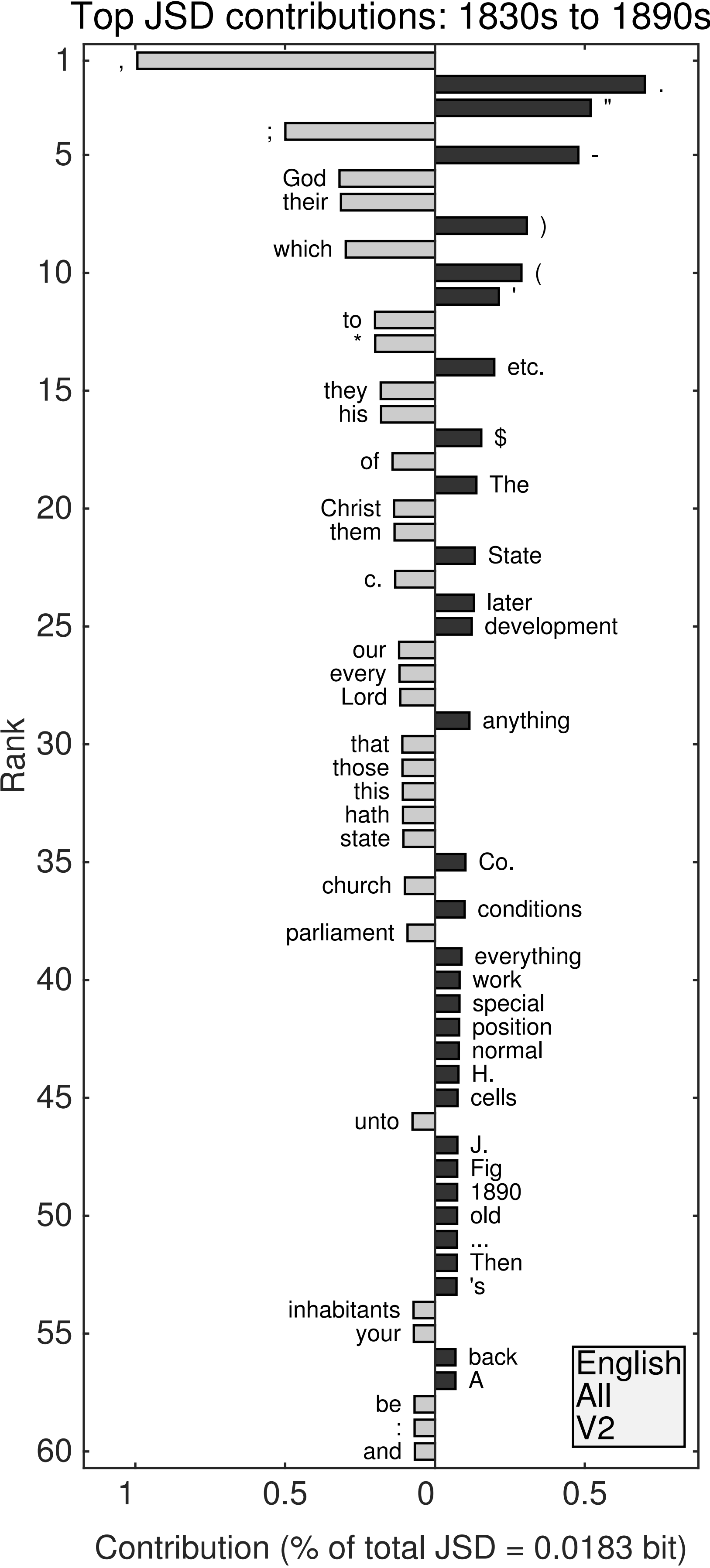
Top JSD contributions: 1830s to 1890s
Rank
Contribution (% of total JSD = 0.0183 bit)
1
5
10
15
20
25
30
35
40
45
50
55
60
1
0.5
0
0.5
,
.
"
;
-
God
their
)
which
(
'
to
*
etc.
they
his
$
of
The
Christ
them
State
c.
later
development
our
every
Lord
anything
that
those
this
hath
state
Co.
church
conditions
parliament
everything
work
special
position
normal
H.
cells
unto
J.
Fig
1890
old
...
Then
's
inhabitants
your
back
A
be
:
and
English
All
V2

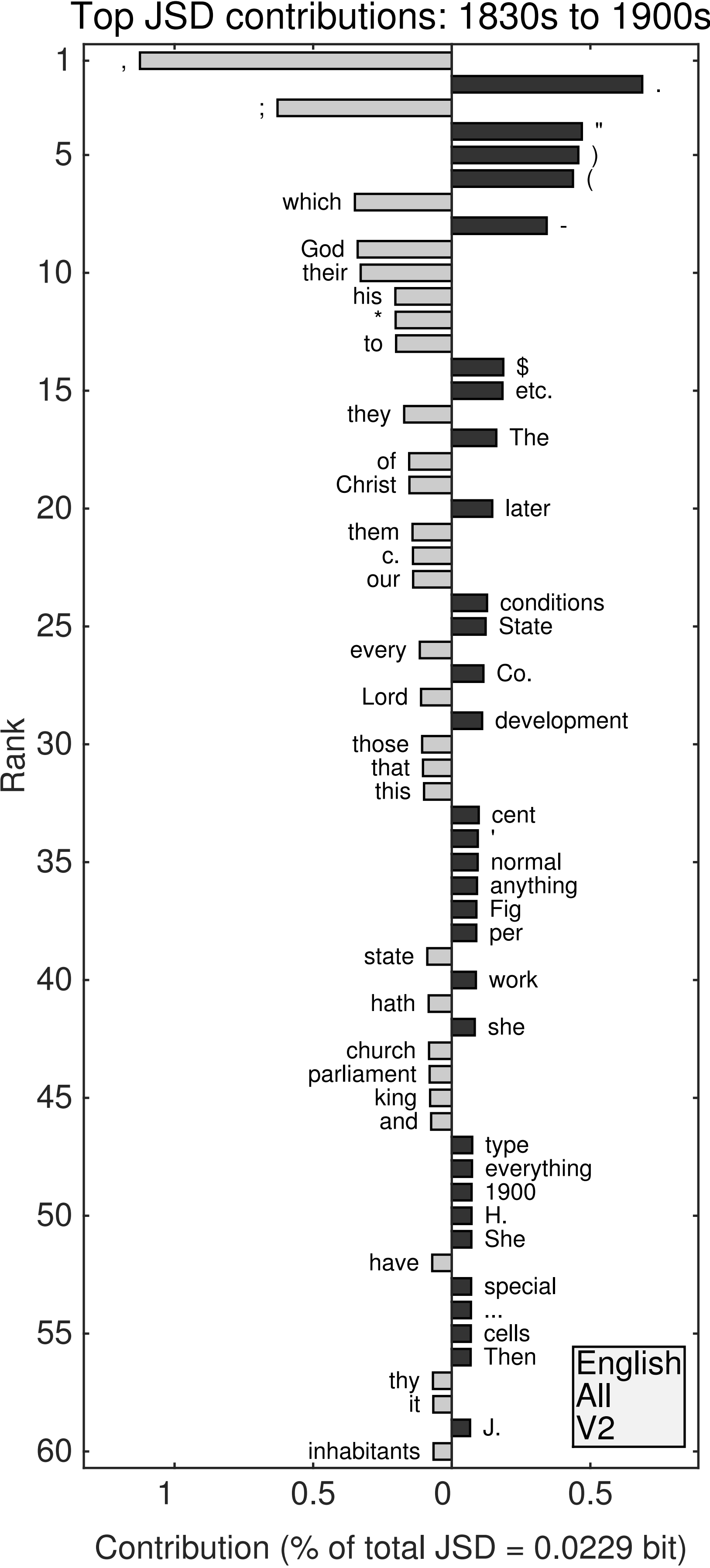

Top JSD contributions: 1830s to 1900s
Rank
Contribution (% of total JSD = 0.0229 bit)
1
5
10
15
20
25
30
35
40
45
50
55
60
1
0.5
0
0.5
,
.
;
"
)
(
which
-
God
their
his
*
to
$
etc.
they
The
of
Christ
later
them
c.
our
conditions
State
every
Co.
Lord
development
those
that
this
cent
'
normal
anything
Fig
per
state
work
hath
she
church
parliament
king
and
type
everything
1900
H.
She
have
special
...
cells
Then
thy
it
J.
inhabitants
English
All
V2

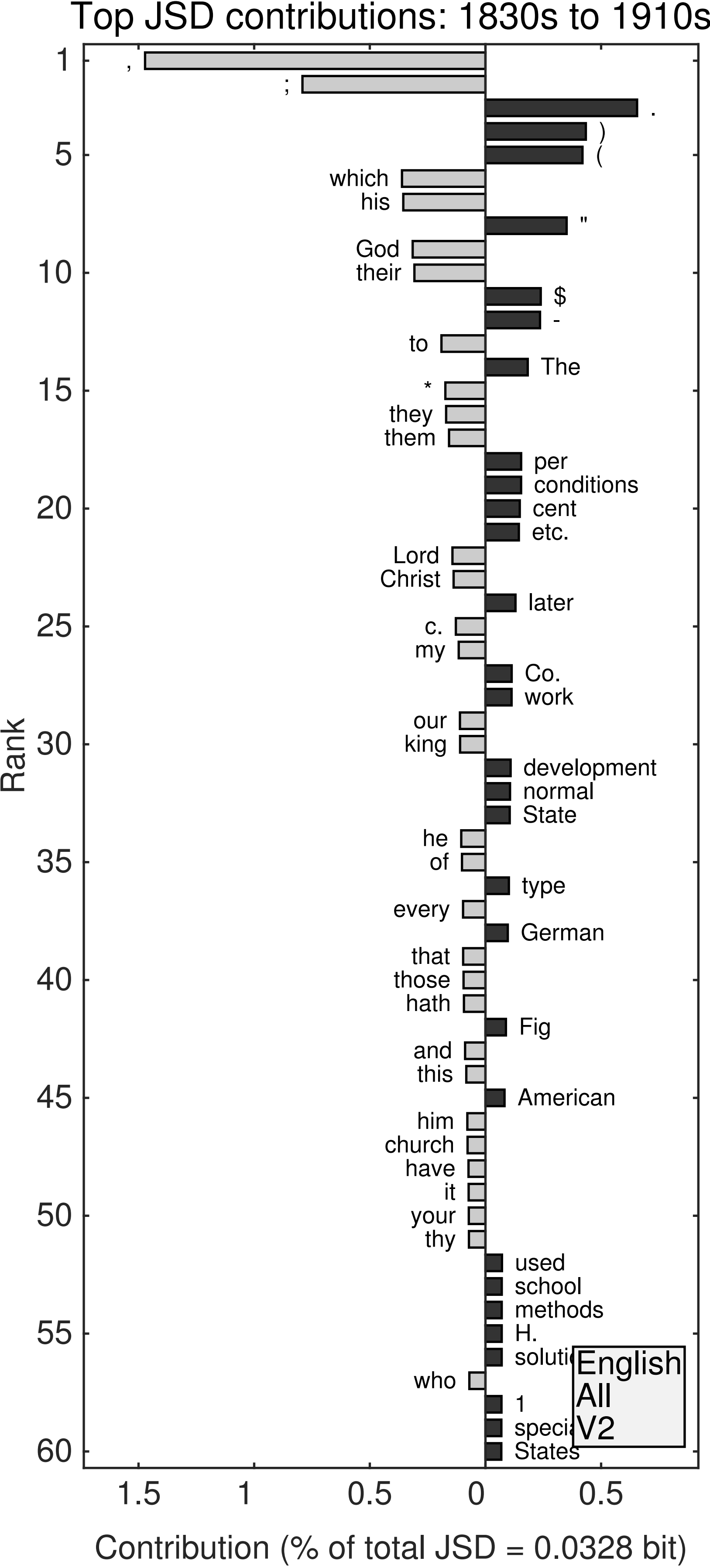
Top JSD contributions: 1830s to 1910s
Rank
Contribution (% of total JSD = 0.0328 bit)
1.5
1
0.5
0
0.5
1
5
10
15
20
25
30
35
40
45
50
55
60
,
;
.
)
(
which
his
"
God
their
$
-
to
The
*
they
them
per
conditions
cent
etc.
Lord
Christ
later
c.
my
Co.
work
our
king
development
normal
State
he
of
type
every
German
that
those
hath
Fig
and
this
American
him
church
have
it
your
thy
used
school
methods
H.
who
1
States
English
All
V2

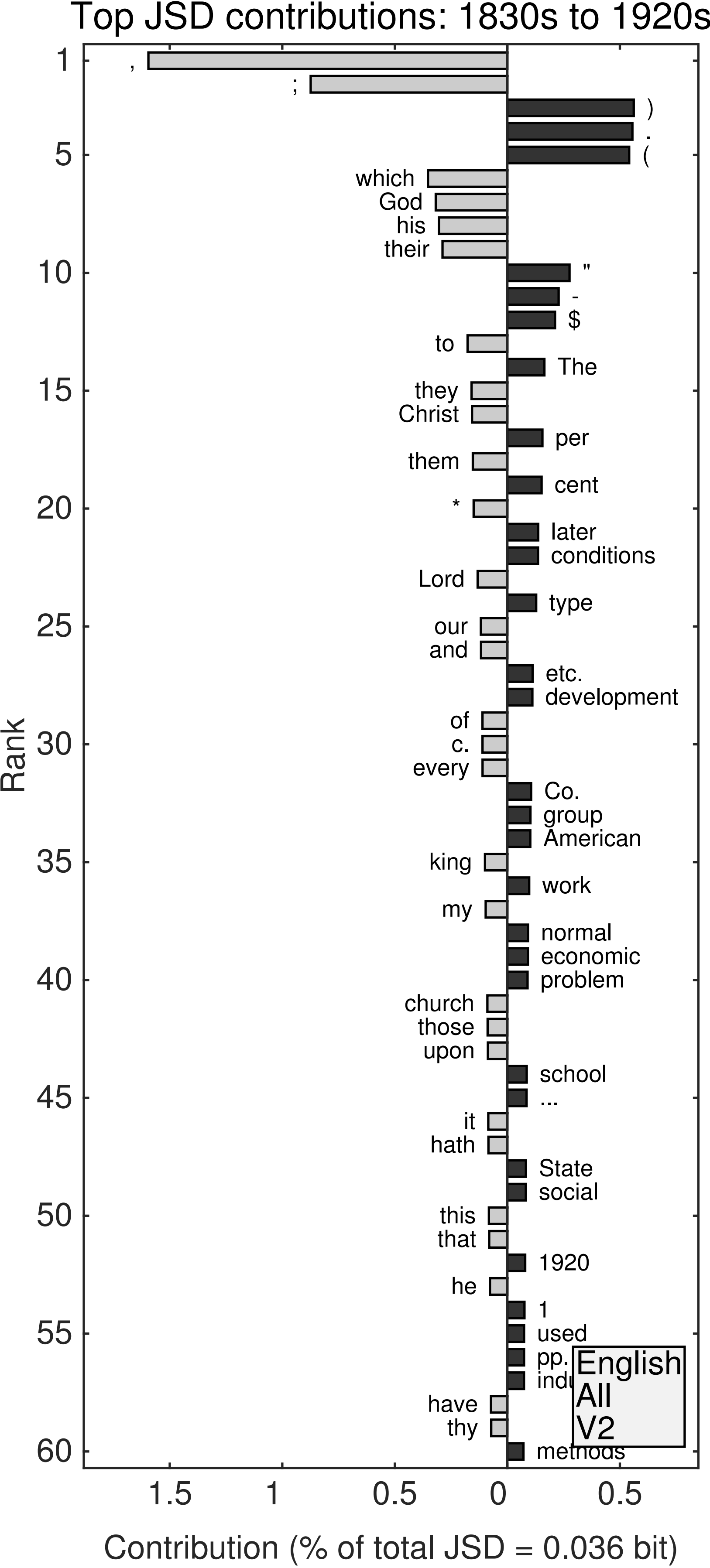

Top JSD contributions: 1830s to 1920s
Rank
Contribution (% of total JSD = 0.036 bit)
1.5
1
0.5
0
0.5
1
5
10
15
20
25
30
35
40
45
50
55
60
,
;
)
.
(
which
God
his
their
"
-
$
to
The
they
Christ
per
them
cent
*
later
conditions
Lord
type
our
and
etc.
development
of
c.
every
Co.
group
American
king
work
my
normal
economic
problem
church
those
upon
school
...
it
hath
State
social
this
that
1920
he
1
used
have
thy
methods
English
All
V2

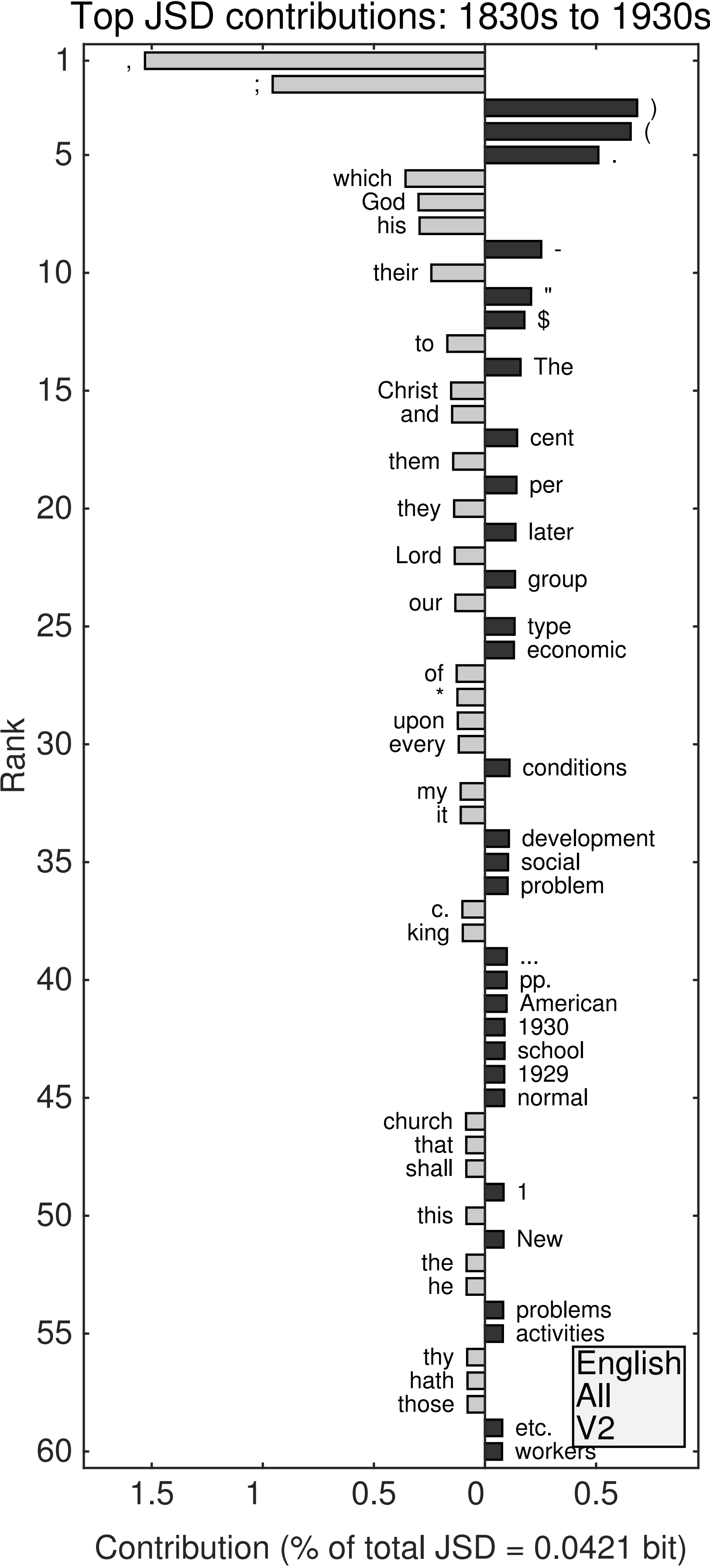
Top JSD contributions: 1830s to 1930s
Rank
Contribution (% of total JSD = 0.0421 bit)
,
;
)
(
.
which
God
his
-
their
"
$
to
The
Christ
and
cent
them
per
they
later
Lord
group
our
type
economic
of
*
upon
every
conditions
my
it
development
social
problem
c.
king
...
pp.
American
1930
school
1929
normal
church
that
shall
1
this
New
the
he
problems
activities
thy
hath
those
etc.
workers
English
All
V2

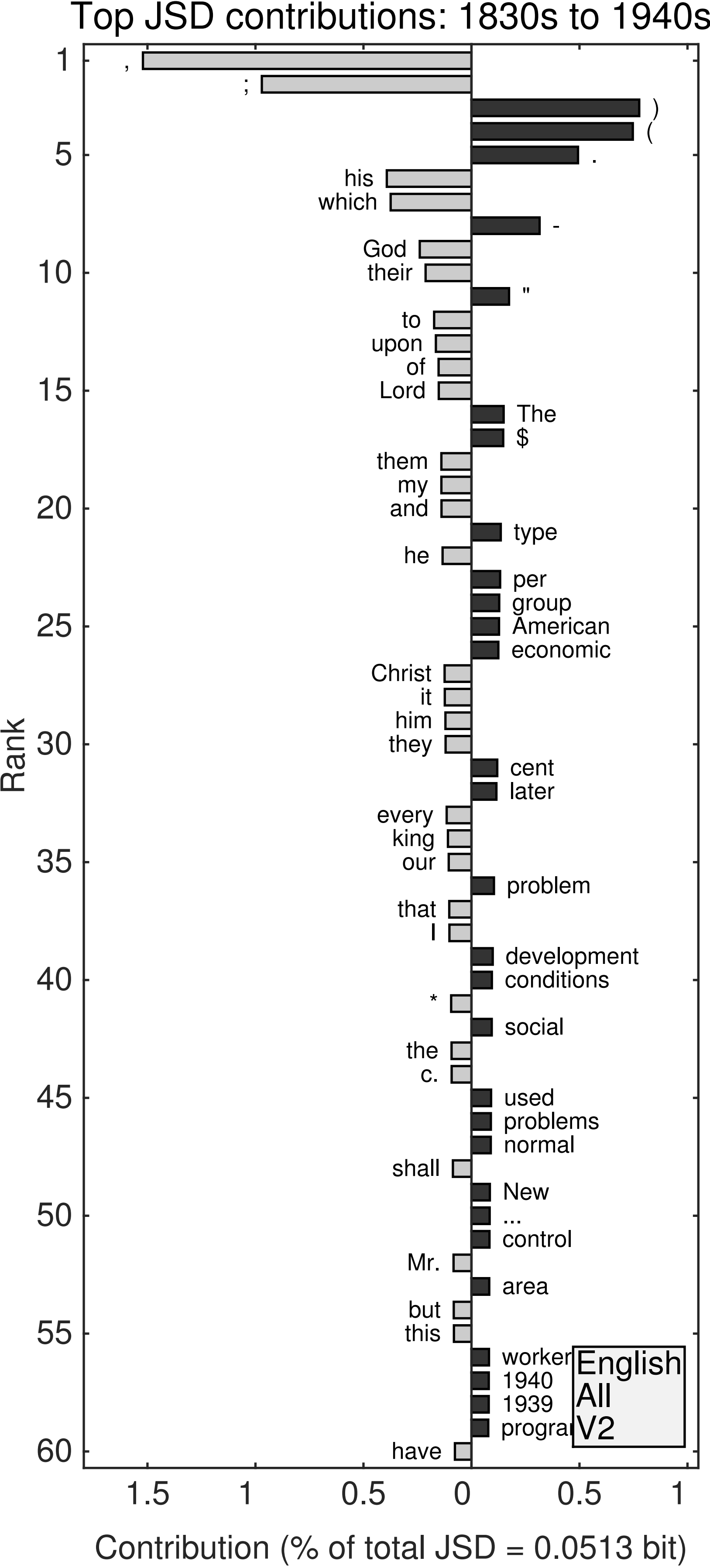
Top JSD contributions: 1830s to 1940s
Rank
1
5
10
15
20
25
30
35
40
45
50
55
60
,
;
)
(
.
his
which
-
God
their
"
to
upon
of
Lord
The
$
them
my
and
type
he
per
group
American
economic
Christ
it
him
they
cent
later
every
king
our
problem
that
I
development
conditions
*
social
the
c.
used
problems
normal
shall
New
...
control
Mr.
area
but
this
worker
1940
1939
progra
have
English
All
V2
1.5
1
0.5
0
0.5
1
Contribution (% of total JSD = 0.0513 bit)

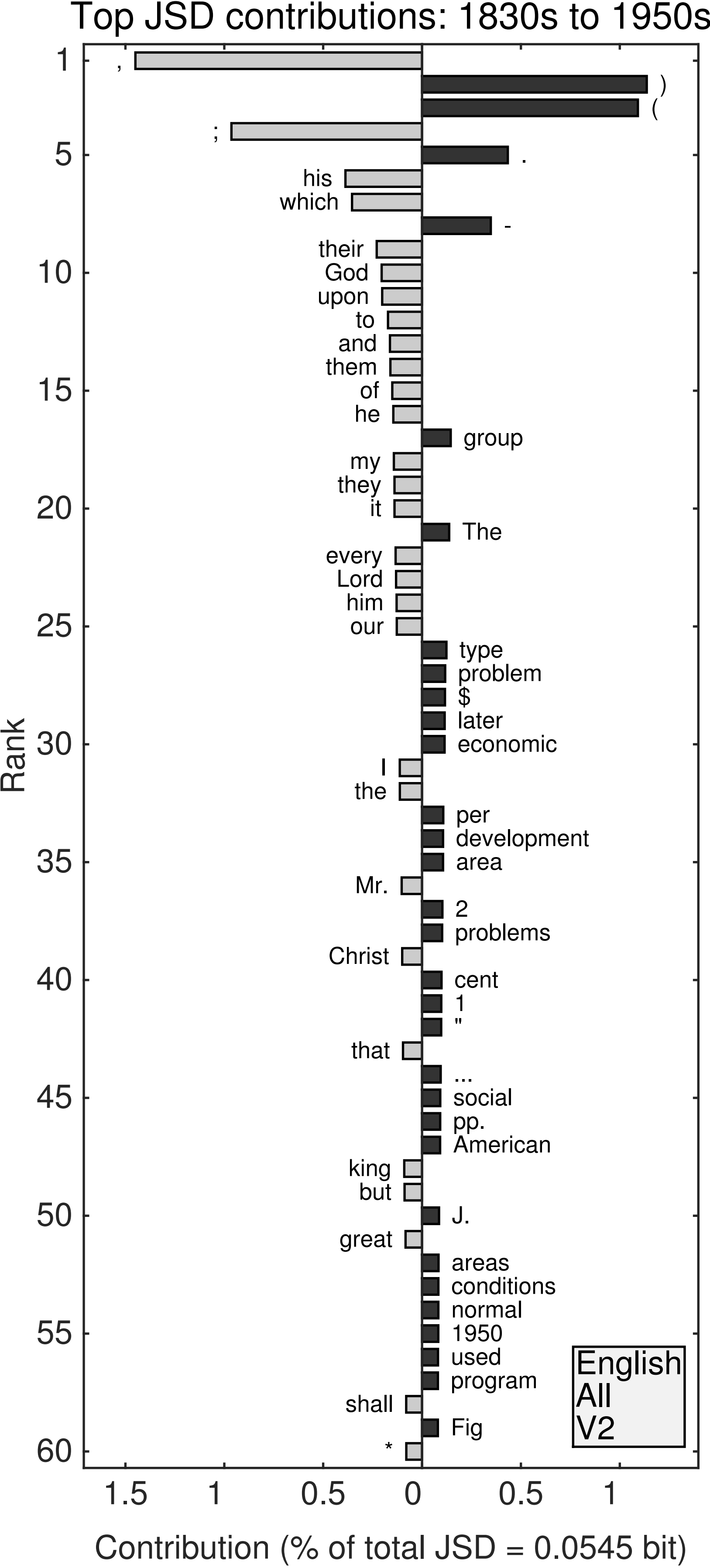

Top JSD contributions: 1830s to 1950s
Rank
Contribution (% of total JSD = 0.0545 bit)
English
All
V2
,
)
(
;
.
his
which
-
their
God
upon
to
and
them
of
he
group
my
they
it
The
every
Lord
him
our
type
problem
$
later
economic
I
the
per
development
area
Mr.
2
problems
Christ
cent
1
"
that
...
social
pp.
American
king
but
J.
great
areas
conditions
normal
1950
used
program
shall
Fig
*

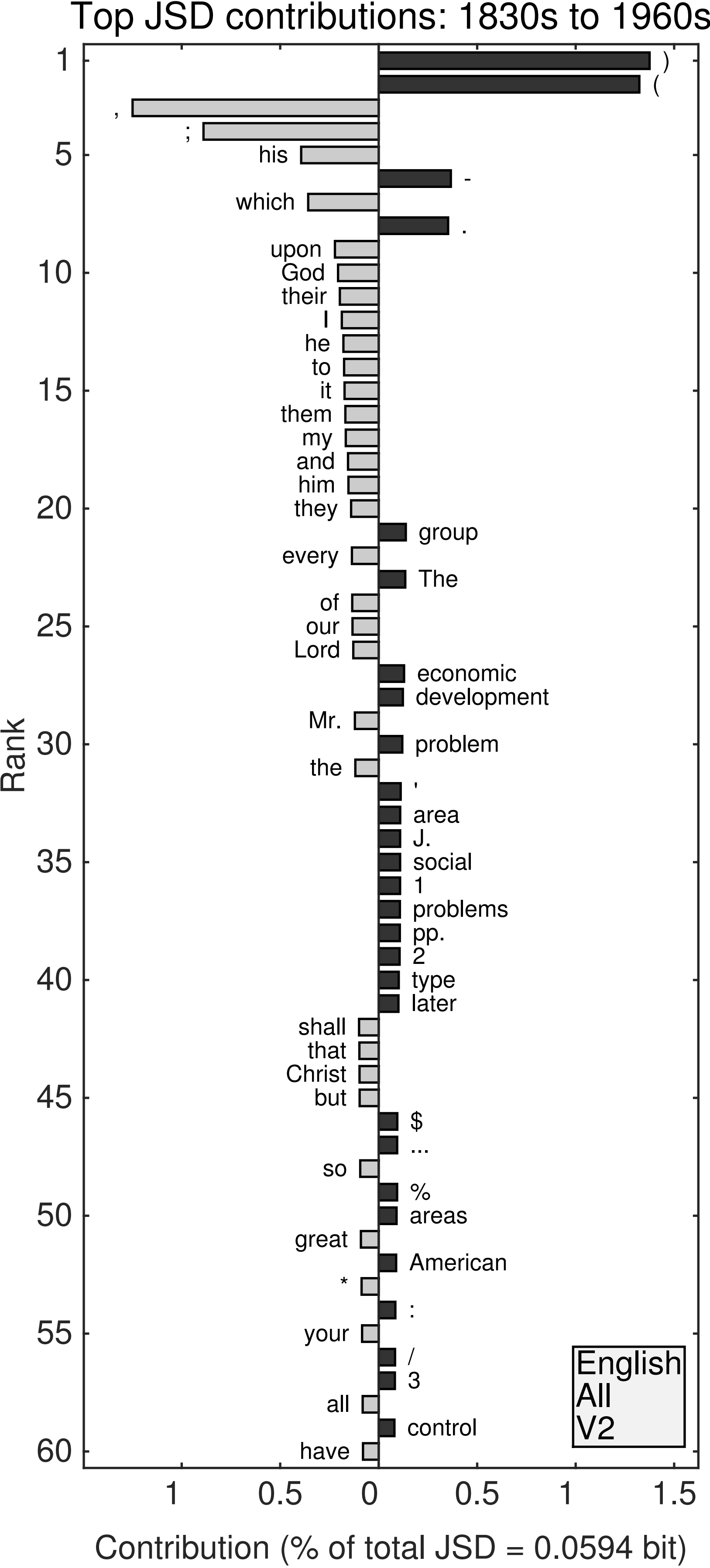

Top JSD contributions: 1830s to 1960s
Rank
Contribution (% of total JSD = 0.0594 bit)
)
(
,
;
his
-
which
.
upon
God
their
I
he
to
it
them
my
and
him
they
group
every
The
of
our
Lord
economic
development
Mr.
problem
the
'
area
J.
social
1
problems
pp.
2
type
later
shall
that
Christ
but
$
...
so
%
areas
great
American
*
:
your
/
3
all
control
have
English
All
V2

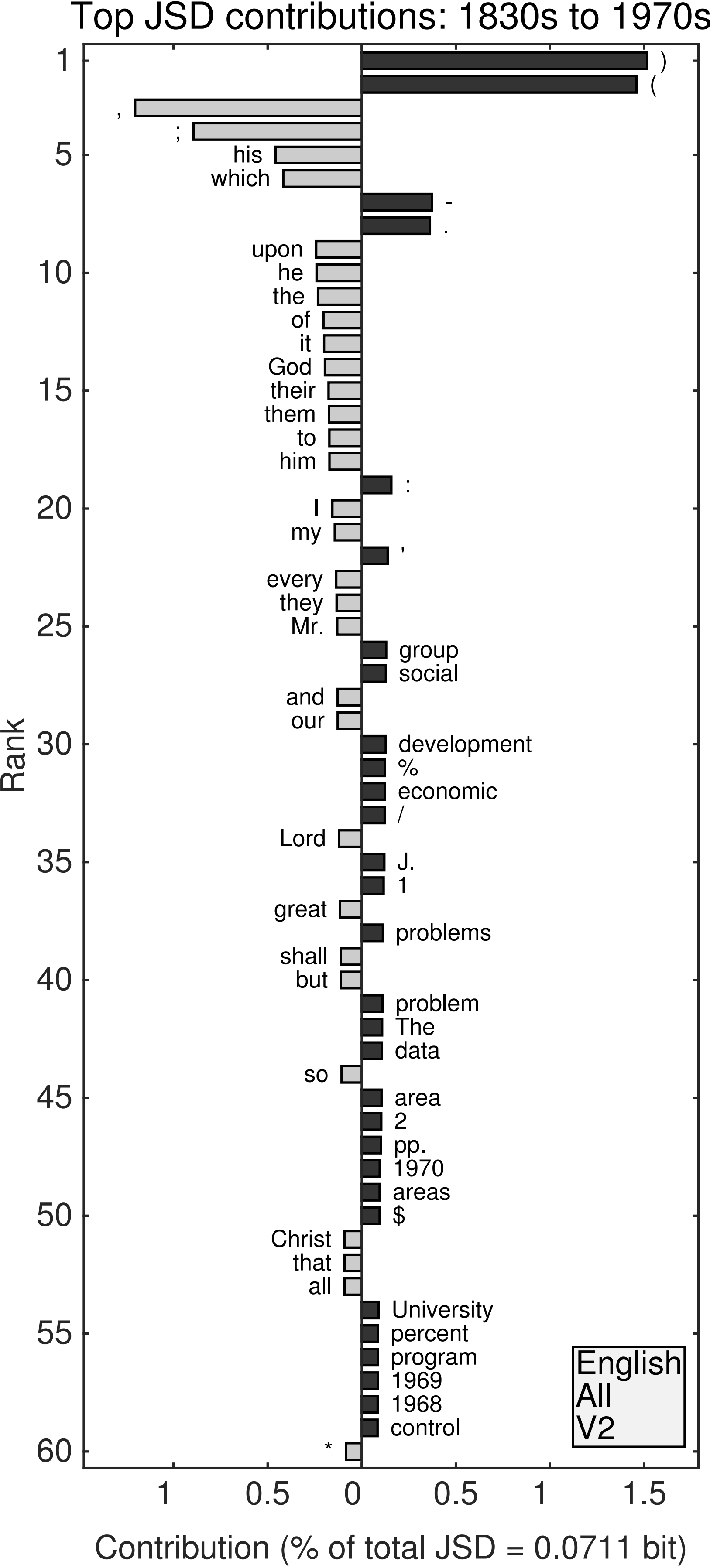
Top JSD contributions: 1830s to 1970s
Rank
Contribution (% of total JSD = 0.0711 bit)
English
All
V2
1
5
10
15
20
25
30
35
40
45
50
55
60
1
0.5
0
0.5
1
1.5
)
(
,
;
his
which
-
.
upon
he
the
of
it
God
their
them
to
him
:
I
my
'
every
they
Mr.
group
social
and
our
development
%
economic
/
Lord
J.
1
great
problems
shall
but
problem
The
data
so
area
2
pp.
1970
areas
$
Christ
that
all
University
percent
program
1969
1968
control
*

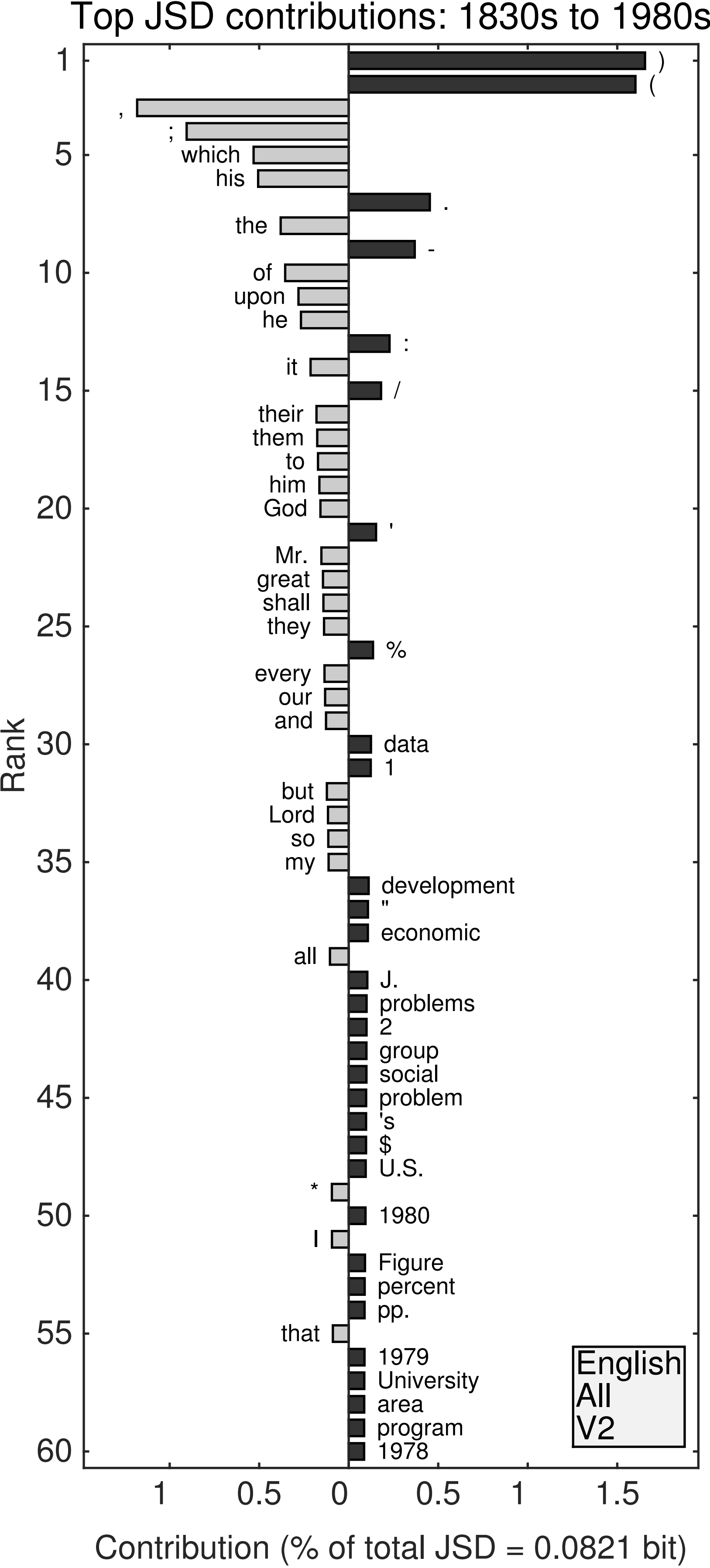

Top JSD contributions: 1830s to 1980s
Rank
Contribution (% of total JSD = 0.0821 bit)
)
(
,
;
which
his
.
the
-
of
upon
he
:
it
/
their
them
to
him
God
'
Mr.
great
shall
they
%
every
our
and
data
1
but
Lord
so
my
development
"
economic
all
J.
problems
2
group
social
problem
's
$
U.S.
*
1980
I
Figure
percent
pp.
that
1979
University
area
program
1978
English
All
V2

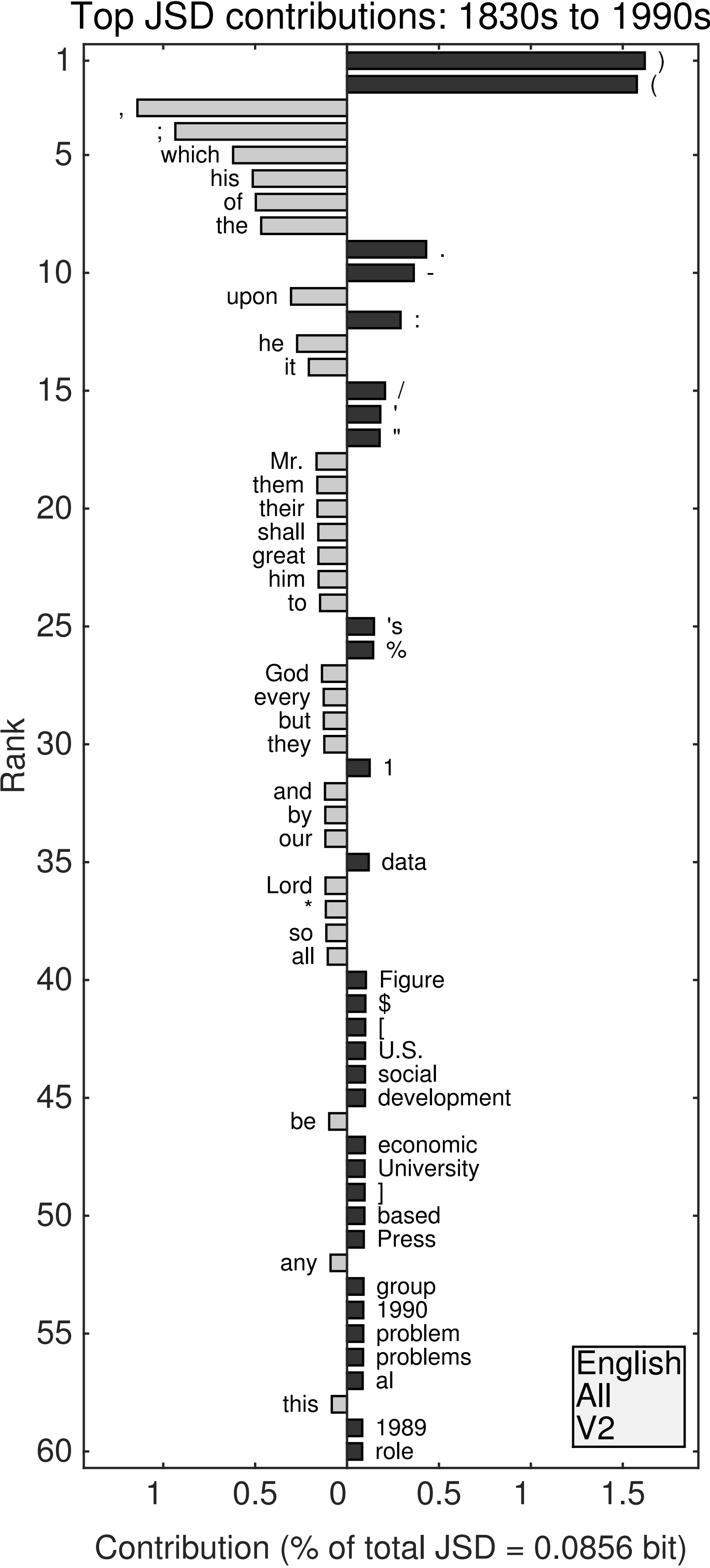

Top JSD contributions: 1830s to 1990s
Rank
Contribution (% of total JSD = 0.0856 bit)
English
All
V2
)
(
,
;
which
his
of
the
.
-
upon
:
he
it
/
'
"
Mr.
them
their
shall
great
him
to
's
%
God
every
but
they
1
and
by
our
data
Lord
*
so
all
Figure
$
[
U.S.
social
development
be
economic
University
]
based
Press
any
group
1990
problem
problems
al
this
1989
role

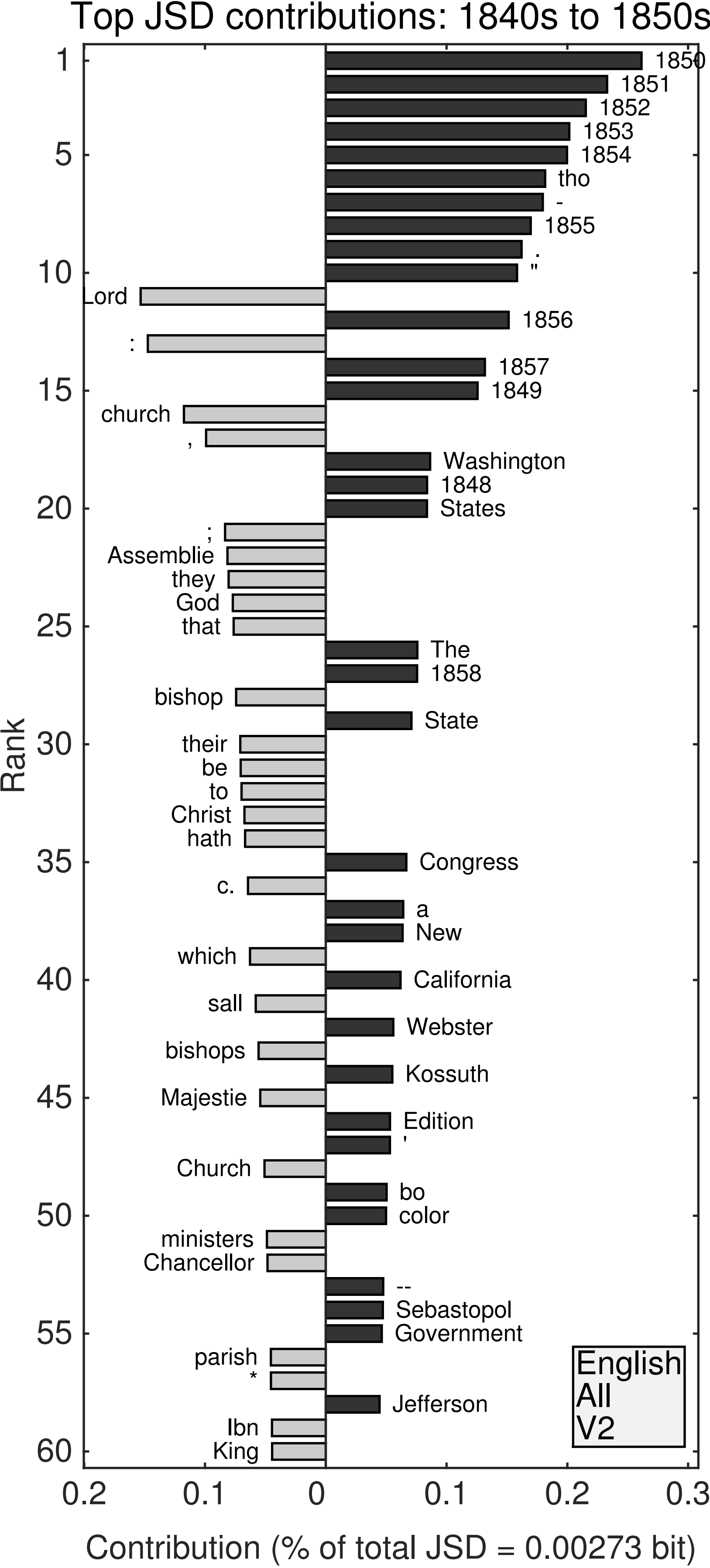
Top JSD contributions: 1840s to 1850s
Rank
Contribution (% of total JSD = 0.00273 bit)
1850
1851
1852
1853
1854
tho
-
1855
.
"
Lord
1856
:
1857
1849
church
,
Washington
1848
States
;
Assemblie
they
God
that
The
1858
bishop
State
their
be
to
Christ
hath
Congress
c.
a
New
which
California
sall
Webster
bishops
Kossuth
Majestie
Edition
'
Church
bo
color
ministers
Chancellor
--
Sebastopol
Government
parish
*
Jefferson
Ibn
King
English
All
V2

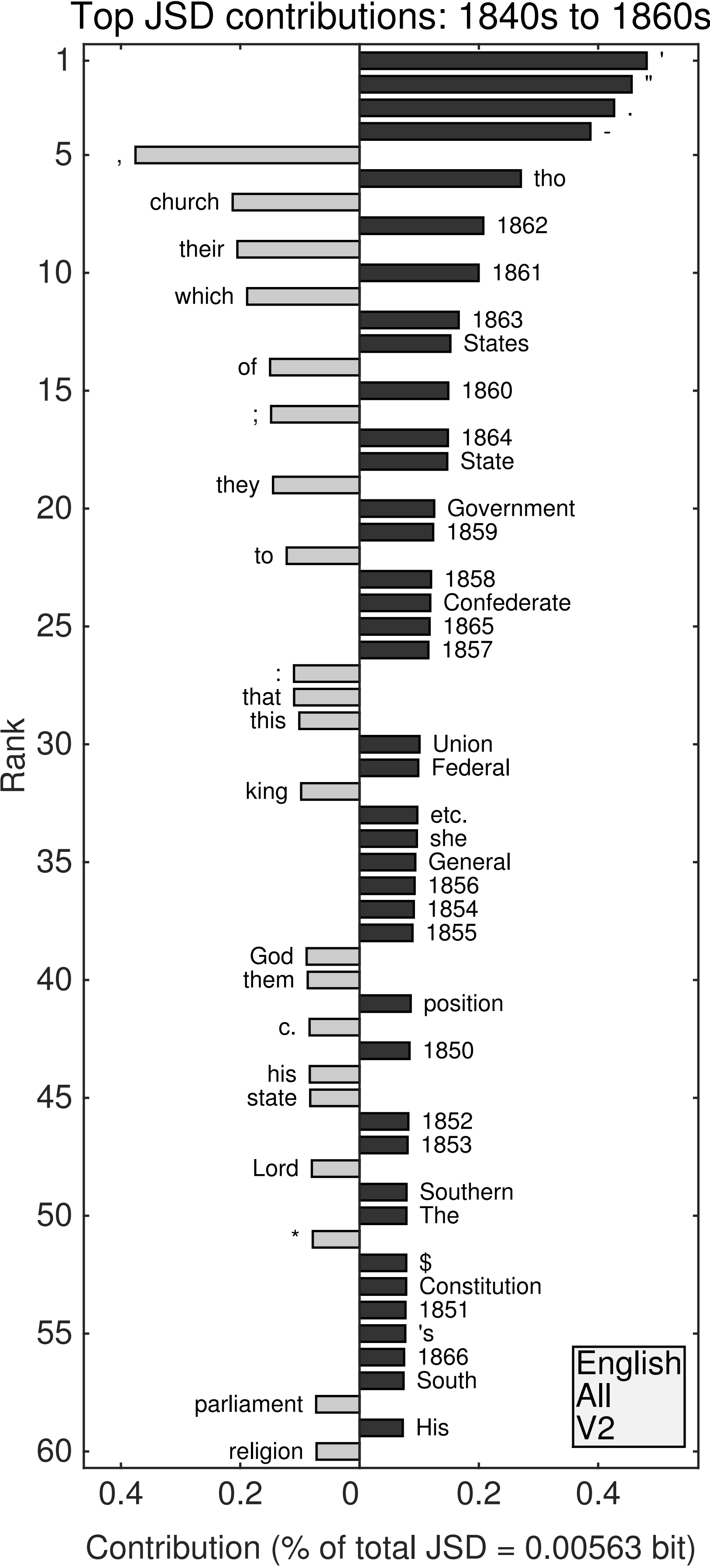

Top JSD contributions: 1840s to 1860s
Rank
1
5
10
15
20
25
30
35
40
45
50
55
60
'
"
.
-
,
tho
church
1862
their
1861
which
1863
States
of
1860
;
1864
State
they
Government
1859
to
1858
Confederate
1865
1857
:
that
this
Union
Federal
king
etc.
she
General
1856
1854
1855
God
them
position
c.
1850
his
state
1852
1853
Lord
Southern
The
*
$
Constitution
1851
's
1866
South
parliament
His
religion
English
All
V2
0.4
0.2
0
0.2
0.4
Contribution (% of total JSD = 0.00563 bit)

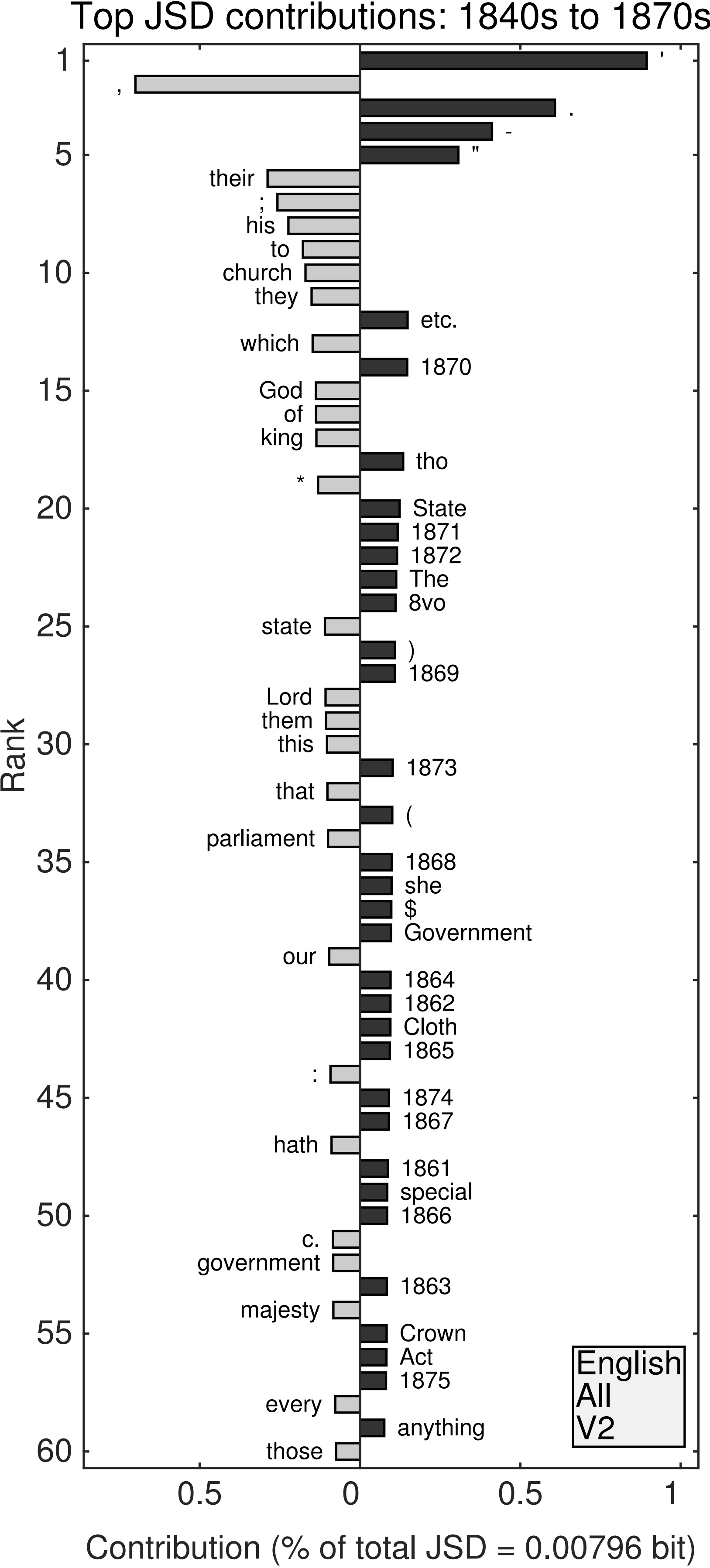
Top JSD contributions: 1840s to 1870s
Rank
1
5
10
15
20
25
30
35
40
45
50
55
60
'
,
.
-
"
their
;
his
to
church
they
etc.
which
1870
God
of
king
tho
*
State
1871
1872
The
8vo
state
)
1869
Lord
them
this
1873
that
(
parliament
1868
she
$
Government
our
1864
1862
Cloth
1865
:
1874
1867
hath
1861
special
1866
c.
government
1863
majesty
Crown
Act
1875
every
anything
those
English
All
V2
0.5
0
0.5
1
Contribution (% of total JSD = 0.00796 bit)

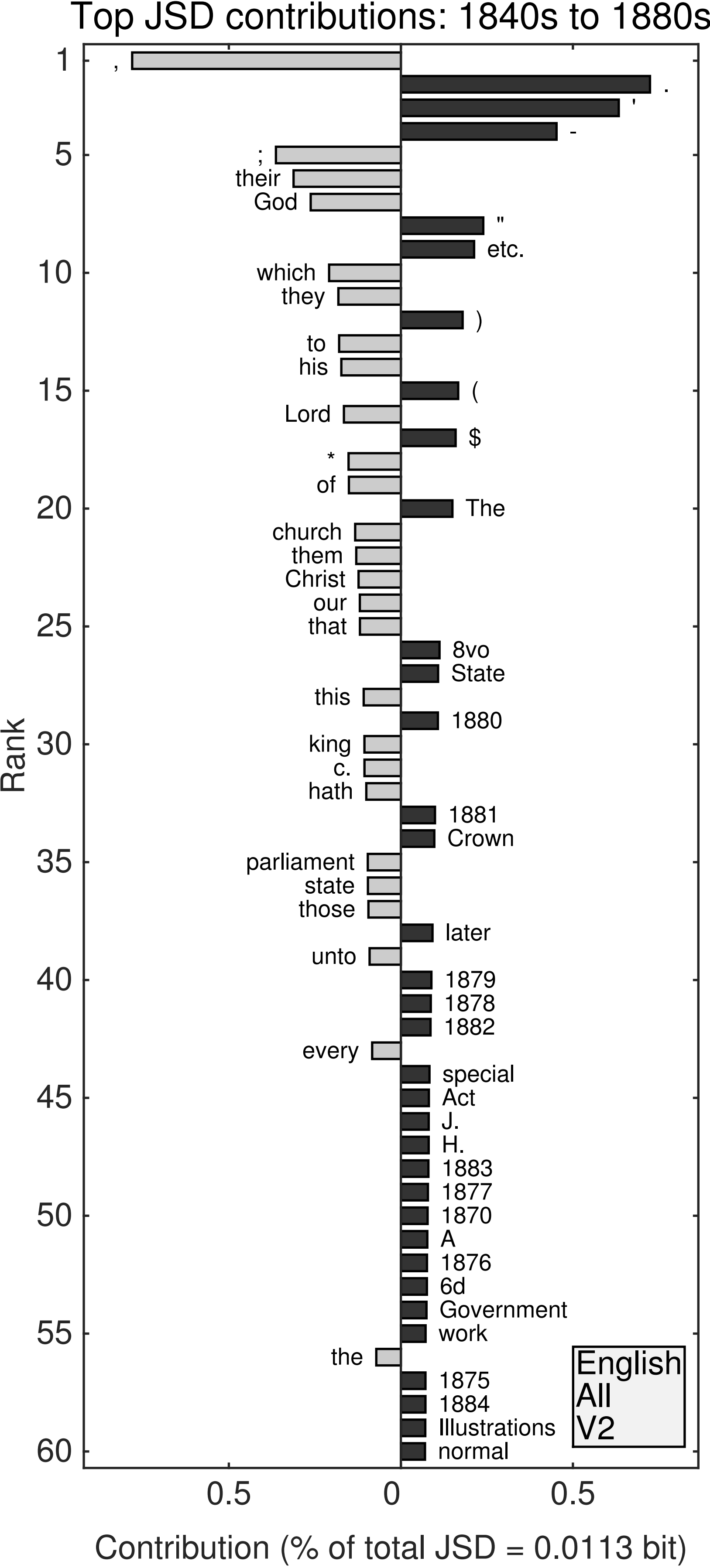
Top JSD contributions: 1840s to 1880s
Rank
Contribution (% of total JSD = 0.0113 bit)
0.5
0
0.5
1
5
10
15
20
25
30
35
40
45
50
55
60
,
.
'
-
;
their
God
"
etc.
which
they
)
to
his
(
Lord
$
*
of
The
church
them
Christ
our
that
8vo
State
this
1880
king
c.
hath
1881
Crown
parliament
state
those
later
unto
1879
1878
1882
every
special
Act
J.
H.
1883
1877
1870
A
1876
6d
Government
work
the
1875
1884
Illustrations
normal
English
All
V2

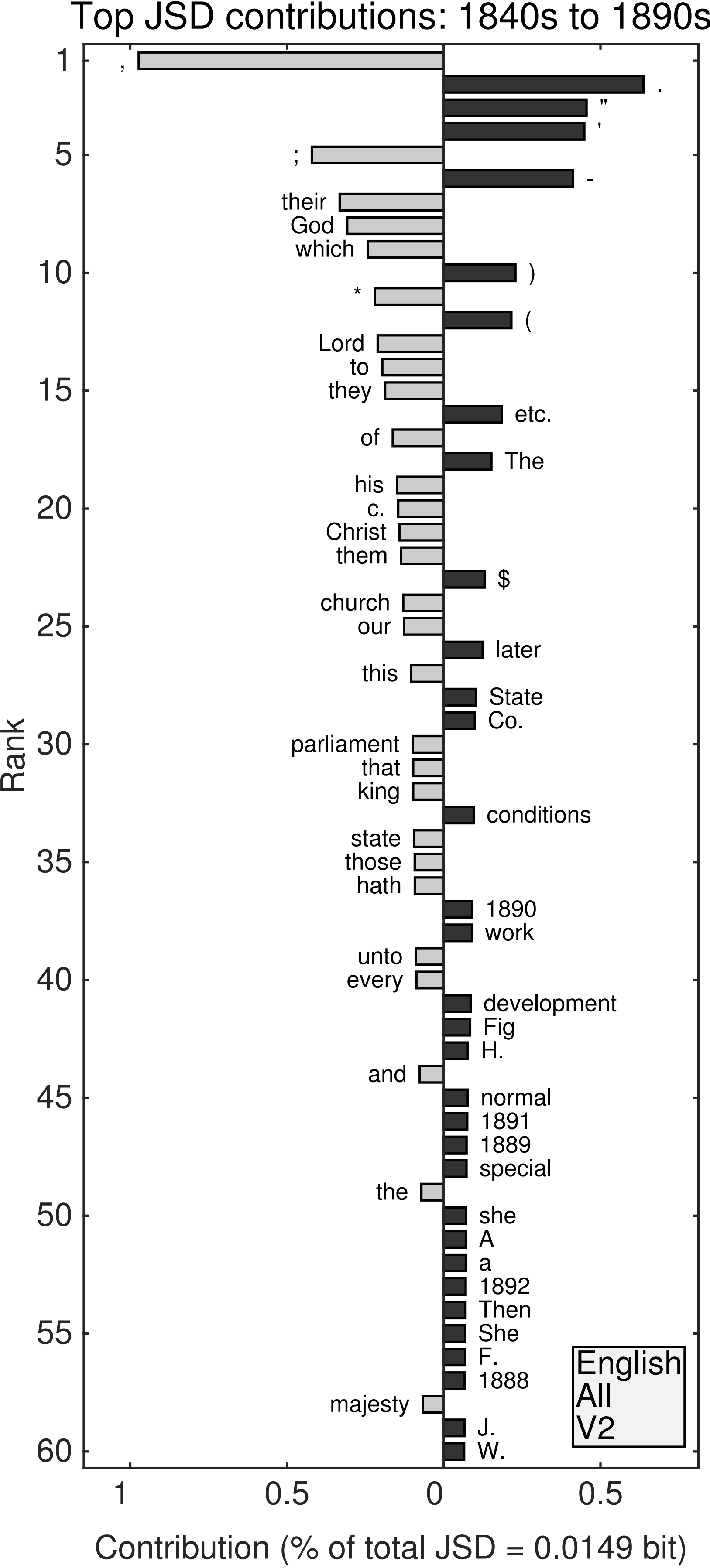
Top JSD contributions: 1840s to 1890s
Rank
Contribution (% of total JSD = 0.0149 bit)
1
5
10
15
20
25
30
35
40
45
50
55
60
1
0.5
0
0.5
,
.
"
'
;
-
their
God
which
)
*
(
Lord
to
they
etc.
of
The
his
c.
Christ
them
$
church
our
later
this
State
Co.
parliament
that
king
conditions
state
those
hath
1890
work
unto
every
development
Fig
H.
and
normal
1891
1889
special
the
she
A
a
1892
Then
She
F.
1888
majesty
J.
W.
English
All
V2

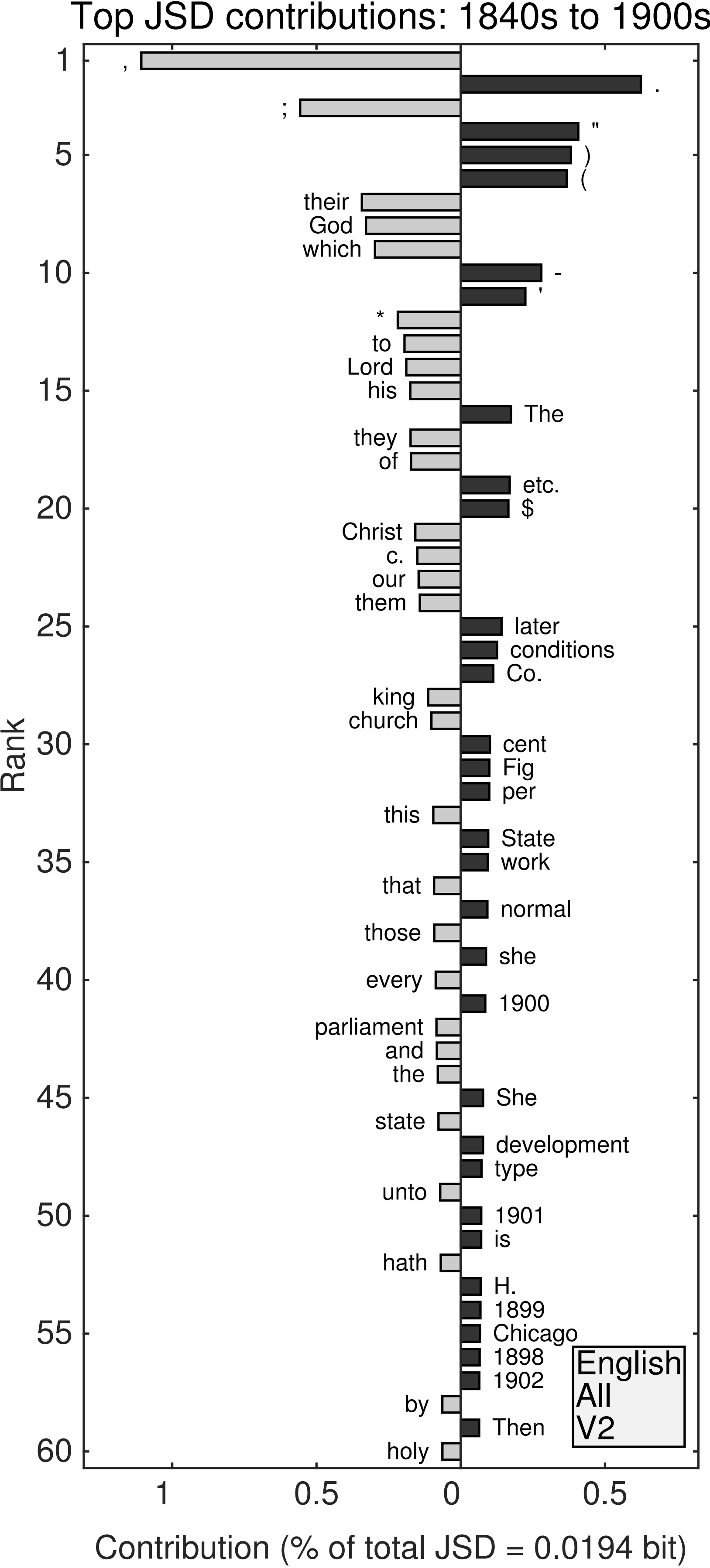
Top JSD contributions: 1840s to 1900s
Rank
Contribution (% of total JSD = 0.0194 bit)
1
5
10
15
20
25
30
35
40
45
50
55
60
1
0.5
0
0.5
,
.
;
"
)
(
their
God
which
-
'
*
to
Lord
his
The
they
of
etc.
$
Christ
c.
our
them
later
conditions
Co.
king
church
cent
Fig
per
this
State
work
that
normal
those
she
every
1900
parliament
and
the
She
state
development
type
unto
1901
is
hath
H.
1899
Chicago
1898
1902
by
Then
holy
English
All
V2

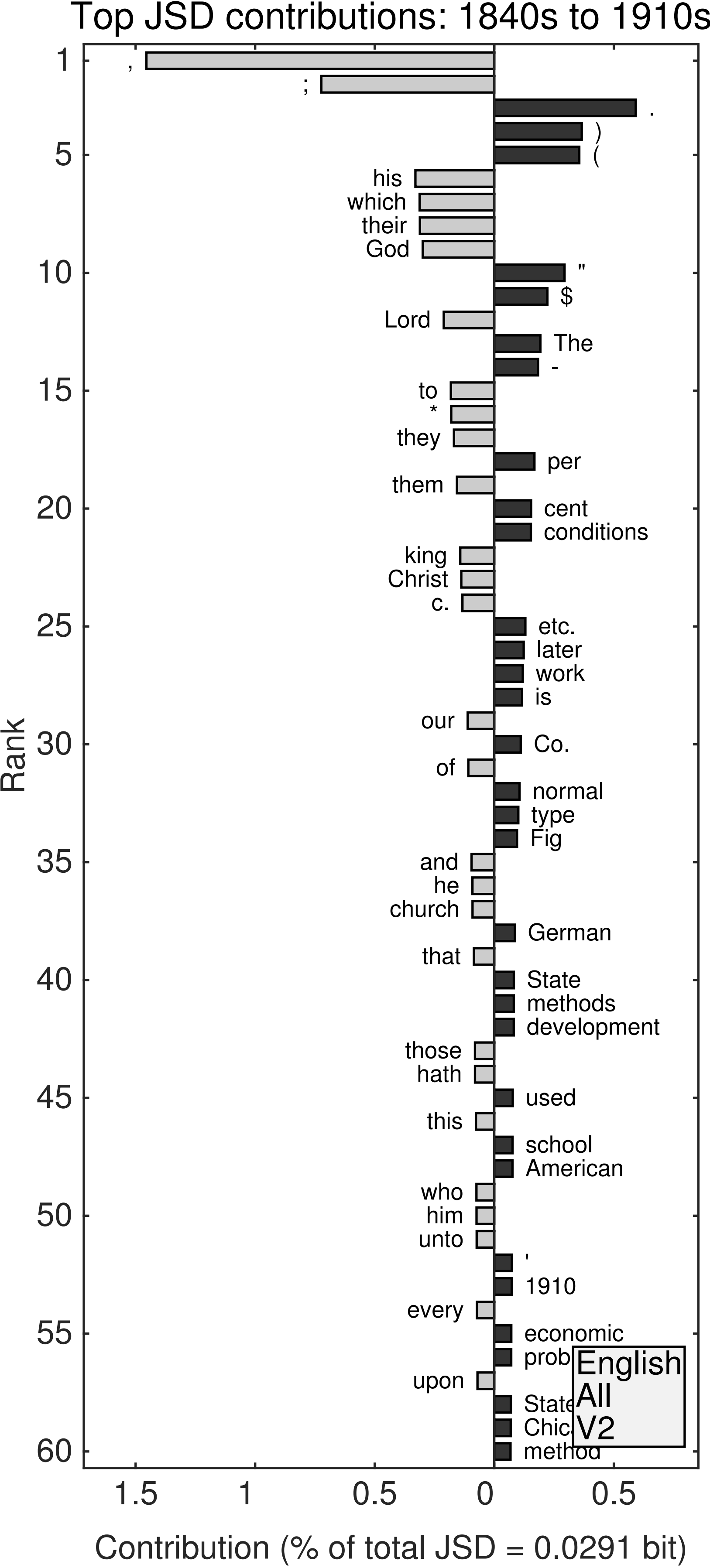

Top JSD contributions: 1840s to 1910s
Rank
Contribution (% of total JSD = 0.0291 bit)
1.5
1
0.5
0
0.5
1
5
10
15
20
25
30
35
40
45
50
55
60
,
;
.
)
(
his
which
their
God
"
$
Lord
The
-
to
*
they
per
them
cent
conditions
king
Christ
c.
etc.
later
work
is
our
Co.
of
normal
type
Fig
and
he
church
German
that
State
methods
development
those
hath
used
this
school
American
who
him
unto
'
1910
every
economic
upon
English
All
V2

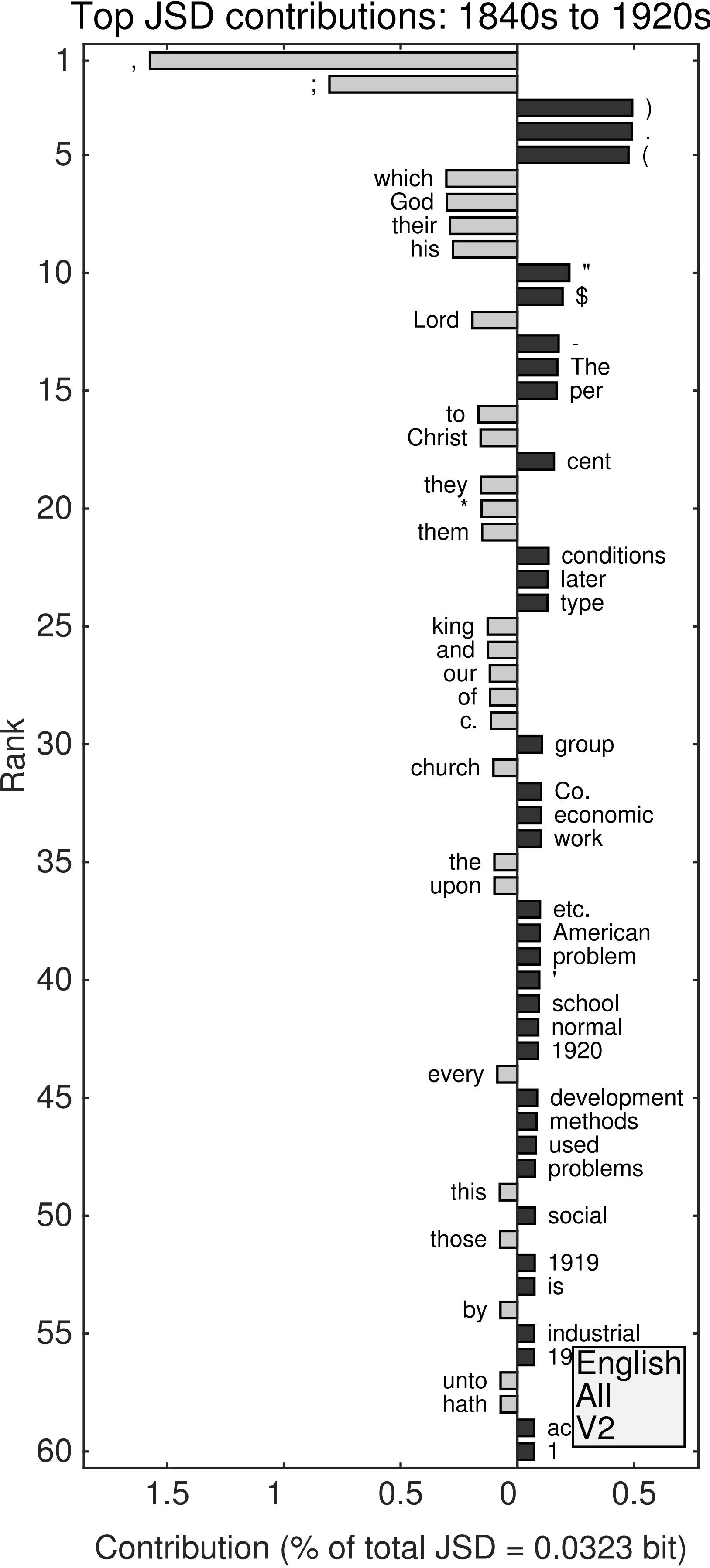

Top JSD contributions: 1840s to 1920s
Rank
Contribution (% of total JSD = 0.0323 bit)
1.5
1
0.5
0
0.5
1
5
10
15
20
25
30
35
40
45
50
55
60
,
;
)
.
(
which
God
their
his
"
$
Lord
-
The
per
to
Christ
cent
they
*
them
conditions
later
type
king
and
our
of
c.
group
church
Co.
economic
work
the
upon
etc.
American
problem
'
school
normal
1920
every
development
methods
used
problems
this
social
those
1919
is
by
industrial
unto
hath
1
English
All
V2

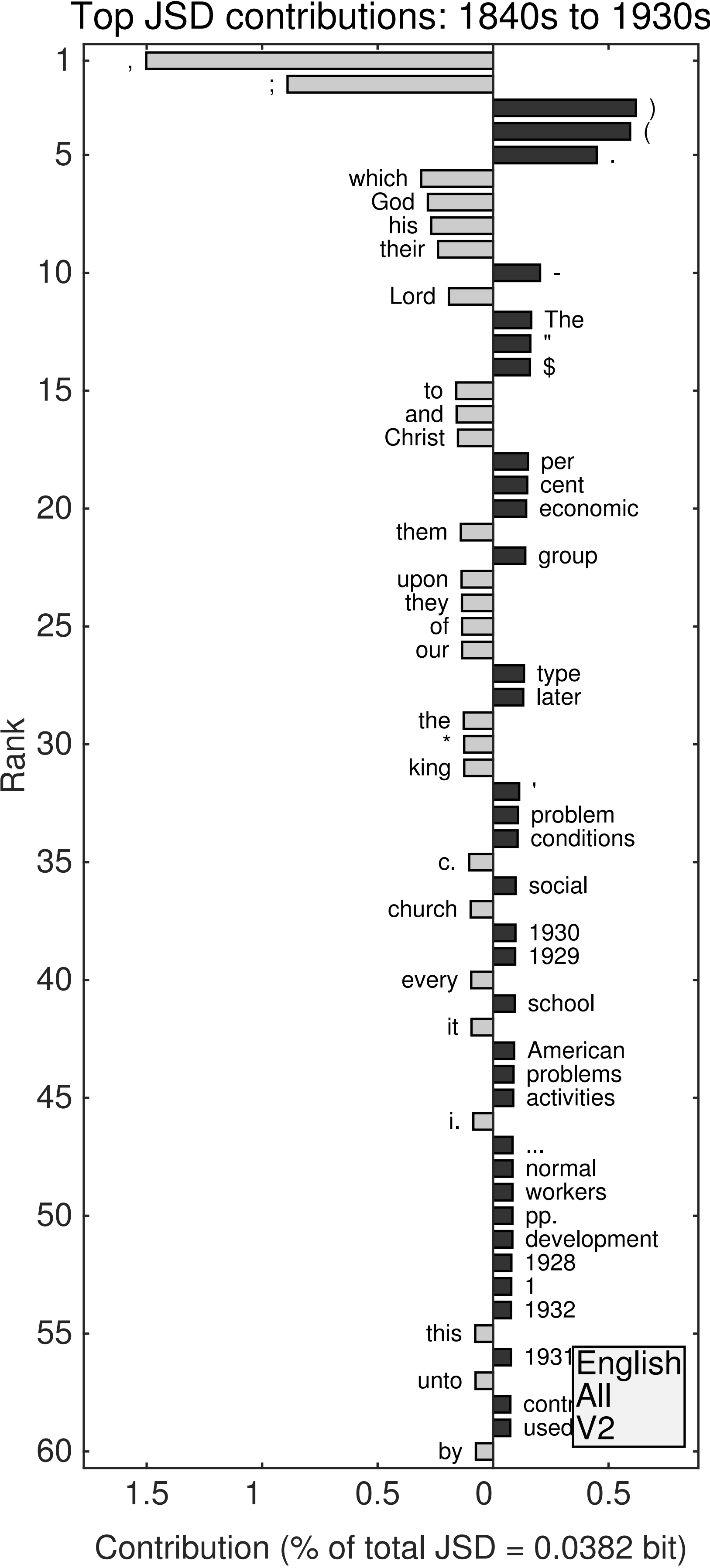

Top JSD contributions: 1840s to 1930s
Rank
Contribution (% of total JSD = 0.0382 bit)
1.5
1
0.5
0
0.5
1
5
10
15
20
25
30
35
40
45
50
55
60
,
;
)
(
.
which
God
his
their
-
Lord
The
"
$
to
and
Christ
per
cent
economic
them
group
upon
they
of
our
type
later
the
*
king
'
problem
conditions
c.
social
church
1930
1929
every
school
it
American
problems
activities
i.
...
normal
workers
pp.
development
1928
1
1932
this
1931
unto
contr
used
by
English
All
V2

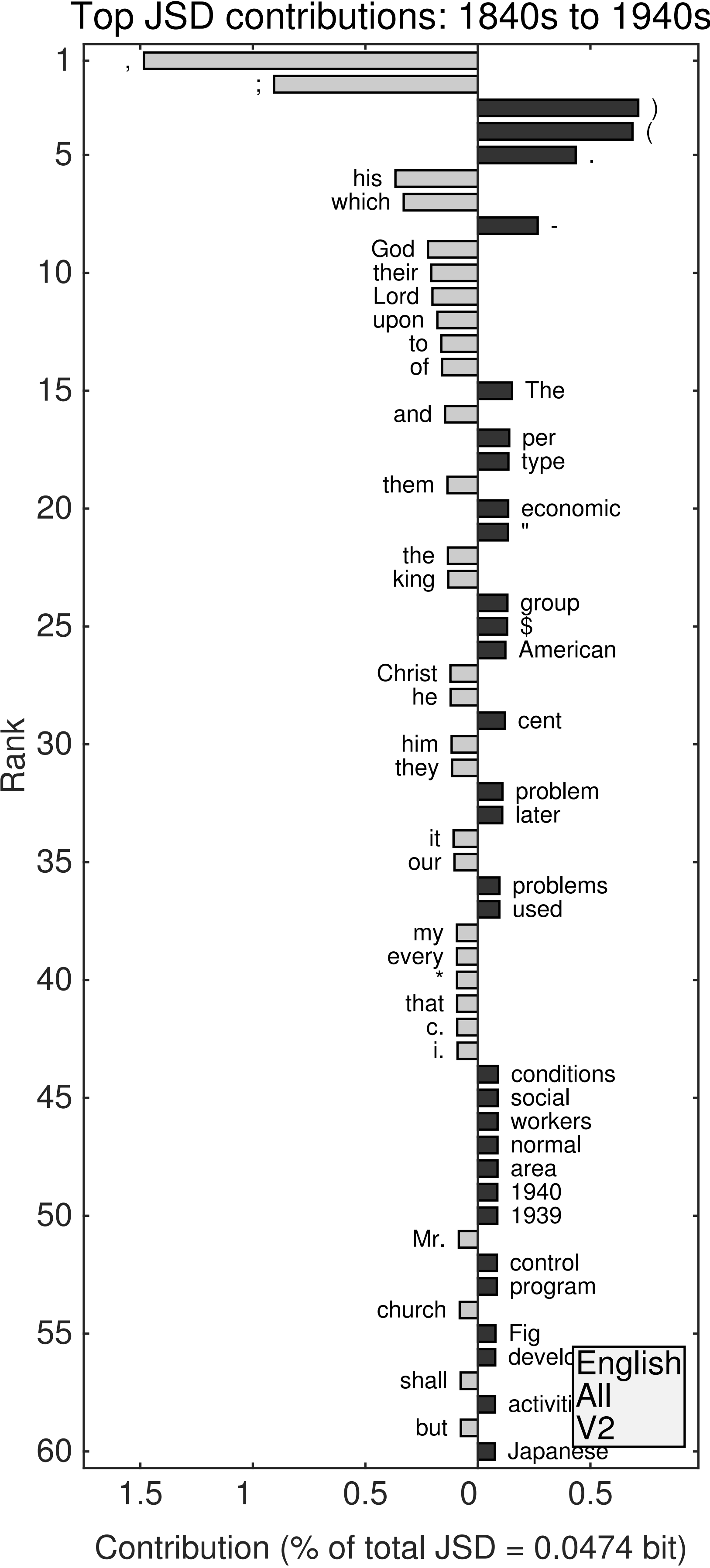

Top JSD contributions: 1840s to 1940s
Rank
Contribution (% of total JSD = 0.0474 bit)
1.5
1
0.5
0
0.5
1
5
10
15
20
25
30
35
40
45
50
55
60
,
;
)
(
.
his
which
-
God
their
Lord
upon
to
of
The
and
per
type
them
economic
"
the
king
group
$
American
Christ
he
cent
him
they
problem
later
it
our
problems
used
my
every
*
that
c.
i.
conditions
social
workers
normal
area
1940
1939
Mr.
control
program
church
Fig
shall
but
Japanese
English
All
V2

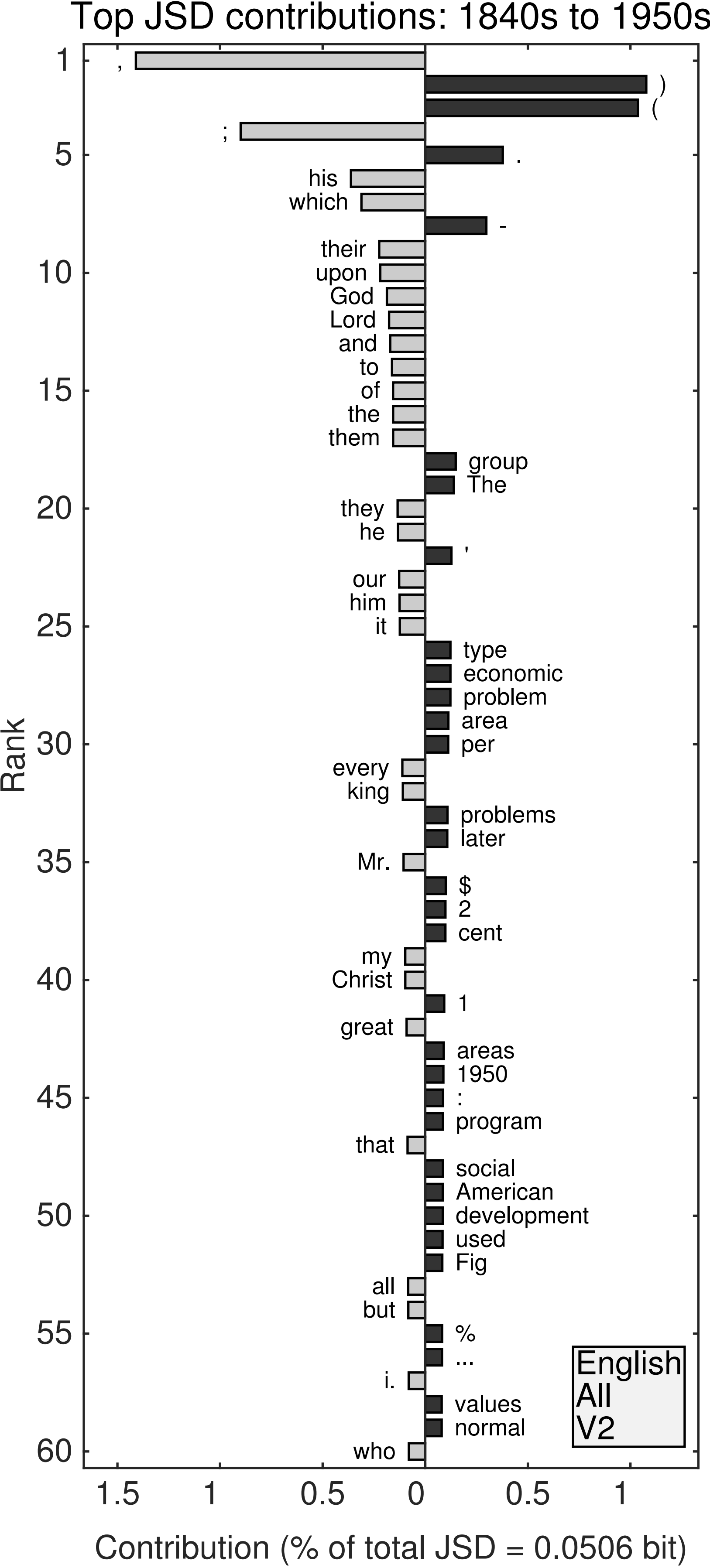
Top JSD contributions: 1840s to 1950s
Rank
Contribution (% of total JSD = 0.0506 bit)
,
)
(
;
.
his
which
-
their
upon
God
Lord
and
to
of
the
them
group
The
they
he
'
our
him
it
type
economic
problem
area
per
every
king
problems
later
Mr.
$
2
cent
my
Christ
1
great
areas
1950
:
program
that
social
American
development
used
Fig
all
but
%
...
i.
values
normal
who
English
All
V2

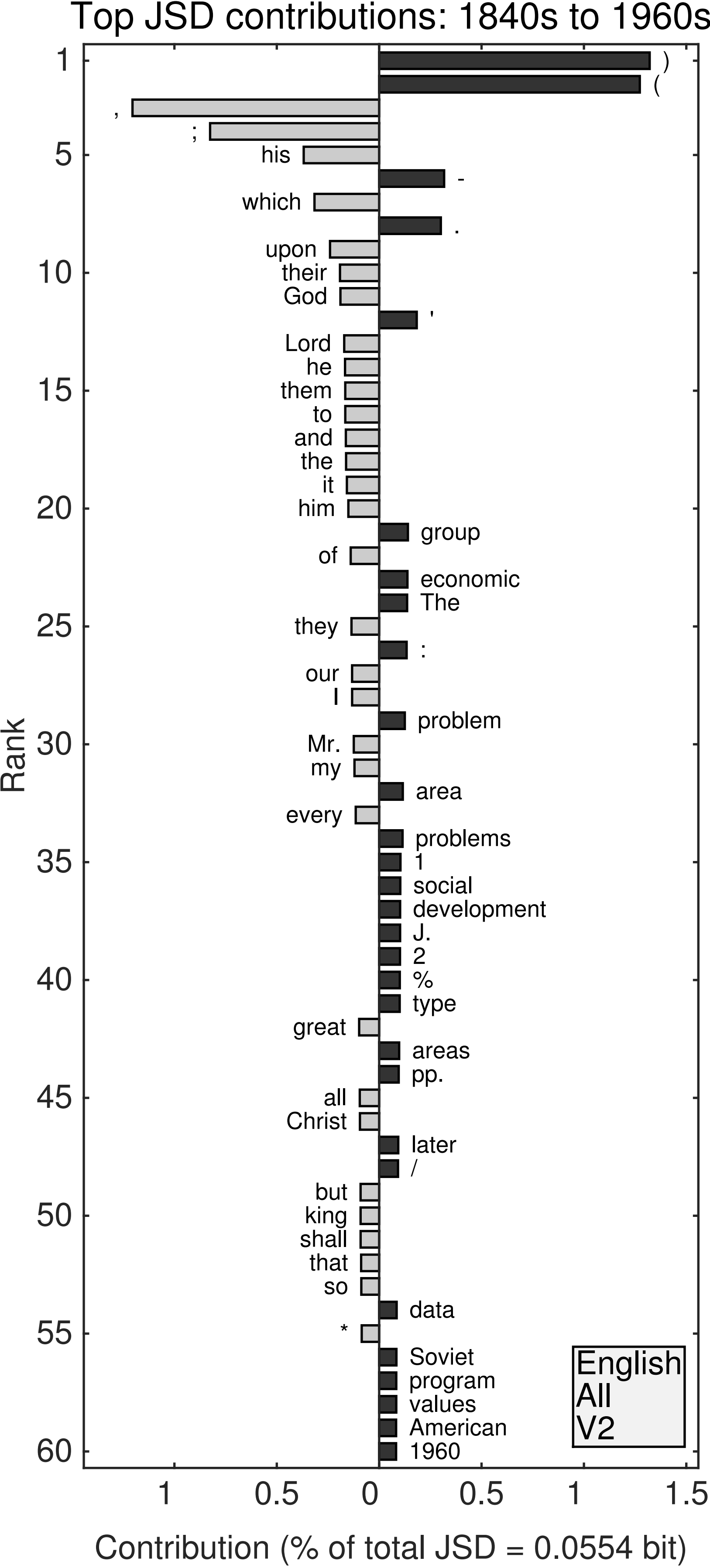

Top JSD contributions: 1840s to 1960s
Rank
Contribution (% of total JSD = 0.0554 bit)
)
(
,
;
his
-
which
.
upon
their
God
'
Lord
he
them
to
and
the
it
him
group
of
economic
The
they
:
our
I
problem
Mr.
my
area
every
problems
1
social
development
J.
2
%
type
great
areas
pp.
all
Christ
later
/
but
king
shall
that
so
data
*
Soviet
program
values
American
1960
English
All
V2

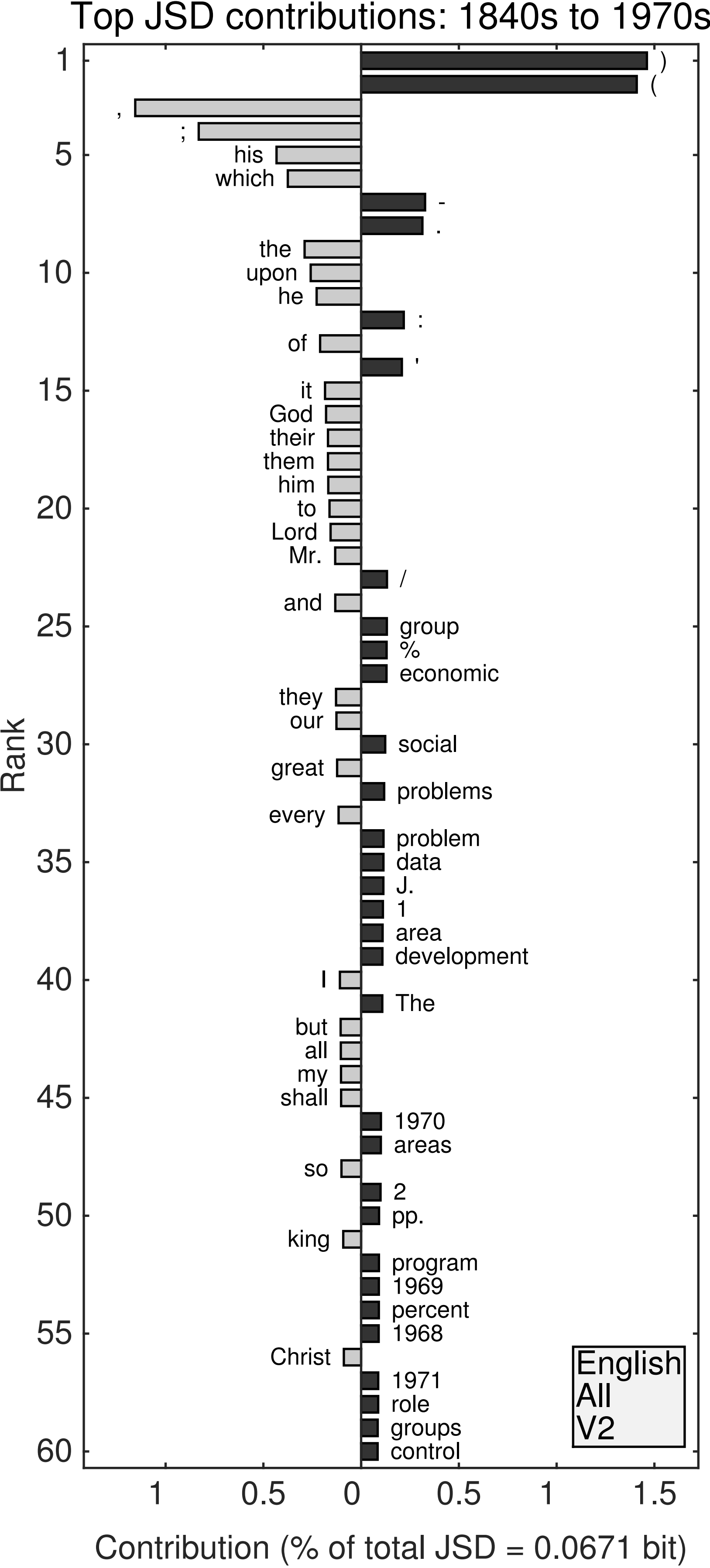
Top JSD contributions: 1840s to 1970s
Rank
Contribution (% of total JSD = 0.0671 bit)
English
All
V2
)
(
,
;
his
which
-
.
the
upon
he
:
of
'
it
God
their
them
him
to
Lord
Mr.
/
and
group
%
economic
they
our
social
great
problems
every
problem
data
J.
1
area
development
I
The
but
all
my
shall
1970
areas
so
2
pp.
king
program
1969
percent
1968
Christ
1971
role
groups
control

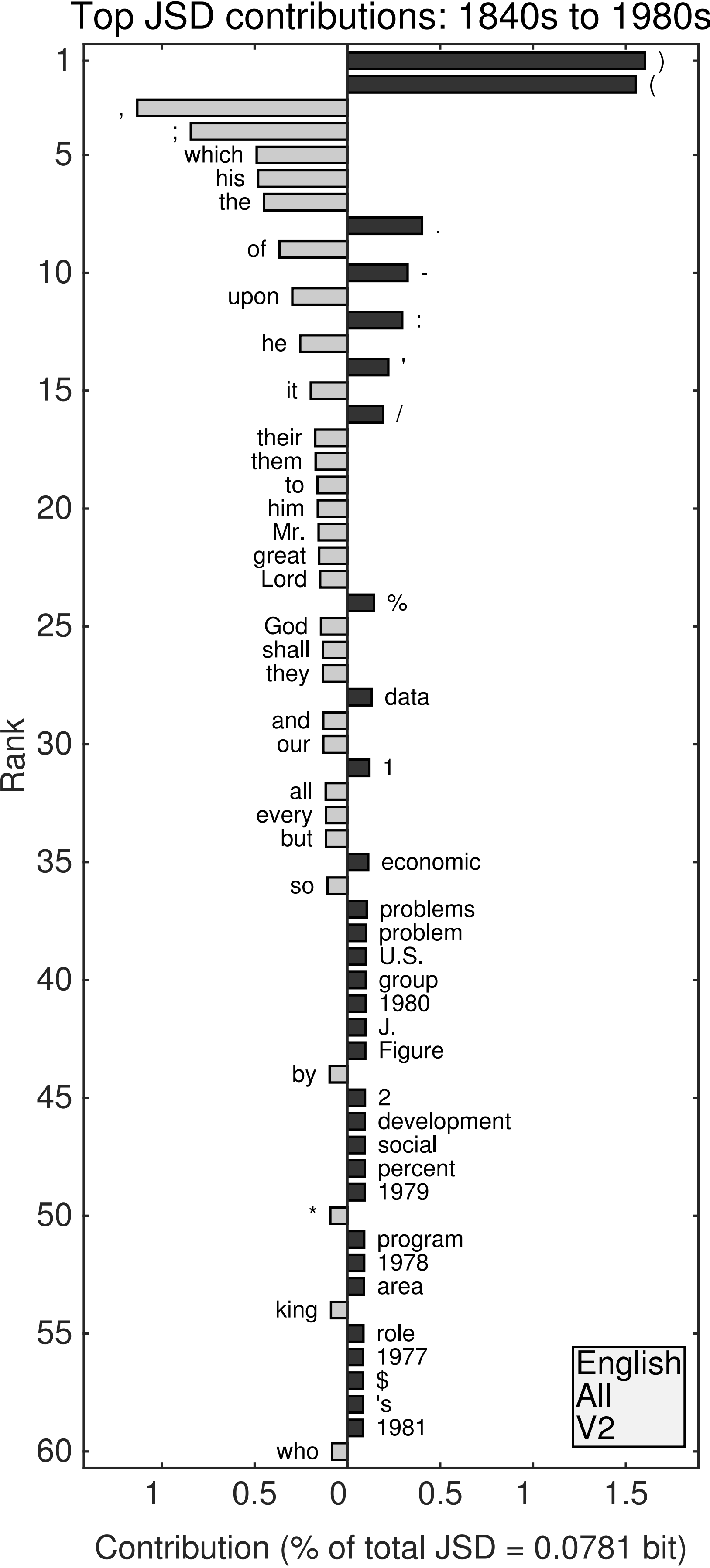

Top JSD contributions: 1840s to 1980s
Rank
Contribution (% of total JSD = 0.0781 bit)
1
5
10
15
20
25
30
35
40
45
50
55
60
1
0.5
0
0.5
1
1.5
)
(
,
;
which
his
the
.
of
-
upon
:
he
'
it
/
their
them
to
him
Mr.
great
Lord
%
God
shall
they
data
and
our
1
all
every
but
economic
so
problems
problem
U.S.
group
1980
J.
Figure
by
2
development
social
percent
1979
*
program
1978
area
king
role
1977
$
's
1981
who
English
All
V2

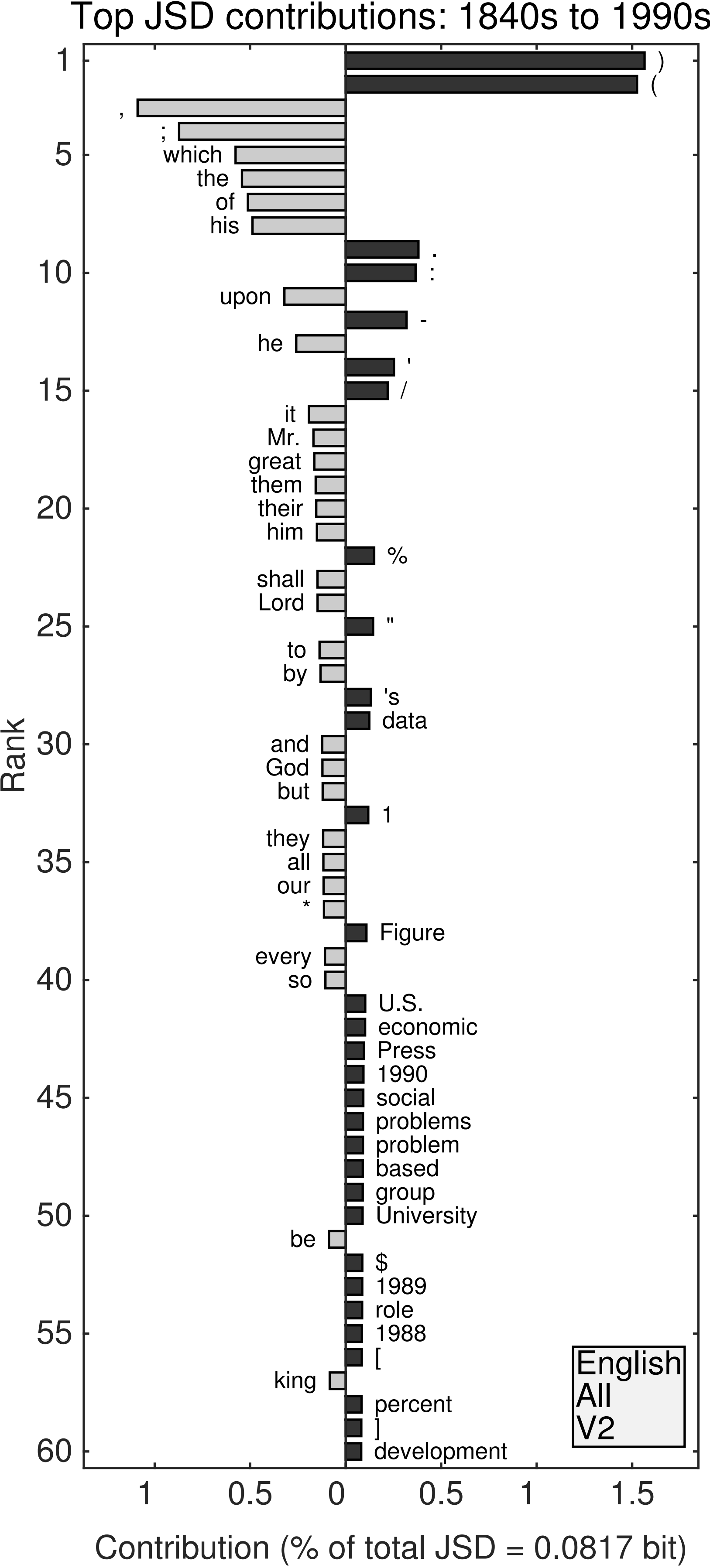

Top JSD contributions: 1840s to 1990s
Rank
Contribution (% of total JSD = 0.0817 bit)
)
(
,
;
which
the
of
his
.
:
upon
-
he
'
/
it
Mr.
great
them
their
him
%
shall
Lord
"
to
by
's
data
and
God
but
1
they
all
our
*
Figure
every
so
U.S.
economic
Press
1990
social
problems
problem
based
group
University
be
$
1989
role
1988
[
king
percent
]
development
English
All
V2

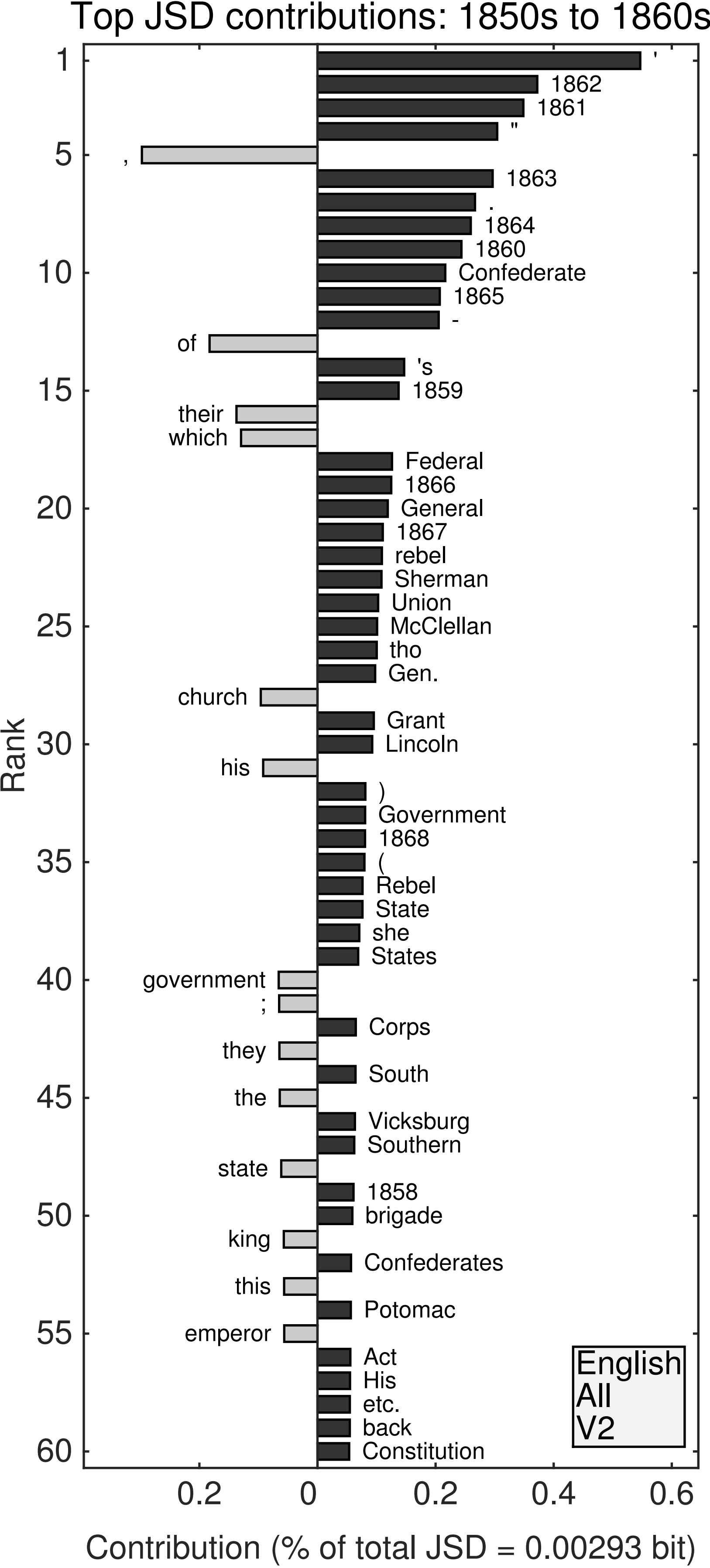

Top JSD contributions: 1850s to 1860s
Rank
Contribution (% of total JSD = 0.00293 bit)
0.2
0
0.2
0.4
0.6
1
5
10
15
20
25
30
35
40
45
50
55
60
'
1862
1861
"
,
1863
.
1864
1860
Confederate
1865
-
of
's
1859
their
which
Federal
1866
General
1867
rebel
Sherman
Union
McClellan
tho
Gen.
church
Grant
Lincoln
his
)
Government
1868
(
Rebel
State
she
States
government
;
Corps
they
South
the
Vicksburg
Southern
state
1858
brigade
king
Confederates
this
Potomac
emperor
Act
His
etc.
back
Constitution
English
All
V2

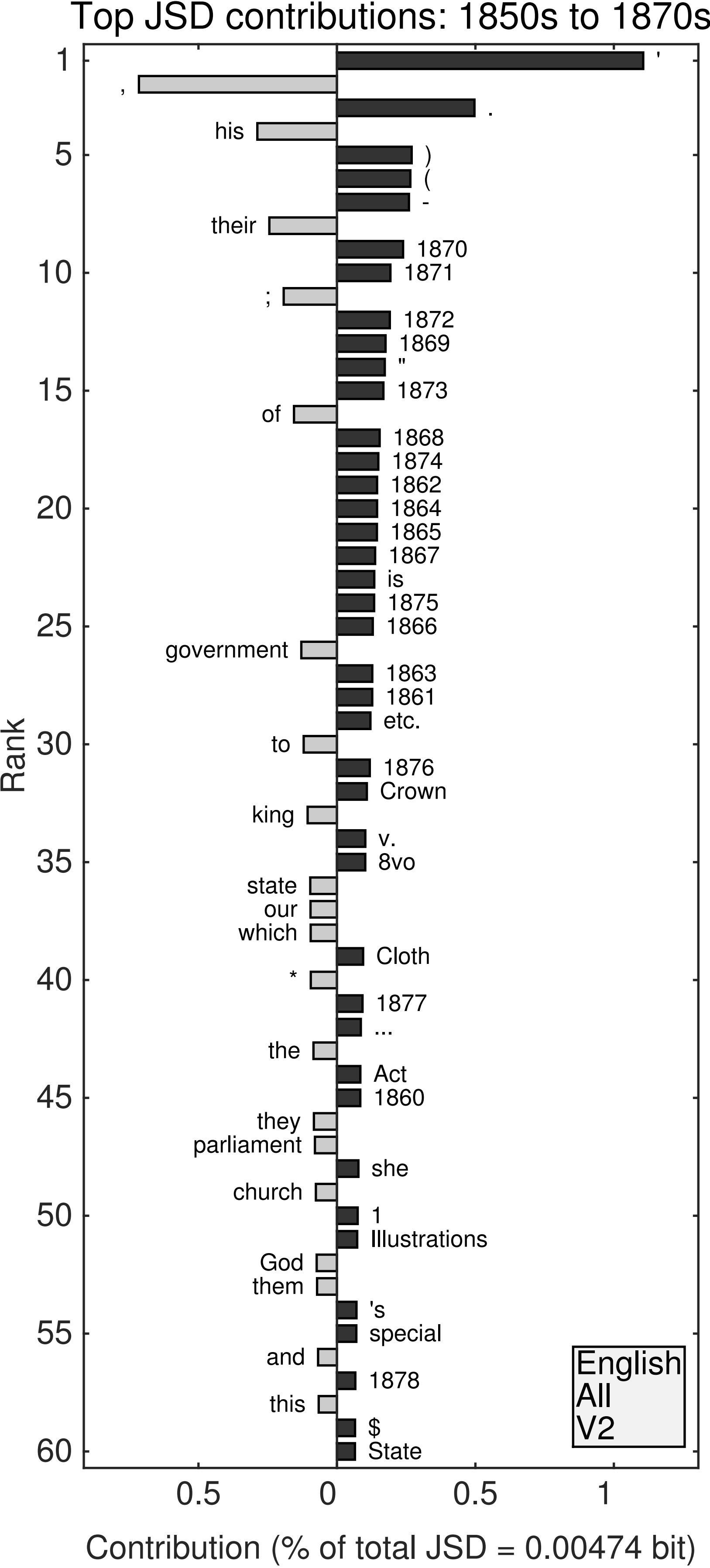

Top JSD contributions: 1850s to 1870s
Rank
Contribution (% of total JSD = 0.00474 bit)
English
All
V2
'
,
.
his
)
(
-
their
1870
1871
;
1872
1869
"
1873
of
1868
1874
1862
1864
1865
1867
is
1875
1866
government
1863
1861
etc.
to
1876
Crown
king
v.
8vo
state
our
which
Cloth
*
1877
...
the
Act
1860
they
parliament
she
church
1
Illustrations
God
them
's
special
and
1878
this
$
State

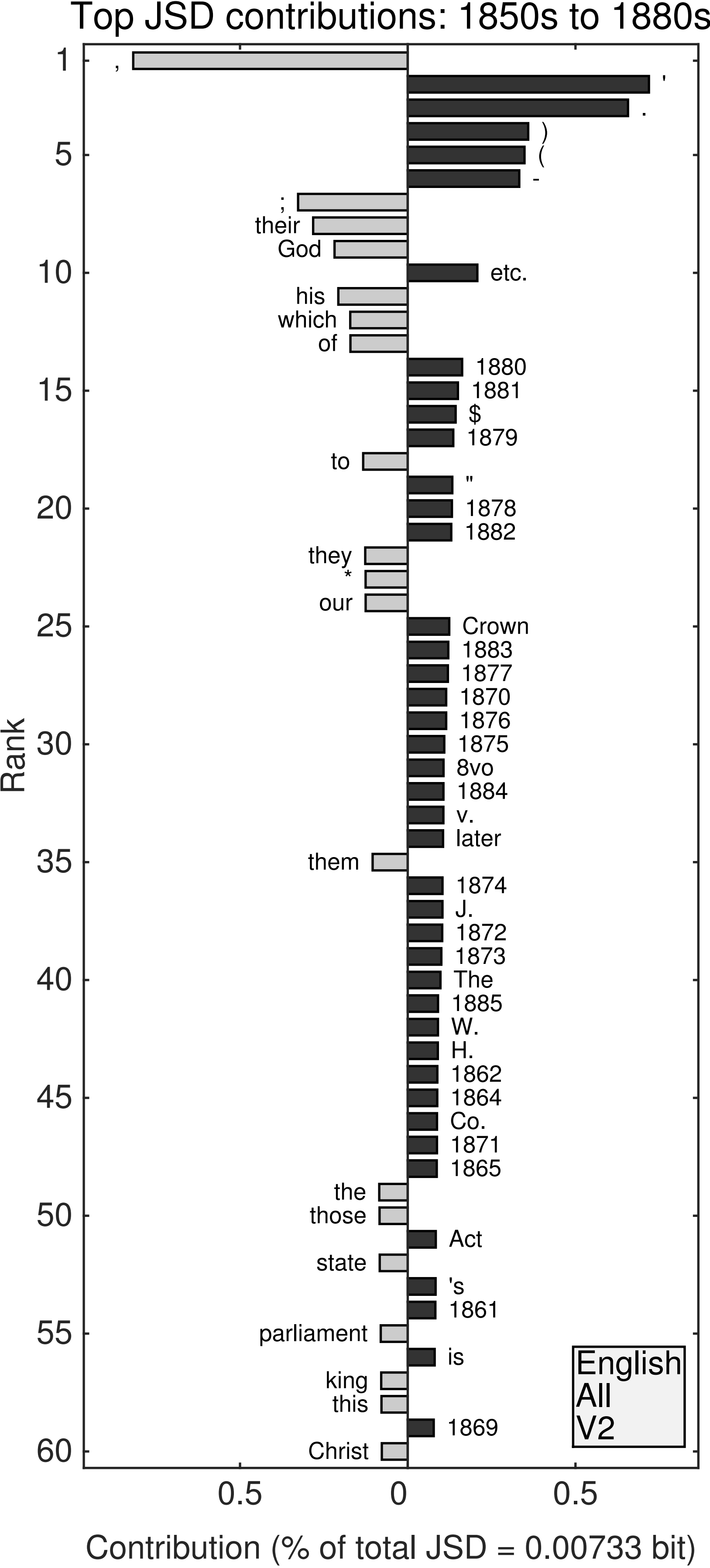

Top JSD contributions: 1850s to 1880s
Rank
Contribution (% of total JSD = 0.00733 bit)
0.5
0
0.5
1
5
10
15
20
25
30
35
40
45
50
55
60
,
'
.
)
(
-
;
their
God
etc.
his
which
of
1880
1881
$
1879
to
"
1878
1882
they
*
our
Crown
1883
1877
1870
1876
1875
8vo
1884
v.
later
them
1874
J.
1872
1873
The
1885
W.
H.
1862
1864
Co.
1871
1865
the
those
Act
state
's
1861
parliament
is
king
this
1869
Christ
English
All
V2

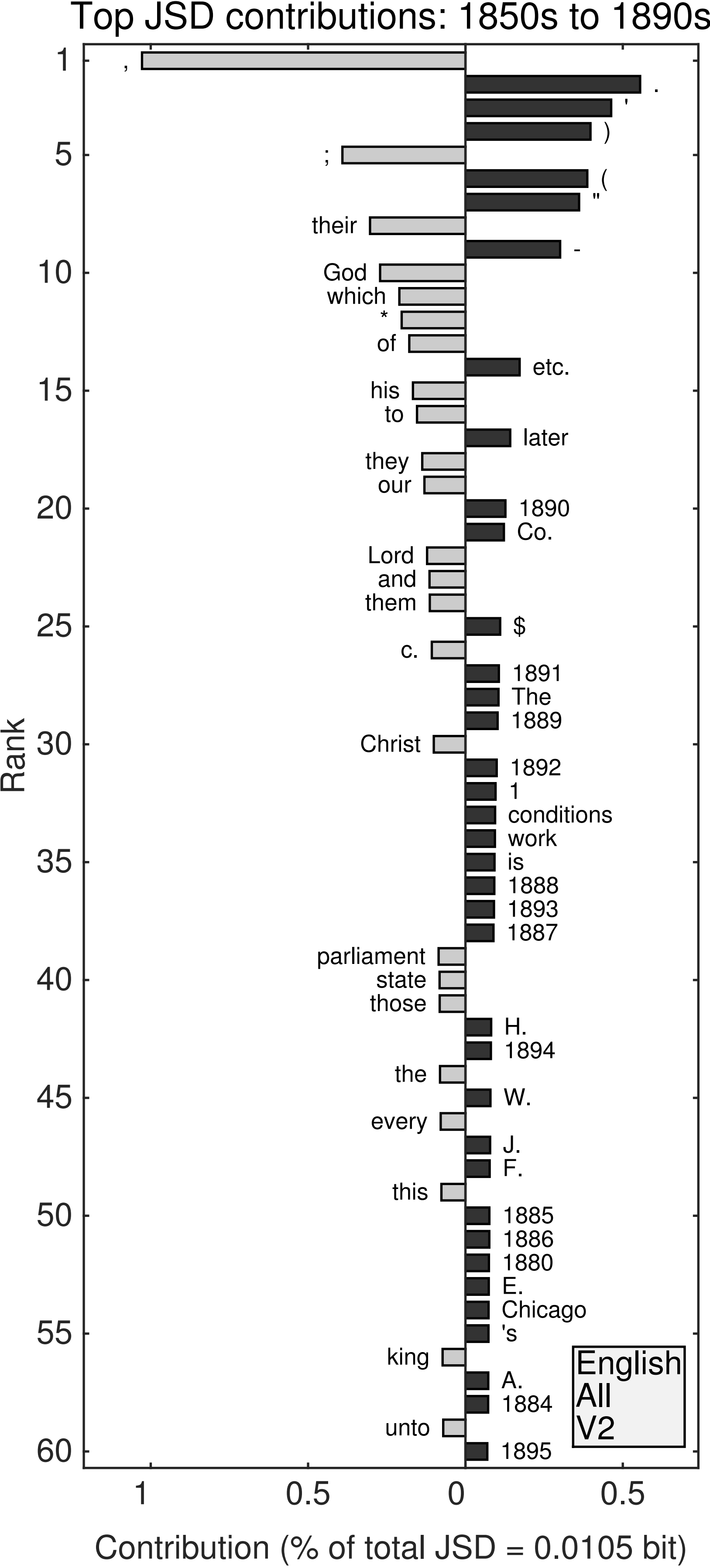
Top JSD contributions: 1850s to 1890s
Rank
Contribution (% of total JSD = 0.0105 bit)
1
5
10
15
20
25
30
35
40
45
50
55
60
1
0.5
0
0.5
,
.
'
)
;
(
"
their
-
God
which
*
of
etc.
his
to
later
they
our
1890
Co.
Lord
and
them
$
c.
1891
The
1889
Christ
1892
1
conditions
work
is
1888
1893
1887
parliament
state
those
H.
1894
the
W.
every
J.
F.
this
1885
1886
1880
E.
Chicago
's
king
A.
1884
unto
1895
English
All
V2

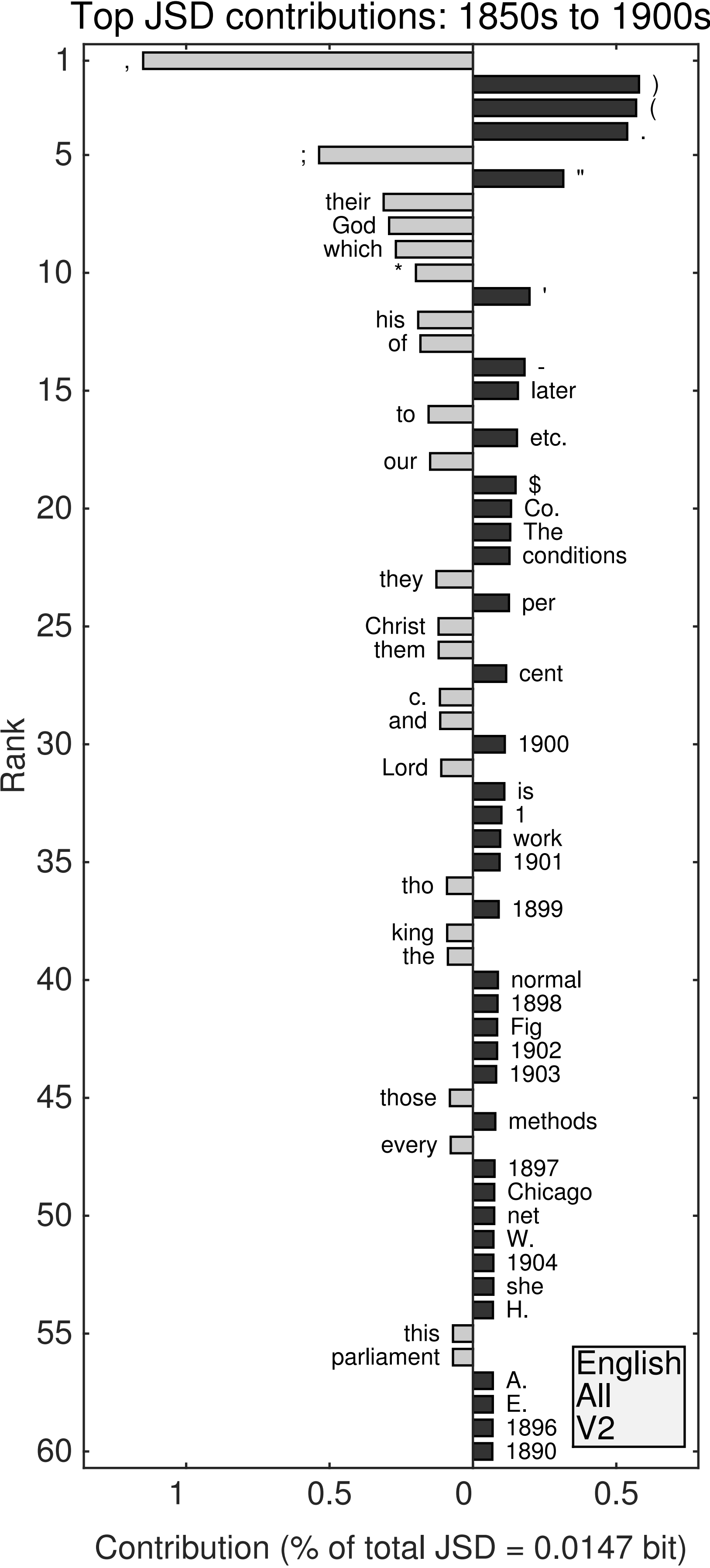

Top JSD contributions: 1850s to 1900s
Rank
Contribution (% of total JSD = 0.0147 bit)
1
5
10
15
20
25
30
35
40
45
50
55
60
1
0.5
0
0.5
,
)
(
.
;
"
their
God
which
*
'
his
of
-
later
to
etc.
our
$
Co.
The
conditions
they
per
Christ
them
cent
c.
and
1900
Lord
is
1
work
1901
tho
1899
king
the
normal
1898
Fig
1902
1903
those
methods
every
1897
Chicago
net
W.
1904
she
H.
this
parliament
A.
E.
1896
1890
English
All
V2

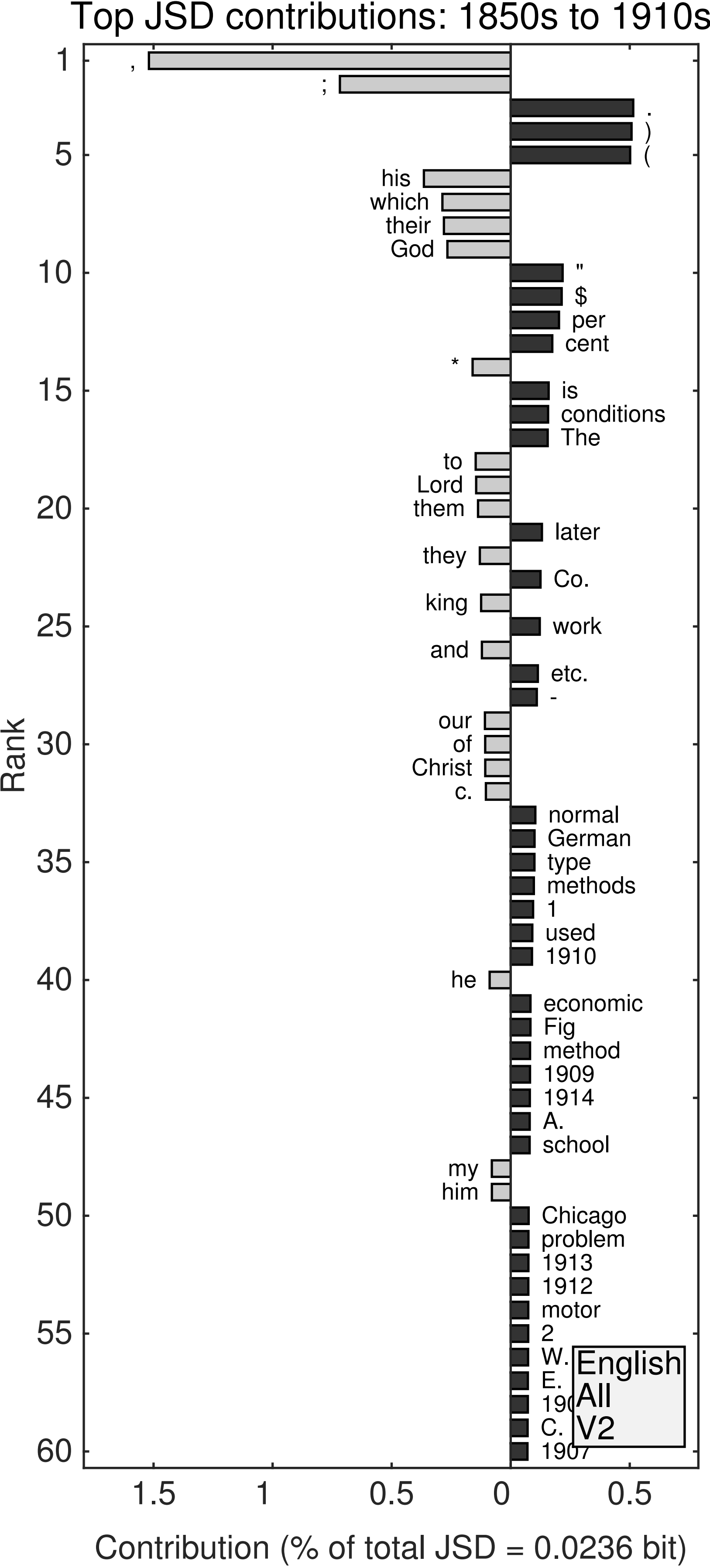
Top JSD contributions: 1850s to 1910s
Rank
Contribution (% of total JSD = 0.0236 bit)
1.5
1
0.5
0
0.5
1
5
10
15
20
25
30
35
40
45
50
55
60
,
;
.
)
(
his
which
their
God
"
$
per
cent
*
is
conditions
The
to
Lord
them
later
they
Co.
king
work
and
etc.
-
our
of
Christ
c.
normal
German
type
methods
1
used
1910
he
economic
Fig
method
1909
1914
A.
school
my
him
Chicago
problem
1913
1912
motor
2
W.
E.
C.
1907
English
All
V2

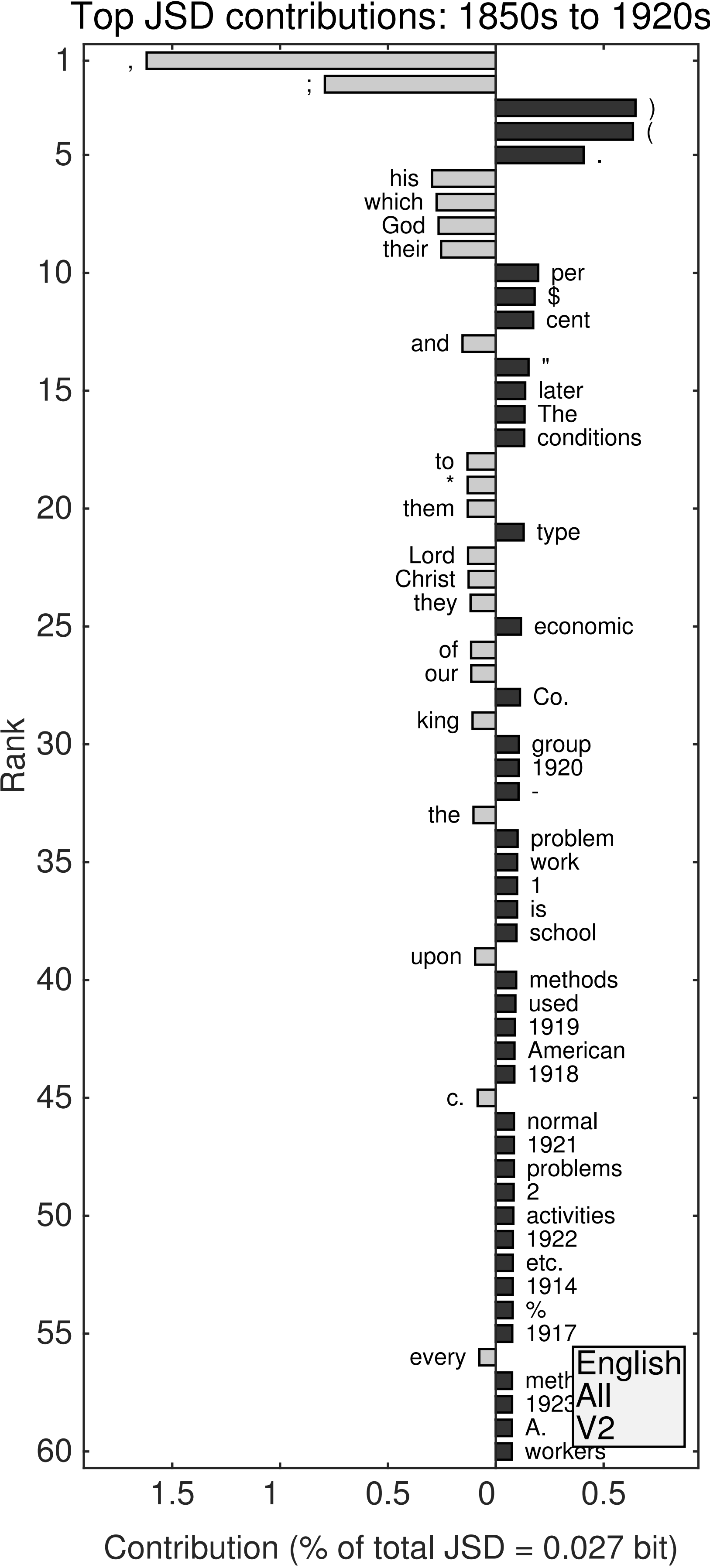

Top JSD contributions: 1850s to 1920s
Rank
Contribution (% of total JSD = 0.027 bit)
1.5
1
0.5
0
0.5
1
5
10
15
20
25
30
35
40
45
50
55
60
,
;
)
(
.
his
which
God
their
per
$
cent
and
"
later
The
conditions
to
*
them
type
Lord
Christ
they
economic
of
our
Co.
king
group
1920
-
the
problem
work
1
is
school
upon
methods
used
1919
American
1918
c.
normal
1921
problems
2
activities
1922
etc.
1914
%
1917
every
A.
workers
English
All
V2

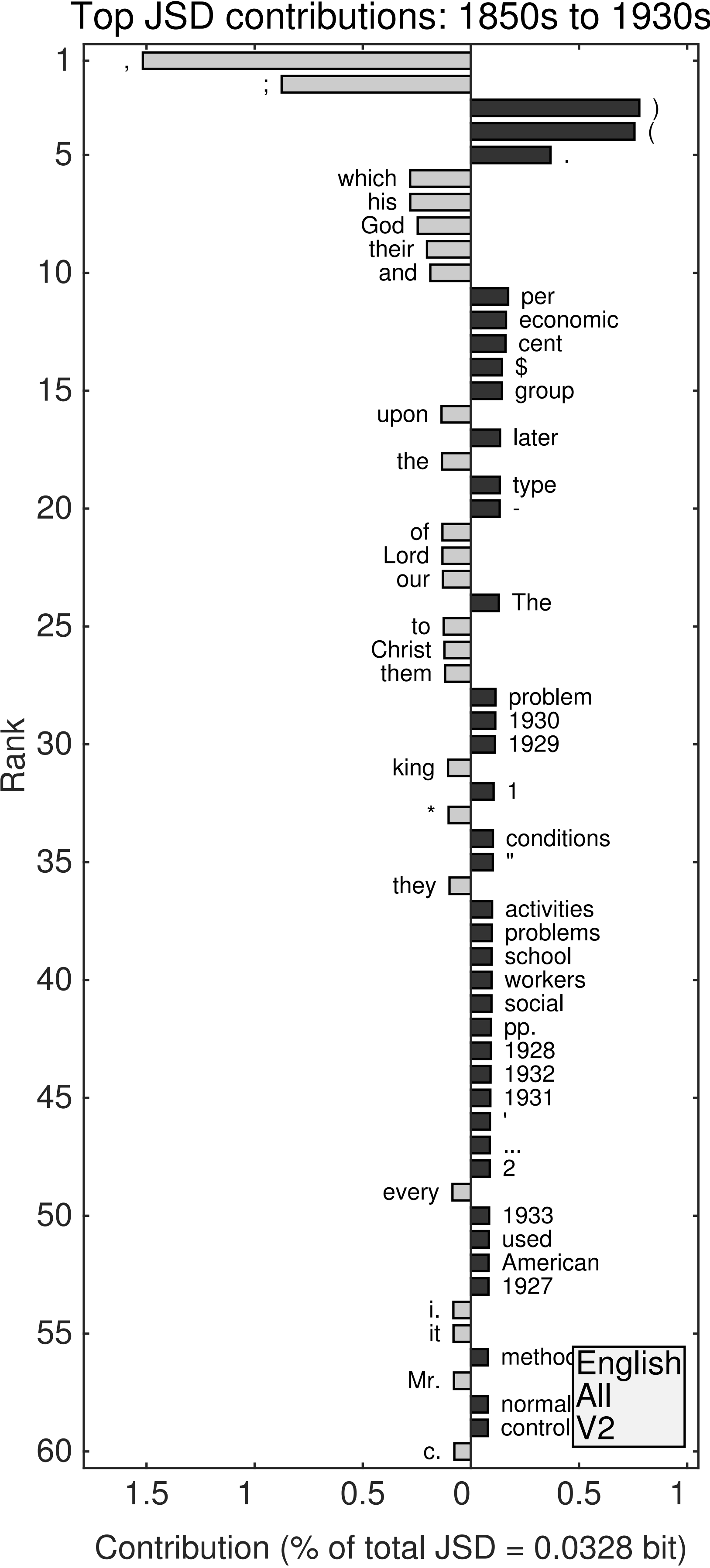

Top JSD contributions: 1850s to 1930s
Rank
1
5
10
15
20
25
30
35
40
45
50
55
60
,
;
)
(
.
which
his
God
their
and
per
economic
cent
$
group
upon
later
the
type
-
of
Lord
our
The
to
Christ
them
problem
1930
1929
king
1
*
conditions
"
they
activities
problems
school
workers
social
pp.
1928
1932
1931
'
...
2
every
1933
used
American
1927
i.
it
method
Mr.
normal
control
c.
English
All
V2
1.5
1
0.5
0
0.5
1
Contribution (% of total JSD = 0.0328 bit)

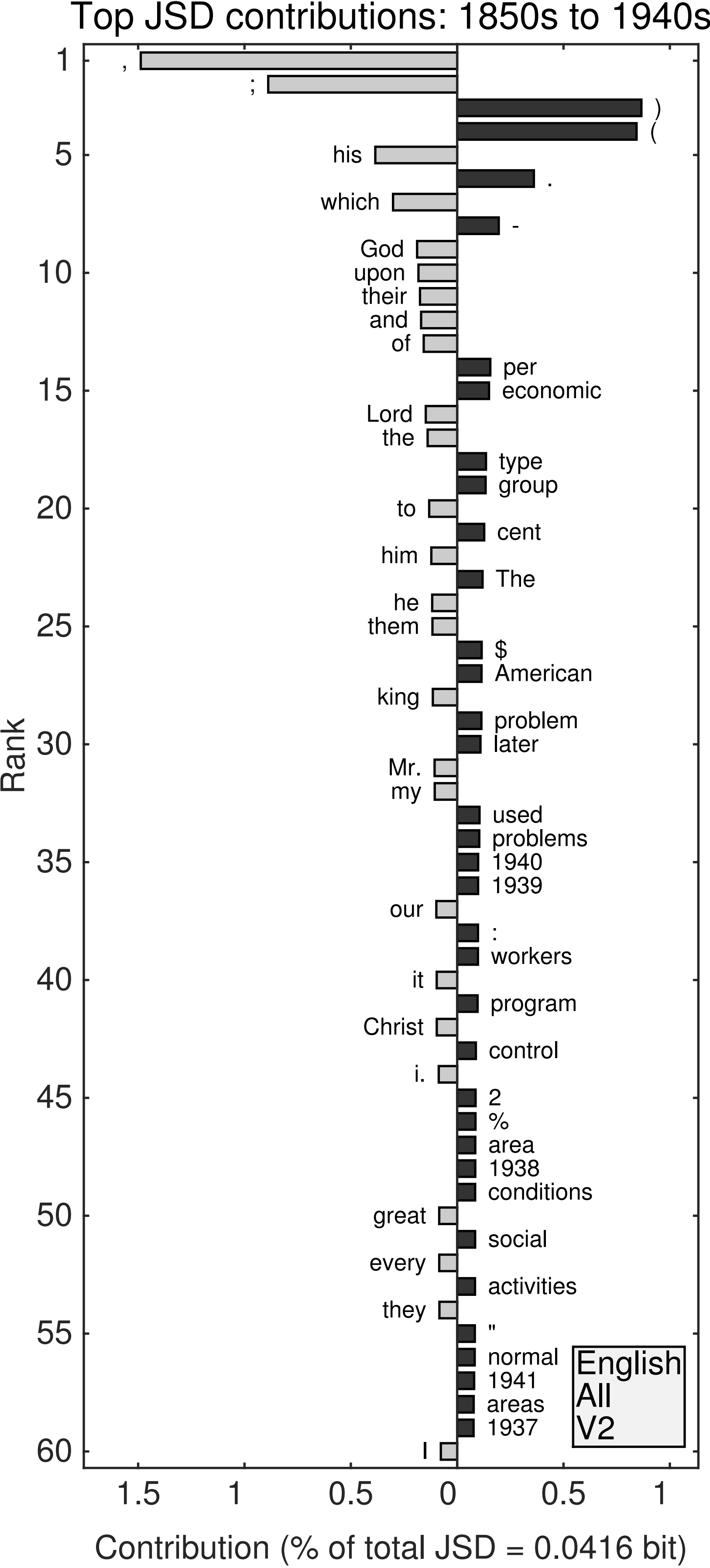

Top JSD contributions: 1850s to 1940s
Rank
Contribution (% of total JSD = 0.0416 bit)
,
;
)
(
his
.
which
-
God
upon
their
and
of
per
economic
Lord
the
type
group
to
cent
him
The
he
them
$
American
king
problem
later
Mr.
my
used
problems
1940
1939
our
:
workers
it
program
Christ
control
i.
2
%
area
1938
conditions
great
social
every
activities
they
"
normal
1941
areas
1937
I
English
All
V2

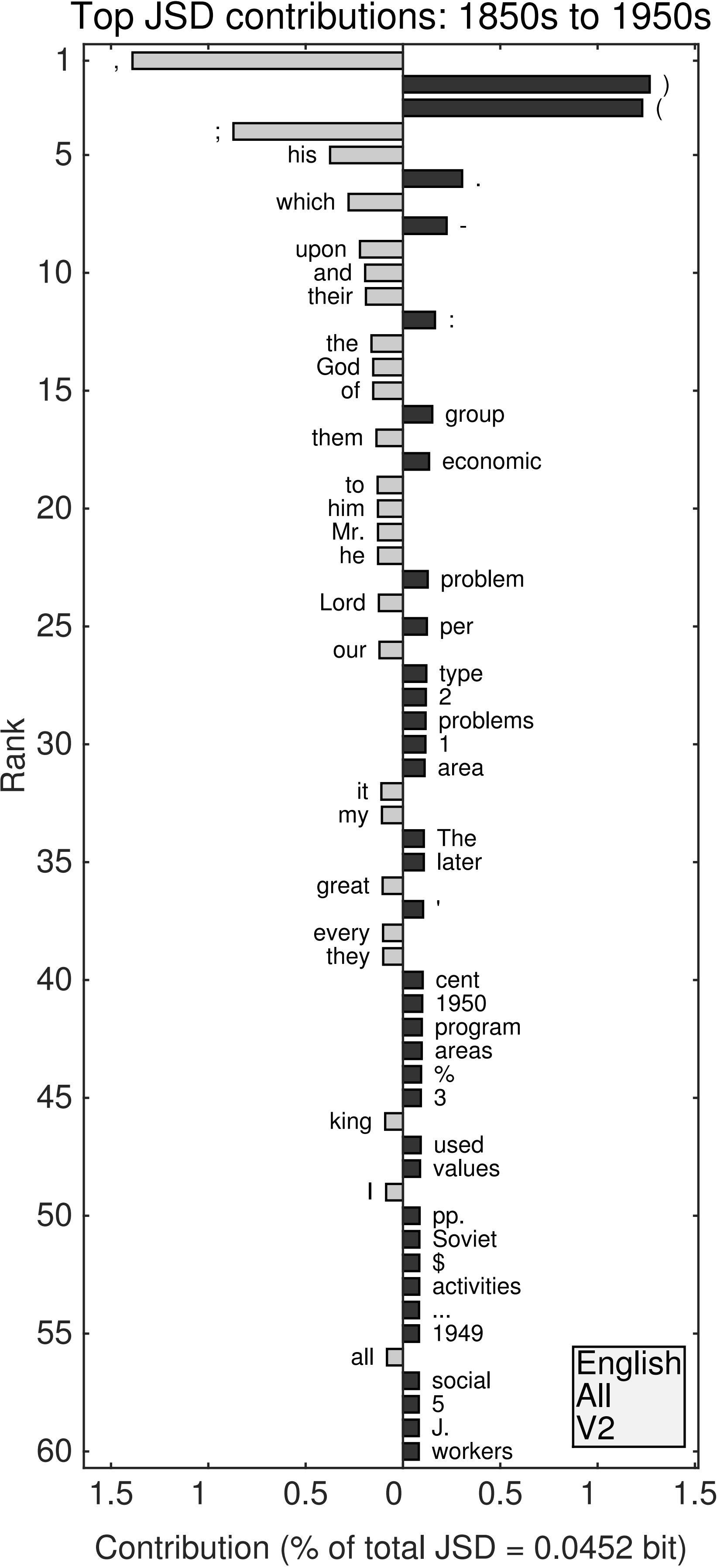
Top JSD contributions: 1850s to 1950s
Rank
Contribution (% of total JSD = 0.0452 bit)
English
All
V2
,
)
(
;
his
.
which
-
upon
and
their
:
the
God
of
group
them
economic
to
him
Mr.
he
problem
Lord
per
our
type
2
problems
1
area
it
my
The
later
great
'
every
they
cent
1950
program
areas
%
3
king
used
values
I
pp.
Soviet
$
activities
...
1949
all
social
5
J.
workers

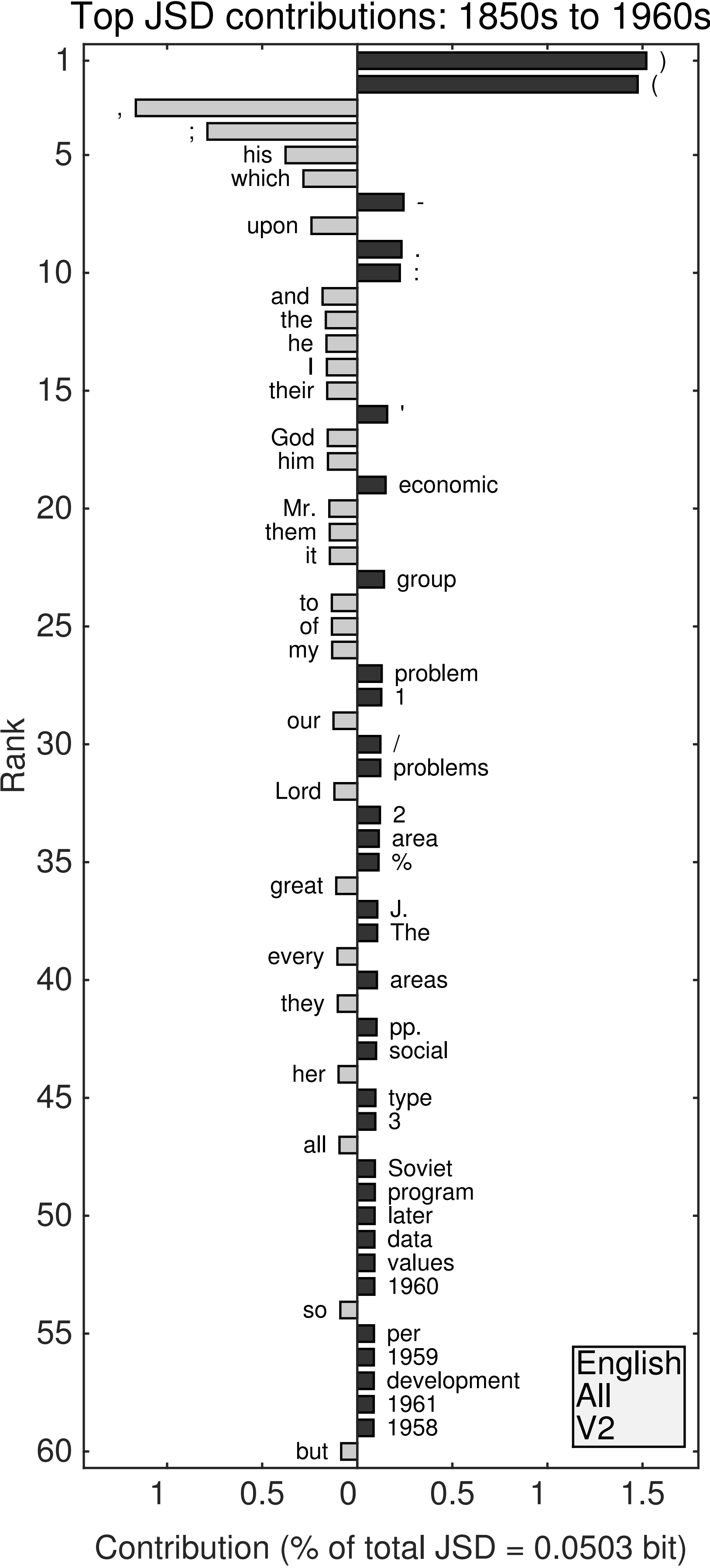
Top JSD contributions: 1850s to 1960s
Rank
Contribution (% of total JSD = 0.0503 bit)
)
(
,
;
his
which
-
upon
.
:
and
the
he
I
their
'
God
him
economic
Mr.
them
it
group
to
of
my
problem
1
our
/
problems
Lord
2
area
%
great
J.
The
every
areas
they
pp.
social
her
type
3
all
Soviet
program
later
data
values
1960
so
per
1959
development
1961
1958
but
English
All
V2

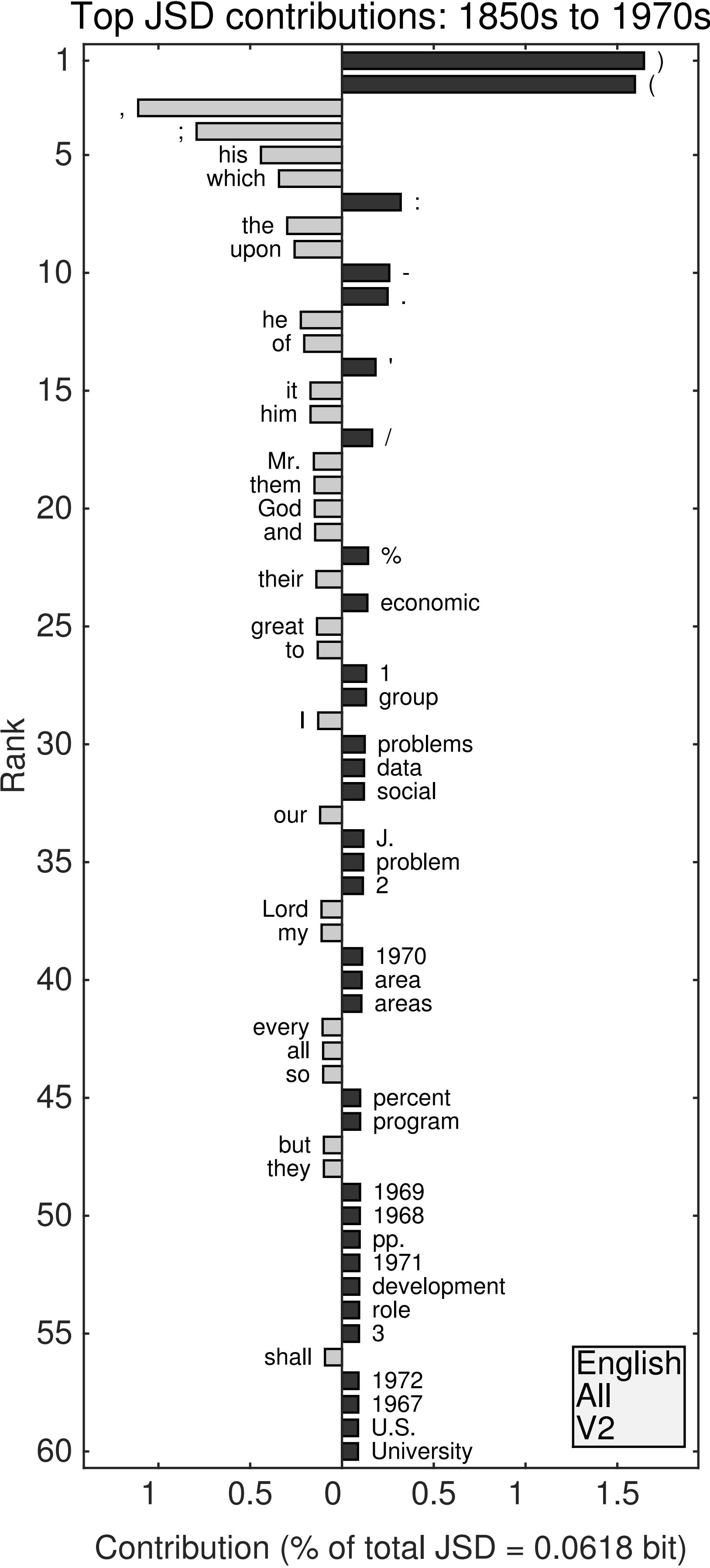
Top JSD contributions: 1850s to 1970s
Rank
Contribution (% of total JSD = 0.0618 bit)
)
(
,
;
his
which
:
the
upon
-
.
he
of
'
it
him
/
Mr.
them
God
and
%
their
economic
great
to
1
group
I
problems
data
social
our
J.
problem
2
Lord
my
1970
area
areas
every
all
so
percent
program
but
they
1969
1968
pp.
1971
development
role
3
shall
1972
1967
U.S.
University
English
All
V2

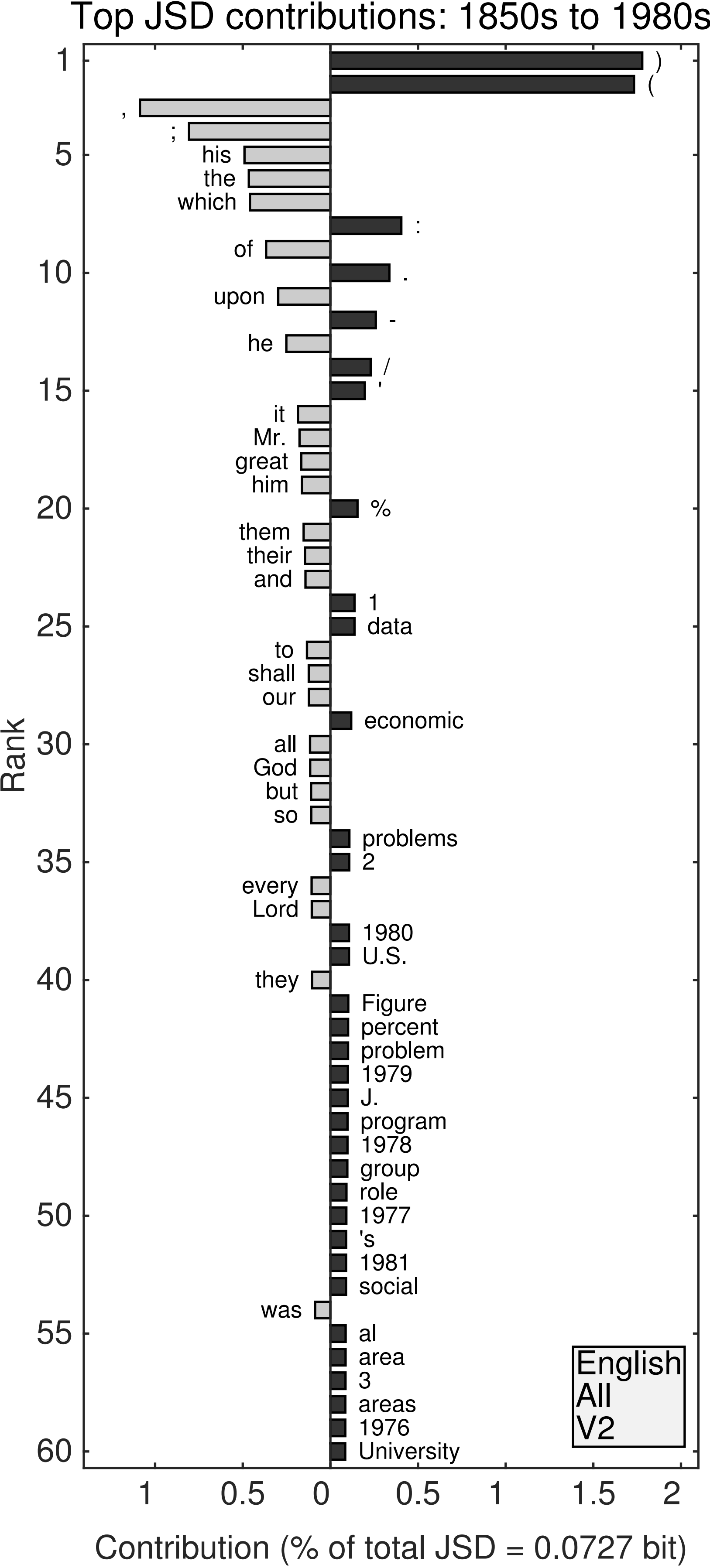

Top JSD contributions: 1850s to 1980s
Rank
Contribution (% of total JSD = 0.0727 bit)
)
(
,
;
his
the
which
:
of
.
upon
-
he
/
'
it
Mr.
great
him
%
them
their
and
1
data
to
shall
our
economic
all
God
but
so
problems
2
every
Lord
1980
U.S.
they
Figure
percent
problem
1979
J.
program
1978
group
role
1977
's
1981
social
was
al
area
3
areas
1976
University
English
All
V2

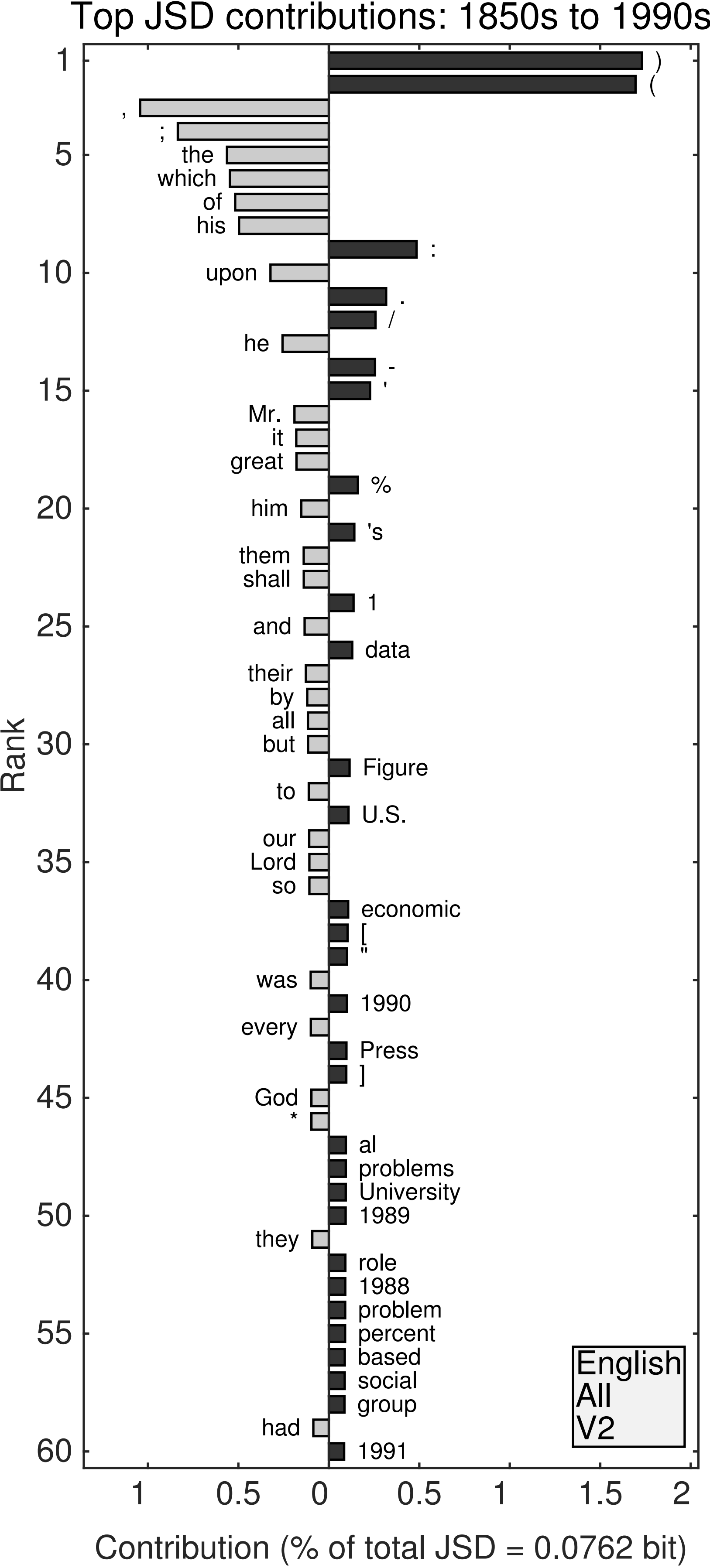

Top JSD contributions: 1850s to 1990s
Rank
Contribution (% of total JSD = 0.0762 bit)
)
(
,
;
the
which
of
his
:
upon
.
/
he
-
'
Mr.
it
great
%
him
's
them
shall
1
and
data
their
by
all
but
Figure
to
U.S.
our
Lord
so
economic
[
"
was
1990
every
Press
]
God
*
al
problems
University
1989
they
role
1988
problem
percent
based
social
group
had
1991
English
All
V2

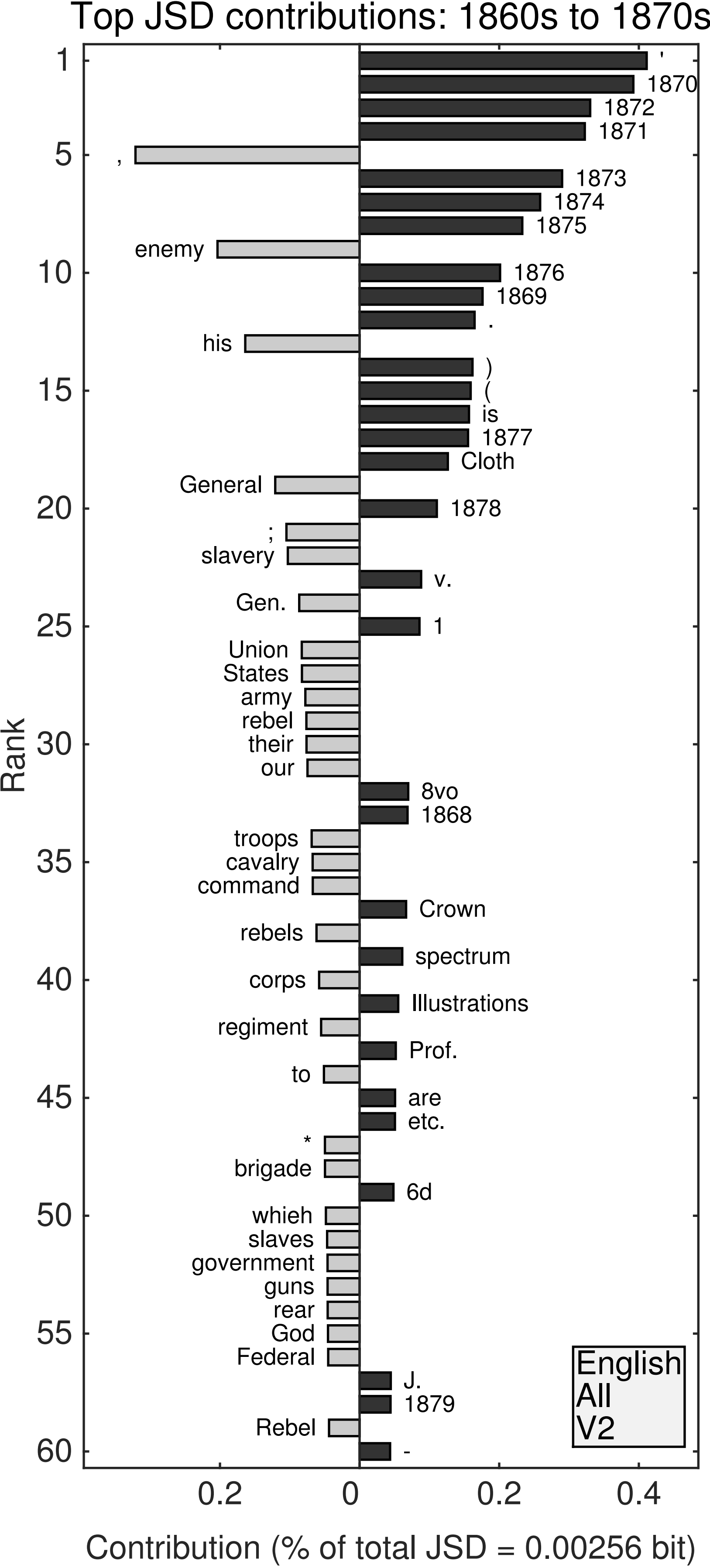

Top JSD contributions: 1860s to 1870s
'
1870
1872
1871
,
1873
1874
1875
enemy
1876
1869
.
his
)
(
is
1877
Cloth
General
1878
;
slavery
v.
Gen.
1
Union
States
army
rebel
their
our
8vo
1868
troops
cavalry
command
Crown
rebels
spectrum
corps
Illustrations
regiment
Prof.
to
are
etc.
*
brigade
6d
whieh
slaves
government
guns
rear
God
Federal
J.
1879
Rebel
-
English
All
V2
Rank
1
5
10
15
20
25
30
35
40
45
50
55
60
0.2
0
0.2
0.4
Contribution (% of total JSD = 0.00256 bit)

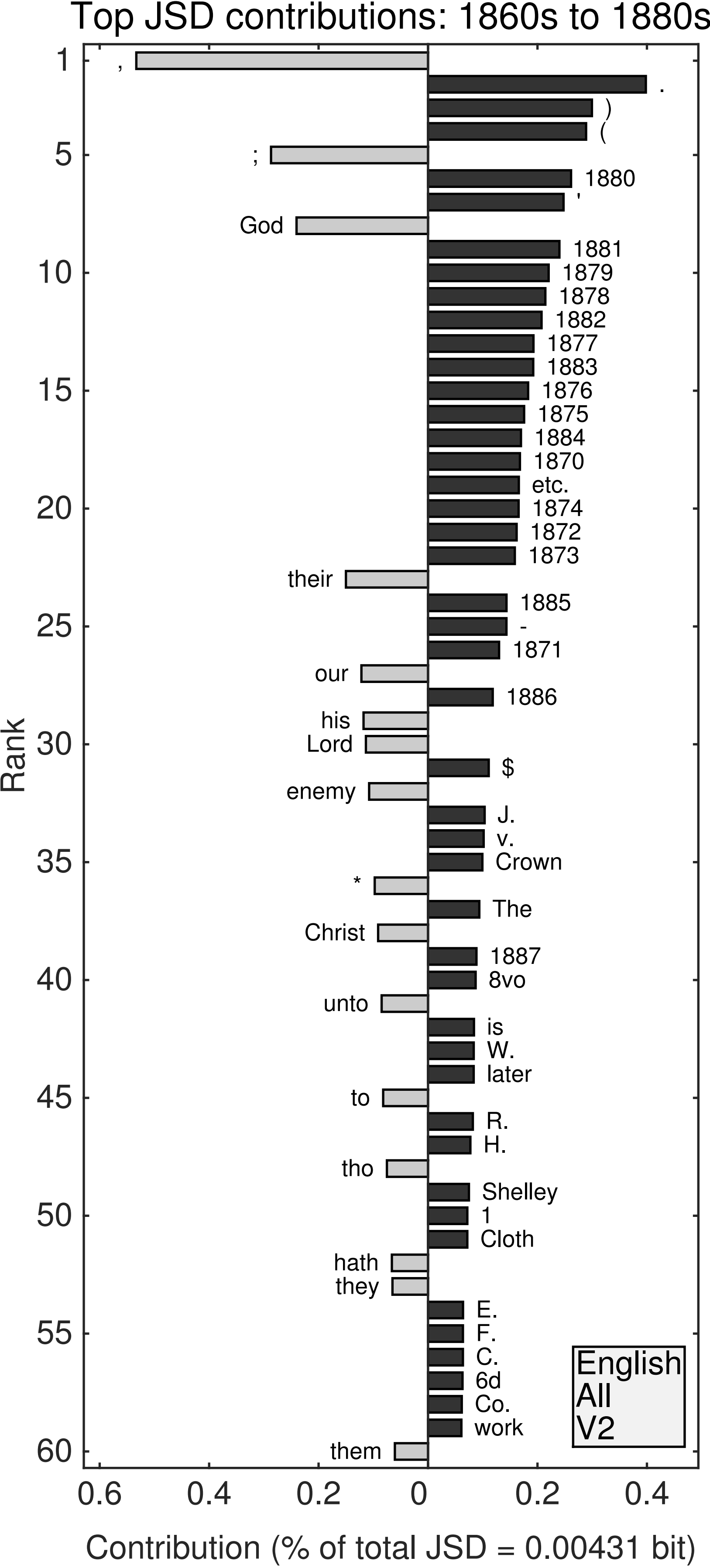

Top JSD contributions: 1860s to 1880s
Rank
Contribution (% of total JSD = 0.00431 bit)
,
.
)
(
;
1880
'
God
1881
1879
1878
1882
1877
1883
1876
1875
1884
1870
etc.
1874
1872
1873
their
1885
-
1871
our
1886
his
Lord
$
enemy
J.
v.
Crown
*
The
Christ
1887
8vo
unto
is
W.
later
to
R.
H.
tho
Shelley
1
Cloth
hath
they
E.
F.
C.
6d
Co.
work
them
English
All
V2

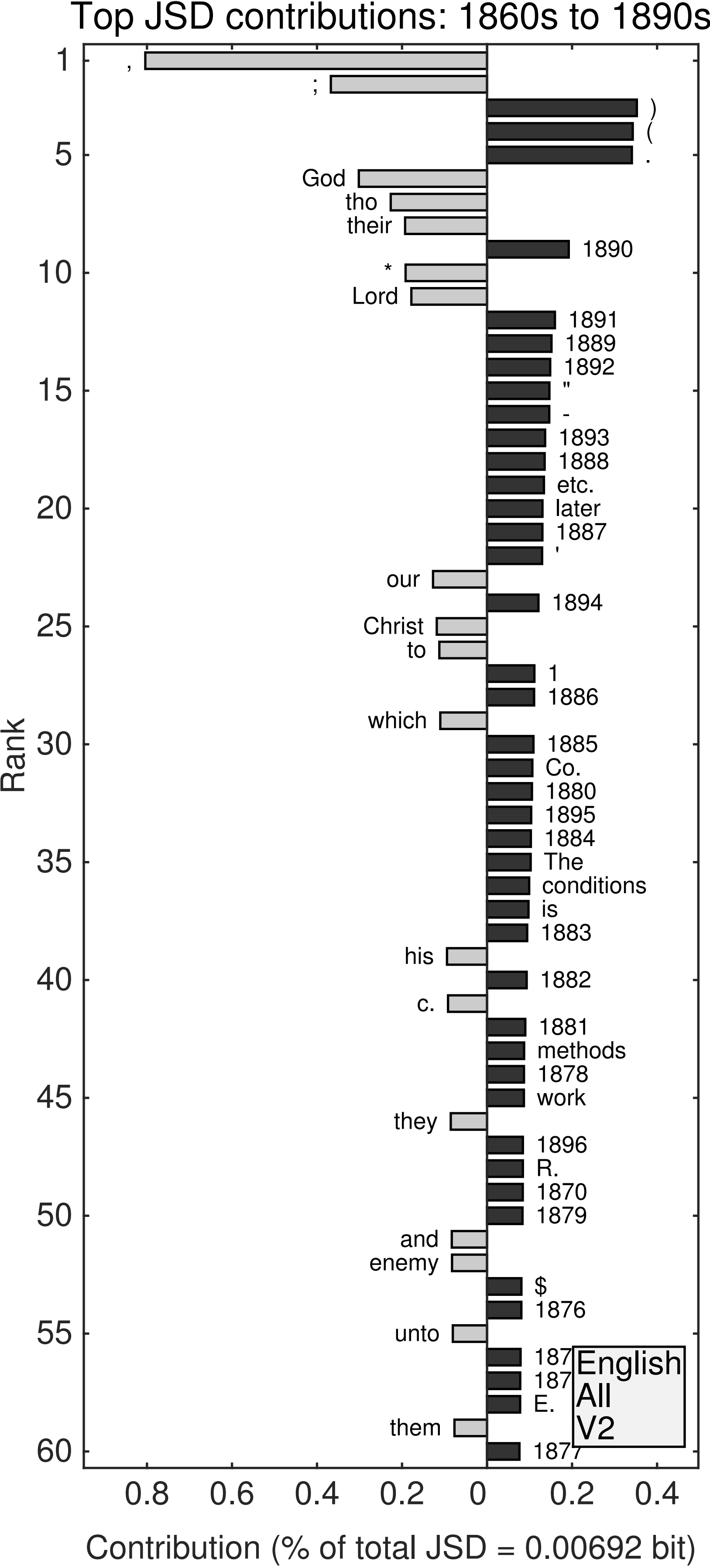
Top JSD contributions: 1860s to 1890s
Rank
Contribution (% of total JSD = 0.00692 bit)
0.8
0.6
0.4
0.2
0
0.2
0.4
1
5
10
15
20
25
30
35
40
45
50
55
60
,
;
)
(
.
God
tho
their
1890
*
Lord
1891
1889
1892
"
-
1893
1888
etc.
later
1887
'
our
1894
Christ
to
1
1886
which
1885
Co.
1880
1895
1884
The
conditions
is
1883
his
1882
c.
1881
methods
1878
work
they
1896
R.
1870
1879
and
enemy
$
1876
unto
E.
them
English
All
V2

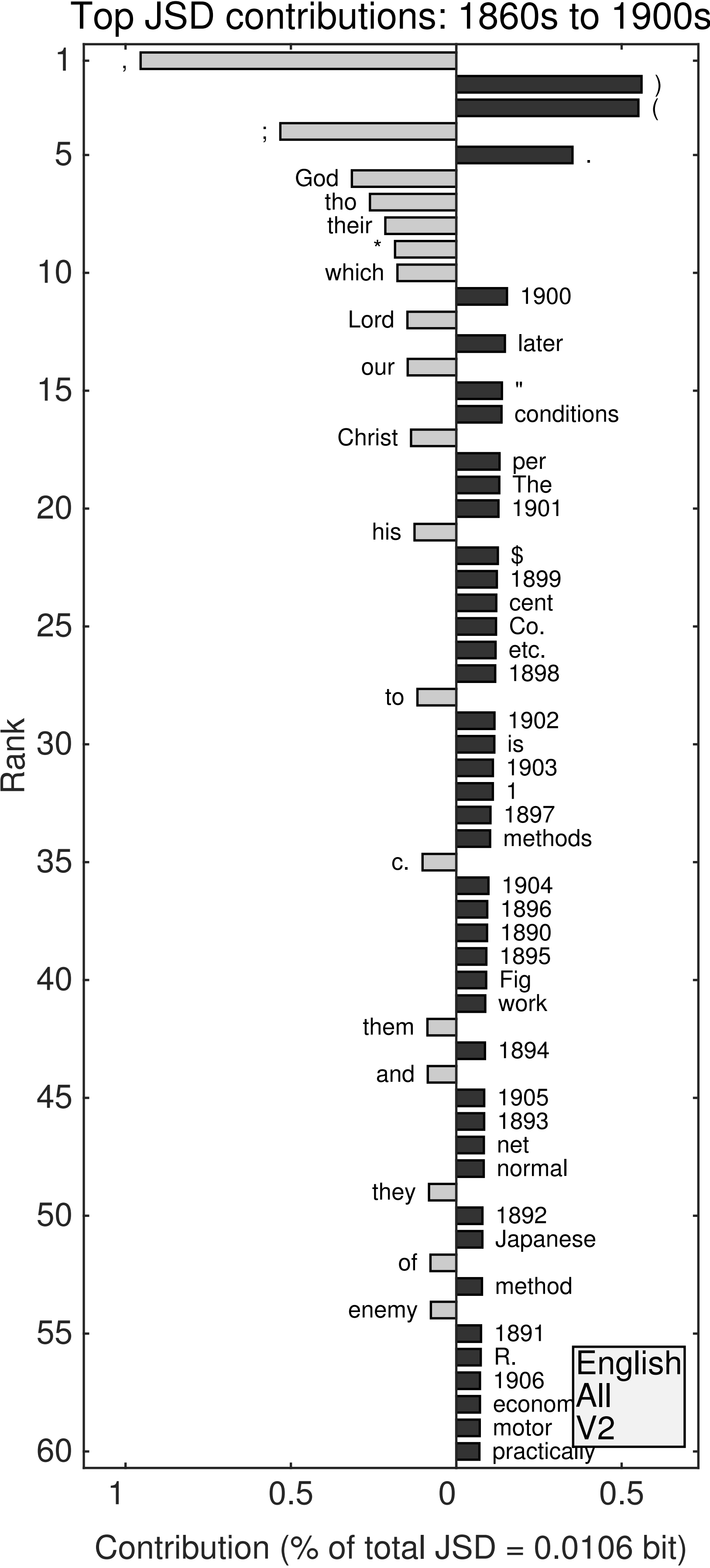

Top JSD contributions: 1860s to 1900s
Rank
Contribution (% of total JSD = 0.0106 bit)
1
5
10
15
20
25
30
35
40
45
50
55
60
1
0.5
0
0.5
,
)
(
;
.
God
tho
their
*
which
1900
Lord
later
our
"
conditions
Christ
per
The
1901
his
$
1899
cent
Co.
etc.
1898
to
1902
is
1903
1
1897
methods
c.
1904
1896
1890
1895
Fig
work
them
1894
and
1905
1893
net
normal
they
1892
Japanese
of
method
enemy
1891
R.
1906
econom
motor
practically
English
All
V2

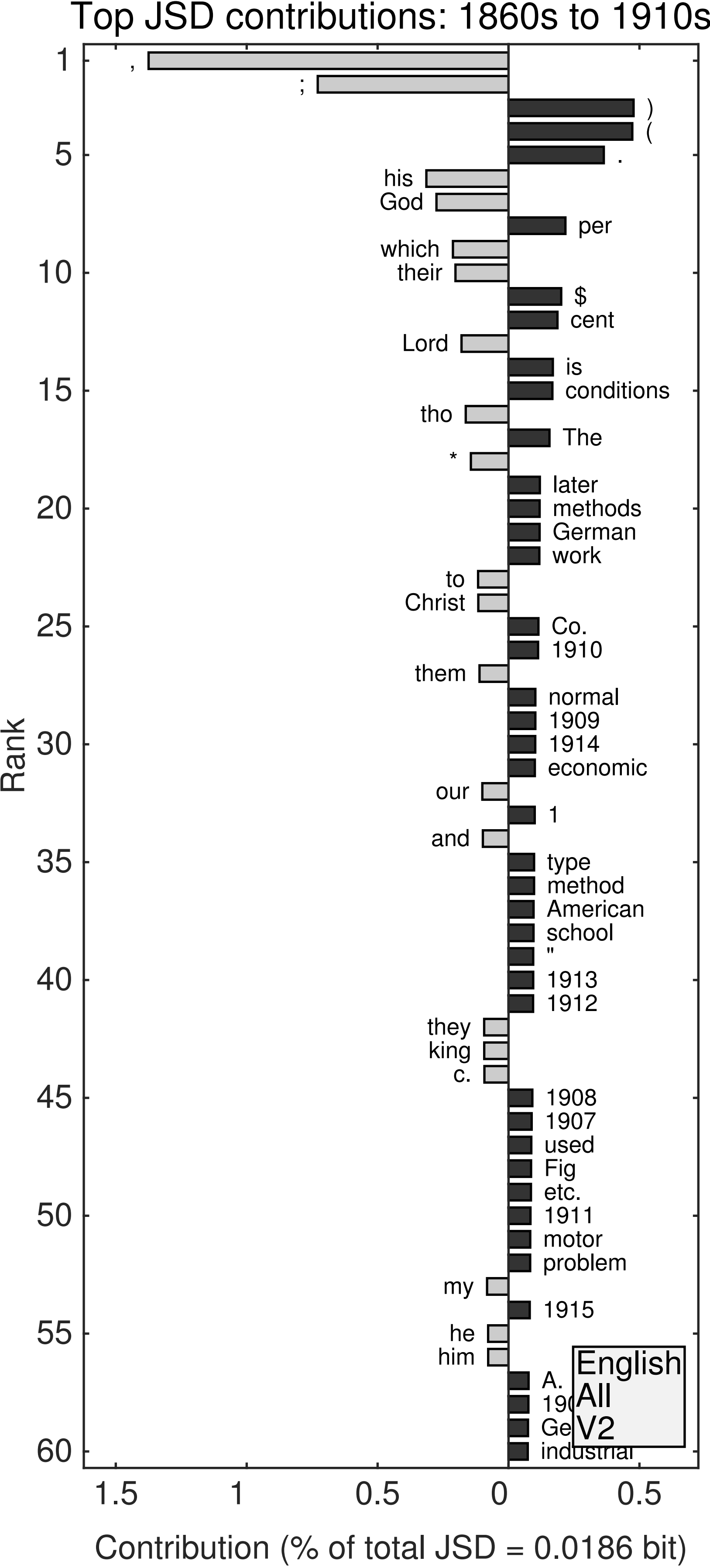

Top JSD contributions: 1860s to 1910s
Rank
Contribution (% of total JSD = 0.0186 bit)
English
All
V2
,
;
)
(
.
his
God
per
which
their
$
cent
Lord
is
conditions
tho
The
*
later
methods
German
work
to
Christ
Co.
1910
them
normal
1909
1914
economic
our
1
and
type
method
American
school
"
1913
1912
they
king
c.
1908
1907
used
Fig
etc.
1911
motor
problem
my
1915
he
him
A.
industrial

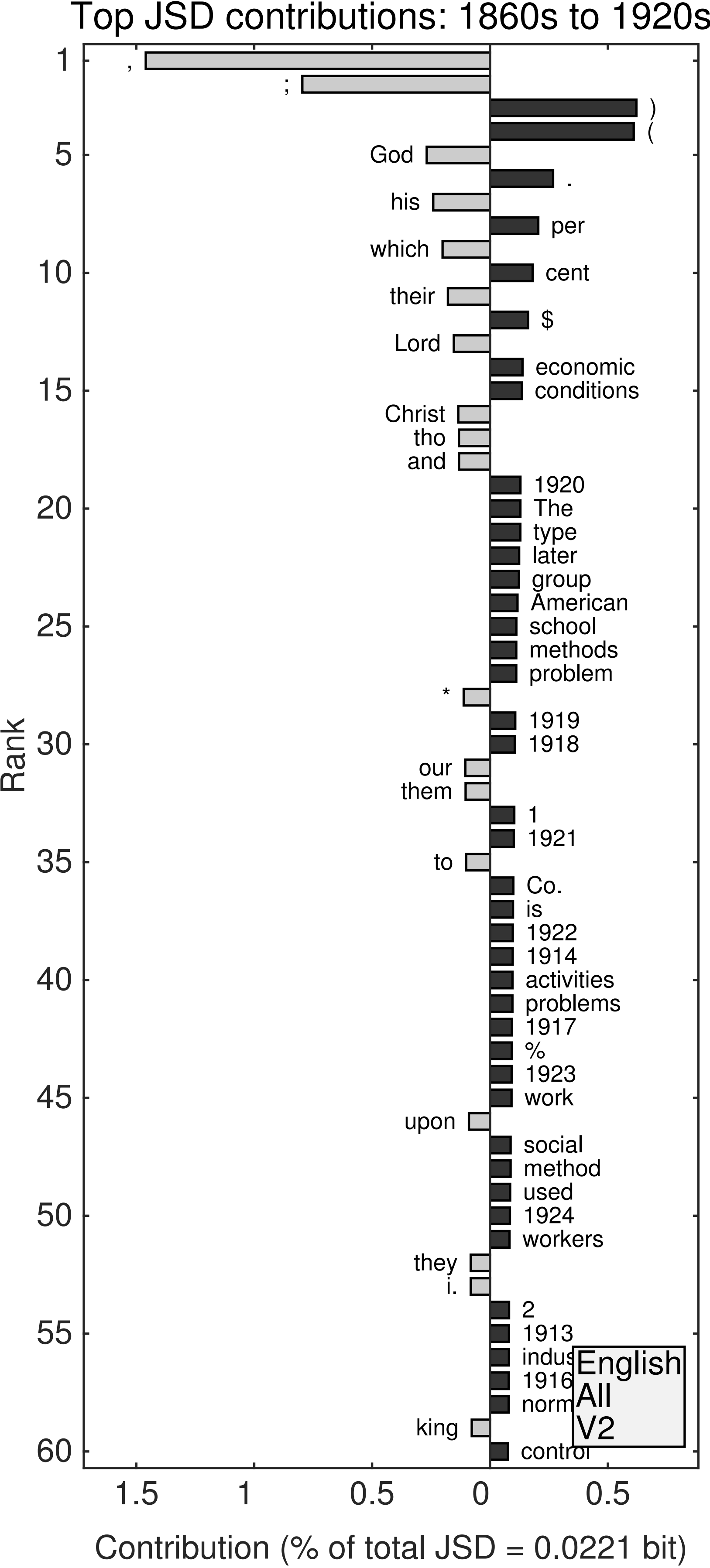

Top JSD contributions: 1860s to 1920s
Rank
Contribution (% of total JSD = 0.0221 bit)
1.5
1
0.5
0
0.5
1
5
10
15
20
25
30
35
40
45
50
55
60
,
;
)
(
God
.
his
per
which
cent
their
$
Lord
economic
conditions
Christ
tho
and
1920
The
type
later
group
American
school
methods
problem
*
1919
1918
our
them
1
1921
to
Co.
is
1922
1914
activities
problems
1917
%
1923
work
upon
social
method
used
1924
workers
they
i.
2
1913
1916
king
English
All
V2

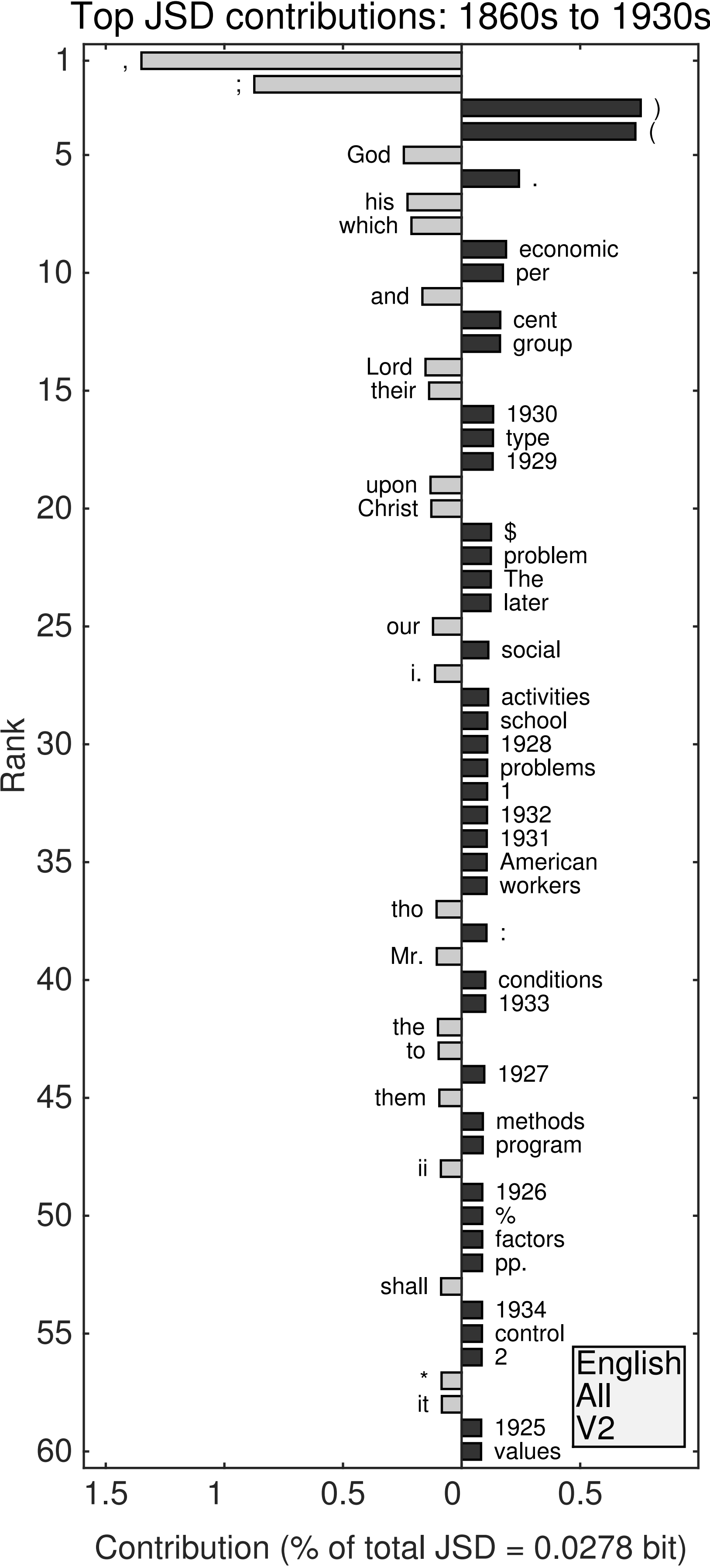
Top JSD contributions: 1860s to 1930s
Rank
Contribution (% of total JSD = 0.0278 bit)
,
;
)
(
God
.
his
which
economic
per
and
cent
group
Lord
their
1930
type
1929
upon
Christ
$
problem
The
later
our
social
i.
activities
school
1928
problems
1
1932
1931
American
workers
tho
:
Mr.
conditions
1933
the
to
1927
them
methods
program
ii
1926
%
factors
pp.
shall
1934
control
2
*
it
1925
values
English
All
V2

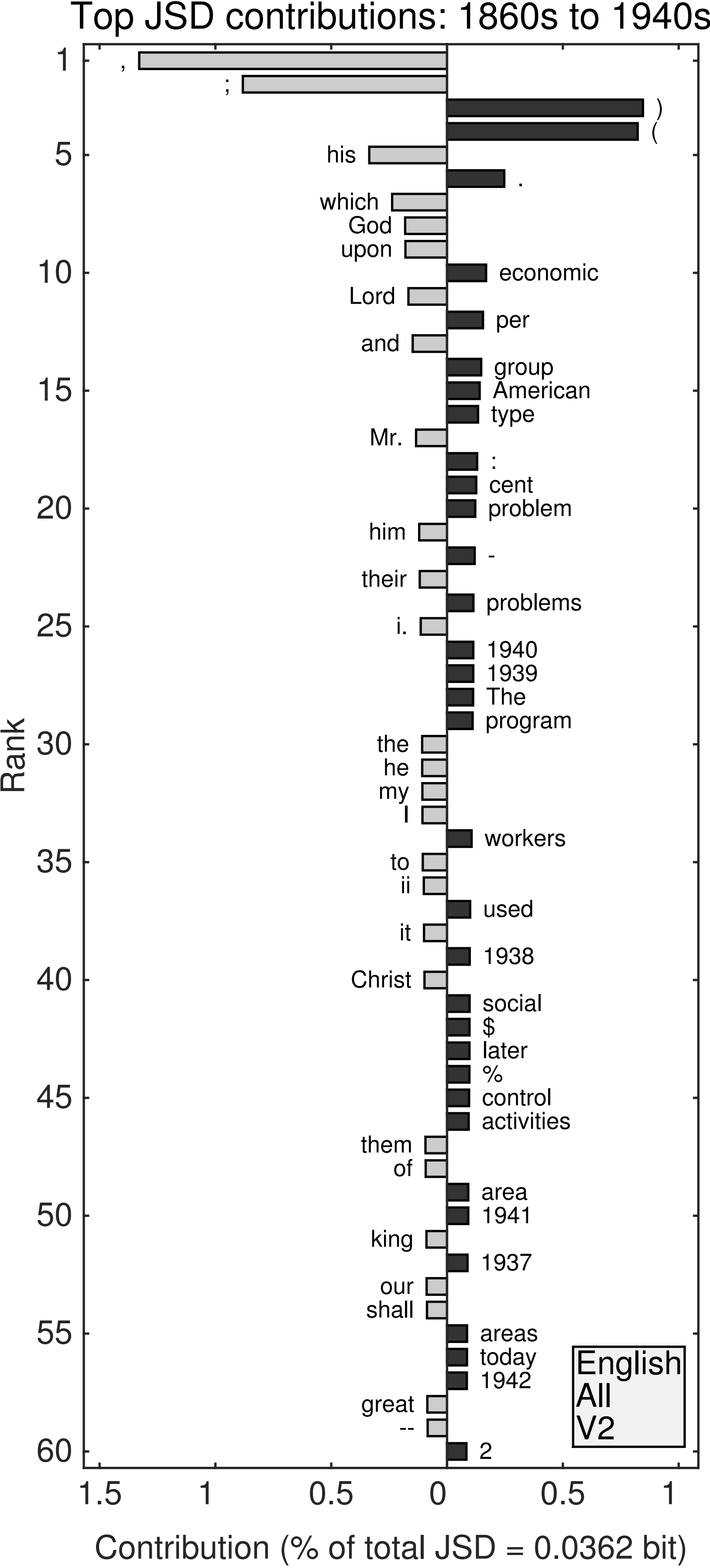

Top JSD contributions: 1860s to 1940s
Rank
Contribution (% of total JSD = 0.0362 bit)
,
;
)
(
his
.
which
God
upon
economic
Lord
per
and
group
American
type
Mr.
:
cent
problem
him
-
their
problems
i.
1940
1939
The
program
the
he
my
I
workers
to
ii
used
it
1938
Christ
social
$
later
%
control
activities
them
of
area
1941
king
1937
our
shall
areas
today
1942
great
--
2
English
All
V2

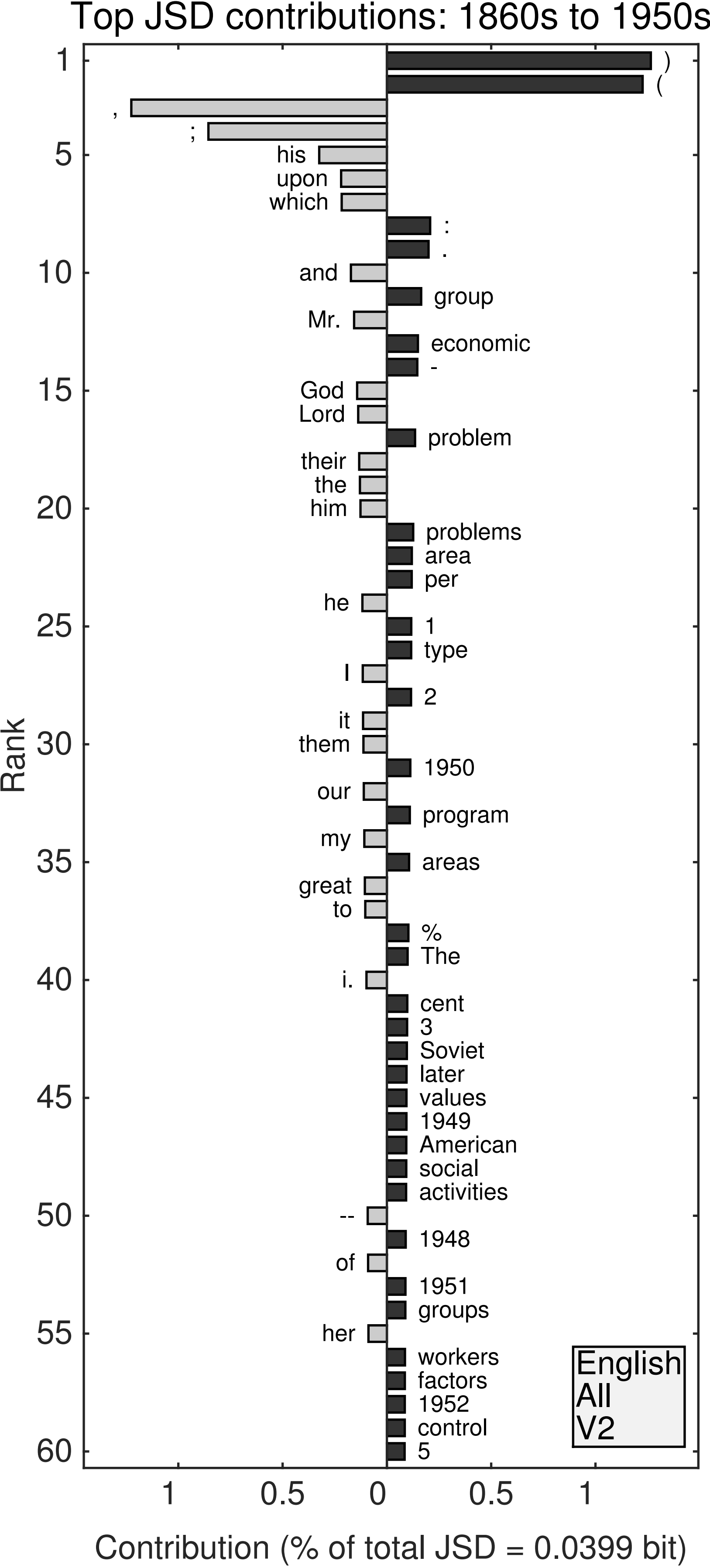

Top JSD contributions: 1860s to 1950s
Rank
Contribution (% of total JSD = 0.0399 bit)
)
(
,
;
his
upon
which
:
.
and
group
Mr.
economic
-
God
Lord
problem
their
the
him
problems
area
per
he
1
type
I
2
it
them
1950
our
program
my
areas
great
to
%
The
i.
cent
3
Soviet
later
values
1949
American
social
activities
--
1948
of
1951
groups
her
workers
factors
1952
control
5
English
All
V2
1 5 10 15 20 25 30 35 40 45 50 55 60
1 0.5 0 0.5 1

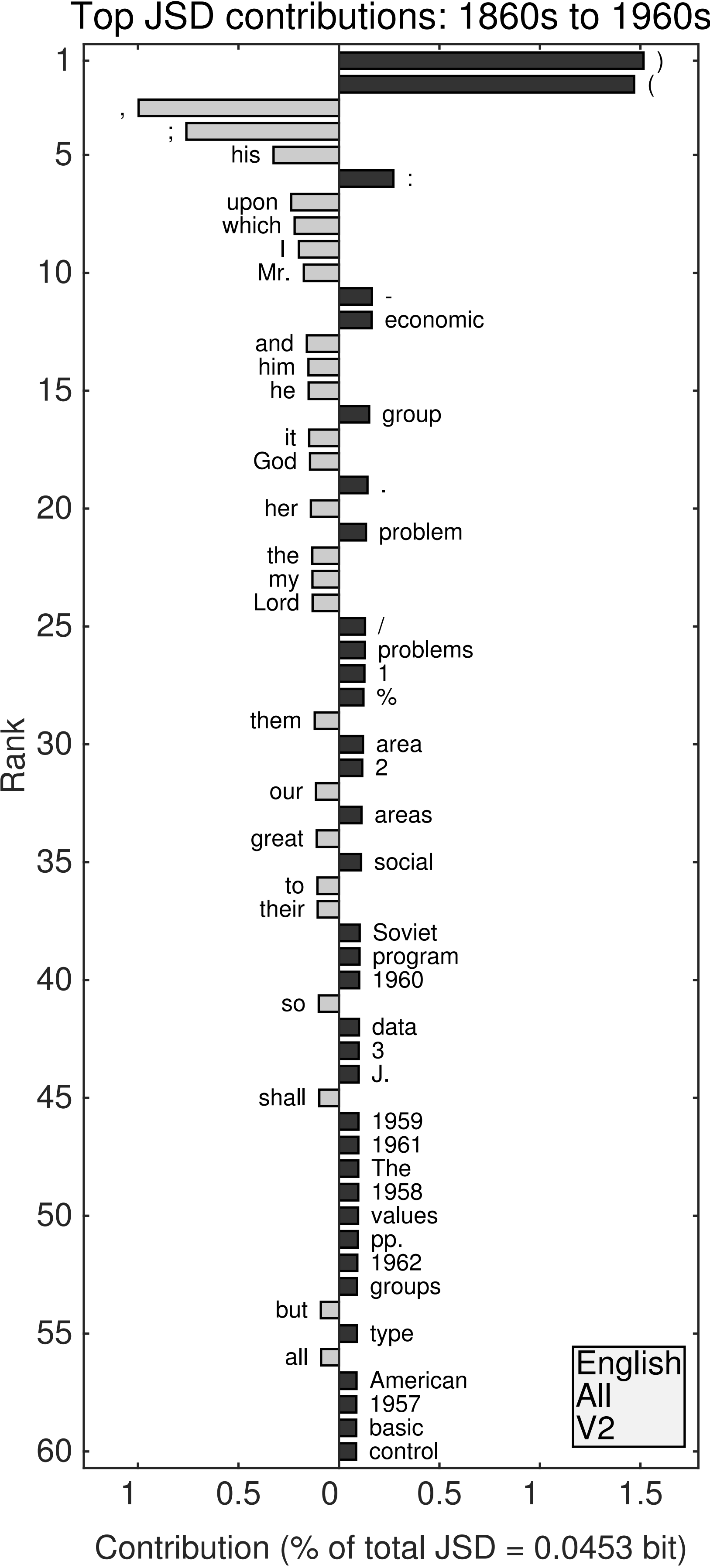

Top JSD contributions: 1860s to 1960s
Rank
Contribution (% of total JSD = 0.0453 bit)
1
5
10
15
20
25
30
35
40
45
50
55
60
1
0.5
0
0.5
1
1.5
)
(
,
;
his
:
upon
which
I
Mr.
-
economic
and
him
he
group
it
God
.
her
problem
the
my
Lord
/
problems
1
%
them
area
2
our
areas
great
social
to
their
Soviet
program
1960
so
data
3
J.
shall
1959
1961
The
1958
values
pp.
1962
groups
but
type
all
American
1957
basic
control
English
All
V2

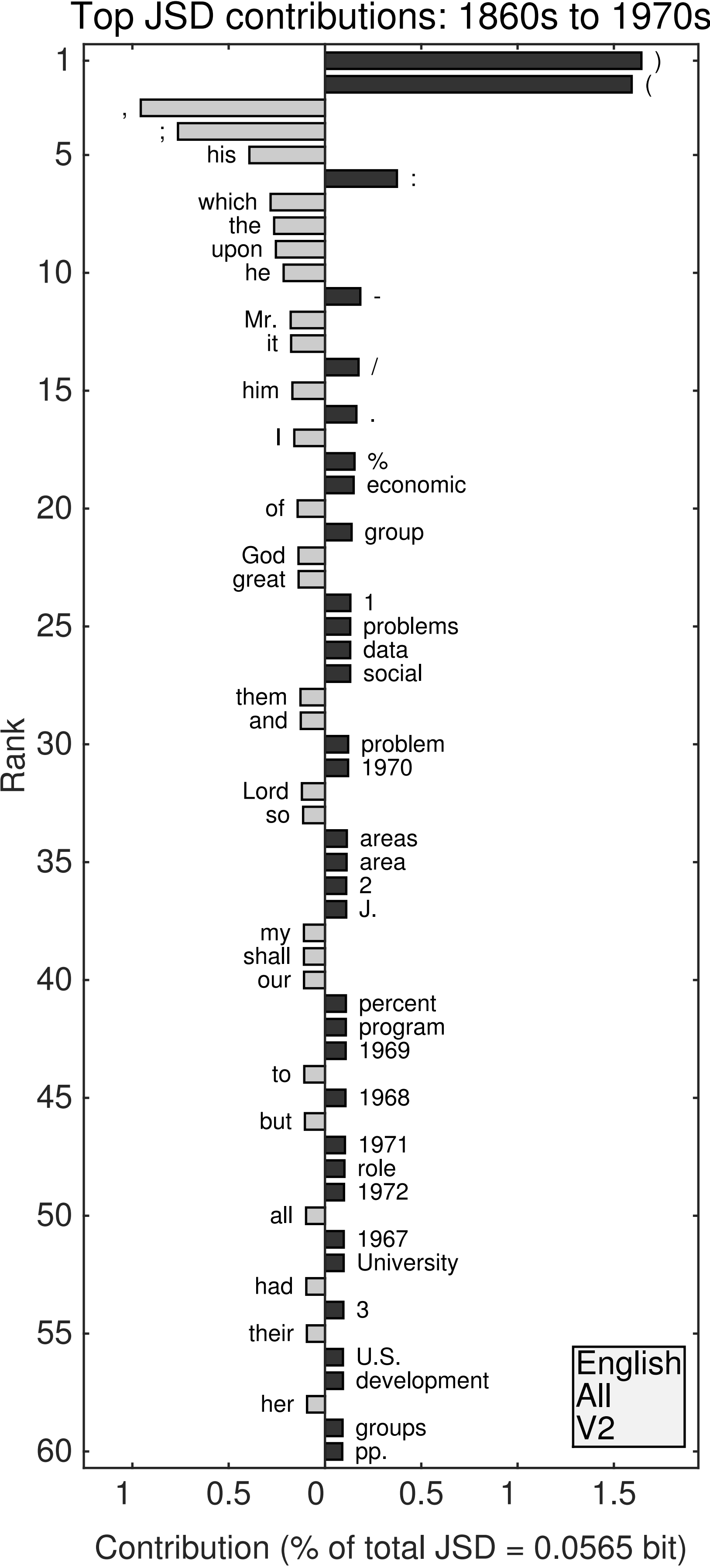
Top JSD contributions: 1860s to 1970s
Rank
Contribution (% of total JSD = 0.0565 bit)
)
(
,
;
his
:
which
the
upon
he
-
Mr.
it
/
him
.
I
%
economic
of
group
God
great
1
problems
data
social
them
and
problem
1970
Lord
so
areas
area
2
J.
my
shall
our
percent
program
1969
to
1968
but
1971
role
1972
all
1967
University
had
3
their
U.S.
development
her
groups
pp.
English
All
V2

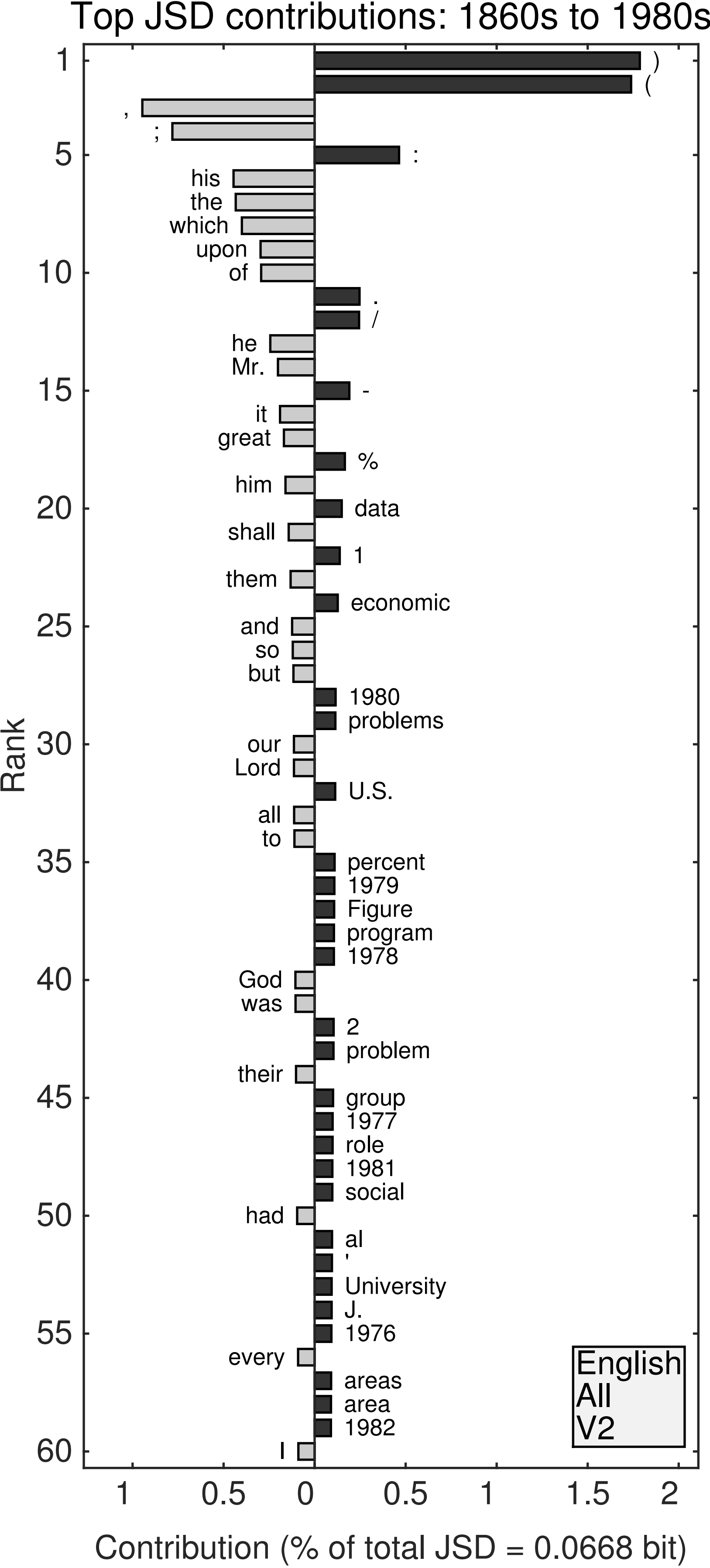
Top JSD contributions: 1860s to 1980s
Rank
Contribution (% of total JSD = 0.0668 bit)
English
All
V2
)
(
,
;
:
his
the
which
upon
of
.
/
he
Mr.
-
it
great
%
him
data
shall
1
them
economic
and
so
but
1980
problems
our
Lord
U.S.
all
to
percent
1979
Figure
program
1978
God
was
2
problem
their
group
1977
role
1981
social
had
al
'
University
J.
1976
every
areas
area
1982
I

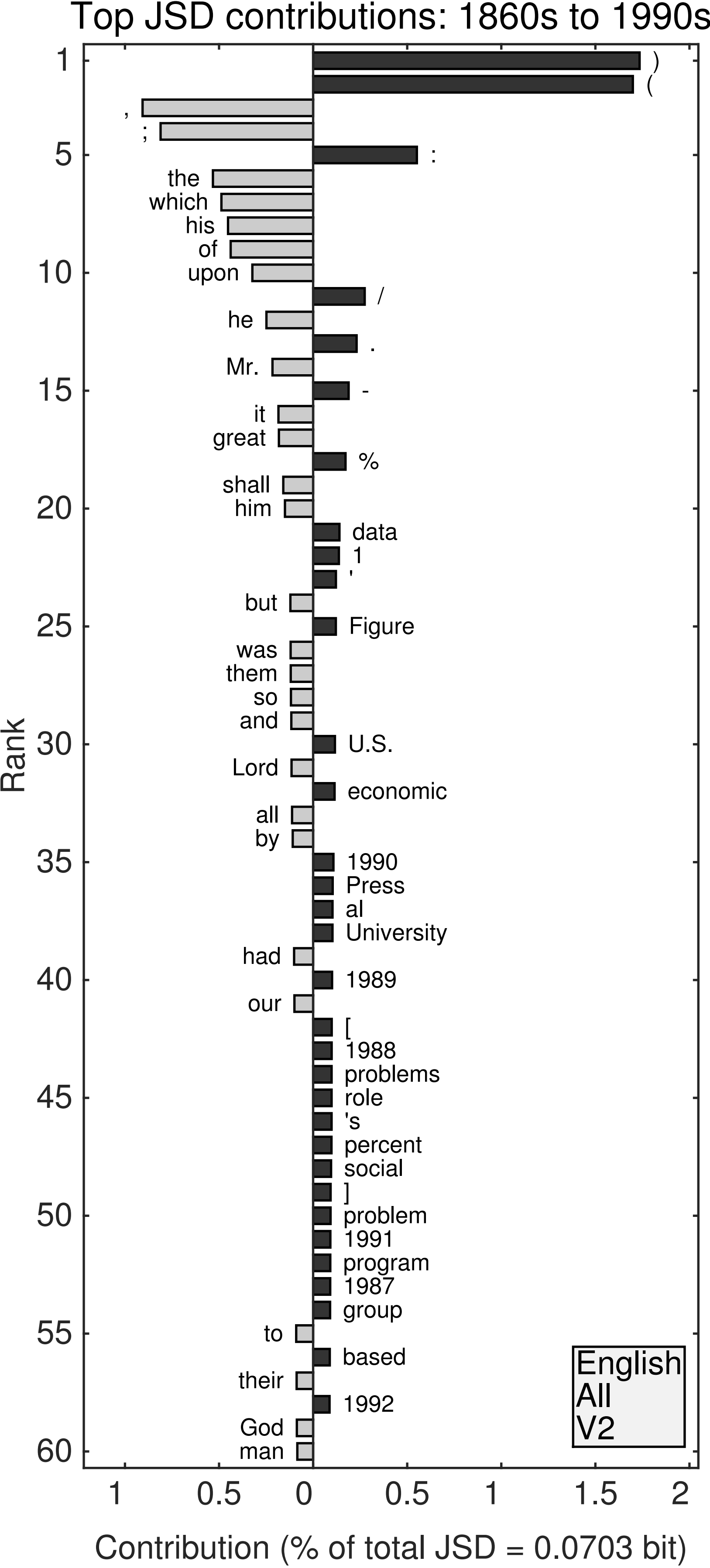

Top JSD contributions: 1860s to 1990s
Rank
Contribution (% of total JSD = 0.0703 bit)
)
(
,
;
:
the
which
his
of
upon
/
he
.
Mr.
-
it
great
%
shall
him
data
1
'
but
Figure
was
them
so
and
U.S.
Lord
economic
all
by
1990
Press
al
University
had
1989
our
[
1988
problems
role
's
percent
social
]
problem
1991
program
1987
group
to
based
their
1992
God
man
English
All
V2

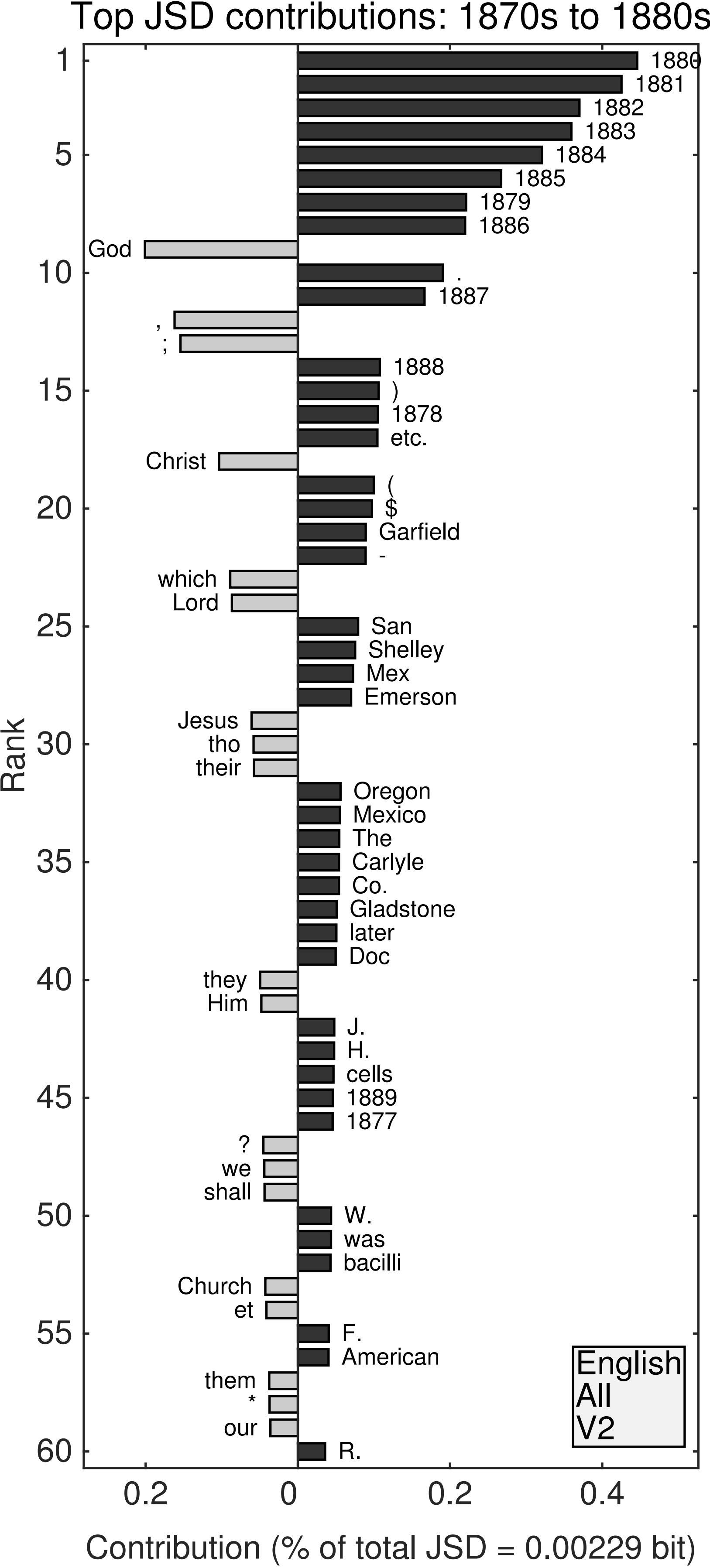

Top JSD contributions: 1870s to 1880s
Rank
Contribution (% of total JSD = 0.00229 bit)
1880
1881
1882
1883
1884
1885
1879
1886
God
.
1887
,
;
1888
)
1878
etc.
Christ
(
$
Garfield
-
which
Lord
San
Shelley
Mex
Emerson
Jesus
tho
their
Oregon
Mexico
The
Carlyle
Co.
Gladstone
later
Doc
they
Him
J.
H.
cells
1889
1877
?
we
shall
W.
was
bacilli
Church
et
F.
American
them
*
our
R.
English
All
V2

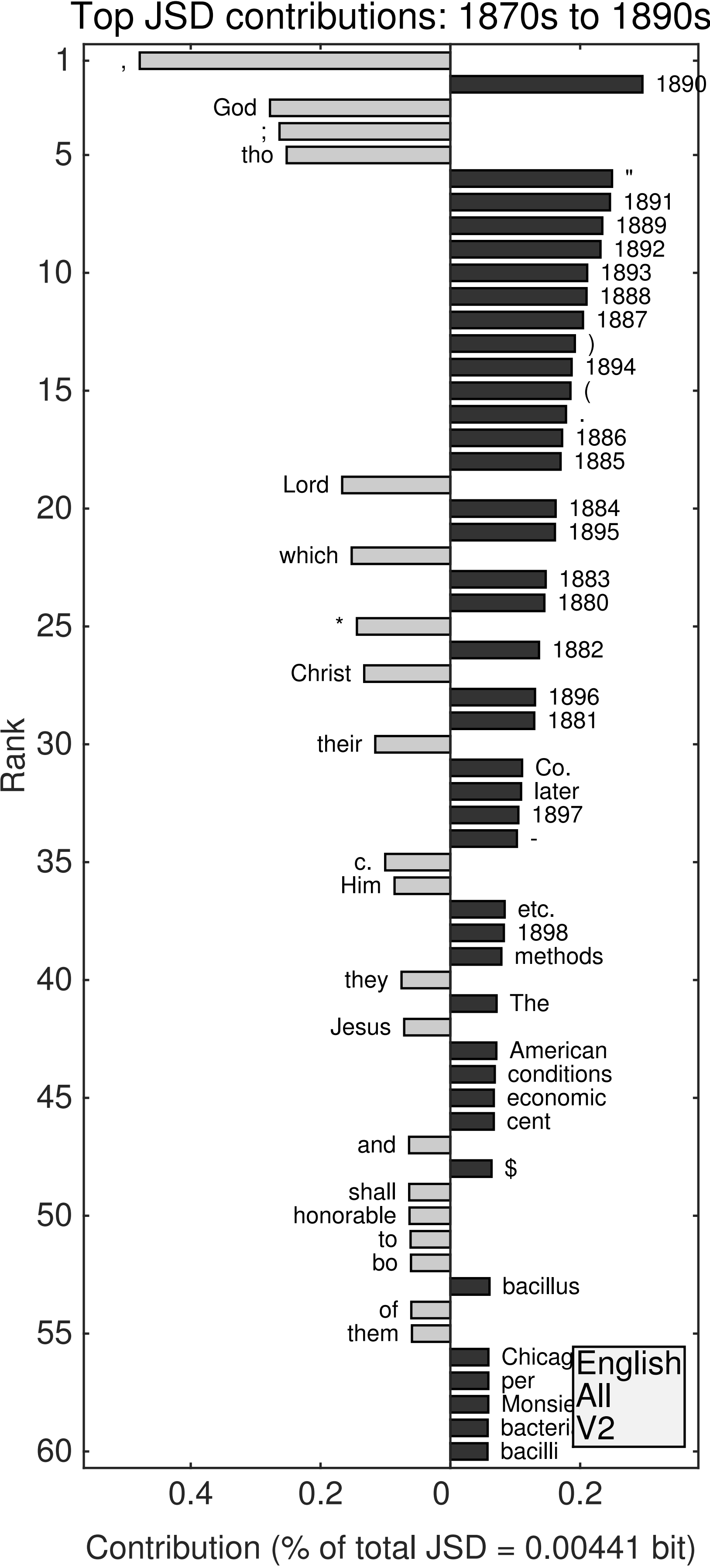
Top JSD contributions: 1870s to 1890s
Rank
Contribution (% of total JSD = 0.00441 bit)
0.4
0.2
0
0.2
1
5
10
15
20
25
30
35
40
45
50
55
60
,
1890
God
;
tho
"
1891
1889
1892
1893
1888
1887
)
1894
(
.
1886
1885
Lord
1884
1895
which
1883
1880
*
1882
Christ
1896
1881
their
Co.
later
1897
-
c.
Him
etc.
1898
methods
they
The
Jesus
American
conditions
economic
cent
and
$
shall
honorable
to
bo
bacillus
of
them
per
bacilli
English
All
V2

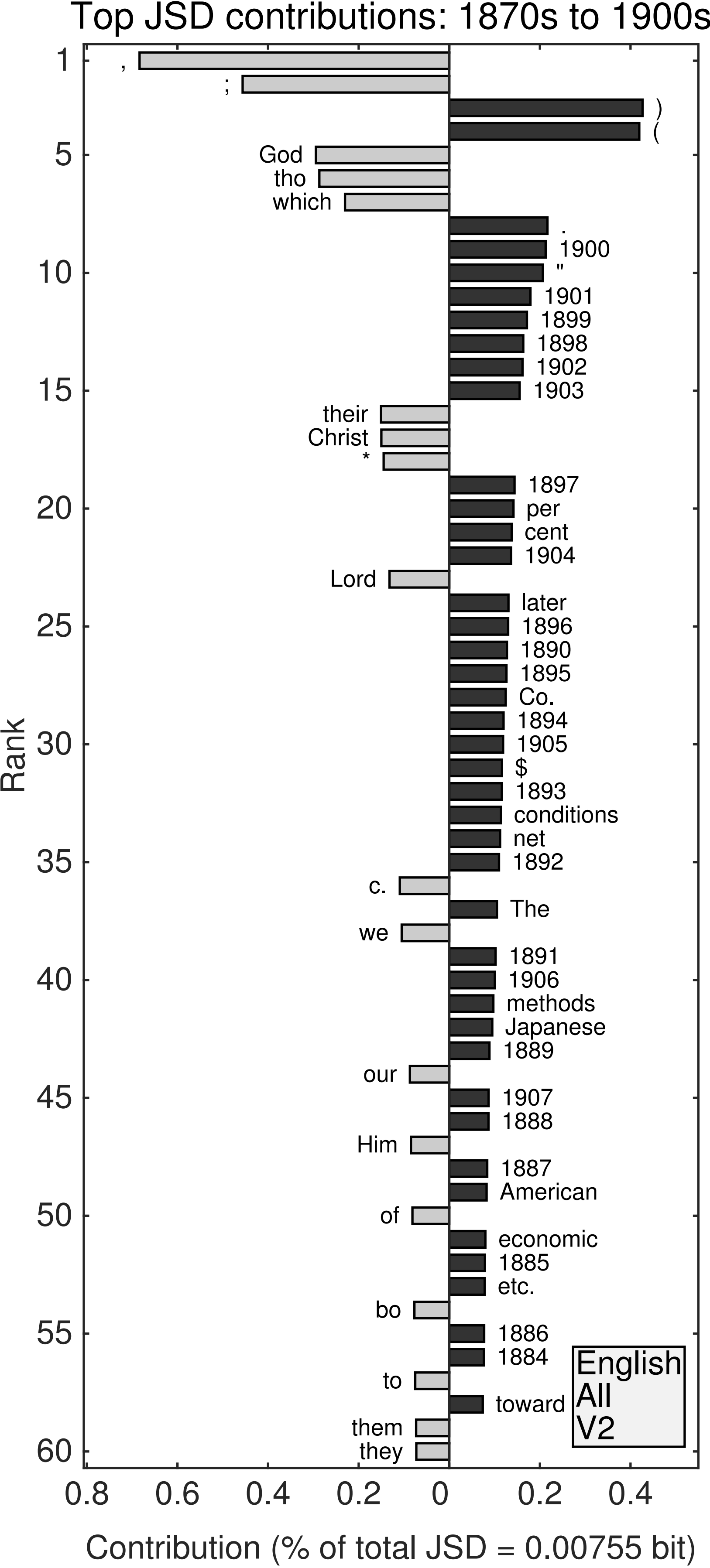

Top JSD contributions: 1870s to 1900s
Rank
Contribution (% of total JSD = 0.00755 bit)
English
All
V2
,
;
)
(
God
tho
which
.
1900
"
1901
1899
1898
1902
1903
their
Christ
*
1897
per
cent
1904
Lord
later
1896
1890
1895
Co.
1894
1905
$
1893
conditions
net
1892
c.
The
we
1891
1906
methods
Japanese
1889
our
1907
1888
Him
1887
American
of
economic
1885
etc.
bo
1886
1884
to
toward
them
they

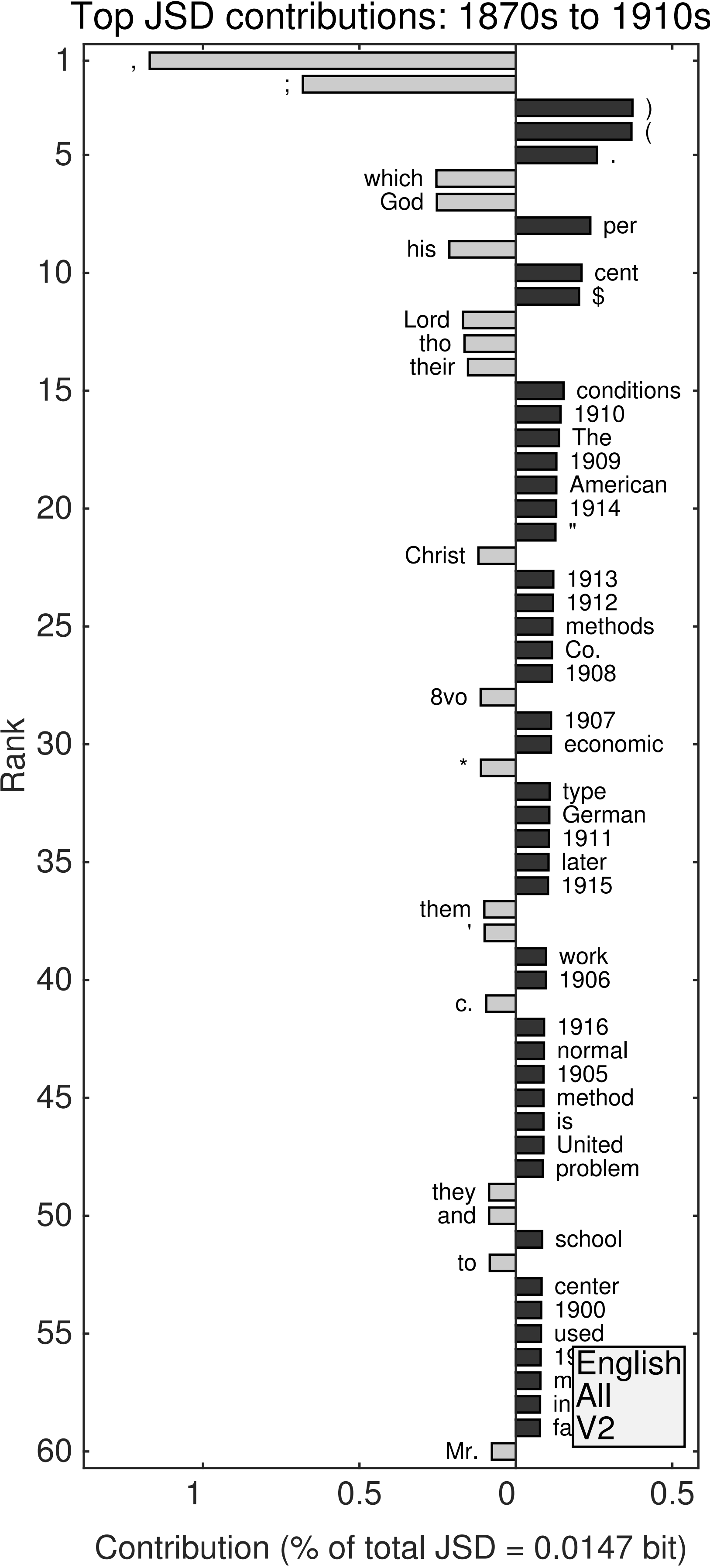

Top JSD contributions: 1870s to 1910s
Rank
1
5
10
15
20
25
30
35
40
45
50
55
60
,
;
)
(
.
which
God
per
his
cent
$
Lord
tho
their
conditions
1910
The
1909
American
1914
"
Christ
1913
1912
methods
Co.
1908
8vo
1907
economic
*
type
German
1911
later
1915
them
'
work
1906
c.
1916
normal
1905
method
is
United
problem
they
and
school
to
center
1900
used
Mr.
English
All
V2
1
0.5
0
0.5
Contribution (% of total JSD = 0.0147 bit)

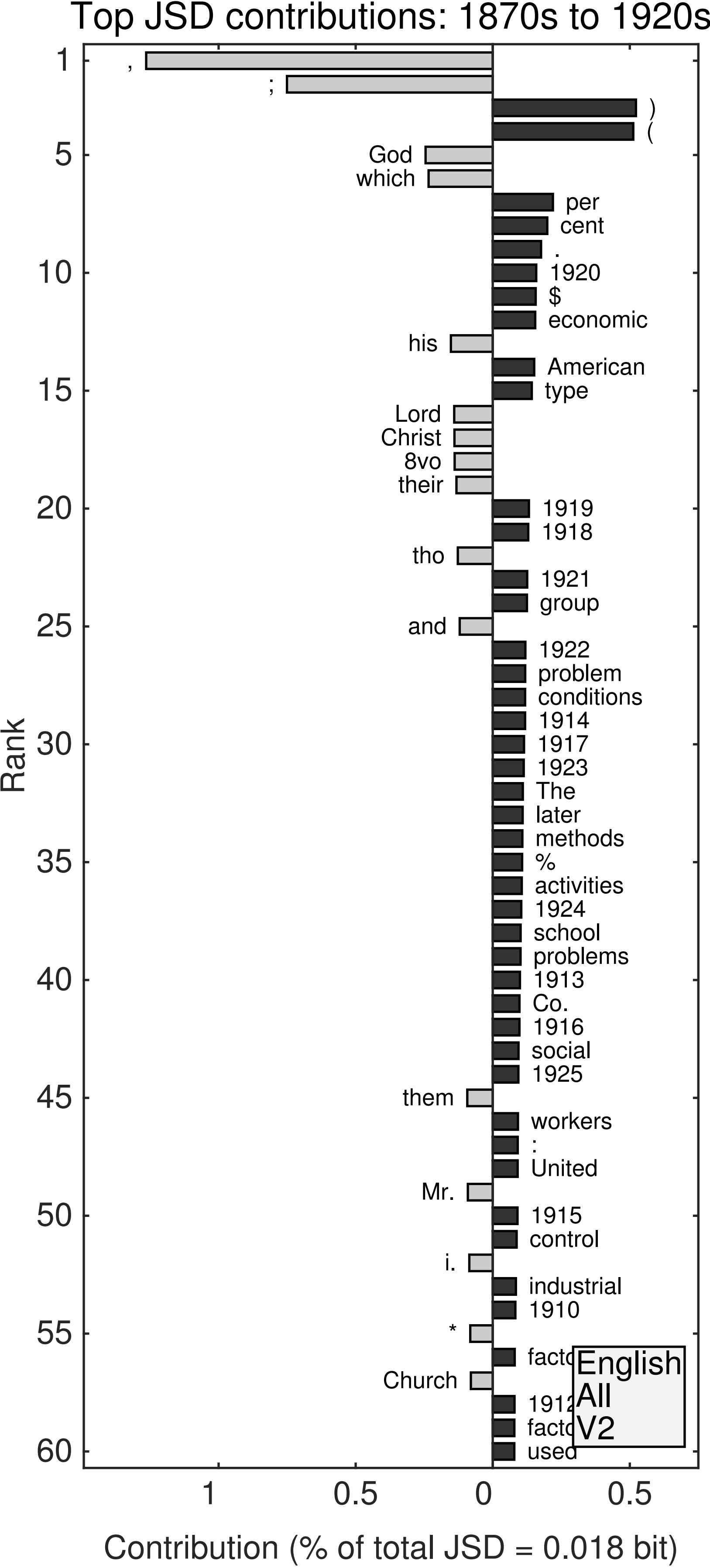

Top JSD contributions: 1870s to 1920s
Rank
Contribution (% of total JSD = 0.018 bit)
,
;
)
(
God
which
per
cent
.
1920
$
economic
his
American
type
Lord
Christ
8vo
their
1919
1918
tho
1921
group
and
1922
problem
conditions
1914
1917
1923
The
later
methods
%
activities
1924
school
problems
1913
Co.
1916
social
1925
them
workers
:
United
Mr.
1915
control
i.
industrial
1910
*
Church
used
English
All
V2

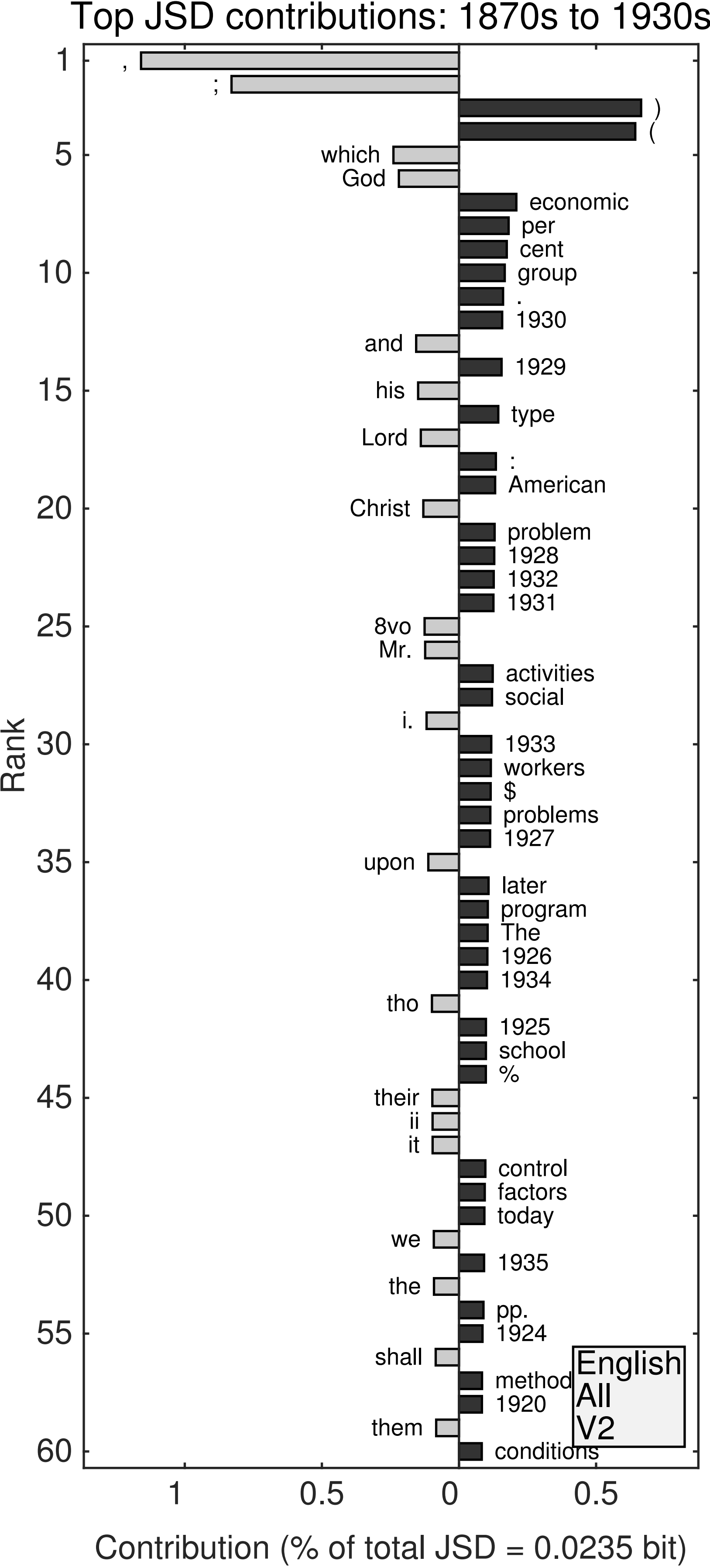
Top JSD contributions: 1870s to 1930s
Rank
Contribution (% of total JSD = 0.0235 bit)
,
;
)
(
which
God
economic
per
cent
group
.
1930
and
1929
his
type
Lord
:
American
Christ
problem
1928
1932
1931
8vo
Mr.
activities
social
i.
1933
workers
$
problems
1927
upon
later
program
The
1926
1934
tho
1925
school
%
their
ii
it
control
factors
today
we
1935
the
pp.
1924
shall
method
1920
them
conditions
English
All
V2

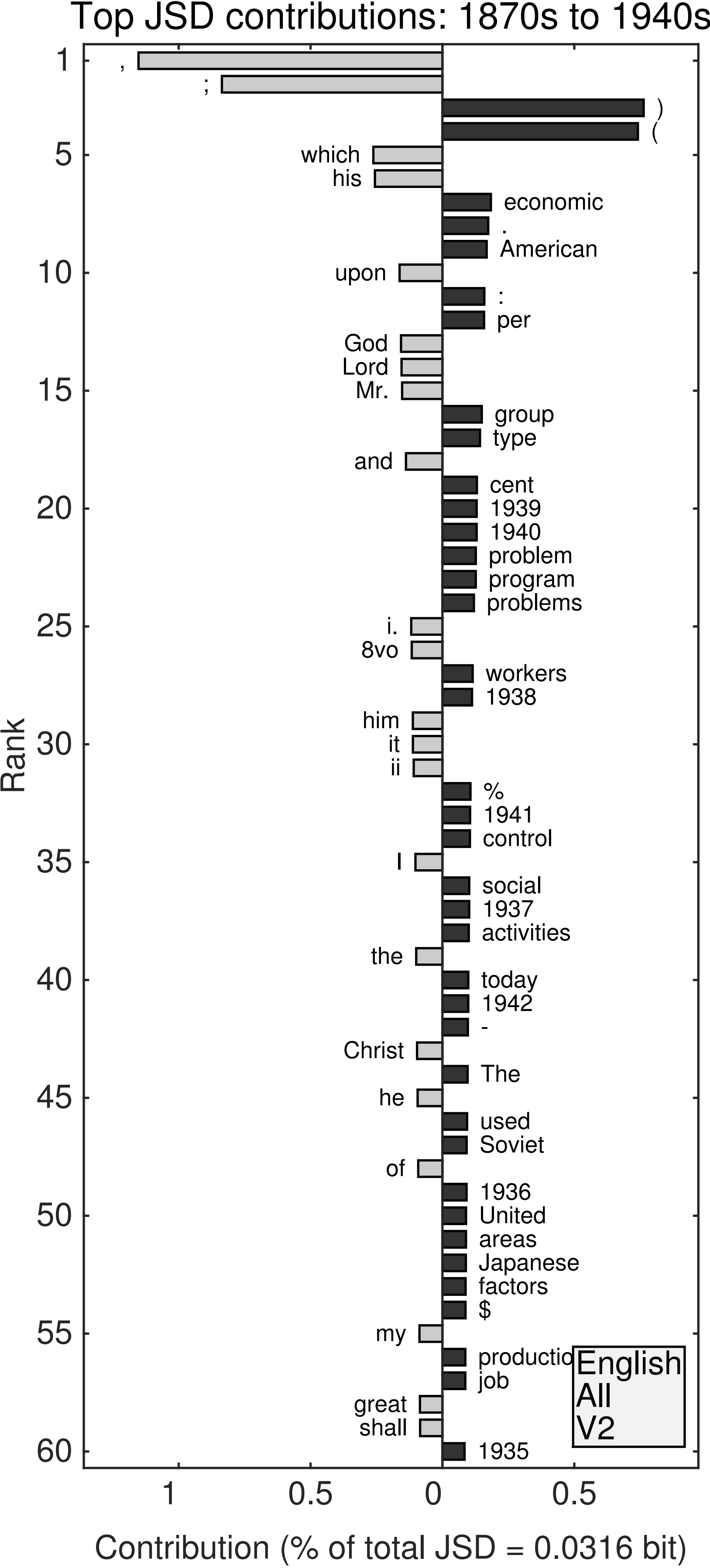

Top JSD contributions: 1870s to 1940s
Rank
Contribution (% of total JSD = 0.0316 bit)
,
;
)
(
which
his
economic
.
American
upon
:
per
God
Lord
Mr.
group
type
and
cent
1939
1940
problem
program
problems
i.
8vo
workers
1938
him
it
ii
%
1941
control
I
social
1937
activities
the
today
1942
-
Christ
The
he
used
Soviet
of
1936
United
areas
Japanese
factors
$
my
productio
job
great
shall
1935
English
All
V2

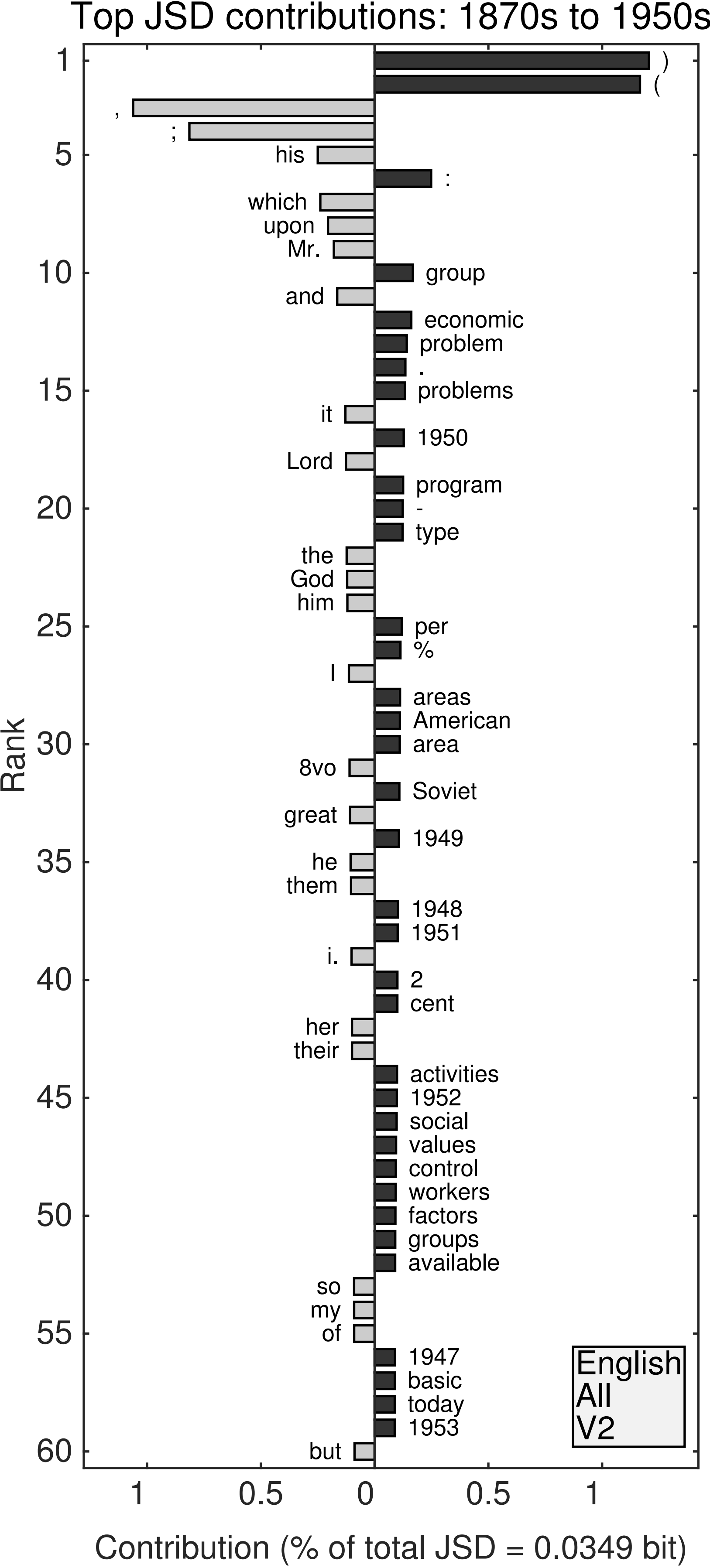
Top JSD contributions: 1870s to 1950s
Rank
)
(
,
;
his
:
which
upon
Mr.
group
and
economic
problem
.
problems
it
1950
Lord
program
-
type
the
God
him
per
%
I
areas
American
area
8vo
Soviet
great
1949
he
them
1948
1951
i.
2
cent
her
their
activities
1952
social
values
control
workers
factors
groups
available
so
my
of
1947
basic
today
1953
but
English
All
V2
1
5
10
15
20
25
30
35
40
45
50
55
60
1
0.5
0
0.5
1
Contribution (% of total JSD = 0.0349 bit)

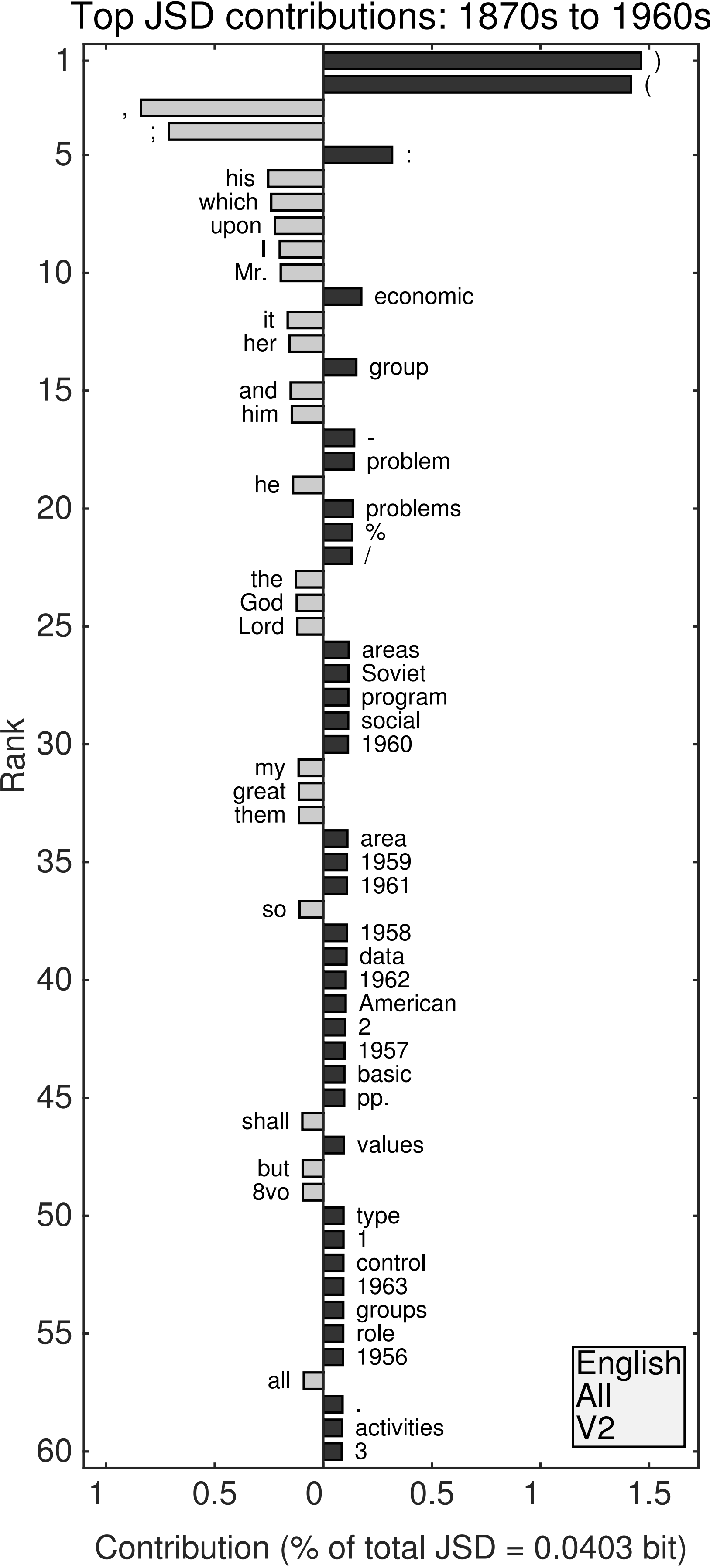
Top JSD contributions: 1870s to 1960s
Rank
Contribution (% of total JSD = 0.0403 bit)
)
(
,
;
:
his
which
upon
I
Mr.
economic
it
her
group
and
him
-
problem
he
problems
%
/
the
God
Lord
areas
Soviet
program
social
1960
my
great
them
area
1959
1961
so
1958
data
1962
American
2
1957
basic
pp.
shall
values
but
8vo
type
1
control
1963
groups
role
1956
all
.
activities
3
English
All
V2

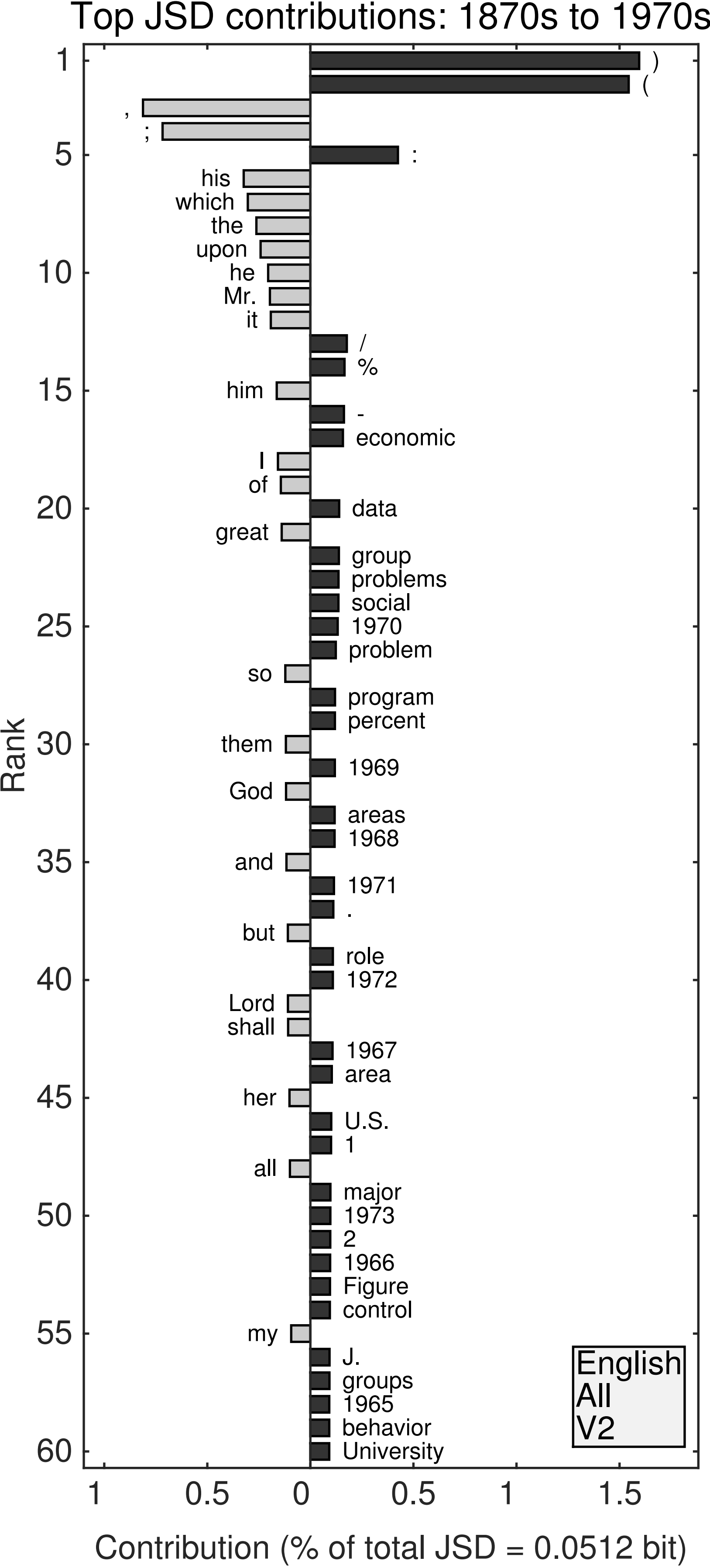

Top JSD contributions: 1870s to 1970s
Rank
Contribution (% of total JSD = 0.0512 bit)
1
5
10
15
20
25
30
35
40
45
50
55
60
1
0.5
0
0.5
1
1.5
)
(
,
;
:
his
which
the
upon
he
Mr.
it
/
%
him
-
economic
I
of
data
great
group
problems
social
1970
problem
so
program
percent
them
1969
God
areas
1968
and
1971
.
but
role
1972
Lord
shall
1967
area
her
U.S.
1
all
major
1973
2
1966
Figure
control
my
J.
groups
1965
behavior
University
English
All
V2

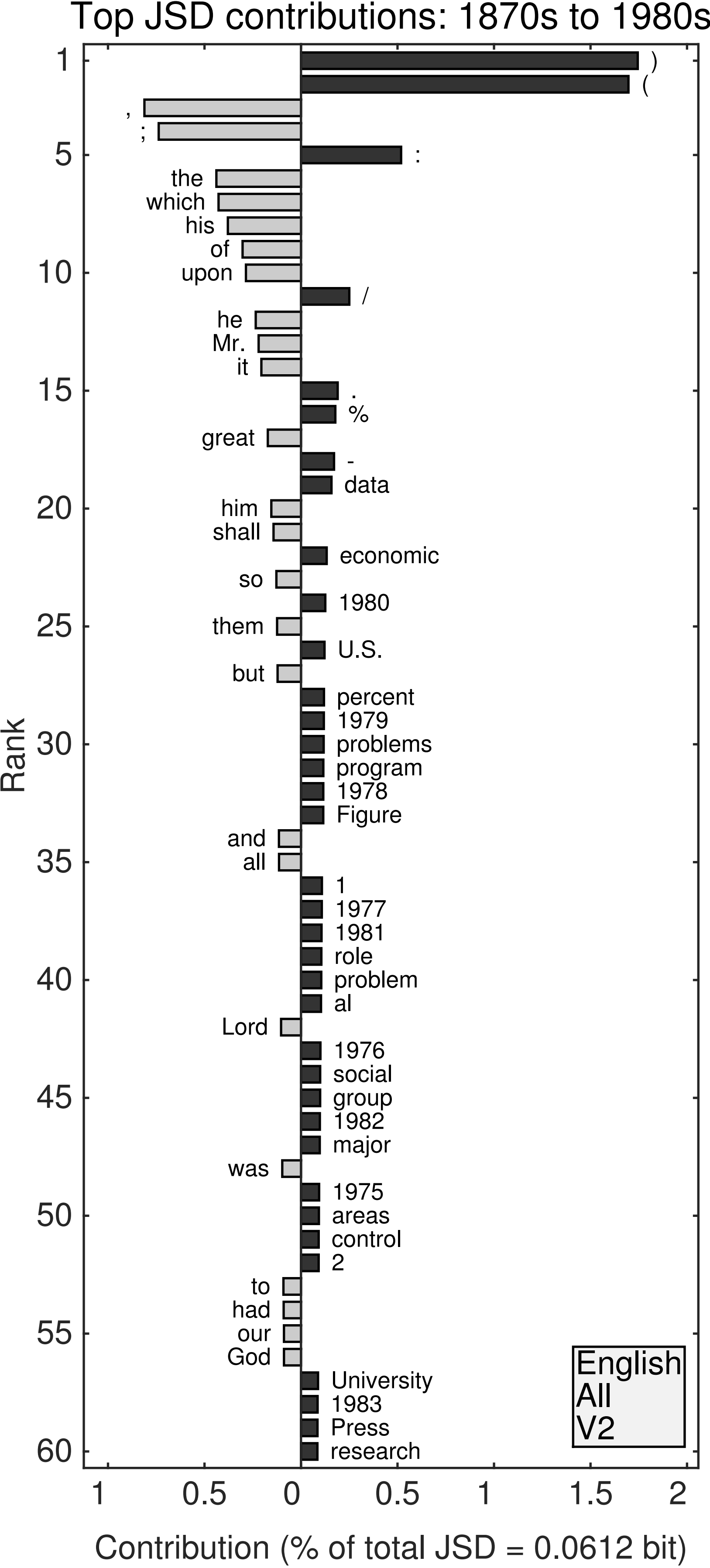

Top JSD contributions: 1870s to 1980s
Rank
1
5
10
15
20
25
30
35
40
45
50
55
60
)
(
,
;
:
the
which
his
of
upon
/
he
Mr.
it
.
%
great
-
data
him
shall
economic
so
1980
them
U.S.
but
percent
1979
problems
program
1978
Figure
and
all
1
1977
1981
role
problem
al
Lord
1976
social
group
1982
major
was
1975
areas
control
2
to
had
our
God
University
1983
Press
research
English
All
V2
1
0.5
0
0.5
1
1.5
2
Contribution (% of total JSD = 0.0612 bit)

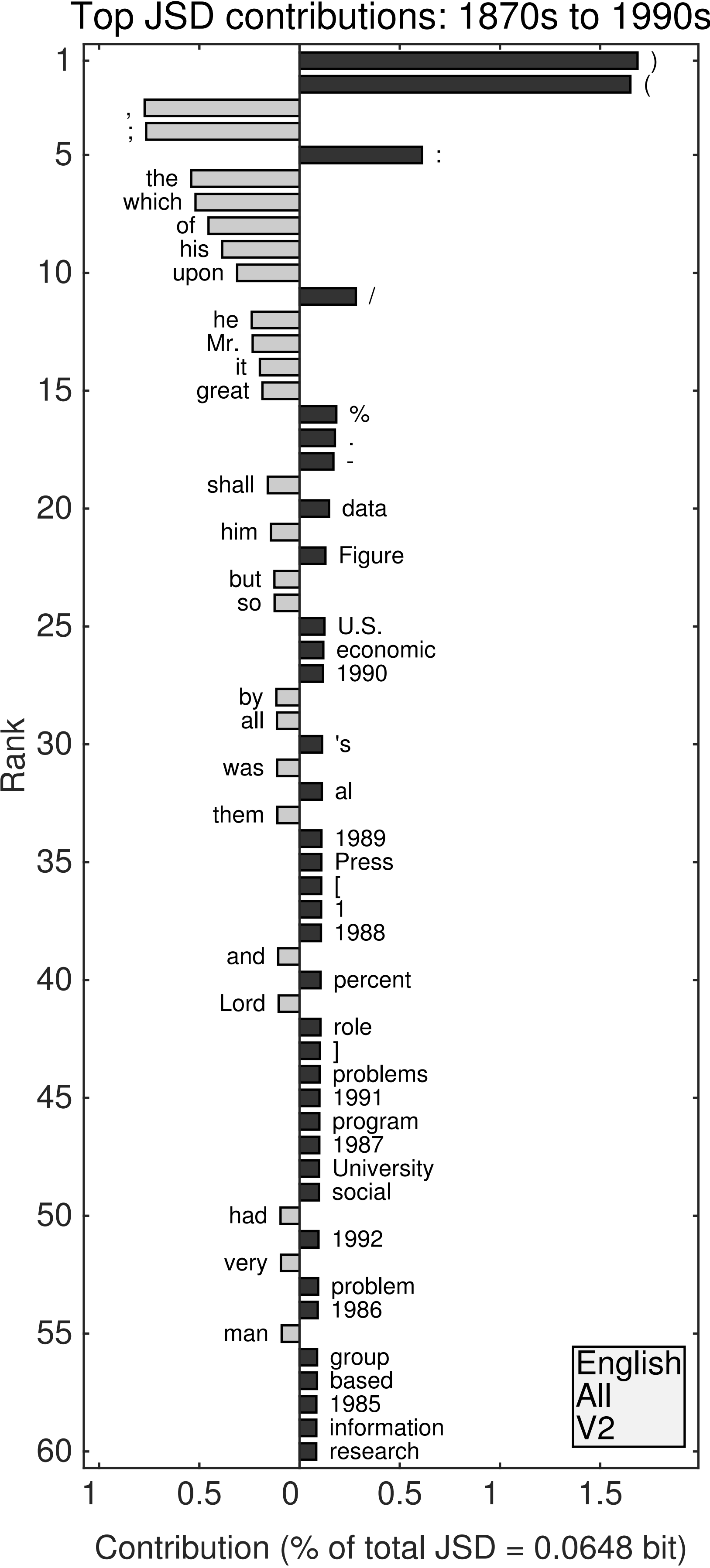
Top JSD contributions: 1870s to 1990s
Rank
Contribution (% of total JSD = 0.0648 bit)
English
All
V2
)
(
,
;
:
the
which
of
his
upon
/
he
Mr.
it
great
%
.
-
shall
data
him
Figure
but
so
U.S.
economic
1990
by
all
's
was
al
them
1989
Press
[
1
1988
and
percent
Lord
role
]
problems
1991
program
1987
University
social
had
1992
very
problem
1986
man
group
based
1985
information
research

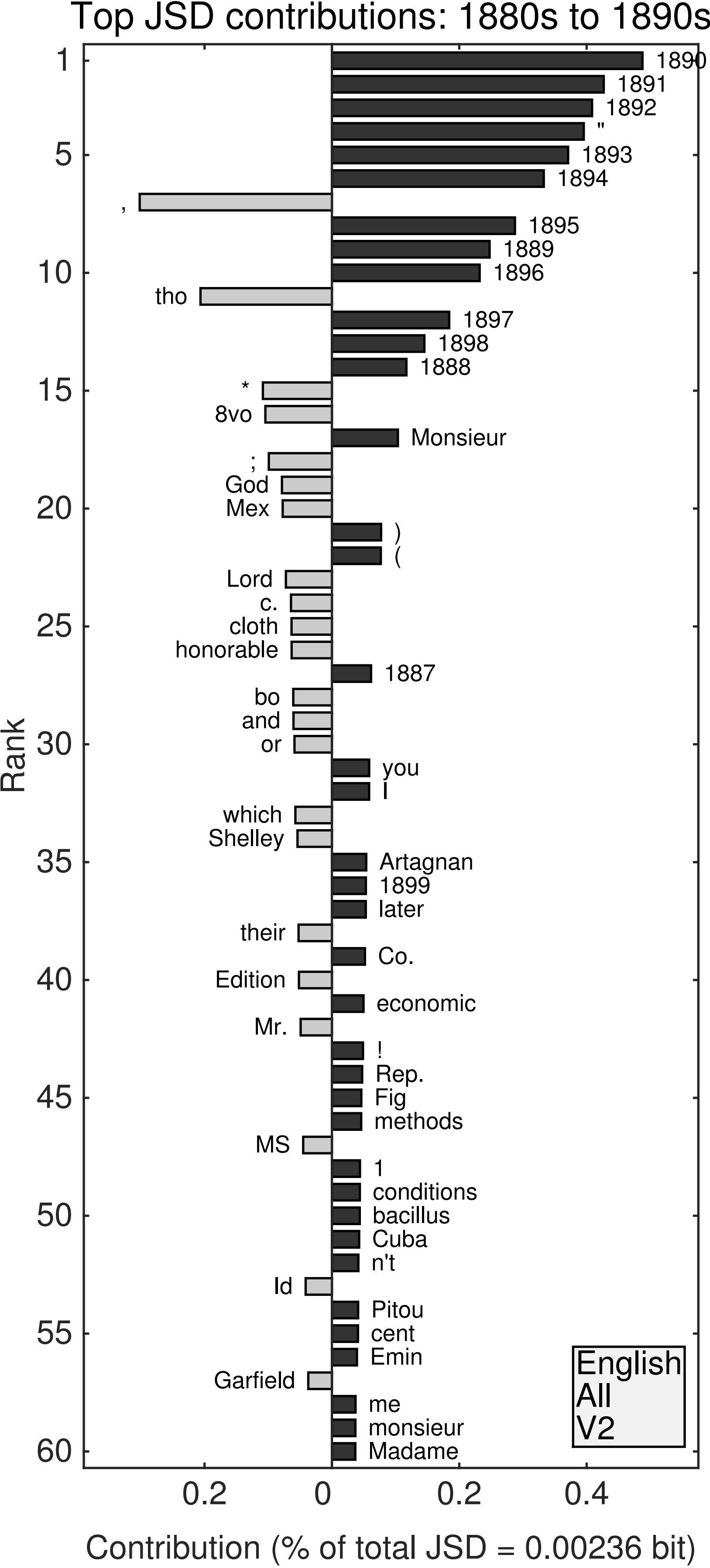

Top JSD contributions: 1880s to 1890s
Rank
1
5
10
15
20
25
30
35
40
45
50
55
60
1890
1891
1892
"
1893
1894
,
1895
1889
1896
tho
1897
1898
1888
*
8vo
Monsieur
;
God
Mex
)
(
Lord
c.
cloth
honorable
1887
bo
and
or
you
I
which
Shelley
Artagnan
1899
later
their
Co.
Edition
economic
Mr.
!
Rep.
Fig
methods
MS
1
conditions
bacillus
Cuba
n't
Id
Pitou
cent
Emin
Garfield
me
monsieur
Madame
English
All
V2
0.2
0
0.2
0.4
Contribution (% of total JSD = 0.00236 bit)

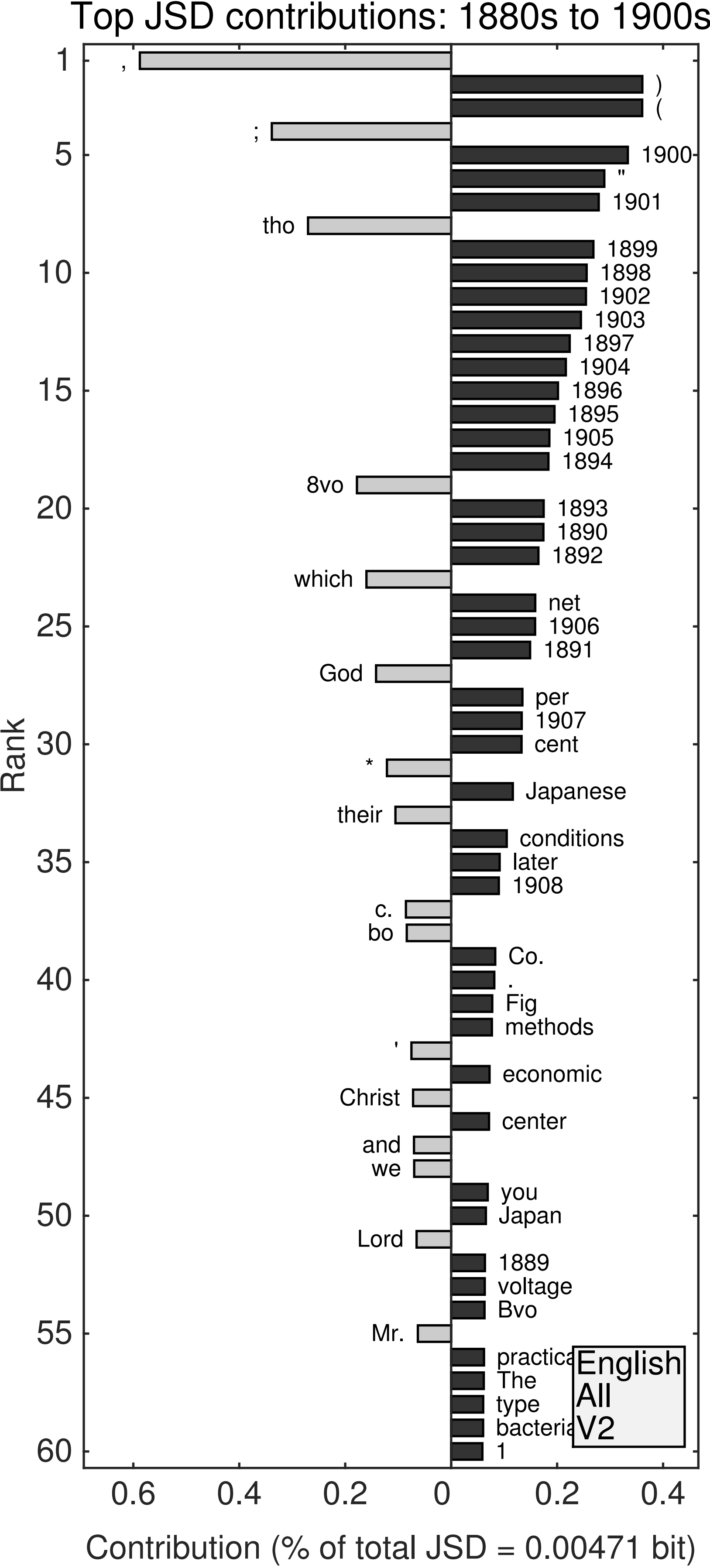
Top JSD contributions: 1880s to 1900s
Rank
Contribution (% of total JSD = 0.00471 bit)
,
)
(
;
1900
"
1901
tho
1899
1898
1902
1903
1897
1904
1896
1895
1905
1894
8vo
1893
1890
1892
which
net
1906
1891
God
per
1907
cent
*
Japanese
their
conditions
later
1908
c.
bo
Co.
.
Fig
methods
'
economic
Christ
center
and
we
you
Japan
Lord
1889
voltage
Bvo
Mr.
The
type
1
English
All
V2

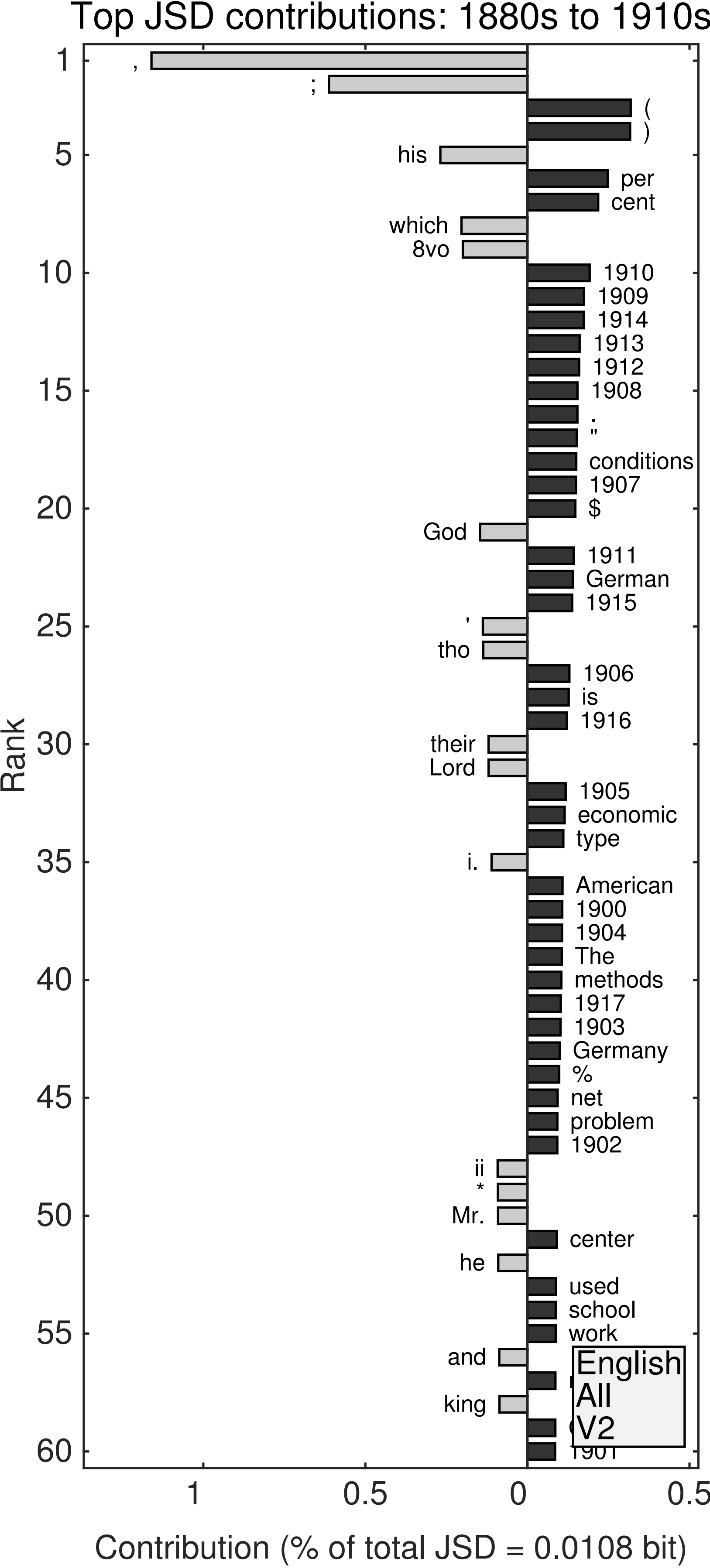
Top JSD contributions: 1880s to 1910s
Rank
Contribution (% of total JSD = 0.0108 bit)
1
5
10
15
20
25
30
35
40
45
50
55
60
1
0.5
0
0.5
,
;
(
)
his
per
cent
which
8vo
1910
1909
1914
1913
1912
1908
:
"
conditions
1907
$
God
1911
German
1915
'
tho
1906
is
1916
their
Lord
1905
economic
type
i.
American
1900
1904
The
methods
1917
1903
Germany
%
net
problem
1902
ii
*
Mr.
center
he
used
school
work
and
king
English
All
V2

Top JSD contributions: 1880s to 1920s

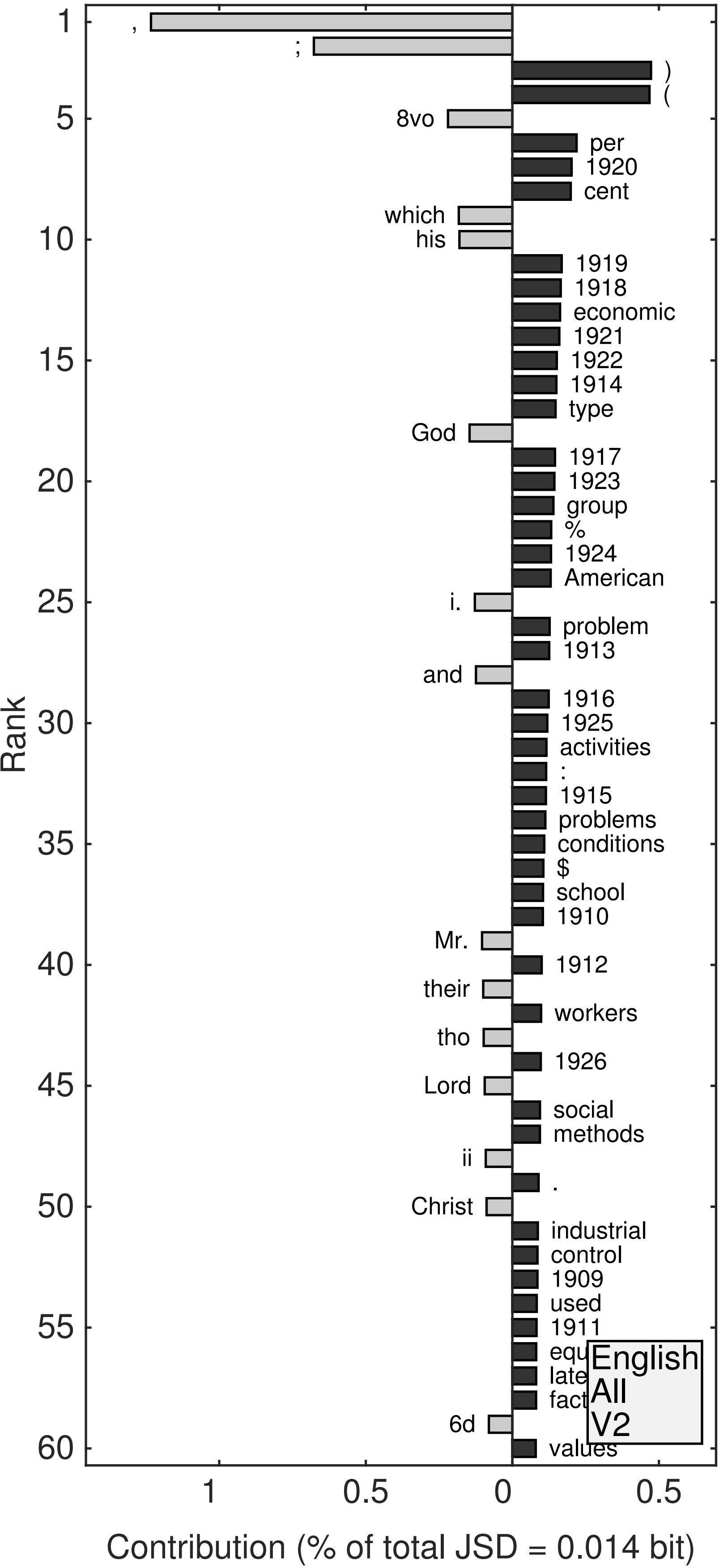

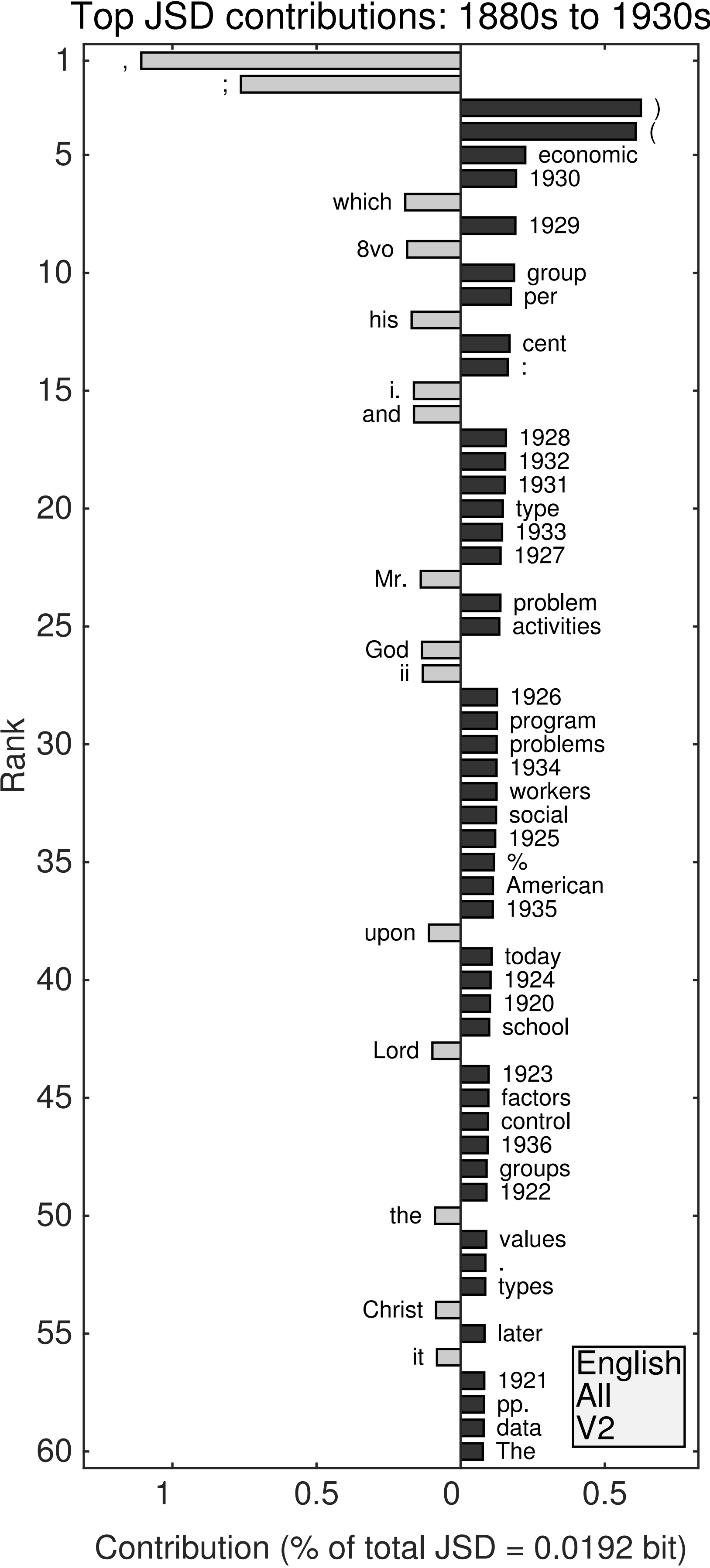
Top JSD contributions: 1880s to 1930s
Rank
Contribution (% of total JSD = 0.0192 bit)
,
;
)
(
economic
1930
which
1929
8vo
group
per
his
cent
:
i.
and
1928
1932
1931
type
1933
1927
Mr.
problem
activities
God
ii
1926
program
problems
1934
workers
social
1925
%
American
1935
upon
today
1924
1920
school
Lord
1923
factors
control
1936
groups
1922
the
values
.
types
Christ
later
it
1921
pp.
data
The
English
All
V2

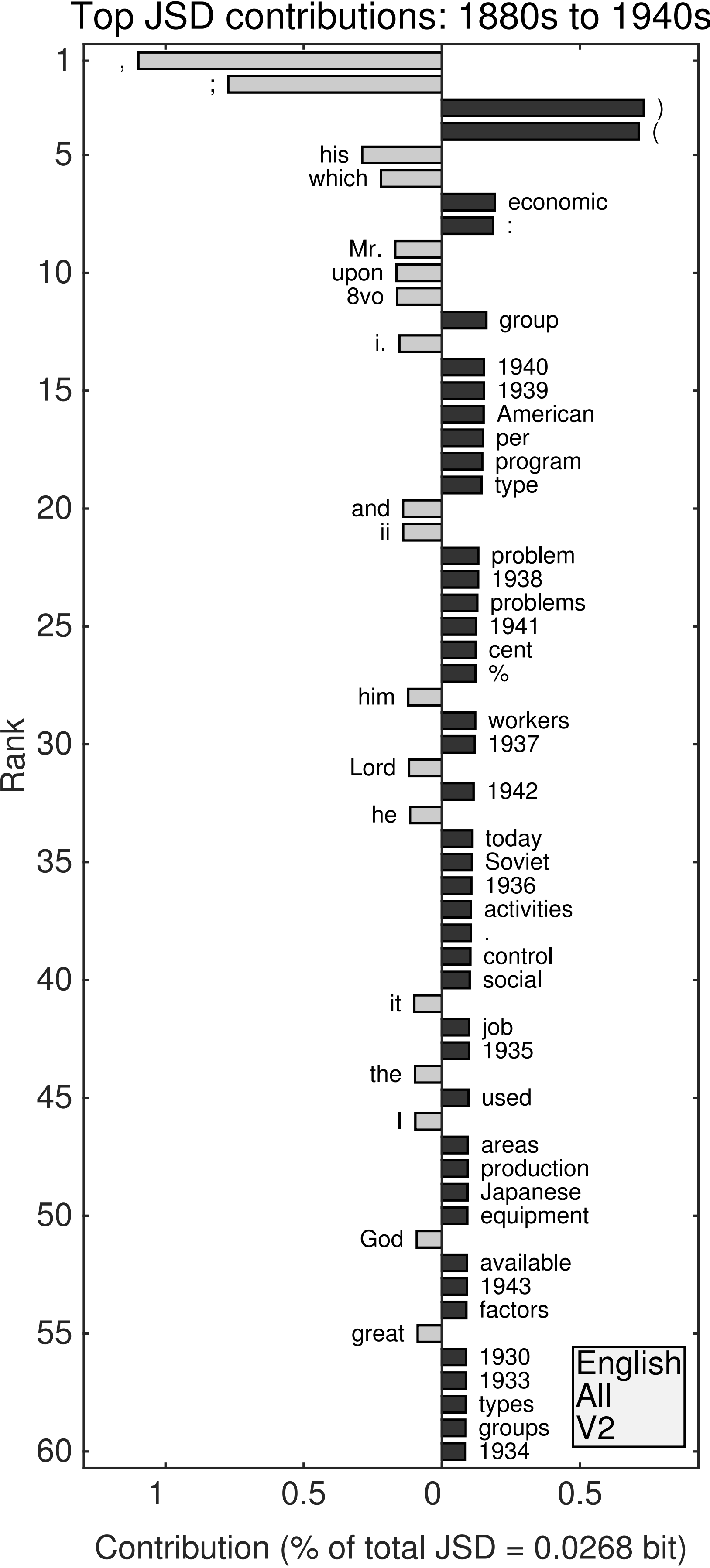

Top JSD contributions: 1880s to 1940s
Rank
Contribution (% of total JSD = 0.0268 bit)
,
;
)
(
his
which
economic
:
Mr.
upon
8vo
group
i.
1940
1939
American
per
program
type
and
ii
problem
1938
problems
1941
cent
%
him
workers
1937
Lord
1942
he
today
Soviet
1936
activities
.
control
social
it
job
1935
the
used
I
areas
production
Japanese
equipment
God
available
1943
factors
great
1930
1933
types
groups
1934
English
All
V2

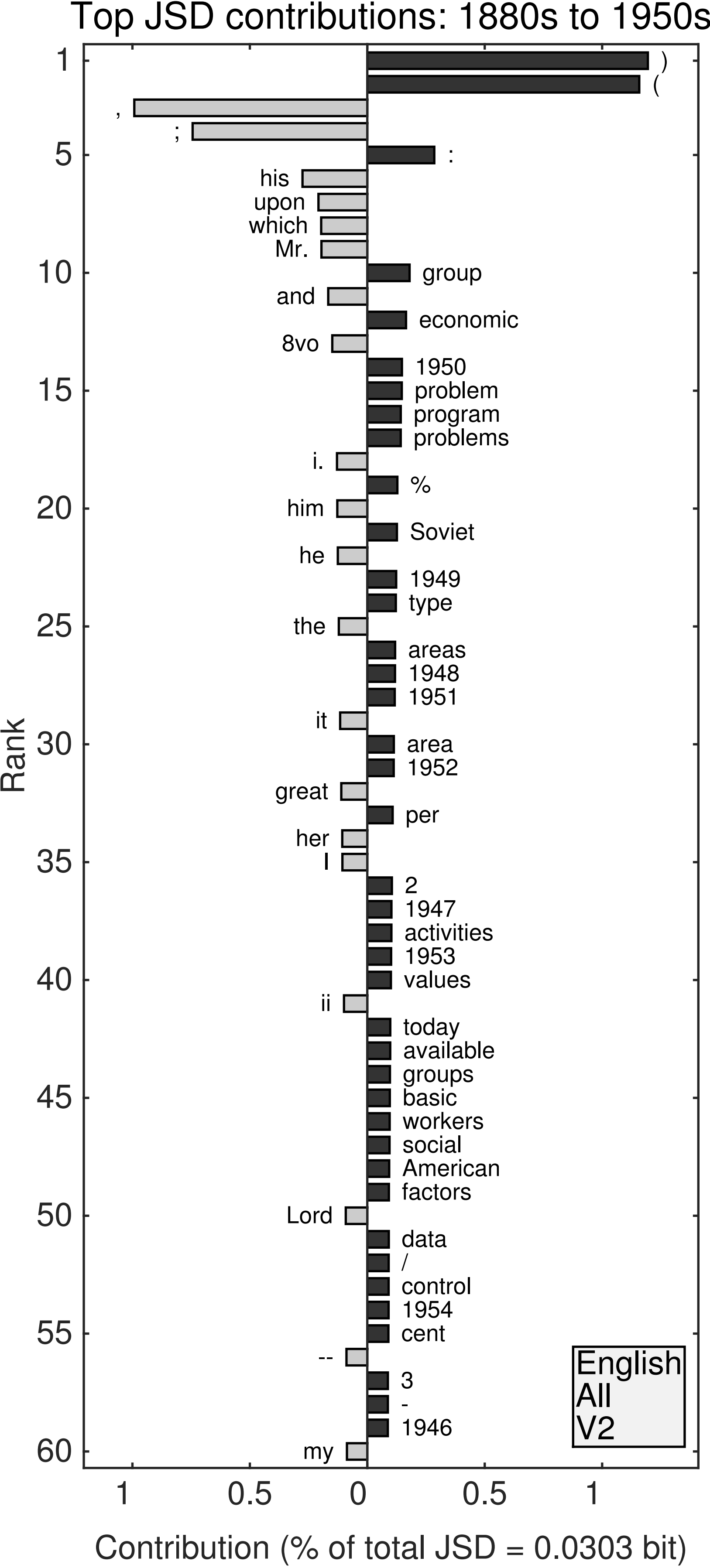

Top JSD contributions: 1880s to 1950s
Rank
)
(
,
;
:
his
upon
which
Mr.
group
and
economic
8vo
1950
problem
program
problems
i.
%
him
Soviet
he
1949
type
the
areas
1948
1951
it
area
1952
great
per
her
I
2
1947
activities
1953
values
ii
today
available
groups
basic
workers
social
American
factors
Lord
data
/
control
1954
cent
--
3
-
1946
my
English
All
V2
Contribution (% of total JSD = 0.0303 bit)

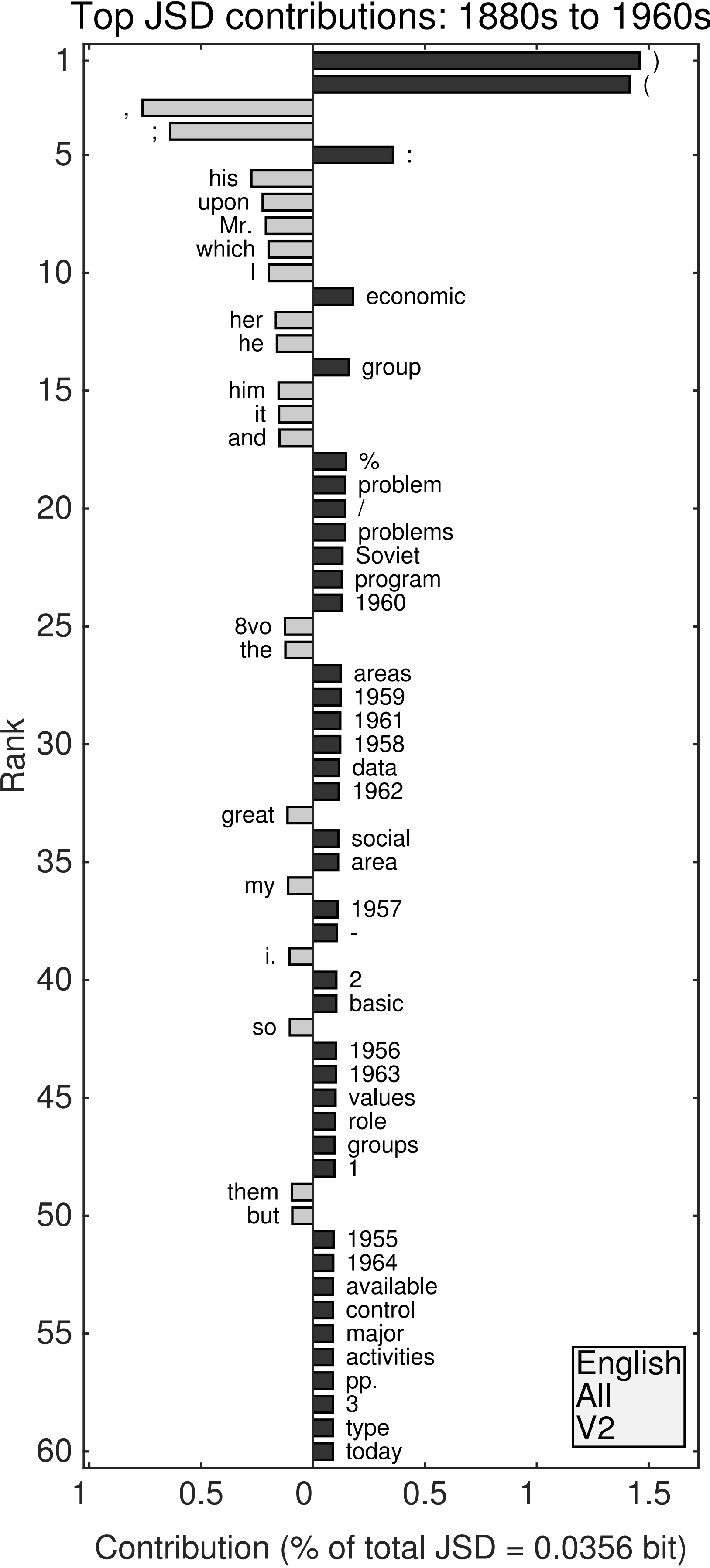
Top JSD contributions: 1880s to 1960s
Rank
Contribution (% of total JSD = 0.0356 bit)
)
(
,
;
:
his
upon
Mr.
which
I
economic
her
he
group
him
it
and
%
problem
/
problems
Soviet
program
1960
8vo
the
areas
1959
1961
1958
data
1962
great
social
area
my
1957
-
i.
2
basic
so
1956
1963
values
role
groups
1
them
but
1955
1964
available
control
major
activities
pp.
3
type
today
English
All
V2

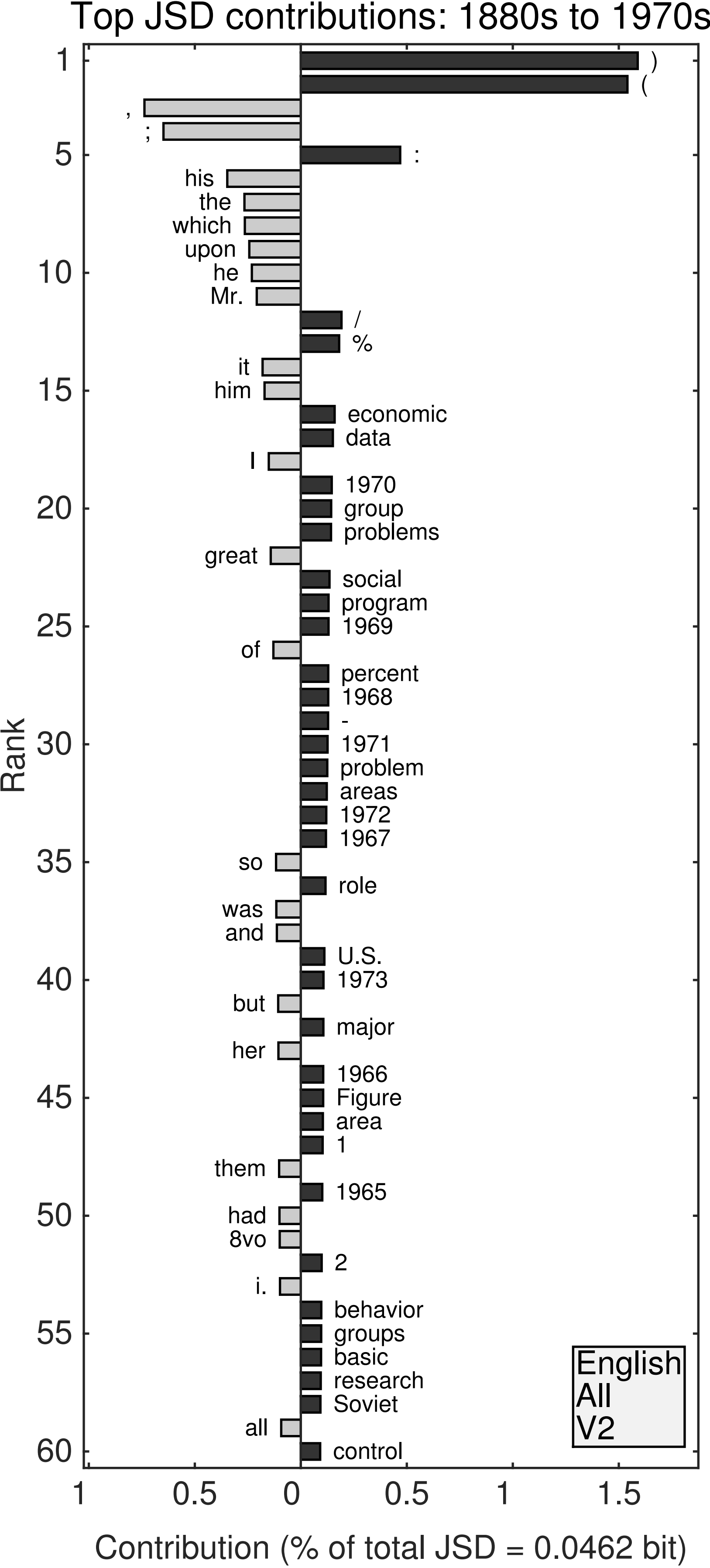

Top JSD contributions: 1880s to 1970s
Rank
1
5
10
15
20
25
30
35
40
45
50
55
60
)
(
,
;
:
his
the
which
upon
he
Mr.
/
%
it
him
economic
data
I
1970
group
problems
great
social
program
1969
of
percent
1968
-
1971
problem
areas
1972
1967
so
role
was
and
U.S.
1973
but
major
her
1966
Figure
area
1
them
1965
had
8vo
2
i.
behavior
groups
basic
research
Soviet
all
control
English
All
V2
1
0.5
0
0.5
1
1.5
Contribution (% of total JSD = 0.0462 bit)

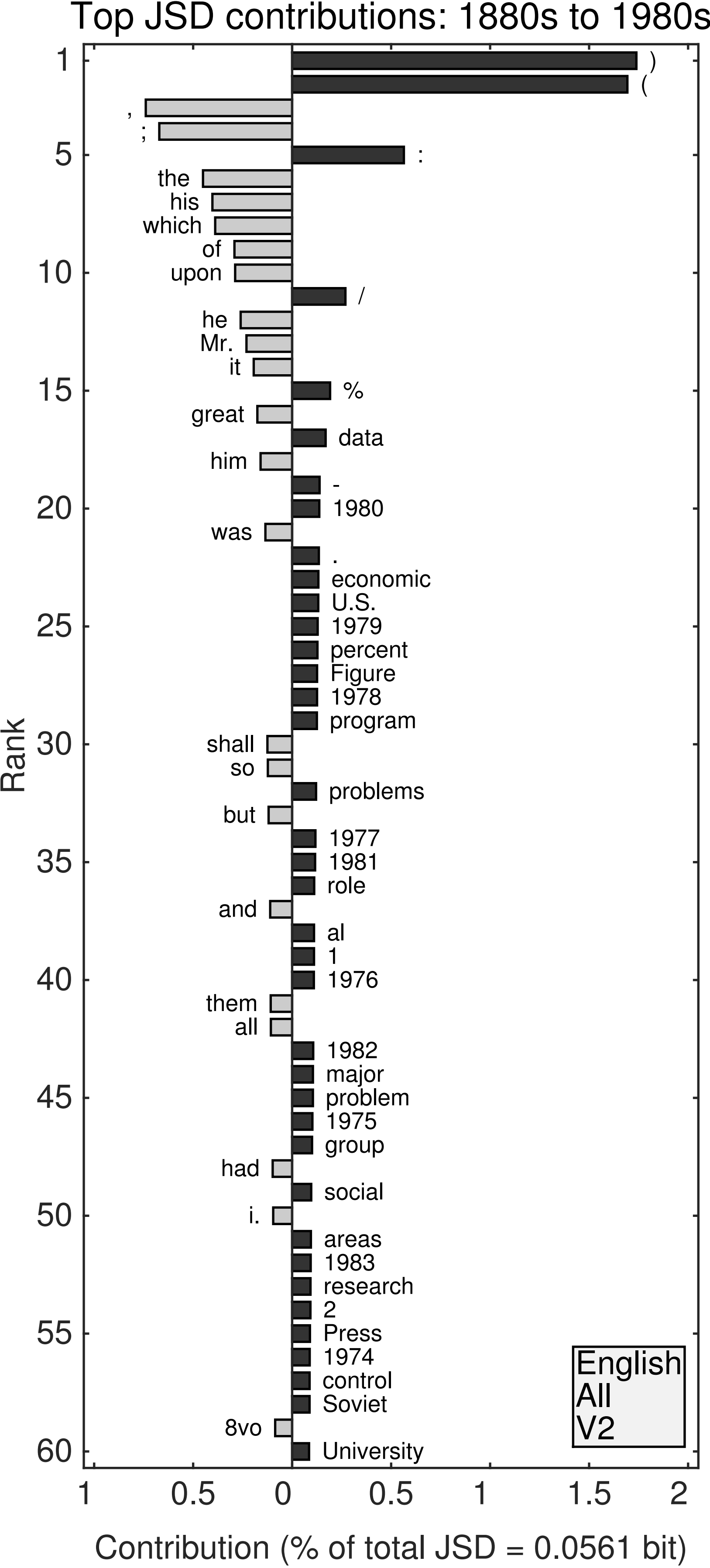

Top JSD contributions: 1880s to 1980s
Rank
Contribution (% of total JSD = 0.0561 bit)
)
(
,
;
:
the
his
which
of
upon
/
he
Mr.
it
%
great
data
him
-
1980
was
.
economic
U.S.
1979
percent
Figure
1978
program
shall
so
problems
but
1977
1981
role
and
al
1
1976
them
all
1982
major
problem
1975
group
had
social
i.
areas
1983
research
2
Press
1974
control
Soviet
8vo
University
English
All
V2

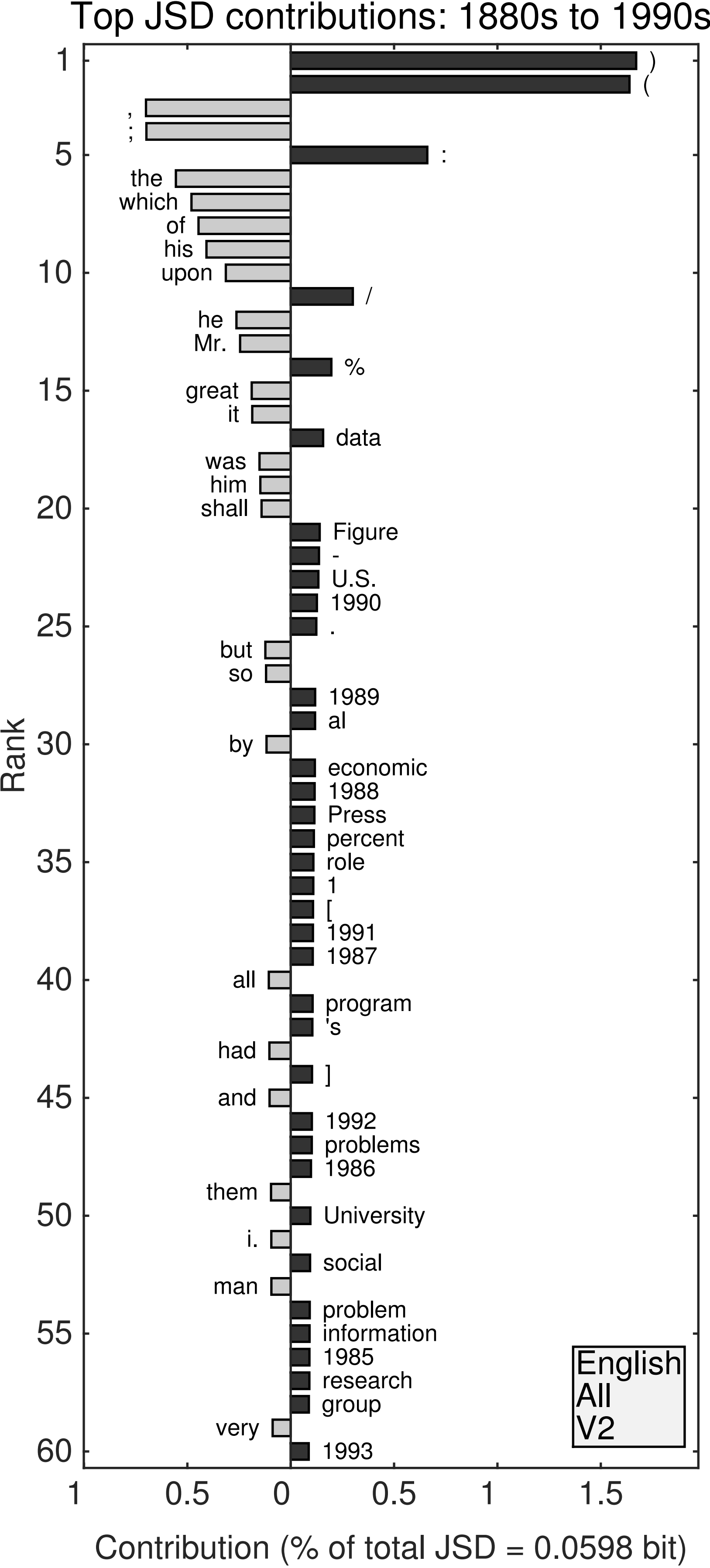

Top JSD contributions: 1880s to 1990s
Rank
Contribution (% of total JSD = 0.0598 bit)
)
(
,
;
:
the
which
of
his
upon
/
he
Mr.
%
great
it
data
was
him
shall
Figure
-
U.S.
1990
.
but
so
1989
al
by
economic
1988
Press
percent
role
1
[
1991
1987
all
program
's
had
]
and
1992
problems
1986
them
University
i.
social
man
problem
information
1985
research
group
very
1993
English
All
V2

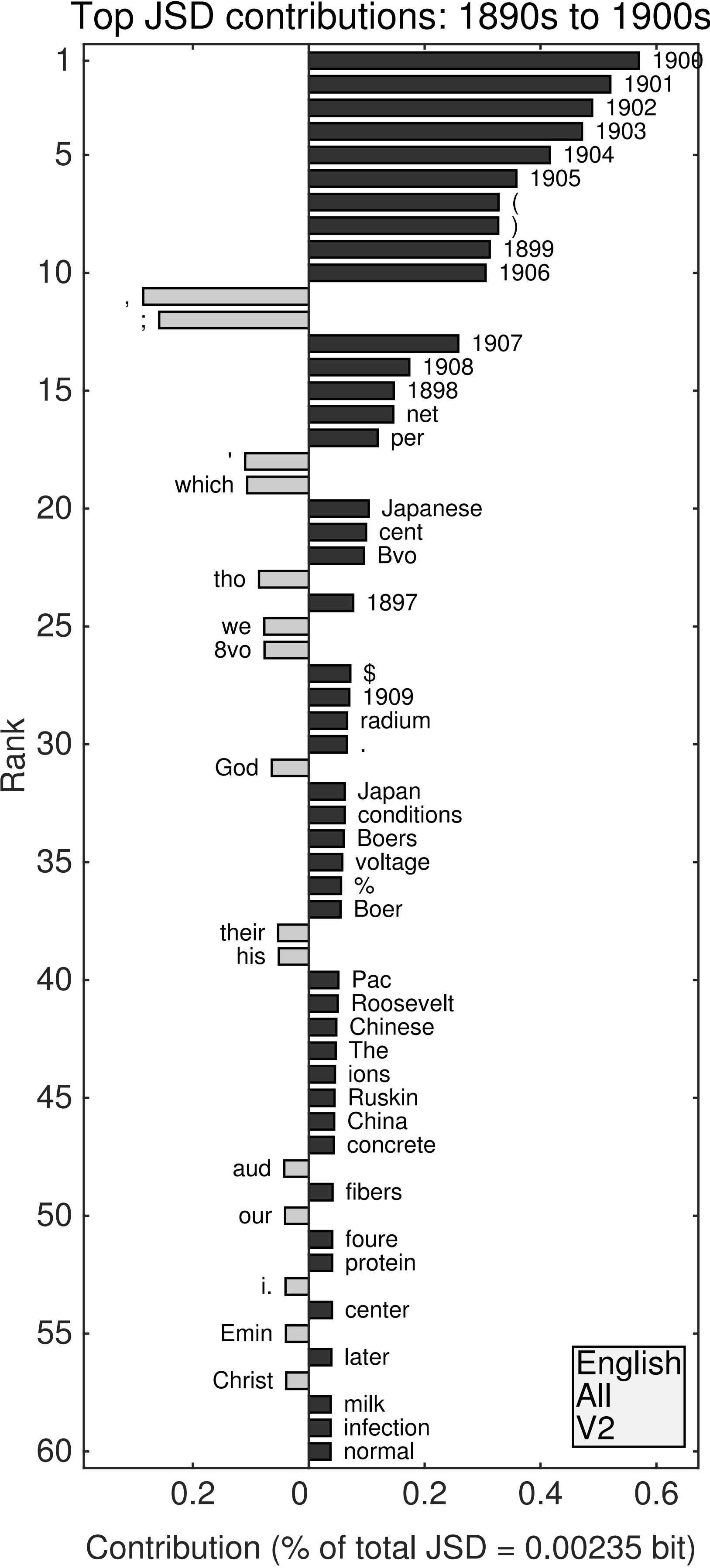

Top JSD contributions: 1890s to 1900s
1900
1901
1902
1903
1904
1905
(
)
1899
1906
,
;
1907
1908
1898
net
per
'
which
Japanese
cent
Bvo
tho
1897
we
8vo
$
1909
radium
.
God
Japan
conditions
Boers
voltage
%
Boer
their
his
Pac
Roosevelt
Chinese
The
ions
Ruskin
China
concrete
aud
fibers
our
foure
protein
i.
center
Emin
later
Christ
milk
infection
normal
English
All
V2
Rank
Contribution (% of total JSD = 0.00235 bit)

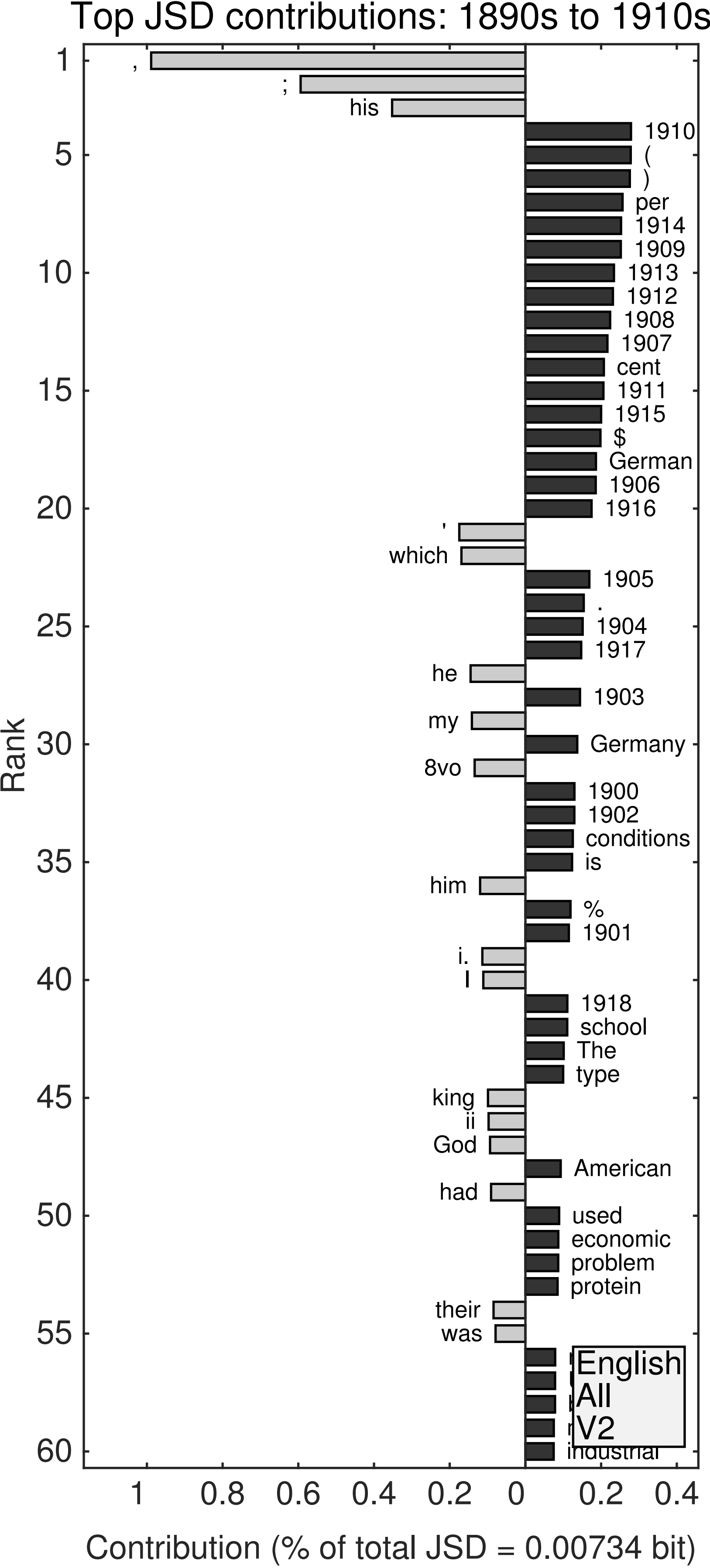
Top JSD contributions: 1890s to 1910s
Rank
1
5
10
15
20
25
30
35
40
45
50
55
60
,
;
his
1910
(
)
per
1914
1909
1913
1912
1908
1907
cent
1911
1915
$
German
1906
1916
'
which
1905
.
1904
1917
he
1903
my
Germany
8vo
1900
1902
conditions
is
him
%
1901
i.
I
1918
school
The
type
king
ii
God
American
had
used
economic
problem
protein
their
was
industrial
English
All
V2
1
0.8
0.6
0.4
0.2
0
0.2
0.4
Contribution (% of total JSD = 0.00734 bit)

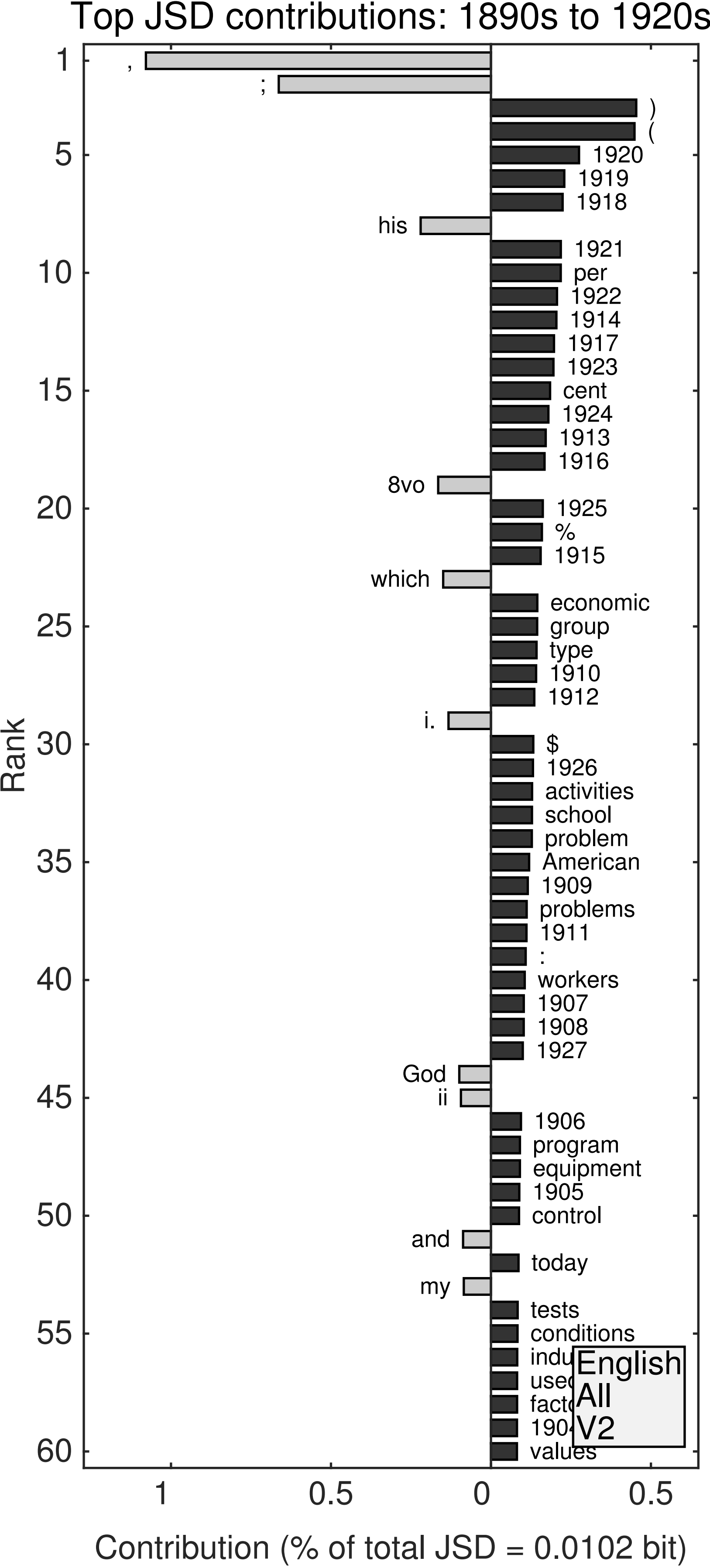
Top JSD contributions: 1890s to 1920s
Rank
Contribution (% of total JSD = 0.0102 bit)
1
5
10
15
20
25
30
35
40
45
50
55
60
1
0.5
0
0.5
,
;
)
(
1920
1919
1918
his
1921
per
1922
1914
1917
1923
cent
1924
1913
1916
8vo
1925
%
1915
which
economic
group
type
1910
1912
i.
$
1926
activities
school
problem
American
1909
problems
1911
:
workers
1907
1908
1927
God
ii
1906
program
equipment
1905
control
and
today
my
tests
conditions
values
English
All
V2

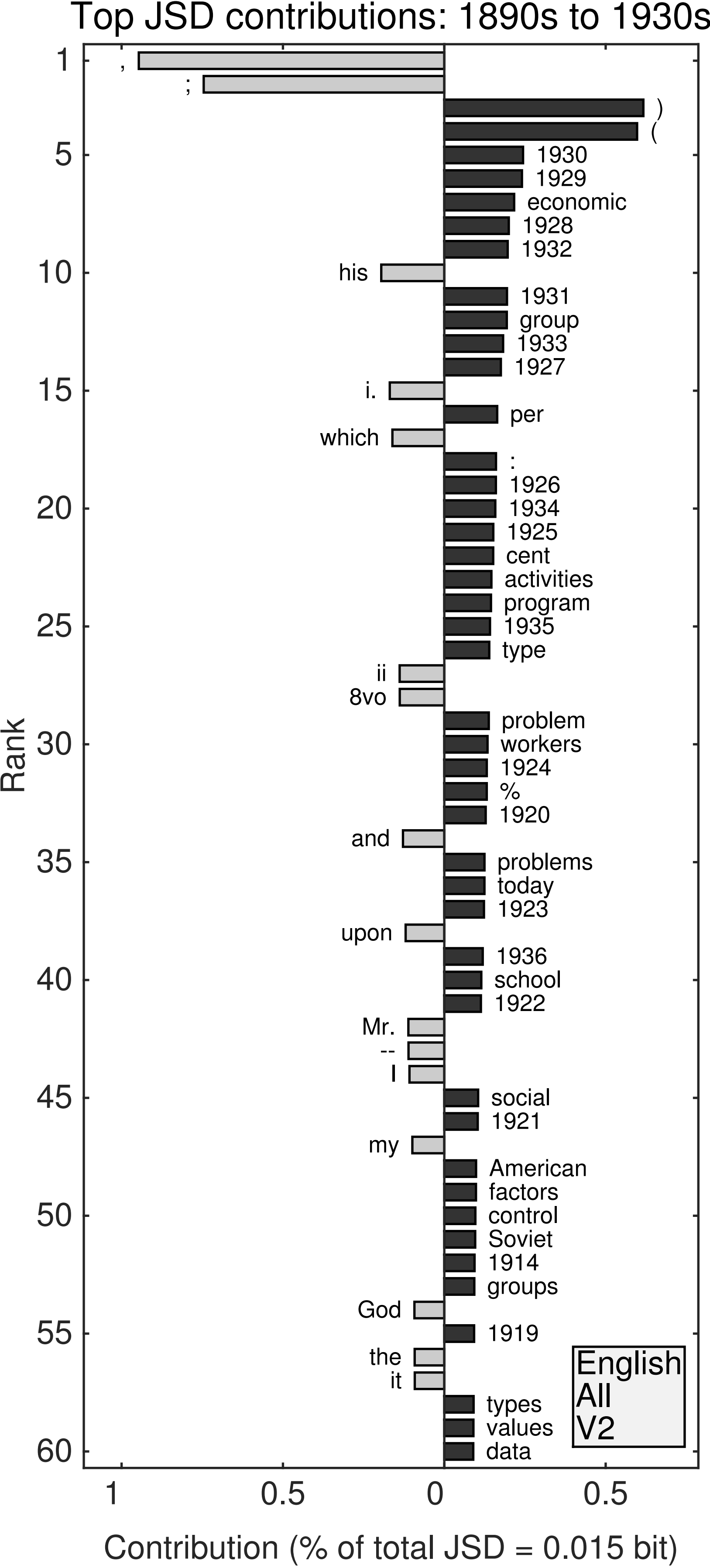
Top JSD contributions: 1890s to 1930s
Rank
Contribution (% of total JSD = 0.015 bit)
1
5
10
15
20
25
30
35
40
45
50
55
60
1
0.5
0
0.5
,
;
)
(
1930
1929
economic
1928
1932
his
1931
group
1933
1927
i.
per
which
:
1926
1934
1925
cent
activities
program
1935
type
ii
8vo
problem
workers
1924
%
1920
and
problems
today
1923
upon
1936
school
1922
Mr.
--
I
social
1921
my
American
factors
control
Soviet
1914
groups
God
1919
the
it
types
values
data
English
All
V2

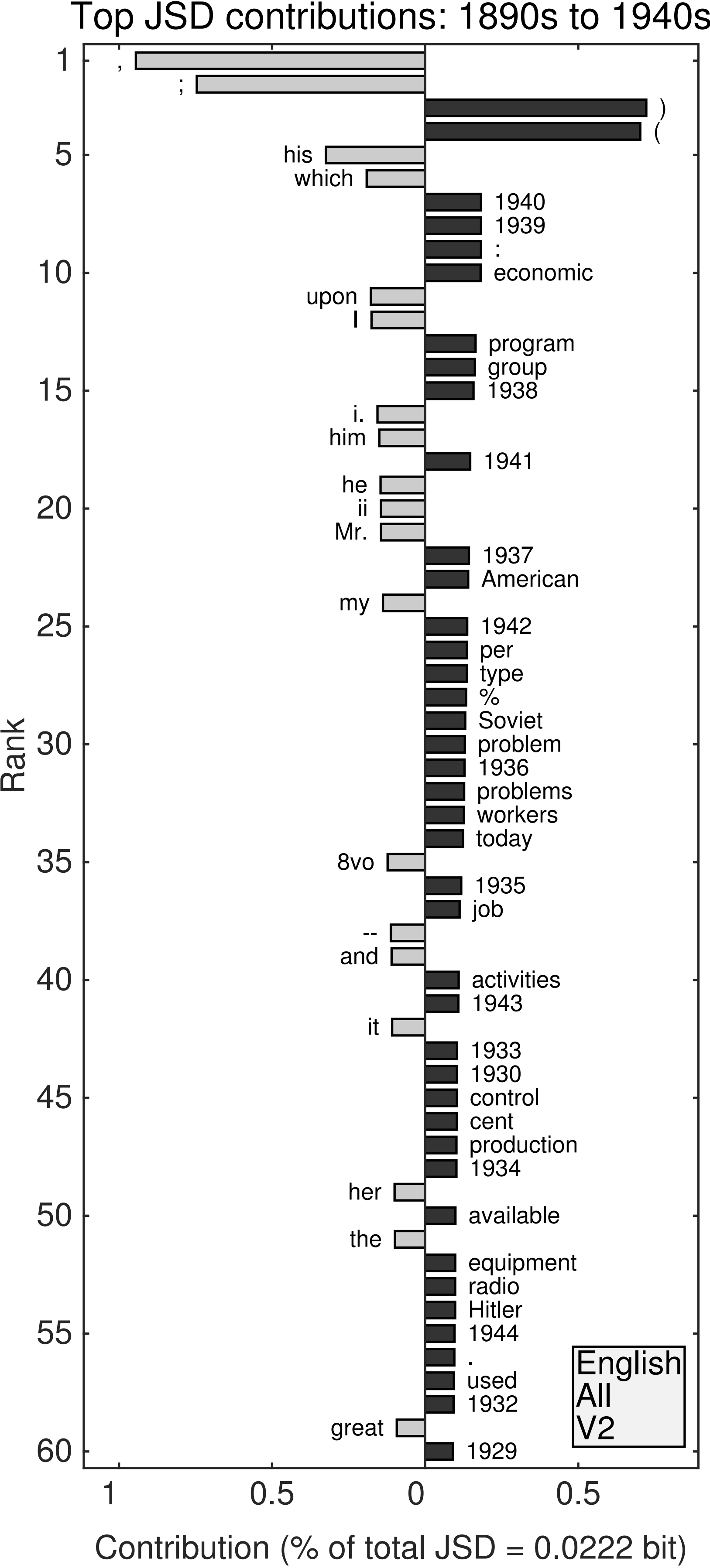

Top JSD contributions: 1890s to 1940s
Rank
1
5
10
15
20
25
30
35
40
45
50
55
60
,
;
)
(
his
which
1940
1939
:
economic
upon
I
program
group
1938
i.
him
1941
he
ii
Mr.
1937
American
my
1942
per
type
%
Soviet
problem
1936
problems
workers
today
8vo
1935
job
--
and
activities
1943
it
1933
1930
control
cent
production
1934
her
available
the
equipment
radio
Hitler
1944
.
used
1932
great
1929
English
All
V2
1
0.5
0
0.5
Contribution (% of total JSD = 0.0222 bit)

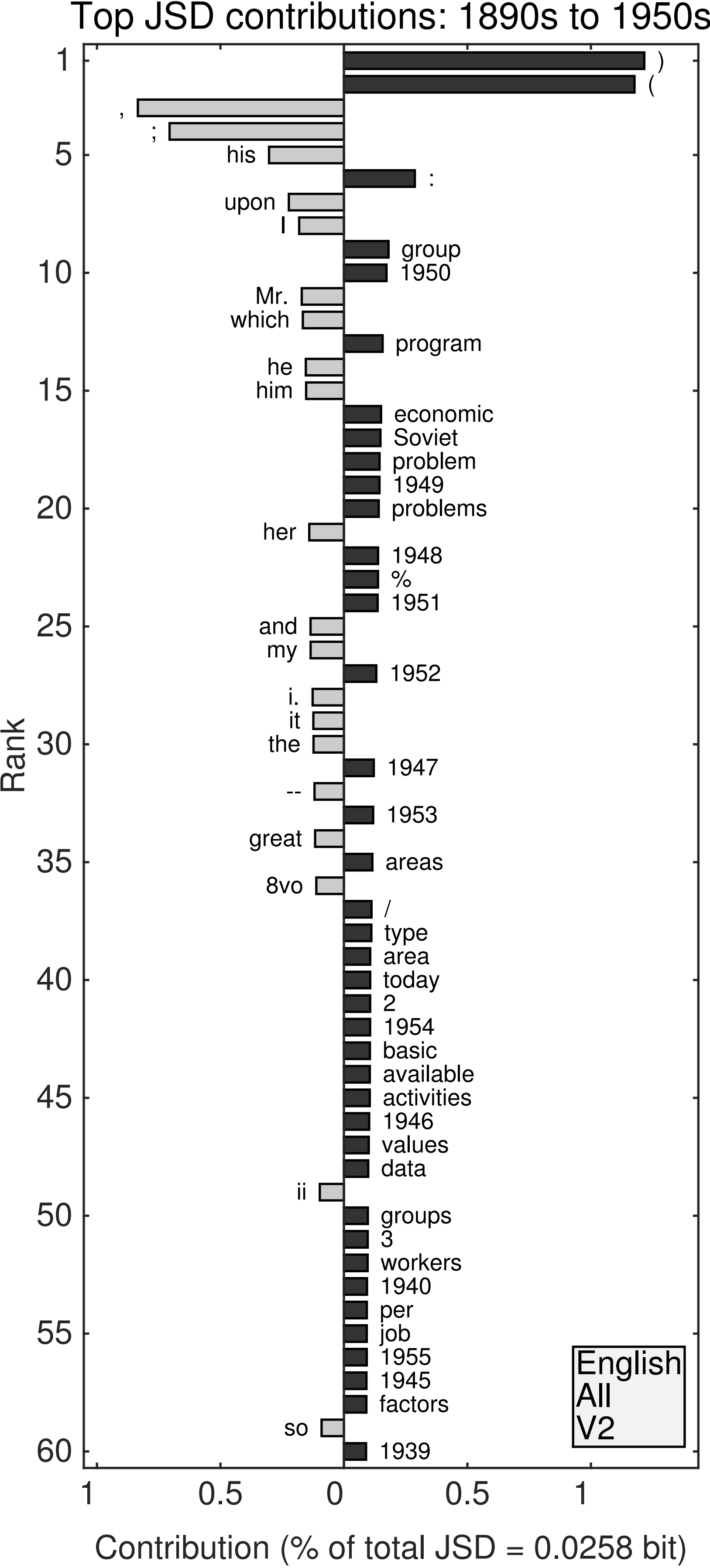
Top JSD contributions: 1890s to 1950s
Rank
Contribution (% of total JSD = 0.0258 bit)
)
(
,
;
his
:
upon
I
group
1950
Mr.
which
program
he
him
economic
Soviet
problem
1949
problems
her
1948
%
1951
and
my
1952
i.
it
the
1947
--
1953
great
areas
8vo
/
type
area
today
2
1954
basic
available
activities
1946
values
data
ii
groups
3
workers
1940
per
job
1955
1945
factors
so
1939
English
All
V2

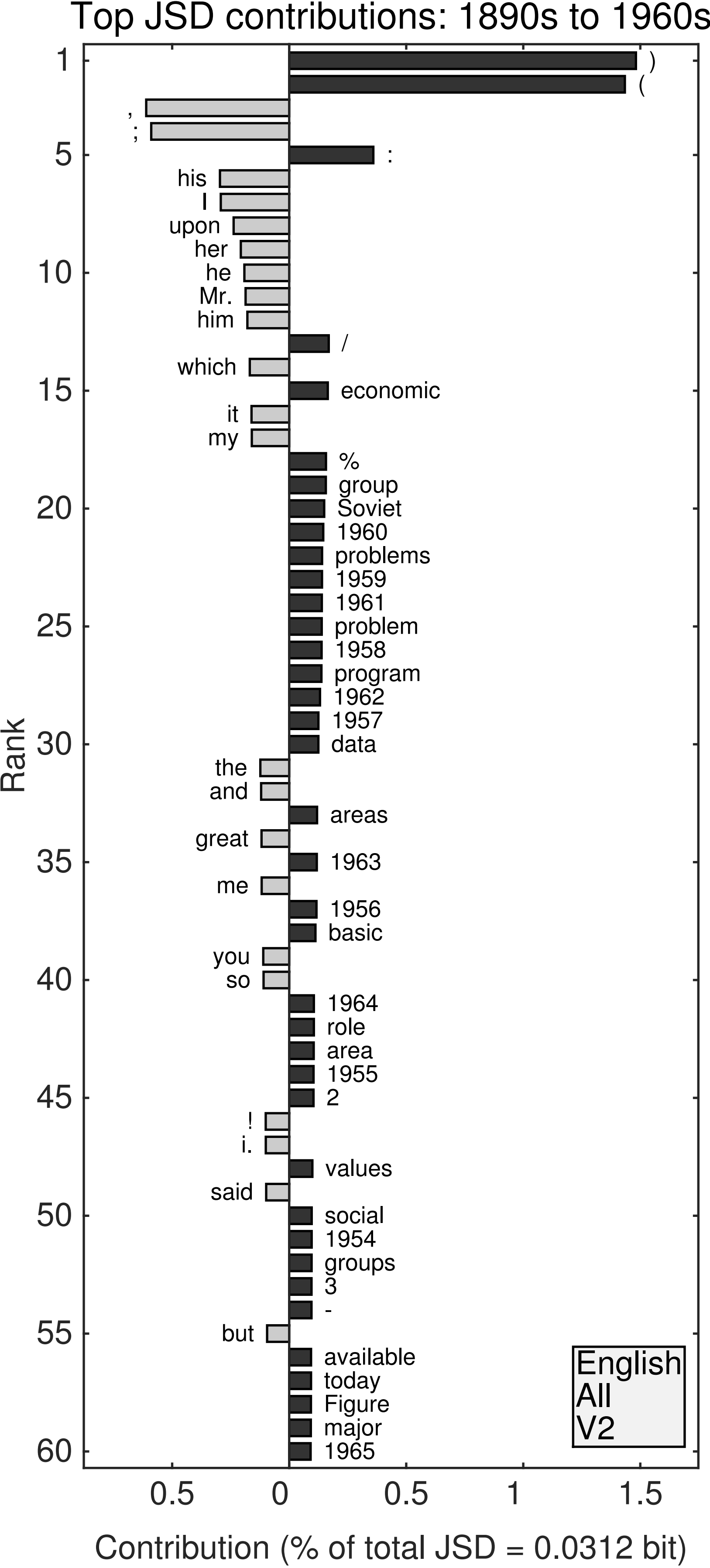
Top JSD contributions: 1890s to 1960s
Rank
Contribution (% of total JSD = 0.0312 bit)
)
(
,
;
:
his
I
upon
her
he
Mr.
him
/
which
economic
it
my
%
group
Soviet
1960
problems
1959
1961
problem
1958
program
1962
1957
data
the
and
areas
great
1963
me
1956
basic
you
so
1964
role
area
1955
2
!
i.
values
said
social
1954
groups
3
-
but
available
today
Figure
major
1965
English
All
V2

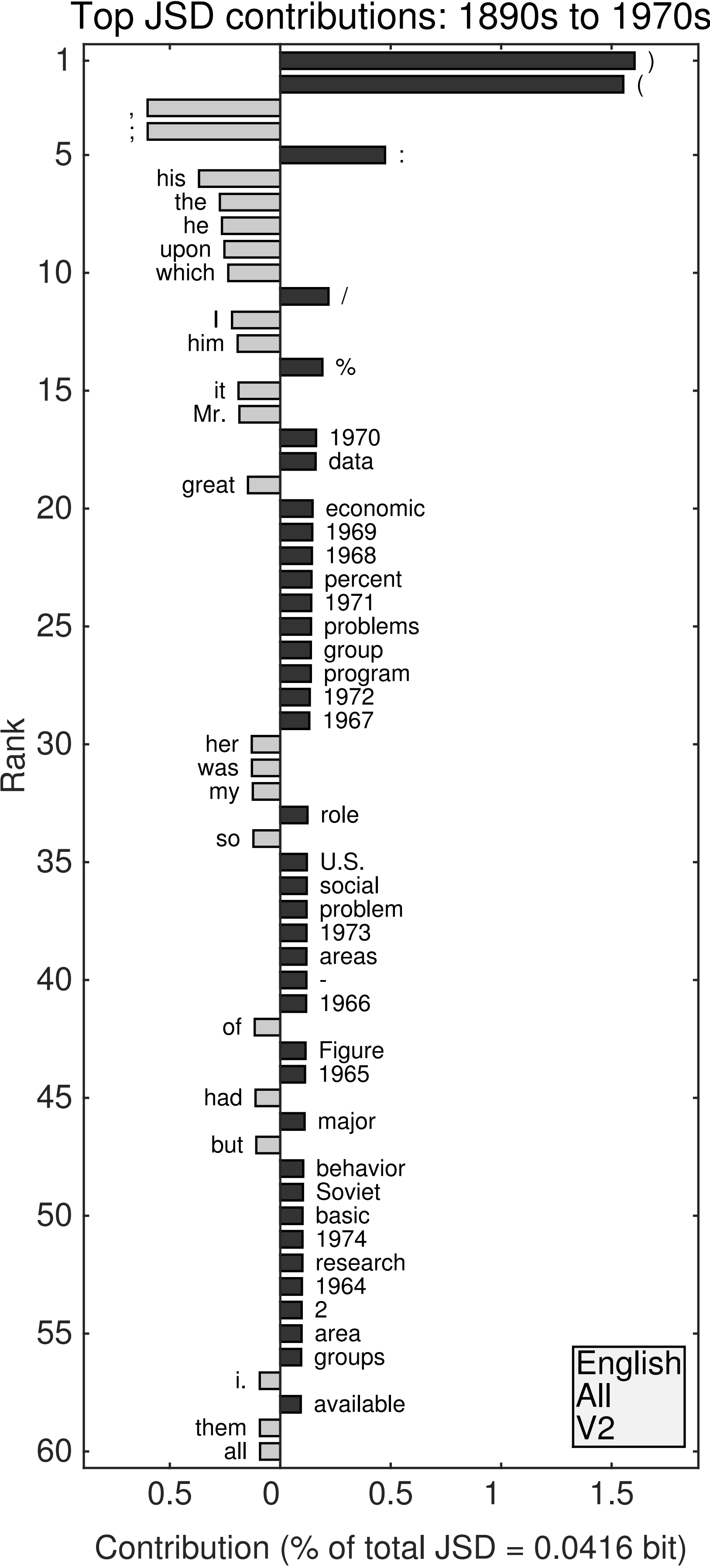

Top JSD contributions: 1890s to 1970s
Rank
Contribution (% of total JSD = 0.0416 bit)
English
All
V2
)
(
,
;
:
his
the
he
upon
which
/
I
him
%
it
Mr.
1970
data
great
economic
1969
1968
percent
1971
problems
group
program
1972
1967
her
was
my
role
so
U.S.
social
problem
1973
areas
-
1966
of
Figure
1965
had
major
but
behavior
Soviet
basic
1974
research
1964
2
area
groups
i.
available
them
all

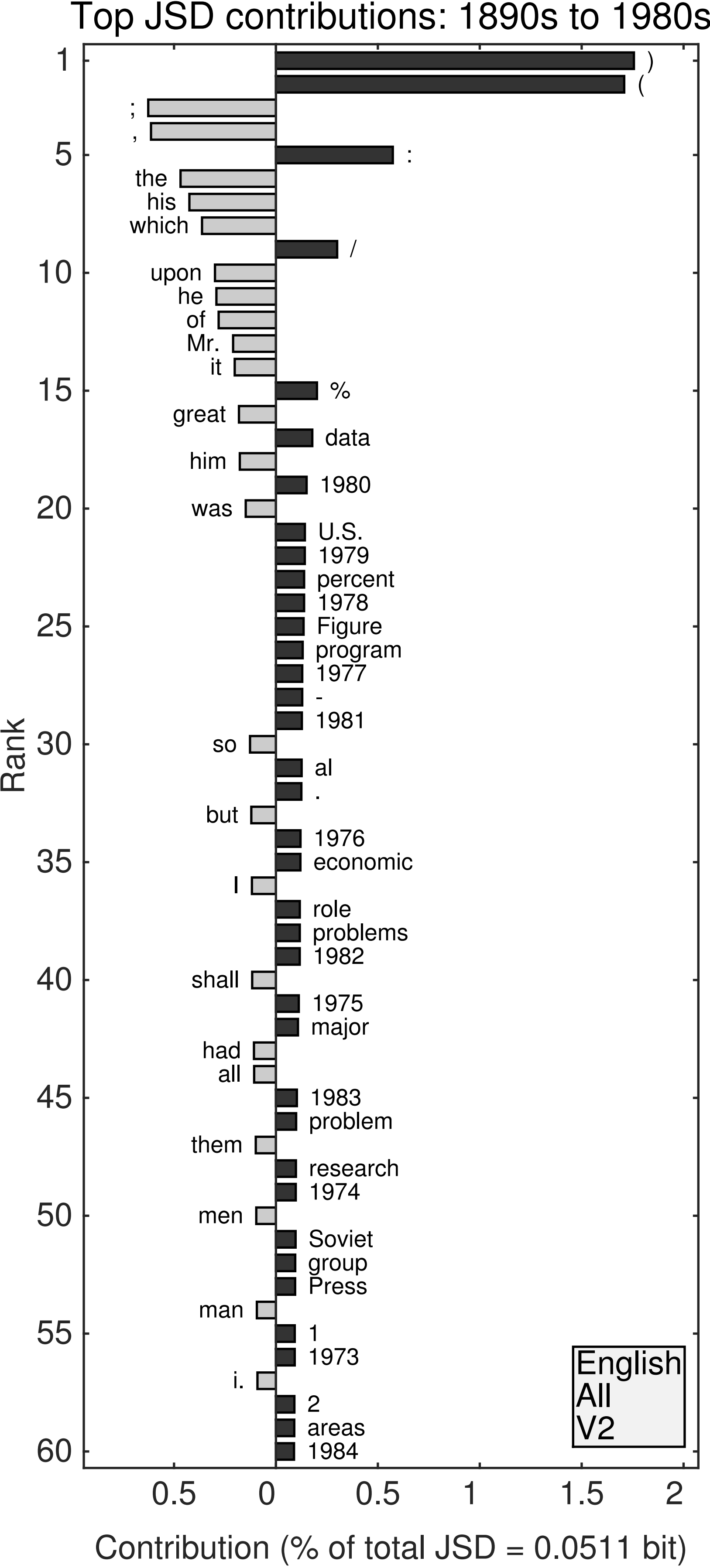

Top JSD contributions: 1890s to 1980s
Rank
Contribution (% of total JSD = 0.0511 bit)
)
(
;
,
:
the
his
which
/
upon
he
of
Mr.
it
%
great
data
him
1980
was
U.S.
1979
percent
1978
Figure
program
1977
-
1981
so
al
.
but
1976
economic
I
role
problems
1982
shall
1975
major
had
all
1983
problem
them
research
1974
men
Soviet
group
Press
man
1
1973
i.
2
areas
1984
English
All
V2

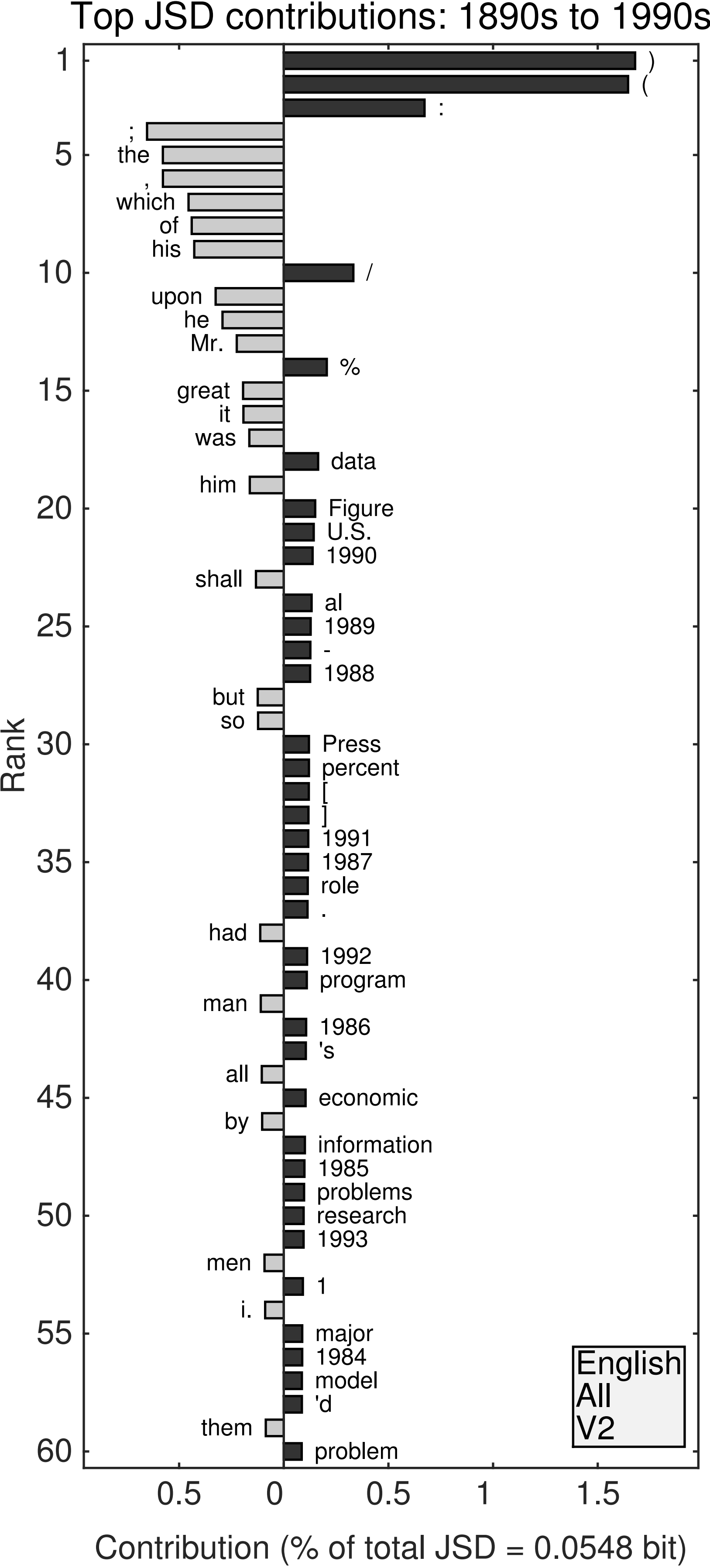

Top JSD contributions: 1890s to 1990s
Rank
Contribution (% of total JSD = 0.0548 bit)
)
(
:
;
the
,
which
of
his
/
upon
he
Mr.
%
great
it
was
data
him
Figure
U.S.
1990
shall
al
1989
-
1988
but
so
Press
percent
[
]
1991
1987
role
.
had
1992
program
man
1986
's
all
economic
by
information
1985
problems
research
1993
men
1
i.
major
1984
model
'd
them
problem
English
All
V2

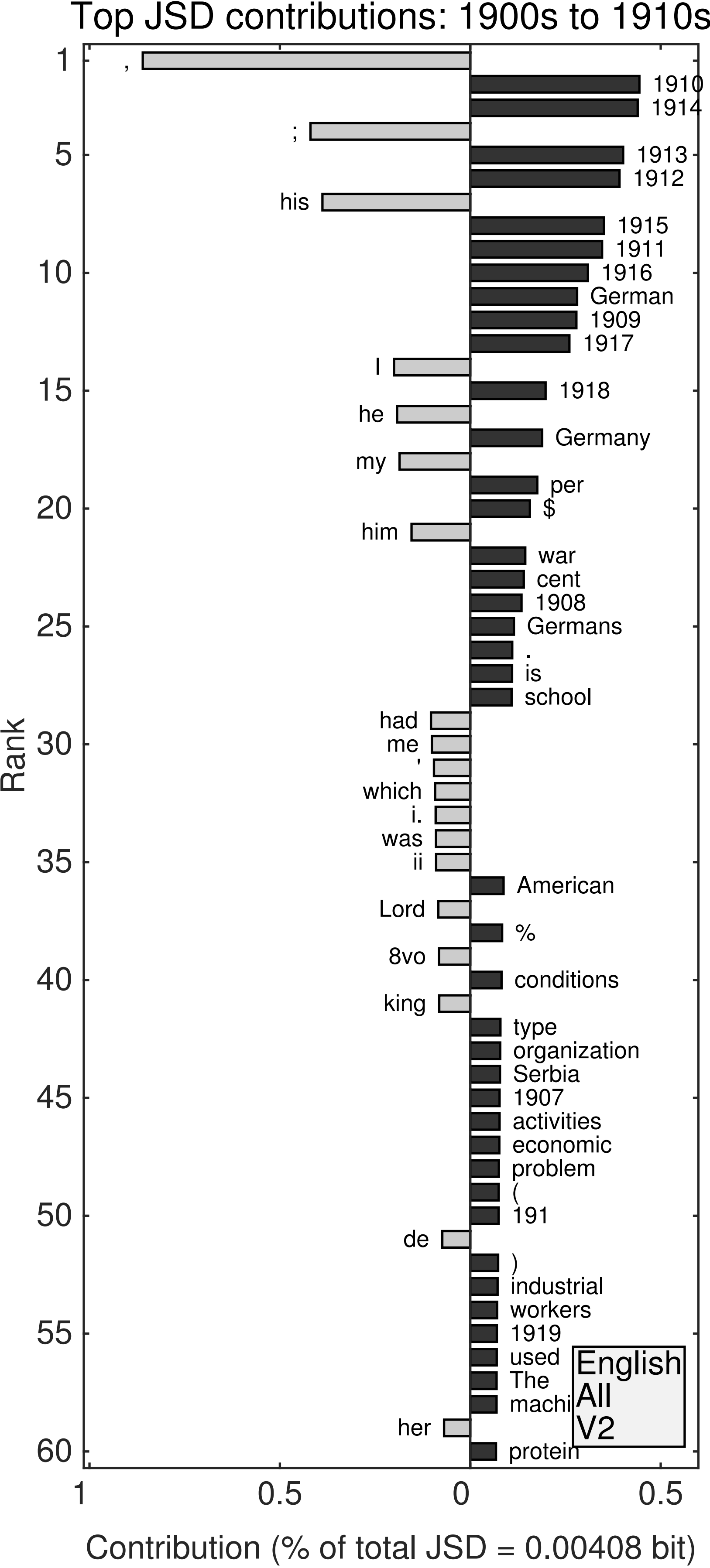

Top JSD contributions: 1900s to 1910s
Rank
Contribution (% of total JSD = 0.00408 bit)
1
5
10
15
20
25
30
35
40
45
50
55
60
1
0.5
0
0.5
,
1910
1914
;
1913
1912
his
1915
1911
1916
German
1909
1917
I
1918
he
Germany
my
per
$
him
war
cent
1908
Germans
.
is
school
had
me
'
which
i.
was
ii
American
Lord
%
8vo
conditions
king
type
organization
Serbia
1907
activities
economic
problem
(
191
de
)
industrial
workers
1919
used
The
machi
her
protein
English
All
V2

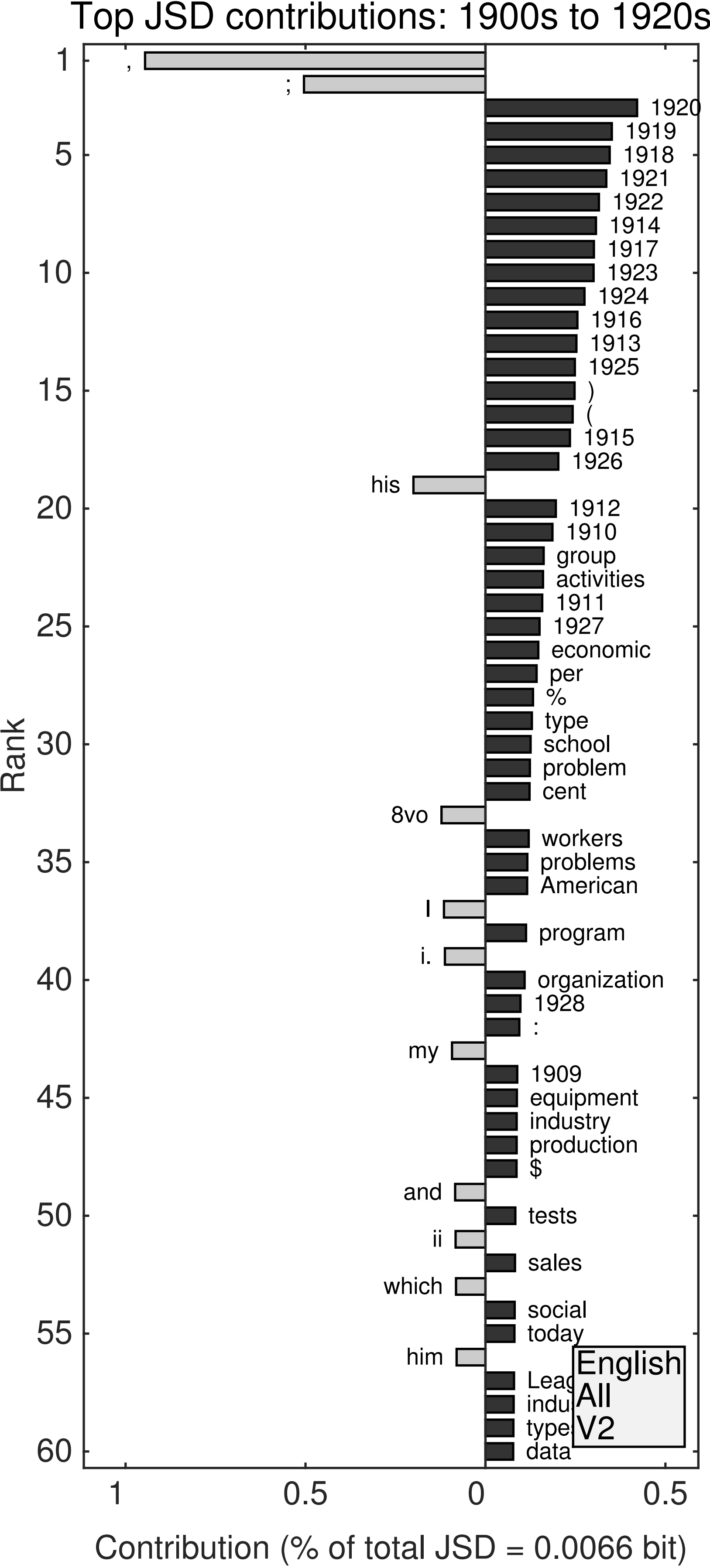

Top JSD contributions: 1900s to 1920s
Rank
Contribution (% of total JSD = 0.0066 bit)
English
All
V2
,
;
1920
1919
1918
1921
1922
1914
1917
1923
1924
1916
1913
1925
)
(
1915
1926
his
1912
1910
group
activities
1911
1927
economic
per
%
type
school
problem
cent
8vo
workers
problems
American
I
program
i.
organization
1928
:
my
1909
equipment
industry
production
$
and
tests
ii
sales
which
social
today
him
data

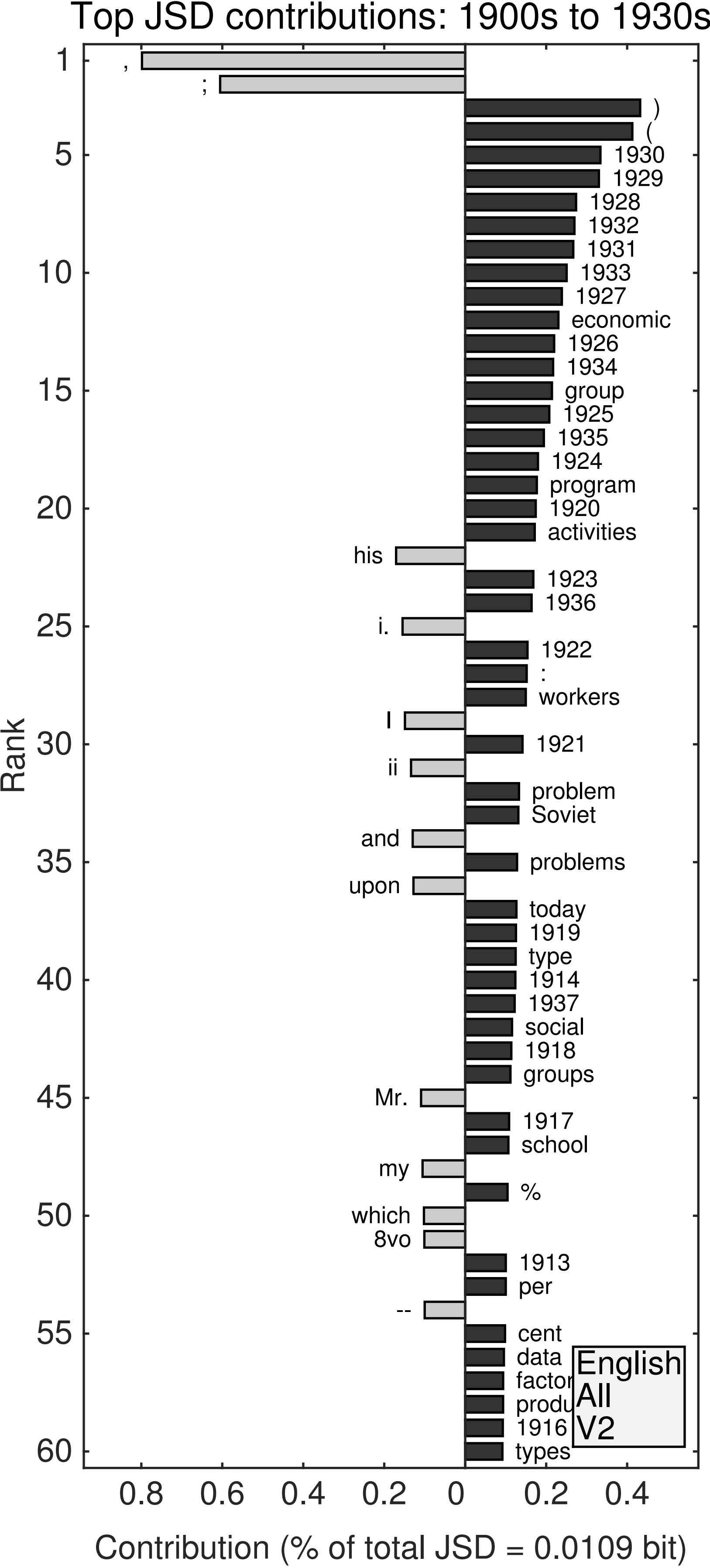
Top JSD contributions: 1900s to 1930s
Rank
Contribution (% of total JSD = 0.0109 bit)
0.8
0.6
0.4
0.2
0
0.2
0.4
1
5
10
15
20
25
30
35
40
45
50
55
60
,
;
)
(
1930
1929
1928
1932
1931
1933
1927
economic
1926
1934
group
1925
1935
1924
program
1920
activities
his
1923
1936
i.
1922
:
workers
I
1921
ii
problem
Soviet
and
problems
upon
today
1919
type
1914
1937
social
1918
groups
Mr.
1917
school
my
%
which
8vo
1913
per
--
cent
data
1916
types
English
All
V2

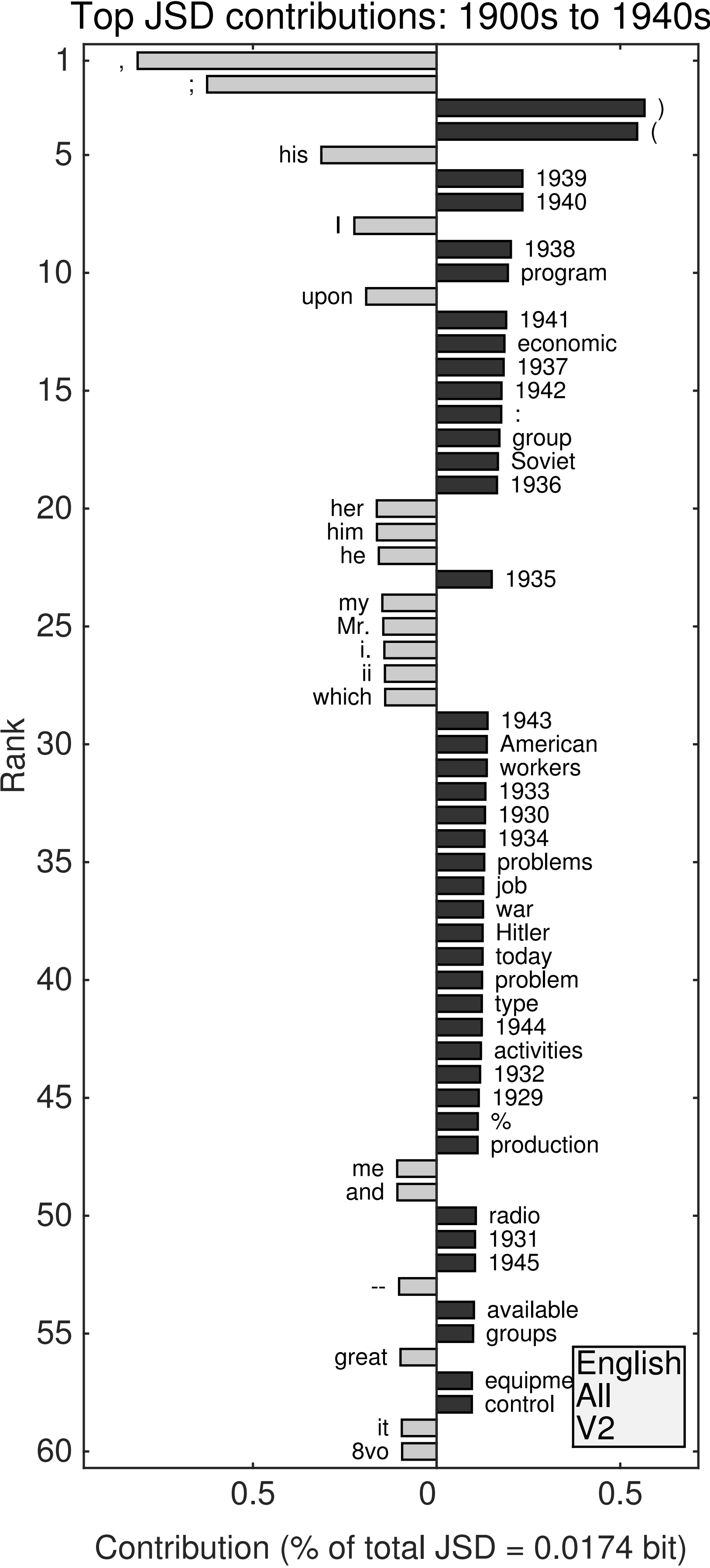
Top JSD contributions: 1900s to 1940s
Rank
Contribution (% of total JSD = 0.0174 bit)
English
All
V2
,
;
)
(
his
1939
1940
I
1938
program
upon
1941
economic
1937
1942
:
group
Soviet
1936
her
him
he
1935
my
Mr.
i.
ii
which
1943
American
workers
1933
1930
1934
problems
job
war
Hitler
today
problem
type
1944
activities
1932
1929
%
production
me
and
radio
1931
1945
--
available
groups
great
equipme
control
it
8vo

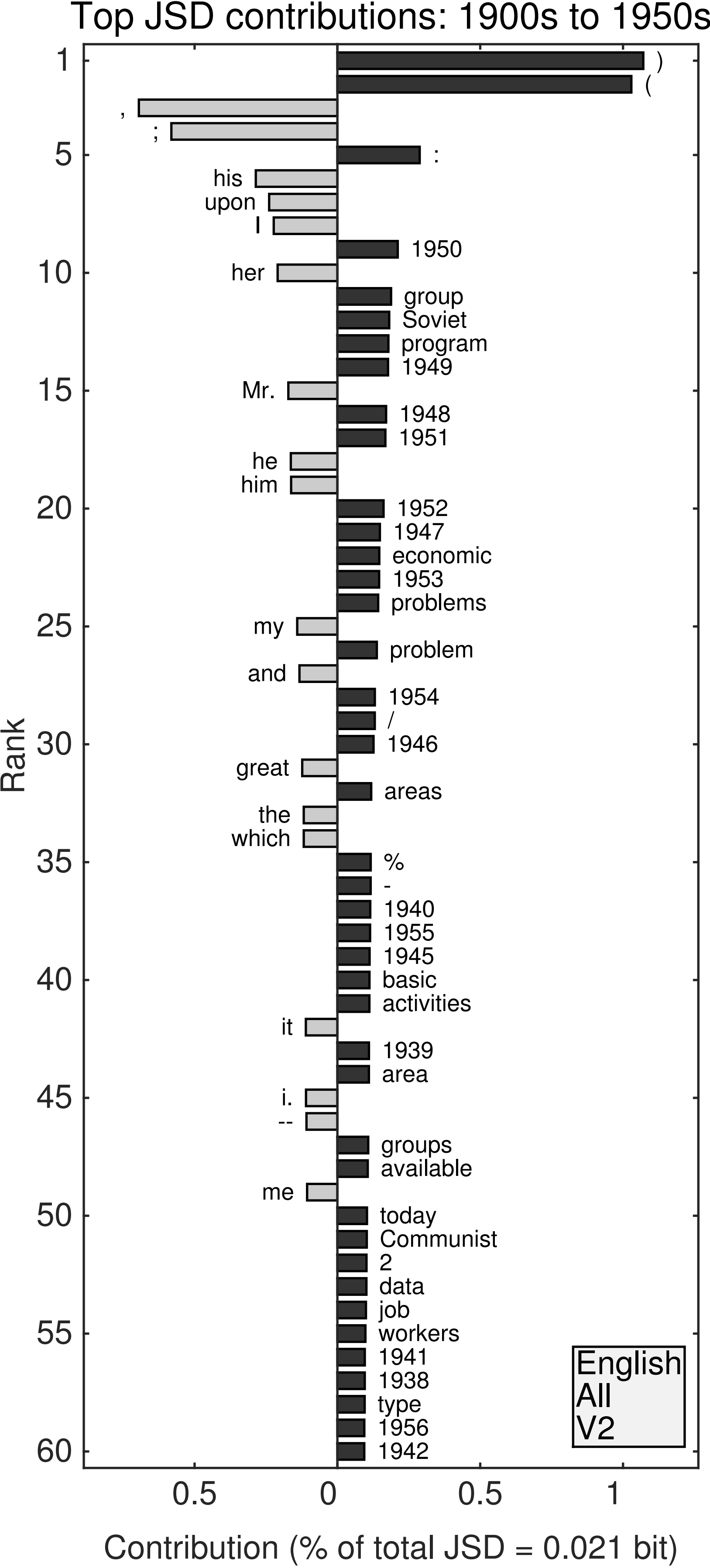
Top JSD contributions: 1900s to 1950s
Rank
Contribution (% of total JSD = 0.021 bit)
)
(
,
;
:
his
upon
I
1950
her
group
Soviet
program
1949
Mr.
1948
1951
he
him
1952
1947
economic
1953
problems
my
problem
and
1954
/
1946
great
areas
the
which
%
-
1940
1955
1945
basic
activities
it
1939
area
i.
--
groups
available
me
today
Communist
2
data
job
workers
1941
1938
type
1956
1942
English
All
V2

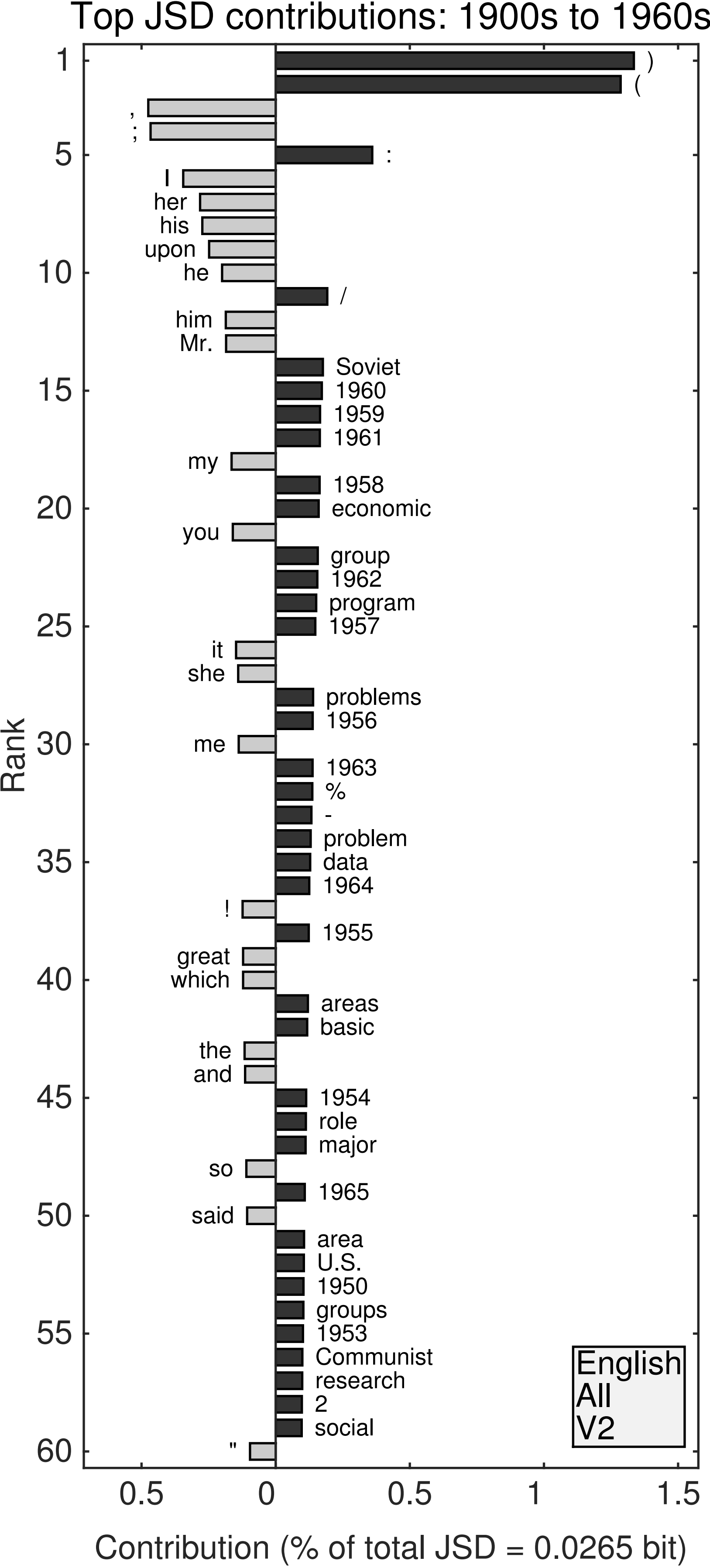
Top JSD contributions: 1900s to 1960s
Rank
)
(
,
;
:
I
her
his
upon
he
/
him
Mr.
Soviet
1960
1959
1961
my
1958
economic
you
group
1962
program
1957
it
she
problems
1956
me
1963
%
-
problem
data
1964
!
1955
great
which
areas
basic
the
and
1954
role
major
so
1965
said
area
U.S.
1950
groups
1953
Communist
research
2
social
"
English
All
V2
Contribution (% of total JSD = 0.0265 bit)

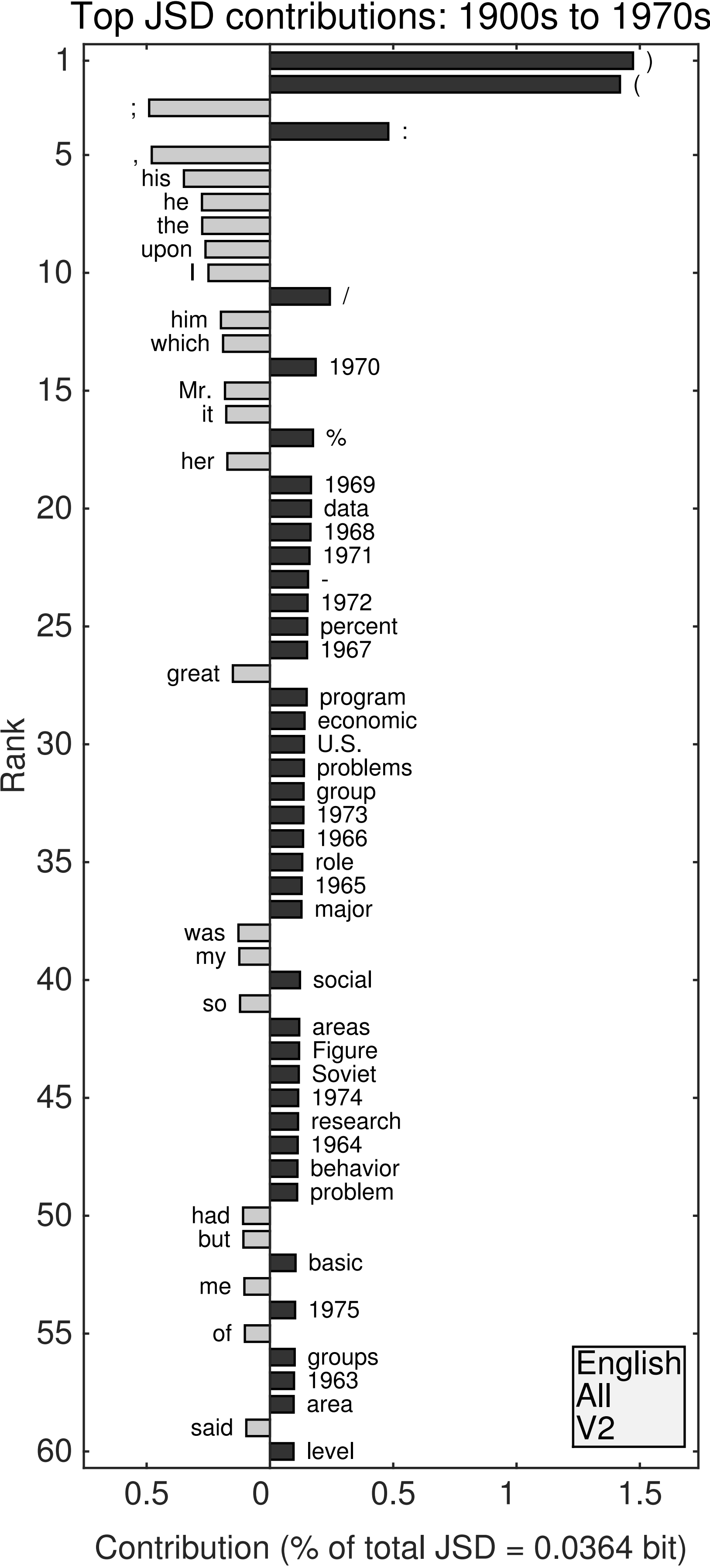

Top JSD contributions: 1900s to 1970s
Rank
)
(
;
:
,
his
he
the
upon
I
/
him
which
1970
Mr.
it
%
her
1969
data
1968
1971
-
1972
percent
1967
great
program
economic
U.S.
problems
group
1973
1966
role
1965
major
was
my
social
so
areas
Figure
Soviet
1974
research
1964
behavior
problem
had
but
basic
me
1975
of
groups
1963
area
said
level
English
All
V2
Contribution (% of total JSD = 0.0364 bit)

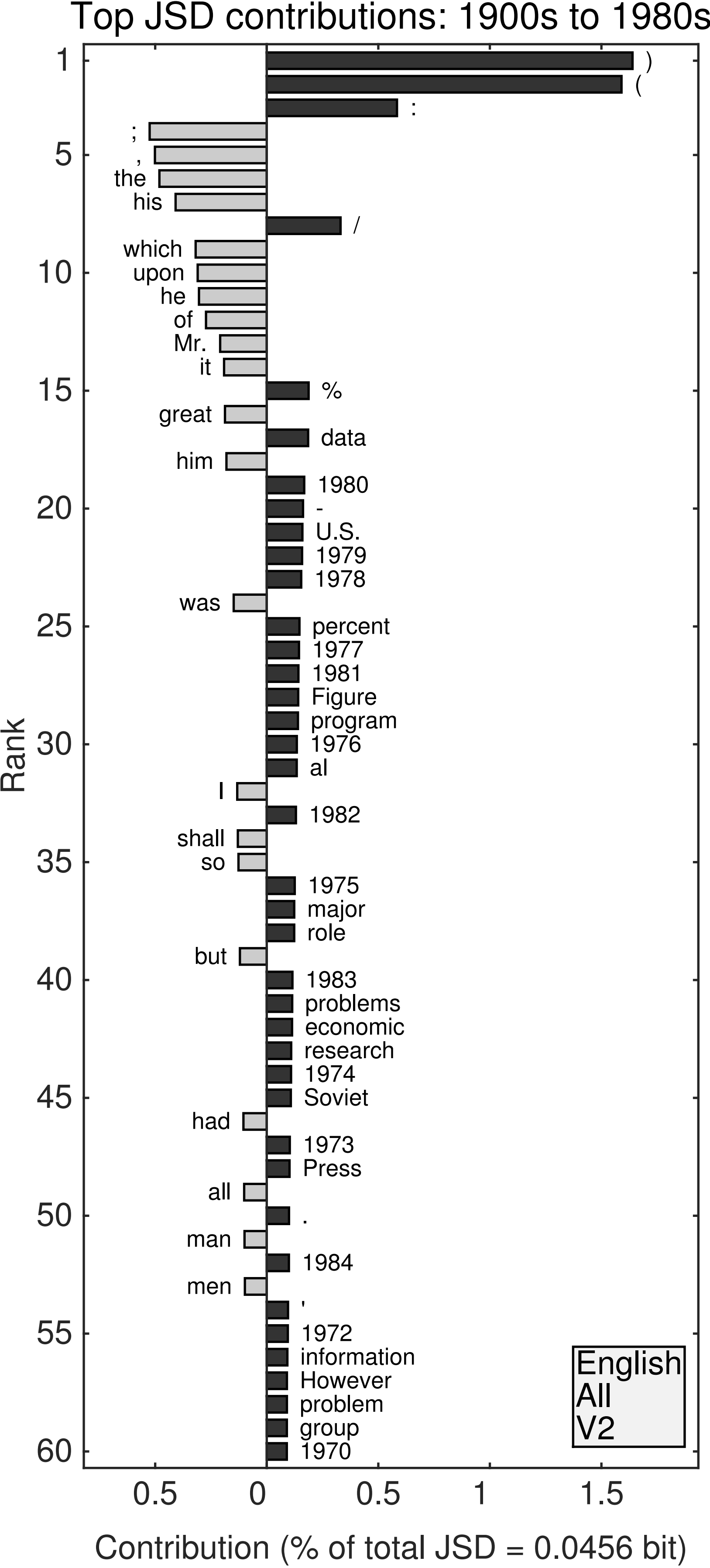

Top JSD contributions: 1900s to 1980s
Rank
Contribution (% of total JSD = 0.0456 bit)
)
(
:
;
,
the
his
/
which
upon
he
of
Mr.
it
%
great
data
him
1980
-
U.S.
1979
1978
was
percent
1977
1981
Figure
program
1976
al
I
1982
shall
so
1975
major
role
but
1983
problems
economic
research
1974
Soviet
had
1973
Press
all
.
man
1984
men
'
1972
information
However
problem
group
1970
English
All
V2

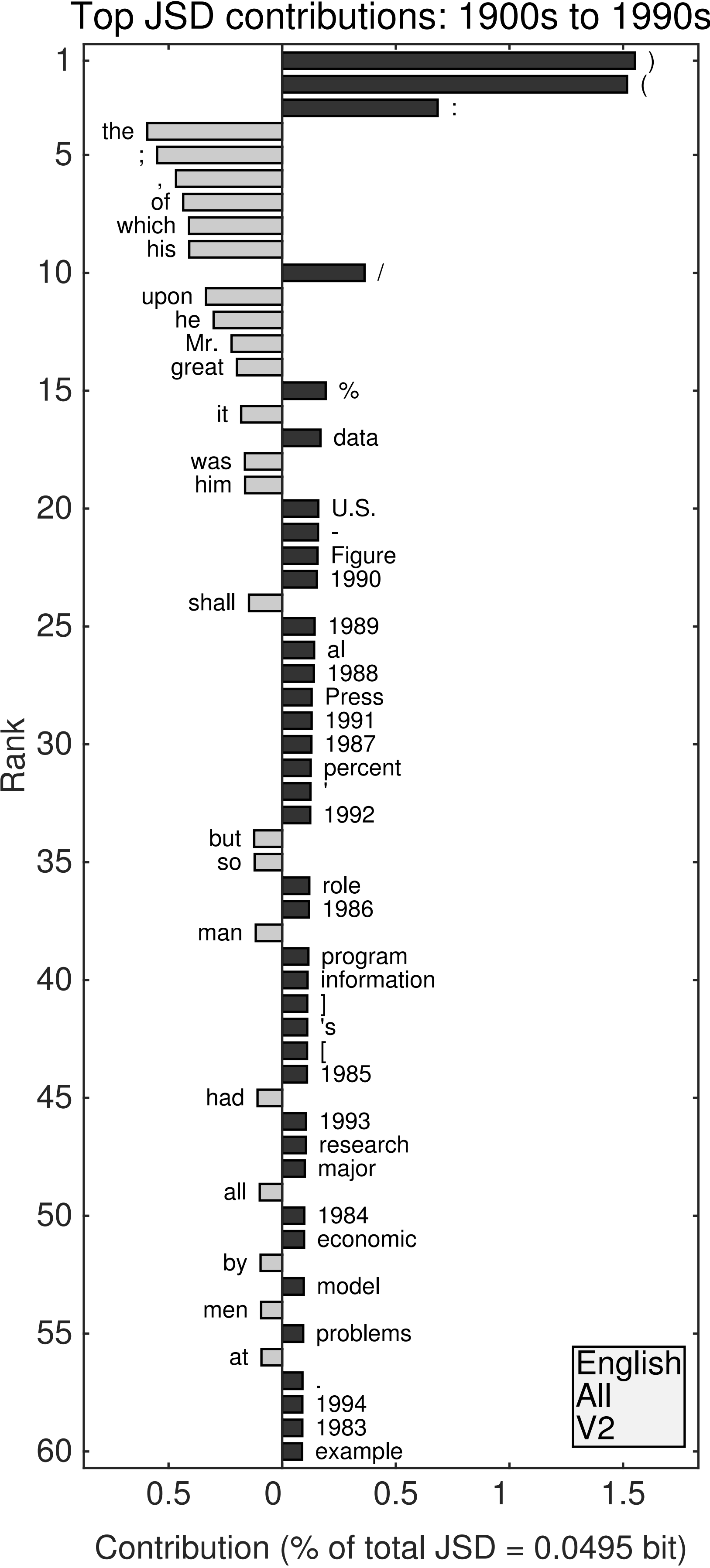
Top JSD contributions: 1900s to 1990s
Rank
Contribution (% of total JSD = 0.0495 bit)
English
All
V2
)
(
:
the
;
,
of
which
his
/
upon
he
Mr.
great
%
it
data
was
him
U.S.
-
Figure
1990
shall
1989
al
1988
Press
1991
1987
percent
'
1992
but
so
role
1986
man
program
information
]
's
[
1985
had
1993
research
major
all
1984
economic
by
model
men
problems
at
.
1994
1983
example

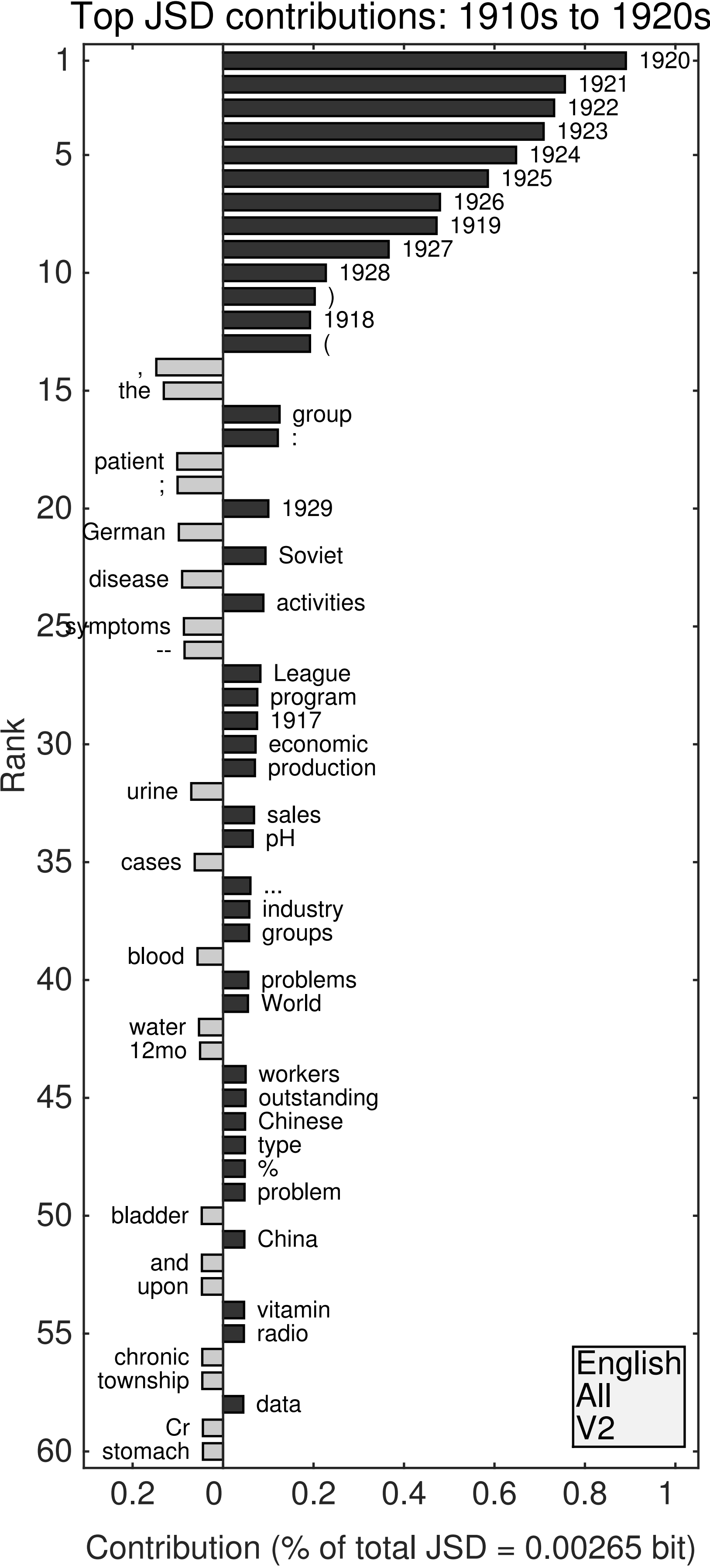
Top JSD contributions: 1910s to 1920s
Rank
Contribution (% of total JSD = 0.00265 bit)
1920
1921
1922
1923
1924
1925
1926
1919
1927
1928
)
1918
(
,
the
group
:
patient
;
1929
German
Soviet
disease
activities
symptoms
--
League
program
1917
economic
production
urine
sales
pH
cases
...
industry
groups
blood
problems
World
water
12mo
workers
outstanding
Chinese
type
%
problem
bladder
China
and
upon
vitamin
radio
chronic
township
data
Cr
stomach
English
All
V2

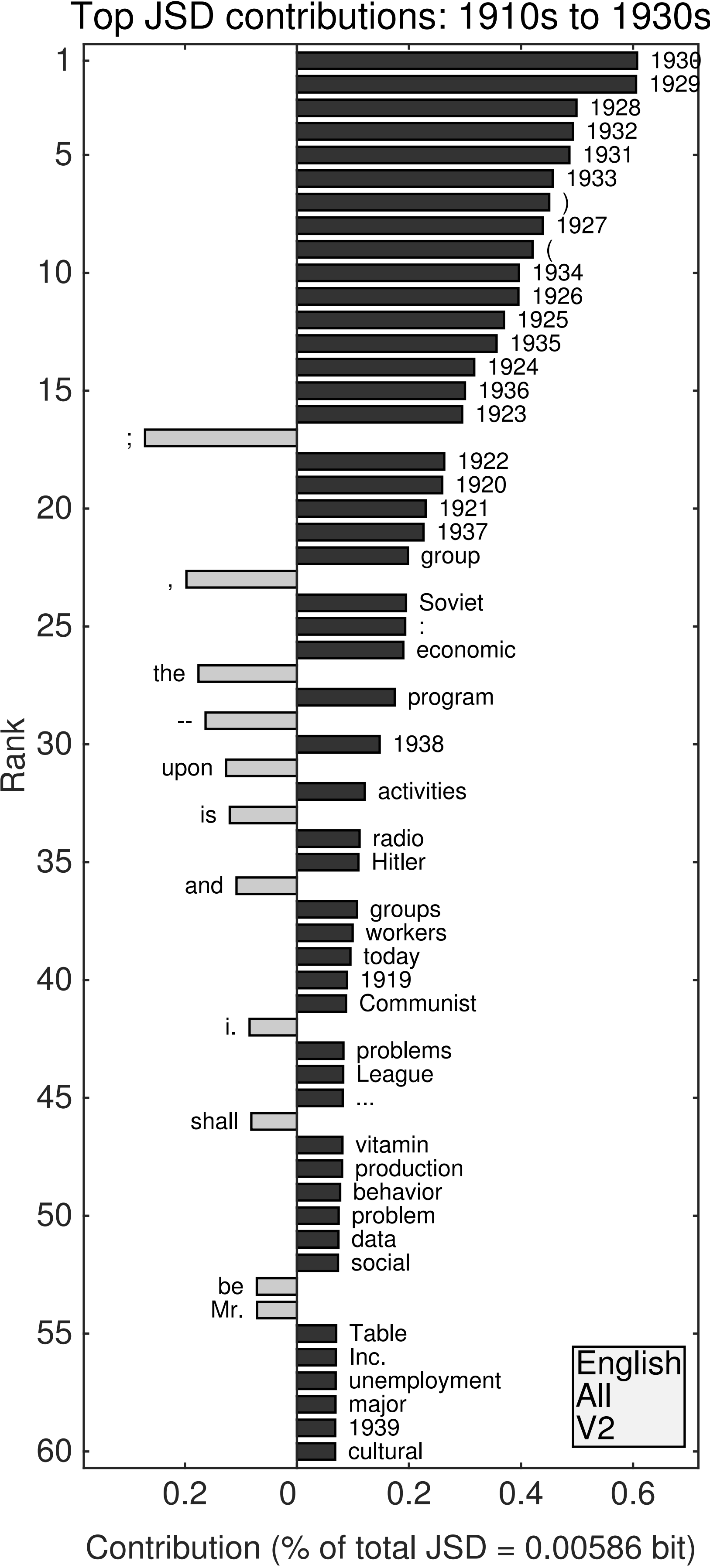

Top JSD contributions: 1910s to 1930s
Rank
1
5
10
15
20
25
30
35
40
45
50
55
60
1930
1929
1928
1932
1931
1933
)
1927
(
1934
1926
1925
1935
1924
1936
1923
;
1922
1920
1921
1937
group
,
Soviet
:
economic
the
program
--
1938
upon
activities
is
radio
Hitler
and
groups
workers
today
1919
Communist
i.
problems
League
...
shall
vitamin
production
behavior
problem
data
social
be
Mr.
Table
Inc.
unemployment
major
1939
cultural
English
All
V2
0.2
0
0.2
0.4
0.6
Contribution (% of total JSD = 0.00586 bit)

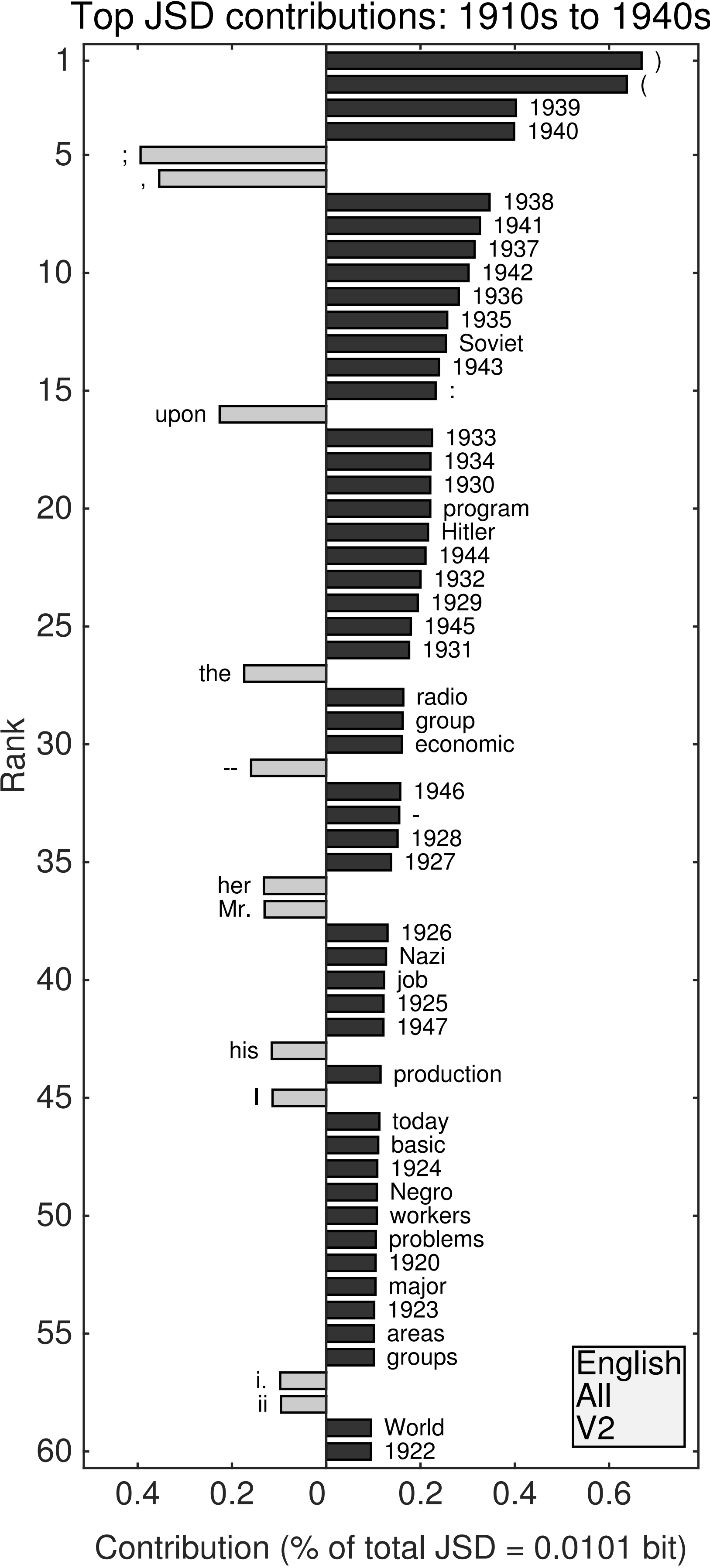

Top JSD contributions: 1910s to 1940s
Rank
1
5
10
15
20
25
30
35
40
45
50
55
60
)
(
1939
1940
;
,
1938
1941
1937
1942
1936
1935
Soviet
1943
:
upon
1933
1934
1930
program
Hitler
1944
1932
1929
1945
1931
the
radio
group
economic
--
1946
-
1928
1927
her
Mr.
1926
Nazi
job
1925
1947
his
production
I
today
basic
1924
Negro
workers
problems
1920
major
1923
areas
groups
i.
ii
World
1922
English
All
V2
0.4
0.2
0
0.2
0.4
0.6
Contribution (% of total JSD = 0.0101 bit)

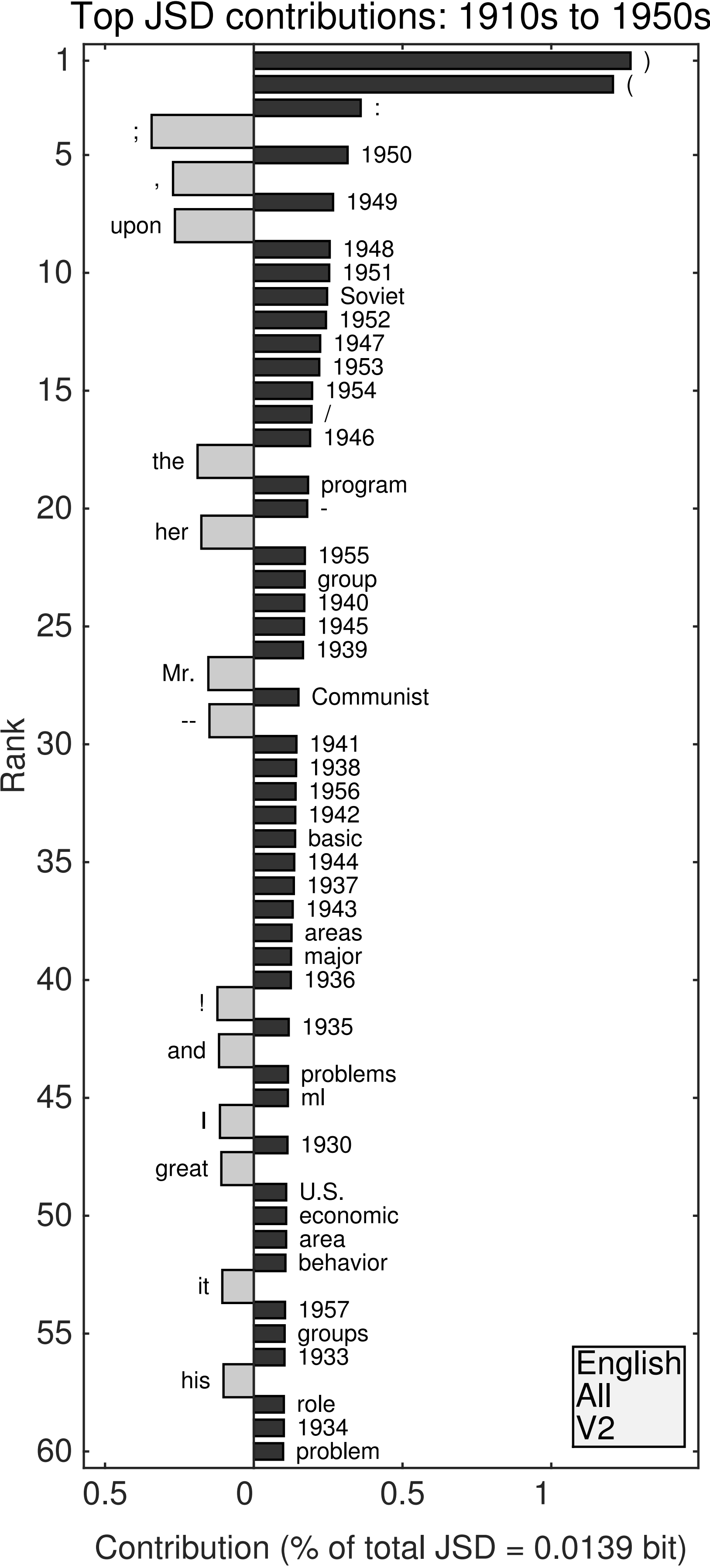

Top JSD contributions: 1910s to 1950s
Rank
Contribution (% of total JSD = 0.0139 bit)
0.5
0
0.5
1
1
5
10
15
20
25
30
35
40
45
50
55
60
)
(
:
;
1950
,
1949
upon
1948
1951
Soviet
1952
1947
1953
1954
/
1946
the
program
-
her
1955
group
1940
1945
1939
Mr.
Communist
--
1941
1938
1956
1942
basic
1944
1937
1943
areas
major
1936
!
1935
and
problems
ml
I
1930
great
U.S.
economic
area
behavior
it
1957
groups
1933
his
role
1934
problem
English
All
V2

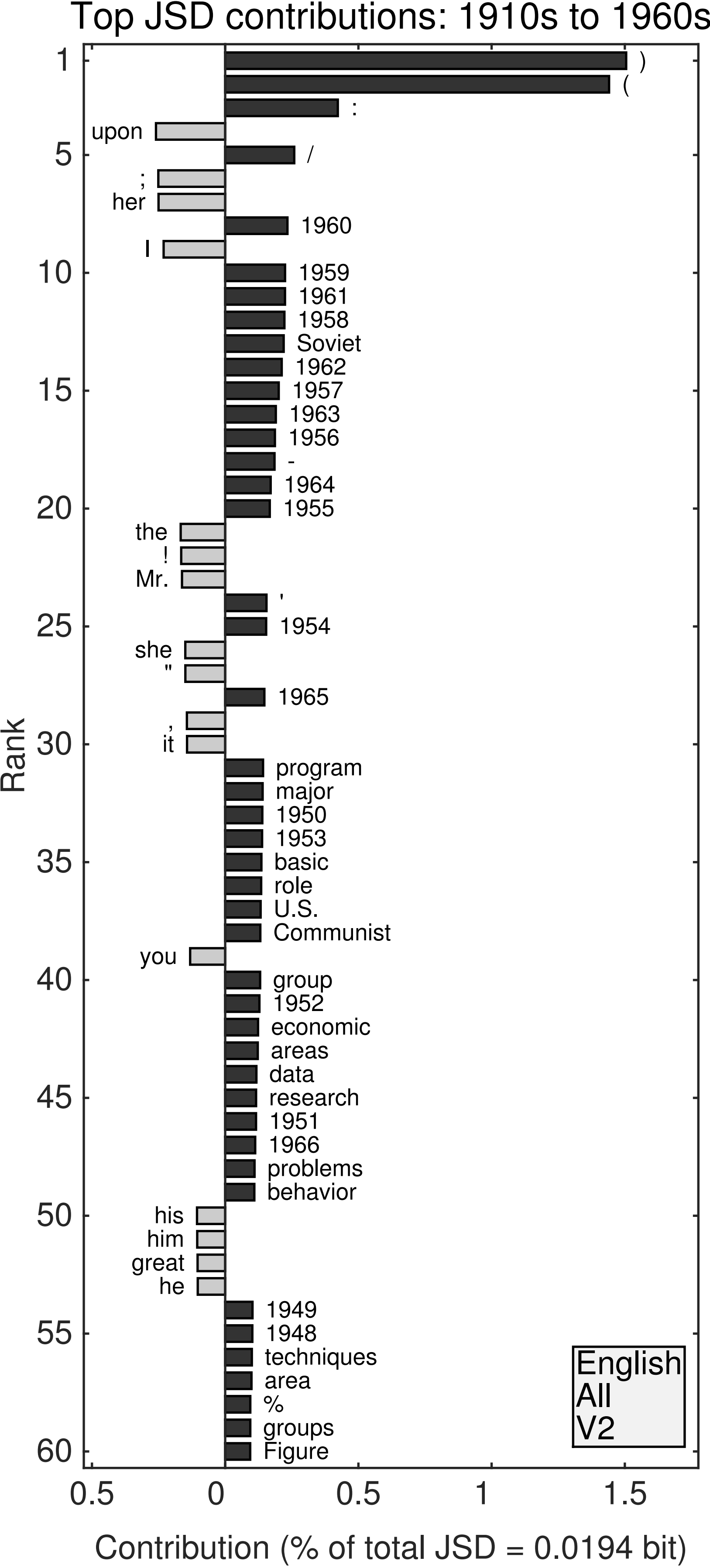
Top JSD contributions: 1910s to 1960s
Rank
1
5
10
15
20
25
30
35
40
45
50
55
60
)
(
:
upon
/
;
her
1960
I
1959
1961
1958
Soviet
1962
1957
1963
1956
-
1964
1955
the
!
Mr.
'
1954
she
"
1965
,
it
program
major
1950
1953
basic
role
U.S.
Communist
you
group
1952
economic
areas
data
research
1951
1966
problems
behavior
his
him
great
he
1949
1948
techniques
area
%
groups
Figure
English
All
V2
0.5
0
0.5
1
1.5
Contribution (% of total JSD = 0.0194 bit)

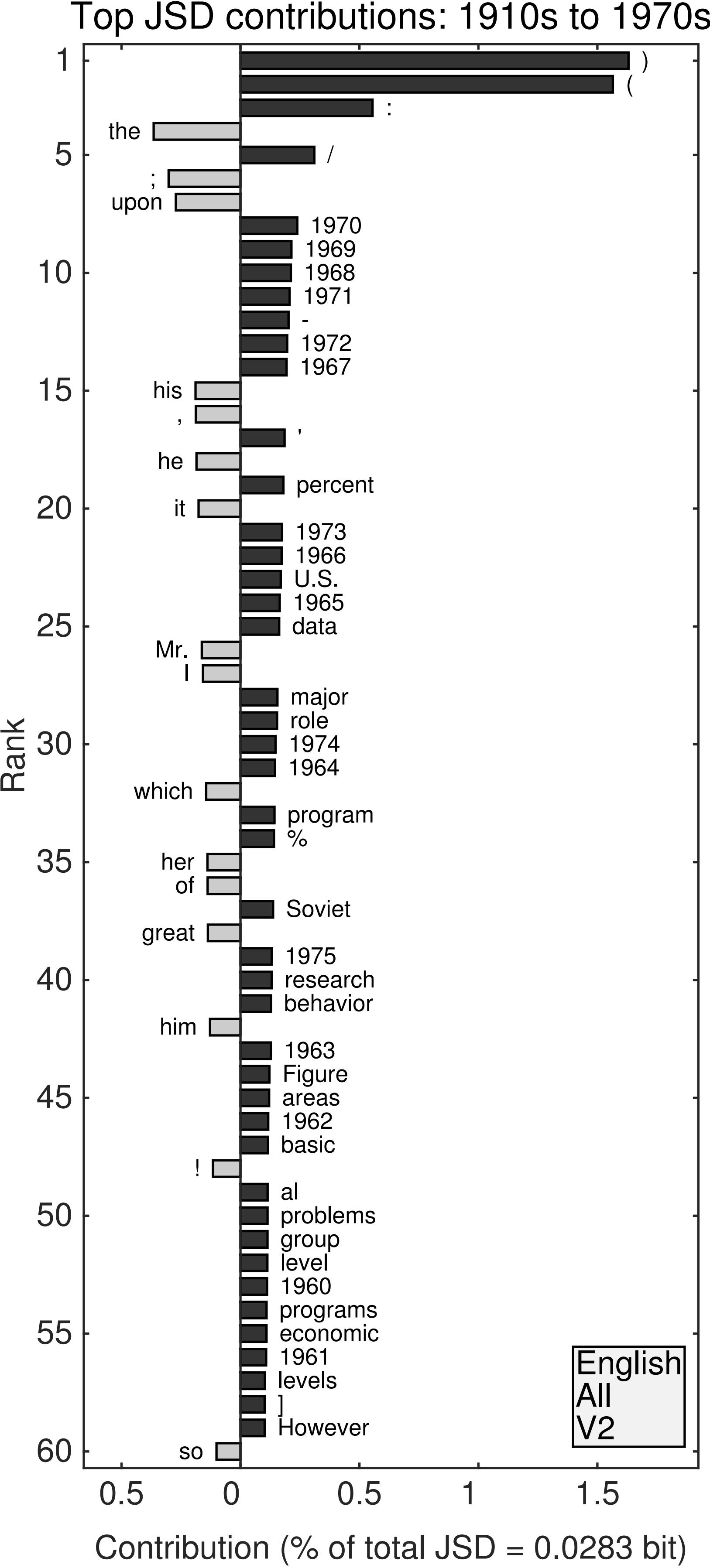

Top JSD contributions: 1910s to 1970s
Rank
Contribution (% of total JSD = 0.0283 bit)
)
(
:
the
/
;
upon
1970
1969
1968
1971
-
1972
1967
his
,
'
he
percent
it
1973
1966
U.S.
1965
data
Mr.
I
major
role
1974
1964
which
program
%
her
of
Soviet
great
1975
research
behavior
him
1963
Figure
areas
1962
basic
!
al
problems
group
level
1960
programs
economic
1961
levels
]
However
so
English
All
V2

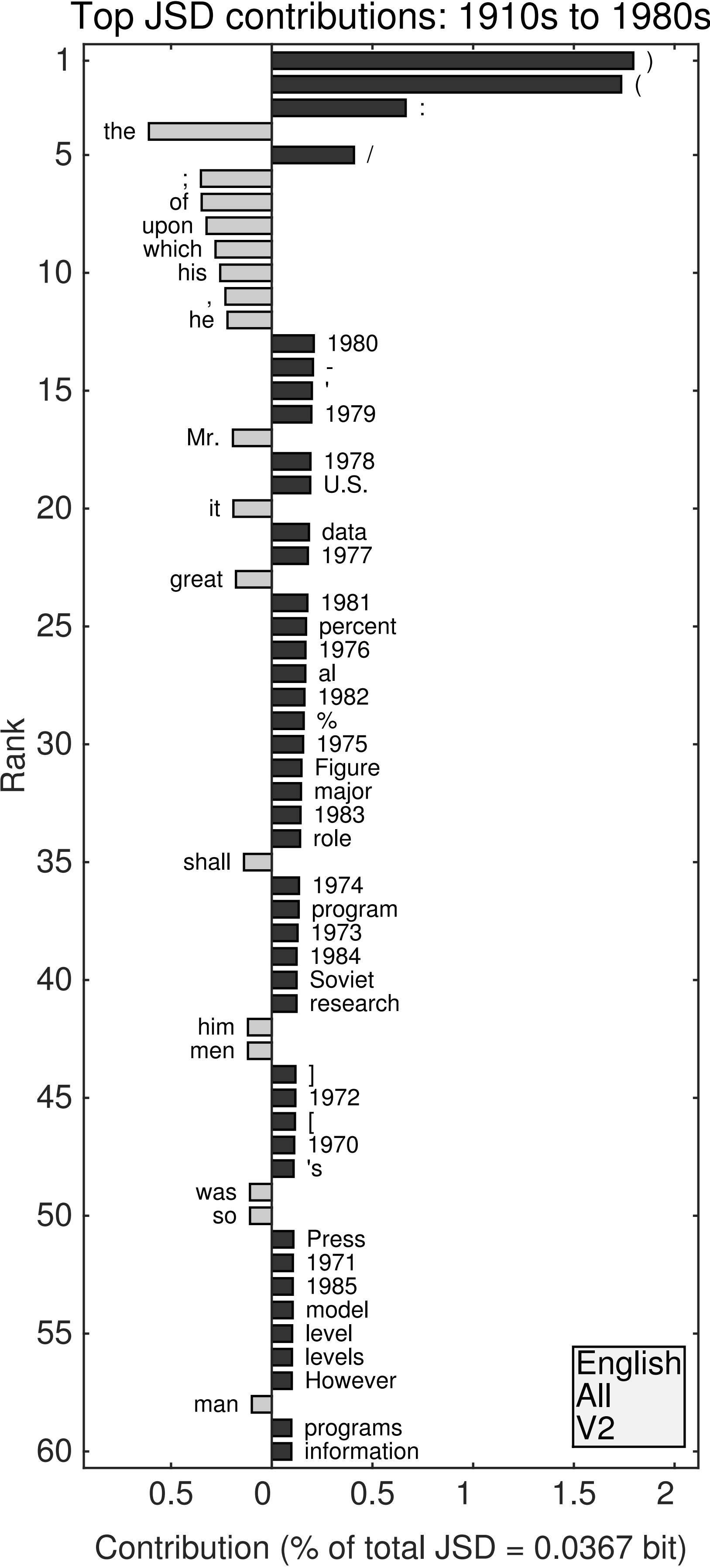

Top JSD contributions: 1910s to 1980s
Rank
1
5
10
15
20
25
30
35
40
45
50
55
60
)
(
:
the
/
;
of
upon
which
his
,
he
1980
-
'
1979
Mr.
1978
U.S.
it
data
1977
great
1981
percent
1976
al
1982
%
1975
Figure
major
1983
role
shall
1974
program
1973
1984
Soviet
research
him
men
]
1972
[
1970
's
was
so
Press
1971
1985
model
level
levels
However
man
programs
information
English
All
V2
0.5
0
0.5
1
1.5
2
Contribution (% of total JSD = 0.0367 bit)

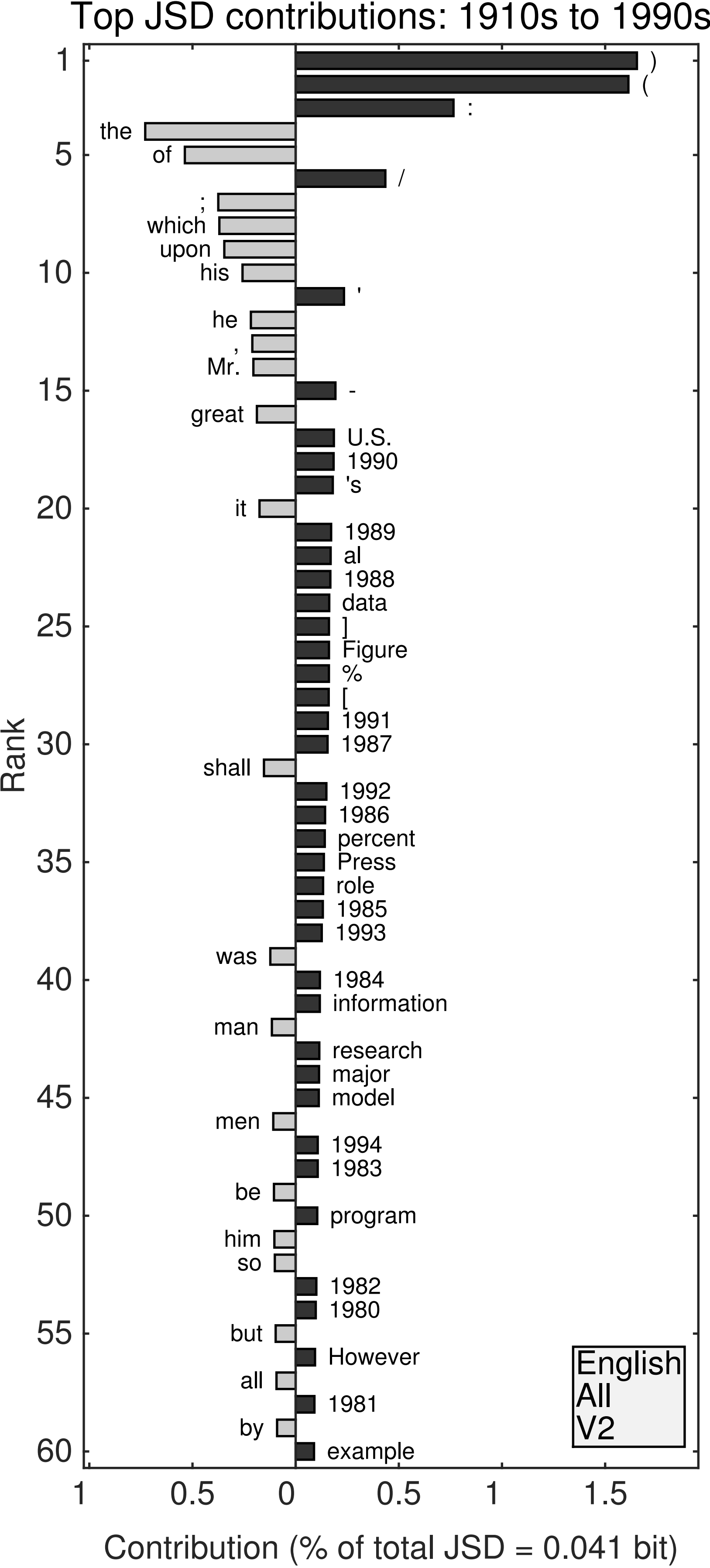
Top JSD contributions: 1910s to 1990s
Rank
Contribution (% of total JSD = 0.041 bit)
)
(
:
the
of
/
;
which
upon
his
'
he
,
Mr.
-
great
U.S.
1990
's
it
1989
al
1988
data
]
Figure
%
[
1991
1987
shall
1992
1986
percent
Press
role
1985
1993
was
1984
information
man
research
major
model
men
1994
1983
be
program
him
so
1982
1980
but
However
all
1981
by
example
English
All
V2

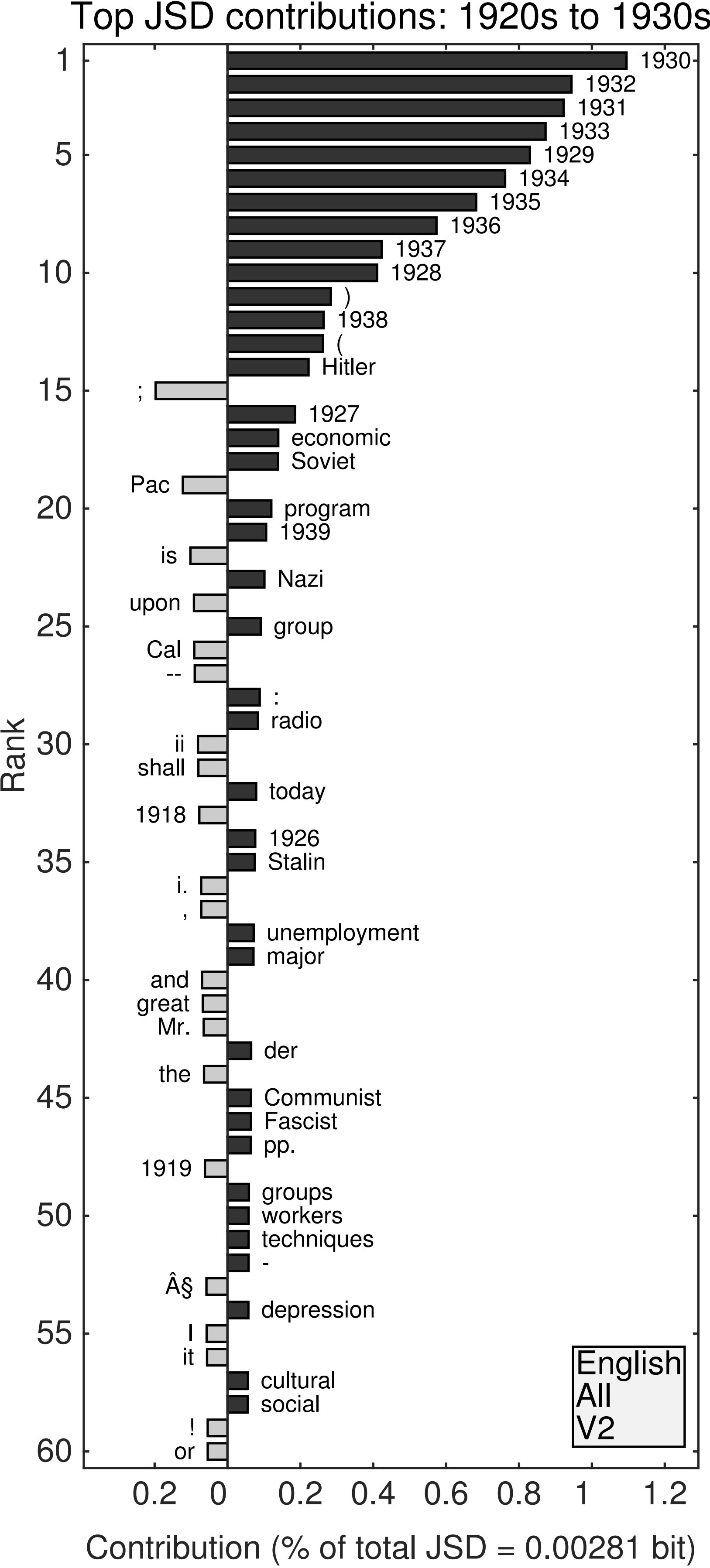

Top JSD contributions: 1920s to 1930s
Rank
Contribution (% of total JSD = 0.00281 bit)
1930
1932
1931
1933
1929
1934
1935
1936
1937
1928
)
1938
(
Hitler
;
1927
economic
Soviet
Pac
program
1939
is
Nazi
upon
group
Cal
--
:
radio
ii
shall
today
1918
1926
Stalin
i.
,
unemployment
major
and
great
Mr.
der
the
Communist
Fascist
pp.
1919
groups
workers
techniques
-
Â§
depression
I
it
cultural
social
!
or
English
All
V2

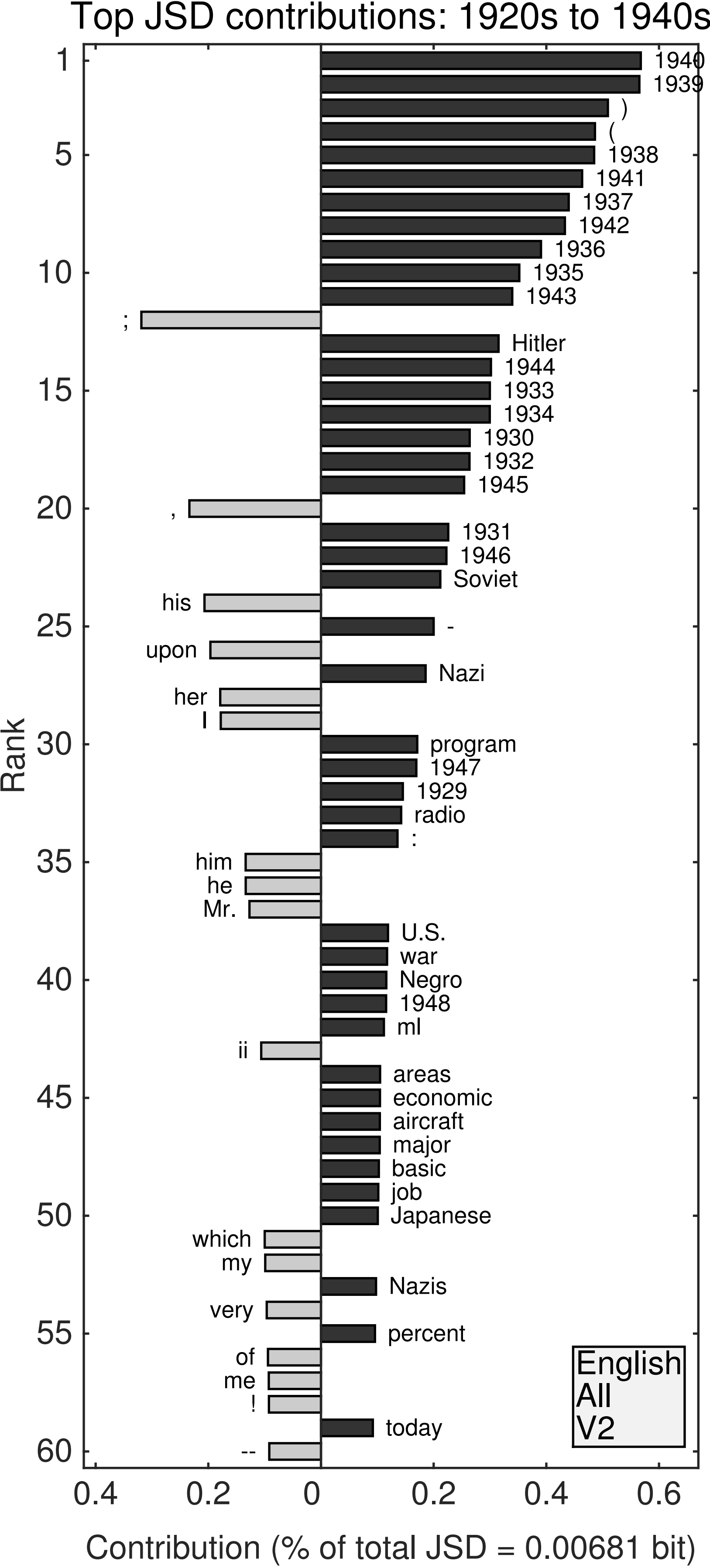
Top JSD contributions: 1920s to 1940s
Rank
Contribution (% of total JSD = 0.00681 bit)
1940
1939
)
(
1938
1941
1937
1942
1936
1935
1943
;
Hitler
1944
1933
1934
1930
1932
1945
,
1931
1946
Soviet
his
-
upon
Nazi
her
I
program
1947
1929
radio
:
him
he
Mr.
U.S.
war
Negro
1948
ml
ii
areas
economic
aircraft
major
basic
job
Japanese
which
my
Nazis
very
percent
of
me
!
today
--
English
All
V2

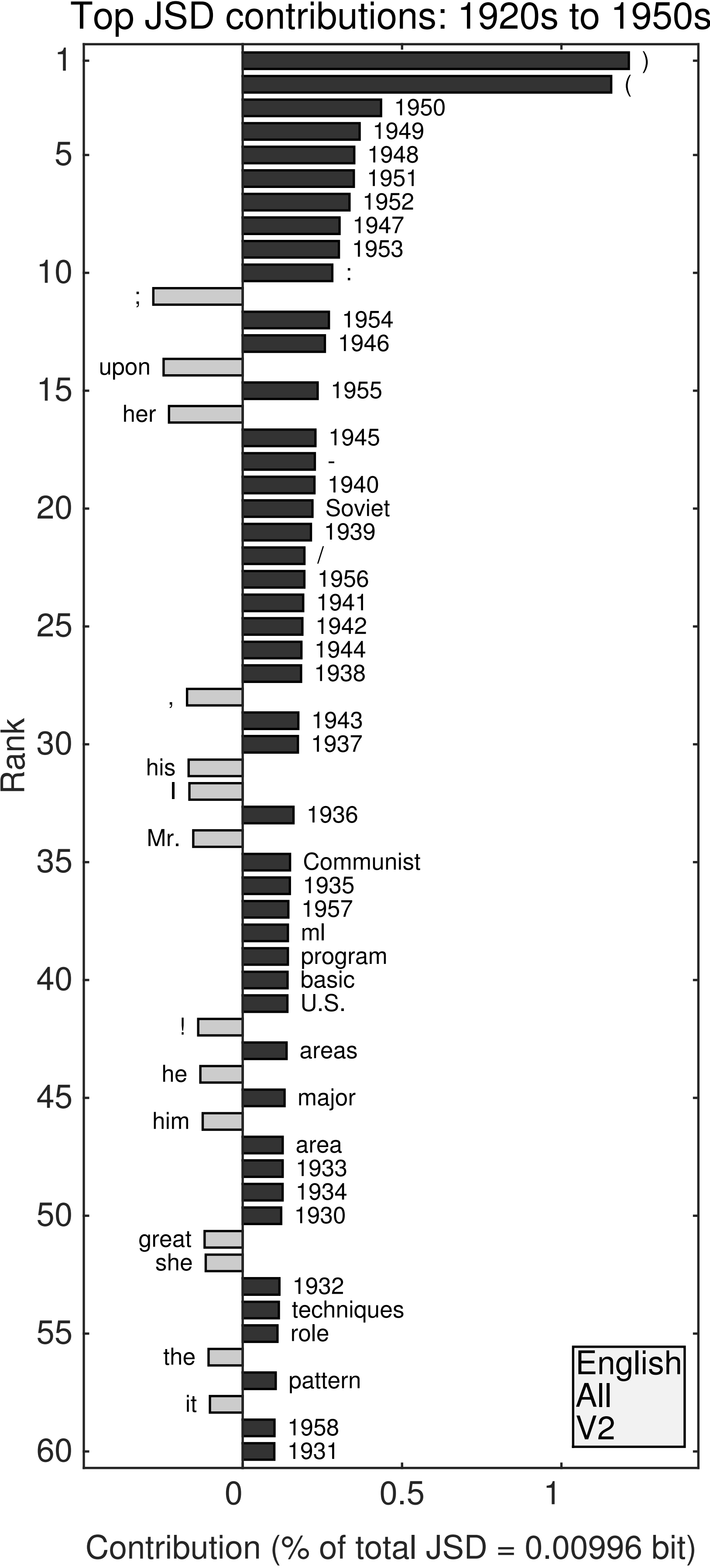

Top JSD contributions: 1920s to 1950s
Rank
Contribution (% of total JSD = 0.00996 bit)
English
All
V2
)
(
1950
1949
1948
1951
1952
1947
1953
:
;
1954
1946
upon
1955
her
1945
-
1940
Soviet
1939
/
1956
1941
1942
1944
1938
,
1943
1937
his
I
1936
Mr.
Communist
1935
1957
ml
program
basic
U.S.
!
areas
he
major
him
area
1933
1934
1930
great
she
1932
techniques
role
the
pattern
it
1958
1931

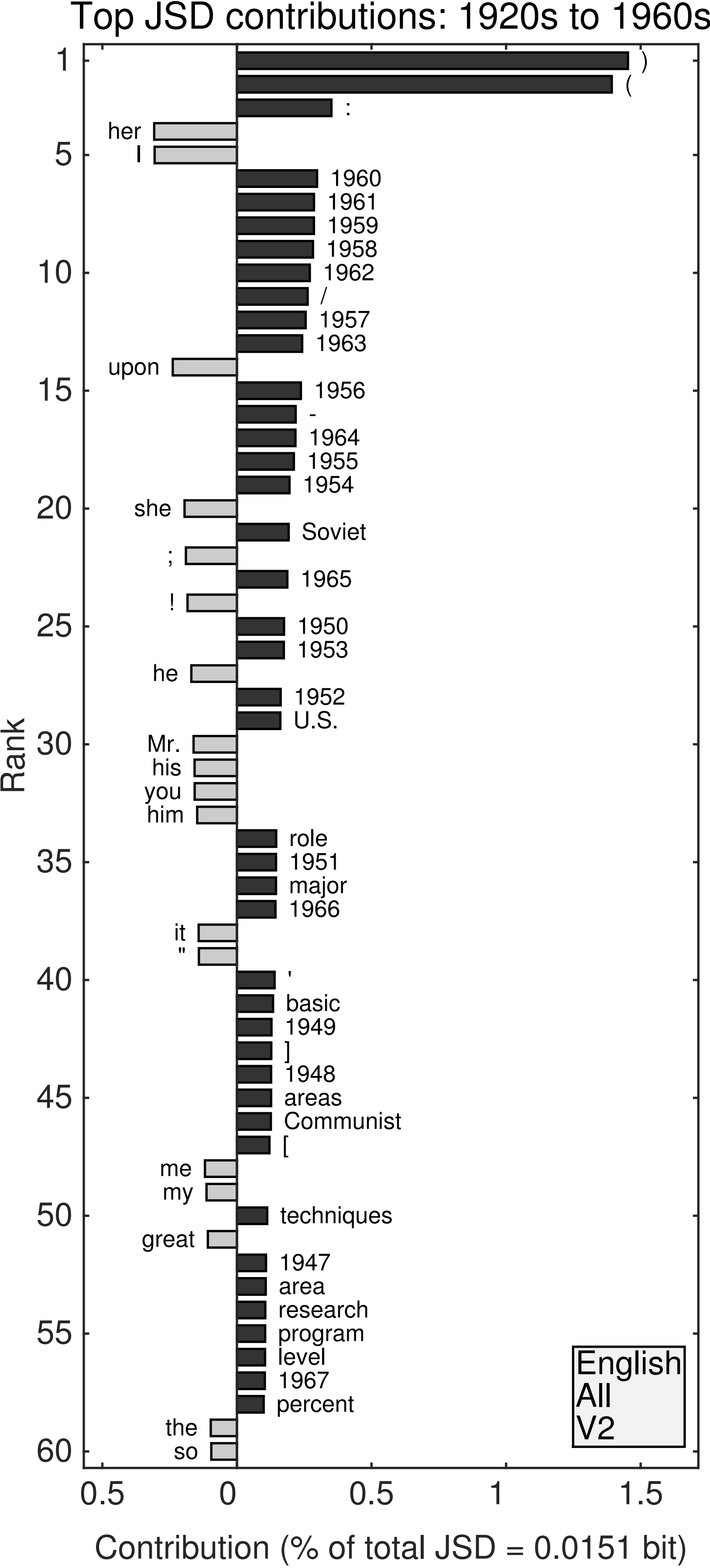
Top JSD contributions: 1920s to 1960s
Rank
)
(
:
her
I
1960
1961
1959
1958
1962
/
1957
1963
upon
1956
-
1964
1955
1954
she
Soviet
;
1965
!
1950
1953
he
1952
U.S.
Mr.
his
you
him
role
1951
major
1966
it
"
'
basic
1949
]
1948
areas
Communist
[
me
my
techniques
great
1947
area
research
program
level
1967
percent
the
so
English
All
V2
Contribution (% of total JSD = 0.0151 bit)

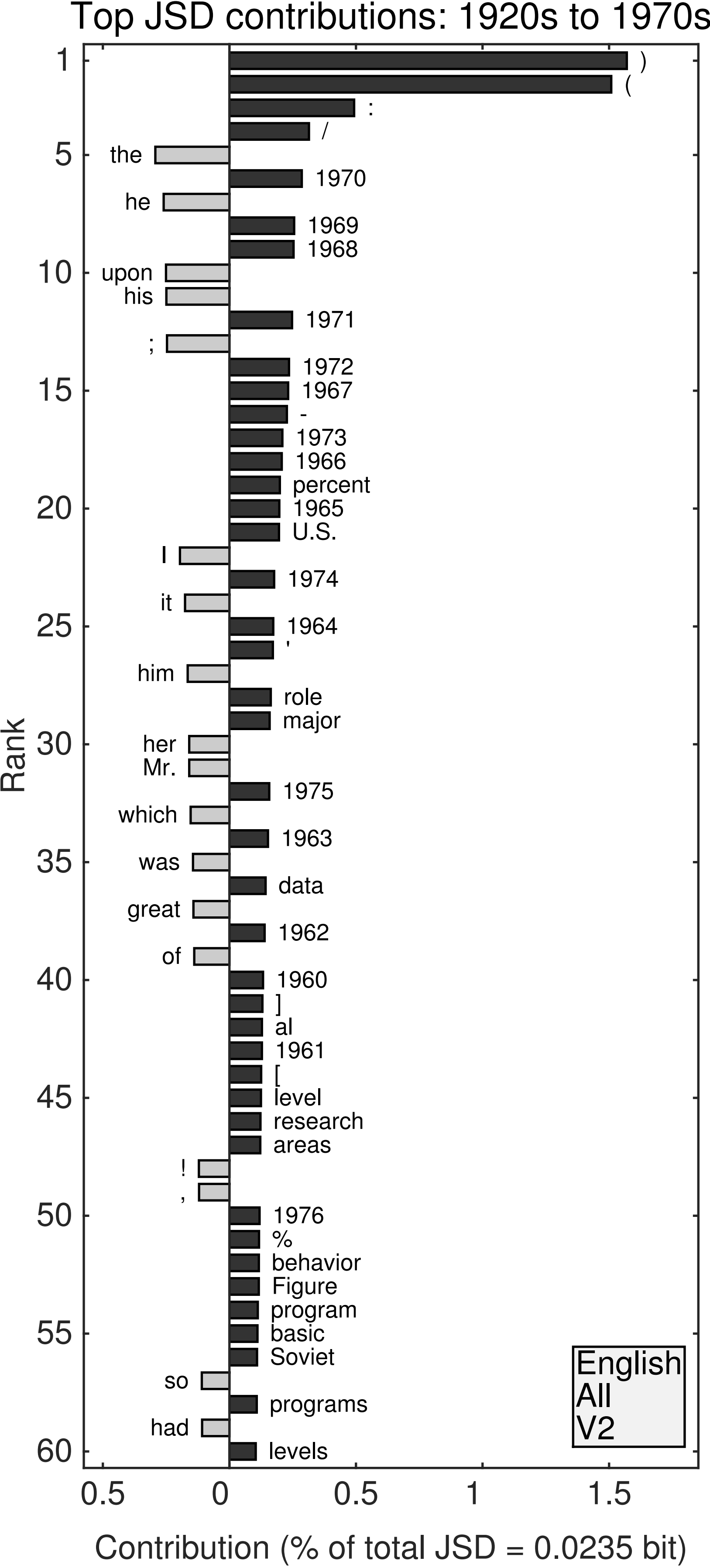

Top JSD contributions: 1920s to 1970s
Rank
Contribution (% of total JSD = 0.0235 bit)
0.5
0
0.5
1
1.5
1
5
10
15
20
25
30
35
40
45
50
55
60
)
(
:
/
the
1970
he
1969
1968
upon
his
1971
;
1972
1967
-
1973
1966
percent
1965
U.S.
I
1974
it
1964
'
him
role
major
her
Mr.
1975
which
1963
was
data
great
1962
of
1960
]
al
1961
[
level
research
areas
!
,
1976
%
behavior
Figure
program
basic
Soviet
so
programs
had
levels
English
All
V2

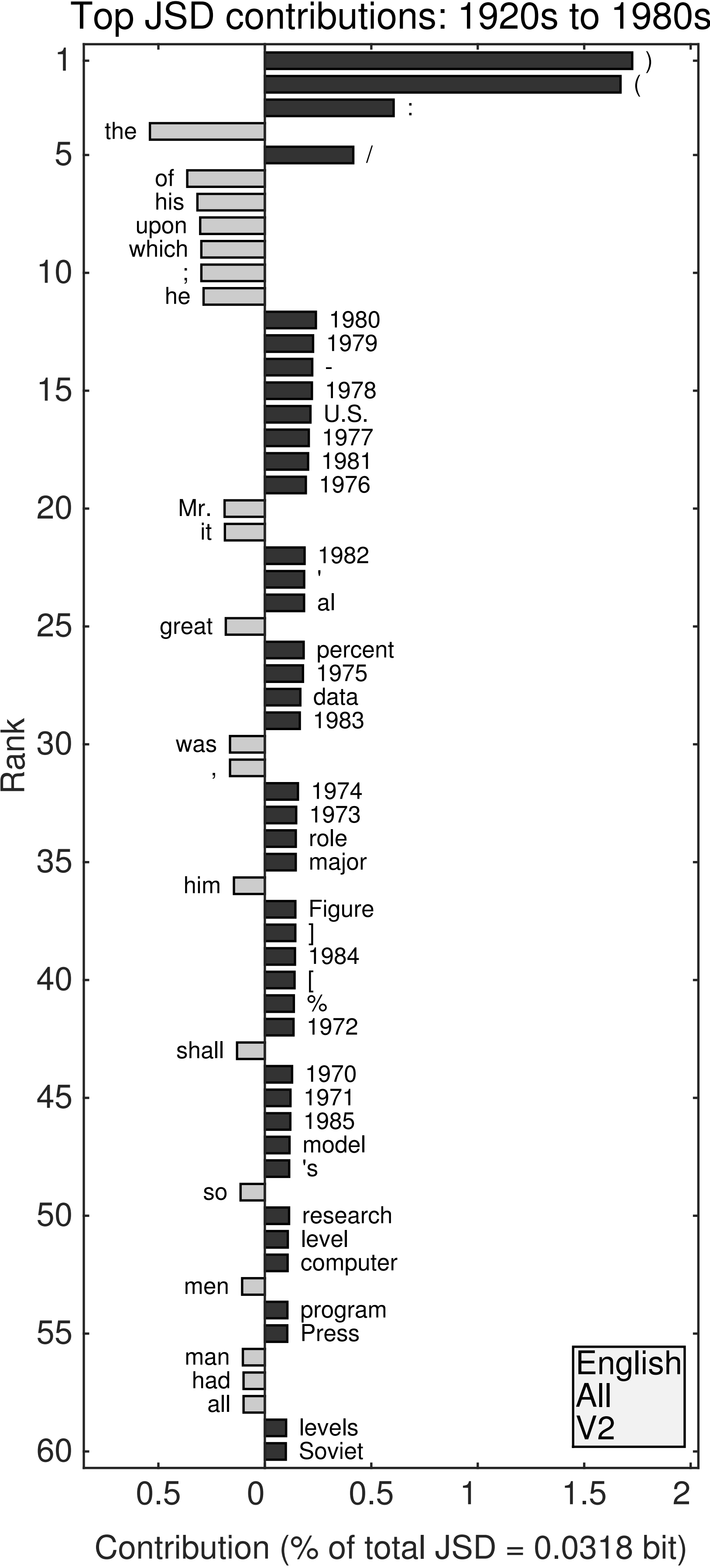

Top JSD contributions: 1920s to 1980s
)
(
:
the
/
of
his
upon
which
;
he
1980
1979
-
1978
U.S.
1977
1981
1976
Mr.
it
1982
'
al
great
percent
1975
data
1983
was
,
1974
1973
role
major
him
Figure
]
1984
[
%
1972
shall
1970
1971
1985
model
's
so
research
level
computer
men
program
Press
man
had
all
levels
Soviet
English
All
V2
Rank
Contribution (% of total JSD = 0.0318 bit)

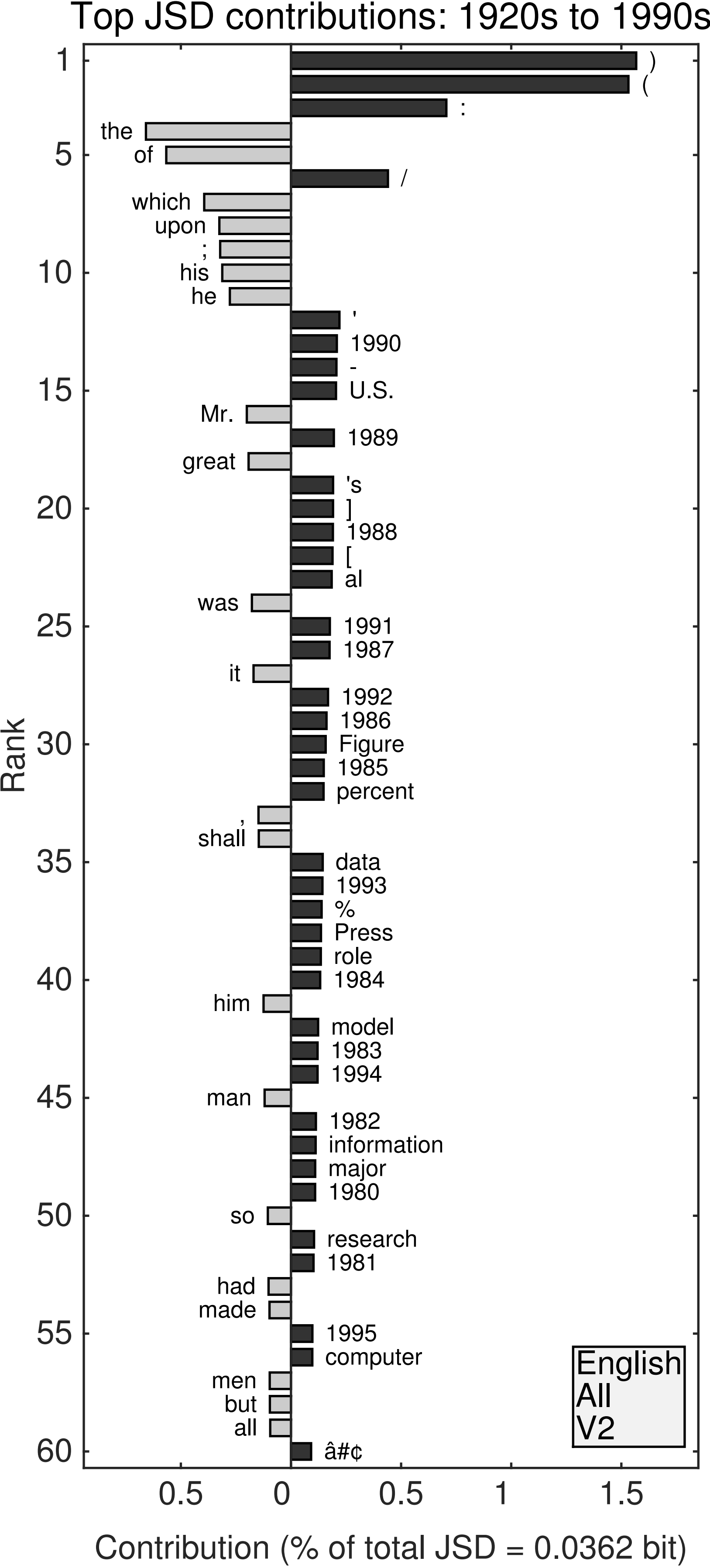

Top JSD contributions: 1920s to 1990s
Rank
1
5
10
15
20
25
30
35
40
45
50
55
60
)
(
:
the
of
/
which
upon
;
his
he
'
1990
-
U.S.
Mr.
1989
great
's
]
1988
[
al
was
1991
1987
it
1992
1986
Figure
1985
percent
,
shall
data
1993
%
Press
role
1984
him
model
1983
1994
man
1982
information
major
1980
so
research
1981
had
made
1995
computer
men
but
all
â#¢
English
All
V2
0.5
0
0.5
1
1.5
Contribution (% of total JSD = 0.0362 bit)

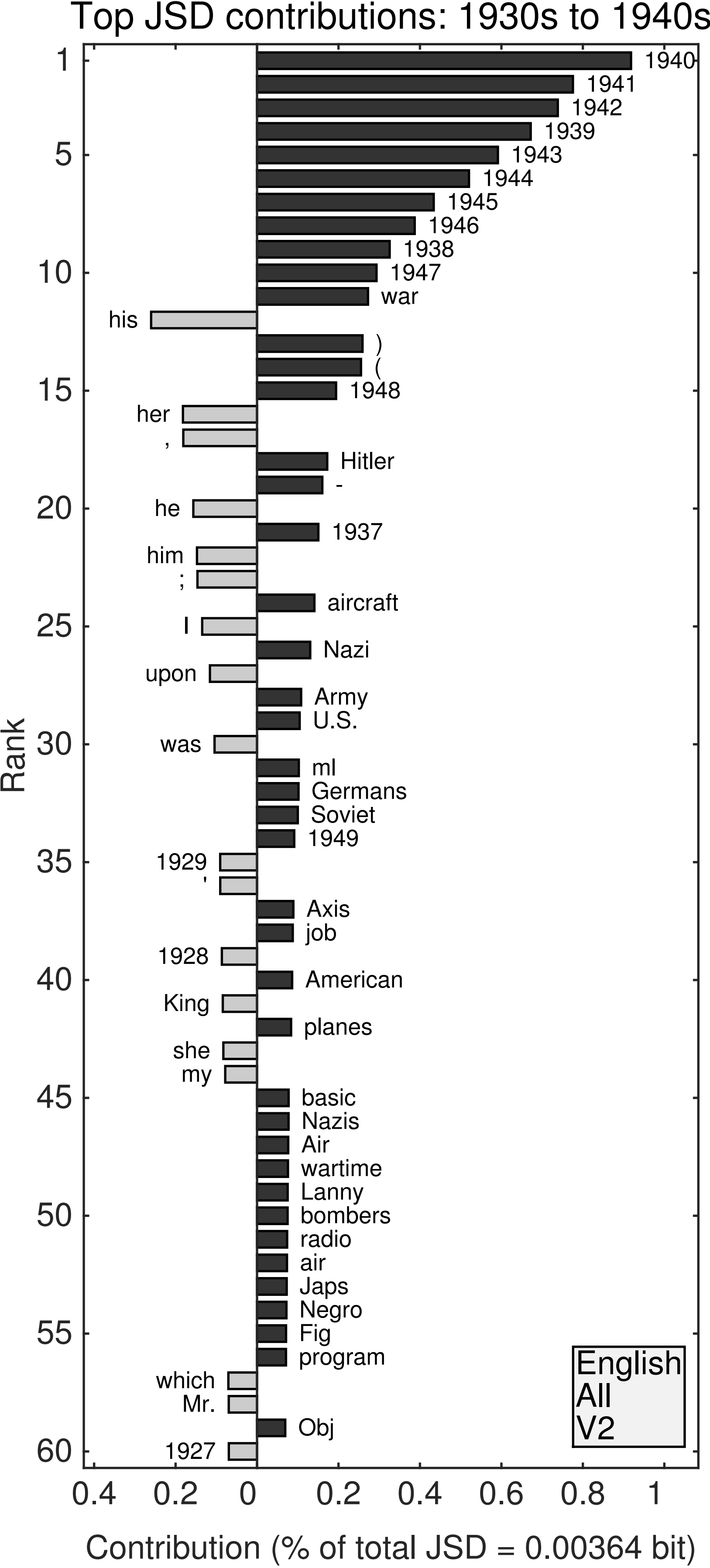

Top JSD contributions: 1930s to 1940s
Rank
Contribution (% of total JSD = 0.00364 bit)
1940
1941
1942
1939
1943
1944
1945
1946
1938
1947
war
his
)
(
1948
her
,
Hitler
-
he
1937
him
;
aircraft
I
Nazi
upon
Army
U.S.
was
ml
Germans
Soviet
1949
1929
'
Axis
job
1928
American
King
planes
she
my
basic
Nazis
Air
wartime
Lanny
bombers
radio
air
Japs
Negro
Fig
program
which
Mr.
Obj
1927
English
All
V2

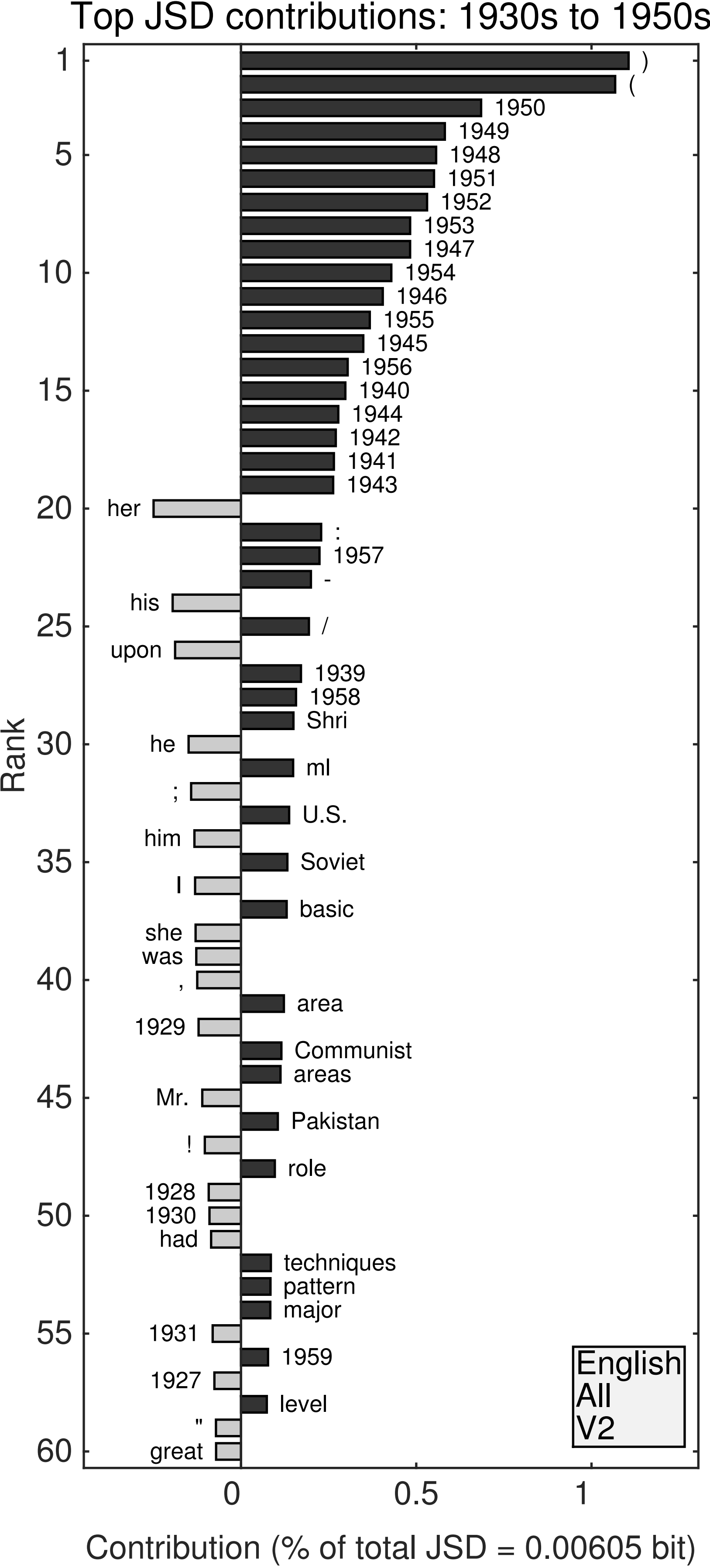

Top JSD contributions: 1930s to 1950s
Rank
Contribution (% of total JSD = 0.00605 bit)
)
(
1950
1949
1948
1951
1952
1953
1947
1954
1946
1955
1945
1956
1940
1944
1942
1941
1943
her
:
1957
-
his
/
upon
1939
1958
Shri
he
ml
;
U.S.
him
Soviet
I
basic
she
was
,
area
1929
Communist
areas
Mr.
Pakistan
!
role
1928
1930
had
techniques
pattern
major
1931
1959
1927
level
"
great
English
All
V2

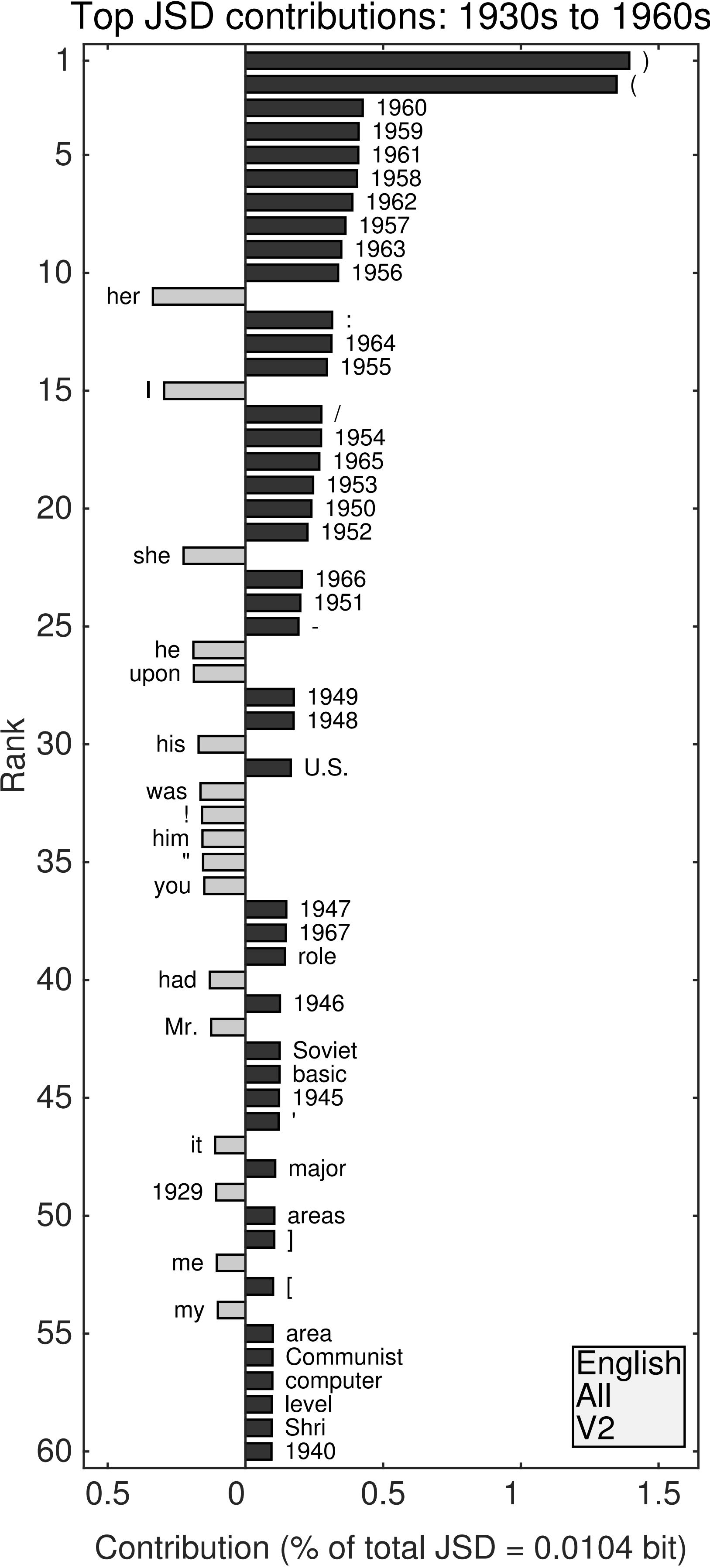
Top JSD contributions: 1930s to 1960s
Rank
Contribution (% of total JSD = 0.0104 bit)
)
(
1960
1959
1961
1958
1962
1957
1963
1956
her
:
1964
1955
I
/
1954
1965
1953
1950
1952
she
1966
1951
-
he
upon
1949
1948
his
U.S.
was
!
him
"
you
1947
1967
role
had
1946
Mr.
Soviet
basic
1945
'
it
major
1929
areas
]
me
[
my
area
Communist
computer
level
Shri
1940
English
All
V2

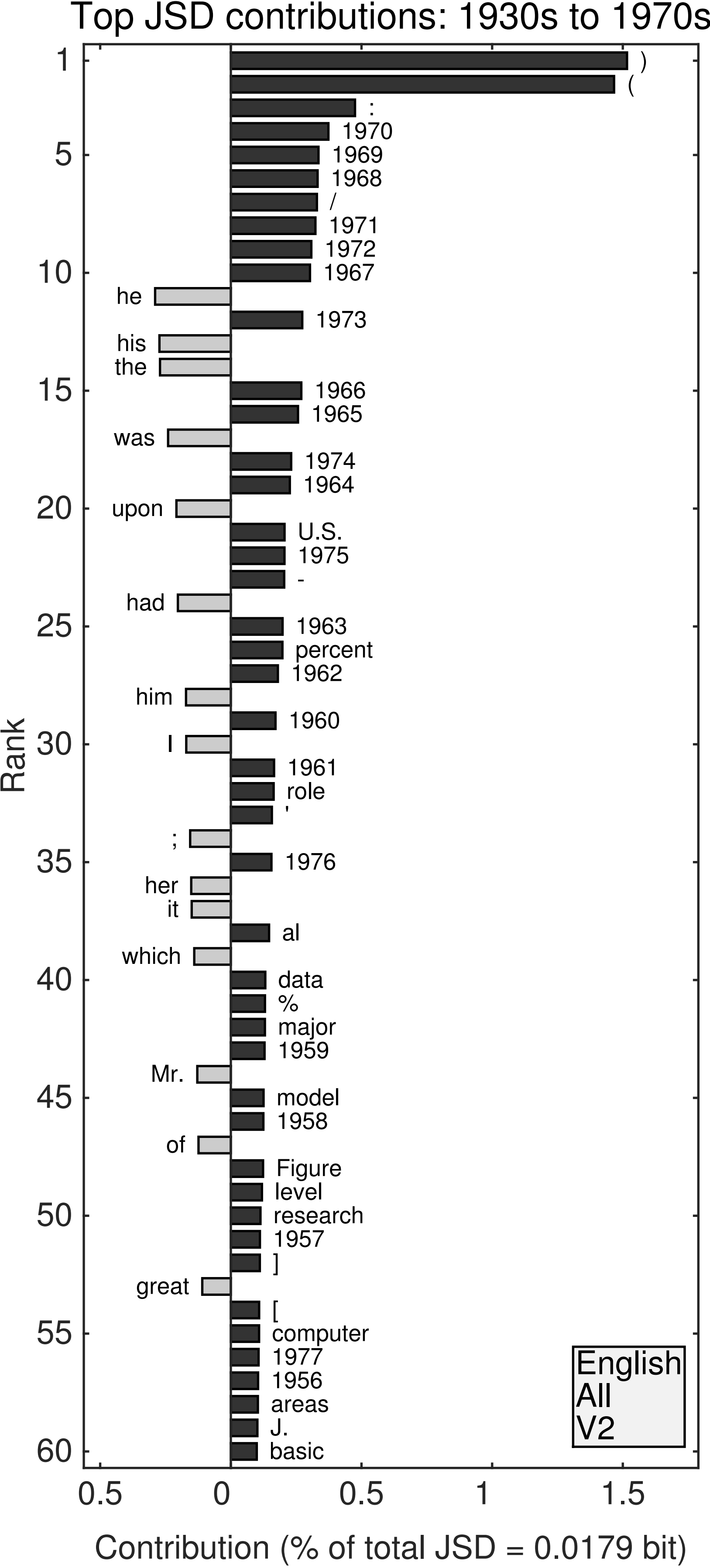

Top JSD contributions: 1930s to 1970s
Rank
Contribution (% of total JSD = 0.0179 bit)
)
(
:
1970
1969
1968
/
1971
1972
1967
he
1973
his
the
1966
1965
was
1974
1964
upon
U.S.
1975
-
had
1963
percent
1962
him
1960
I
1961
role
'
;
1976
her
it
al
which
data
%
major
1959
Mr.
model
1958
of
Figure
level
research
1957
]
great
[
computer
1977
1956
areas
J.
basic
English
All
V2

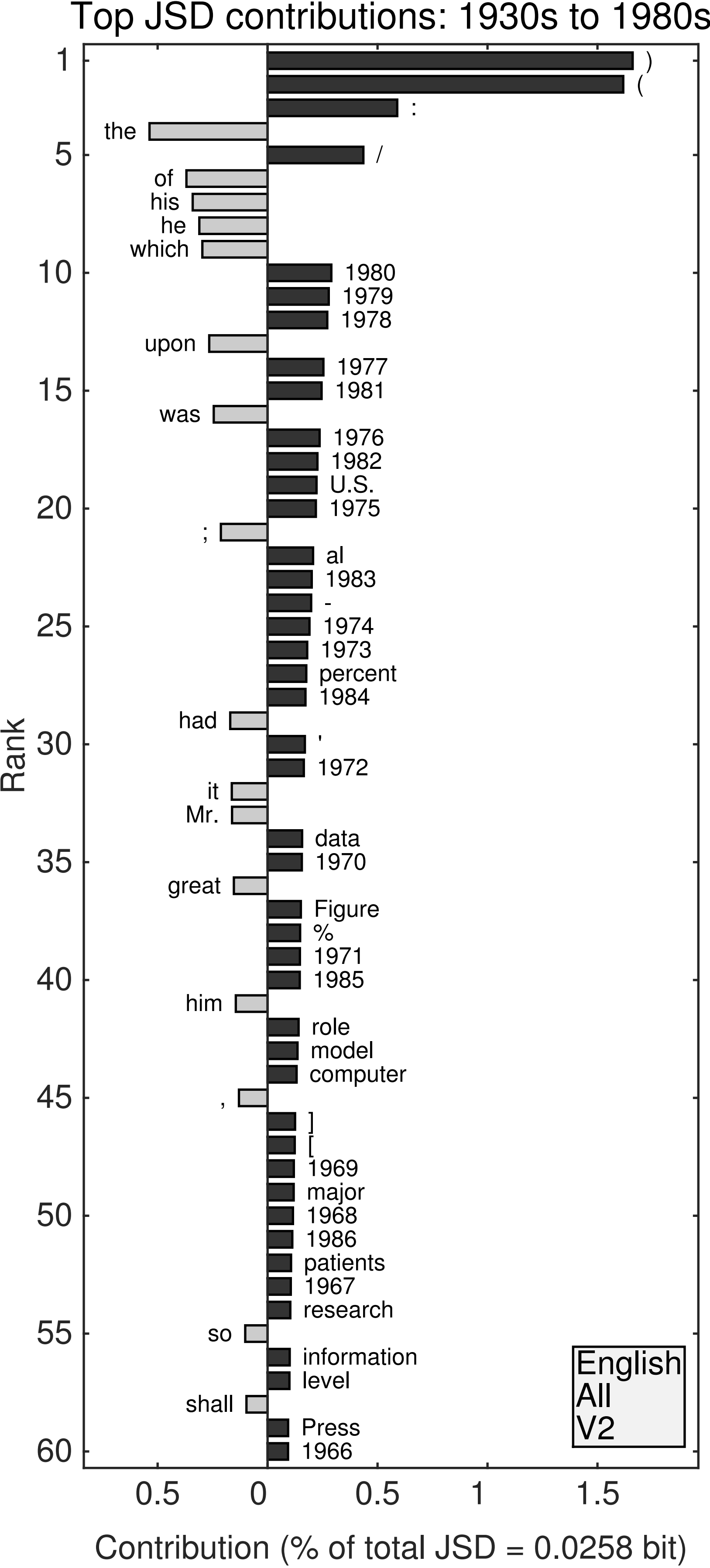
Top JSD contributions: 1930s to 1980s
Rank
Contribution (% of total JSD = 0.0258 bit)
)
(
:
the
/
of
his
he
which
1980
1979
1978
upon
1977
1981
was
1976
1982
U.S.
1975
;
al
1983
-
1974
1973
percent
1984
had
'
1972
it
Mr.
data
1970
great
Figure
%
1971
1985
him
role
model
computer
,
]
[
1969
major
1968
1986
patients
1967
research
so
information
level
shall
Press
1966
English
All
V2

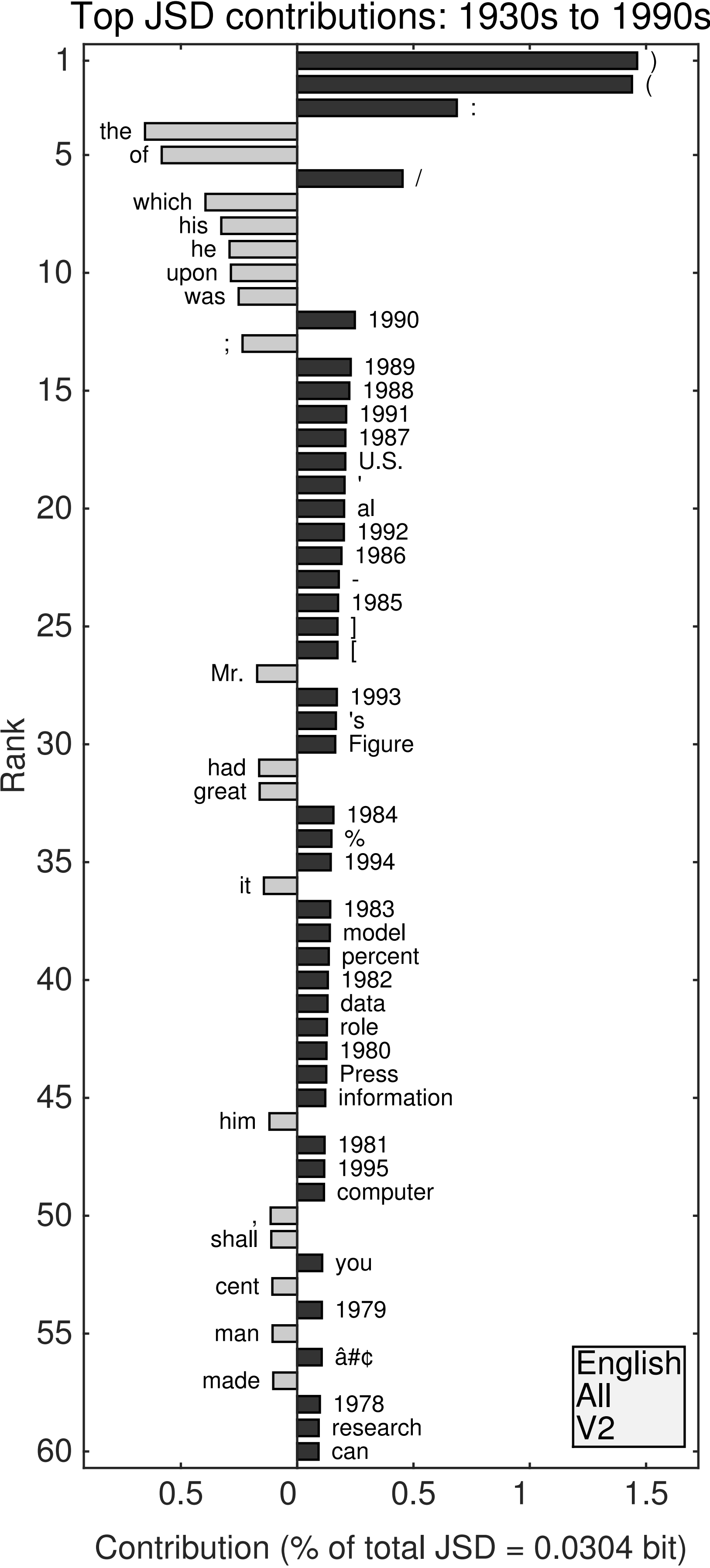

Top JSD contributions: 1930s to 1990s
Rank
Contribution (% of total JSD = 0.0304 bit)
)
(
:
the
of
/
which
his
he
upon
was
1990
;
1989
1988
1991
1987
U.S.
'
al
1992
1986
-
1985
]
[
Mr.
1993
's
Figure
had
great
1984
%
1994
it
1983
model
percent
1982
data
role
1980
Press
information
him
1981
1995
computer
,
shall
you
cent
1979
man
â#¢
made
1978
research
can
English
All
V2

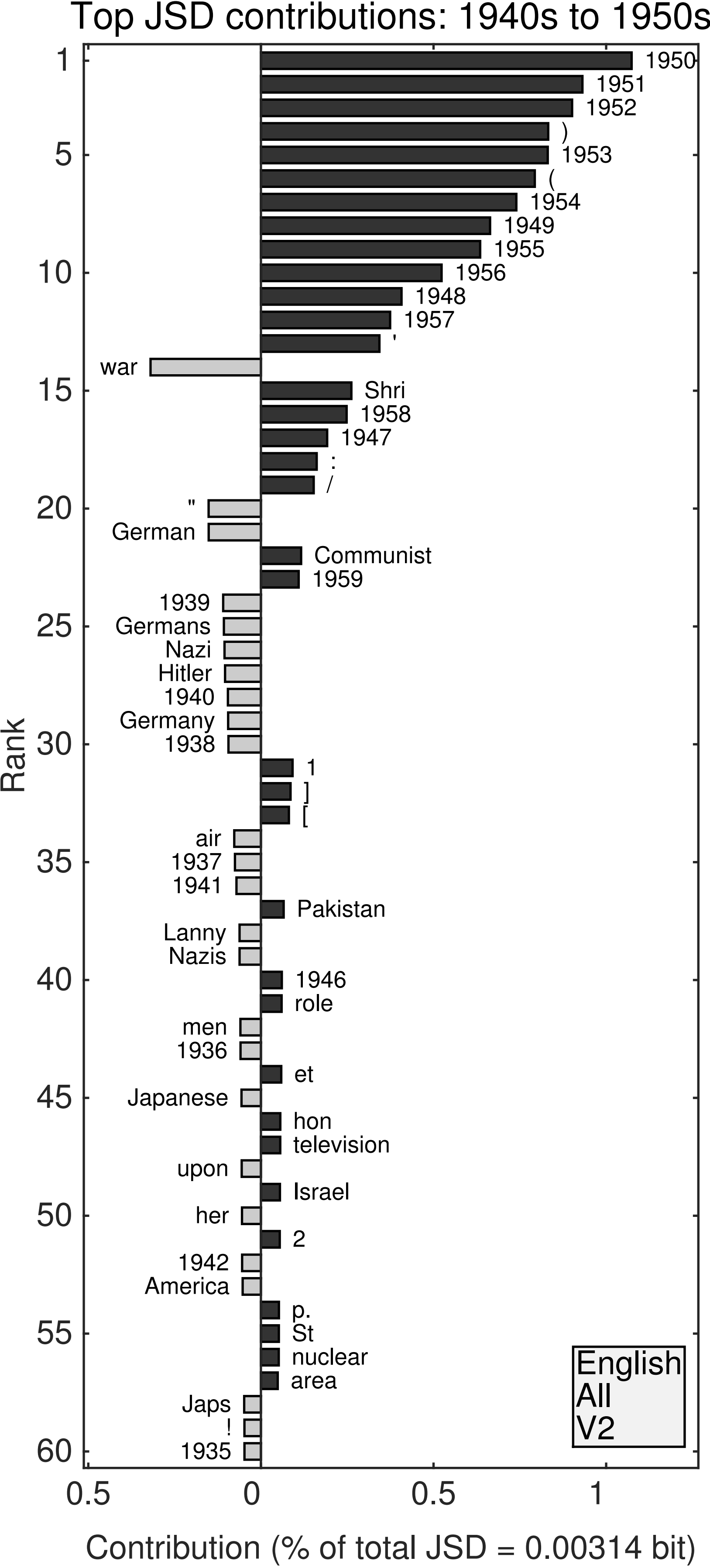
Top JSD contributions: 1940s to 1950s
Rank
Contribution (% of total JSD = 0.00314 bit)
1950
1951
1952
)
1953
(
1954
1949
1955
1956
1948
1957
'
war
Shri
1958
1947
:
/
"
German
Communist
1959
1939
Germans
Nazi
Hitler
1940
Germany
1938
1
]
[
air
1937
1941
Pakistan
Lanny
Nazis
1946
role
men
1936
et
Japanese
hon
television
upon
Israel
her
2
1942
America
p.
St
nuclear
area
Japs
!
1935
English
All
V2

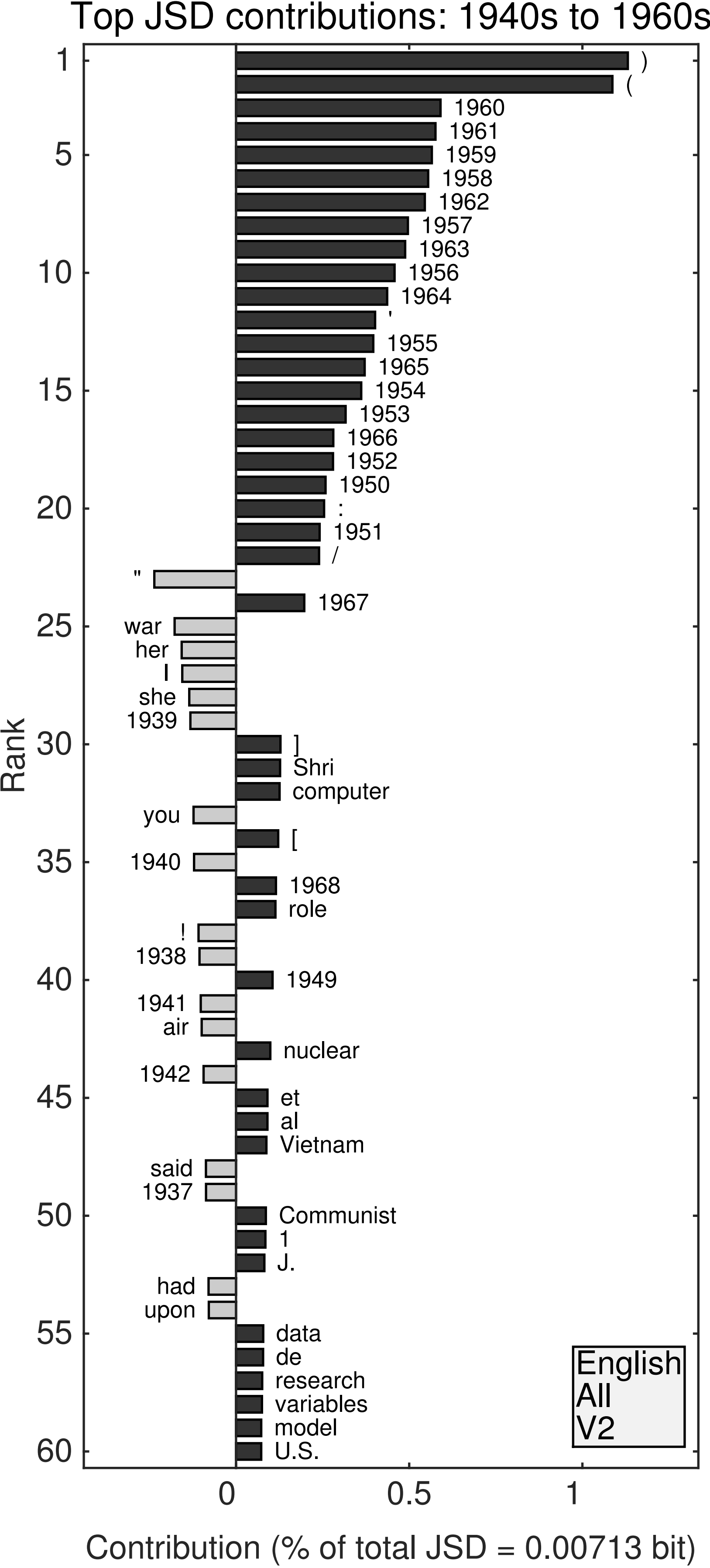

Top JSD contributions: 1940s to 1960s
Rank
Contribution (% of total JSD = 0.00713 bit)
English
All
V2
)
(
1960
1961
1959
1958
1962
1957
1963
1956
1964
'
1955
1965
1954
1953
1966
1952
1950
:
1951
/
"
1967
war
her
I
she
1939
]
Shri
computer
you
[
1940
1968
role
!
1938
1949
1941
air
nuclear
1942
et
al
Vietnam
said
1937
Communist
1
J.
had
upon
data
de
research
variables
model
U.S.

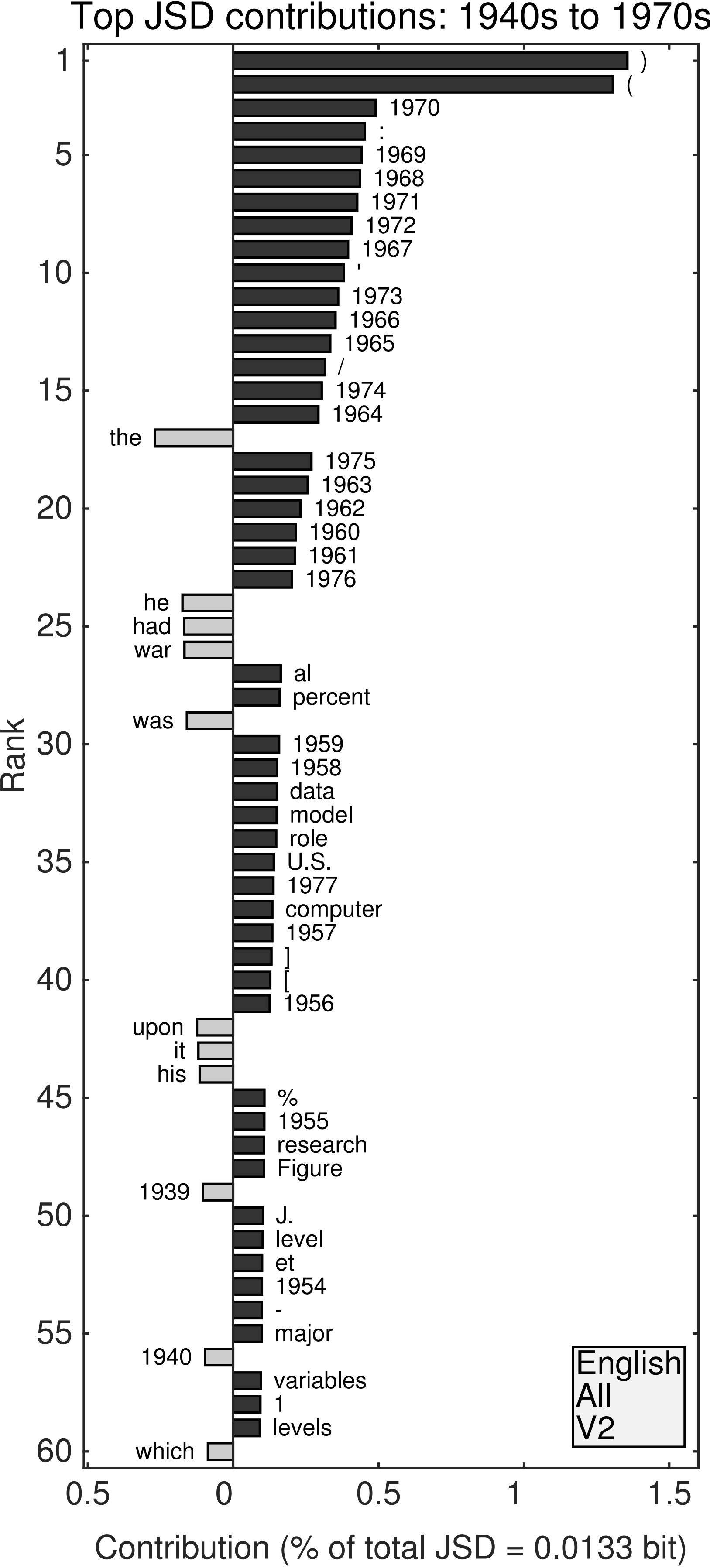

Top JSD contributions: 1940s to 1970s
Rank
Contribution (% of total JSD = 0.0133 bit)
)
(
1970
:
1969
1968
1971
1972
1967
'
1973
1966
1965
/
1974
1964
the
1975
1963
1962
1960
1961
1976
he
had
war
al
percent
was
1959
1958
data
model
role
U.S.
1977
computer
1957
]
[
1956
upon
it
his
%
1955
research
Figure
1939
J.
level
et
1954
-
major
1940
variables
1
levels
which
English
All
V2

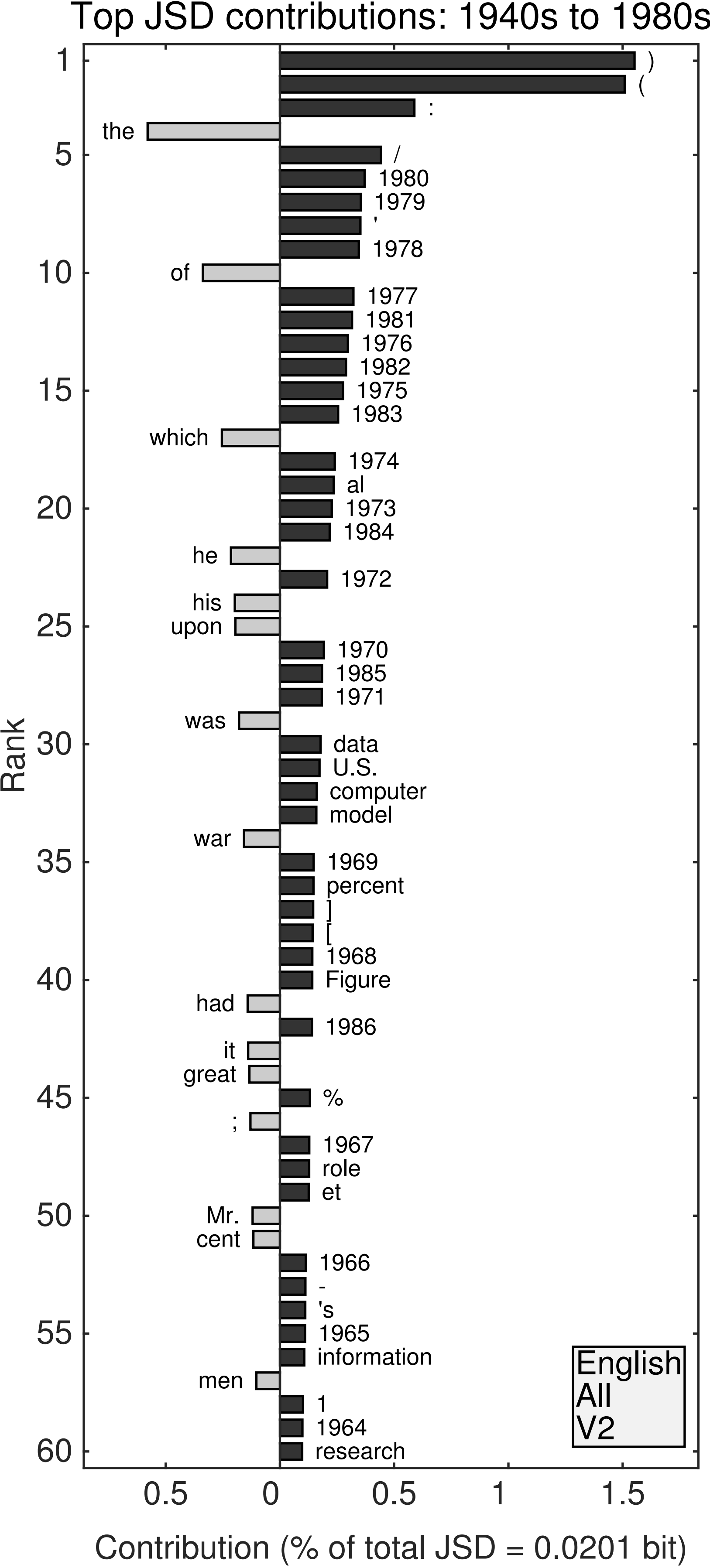
Top JSD contributions: 1940s to 1980s
Rank
Contribution (% of total JSD = 0.0201 bit)
0.5
0
0.5
1
1.5
1
5
10
15
20
25
30
35
40
45
50
55
60
)
(
:
the
/
1980
1979
'
1978
of
1977
1981
1976
1982
1975
1983
which
1974
al
1973
1984
he
1972
his
upon
1970
1985
1971
was
data
U.S.
computer
model
war
1969
percent
]
[
1968
Figure
had
1986
it
great
%
;
1967
role
et
Mr.
cent
1966
-
's
1965
information
men
1
1964
research
English
All
V2

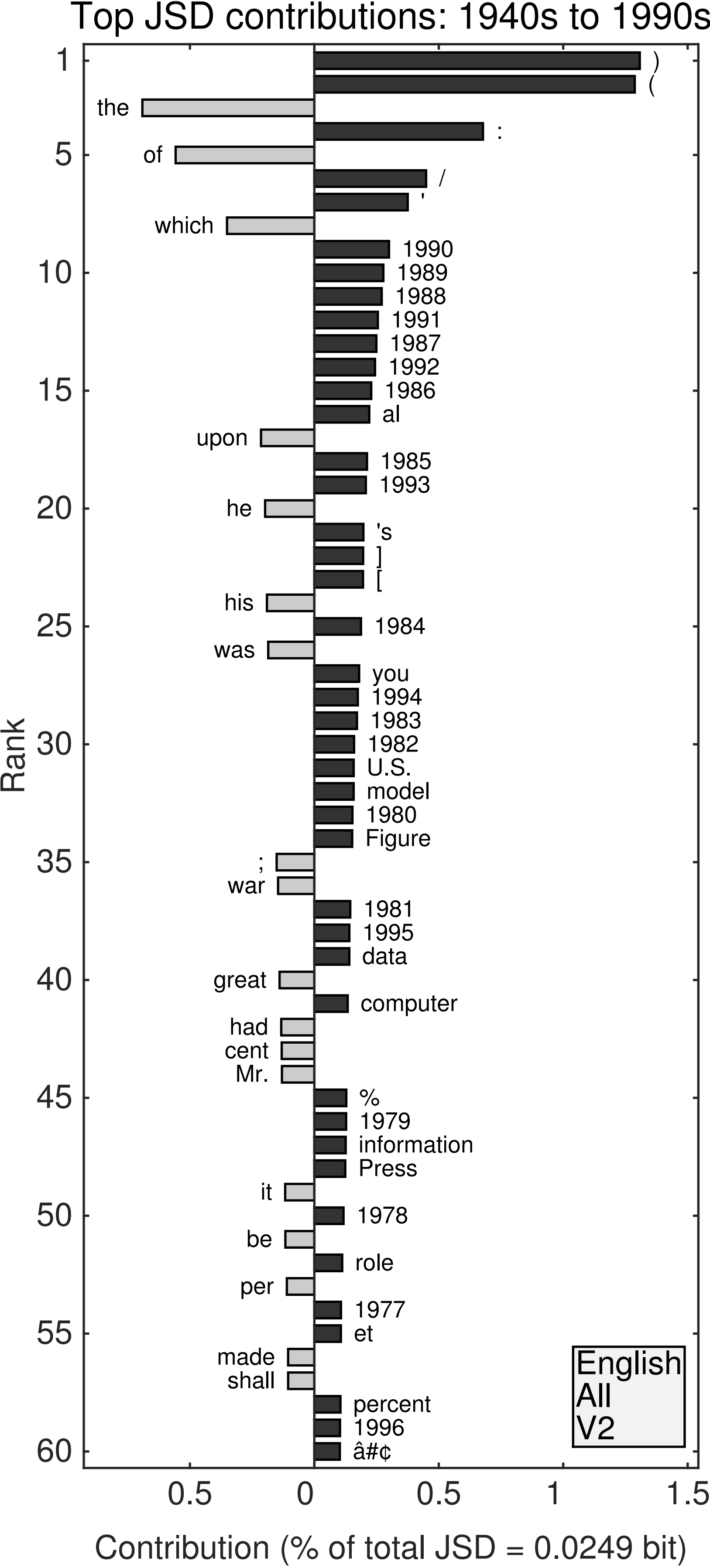

Top JSD contributions: 1940s to 1990s
Rank
Contribution (% of total JSD = 0.0249 bit)
)
(
the
:
of
/
'
which
1990
1989
1988
1991
1987
1992
1986
al
upon
1985
1993
he
's
]
[
his
1984
was
you
1994
1983
1982
U.S.
model
1980
Figure
;
war
1981
1995
data
great
computer
had
cent
Mr.
%
1979
information
Press
it
1978
be
role
per
1977
et
made
shall
percent
1996
â#¢
English
All
V2

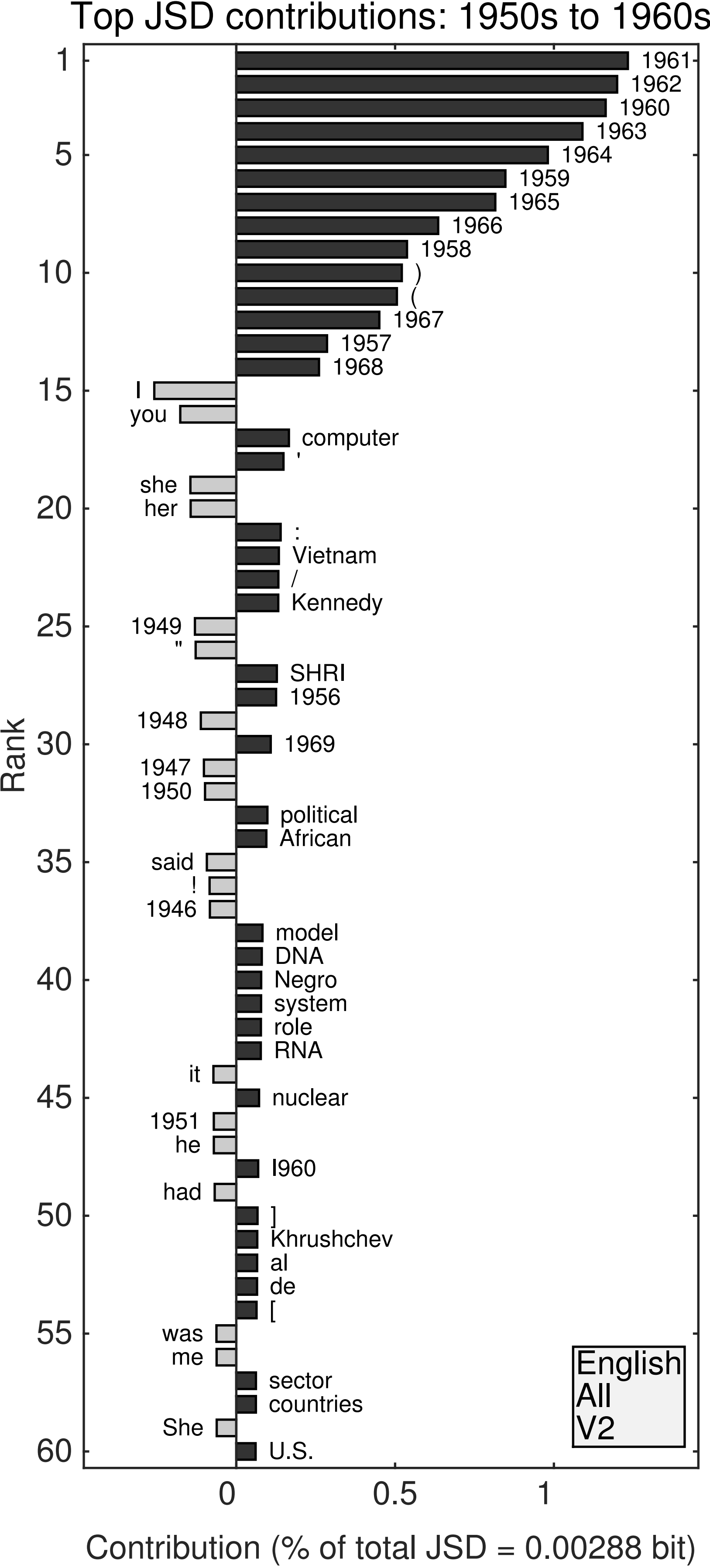
Top JSD contributions: 1950s to 1960s
Rank
1961
1962
1960
1963
1964
1959
1965
1966
1958
)
(
1967
1957
1968
I
you
computer
'
she
her
:
Vietnam
/
Kennedy
1949
"
SHRI
1956
1948
1969
1947
1950
political
African
said
!
1946
model
DNA
Negro
system
role
RNA
it
nuclear
1951
he
I960
had
]
Khrushchev
al
de
[
was
me
sector
countries
She
U.S.
English
All
V2
0
0.5
1
Contribution (% of total JSD = 0.00288 bit)

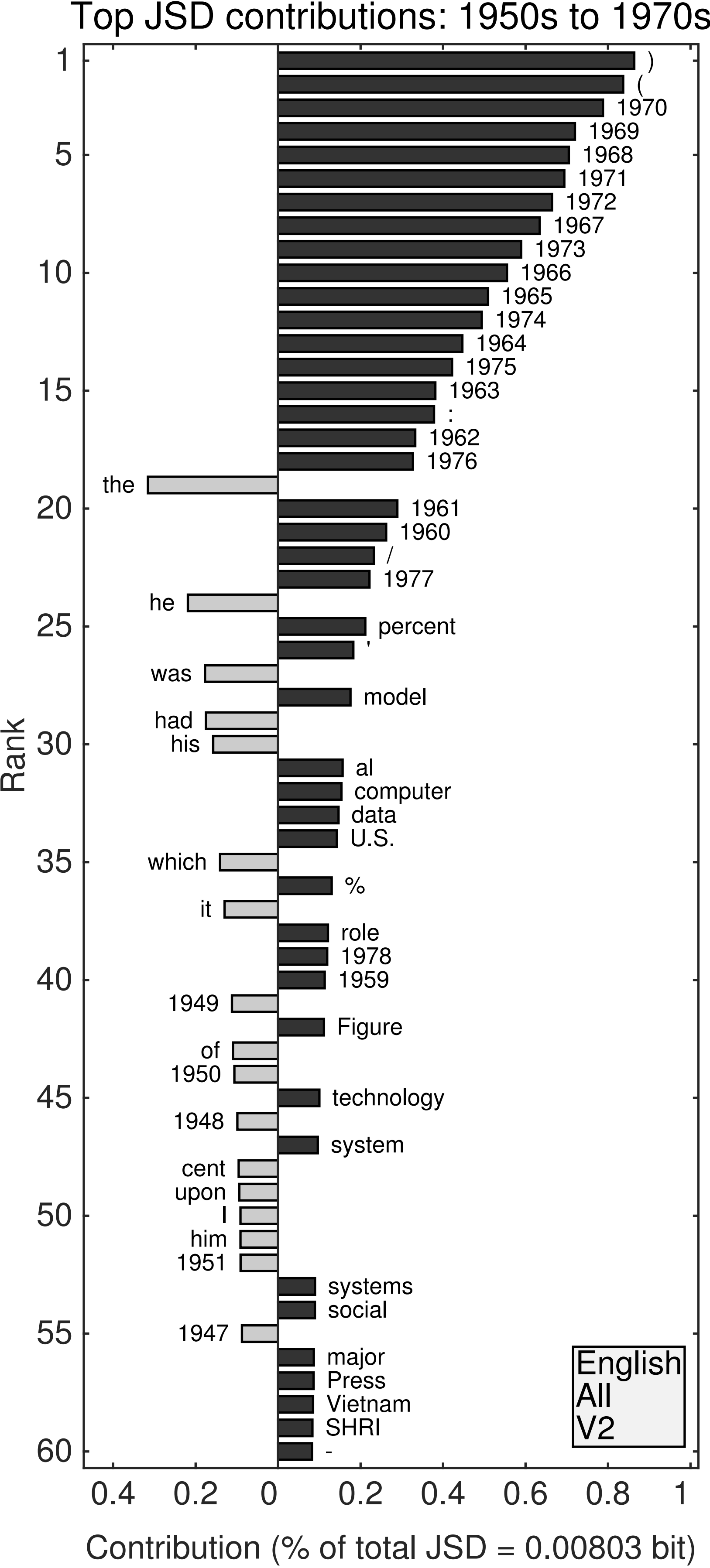
Top JSD contributions: 1950s to 1970s
Rank
Contribution (% of total JSD = 0.00803 bit)
English
All
V2
)
(
1970
1969
1968
1971
1972
1967
1973
1966
1965
1974
1964
1975
1963
:
1962
1976
the
1961
1960
/
1977
he
percent
'
was
model
had
his
al
computer
data
U.S.
which
%
it
role
1978
1959
1949
Figure
of
1950
technology
1948
system
cent
upon
I
him
1951
systems
social
1947
major
Press
Vietnam
SHRI
-

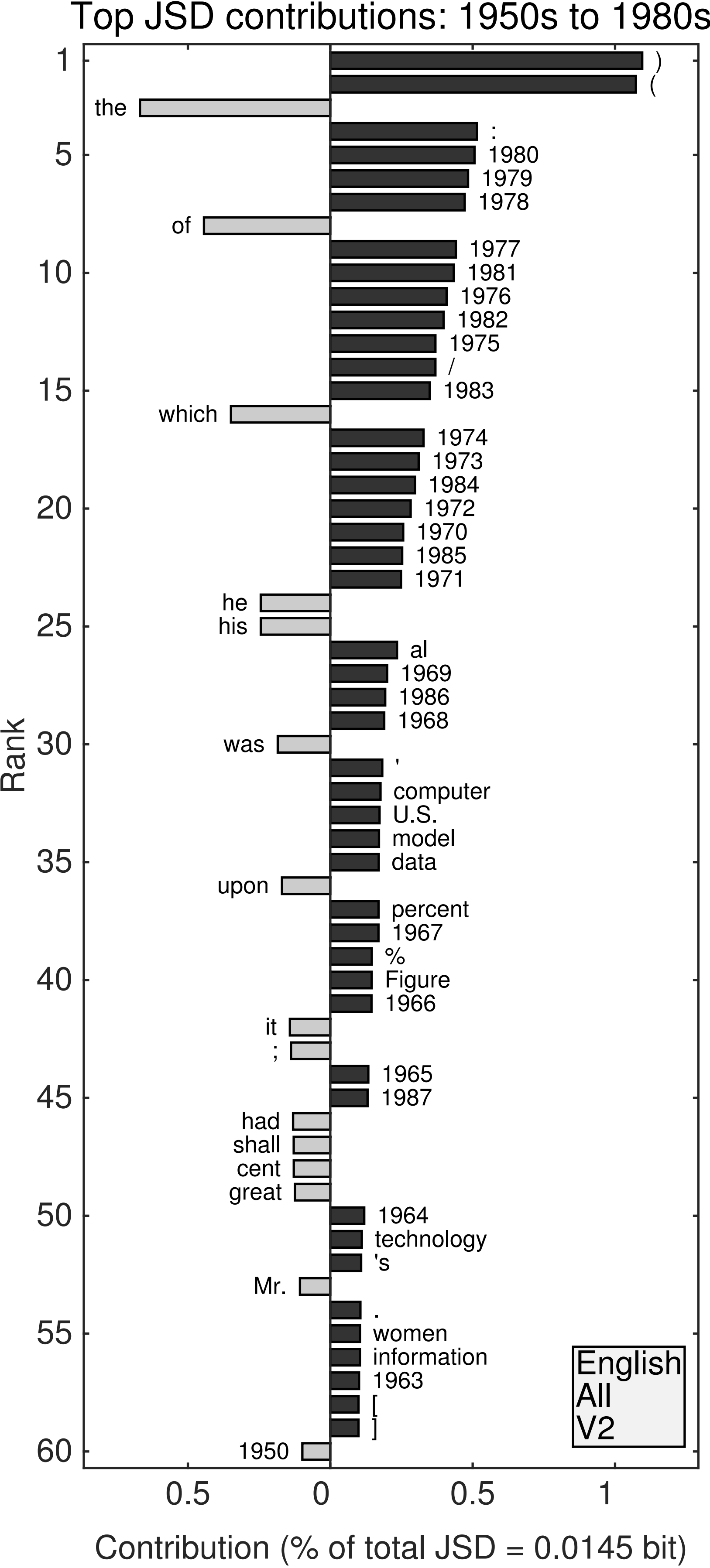

Top JSD contributions: 1950s to 1980s
Rank
1
5
10
15
20
25
30
35
40
45
50
55
60
)
(
the
:
1980
1979
1978
of
1977
1981
1976
1982
1975
/
1983
which
1974
1973
1984
1972
1970
1985
1971
he
his
al
1969
1986
1968
was
'
computer
U.S.
model
data
upon
percent
1967
%
Figure
1966
it
;
1965
1987
had
shall
cent
great
1964
technology
's
Mr.
.
women
information
1963
[
]
1950
English
All
V2
0.5
0
0.5
1
Contribution (% of total JSD = 0.0145 bit)

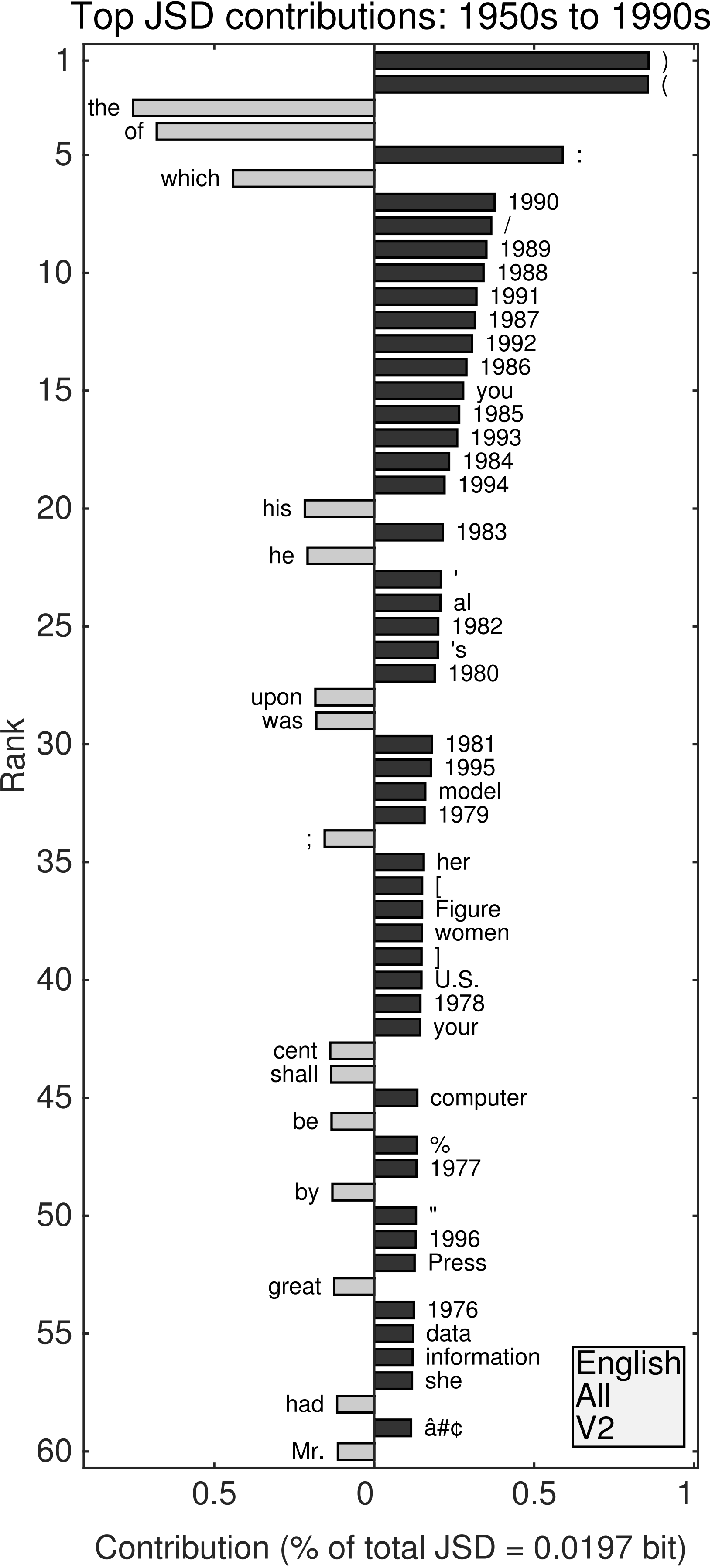
Top JSD contributions: 1950s to 1990s
Rank
Contribution (% of total JSD = 0.0197 bit)
)
(
the
of
:
which
1990
/
1989
1988
1991
1987
1992
1986
you
1985
1993
1984
1994
his
1983
he
'
al
1982
's
1980
upon
was
1981
1995
model
1979
;
her
[
Figure
women
]
U.S.
1978
your
cent
shall
computer
be
%
1977
by
"
1996
Press
great
1976
data
information
she
had
â#¢
Mr.
English
All
V2

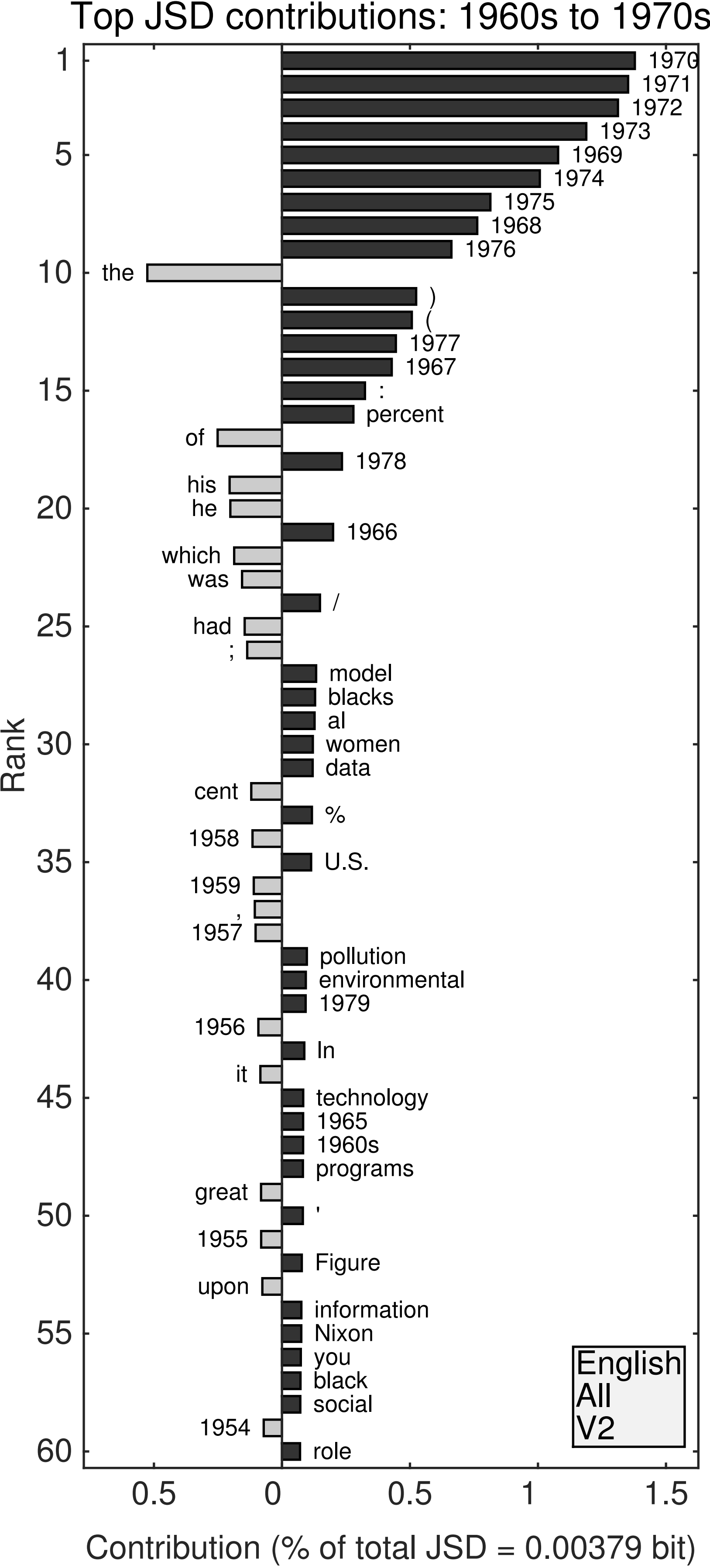

Top JSD contributions: 1960s to 1970s
Rank
Contribution (% of total JSD = 0.00379 bit)
1970
1971
1972
1973
1969
1974
1975
1968
1976
the
)
(
1977
1967
:
percent
of
1978
his
he
1966
which
was
/
had
;
model
blacks
al
women
data
cent
%
1958
U.S.
1959
,
1957
pollution
environmental
1979
1956
In
it
technology
1965
1960s
programs
great
'
1955
Figure
upon
information
Nixon
you
black
social
1954
role
English
All
V2

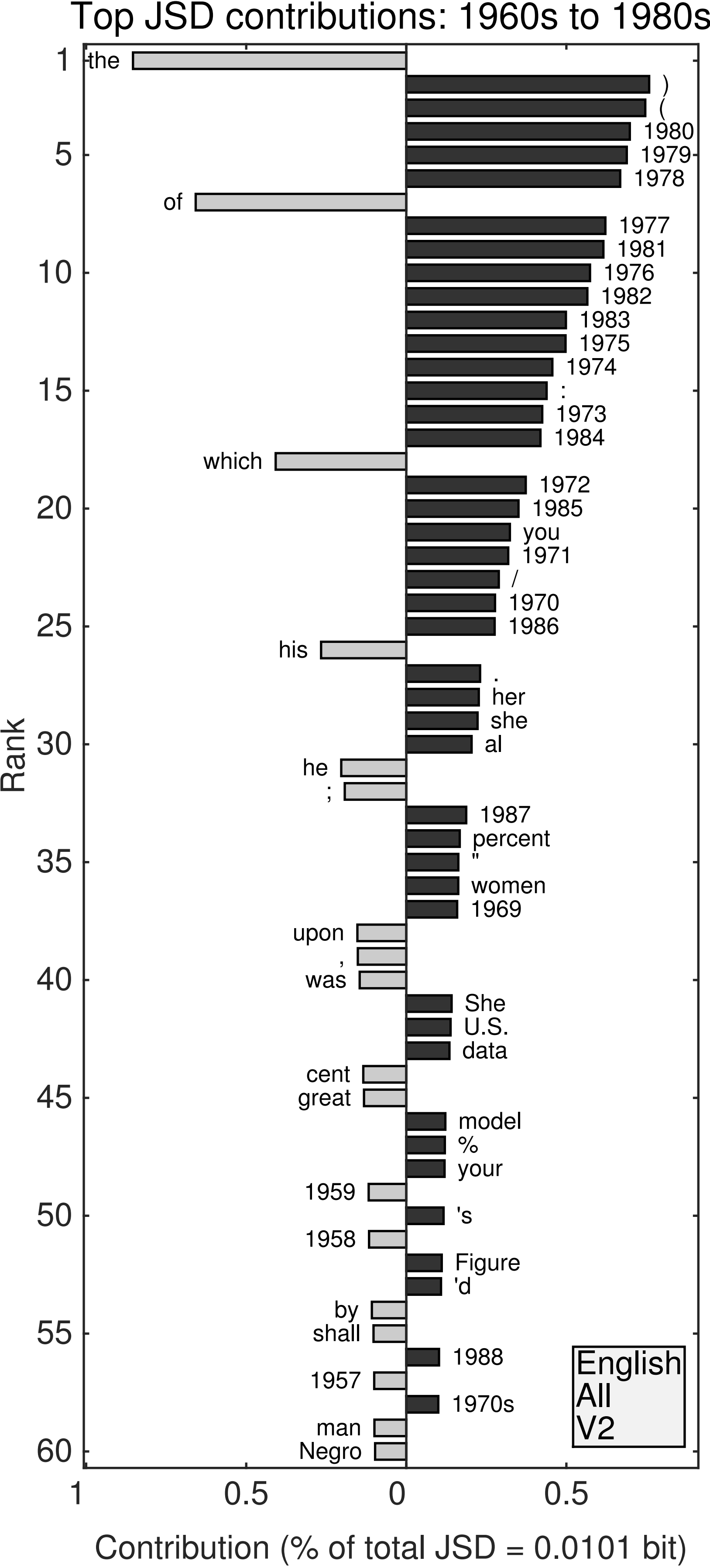
Top JSD contributions: 1960s to 1980s
Rank
the
)
(
1980
1979
1978
of
1977
1981
1976
1982
1983
1975
1974
:
1973
1984
which
1972
1985
you
1971
/
1970
1986
his
.
her
she
al
he
;
1987
percent
"
women
1969
upon
,
was
She
U.S.
data
cent
great
model
%
your
1959
's
1958
Figure
'd
by
shall
1988
1957
1970s
man
Negro
English
All
V2
1
0.5
0
0.5
Contribution (% of total JSD = 0.0101 bit)

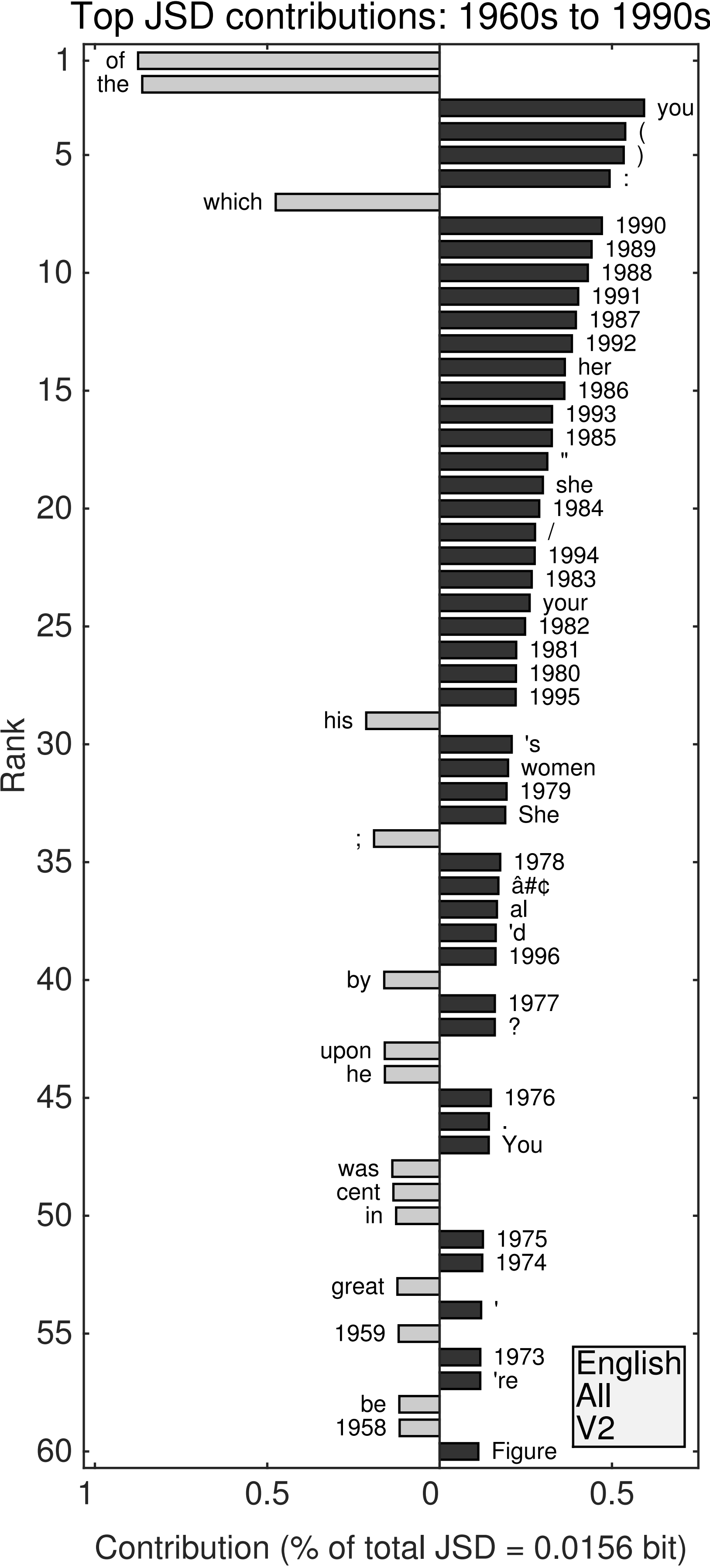
Top JSD contributions: 1960s to 1990s
Rank
Contribution (% of total JSD = 0.0156 bit)
English
All
V2
of
the
you
(
)
:
which
1990
1989
1988
1991
1987
1992
her
1986
1993
1985
"
she
1984
/
1994
1983
your
1982
1981
1980
1995
his
's
women
1979
She
;
1978
â#¢
al
'd
1996
by
1977
?
upon
he
1976
.
You
was
cent
in
1975
1974
great
'
1959
1973
're
be
1958
Figure

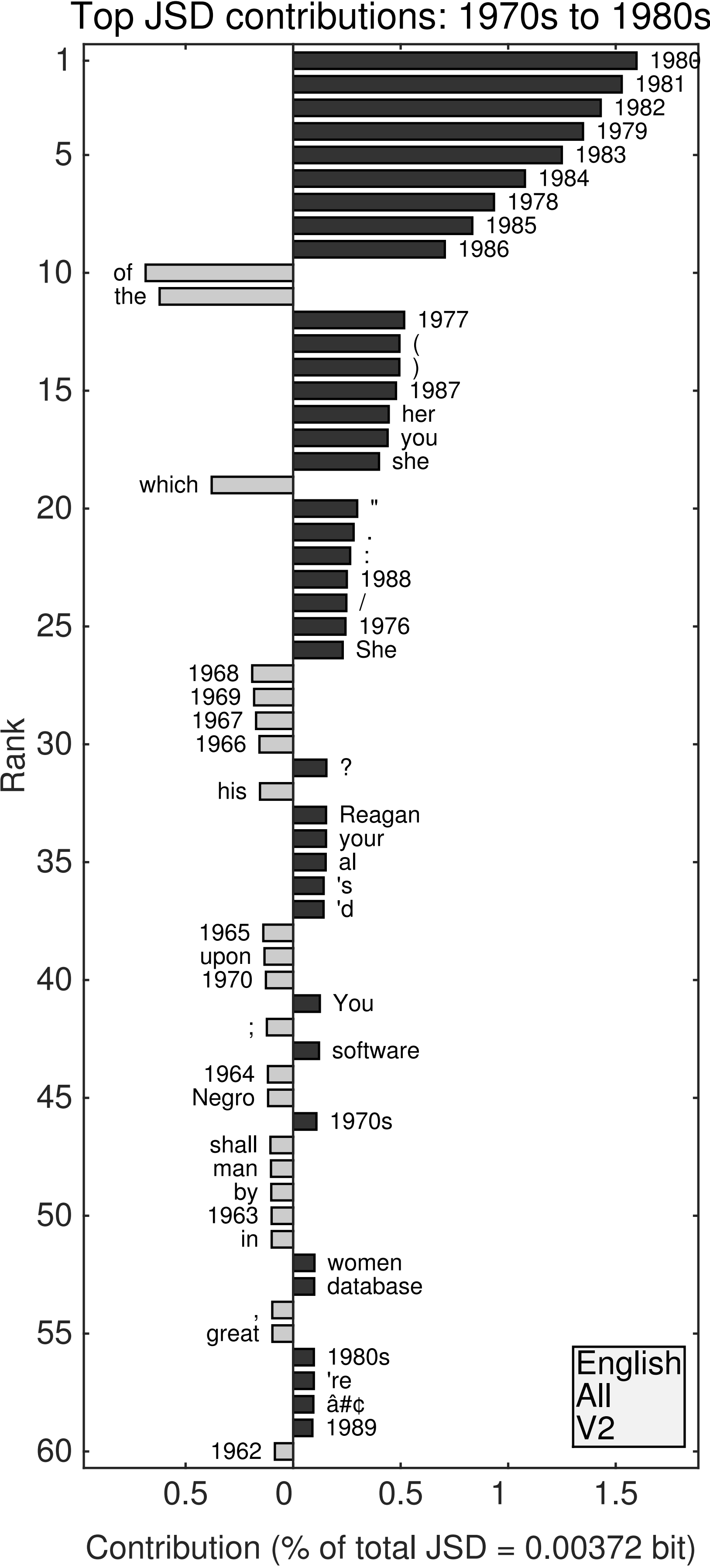

Top JSD contributions: 1970s to 1980s
Rank
Contribution (% of total JSD = 0.00372 bit)
0.5
0
0.5
1
1.5
1
5
10
15
20
25
30
35
40
45
50
55
60
1980
1981
1982
1979
1983
1984
1978
1985
1986
of
the
1977
(
)
1987
her
you
she
which
"
.
:
1988
/
1976
She
1968
1969
1967
1966
?
his
Reagan
your
al
's
'd
1965
upon
1970
You
;
software
1964
Negro
1970s
shall
man
by
1963
in
women
database
,
great
1980s
're
â#¢
1989
1962
English
All
V2

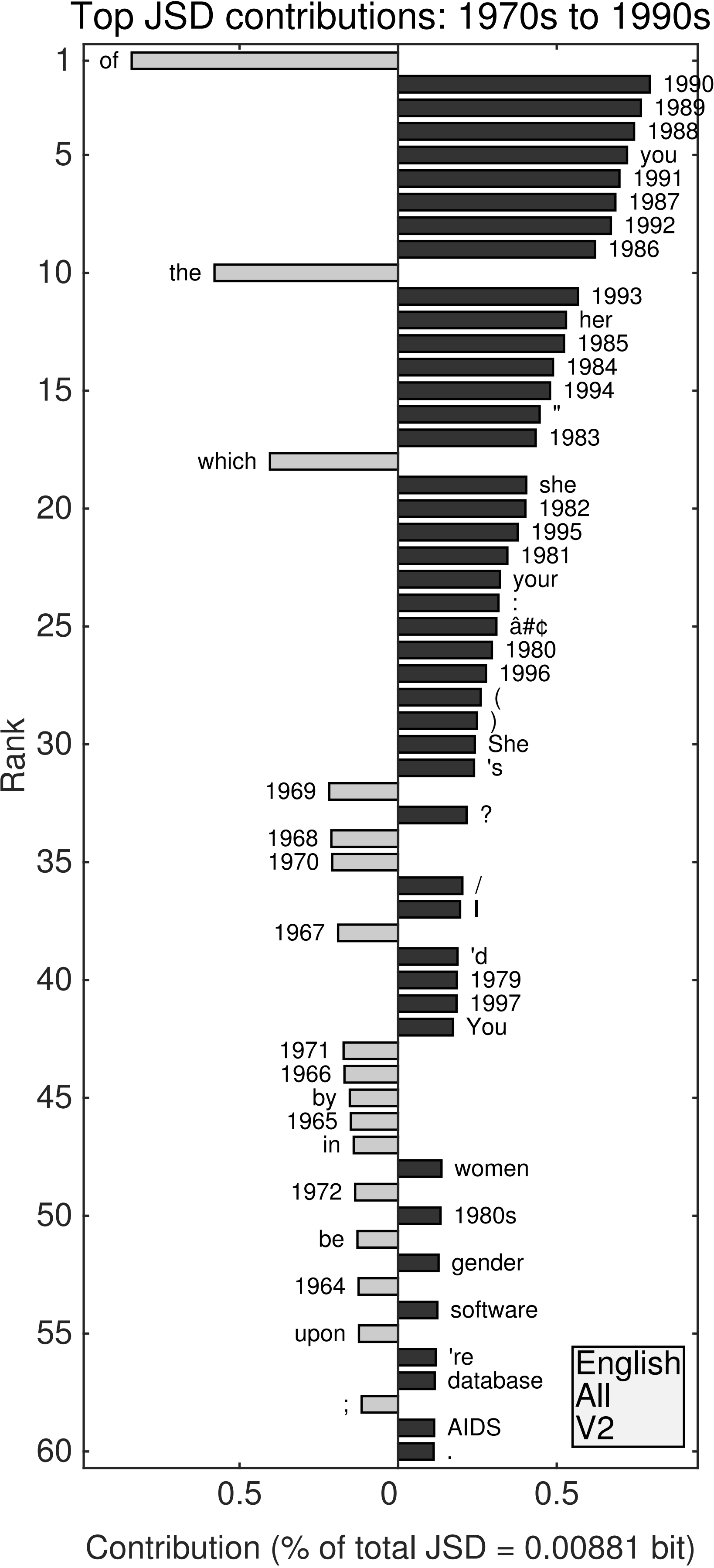

Top JSD contributions: 1970s to 1990s
Rank
Contribution (% of total JSD = 0.00881 bit)
of
1990
1989
1988
you
1991
1987
1992
1986
the
1993
her
1985
1984
1994
"
1983
which
she
1982
1995
1981
your
:
â#¢
1980
1996
(
)
She
's
1969
?
1968
1970
/
I
1967
'd
1979
1997
You
1971
1966
by
1965
in
women
1972
1980s
be
gender
1964
software
upon
're
database
;
AIDS
.
English
All
V2

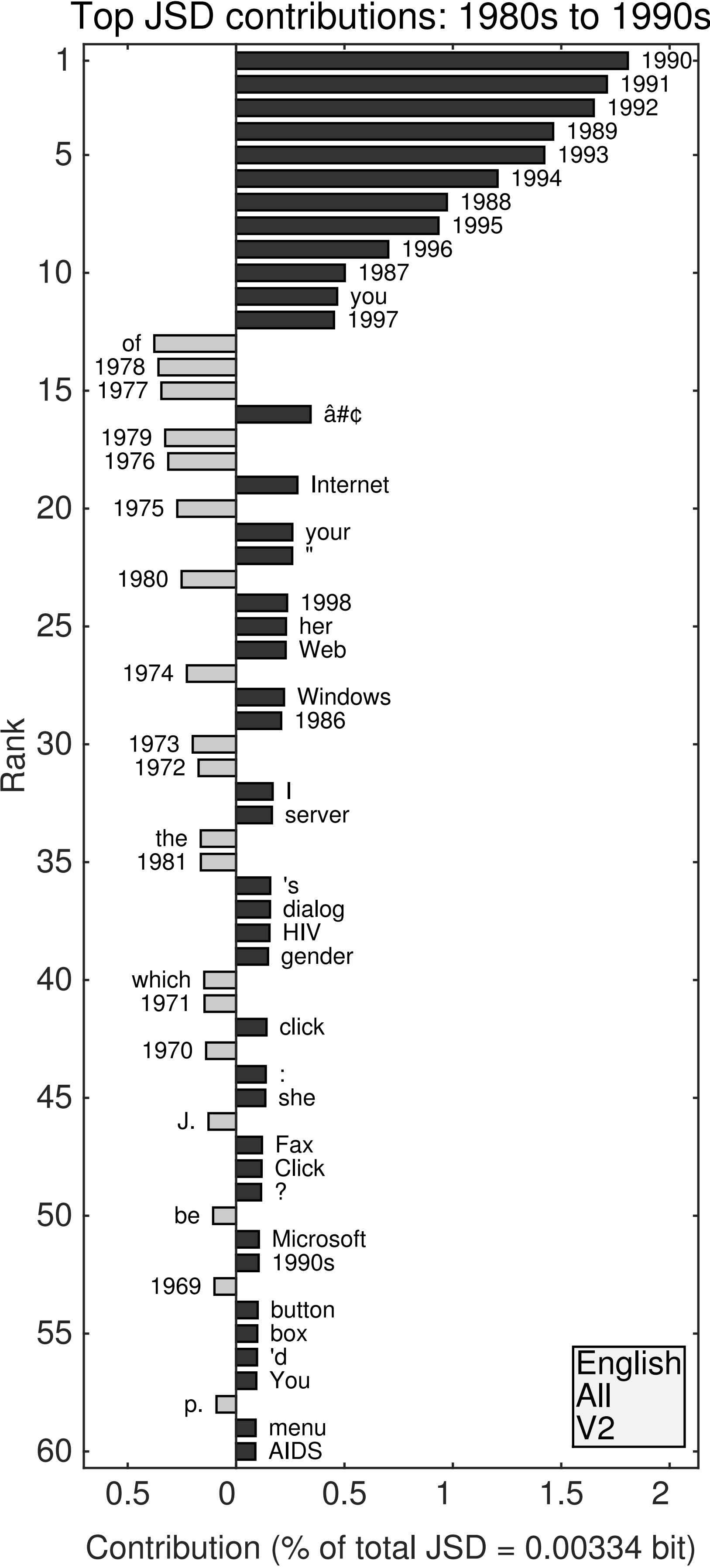

Top JSD contributions: 1980s to 1990s
Rank
Contribution (% of total JSD = 0.00334 bit)
1990
1991
1992
1989
1993
1994
1988
1995
1996
1987
you
1997
of
1978
1977
â#¢
1979
1976
Internet
1975
your
"
1980
1998
her
Web
1974
Windows
1986
1973
1972
I
server
the
1981
's
dialog
HIV
gender
which
1971
click
1970
:
she
J.
Fax
Click
?
be
Microsoft
1990s
1969
button
box
'd
You
p.
menu
AIDS
English
All
V2
