# Supplementary material for: Characterizing the Google Books Corpus: Strong Limits to Inferences of Socio-Cultural and Linguistic Evolution
Source: PLoS One. 2015 Oct 7;10(10):e0137041. doi: 10.1371/journal.pone.0137041 (PMC4596490; doi:10.1371/journal.pone.0137041)
Supplement: S2 File — (PDF) [file pone.0137041.s002.pdf]

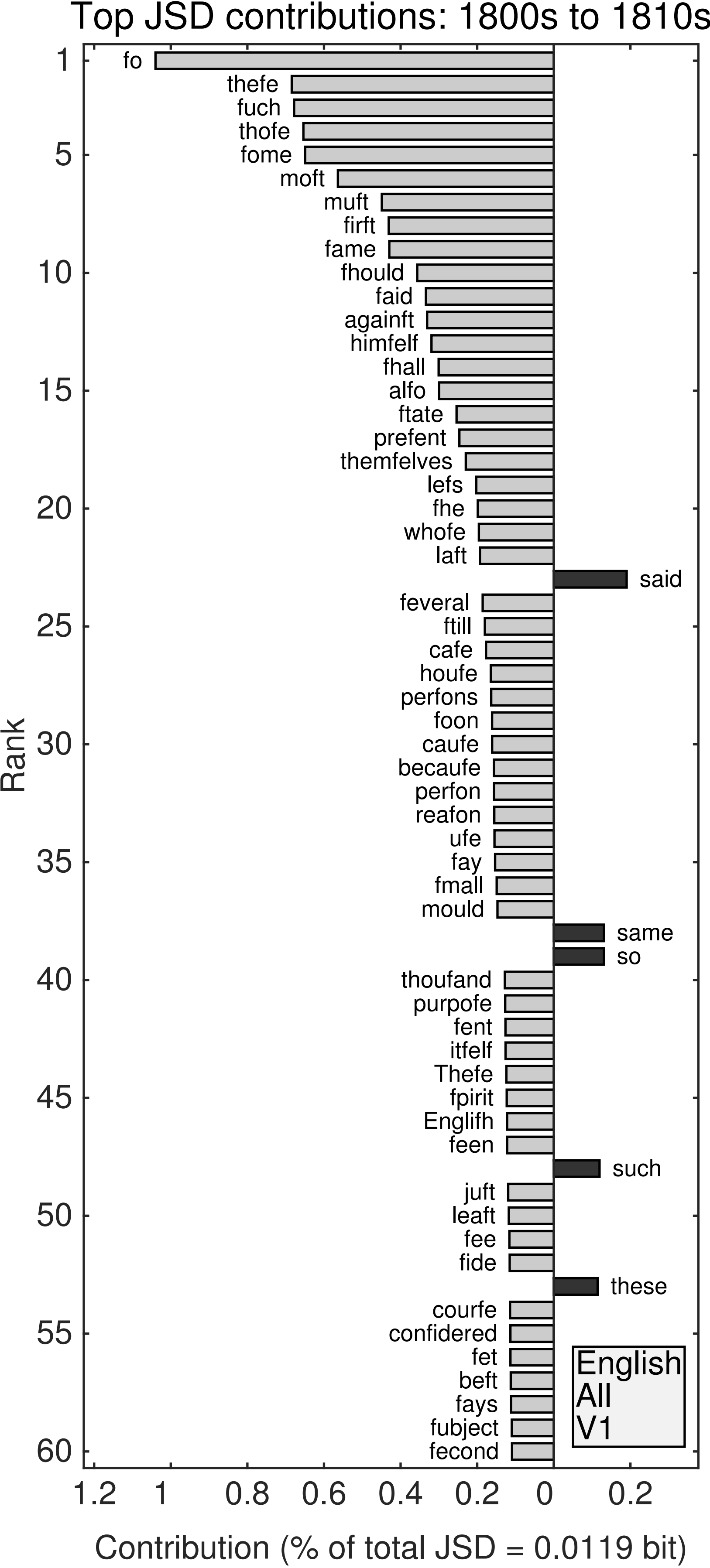

Top JSD contributions: 1800s to 1810s
Rank
fo
thefe
fuch
thofe
fome
moft
muft
firft
fame
fhould
faid
againft
himfelf
fhall
alfo
ftate
prefent
themfelves
lefs
fhe
whofe
laft
said
feveral
ftill
cafe
houfe
perfons
foon
caufe
becaufe
perfon
reafon
ufe
fay
fmall
mould
same
so
thoufand
purpofe
fent
itfelf
Thefe
fpirit
Englifh
feen
such
juft
leaft
fee
fide
these
courfe
confidered
fet
beft
fays
fubject
fecond
English
All
V1
Contribution (% of total JSD = 0.0119 bit)

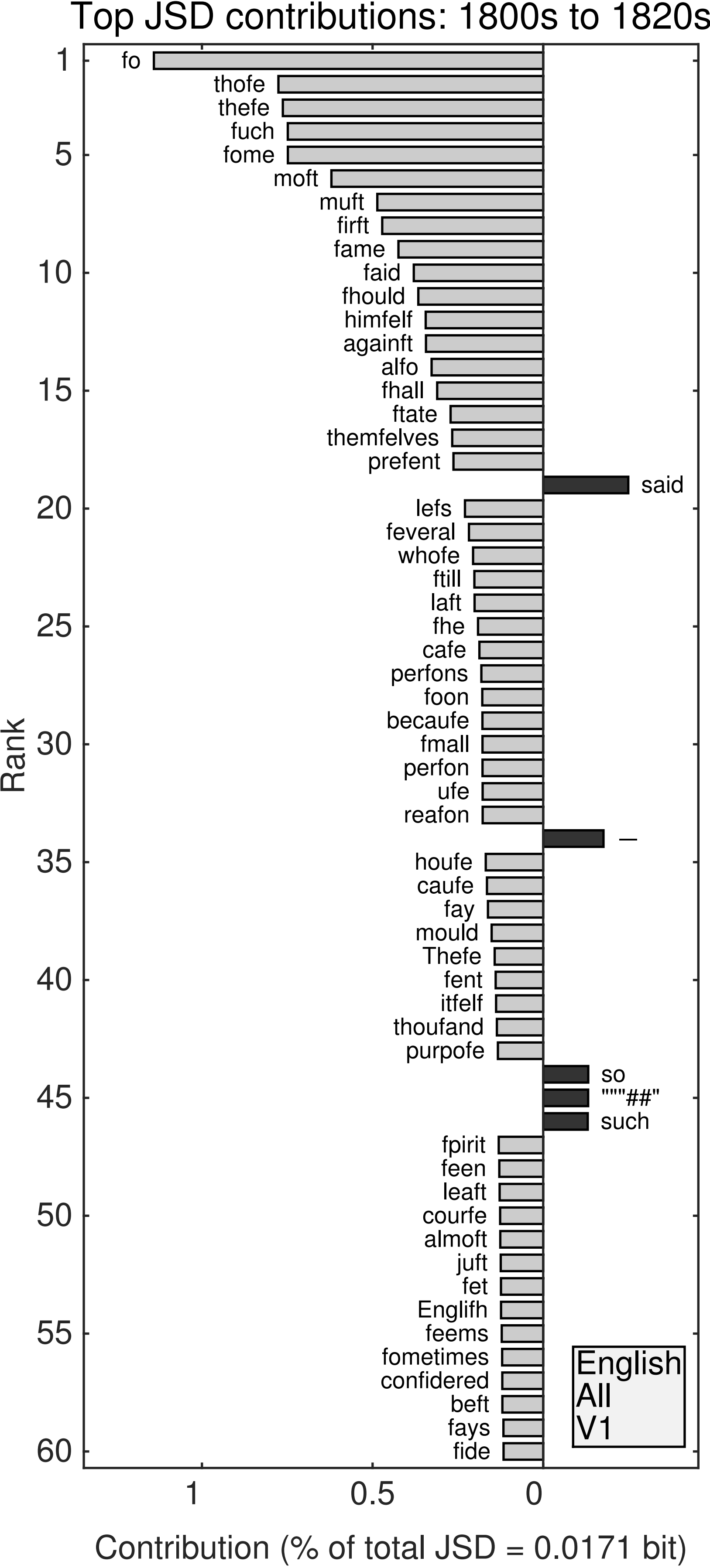

Top JSD contributions: 1800s to 1820s
Rank
Contribution (% of total JSD = 0.0171 bit)
fo
thofe
thefe
fuch
fome
moft
muft
firft
fame
faid
fhould
himfelf
againft
alfo
fhall
ftate
themfelves
prefent
said
lefs
feveral
whofe
ftill
laft
fhe
cafe
perfons
foon
becaufe
fmall
perfon
ufe
reafon
—
houfe
caufe
fay
mould
Thefe
fent
itfelf
thoufand
purpofe
so
"""##"
such
fpirit
feen
leaft
courfe
almoft
juft
fet
Englifh
feems
fometimes
confidered
beft
fays
fide
English
All
V1

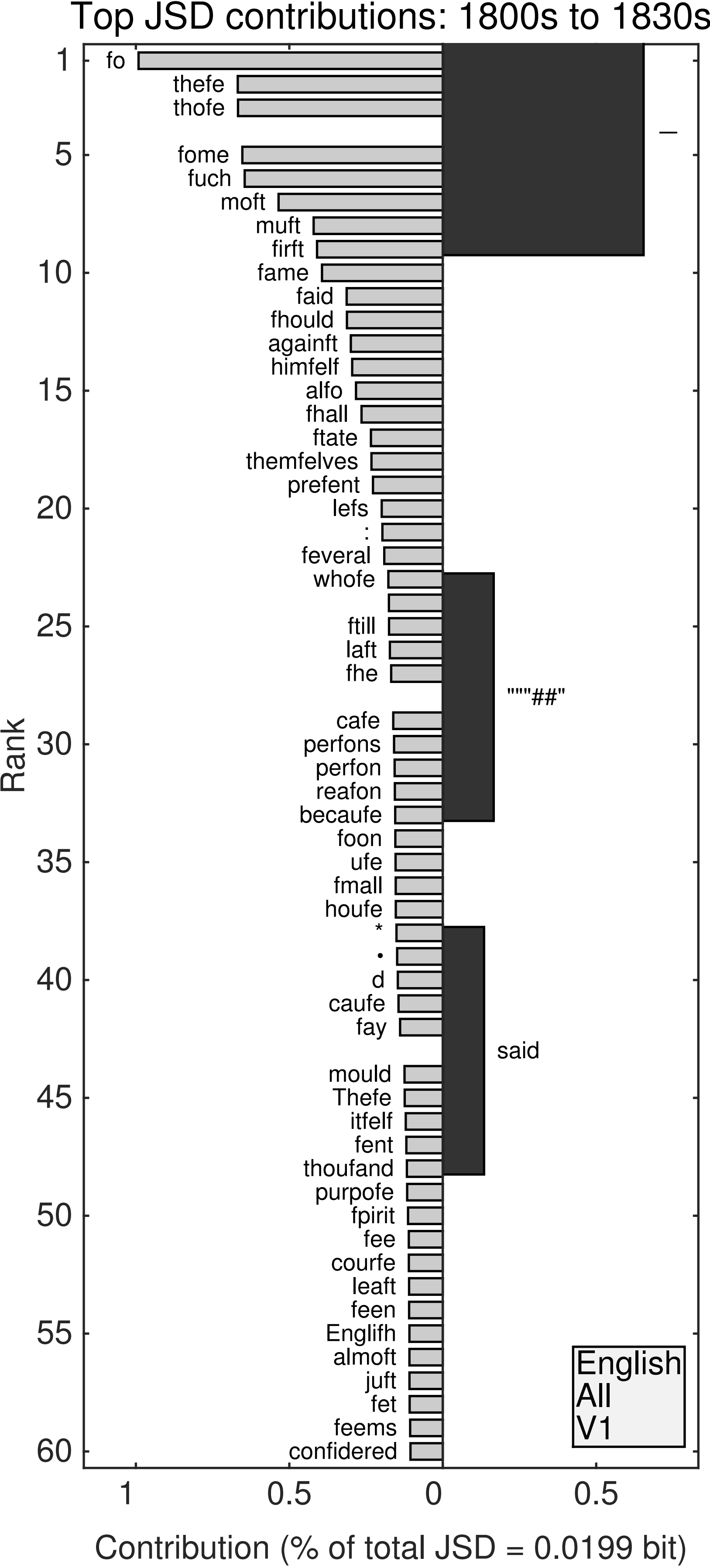

Top JSD contributions: 1800s to 1830s
Rank
fo
thefe
thofe
fome
fuch
moft
muft
firft
fame
faid
fhould
againft
himfelf
alfo
fhall
ftate
themfelves
prefent
lefs
:
feveral
whofe
ftill
laft
fhe
cafe
perfons
perfon
reafon
becaufe
foon
ufe
fmall
houfe
*
•
d
caufe
fay
mould
Thefe
itfelf
fent
thoufand
purpofe
fpirit
fee
courfe
leaft
feen
Englifh
almoft
juft
fet
feems
confidered
—
"""##"
said
English
All
V1
1
5
10
15
20
25
30
35
40
45
50
55
60
1
0.5
0
0.5
Contribution (% of total JSD = 0.0199 bit)

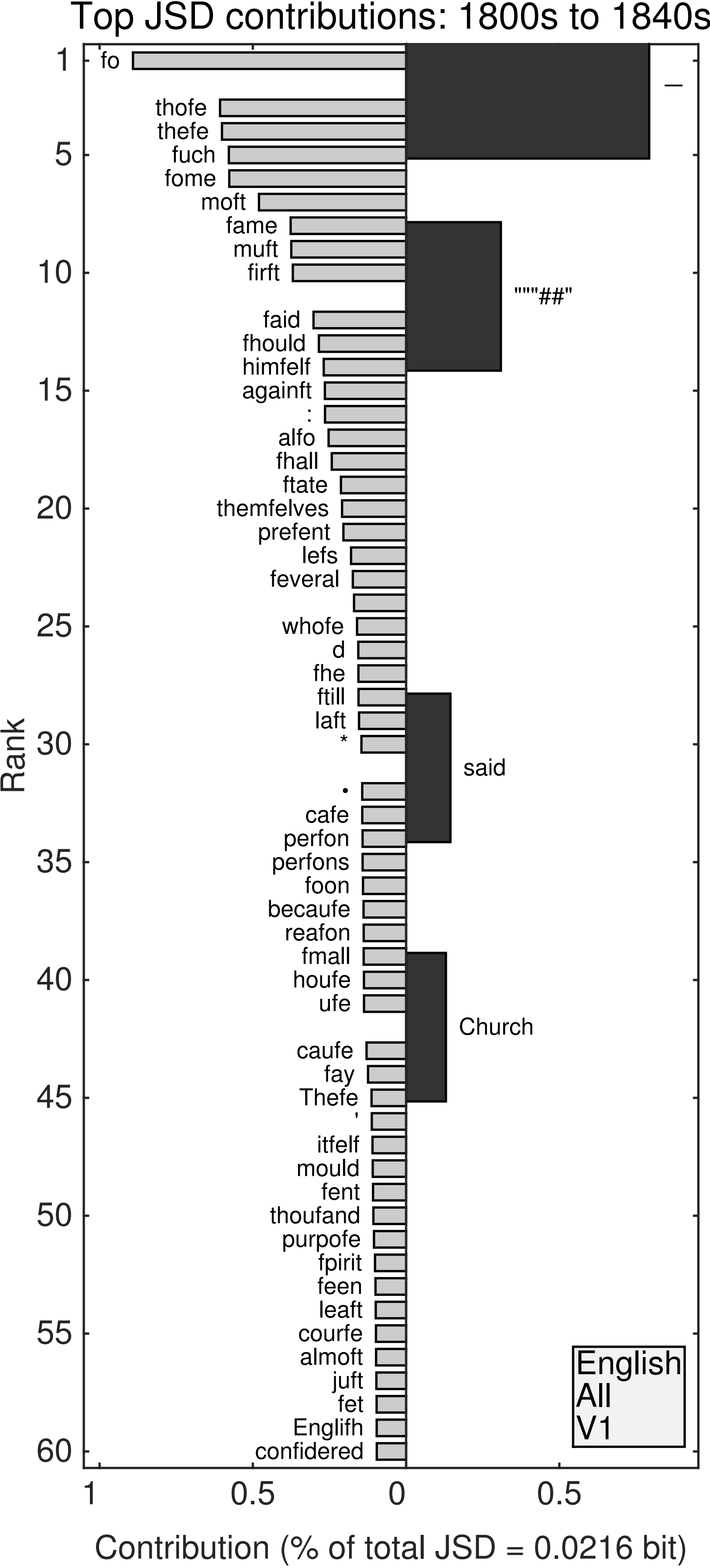

Top JSD contributions: 1800s to 1840s
Rank
Contribution (% of total JSD = 0.0216 bit)
1
5
10
15
20
25
30
35
40
45
50
55
60
1
0.5
0
0.5
fo
thofe
thefe
fuch
fome
moft
fame
muft
firft
faid
fhould
himfelf
againft
:
alfo
fhall
ftate
themfelves
prefent
lefs
feveral
whofe
d
fhe
ftill
laft
*
•
cafe
perfon
perfons
foon
becaufe
reafon
fmall
houfe
ufe
caufe
fay
Thefe
'
itfelf
mould
fent
thoufand
purpofe
fpirit
feen
leaft
courfe
almoft
juft
fet
Englifh
confidered
—
"""##"
said
Church
English
All
V1

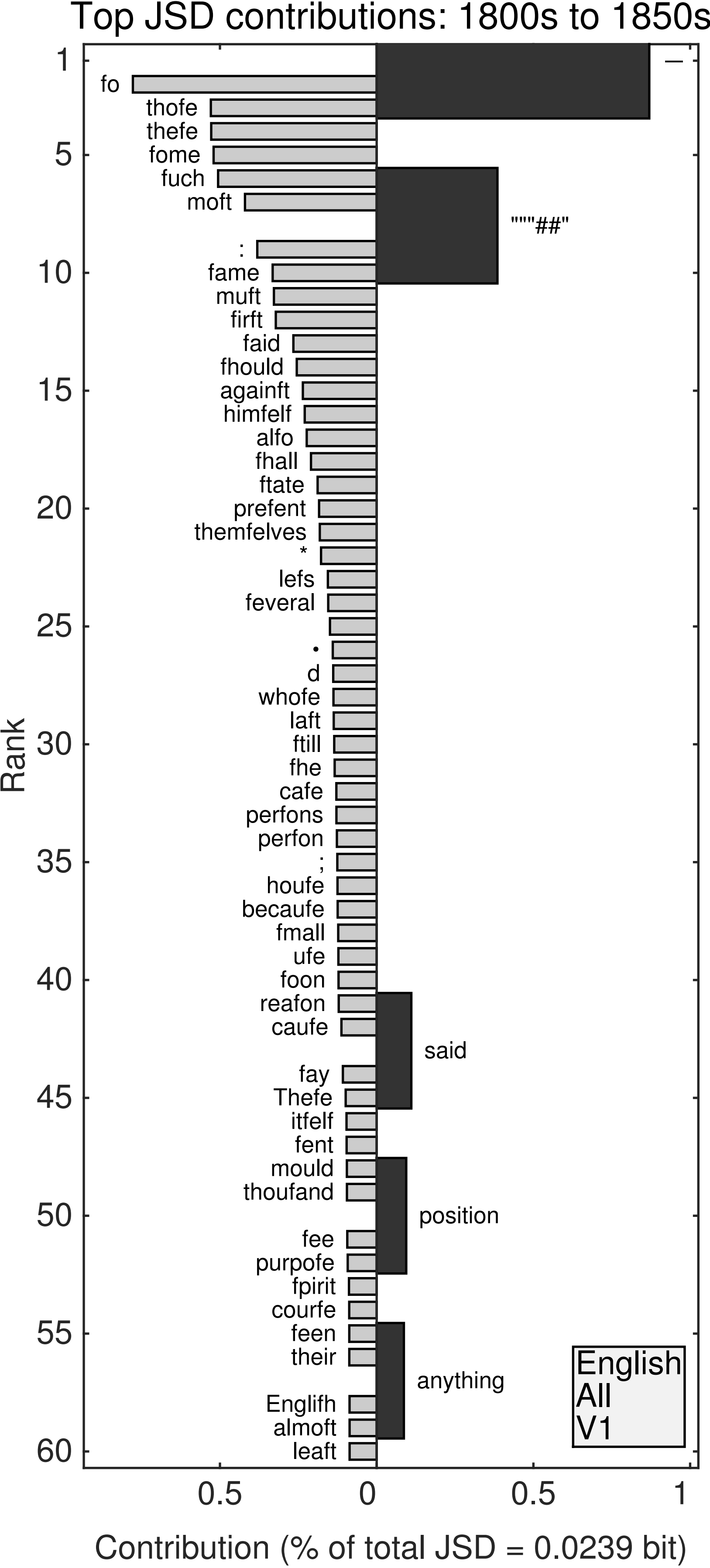

Top JSD contributions: 1800s to 1850s
Rank
fo
thofe
thefe
fome
fuch
moft
:
fame
muft
firft
faid
fhould
againft
himfelf
alfo
fhall
ftate
prefent
themfelves
*
lefs
feveral
•
d
whofe
laft
ftill
fhe
cafe
perfons
perfon
;
houfe
becaufe
fmall
ufe
foon
reafon
caufe
fay
Thefe
itfelf
fent
mould
thoufand
fee
purpofe
fpirit
courfe
feen
their
Englifh
almoft
leaft
"""##"
said
position
anything
English
All
V1
Contribution (% of total JSD = 0.0239 bit)

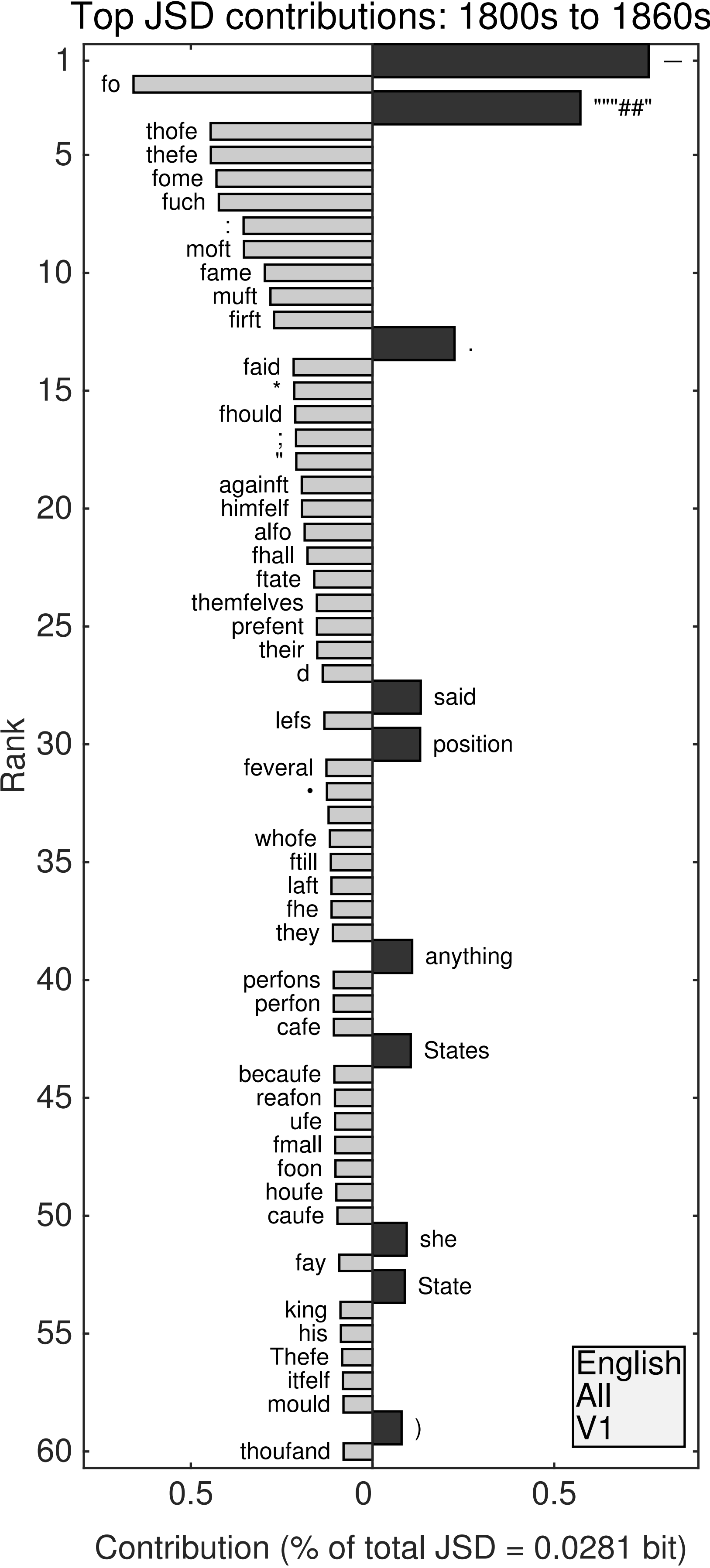
Top JSD contributions: 1800s to 1860s
Rank
Contribution (% of total JSD = 0.0281 bit)
—
fo
"""##"
thofe
thefe
fome
fuch
:
moft
fame
muft
firft
.
faid
*
fhould
;
"
againft
himfelf
alfo
fhall
ftate
themfelves
prefent
their
d
said
lefs
position
feveral
•
whofe
ftill
laft
fhe
they
anything
perfons
perfon
cafe
States
becaufe
reafon
ufe
fmall
foon
houfe
caufe
she
fay
State
king
his
Thefe
itfelf
mould
)
thoufand
English
All
V1

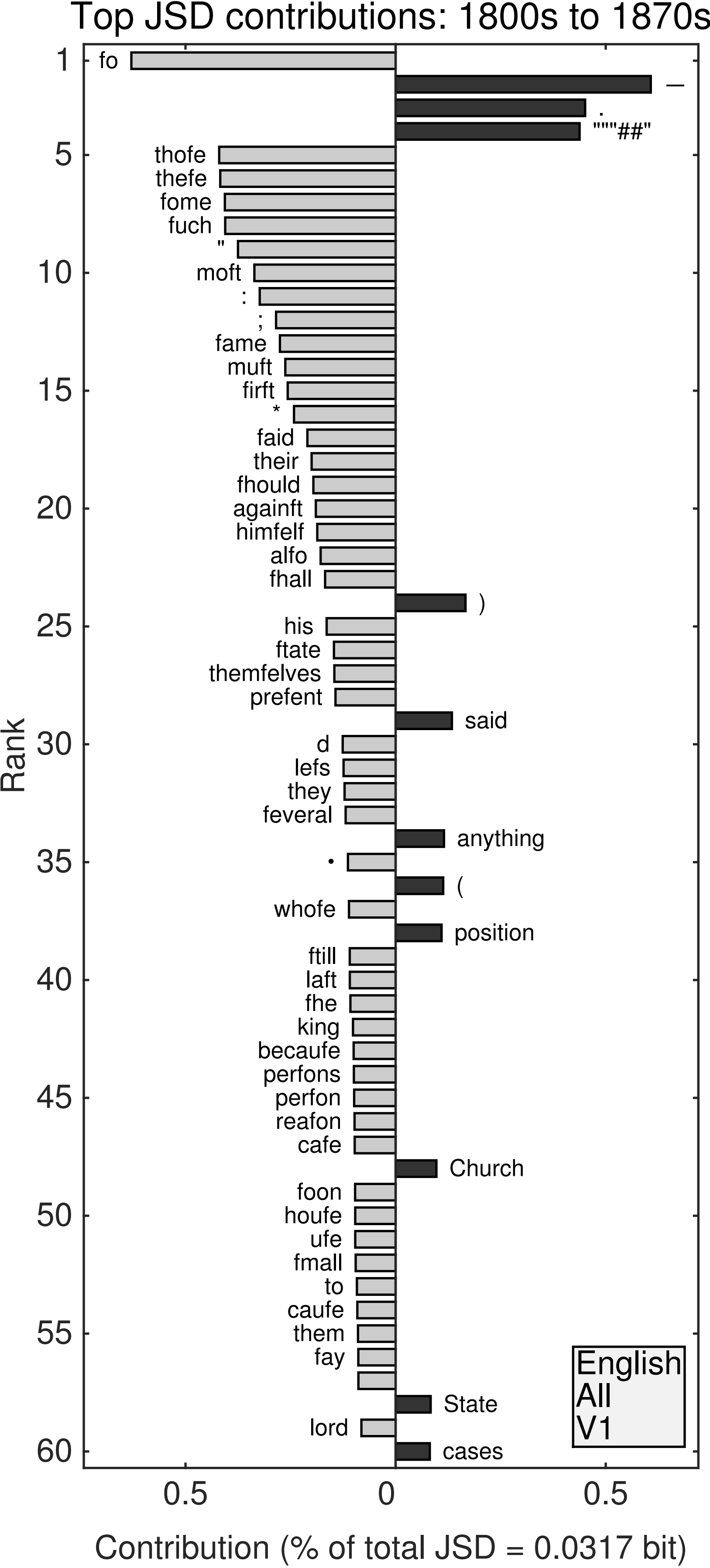
Top JSD contributions: 1800s to 1870s
Rank
Contribution (% of total JSD = 0.0317 bit)
0.5
0
0.5
1
5
10
15
20
25
30
35
40
45
50
55
60
fo
—
.
"""##"
thofe
thefe
fome
fuch
"
moft
:
;
fame
muft
firft
*
faid
their
fhould
againft
himfelf
alfo
fhall
)
his
ftate
themfelves
prefent
said
d
lefs
they
feveral
anything
•
(
whofe
position
ftill
laft
fhe
king
becaufe
perfons
perfon
reafon
cafe
Church
foon
houfe
ufe
fmall
to
caufe
them
fay
State
lord
cases
English
All
V1

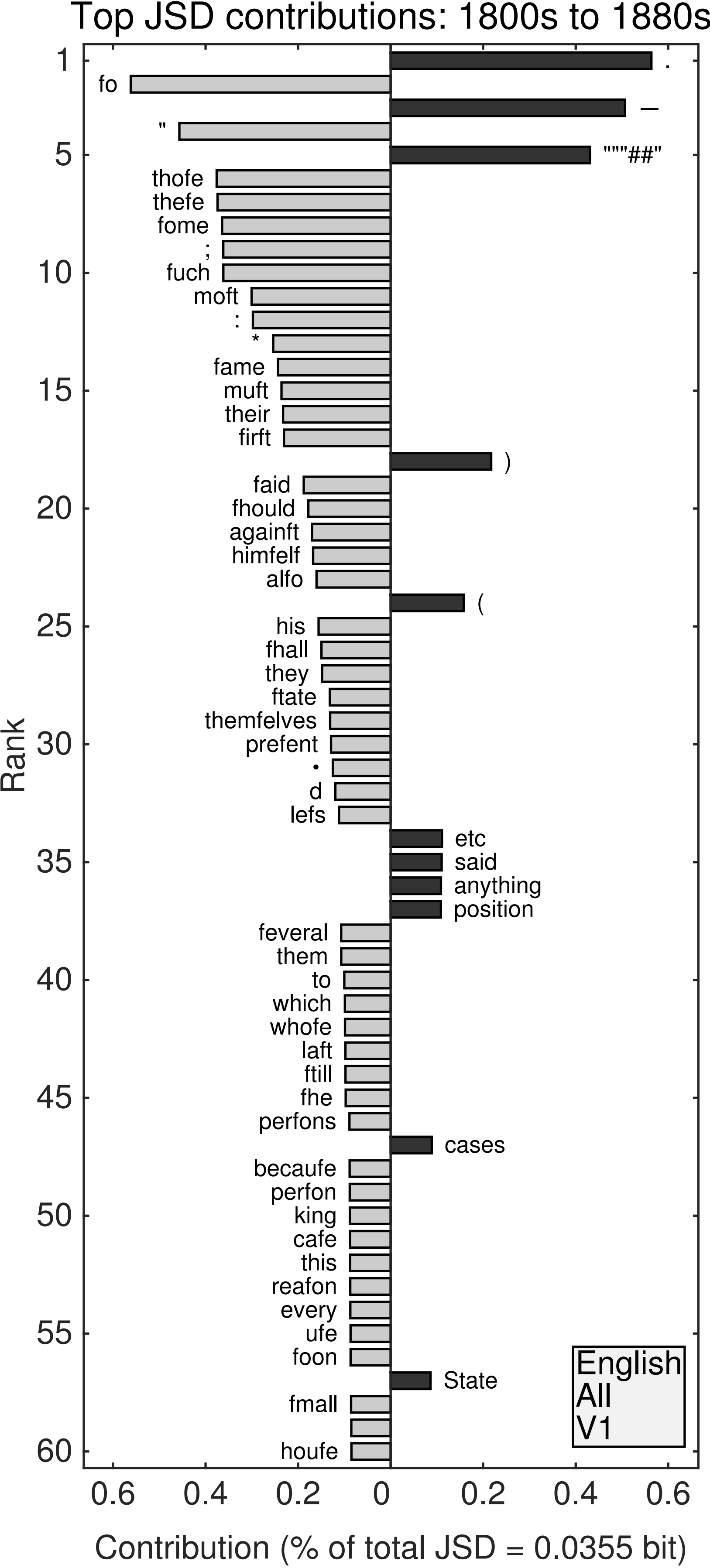
Top JSD contributions: 1800s to 1880s
Rank
Contribution (% of total JSD = 0.0355 bit)
0.6
0.4
0.2
0
0.2
0.4
0.6
1
5
10
15
20
25
30
35
40
45
50
55
60
.
fo
—
"
"""##"
thofe
thefe
fome
;
fuch
moft
:
*
fame
muft
their
firft
)
faid
fhould
againft
himfelf
alfo
(
his
fhall
they
ftate
themfelves
prefent
•
d
lefs
etc
said
anything
position
feveral
them
to
which
whofe
laft
ftill
fhe
perfons
cases
becaufe
perfon
king
cafe
this
reafon
every
ufe
foon
State
fmall
houfe
English
All
V1

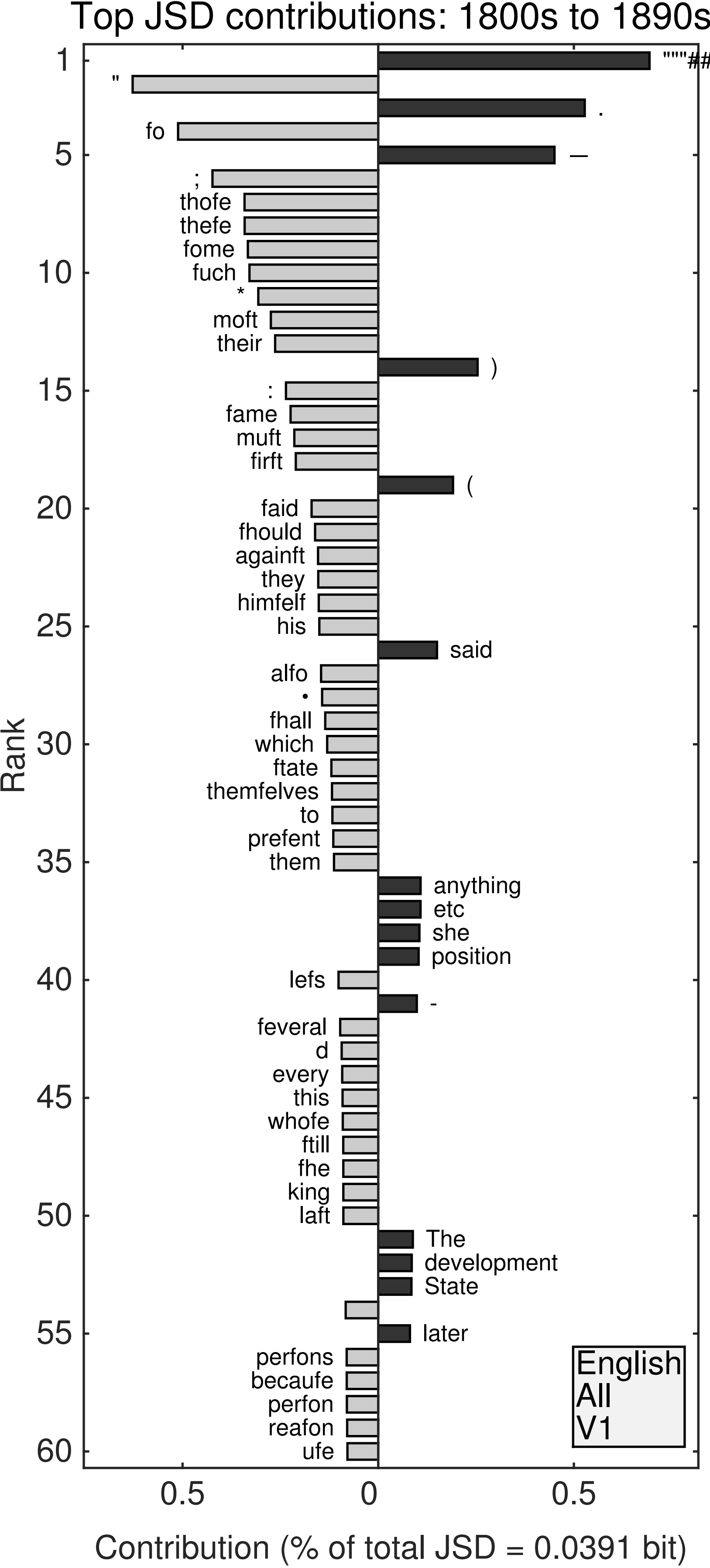
Top JSD contributions: 1800s to 1890s
Rank
Contribution (% of total JSD = 0.0391 bit)
0.5
0
0.5
1
5
10
15
20
25
30
35
40
45
50
55
60
"""##
"
.
fo
—
;
thofe
thefe
fome
fuch
*
moft
their
)
:
fame
muft
firft
(
faid
fhould
againft
they
himfelf
his
said
alfo
•
fhall
which
ftate
themfelves
to
prefent
them
anything
etc
she
position
lefs
-
feveral
d
every
this
whofe
ftill
fhe
king
laft
The
development
State
later
perfons
becaufe
perfon
reafon
ufe
English
All
V1

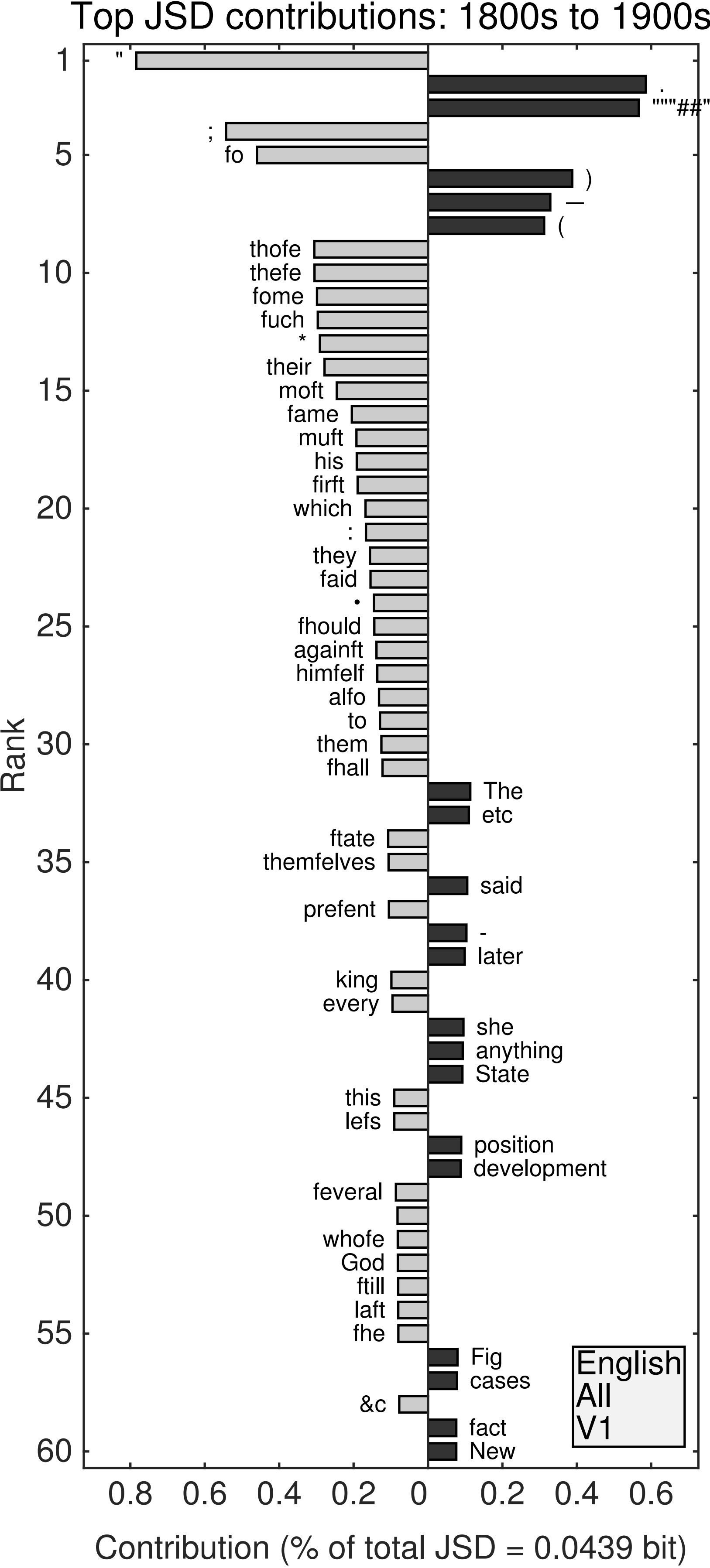

Top JSD contributions: 1800s to 1900s
"
.
"""##"
;
fo
)
—
(
thofe
thefe
fome
fuch
*
their
moft
fame
muft
his
firft
which
:
they
faid
•
fhould
againft
himfelf
alfo
to
them
fhall
The
etc
ftate
themfelves
said
prefent
-
later
king
every
she
anything
State
this
lefs
position
development
feveral
whofe
God
ftill
laft
fhe
Fig
cases
&c
fact
New
English
All
V1
Rank
Contribution (% of total JSD = 0.0439 bit)

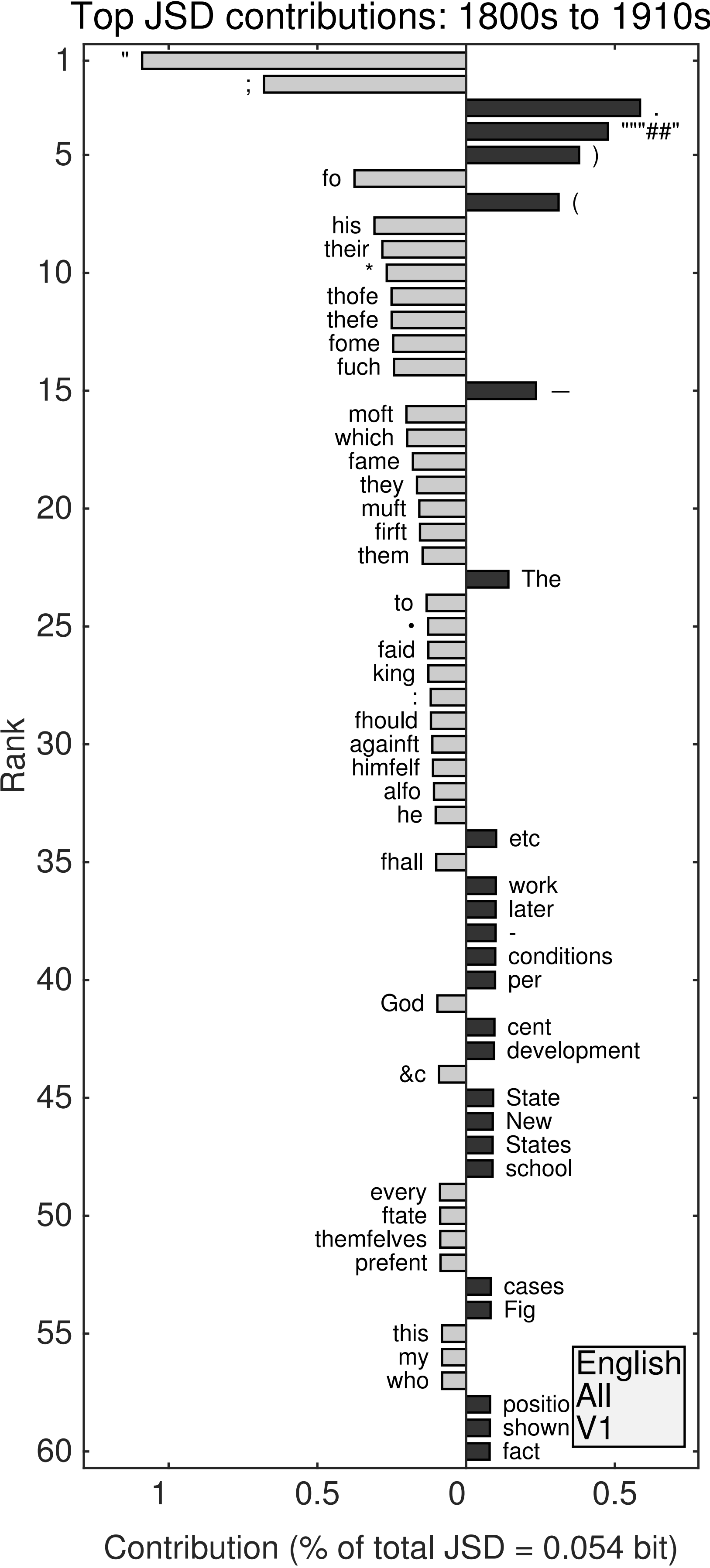

Top JSD contributions: 1800s to 1910s
Rank
Contribution (% of total JSD = 0.054 bit)
"
;
.
"""##"
)
fo
(
his
their
*
thofe
thefe
fome
fuch
—
moft
which
fame
they
muft
firft
them
The
to
•
faid
king
:
fhould
againft
himfelf
alfo
he
etc
fhall
work
later
-
conditions
per
God
cent
development
&c
State
New
States
school
every
ftate
themfelves
prefent
cases
Fig
this
my
who
positio
shown
fact
English
All
V1

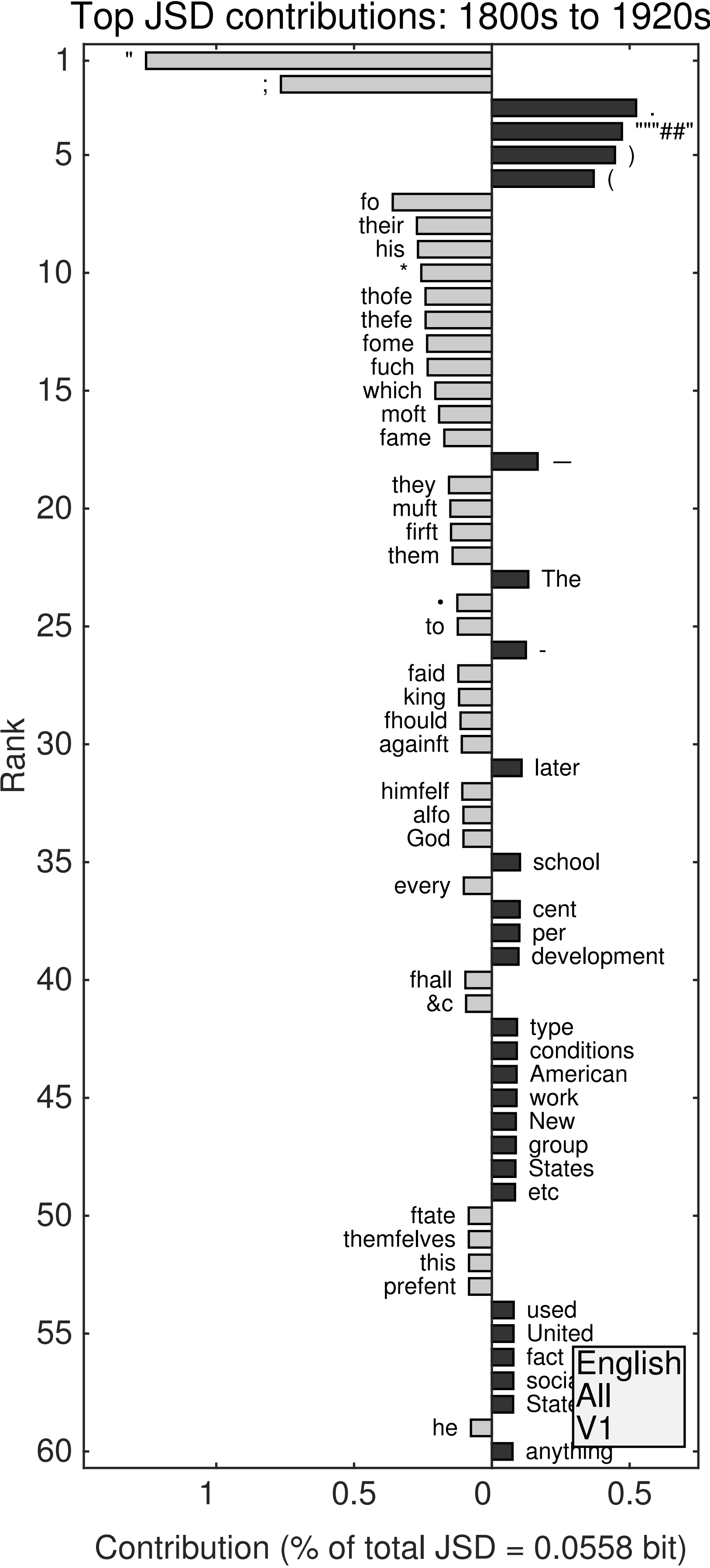

Top JSD contributions: 1800s to 1920s
Rank
"
;
.
""""##"
)
(
fo
their
his
*
thofe
thefe
fome
fuch
which
moft
fame
—
they
muft
firft
them
The
•
to
-
faid
king
fhould
againft
later
himfelf
alfo
God
school
every
cent
per
development
fhall
&c
type
conditions
American
work
New
group
States
etc
ftate
themfelves
this
prefent
used
United
fact
he
anything
English
All
V1
Contribution (% of total JSD = 0.0558 bit)

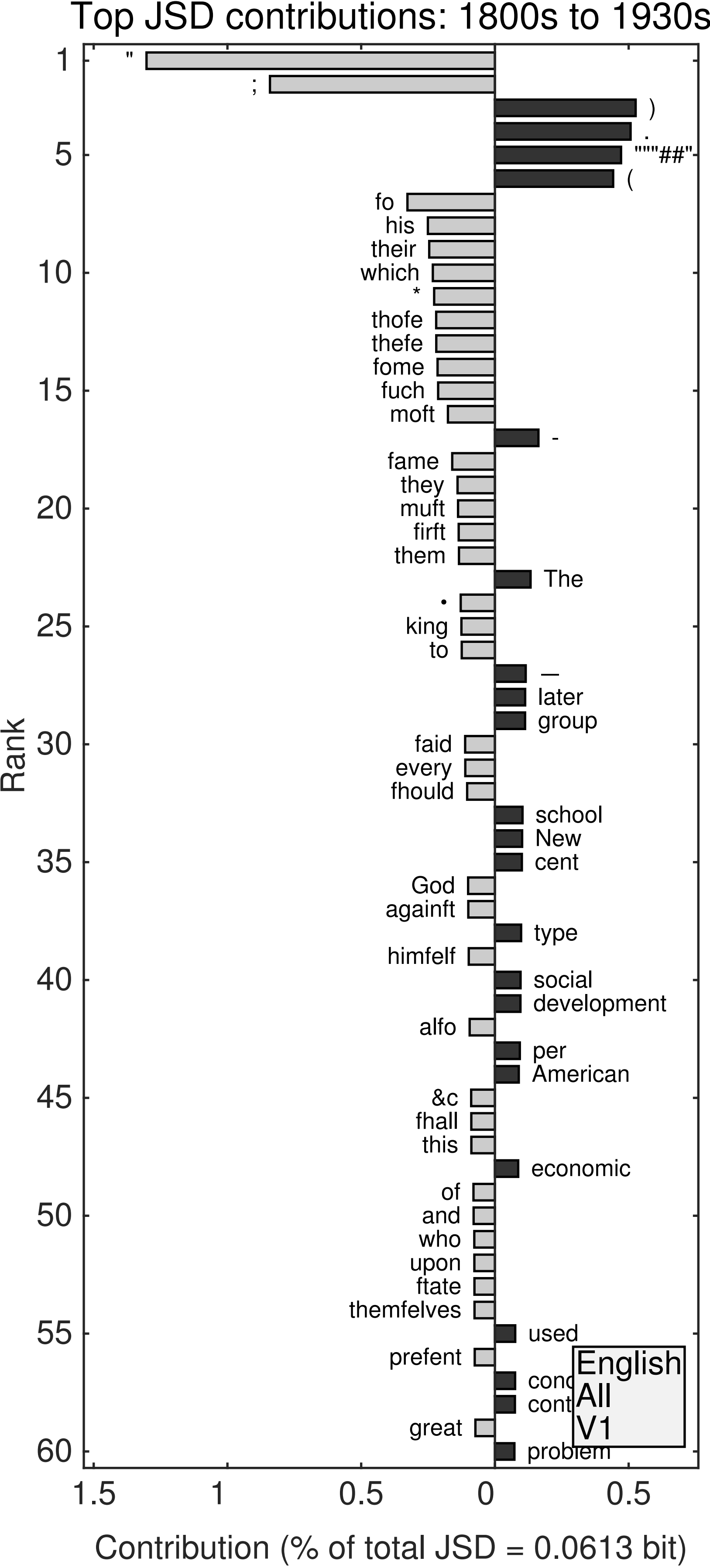

Top JSD contributions: 1800s to 1930s
Rank
Contribution (% of total JSD = 0.0613 bit)
1.5
1
0.5
0
0.5
1
5
10
15
20
25
30
35
40
45
50
55
60
"
;
)
.
"""##"
(
fo
his
their
which
*
thofe
thefe
fome
fuch
moft
-
fame
they
muft
firft
them
The
•
king
to
—
later
group
faid
every
fhould
school
New
cent
God
againft
type
himfelf
social
development
alfo
per
American
&c
fhall
this
economic
of
and
who
upon
ftate
themfelves
used
prefent
great
problem
English
All
V1

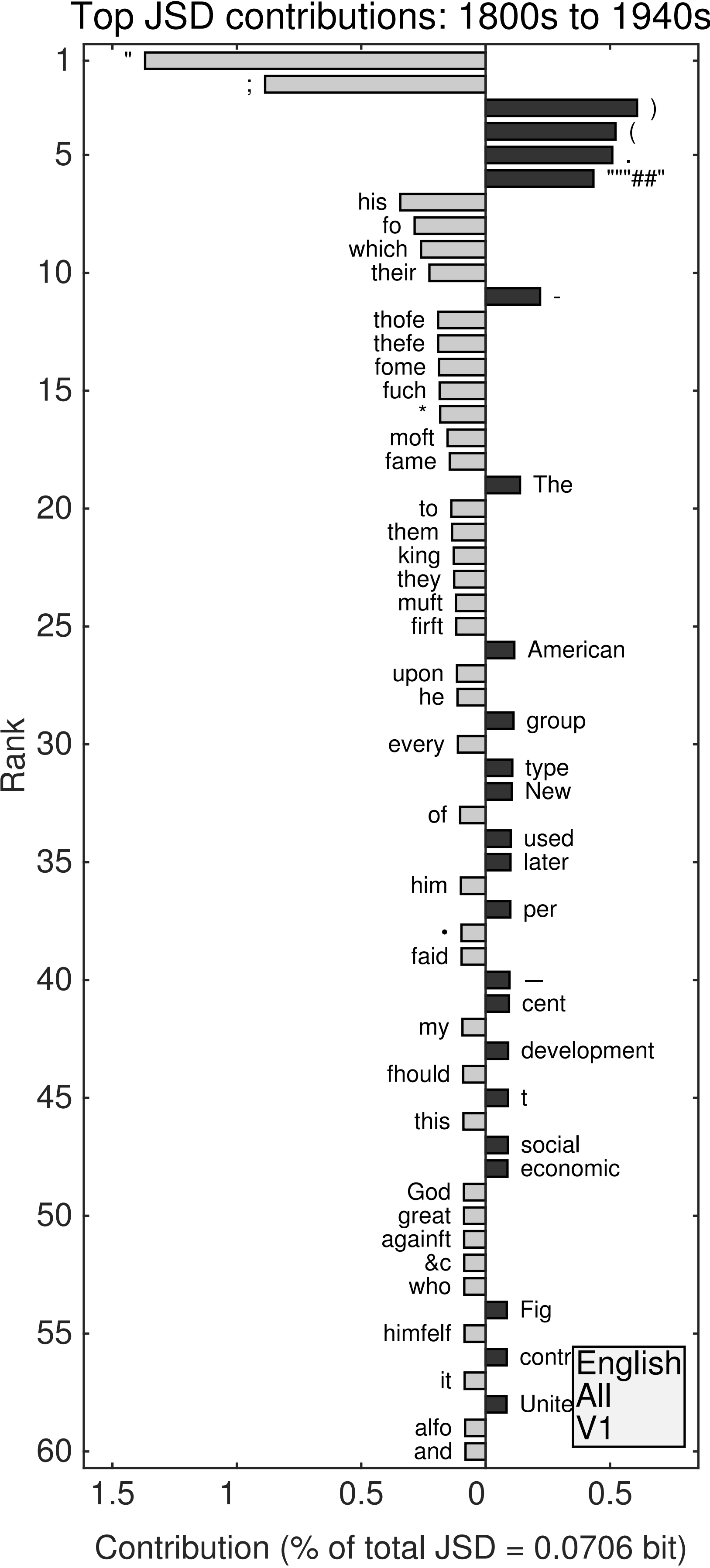

Top JSD contributions: 1800s to 1940s
Rank
Contribution (% of total JSD = 0.0706 bit)
"
;
)
(
.
"""##"
his
fo
which
their
-
thofe
thefe
fome
fuch
*
moft
fame
The
to
them
king
they
muft
firft
American
upon
he
group
every
type
New
of
used
later
him
per
•
faid
—
cent
my
development
fhould
t
this
social
economic
God
great
againft
&c
who
Fig
himfelf
contr
it
Unite
alfo
and
English
All
V1

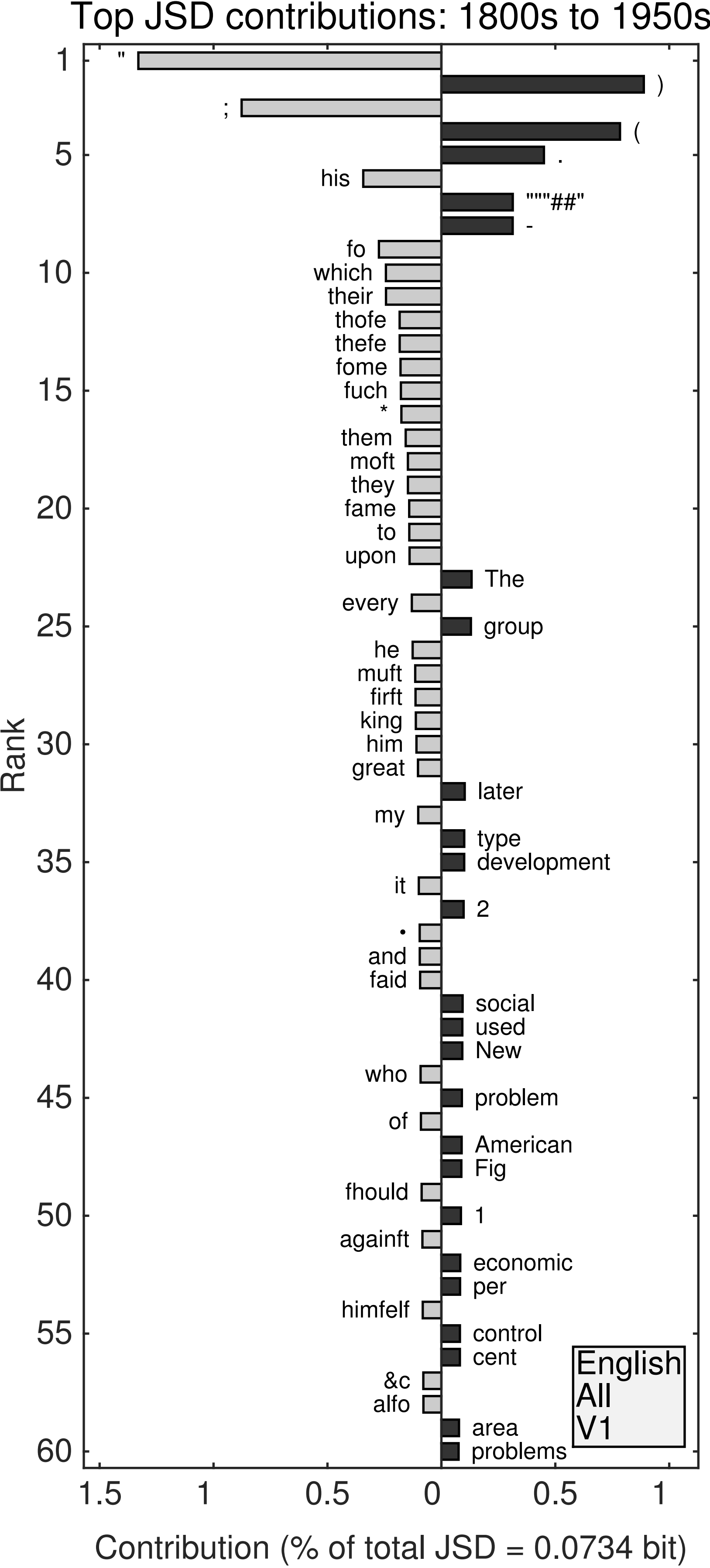

Top JSD contributions: 1800s to 1950s
Rank
Contribution (% of total JSD = 0.0734 bit)
" ) ; ( . his """##" - fo which their thofe thefe fome fuch * them moft they fame to upon The every group he muft firft king him great later my type development it 2 • and faid social used New who problem of American Fig fhould 1 againft economic per himfelf control cent &c alfo area problems
English
All
V1

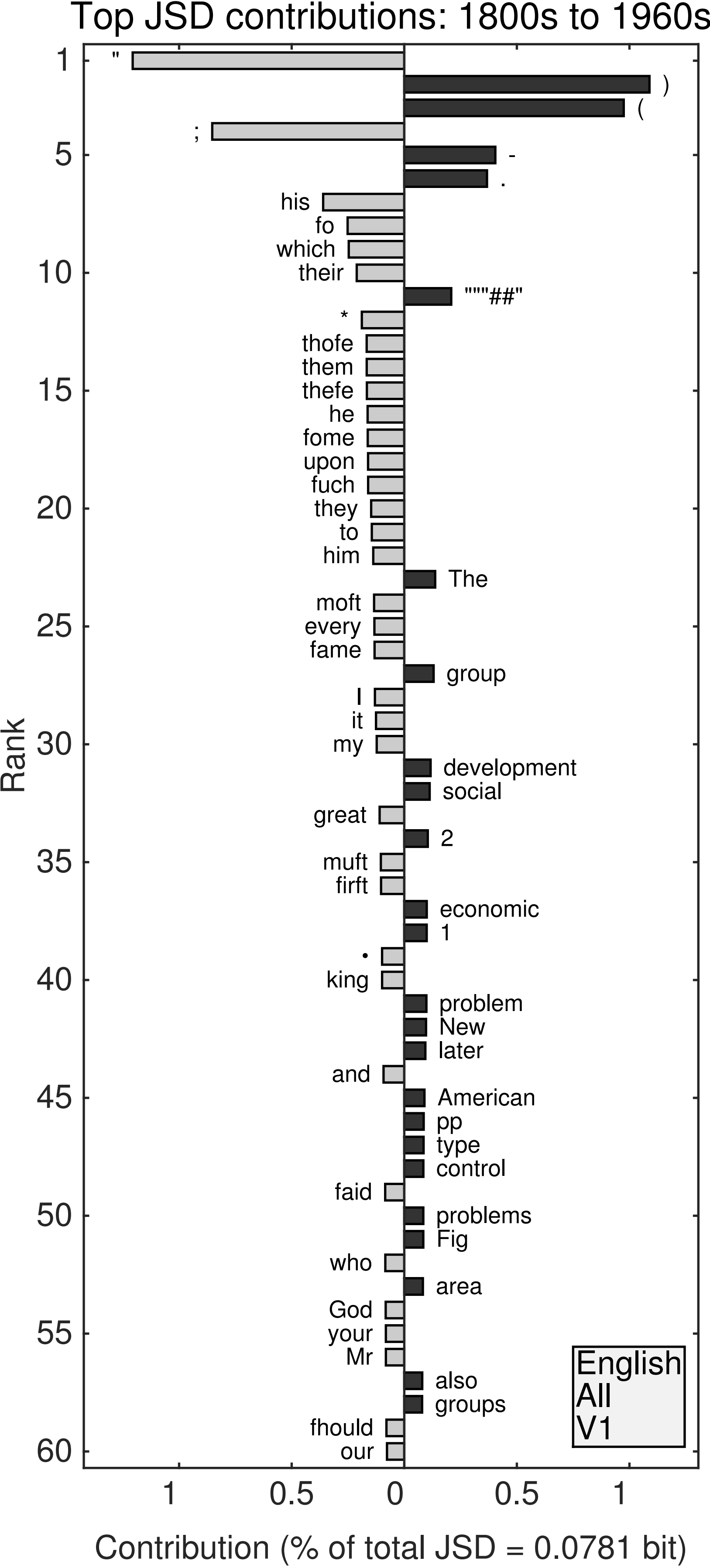

Top JSD contributions: 1800s to 1960s
Rank
Contribution (% of total JSD = 0.0781 bit)
English
All
V1
"
)
(
;
-
.
his
fo
which
their
"""##"
*
thofe
them
thefe
he
fome
upon
fuch
they
to
him
The
moft
every
fame
group
I
it
my
development
social
great
2
muft
firft
economic
1
•
king
problem
New
later
and
American
pp
type
control
faid
problems
Fig
who
area
God
your
Mr
also
groups
fhould
our

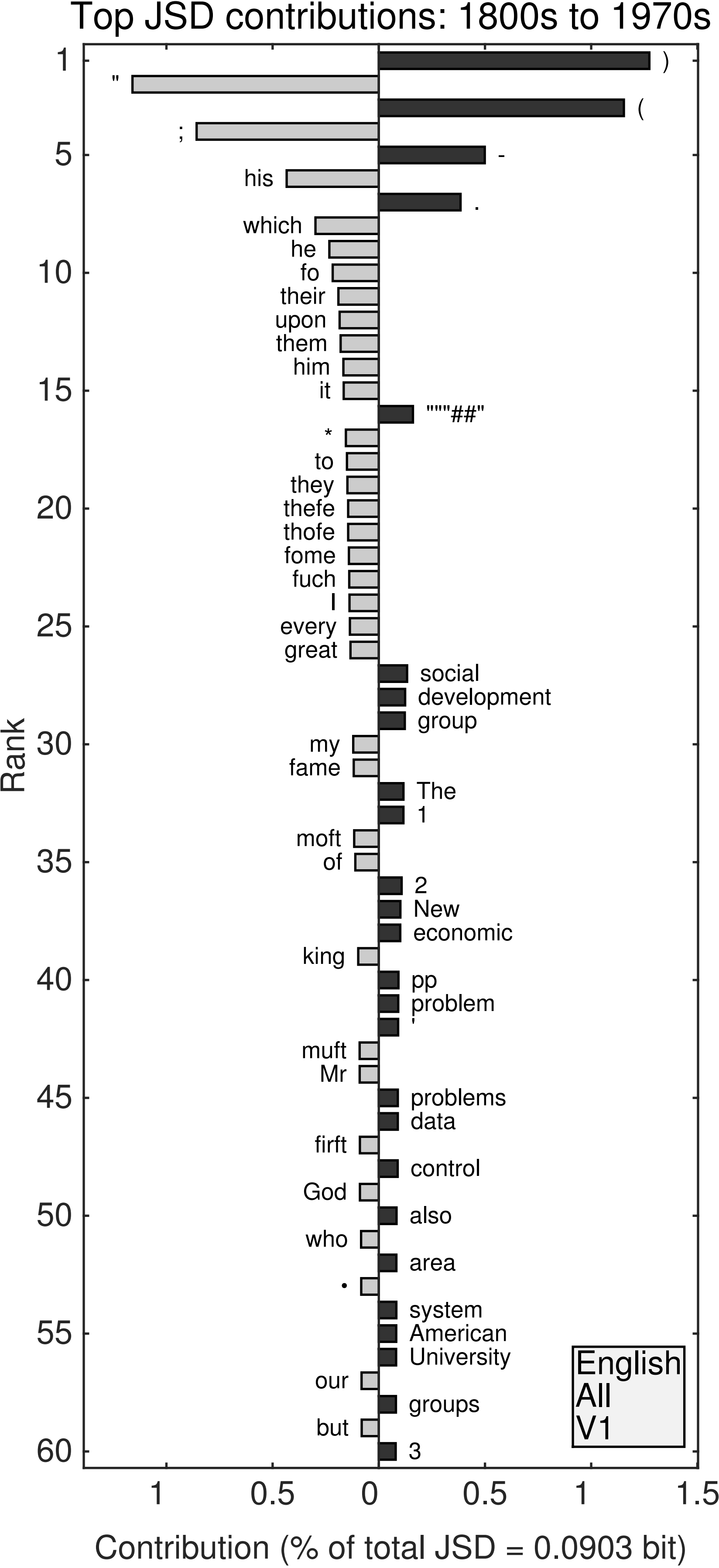

Top JSD contributions: 1800s to 1970s
Rank
Contribution (% of total JSD = 0.0903 bit)
)
"
(
;
-
his
.
which
he
fo
their
upon
them
him
it
"""##"
*
to
they
thefe
thofe
fome
fuch
I
every
great
social
development
group
my
fame
The
1
moft
of
2
New
economic
king
pp
problem
'
muft
Mr
problems
data
firft
control
God
also
who
area
•
system
American
University
our
groups
but
3
English
All
V1

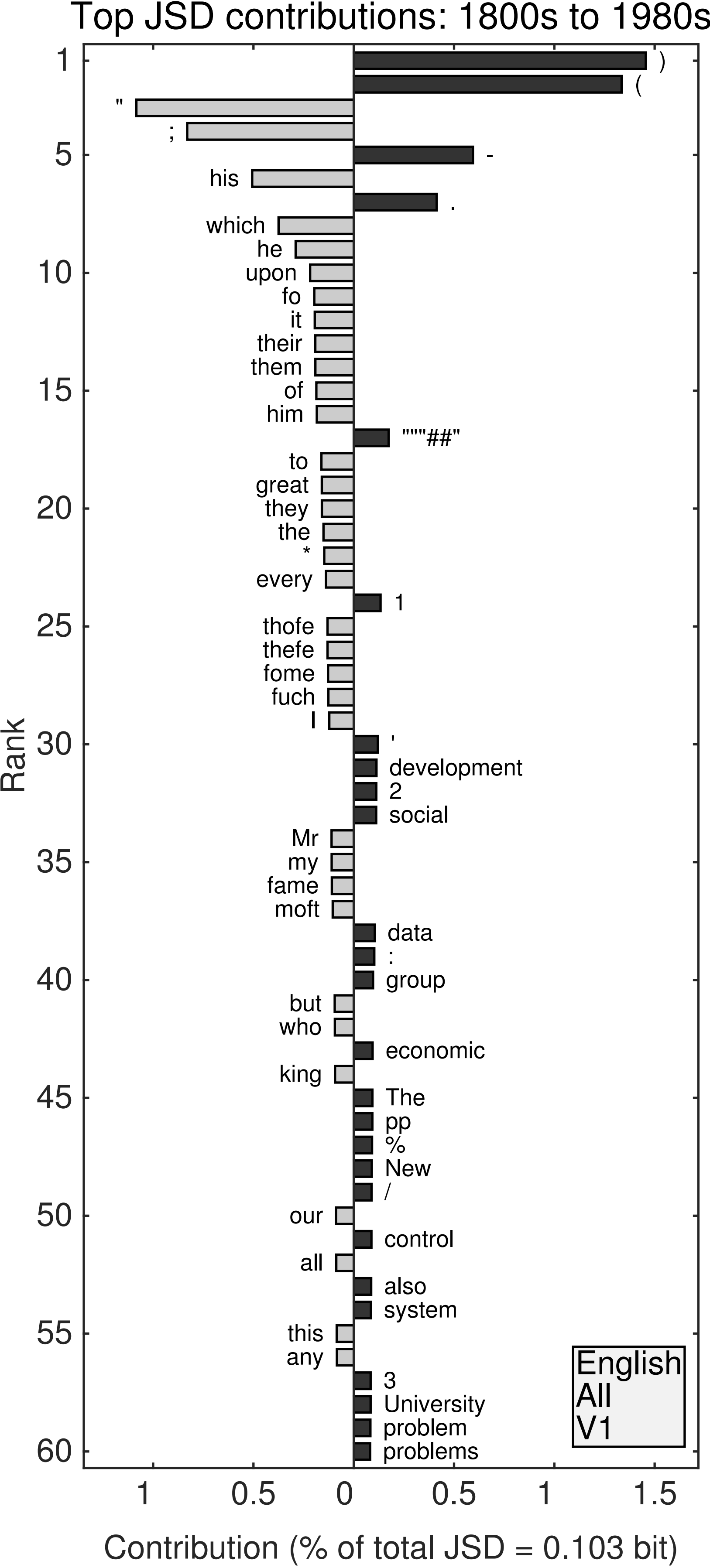

Top JSD contributions: 1800s to 1980s
Rank
Contribution (% of total JSD = 0.103 bit)
)
(
"
;
-
his
.
which
he
upon
fo
it
their
them
of
him
"""##"
to
great
they
the
*
every
1
thofe
thefe
fome
fuch
I
'
development
2
social
Mr
my
fame
moft
data
:
group
but
who
economic
king
The
pp
%
New
/
our
control
all
also
system
this
any
3
University
problem
problems
English
All
V1

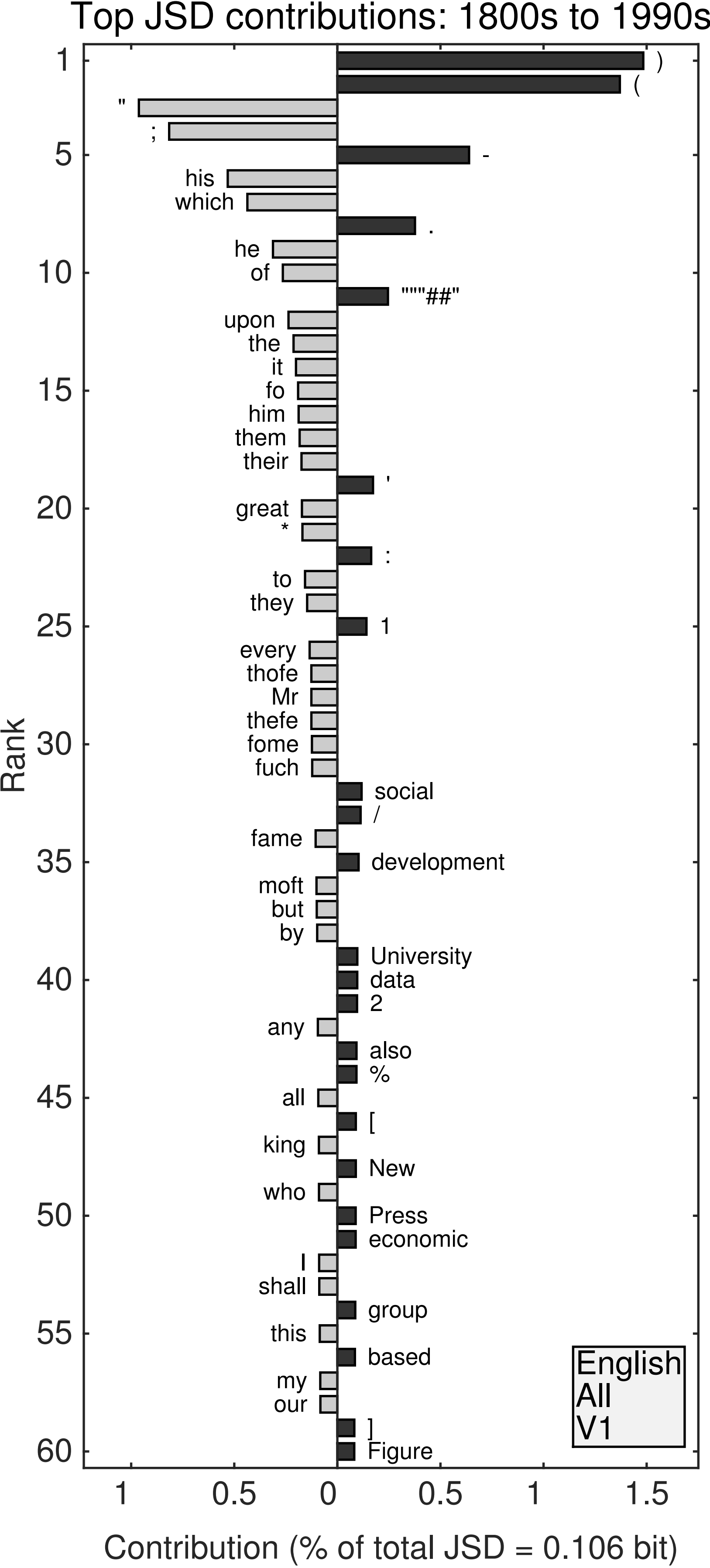

Top JSD contributions: 1800s to 1990s
Rank
Contribution (% of total JSD = 0.106 bit)
English
All
V1
)
(
"
;
-
his
which
.
he
of
"""##"
upon
the
it
fo
him
them
their
'
great
*
:
to
they
1
every
thofe
Mr
thefe
fome
fuch
social
/
fame
development
moft
but
by
University
data
2
any
also
%
all
[
king
New
who
Press
economic
I
shall
group
this
based
my
our
]
Figure

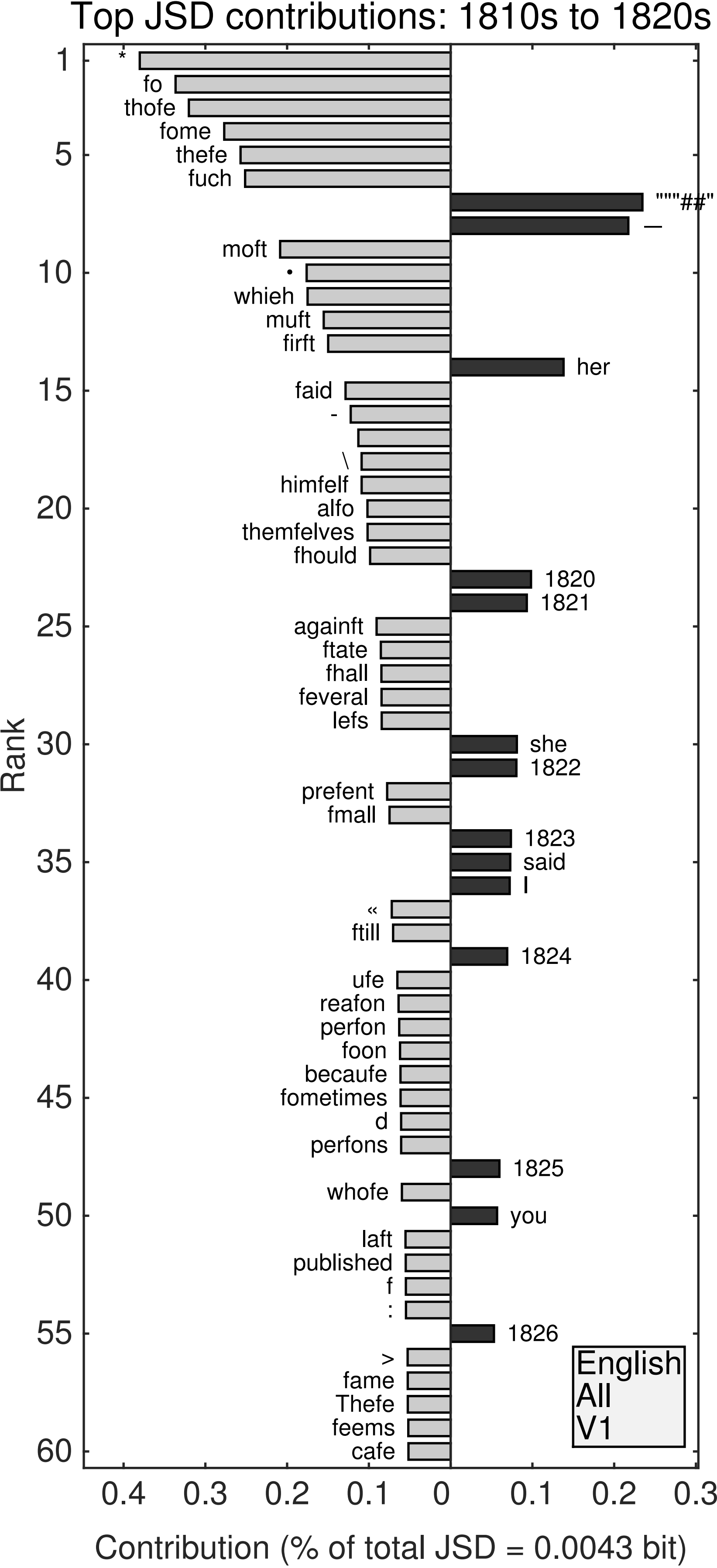

Top JSD contributions: 1810s to 1820s
Rank
Contribution (% of total JSD = 0.0043 bit)
English
All
V1
*
fo
thofe
fome
thefe
fuch
"""##"
—
moft
•
whieh
muft
firft
her
faid
-
\
himfelf
alfo
themfelves
fhould
1820
1821
againft
ftate
fhall
feveral
lefs
she
1822
prefent
fmall
1823
said
I
«
ftill
1824
ufe
reafon
perfon
foon
becaufe
fometimes
d
perfons
1825
whofe
you
laft
published
f
:
1826
>
fame
Thefe
feems
cafe

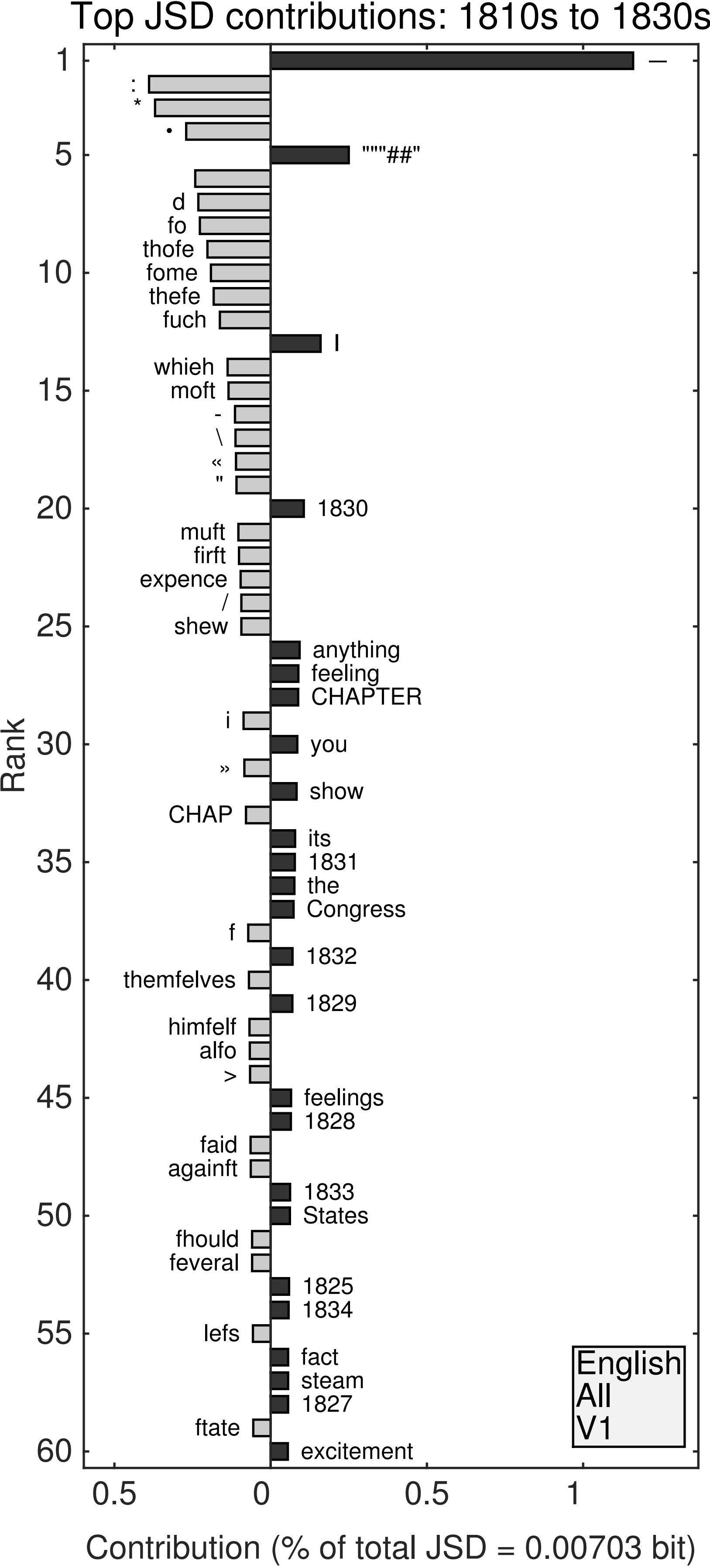
Top JSD contributions: 1810s to 1830s
Rank
1
5
10
15
20
25
30
35
40
45
50
55
60
—
:
*
•
"""##"
d
fo
thofe
fome
thefe
fuch
I
whieh
moft
-
\
«
"
1830
muft
firft
expence
/
shew
anything
feeling
CHAPTER
i
you
»
show
CHAP
its
1831
the
Congress
f
1832
themfelves
1829
himfelf
alfo
>
feelings
1828
faid
againft
1833
States
fhould
feveral
1825
1834
lefs
fact
steam
1827
ftate
excitement
English
All
V1
0.5
0
0.5
1
Contribution (% of total JSD = 0.00703 bit)

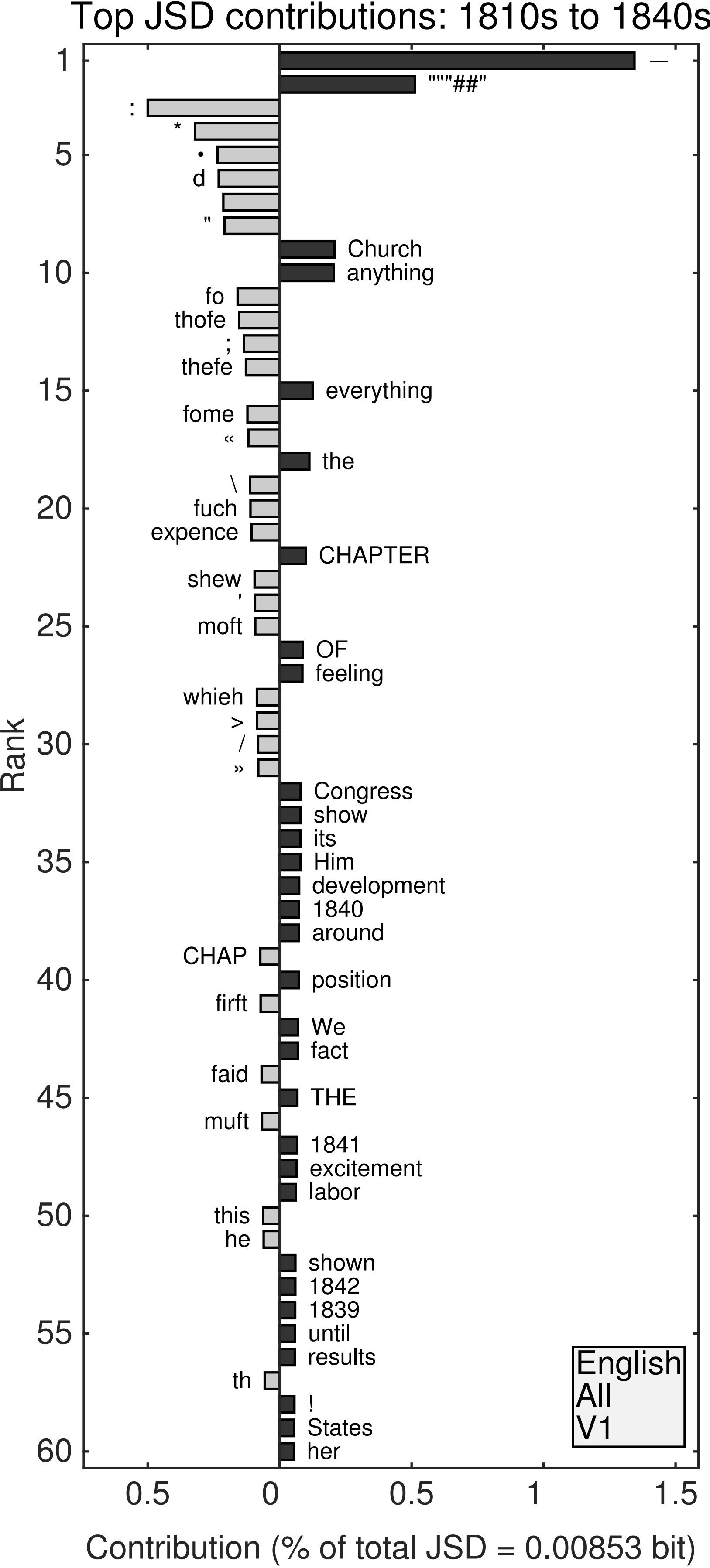

Top JSD contributions: 1810s to 1840s
Rank
Contribution (% of total JSD = 0.00853 bit)
—
""""##"
:
*
•
d
"
Church
anything
fo
thofe
;
thefe
everything
fome
«
the
\
fuch
expence
CHAPTER
shew
'
moft
OF
feeling
whieh
>
/
»
Congress
show
its
Him
development
1840
around
CHAP
position
firft
We
fact
faid
THE
muft
1841
excitement
labor
this
he
shown
1842
1839
until
results
th
!
States
her
English
All
V1

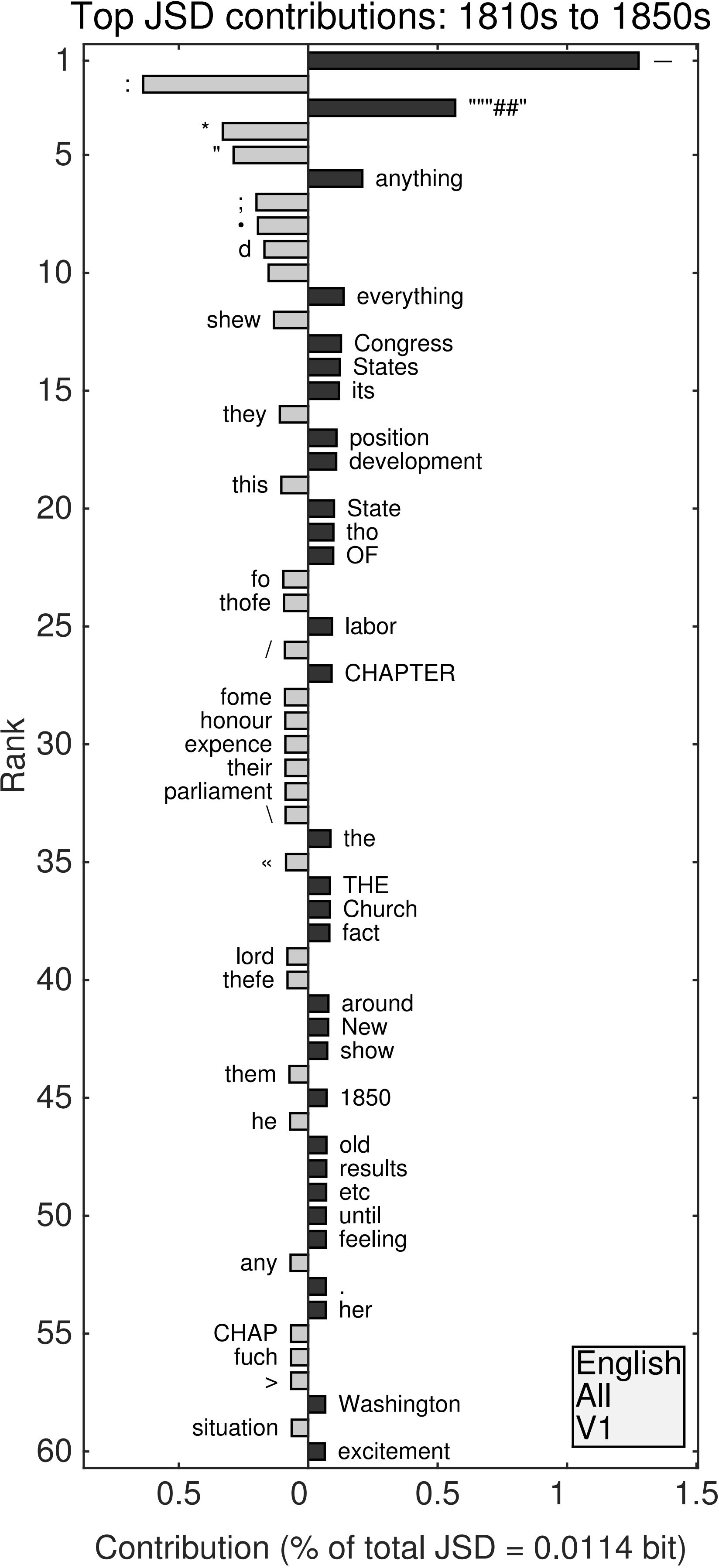
Top JSD contributions: 1810s to 1850s
Rank
1
5
10
15
20
25
30
35
40
45
50
55
60
—
:
"""##"
*
"
anything
;
•
d
everything
shew
Congress
States
its
they
position
development
this
State
tho
OF
fo
thofe
labor
/
CHAPTER
fome
honour
expence
their
parliament
\
the
«
THE
Church
fact
lord
thefe
around
New
show
them
1850
he
old
results
etc
until
feeling
any
.
her
CHAP
fuch
>
Washington
situation
excitement
English
All
V1
0.5
0
0.5
1
1.5
Contribution (% of total JSD = 0.0114 bit)

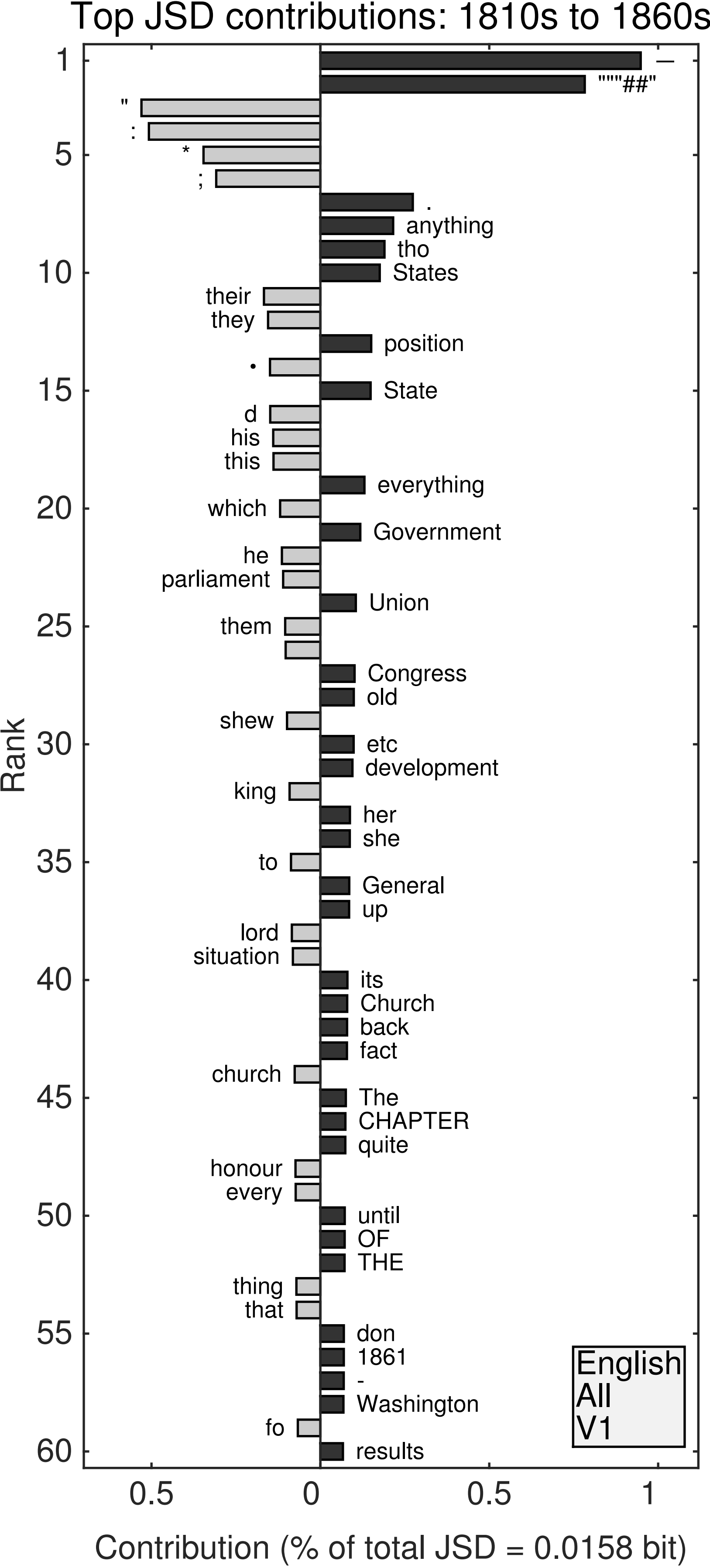
Top JSD contributions: 1810s to 1860s
Rank
Contribution (% of total JSD = 0.0158 bit)
English
All
V1
—
"""##"
"
:
*
;
.
anything
tho
States
their
they
position
•
State
d
his
this
everything
which
Government
he
parliament
Union
them
Congress
old
shew
etc
development
king
her
she
to
General
up
lord
situation
its
Church
back
fact
church
The
CHAPTER
quite
honour
every
until
OF
THE
thing
that
don
1861
-
Washington
fo
results

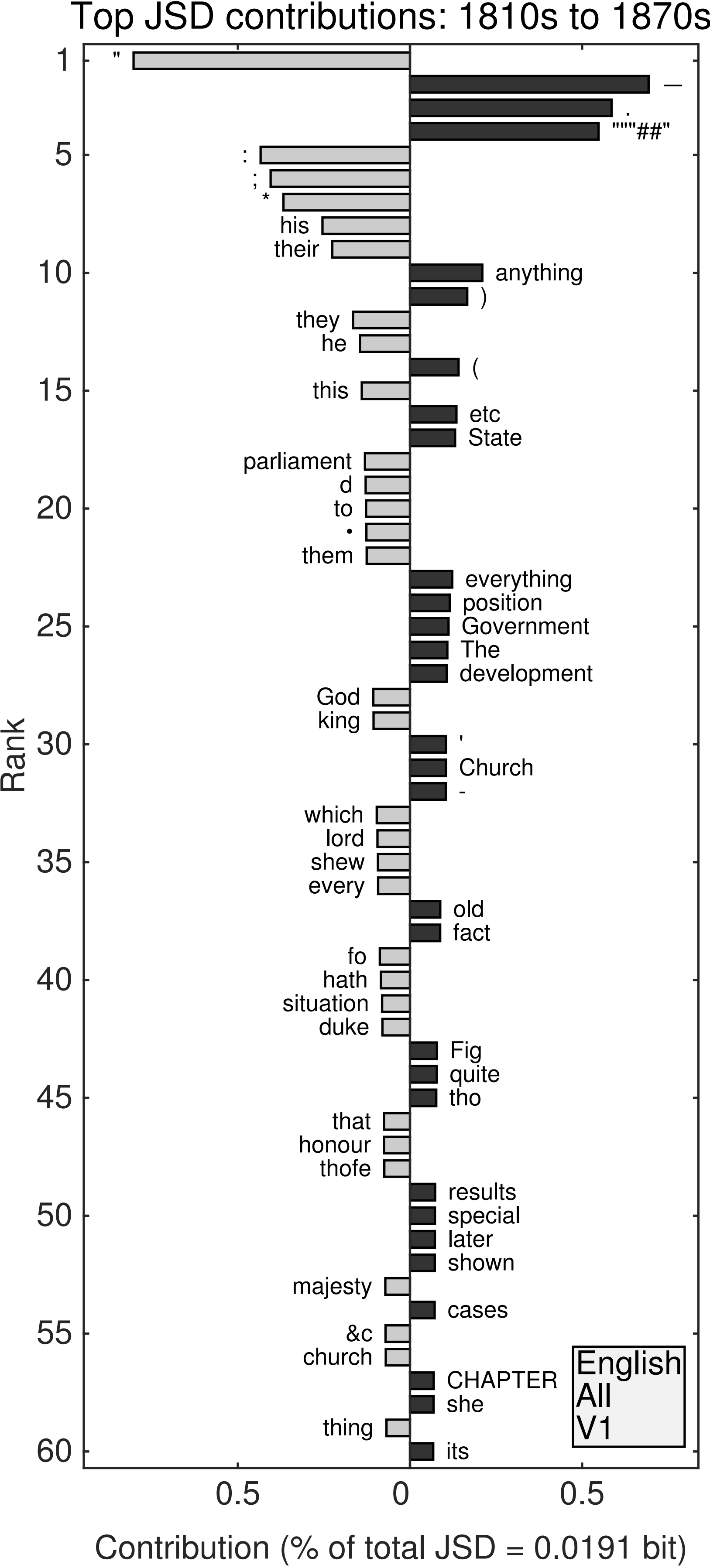
Top JSD contributions: 1810s to 1870s
Rank
Contribution (% of total JSD = 0.0191 bit)
0.5
0
0.5
1
5
10
15
20
25
30
35
40
45
50
55
60
"
—
.
"""##"
:
;
*
his
their
anything
)
they
he
(
this
etc
State
parliament
d
to
•
them
everything
position
Government
The
development
God
king
'
Church
-
which
lord
shew
every
old
fact
fo
hath
situation
duke
Fig
quite
tho
that
honour
thofe
results
special
later
shown
majesty
cases
&c
church
CHAPTER
she
thing
its
English
All
V1

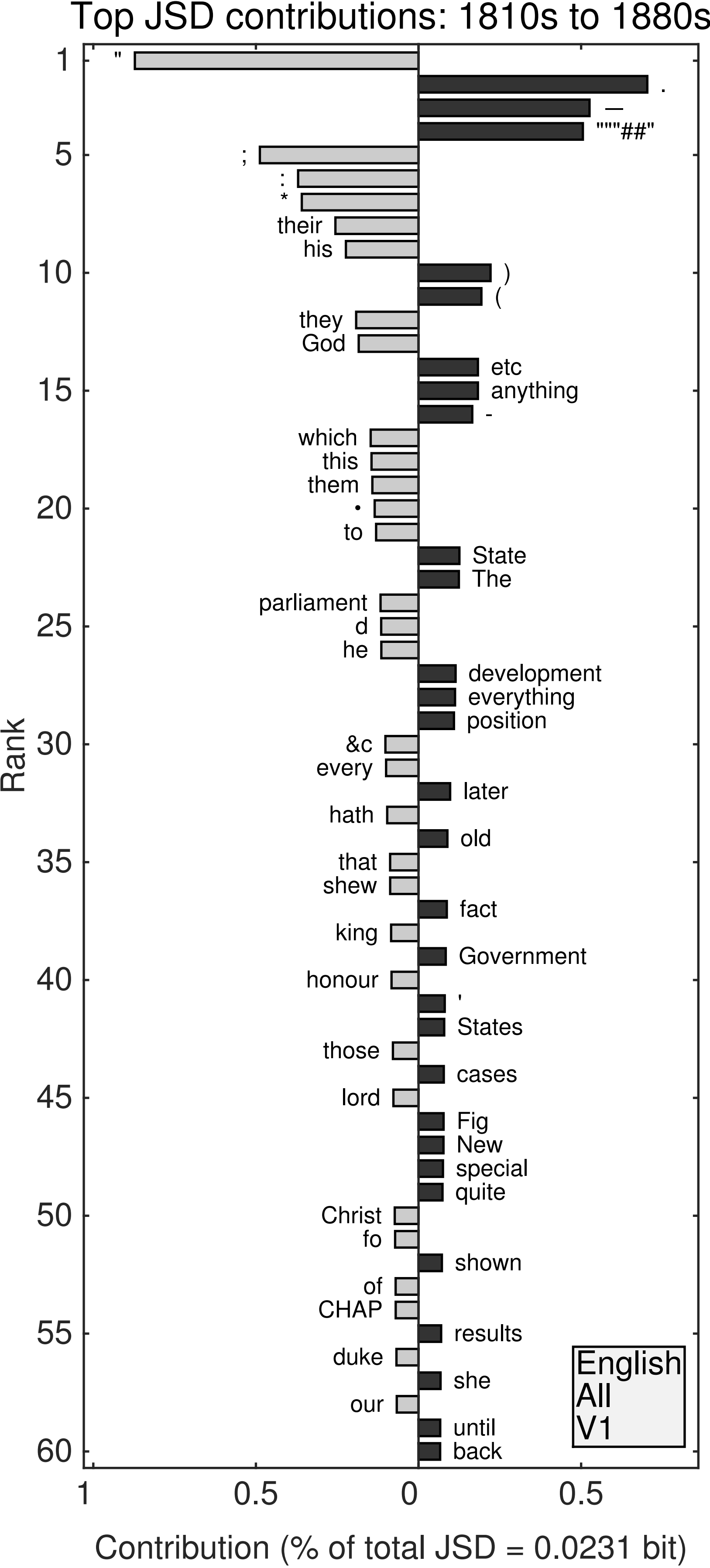

Top JSD contributions: 1810s to 1880s
Rank
Contribution (% of total JSD = 0.0231 bit)
1
5
10
15
20
25
30
35
40
45
50
55
60
1
0.5
0
0.5
"
.
—
"""##"
;
:
*
their
his
)
(
they
God
etc
anything
-
which
this
them
•
to
State
The
parliament
d
he
development
everything
position
&c
every
later
hath
old
that
shew
fact
king
Government
honour
'
States
those
cases
lord
Fig
New
special
quite
Christ
fo
shown
of
CHAP
results
duke
she
our
until
back
English
All
V1

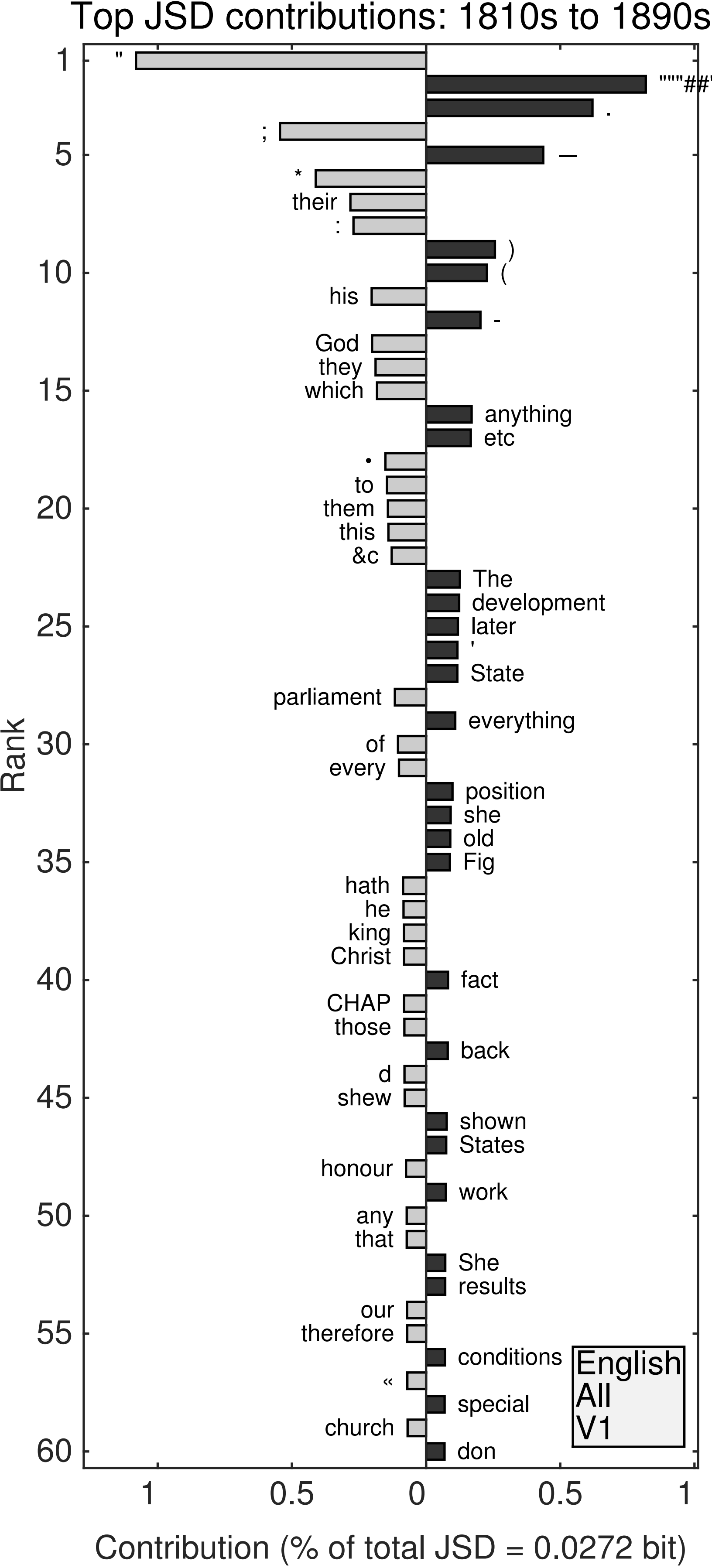
Top JSD contributions: 1810s to 1890s
Rank
Contribution (% of total JSD = 0.0272 bit)
English
All
V1
"
.
;
—
*
their
:
)
(
his
-
God
they
which
anything
etc
•
to
them
this
&c
The
development
later
'
State
parliament
everything
of
every
position
she
old
Fig
hath
he
king
Christ
fact
CHAP
those
back
d
shew
shown
States
honour
work
any
that
She
results
our
therefore
conditions
special
church
don

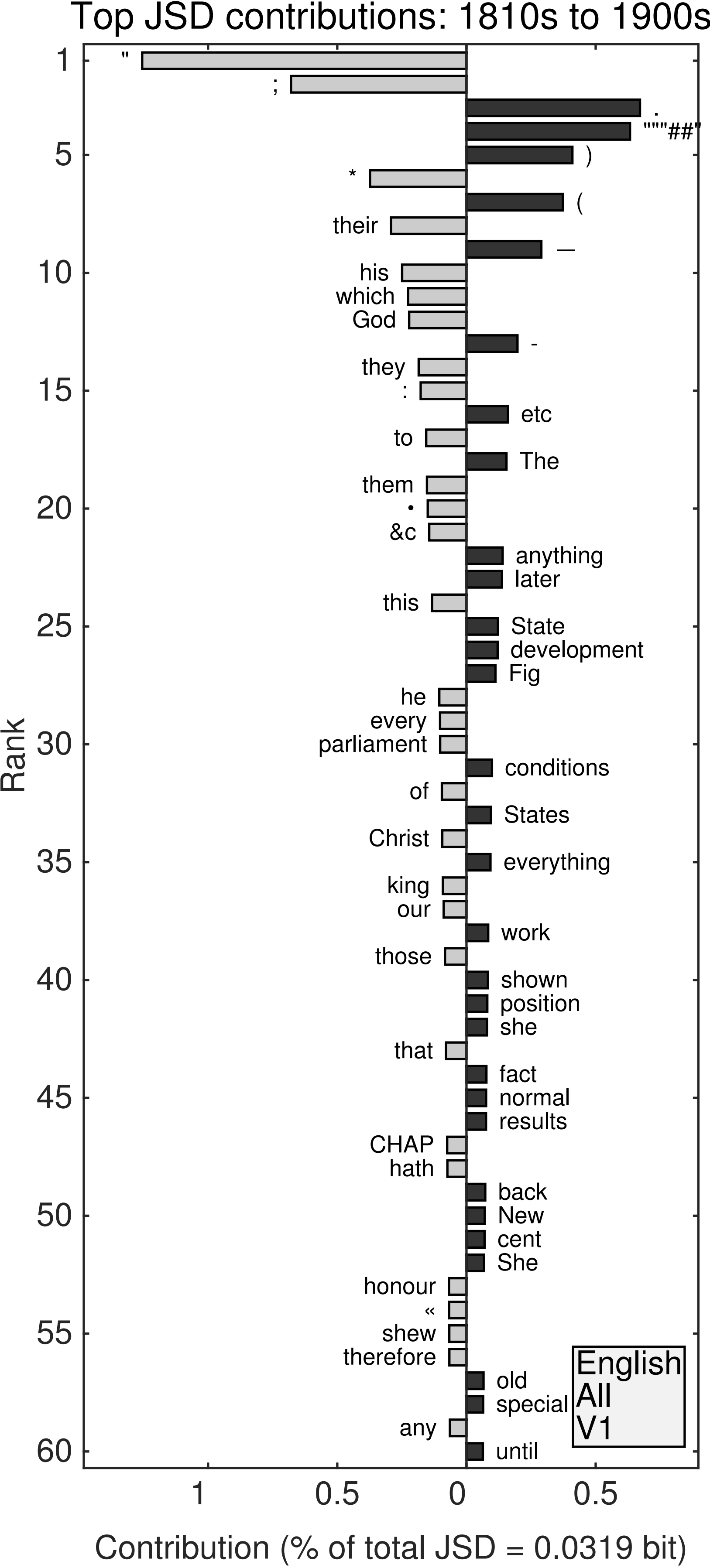
Top JSD contributions: 1810s to 1900s
Rank
Contribution (% of total JSD = 0.0319 bit)
1
5
10
15
20
25
30
35
40
45
50
55
60
1
0.5
0
0.5
"
;
.
""""##"
)
*
(
their
—
his
which
God
-
they
:
etc
to
The
them
•
&c
anything
later
this
State
development
Fig
he
every
parliament
conditions
of
States
Christ
everything
king
our
work
those
shown
position
she
that
fact
normal
results
CHAP
hath
back
New
cent
She
honour
«
shew
therefore
old
special
any
until
English
All
V1

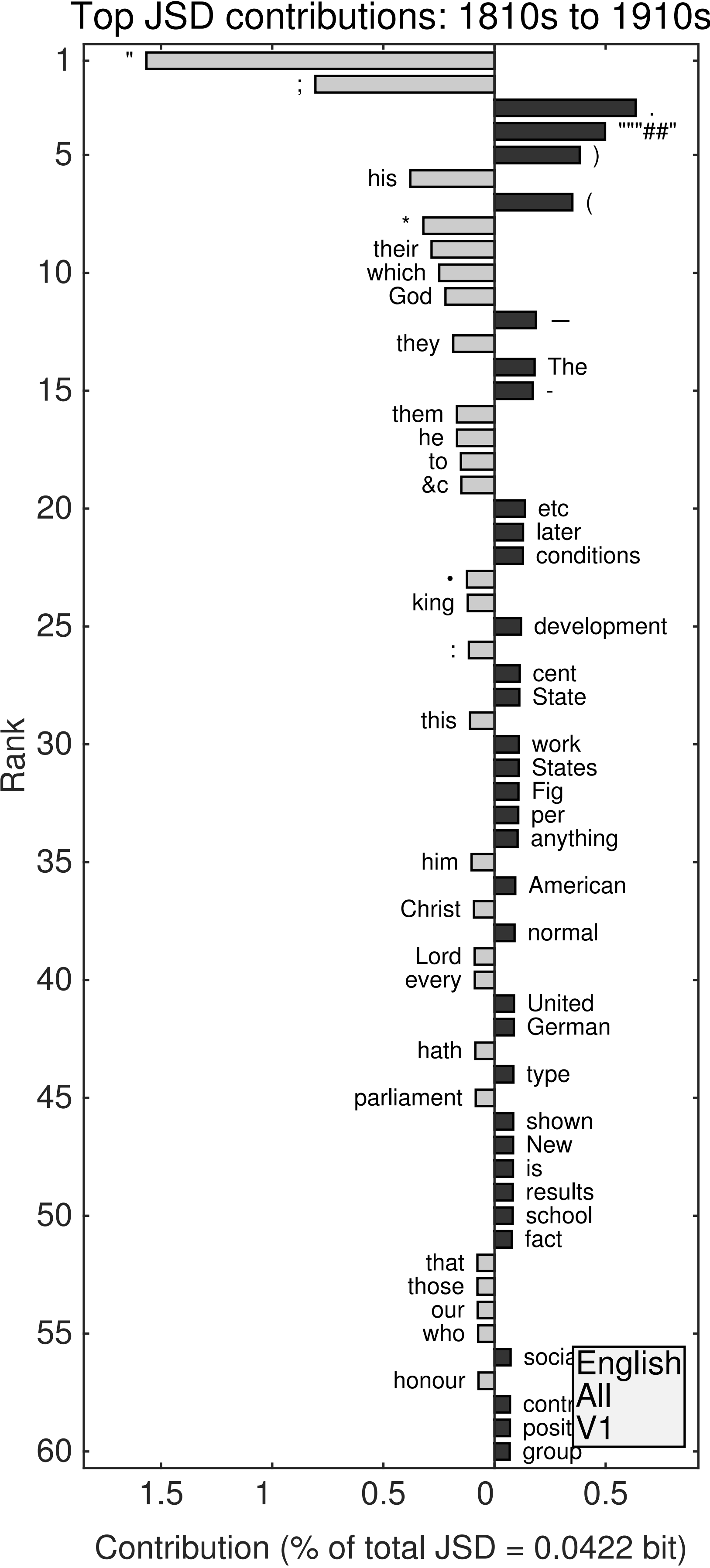
Top JSD contributions: 1810s to 1910s
Rank
Contribution (% of total JSD = 0.0422 bit)
"
;
.
"""##"
)
his
(
*
their
which
God
—
they
The
-
them
he
to
&c
etc
later
conditions
•
king
development
:
cent
State
this
work
States
Fig
per
anything
him
American
Christ
normal
Lord
every
United
German
hath
type
parliament
shown
New
is
results
school
fact
that
those
our
who
honour
group
English
All
V1

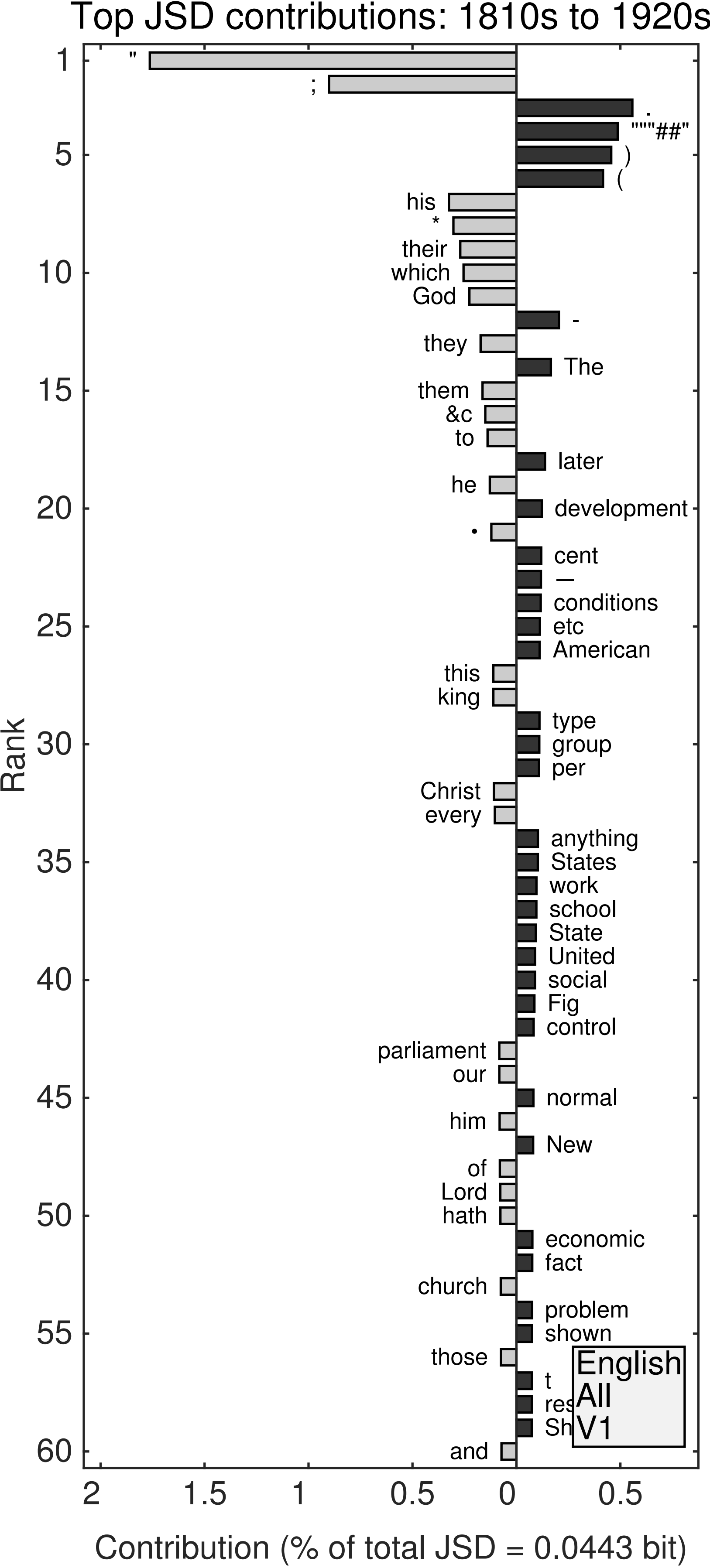

Top JSD contributions: 1810s to 1920s
Rank
1
5
10
15
20
25
30
35
40
45
50
55
60
"
;
.
"""##"
)
(
his
*
their
which
God
-
they
The
them
&c
to
later
he
development
•
cent
—
conditions
etc
American
this
king
type
group
per
Christ
every
anything
States
work
school
State
United
social
Fig
control
parliament
our
normal
him
New
of
Lord
hath
economic
fact
church
problem
shown
those
t
res
Sh
and
English
All
V1
2
1.5
1
0.5
0
0.5
Contribution (% of total JSD = 0.0443 bit)

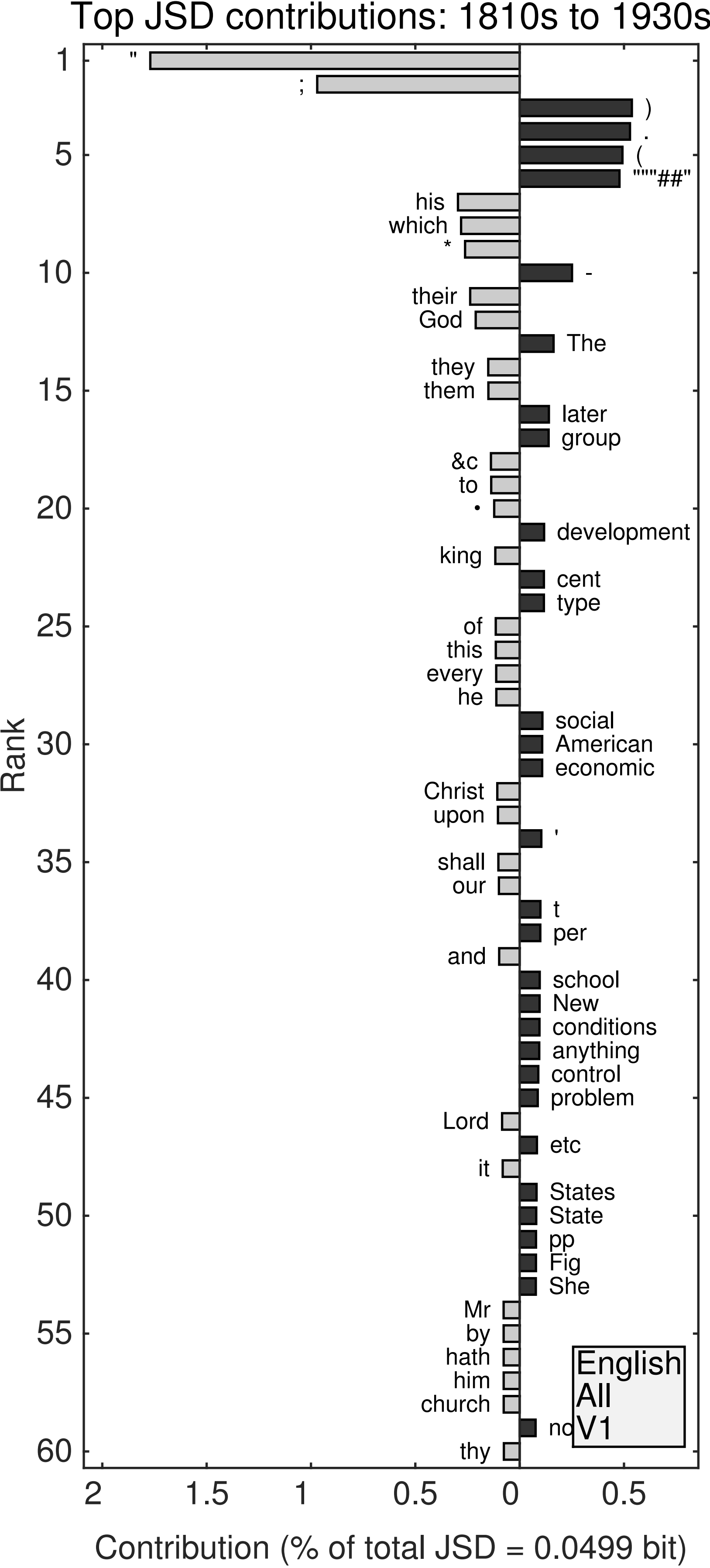
Top JSD contributions: 1810s to 1930s
Rank
Contribution (% of total JSD = 0.0499 bit)
"
;
)
.
(
"""##"
his
which
*
-
their
God
The
they
them
later
group
&c
to
•
development
king
cent
type
of
this
every
he
social
American
economic
Christ
upon
'
shall
our
t
per
and
school
New
conditions
anything
control
problem
Lord
etc
it
States
State
pp
Fig
She
Mr
by
hath
him
church
no
thy
English
All
V1

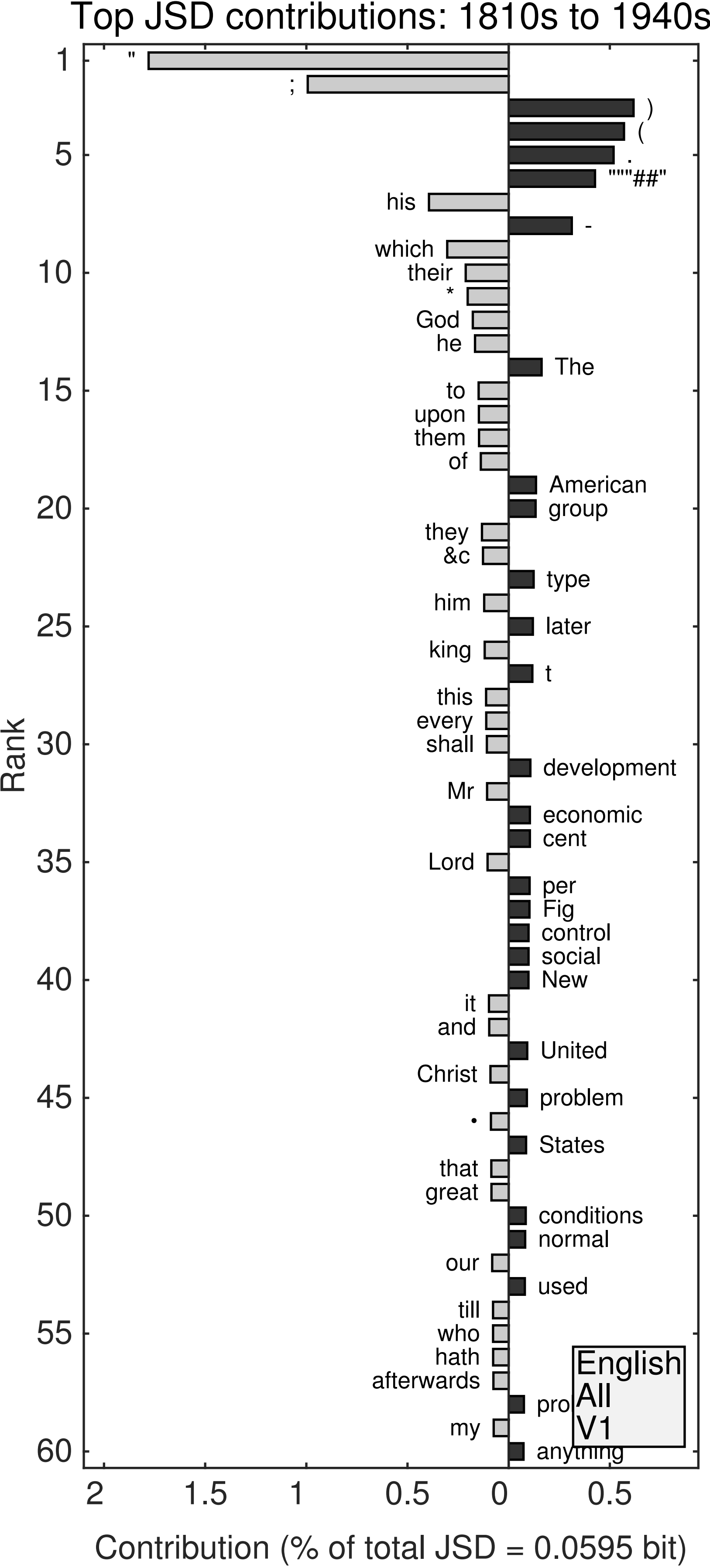

Top JSD contributions: 1810s to 1940s
"
;
)
(
.
"""##"
his
-
which
their
*
God
he
The
to
upon
them
of
American
group
they
&c
type
him
later
king
t
this
every
shall
development
Mr
economic
cent
Lord
per
Fig
control
social
New
it
and
United
Christ
problem
•
States
that
great
conditions
normal
our
used
till
who
hath
afterwards
my
English
All
V1
Rank
1
5
10
15
20
25
30
35
40
45
50
55
60
2
1.5
1
0.5
0
0.5
Contribution (% of total JSD = 0.0595 bit)

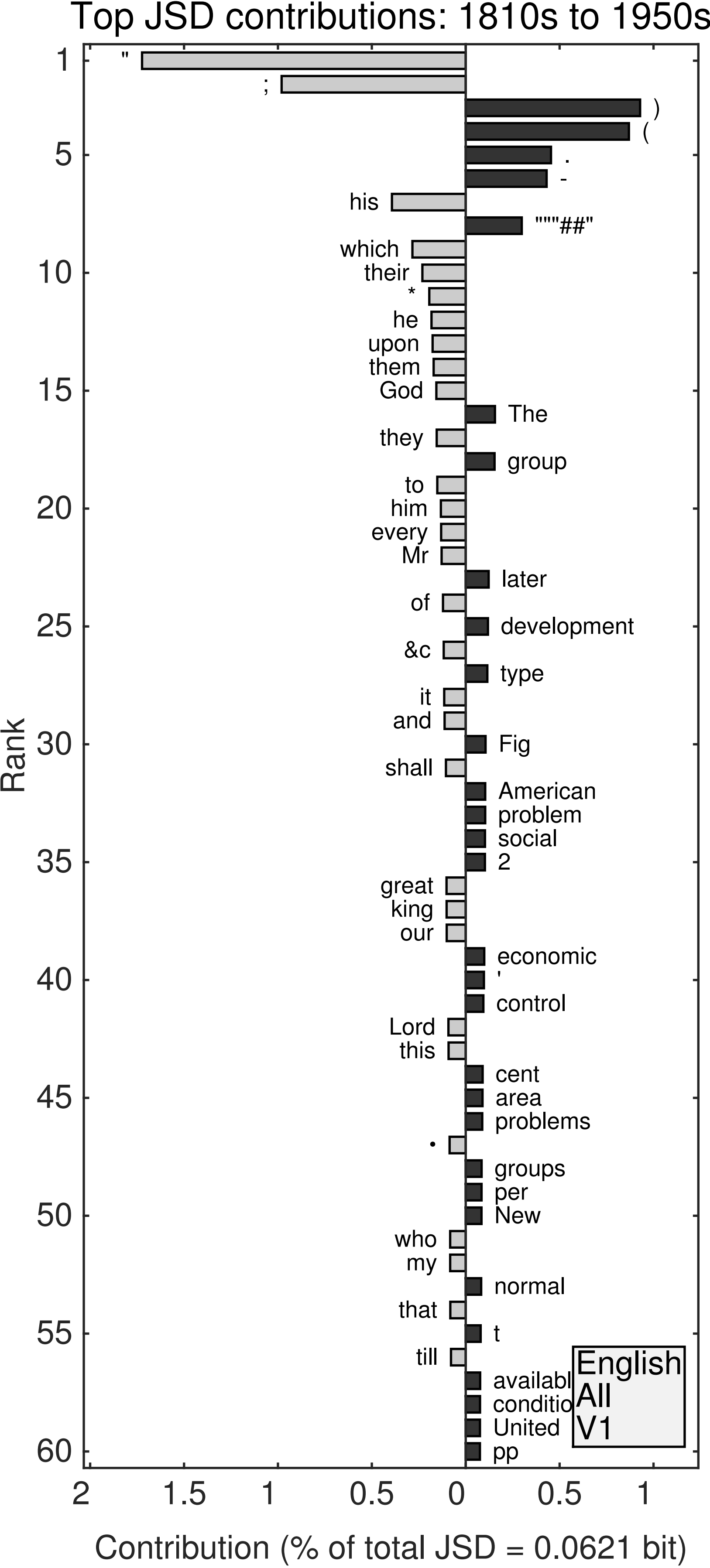
Top JSD contributions: 1810s to 1950s
Rank
"
;
)
(
.
-
his
"""##"
which
their
*
he
upon
them
God
The
they
group
to
him
every
Mr
later
of
development
&c
type
it
and
Fig
shall
American
problem
social
2
great
king
our
economic
'
control
Lord
this
cent
area
problems
•
groups
per
New
who
my
normal
that
t
till
availabl
conditio
United
pp
English
All
V1
Contribution (% of total JSD = 0.0621 bit)

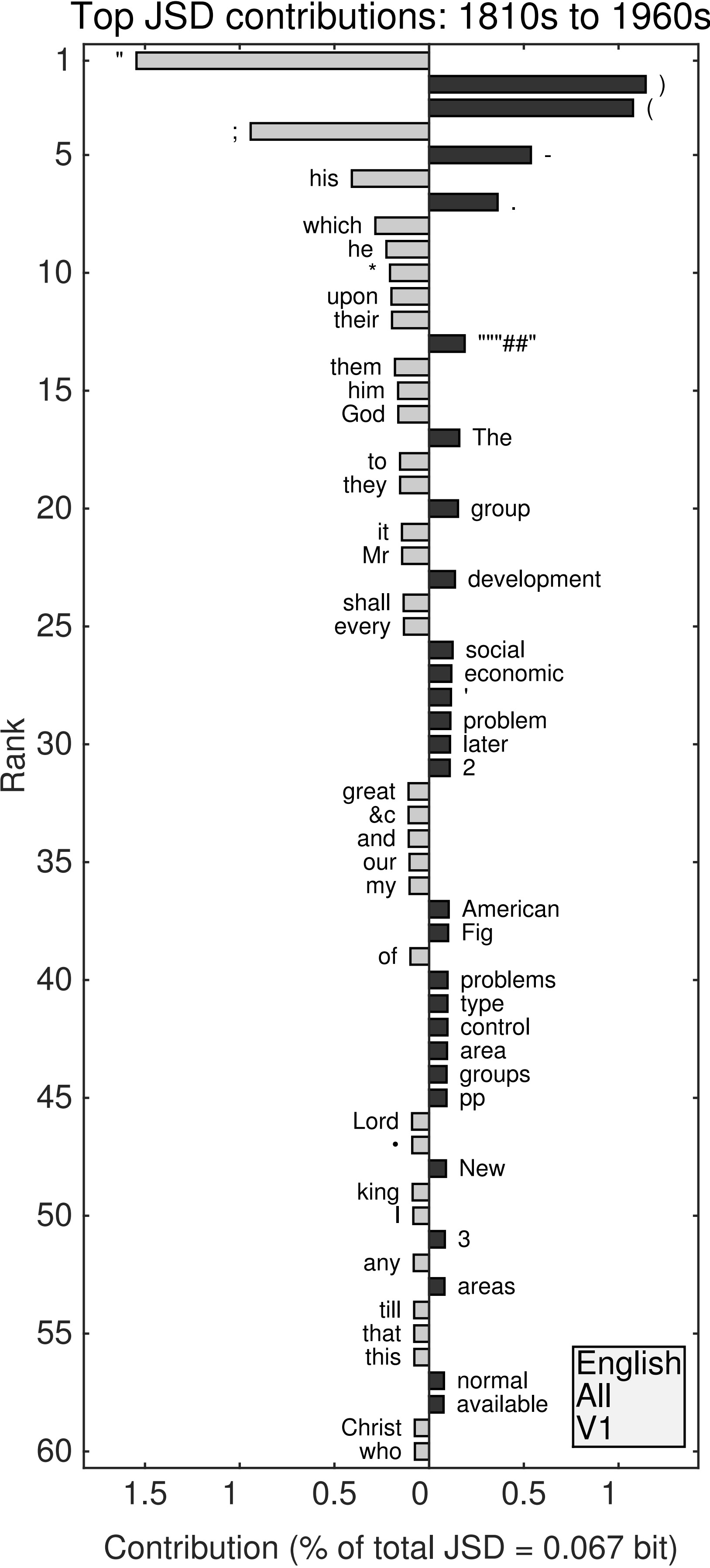
Top JSD contributions: 1810s to 1960s
Rank
Contribution (% of total JSD = 0.067 bit)
"
)
(
;
-
his
.
which
he
*
upon
their
"""##"
them
him
God
The
to
they
group
it
Mr
development
shall
every
social
economic
'
problem
later
2
great
&c
and
our
my
American
Fig
of
problems
type
control
area
groups
pp
Lord
•
New
king
I
3
any
areas
till
that
this
normal
available
Christ
who
English
All
V1

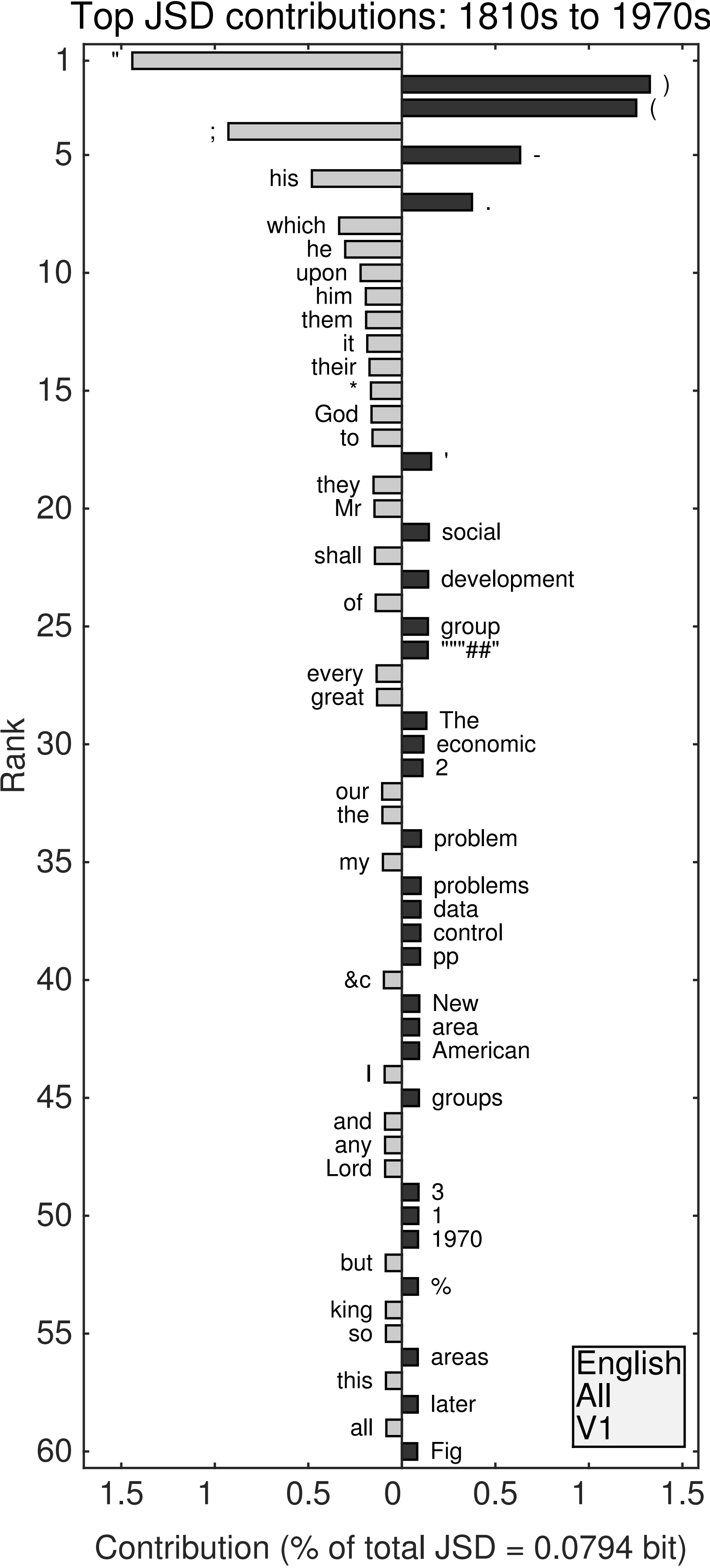

Top JSD contributions: 1810s to 1970s
Rank
Contribution (% of total JSD = 0.0794 bit)
English
All
V1
"
)
(
;
-
his
.
which
he
upon
him
them
it
their
*
God
to
'
they
Mr
social
shall
development
of
group
"""##"
every
great
The
economic
2
our
the
problem
my
problems
data
control
pp
&c
New
area
American
I
groups
and
any
Lord
3
1
1970
but
%
king
so
areas
this
later
all
Fig

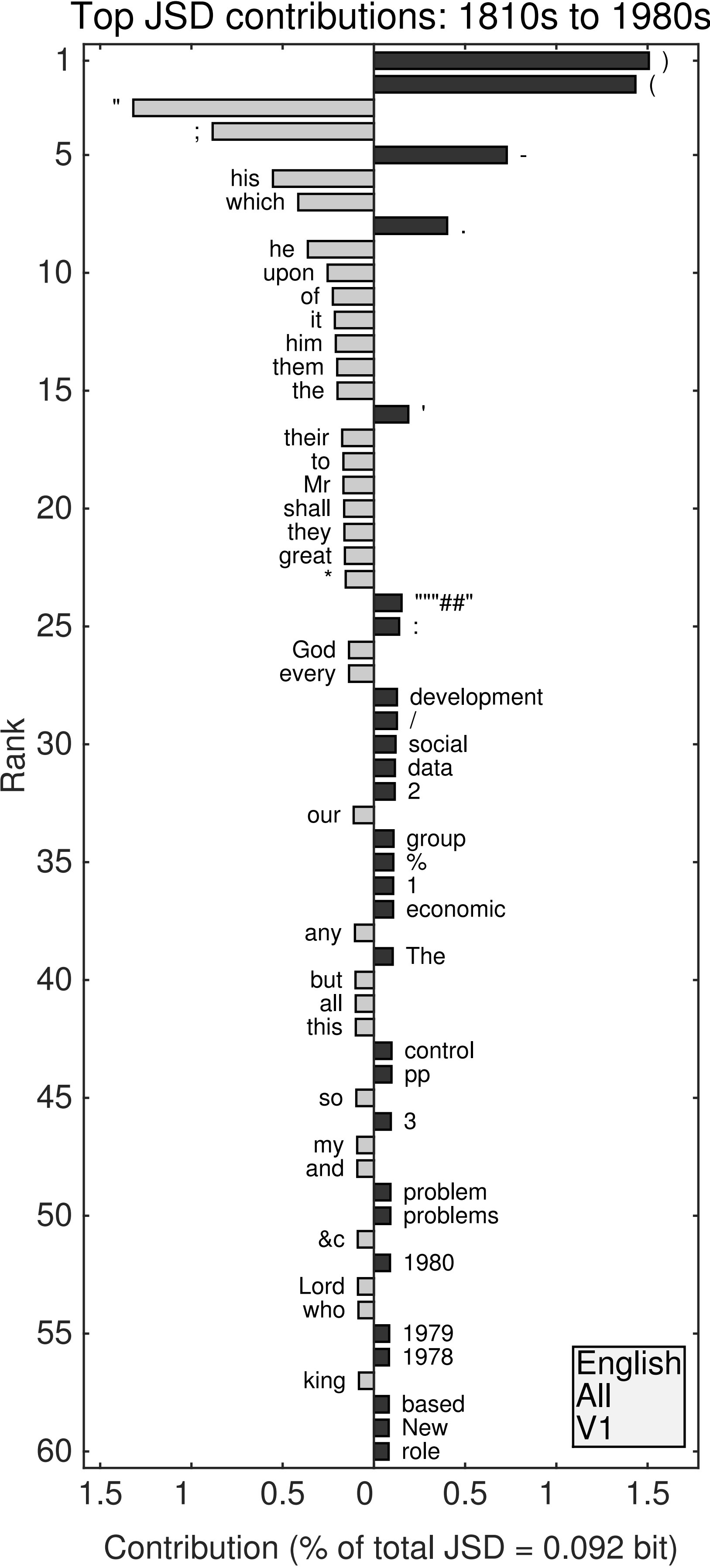

Top JSD contributions: 1810s to 1980s
Rank
Contribution (% of total JSD = 0.092 bit)
)
(
"
;
-
his
which
.
he
upon
of
it
him
them
the
'
their
to
Mr
shall
they
great
*
"""##"
:
God
every
development
/
social
data
2
our
group
%
1
economic
any
The
but
all
this
control
pp
so
3
my
and
problem
problems
&c
1980
Lord
who
1979
1978
king
based
New
role
English
All
V1

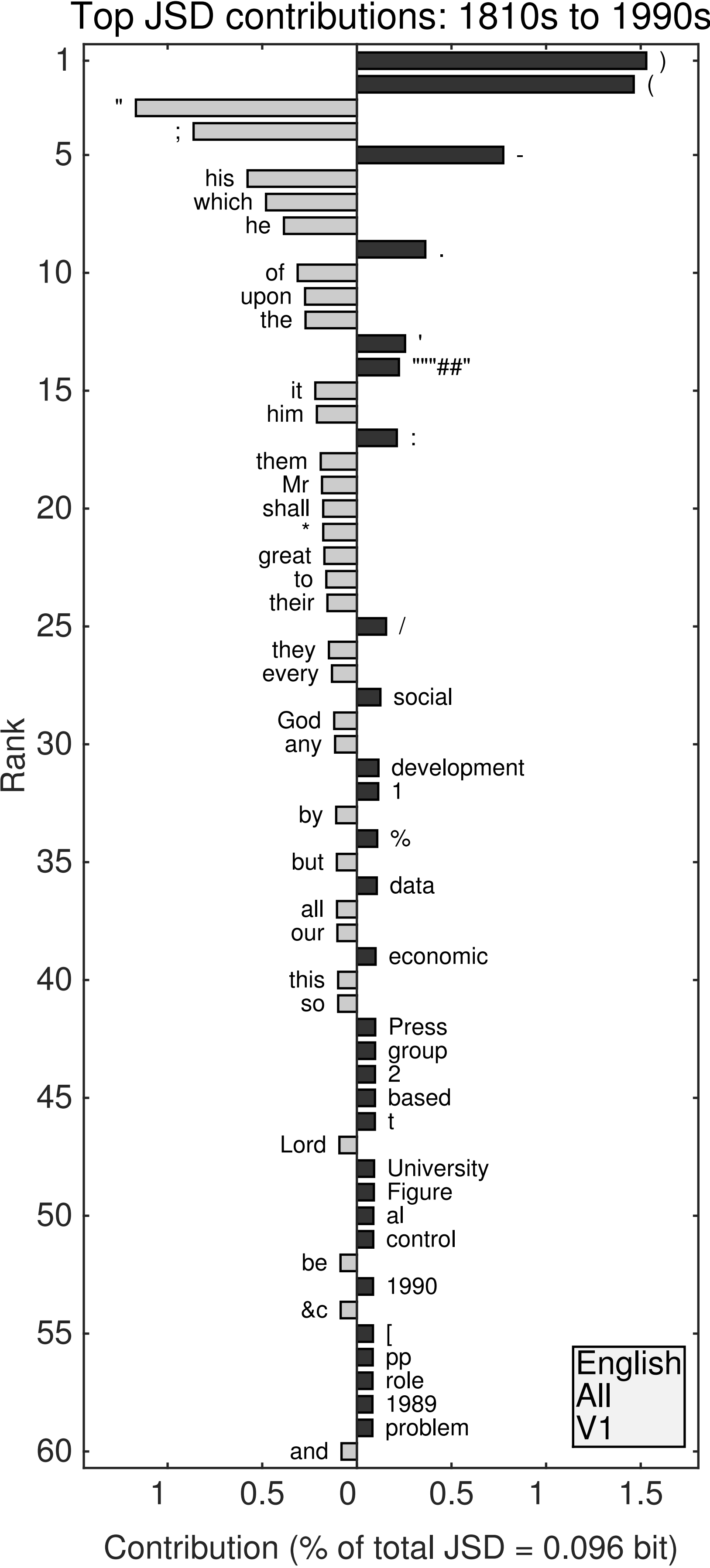

Top JSD contributions: 1810s to 1990s
Rank
Contribution (% of total JSD = 0.096 bit)
1
5
10
15
20
25
30
35
40
45
50
55
60
1
0.5
0
0.5
1
1.5
)
(
"
;
-
his
which
he
.
of
upon
the
'
"""##"
it
him
:
them
Mr
shall
*
great
to
their
/
they
every
social
God
any
development
1
by
%
but
data
all
our
economic
this
so
Press
group
2
based
t
Lord
University
Figure
al
control
be
1990
&c
[
pp
role
1989
problem
and
English
All
V1

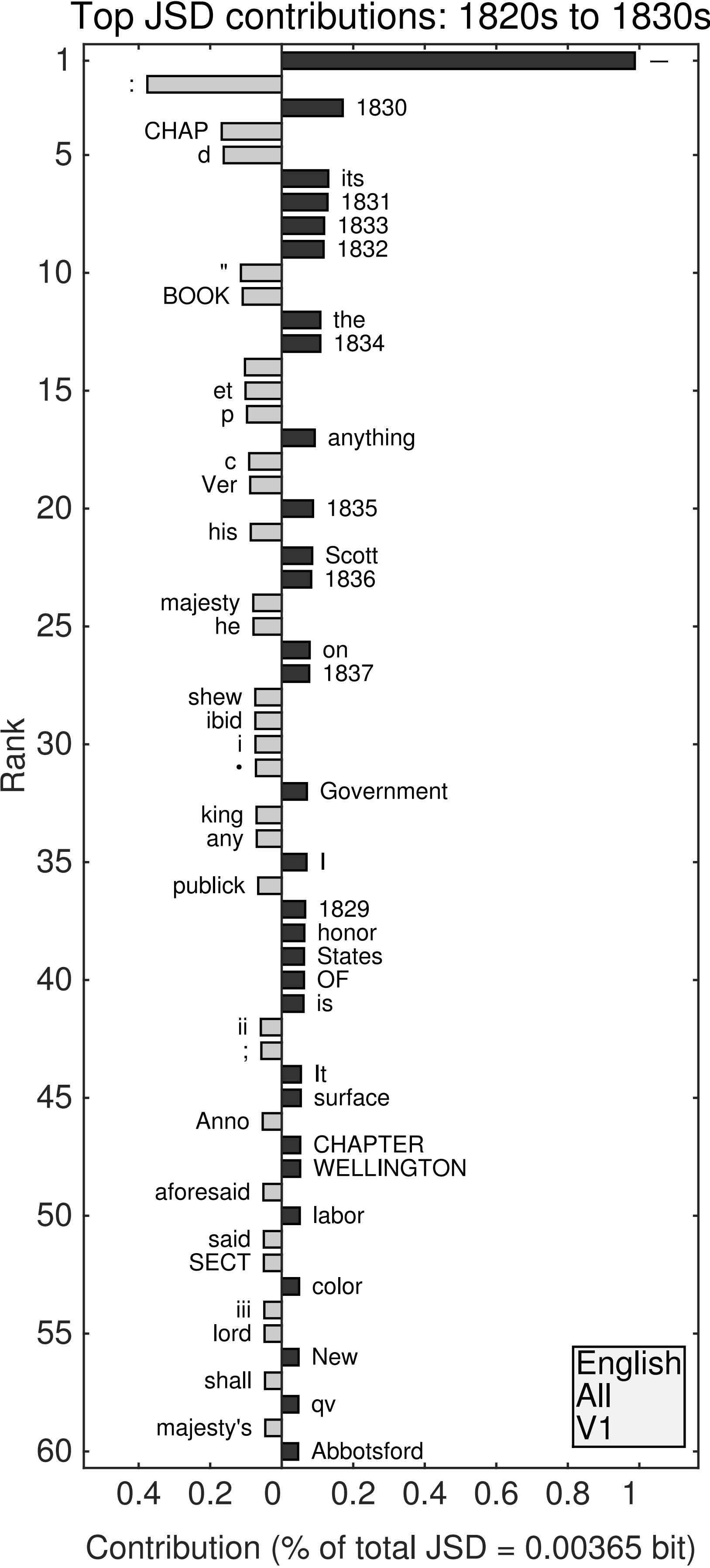
Top JSD contributions: 1820s to 1830s
Rank
Contribution (% of total JSD = 0.00365 bit)
English
All
V1
—
:
1830
CHAP
d
its
1831
1833
1832
"
BOOK
the
1834
et
p
anything
c
Ver
1835
his
Scott
1836
majesty
he
on
1837
shew
ibid
i
•
Government
king
any
I
publick
1829
honor
States
OF
is
ii
;
It
surface
Anno
CHAPTER
WELLINGTON
aforesaid
labor
said
SECT
color
iii
lord
New
shall
qv
majesty's
Abbotsford

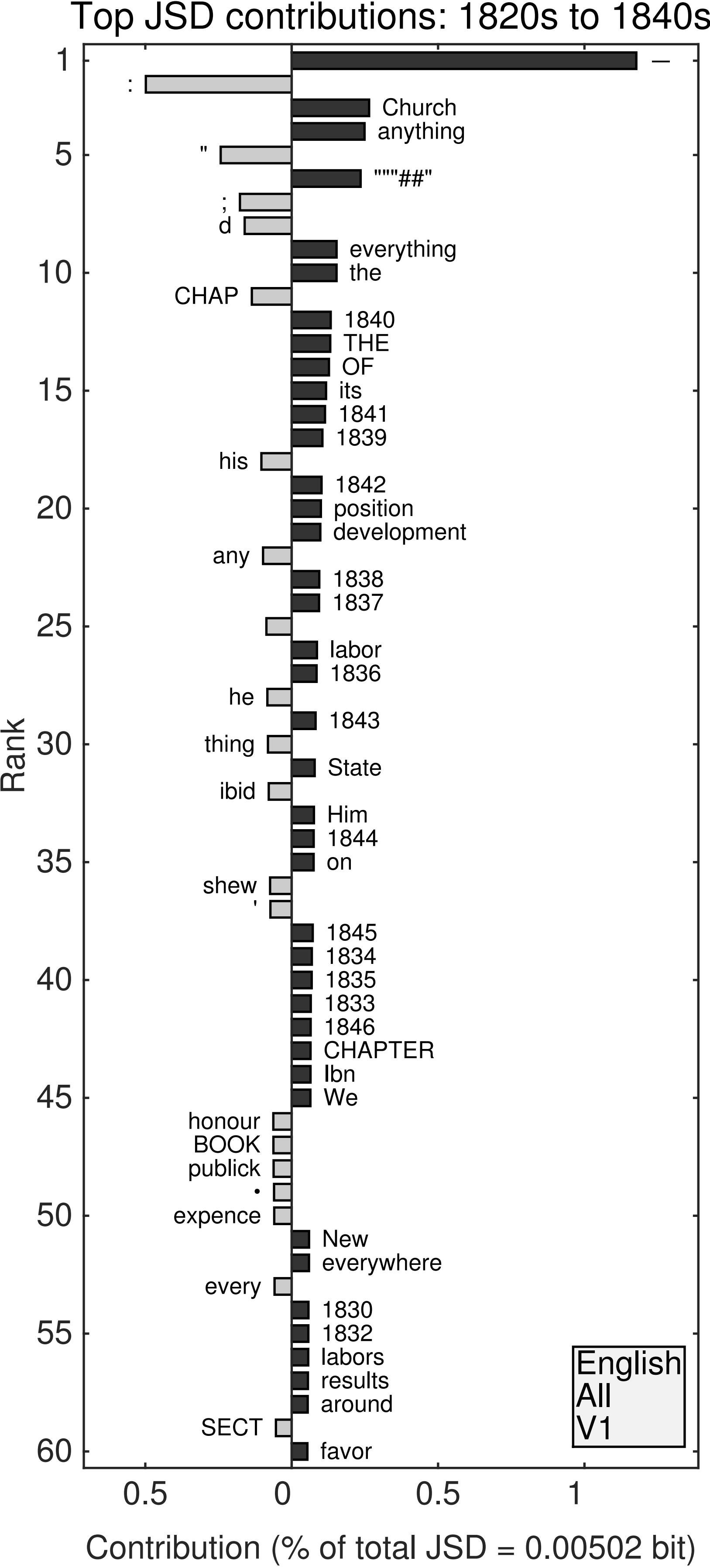
Top JSD contributions: 1820s to 1840s
Rank
Contribution (% of total JSD = 0.00502 bit)
English
All
V1
—
:
Church
anything
"
"""##"
;
d
everything
the
CHAP
1840
THE
OF
its
1841
1839
his
1842
position
development
any
1838
1837
labor
1836
he
1843
thing
State
ibid
Him
1844
on
shew
'
1845
1834
1835
1833
1846
CHAPTER
Ibn
We
honour
BOOK
publick
•
expence
New
everywhere
every
1830
1832
labors
results
around
SECT
favor

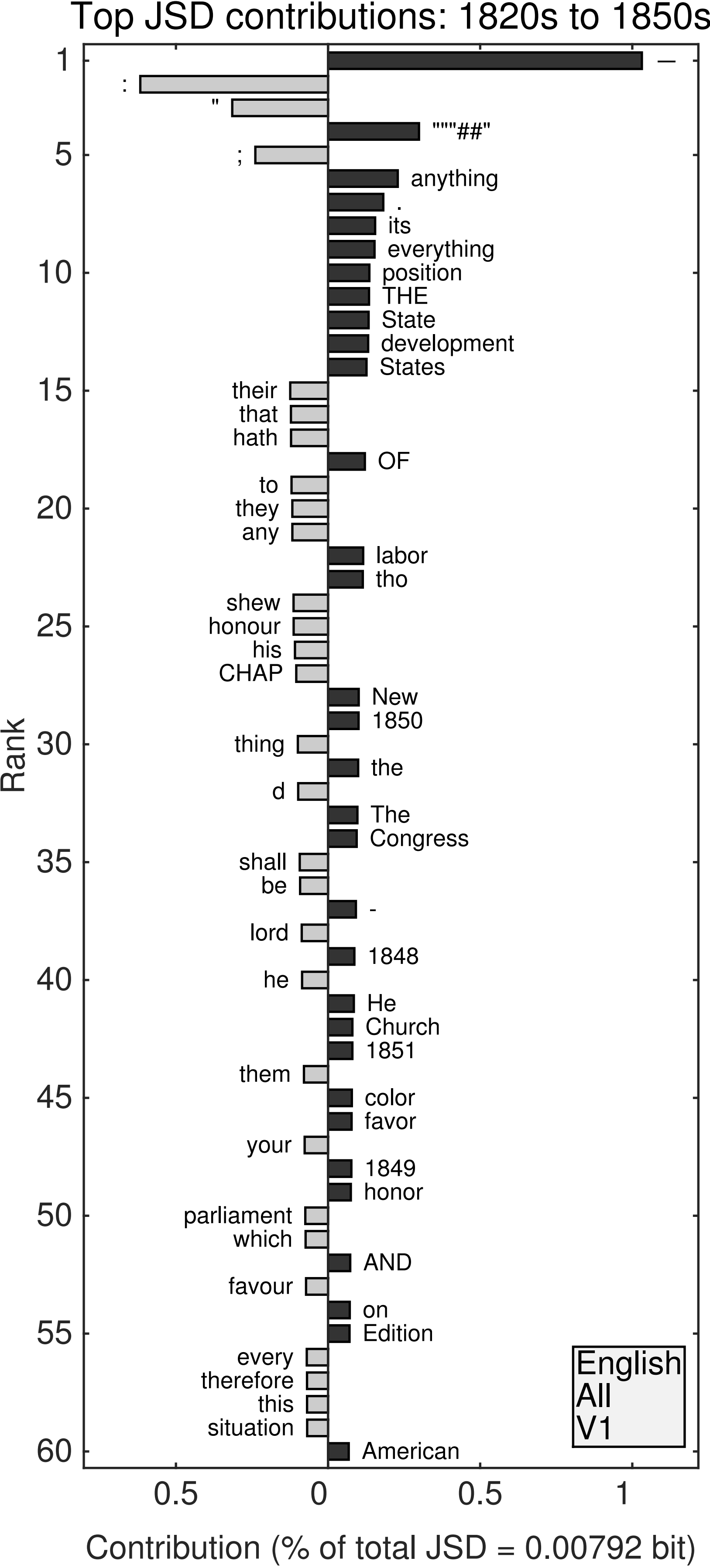
Top JSD contributions: 1820s to 1850s
Rank
Contribution (% of total JSD = 0.00792 bit)
—
:
"
"""##"
;
anything
.
its
everything
position
THE
State
development
States
their
that
hath
OF
to
they
any
labor
tho
shew
honour
his
CHAP
New
1850
thing
the
d
The
Congress
shall
be
-
lord
1848
he
He
Church
1851
them
color
favor
your
1849
honor
parliament
which
AND
favour
on
Edition
every
therefore
this
situation
American
English
All
V1

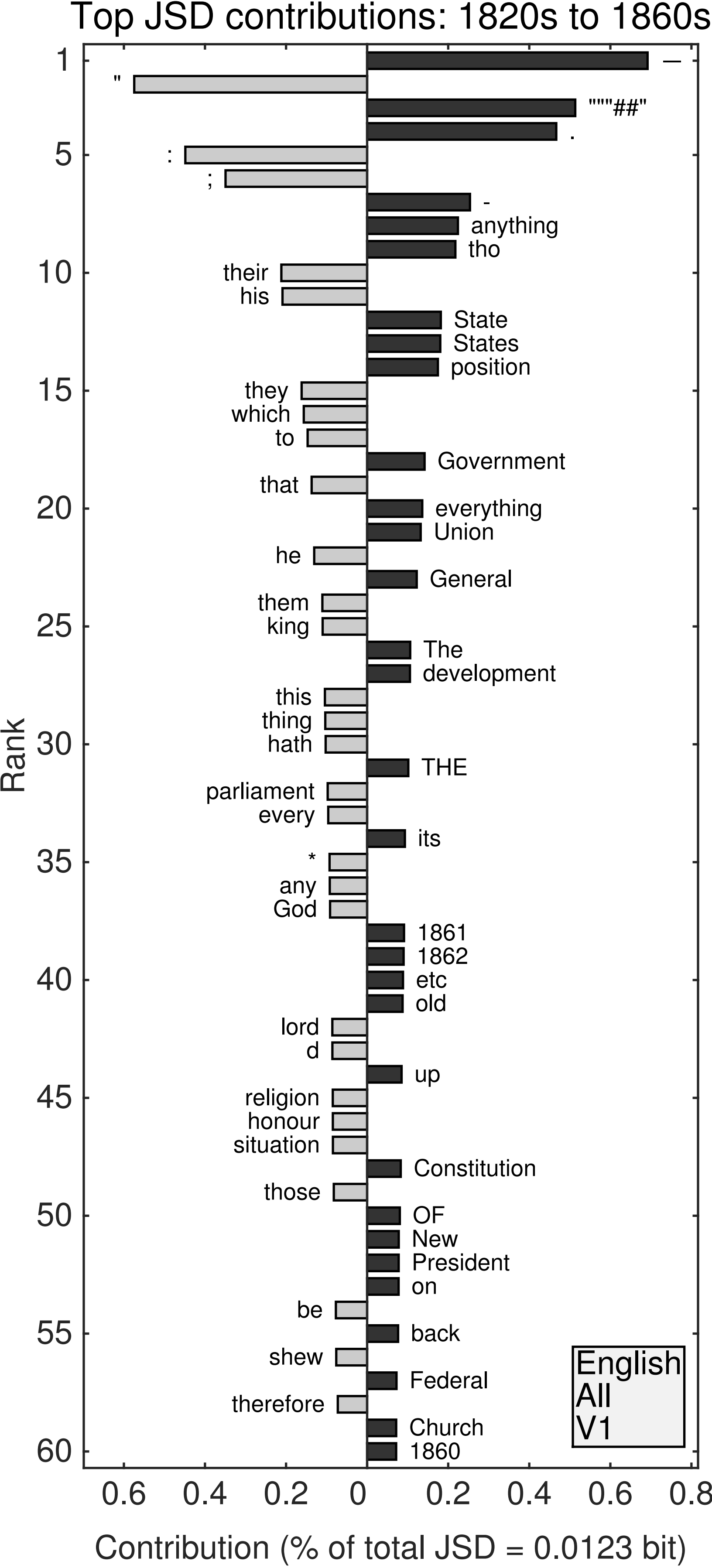
Top JSD contributions: 1820s to 1860s
Rank
Contribution (% of total JSD = 0.0123 bit)
1
5
10
15
20
25
30
35
40
45
50
55
60
0.6
0.4
0.2
0
0.2
0.4
0.6
0.8
—
"
"""##"
.
:
;
-
anything
tho
their
his
State
States
position
they
which
to
Government
that
everything
Union
he
General
them
king
The
development
this
thing
hath
THE
parliament
every
its
*
any
God
1861
1862
etc
old
lord
d
up
religion
honour
situation
Constitution
those
OF
New
President
on
be
back
shew
Federal
therefore
Church
1860
English
All
V1

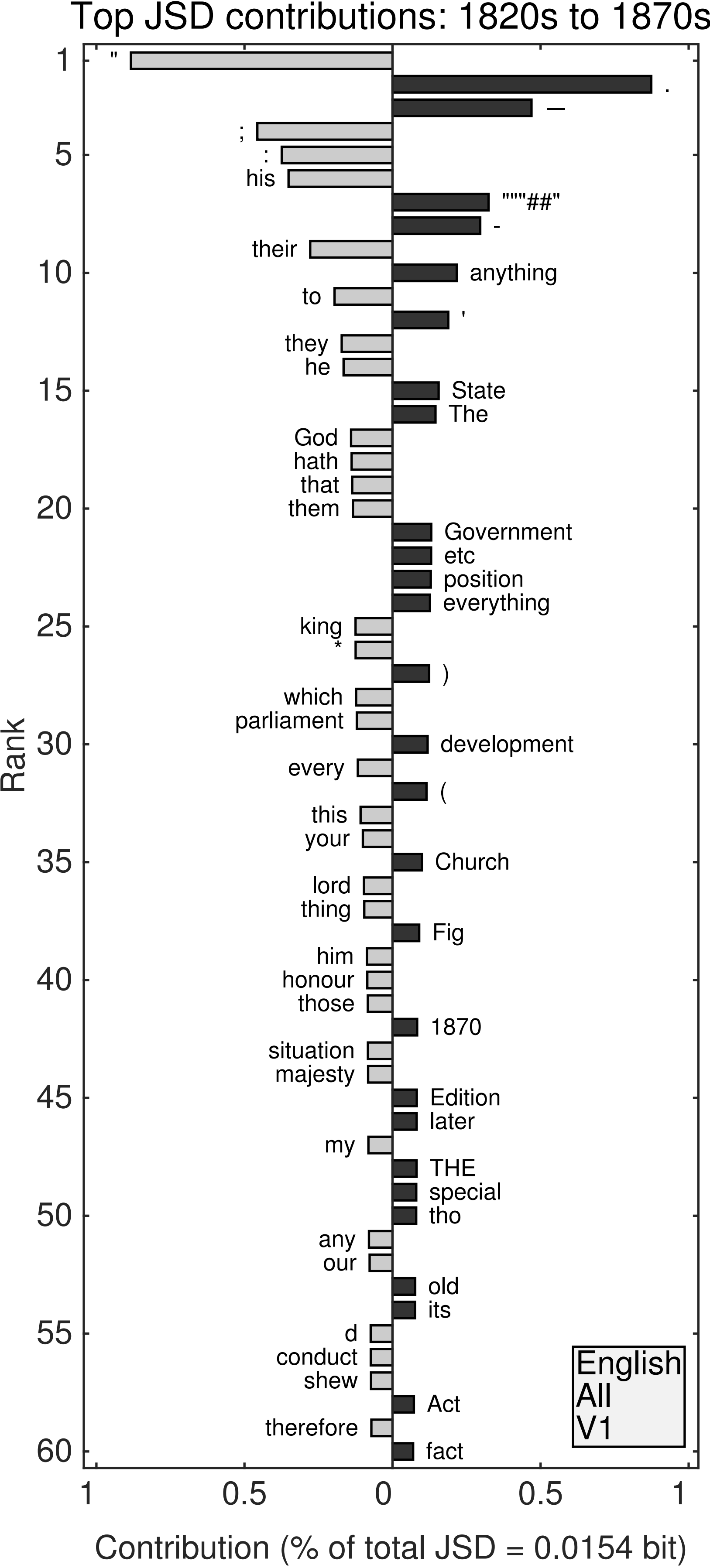

Top JSD contributions: 1820s to 1870s
Rank
Contribution (% of total JSD = 0.0154 bit)
"
.
—
;
:
his
"""##"
-
their
anything
to
'
they
he
State
The
God
hath
that
them
Government
etc
position
everything
king
*
)
which
parliament
development
every
(
this
your
Church
lord
thing
Fig
him
honour
those
1870
situation
majesty
Edition
later
my
THE
special
tho
any
our
old
its
d
conduct
shew
Act
therefore
fact
English
All
V1

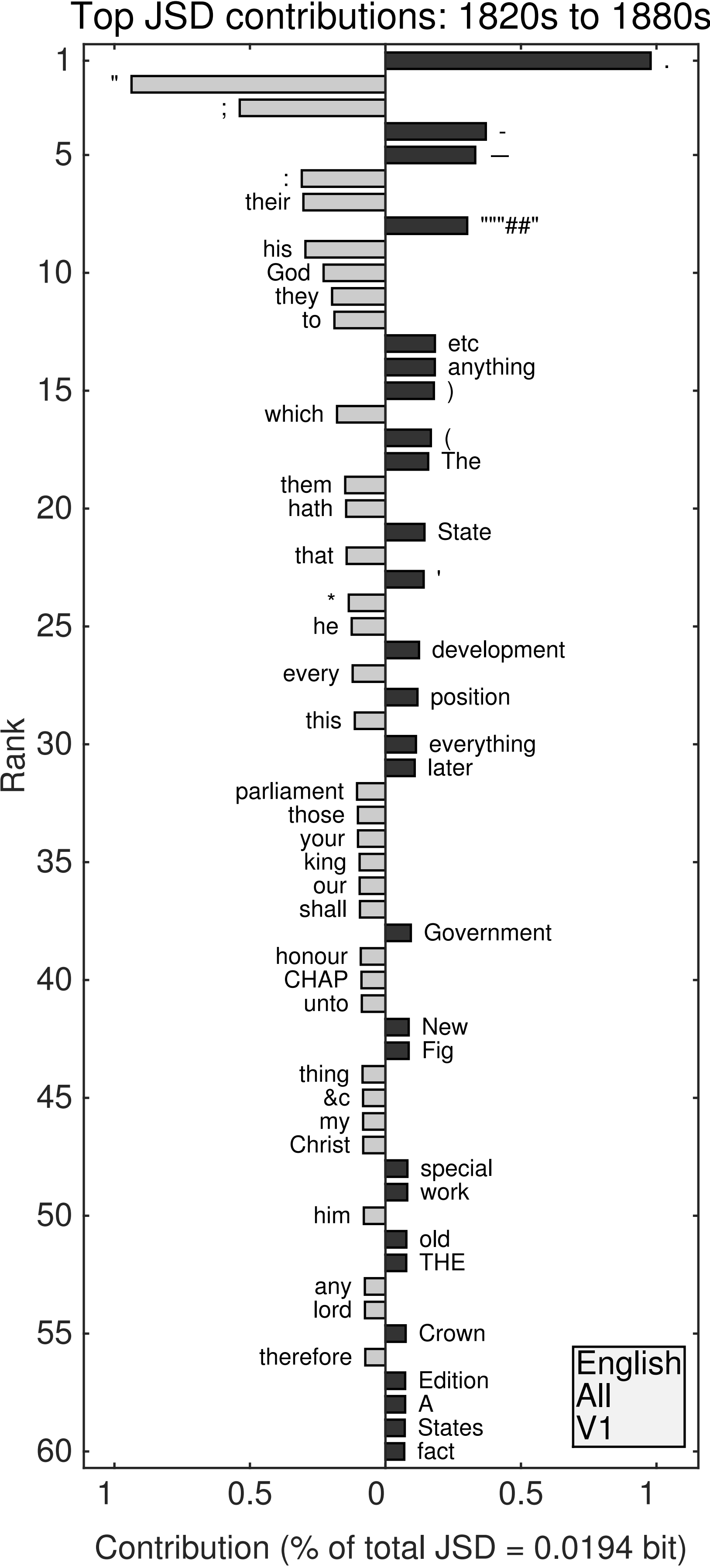
Top JSD contributions: 1820s to 1880s
Rank
Contribution (% of total JSD = 0.0194 bit)
1
5
10
15
20
25
30
35
40
45
50
55
60
1
0.5
0
0.5
1
.
"
;
-
—
:
their
"""##"
his
God
they
to
etc
anything
)
which
(
The
them
hath
State
that
'
*
he
development
every
position
this
everything
later
parliament
those
your
king
our
shall
Government
honour
CHAP
unto
New
Fig
thing
&c
my
Christ
special
work
him
old
THE
any
lord
Crown
therefore
Edition
A
States
fact
English
All
V1

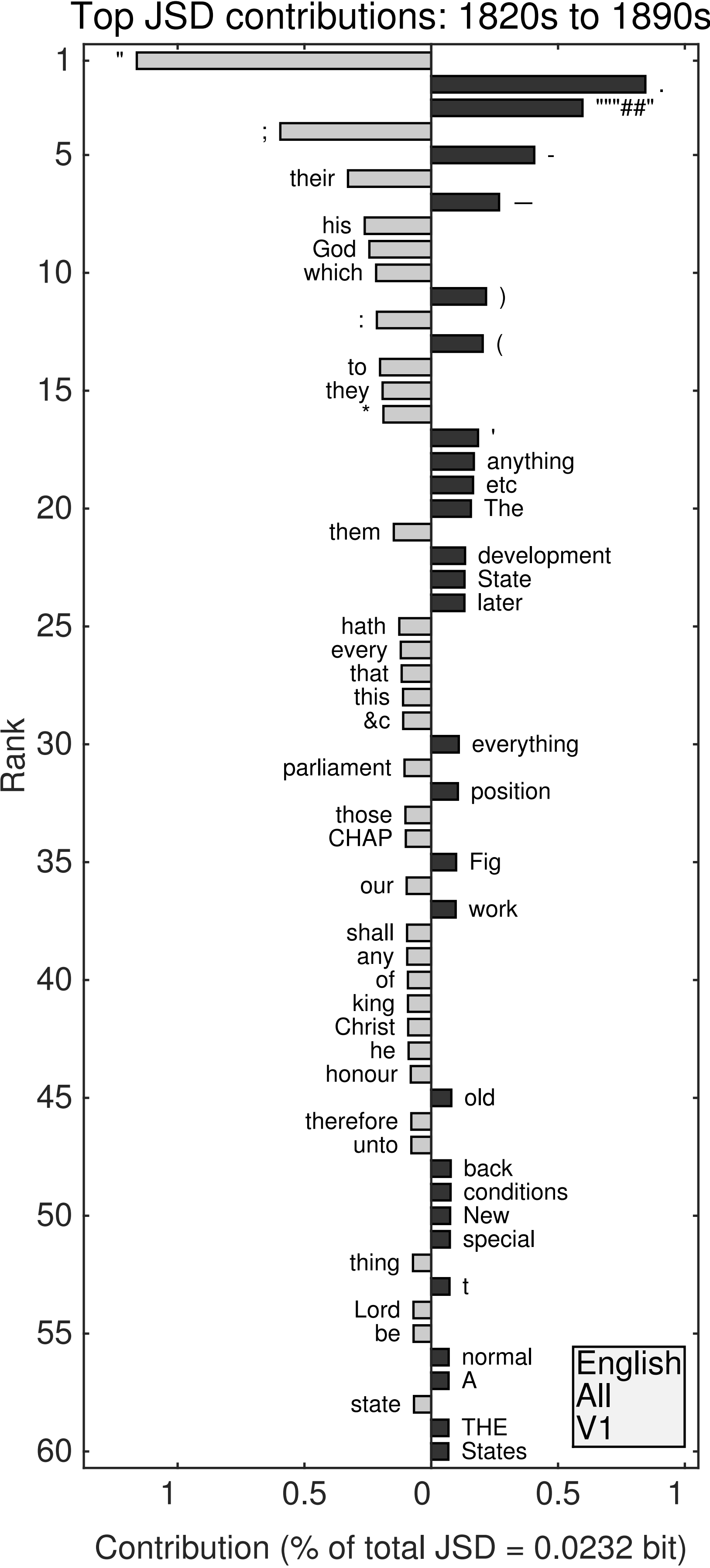

Top JSD contributions: 1820s to 1890s
Rank
Contribution (% of total JSD = 0.0232 bit)
"
.
"""##"
;
-
their
—
his
God
which
)
:
(
to
they
*
'
anything
etc
The
them
development
State
later
hath
every
that
this
&c
everything
parliament
position
those
CHAP
Fig
our
work
shall
any
of
king
Christ
he
honour
old
therefore
unto
back
conditions
New
special
thing
t
Lord
be
normal
A
state
THE
States
English
All
V1

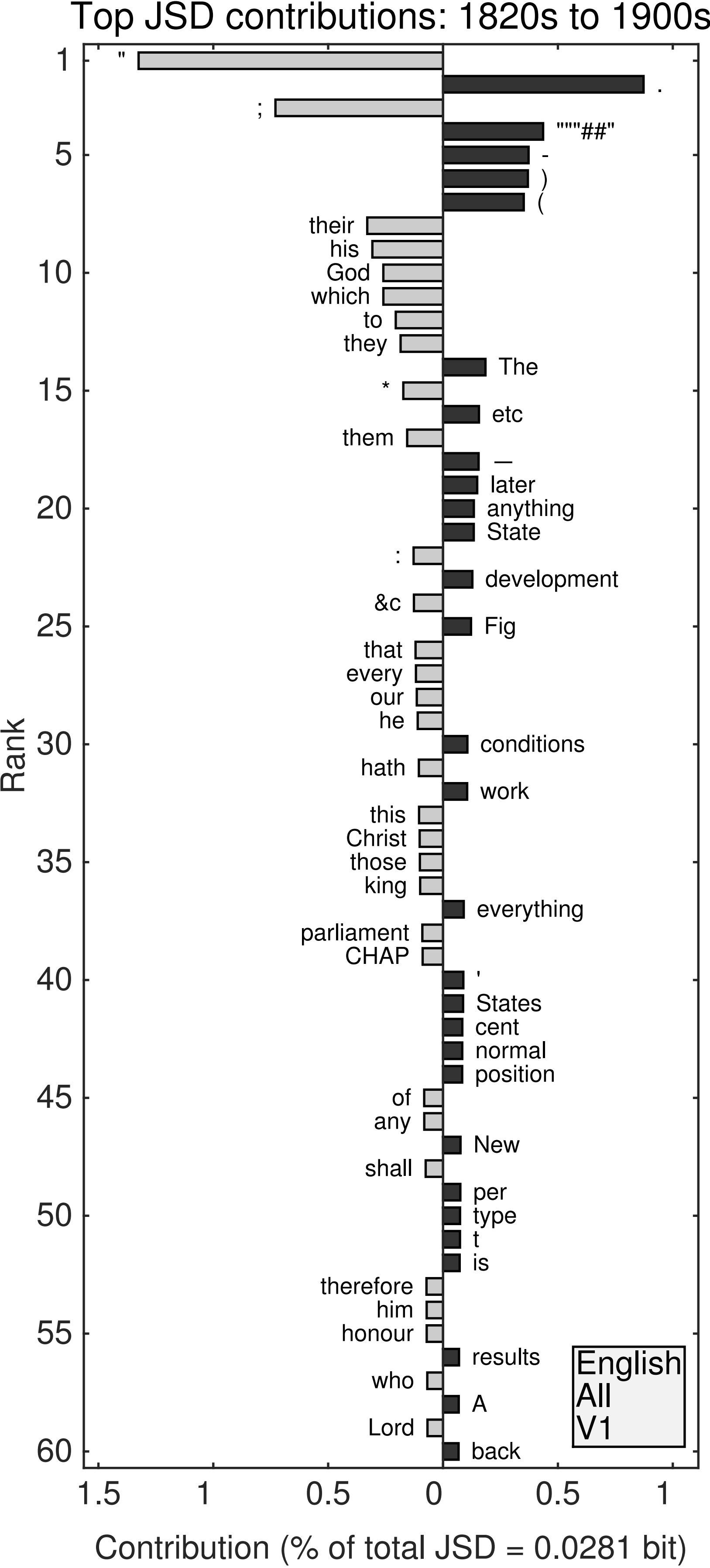

Top JSD contributions: 1820s to 1900s
Rank
Contribution (% of total JSD = 0.0281 bit)
1.5
1
0.5
0
0.5
1
1
5
10
15
20
25
30
35
40
45
50
55
60
"
.
;
"""##"
-
)
(
their
his
God
which
to
they
The
*
etc
them
—
later
anything
State
:
development
&c
Fig
that
every
our
he
conditions
hath
work
this
Christ
those
king
everything
parliament
CHAP
'
States
cent
normal
position
of
any
New
shall
per
type
t
is
therefore
him
honour
results
who
A
Lord
back
English
All
V1

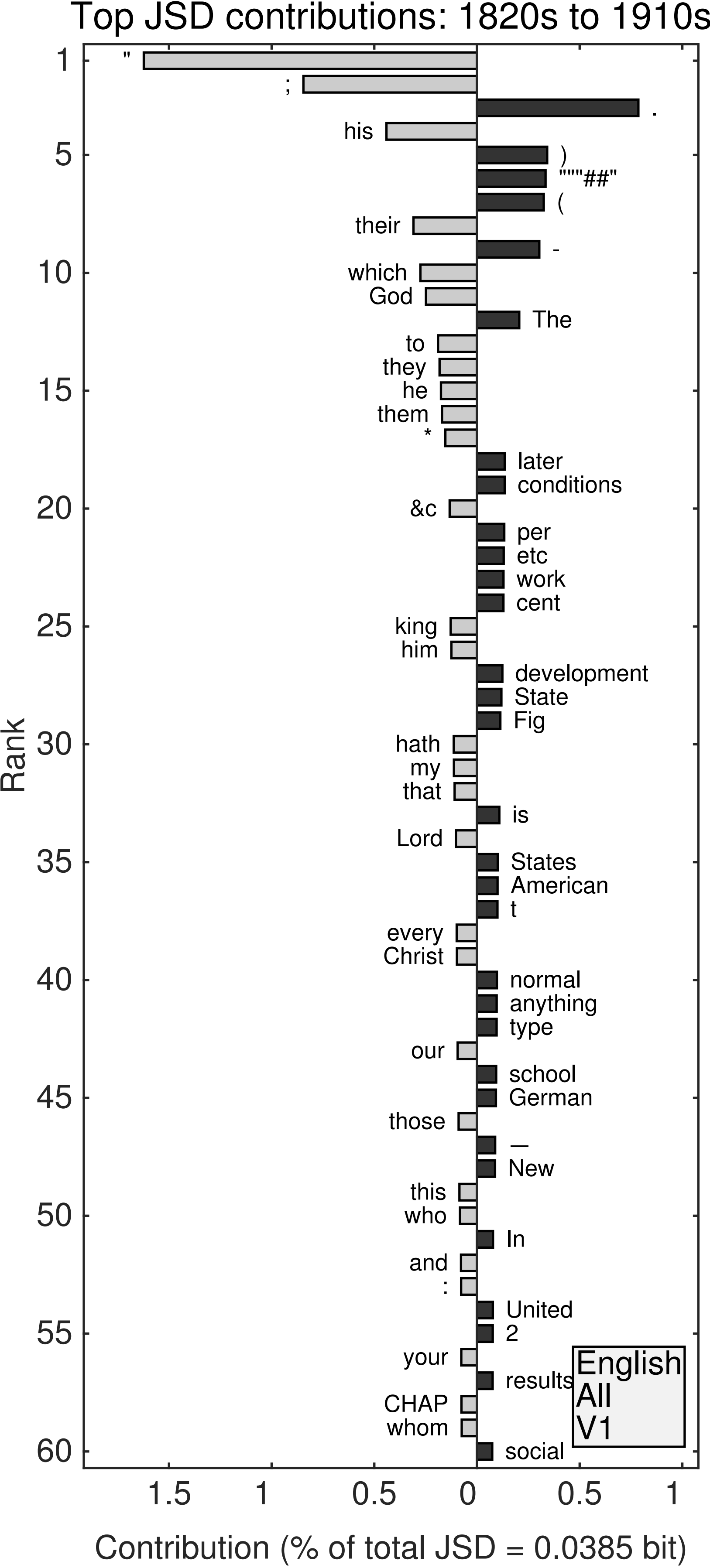

Top JSD contributions: 1820s to 1910s
Rank
1
5
10
15
20
25
30
35
40
45
50
55
60
"
;
.
his
)
"""##"
(
their
-
which
God
The
to
they
he
them
*
later
conditions
&c
per
etc
work
cent
king
him
development
State
Fig
hath
my
that
is
Lord
States
American
t
every
Christ
normal
anything
type
our
school
German
those
—
New
this
who
In
and
:
United
2
your
results
CHAP
whom
social
English
All
V1
1.5
1
0.5
0
0.5
1
Contribution (% of total JSD = 0.0385 bit)

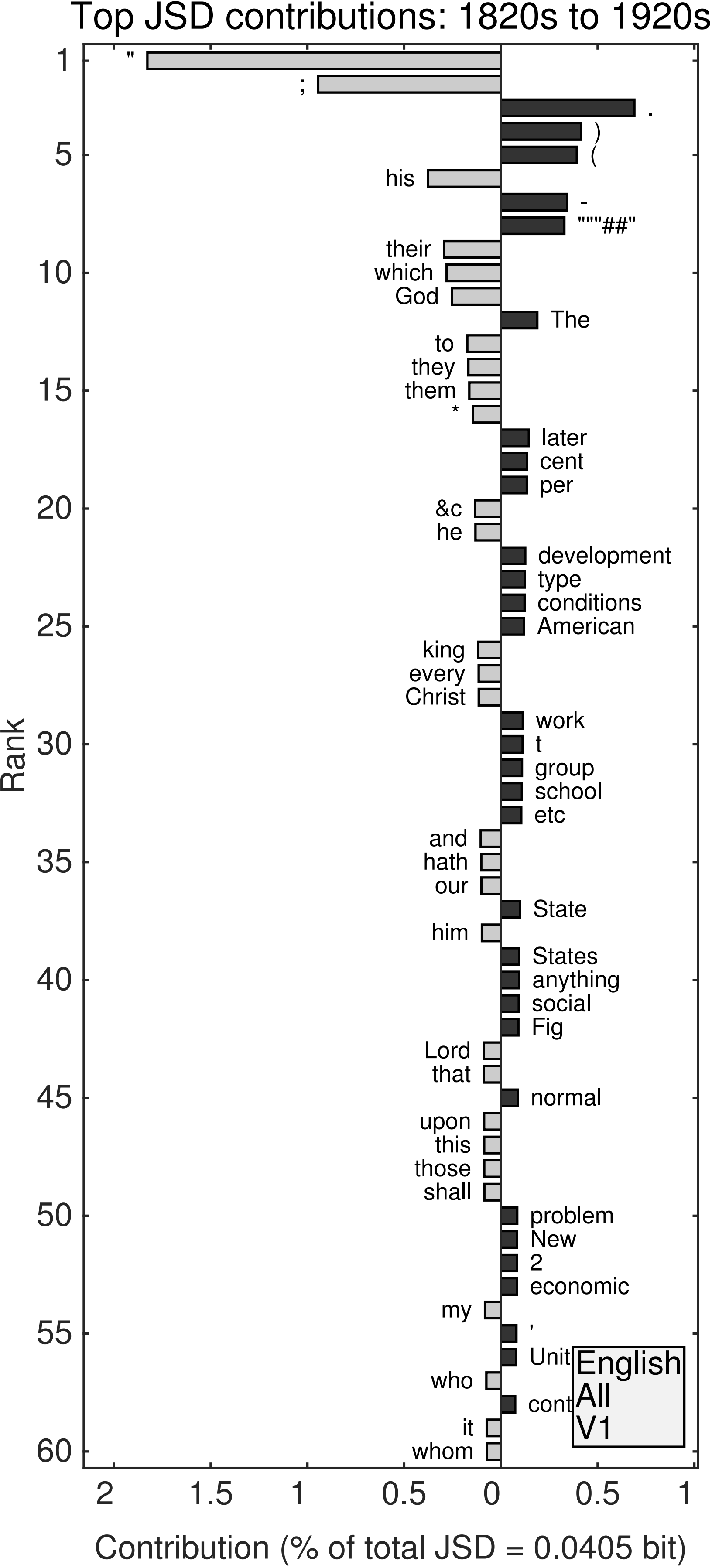

Top JSD contributions: 1820s to 1920s
Rank
Contribution (% of total JSD = 0.0405 bit)
English
All
V1
"
;
.
)
(
his
-
"""##"
their
which
God
The
to
they
them
*
later
cent
per
&c
he
development
type
conditions
American
king
every
Christ
work
t
group
school
etc
and
hath
our
State
him
States
anything
social
Fig
Lord
that
normal
upon
this
those
shall
problem
New
2
economic
my
'
Unit
who
cont
it
whom

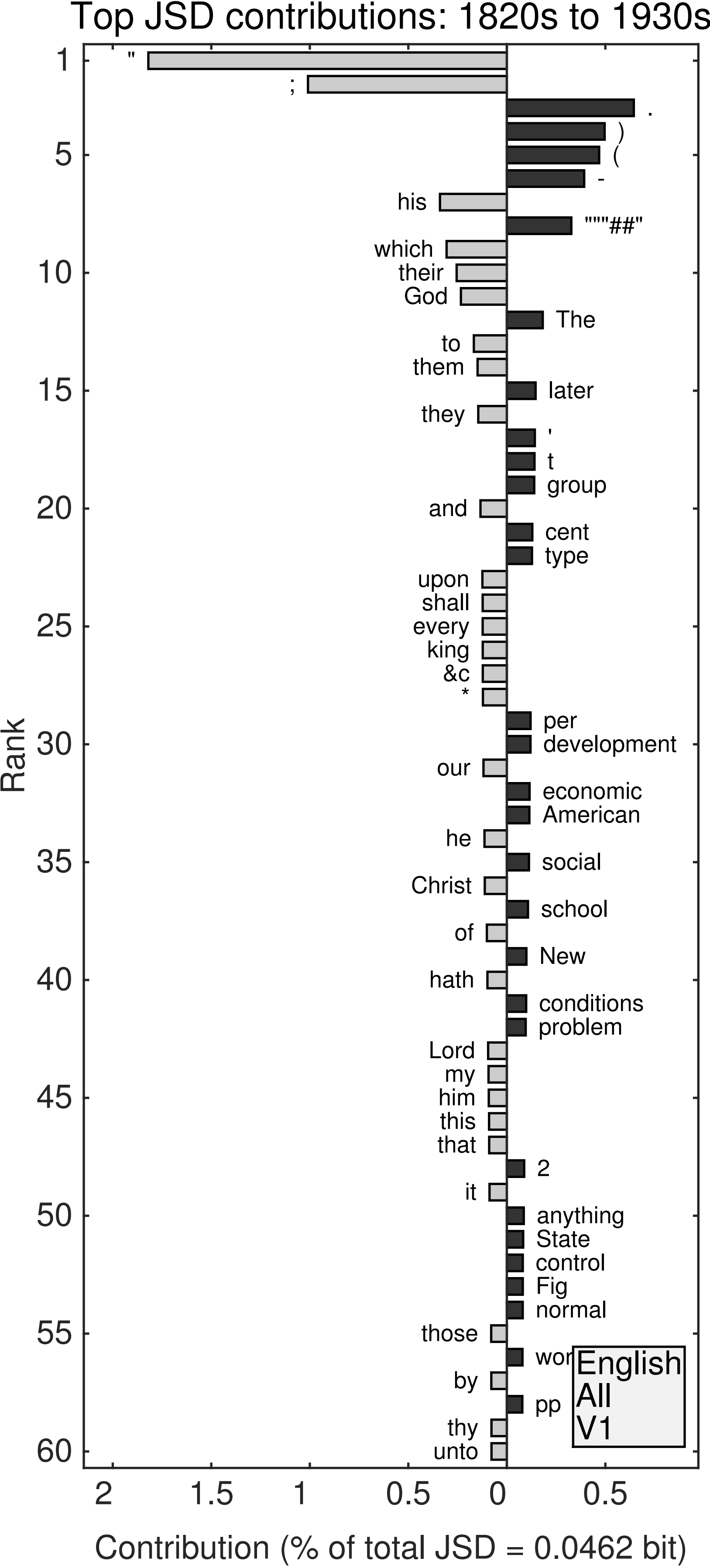

Top JSD contributions: 1820s to 1930s
Rank
Contribution (% of total JSD = 0.0462 bit)
English
All
V1
"
;
.
)
(
-
his
"""##"
which
their
God
The
to
them
later
they
'
t
group
and
cent
type
upon
shall
every
king
&c
*
per
development
our
economic
American
he
social
Christ
school
of
New
hath
conditions
problem
Lord
my
him
this
that
2
it
anything
State
control
Fig
normal
those
by
pp
thy
unto

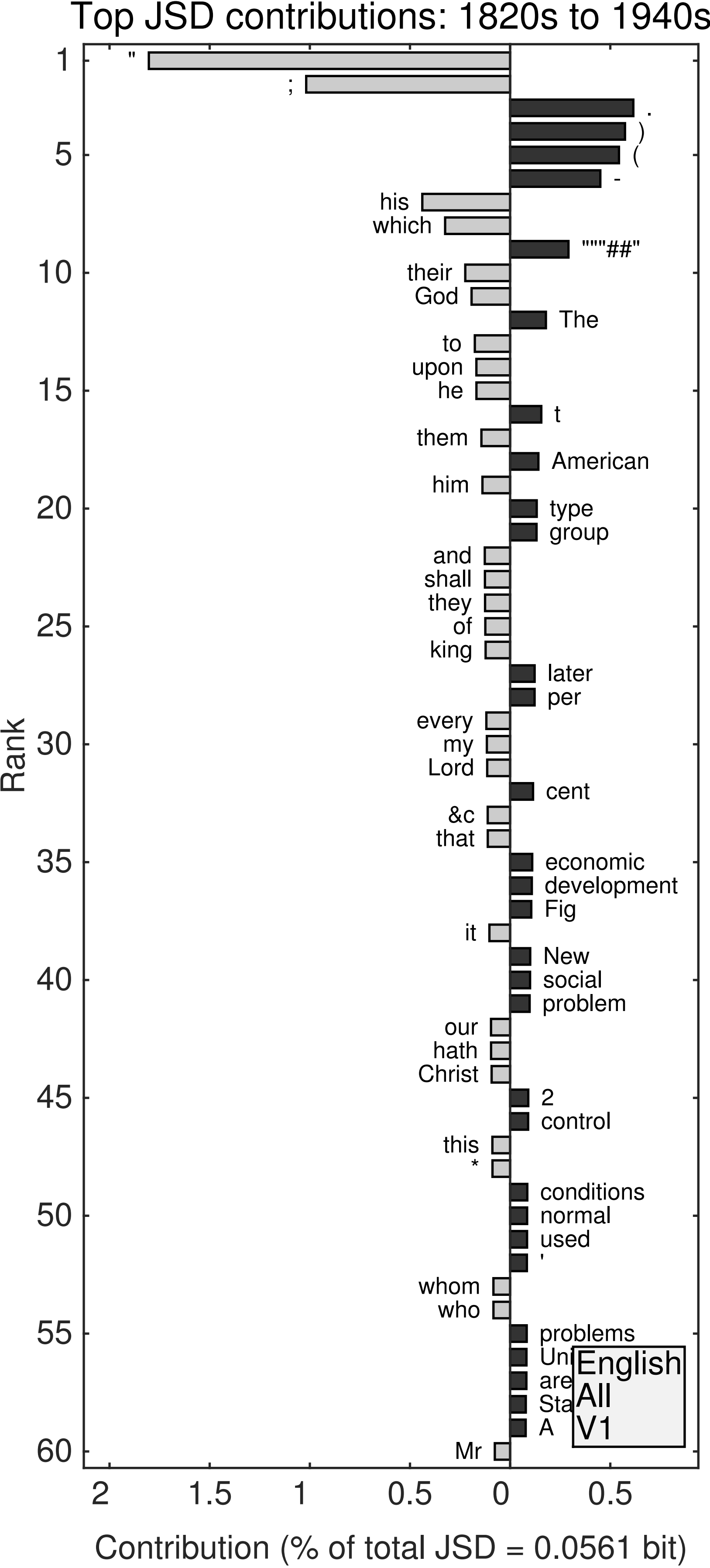

Top JSD contributions: 1820s to 1940s
Rank
1
5
10
15
20
25
30
35
40
45
50
55
60
"
;
.
)
(
-
his
which
"""##"
their
God
The
to
upon
he
t
them
American
him
type
group
and
shall
they
of
king
later
per
every
my
Lord
cent
&c
that
economic
development
Fig
it
New
social
problem
our
hath
Christ
2
control
this
*
conditions
normal
used
'
whom
who
problems
are
A
Mr
English
All
V1
2
1.5
1
0.5
0
0.5
Contribution (% of total JSD = 0.0561 bit)

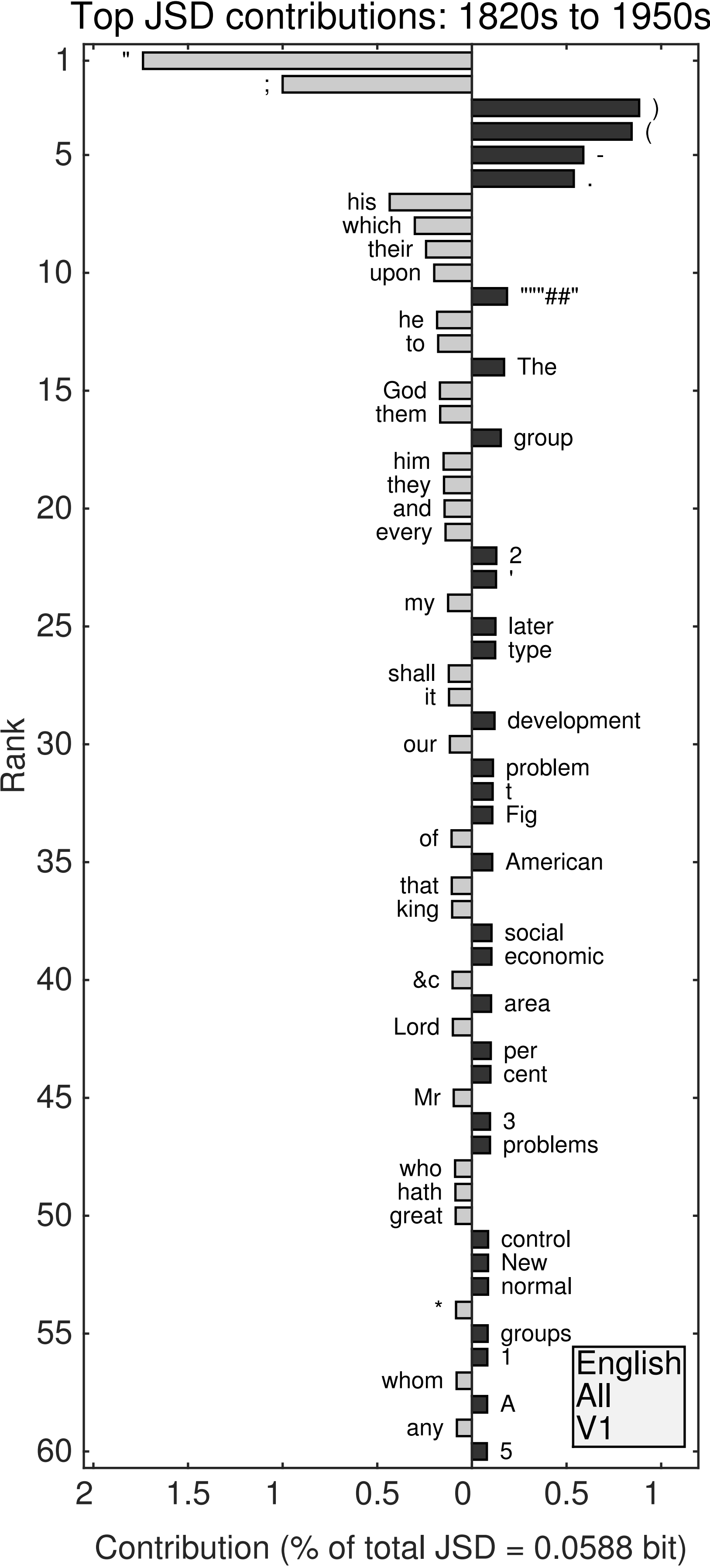

Top JSD contributions: 1820s to 1950s
Rank
Contribution (% of total JSD = 0.0588 bit)
"
;
)
(
-
.
his
which
their
upon
"""##"
he
to
The
God
them
group
him
they
and
every
2
'
my
later
type
shall
it
development
our
problem
t
Fig
of
American
that
king
social
economic
&c
area
Lord
per
cent
Mr
3
problems
who
hath
great
control
New
normal
*
groups
1
whom
A
any
5
English
All
V1

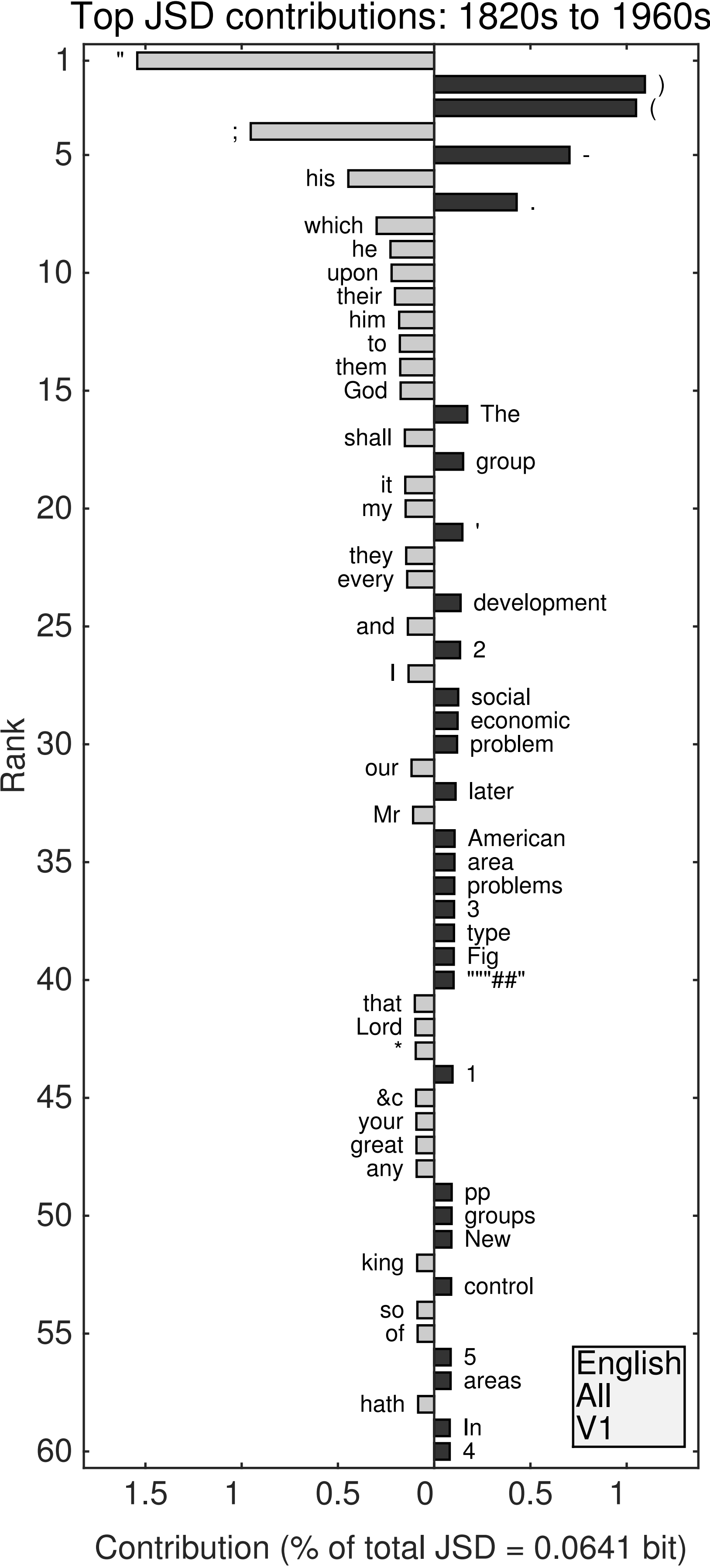

Top JSD contributions: 1820s to 1960s
Rank
Contribution (% of total JSD = 0.0641 bit)
"
)
(
;
-
his
.
which
he
upon
their
him
to
them
God
The
shall
group
it
my
'
they
every
development
and
2
I
social
economic
problem
our
later
Mr
American
area
problems
3
type
Fig
"""##"
that
Lord
*
1
&c
your
great
any
pp
groups
New
king
control
so
of
5
areas
hath
In
4
English
All
V1

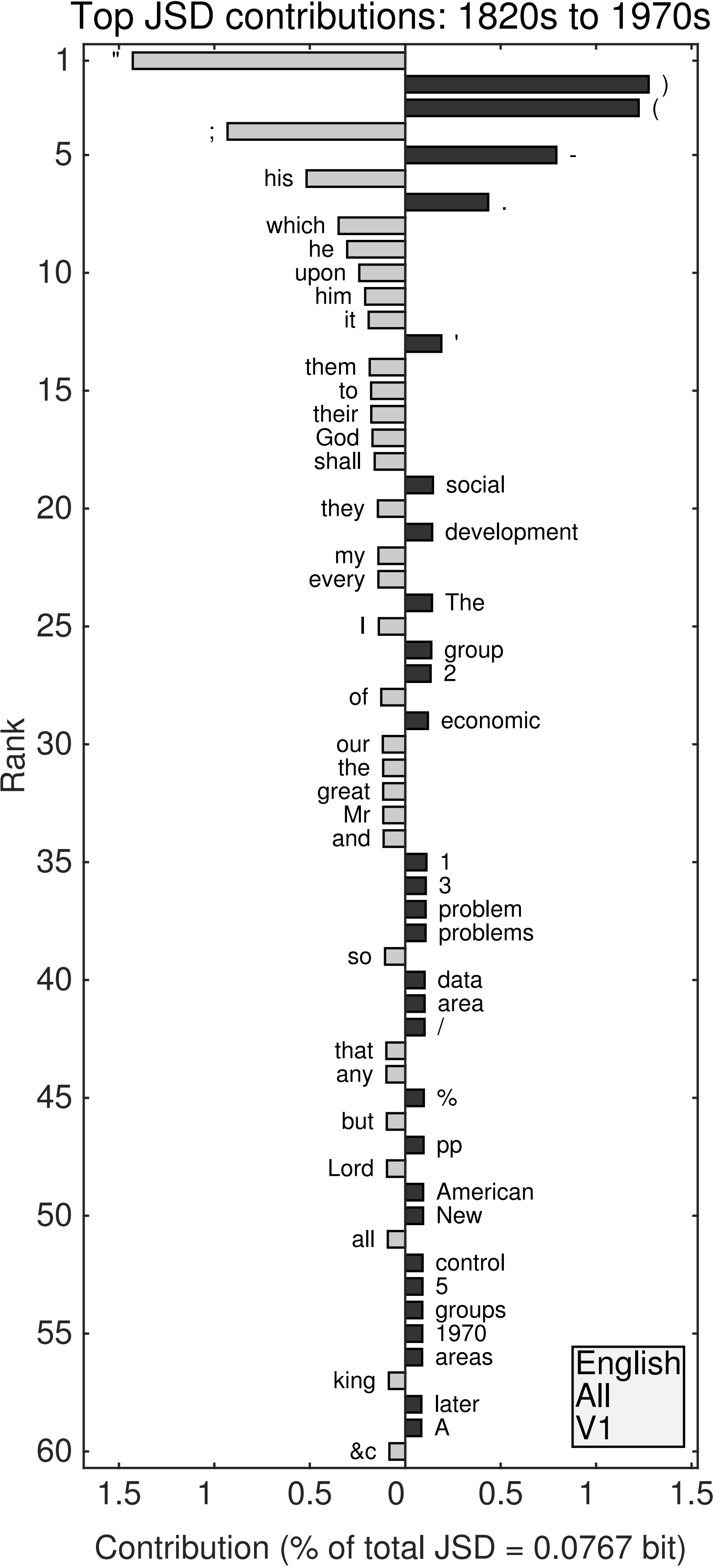

Top JSD contributions: 1820s to 1970s
Rank
Contribution (% of total JSD = 0.0767 bit)
"
)
(
;
-
his
.
which
he
upon
him
it
'
them
to
their
God
shall
social
they
development
my
every
The
I
group
2
of
economic
our
the
great
Mr
and
1
3
problem
problems
so
data
area
/
that
any
%
but
pp
Lord
American
New
all
control
5
groups
1970
areas
king
later
A
&c
English
All
V1

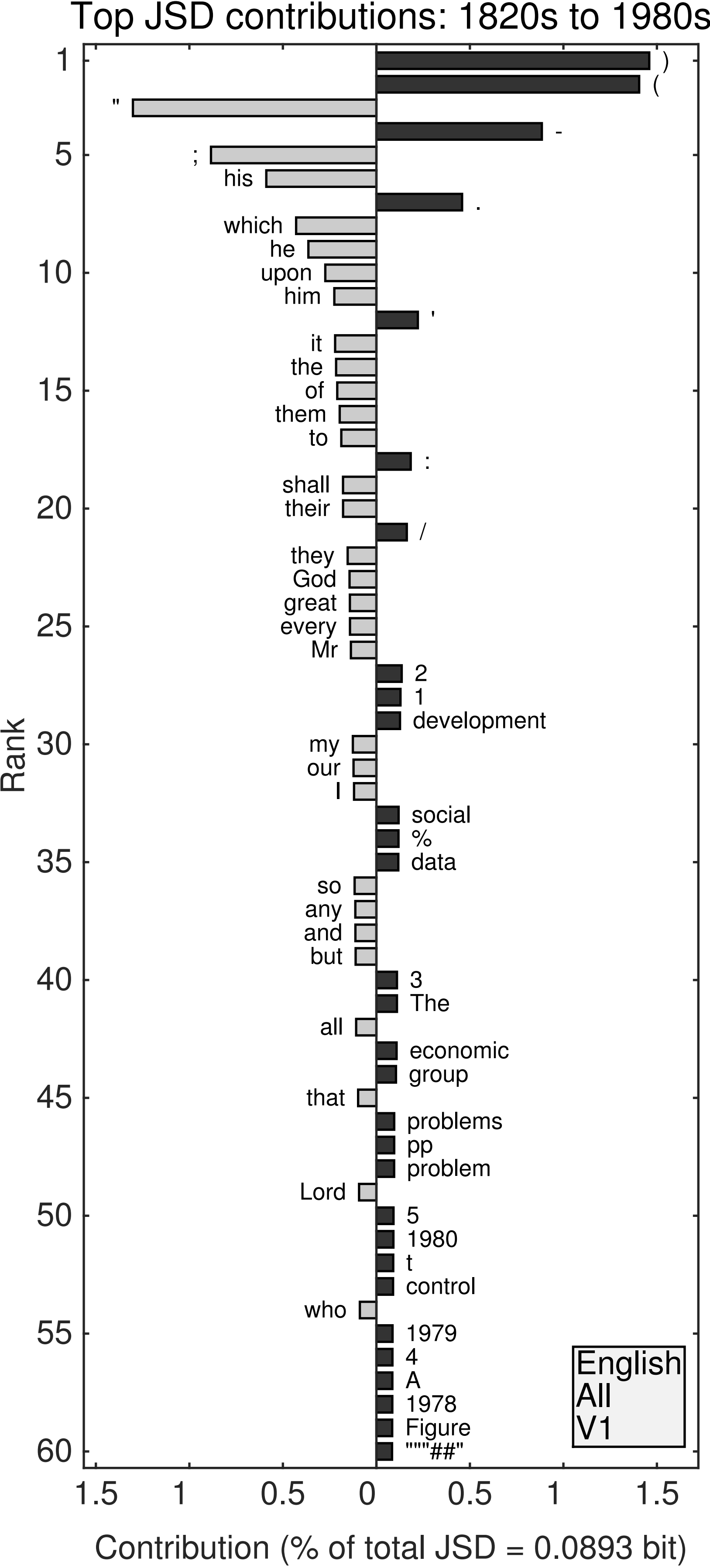
Top JSD contributions: 1820s to 1980s
Rank
Contribution (% of total JSD = 0.0893 bit)
)
(
"
-
;
his
.
which
he
upon
him
'
it
the
of
them
to
:
shall
their
/
they
God
great
every
Mr
2
1
development
my
our
I
social
%
data
so
any
and
but
3
The
all
economic
group
that
problems
pp
problem
Lord
5
1980
t
control
who
1979
4
A
1978
Figure
"""##"
English
All
V1

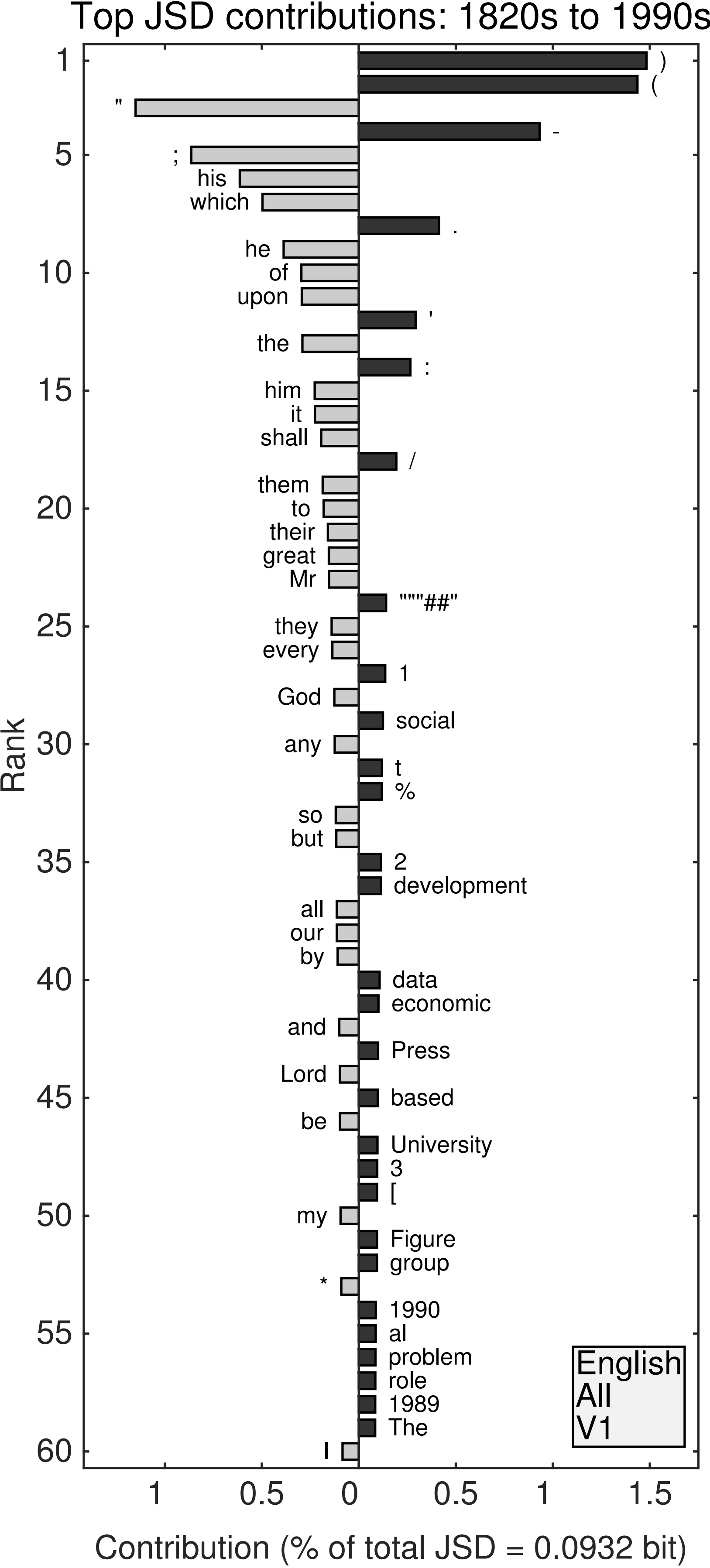

Top JSD contributions: 1820s to 1990s
Rank
Contribution (% of total JSD = 0.0932 bit)
)
(
"
-
;
his
which
.
he
of
upon
'
the
:
him
it
shall
/
them
to
their
great
Mr
"""##"
they
every
1
God
social
any
t
%
so
but
2
development
all
our
by
data
economic
and
Press
Lord
based
be
University
3
[
my
Figure
group
*
1990
al
problem
role
1989
The
I
English
All
V1

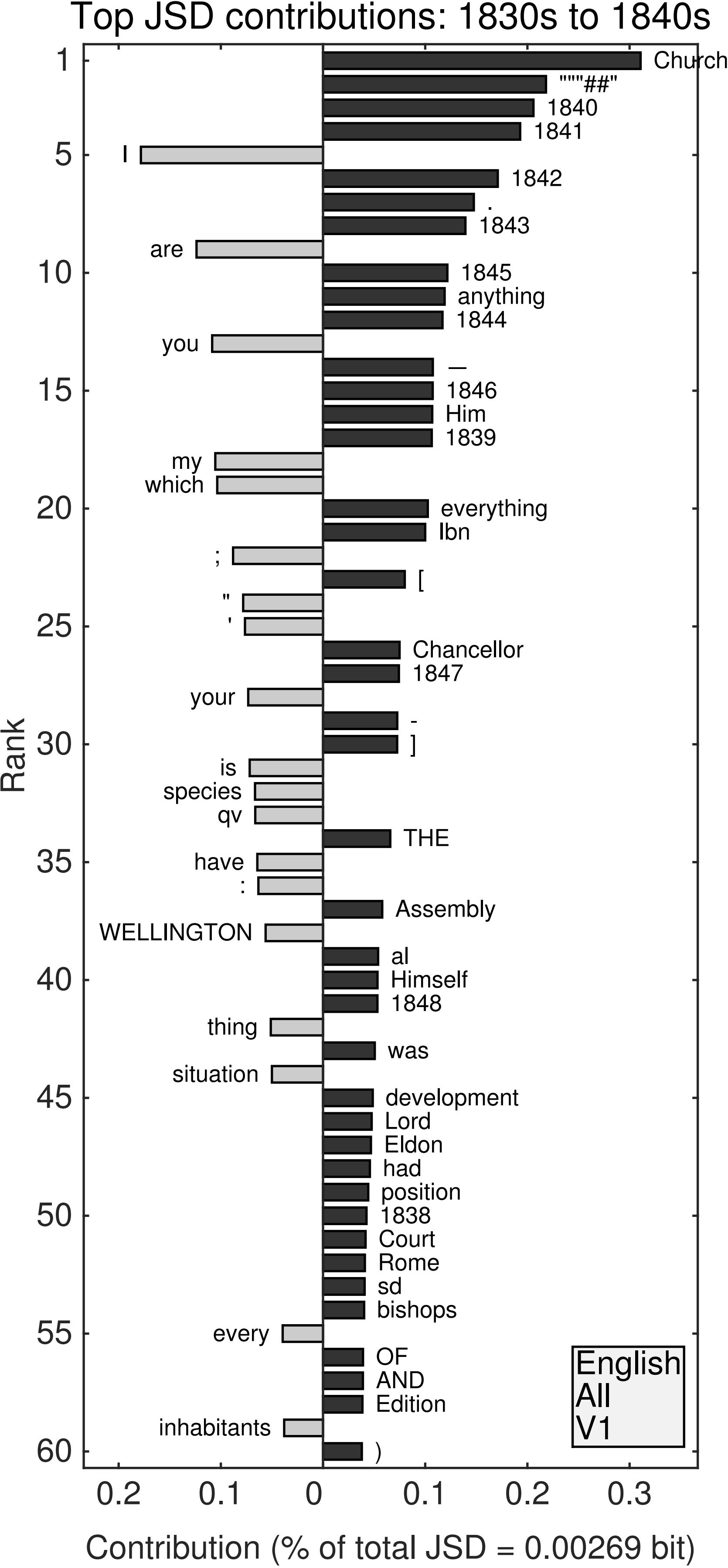

Top JSD contributions: 1830s to 1840s
Church
"""##"
1840
1841
I
1842
.
1843
are
1845
anything
1844
you
—
1846
Him
1839
my
which
everything
Ibn
;
[
"
'
Chancellor
1847
your
-
]
is
species
qv
THE
have
:
Assembly
WELLINGTON
al
Himself
1848
thing
was
situation
development
Lord
Eldon
had
position
1838
Court
Rome
sd
bishops
every
OF
AND
Edition
inhabitants
)
English
All
V1
Rank
1
5
10
15
20
25
30
35
40
45
50
55
60
0.2
0.1
0
0.1
0.2
0.3
Contribution (% of total JSD = 0.00269 bit)

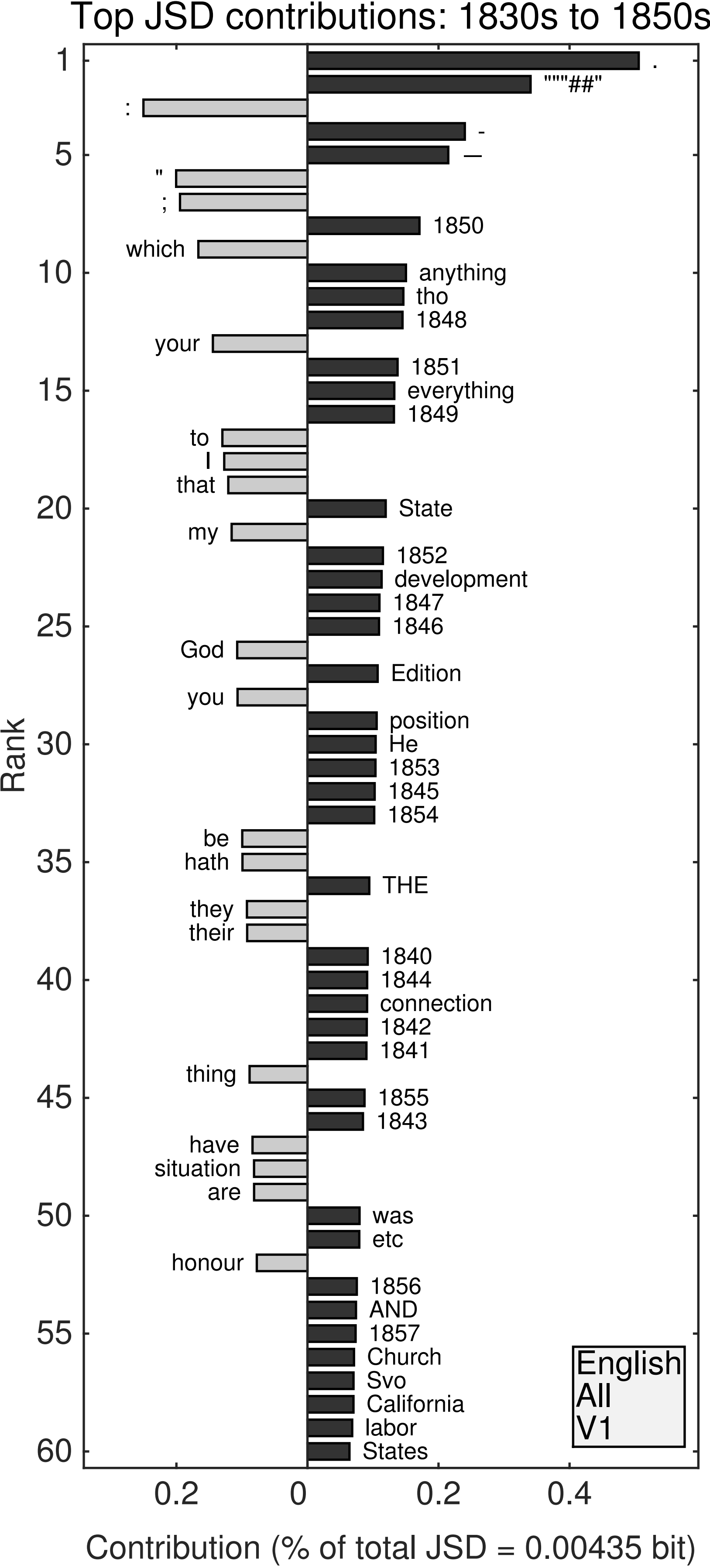

Top JSD contributions: 1830s to 1850s
Rank
Contribution (% of total JSD = 0.00435 bit)
.
"""##"
:
-
—
"
;
1850
which
anything
tho
1848
your
1851
everything
1849
to
I
that
State
my
1852
development
1847
1846
God
Edition
you
position
He
1853
1845
1854
be
hath
THE
they
their
1840
1844
connection
1842
1841
thing
1855
1843
have
situation
are
was
etc
honour
1856
AND
1857
Church
Svo
California
labor
States
English
All
V1

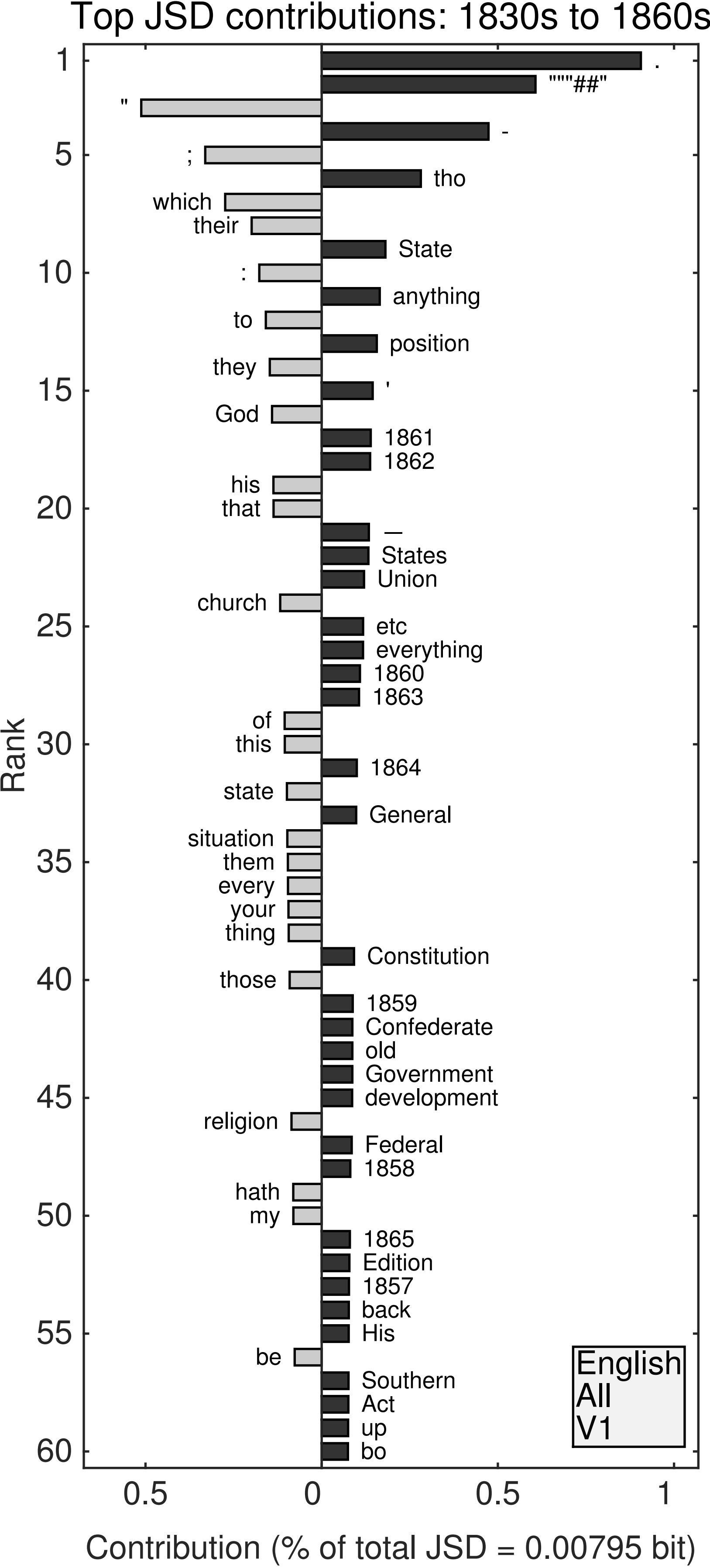

Top JSD contributions: 1830s to 1860s
Rank
Contribution (% of total JSD = 0.00795 bit)
.
"""##"
"
-
;
tho
which
their
State
:
anything
to
position
they
'
God
1861
1862
his
that
—
States
Union
church
etc
everything
1860
1863
of
this
1864
state
General
situation
them
every
your
thing
Constitution
those
1859
Confederate
old
Government
development
religion
Federal
1858
hath
my
1865
Edition
1857
back
His
be
Southern
Act
up
bo
English
All
V1

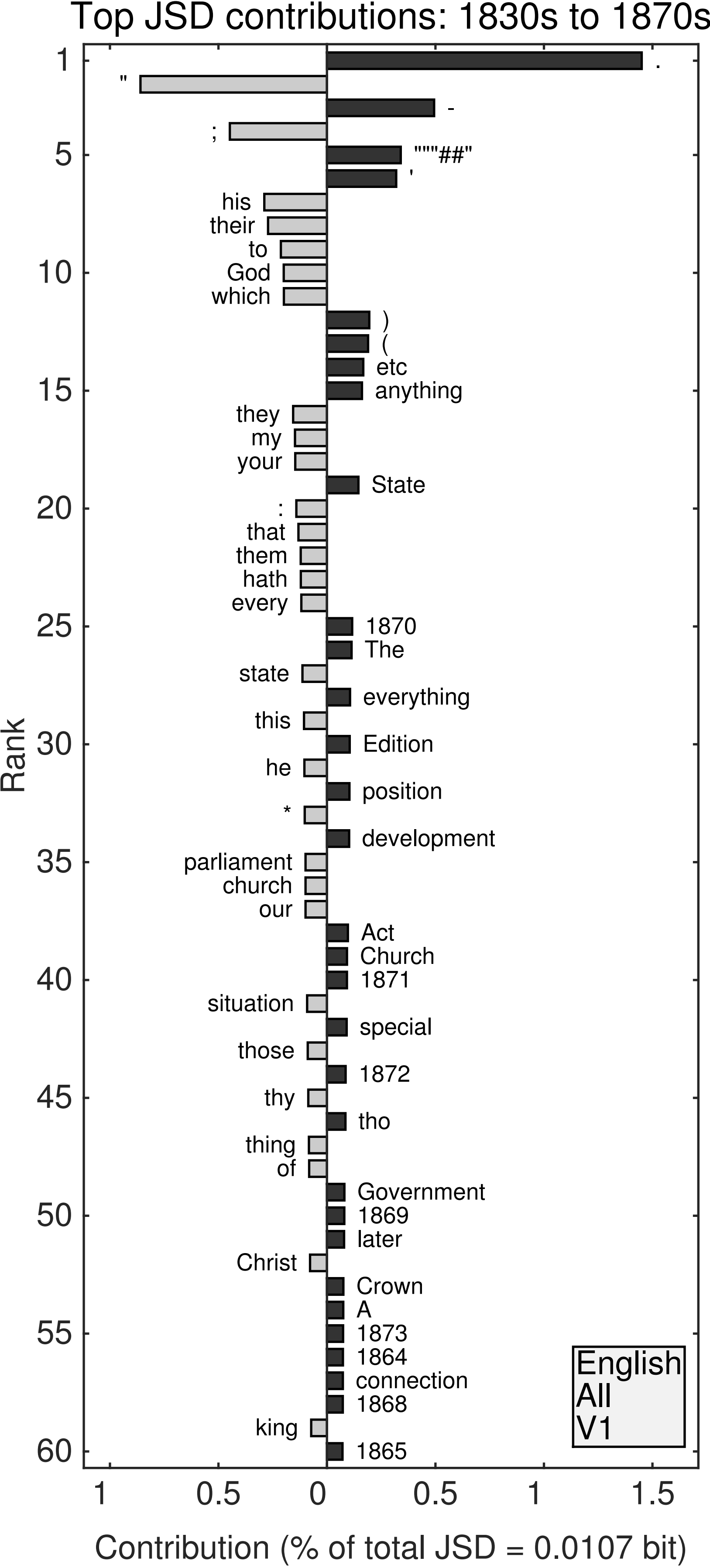

Top JSD contributions: 1830s to 1870s
Rank
Contribution (% of total JSD = 0.0107 bit)
.
"
-
;
"""##"
'
his
their
to
God
which
)
(
etc
anything
they
my
your
State
:
that
them
hath
every
1870
The
state
everything
this
Edition
he
position
*
development
parliament
church
our
Act
Church
1871
situation
special
those
1872
thy
tho
thing
of
Government
1869
later
Christ
Crown
A
1873
1864
connection
1868
king
1865
English
All
V1

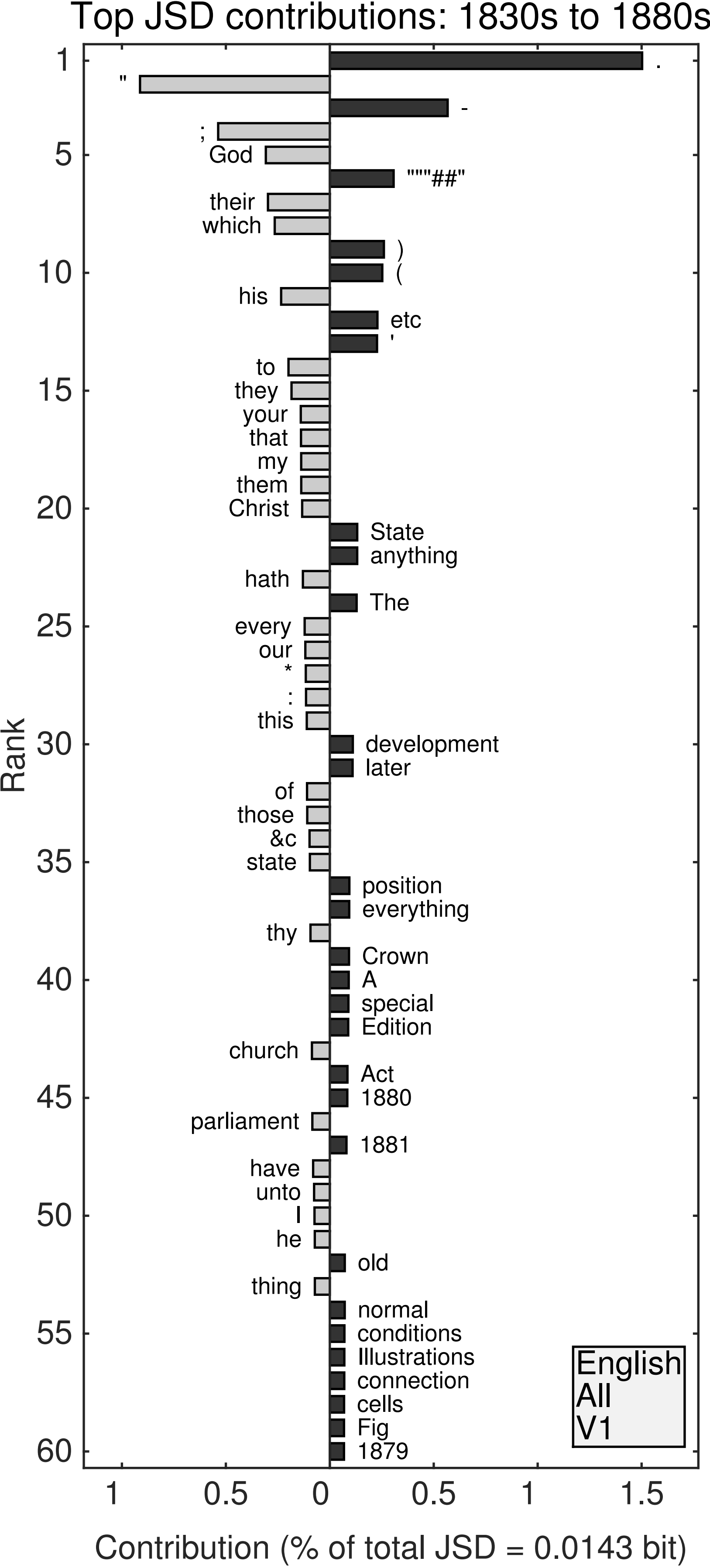
Top JSD contributions: 1830s to 1880s
Rank
Contribution (% of total JSD = 0.0143 bit)
English
All
V1
.
"
-
;
God
"""##"
their
which
)
(
his
etc
'
to
they
your
that
my
them
Christ
State
anything
hath
The
every
our
*
:
this
development
later
of
those
&c
state
position
everything
thy
Crown
A
special
Edition
church
Act
1880
parliament
1881
have
unto
I
he
old
thing
normal
conditions
Illustrations
connection
cells
Fig
1879

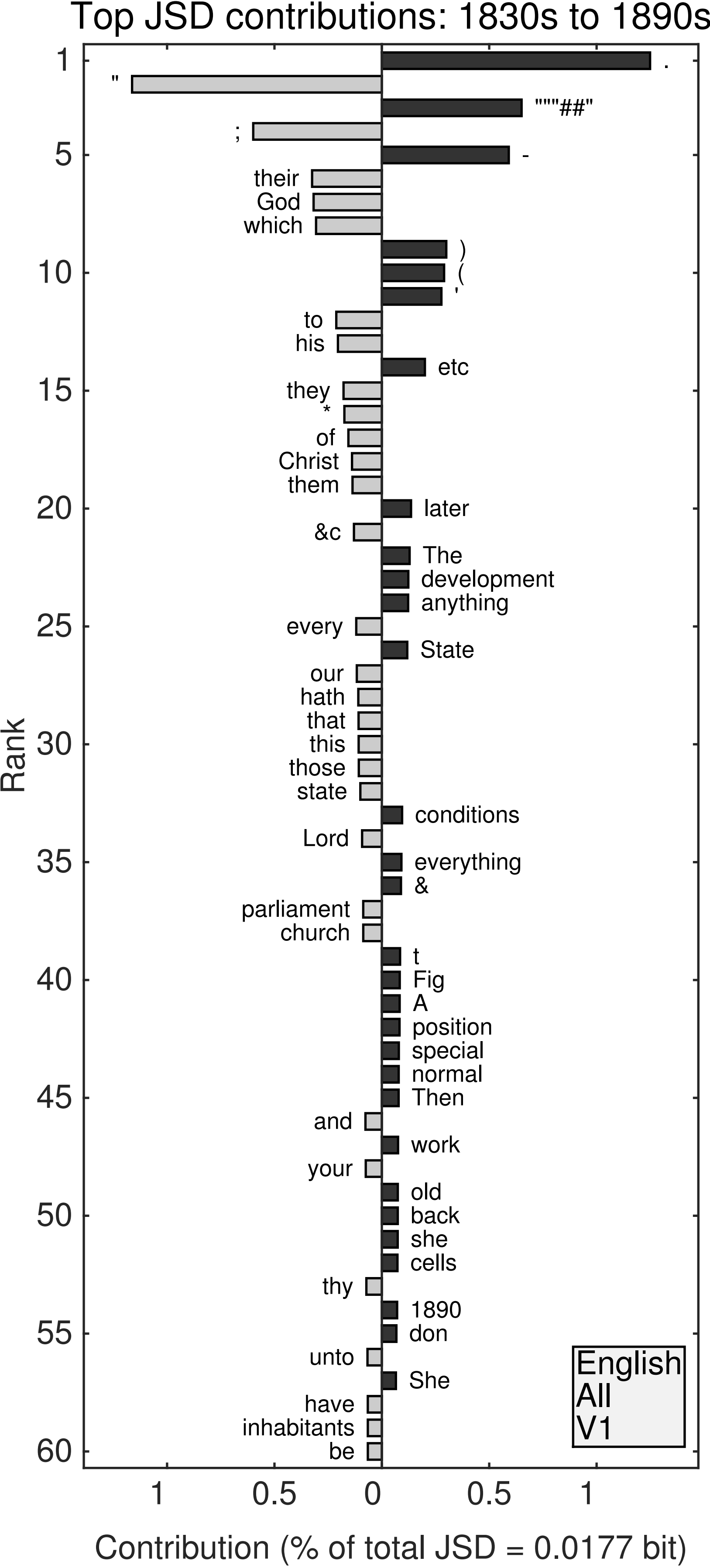

Top JSD contributions: 1830s to 1890s
Rank
1
5
10
15
20
25
30
35
40
45
50
55
60
.
"
"""##"
;
-
their
God
which
)
(
'
to
his
etc
they
*
of
Christ
them
later
&c
The
development
anything
every
State
our
hath
that
this
those
state
conditions
Lord
everything
&
parliament
church
t
Fig
A
position
special
normal
Then
and
work
your
old
back
she
cells
thy
1890
don
unto
She
have
inhabitants
be
English
All
V1
1
0.5
0
0.5
1
Contribution (% of total JSD = 0.0177 bit)

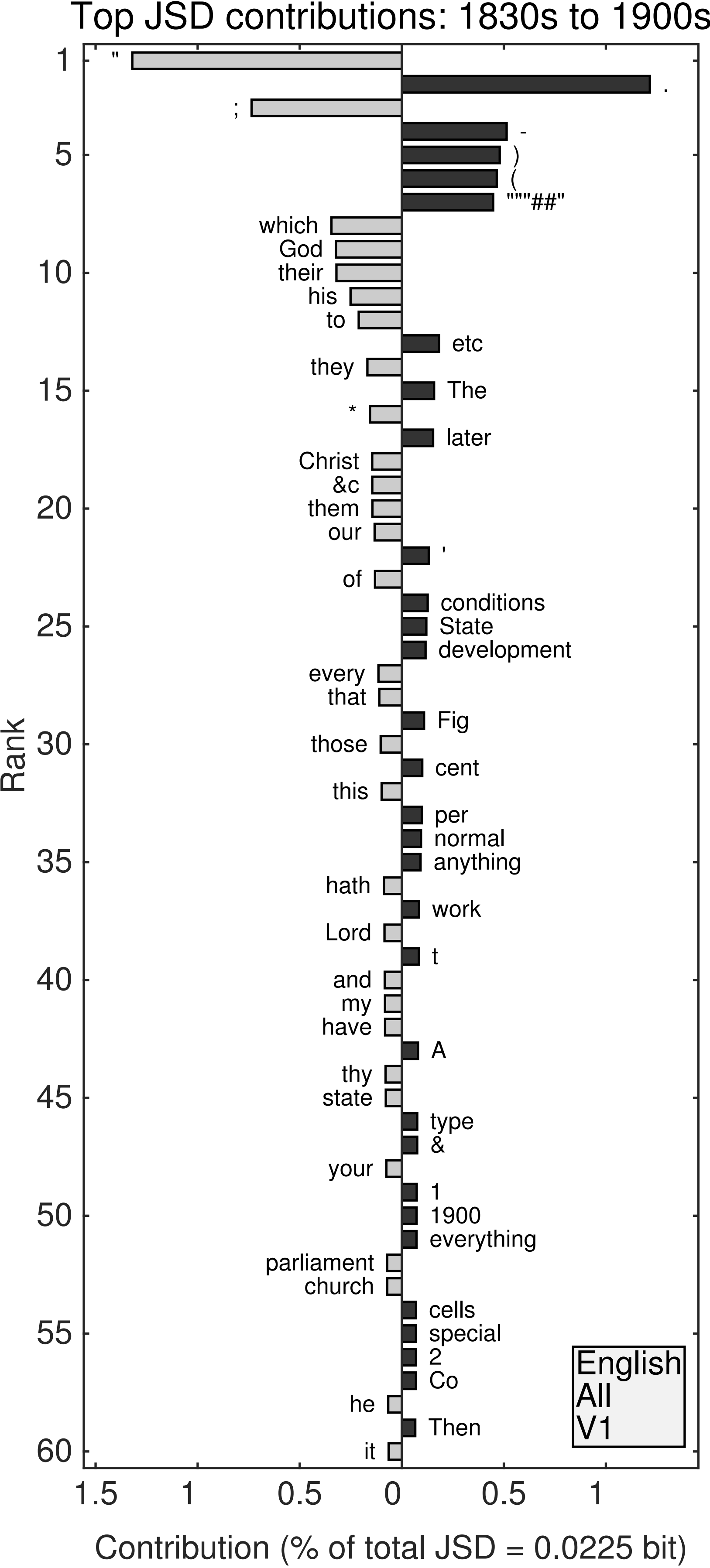
Top JSD contributions: 1830s to 1900s
Rank
Contribution (% of total JSD = 0.0225 bit)
1.5
1
0.5
0
0.5
1
1
5
10
15
20
25
30
35
40
45
50
55
60
"
.
;
-
)
(
"""##"
which
God
their
his
to
etc
they
The
*
later
Christ
&c
them
our
'
of
conditions
State
development
every
that
Fig
those
cent
this
per
normal
anything
hath
work
Lord
t
and
my
have
A
thy
state
type
&
your
1
1900
everything
parliament
church
cells
special
2
Co
he
Then
it
English
All
V1

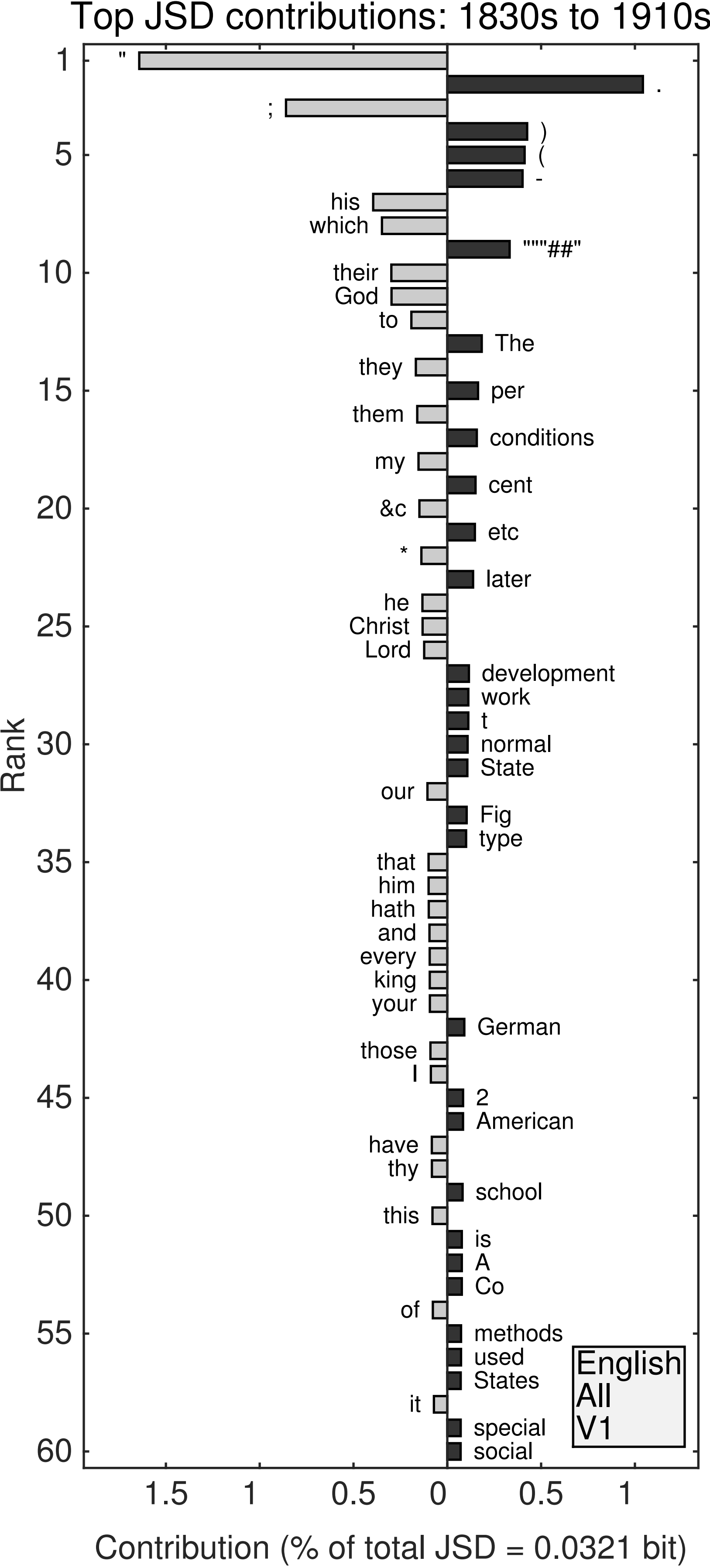
Top JSD contributions: 1830s to 1910s
Rank
Contribution (% of total JSD = 0.0321 bit)
1
5
10
15
20
25
30
35
40
45
50
55
60
1.5
1
0.5
0
0.5
1
"
.
;
)
(
-
his
which
"""##"
their
God
to
The
they
per
them
conditions
my
cent
&c
etc
*
later
he
Christ
Lord
development
work
t
normal
State
our
Fig
type
that
him
hath
and
every
king
your
German
those
I
2
American
have
thy
school
this
is
A
Co
of
methods
used
States
it
special
social
English
All
V1

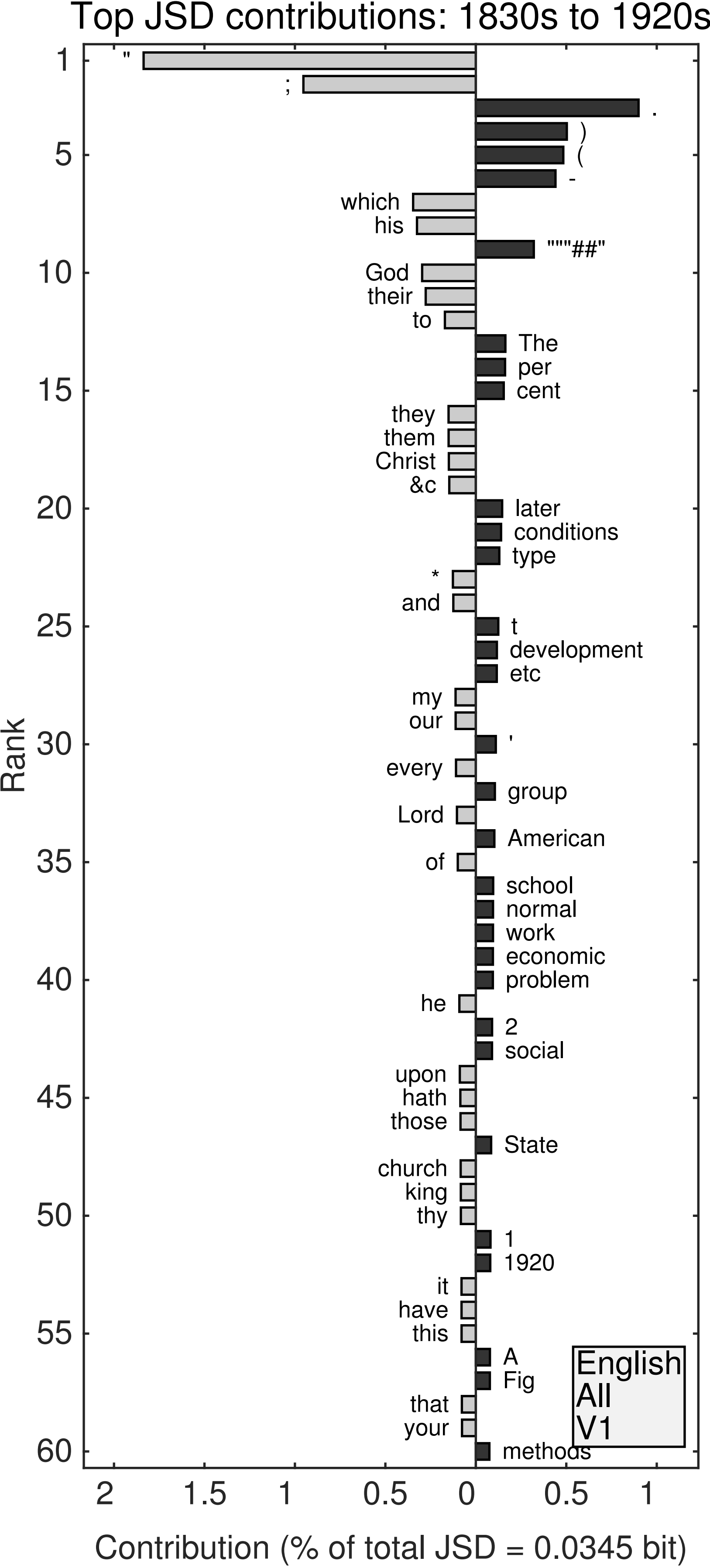
Top JSD contributions: 1830s to 1920s
Rank
Contribution (% of total JSD = 0.0345 bit)
"
;
.
)
(
-
which
his
"""##"
God
their
to
The
per
cent
they
them
Christ
&c
later
conditions
type
*
and
t
development
etc
my
our
'
every
group
Lord
American
of
school
normal
work
economic
problem
he
2
social
upon
hath
those
State
church
king
thy
1
1920
it
have
this
A
Fig
that
your
methods
English
All
V1

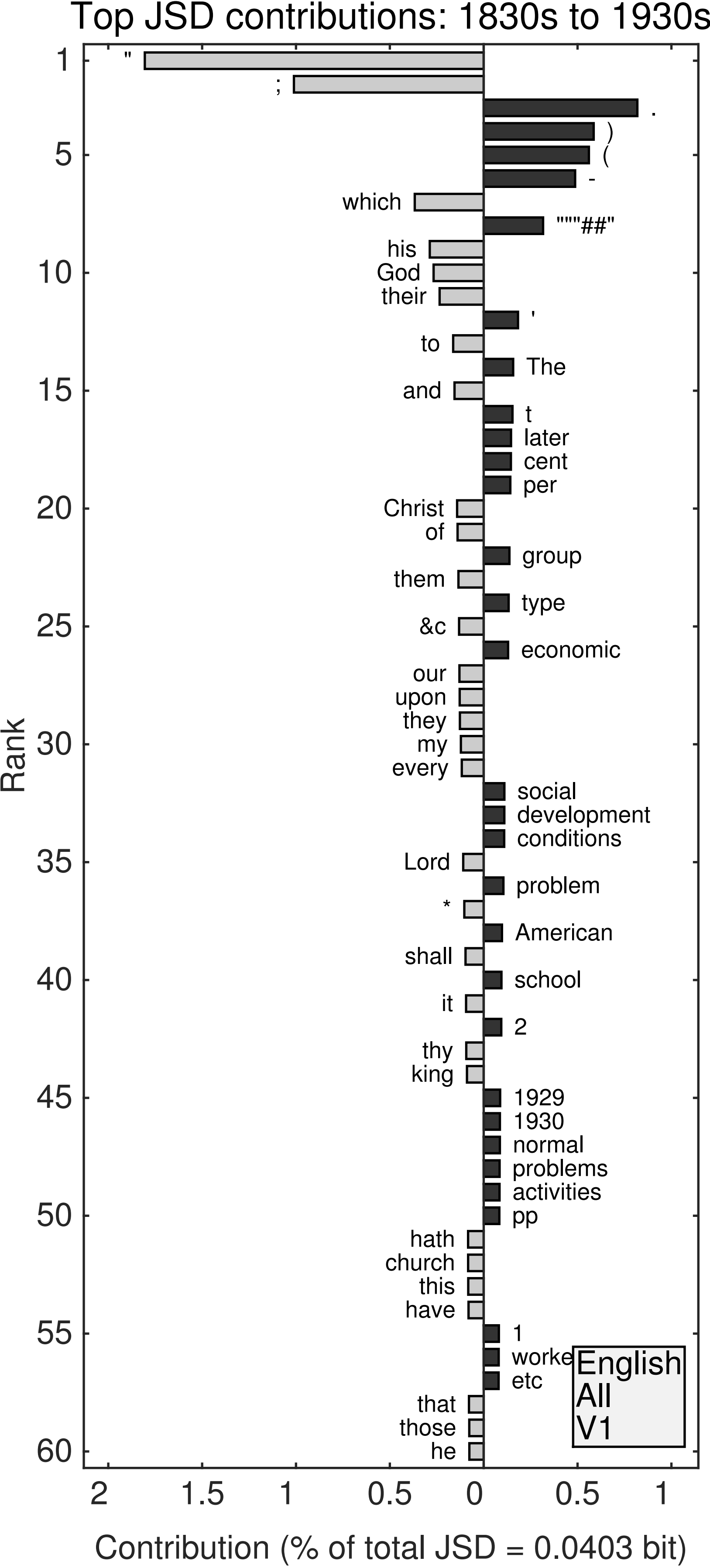
Top JSD contributions: 1830s to 1930s
Rank
Contribution (% of total JSD = 0.0403 bit)
"
;
.
)
(
-
which
"""##"
his
God
their
'
to
The
and
t
later
cent
per
Christ
of
group
them
type
&c
economic
our
upon
they
my
every
social
development
conditions
Lord
problem
*
American
shall
school
it
2
thy
king
1929
1930
normal
problems
activities
pp
hath
church
this
have
1
worke
etc
that
those
he
English
All
V1

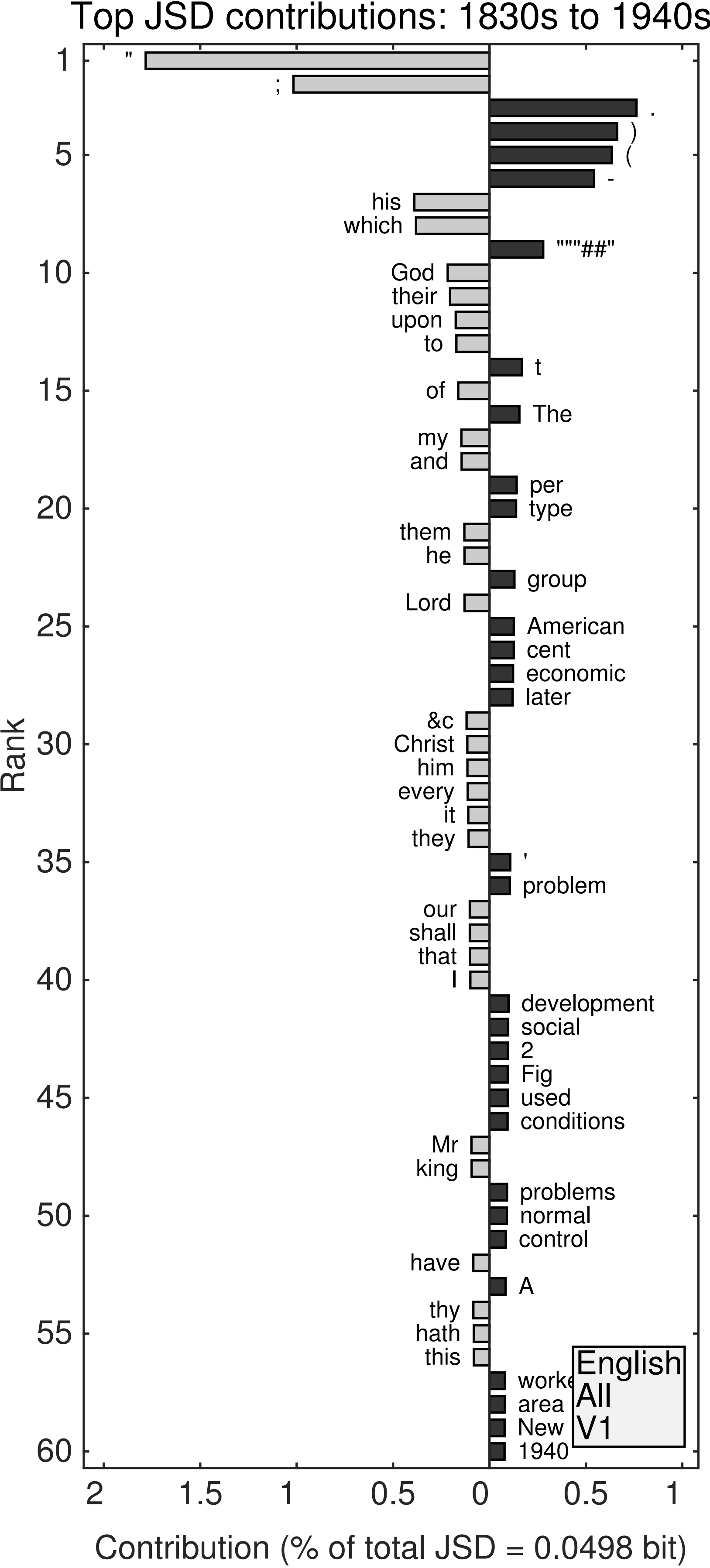

Top JSD contributions: 1830s to 1940s
Rank
Contribution (% of total JSD = 0.0498 bit)
"
;
.
)
(
-
his
which
"""##"
God
their
upon
to
t
of
The
my
and
per
type
them
he
group
Lord
American
cent
economic
later
&c
Christ
him
every
it
they
'
problem
our
shall
that
I
development
social
2
Fig
used
conditions
Mr
king
problems
normal
control
have
A
thy
hath
this
worke
area
New
1940
English
All
V1

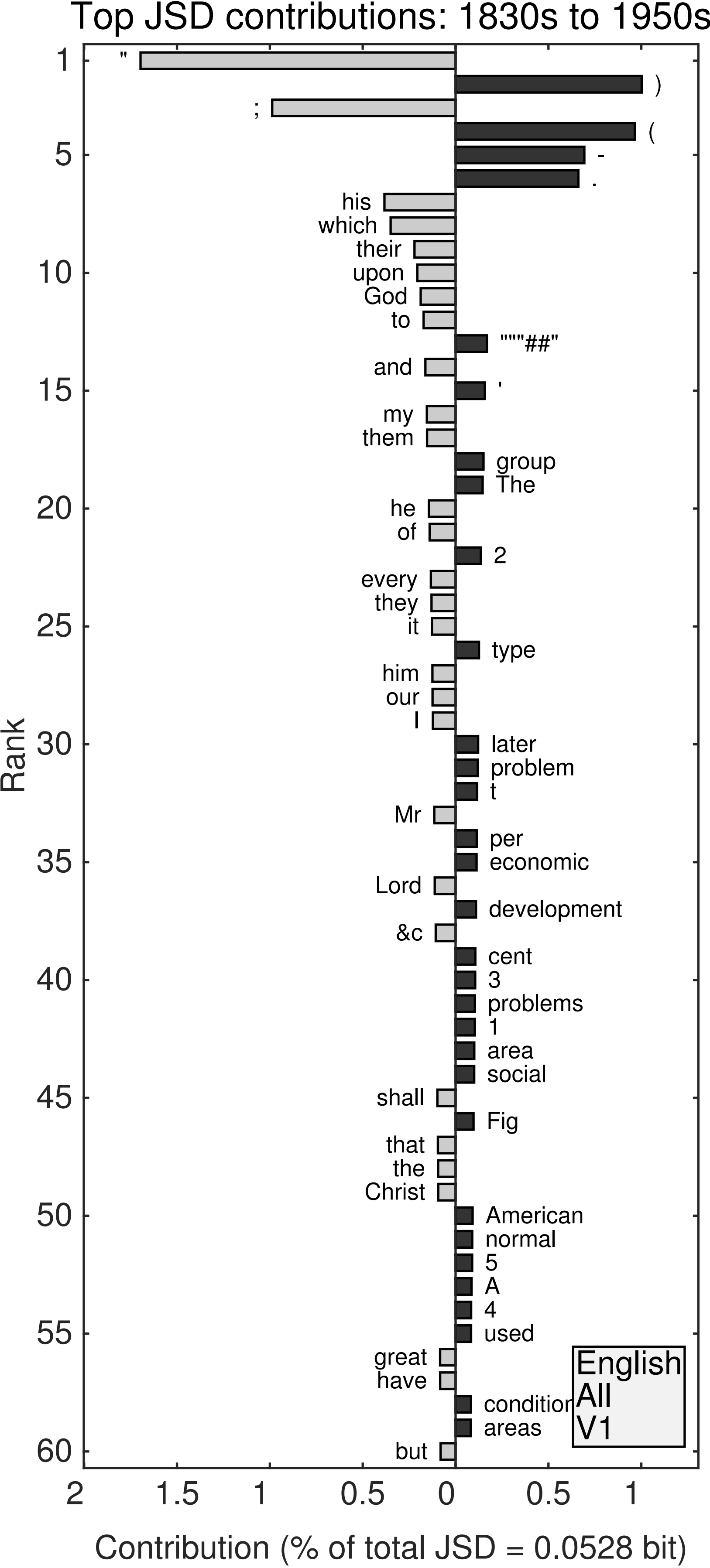
Top JSD contributions: 1830s to 1950s
Rank
Contribution (% of total JSD = 0.0528 bit)
"
)
;
(
-
.
his
which
their
upon
God
to
"""##"
and
'
my
them
group
The
he
of
2
every
they
it
type
him
our
I
later
problem
t
Mr
per
economic
Lord
development
&c
cent
3
problems
1
area
social
shall
Fig
that
the
Christ
American
normal
5
A
4
used
great
have
condition
areas
but
English
All
V1

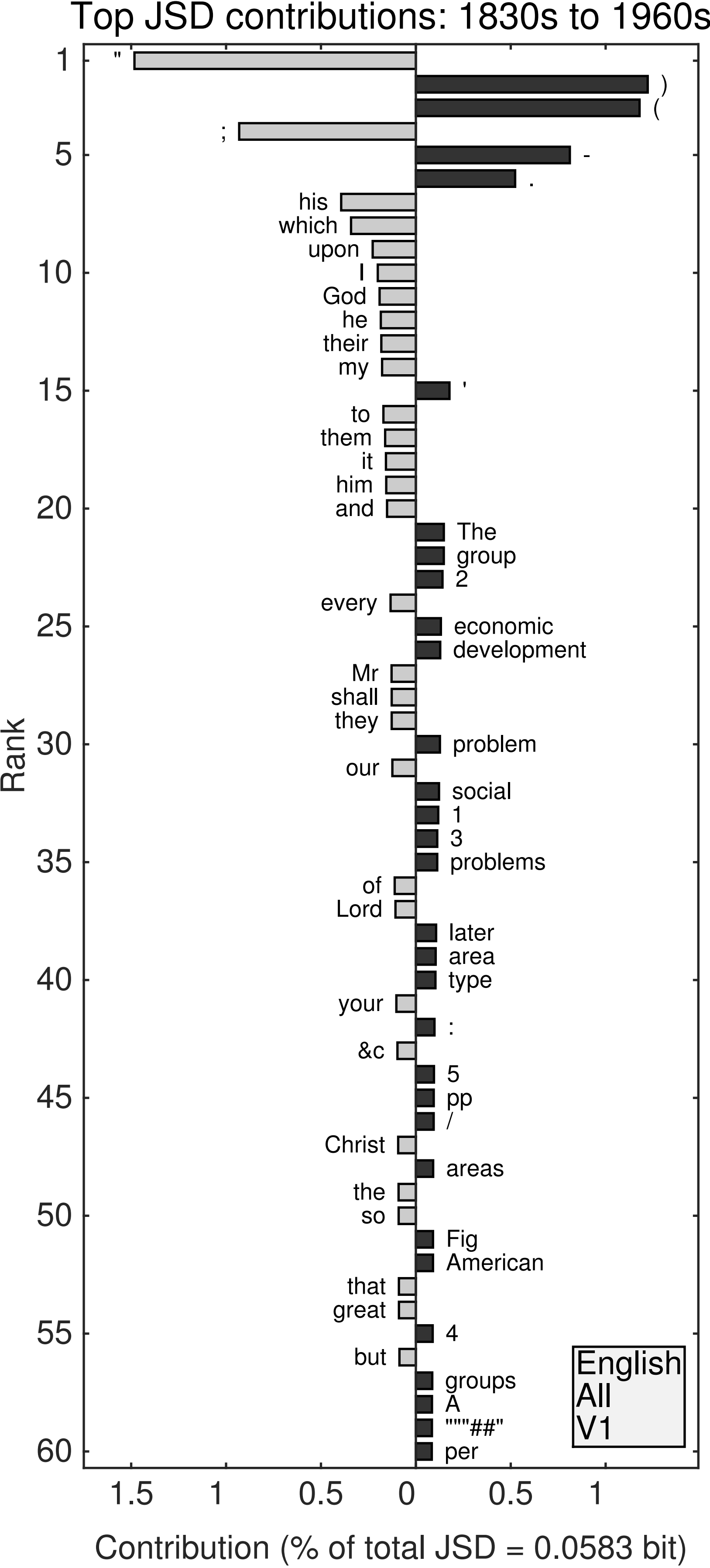

Top JSD contributions: 1830s to 1960s
Rank
Contribution (% of total JSD = 0.0583 bit)
"
)
(
;
-
.
his
which
upon
I
God
he
their
my
'
to
them
it
him
and
The
group
2
every
economic
development
Mr
shall
they
problem
our
social
1
3
problems
of
Lord
later
area
type
your
:
&c
5
pp
/
Christ
areas
the
so
Fig
American
that
great
4
but
groups
A
"""##"
per
English
All
V1

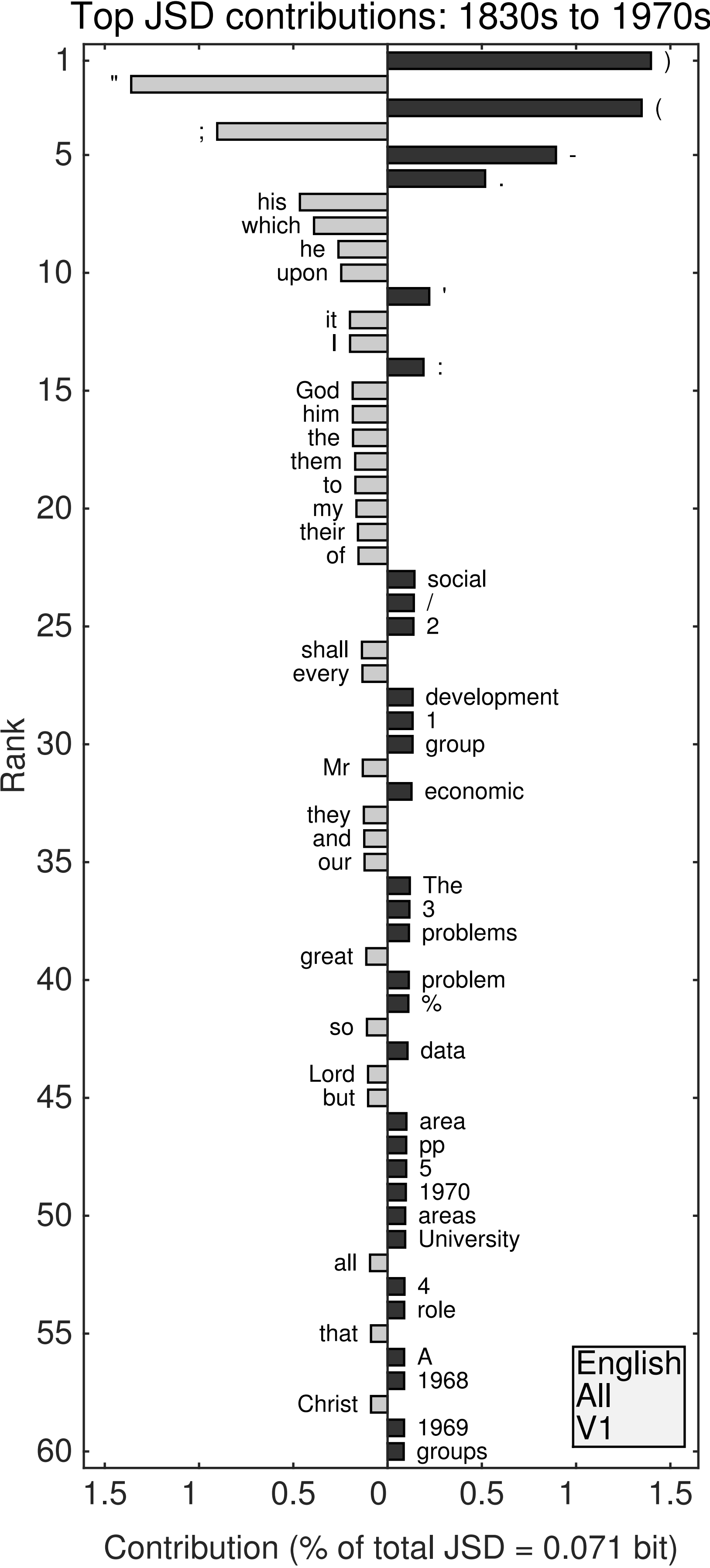
Top JSD contributions: 1830s to 1970s
Rank
Contribution (% of total JSD = 0.071 bit)
1.5
1
0.5
0
0.5
1
1.5
1
5
10
15
20
25
30
35
40
45
50
55
60
)
"
(
;
-
.
his
which
he
upon
'
it
I
:
God
him
the
them
to
my
their
of
social
/
2
shall
every
development
1
group
Mr
economic
they
and
our
The
3
problems
great
problem
%
so
data
Lord
but
area
pp
5
1970
areas
University
all
4
role
that
A
1968
Christ
1969
groups
English
All
V1

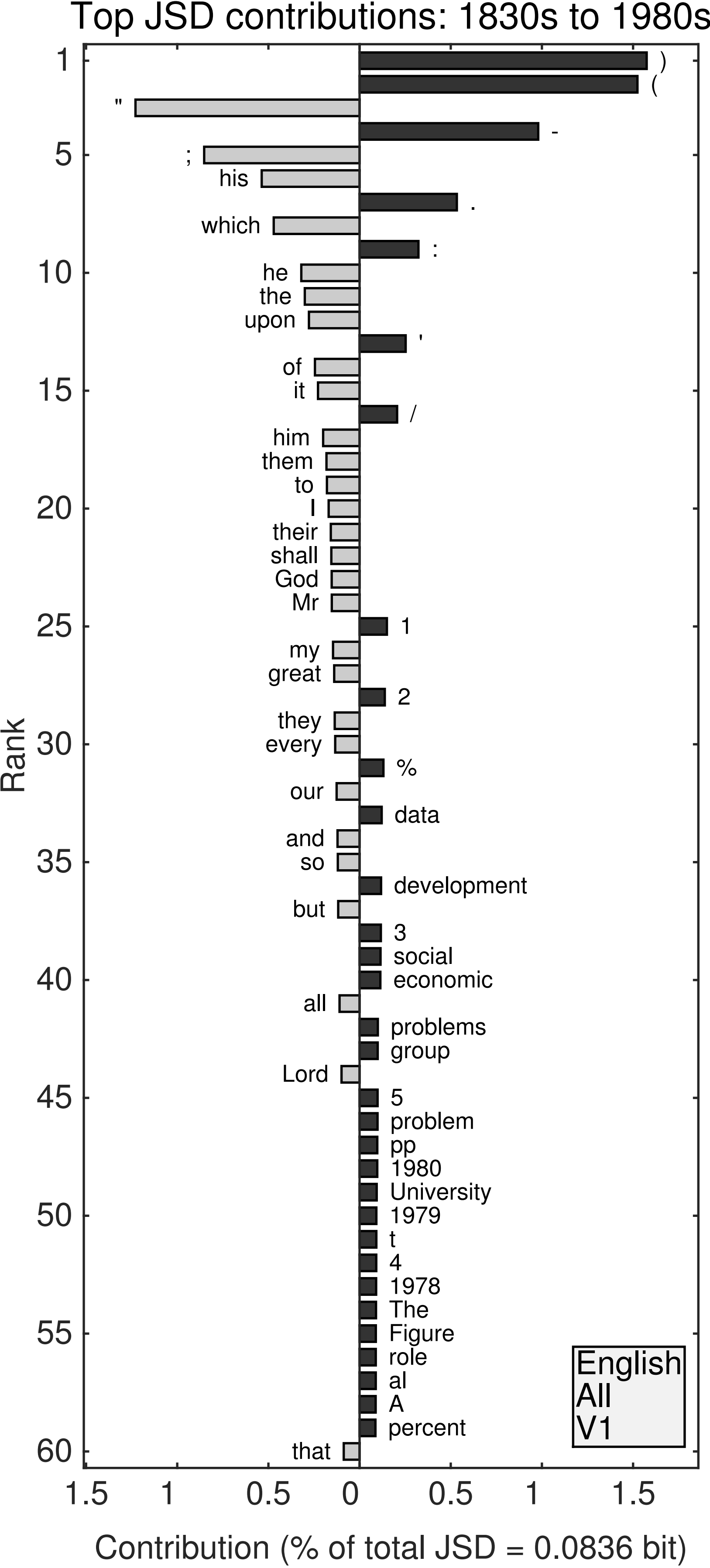

Top JSD contributions: 1830s to 1980s
Rank
Contribution (% of total JSD = 0.0836 bit)
)
(
"
-
;
his
.
which
:
he
the
upon
'
of
it
/
him
them
to
I
their
shall
God
Mr
1
my
great
2
they
every
%
our
data
and
so
development
but
3
social
economic
all
problems
group
Lord
5
problem
pp
1980
University
1979
t
4
1978
The
Figure
role
al
A
percent
that
English
All
V1

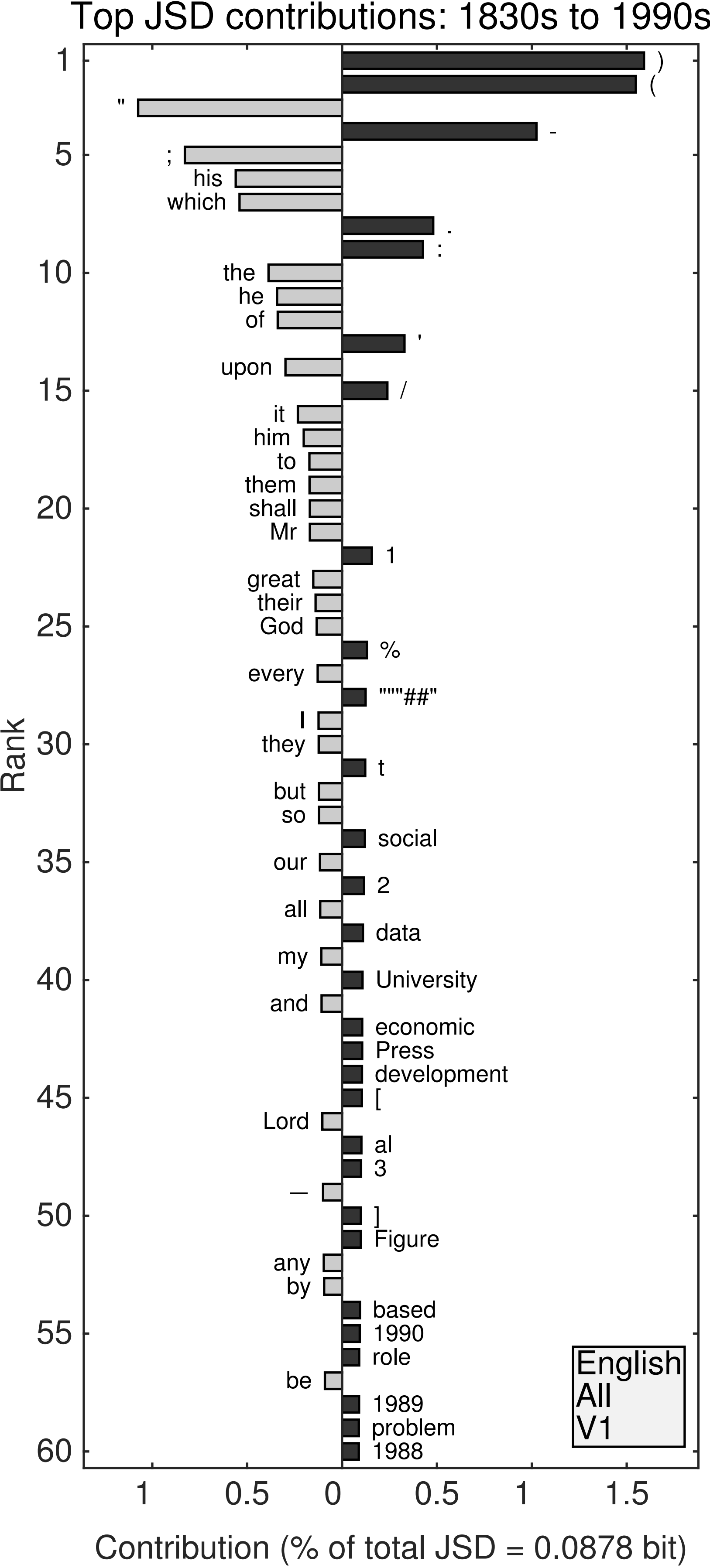

Top JSD contributions: 1830s to 1990s
Rank
Contribution (% of total JSD = 0.0878 bit)
)
(
"
-
;
his
which
.
:
the
he
of
'
upon
/
it
him
to
them
shall
Mr
1
great
their
God
%
every
"""##"
I
they
t
but
so
social
our
2
all
data
my
University
and
economic
Press
development
[
Lord
al
3
—
]
Figure
any
by
based
1990
role
be
1989
problem
1988
English
All
V1

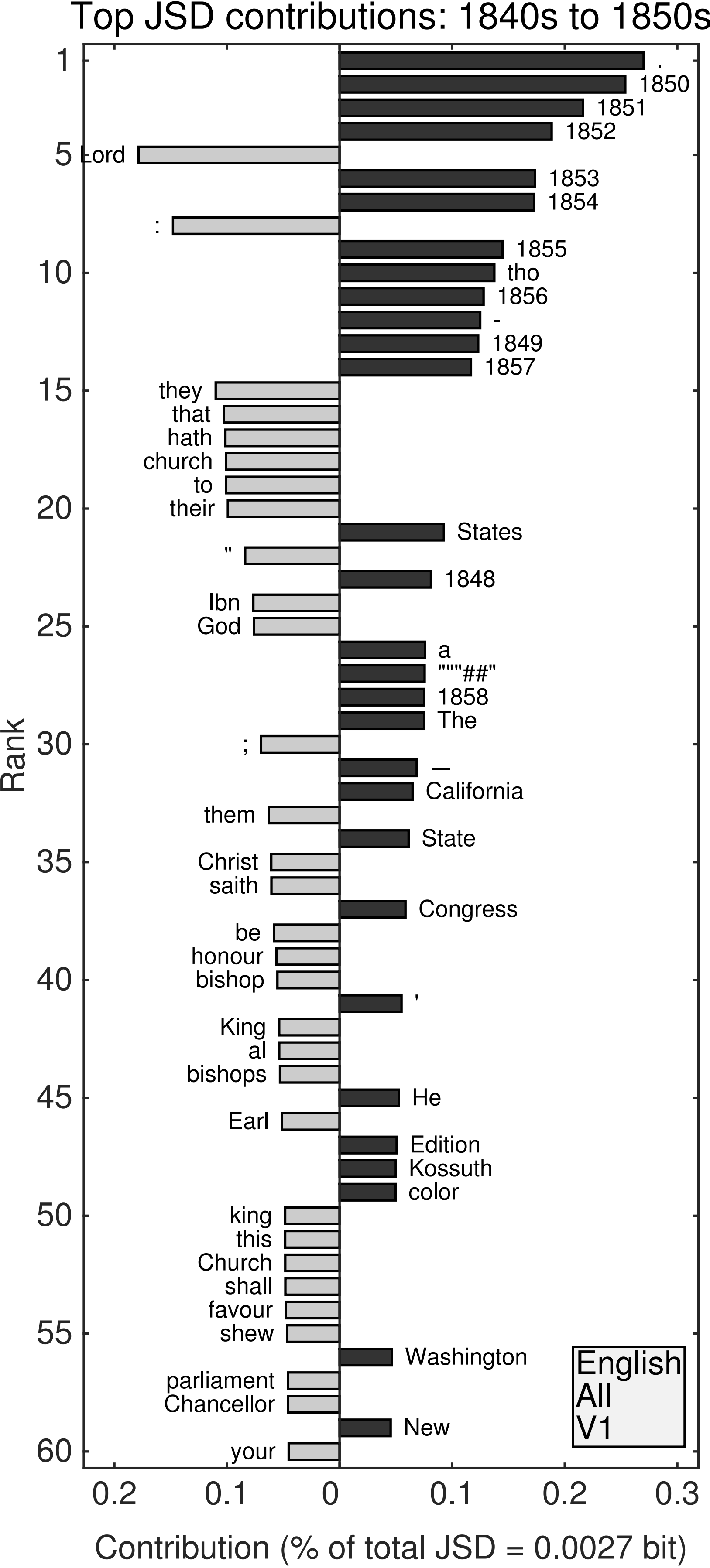

Top JSD contributions: 1840s to 1850s
Rank
1
5
10
15
20
25
30
35
40
45
50
55
60
.
1850
1851
1852
Lord
1853
1854
:
1855
tho
1856
-
1849
1857
they
that
hath
church
to
their
States
"
1848
Ibn
God
a
"""##"
1858
The
;
—
California
them
State
Christ
saith
Congress
be
honour
bishop
'
King
al
bishops
He
Earl
Edition
Kossuth
color
king
this
Church
shall
favour
shew
Washington
parliament
Chancellor
New
your
English
All
V1
0.2
0.1
0
0.1
0.2
0.3
Contribution (% of total JSD = 0.0027 bit)

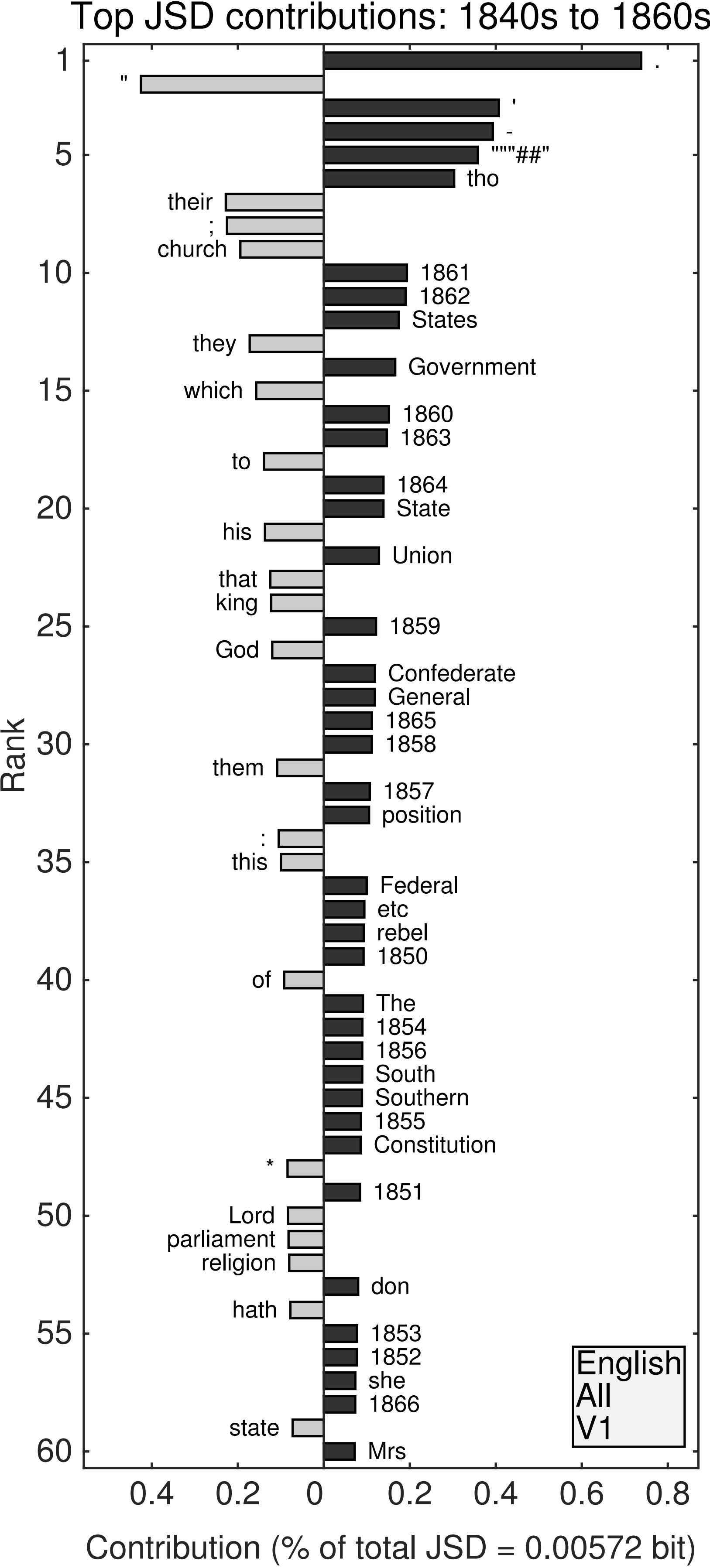

Top JSD contributions: 1840s to 1860s
Rank
Contribution (% of total JSD = 0.00572 bit)
.
"
'
-
"""##"
tho
their
;
church
1861
1862
States
they
Government
which
1860
1863
to
1864
State
his
Union
that
king
1859
God
Confederate
General
1865
1858
them
1857
position
:
this
Federal
etc
rebel
1850
of
The
1854
1856
South
Southern
1855
Constitution
*
1851
Lord
parliament
religion
don
hath
1853
1852
she
1866
state
Mrs
English
All
V1

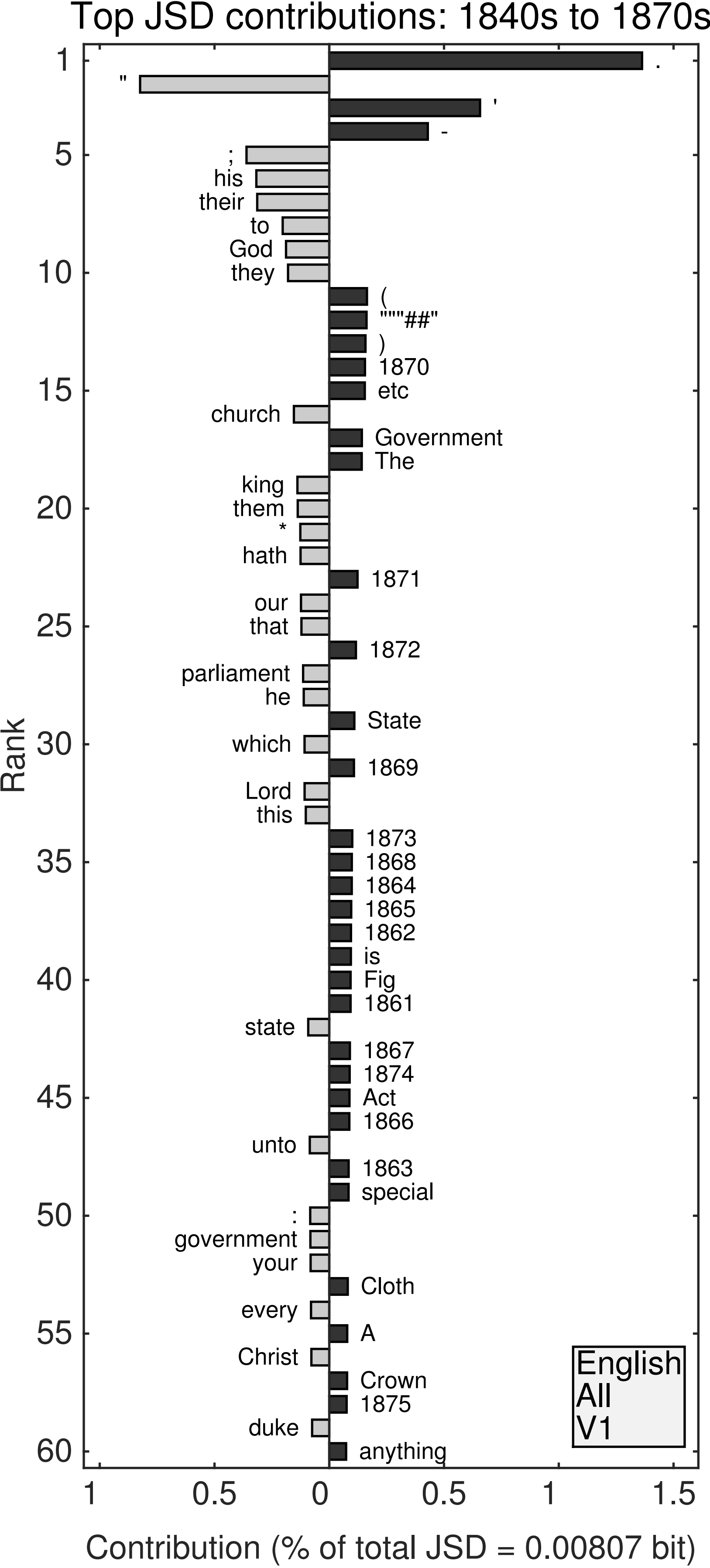

Top JSD contributions: 1840s to 1870s
Rank
Contribution (% of total JSD = 0.00807 bit)
English
All
V1
.
"
'
-
;
his
their
to
God
they
(
"""##"
)
1870
etc
church
Government
The
king
them
*
hath
1871
our
that
1872
parliament
he
State
which
1869
Lord
this
1873
1868
1864
1865
1862
is
Fig
1861
state
1867
1874
Act
1866
unto
1863
special
:
government
your
Cloth
every
A
Christ
Crown
1875
duke
anything

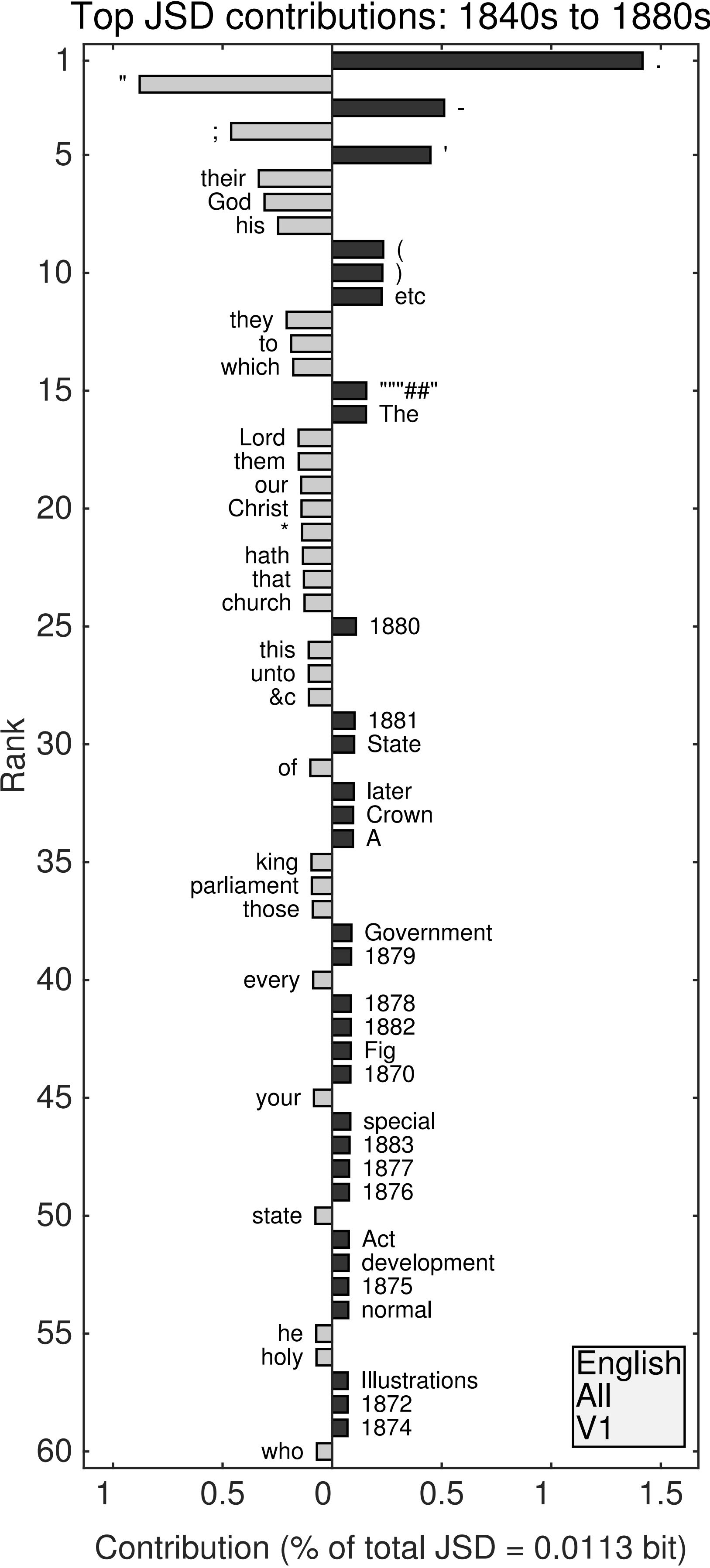
Top JSD contributions: 1840s to 1880s
.
"
-
;
'
their
God
his
(
)
etc
they
to
which
"""##"
The
Lord
them
our
Christ
*
hath
that
church
1880
this
unto
&c
1881
State
of
later
Crown
A
king
parliament
those
Government
1879
every
1878
1882
Fig
1870
your
special
1883
1877
1876
state
Act
development
1875
normal
he
holy
Illustrations
1872
1874
who
English
All
V1
Rank
Contribution (% of total JSD = 0.0113 bit)

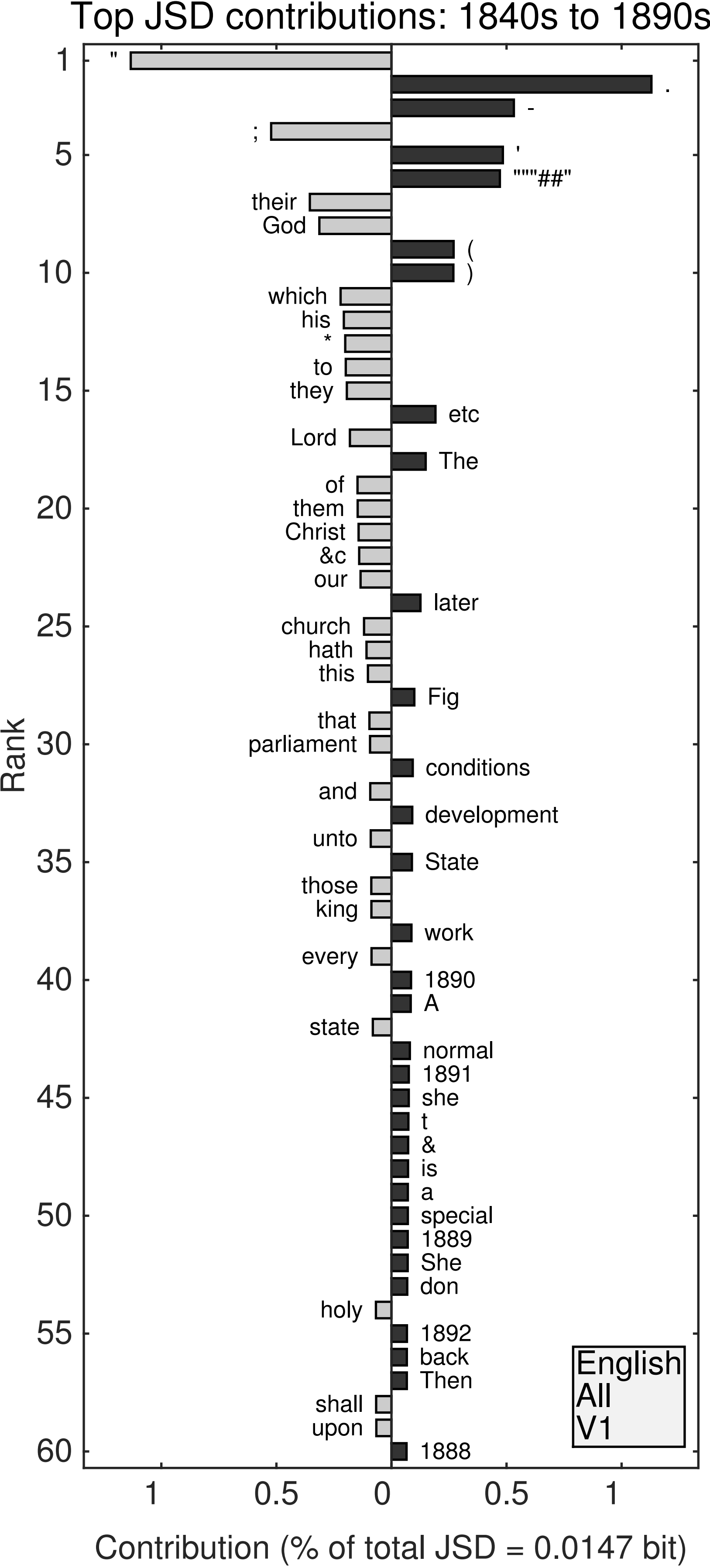

Top JSD contributions: 1840s to 1890s
Rank
"
.
-
;
'
"""##"
their
God
(
)
which
his
*
to
they
etc
Lord
The
of
them
Christ
&c
our
later
church
hath
this
Fig
that
parliament
conditions
and
development
unto
State
those
king
work
every
1890
A
state
normal
1891
she
t
&
is
a
special
1889
She
don
holy
1892
back
Then
shall
upon
1888
English
All
V1
Contribution (% of total JSD = 0.0147 bit)

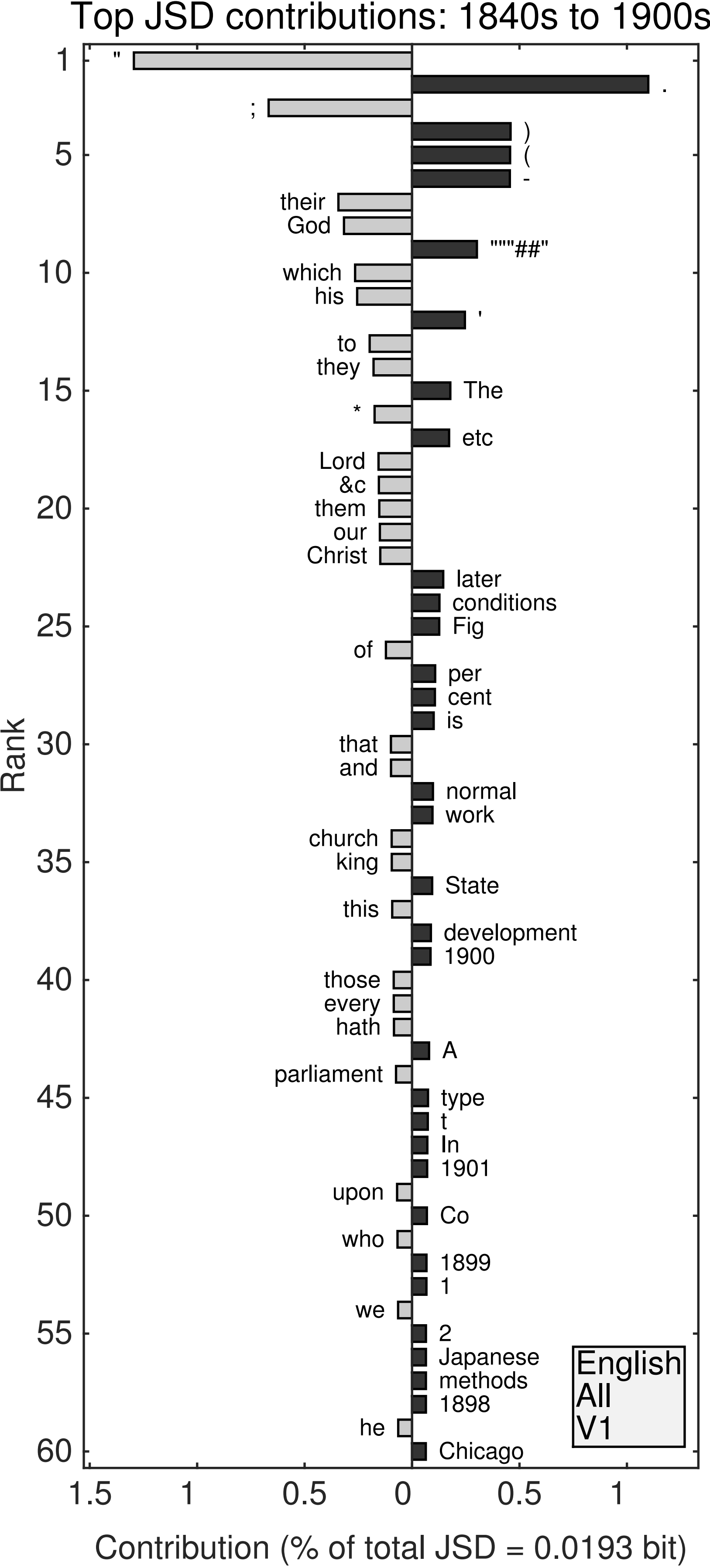

Top JSD contributions: 1840s to 1900s
Rank
Contribution (% of total JSD = 0.0193 bit)
"
.
;
)
(
-
their
God
"""##"
which
his
'
to
they
The
*
etc
Lord
&c
them
our
Christ
later
conditions
Fig
of
per
cent
is
that
and
normal
work
church
king
State
this
development
1900
those
every
hath
A
parliament
type
t
In
1901
upon
Co
who
1899
1
we
2
Japanese
methods
1898
he
Chicago
English
All
V1

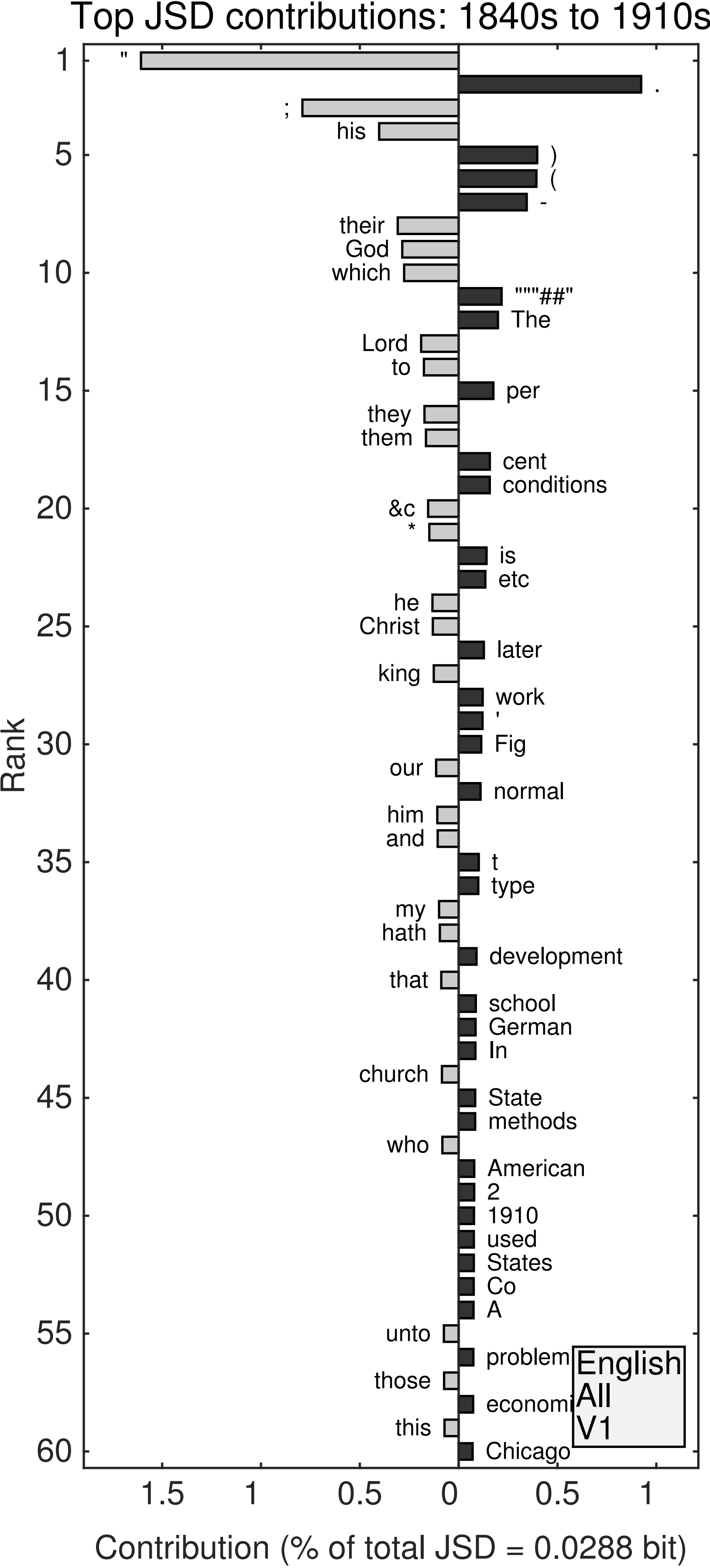
Top JSD contributions: 1840s to 1910s
Rank
"
.
;
his
)
(
-
their
God
which
"""##"
The
Lord
to
per
they
them
cent
conditions
&c
*
is
etc
he
Christ
later
king
work
'
Fig
our
normal
him
and
t
type
my
hath
development
that
school
German
In
church
State
methods
who
American
2
1910
used
States
Co
A
unto
problem
those
economi
this
Chicago
English
All
V1
Contribution (% of total JSD = 0.0288 bit)

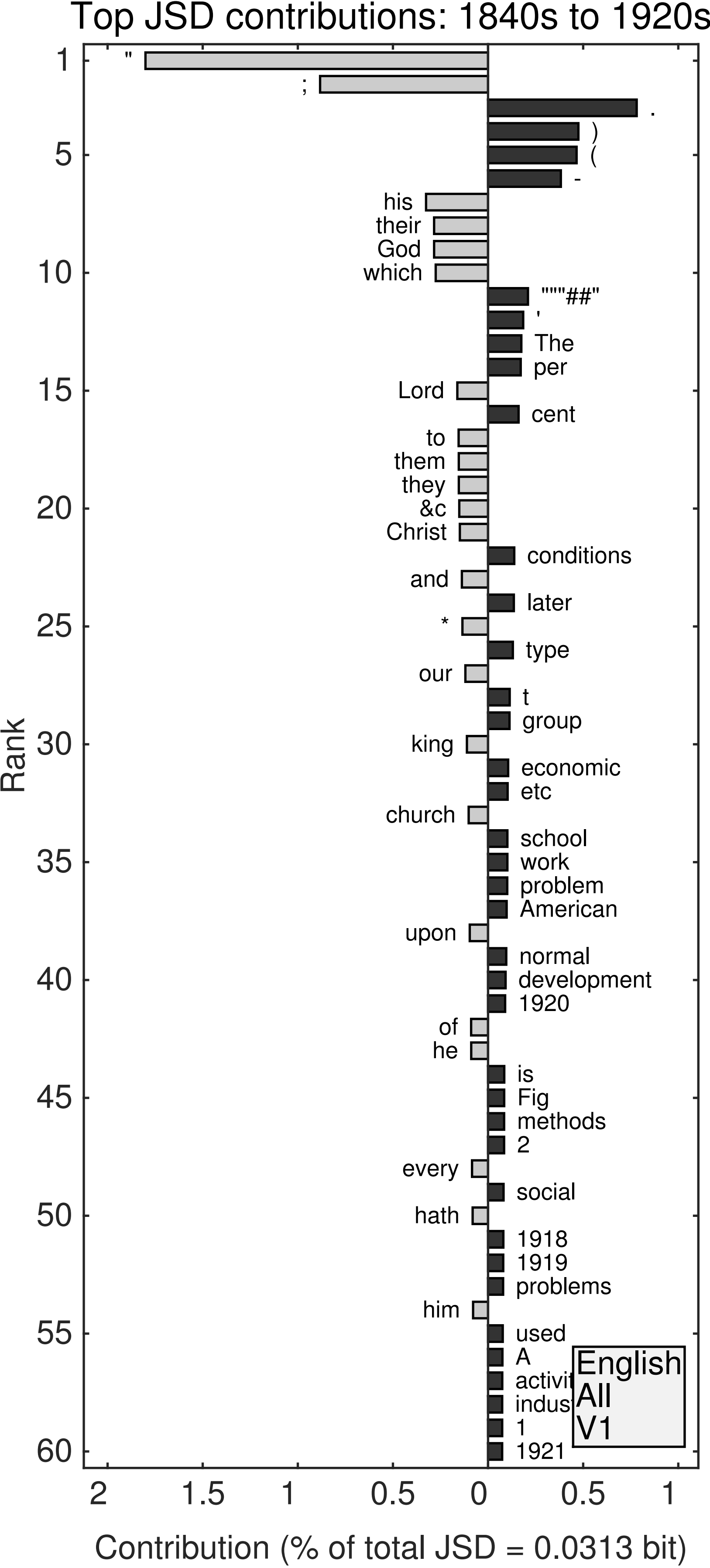

Top JSD contributions: 1840s to 1920s
Rank
Contribution (% of total JSD = 0.0313 bit)
"
;
.
)
(
-
his
their
God
which
"""##"
'
The
per
Lord
cent
to
them
they
&c
Christ
conditions
and
later
*
type
our
t
group
king
economic
etc
church
school
work
problem
American
upon
normal
development
1920
of
he
is
Fig
methods
2
every
social
hath
1918
1919
problems
him
used
A
1
1921
English
All
V1

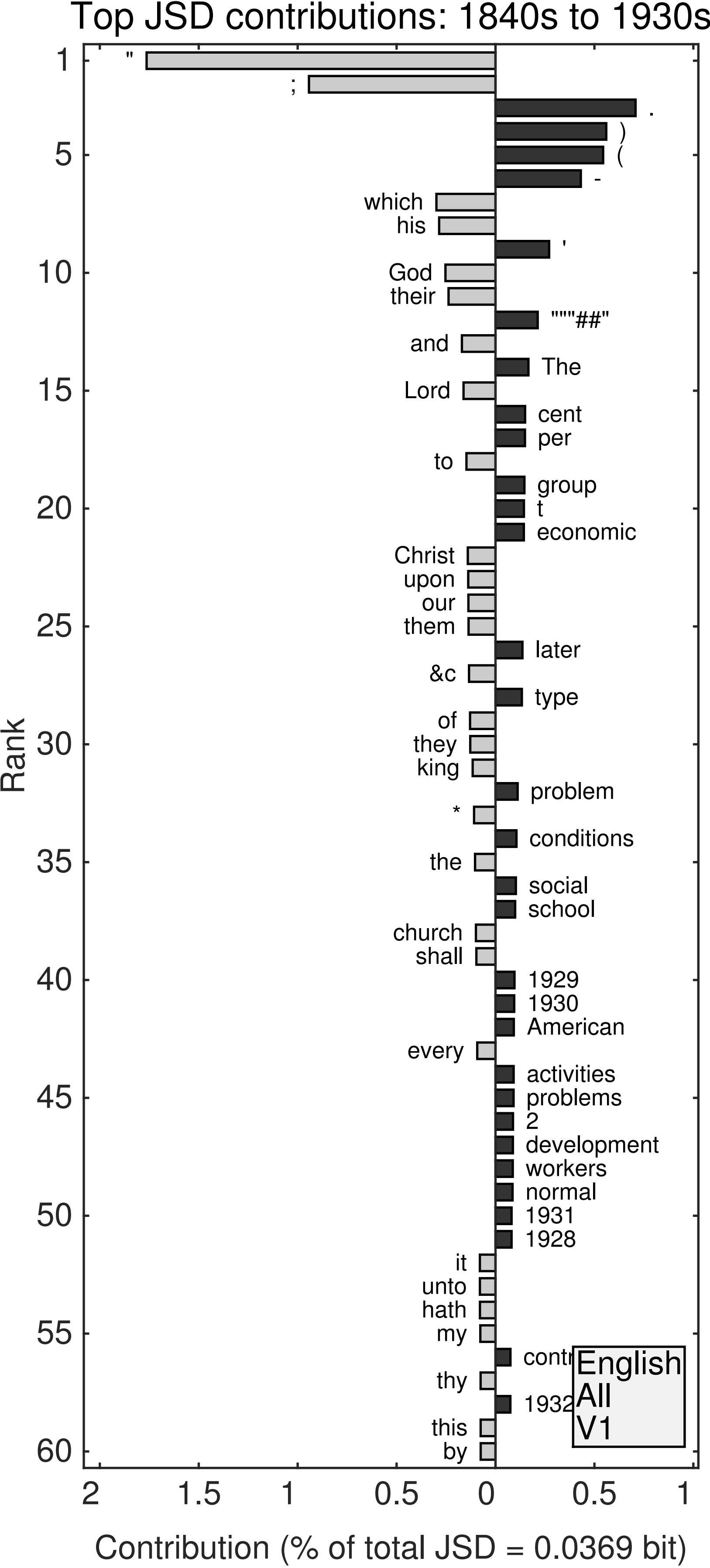

Top JSD contributions: 1840s to 1930s
Rank
"
;
.
)
(
-
which
his
'
God
their
"""##"
and
The
Lord
cent
per
to
group
t
economic
Christ
upon
our
them
later
&c
type
of
they
king
problem
*
conditions
the
social
school
church
shall
1929
1930
American
every
activities
problems
2
development
workers
normal
1931
1928
it
unto
hath
my
thy
this
by
English
All
V1
Contribution (% of total JSD = 0.0369 bit)

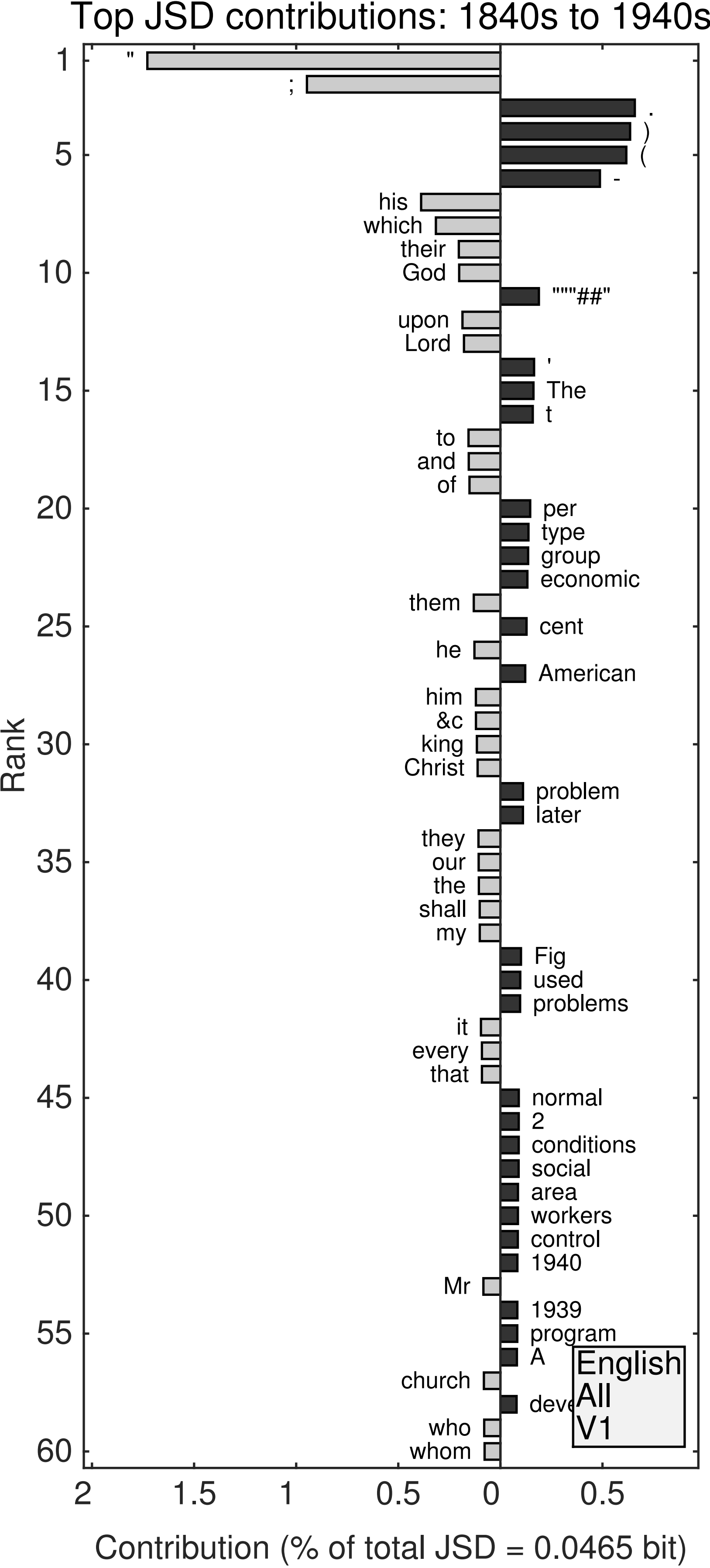

Top JSD contributions: 1840s to 1940s
Rank
Contribution (% of total JSD = 0.0465 bit)
"
;
.
)
(
-
his
which
their
God
"""##"
upon
Lord
'
The
t
to
and
of
per
type
group
economic
them
cent
he
American
him
&c
king
Christ
problem
later
they
our
the
shall
my
Fig
used
problems
it
every
that
normal
2
conditions
social
area
workers
control
1940
Mr
1939
program
A
church
who
whom
English
All
V1

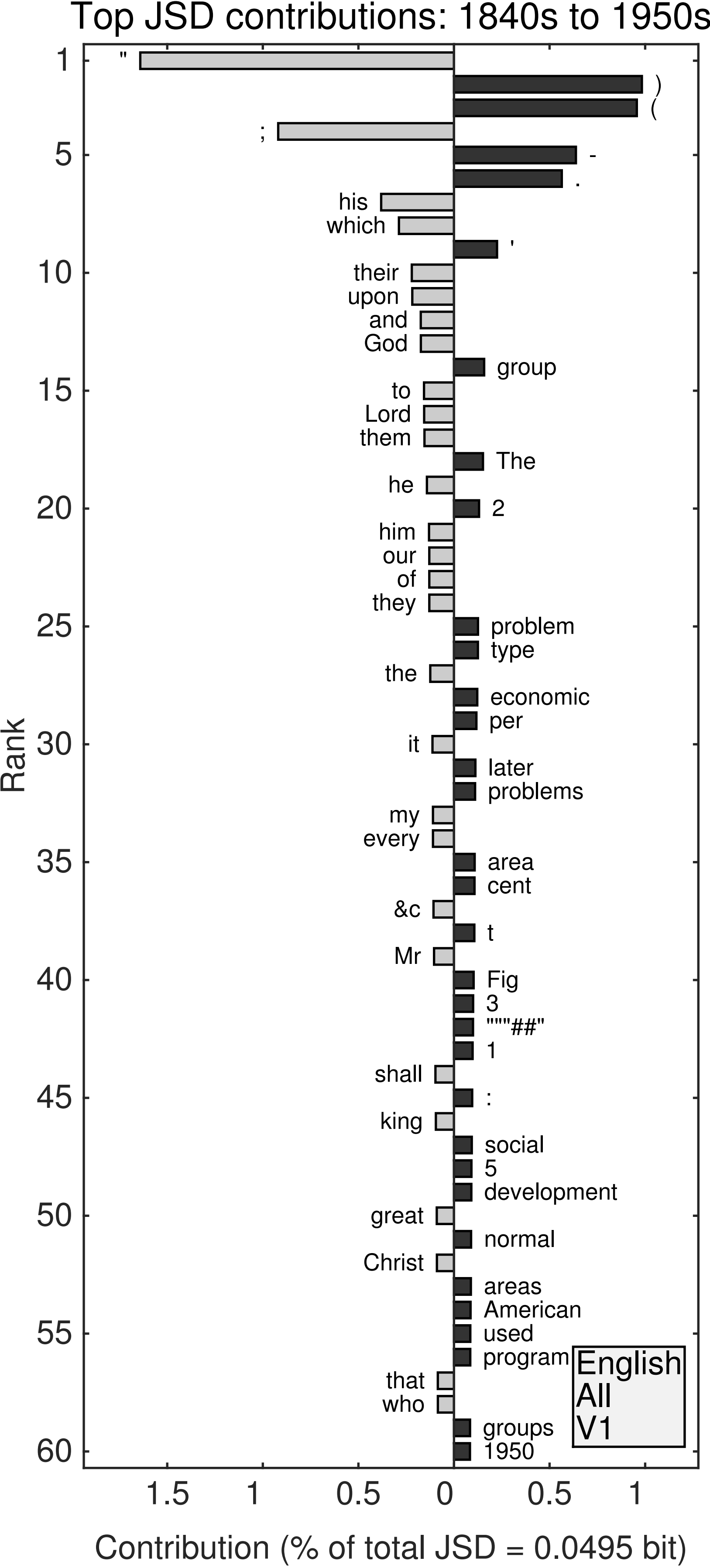

Top JSD contributions: 1840s to 1950s
Rank
Contribution (% of total JSD = 0.0495 bit)
"
)
(
;
-
.
his
which
'
their
upon
and
God
group
to
Lord
them
The
he
2
him
our
of
they
problem
type
the
economic
per
it
later
problems
my
every
area
cent
&c
t
Mr
Fig
3
"""##"
1
shall
:
king
social
5
development
great
normal
Christ
areas
American
used
program
that
who
groups
1950
English
All
V1

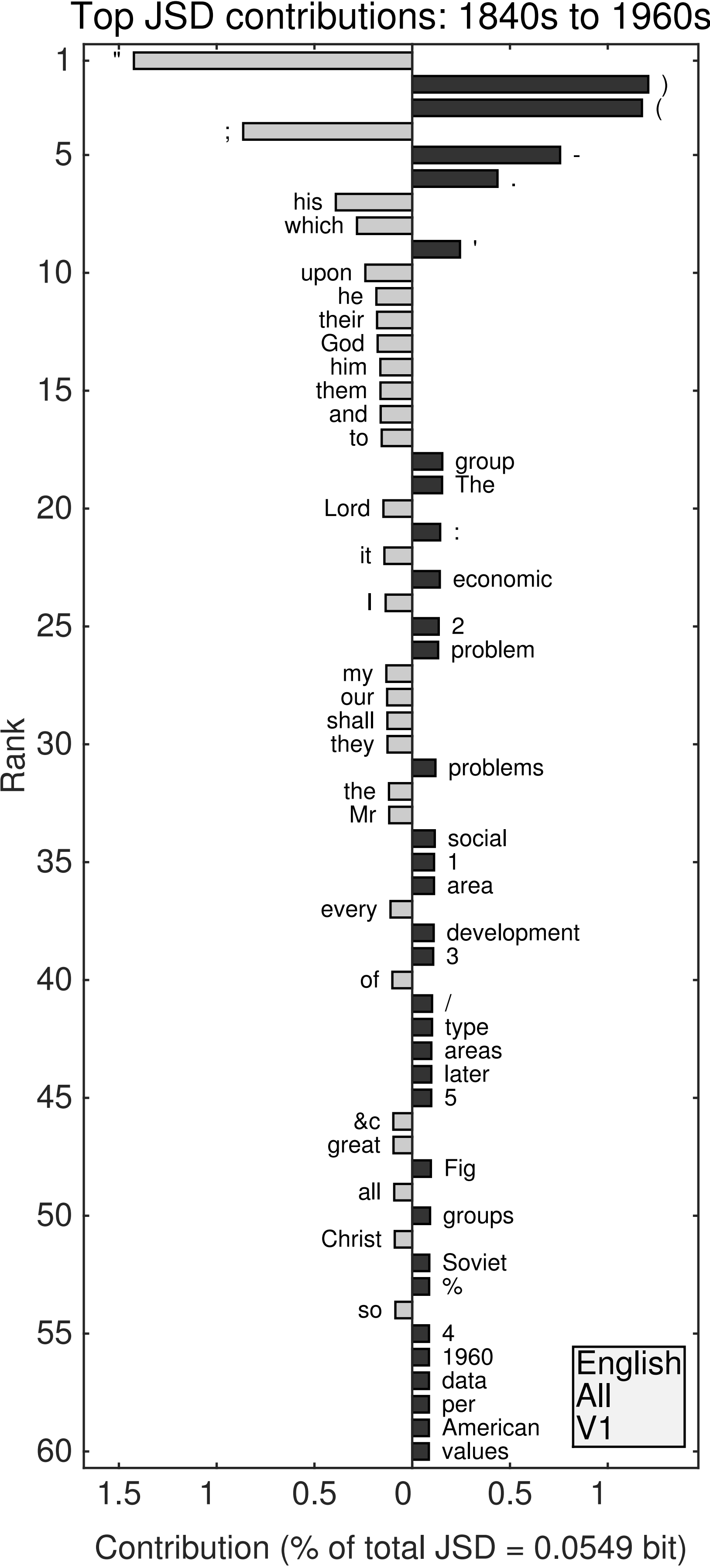

Top JSD contributions: 1840s to 1960s
Rank
Contribution (% of total JSD = 0.0549 bit)
1.5
1
0.5
0
0.5
1
1
5
10
15
20
25
30
35
40
45
50
55
60
"
)
(
;
-
.
his
which
'
upon
he
their
God
him
them
and
to
group
The
Lord
:
it
economic
I
2
problem
my
our
shall
they
problems
the
Mr
social
1
area
every
development
3
of
/
type
areas
later
5
&c
great
Fig
all
groups
Christ
Soviet
%
so
4
1960
data
per
American
values
English
All
V1

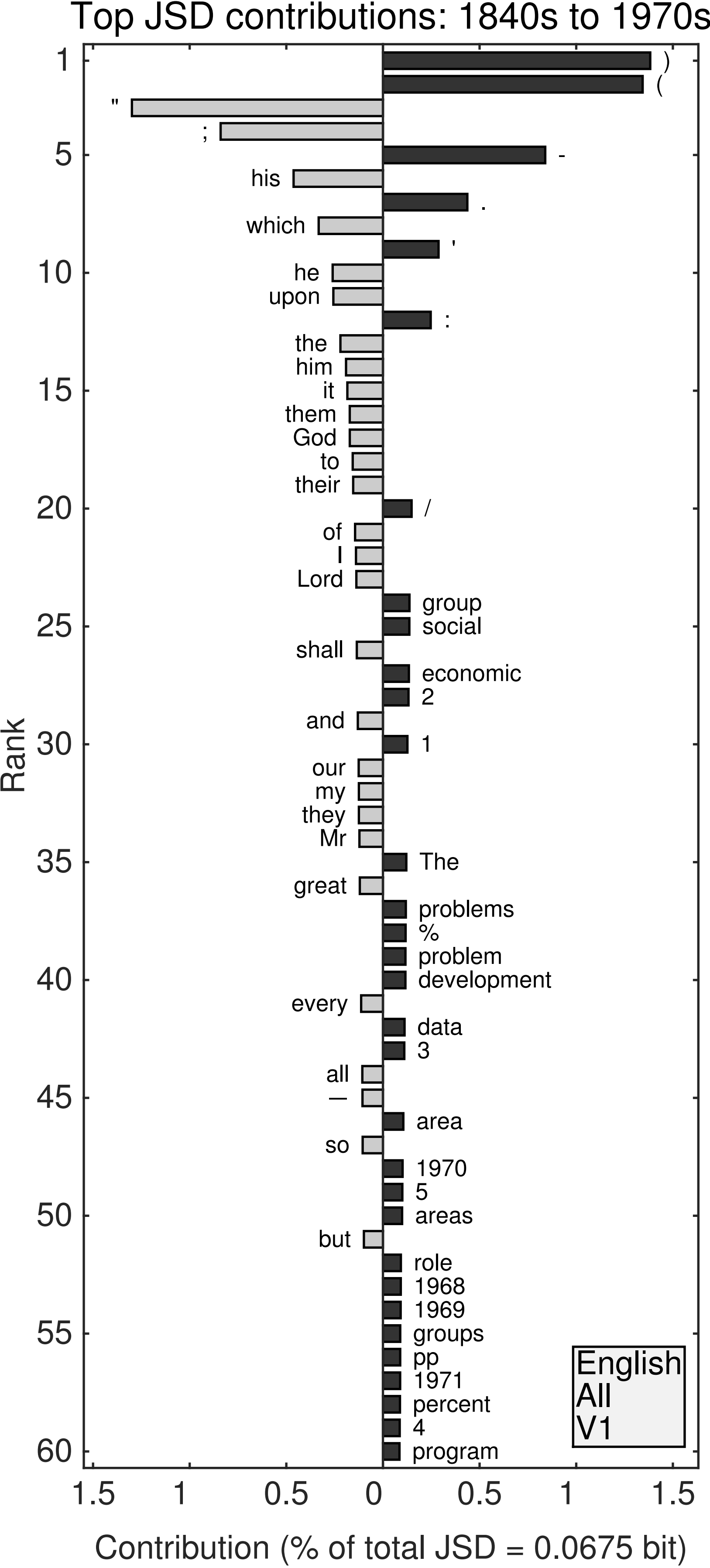

Top JSD contributions: 1840s to 1970s
Rank
Contribution (% of total JSD = 0.0675 bit)
)
(
"
;
-
his
.
which
'
he
upon
:
the
him
it
them
God
to
their
/
of
I
Lord
group
social
shall
economic
2
and
1
our
my
they
Mr
The
great
problems
%
problem
development
every
data
3
all
—
area
so
1970
5
areas
but
role
1968
1969
groups
pp
1971
percent
4
program
English
All
V1

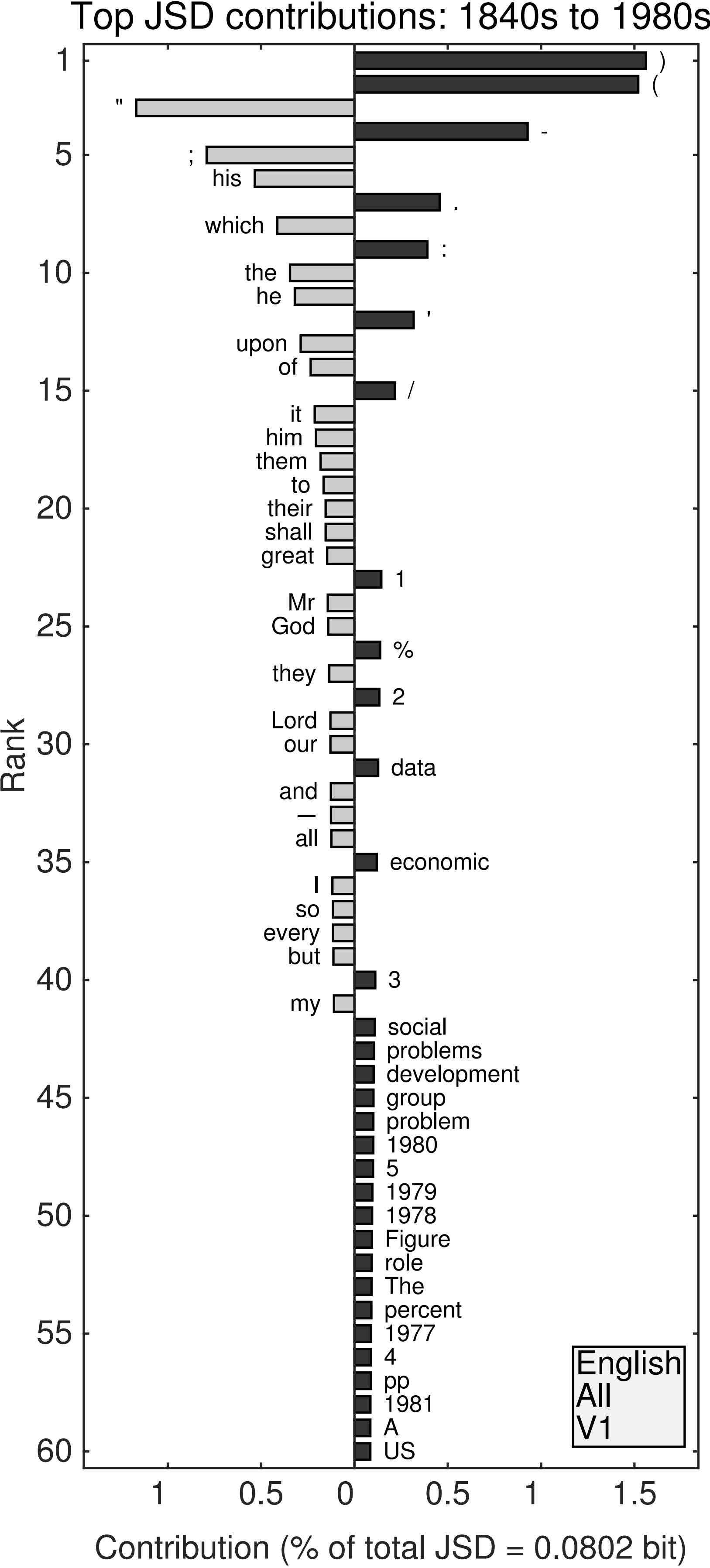

Top JSD contributions: 1840s to 1980s
Rank
Contribution (% of total JSD = 0.0802 bit)
)
(
"
-
;
his
.
which
:
the
he
'
upon
of
/
it
him
them
to
their
shall
great
1
Mr
God
%
they
2
Lord
our
data
and
—
all
economic
I
so
every
but
3
my
social
problems
development
group
problem
1980
5
1979
1978
Figure
role
The
percent
1977
4
pp
1981
A
US
English
All
V1

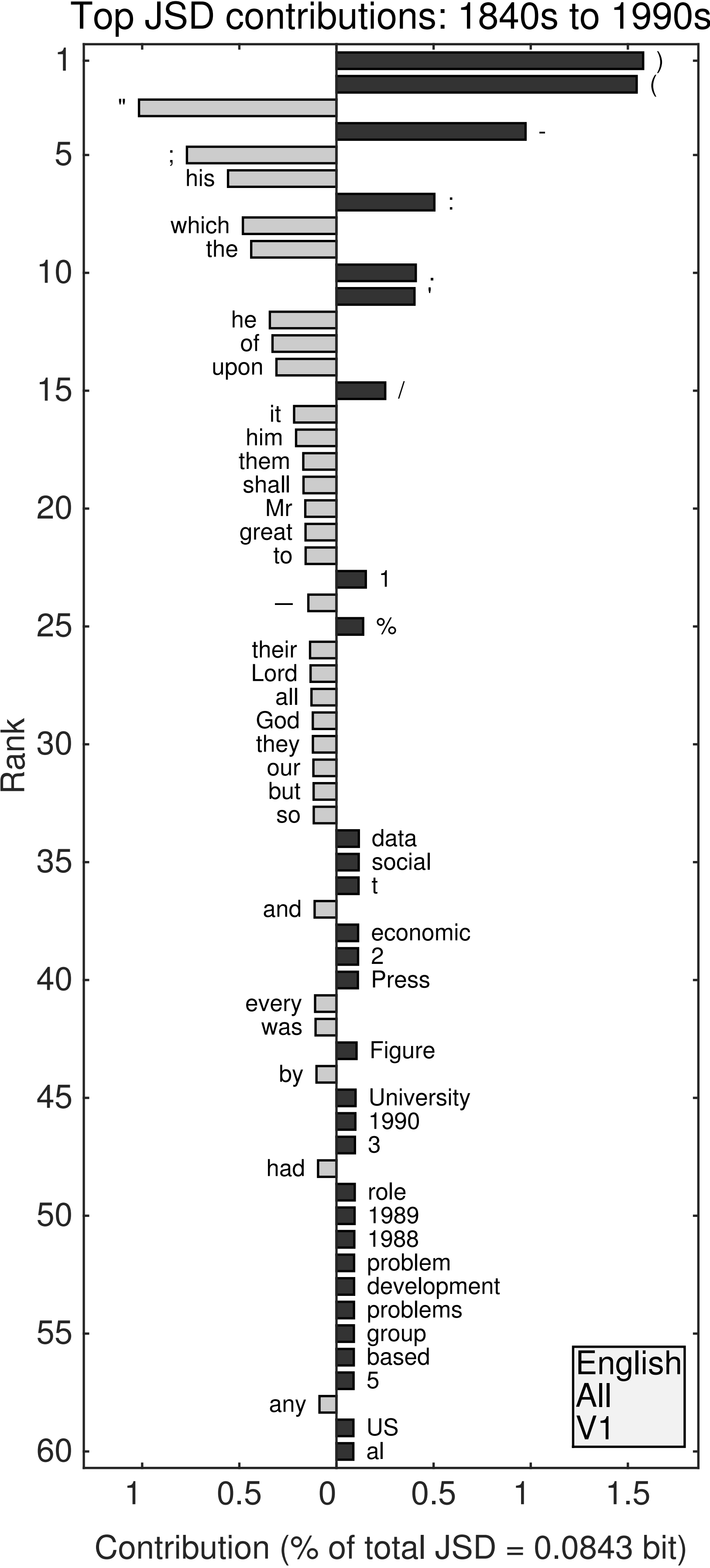
Top JSD contributions: 1840s to 1990s
Rank
Contribution (% of total JSD = 0.0843 bit)
)
(
"
-
;
his
:
which
the
;
'
he
of
upon
/
it
him
them
shall
Mr
great
to
1
—
%
their
Lord
all
God
they
our
but
so
data
social
t
and
economic
2
Press
every
was
Figure
by
University
1990
3
had
role
1989
1988
problem
development
problems
group
based
5
any
US
al
English
All
V1

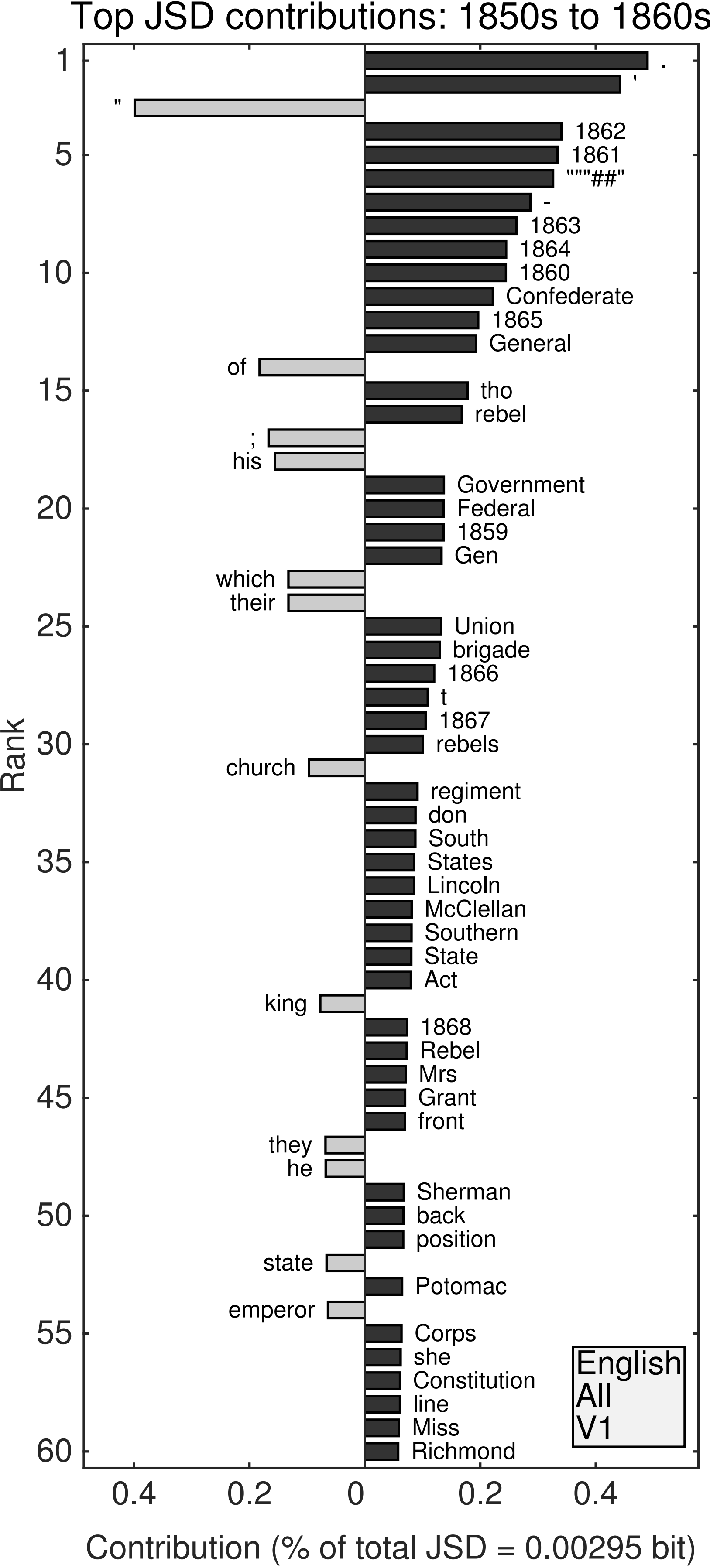

Top JSD contributions: 1850s to 1860s
.
'
"
1862
1861
"""##"
-
1863
1864
1860
Confederate
1865
General
of
tho
rebel
;
his
Government
Federal
1859
Gen
which
their
Union
brigade
1866
t
1867
rebels
church
regiment
don
South
States
Lincoln
McClellan
Southern
State
Act
king
1868
Rebel
Mrs
Grant
front
they
he
Sherman
back
position
state
Potomac
emperor
Corps
she
Constitution
line
Miss
Richmond
English
All
V1
Rank
1
5
10
15
20
25
30
35
40
45
50
55
60
0.4
0.2
0
0.2
0.4
Contribution (% of total JSD = 0.00295 bit)

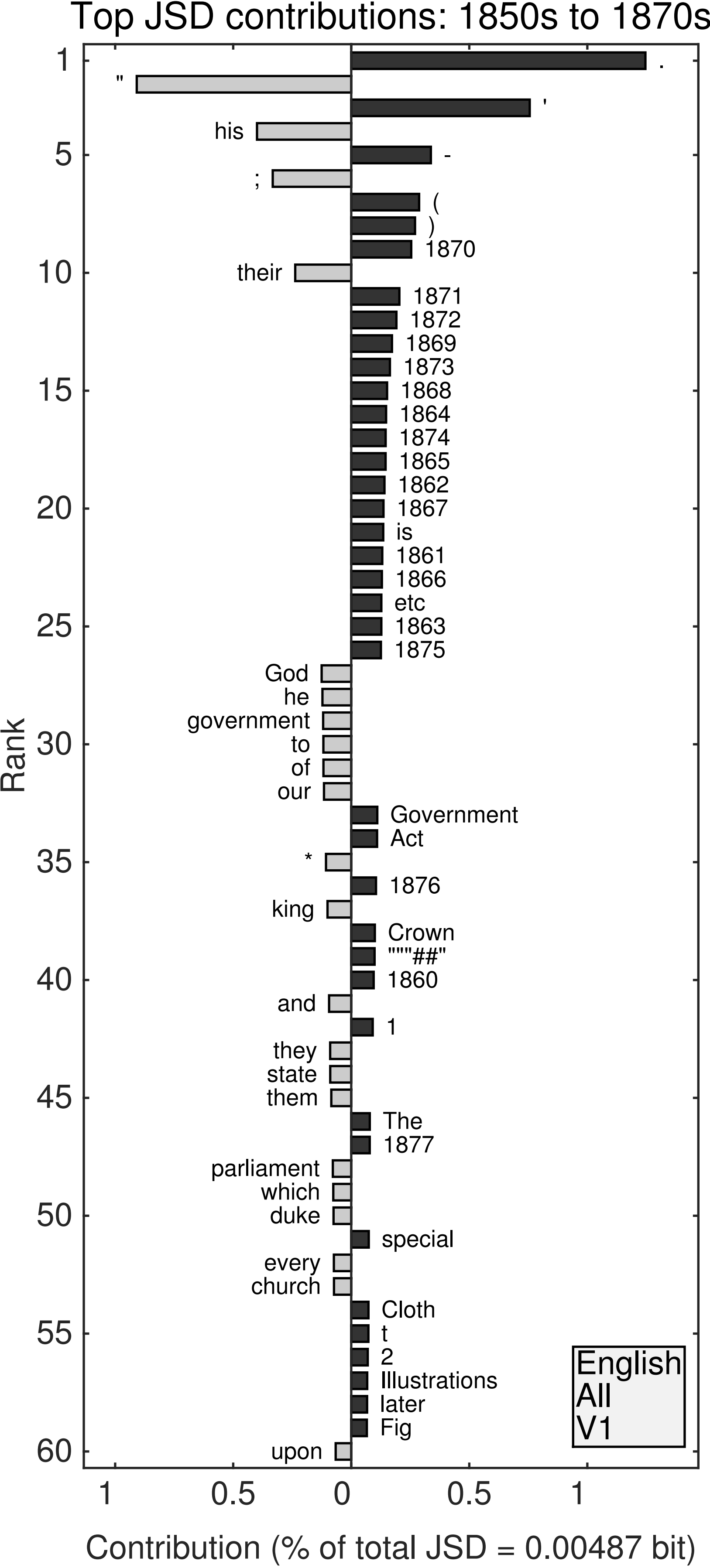
Top JSD contributions: 1850s to 1870s
Rank
Contribution (% of total JSD = 0.00487 bit)
.
"
'
his
-
;
(
)
1870
their
1871
1872
1869
1873
1868
1864
1874
1865
1862
1867
is
1861
1866
etc
1863
1875
God
he
government
to
of
our
Government
Act
*
1876
king
Crown
"""##"
1860
and
1
they
state
them
The
1877
parliament
which
duke
special
every
church
Cloth
t
2
Illustrations
later
Fig
upon
English
All
V1

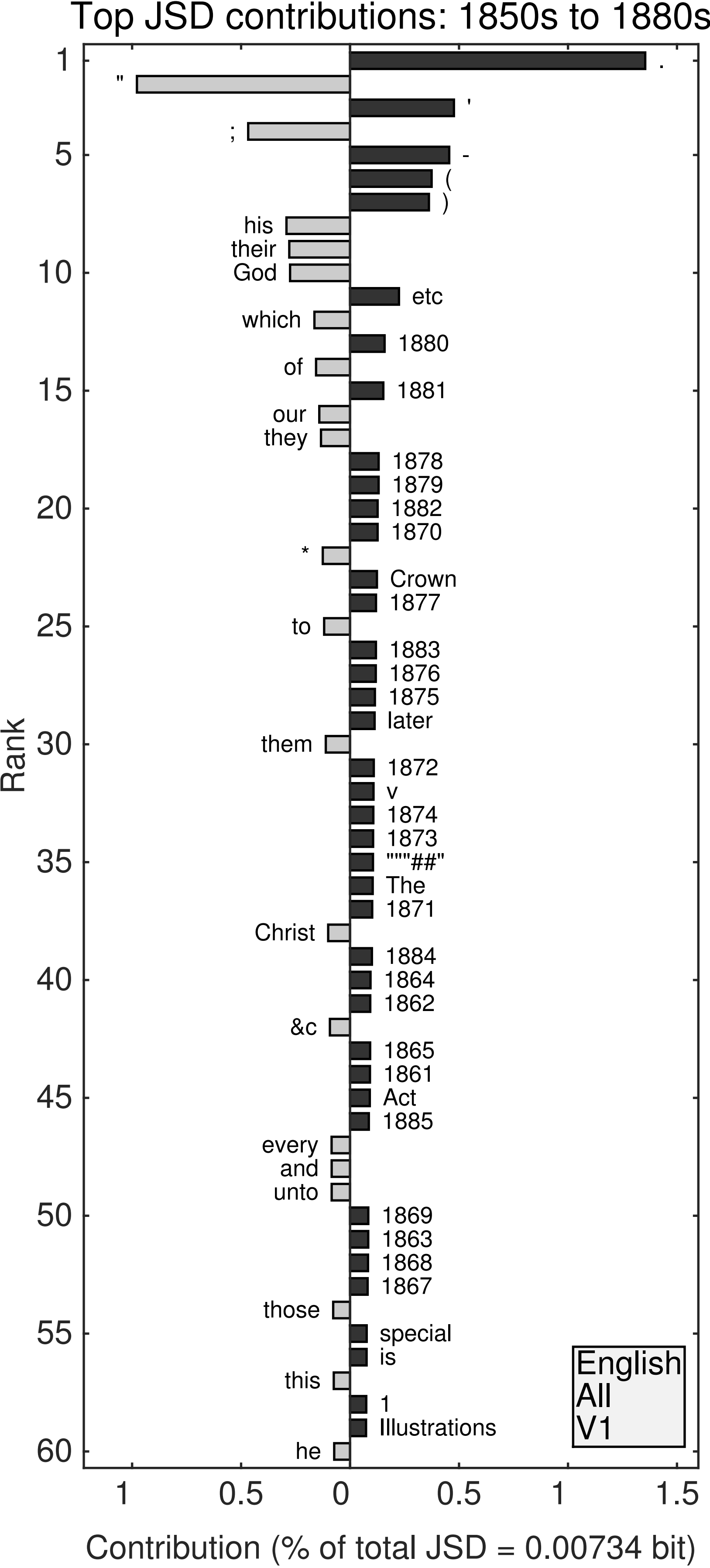
Top JSD contributions: 1850s to 1880s
Rank
1
5
10
15
20
25
30
35
40
45
50
55
60
.
"
'
;
-
(
)
his
their
God
etc
which
1880
of
1881
our
they
1878
1879
1882
1870
*
Crown
1877
to
1883
1876
1875
later
them
1872
v
1874
1873
""""##"
The
1871
Christ
1884
1864
1862
&c
1865
1861
Act
1885
every
and
unto
1869
1863
1868
1867
those
special
is
this
1
Illustrations
he
English
All
V1
1
0.5
0
0.5
1
1.5
Contribution (% of total JSD = 0.00734 bit)

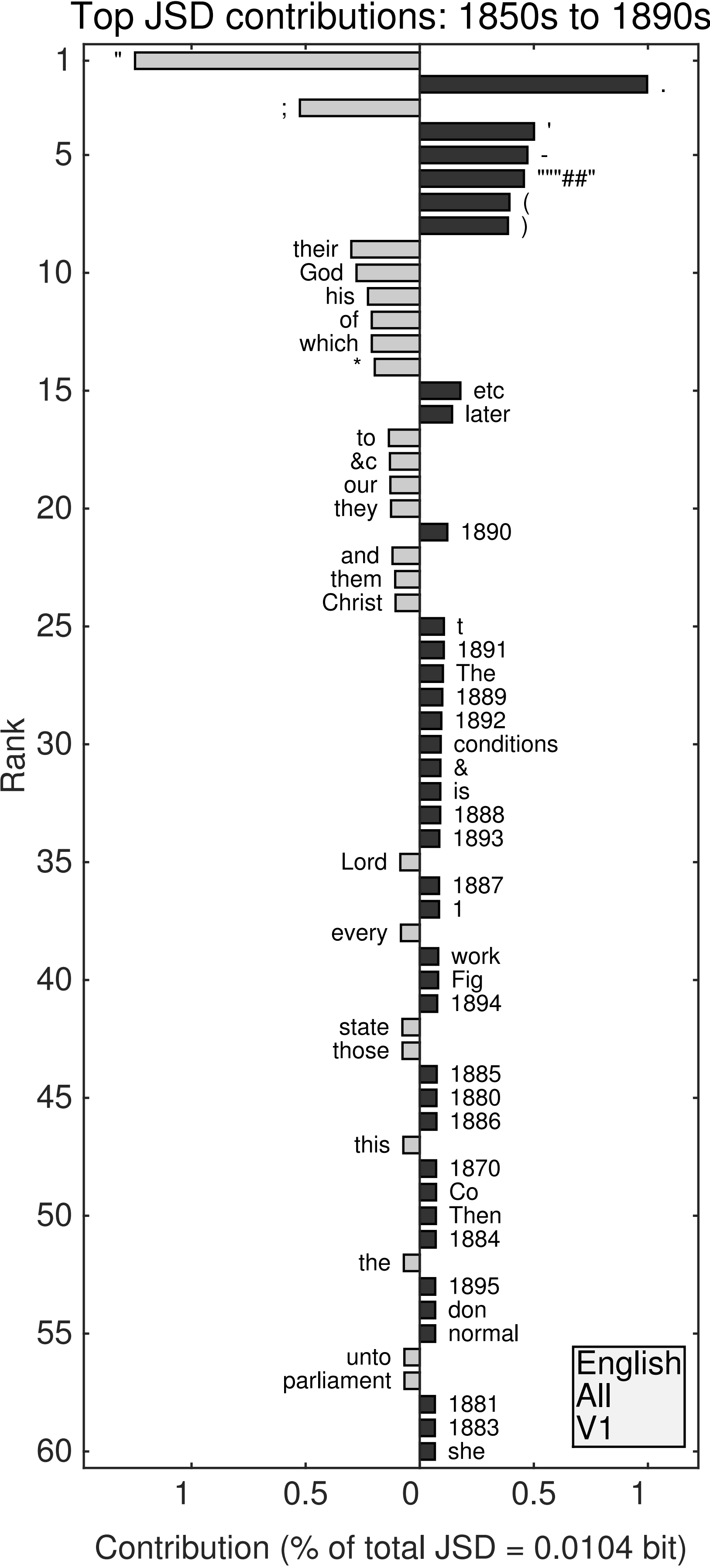
Top JSD contributions: 1850s to 1890s
Rank
Contribution (% of total JSD = 0.0104 bit)
"
.
;
'
-
"""##"
(
)
their
God
his
of
which
*
etc
later
to
&c
our
they
1890
and
them
Christ
t
1891
The
1889
1892
conditions
&
is
1888
1893
Lord
1887
1
every
work
Fig
1894
state
those
1885
1880
1886
this
1870
Co
Then
1884
the
1895
don
normal
unto
parliament
1881
1883
she
English
All
V1

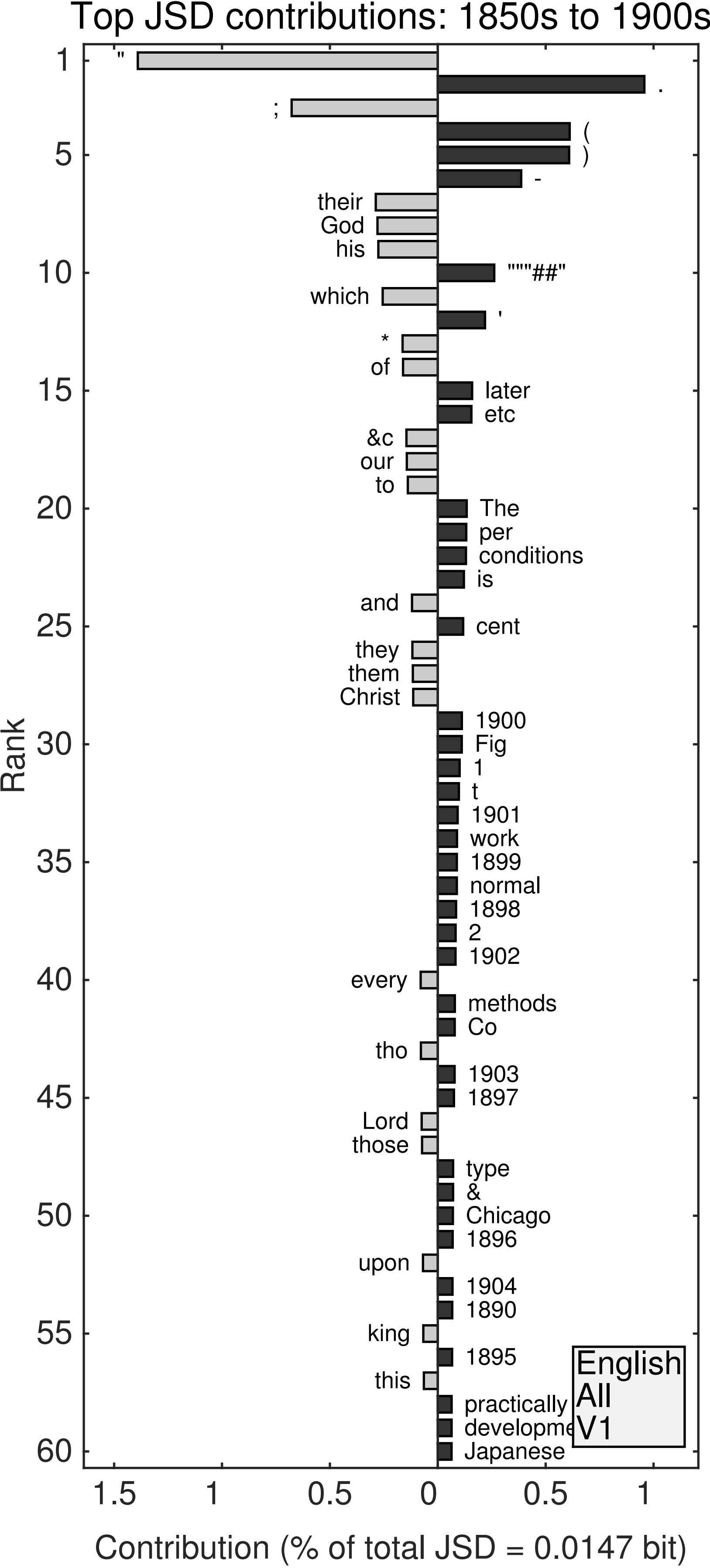

Top JSD contributions: 1850s to 1900s
Rank
Contribution (% of total JSD = 0.0147 bit)
"
.
;
(
)
-
their
God
his
"""##"
which
'
*
of
later
etc
&c
our
to
The
per
conditions
is
and
cent
they
them
Christ
1900
Fig
1
t
1901
work
1899
normal
1898
2
1902
every
methods
Co
tho
1903
1897
Lord
those
type
&
Chicago
1896
upon
1904
1890
king
1895
this
practically
developme
Japanese
English
All
V1

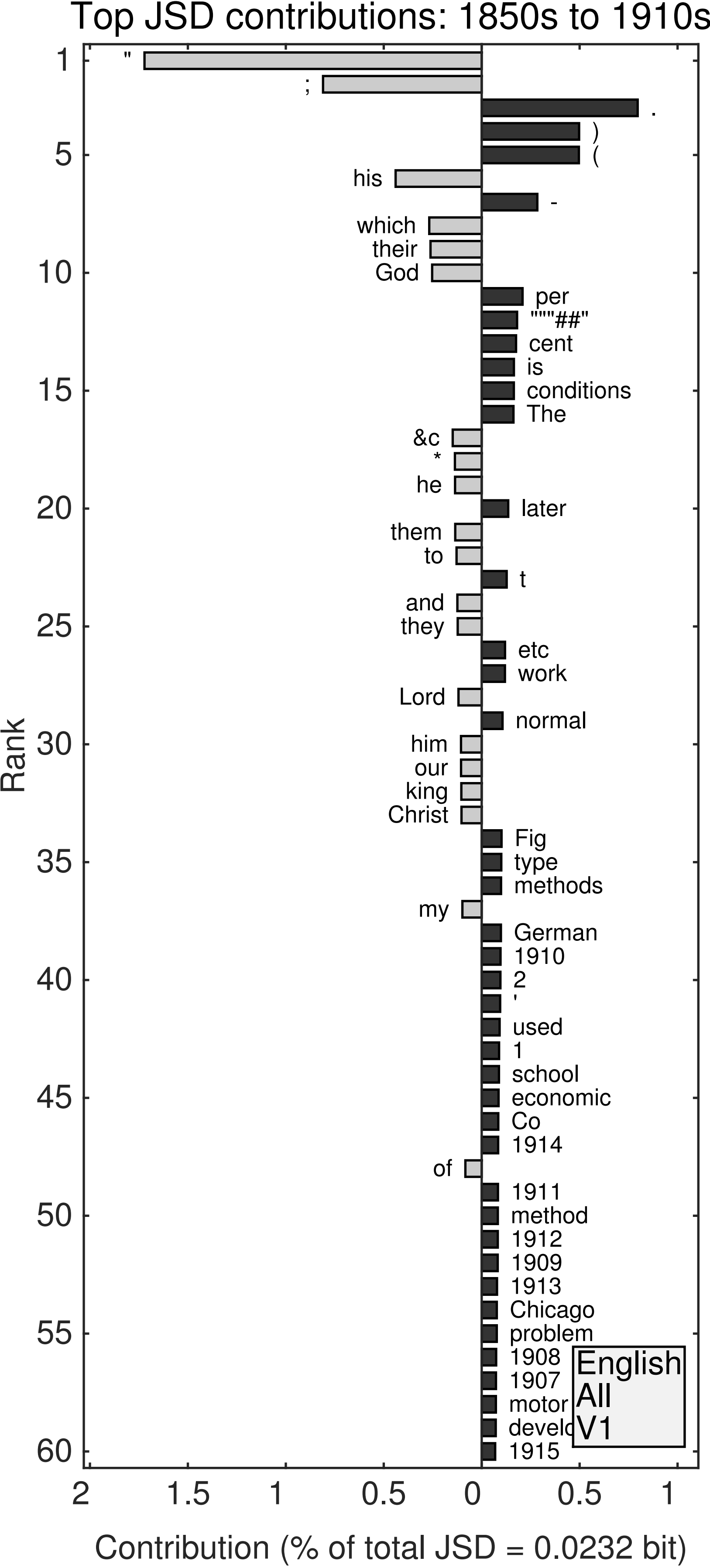
Top JSD contributions: 1850s to 1910s
Rank
Contribution (% of total JSD = 0.0232 bit)
"
;
.
)
(
his
-
which
their
God
per
"""##"
cent
is
conditions
The
&c
*
he
later
them
to
t
and
they
etc
work
Lord
normal
him
our
king
Christ
Fig
type
methods
my
German
1910
2
'
used
1
school
economic
Co
1914
of
1911
method
1912
1909
1913
Chicago
problem
1908
1907
motor
develo
1915
English
All
V1

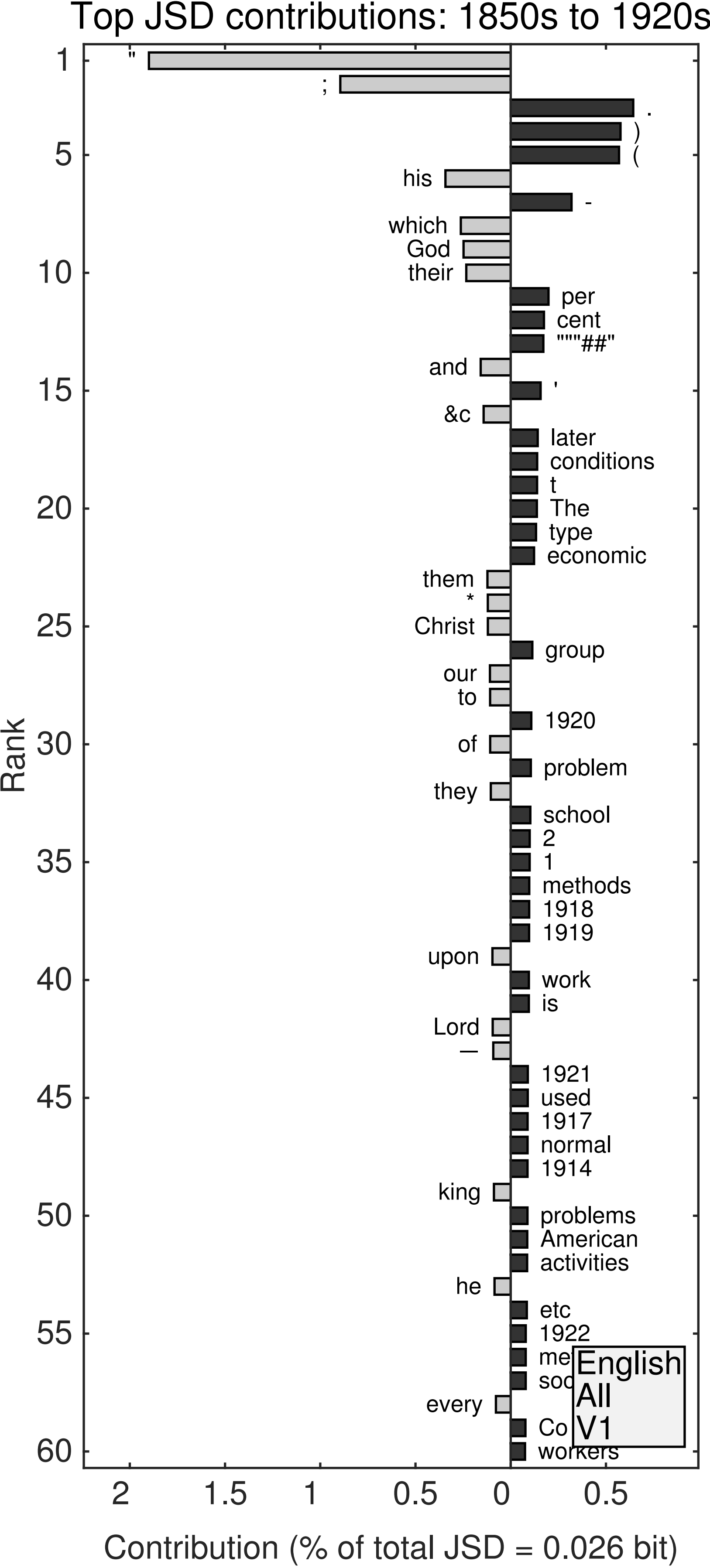

Top JSD contributions: 1850s to 1920s
Rank
Contribution (% of total JSD = 0.026 bit)
"
;
.
)
(
his
-
which
God
their
per
cent
"""##"
and
'
&c
later
conditions
t
The
type
economic
them
*
Christ
group
our
to
1920
of
problem
they
school
2
1
methods
1918
1919
upon
work
is
Lord
—
1921
used
1917
normal
1914
king
problems
American
activities
he
etc
1922
every
workers
English
All
V1

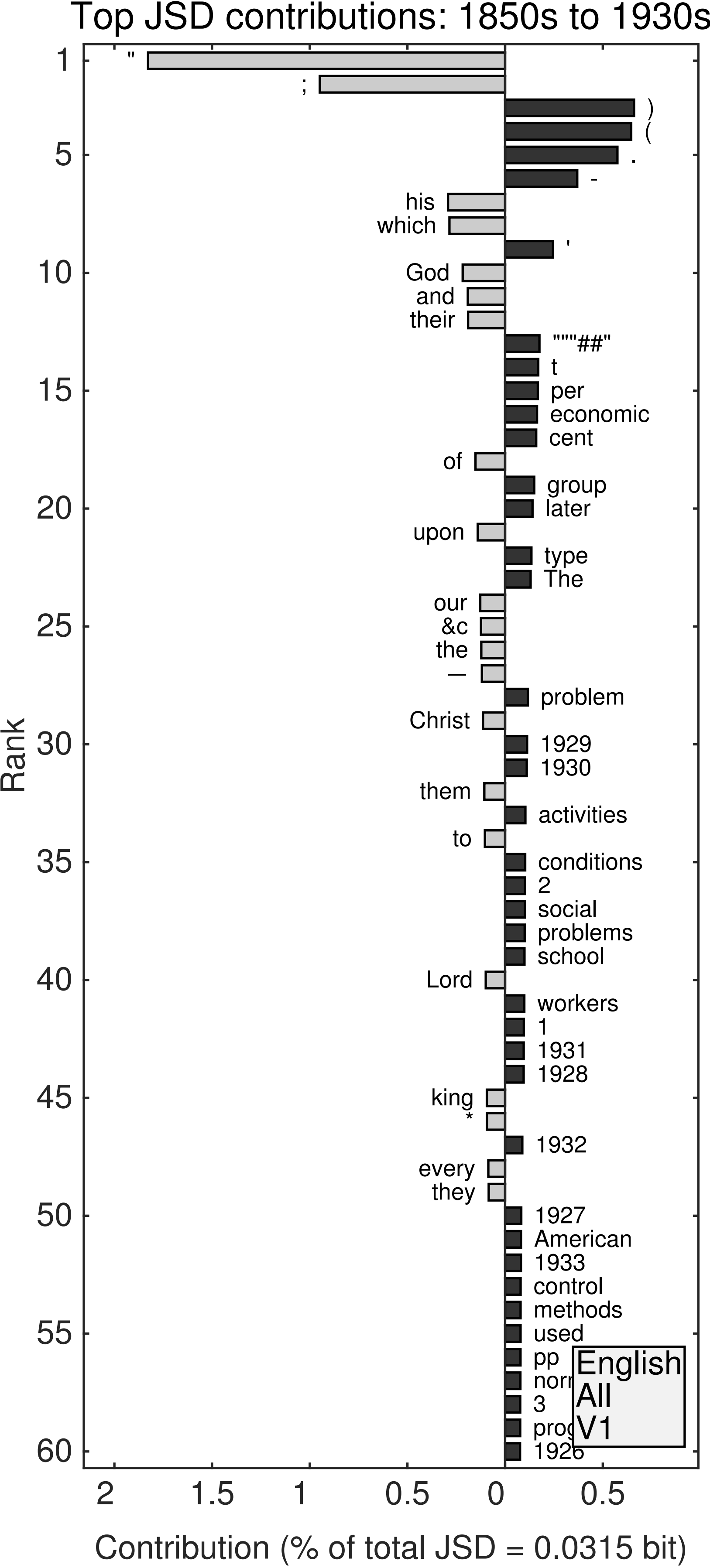

Top JSD contributions: 1850s to 1930s
Rank
Contribution (% of total JSD = 0.0315 bit)
"
;
)
(
.
-
his
which
'
God
and
their
"""##"
t
per
economic
cent
of
group
later
upon
type
The
our
&c
the
—
problem
Christ
1929
1930
them
activities
to
conditions
2
social
problems
school
Lord
workers
1
1931
1928
king
*
1932
every
they
1927
American
1933
control
methods
used
pp
3
1926
English
All
V1

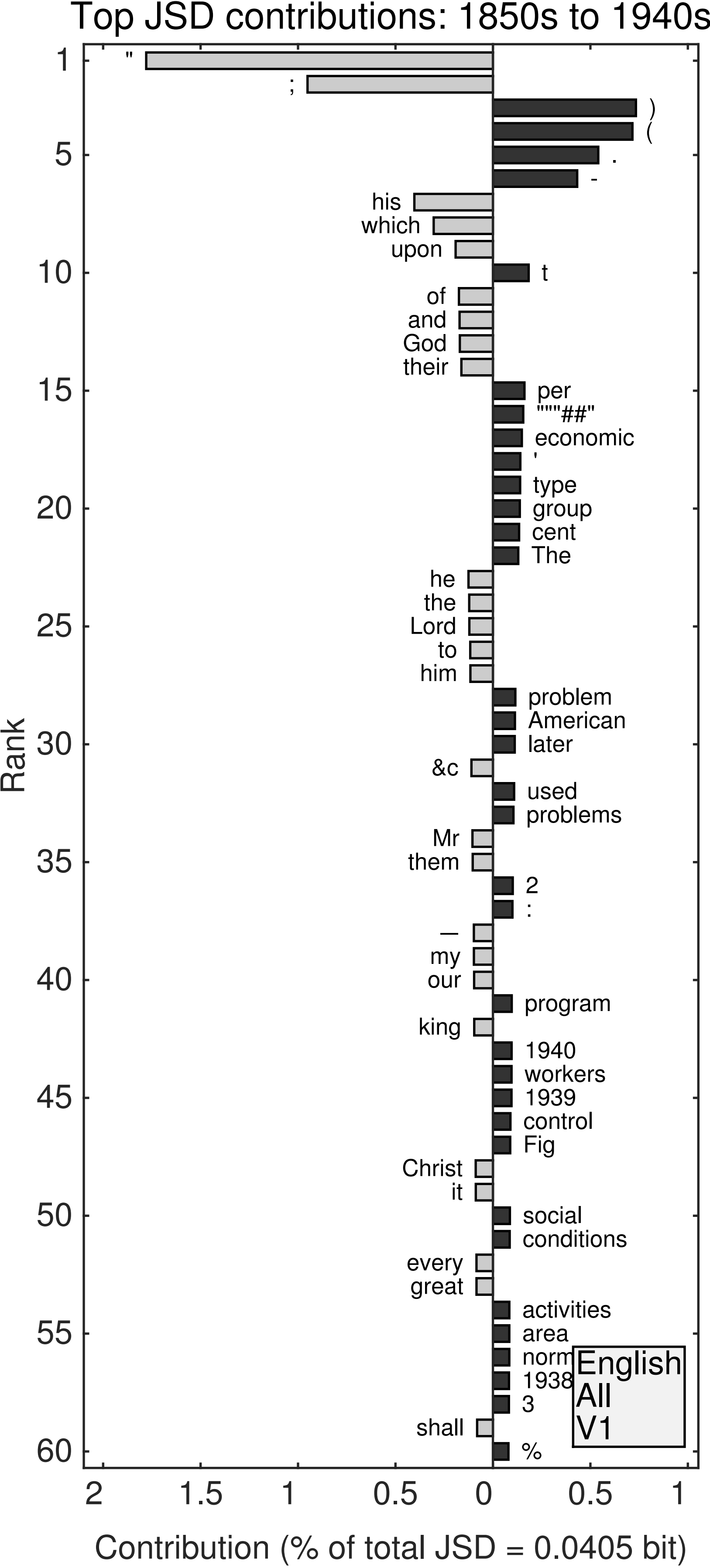
Top JSD contributions: 1850s to 1940s
Rank
Contribution (% of total JSD = 0.0405 bit)
"
;
)
(
.
-
his
which
upon
t
of
and
God
their
per
"""##"
economic
'
type
group
cent
The
he
the
Lord
to
him
problem
American
later
&c
used
problems
Mr
them
2
:
—
my
our
program
king
1940
workers
1939
control
Fig
Christ
it
social
conditions
every
great
activities
area
norm
1938
3
shall
%
English
All
V1

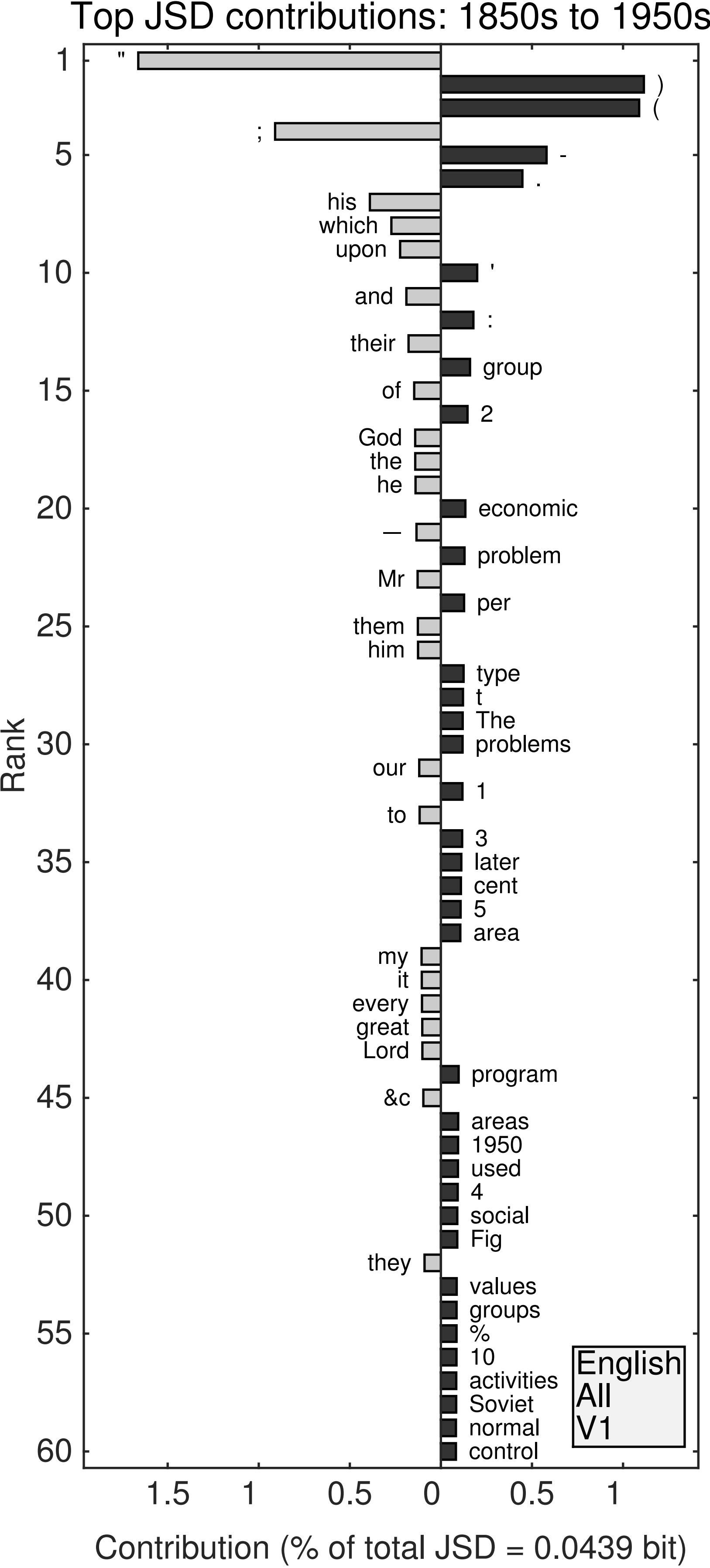

Top JSD contributions: 1850s to 1950s
Rank
Contribution (% of total JSD = 0.0439 bit)
"
)
(
;
-
.
his
which
upon
'
and
:
their
group
of
2
God
the
he
economic
—
problem
Mr
per
them
him
type
t
The
problems
our
1
to
3
later
cent
5
area
my
it
every
great
Lord
program
&c
areas
1950
used
4
social
Fig
they
values
groups
%
10
activities
Soviet
normal
control
English
All
V1

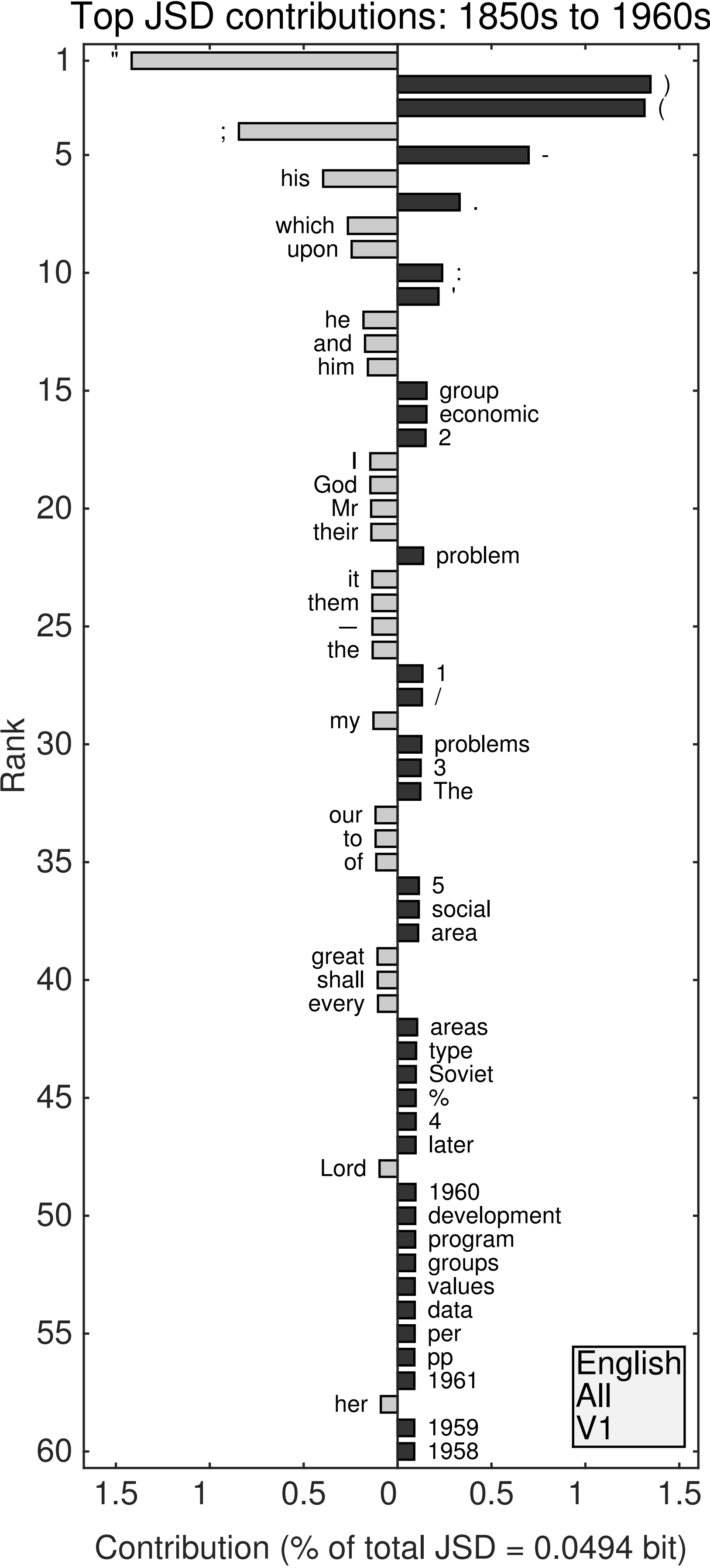

Top JSD contributions: 1850s to 1960s
Rank
Contribution (% of total JSD = 0.0494 bit)
"
)
(
;
-
his
.
which
upon
:
'
he
and
him
group
economic
2
I
God
Mr
their
problem
it
them
—
the
1
/
my
problems
3
The
our
to
of
5
social
area
great
shall
every
areas
type
Soviet
%
4
later
Lord
1960
development
program
groups
values
data
per
pp
1961
her
1959
1958
English
All
V1

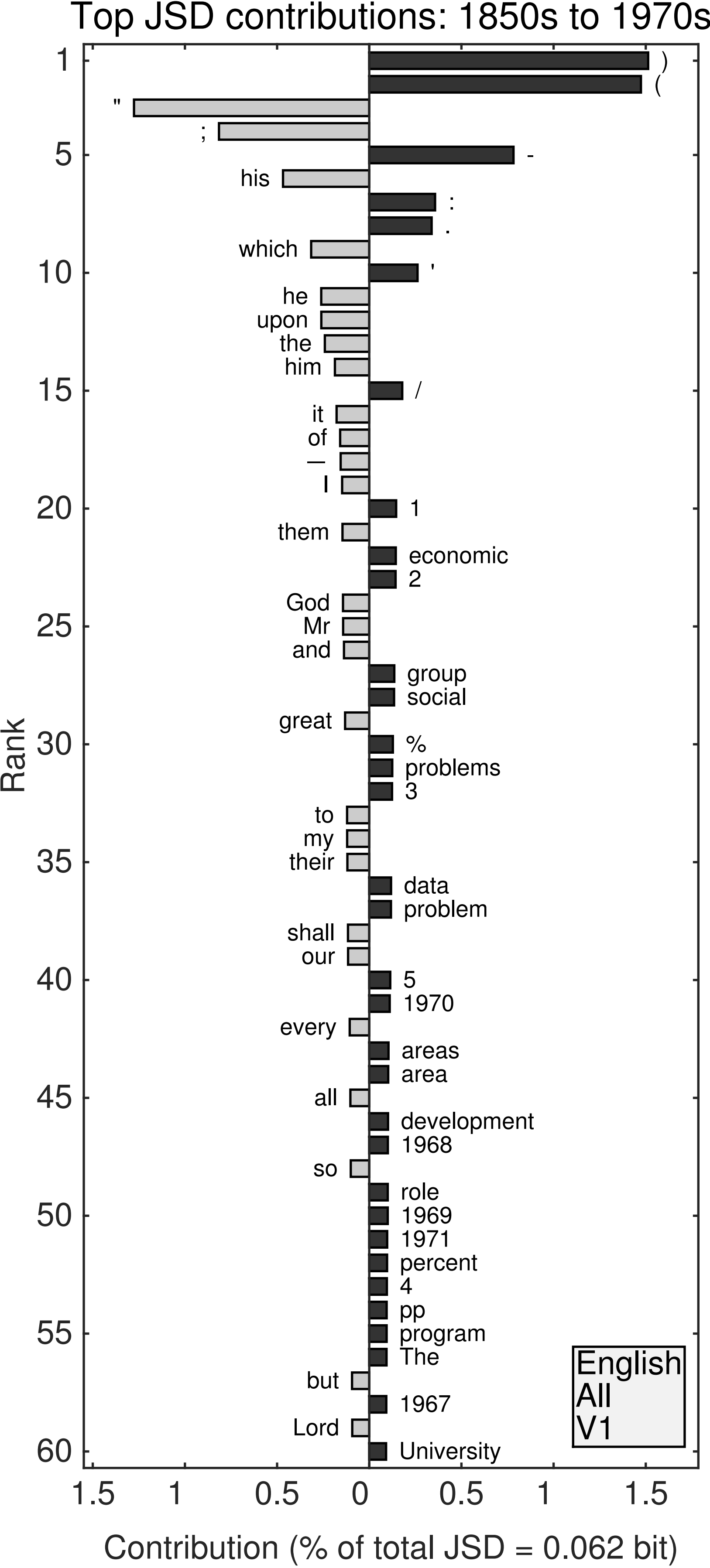
Top JSD contributions: 1850s to 1970s
Rank
Contribution (% of total JSD = 0.062 bit)
)
(
"
;
-
his
:
.
which
'
he
upon
the
him
/
it
of
—
I
1
them
economic
2
God
Mr
and
group
social
great
%
problems
3
to
my
their
data
problem
shall
our
5
1970
every
areas
area
all
development
1968
so
role
1969
1971
percent
4
pp
program
The
but
1967
Lord
University
English
All
V1

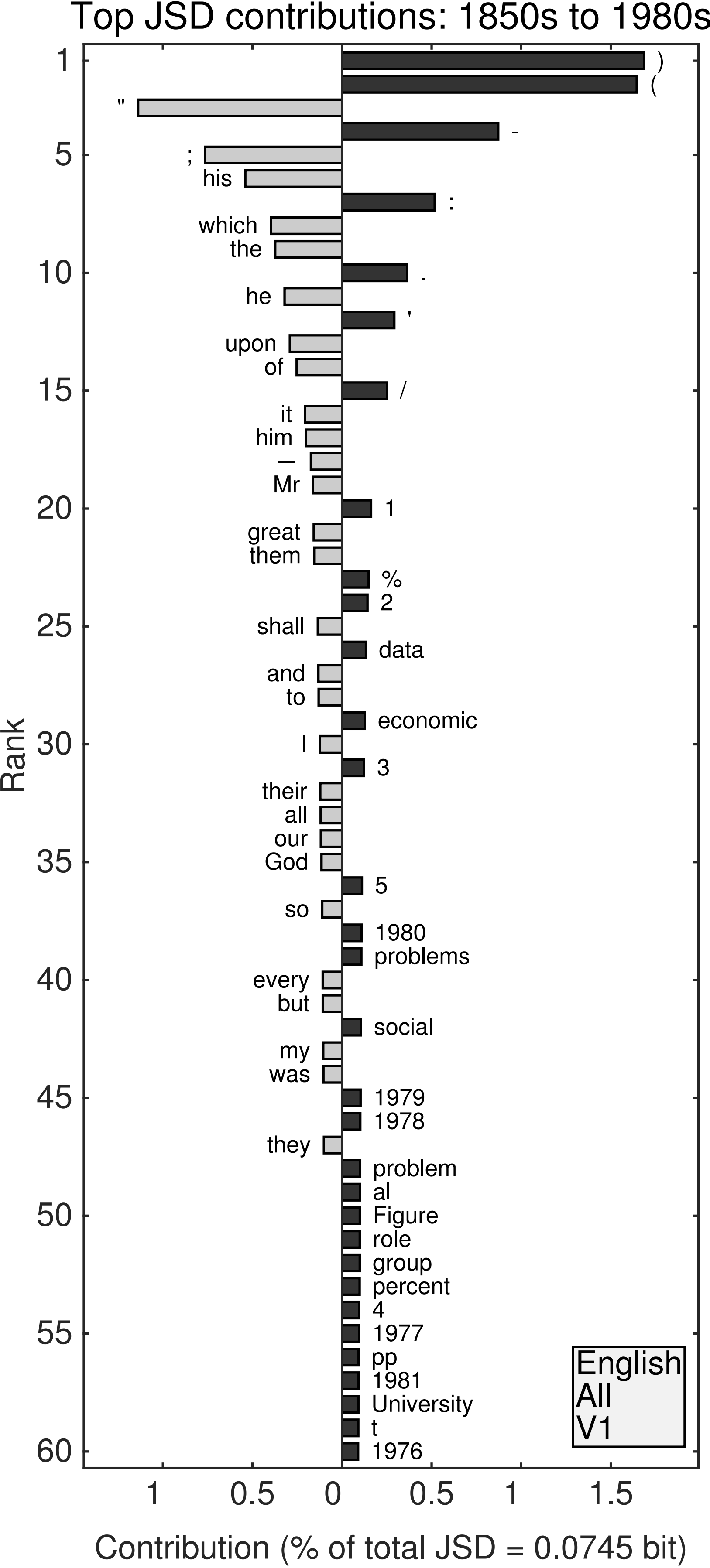
Top JSD contributions: 1850s to 1980s
Rank
Contribution (% of total JSD = 0.0745 bit)
)
(
"
-
;
his
:
which
the
.
he
'
upon
of
/
it
him
—
Mr
1
great
them
%
2
shall
data
and
to
economic
I
3
their
all
our
God
5
so
1980
problems
every
but
social
my
was
1979
1978
they
problem
al
Figure
role
group
percent
4
1977
pp
1981
University
t
1976
English
All
V1

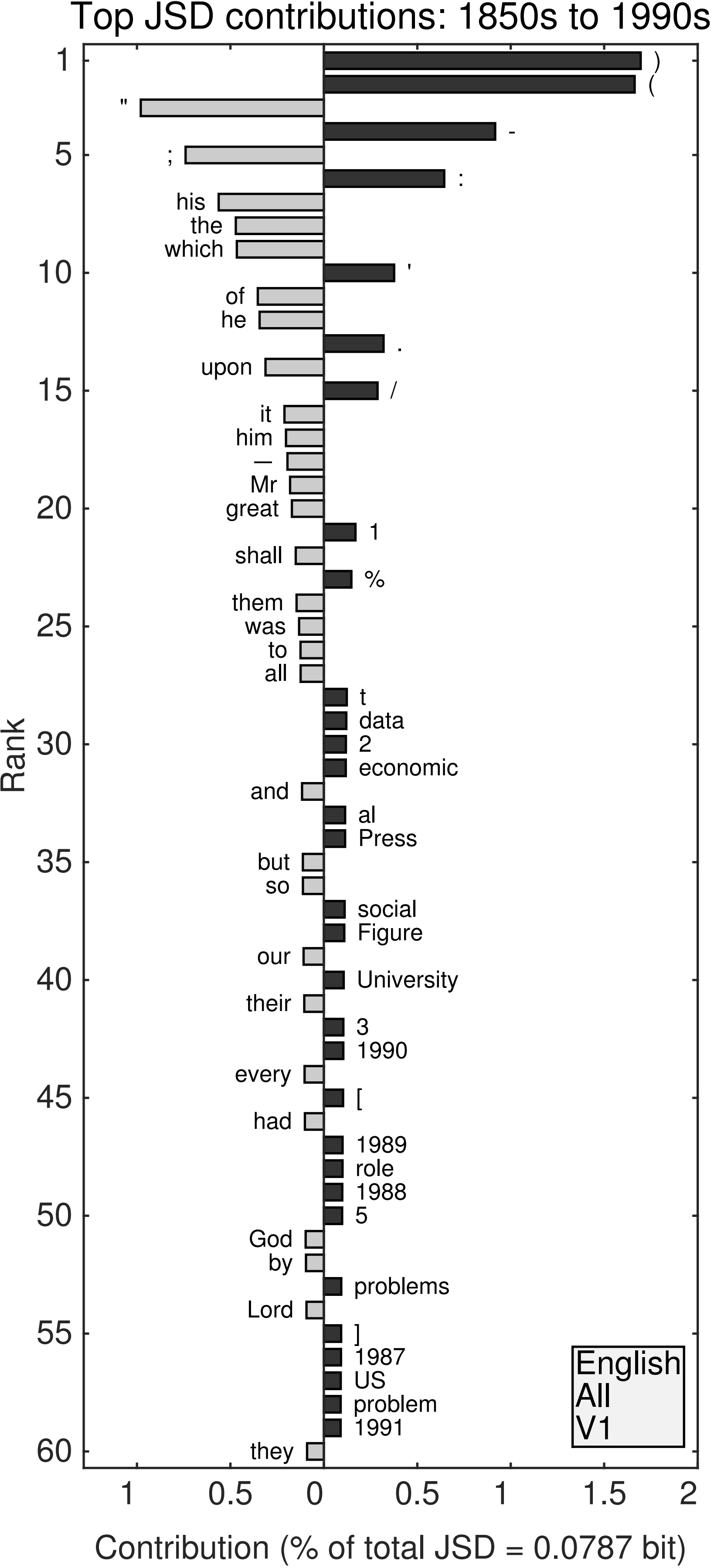
Top JSD contributions: 1850s to 1990s
Rank
Contribution (% of total JSD = 0.0787 bit)
)
(
"
-
;
:
his
the
which
'
of
he
.
upon
/
it
him
—
Mr
great
1
shall
%
them
was
to
all
t
data
2
economic
and
al
Press
but
so
social
Figure
our
University
their
3
1990
every
[
had
1989
role
1988
5
God
by
problems
Lord
]
1987
US
problem
1991
they
English
All
V1

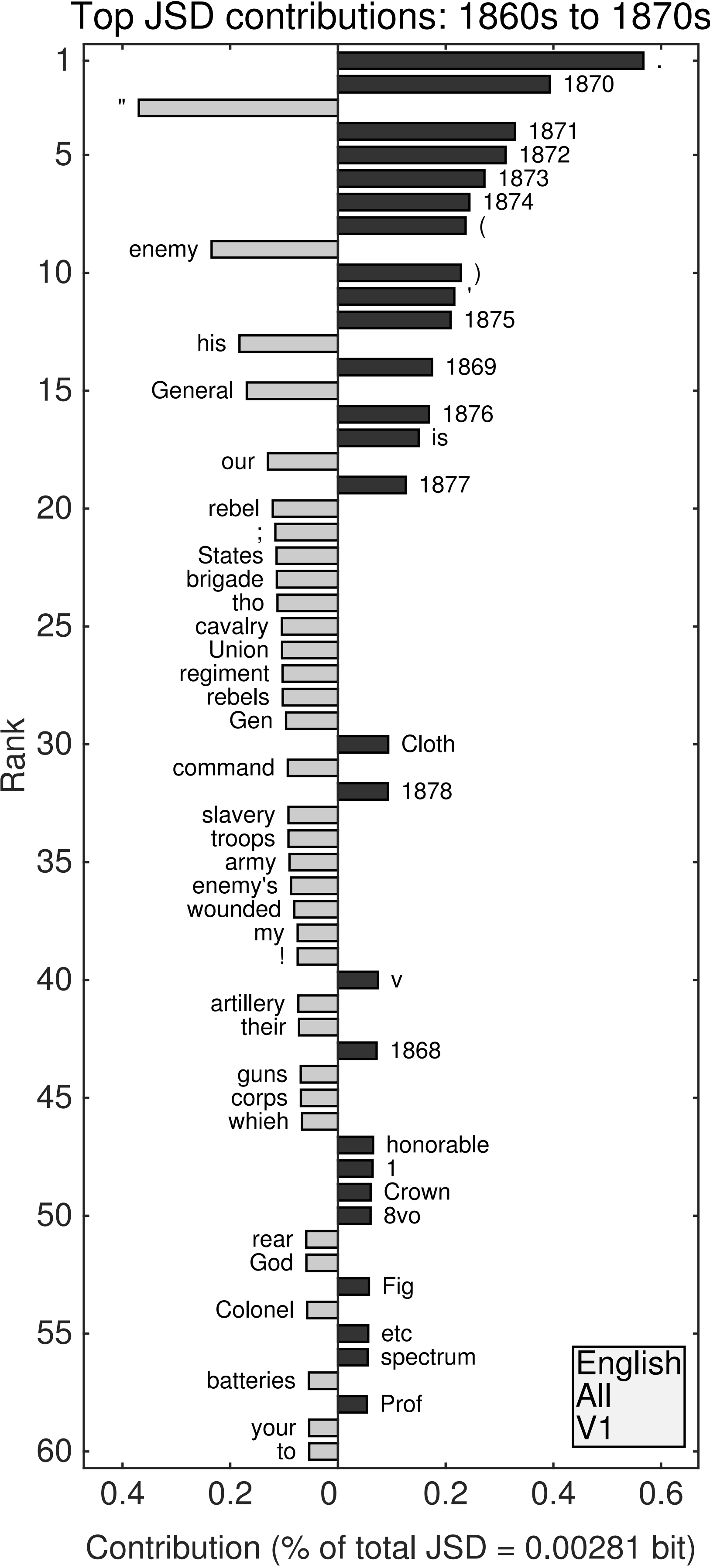
Top JSD contributions: 1860s to 1870s
Rank
Contribution (% of total JSD = 0.00281 bit)
.
1870
"
1871
1872
1873
1874
(
enemy
)
'
1875
his
1869
General
1876
is
our
1877
rebel
;
States
brigade
tho
cavalry
Union
regiment
rebels
Gen
Cloth
command
1878
slavery
troops
army
enemy's
wounded
my
!
v
artillery
their
1868
guns
corps
whieh
honorable
1
Crown
8vo
rear
God
Fig
Colonel
etc
spectrum
batteries
Prof
your
to
English
All
V1

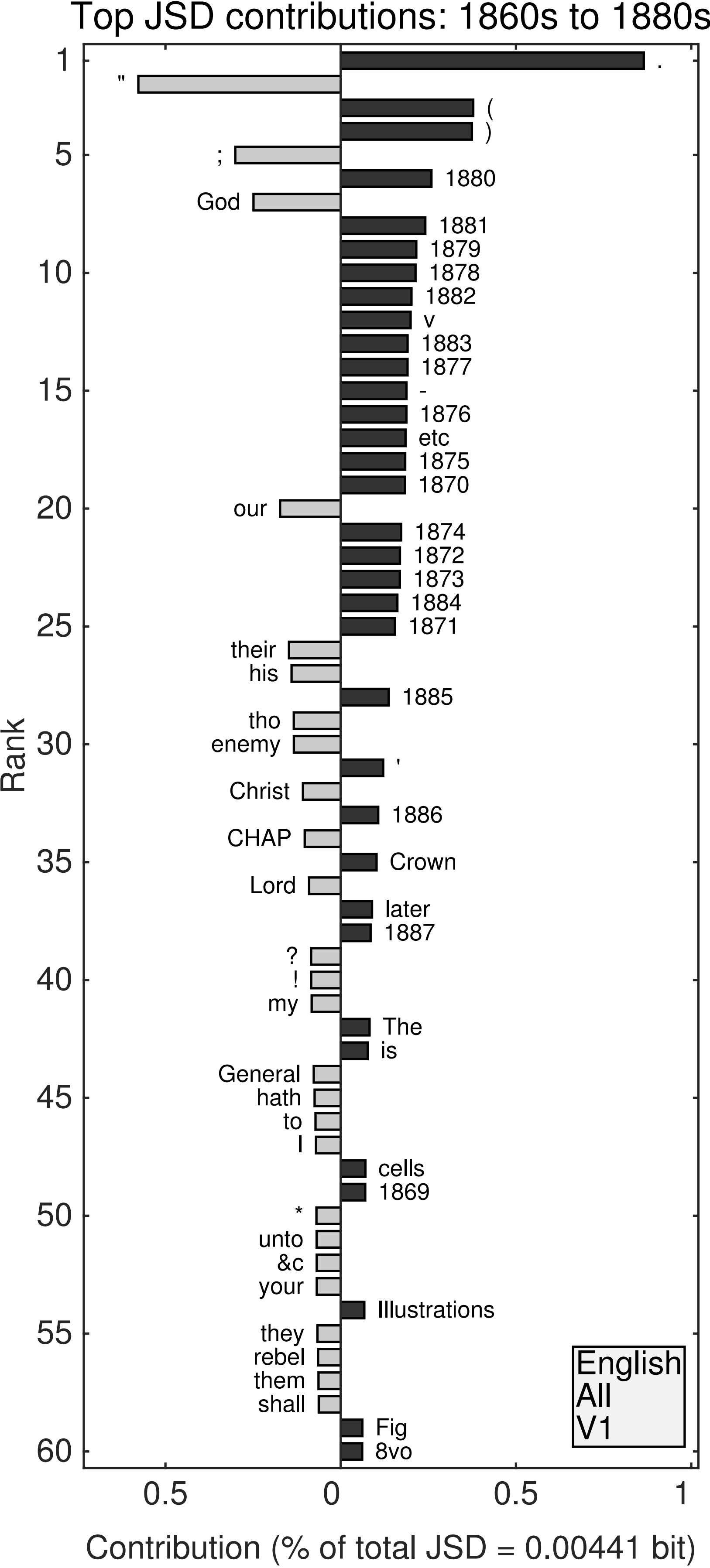
Top JSD contributions: 1860s to 1880s
Rank
Contribution (% of total JSD = 0.00441 bit)
English
All
V1
.
"
(
)
;
1880
God
1881
1879
1878
1882
v
1883
1877
-
1876
etc
1875
1870
our
1874
1872
1873
1884
1871
their
his
1885
tho
enemy
'
Christ
1886
CHAP
Crown
Lord
later
1887
?
!
my
The
is
General
hath
to
I
cells
1869
*
unto
&c
your
Illustrations
they
rebel
them
shall
Fig
8vo

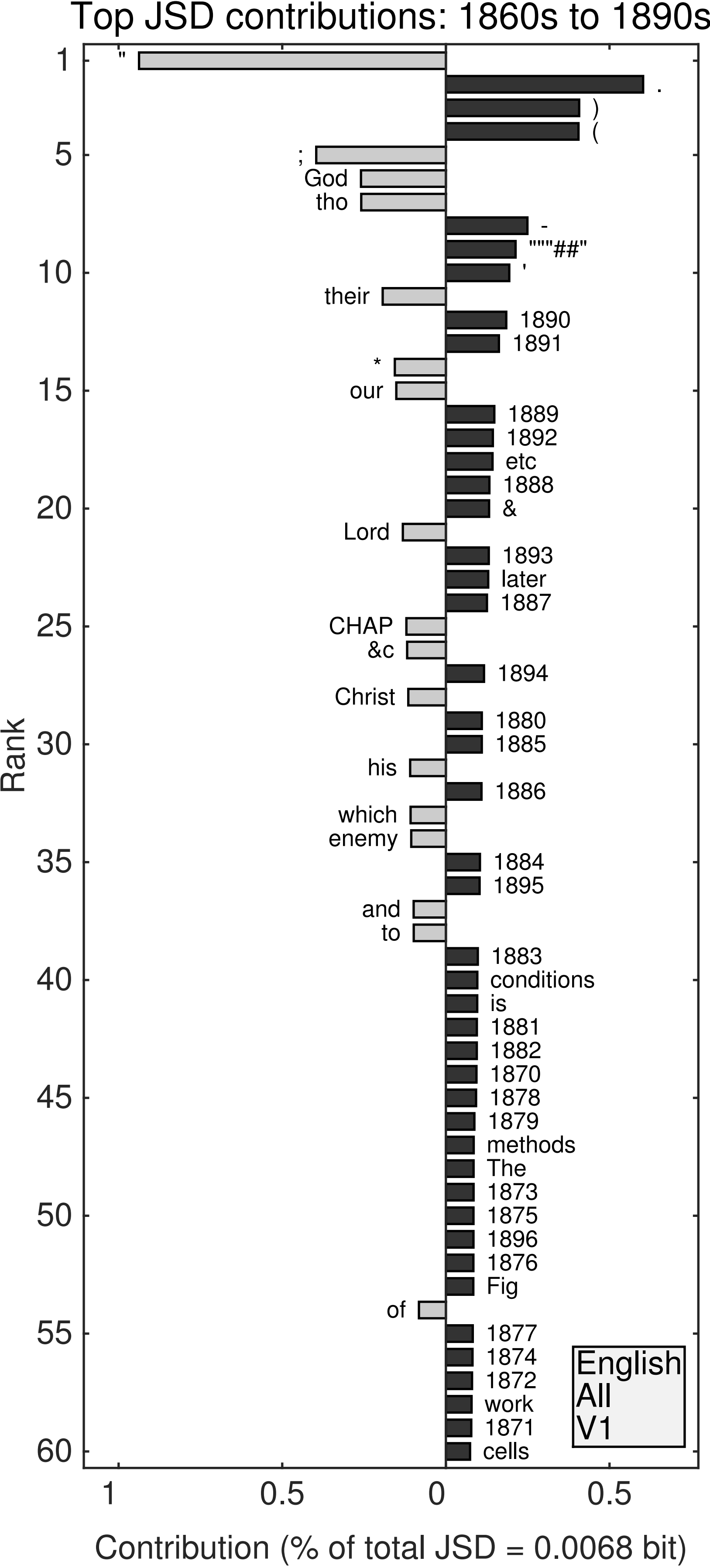
Top JSD contributions: 1860s to 1890s
Rank
Contribution (% of total JSD = 0.0068 bit)
"
.
)
(
;
God
tho
-
"""##"
'
their
1890
1891
*
our
1889
1892
etc
1888
&
Lord
1893
later
1887
CHAP
&c
1894
Christ
1880
1885
his
1886
which
enemy
1884
1895
and
to
1883
conditions
is
1881
1882
1870
1878
1879
methods
The
1873
1875
1896
1876
Fig
of
1877
1874
1872
work
1871
cells
English
All
V1

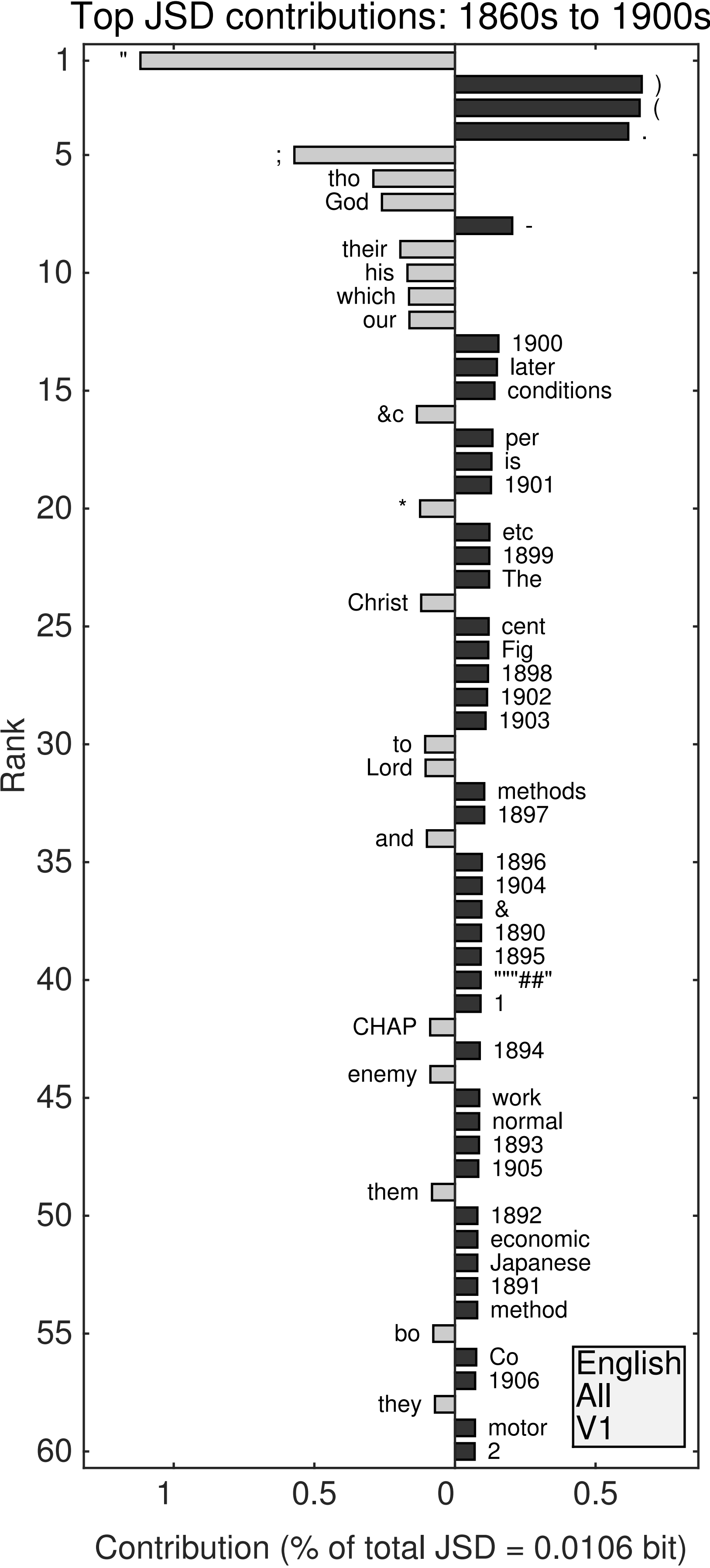
Top JSD contributions: 1860s to 1900s
Rank
Contribution (% of total JSD = 0.0106 bit)
"
)
(
.
;
tho
God
-
their
his
which
our
1900
later
conditions
&c
per
is
1901
*
etc
1899
The
Christ
cent
Fig
1898
1902
1903
to
Lord
methods
1897
and
1896
1904
&
1890
1895
"""##"
1
CHAP
1894
enemy
work
normal
1893
1905
them
1892
economic
Japanese
1891
method
bo
Co
1906
they
motor
2
English
All
V1

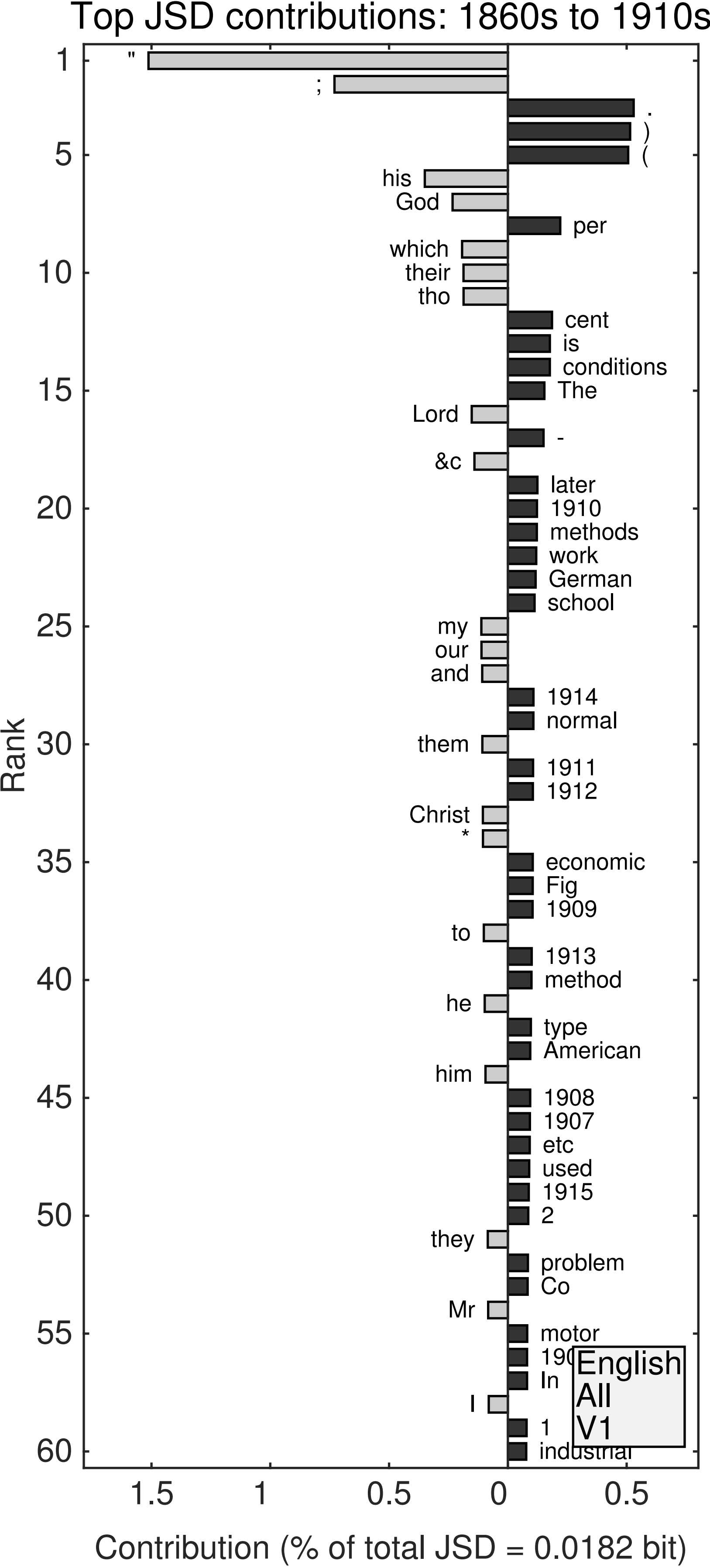
Top JSD contributions: 1860s to 1910s
Rank
Contribution (% of total JSD = 0.0182 bit)
1.5
1
0.5
0
0.5
1
5
10
15
20
25
30
35
40
45
50
55
60
"
;
.
)
(
his
God
per
which
their
tho
cent
is
conditions
The
Lord
-
&c
later
1910
methods
work
German
school
my
our
and
1914
normal
them
1911
1912
Christ
*
economic
Fig
1909
to
1913
method
he
type
American
him
1908
1907
etc
used
1915
2
they
problem
Co
Mr
motor
In
I
1
English
All
V1

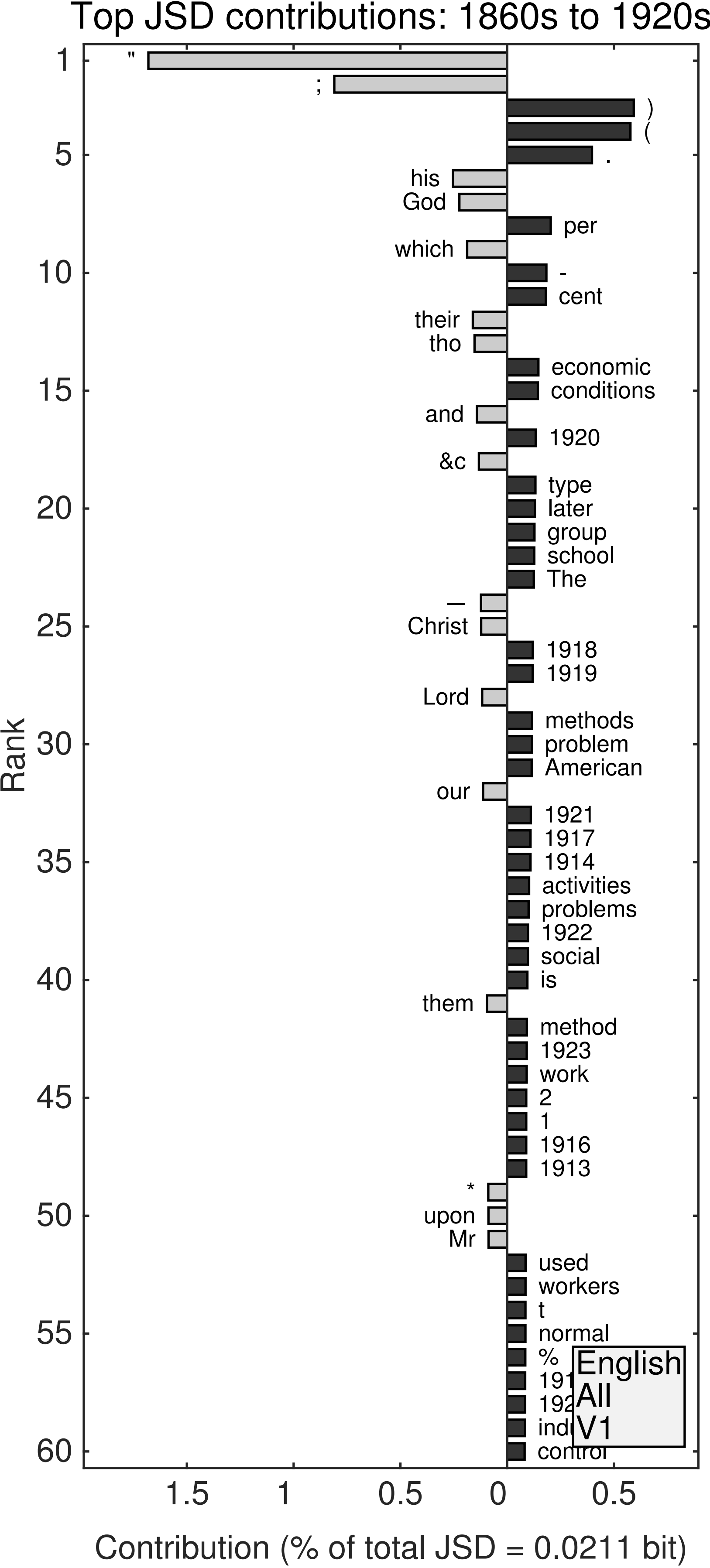
Top JSD contributions: 1860s to 1920s
Rank
Contribution (% of total JSD = 0.0211 bit)
1.5
1
0.5
0
0.5
1
5
10
15
20
25
30
35
40
45
50
55
60
"
;
)
(
.
his
God
per
which
-
cent
their
tho
economic
conditions
and
1920
&c
type
later
group
school
The
—
Christ
1918
1919
Lord
methods
problem
American
our
1921
1917
1914
activities
problems
1922
social
is
them
method
1923
work
2
1
1916
1913
*
upon
Mr
used
workers
t
normal
%
English
All
V1

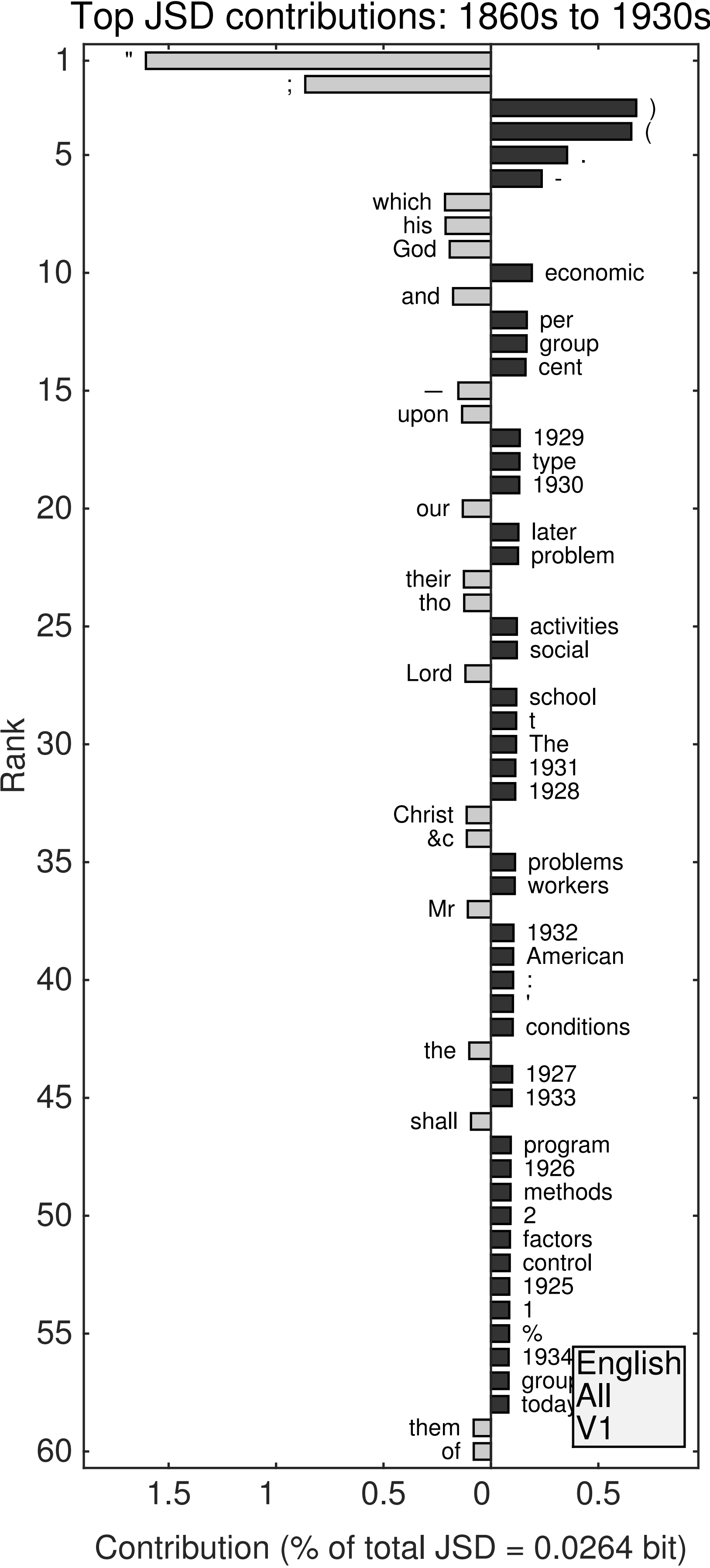

Top JSD contributions: 1860s to 1930s
Rank
Contribution (% of total JSD = 0.0264 bit)
"
;
)
(
.
-
which
his
God
economic
and
per
group
cent
—
upon
1929
type
1930
our
later
problem
their
tho
activities
social
Lord
school
t
The
1931
1928
Christ
&c
problems
workers
Mr
1932
American
:
'
conditions
the
1927
1933
shall
program
1926
methods
2
factors
control
1925
1
%
1934
group
today
them
of
English
All
V1

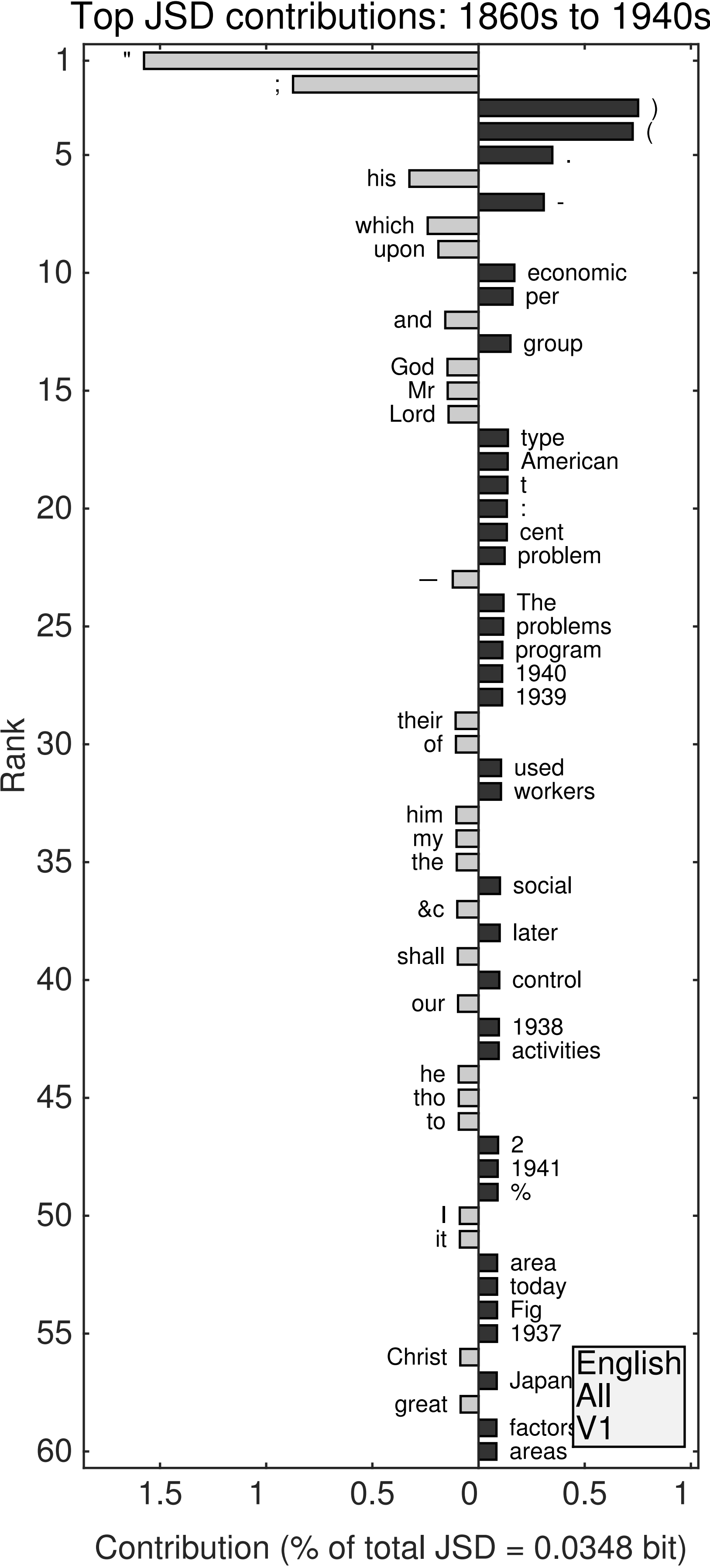

Top JSD contributions: 1860s to 1940s
Rank
Contribution (% of total JSD = 0.0348 bit)
"
;
)
(
.
his
-
which
upon
economic
per
and
group
God
Mr
Lord
type
American
t
:
cent
problem
—
The
problems
program
1940
1939
their
of
used
workers
him
my
the
social
&c
later
shall
control
our
1938
activities
he
tho
to
2
1941
%
I
it
area
today
Fig
1937
Christ
Japan
great
factors
areas
English
All
V1

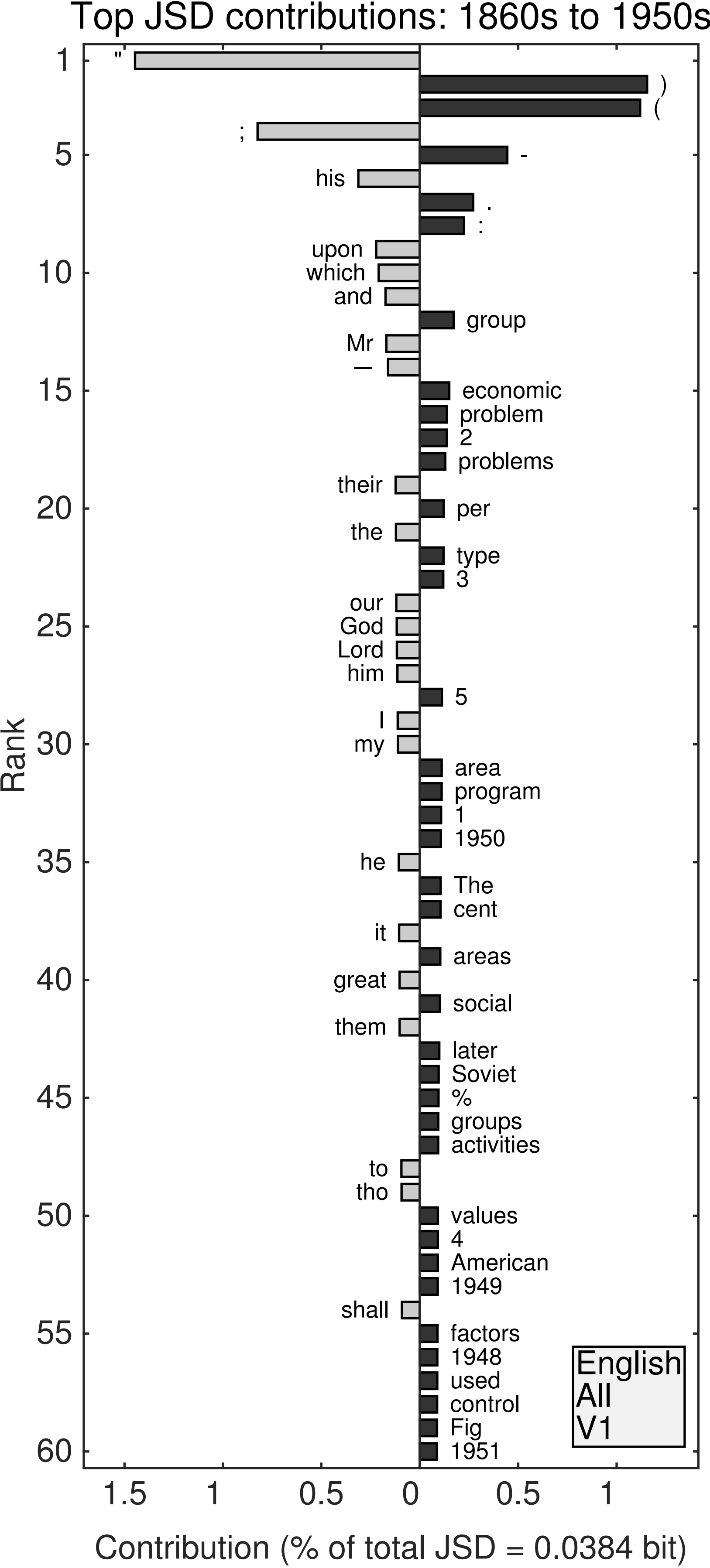

Top JSD contributions: 1860s to 1950s
Rank
Contribution (% of total JSD = 0.0384 bit)
"
)
(
;
-
his
.
:
upon
which
and
group
Mr
—
economic
problem
2
problems
their
per
the
type
3
our
God
Lord
him
5
I
my
area
program
1
1950
he
The
cent
it
areas
great
social
them
later
Soviet
%
groups
activities
to
tho
values
4
American
1949
shall
factors
1948
used
control
Fig
1951
English
All
V1

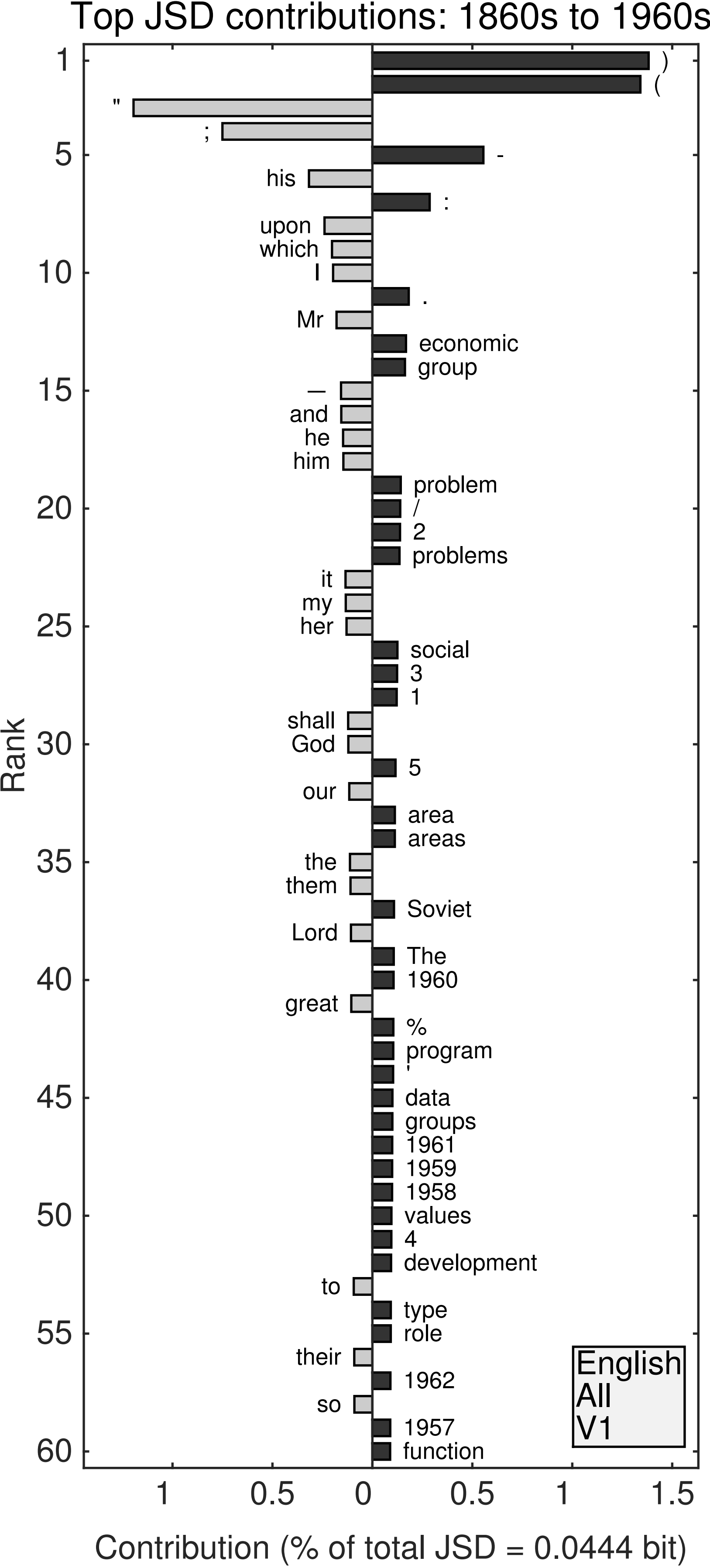
Top JSD contributions: 1860s to 1960s
Rank
Contribution (% of total JSD = 0.0444 bit)
)
(
"
;
-
his
:
upon
which
I
.
Mr
economic
group
—
and
he
him
problem
/
2
problems
it
my
her
social
3
1
shall
God
5
our
area
areas
the
them
Soviet
Lord
The
1960
great
%
program
'
data
groups
1961
1959
1958
values
4
development
to
type
role
their
1962
so
1957
function
English
All
V1

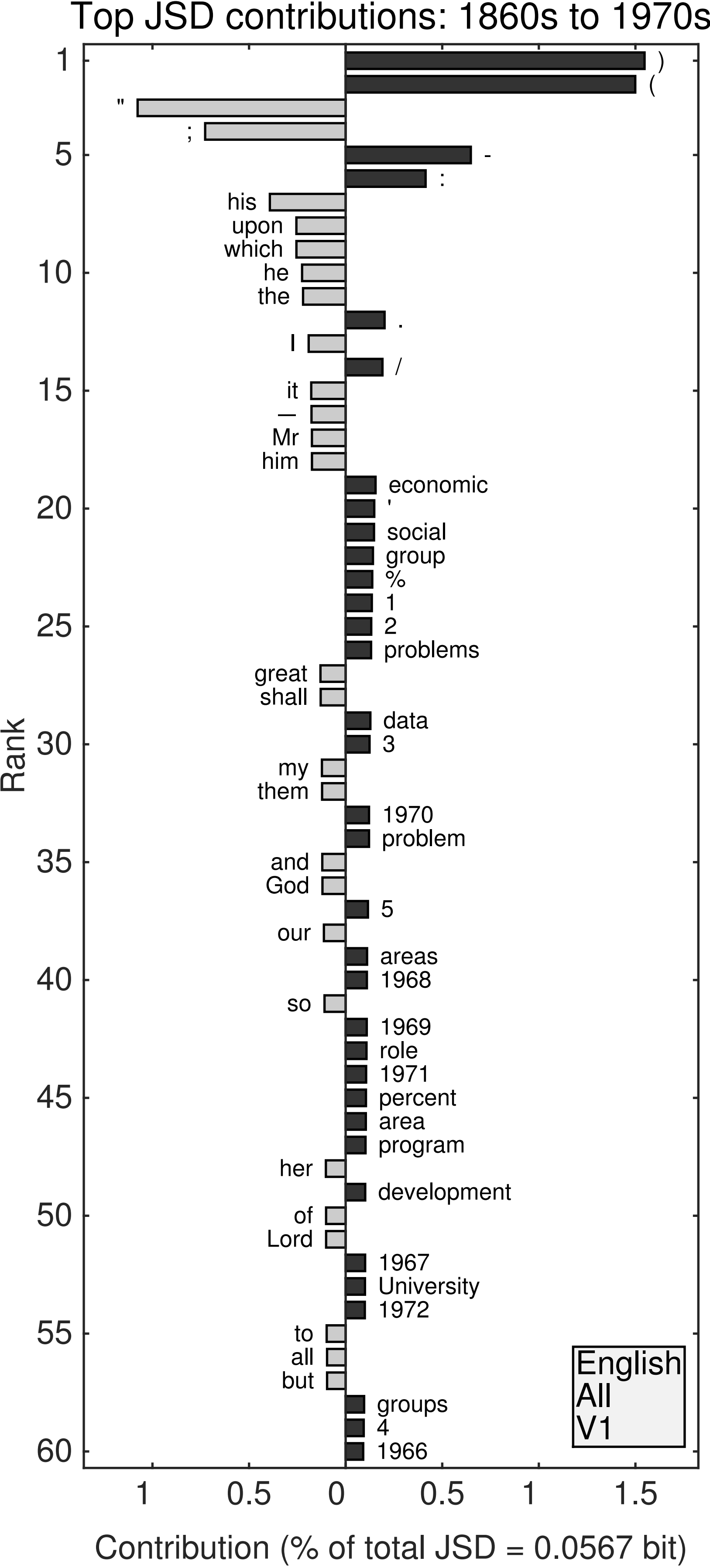

Top JSD contributions: 1860s to 1970s
Rank
)
(
"
;
-
:
his
upon
which
he
the
.
I
/
it
—
Mr
him
economic
'
social
group
%
1
2
problems
great
shall
data
3
my
them
1970
problem
and
God
5
our
areas
1968
so
1969
role
1971
percent
area
program
her
development
of
Lord
1967
University
1972
to
all
but
groups
4
1966
English
All
V1
Contribution (% of total JSD = 0.0567 bit)

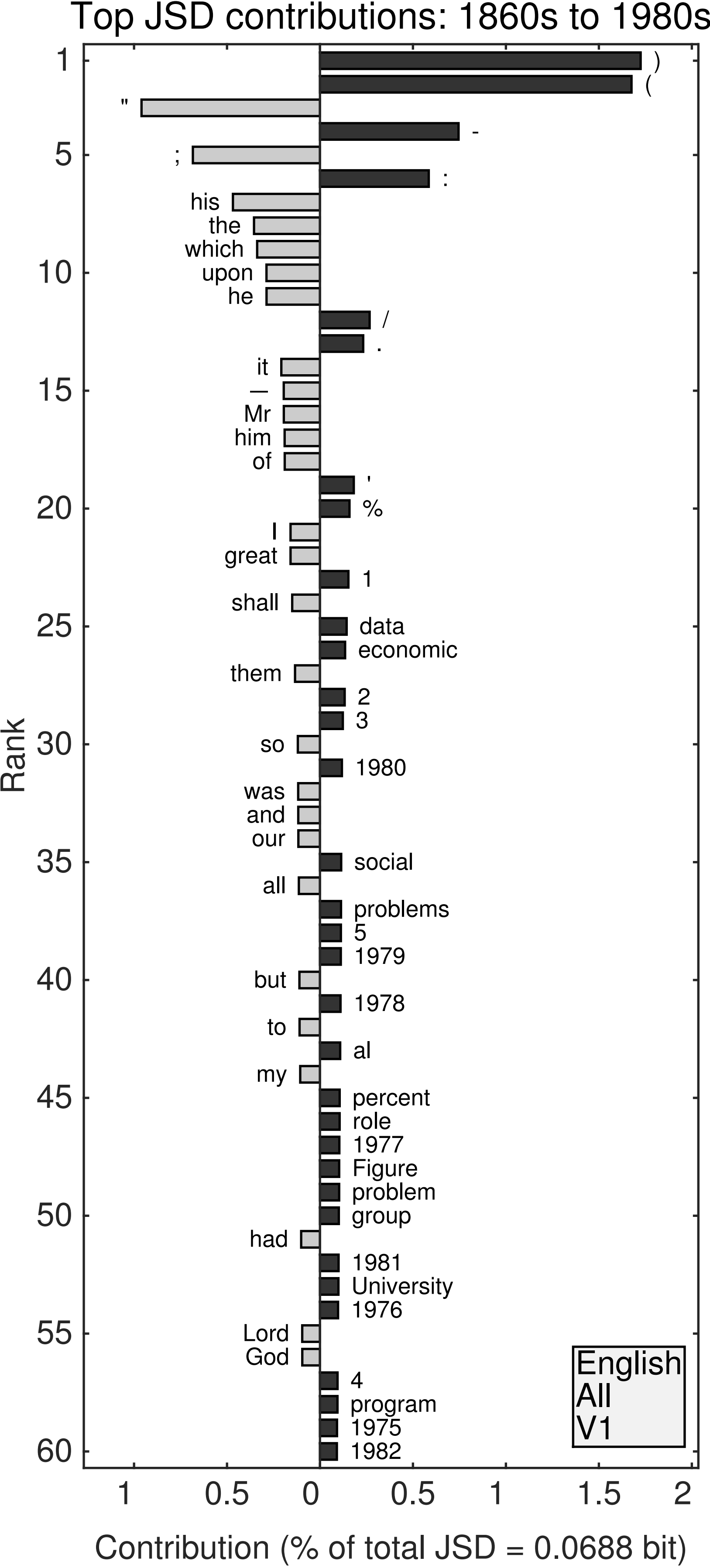
Top JSD contributions: 1860s to 1980s
Rank
Contribution (% of total JSD = 0.0688 bit)
)
(
"
-
;
:
his
the
which
upon
he
/
.
it
—
Mr
him
of
'
%
I
great
1
shall
data
economic
them
2
3
so
1980
was
and
our
social
all
problems
5
1979
but
1978
to
al
my
percent
role
1977
Figure
problem
group
had
1981
University
1976
Lord
God
4
program
1975
1982
English
All
V1

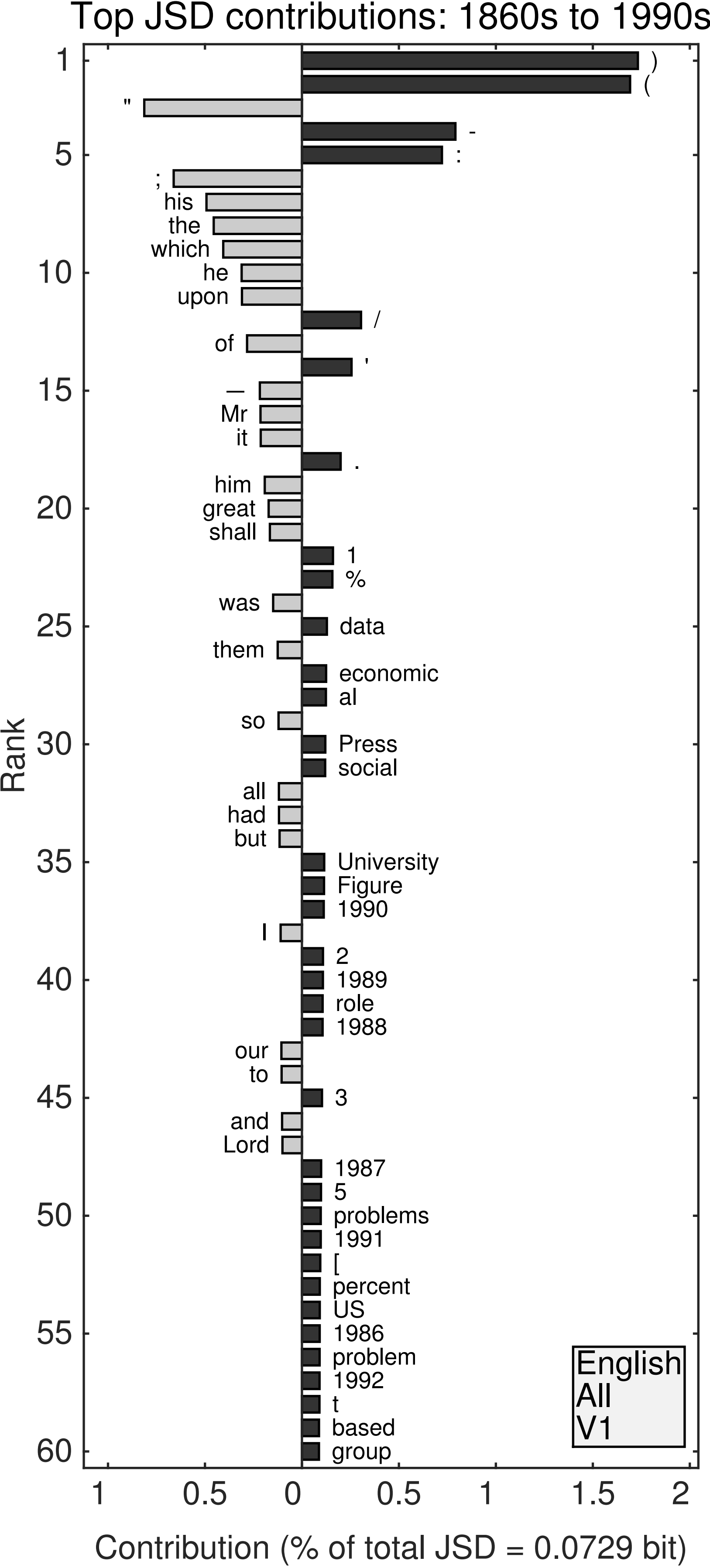
Top JSD contributions: 1860s to 1990s
Rank
Contribution (% of total JSD = 0.0729 bit)
)
(
"
-
:
;
his
the
which
he
upon
/
of
'
—
Mr
it
.
him
great
shall
1
%
was
data
them
economic
al
so
Press
social
all
had
but
University
Figure
1990
I
2
1989
role
1988
our
to
3
and
Lord
1987
5
problems
1991
[
percent
US
1986
problem
1992
t
based
group
English
All
V1

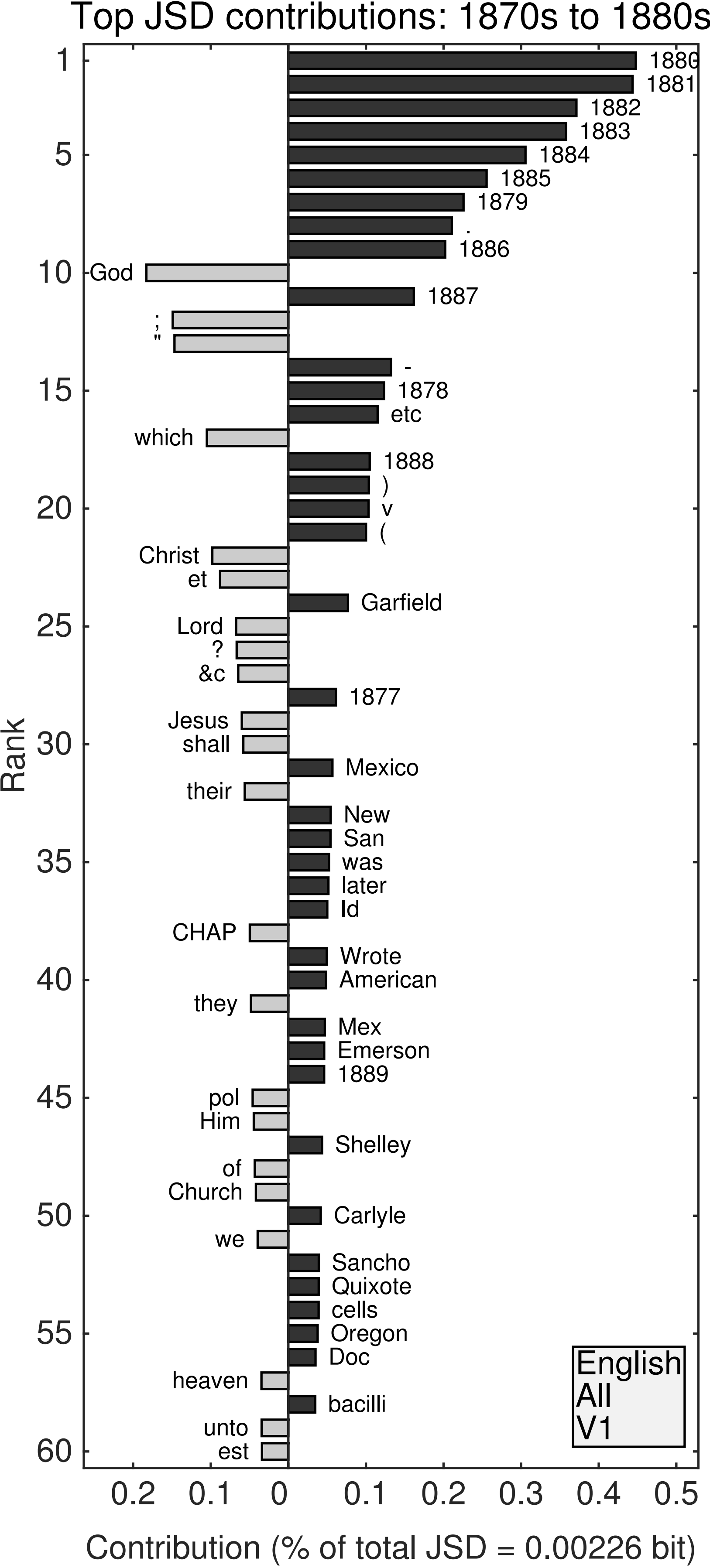

Top JSD contributions: 1870s to 1880s
Rank
1880
1881
1882
1883
1884
1885
1879
.
1886
God
1887
;
"
-
1878
etc
which
1888
)
v
(
Christ
et
Garfield
Lord
?
&c
1877
Jesus
shall
Mexico
their
New
San
was
later
Id
CHAP
Wrote
American
they
Mex
Emerson
1889
pol
Him
Shelley
of
Church
Carlyle
we
Sancho
Quixote
cells
Oregon
Doc
heaven
bacilli
unto
est
English
All
V1
Contribution (% of total JSD = 0.00226 bit)

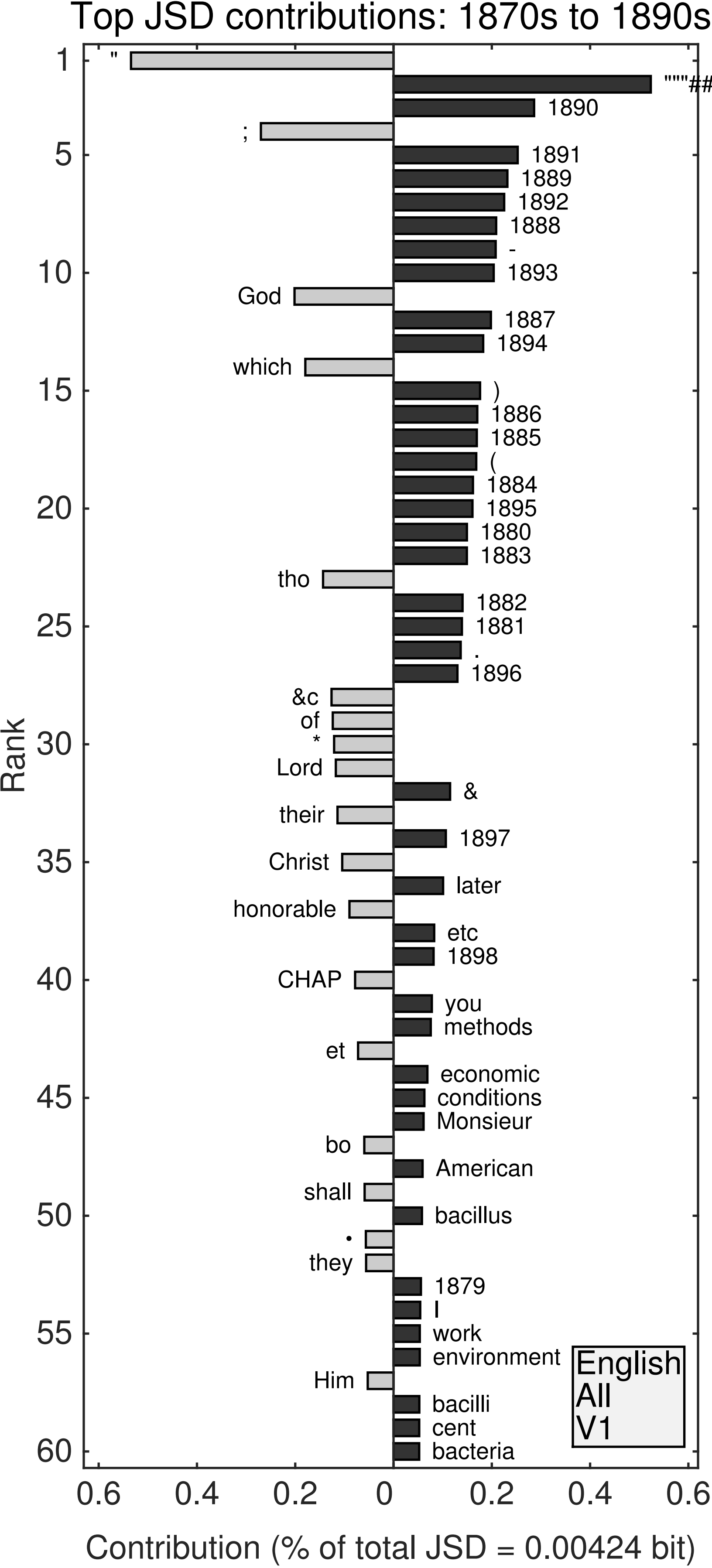
Top JSD contributions: 1870s to 1890s
"
"""##
1890
;
1891
1889
1892
1888
-
1893
God
1887
1894
which
)
1886
1885
(
1884
1895
1880
1883
tho
1882
1881
.
1896
&c
of
*
Lord
&
their
1897
Christ
later
honorable
etc
1898
CHAP
you
methods
et
economic
conditions
Monsieur
bo
American
shall
bacillus
•
they
1879
I
work
environment
Him
bacilli
cent
bacteria
English
All
V1
Rank
Contribution (% of total JSD = 0.00424 bit)

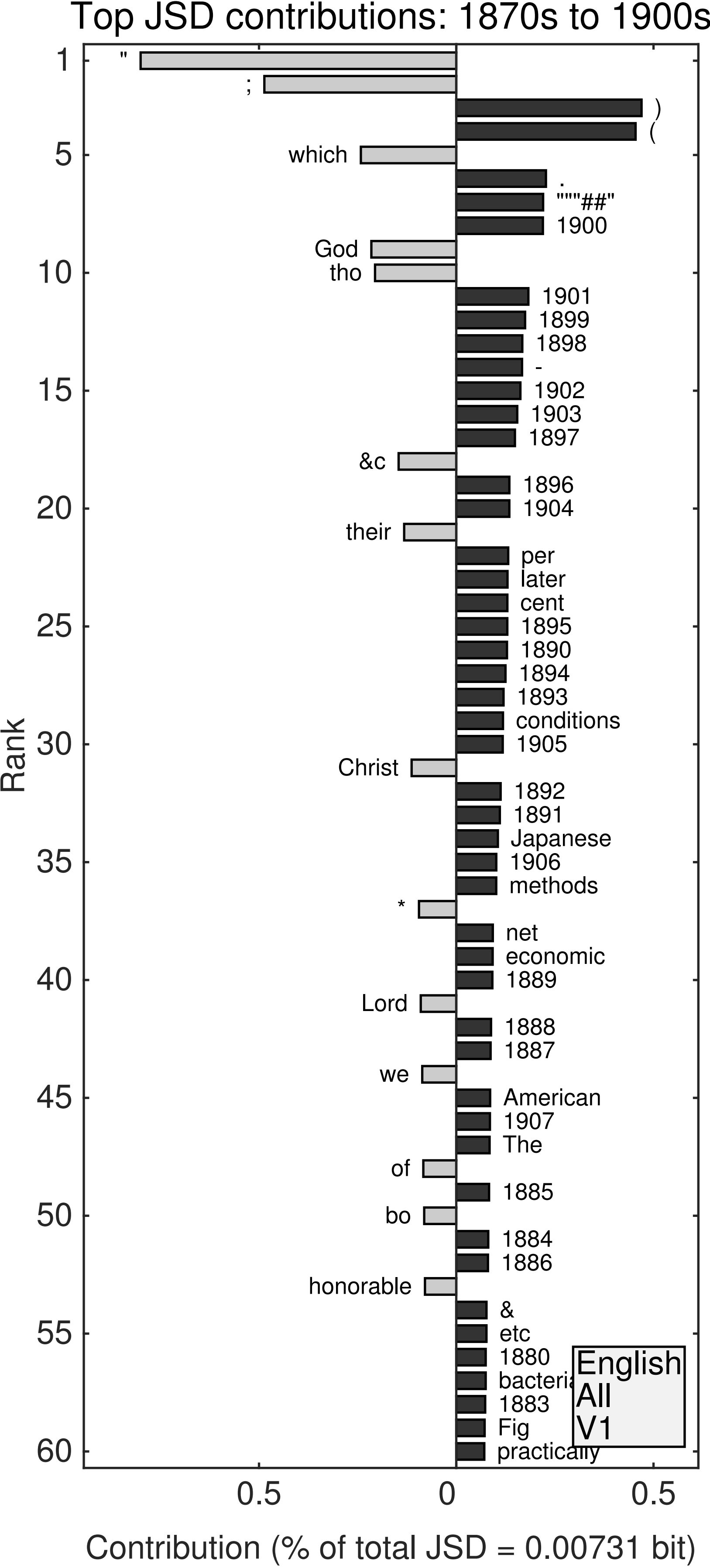

Top JSD contributions: 1870s to 1900s
Rank
Contribution (% of total JSD = 0.00731 bit)
"
;
)
(
which
.
"""##"
1900
God
tho
1901
1899
1898
-
1902
1903
1897
&c
1896
1904
their
per
later
cent
1895
1890
1894
1893
conditions
1905
Christ
1892
1891
Japanese
1906
methods
*
net
economic
1889
Lord
1888
1887
we
American
1907
The
of
1885
bo
1884
1886
honorable
&
etc
1880
bacteria
1883
Fig
practically
English
All
V1

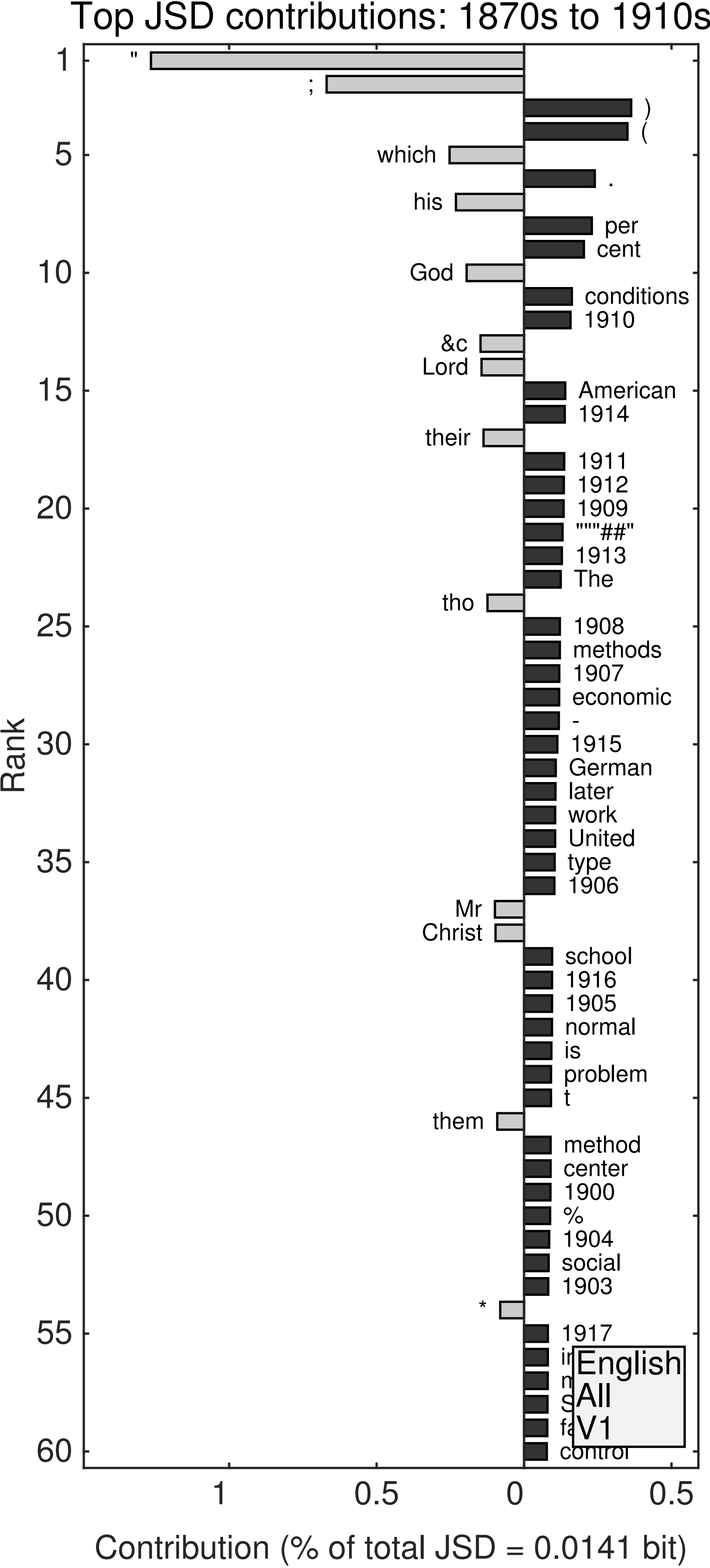
Top JSD contributions: 1870s to 1910s
Rank
"
;
)
(
which
.
his
per
cent
God
conditions
1910
&c
Lord
American
1914
their
1911
1912
1909
"""##"
1913
The
tho
1908
methods
1907
economic
-
1915
German
later
work
United
type
1906
Mr
Christ
school
1916
1905
normal
is
problem
t
them
method
center
1900
%
1904
social
1903
*
1917
control
English
All
V1
1 5 10 15 20 25 30 35 40 45 50 55 60
1 0.5 0 0.5
Contribution (% of total JSD = 0.0141 bit)

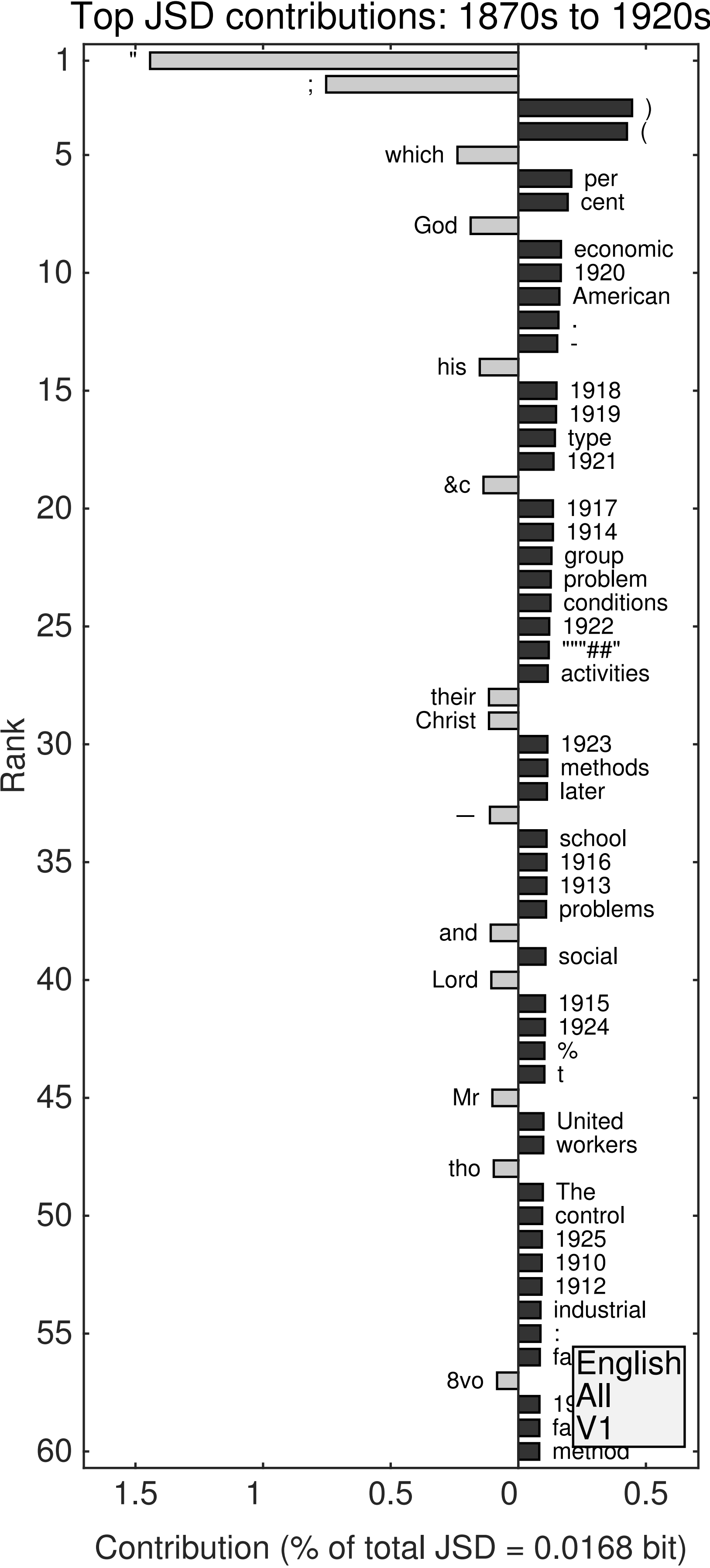

Top JSD contributions: 1870s to 1920s
Rank
Contribution (% of total JSD = 0.0168 bit)
1.5
1
0.5
0
0.5
"
;
)
(
which
per
cent
God
economic
1920
American
.
-
his
1918
1919
type
1921
&c
1917
1914
group
problem
conditions
1922
"""##"
activities
their
Christ
1923
methods
later
—
school
1916
1913
problems
and
social
Lord
1915
1924
%
t
Mr
United
workers
tho
The
control
1925
1910
1912
industrial
:
8vo
method
English
All
V1

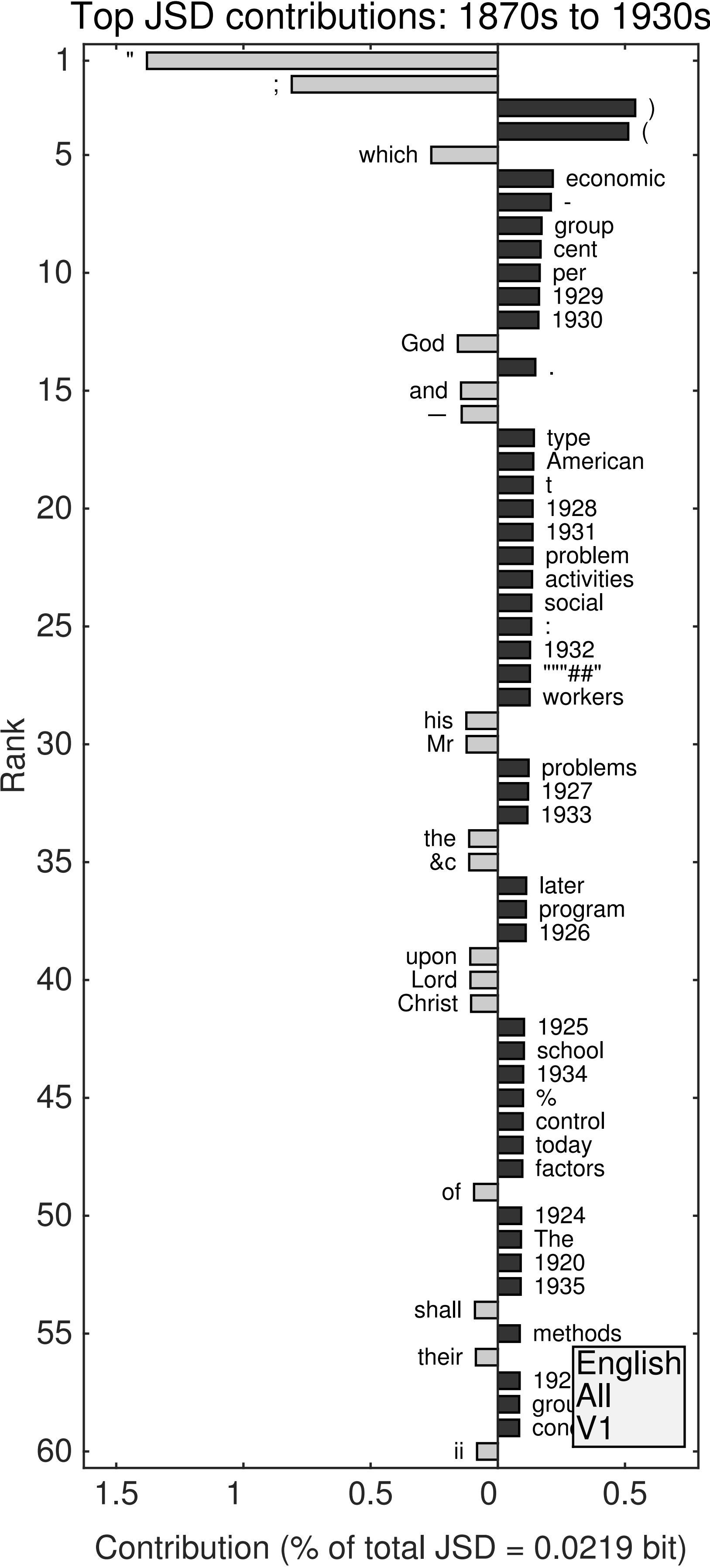

Top JSD contributions: 1870s to 1930s
Rank
Contribution (% of total JSD = 0.0219 bit)
"
;
)
(
which
economic
-
group
cent
per
1929
1930
God
.
and
—
type
American
t
1928
1931
problem
activities
social
:
1932
"""##"
workers
his
Mr
problems
1927
1933
the
&c
later
program
1926
upon
Lord
Christ
1925
school
1934
%
control
today
factors
of
1924
The
1920
1935
shall
methods
their
ii
English
All
V1

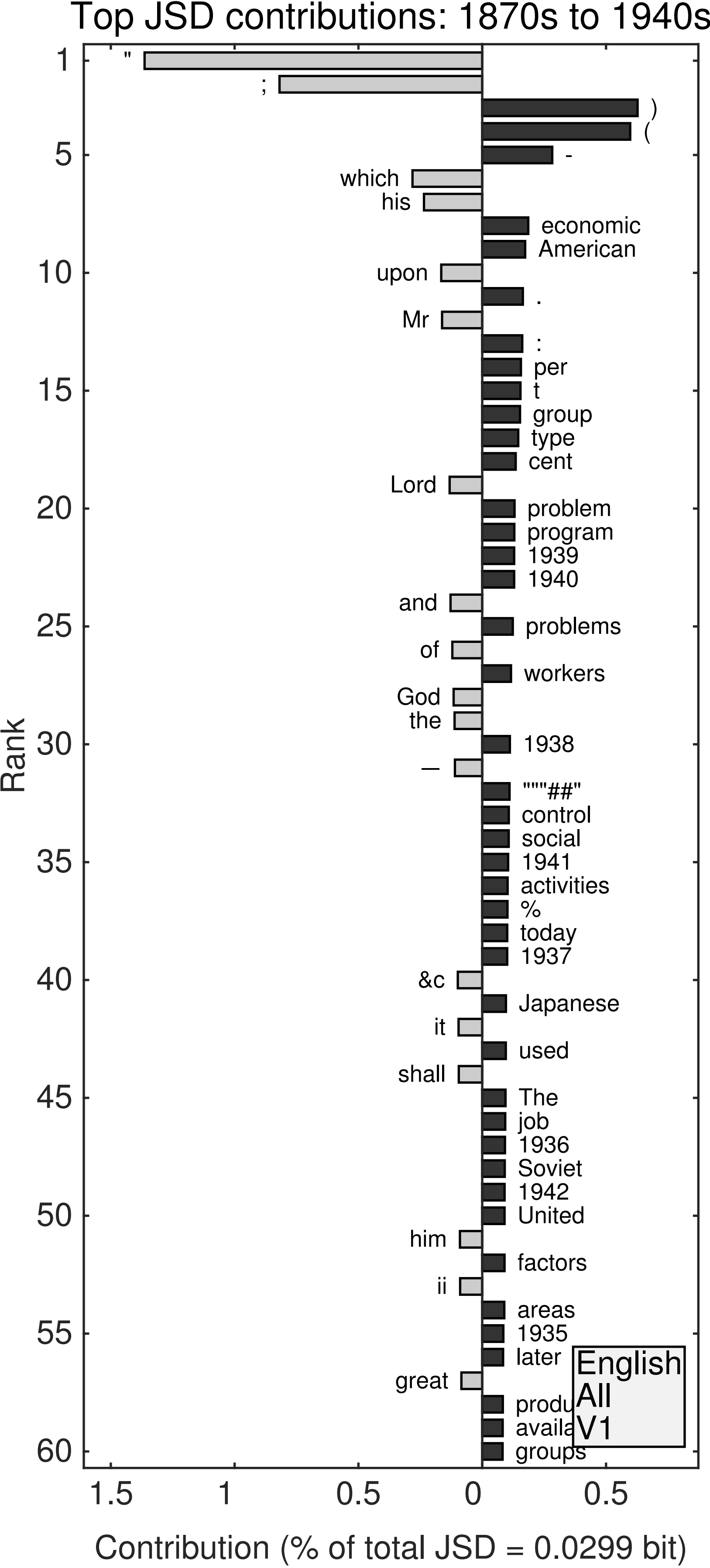

Top JSD contributions: 1870s to 1940s
Rank
Contribution (% of total JSD = 0.0299 bit)
1.5
1
0.5
0
0.5
1
5
10
15
20
25
30
35
40
45
50
55
60
"
;
)
(
-
which
his
economic
American
upon
.
Mr
:
per
t
group
type
cent
Lord
problem
program
1939
1940
and
problems
of
workers
God
the
1938
—
"""##"
control
social
1941
activities
%
today
1937
&c
Japanese
it
used
shall
The
job
1936
Soviet
1942
United
him
factors
ii
areas
1935
later
great
groups
English
All
V1

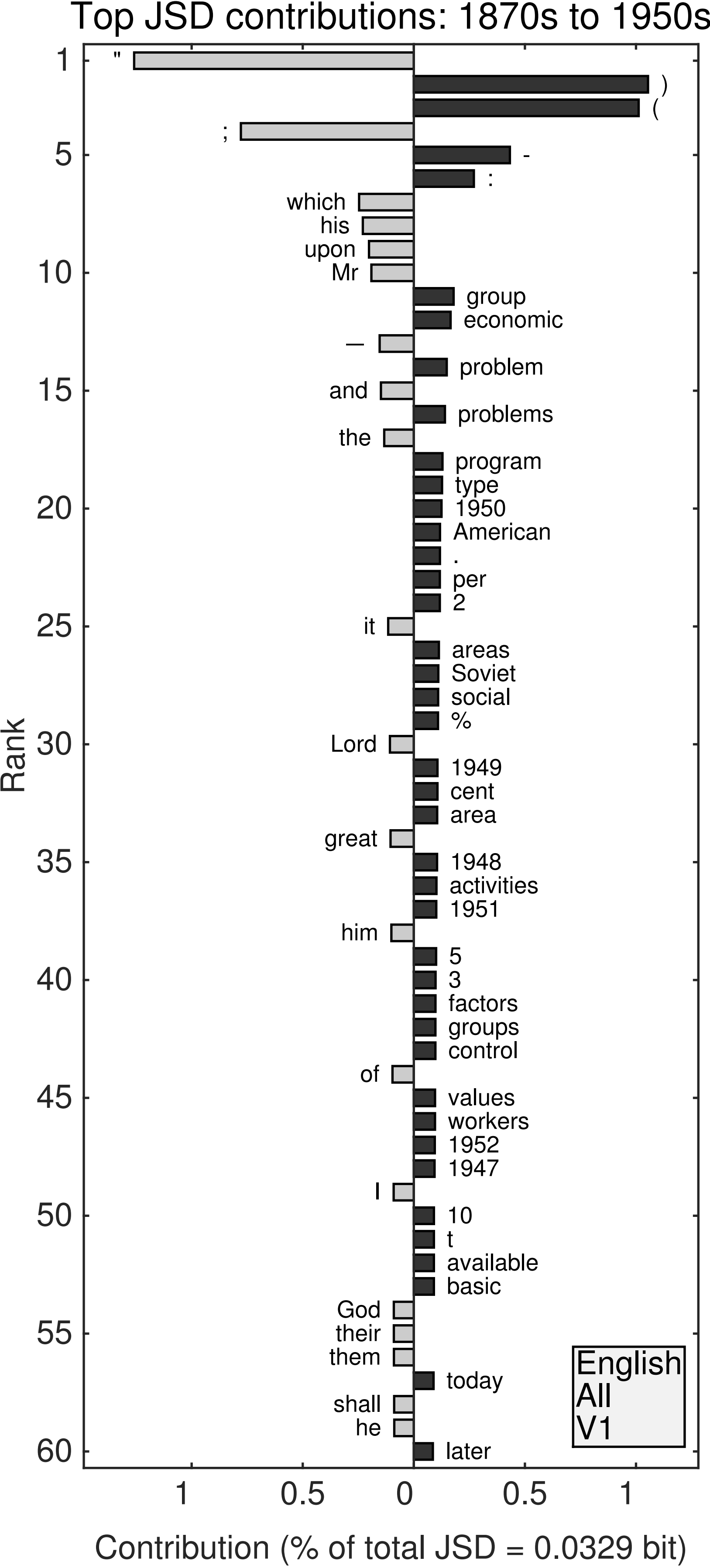
Top JSD contributions: 1870s to 1950s
Rank
Contribution (% of total JSD = 0.0329 bit)
"
)
(
;
-
:
which
his
upon
Mr
group
economic
—
problem
and
problems
the
program
type
1950
American
.
per
2
it
areas
Soviet
social
%
Lord
1949
cent
area
great
1948
activities
1951
him
5
3
factors
groups
control
of
values
workers
1952
1947
I
10
t
available
basic
God
their
them
today
shall
he
later
English
All
V1

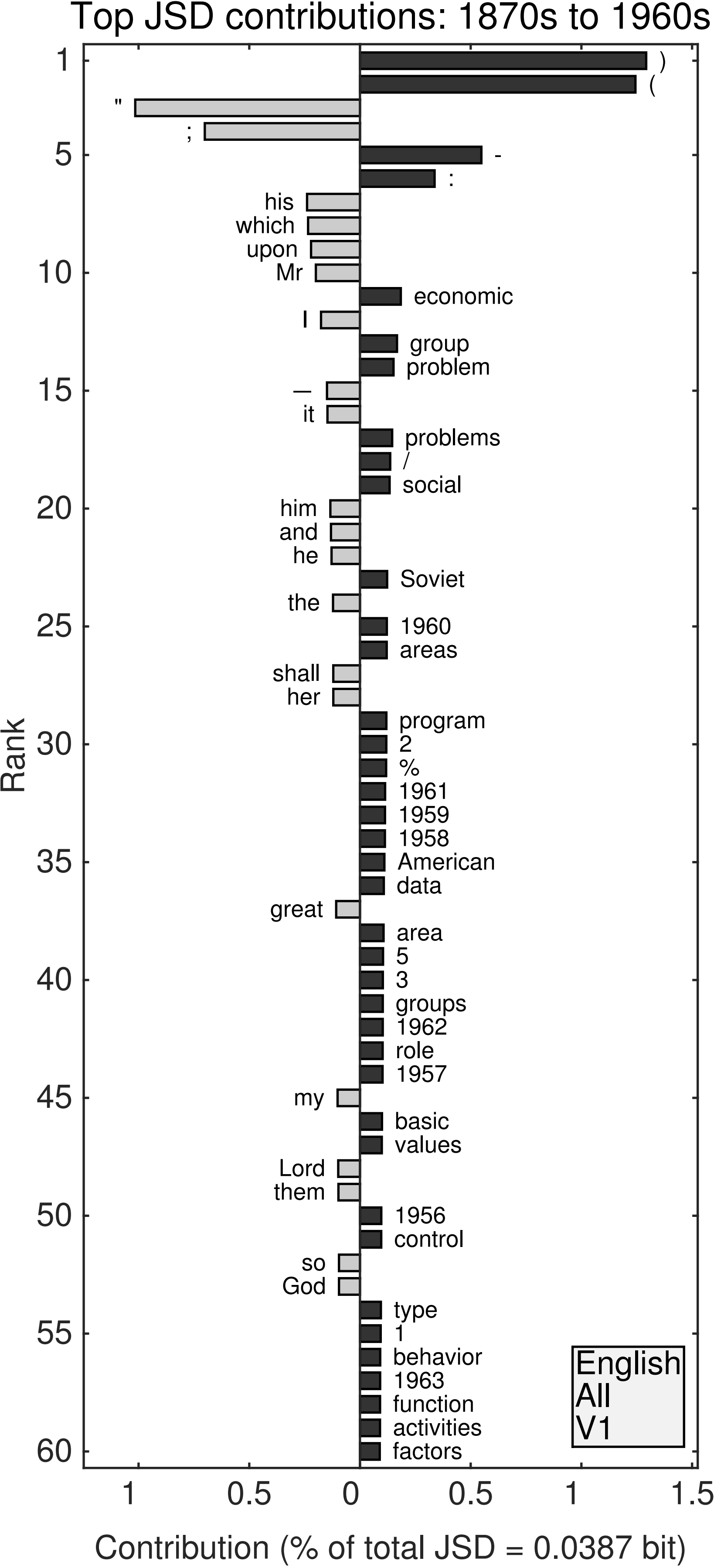

Top JSD contributions: 1870s to 1960s
Rank
Contribution (% of total JSD = 0.0387 bit)
1 0.5 0 0.5 1 1.5
1 5 10 15 20 25 30 35 40 45 50 55 60
)
(
"
;
-
:
his
which
upon
Mr
economic
I
group
problem
—
it
problems
/
social
him
and
he
Soviet
the
1960
areas
shall
her
program
2
%
1961
1959
1958
American
data
great
area
5
3
groups
1962
role
1957
my
basic
values
Lord
them
1956
control
so
God
type
1
behavior
1963
function
activities
factors
English
All
V1

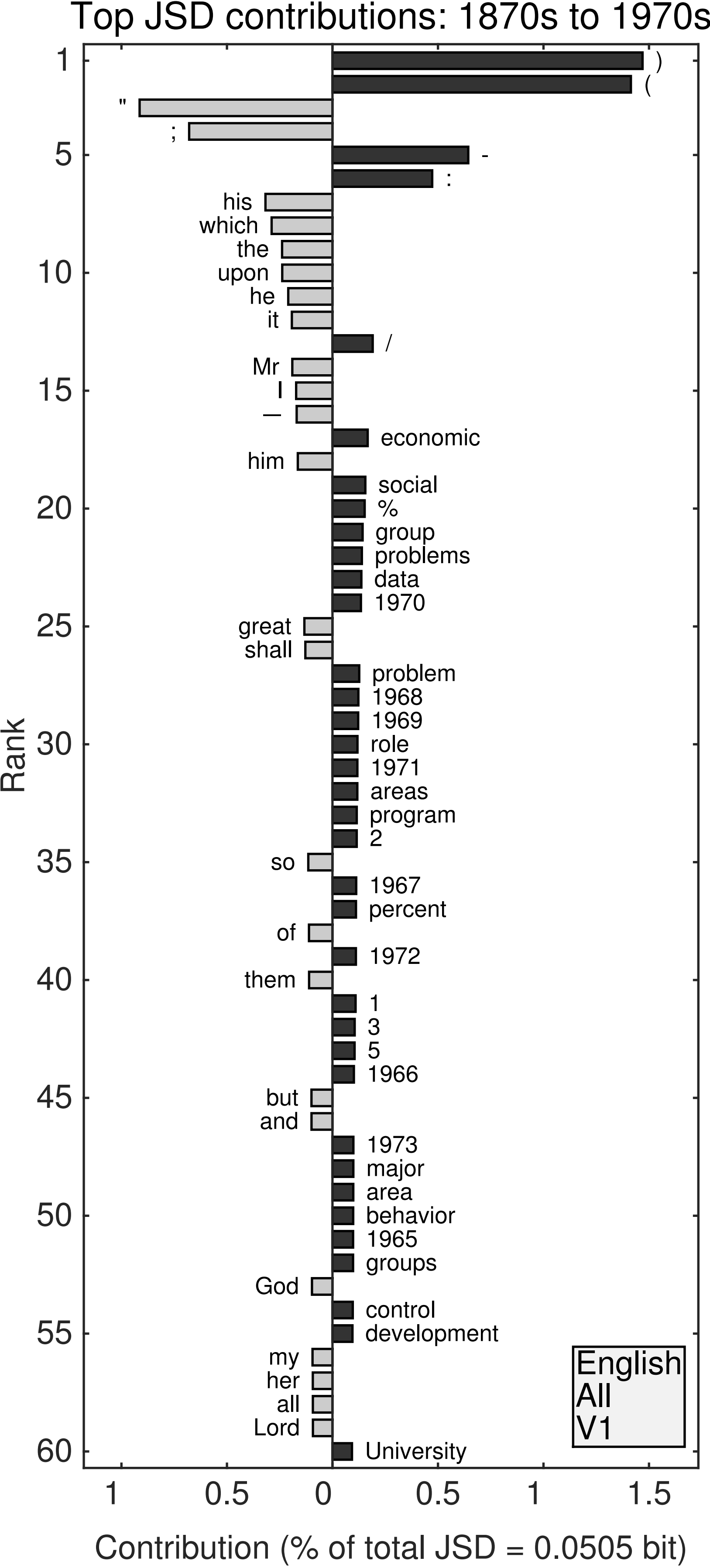

Top JSD contributions: 1870s to 1970s
Rank
1
5
10
15
20
25
30
35
40
45
50
55
60
)
(
"
;
-
:
his
which
the
upon
he
it
/
Mr
I
—
economic
him
social
%
group
problems
data
1970
great
shall
problem
1968
1969
role
1971
areas
program
2
so
1967
percent
of
1972
them
1
3
5
1966
but
and
1973
major
area
behavior
1965
groups
God
control
development
my
her
all
Lord
University
English
All
V1
1
0.5
0
0.5
1
1.5
Contribution (% of total JSD = 0.0505 bit)

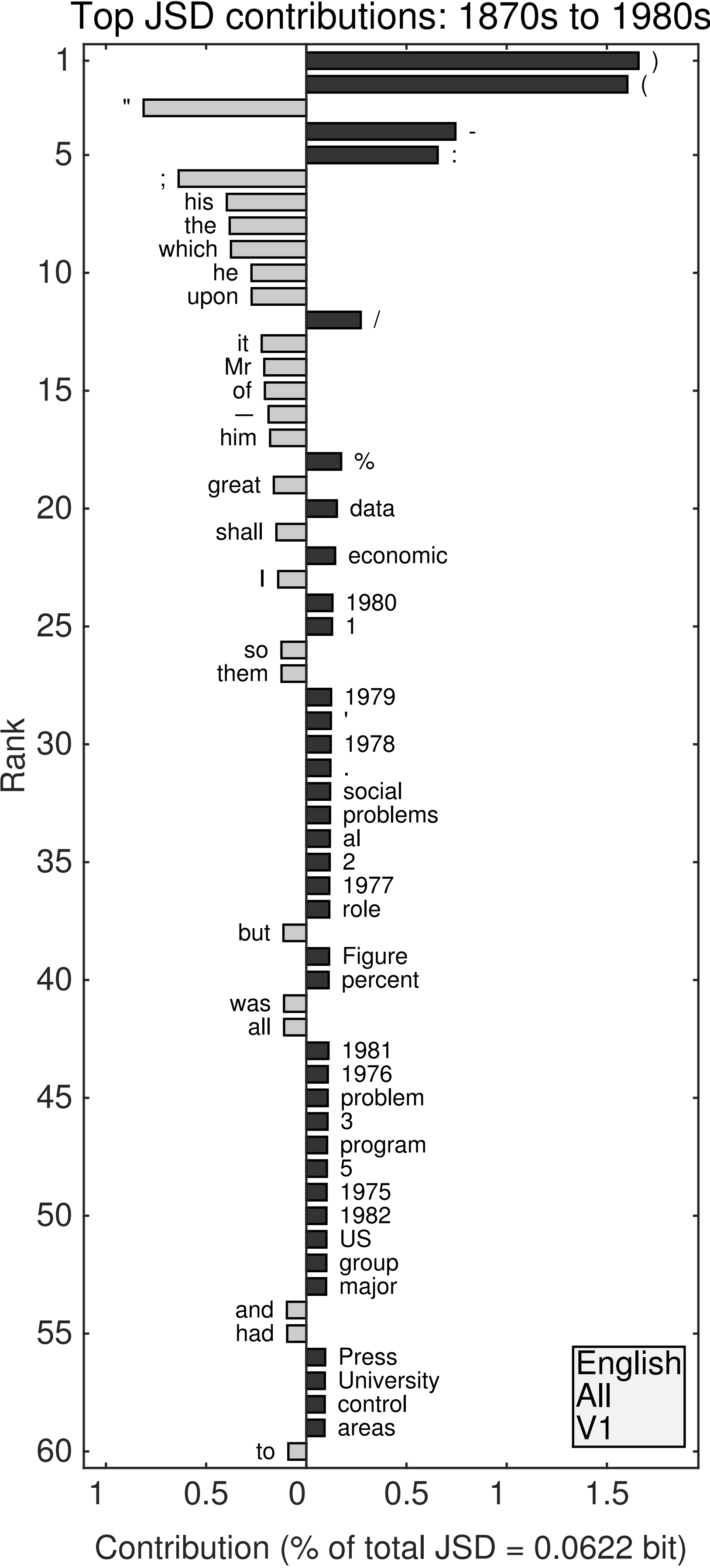

Top JSD contributions: 1870s to 1980s
Rank
Contribution (% of total JSD = 0.0622 bit)
)
(
"
-
:
;
his
the
which
he
upon
/
it
Mr
of
—
him
%
great
data
shall
economic
I
1980
1
so
them
1979
'
1978
.
social
problems
al
2
1977
role
but
Figure
percent
was
all
1981
1976
problem
3
program
5
1975
1982
US
group
major
and
had
Press
University
control
areas
to
English
All
V1

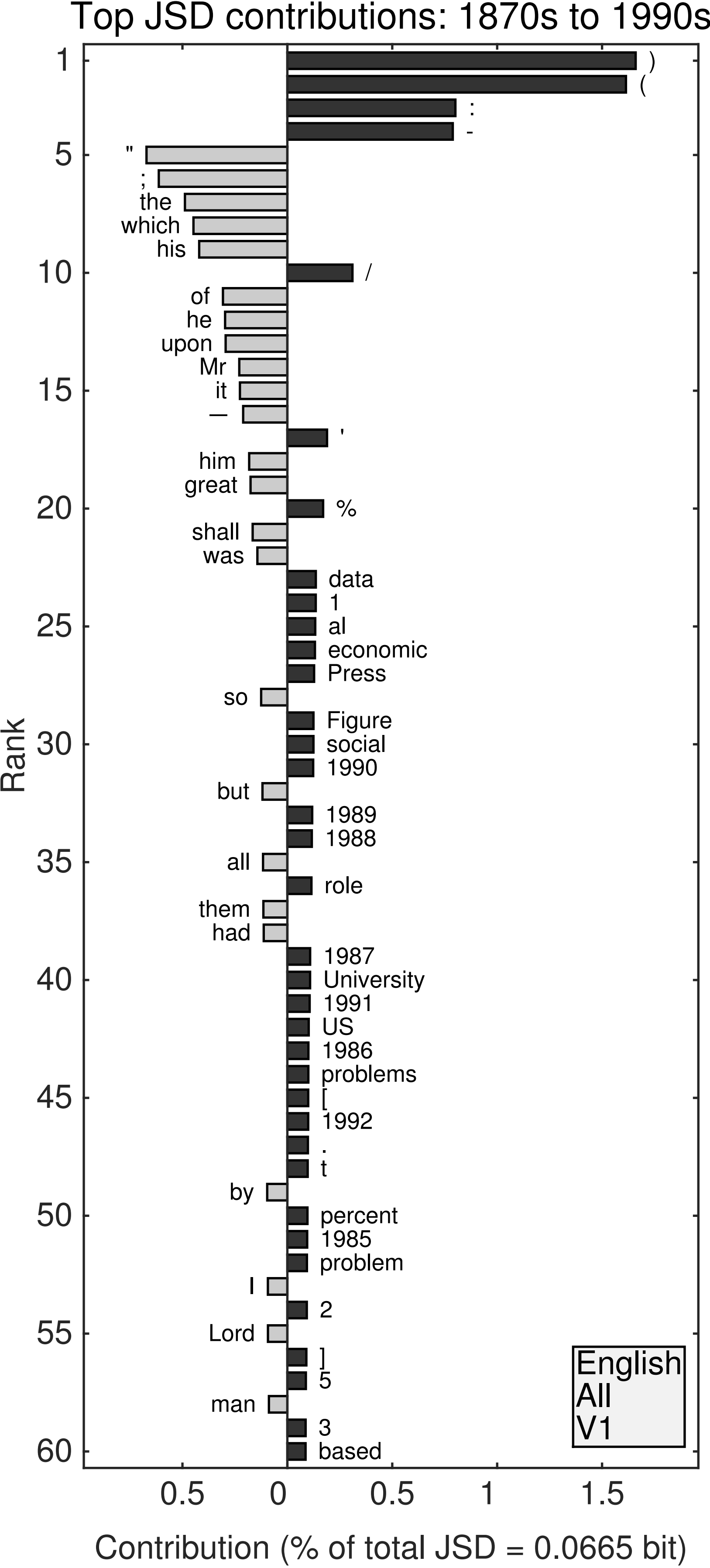

Top JSD contributions: 1870s to 1990s
Rank
Contribution (% of total JSD = 0.0665 bit)
English
All
V1
1
5
10
15
20
25
30
35
40
45
50
55
60
0.5
0
0.5
1
1.5
)
(
:
-
"
;
the
which
his
/
of
he
upon
Mr
it
—
'
him
great
%
shall
was
data
1
al
economic
Press
so
Figure
social
1990
but
1989
1988
all
role
them
had
1987
University
1991
US
1986
problems
[
1992
.
t
by
percent
1985
problem
I
2
Lord
]
5
man
3
based

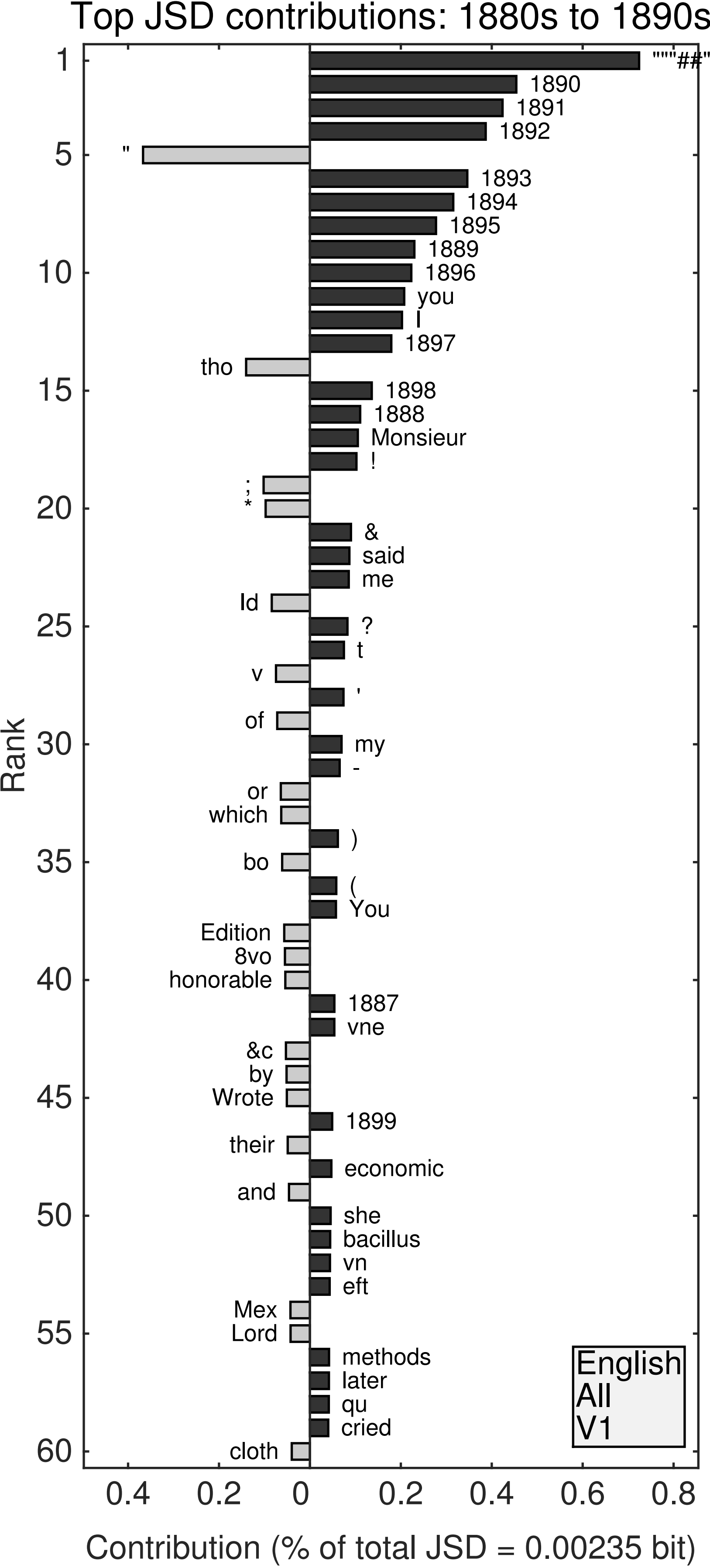
Top JSD contributions: 1880s to 1890s
Rank
Contribution (% of total JSD = 0.00235 bit)
"""##"
1890
1891
1892
"
1893
1894
1895
1889
1896
you
I
1897
tho
1898
1888
Monsieur
!
;
*
&
said
me
Id
?
t
v
'
of
my
-
or
which
)
bo
(
You
Edition
8vo
honorable
1887
vne
&c
by
Wrote
1899
their
economic
and
she
bacillus
vn
eft
Mex
Lord
methods
later
qu
cried
cloth
English
All
V1

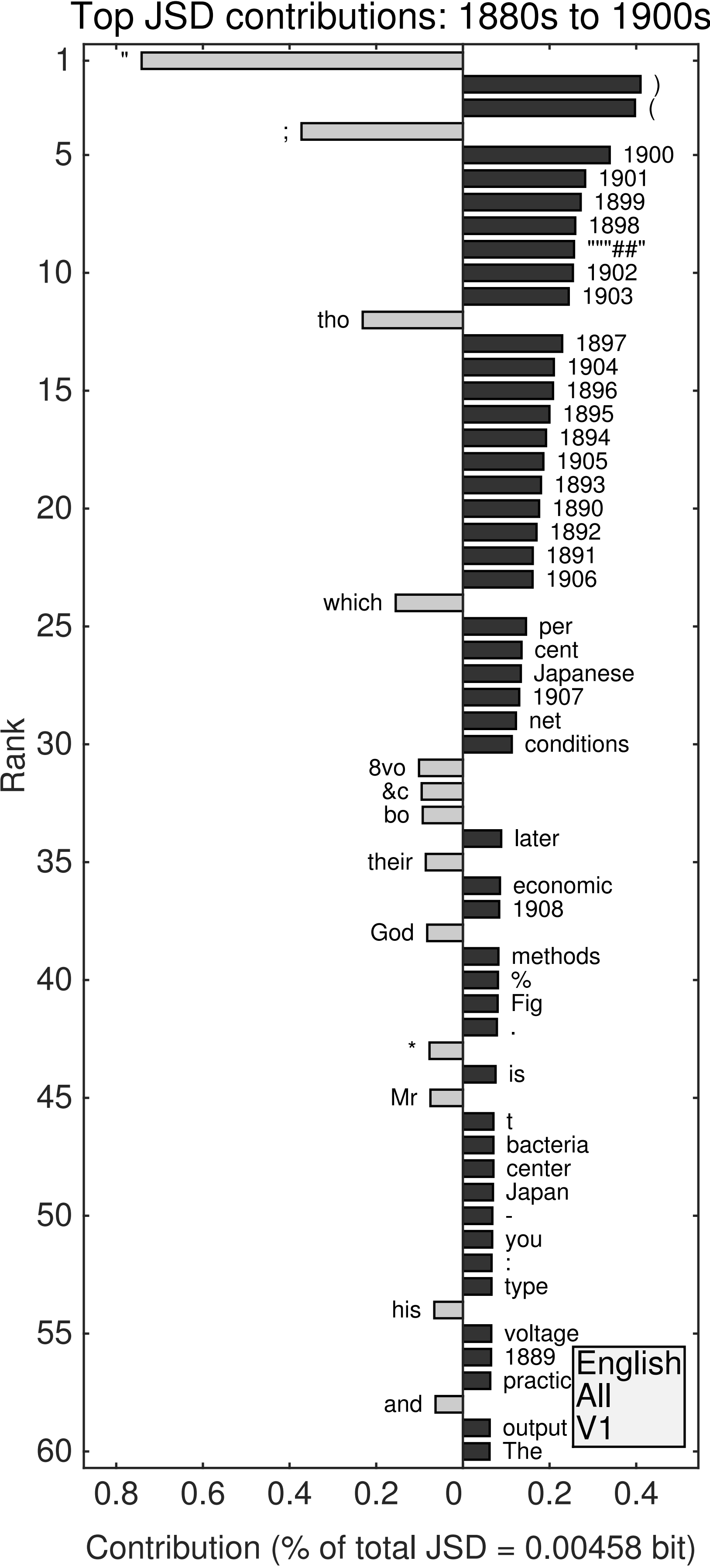

Top JSD contributions: 1880s to 1900s
Rank
Contribution (% of total JSD = 0.00458 bit)
"
)
(
;
1900
1901
1899
1898
"""##"
1902
1903
tho
1897
1904
1896
1895
1894
1905
1893
1890
1892
1891
1906
which
per
cent
Japanese
1907
net
conditions
8vo
&c
bo
later
their
economic
1908
God
methods
%
Fig
.
*
is
Mr
t
bacteria
center
Japan
-
you
:
type
his
voltage
1889
practic
and
output
The
English
All
V1

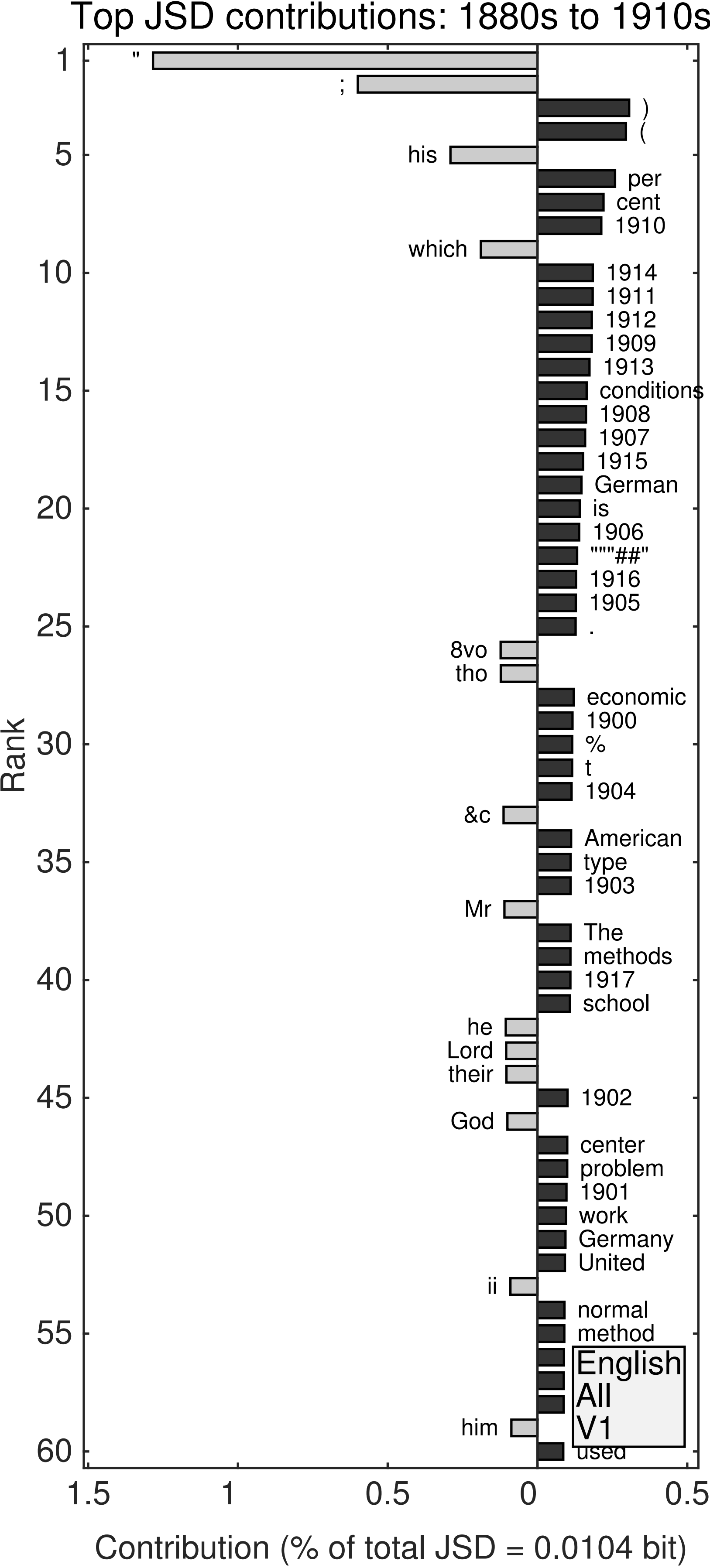
Top JSD contributions: 1880s to 1910s
"
;
)
(
his
per
cent
1910
which
1914
1911
1912
1909
1913
conditions
1908
1907
1915
German
is
1906
"""##"
1916
1905
.
8vo
tho
economic
1900
%
t
1904
&c
American
type
1903
Mr
The
methods
1917
school
he
Lord
their
1902
God
center
problem
1901
work
Germany
United
ii
normal
method
him
used
English
All
V1
Rank
1
5
10
15
20
25
30
35
40
45
50
55
60
1.5
1
0.5
0
0.5
Contribution (% of total JSD = 0.0104 bit)

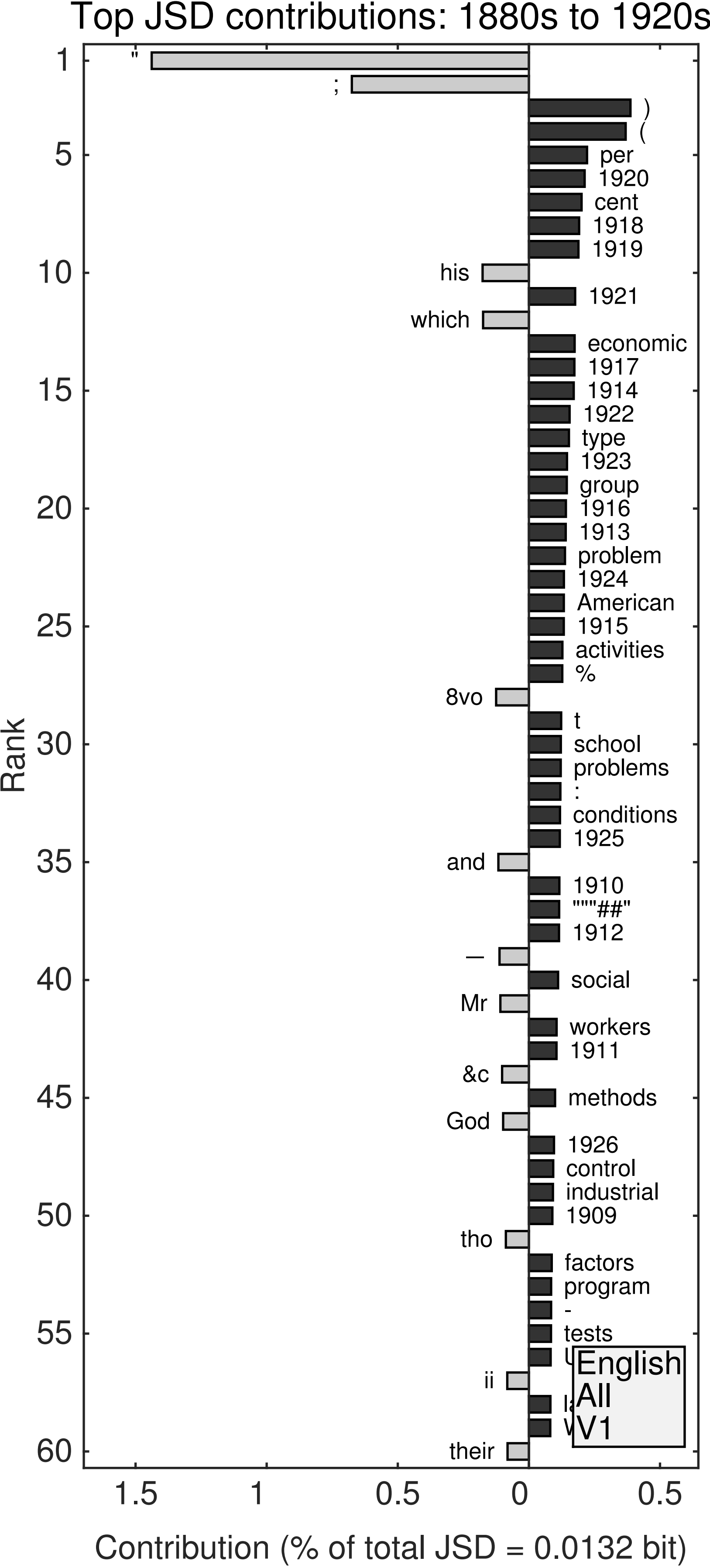

Top JSD contributions: 1880s to 1920s
Rank
"
;
)
(
per
1920
cent
1918
1919
his
1921
which
economic
1917
1914
1922
type
1923
group
1916
1913
problem
1924
American
1915
activities
%
8vo
t
school
problems
:
conditions
1925
and
1910
"""##"
1912
—
social
Mr
workers
1911
&c
methods
God
1926
control
industrial
1909
tho
factors
program
-
tests
ii
their
English
All
V1
Contribution (% of total JSD = 0.0132 bit)

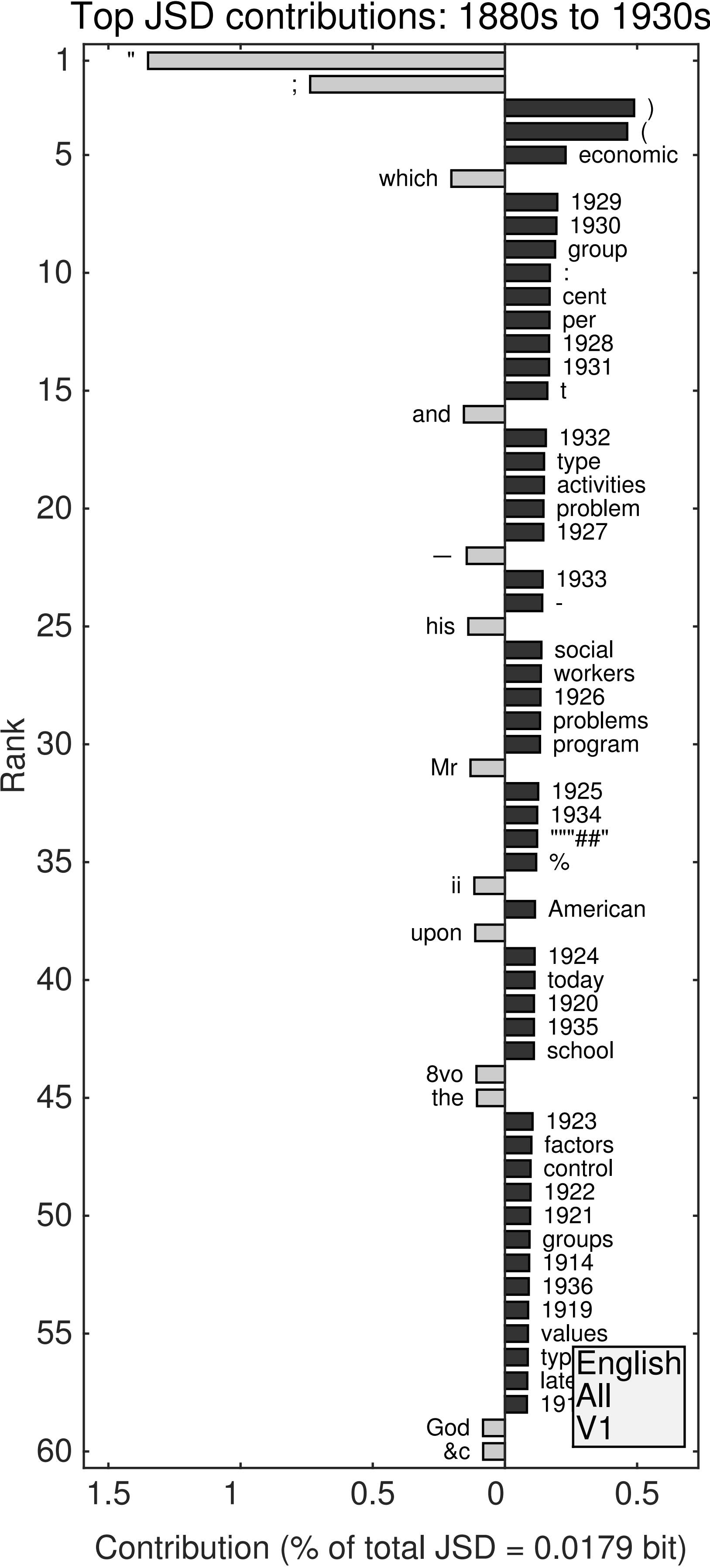

Top JSD contributions: 1880s to 1930s
Rank
Contribution (% of total JSD = 0.0179 bit)
"
;
)
(
economic
which
1929
1930
group
:
cent
per
1928
1931
t
and
1932
type
activities
problem
1927
—
1933
-
his
social
workers
1926
problems
program
Mr
1925
1934
"""##"
%
ii
American
upon
1924
today
1920
1935
school
8vo
the
1923
factors
control
1922
1921
groups
1914
1936
1919
values
God
&c
English
All
V1

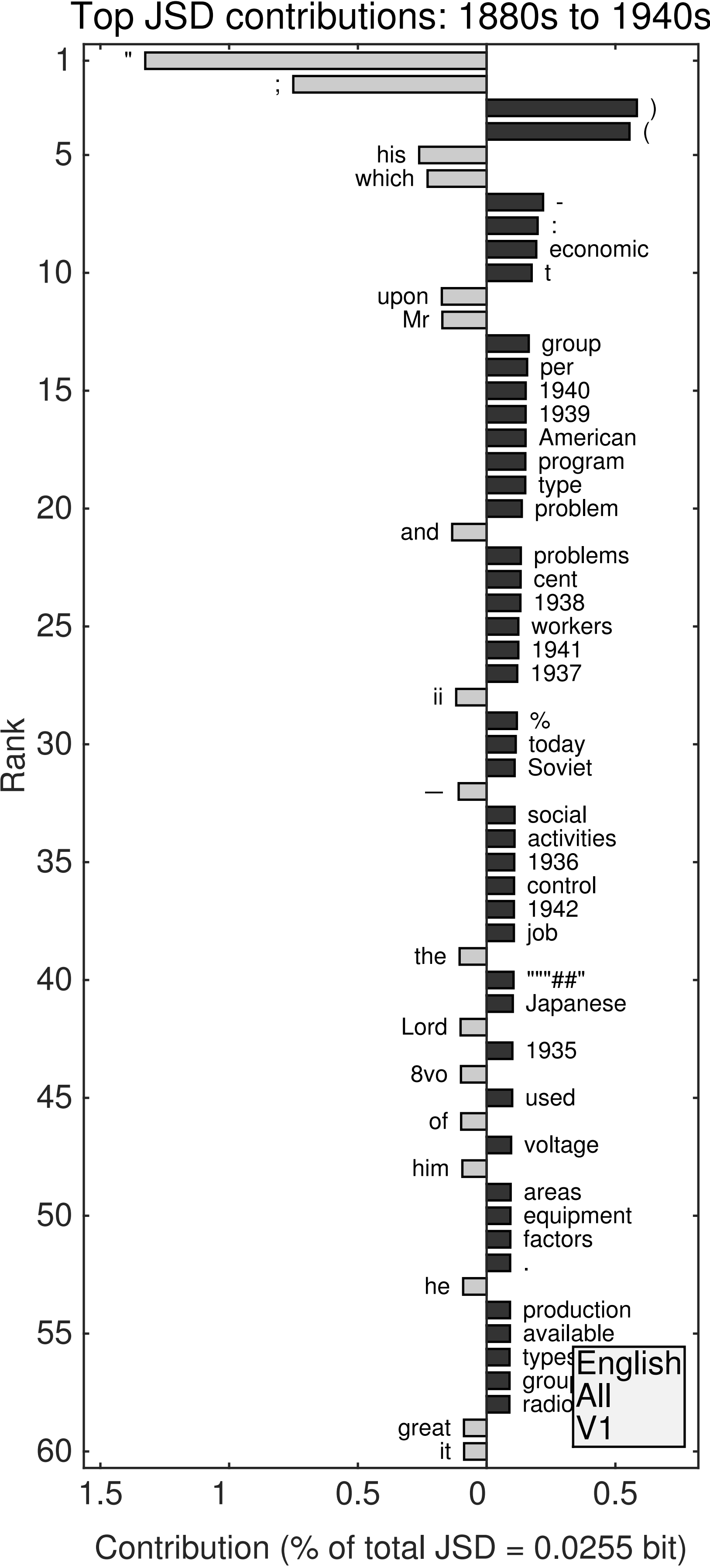
Top JSD contributions: 1880s to 1940s
Rank
"
;
)
(
his
which
-
:
economic
t
upon
Mr
group
per
1940
1939
American
program
type
problem
and
problems
cent
1938
workers
1941
1937
ii
%
today
Soviet
—
social
activities
1936
control
1942
job
the
"""##"
Japanese
Lord
1935
8vo
used
of
voltage
him
areas
equipment
factors
.
he
production
available
great
it
English
All
V1
Contribution (% of total JSD = 0.0255 bit)

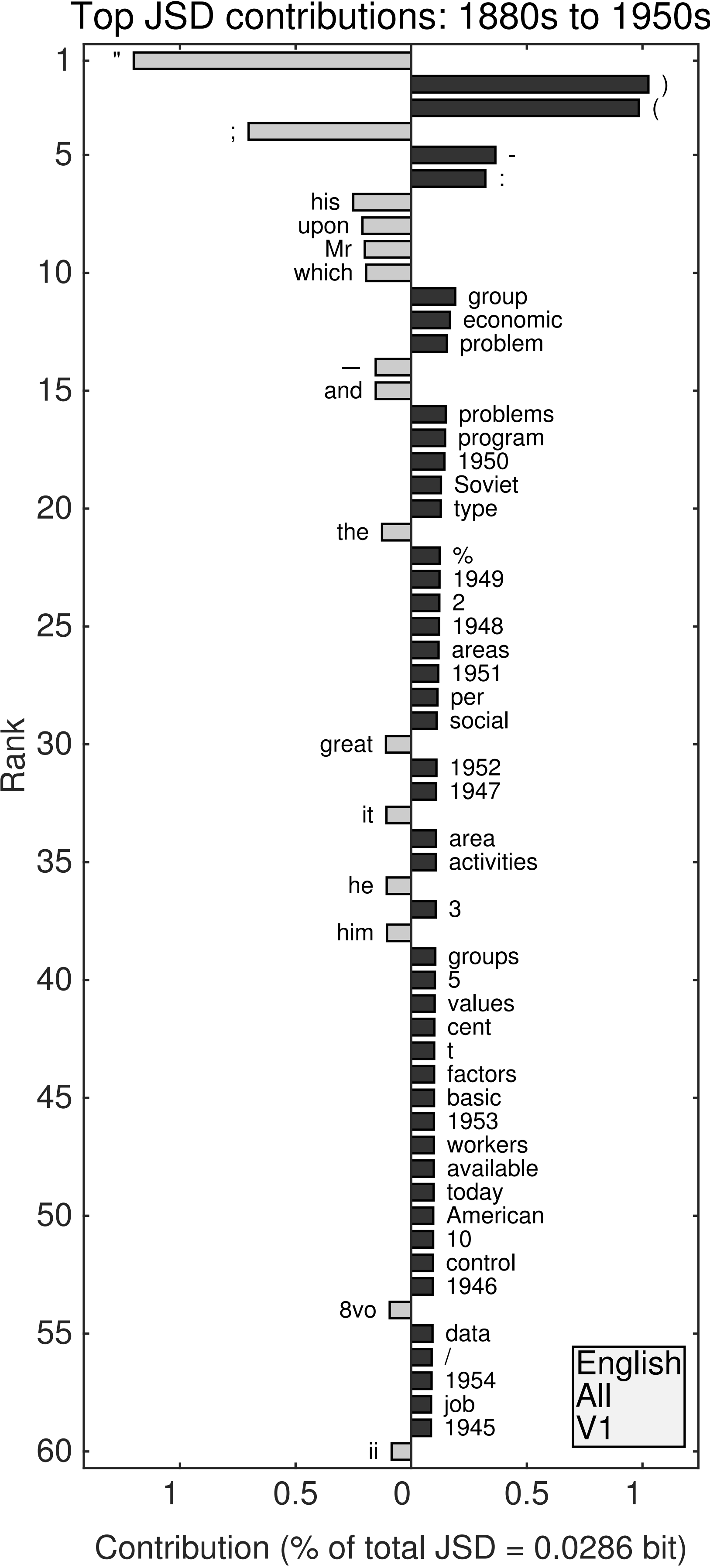

Top JSD contributions: 1880s to 1950s
"
)
(
;
-
:
his
upon
Mr
which
group
economic
problem
—
and
problems
program
1950
Soviet
type
the
%
1949
2
1948
areas
1951
per
social
great
1952
1947
it
area
activities
he
3
him
groups
5
values
cent
t
factors
basic
1953
workers
available
today
American
10
control
1946
8vo
data
/
1954
job
1945
ii
English
All
V1
Rank
1 5 10 15 20 25 30 35 40 45 50 55 60
1 0.5 0 0.5 1
Contribution (% of total JSD = 0.0286 bit)

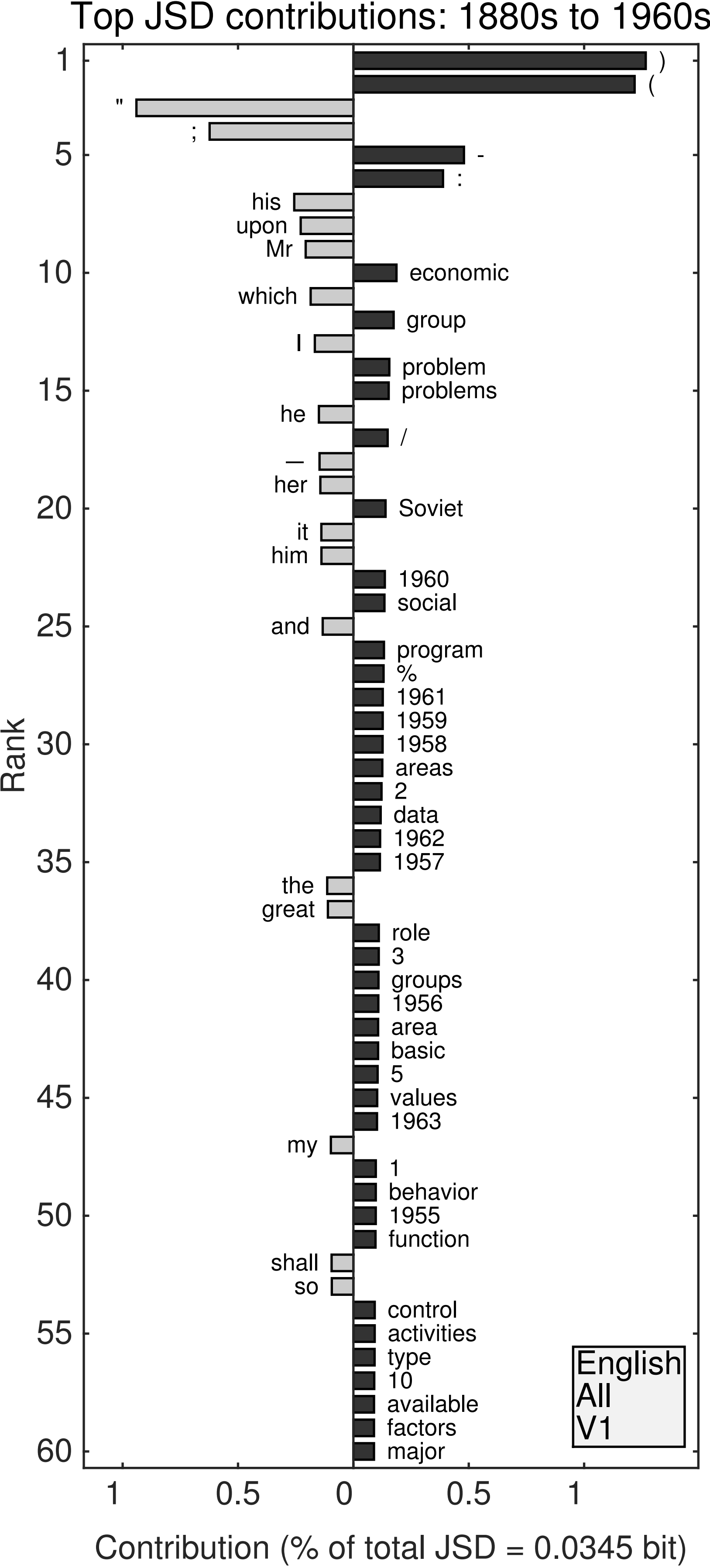

Top JSD contributions: 1880s to 1960s
Rank
1
5
10
15
20
25
30
35
40
45
50
55
60
)
(
"
;
-
:
his
upon
Mr
economic
which
group
I
problem
problems
he
/
—
her
Soviet
it
him
1960
social
and
program
%
1961
1959
1958
areas
2
data
1962
1957
the
great
role
3
groups
1956
area
basic
5
values
1963
my
1
behavior
1955
function
shall
so
control
activities
type
10
available
factors
major
English
All
V1
1
0.5
0
0.5
1
Contribution (% of total JSD = 0.0345 bit)

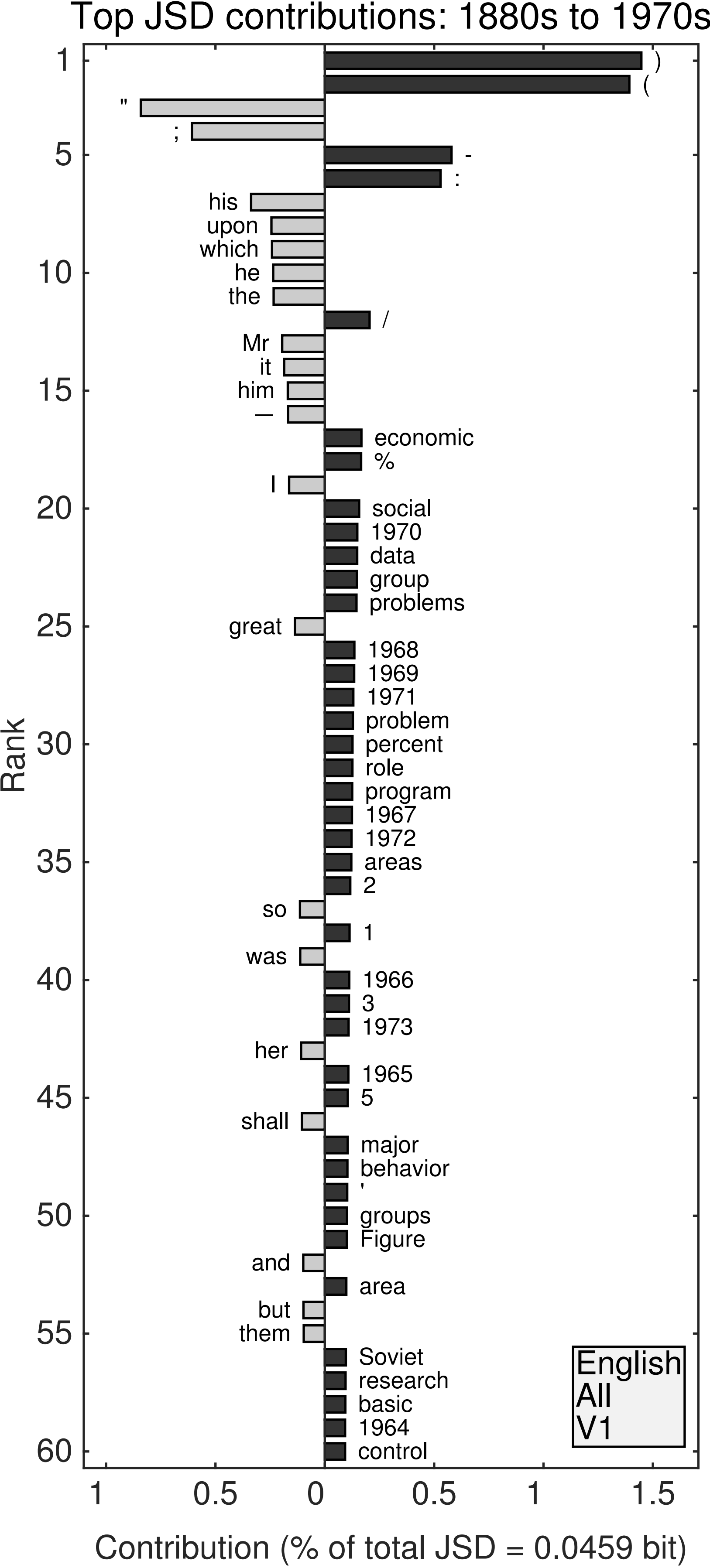
Top JSD contributions: 1880s to 1970s
Rank
Contribution (% of total JSD = 0.0459 bit)
)
(
"
;
-
:
his
upon
which
he
the
/
Mr
it
him
—
economic
%
I
social
1970
data
group
problems
great
1968
1969
1971
problem
percent
role
program
1967
1972
areas
2
so
1
was
1966
3
1973
her
1965
5
shall
major
behavior
'
groups
Figure
and
area
but
them
Soviet
research
basic
1964
control
English
All
V1

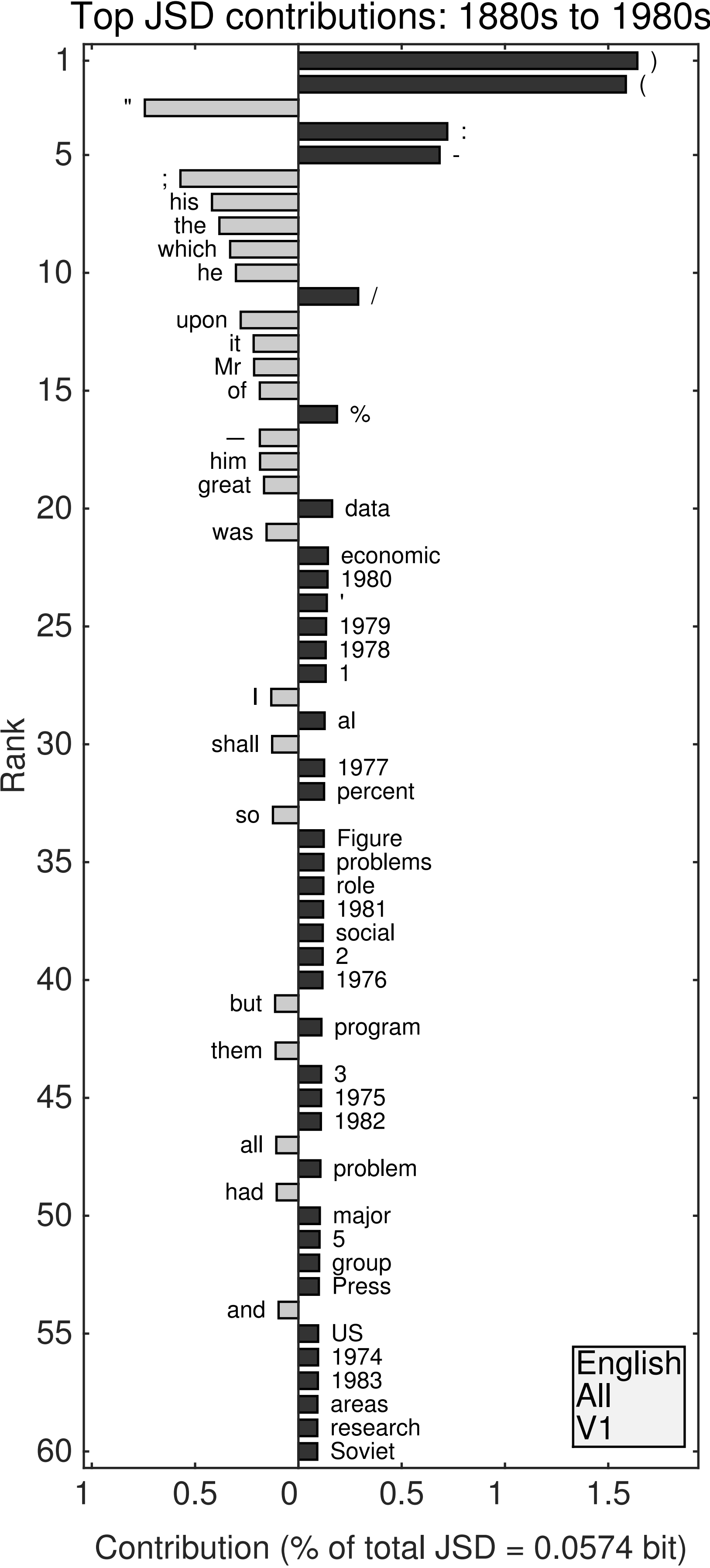

Top JSD contributions: 1880s to 1980s
Rank
Contribution (% of total JSD = 0.0574 bit)
)
(
"
:
-
;
his
the
which
he
/
upon
it
Mr
of
%
—
him
great
data
was
economic
1980
'
1979
1978
1
I
al
shall
1977
percent
so
Figure
problems
role
1981
social
2
1976
but
program
them
3
1975
1982
all
problem
had
major
5
group
Press
and
US
1974
1983
areas
research
Soviet
English
All
V1

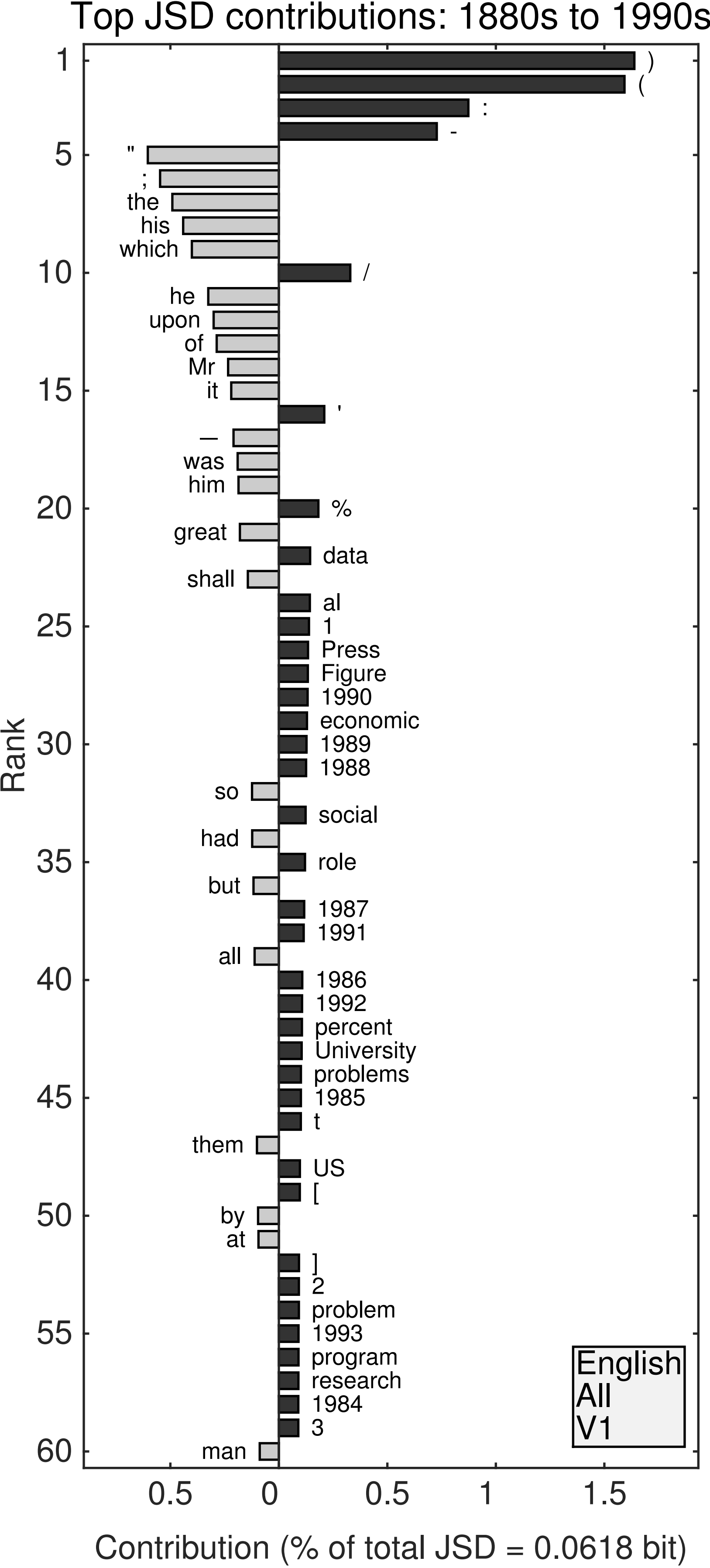
Top JSD contributions: 1880s to 1990s
Rank
Contribution (% of total JSD = 0.0618 bit)
)
(
:
-
"
;
the
his
which
/
he
upon
of
Mr
it
'
—
was
him
%
great
data
shall
al
1
Press
Figure
1990
economic
1989
1988
so
social
had
role
but
1987
1991
all
1986
1992
percent
University
problems
1985
t
them
US
[
by
at
]
2
problem
1993
program
research
1984
3
man
English
All
V1

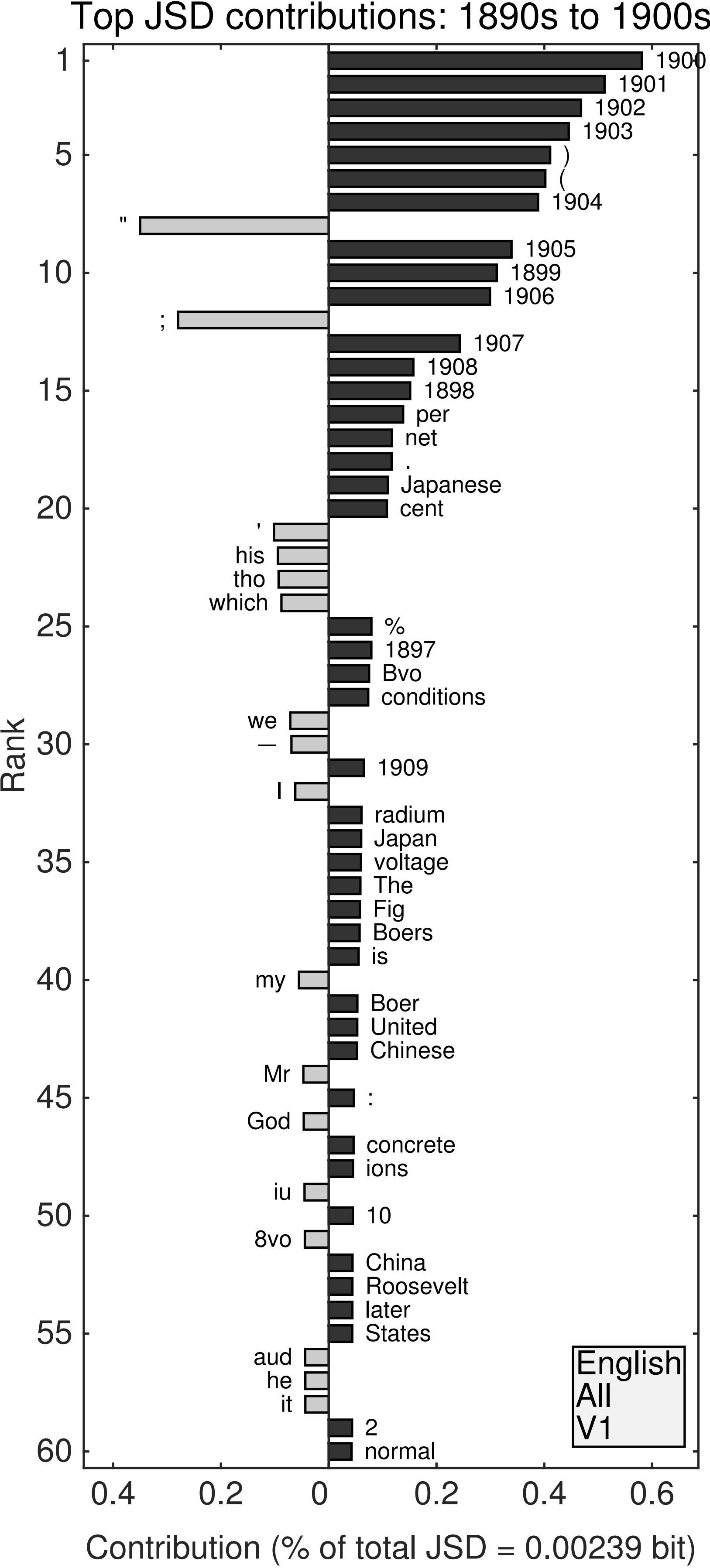
Top JSD contributions: 1890s to 1900s
Rank
Contribution (% of total JSD = 0.00239 bit)
1900
1901
1902
1903
)
(
1904
"
1905
1899
1906
;
1907
1908
1898
per
net
.
Japanese
cent
'
his
tho
which
%
1897
Bvo
conditions
we
—
1909
I
radium
Japan
voltage
The
Fig
Boers
is
my
Boer
United
Chinese
Mr
:
God
concrete
ions
iu
10
8vo
China
Roosevelt
later
States
aud
he
it
2
normal
English
All
V1

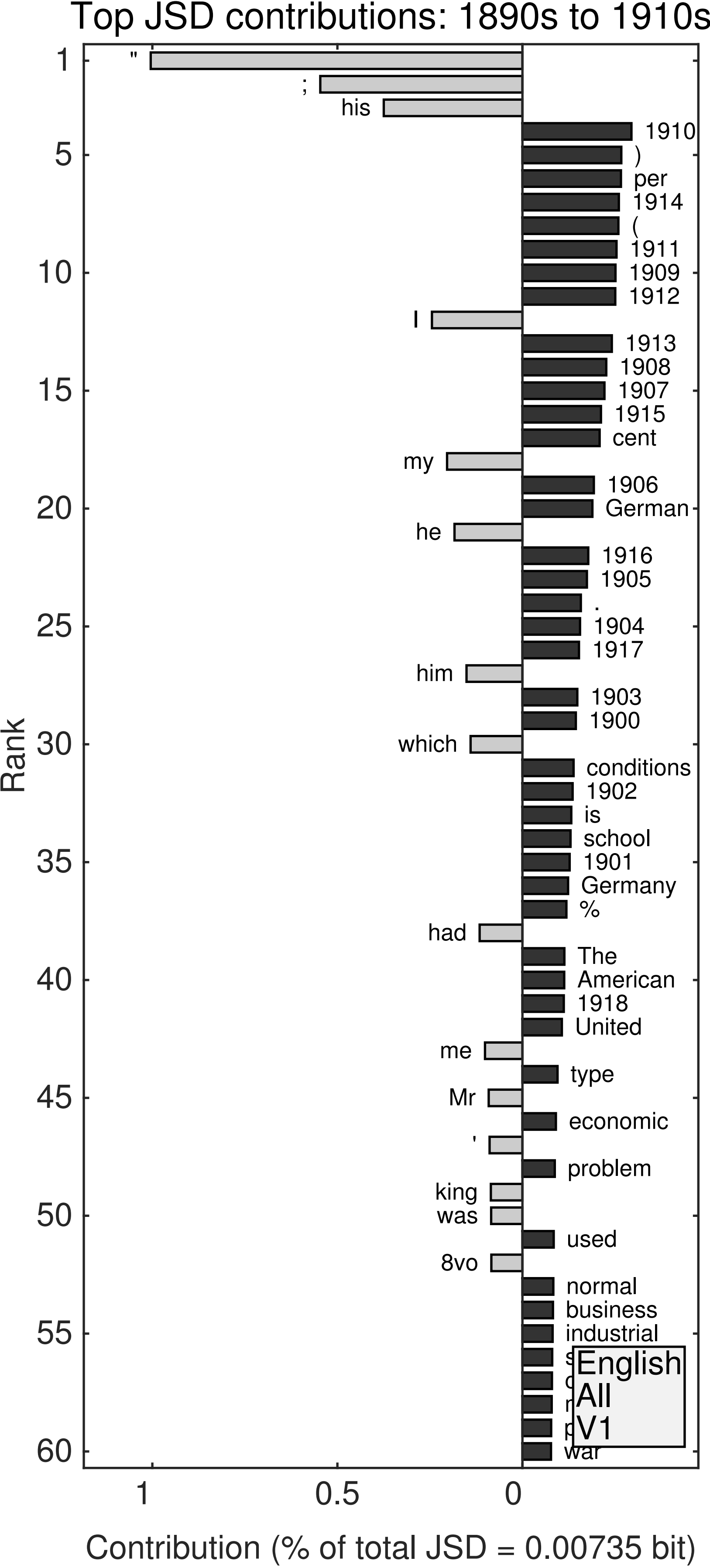

Top JSD contributions: 1890s to 1910s
Rank
1
5
10
15
20
25
30
35
40
45
50
55
60
"
;
his
1910
)
per
1914
(
1911
1909
1912
I
1913
1908
1907
1915
cent
my
1906
German
he
1916
1905
.
1904
1917
him
1903
1900
which
conditions
1902
is
school
1901
Germany
%
had
The
American
1918
United
me
type
Mr
economic
'
problem
king
was
used
8vo
normal
business
industrial
war
English
All
V1
1
0.5
0
Contribution (% of total JSD = 0.00735 bit)

Top JSD contributions: 1890s to 1920s

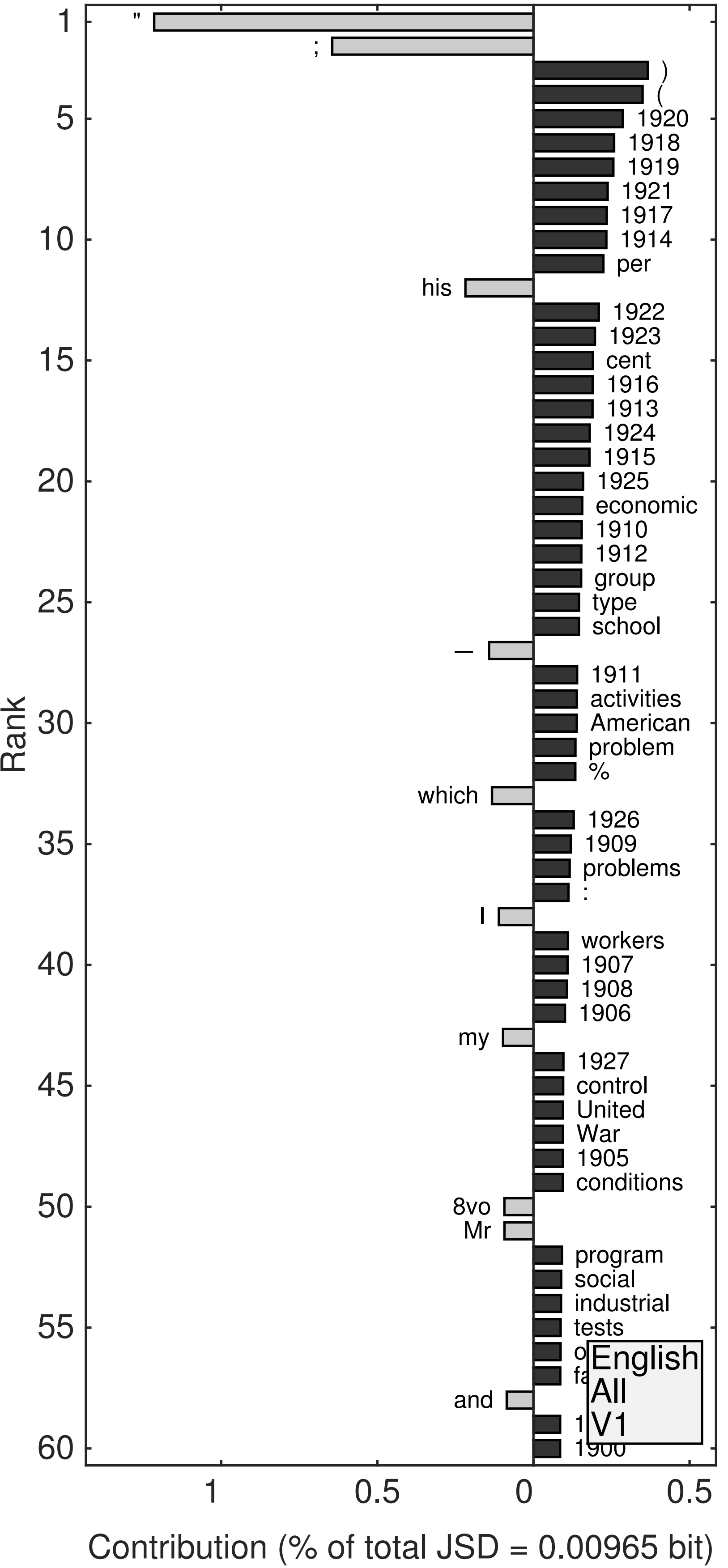

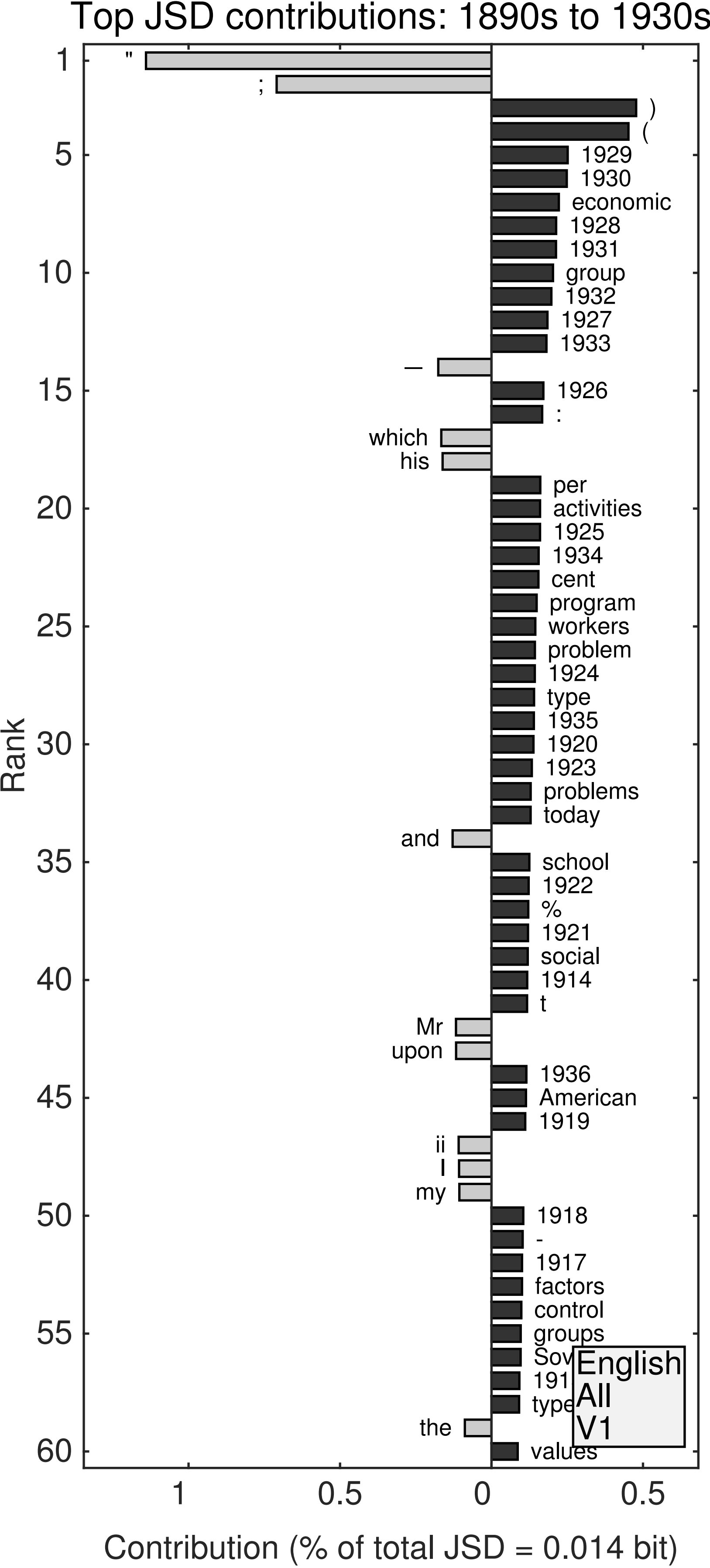

Top JSD contributions: 1890s to 1930s
"
;
)
(
1929
1930
economic
1928
1931
group
1932
1927
1933
—
1926
:
which
his
per
activities
1925
1934
cent
program
workers
problem
1924
type
1935
1920
1923
problems
today
and
school
1922
%
1921
social
1914
t
Mr
upon
1936
American
1919
ii
I
my
1918
-
1917
factors
control
groups
the
values
English
All
V1
Rank
1
5
10
15
20
25
30
35
40
45
50
55
60
1
0.5
0
0.5
Contribution (% of total JSD = 0.014 bit)

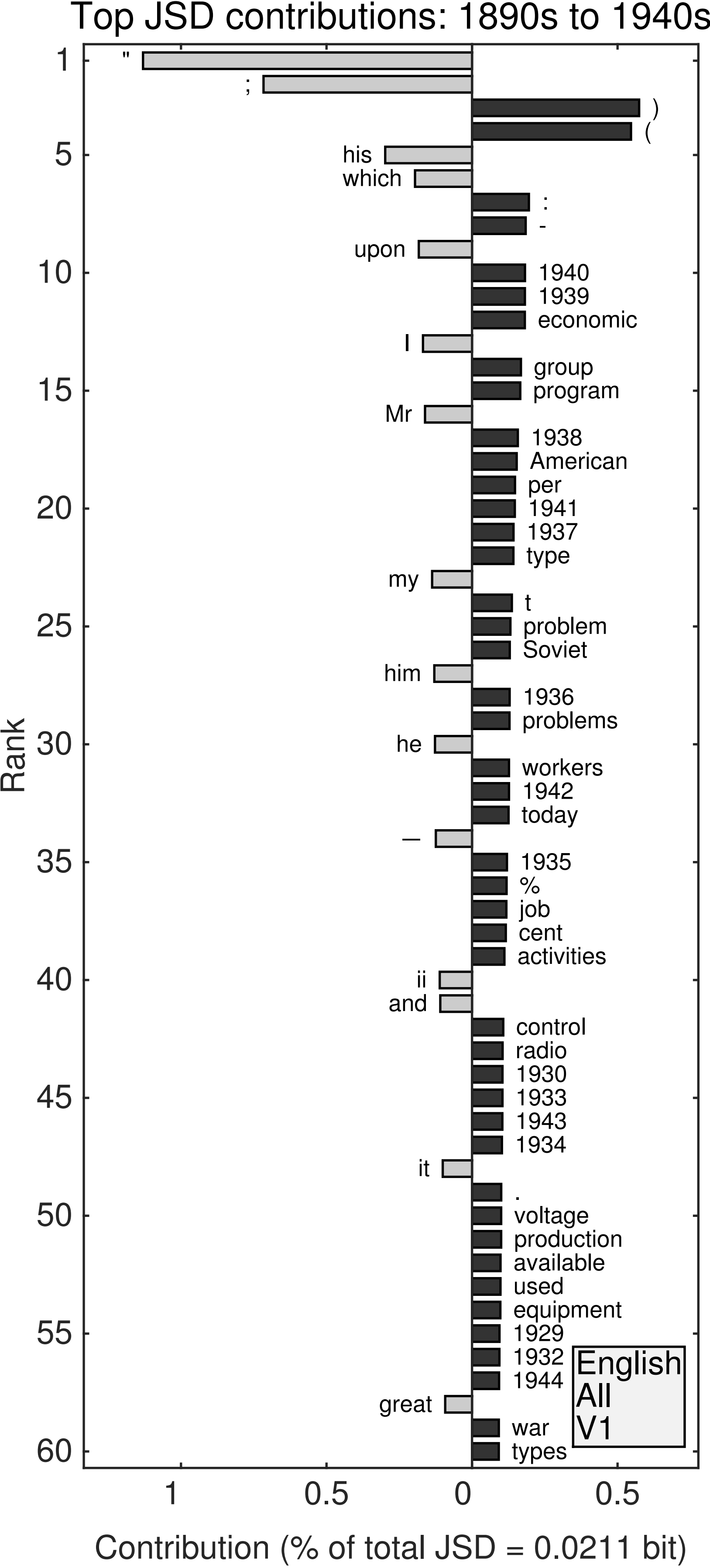

Top JSD contributions: 1890s to 1940s
Rank
Contribution (% of total JSD = 0.0211 bit)
"
;
)
(
his
which
:
-
upon
1940
1939
economic
I
group
program
Mr
1938
American
per
1941
1937
type
my
t
problem
Soviet
him
1936
problems
he
workers
1942
today
—
1935
%
job
cent
activities
ii
and
control
radio
1930
1933
1943
1934
it
.
voltage
production
available
used
equipment
1929
1932
1944
great
war
types
English
All
V1

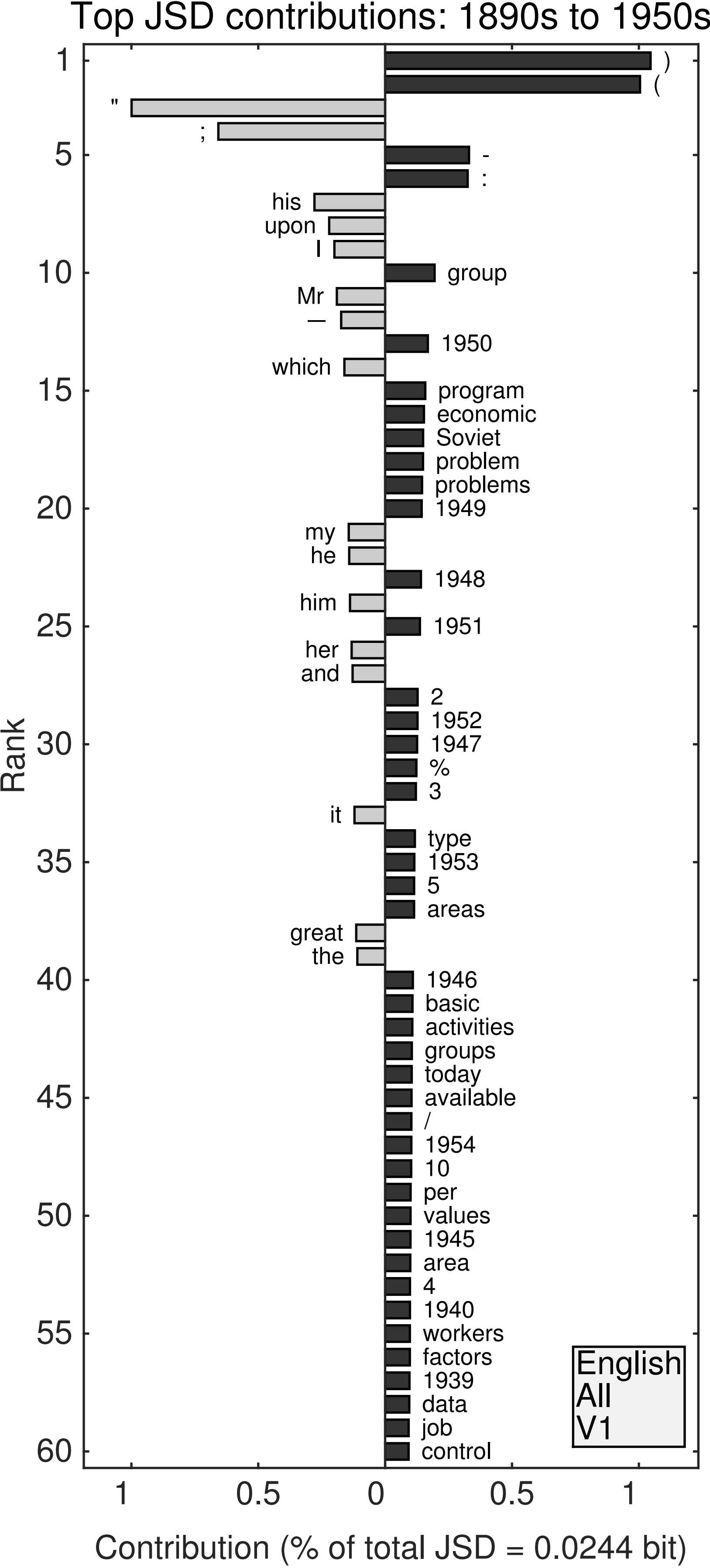
Top JSD contributions: 1890s to 1950s
Rank
Contribution (% of total JSD = 0.0244 bit)
)
(
"
;
-
:
his
upon
I
group
Mr
—
1950
which
program
economic
Soviet
problem
problems
1949
my
he
1948
him
1951
her
and
2
1952
1947
%
3
it
type
1953
5
areas
great
the
1946
basic
activities
groups
today
available
/
1954
10
per
values
1945
area
4
1940
workers
factors
1939
data
job
control
English
All
V1

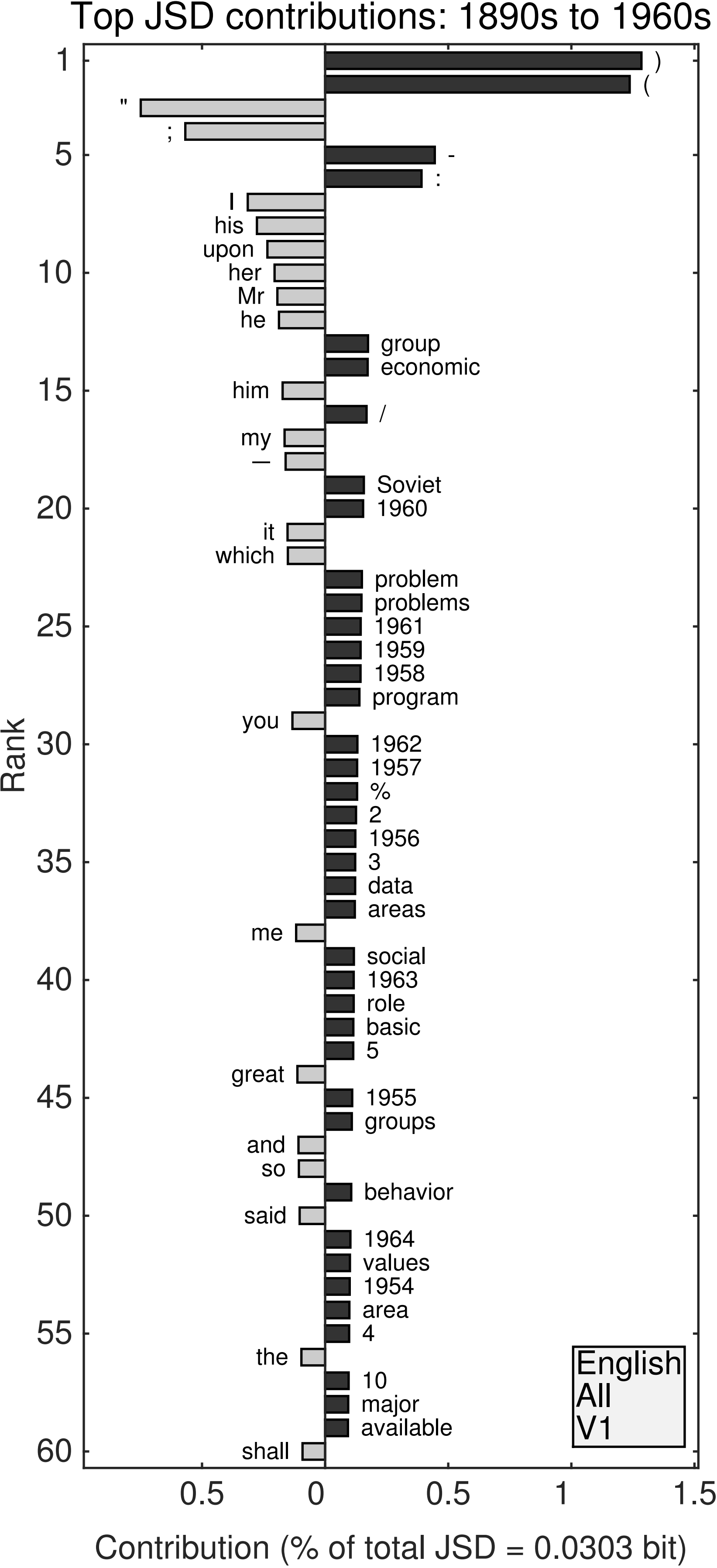

Top JSD contributions: 1890s to 1960s
Rank
Contribution (% of total JSD = 0.0303 bit)
1
5
10
15
20
25
30
35
40
45
50
55
60
0.5
0
0.5
1
1.5
)
(
"
;
-
:
I
his
upon
her
Mr
he
group
economic
him
/
my
—
Soviet
1960
it
which
problem
problems
1961
1959
1958
program
you
1962
1957
%
2
1956
3
data
areas
me
social
1963
role
basic
5
great
1955
groups
and
so
behavior
said
1964
values
1954
area
4
the
10
major
available
shall
English
All
V1

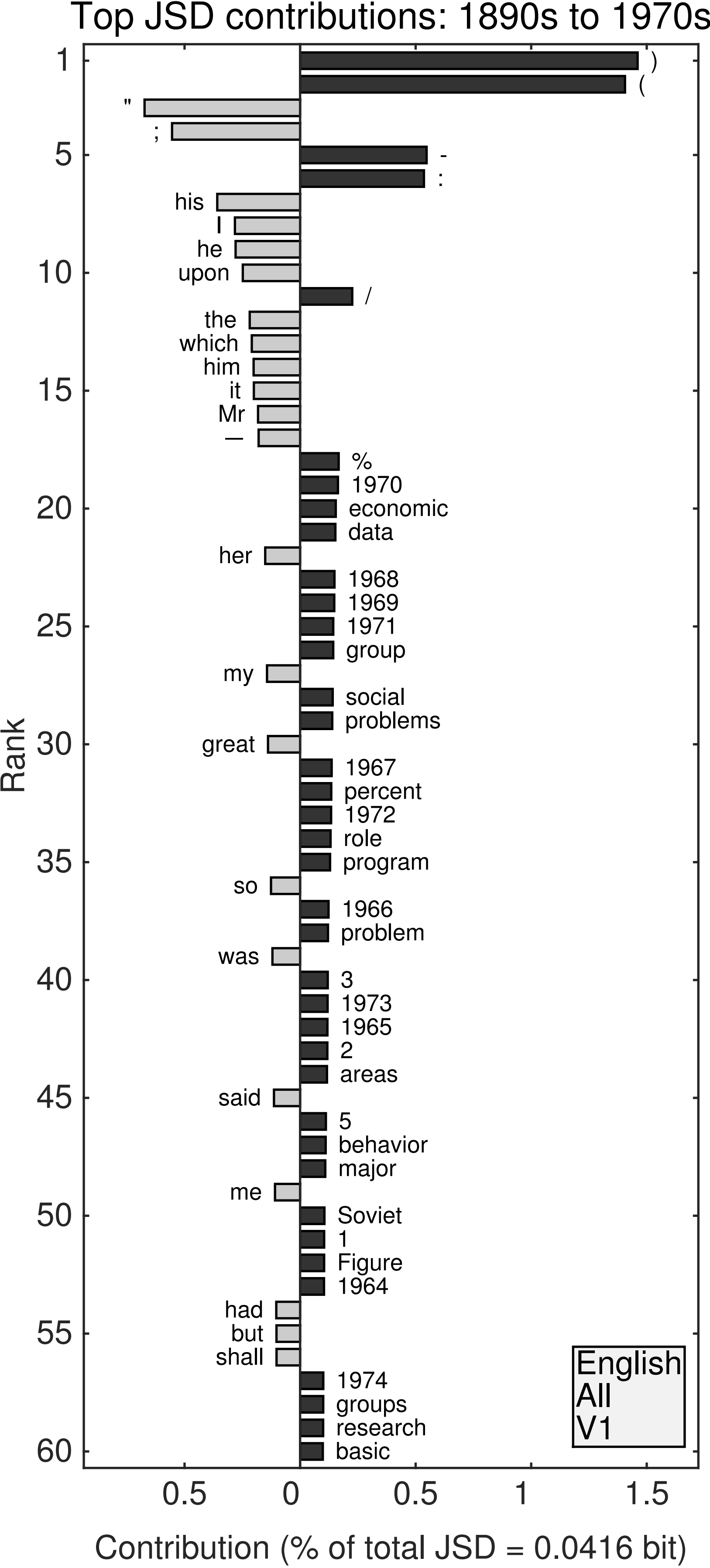

Top JSD contributions: 1890s to 1970s
Rank
Contribution (% of total JSD = 0.0416 bit)
)
(
"
;
-
:
his
I
he
upon
/
the
which
him
it
Mr
—
%
1970
economic
data
her
1968
1969
1971
group
my
social
problems
great
1967
percent
1972
role
program
so
1966
problem
was
3
1973
1965
2
areas
said
5
behavior
major
me
Soviet
1
Figure
1964
had
but
shall
1974
groups
research
basic
English
All
V1

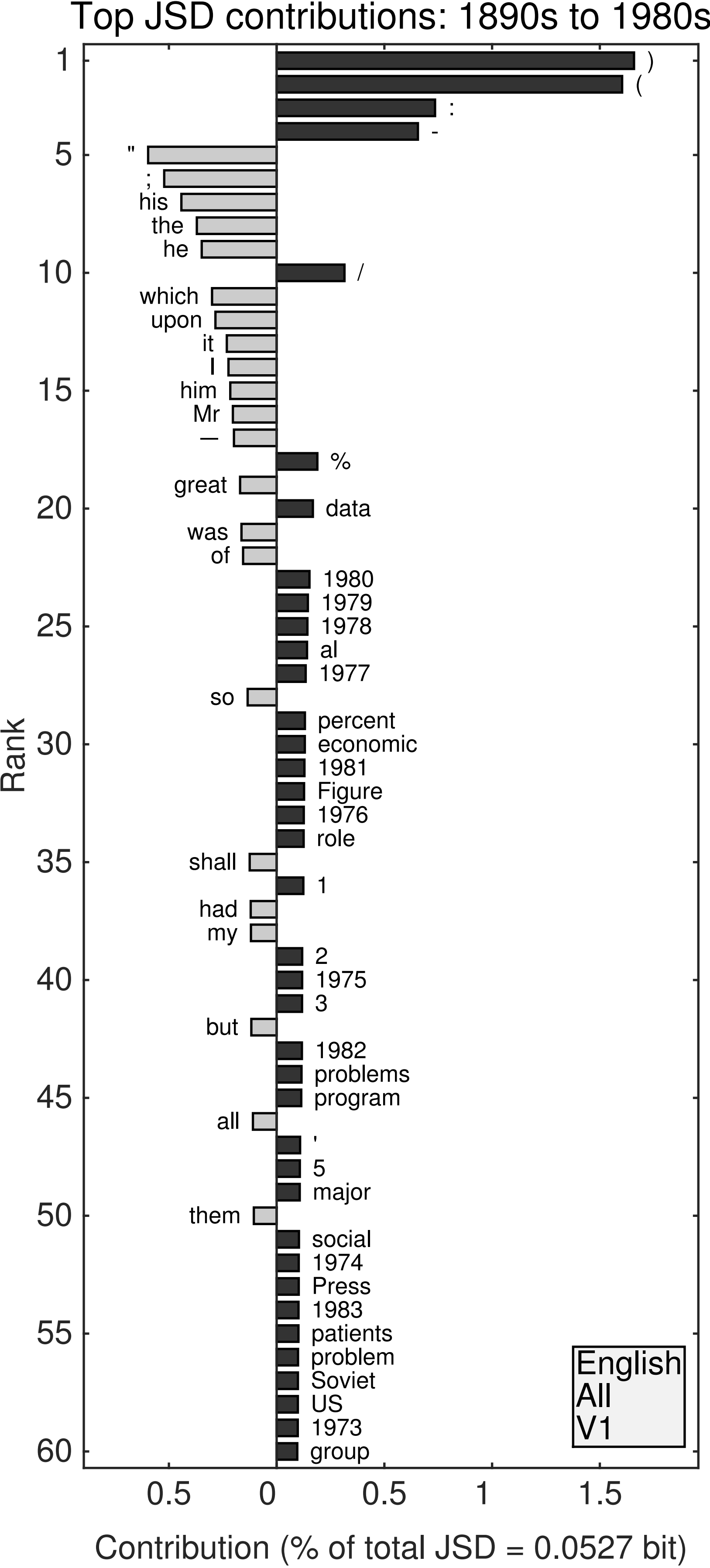
Top JSD contributions: 1890s to 1980s
Rank
Contribution (% of total JSD = 0.0527 bit)
0.5
0
0.5
1
1.5
1
5
10
15
20
25
30
35
40
45
50
55
60
)
(
:
-
"
;
his
the
he
/
which
upon
it
I
him
Mr
—
%
great
data
was
of
1980
1979
1978
al
1977
so
percent
economic
1981
Figure
1976
role
shall
1
had
my
2
1975
3
but
1982
problems
program
all
'
5
major
them
social
1974
Press
1983
patients
problem
Soviet
US
1973
group
English
All
V1

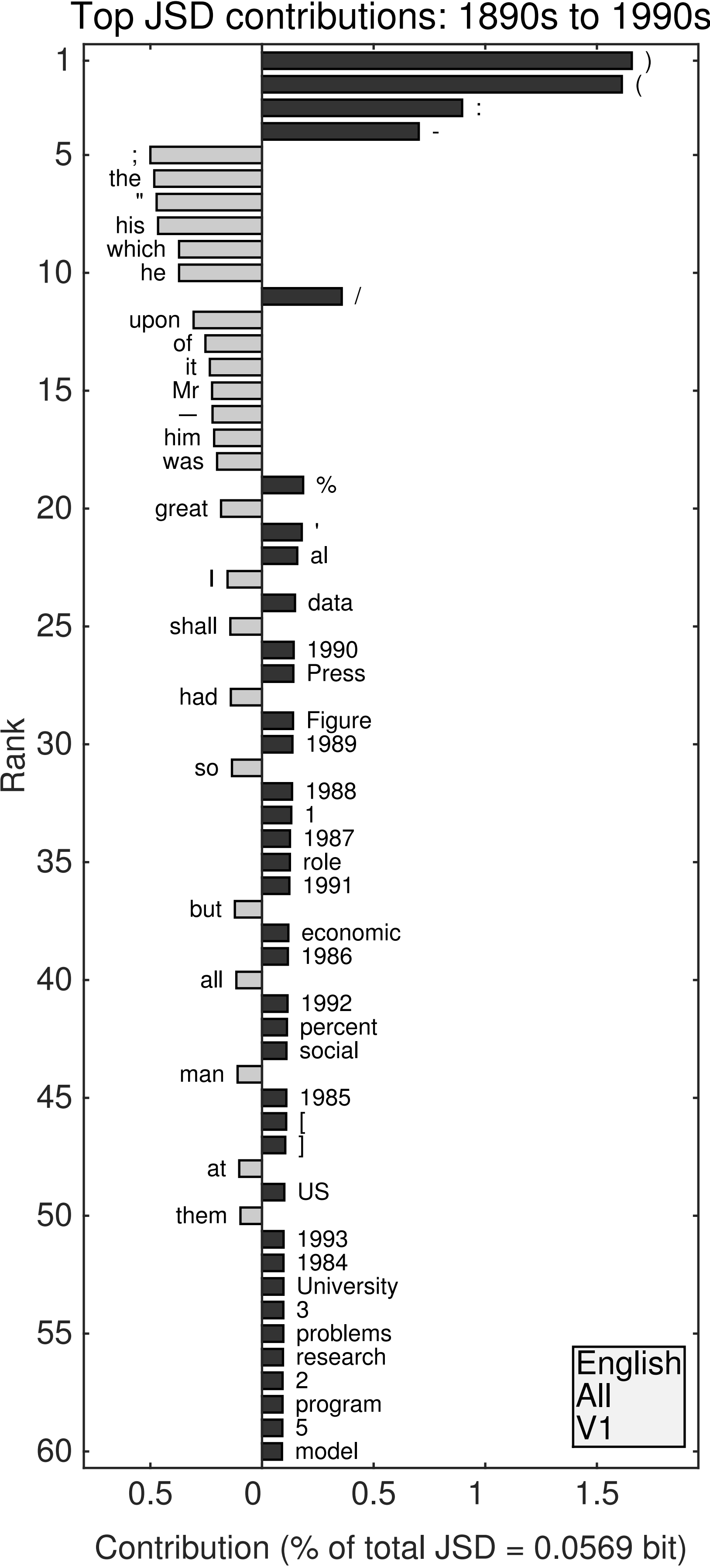
Top JSD contributions: 1890s to 1990s
Rank
Contribution (% of total JSD = 0.0569 bit)
)
(
:
-
;
the
"
his
which
he
/
upon
of
it
Mr
—
him
was
%
great
'
al
I
data
shall
1990
Press
had
Figure
1989
so
1988
1
1987
role
1991
but
economic
1986
all
1992
percent
social
man
1985
[
]
at
US
them
1993
1984
University
3
problems
research
2
program
5
model
English
All
V1

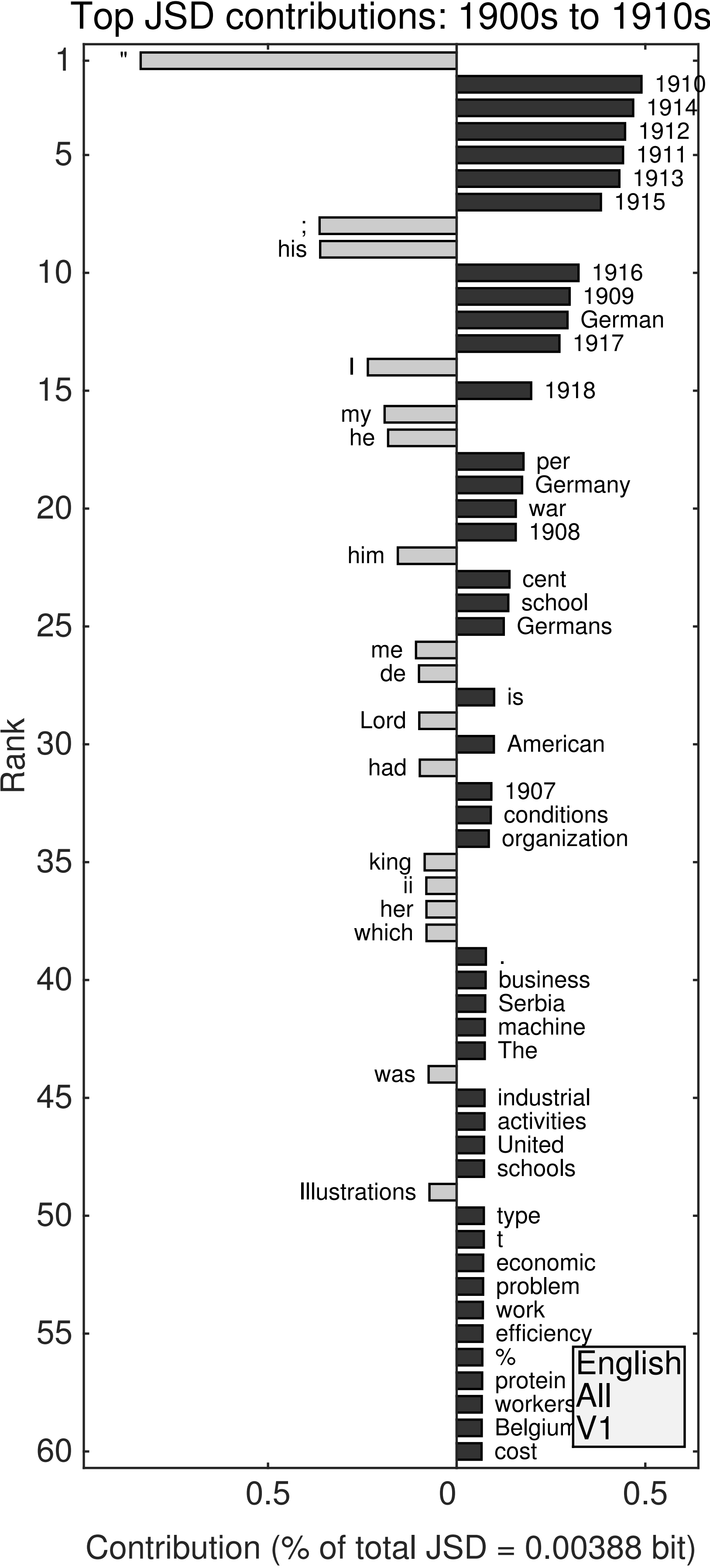
Top JSD contributions: 1900s to 1910s
Rank
Contribution (% of total JSD = 0.00388 bit)
"
1910
1914
1912
1911
1913
1915
;
his
1916
1909
German
1917
I
1918
my
he
per
Germany
war
1908
him
cent
school
Germans
me
de
is
Lord
American
had
1907
conditions
organization
king
ii
her
which
.
business
Serbia
machine
The
was
industrial
activities
United
schools
Illustrations
type
t
economic
problem
work
efficiency
%
protein
workers
Belgium
cost
English
All
V1

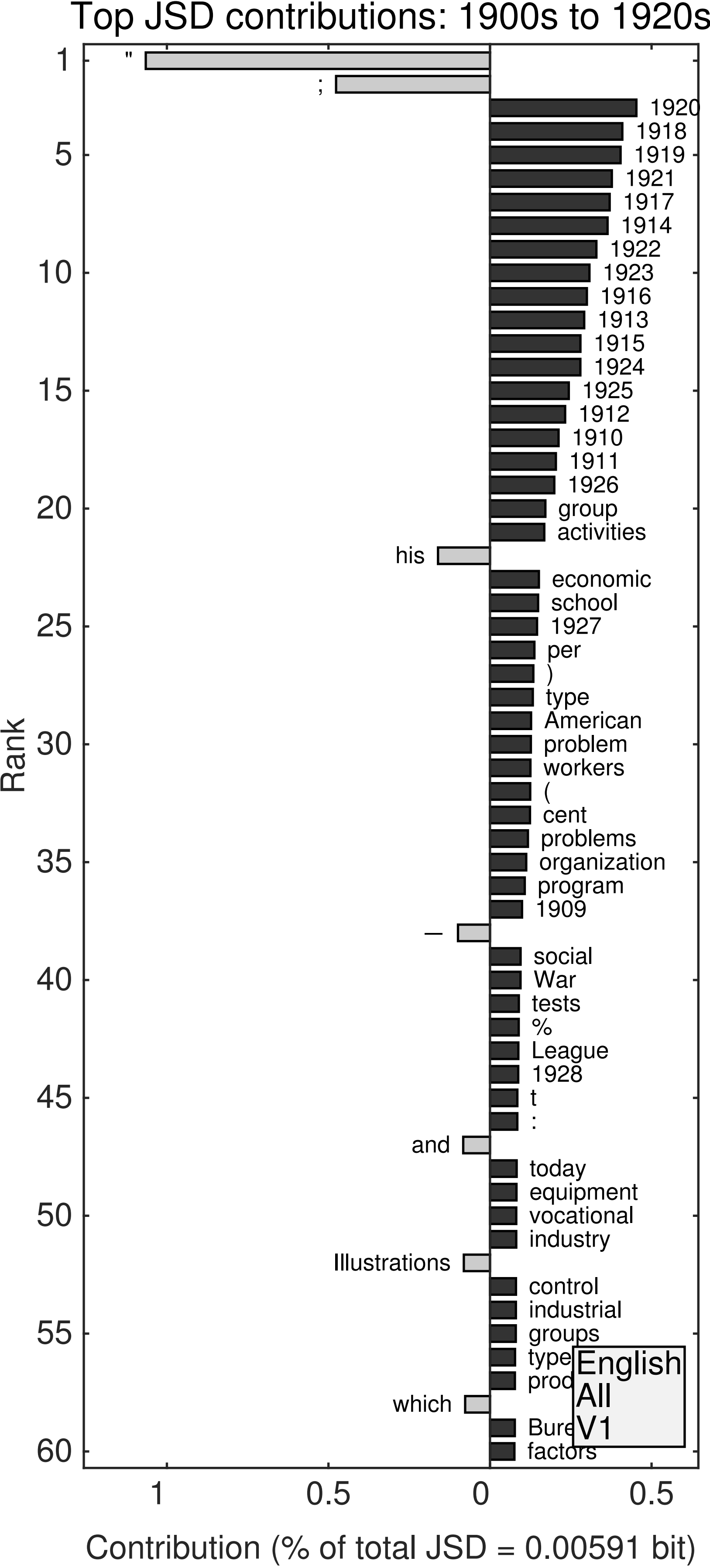

Top JSD contributions: 1900s to 1920s
Rank
Contribution (% of total JSD = 0.00591 bit)
"
;
1920
1918
1919
1921
1917
1914
1922
1923
1916
1913
1915
1924
1925
1912
1910
1911
1926
group
activities
his
economic
school
1927
per
)
type
American
problem
workers
(
cent
problems
organization
program
1909
—
social
War
tests
%
League
1928
t
:
and
today
equipment
vocational
industry
Illustrations
control
industrial
groups
which
factors
English
All
V1

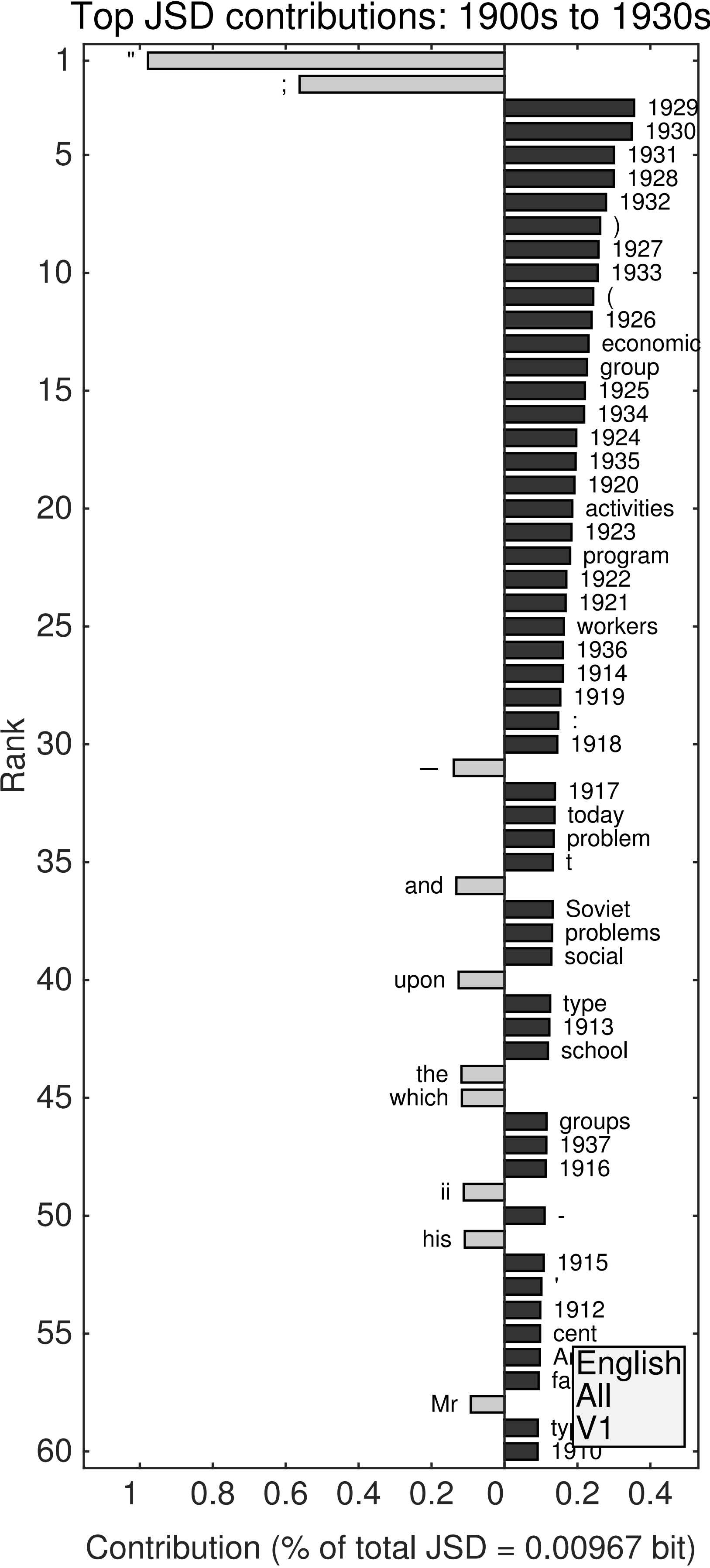
Top JSD contributions: 1900s to 1930s
Rank
Contribution (% of total JSD = 0.00967 bit)
1
5
10
15
20
25
30
35
40
45
50
55
60
1
0.8
0.6
0.4
0.2
0
0.2
0.4
"
;
1929
1930
1931
1928
1932
)
1927
1933
(
1926
economic
group
1925
1934
1924
1935
1920
activities
1923
program
1922
1921
workers
1936
1914
1919
:
1918
—
1917
today
problem
t
and
Soviet
problems
social
upon
type
1913
school
the
which
groups
1937
1916
ii
-
his
1915
'
1912
cent
Mr
English
All
V1

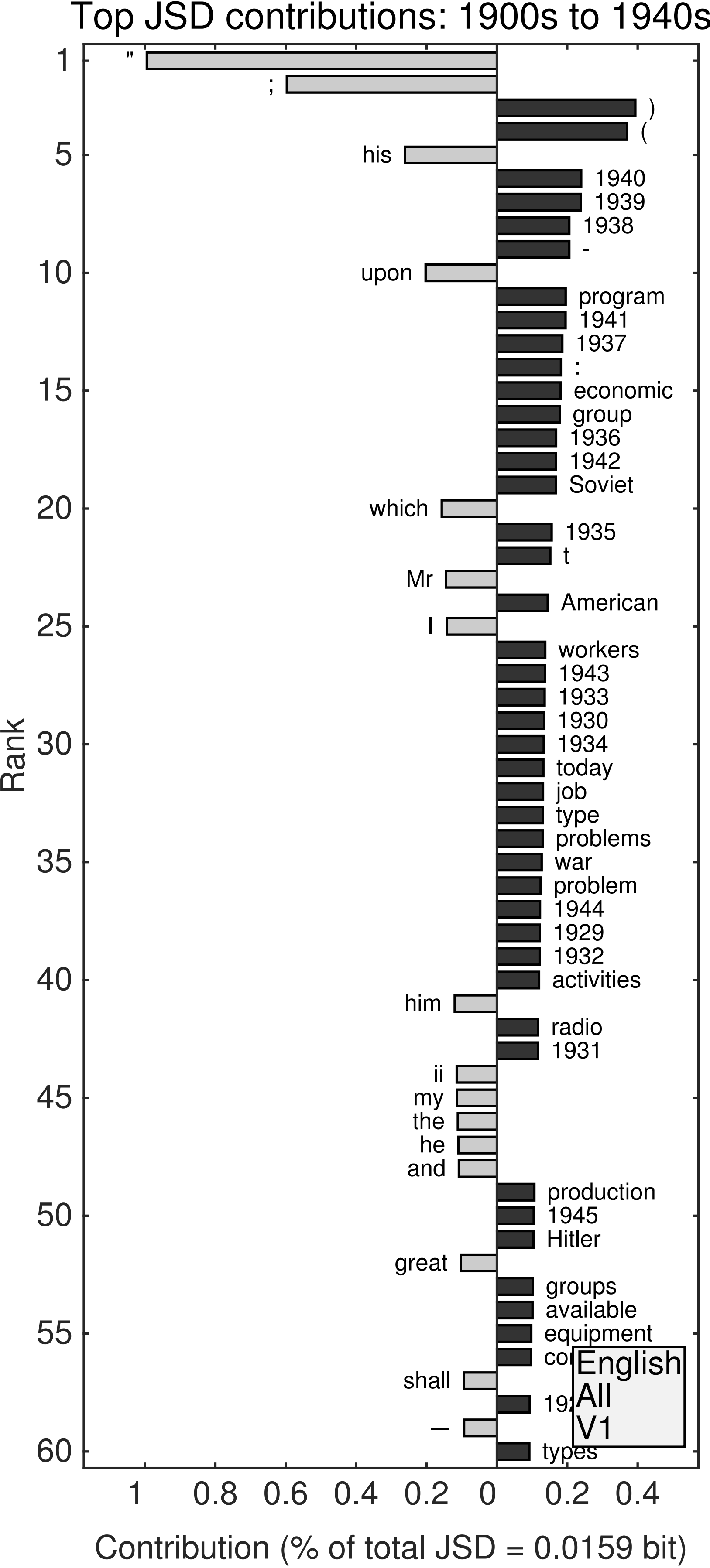
Top JSD contributions: 1900s to 1940s
Rank
Contribution (% of total JSD = 0.0159 bit)
"
;
)
(
his
1940
1939
1938
-
upon
program
1941
1937
:
economic
group
1936
1942
Soviet
which
1935
t
Mr
American
I
workers
1943
1933
1930
1934
today
job
type
problems
war
problem
1944
1929
1932
activities
him
radio
1931
ii
my
the
he
and
production
1945
Hitler
great
groups
available
equipment
shall
—
types
English
All
V1

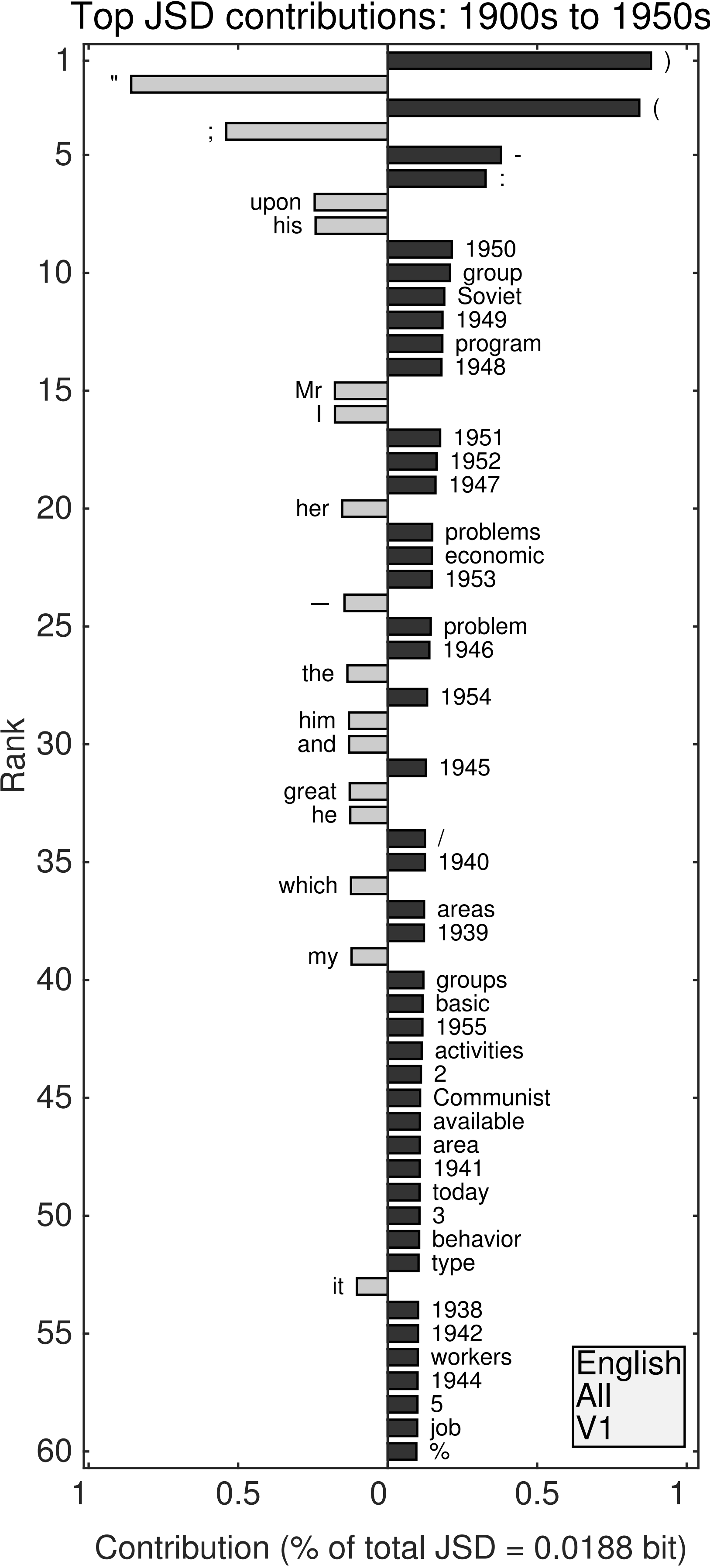

Top JSD contributions: 1900s to 1950s
Rank
)
"
(
;
-
:
upon
his
1950
group
Soviet
1949
program
1948
Mr
I
1951
1952
1947
her
problems
economic
1953
—
problem
1946
the
1954
him
and
1945
great
he
/
1940
which
areas
1939
my
groups
basic
1955
activities
2
Communist
available
area
1941
today
3
behavior
type
it
1938
1942
workers
1944
5
job
%
English
All
V1
Contribution (% of total JSD = 0.0188 bit)

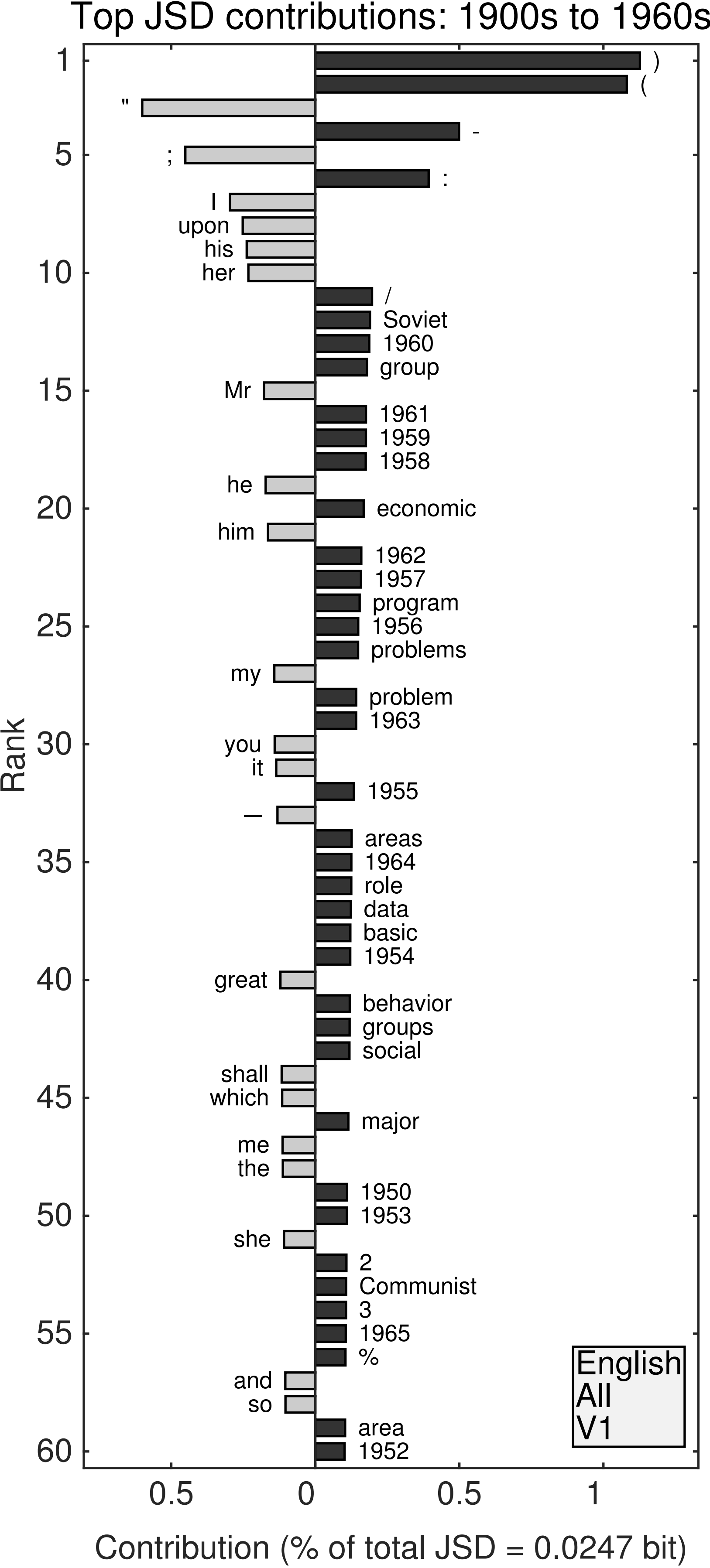
Top JSD contributions: 1900s to 1960s
Rank
Contribution (% of total JSD = 0.0247 bit)
)
(
"
-
;
:
I
upon
his
her
/
Soviet
1960
group
Mr
1961
1959
1958
he
economic
him
1962
1957
program
1956
problems
my
problem
1963
you
it
1955
—
areas
1964
role
data
basic
1954
great
behavior
groups
social
shall
which
major
me
the
1950
1953
she
2
Communist
3
1965
%
and
so
area
1952
English
All
V1

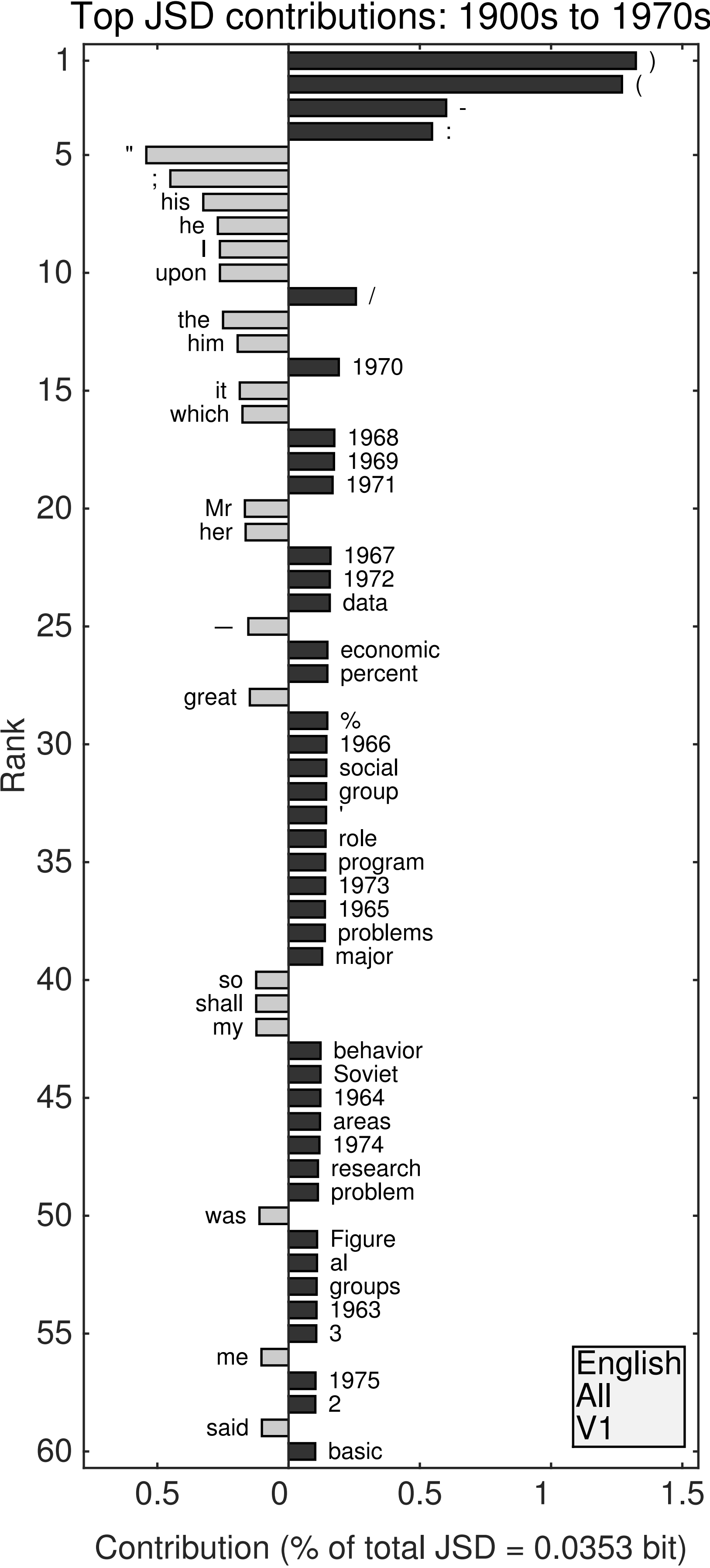

Top JSD contributions: 1900s to 1970s
Rank
Contribution (% of total JSD = 0.0353 bit)
)
(
-
:
"
;
his
he
I
upon
/
the
him
1970
it
which
1968
1969
1971
Mr
her
1967
1972
data
—
economic
percent
great
%
1966
social
group
'
role
program
1973
1965
problems
major
so
shall
my
behavior
Soviet
1964
areas
1974
research
problem
was
Figure
al
groups
1963
3
me
1975
2
said
basic
English
All
V1

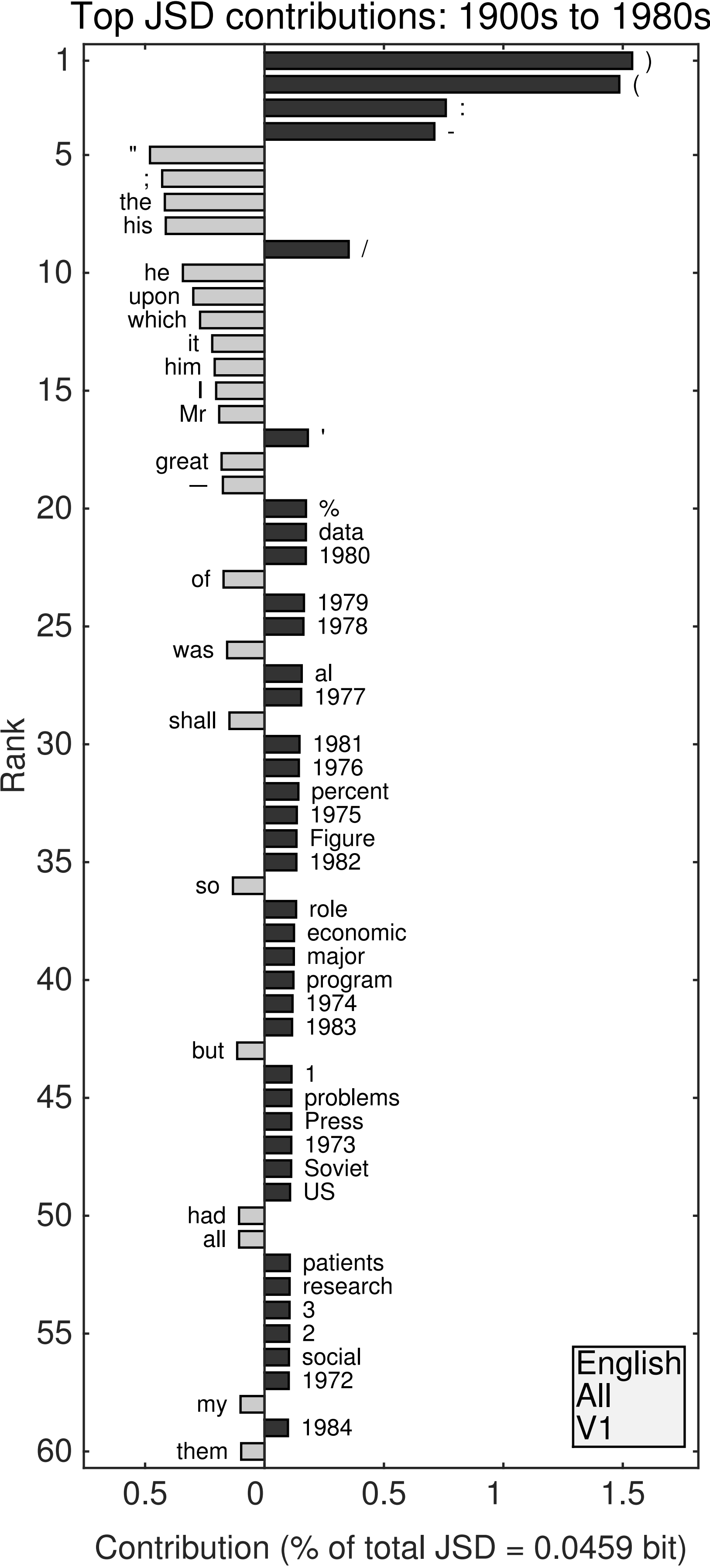

Top JSD contributions: 1900s to 1980s
Rank
Contribution (% of total JSD = 0.0459 bit)
)
(
:
-
"
;
the
his
/
he
upon
which
it
him
I
Mr
'
great
—
%
data
1980
of
1979
1978
was
al
1977
shall
1981
1976
percent
1975
Figure
1982
so
role
economic
major
program
1974
1983
but
1
problems
Press
1973
Soviet
US
had
all
patients
research
3
2
social
1972
my
1984
them
English
All
V1

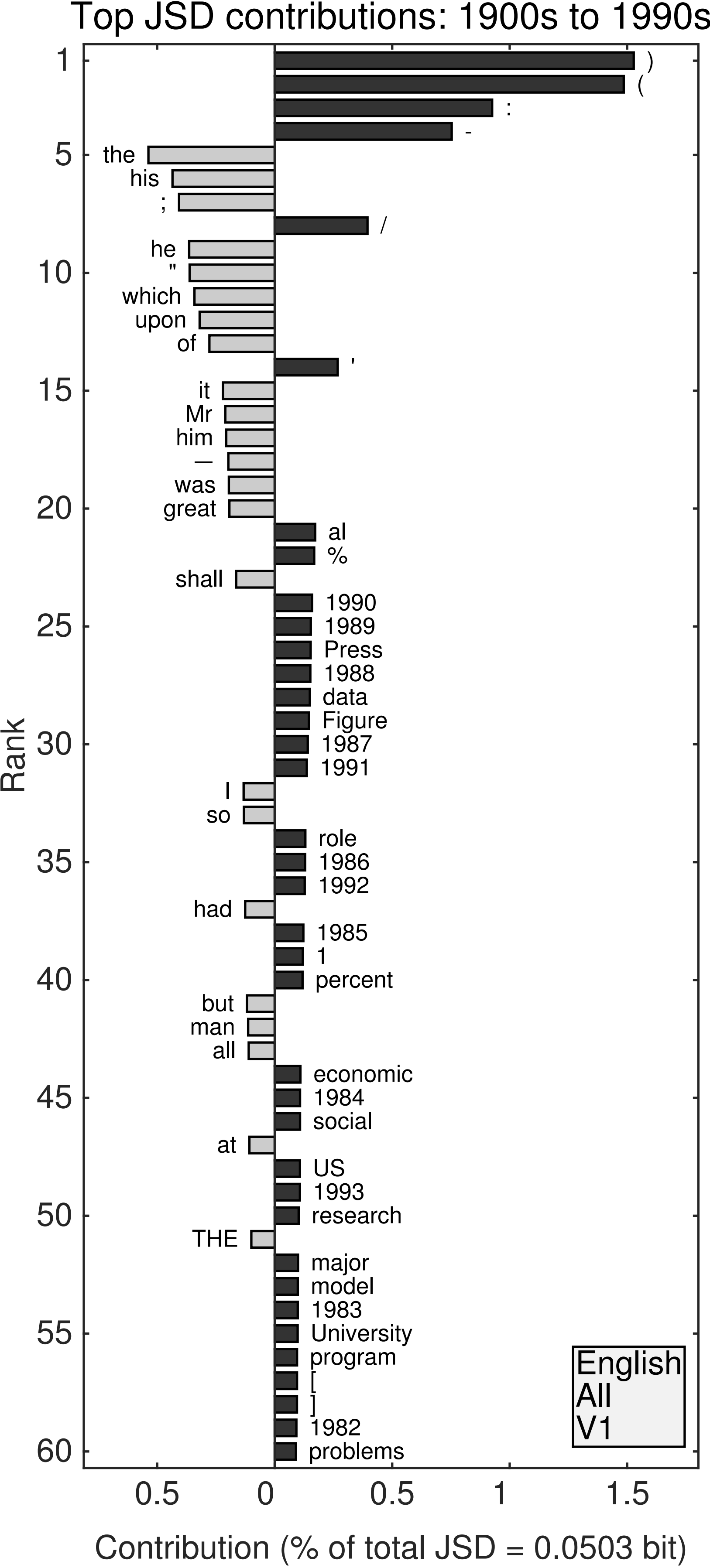
Top JSD contributions: 1900s to 1990s
Rank
Contribution (% of total JSD = 0.0503 bit)
0.5
0
0.5
1
1.5
1
5
10
15
20
25
30
35
40
45
50
55
60
)
(
:
-
the
his
;
/
he
"
which
upon
of
'
it
Mr
him
—
was
great
al
%
shall
1990
1989
Press
1988
data
Figure
1987
1991
I
so
role
1986
1992
had
1985
1
percent
but
man
all
economic
1984
social
at
US
1993
research
THE
major
model
1983
University
program
[
]
1982
problems
English
All
V1

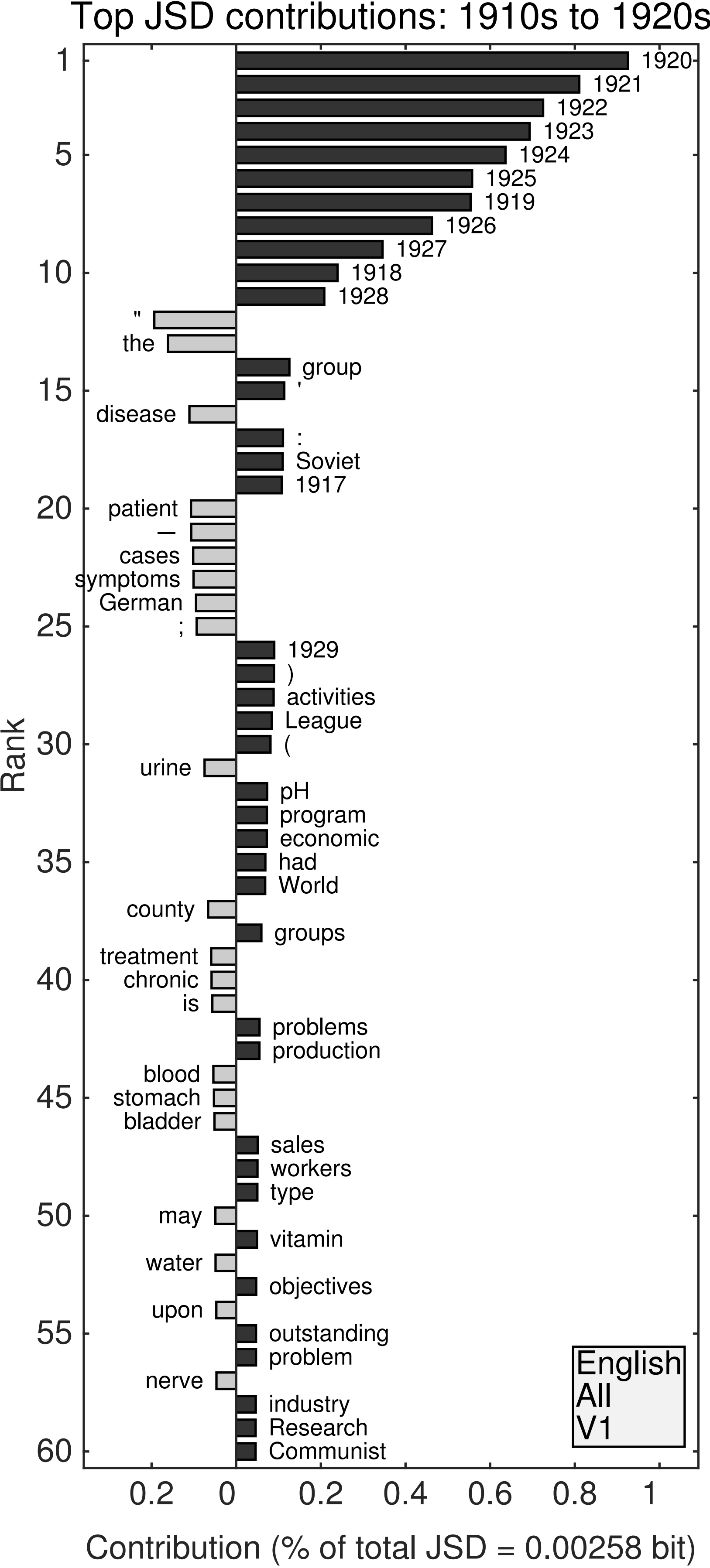
Top JSD contributions: 1910s to 1920s
Rank
Contribution (% of total JSD = 0.00258 bit)
1920
1921
1922
1923
1924
1925
1919
1926
1927
1918
1928
"
the
group
'
disease
:
Soviet
1917
patient
—
cases
symptoms
German
;
1929
)
activities
League
(
urine
pH
program
economic
had
World
county
groups
treatment
chronic
is
problems
production
blood
stomach
bladder
sales
workers
type
may
vitamin
water
objectives
upon
outstanding
problem
nerve
industry
Research
Communist
English
All
V1

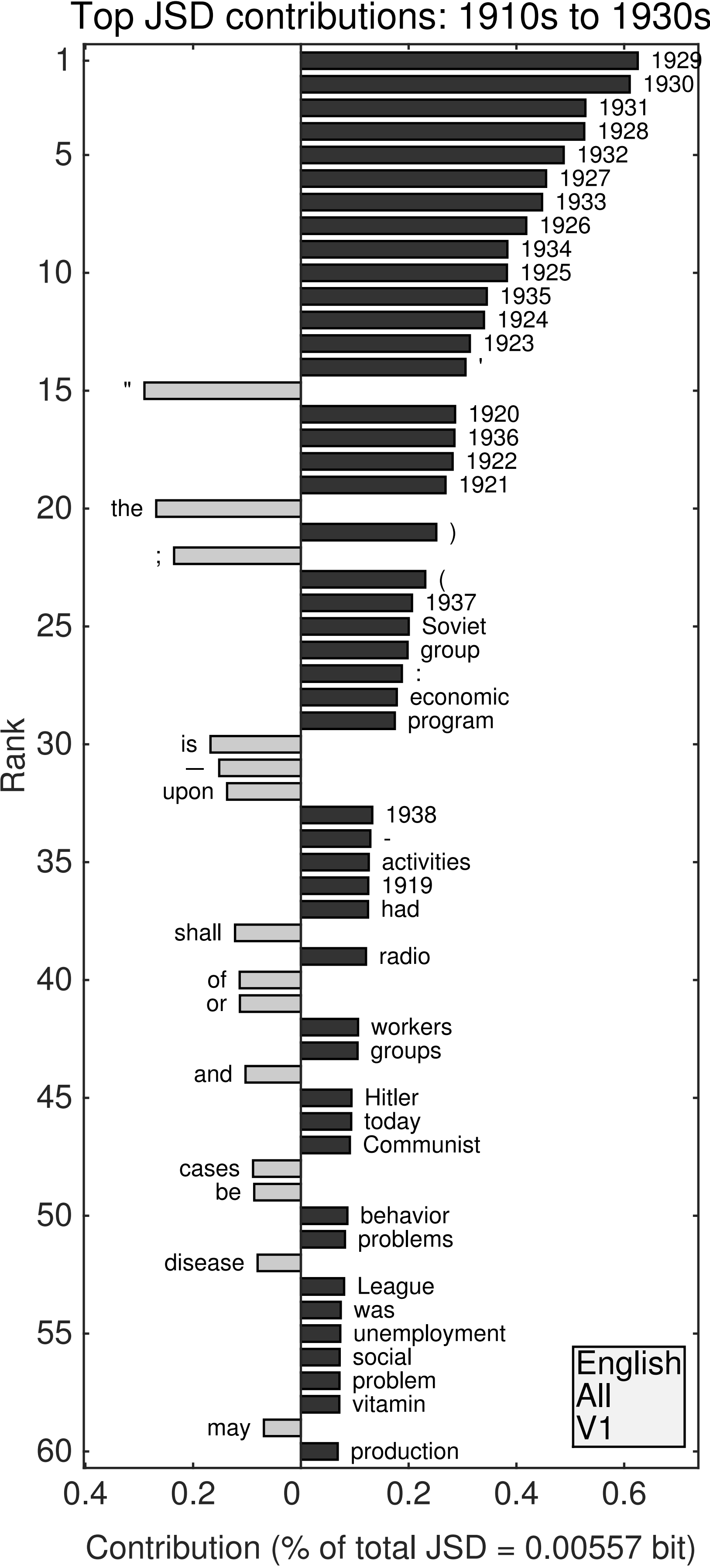
Top JSD contributions: 1910s to 1930s
Rank
Contribution (% of total JSD = 0.00557 bit)
1929
1930
1931
1928
1932
1927
1933
1926
1934
1925
1935
1924
1923
'
"
1920
1936
1922
1921
the
)
;
(
1937
Soviet
group
:
economic
program
is
—
upon
1938
-
activities
1919
had
shall
radio
of
or
workers
groups
and
Hitler
today
Communist
cases
be
behavior
problems
disease
League
was
unemployment
social
problem
vitamin
may
production
English
All
V1

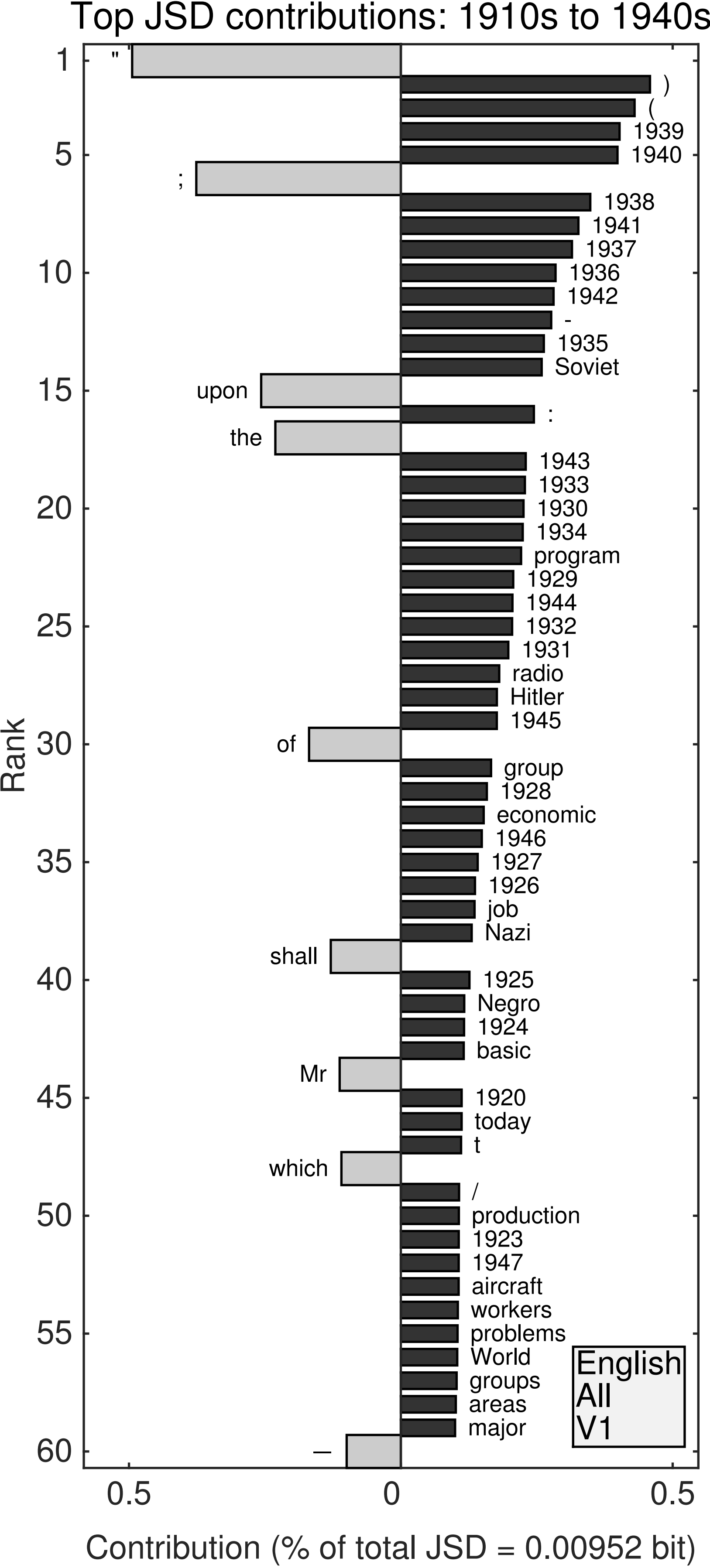

Top JSD contributions: 1910s to 1940s
Rank
Contribution (% of total JSD = 0.00952 bit)
English
All
V1
"
)
(
1939
1940
;
1938
1941
1937
1936
1942
-
1935
Soviet
upon
:
the
1943
1933
1930
1934
program
1929
1944
1932
1931
radio
Hitler
1945
of
group
1928
economic
1946
1927
1926
job
Nazi
shall
1925
Negro
1924
basic
Mr
1920
today
t
which
/
production
1923
1947
aircraft
workers
problems
World
groups
areas
major
—

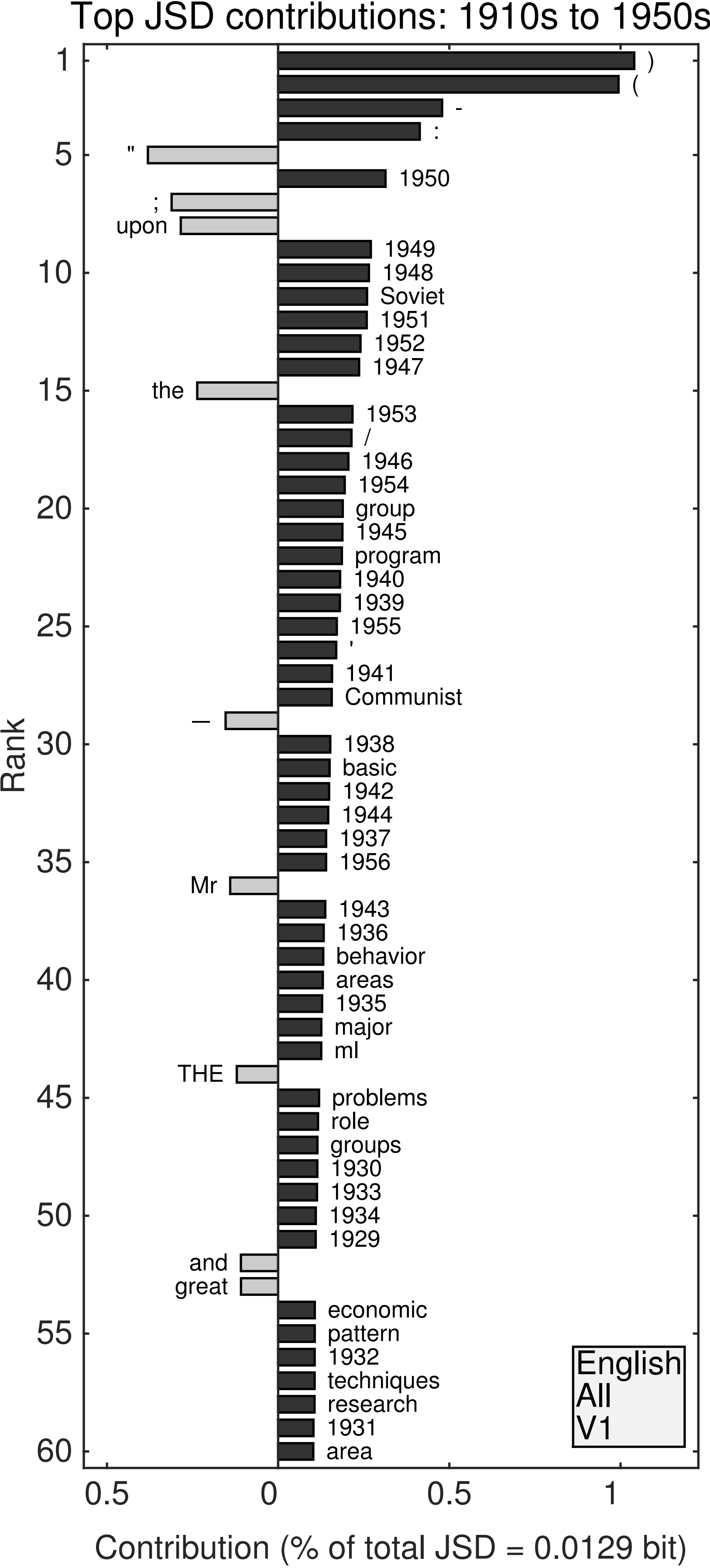
Top JSD contributions: 1910s to 1950s
Rank
Contribution (% of total JSD = 0.0129 bit)
English
All
V1
)
(
-
:
"
1950
;
upon
1949
1948
Soviet
1951
1952
1947
the
1953
/
1946
1954
group
1945
program
1940
1939
1955
'
1941
Communist
—
1938
basic
1942
1944
1937
1956
Mr
1943
1936
behavior
areas
1935
major
ml
THE
problems
role
groups
1930
1933
1934
1929
and
great
economic
pattern
1932
techniques
research
1931
area
1
5
10
15
20
25
30
35
40
45
50
55
60
0.5
0
0.5
1

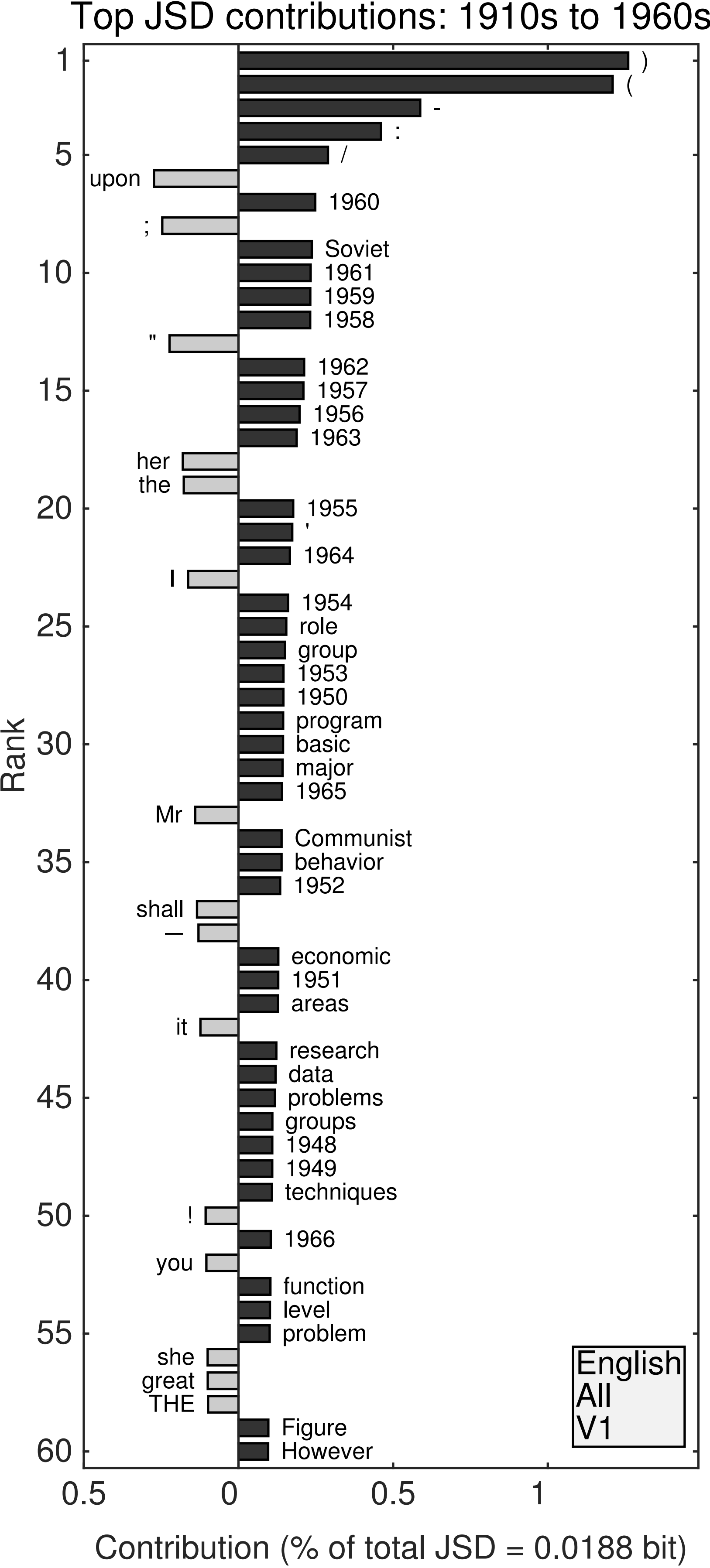

Top JSD contributions: 1910s to 1960s
Rank
Contribution (% of total JSD = 0.0188 bit)
)
(
-
:
/
upon
1960
;
Soviet
1961
1959
1958
"
1962
1957
1956
1963
her
the
1955
'
1964
I
1954
role
group
1953
1950
program
basic
major
1965
Mr
Communist
behavior
1952
shall
—
economic
1951
areas
it
research
data
problems
groups
1948
1949
techniques
!
1966
you
function
level
problem
she
great
THE
Figure
However
English
All
V1

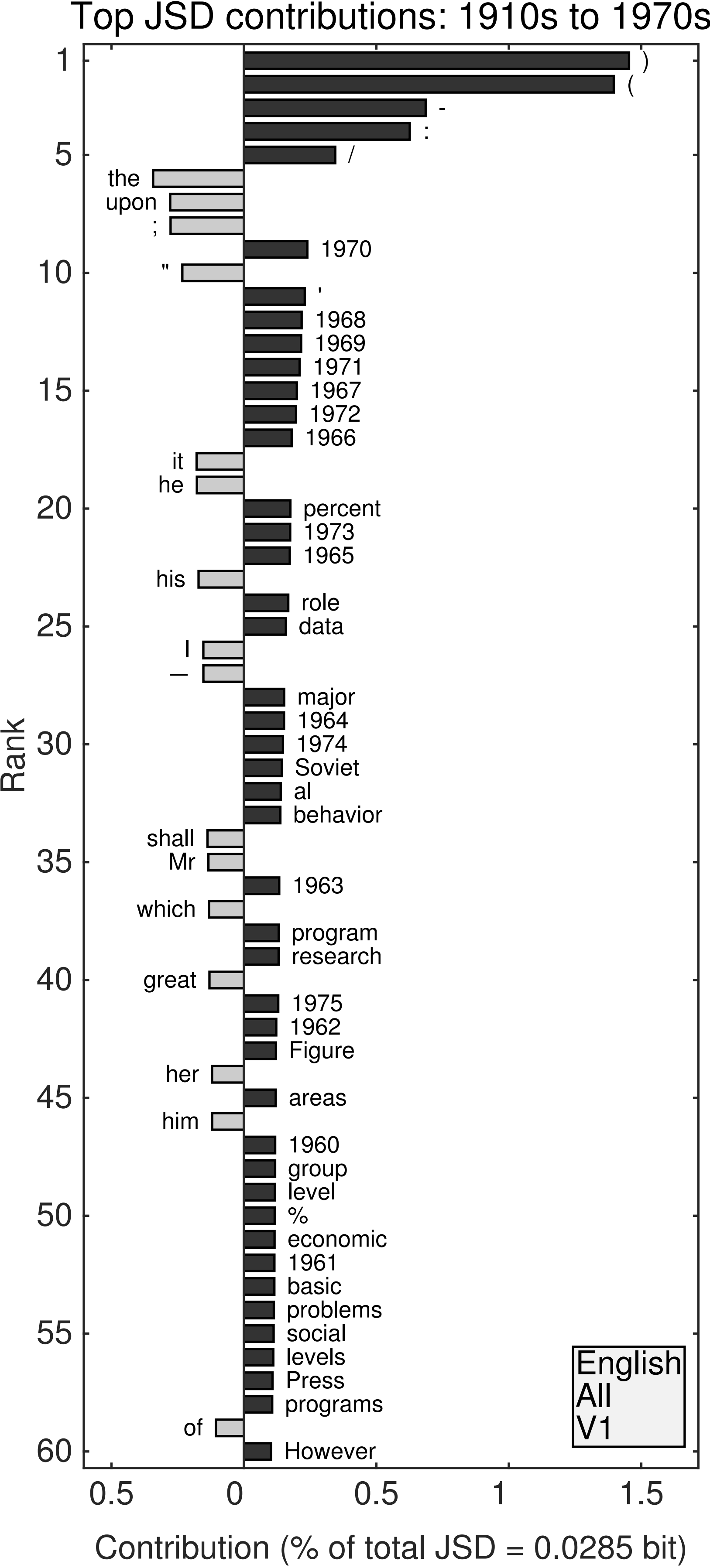
Top JSD contributions: 1910s to 1970s
Rank
Contribution (% of total JSD = 0.0285 bit)
)
(
-
:
/
the
upon
;
1970
"
'
1968
1969
1971
1967
1972
1966
it
he
percent
1973
1965
his
role
data
I
—
major
1964
1974
Soviet
al
behavior
shall
Mr
1963
which
program
research
great
1975
1962
Figure
her
areas
him
1960
group
level
%
economic
1961
basic
problems
social
levels
Press
programs
of
However
English
All
V1

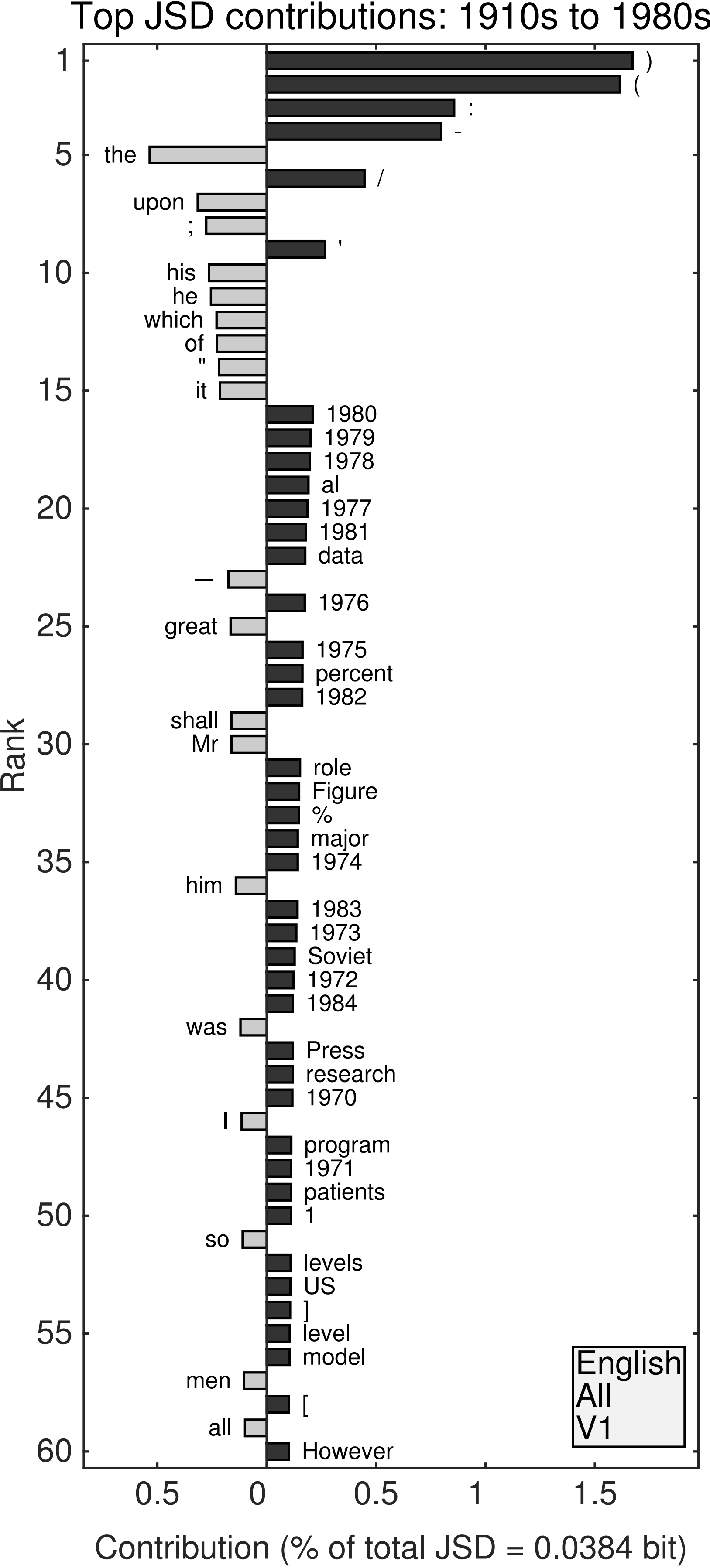
Top JSD contributions: 1910s to 1980s
Rank
Contribution (% of total JSD = 0.0384 bit)
)
(
:
-
the
/
upon
;
'
his
he
which
of
"
it
1980
1979
1978
al
1977
1981
data
—
1976
great
1975
percent
1982
shall
Mr
role
Figure
%
major
1974
him
1983
1973
Soviet
1972
1984
was
Press
research
1970
I
program
1971
patients
1
so
levels
US
]
level
model
men
[
all
However
English
All
V1

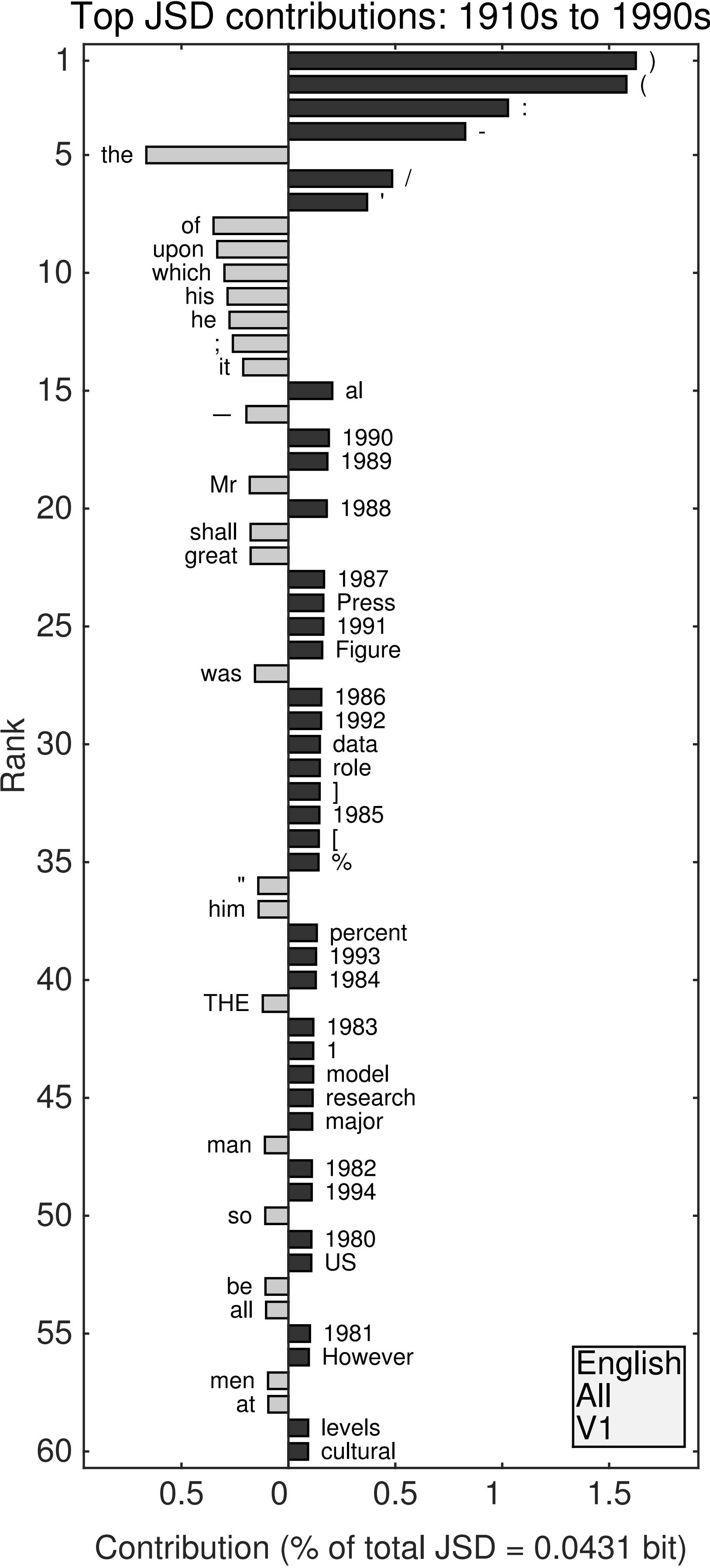
Top JSD contributions: 1910s to 1990s
Rank
Contribution (% of total JSD = 0.0431 bit)
)
(
:
-
the
/
'
of
upon
which
his
he
;
it
al
—
1990
1989
Mr
1988
shall
great
1987
Press
1991
Figure
was
1986
1992
data
role
]
1985
[
%
"
him
percent
1993
1984
THE
1983
1
model
research
major
man
1982
1994
so
1980
US
be
all
1981
However
men
at
levels
cultural
English
All
V1

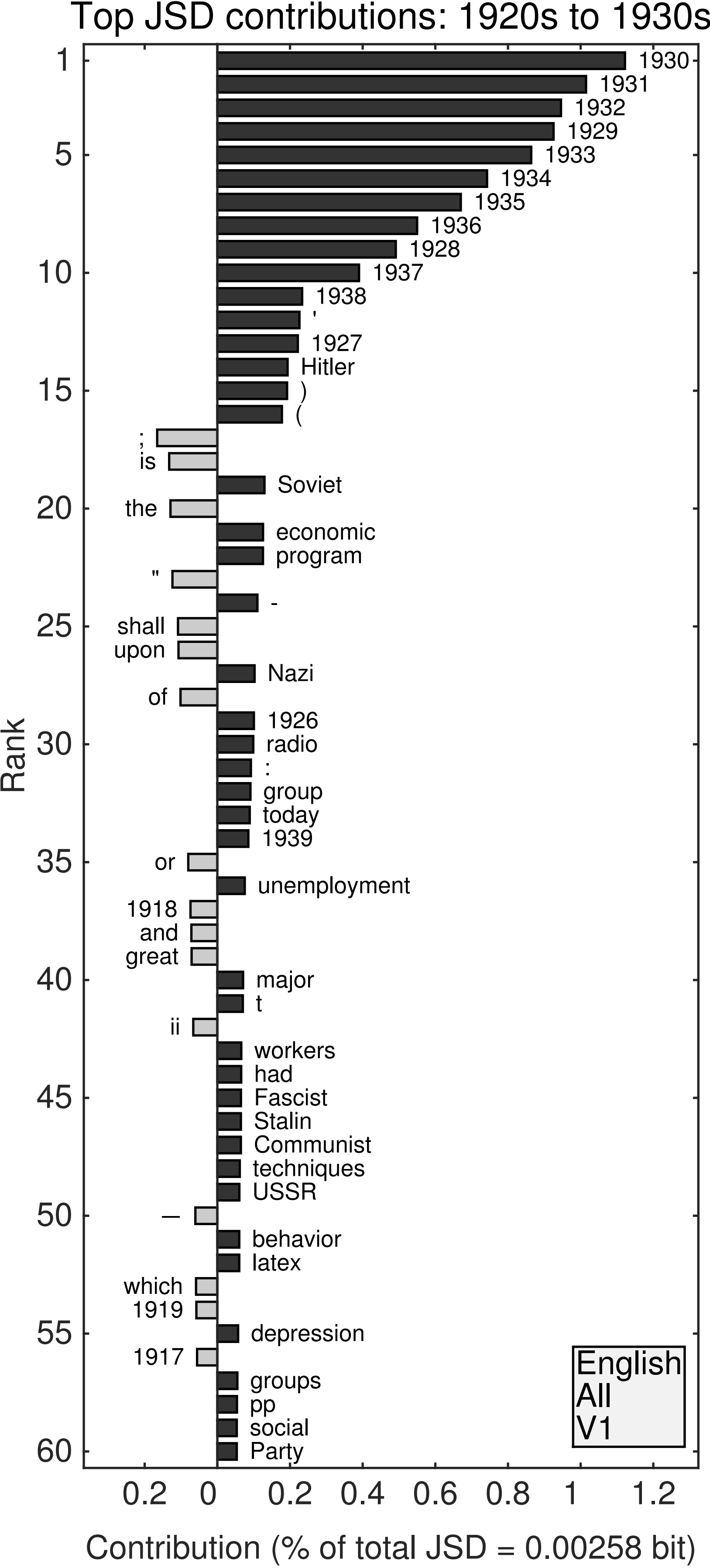

Top JSD contributions: 1920s to 1930s
Rank
Contribution (% of total JSD = 0.00258 bit)
1930
1931
1932
1929
1933
1934
1935
1936
1928
1937
1938
'
1927
Hitler
)
(
;
is
Soviet
the
economic
program
"
-
shall
upon
Nazi
of
1926
radio
:
group
today
1939
or
unemployment
1918
and
great
major
t
ii
workers
had
Fascist
Stalin
Communist
techniques
USSR
—
behavior
latex
which
1919
depression
1917
groups
pp
social
Party
English
All
V1

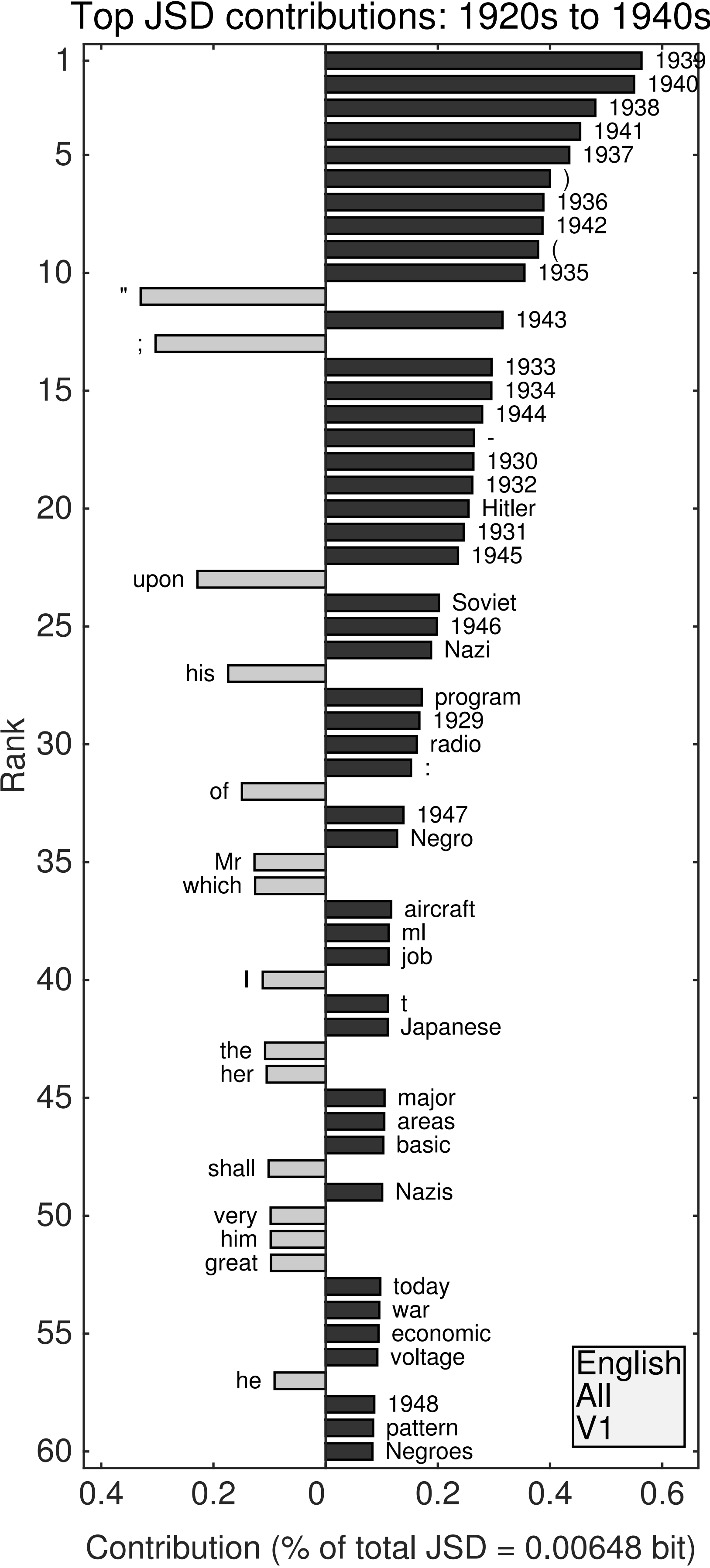
Top JSD contributions: 1920s to 1940s
Rank
Contribution (% of total JSD = 0.00648 bit)
1939
1940
1938
1941
1937
)
1936
1942
(
1935
"
1943
;
1933
1934
1944
-
1930
1932
Hitler
1931
1945
upon
Soviet
1946
Nazi
his
program
1929
radio
:
of
1947
Negro
Mr
which
aircraft
ml
job
I
t
Japanese
the
her
major
areas
basic
shall
Nazis
very
him
great
today
war
economic
voltage
he
1948
pattern
Negroes
English
All
V1

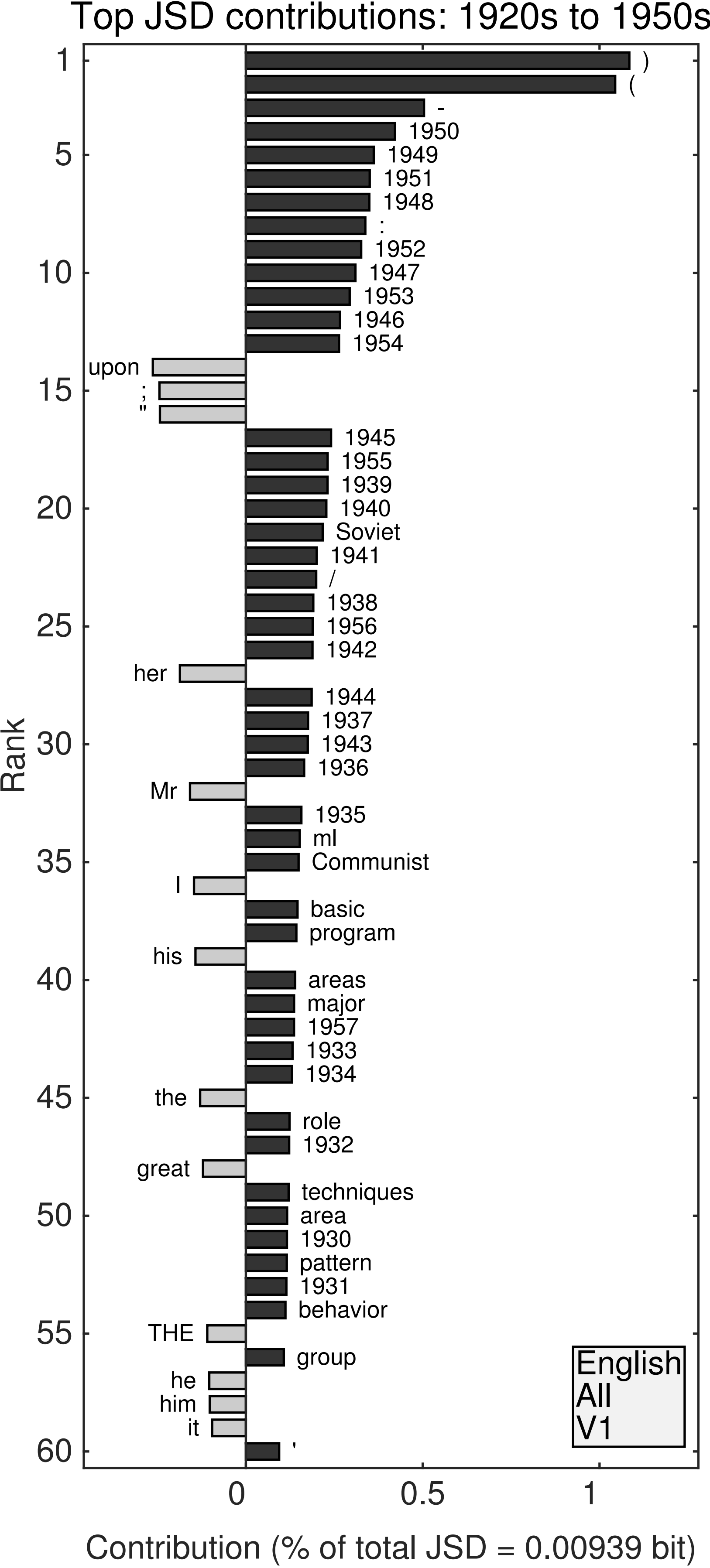
Top JSD contributions: 1920s to 1950s
Rank
Contribution (% of total JSD = 0.00939 bit)
0
0.5
1
1
5
10
15
20
25
30
35
40
45
50
55
60
)
(
-
1950
1949
1951
1948
:
1952
1947
1953
1946
1954
upon
;
"
1945
1955
1939
1940
Soviet
1941
/
1938
1956
1942
her
1944
1937
1943
1936
Mr
1935
ml
Communist
I
basic
program
his
areas
major
1957
1933
1934
the
role
1932
great
techniques
area
1930
pattern
1931
behavior
THE
group
he
him
it
'
English
All
V1

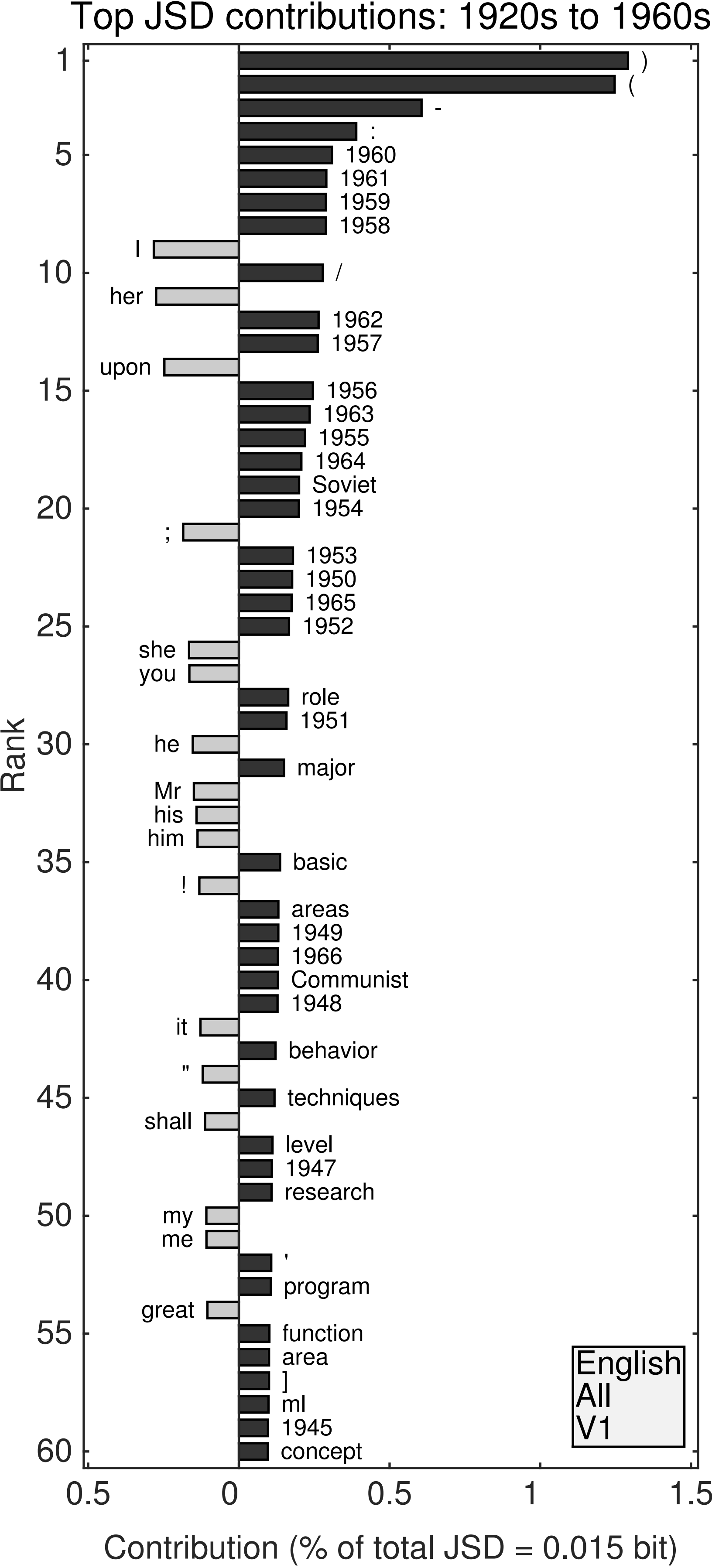

Top JSD contributions: 1920s to 1960s
Rank
Contribution (% of total JSD = 0.015 bit)
)
(
-
:
1960
1961
1959
1958
I
/
her
1962
1957
upon
1956
1963
1955
1964
Soviet
1954
;
1953
1950
1965
1952
she
you
role
1951
he
major
Mr
his
him
basic
!
areas
1949
1966
Communist
1948
it
behavior
"
techniques
shall
level
1947
research
my
me
'
program
great
function
area
]
ml
1945
concept
English
All
V1

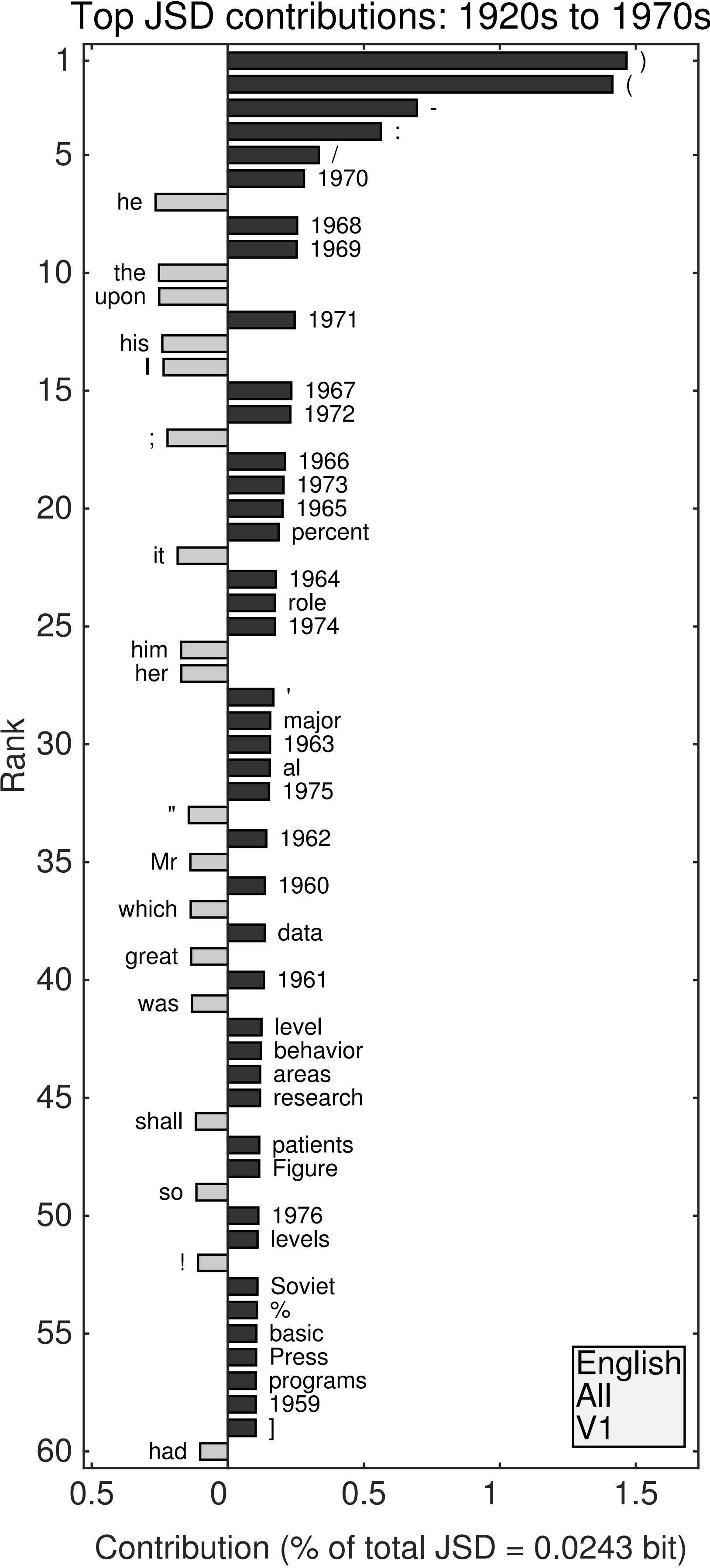

Top JSD contributions: 1920s to 1970s
Rank
Contribution (% of total JSD = 0.0243 bit)
0.5
0
0.5
1
1.5
1
5
10
15
20
25
30
35
40
45
50
55
60
)
(
-
:
/
1970
he
1968
1969
the
upon
1971
his
I
1967
1972
;
1966
1973
1965
percent
it
1964
role
1974
him
her
'
major
1963
al
1975
"
1962
Mr
1960
which
data
great
1961
was
level
behavior
areas
research
shall
patients
Figure
so
1976
levels
!
Soviet
%
basic
Press
programs
1959
]
had
English
All
V1

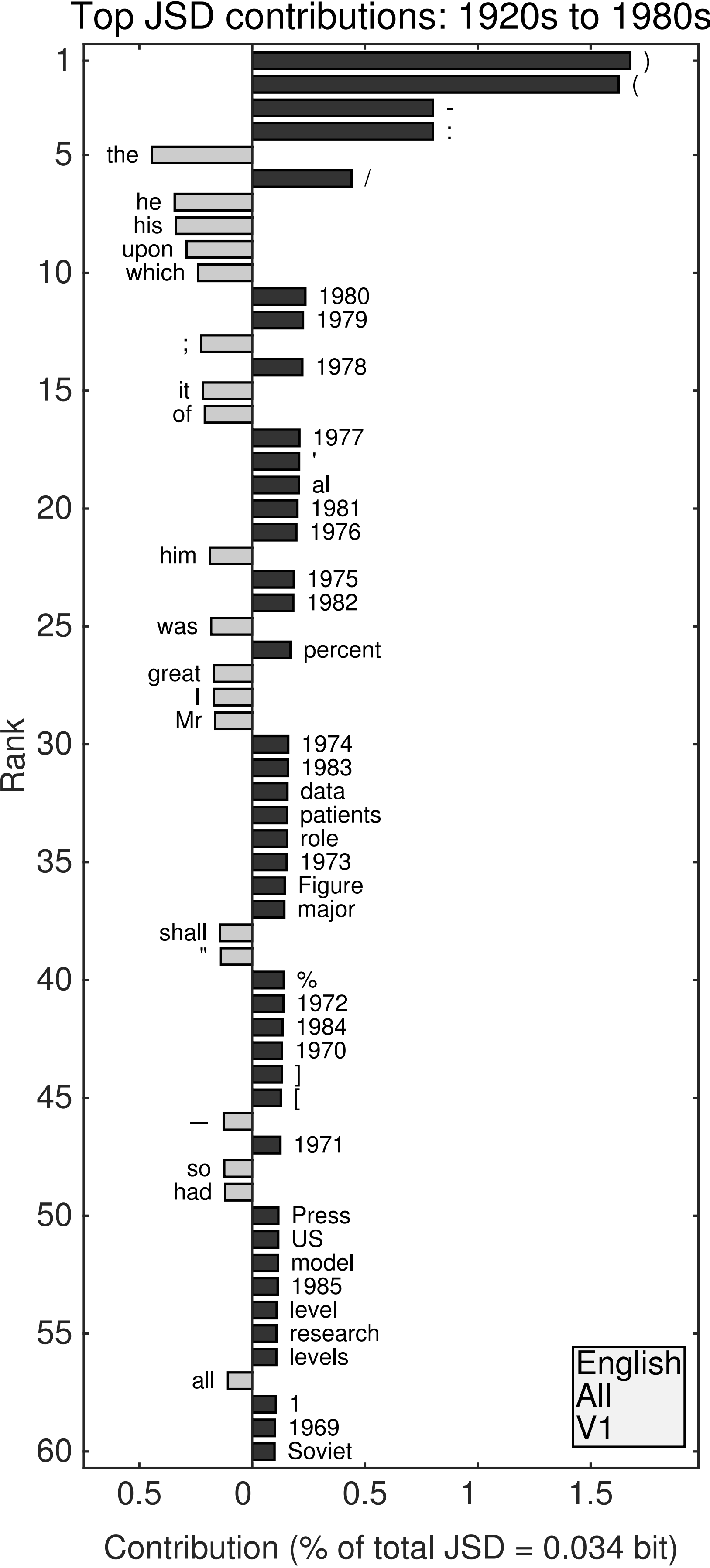
Top JSD contributions: 1920s to 1980s
Rank
Contribution (% of total JSD = 0.034 bit)
English
All
V1
)
(
-
:
the
/
he
his
upon
which
1980
1979
;
1978
it
of
1977
'
al
1981
1976
him
1975
1982
was
percent
great
I
Mr
1974
1983
data
patients
role
1973
Figure
major
shall
"
%
1972
1984
1970
]
[
—
1971
so
had
Press
US
model
1985
level
research
levels
all
1
1969
Soviet

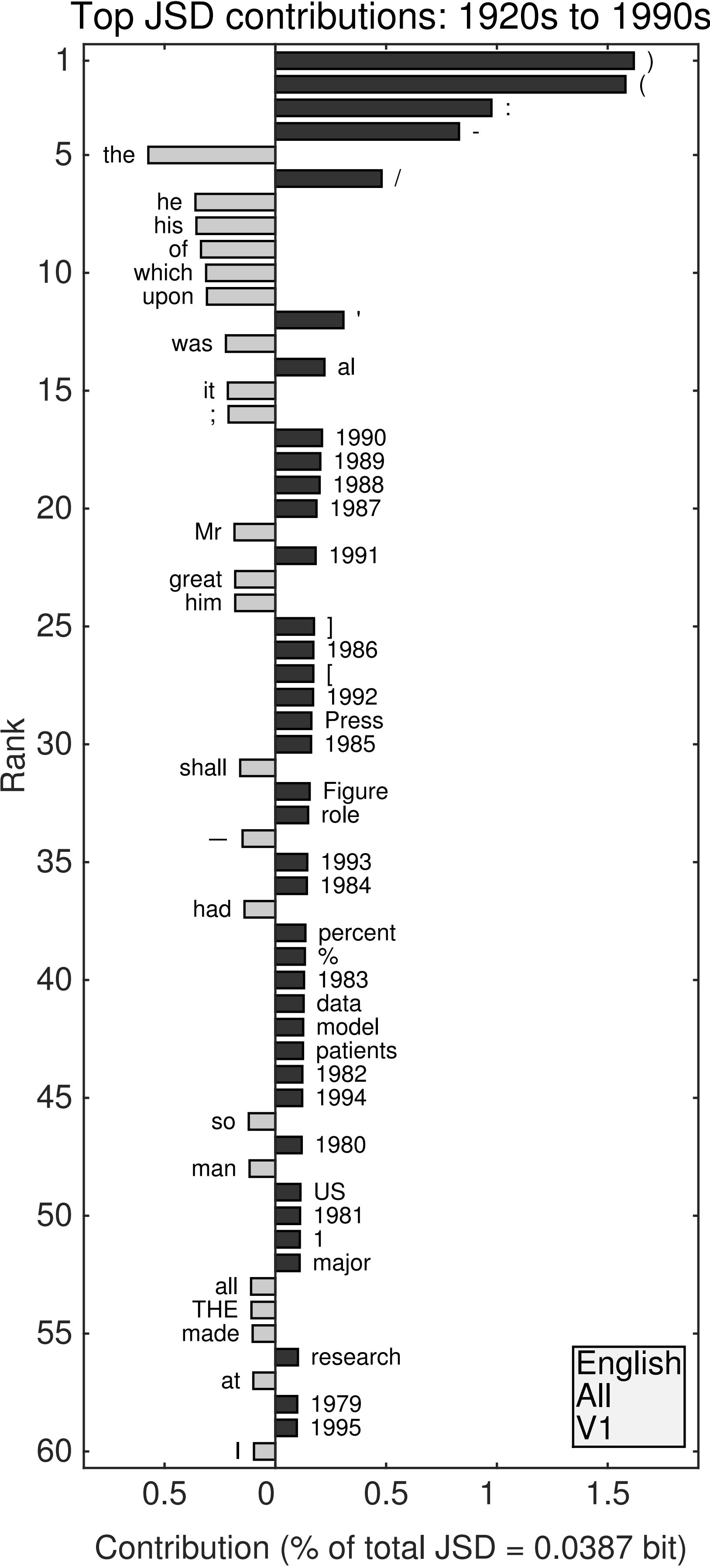
Top JSD contributions: 1920s to 1990s
Rank
Contribution (% of total JSD = 0.0387 bit)
English
All
V1
)
(
:
-
the
/
he
his
of
which
upon
'
was
al
it
;
1990
1989
1988
1987
Mr
1991
great
him
]
1986
[
1992
Press
1985
shall
Figure
role
—
1993
1984
had
percent
%
1983
data
model
patients
1982
1994
so
1980
man
US
1981
1
major
all
THE
made
research
at
1979
1995
I

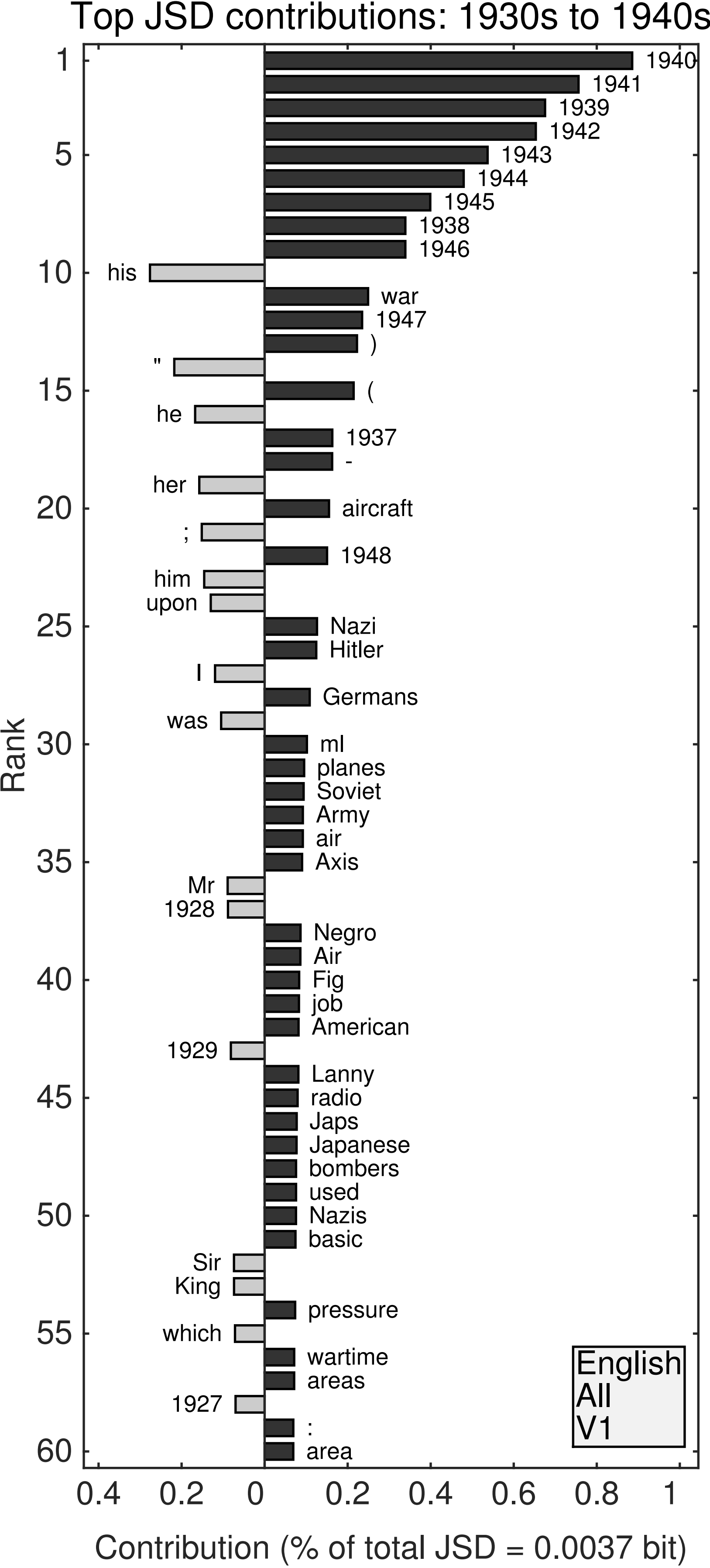

Top JSD contributions: 1930s to 1940s
Rank
Contribution (% of total JSD = 0.0037 bit)
1940
1941
1939
1942
1943
1944
1945
1938
1946
his
war
1947
)
"
(
he
1937
-
her
aircraft
;
1948
him
upon
Nazi
Hitler
I
Germans
was
ml
planes
Soviet
Army
air
Axis
Mr
1928
Negro
Air
Fig
job
American
1929
Lanny
radio
Japs
Japanese
bombers
used
Nazis
basic
Sir
King
pressure
which
wartime
areas
1927
:
area
English
All
V1

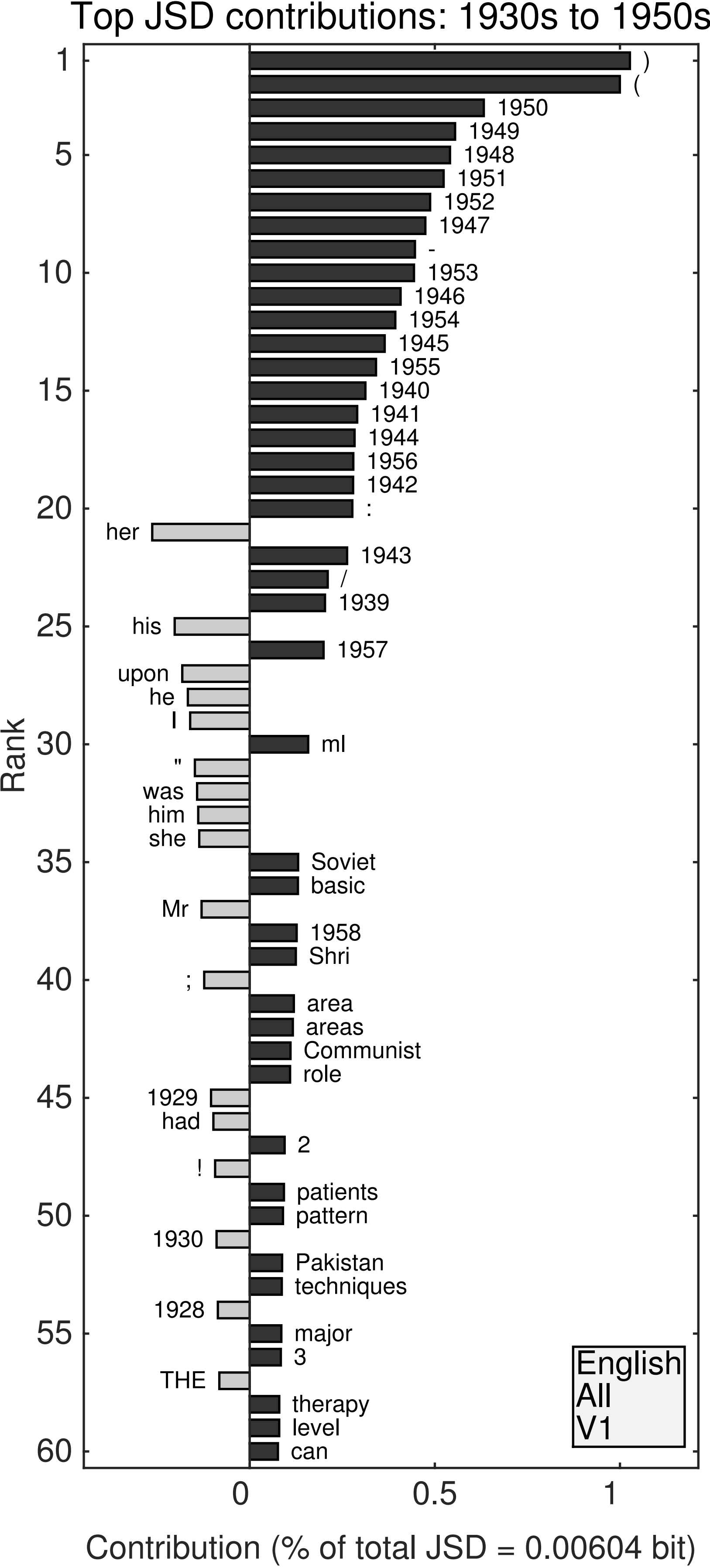

Top JSD contributions: 1930s to 1950s
Rank
Contribution (% of total JSD = 0.00604 bit)
)
(
1950
1949
1948
1951
1952
1947
-
1953
1946
1954
1945
1955
1940
1941
1944
1956
1942
:
her
1943
/
1939
his
1957
upon
he
I
ml
"
was
him
she
Soviet
basic
Mr
1958
Shri
;
area
areas
Communist
role
1929
had
2
!
patients
pattern
1930
Pakistan
techniques
1928
major
3
THE
therapy
level
can
English
All
V1

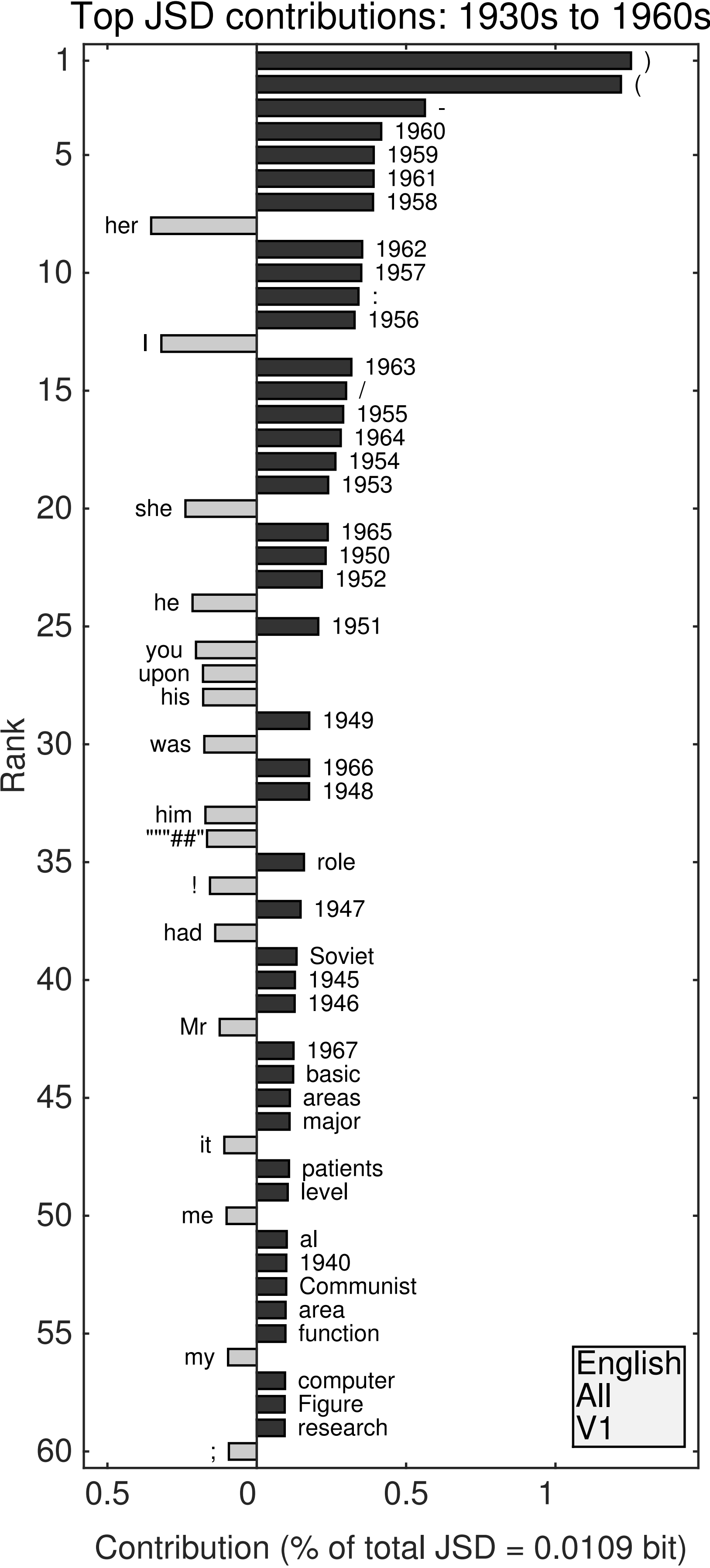

Top JSD contributions: 1930s to 1960s
Rank
Contribution (% of total JSD = 0.0109 bit)
)
(
-
1960
1959
1961
1958
her
1962
1957
:
1956
I
1963
/
1955
1964
1954
1953
she
1965
1950
1952
he
1951
you
upon
his
1949
was
1966
1948
him
"""##"
role
!
1947
had
Soviet
1945
1946
Mr
1967
basic
areas
major
it
patients
level
me
al
1940
Communist
area
function
my
computer
Figure
research
;
English
All
V1

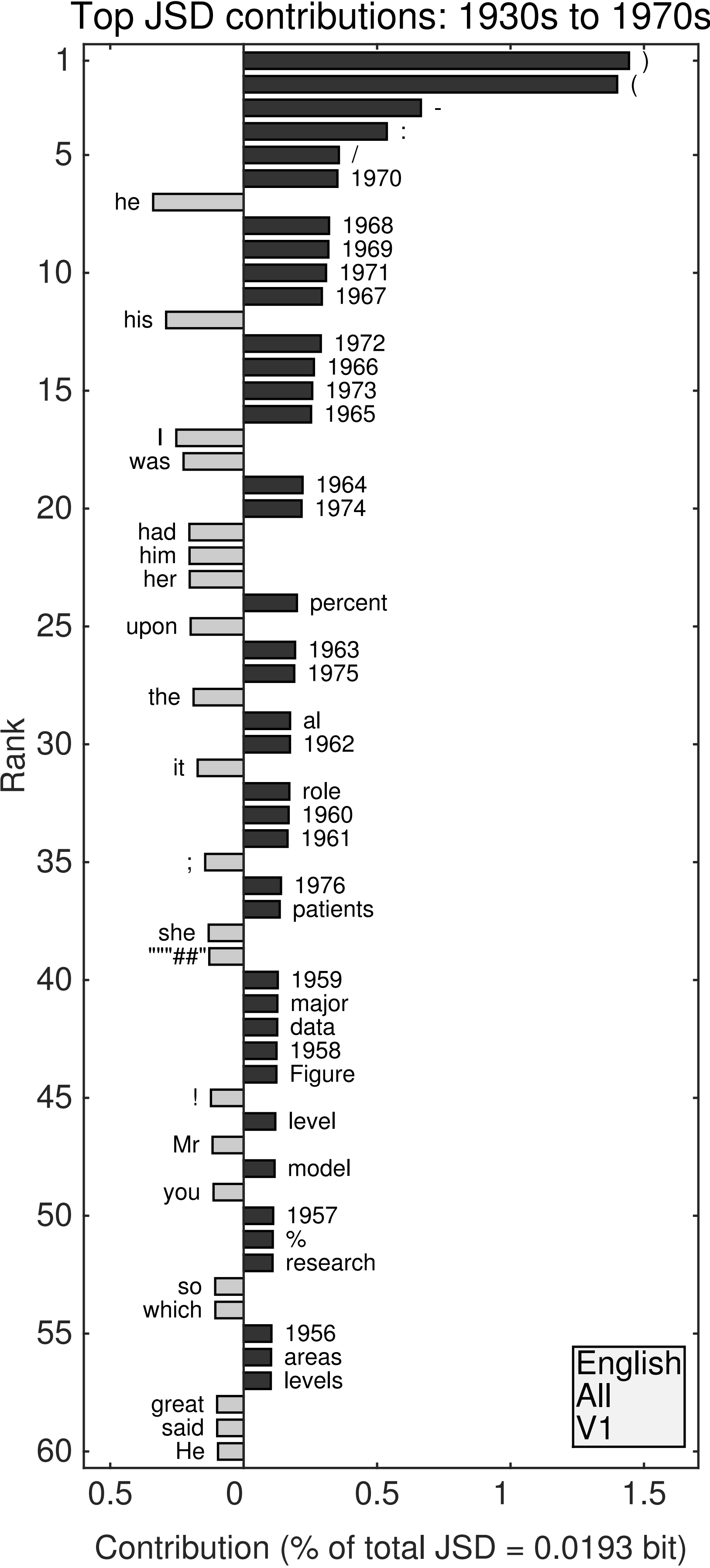
Top JSD contributions: 1930s to 1970s
Rank
Contribution (% of total JSD = 0.0193 bit)
)
(
-
:
/
1970
he
1968
1969
1971
1967
his
1972
1966
1973
1965
I
was
1964
1974
had
him
her
percent
upon
1963
1975
the
al
1962
it
role
1960
1961
;
1976
patients
she
"""##"
1959
major
data
1958
Figure
!
level
Mr
model
you
1957
%
research
so
which
1956
areas
levels
great
said
He
English
All
V1

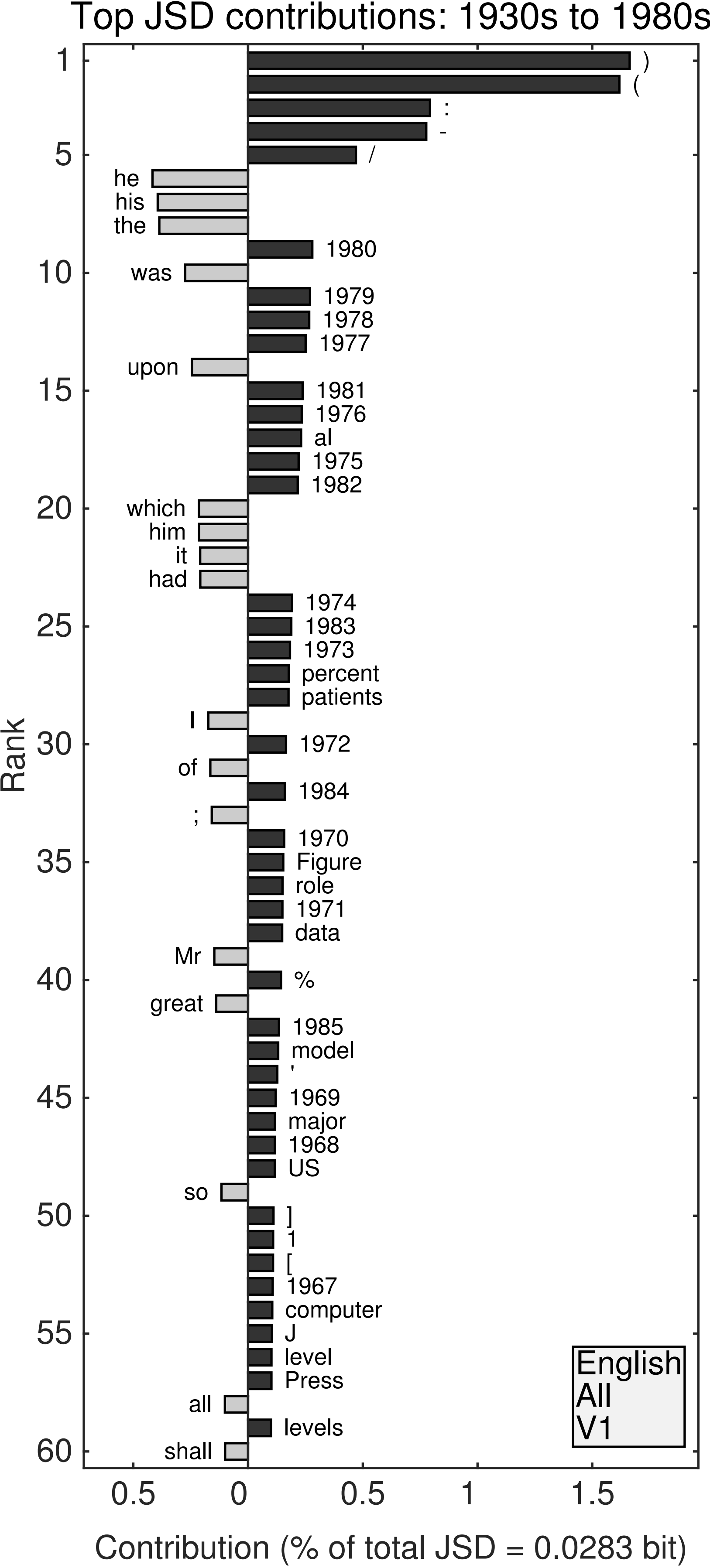

Top JSD contributions: 1930s to 1980s
Rank
Contribution (% of total JSD = 0.0283 bit)
)
(
:
-
/
he
his
the
1980
was
1979
1978
1977
upon
1981
1976
al
1975
1982
which
him
it
had
1974
1983
1973
percent
patients
I
1972
of
1984
;
1970
Figure
role
1971
data
Mr
%
great
1985
model
'
1969
major
1968
US
so
]
1
[
1967
computer
J
level
Press
all
levels
shall
English
All
V1

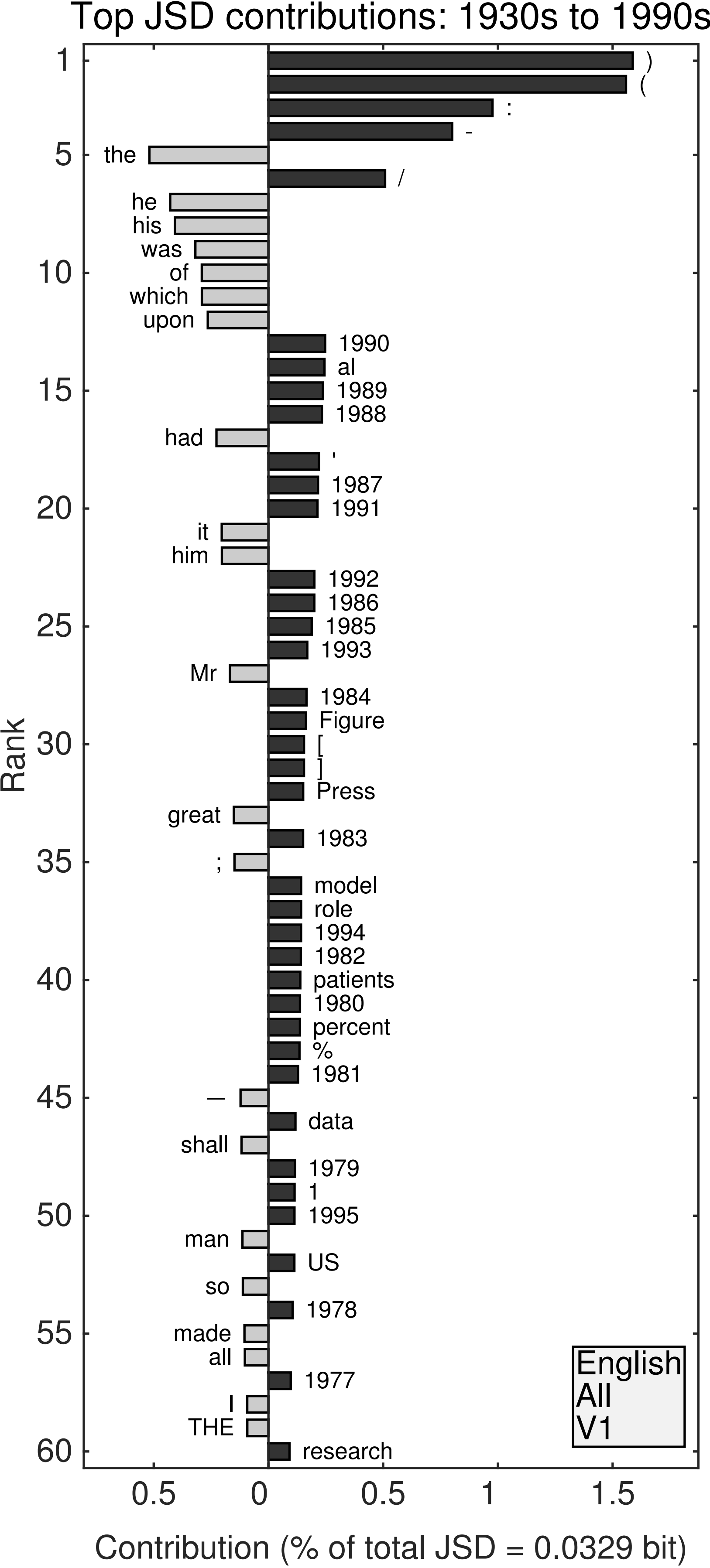
Top JSD contributions: 1930s to 1990s
Rank
Contribution (% of total JSD = 0.0329 bit)
English
All
V1
)
(
:
-
the
/
he
his
was
of
which
upon
1990
al
1989
1988
had
'
1987
1991
it
him
1992
1986
1985
1993
Mr
1984
Figure
[
]
Press
great
1983
;
model
role
1994
1982
patients
1980
percent
%
1981
—
data
shall
1979
1
1995
man
US
so
1978
made
all
1977
I
THE
research

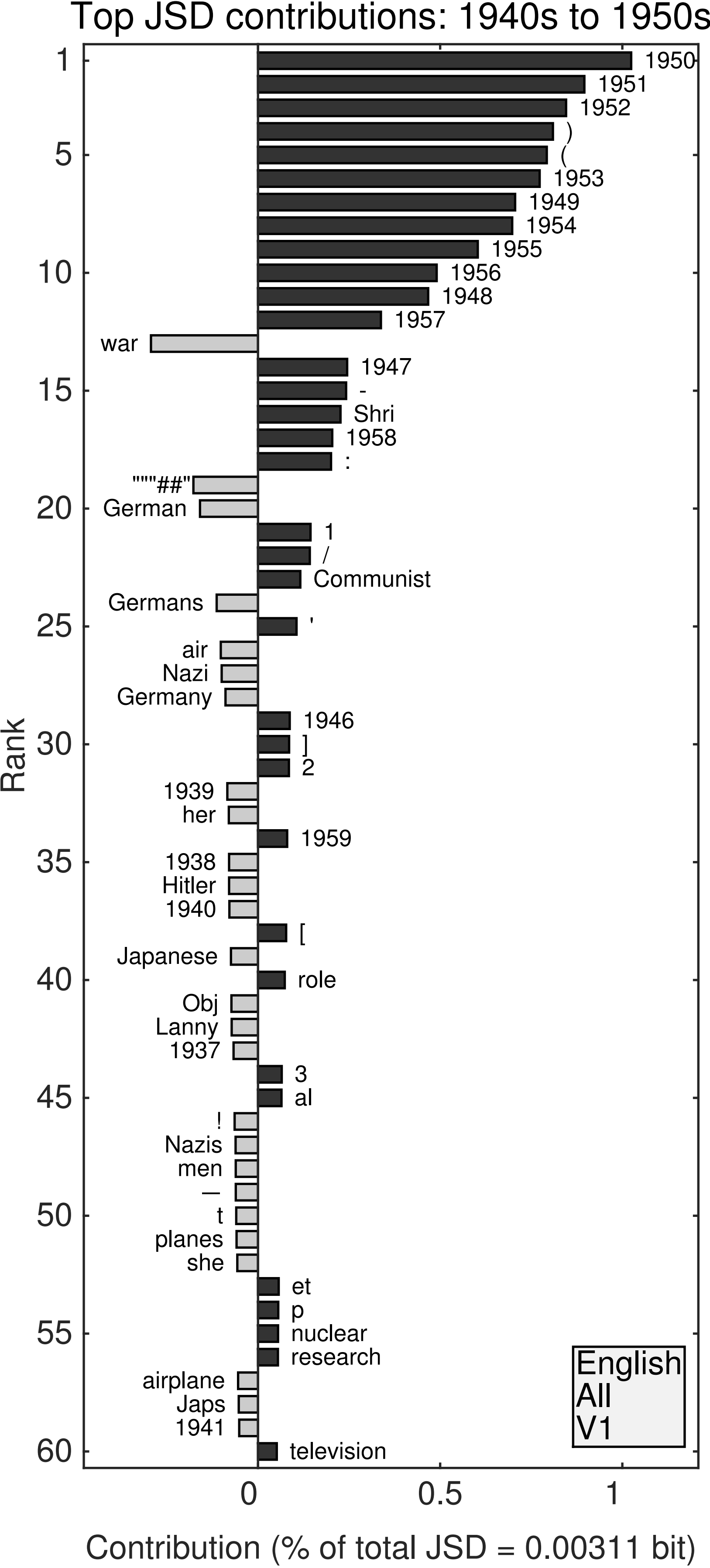

Top JSD contributions: 1940s to 1950s
Rank
1
5
10
15
20
25
30
35
40
45
50
55
60
1950
1951
1952
)
(
1953
1949
1954
1955
1956
1948
1957
war
1947
-
Shri
1958
:
"""##"
German
1
/
Communist
Germans
'
air
Nazi
Germany
1946
]
2
1939
her
1959
1938
Hitler
1940
[
Japanese
role
Obj
Lanny
1937
3
al
!
Nazis
men
—
t
planes
she
et
p
nuclear
research
airplane
Japs
1941
television
English
All
V1
0
0.5
1
Contribution (% of total JSD = 0.00311 bit)

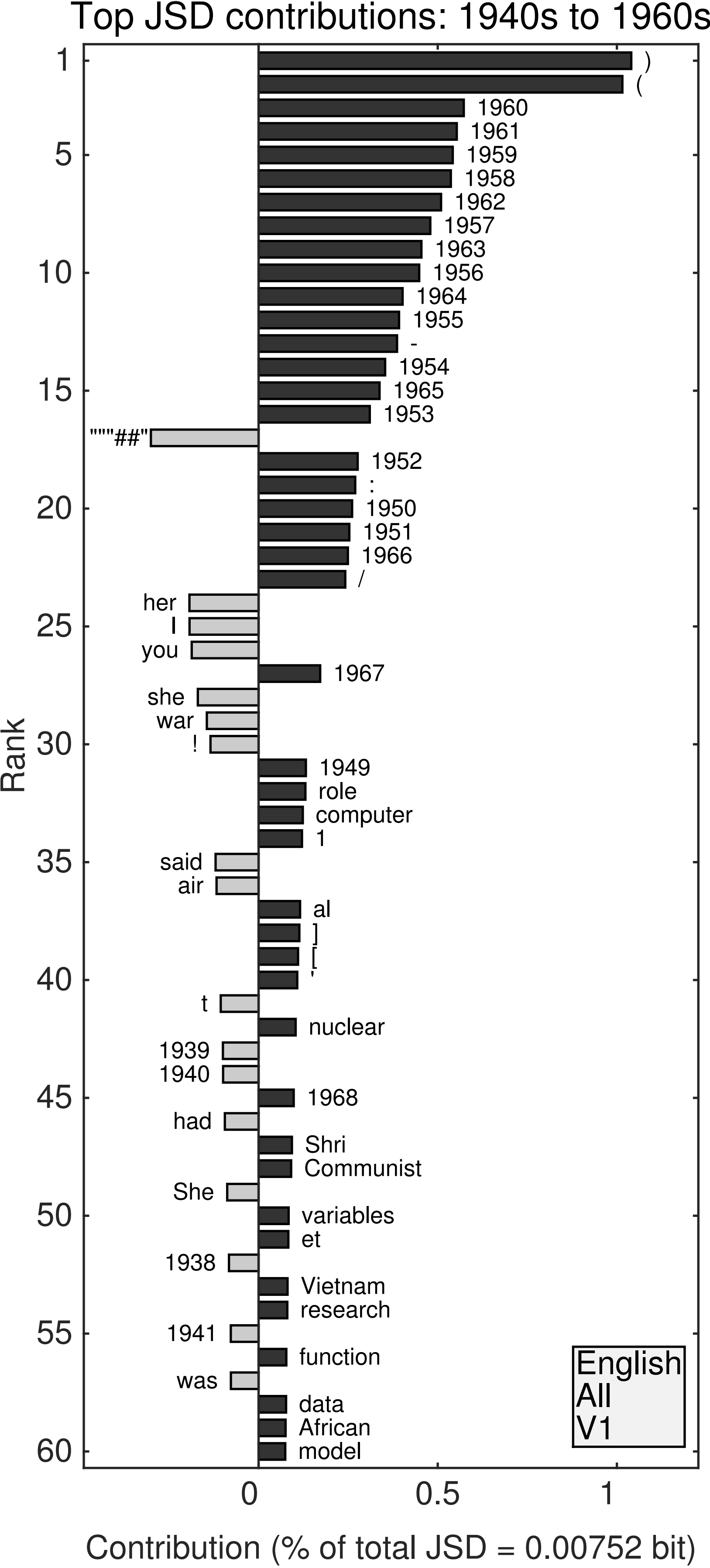
Top JSD contributions: 1940s to 1960s
Rank
Contribution (% of total JSD = 0.00752 bit)
)
(
1960
1961
1959
1958
1962
1957
1963
1956
1964
1955
-
1954
1965
1953
"""##"
1952
:
1950
1951
1966
/
her
I
you
1967
she
war
!
1949
role
computer
1
said
air
al
]
[
'
t
nuclear
1939
1940
1968
had
Shri
Communist
She
variables
et
1938
Vietnam
research
1941
function
was
data
African
model
English
All
V1

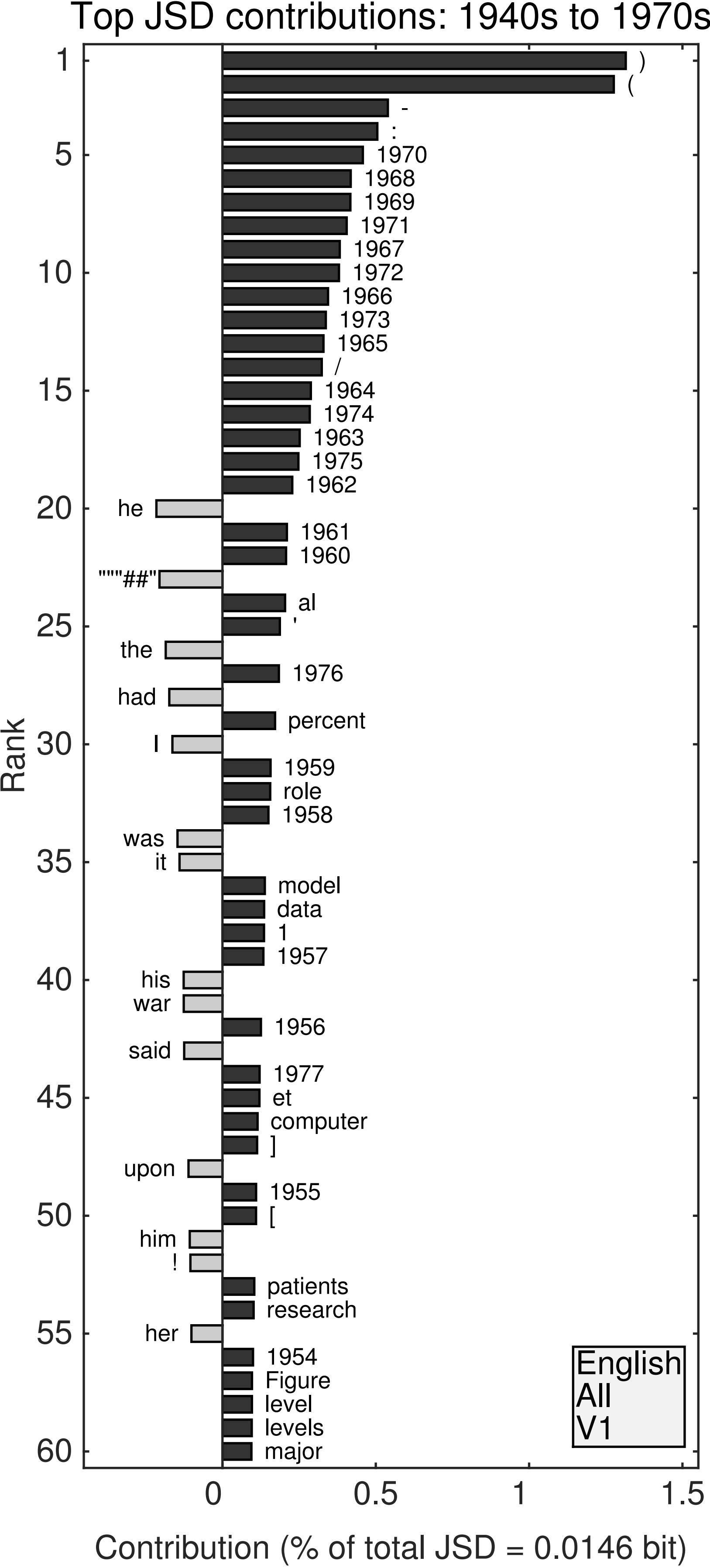

Top JSD contributions: 1940s to 1970s
Rank
Contribution (% of total JSD = 0.0146 bit)
)
(
-
:
1970
1968
1969
1971
1967
1972
1966
1973
1965
/
1964
1974
1963
1975
1962
he
1961
1960
"""##"
al
'
the
1976
had
percent
I
1959
role
1958
was
it
model
data
1
1957
his
war
1956
said
1977
et
computer
]
upon
1955
[
him
!
patients
research
her
1954
Figure
level
levels
major
English
All
V1

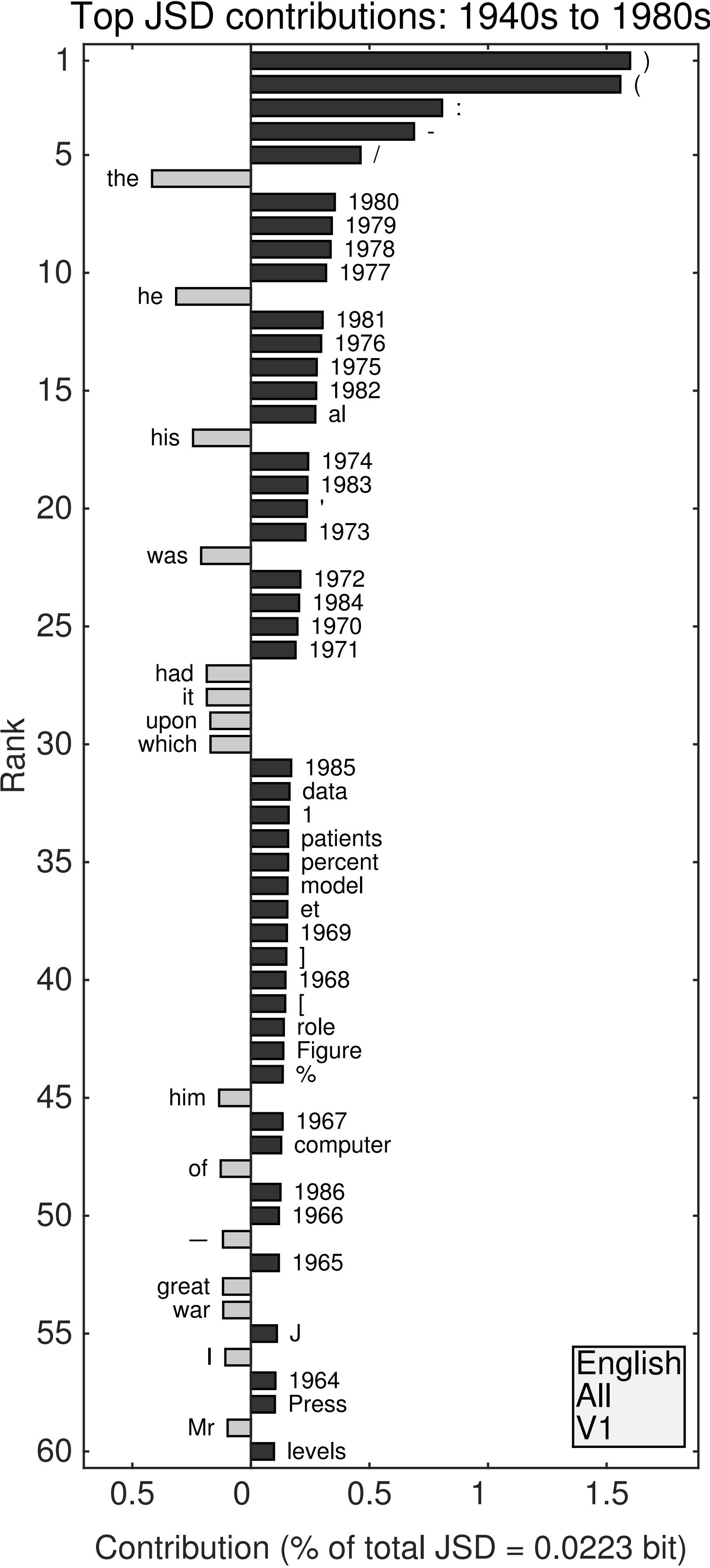
Top JSD contributions: 1940s to 1980s
Rank
Contribution (% of total JSD = 0.0223 bit)
English
All
V1
)
(
:
-
/
the
1980
1979
1978
1977
he
1981
1976
1975
1982
al
his
1974
1983
'
1973
was
1972
1984
1970
1971
had
it
upon
which
1985
data
1
patients
percent
model
et
1969
]
1968
[
role
Figure
%
him
1967
computer
of
1986
1966
—
1965
great
war
J
I
1964
Press
Mr
levels

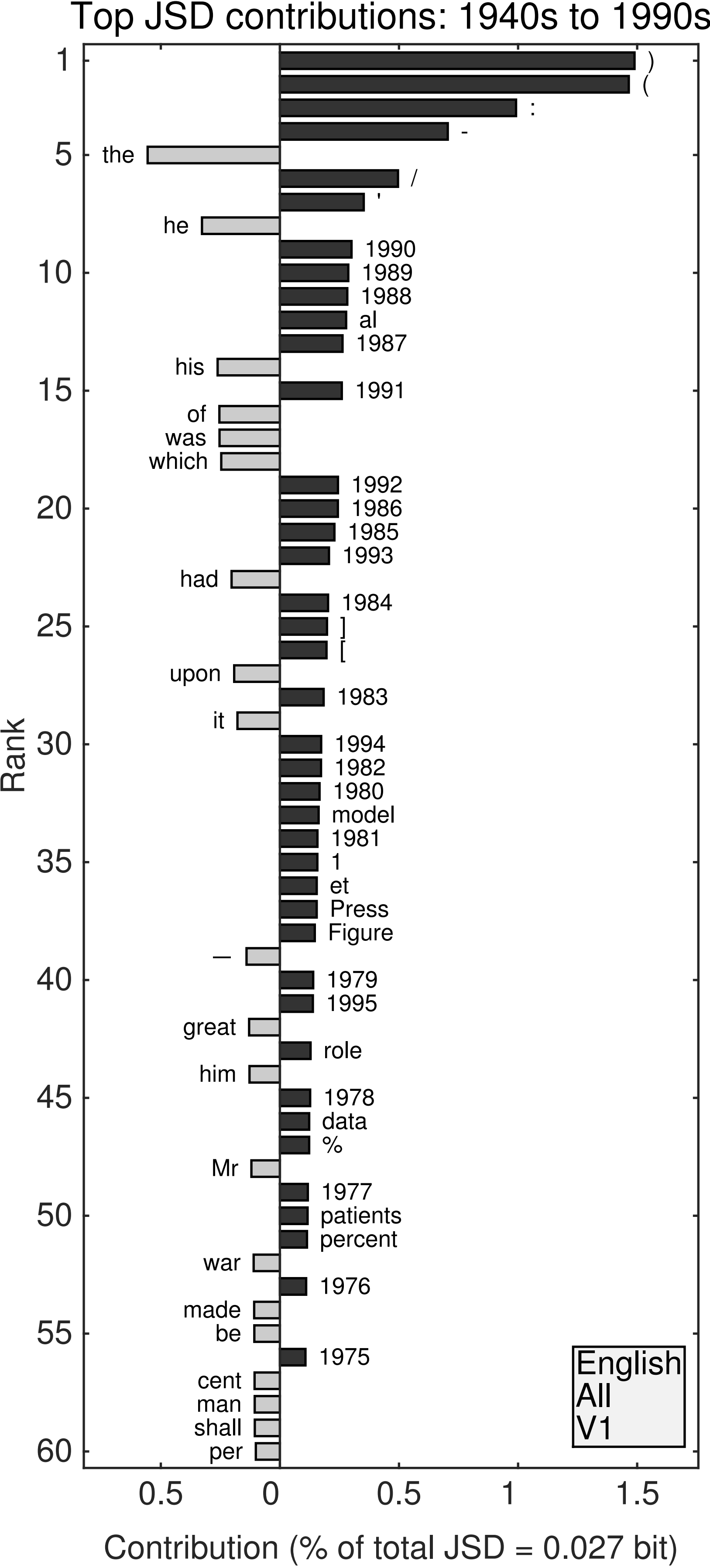
Top JSD contributions: 1940s to 1990s
Rank
Contribution (% of total JSD = 0.027 bit)
)
(
:
-
the
/
'
he
1990
1989
1988
al
1987
his
1991
of
was
which
1992
1986
1985
1993
had
1984
]
[
upon
1983
it
1994
1982
1980
model
1981
1
et
Press
Figure
—
1979
1995
great
role
him
1978
data
%
Mr
1977
patients
percent
war
1976
made
be
1975
cent
man
shall
per
English
All
V1

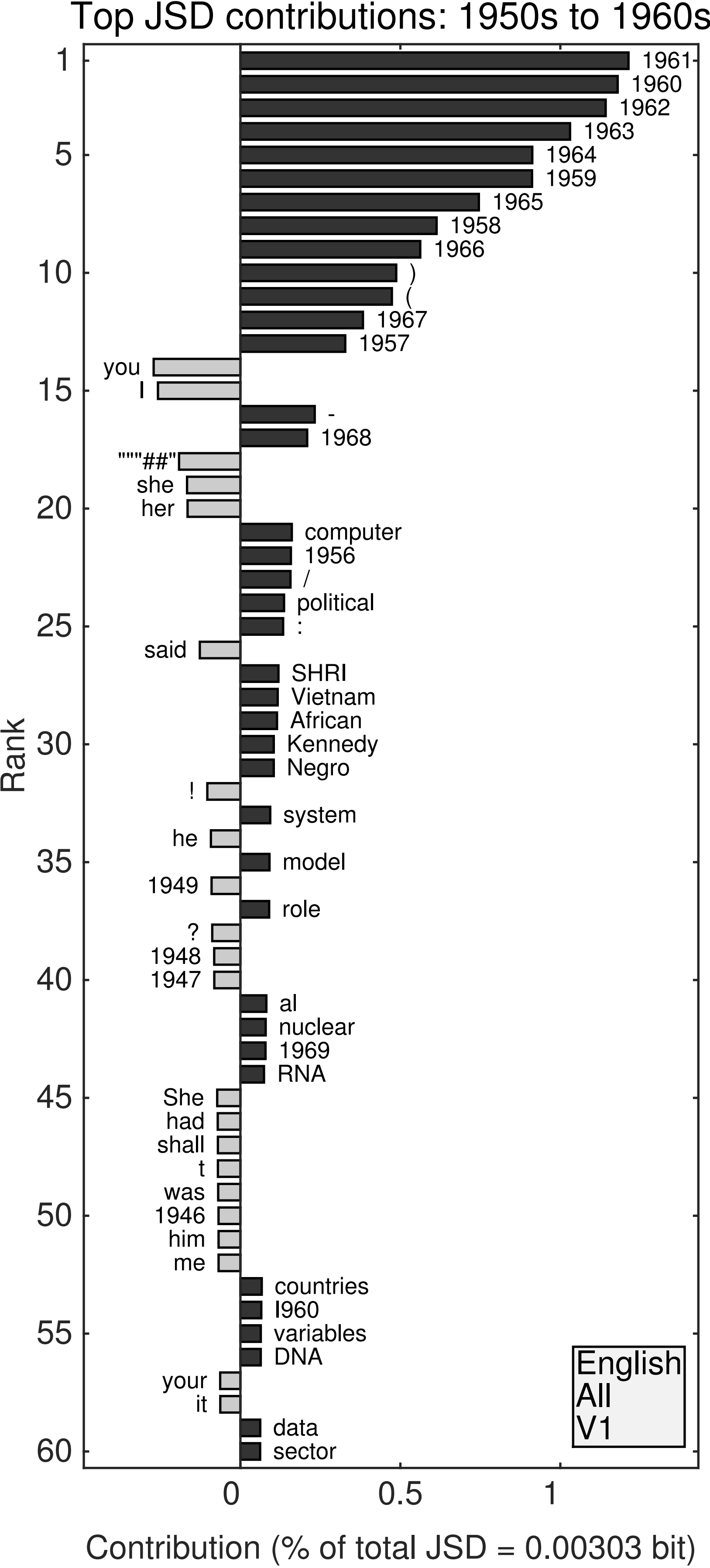
Top JSD contributions: 1950s to 1960s
Rank
Contribution (% of total JSD = 0.00303 bit)
1961
1960
1962
1963
1964
1959
1965
1958
1966
)
(
1967
1957
you
I
-
1968
"""##"
she
her
computer
1956
/
political
:
said
SHRI
Vietnam
African
Kennedy
Negro
!
system
he
model
1949
role
?
1948
1947
al
nuclear
1969
RNA
She
had
shall
t
was
1946
him
me
countries
I960
variables
DNA
your
it
data
sector
English
All
V1
1
5
10
15
20
25
30
35
40
45
50
55
60
0
0.5
1

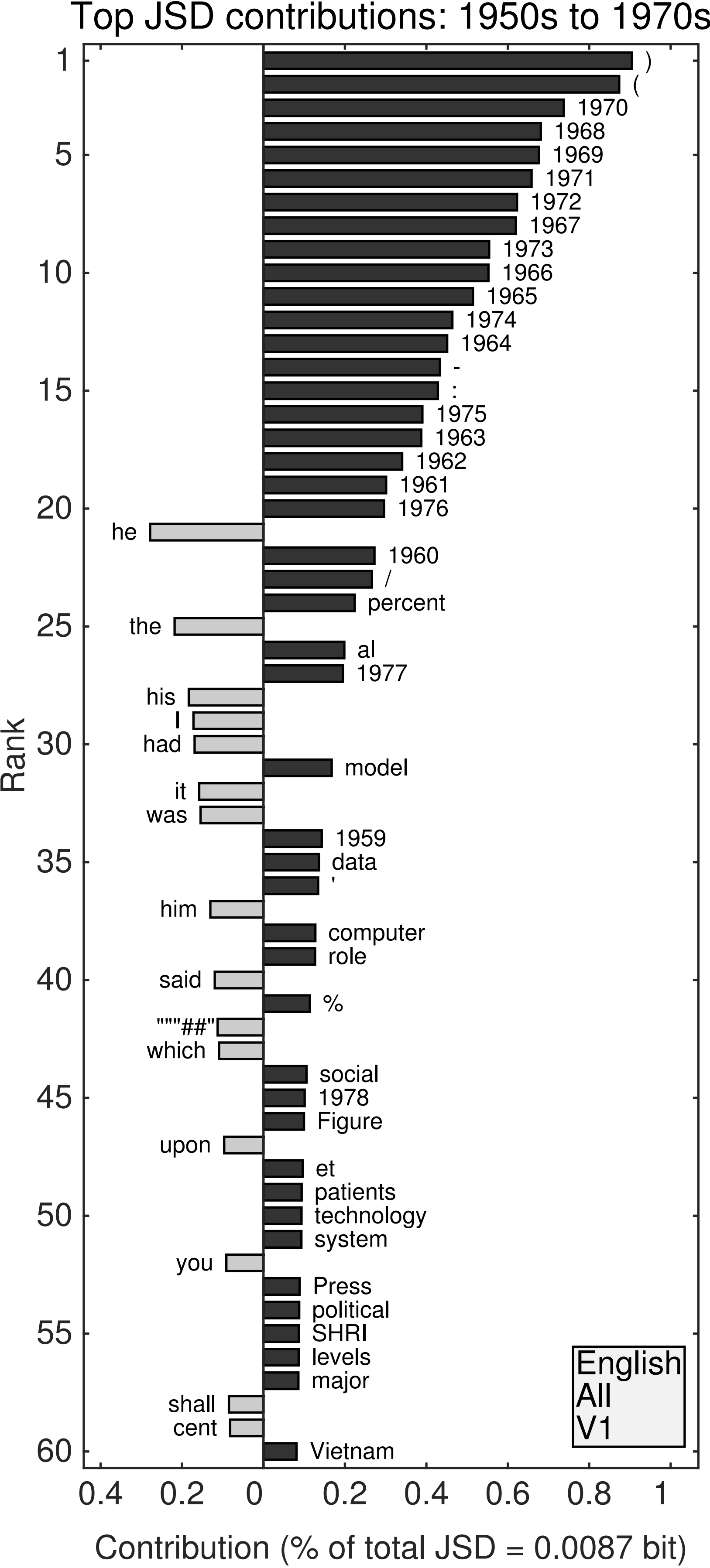

Top JSD contributions: 1950s to 1970s
Rank
Contribution (% of total JSD = 0.0087 bit)
)
(
1970
1968
1969
1971
1972
1967
1973
1966
1965
1974
1964
-
:
1975
1963
1962
1961
1976
he
1960
/
percent
the
al
1977
his
I
had
model
it
was
1959
data
'
him
computer
role
said
%
"""##"
which
social
1978
Figure
upon
et
patients
technology
system
you
Press
political
SHRI
levels
major
shall
cent
Vietnam
English
All
V1

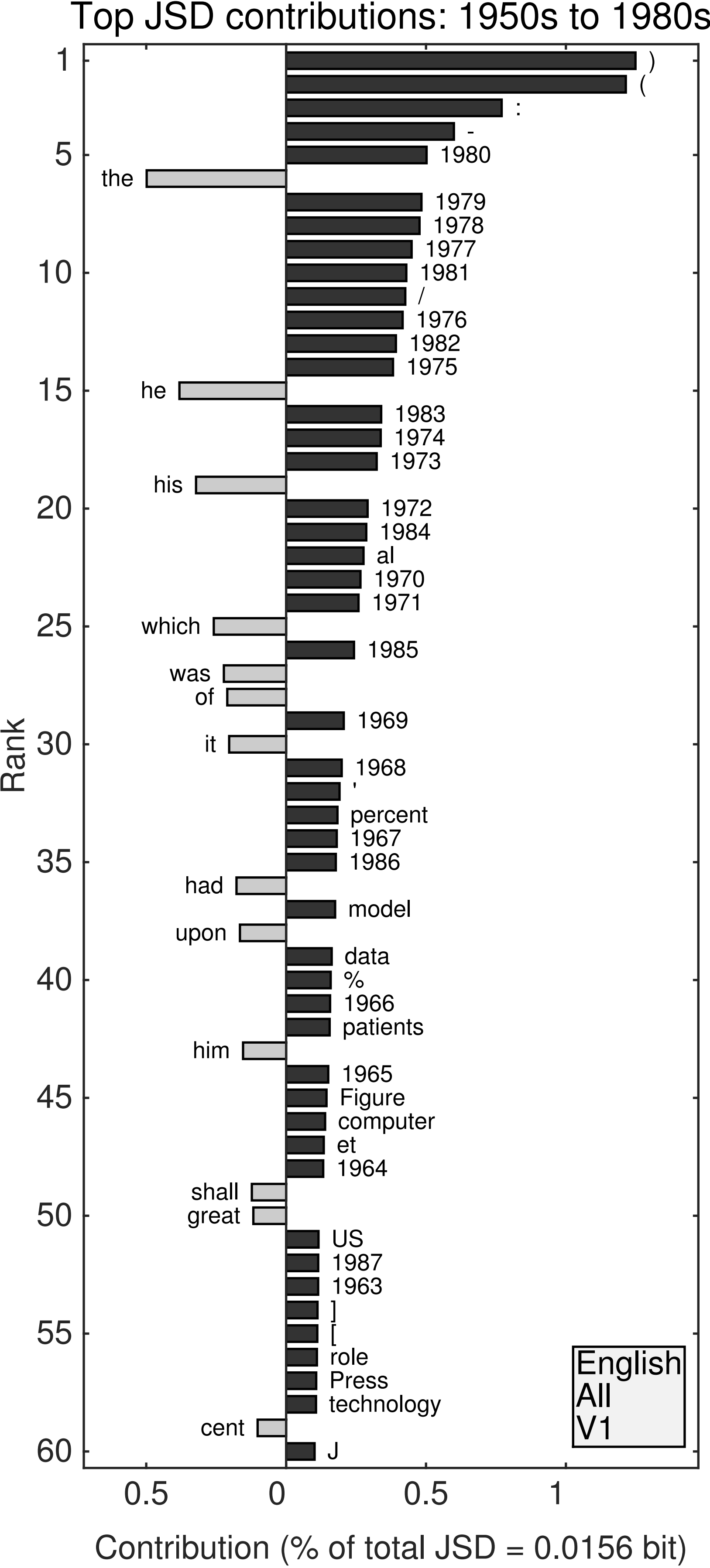

Top JSD contributions: 1950s to 1980s
Rank
Contribution (% of total JSD = 0.0156 bit)
)
(
:
-
1980
the
1979
1978
1977
1981
/
1976
1982
1975
he
1983
1974
1973
his
1972
1984
al
1970
1971
which
1985
was
of
1969
it
1968
'
percent
1967
1986
had
model
upon
data
%
1966
patients
him
1965
Figure
computer
et
1964
shall
great
US
1987
1963
]
[
role
Press
technology
cent
J
English
All
V1

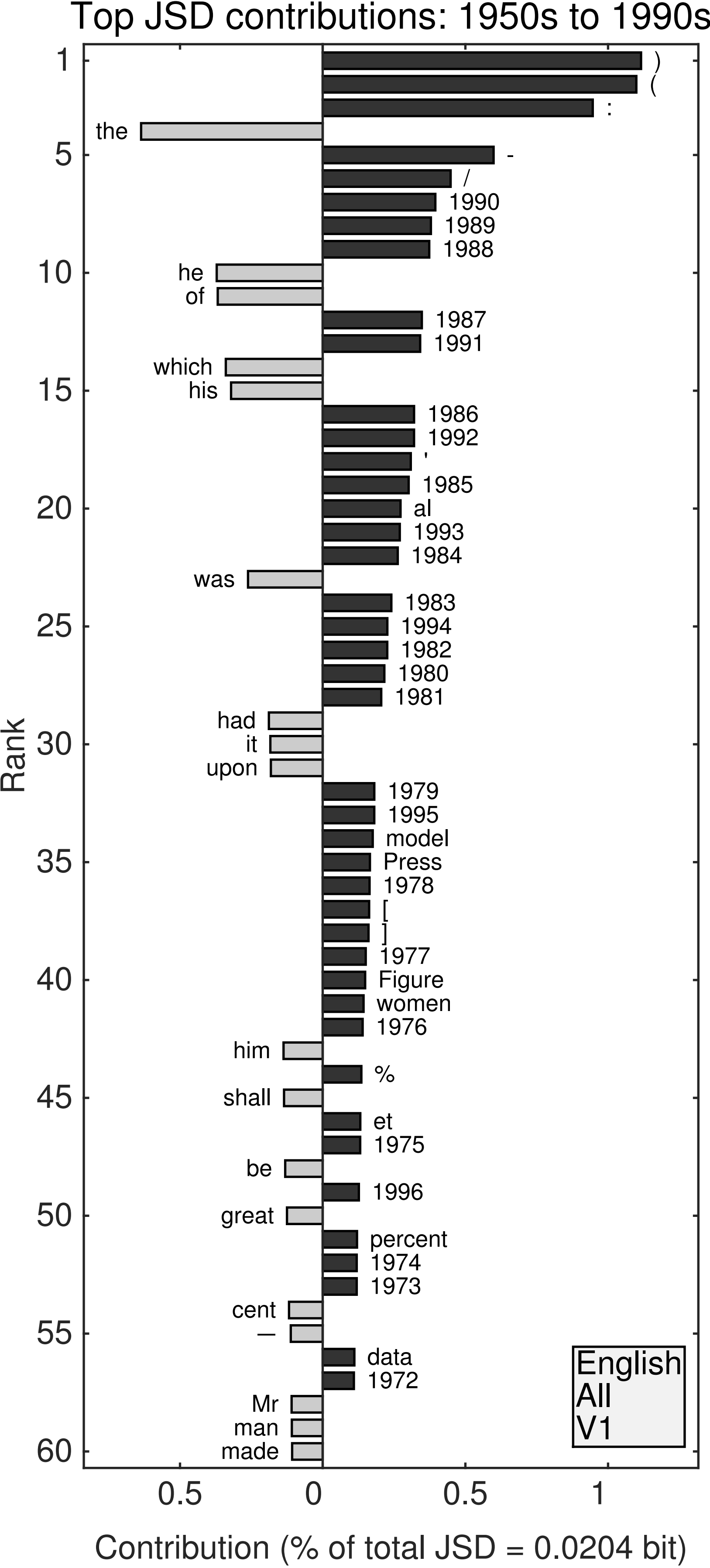
Top JSD contributions: 1950s to 1990s
Rank
Contribution (% of total JSD = 0.0204 bit)
0.5
0
0.5
1
1
5
10
15
20
25
30
35
40
45
50
55
60
)
(
:
the
-
/
1990
1989
1988
he
of
1987
1991
which
his
1986
1992
'
1985
al
1993
1984
was
1983
1994
1982
1980
1981
had
it
upon
1979
1995
model
Press
1978
[
]
1977
Figure
women
1976
him
%
shall
et
1975
be
1996
great
percent
1974
1973
cent
—
data
1972
Mr
man
made
English
All
V1

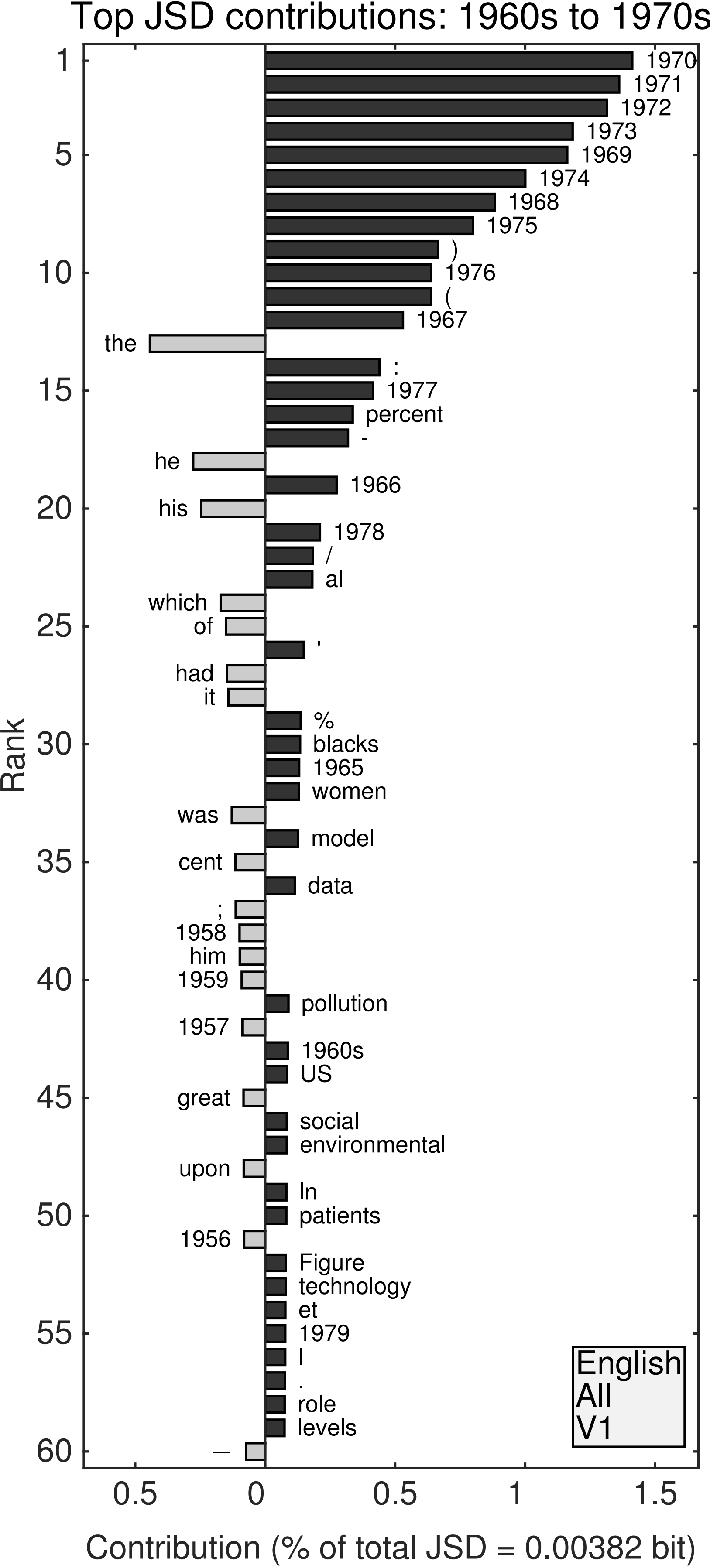

Top JSD contributions: 1960s to 1970s
1970
1971
1972
1973
1969
1974
1968
1975
)
1976
(
1967
the
:
1977
percent
-
he
1966
his
1978
/
al
which
of
'
had
it
%
blacks
1965
women
was
model
cent
data
;
1958
him
1959
pollution
1957
1960s
US
great
social
environmental
upon
ln
patients
1956
Figure
technology
et
1979
l
.
role
levels
—
English
All
V1
Rank
1
5
10
15
20
25
30
35
40
45
50
55
60
0.5
0
0.5
1
1.5
Contribution (% of total JSD = 0.00382 bit)

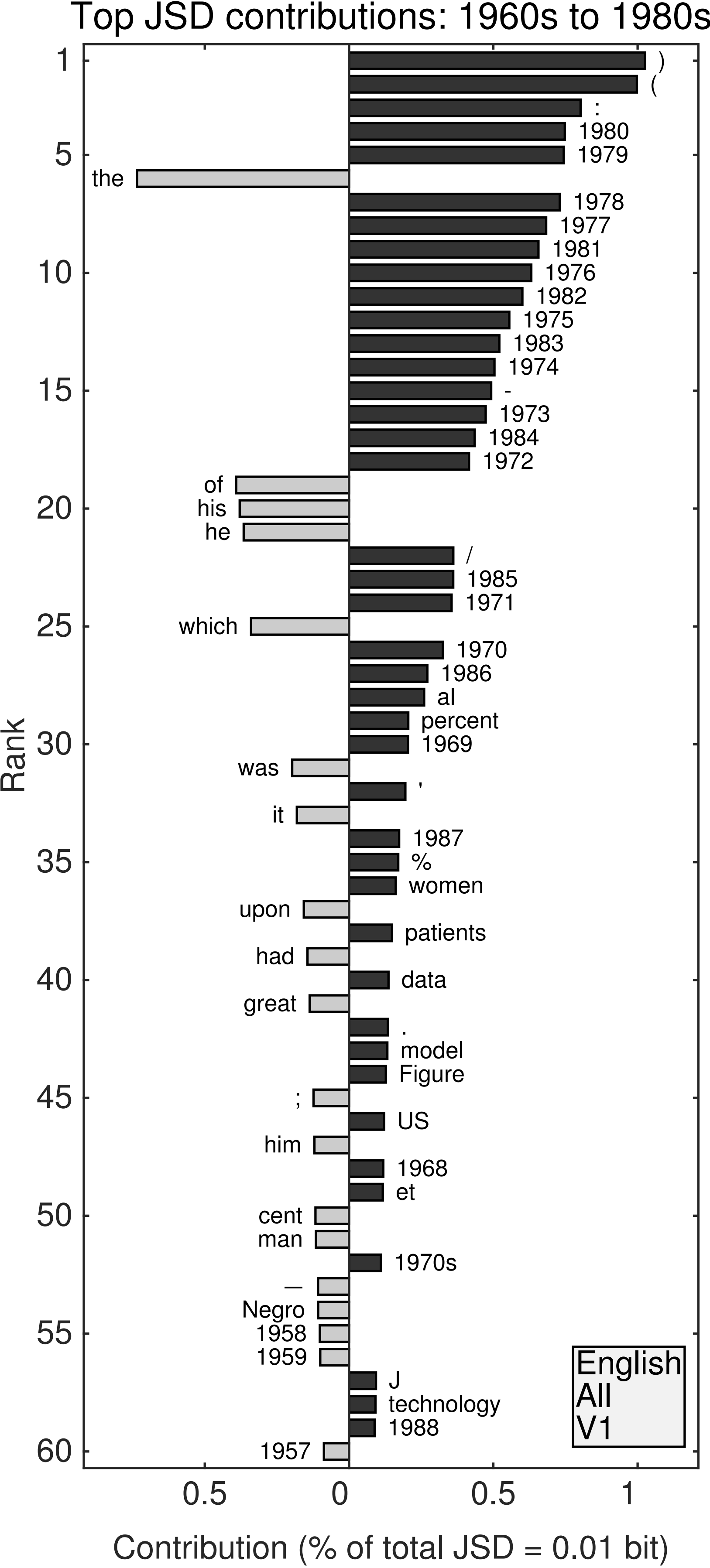

Top JSD contributions: 1960s to 1980s
Rank
Contribution (% of total JSD = 0.01 bit)
)
(
:
1980
1979
the
1978
1977
1981
1976
1982
1975
1983
1974
-
1973
1984
1972
of
his
he
/
1985
1971
which
1970
1986
al
percent
1969
was
'
it
1987
%
women
upon
patients
had
data
great
.
model
Figure
;
US
him
1968
et
cent
man
1970s
—
Negro
1958
1959
J
technology
1988
1957
English
All
V1

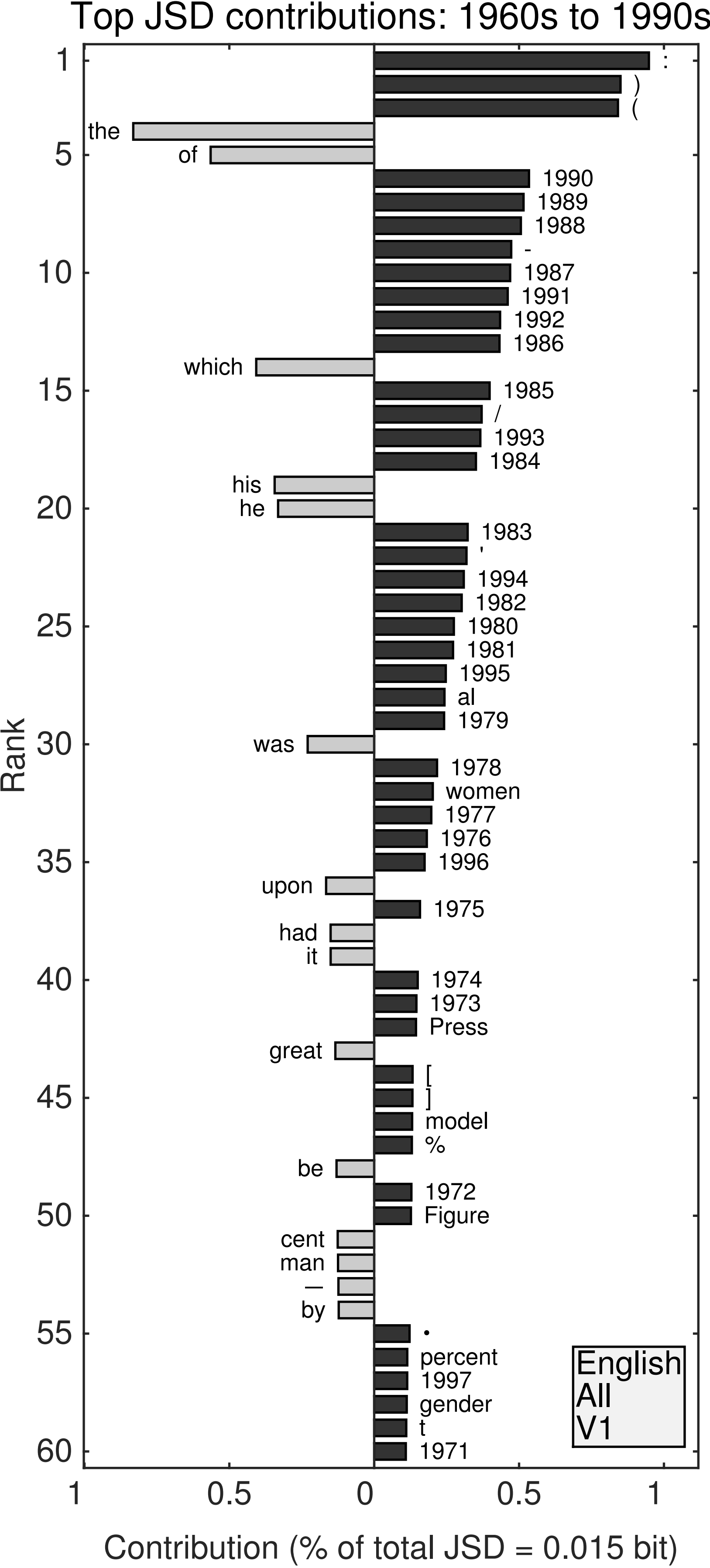

Top JSD contributions: 1960s to 1990s
Rank
Contribution (% of total JSD = 0.015 bit)
English
All
V1
:
)
(
the
of
1990
1989
1988
-
1987
1991
1992
1986
which
1985
/
1993
1984
his
he
1983
'
1994
1982
1980
1981
1995
al
1979
was
1978
women
1977
1976
1996
upon
1975
had
it
1974
1973
Press
great
[
]
model
%
be
1972
Figure
cent
man
—
by
•
percent
1997
gender
t
1971

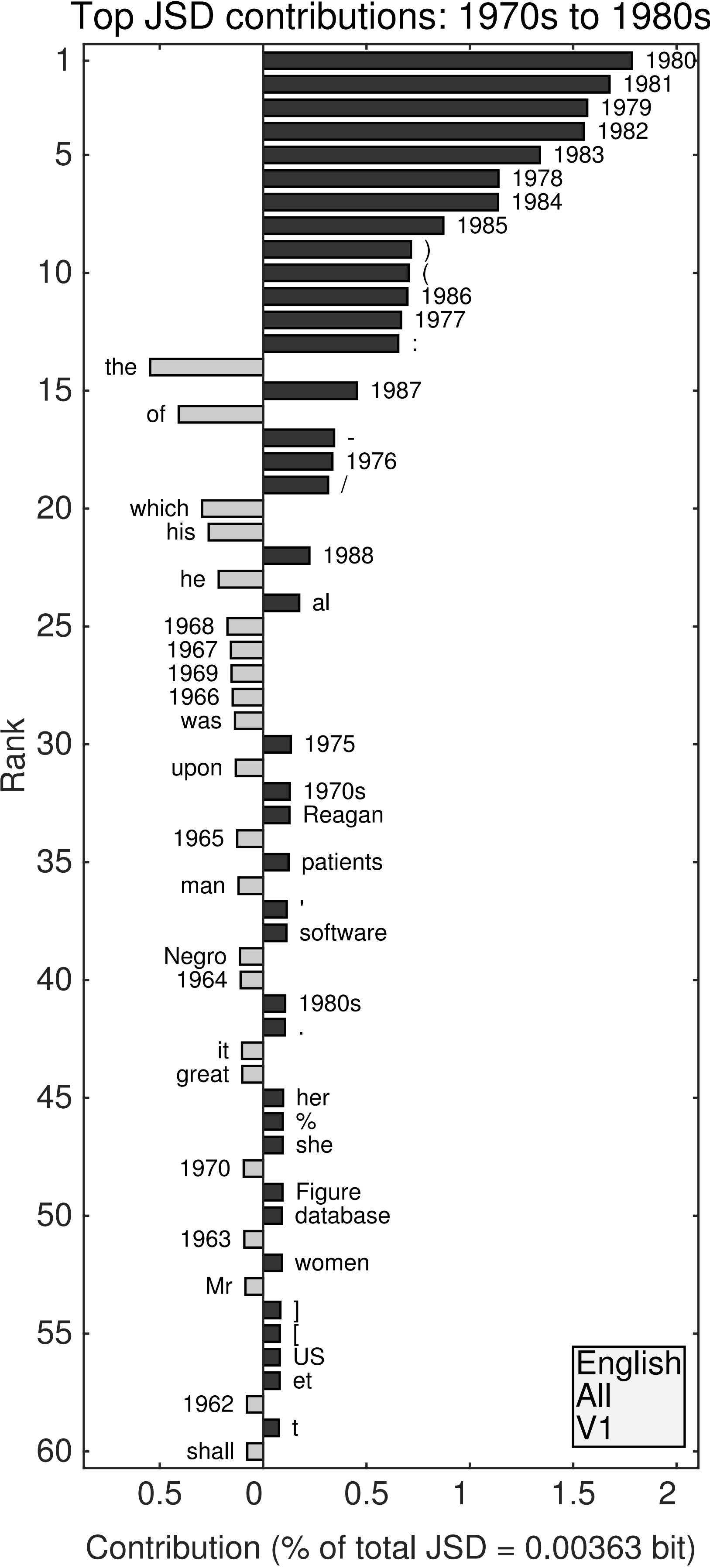
Top JSD contributions: 1970s to 1980s
Rank
Contribution (% of total JSD = 0.00363 bit)
1980
1981
1979
1982
1983
1978
1984
1985
)
(
1986
1977
:
the
1987
of
-
1976
/
which
his
1988
he
al
1968
1967
1969
1966
was
1975
upon
1970s
Reagan
1965
patients
man
'
software
Negro
1964
1980s
.
it
great
her
%
she
1970
Figure
database
1963
women
Mr
]
[
US
et
1962
t
shall
English
All
V1

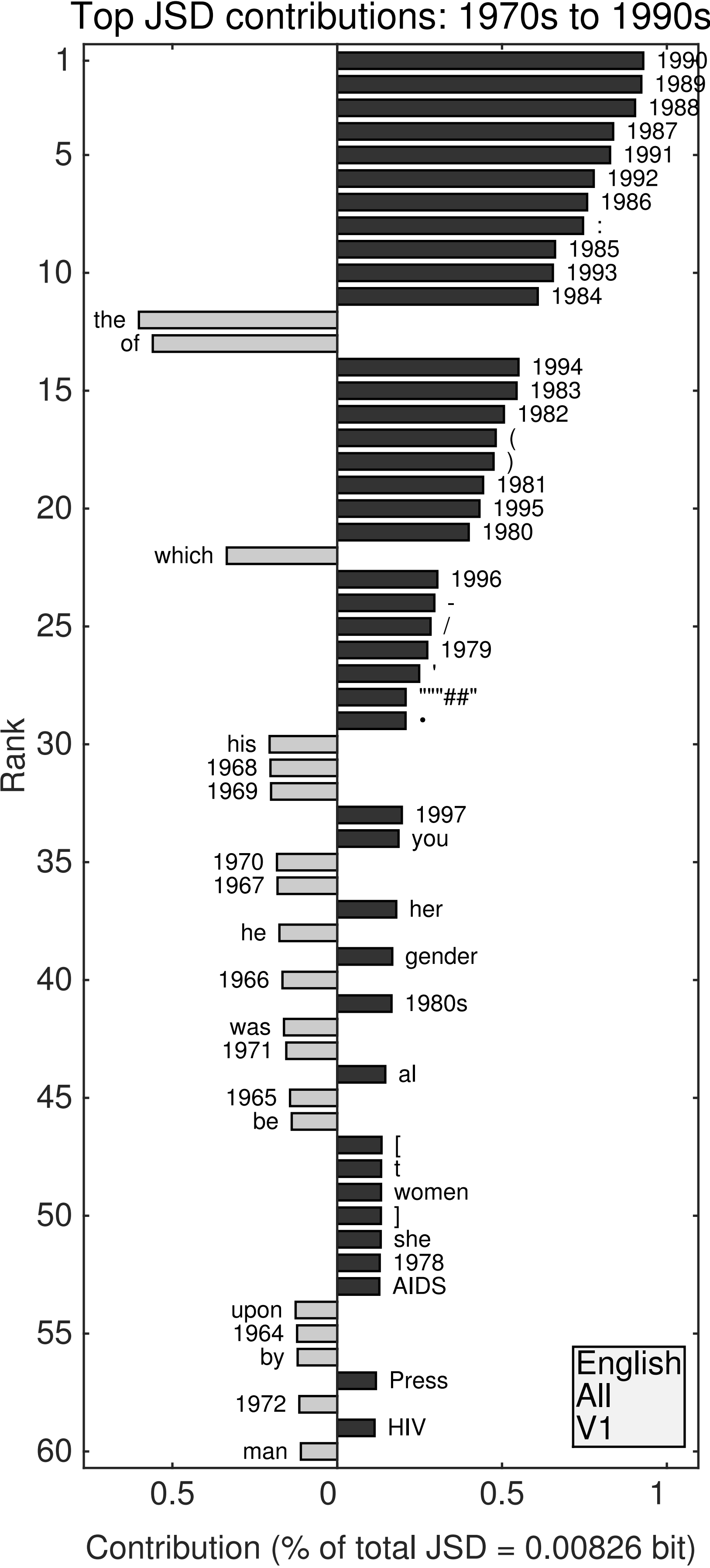

Top JSD contributions: 1970s to 1990s
Rank
Contribution (% of total JSD = 0.00826 bit)
1990
1989
1988
1987
1991
1992
1986
:
1985
1993
1984
the
of
1994
1983
1982
(
)
1981
1995
1980
which
1996
-
/
1979
'
"""##"
•
his
1968
1969
1997
you
1970
1967
her
he
gender
1966
1980s
was
1971
al
1965
be
[
t
women
]
she
1978
AIDS
upon
1964
by
Press
1972
HIV
man
English
All
V1

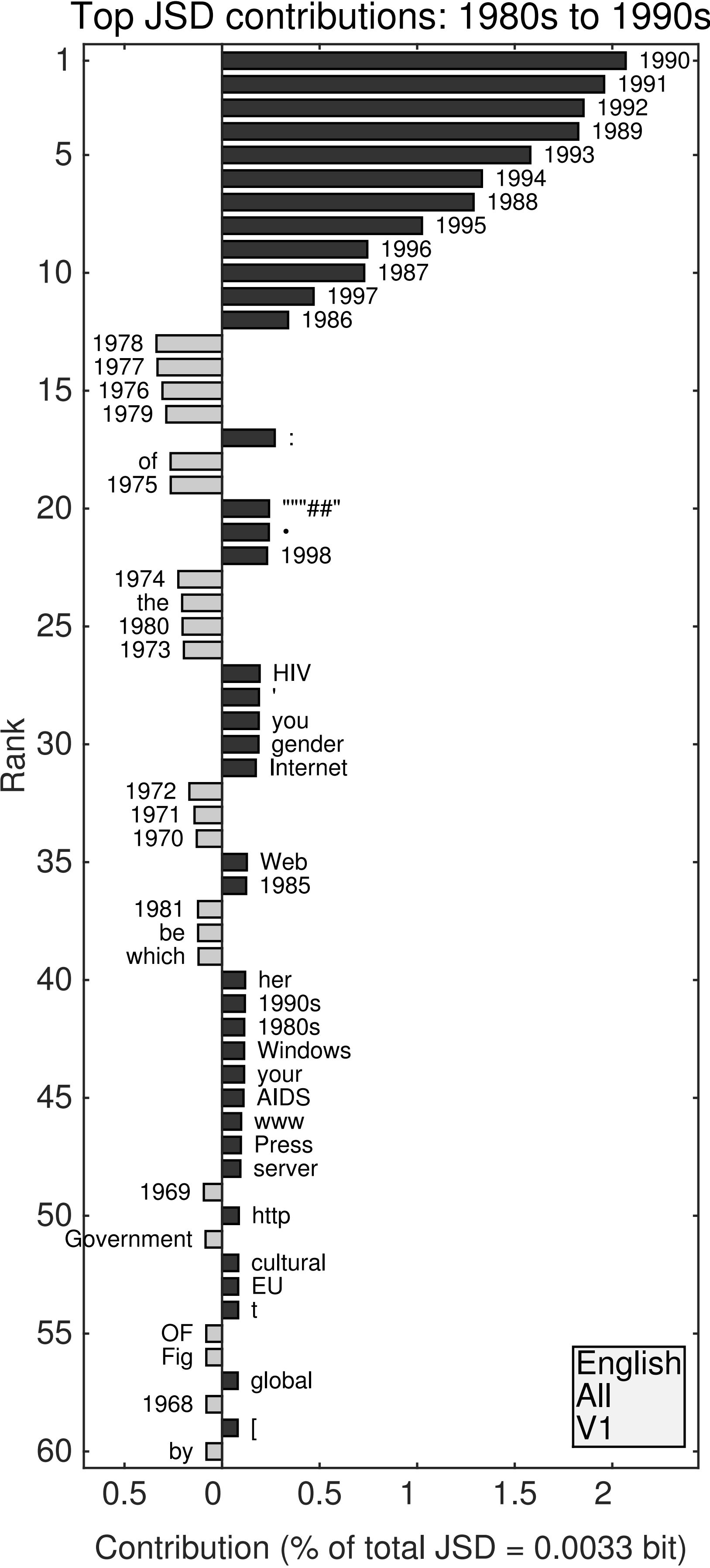
Top JSD contributions: 1980s to 1990s
Rank
Contribution (% of total JSD = 0.0033 bit)
1990
1991
1992
1989
1993
1994
1988
1995
1996
1987
1997
1986
1978
1977
1976
1979
:
of
1975
"""##"
•
1998
1974
the
1980
1973
HIV
'
you
gender
Internet
1972
1971
1970
Web
1985
1981
be
which
her
1990s
1980s
Windows
your
AIDS
www
Press
server
1969
http
Government
cultural
EU
t
OF
Fig
global
1968
[
by
English
All
V1
