# Supplementary material for: Characterizing the Google Books Corpus: Strong Limits to Inferences of Socio-Cultural and Linguistic Evolution
Source: PLoS One. 2015 Oct 7;10(10):e0137041. doi: 10.1371/journal.pone.0137041 (PMC4596490; doi:10.1371/journal.pone.0137041)
Supplement: S3 File — (PDF) [file pone.0137041.s003.pdf]

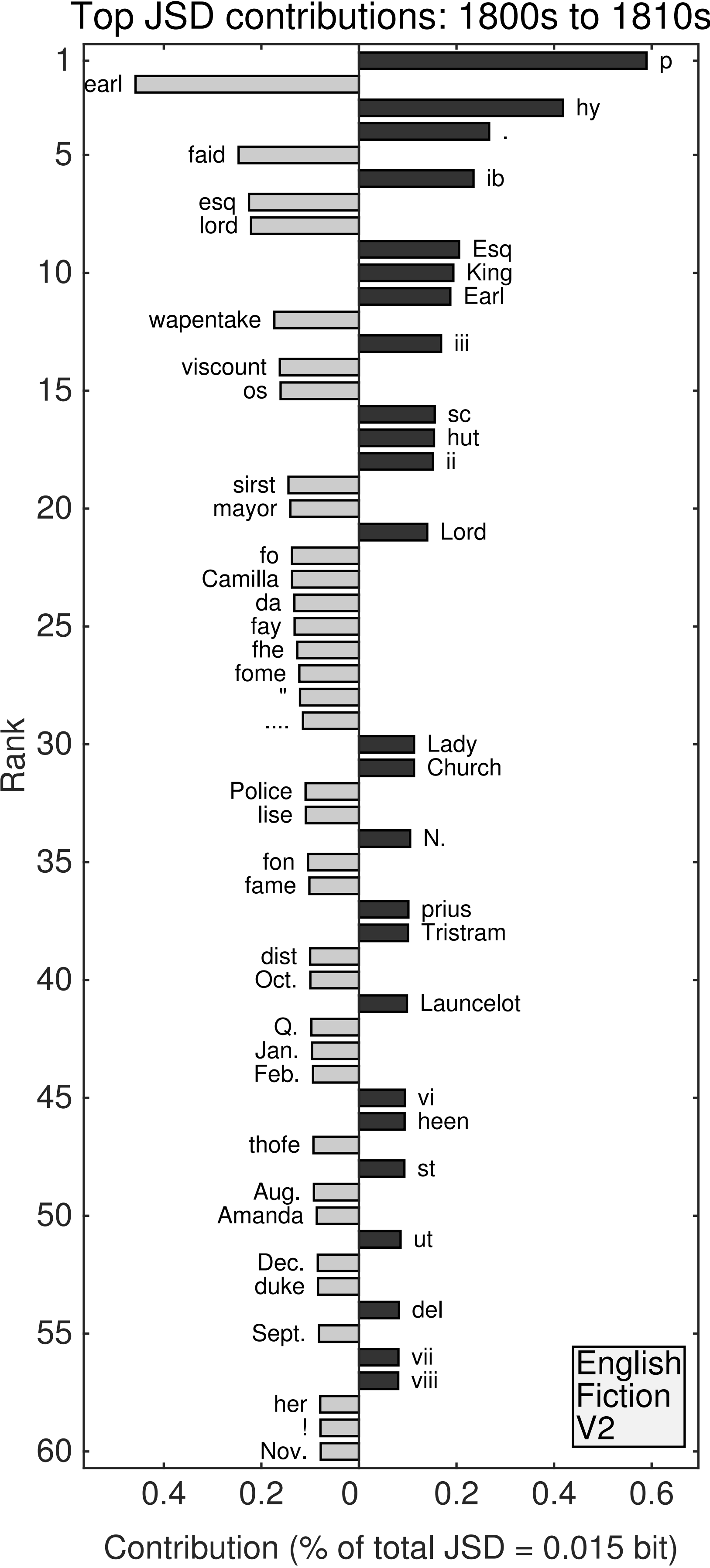

Top JSD contributions: 1800s to 1810s
Rank
Contribution (% of total JSD = 0.015 bit)
p
earl
hy
.
faid
ib
esq
lord
Esq
King
Earl
wapentake
iii
viscount
os
sc
hut
ii
sirst
mayor
Lord
fo
Camilla
da
fay
fhe
fome
"
....
Lady
Church
Police
lise
N.
fon
fame
prius
Tristram
dist
Oct.
Launcelot
Q.
Jan.
Feb.
vi
heen
thofe
st
Aug.
Amanda
ut
Dec.
duke
del
Sept.
vii
viii
her
!
Nov.
English
Fiction
V2

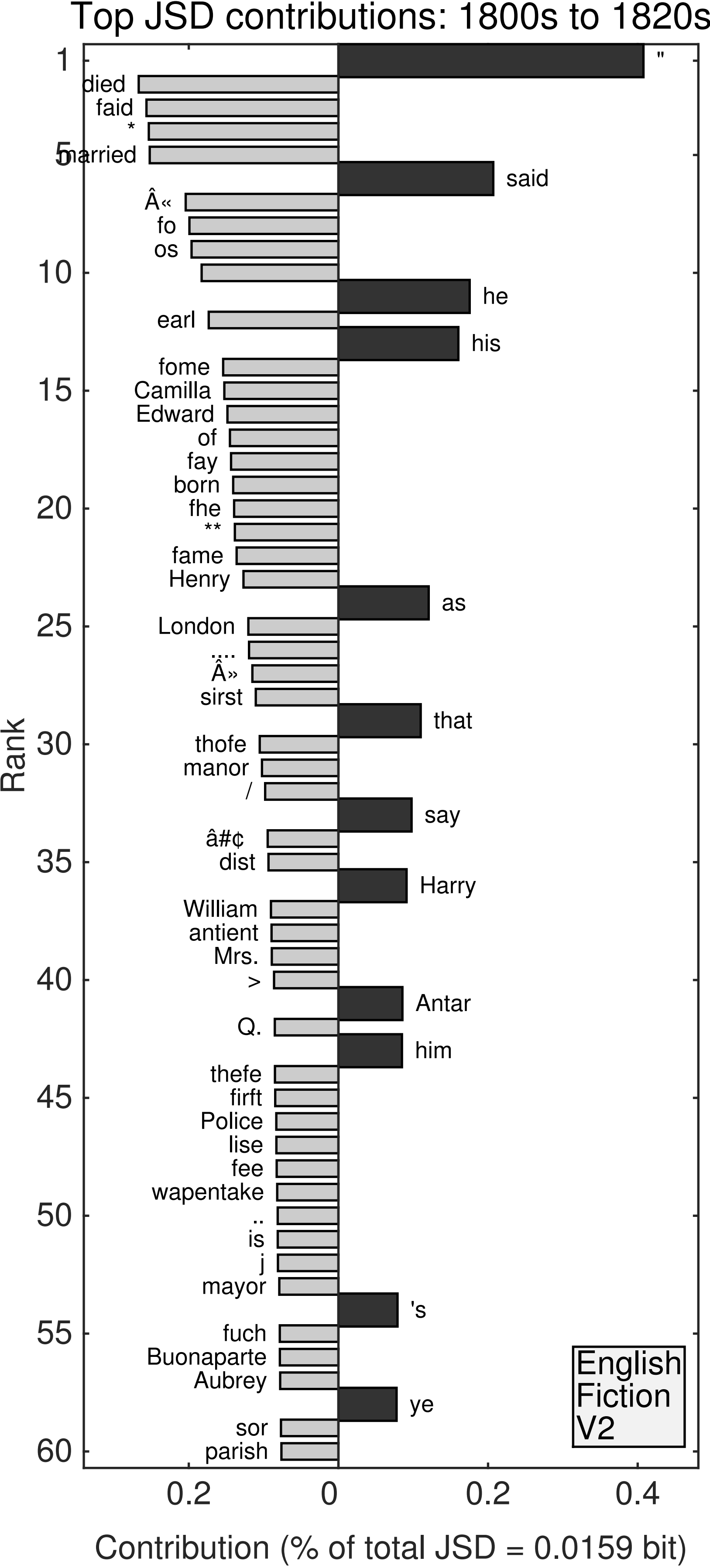
Top JSD contributions: 1800s to 1820s
Rank
Contribution (% of total JSD = 0.0159 bit)
"
died
faid
*
married
said
Â«
fo
os
he
earl
his
fome
Camilla
Edward
of
fay
born
fhe
**
fame
Henry
as
London
....
Â»
sirst
that
thofe
manor
/
say
â#¢
dist
Harry
William
antient
Mrs.
>
Antar
Q.
him
thefe
firft
Police
lise
fee
wapentake
..
is
j
mayor
's
fuch
Buonaparte
Aubrey
ye
sor
parish
English Fiction V2

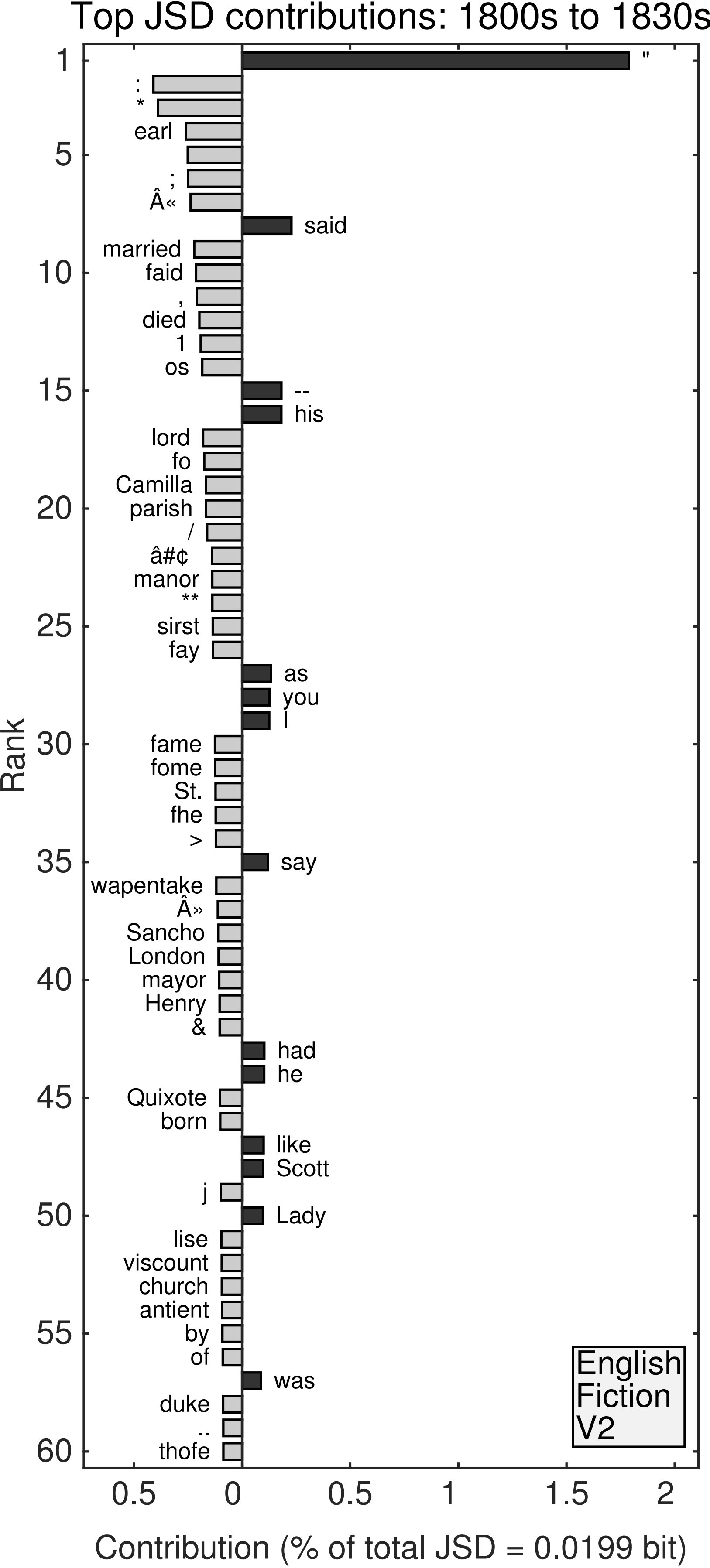

Top JSD contributions: 1800s to 1830s
Rank
Contribution (% of total JSD = 0.0199 bit)
"
:
*
earl
;
Â«
said
married
faid
,
died
1
os
--
his
lord
fo
Camilla
parish
/
â#¢
manor
**
sirst
fay
as
you
I
fame
fome
St.
fhe
>
say
wapentake
Â»
Sancho
London
mayor
Henry
&
had
he
Quixote
born
like
Scott
j
Lady
lise
viscount
church
antient
by
of
was
duke
..
thofe
English Fiction V2

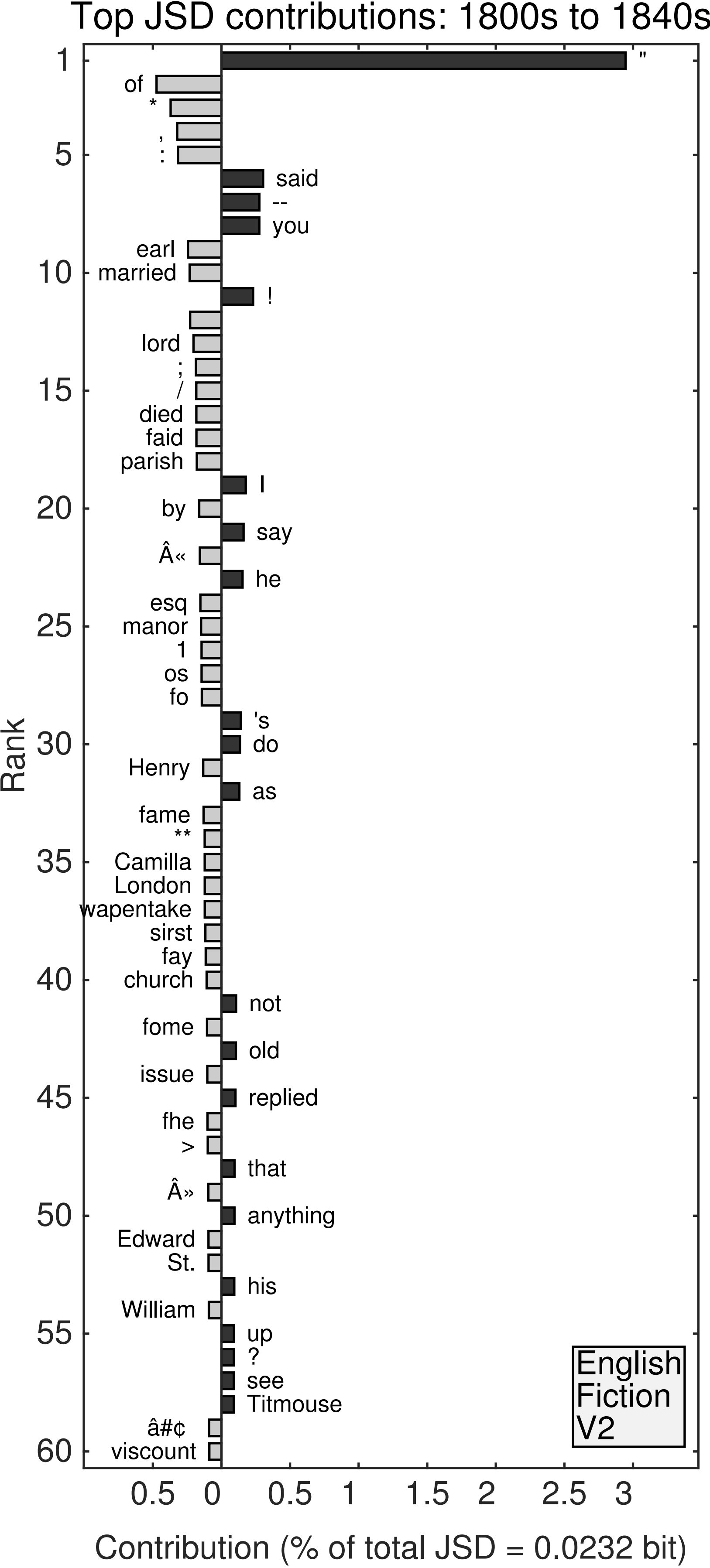

Top JSD contributions: 1800s to 1840s
Rank
Contribution (% of total JSD = 0.0232 bit)
English Fiction V2
"
of
*
,
:
said
--
you
earl
married
!
lord
;
/
died
faid
parish
I
by
say
Â«
he
esq
manor
1
os
fo
's
do
Henry
as
fame
**
Camilla
London
wapentake
sirst
fay
church
not
fome
old
issue
replied
fhe
>
that
Â»
anything
Edward
St.
his
William
up
?
see
Titmouse
â#¢
viscount
1 5 10 15 20 25 30 35 40 45 50 55 60
0.5 0 0.5 1 1.5 2 2.5 3

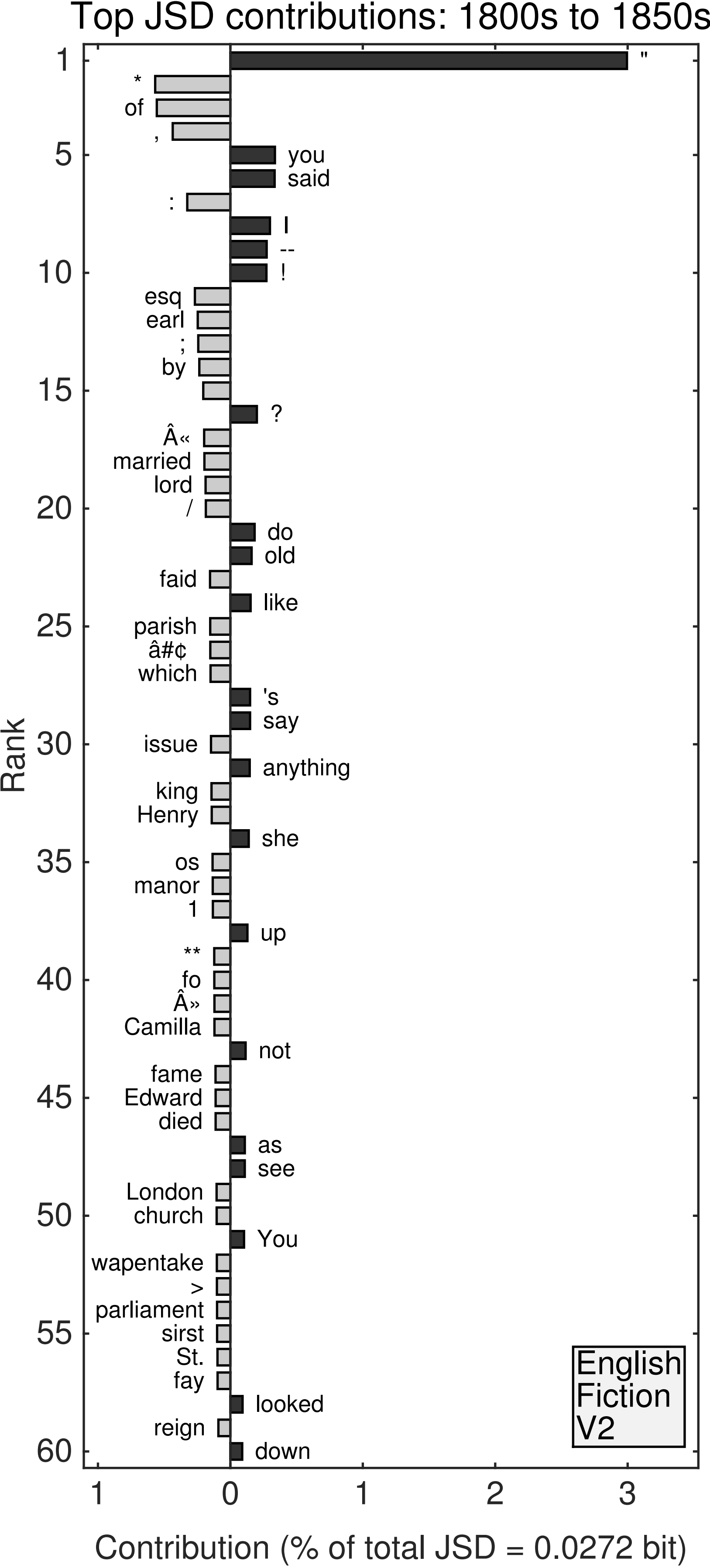

Top JSD contributions: 1800s to 1850s
Rank
Contribution (% of total JSD = 0.0272 bit)
"
*
of
,
you
said
:
I
--
!
esq
earl
;
by
?
Â«
married
lord
/
do
old
faid
like
parish
â#¢
which
's
say
issue
anything
king
Henry
she
os
manor
1
up
**
fo
Â»
Camilla
not
fame
Edward
died
as
see
London
church
You
wapentake
>
parliament
sirst
St.
fay
looked
reign
down
English
Fiction
V2

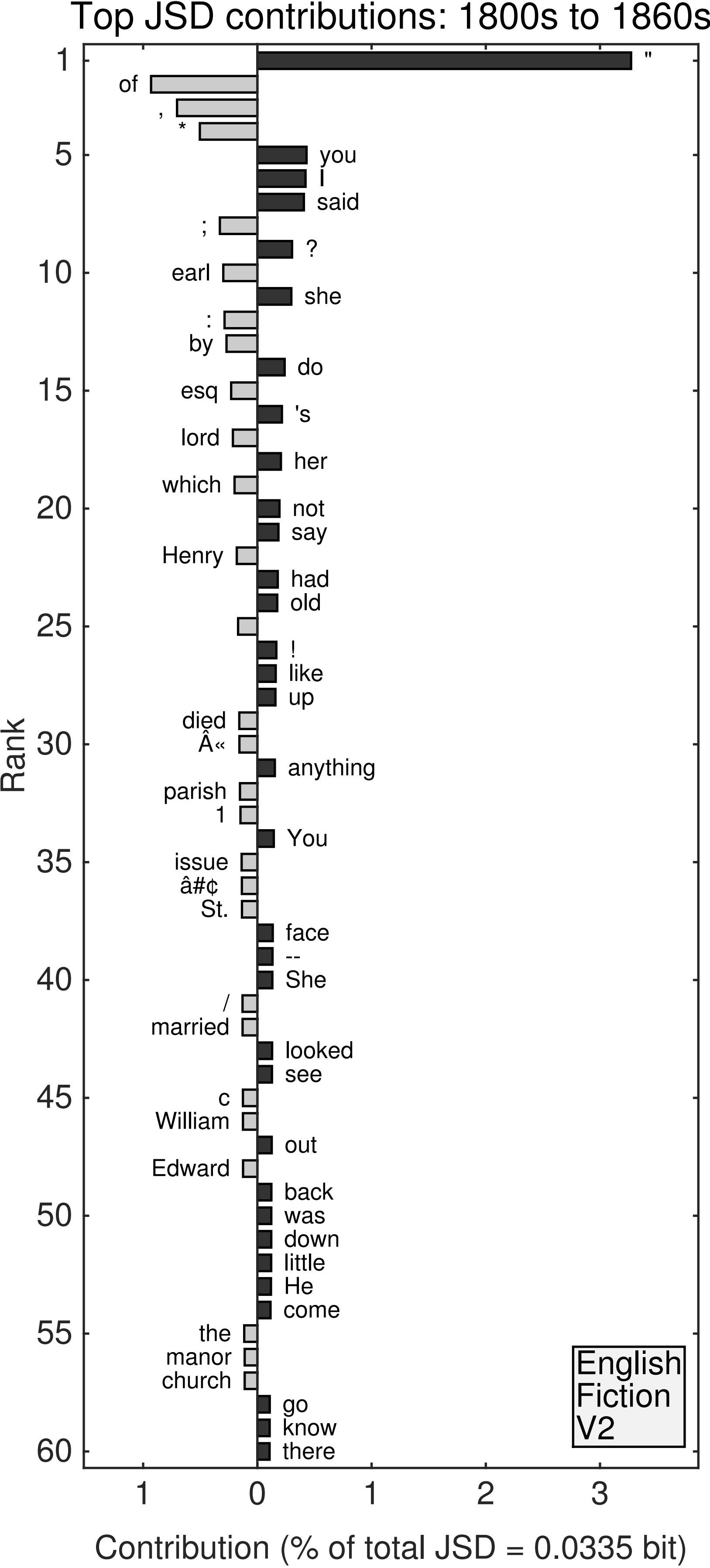
Top JSD contributions: 1800s to 1860s
Rank
Contribution (% of total JSD = 0.0335 bit)
English Fiction V2
"
of
,
*
you
I
said
;
?
earl
she
:
by
do
esq
's
lord
her
which
not
say
Henry
had
old
!
like
up
died
Â«
anything
parish
1
You
issue
â#¢
St.
face
--
She
/
married
looked
see
c
William
out
Edward
back
was
down
little
He
come
the
manor
church
go
know
there

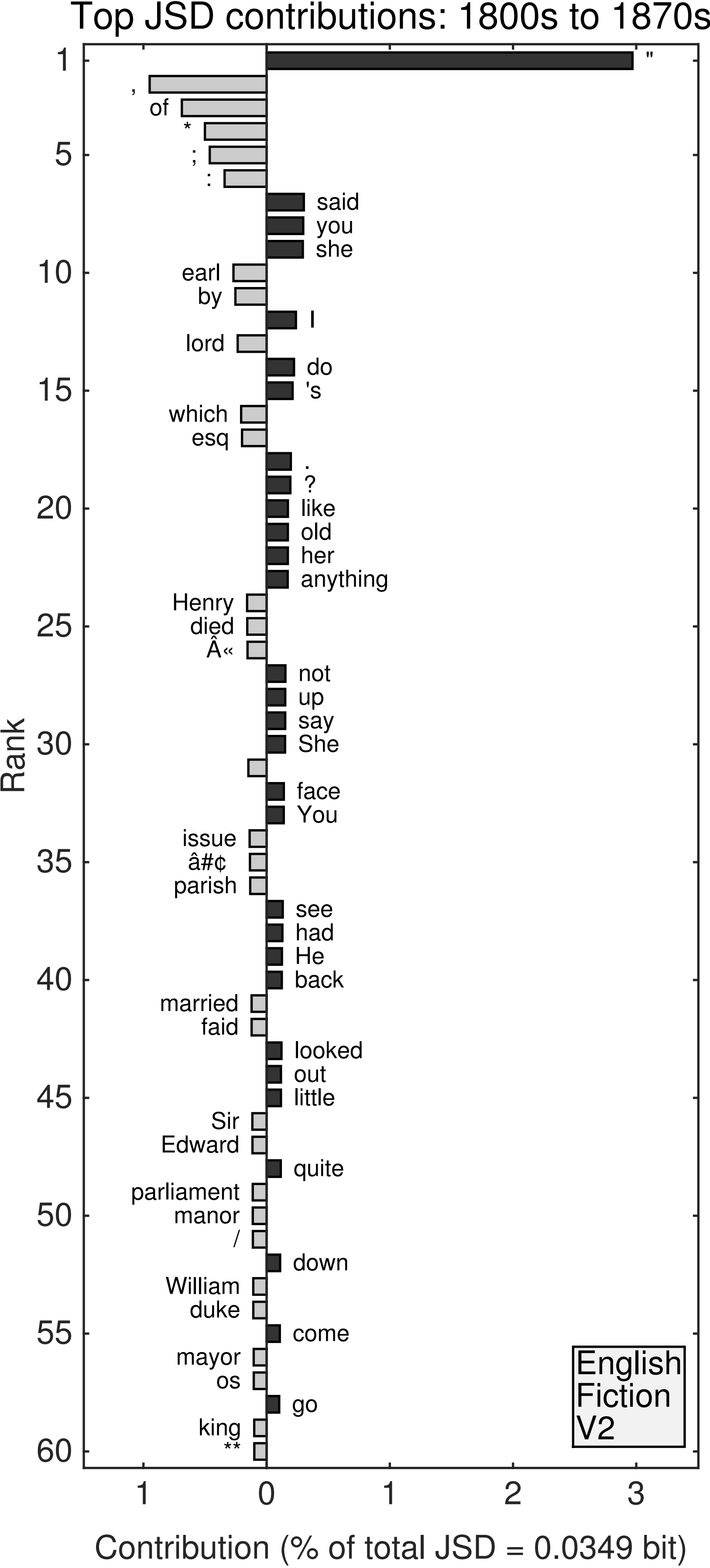
Top JSD contributions: 1800s to 1870s
Rank
Contribution (% of total JSD = 0.0349 bit)
"
,
of
*
;
:
said
you
she
earl
by
I
lord
do
's
which
esq
.
?
like
old
her
anything
Henry
died
Â«
not
up
say
She
face
You
issue
â#¢
parish
see
had
He
back
married
faid
looked
out
little
Sir
Edward
quite
parliament
manor
/
down
William
duke
come
mayor
os
go
king
**
English
Fiction
V2

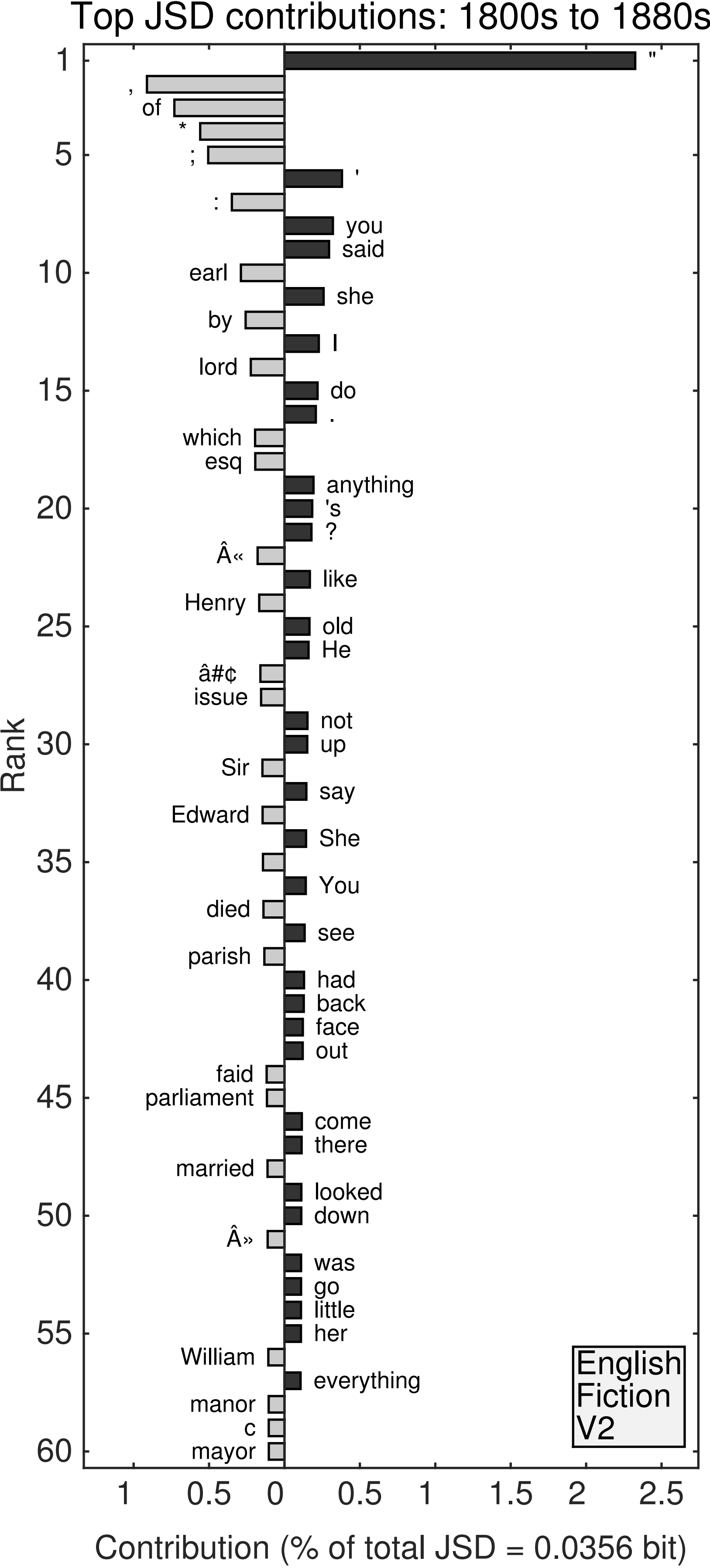
Top JSD contributions: 1800s to 1880s
Rank
Contribution (% of total JSD = 0.0356 bit)
English Fiction V2
"
,
of
*
;
'
:
you
said
earl
she
by
I
lord
do
.
which
esq
anything
's
?
Â«
like
Henry
old
He
â#¢
issue
not
up
Sir
say
Edward
She
You
died
see
parish
had
back
face
out
faid
parliament
come
there
married
looked
down
Â»
was
go
little
her
William
everything
manor
c
mayor

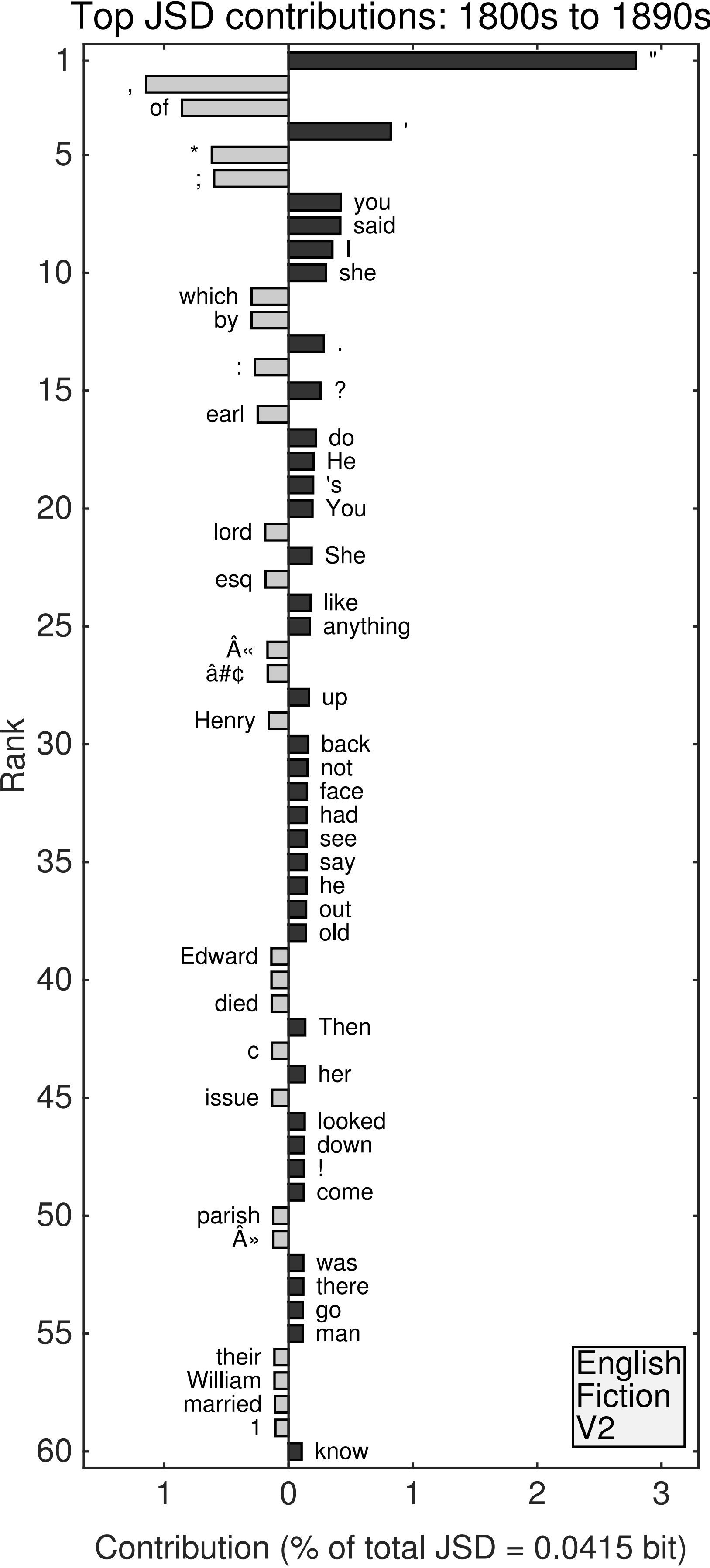
Top JSD contributions: 1800s to 1890s
Rank
Contribution (% of total JSD = 0.0415 bit)
"
,
of
'
*
;
you
said
I
she
which
by
.
:
?
earl
do
He
's
You
lord
She
esq
like
anything
Â«
â#¢
up
Henry
back
not
face
had
see
say
he
out
old
Edward
died
Then
c
her
issue
looked
down
!
come
parish
Â»
was
there
go
man
their
William
married
1
know
English
Fiction
V2

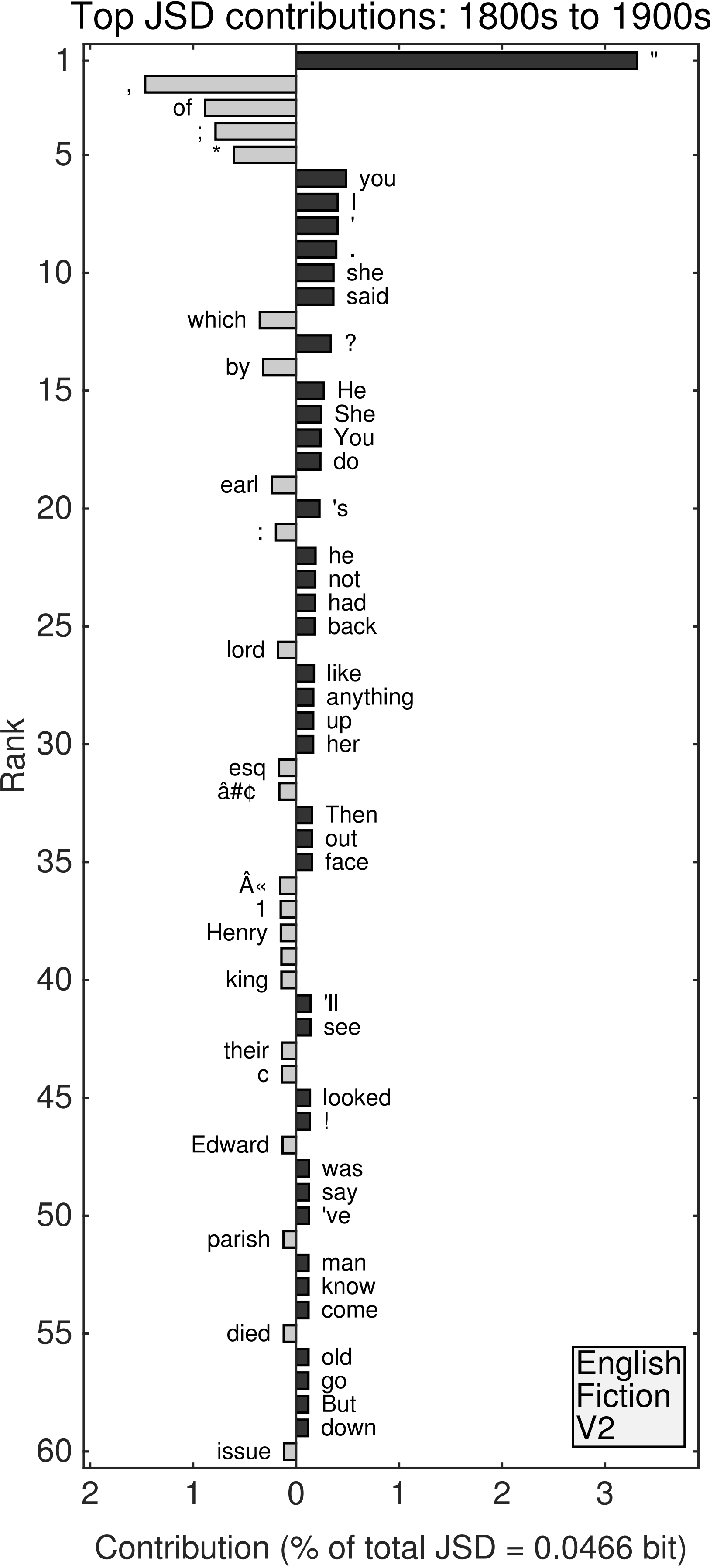
Top JSD contributions: 1800s to 1900s
Rank
Contribution (% of total JSD = 0.0466 bit)
English Fiction V2
"
,
of
;
*
you
!
'
.
she
said
which
?
by
He
She
You
do
earl
's
:
he
not
had
back
lord
like
anything
up
her
esq
â#¢
Then
out
face
Â«
1
Henry
king
'll
see
their
c
looked
!
Edward
was
say
've
parish
man
know
come
died
old
go
But
down
issue

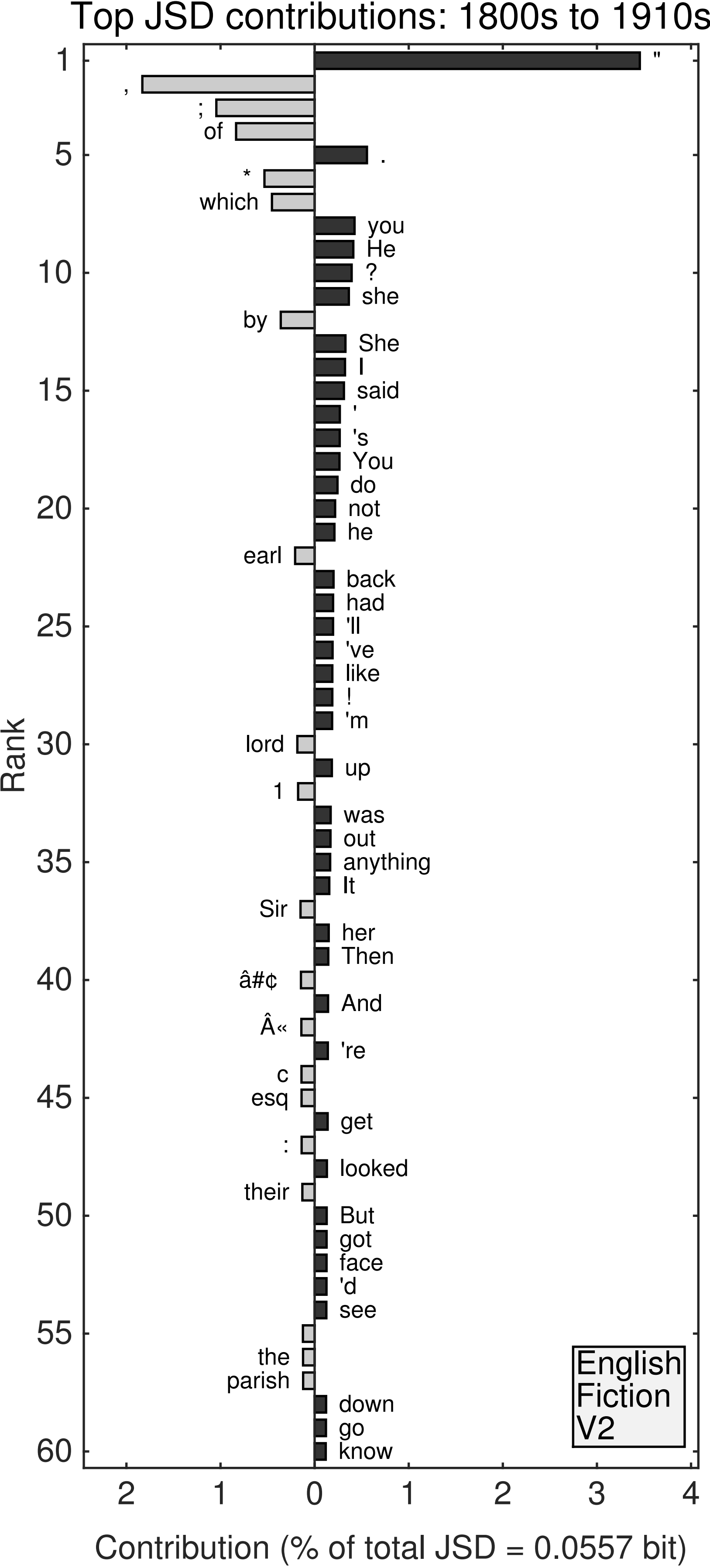

Top JSD contributions: 1800s to 1910s
Rank
Contribution (% of total JSD = 0.0557 bit)
English Fiction V2
"
,
;
of
.
*
which
you
He
?
she
by
She
I
said
'
's
You
do
not
he
earl
back
had
'll
've
like
!
'm
lord
up
1
was
out
anything
It
Sir
her
Then
â#¢
And
Â«
're
c
esq
get
:
looked
their
But
got
face
'd
see
the
parish
down
go
know

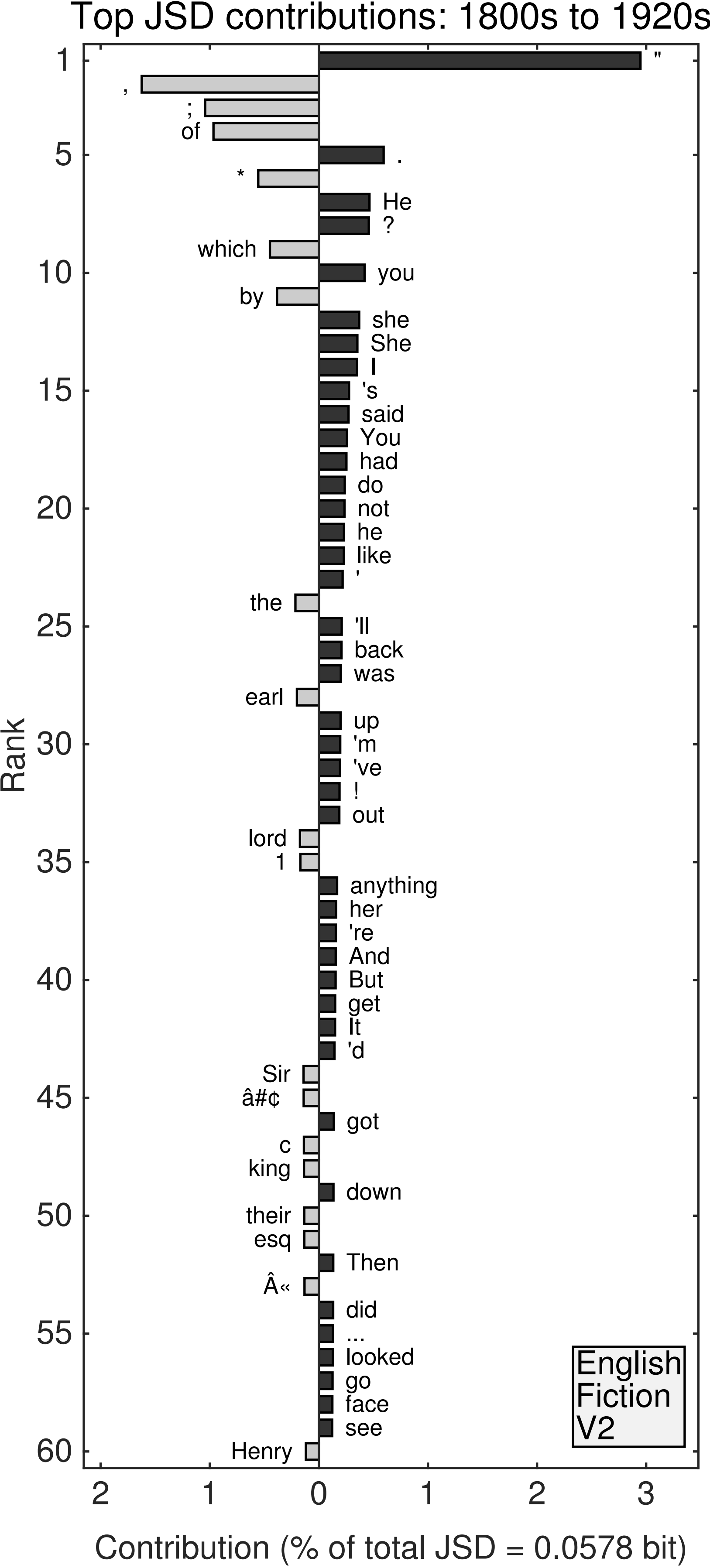
Top JSD contributions: 1800s to 1920s
Rank
Contribution (% of total JSD = 0.0578 bit)
English Fiction V2
"
,
;
of
.
*
He
?
which
you
by
she
She
I
's
said
You
had
do
not
he
like
'
the
'll
back
was
earl
up
'm
've
!
out
lord
1
anything
her
're
And
But
get
It
'd
Sir
â#¢
got
c
king
down
their
esq
Then
Â«
did
...
looked
go
face
see
Henry

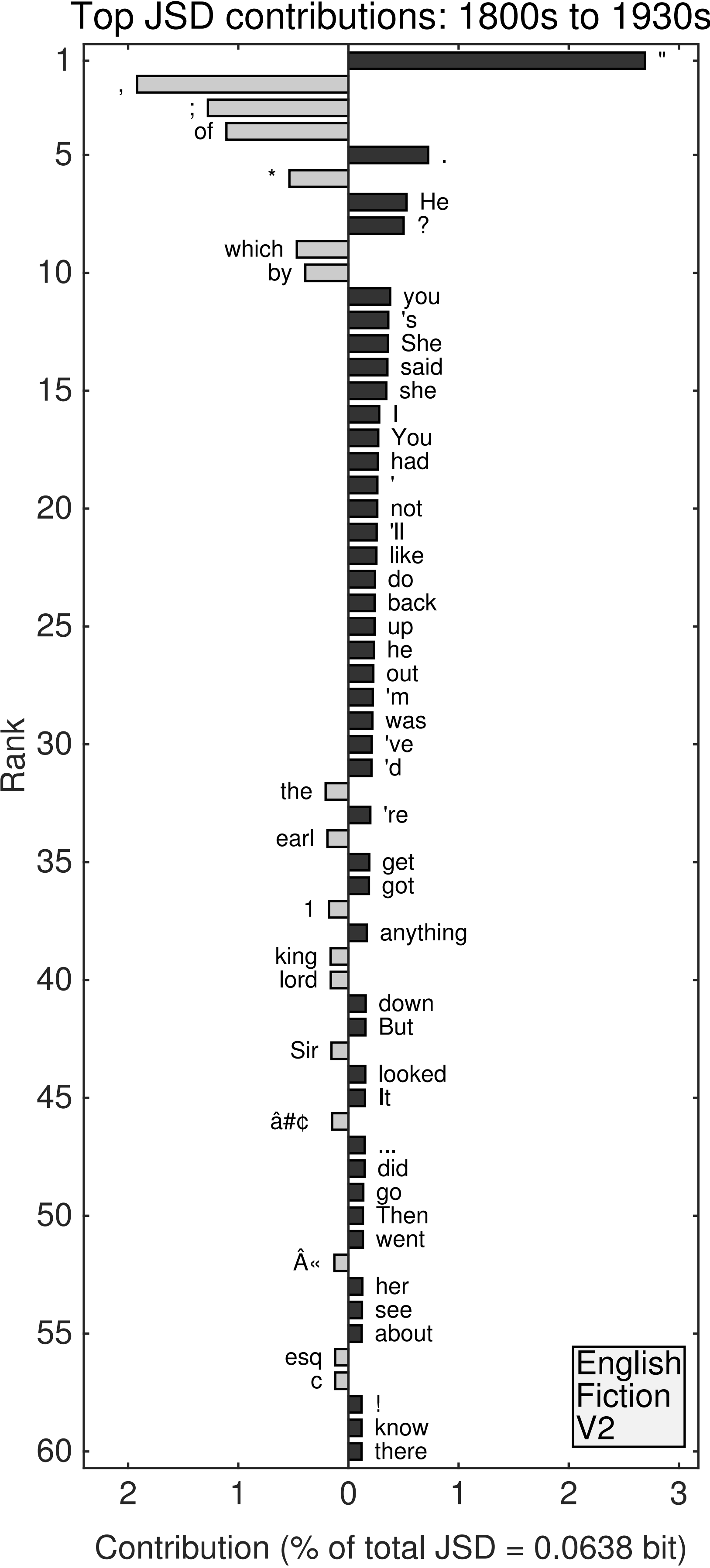

Top JSD contributions: 1800s to 1930s
Rank
Contribution (% of total JSD = 0.0638 bit)
English Fiction V2
"
,
;
of
.
*
He
?
which
by
you
's
She
said
she
I
You
had
'
not
'll
like
do
back
up
he
out
'm
was
've
'd
the
're
earl
get
got
1
anything
king
lord
down
But
Sir
looked
It
â#¢
...
did
go
Then
went
Â«
her
see
about
esq
c
!
know
there

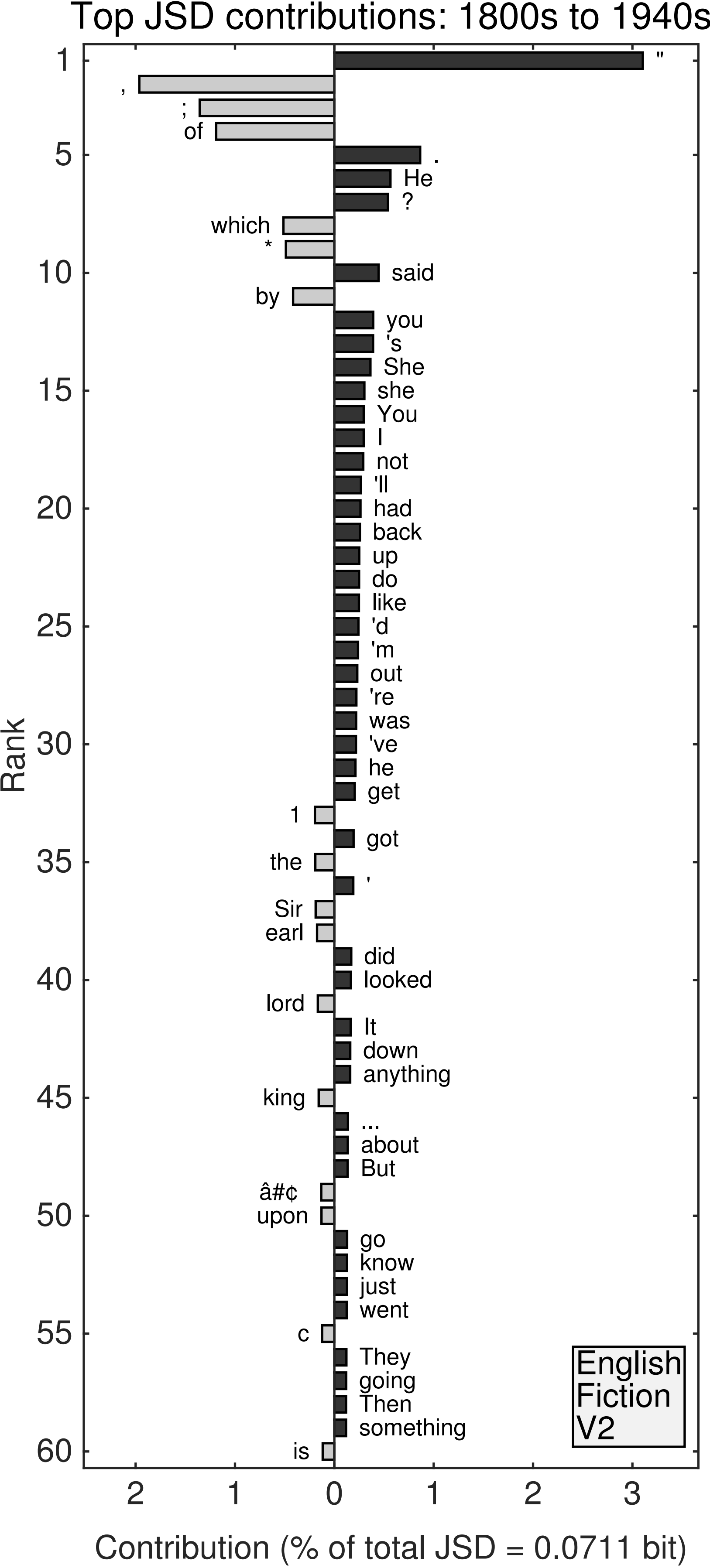

Top JSD contributions: 1800s to 1940s
Rank
Contribution (% of total JSD = 0.0711 bit)
English Fiction V2
"
,
;
of
.
He
?
which
*
said
by
you
's
She
she
You
I
not
'll
had
back
up
do
like
'd
'm
out
're
was
've
he
get
1
got
the
'
Sir
earl
did
looked
lord
It
down
anything
king
...
about
But
â#¢
upon
go
know
just
went
c
They
going
Then
something
is

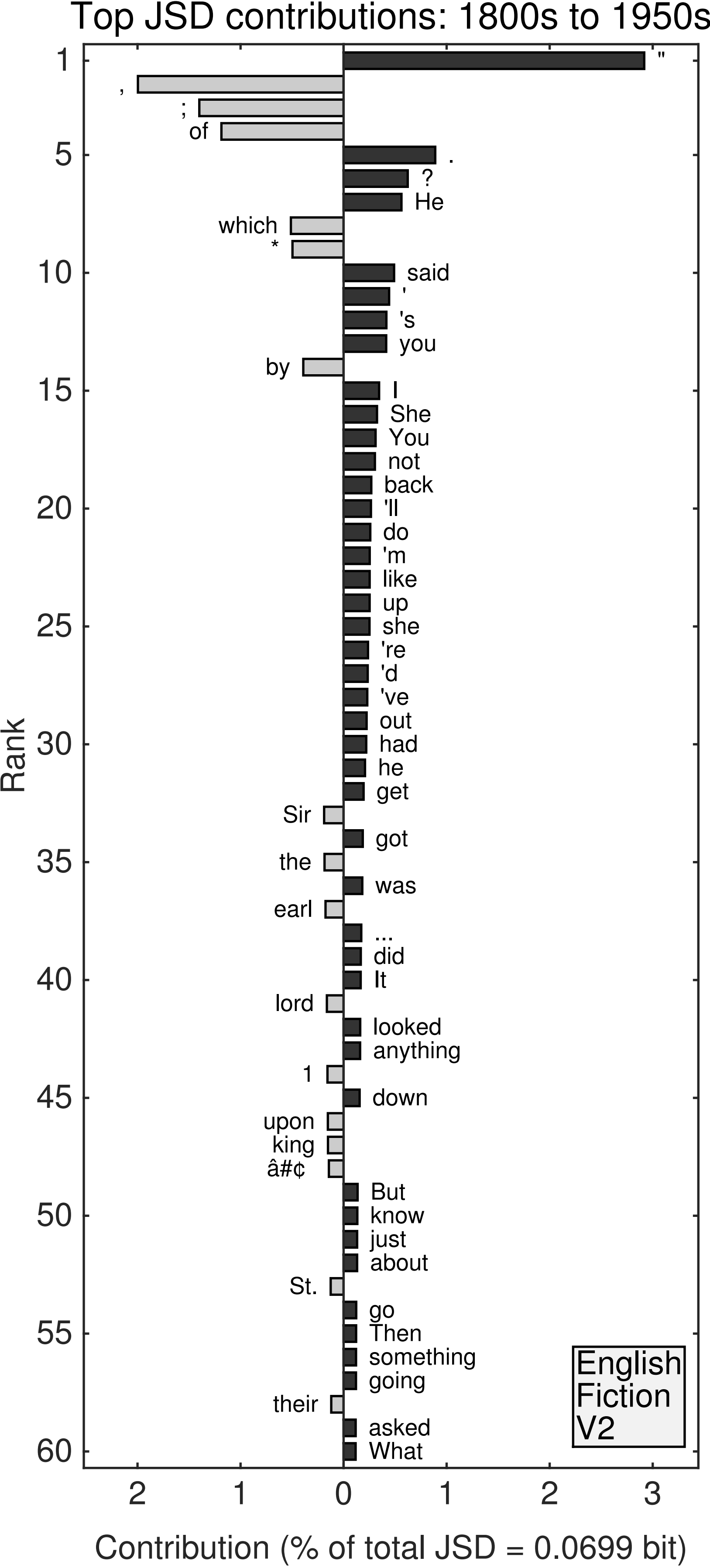
Top JSD contributions: 1800s to 1950s
Rank
Contribution (% of total JSD = 0.0699 bit)
English Fiction V2
"
,
;
of
.
?
He
which
*
said
'
's
you
by
I
She
You
not
back
'll
do
'm
like
up
she
're
'd
've
out
had
he
get
Sir
got
the
was
earl
...
did
It
lord
looked
anything
1
down
upon
king
â#¢
But
know
just
about
St.
go
Then
something
going
their
asked
What
1
5
10
15
20
25
30
35
40
45
50
55
60
2
1
0
1
2
3

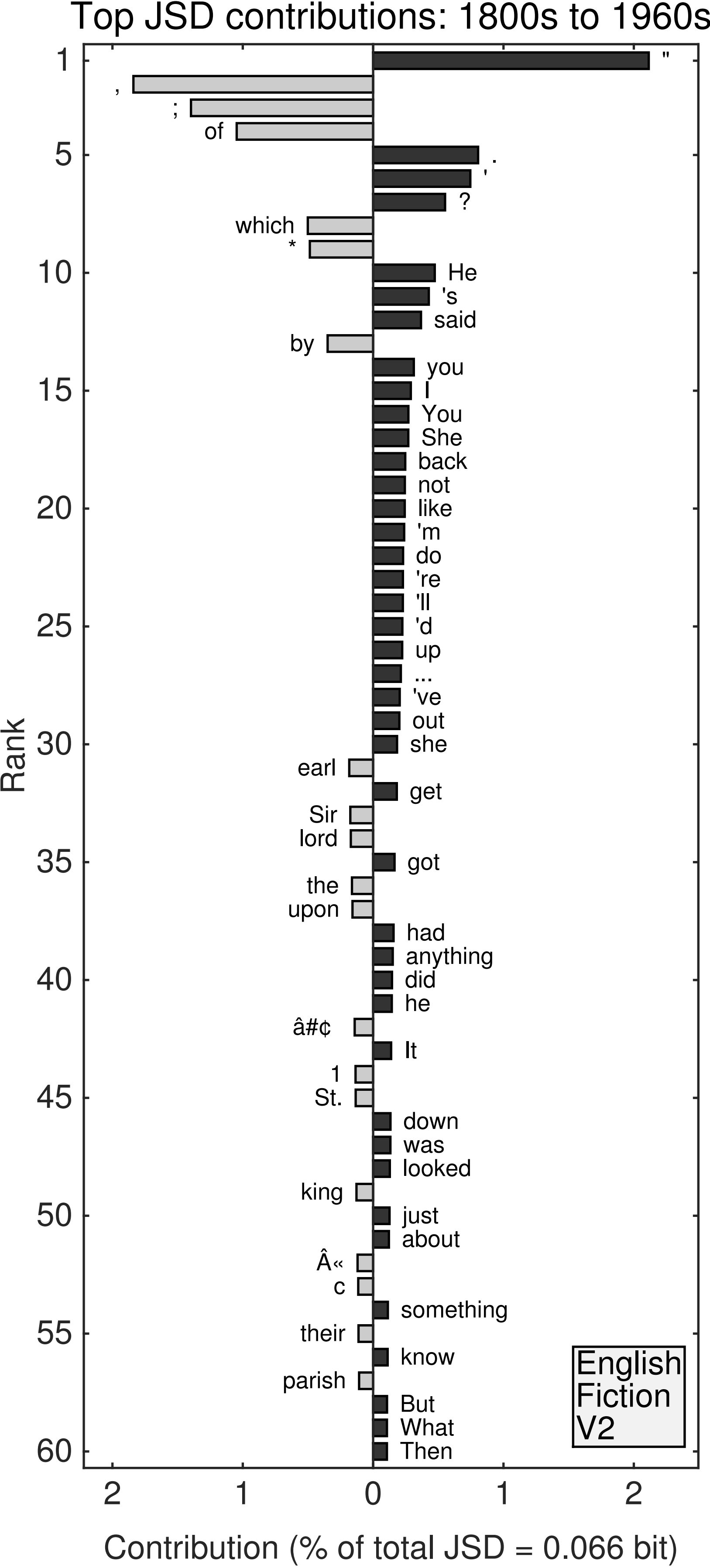
Top JSD contributions: 1800s to 1960s
Rank
Contribution (% of total JSD = 0.066 bit)
English Fiction V2
"
,
;
of
.
'
?
which
*
He
's
said
by
you
I
You
She
back
not
like
'm
do
're
'll
'd
up
...
've
out
she
earl
get
Sir
lord
got
the
upon
had
anything
did
he
â#¢
It
1
St.
down
was
looked
king
just
about
Â«
c
something
their
know
parish
But
What
Then

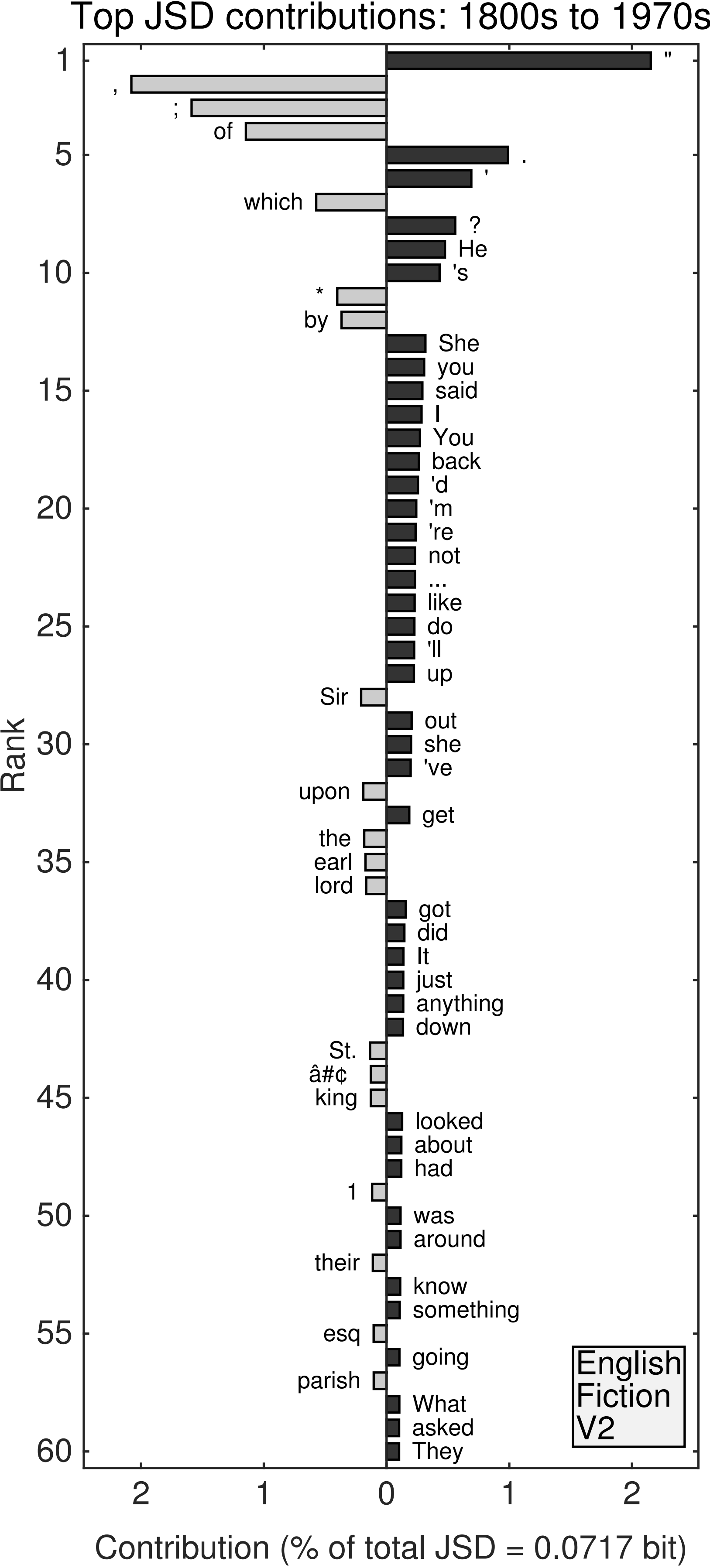

Top JSD contributions: 1800s to 1970s
Rank
Contribution (% of total JSD = 0.0717 bit)
English Fiction V2
"
,
;
of
.
'
which
?
He
's
*
by
She
you
said
I
You
back
'd
'm
're
not
...
like
do
'll
up
Sir
out
she
've
upon
get
the
earl
lord
got
did
It
just
anything
down
St.
â#¢
king
looked
about
had
1
was
around
their
know
something
esq
going
parish
What
asked
They

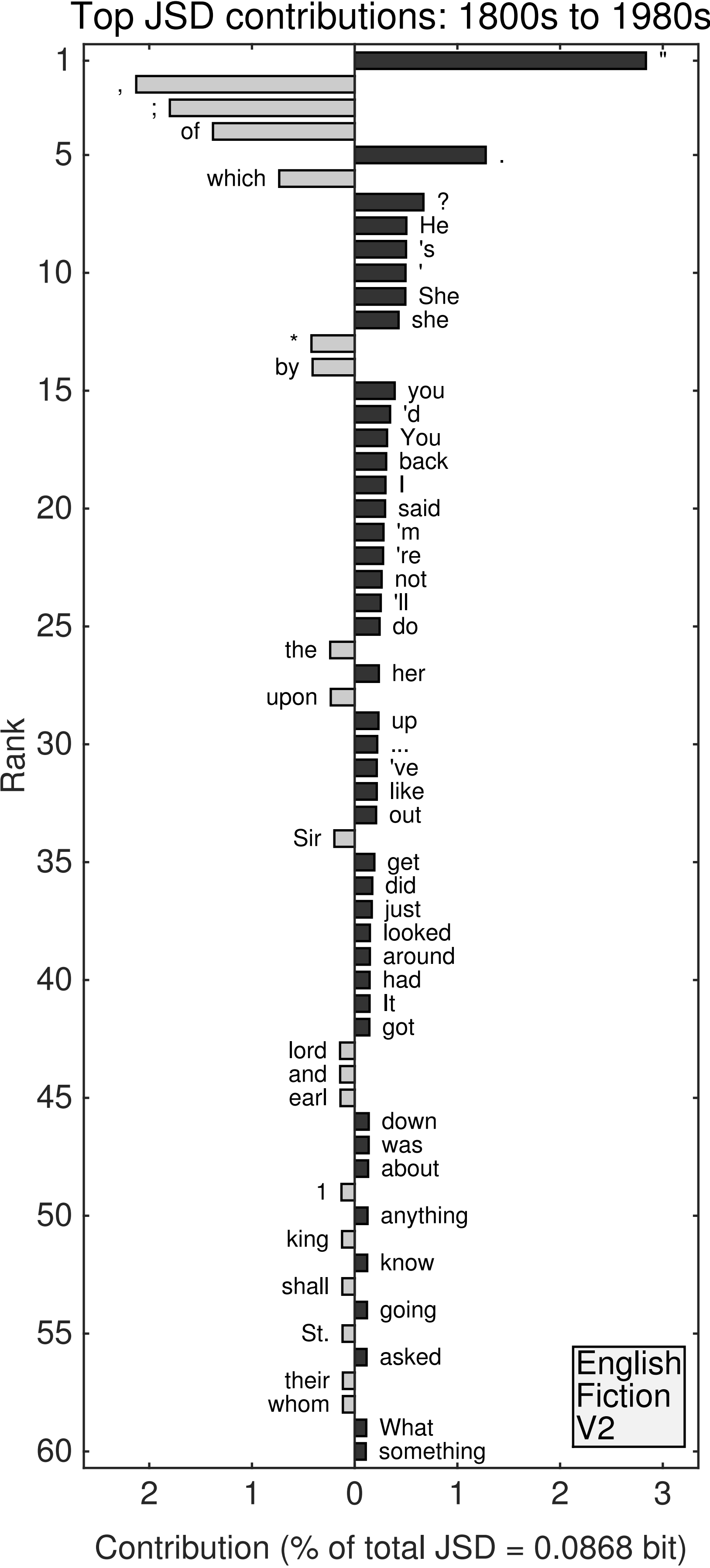

Top JSD contributions: 1800s to 1980s
Rank
Contribution (% of total JSD = 0.0868 bit)
English Fiction V2
"
,
;
of
.
which
?
He
's
'
She
she
*
by
you
'd
You
back
I
said
'm
're
not
'll
do
the
her
upon
up
...
've
like
out
Sir
get
did
just
looked
around
had
It
got
lord
and
earl
down
was
about
1
anything
king
know
shall
going
St.
asked
their
whom
What
something

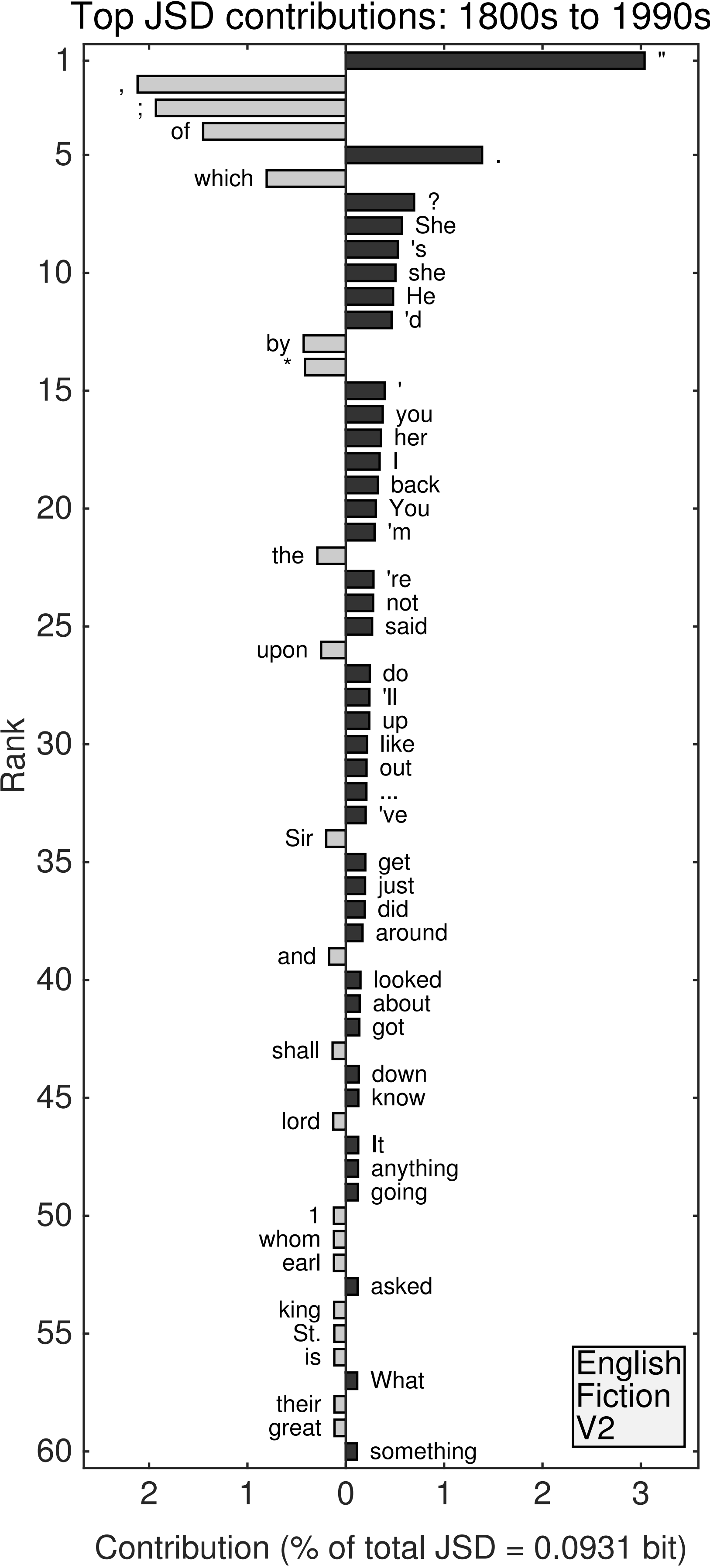

Top JSD contributions: 1800s to 1990s
Rank
"
,
;
of
.
which
?
She
's
she
He
'd
by
*
'
you
her
I
back
You
'm
the
're
not
said
upon
do
'll
up
like
out
...
've
Sir
get
just
did
around
and
looked
about
got
shall
down
know
lord
It
anything
going
1
whom
earl
asked
king
St.
is
What
their
great
something
English Fiction V2
Contribution (% of total JSD = 0.0931 bit)

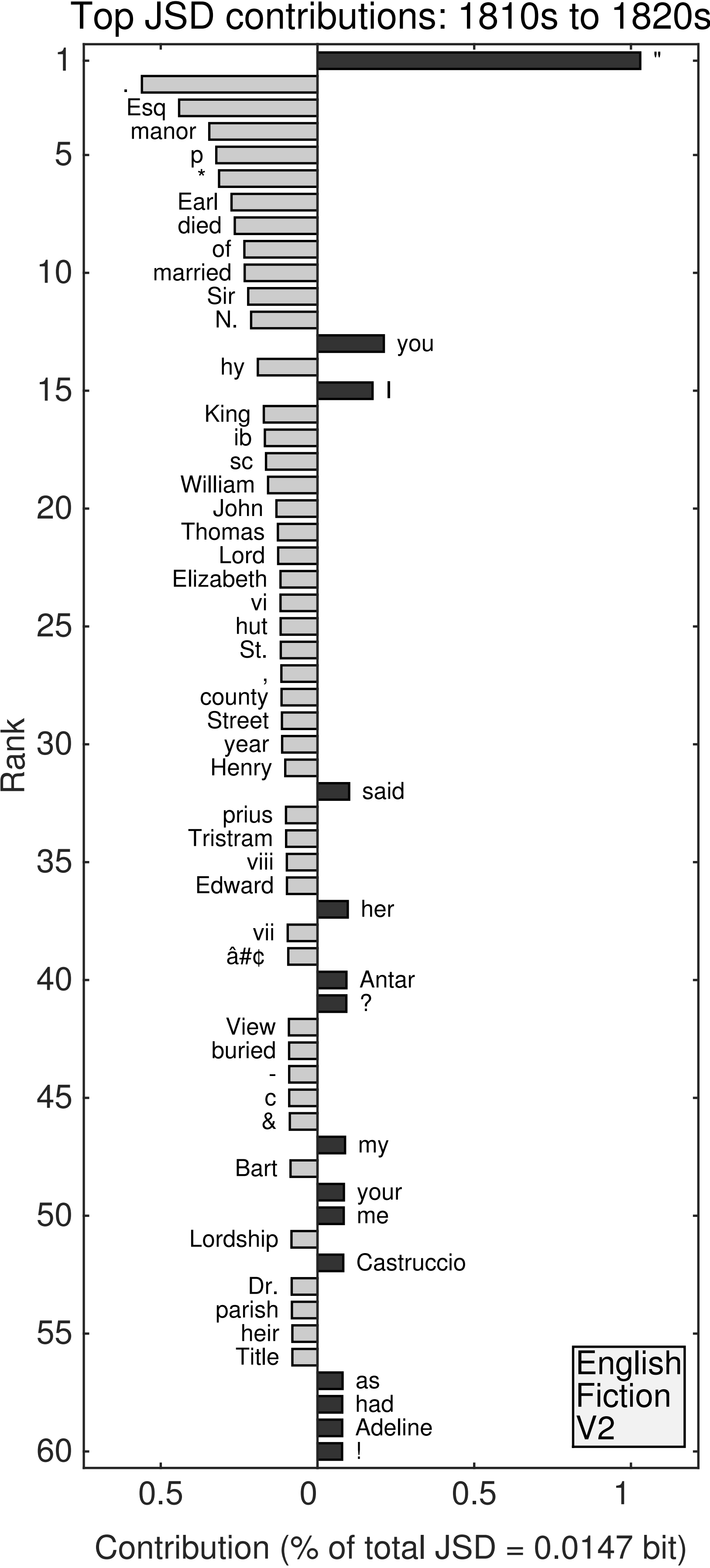

Top JSD contributions: 1810s to 1820s
Rank
Contribution (% of total JSD = 0.0147 bit)
English Fiction V2
"
.
Esq
manor
p
*
Earl
died
of
married
Sir
N.
you
hy
I
King
ib
sc
William
John
Thomas
Lord
Elizabeth
vi
hut
St.
,
county
Street
year
Henry
said
prius
Tristram
viii
Edward
her
vii
â#¢
Antar
?
View
buried
-
c
&
my
Bart
your
me
Lordship
Castruccio
Dr.
parish
heir
Title
as
had
Adeline
!

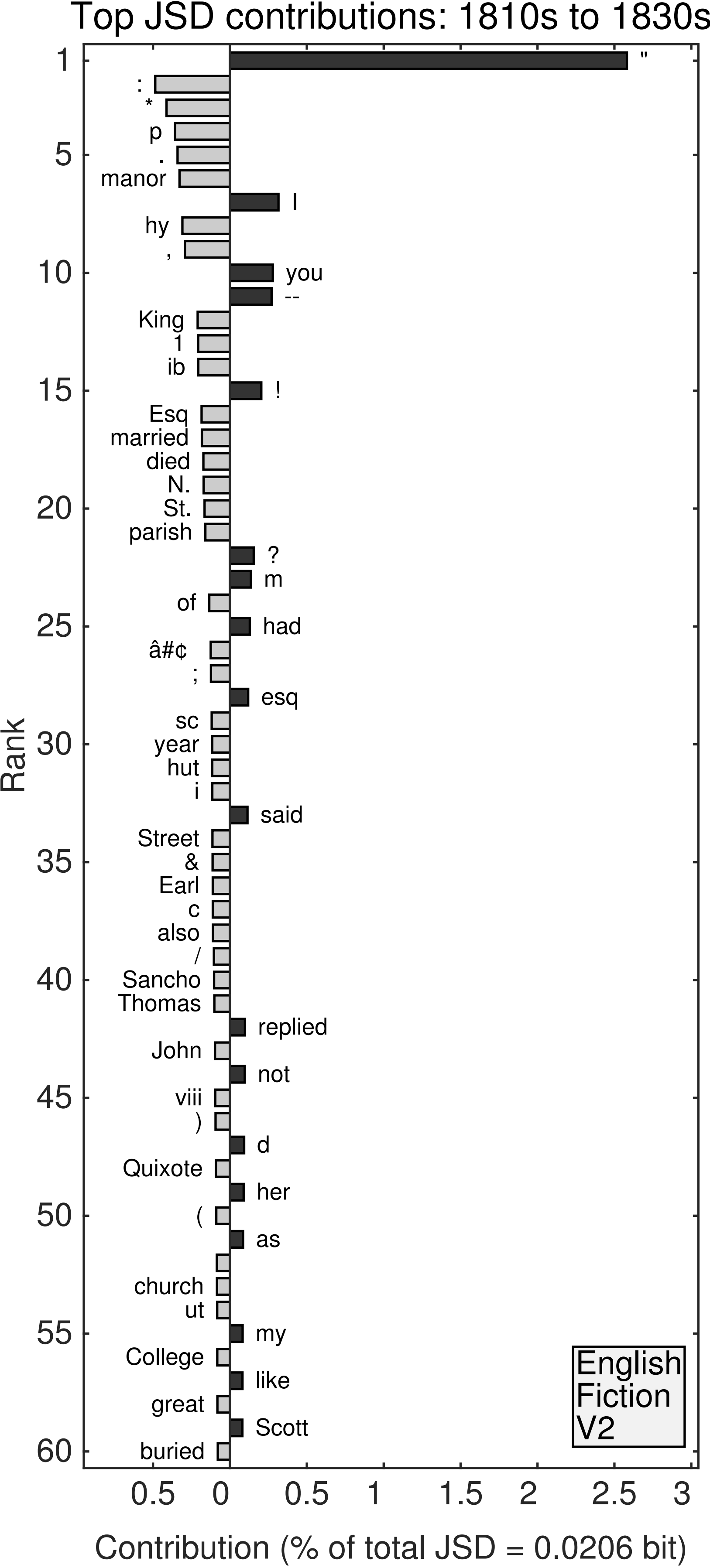

Top JSD contributions: 1810s to 1830s
Rank
Contribution (% of total JSD = 0.0206 bit)
0.5
0
0.5
1
1.5
2
2.5
3
1
5
10
15
20
25
30
35
40
45
50
55
60
"
:
*
p
.
manor
I
hy
,
you
--
King
1
ib
!
Esq
married
died
N.
St.
parish
?
m
of
had
â#¢
;
esq
sc
year
hut
i
said
Street
&
Earl
c
also
/
Sancho
Thomas
replied
John
not
viii
)
d
Quixote
her
(
as
church
ut
my
College
like
great
Scott
buried
English
Fiction
V2

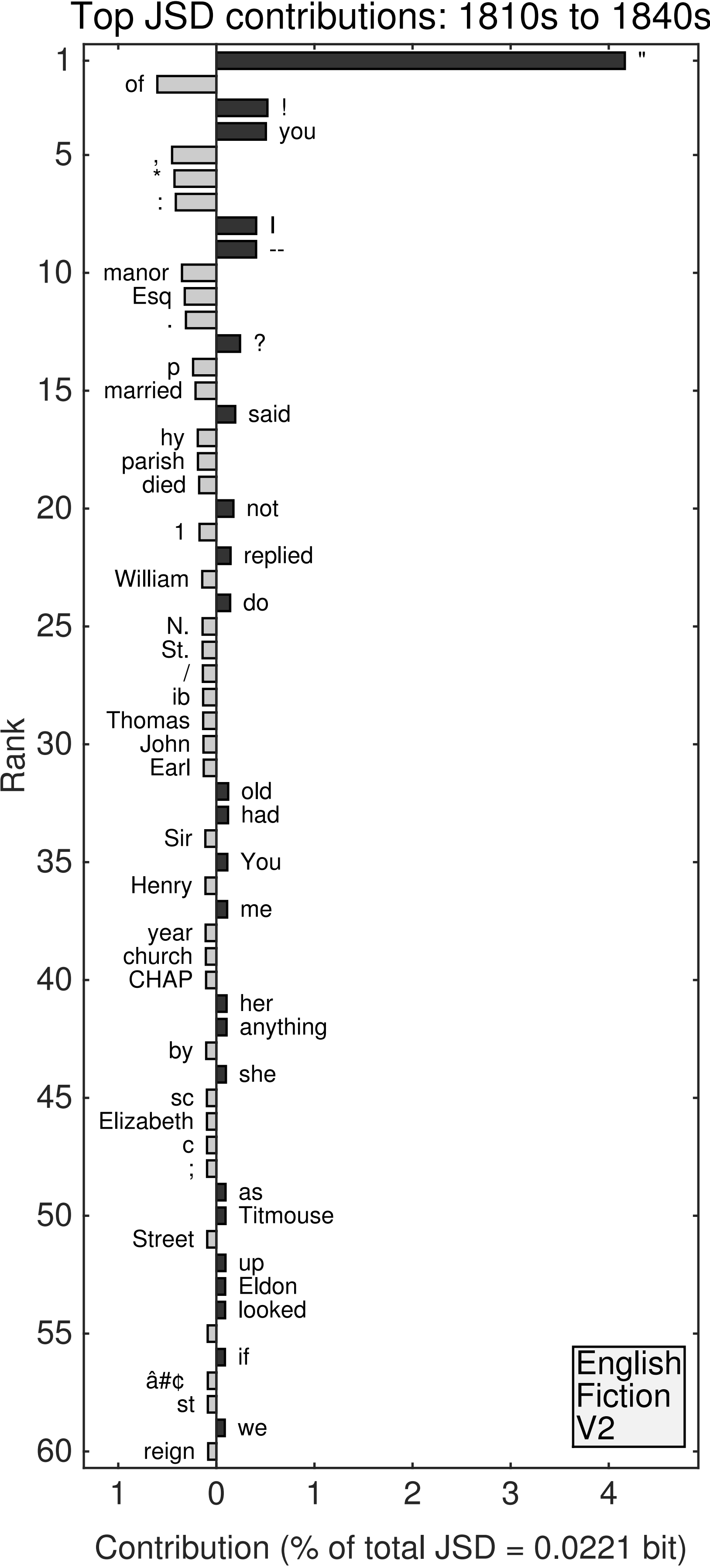

Top JSD contributions: 1810s to 1840s
Rank
Contribution (% of total JSD = 0.0221 bit)
English Fiction V2
"
of
!
you
,
*
:
I
--
manor
Esq
.
?
p
married
said
hy
parish
died
not
1
replied
William
do
N.
St.
/
ib
Thomas
John
Earl
old
had
Sir
You
Henry
me
year
church
CHAP
her
anything
by
she
sc
Elizabeth
c
;
as
Titmouse
Street
up
Eldon
looked
if
â#¢
st
we
reign

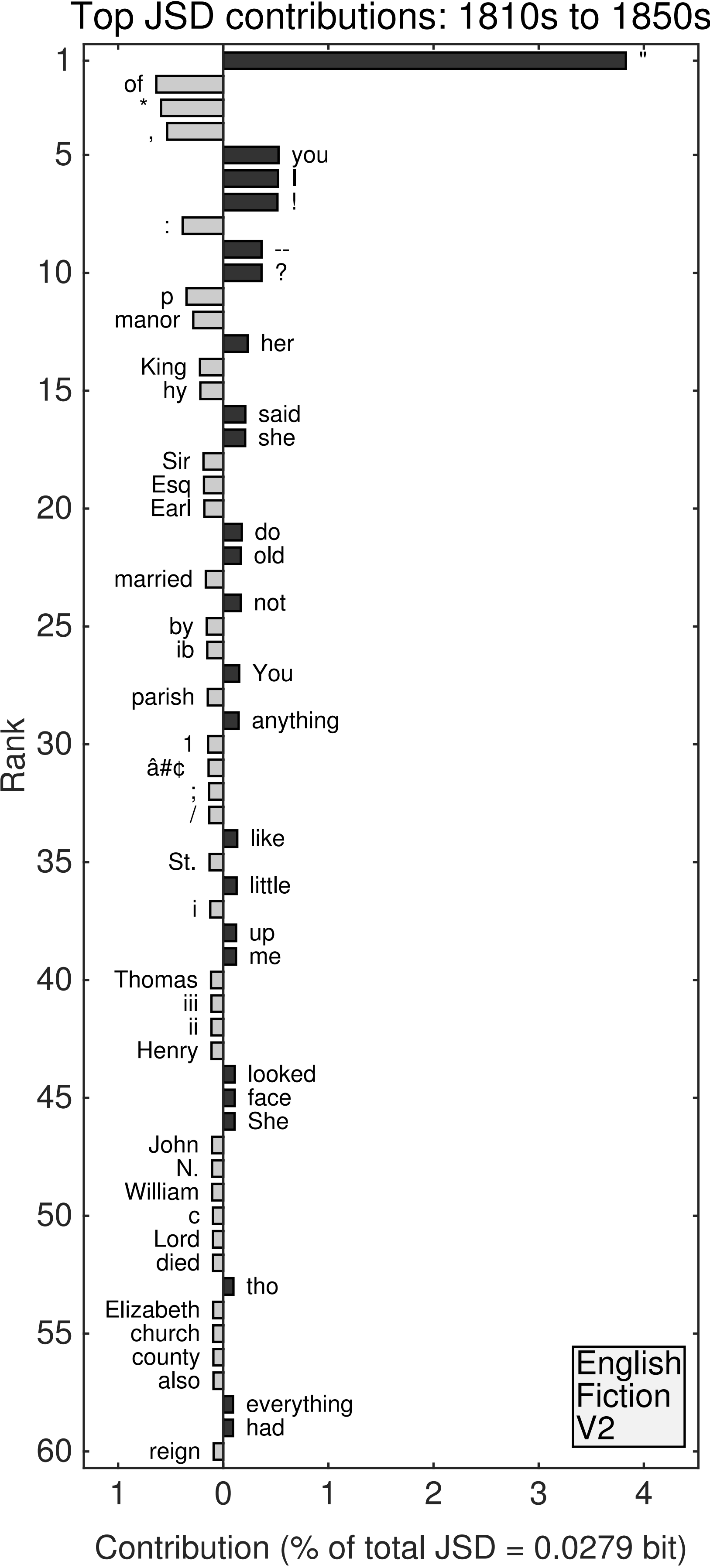

Top JSD contributions: 1810s to 1850s
Rank
Contribution (% of total JSD = 0.0279 bit)
"
of
*
,
you
I
!
:
--
?
p
manor
her
King
hy
said
she
Sir
Esq
Earl
do
old
married
not
by
ib
You
parish
anything
1
â#¢
;
/
like
St.
little
i
up
me
Thomas
iii
ii
Henry
looked
face
She
John
N.
William
c
Lord
died
tho
Elizabeth
church
county
also
everything
had
reign
English Fiction V2

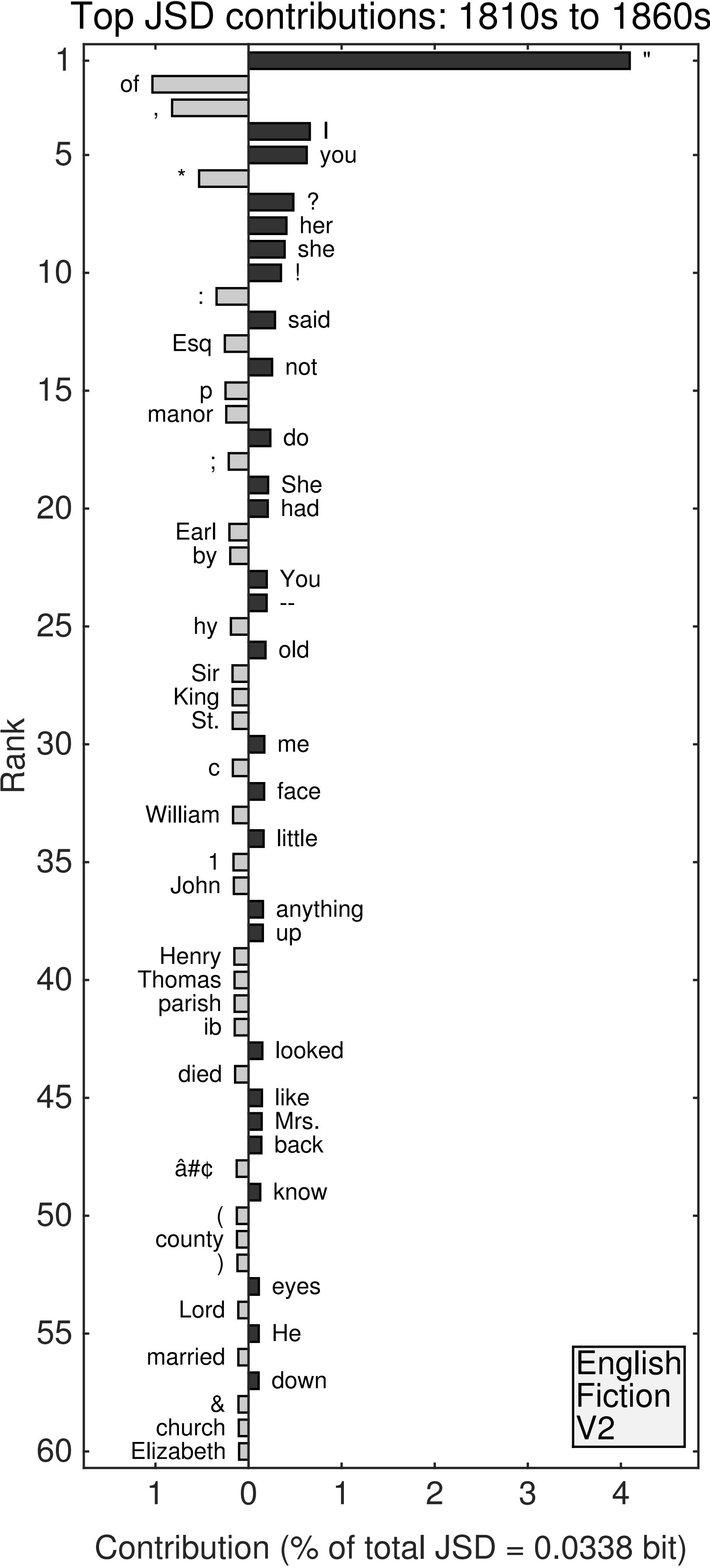

Top JSD contributions: 1810s to 1860s
Rank
Contribution (% of total JSD = 0.0338 bit)
English Fiction V2
"
of
,
I
you
*
?
her
she
!
:
said
Esq
not
p
manor
do
;
She
had
Earl
by
You
--
hy
old
Sir
King
St.
me
c
face
William
little
1
John
anything
up
Henry
Thomas
parish
ib
looked
died
like
Mrs.
back
â#¢
know
(
county
)
eyes
Lord
He
married
down
&
church
Elizabeth
1
5
10
15
20
25
30
35
40
45
50
55
60
1
0
1
2
3
4

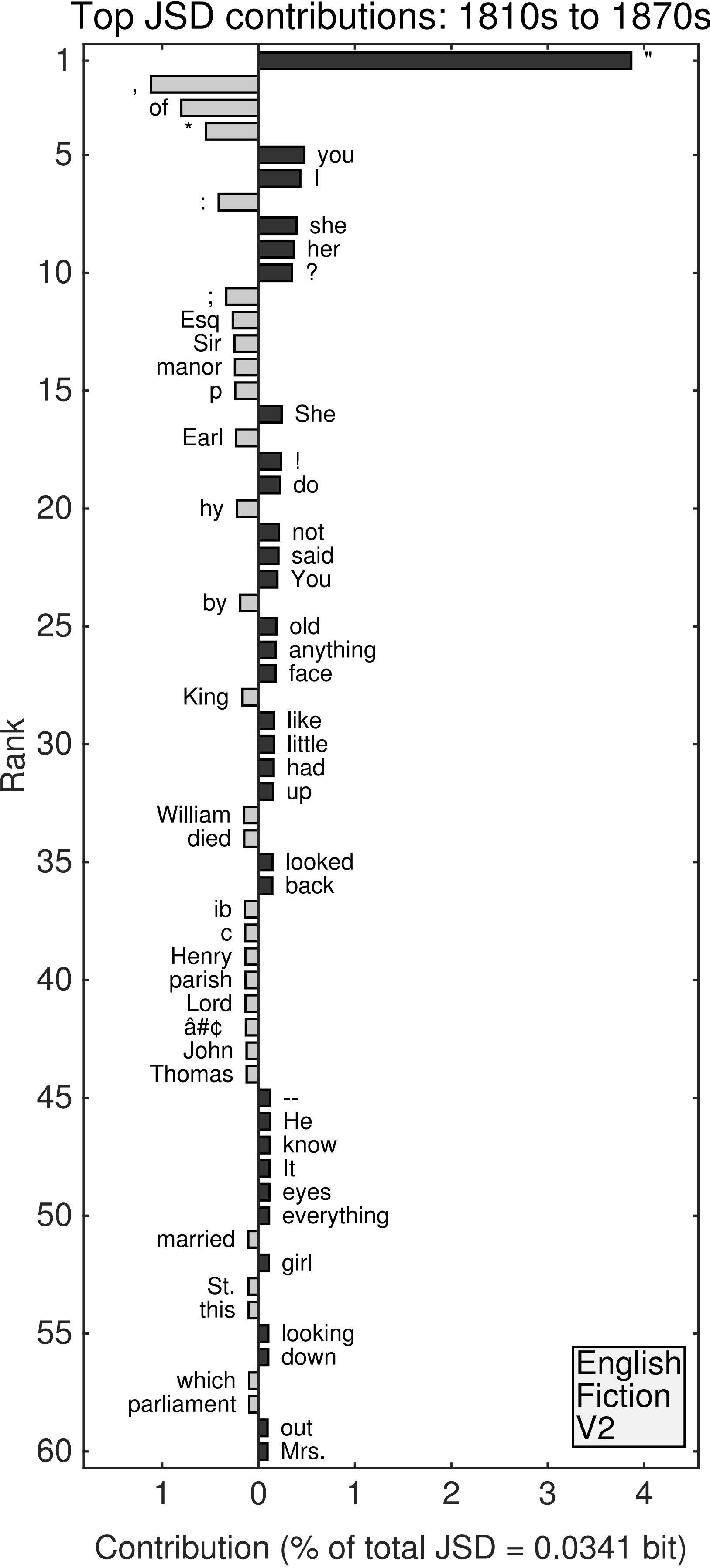
Top JSD contributions: 1810s to 1870s
Rank
Contribution (% of total JSD = 0.0341 bit)
English Fiction V2
"
,
of
*
you
I
:
she
her
?
;
Esq
Sir
manor
p
She
Earl
!
do
hy
not
said
You
by
old
anything
face
King
like
little
had
up
William
died
looked
back
ib
c
Henry
parish
Lord
â#¢
John
Thomas
--
He
know
It
eyes
everything
married
girl
St.
this
looking
down
which
parliament
out
Mrs.

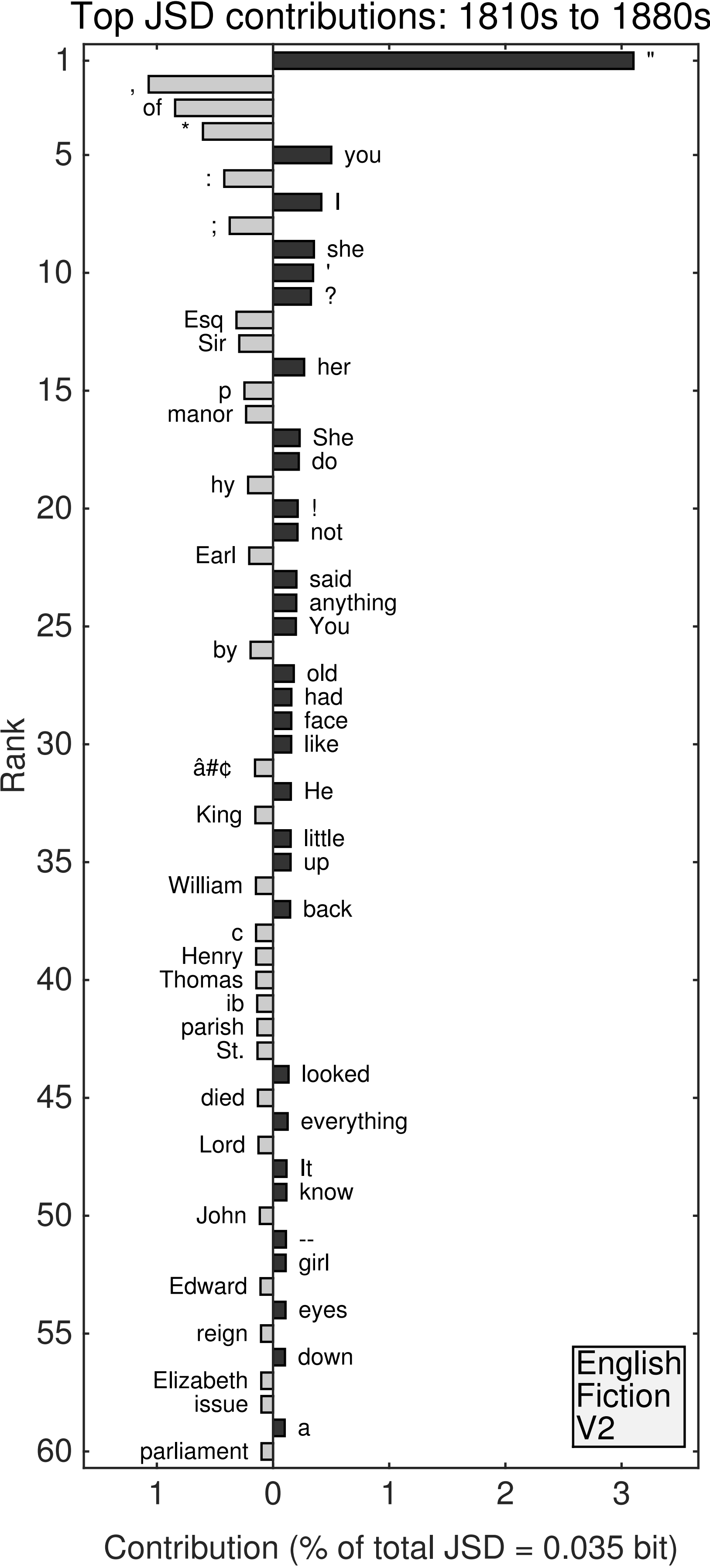

Top JSD contributions: 1810s to 1880s
Rank
Contribution (% of total JSD = 0.035 bit)
English Fiction V2
"
,
of
*
you
:
I
;
she
'
?
Esq
Sir
her
p
manor
She
do
hy
!
not
Earl
said
anything
You
by
old
had
face
like
â#¢
He
King
little
up
William
back
c
Henry
Thomas
ib
parish
St.
looked
died
everything
Lord
It
know
John
--
girl
Edward
eyes
reign
down
Elizabeth
issue
a
parliament

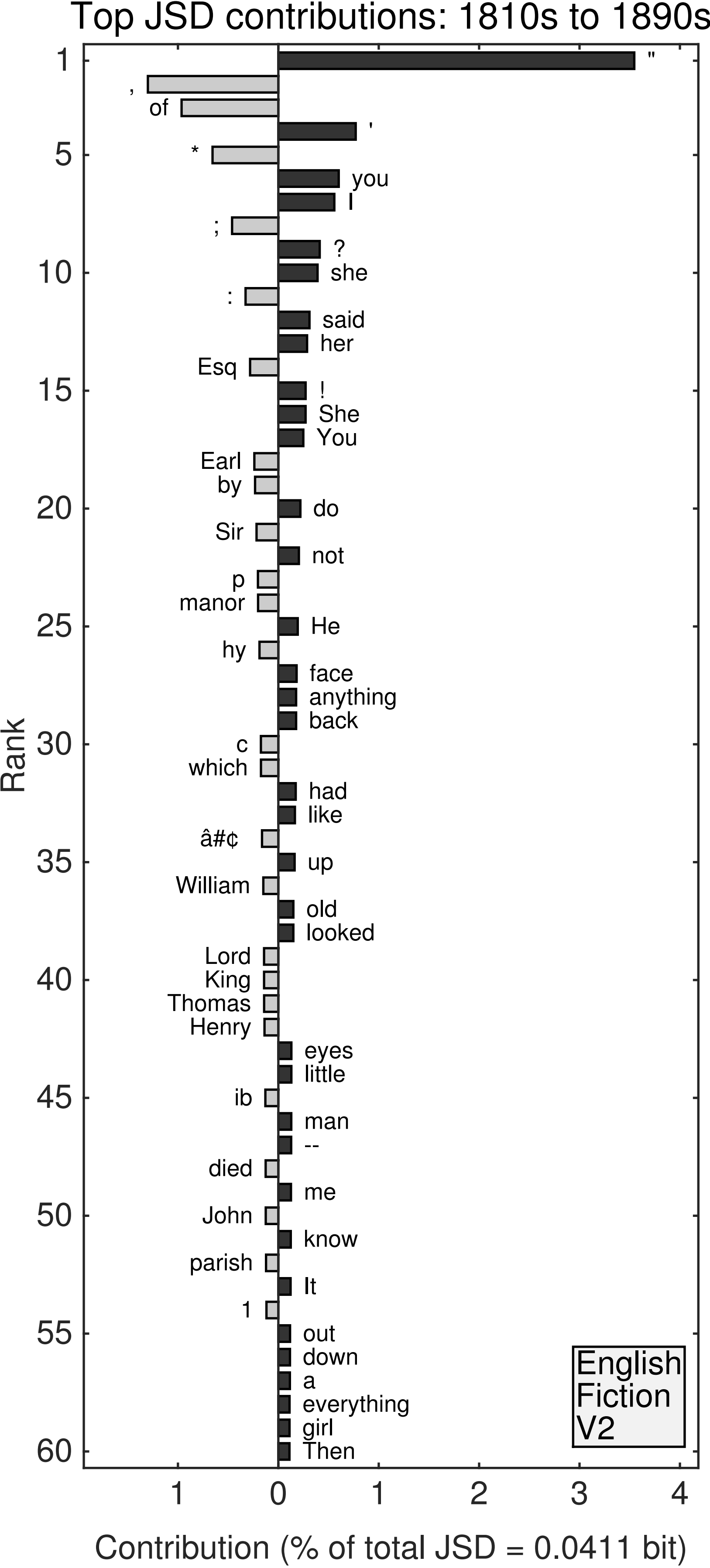
Top JSD contributions: 1810s to 1890s
Rank
"
,
of
'
*
you
I
;
?
she
:
said
her
Esq
!
She
You
Earl
by
do
Sir
not
p
manor
He
hy
face
anything
back
c
which
had
like
â#¢
up
William
old
looked
Lord
King
Thomas
Henry
eyes
little
ib
man
--
died
me
John
know
parish
It
1
out
down
a
everything
girl
Then
English
Fiction
V2
Contribution (% of total JSD = 0.0411 bit)

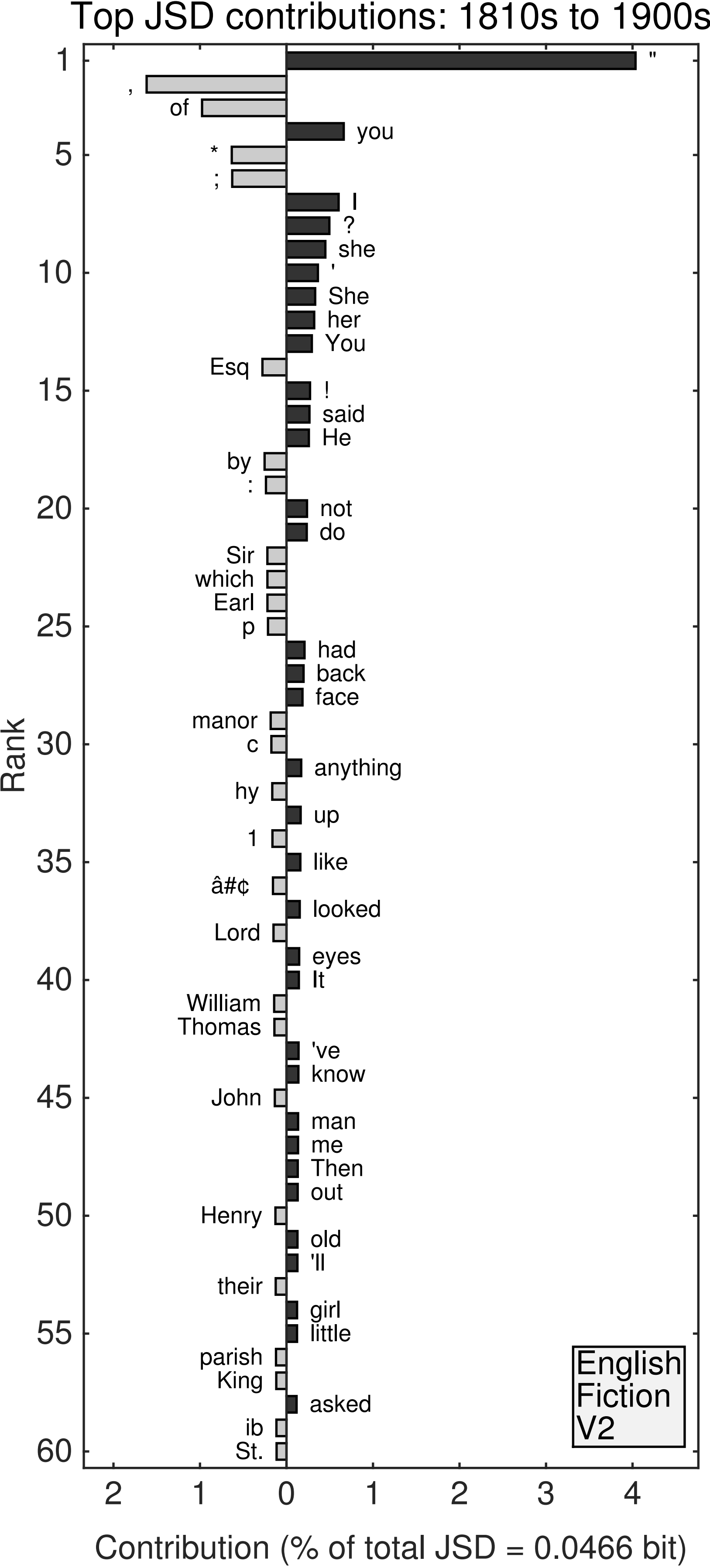
Top JSD contributions: 1810s to 1900s
Rank
Contribution (% of total JSD = 0.0466 bit)
English Fiction V2
"
,
of
you
*
;
I
?
she
'
She
her
You
Esq
!
said
He
by
:
not
do
Sir
which
Earl
p
had
back
face
manor
c
anything
hy
up
1
like
â#¢
looked
Lord
eyes
It
William
Thomas
've
know
John
man
me
Then
out
Henry
old
'll
their
girl
little
parish
King
asked
ib
St.

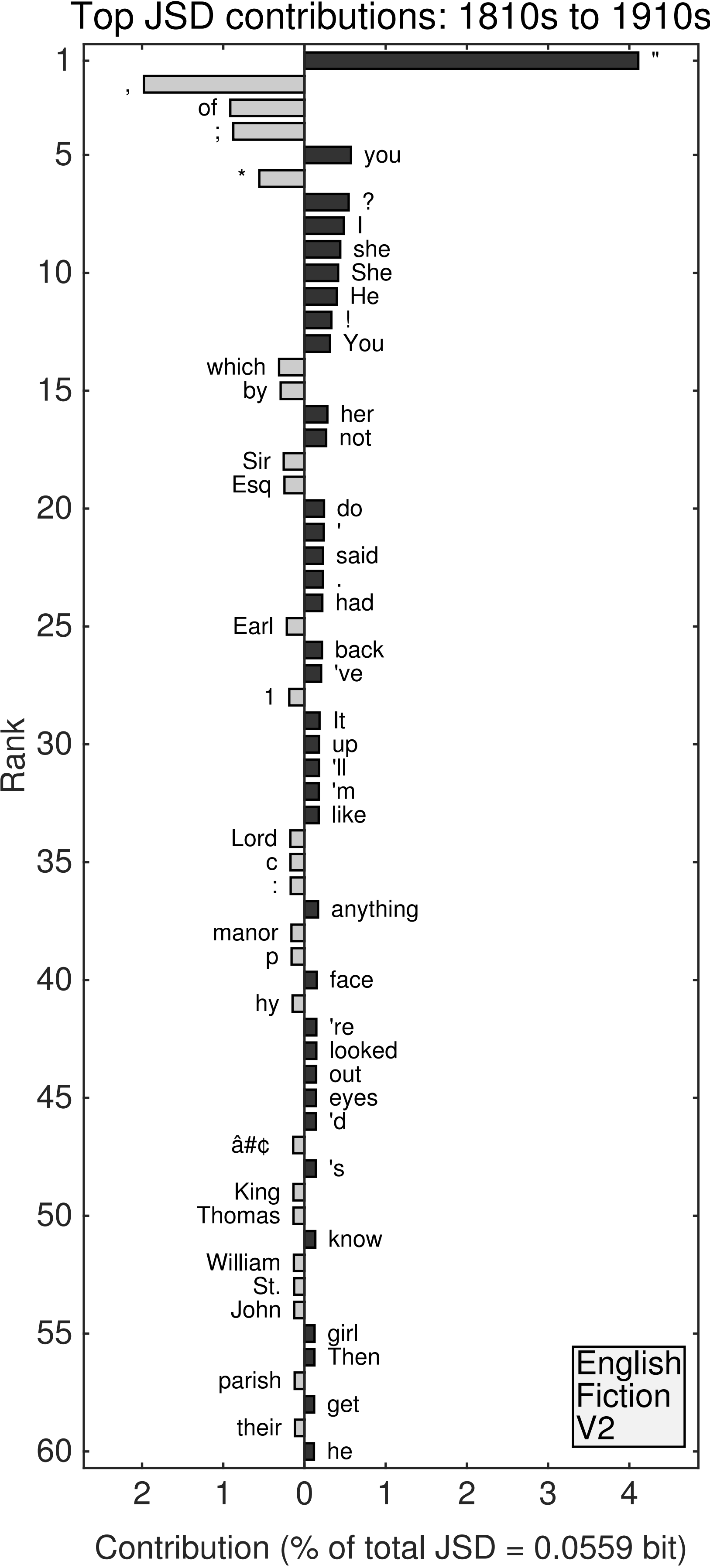
Top JSD contributions: 1810s to 1910s
Rank
Contribution (% of total JSD = 0.0559 bit)
"
,
of
;
you
*
?
I
she
She
He
!
You
which
by
her
not
Sir
Esq
do
'
said
.
had
Earl
back
've
1
It
up
'll
'm
like
Lord
c
:
anything
manor
p
face
hy
're
looked
out
eyes
'd
â#¢
's
King
Thomas
know
William
St.
John
girl
Then
parish
get
their
he
English Fiction V2

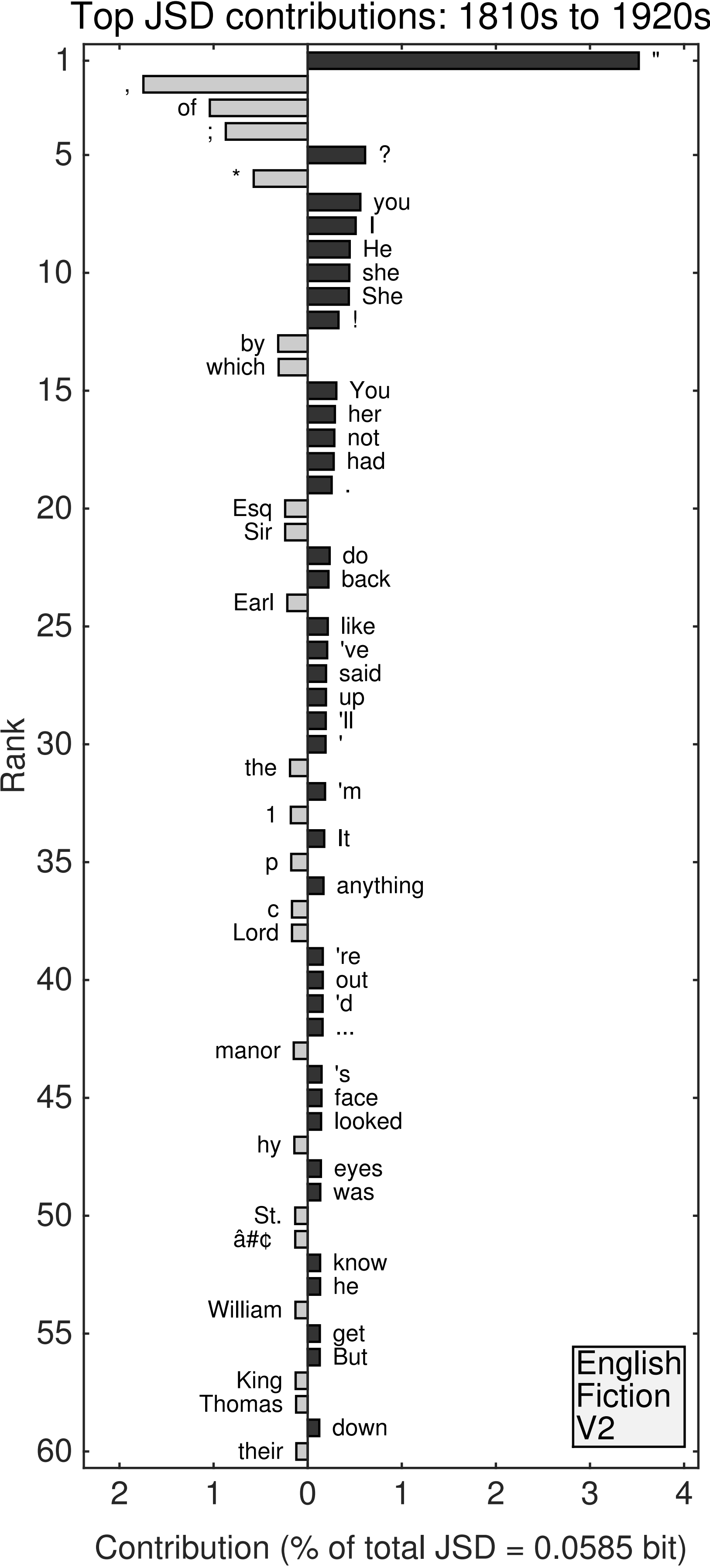

Top JSD contributions: 1810s to 1920s
Rank
Contribution (% of total JSD = 0.0585 bit)
English Fiction V2
"
,
of
;
?
*
you
I
He
she
She
!
by
which
You
her
not
had
.
Esq
Sir
do
back
Earl
like
've
said
up
'll
'
the
'm
1
It
p
anything
c
Lord
're
out
'd
...
manor
's
face
looked
hy
eyes
was
St.
â#¢
know
he
William
get
But
King
Thomas
down
their

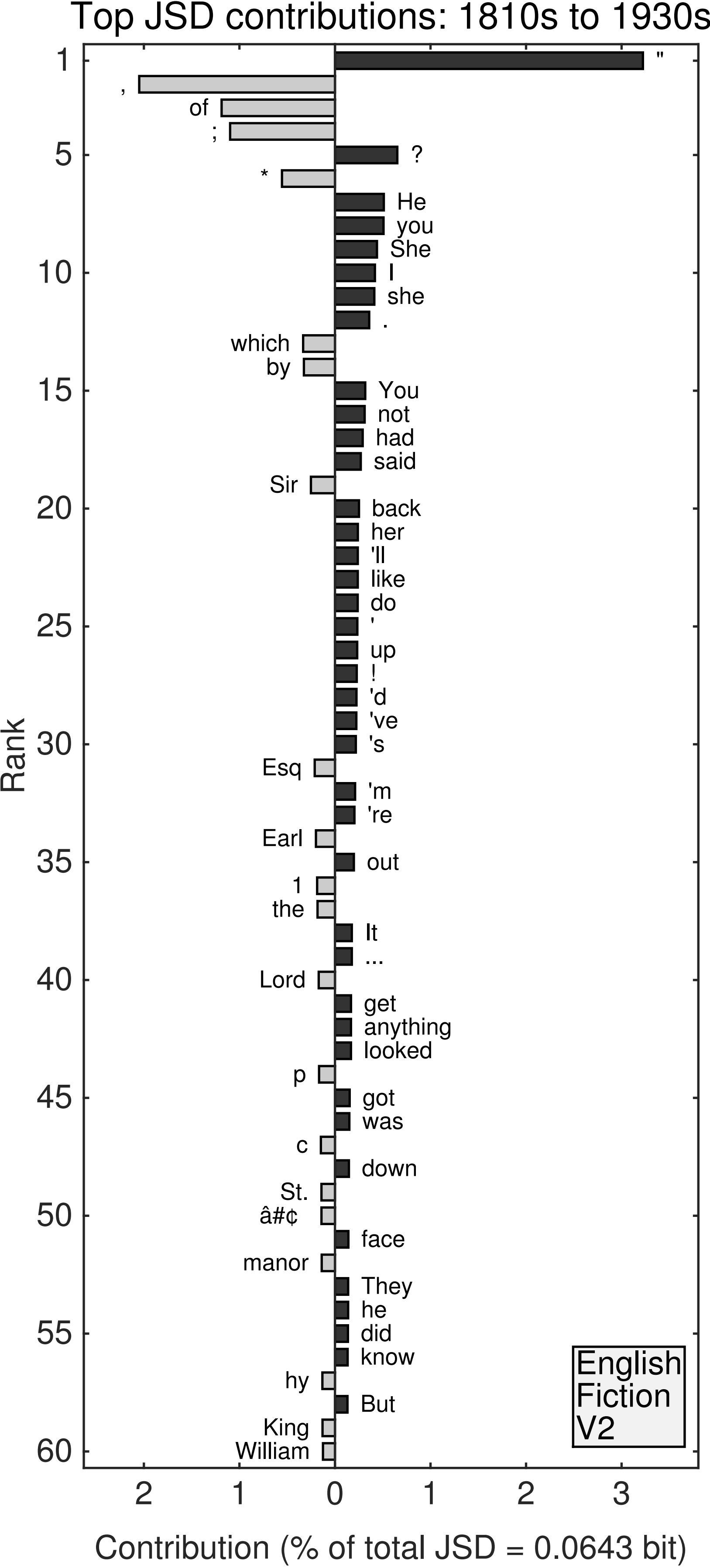
Top JSD contributions: 1810s to 1930s
Rank
Contribution (% of total JSD = 0.0643 bit)
"
,
of
;
?
*
He
you
She
I
she
.
which
by
You
not
had
said
Sir
back
her
'll
like
do
'
up
!
'd
've
's
Esq
'm
're
Earl
out
1
the
It
...
Lord
get
anything
looked
p
got
was
c
down
St.
â#¢
face
manor
They
he
did
know
hy
But
King
William
English
Fiction
V2

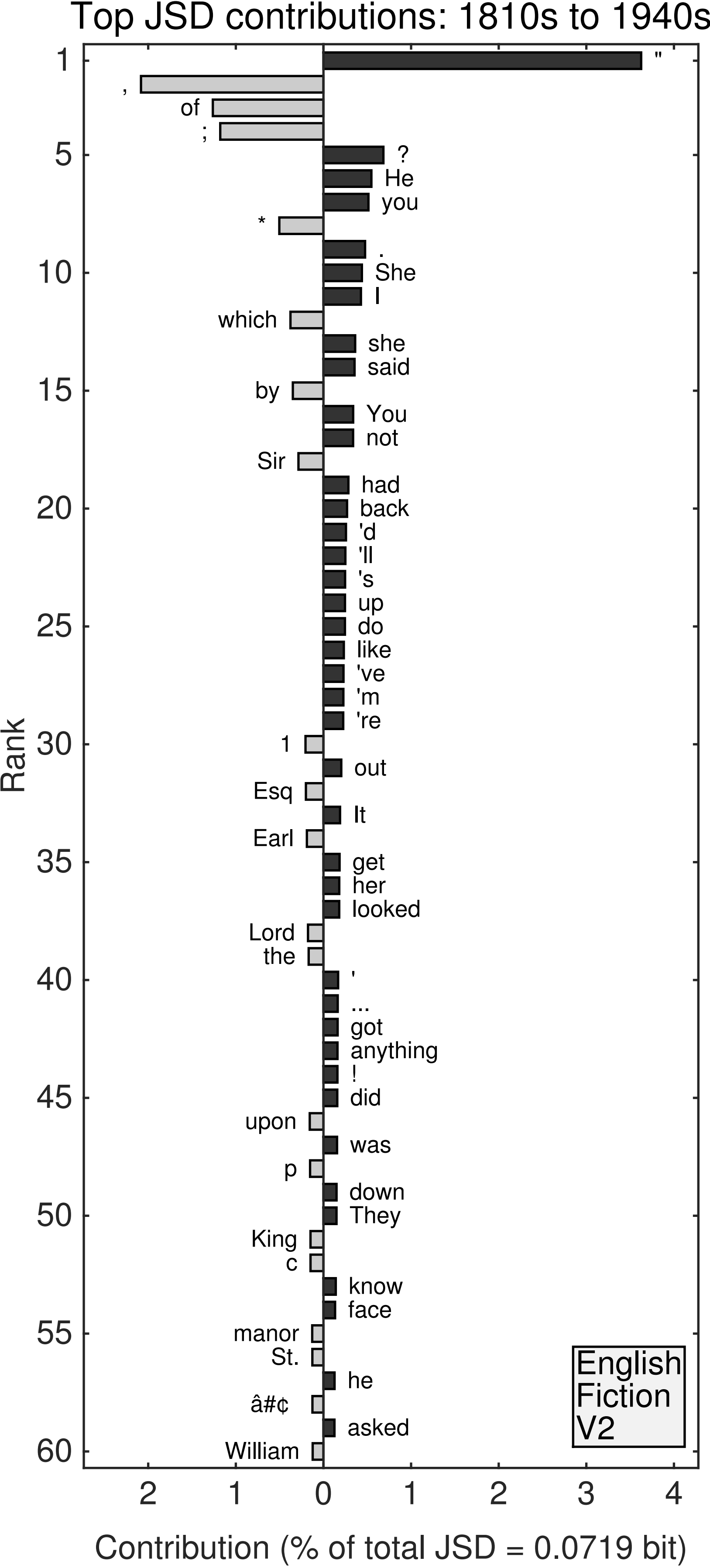
Top JSD contributions: 1810s to 1940s
Rank
Contribution (% of total JSD = 0.0719 bit)
"
,
of
;
?
He
you
*
.
She
I
which
she
said
by
You
not
Sir
had
back
'd
'll
's
up
do
like
've
'm
're
1
out
Esq
It
Earl
get
her
looked
Lord
the
'
...
got
anything
!
did
upon
was
p
down
They
King
c
know
face
manor
St.
he
â#¢
asked
William
English Fiction V2

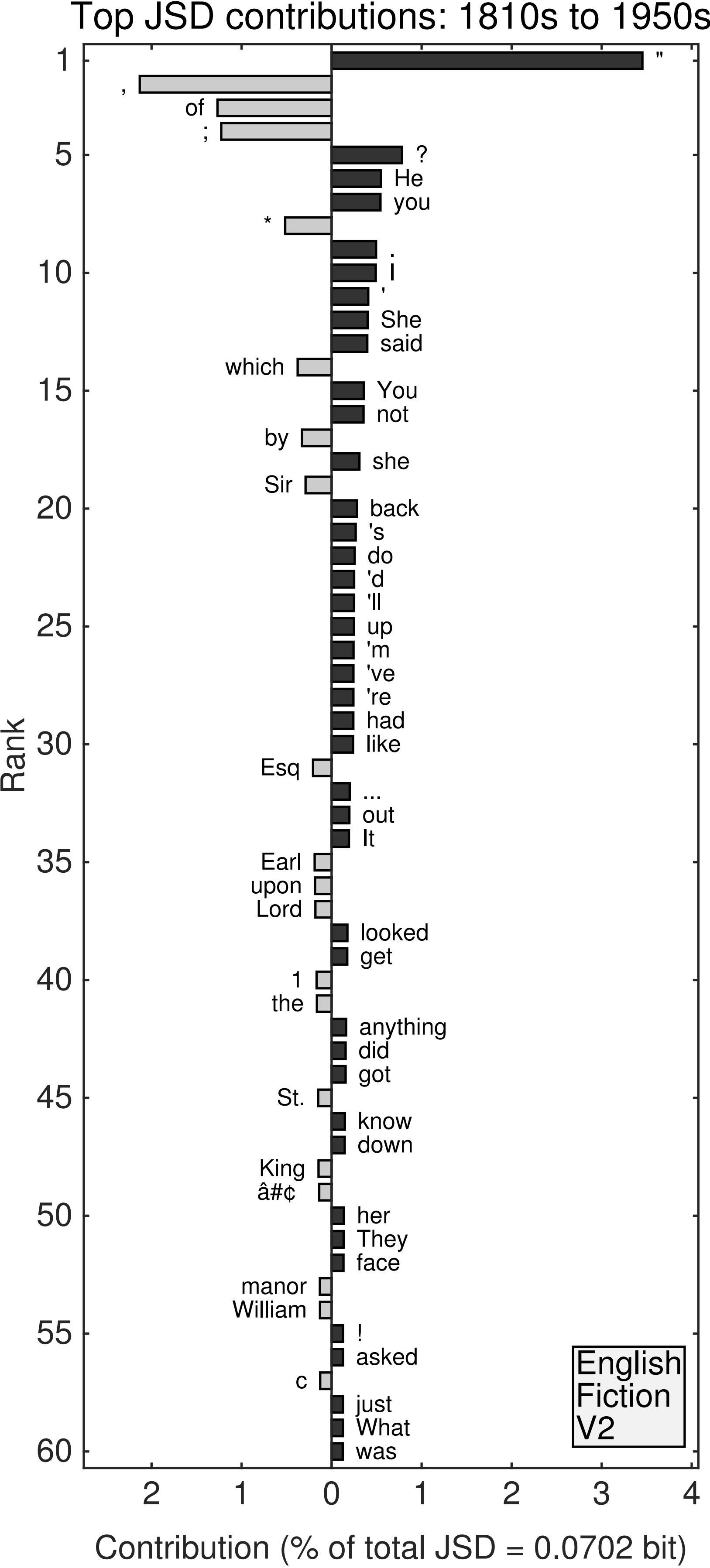
Top JSD contributions: 1810s to 1950s
Rank
Contribution (% of total JSD = 0.0702 bit)
English Fiction V2
"
,
of
;
?
He
you
*
.
i
'
She
said
which
You
not
by
she
Sir
back
's
do
'd
'll
up
'm
've
're
had
like
Esq
...
out
It
Earl
upon
Lord
looked
get
1
the
anything
did
got
St.
know
down
King
â#¢
her
They
face
manor
William
!
asked
c
just
What
was

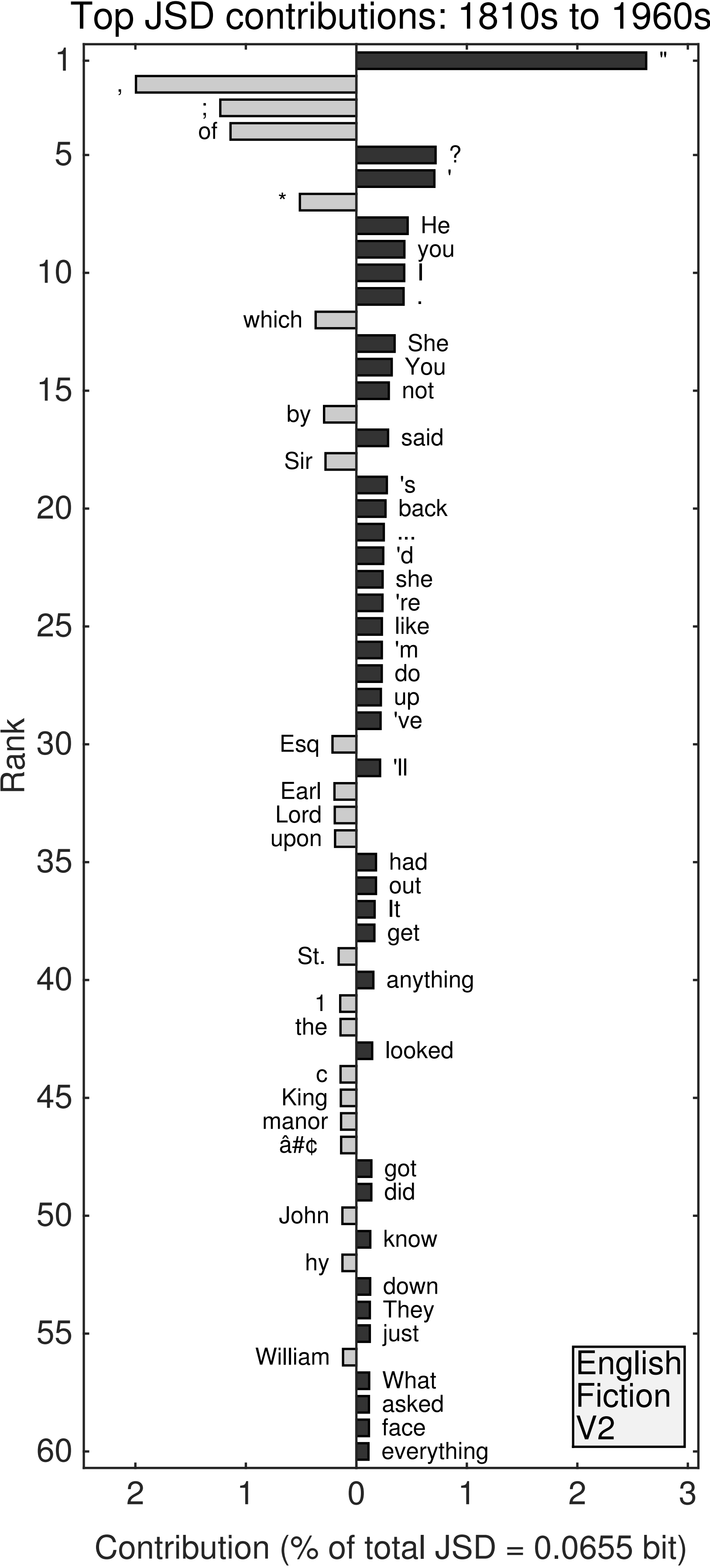
Top JSD contributions: 1810s to 1960s
Rank
Contribution (% of total JSD = 0.0655 bit)
English Fiction V2
"
,
;
of
?
'
*
He
you
I
.
which
She
You
not
by
said
Sir
's
back
...
'd
she
're
like
'm
do
up
've
Esq
'll
Earl
Lord
upon
had
out
It
get
St.
anything
1
the
looked
c
King
manor
â#¢
got
did
John
know
hy
down
They
just
William
What
asked
face
everything

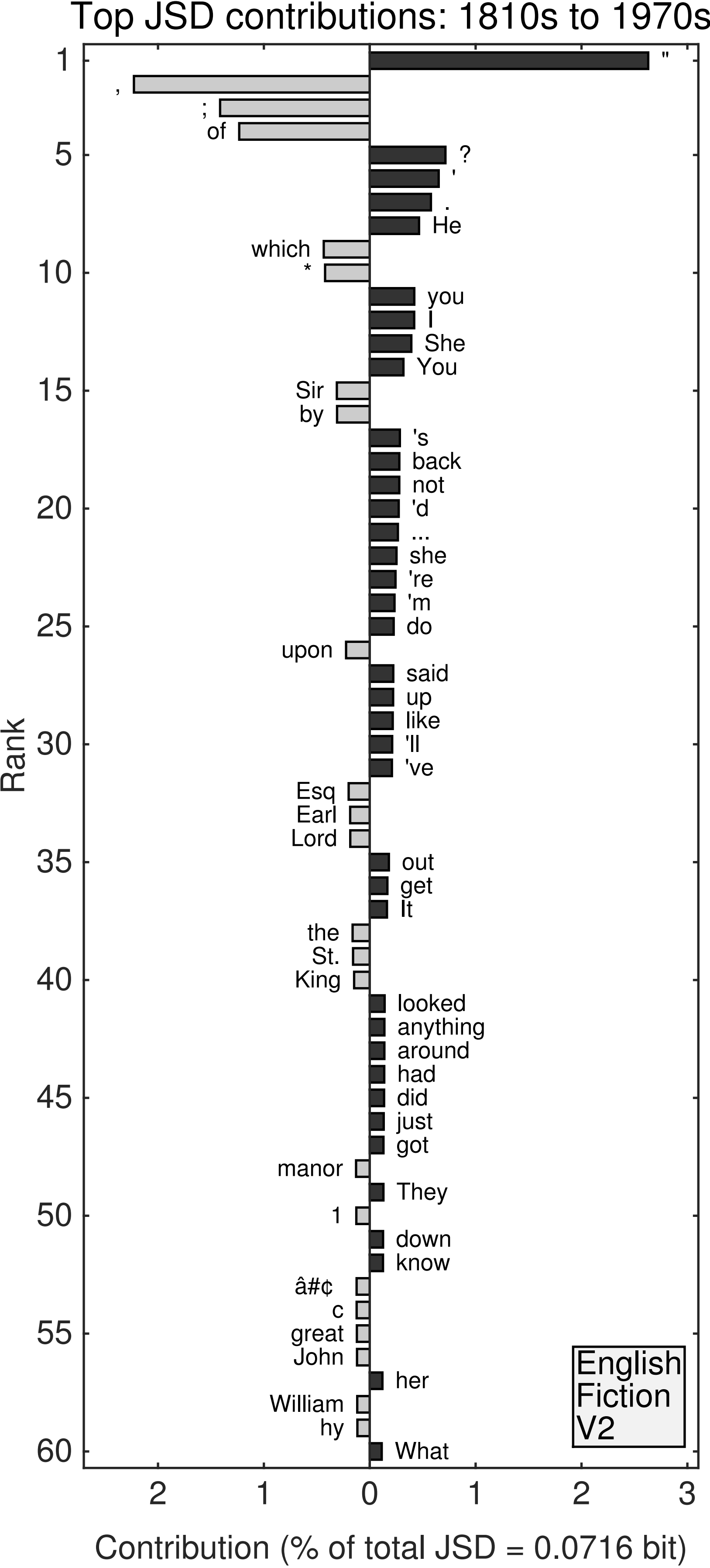
Top JSD contributions: 1810s to 1970s
Rank
"
,
;
of
?
'
.
He
which
*
you
I
She
You
Sir
by
's
back
not
'd
...
she
're
'm
do
upon
said
up
like
'll
've
Esq
Earl
Lord
out
get
It
the
St.
King
looked
anything
around
had
did
just
got
manor
They
1
down
know
â#¢
c
great
John
her
William
hy
What
English Fiction V2
Contribution (% of total JSD = 0.0716 bit)

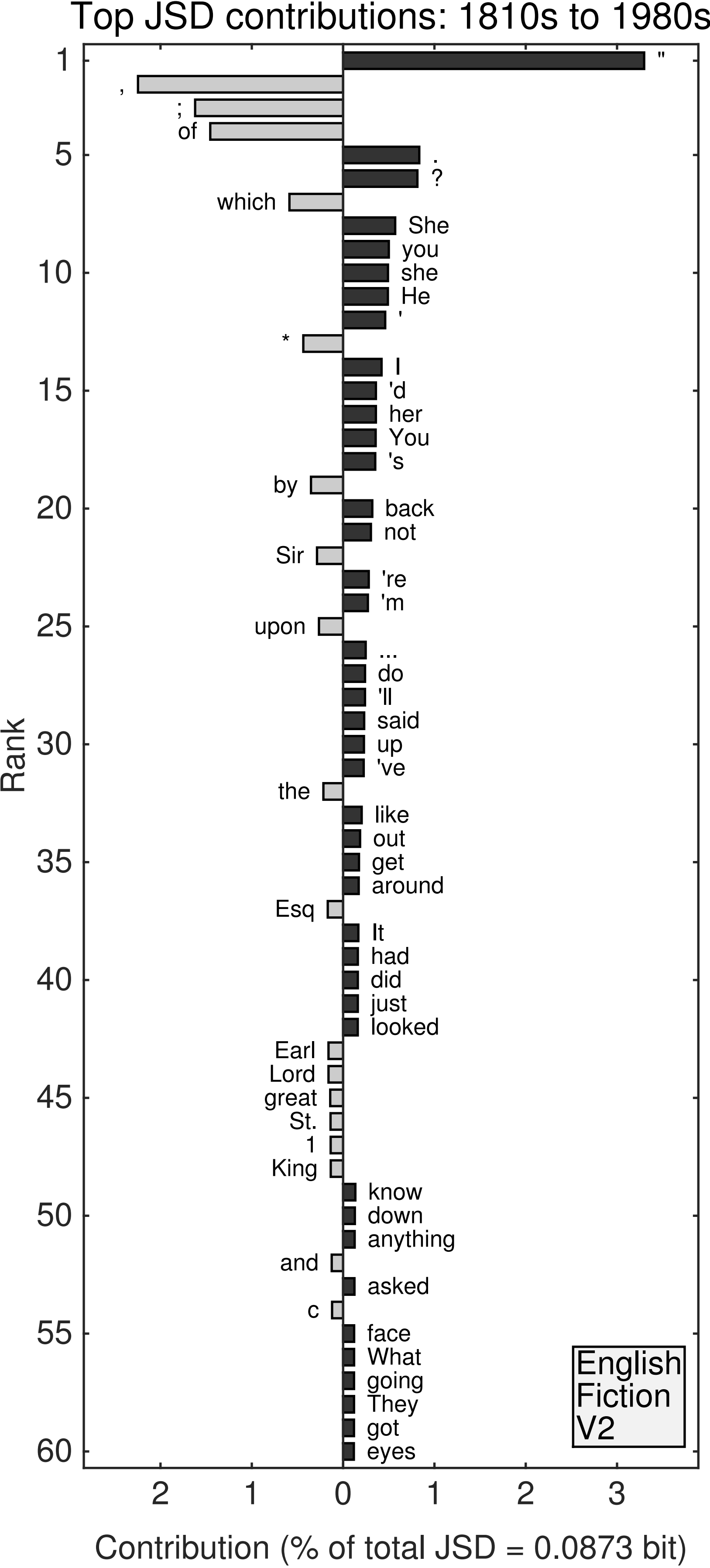
Top JSD contributions: 1810s to 1980s
Rank
Contribution (% of total JSD = 0.0873 bit)
English Fiction V2
"
,
;
of
.
?
which
She
you
she
He
'
*
I
'd
her
You
's
by
back
not
Sir
're
'm
upon
...
do
'll
said
up
've
the
like
out
get
around
Esq
It
had
did
just
looked
Earl
Lord
great
St.
1
King
know
down
anything
and
asked
c
face
What
going
They
got
eyes

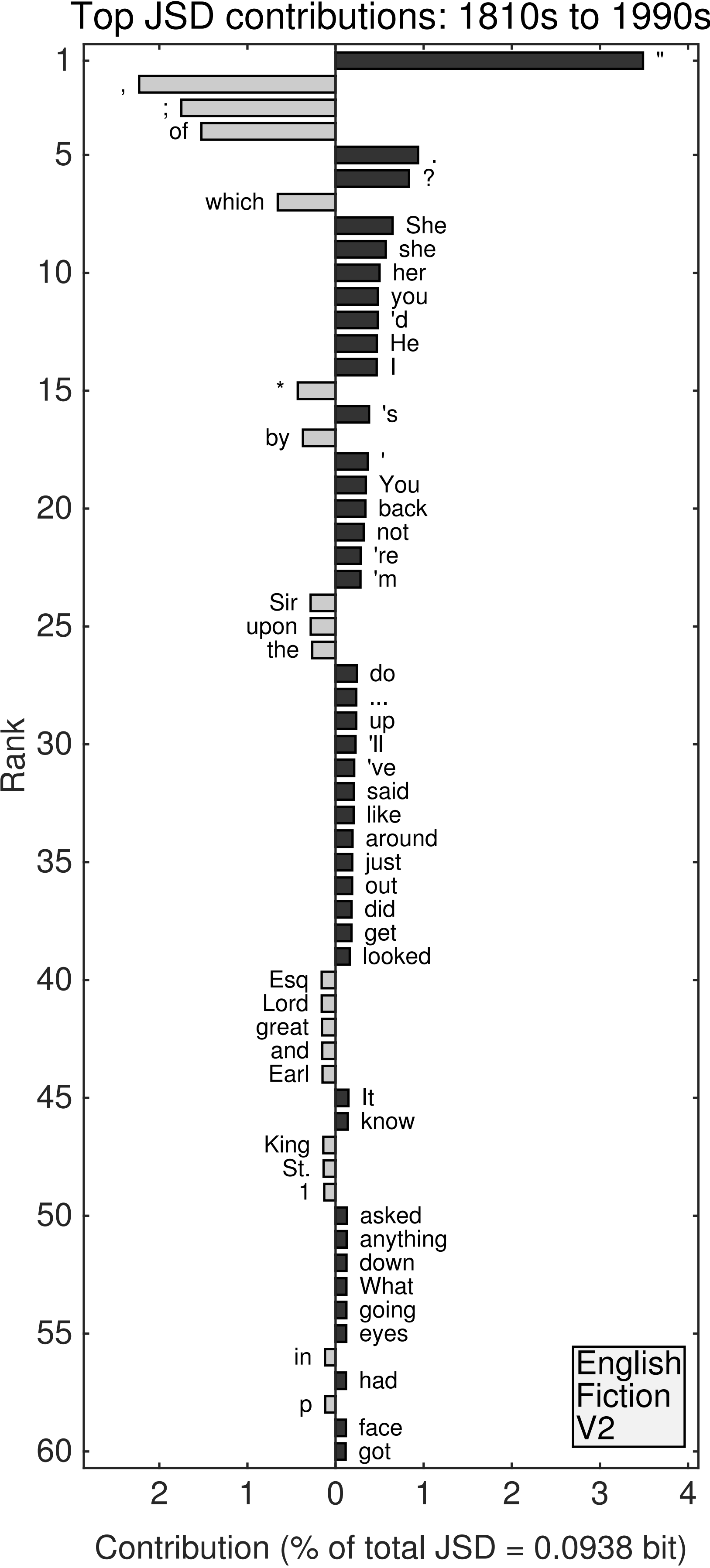

Top JSD contributions: 1810s to 1990s
Rank
Contribution (% of total JSD = 0.0938 bit)
English Fiction V2
"
,
;
of
.
?
which
She
she
her
you
'd
He
I
*
's
by
'
You
back
not
're
'm
Sir
upon
the
do
...
up
'll
've
said
like
around
just
out
did
get
looked
Esq
Lord
great
and
Earl
It
know
King
St.
1
asked
anything
down
What
going
eyes
in
had
p
face
got

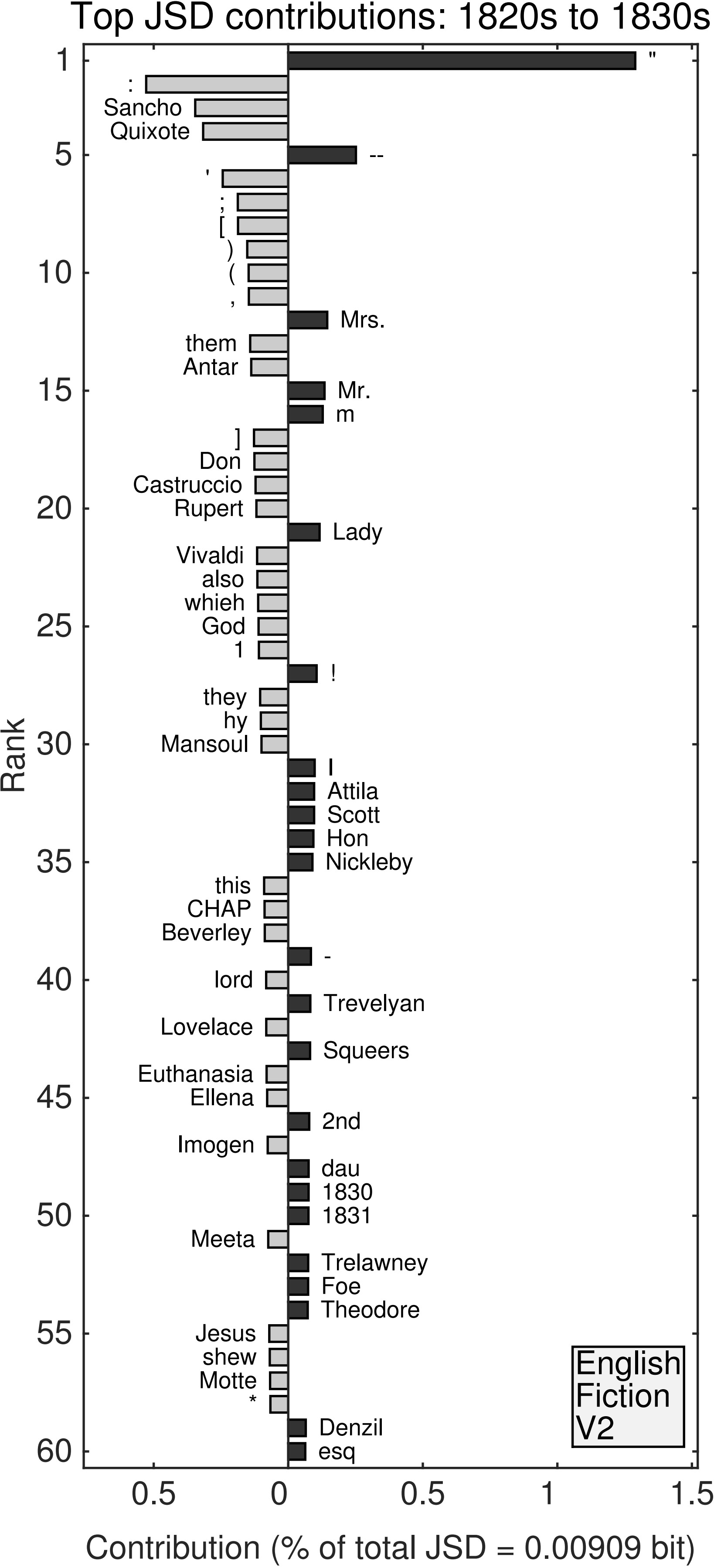

Top JSD contributions: 1820s to 1830s
Rank
Contribution (% of total JSD = 0.00909 bit)
"
:
Sancho
Quixote
--
'
;
[
)
(
,
Mrs.
them
Antar
Mr.
m
]
Don
Castruccio
Rupert
Lady
Vivaldi
also
whieh
God
1
!
they
hy
Mansoul
I
Attila
Scott
Hon
Nickleby
this
CHAP
Beverley
-
lord
Trevelyan
Lovelace
Squeers
Euthanasia
Ellena
2nd
Imogen
dau
1830
1831
Meeta
Trelawney
Foe
Theodore
Jesus
shew
Motte
*
Denzil
esq
English Fiction V2

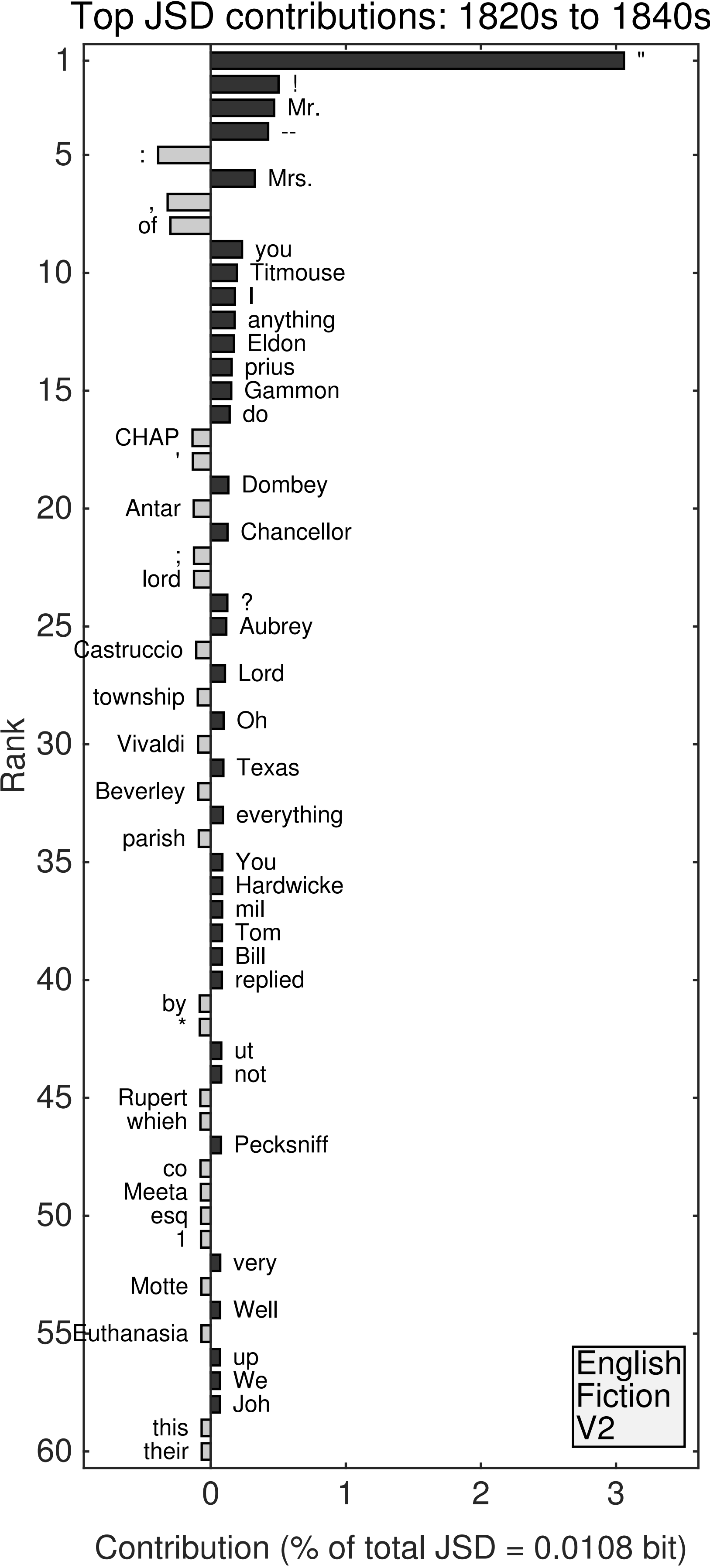
Top JSD contributions: 1820s to 1840s
Rank
Contribution (% of total JSD = 0.0108 bit)
English Fiction V2
"
!
Mr.
--
:
Mrs.
,
of
you
Titmouse
I
anything
Eldon
prius
Gammon
do
CHAP
'
Dombey
Antar
Chancellor
;
lord
?
Aubrey
Castruccio
Lord
township
Oh
Vivaldi
Texas
Beverley
everything
parish
You
Hardwicke
mil
Tom
Bill
replied
by
*
ut
not
Rupert
whieh
Pecksniff
co
Meeta
esq
1
very
Motte
Well
Euthanasia
up
We
Joh
this
their

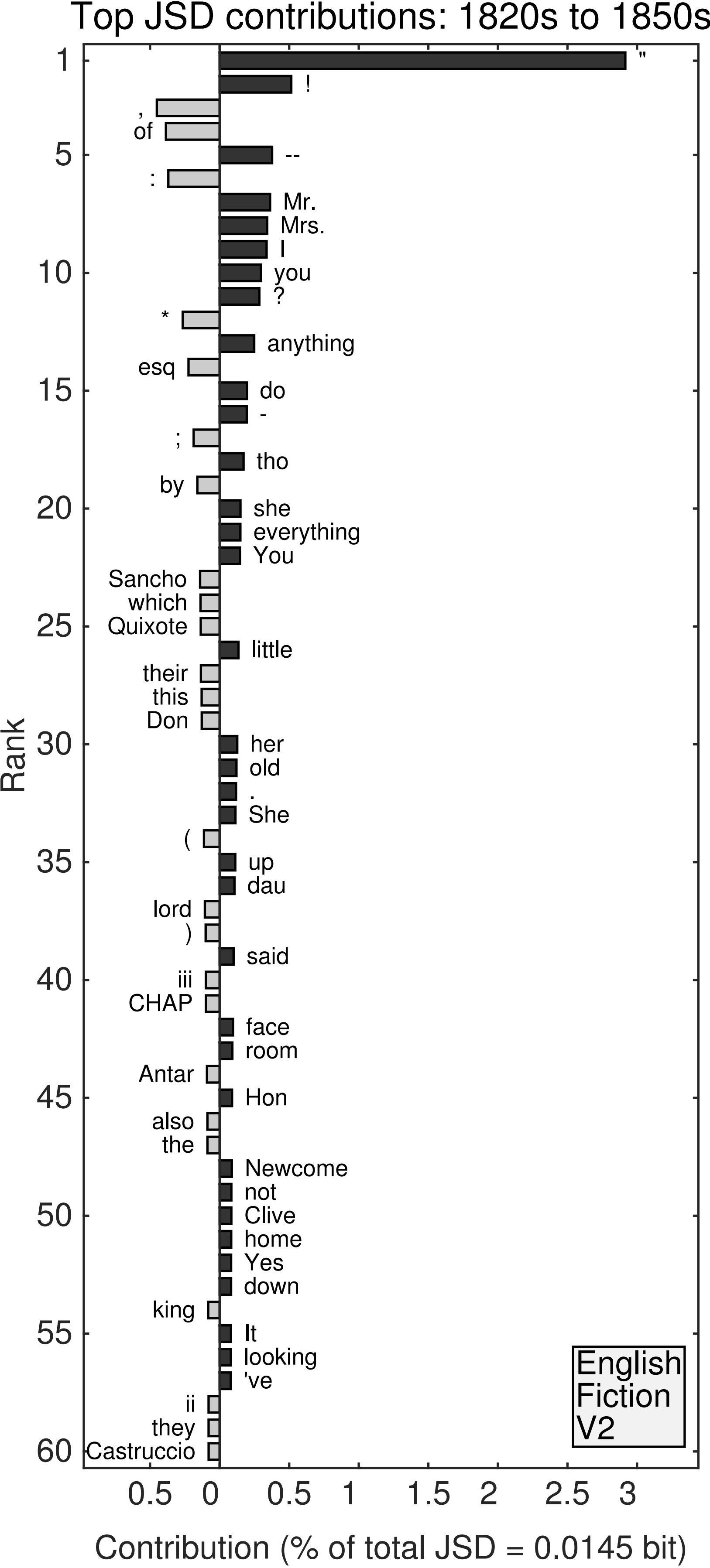
Top JSD contributions: 1820s to 1850s
Rank
Contribution (% of total JSD = 0.0145 bit)
English
Fiction
V2
"
!
,
of
--
:
Mr.
Mrs.
I
you
?
*
anything
esq
do
-
;
tho
by
she
everything
You
Sancho
which
Quixote
little
their
this
Don
her
old
.
She
(
up
dau
lord
)
said
iii
CHAP
face
room
Antar
Hon
also
the
Newcome
not
Clive
home
Yes
down
king
It
looking
've
ii
they
Castruccio

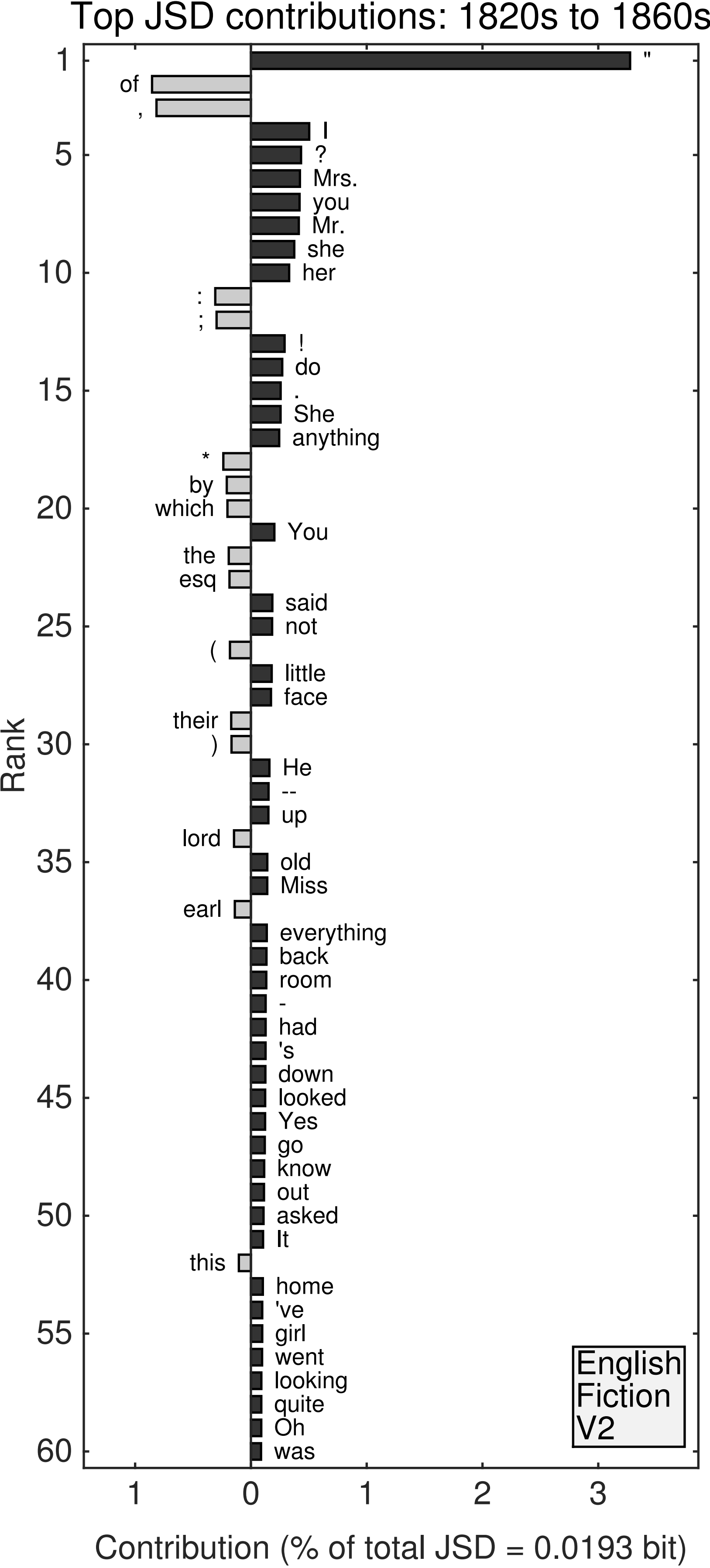

Top JSD contributions: 1820s to 1860s
Rank
Contribution (% of total JSD = 0.0193 bit)
English
Fiction
V2
"
of
,
I
?
Mrs.
you
Mr.
she
her
:
;
!
do
.
She
anything
*
by
which
You
the
esq
said
not
(
little
face
their
)
He
--
up
lord
old
Miss
earl
everything
back
room
-
had
's
down
looked
Yes
go
know
out
asked
It
this
home
've
girl
went
looking
quite
Oh
was

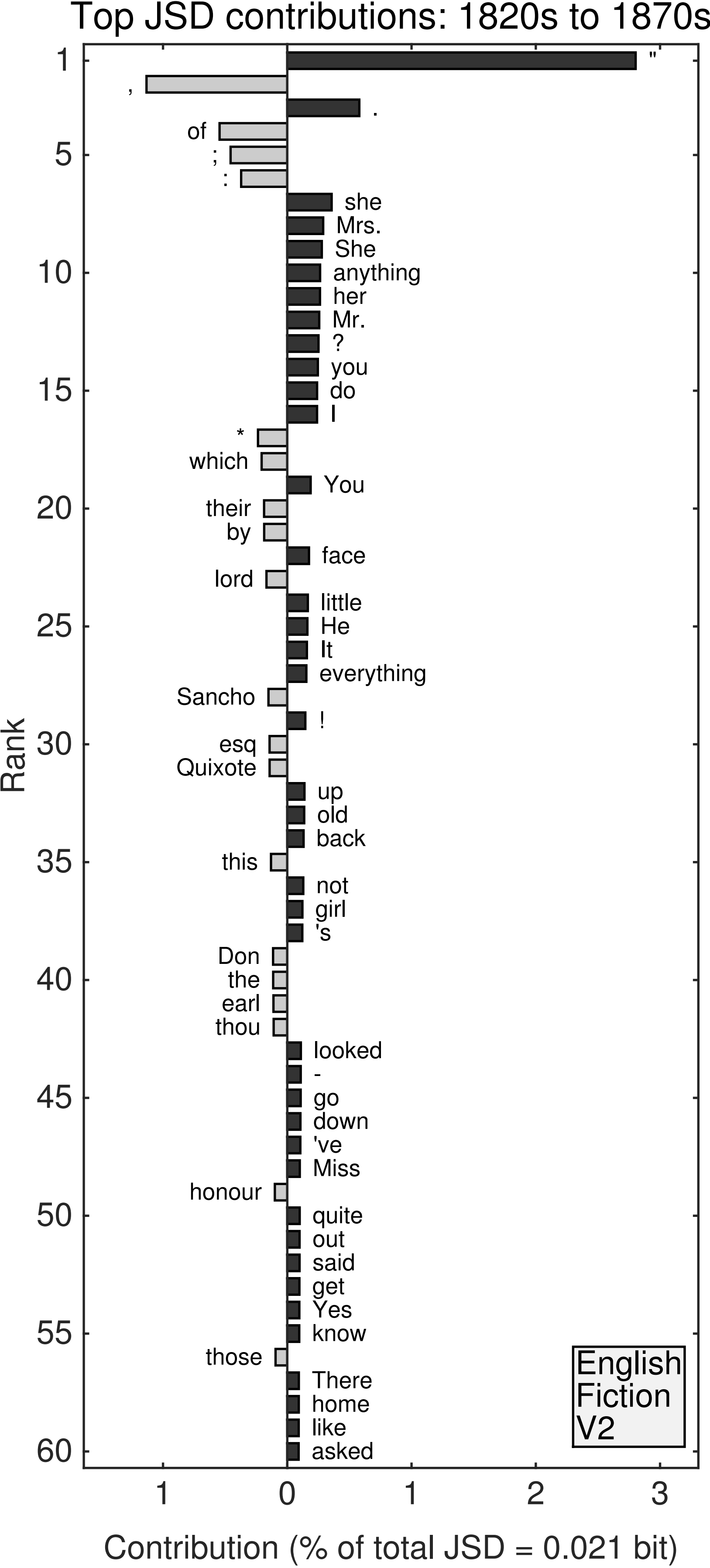
Top JSD contributions: 1820s to 1870s
Rank
Contribution (% of total JSD = 0.021 bit)
English Fiction V2
"
,
.
of
;
:
she
Mrs.
She
anything
her
Mr.
?
you
do
I
*
which
You
their
by
face
lord
little
He
It
everything
Sancho
!
esq
Quixote
up
old
back
this
not
girl
's
Don
the
earl
thou
looked
-
go
down
've
Miss
honour
quite
out
said
get
Yes
know
those
There
home
like
asked

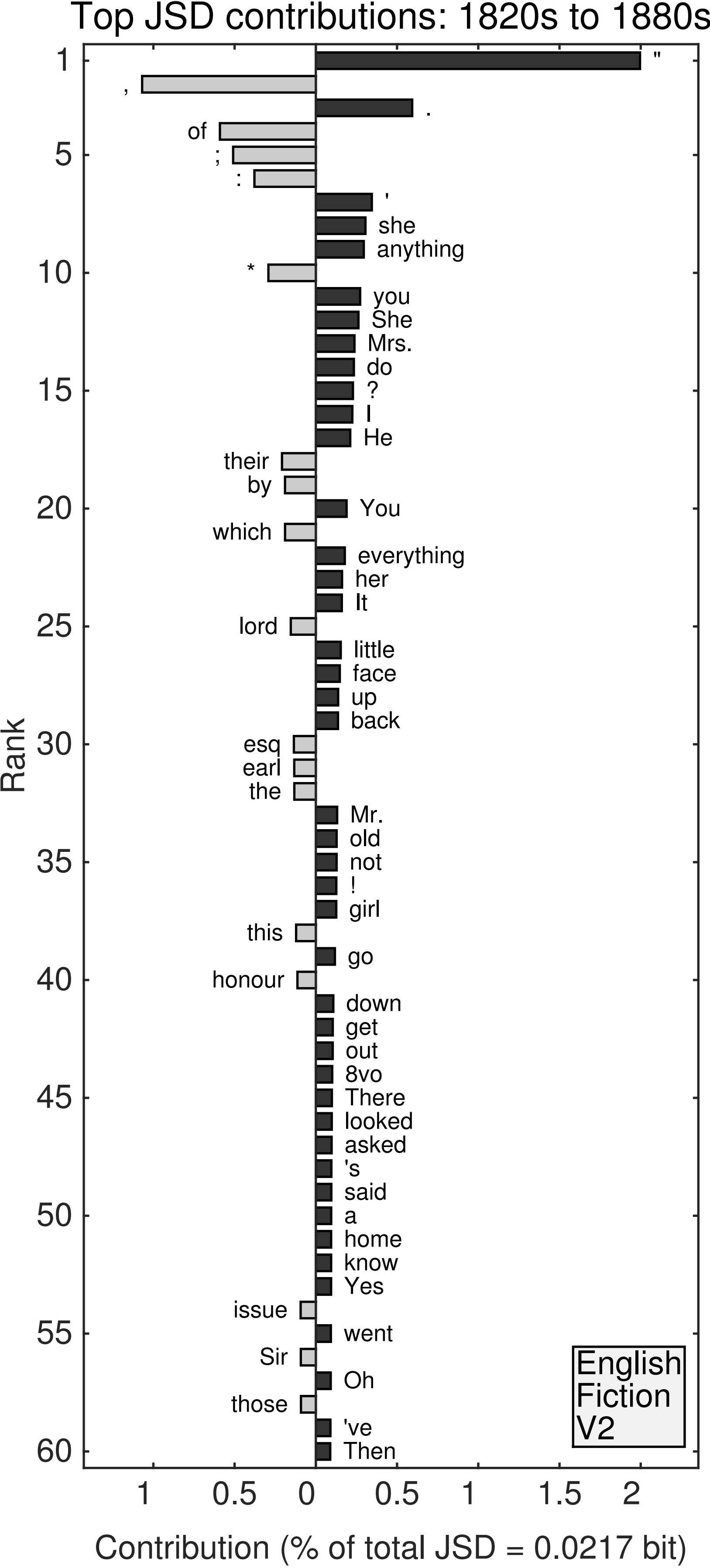

Top JSD contributions: 1820s to 1880s
Rank
Contribution (% of total JSD = 0.0217 bit)
English Fiction V2
"
,
.
of
;
:
'
she
anything
*
you
She
Mrs.
do
?
I
He
their
by
You
which
everything
her
It
lord
little
face
up
back
esq
earl
the
Mr.
old
not
!
girl
this
go
honour
down
get
out
8vo
There
looked
asked
's
said
a
home
know
Yes
issue
went
Sir
Oh
those
've
Then

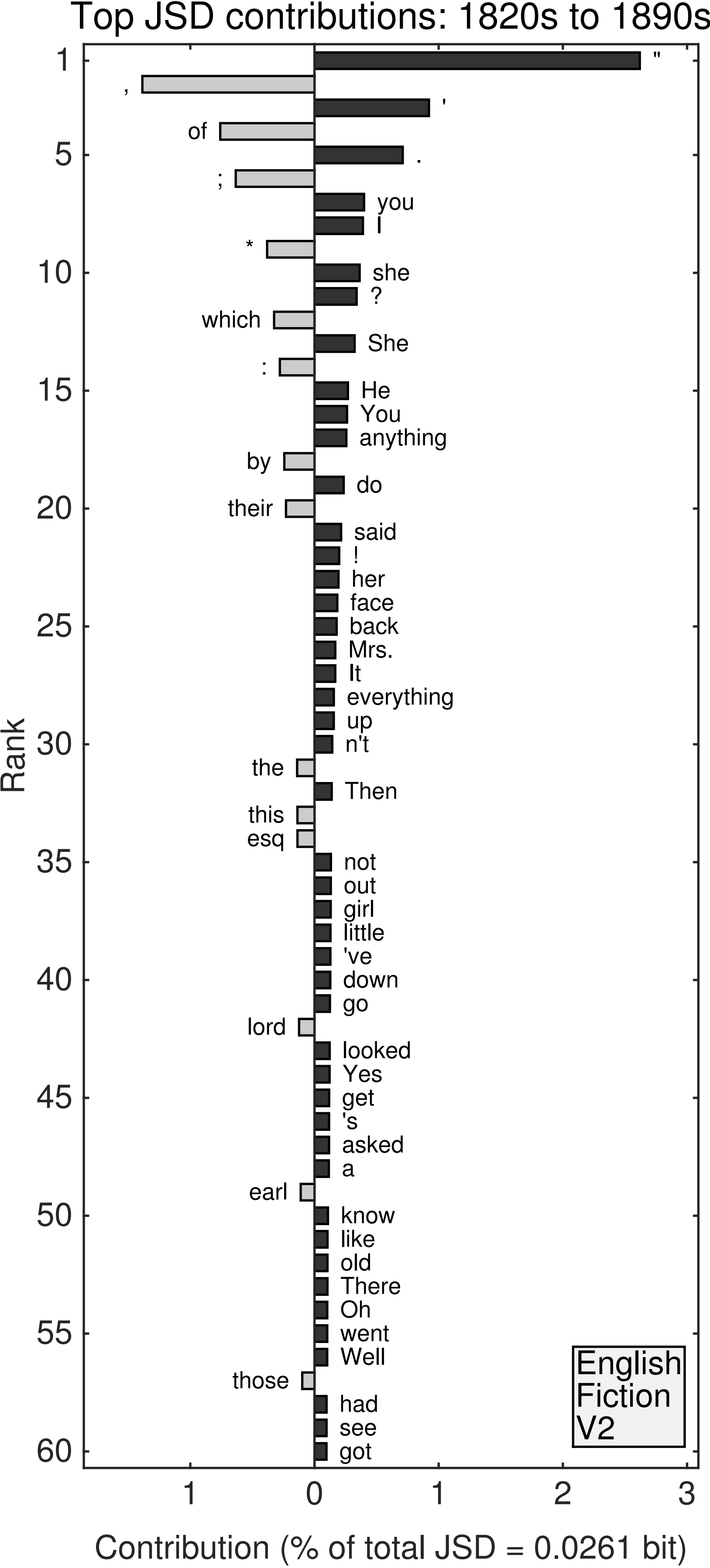
Top JSD contributions: 1820s to 1890s
Rank
Contribution (% of total JSD = 0.0261 bit)
English Fiction V2
"
,
'
of
.
;
you
I
*
she
?
which
She
:
He
You
anything
by
do
their
said
!
her
face
back
Mrs.
It
everything
up
n't
the
Then
this
esq
not
out
girl
little
've
down
go
lord
looked
Yes
get
's
asked
a
earl
know
like
old
There
Oh
went
Well
those
had
see
got

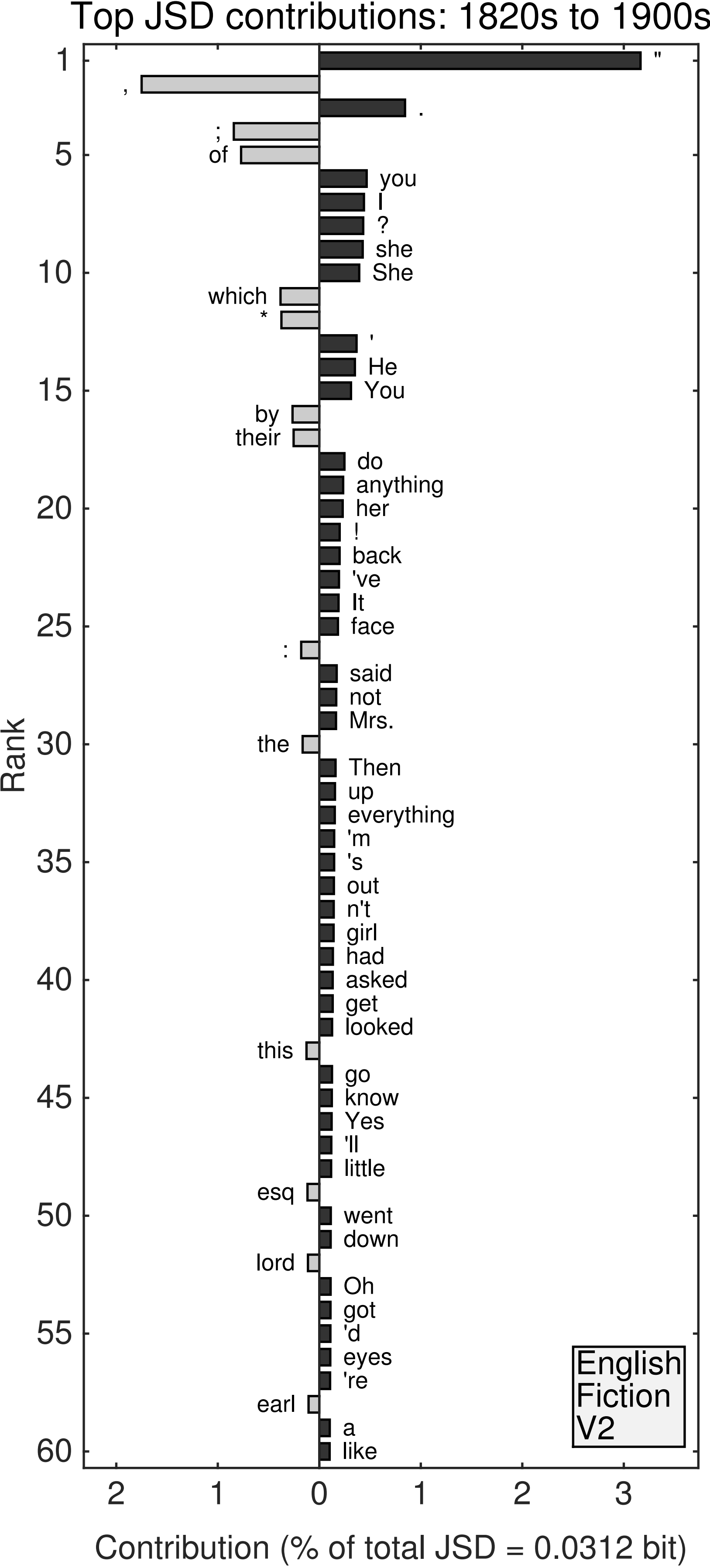

Top JSD contributions: 1820s to 1900s
Rank
Contribution (% of total JSD = 0.0312 bit)
English Fiction V2
"
,
.
;
of
you
I
?
she
She
which
*
'
He
You
by
their
do
anything
her
!
back
've
It
face
:
said
not
Mrs.
the
Then
up
everything
'm
's
out
n't
girl
had
asked
get
looked
this
go
know
Yes
'll
little
esq
went
down
lord
Oh
got
'd
eyes
're
earl
a
like
1
5
10
15
20
25
30
35
40
45
50
55
60
2
1
0
1
2
3

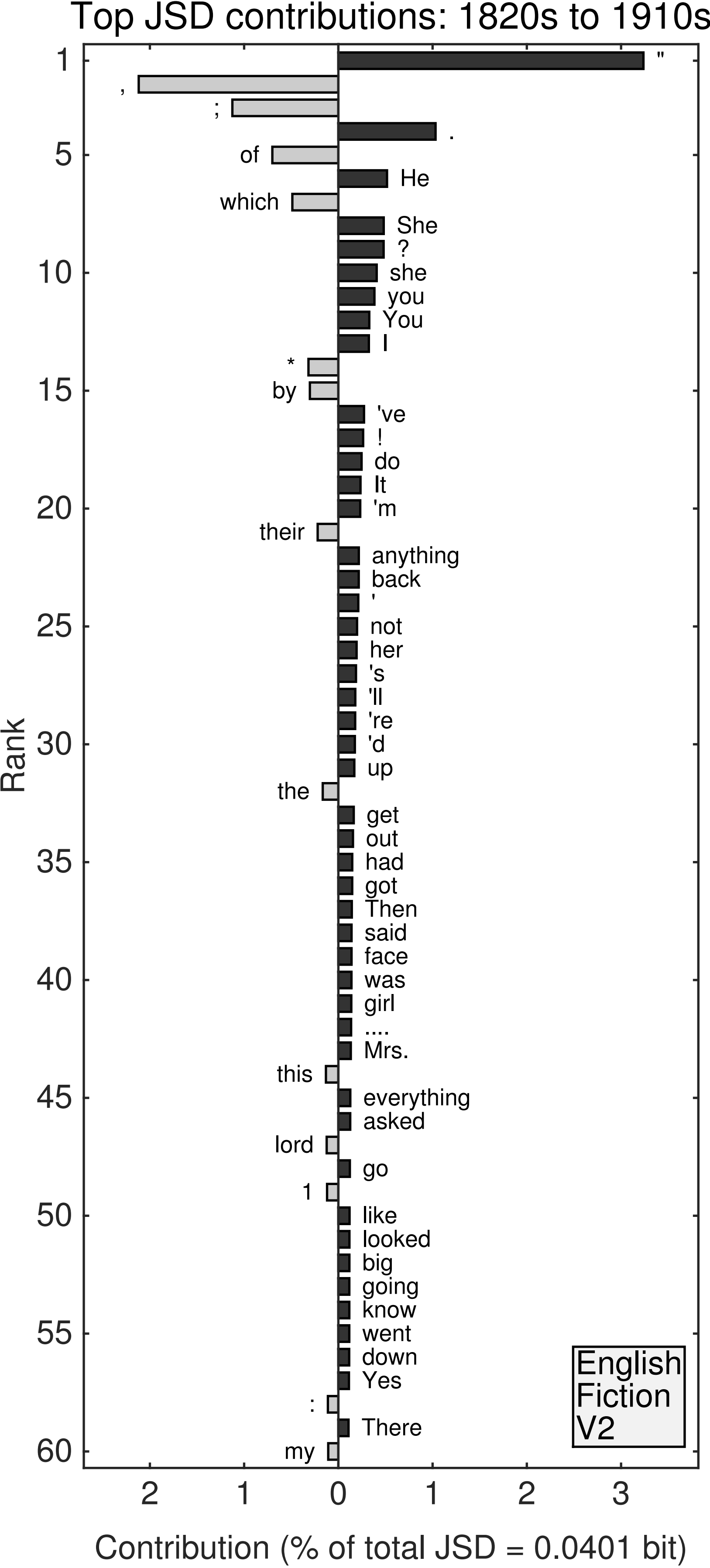

Top JSD contributions: 1820s to 1910s
Rank
Contribution (% of total JSD = 0.0401 bit)
English Fiction V2
"
,
;
.
of
He
which
She
?
she
you
You
I
*
by
've
!
do
It
'm
their
anything
back
'
not
her
's
'll
're
'd
up
the
get
out
had
got
Then
said
face
was
girl
....
Mrs.
this
everything
asked
lord
go
1
like
looked
big
going
know
went
down
Yes
:
There
my

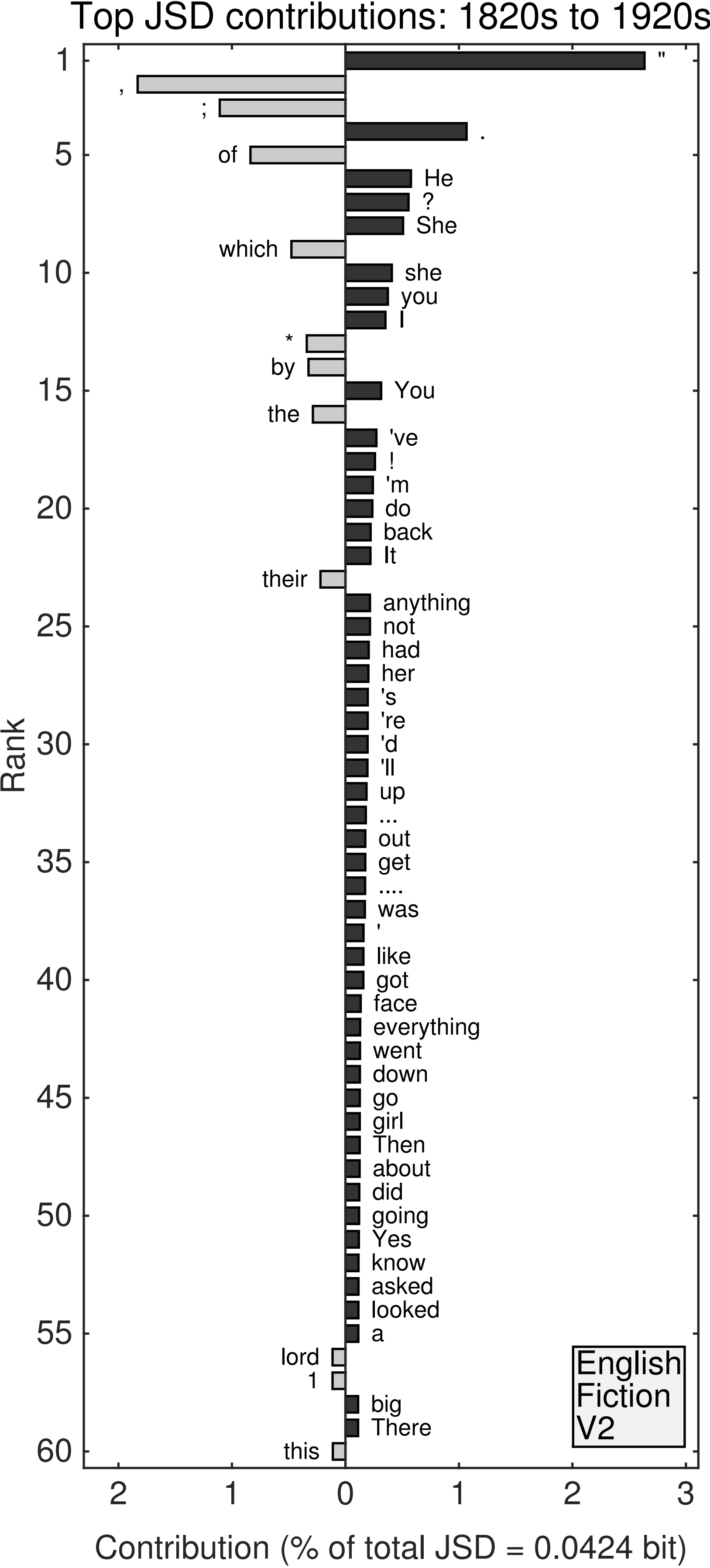

Top JSD contributions: 1820s to 1920s
Rank
Contribution (% of total JSD = 0.0424 bit)
English Fiction V2
"
,
;
.
of
He
?
She
which
she
you
I
*
by
You
the
've
!
'm
do
back
It
their
anything
not
had
her
's
're
'd
'll
up
...
out
get
....
was
'
like
got
face
everything
went
down
go
girl
Then
about
did
going
Yes
know
asked
looked
a
lord
1
big
There
this

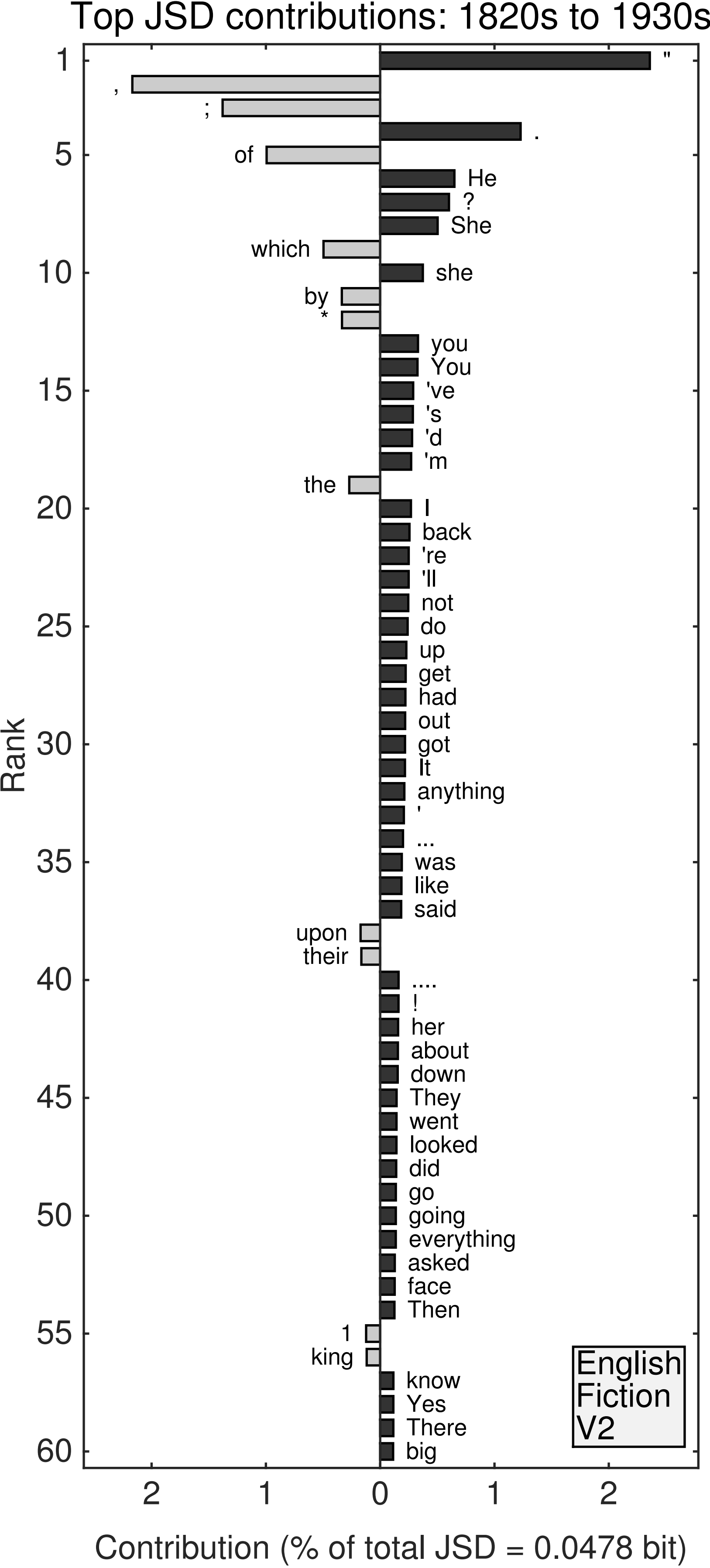
Top JSD contributions: 1820s to 1930s
Rank
1
5
10
15
20
25
30
35
40
45
50
55
60
"
,
;
.
of
He
?
She
which
she
by
*
you
You
've
's
'd
'm
the
I
back
're
'll
not
do
up
get
had
out
got
It
anything
'
...
was
like
said
upon
their
....
!
her
about
down
They
went
looked
did
go
going
everything
asked
face
Then
1
king
know
Yes
There
big
English
Fiction
V2
2
1
0
1
2
Contribution (% of total JSD = 0.0478 bit)

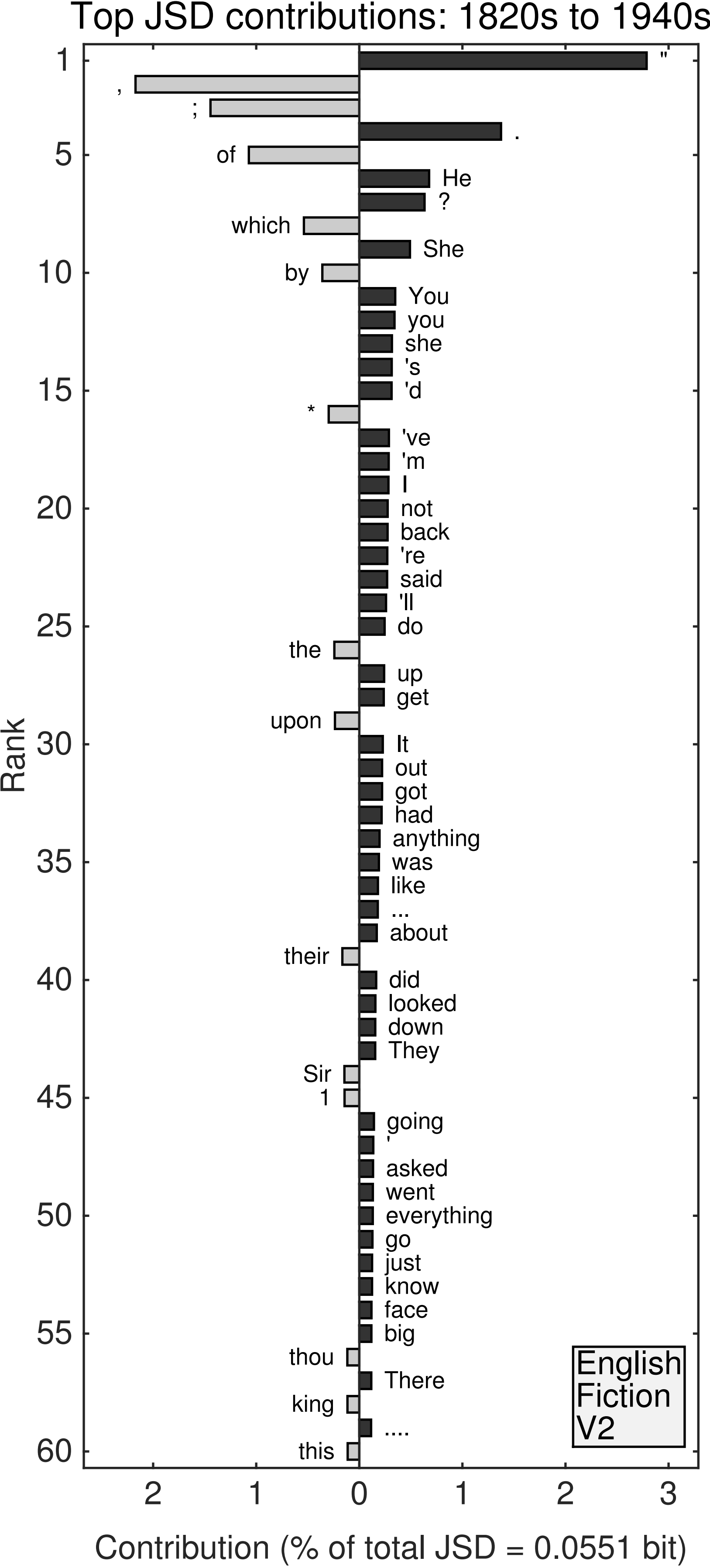

Top JSD contributions: 1820s to 1940s
Rank
Contribution (% of total JSD = 0.0551 bit)
English Fiction V2
"
,
;
.
of
He
?
which
She
by
You
you
she
's
'd
*
've
'm
I
not
back
're
said
'll
do
the
up
get
upon
It
out
got
had
anything
was
like
...
about
their
did
looked
down
They
Sir
1
going
'
asked
went
everything
go
just
know
face
big
thou
There
king
....
this

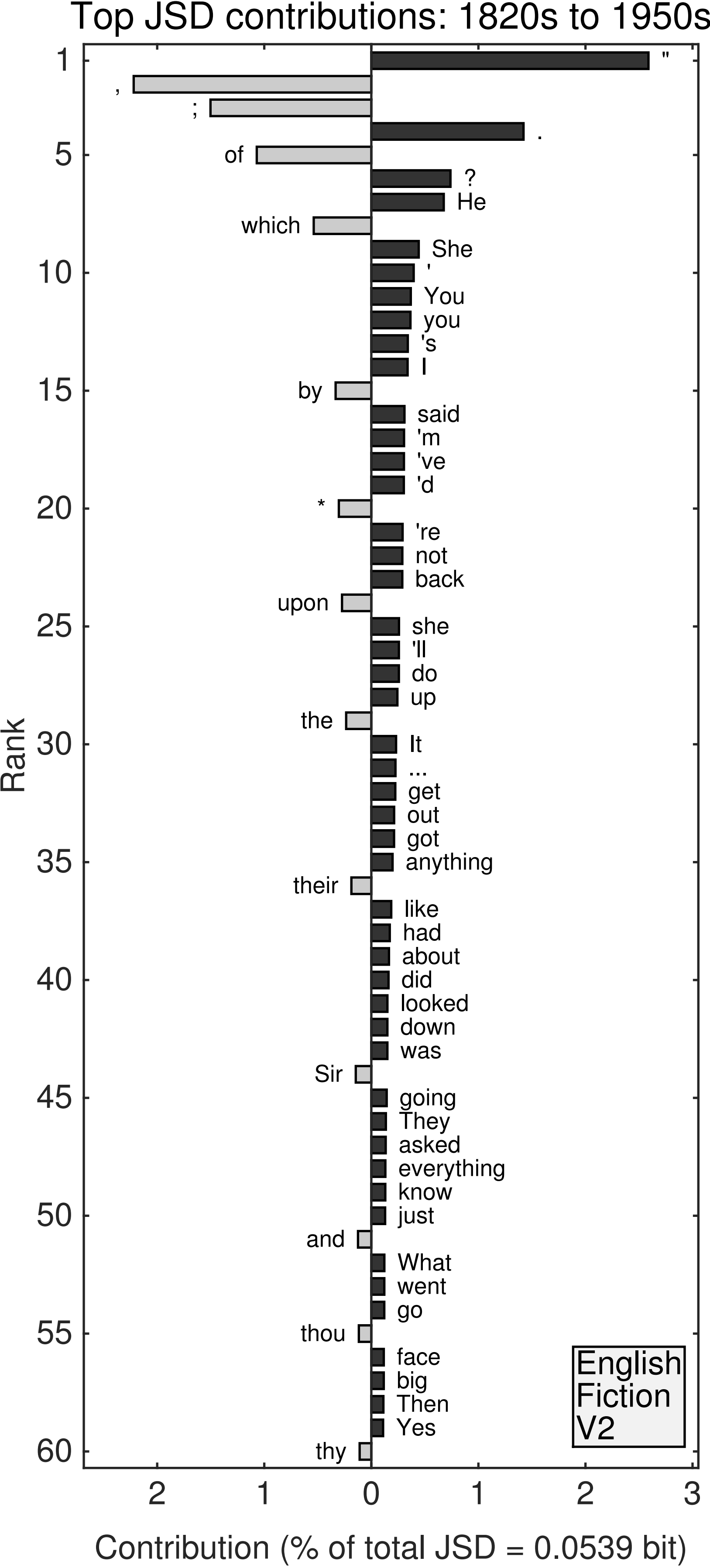

Top JSD contributions: 1820s to 1950s
Rank
Contribution (% of total JSD = 0.0539 bit)
English Fiction V2
"
,
;
.
of
?
He
which
She
'
You
you
's
I
by
said
'm
've
'd
*
're
not
back
upon
she
'll
do
up
the
It
...
get
out
got
anything
their
like
had
about
did
looked
down
was
Sir
going
They
asked
everything
know
just
and
What
went
go
thou
face
big
Then
Yes
thy

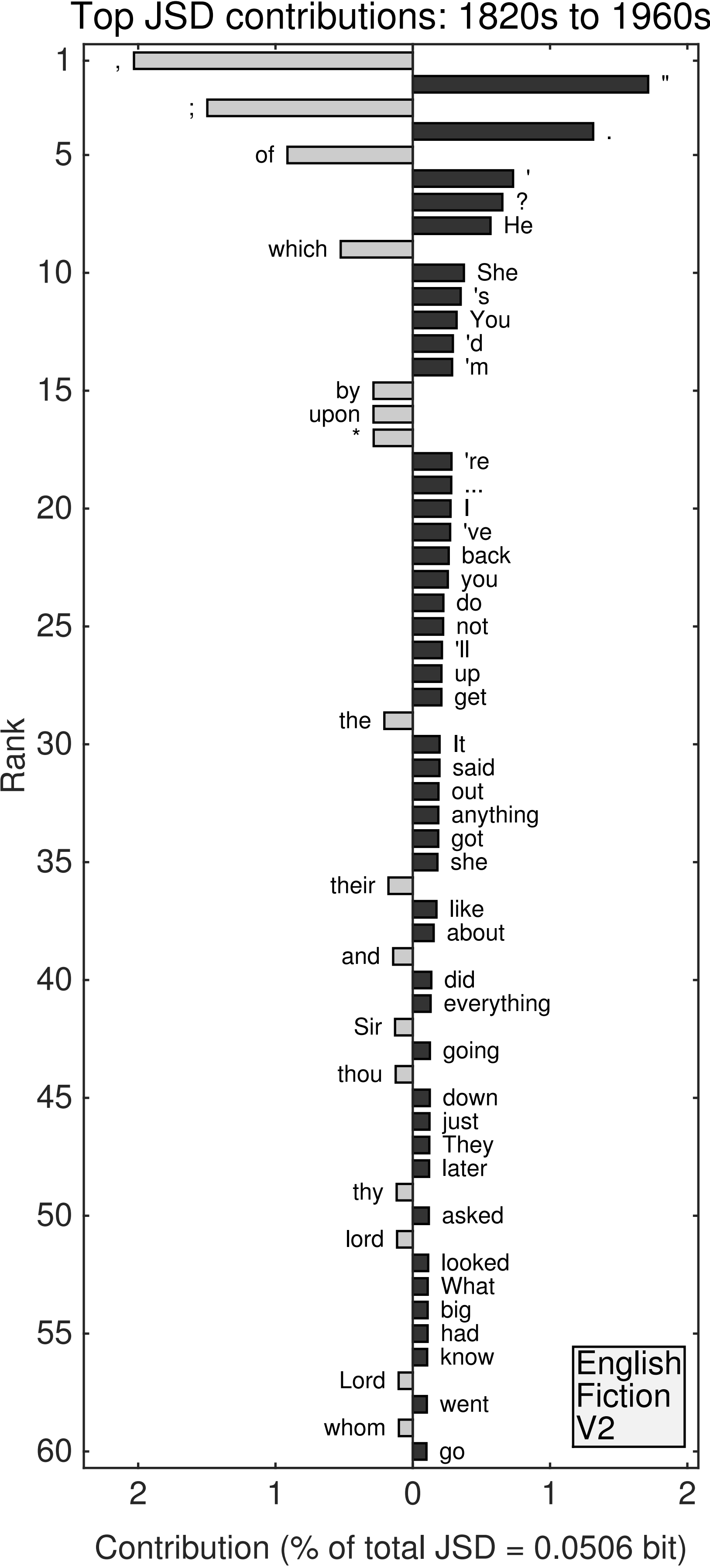

Top JSD contributions: 1820s to 1960s
Rank
Contribution (% of total JSD = 0.0506 bit)
English Fiction V2
,
"
;
.
of
'
?
He
which
She
's
You
'd
'm
by
upon
*
're
...
I
've
back
you
do
not
'll
up
get
the
It
said
out
anything
got
she
their
like
about
and
did
everything
Sir
going
thou
down
just
They
later
thy
asked
lord
looked
What
big
had
know
Lord
went
whom
go

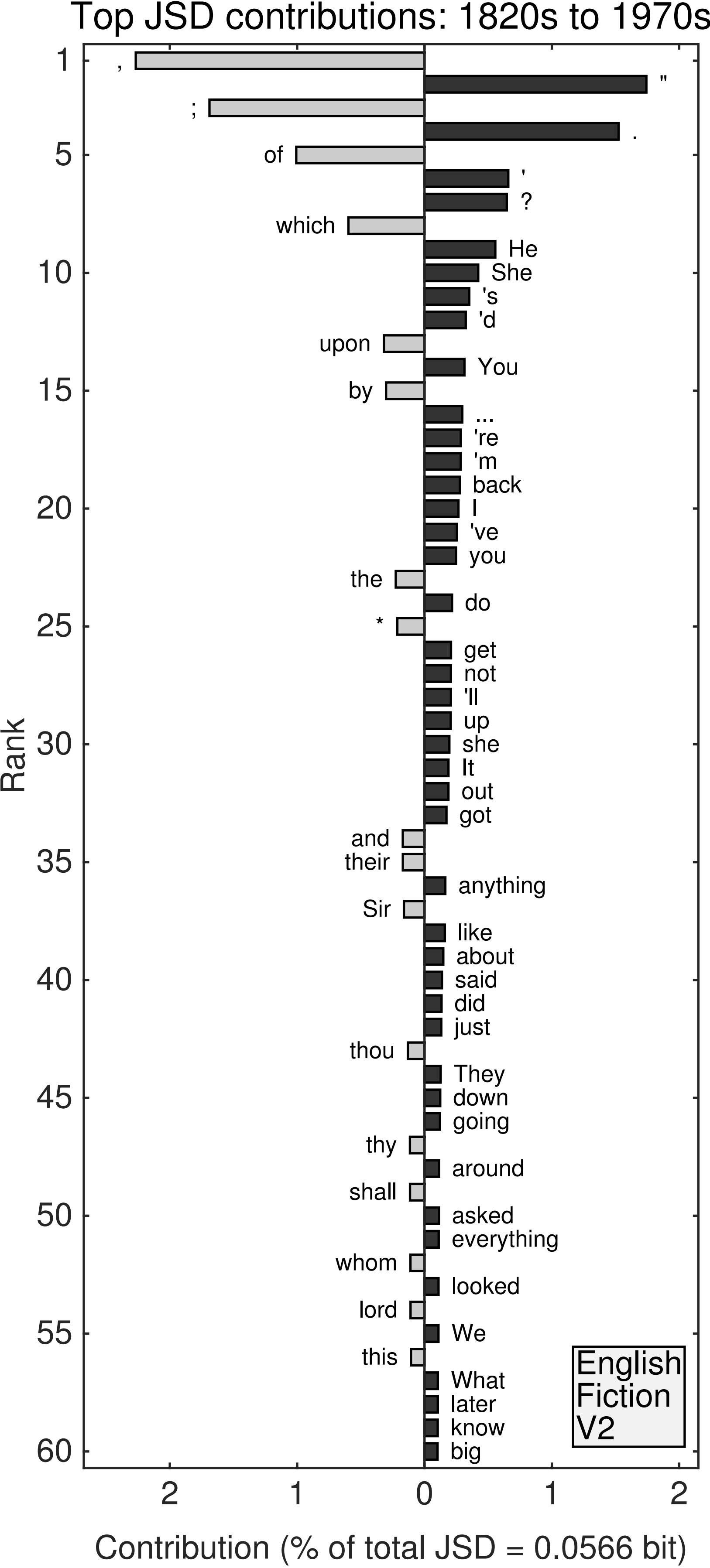

Top JSD contributions: 1820s to 1970s
Rank
Contribution (% of total JSD = 0.0566 bit)
English Fiction V2
1
5
10
15
20
25
30
35
40
45
50
55
60
2
1
0
1
2
,
"
;
.
of
'
?
which
He
She
's
'd
upon
You
by
...
're
'm
back
I
've
you
the
do
*
get
not
'll
up
she
It
out
got
and
their
anything
Sir
like
about
said
did
just
thou
They
down
going
thy
around
shall
asked
everything
whom
looked
lord
We
this
What
later
know
big

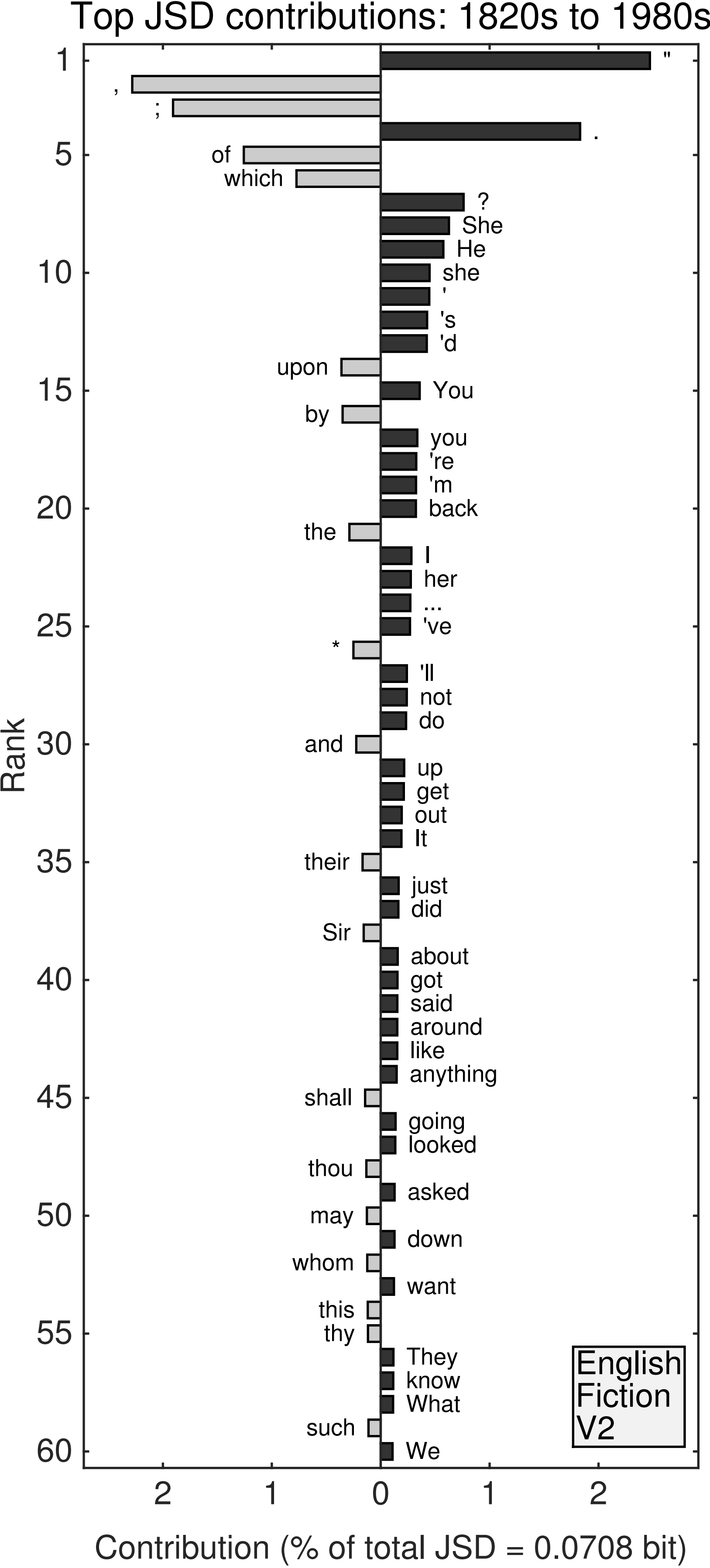

Top JSD contributions: 1820s to 1980s
Rank
Contribution (% of total JSD = 0.0708 bit)
English Fiction V2
"
,
;
.
of
which
?
She
He
she
'
's
'd
upon
You
by
you
're
'm
back
the
I
her
...
've
*
'll
not
do
and
up
get
out
It
their
just
did
Sir
about
got
said
around
like
anything
shall
going
looked
thou
asked
may
down
whom
want
this
thy
They
know
What
such
We

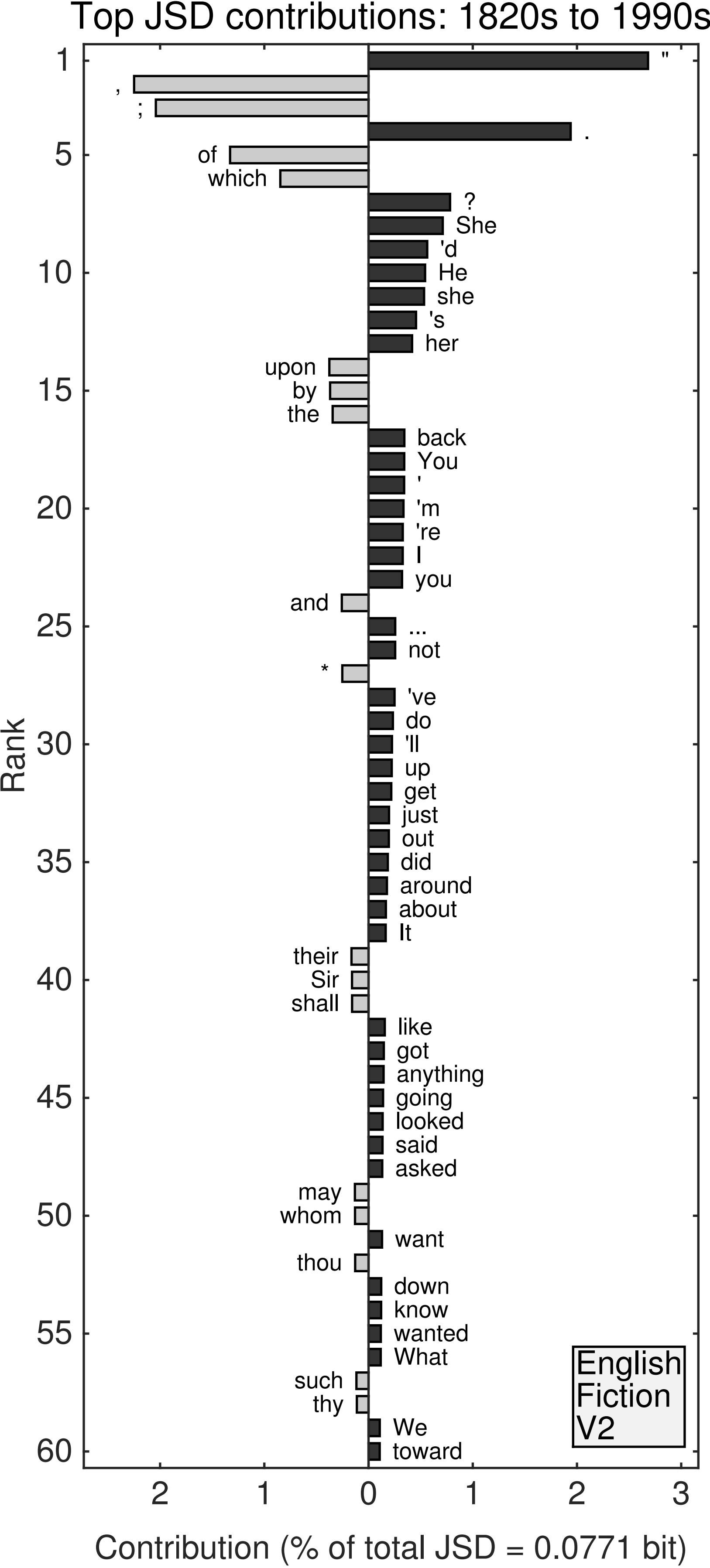
Top JSD contributions: 1820s to 1990s
Rank
Contribution (% of total JSD = 0.0771 bit)
English Fiction V2
"
,
;
.
of
which
?
She
'd
He
she
's
her
upon
by
the
back
You
'
'm
're
I
you
and
...
not
*
've
do
'll
up
get
just
out
did
around
about
It
their
Sir
shall
like
got
anything
going
looked
said
asked
may
whom
want
thou
down
know
wanted
What
such
thy
We
toward

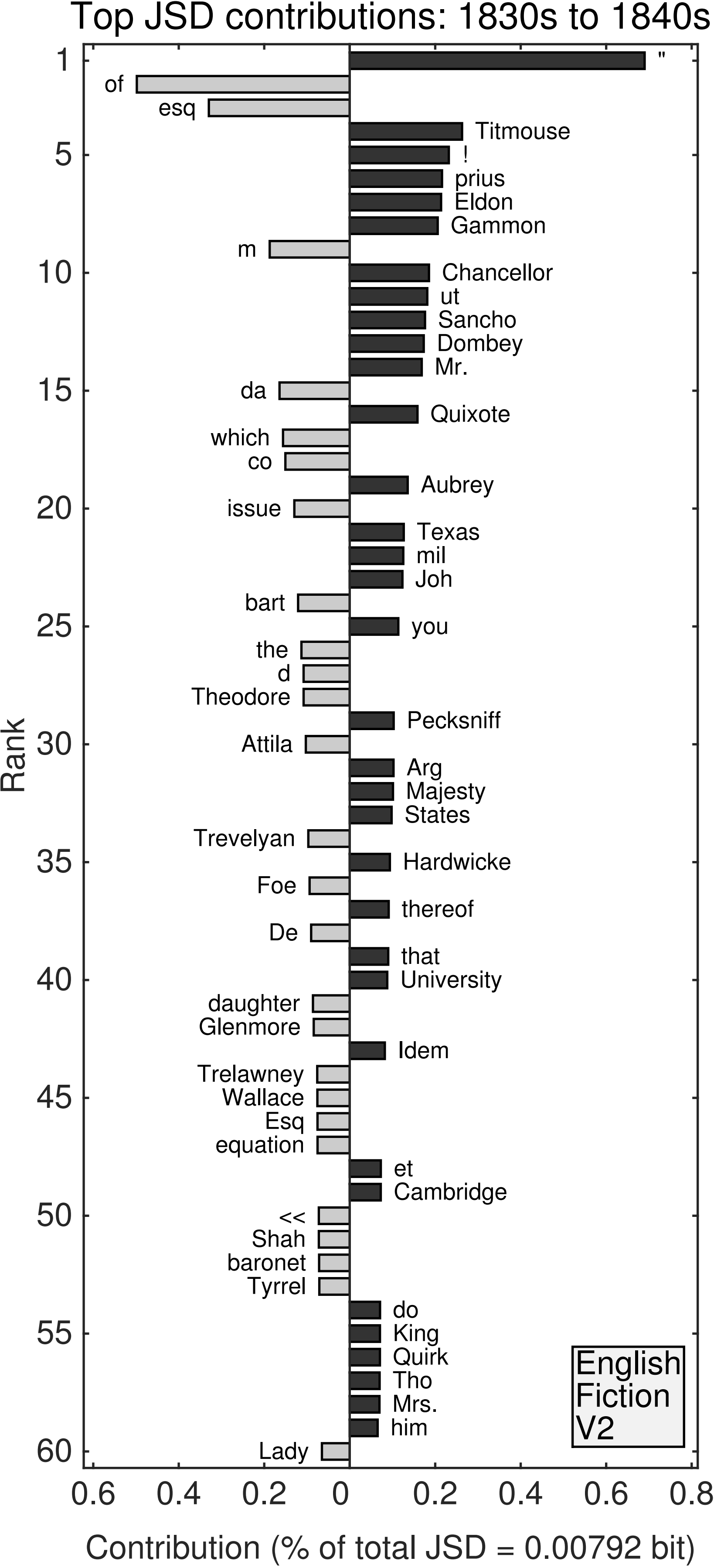
Top JSD contributions: 1830s to 1840s
Rank
Contribution (% of total JSD = 0.00792 bit)
English Fiction V2
"
of
esq
Titmouse
!
prius
Eldon
Gammon
m
Chancellor
ut
Sancho
Dombey
Mr.
da
Quixote
which
co
Aubrey
issue
Texas
mil
Joh
bart
you
the
d
Theodore
Pecksniff
Attila
Arg
Majesty
States
Trevelyan
Hardwicke
Foe
thereof
De
that
University
daughter
Glenmore
Idem
Trelawney
Wallace
Esq
equation
et
Cambridge
<<
Shah
baronet
Tyrrel
do
King
Quirk
Tho
Mrs.
him
Lady

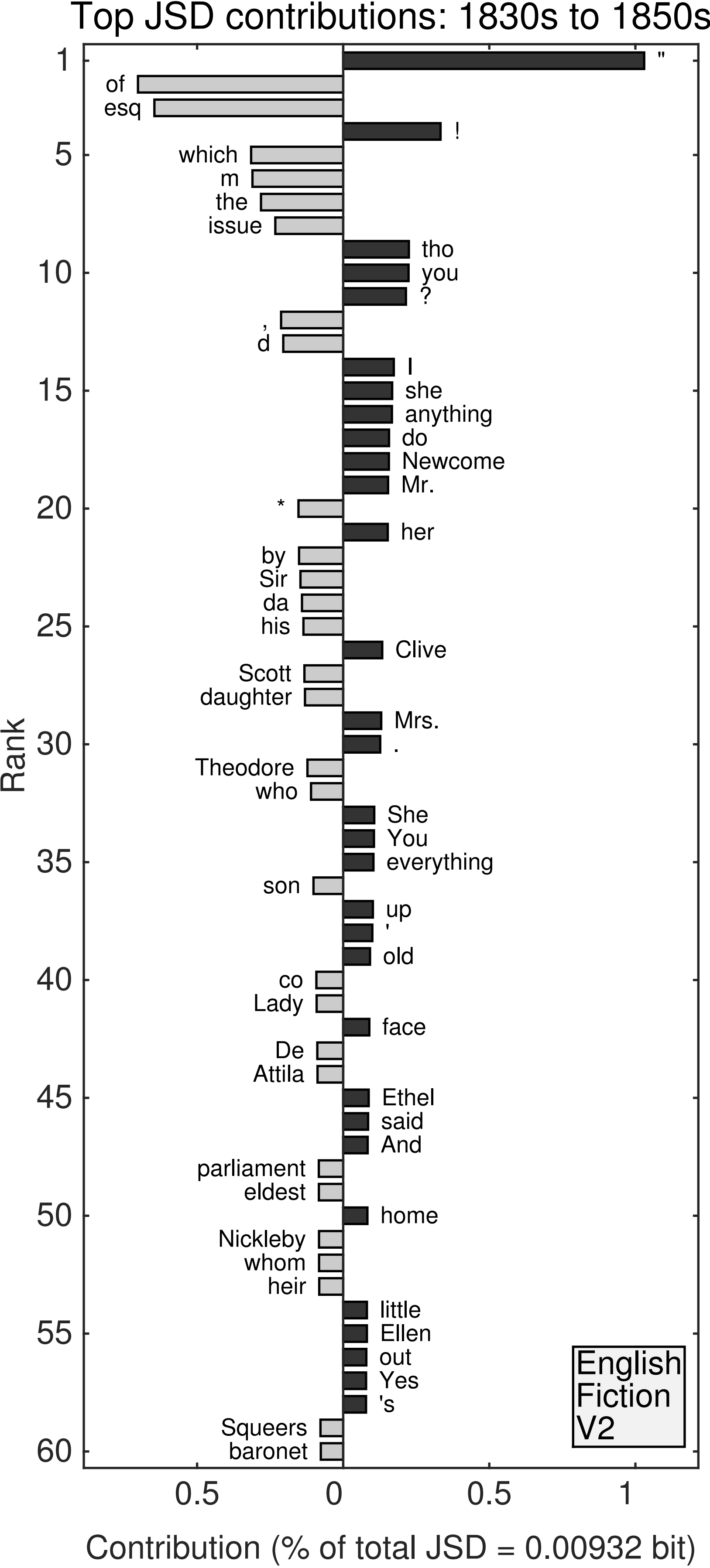
Top JSD contributions: 1830s to 1850s
Rank
Contribution (% of total JSD = 0.00932 bit)
English Fiction V2
"
of
esq
!
which
m
the
issue
tho
you
?
,
d
I
she
anything
do
Newcome
Mr.
*
her
by
Sir
da
his
Clive
Scott
daughter
Mrs.
.
Theodore
who
She
You
everything
son
up
'
old
co
Lady
face
De
Attila
Ethel
said
And
parliament
eldest
home
Nickleby
whom
heir
little
Ellen
out
Yes
's
Squeers
baronet

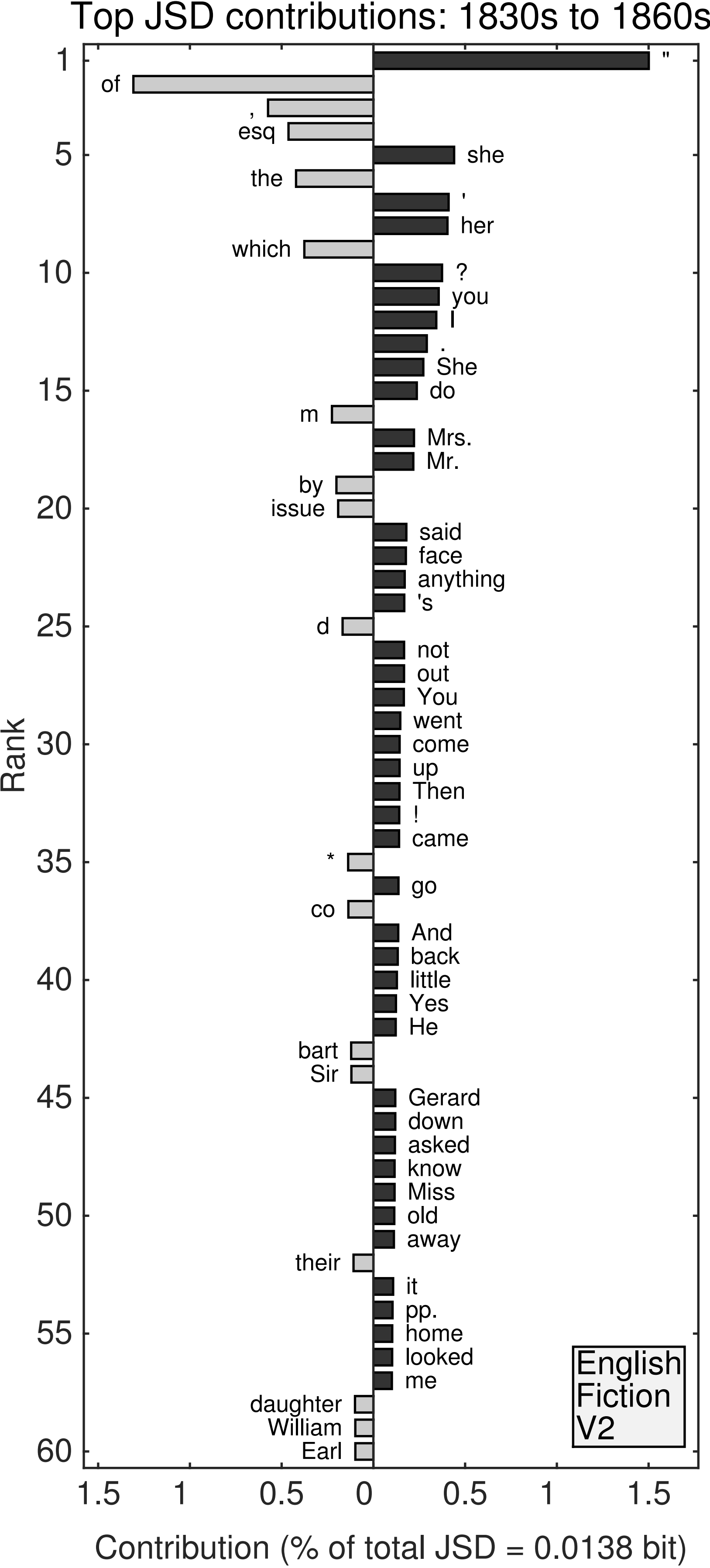

Top JSD contributions: 1830s to 1860s
Rank
"
of
,
esq
she
the
'
her
which
?
you
I
.
She
do
m
Mrs.
Mr.
by
issue
said
face
anything
's
d
not
out
You
went
come
up
Then
!
came
*
go
co
And
back
little
Yes
He
bart
Sir
Gerard
down
asked
know
Miss
old
away
their
it
pp.
home
looked
me
daughter
William
Earl
English Fiction V2
Contribution (% of total JSD = 0.0138 bit)

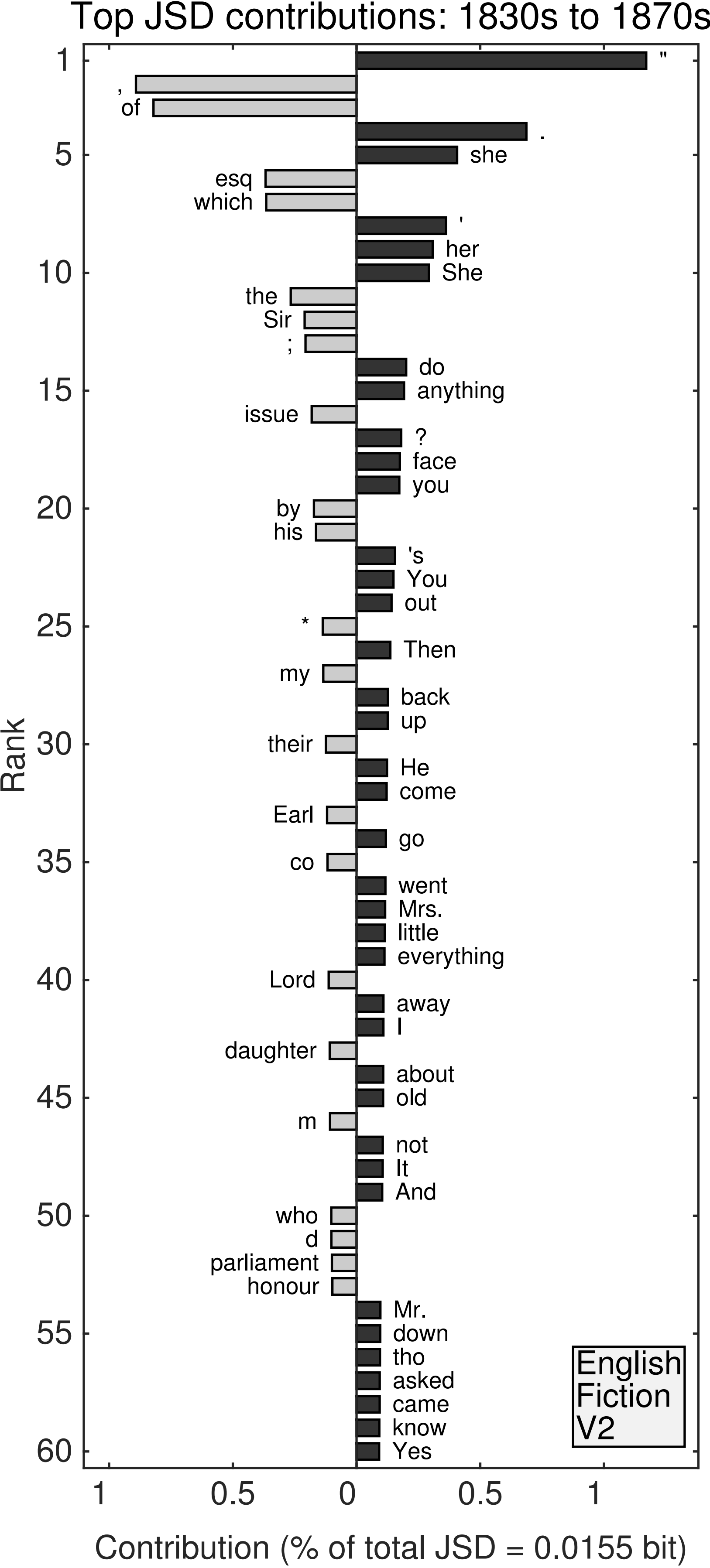
Top JSD contributions: 1830s to 1870s
Rank
Contribution (% of total JSD = 0.0155 bit)
"
,
of
.
she
esq
which
'
her
She
the
Sir
;
do
anything
issue
?
face
you
by
his
's
You
out
*
Then
my
back
up
their
He
come
Earl
go
co
went
Mrs.
little
everything
Lord
away
I
daughter
about
old
m
not
It
And
who
d
parliament
honour
Mr.
down
tho
asked
came
know
Yes
English
Fiction
V2

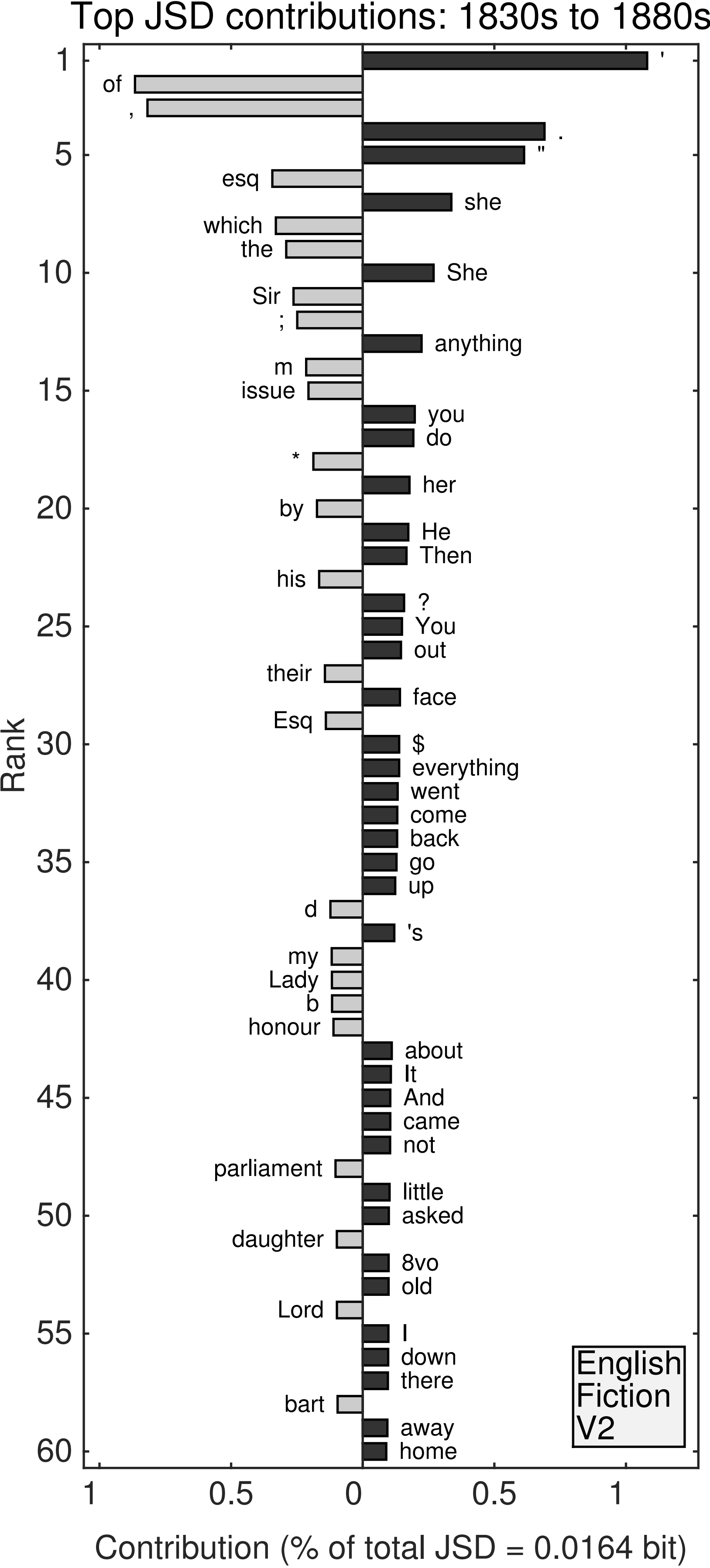

Top JSD contributions: 1830s to 1880s
Rank
Contribution (% of total JSD = 0.0164 bit)
English Fiction V2
1
5
10
15
20
25
30
35
40
45
50
55
60
1
0.5
0
0.5
1
'
of
,
.
"
esq
she
which
the
She
Sir
;
anything
m
issue
you
do
*
her
by
He
Then
his
?
You
out
their
face
Esq
$
everything
went
come
back
go
up
d
's
my
Lady
b
honour
about
It
And
came
not
parliament
little
asked
daughter
8vo
old
Lord
I
down
there
bart
away
home

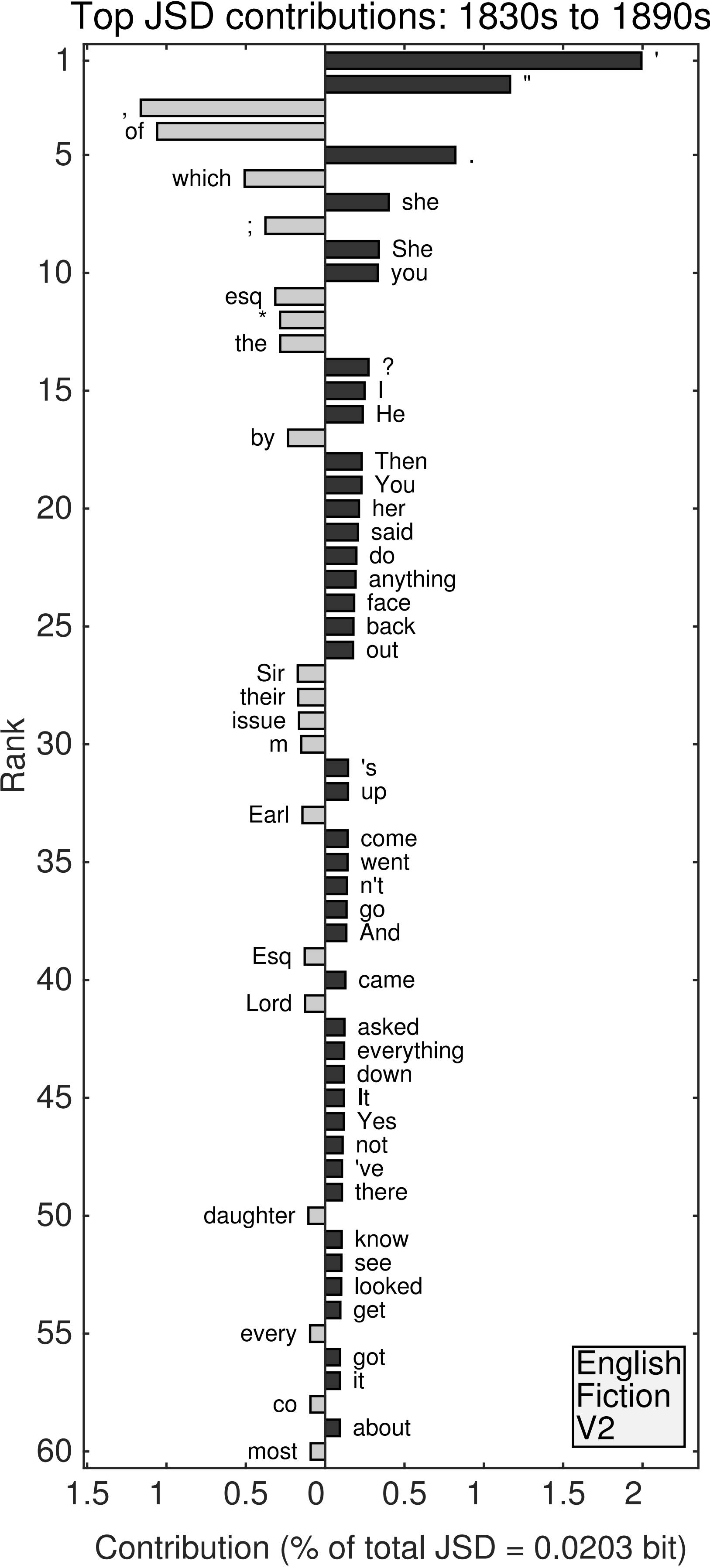
Top JSD contributions: 1830s to 1890s
Rank
Contribution (% of total JSD = 0.0203 bit)
English
Fiction
V2
'
"
,
of
.
which
she
;
She
you
esq
*
the
?
I
He
by
Then
You
her
said
do
anything
face
back
out
Sir
their
issue
m
's
up
Earl
come
went
n't
go
And
Esq
came
Lord
asked
everything
down
It
Yes
not
've
there
daughter
know
see
looked
get
every
got
it
co
about
most

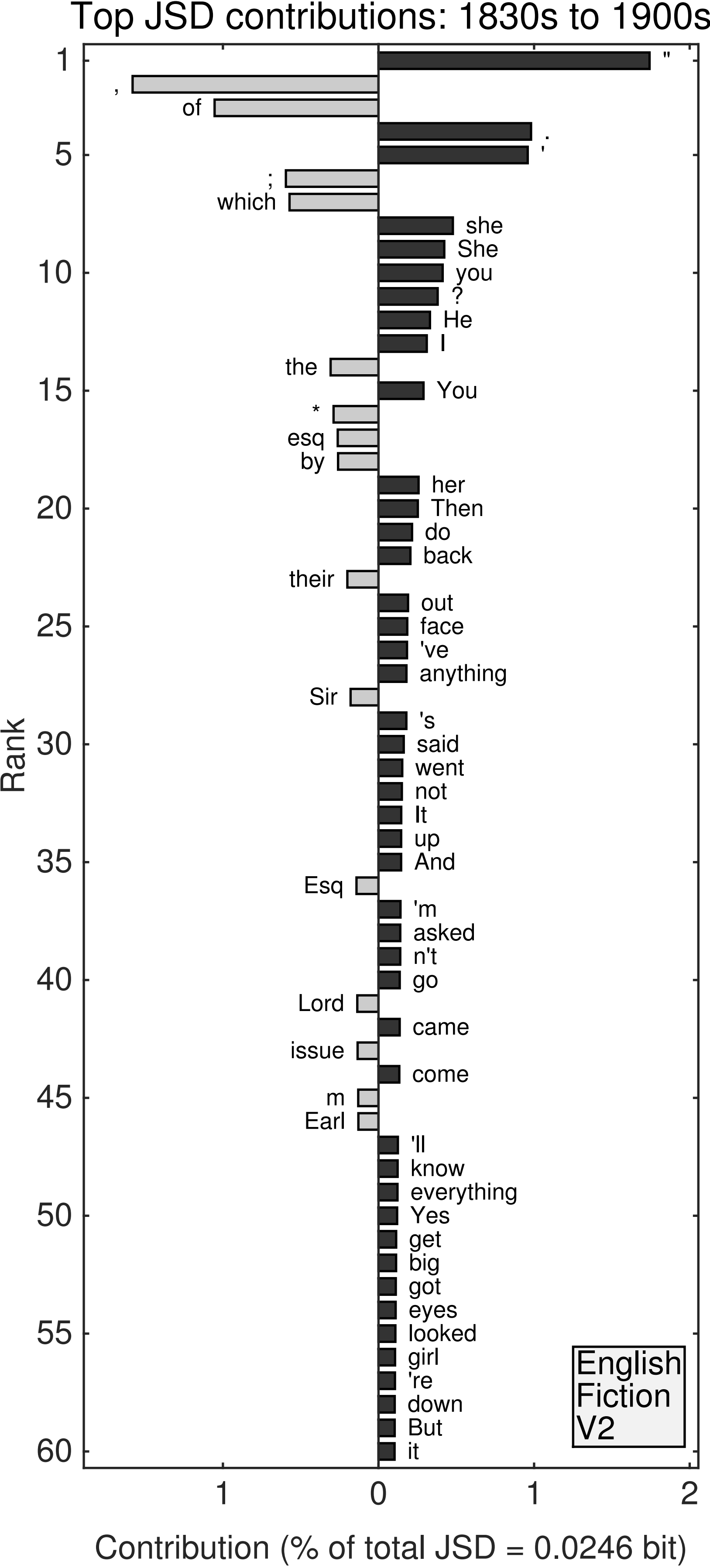

Top JSD contributions: 1830s to 1900s
Rank
Contribution (% of total JSD = 0.0246 bit)
English Fiction V2
"
,
of
.
'
;
which
she
She
you
?
He
I
the
You
*
esq
by
her
Then
do
back
their
out
face
've
anything
Sir
's
said
went
not
It
up
And
Esq
'm
asked
n't
go
Lord
came
issue
come
m
Earl
'll
know
everything
Yes
get
big
got
eyes
looked
girl
're
down
But
it

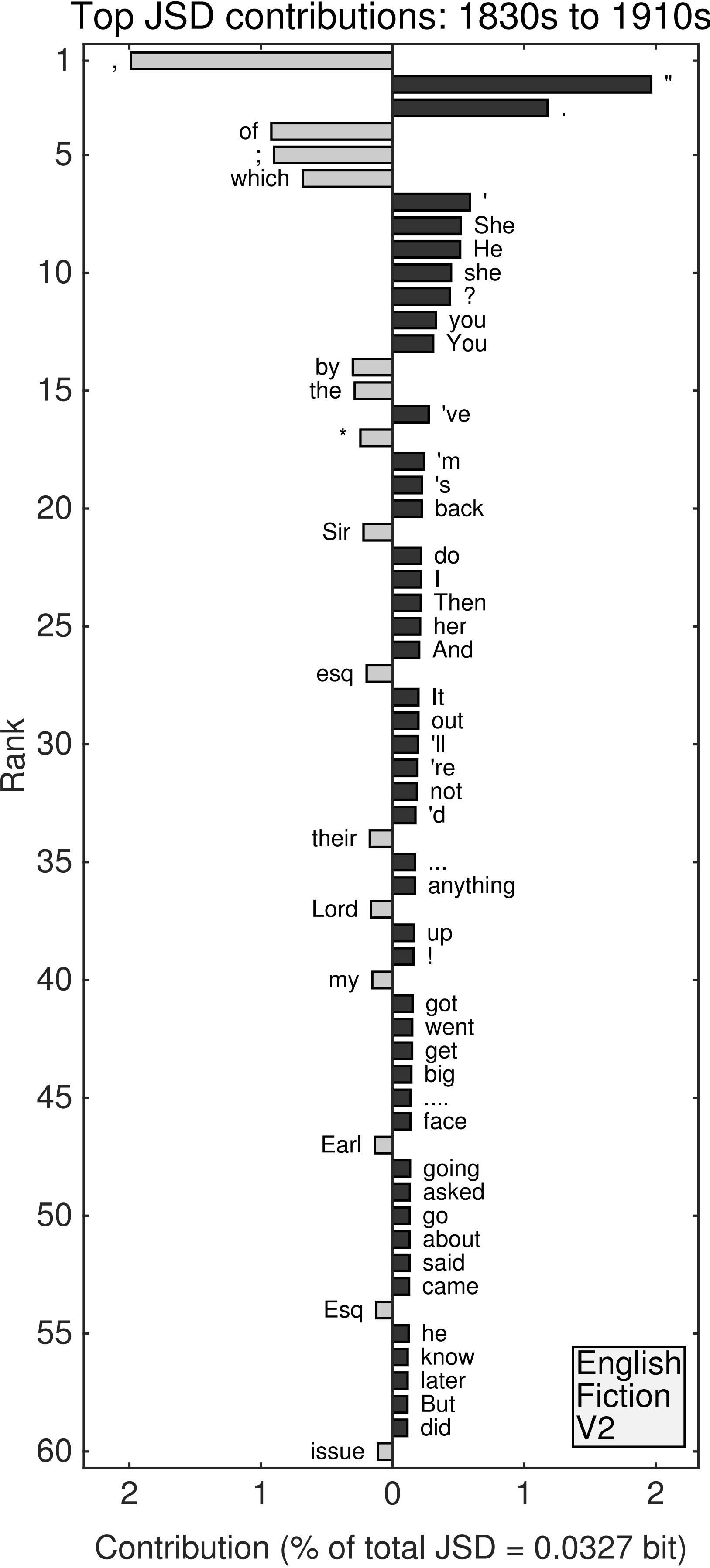
Top JSD contributions: 1830s to 1910s
Rank
Contribution (% of total JSD = 0.0327 bit)
English Fiction V2
,
"
.
of
;
which
'
She
He
she
?
you
You
by
the
've
*
'm
's
back
Sir
do
I
Then
her
And
esq
It
out
'll
're
not
'd
their
...
anything
Lord
up
!
my
got
went
get
big
....
face
Earl
going
asked
go
about
said
came
Esq
he
know
later
But
did
issue
1
5
10
15
20
25
30
35
40
45
50
55
60
2
1
0
1
2

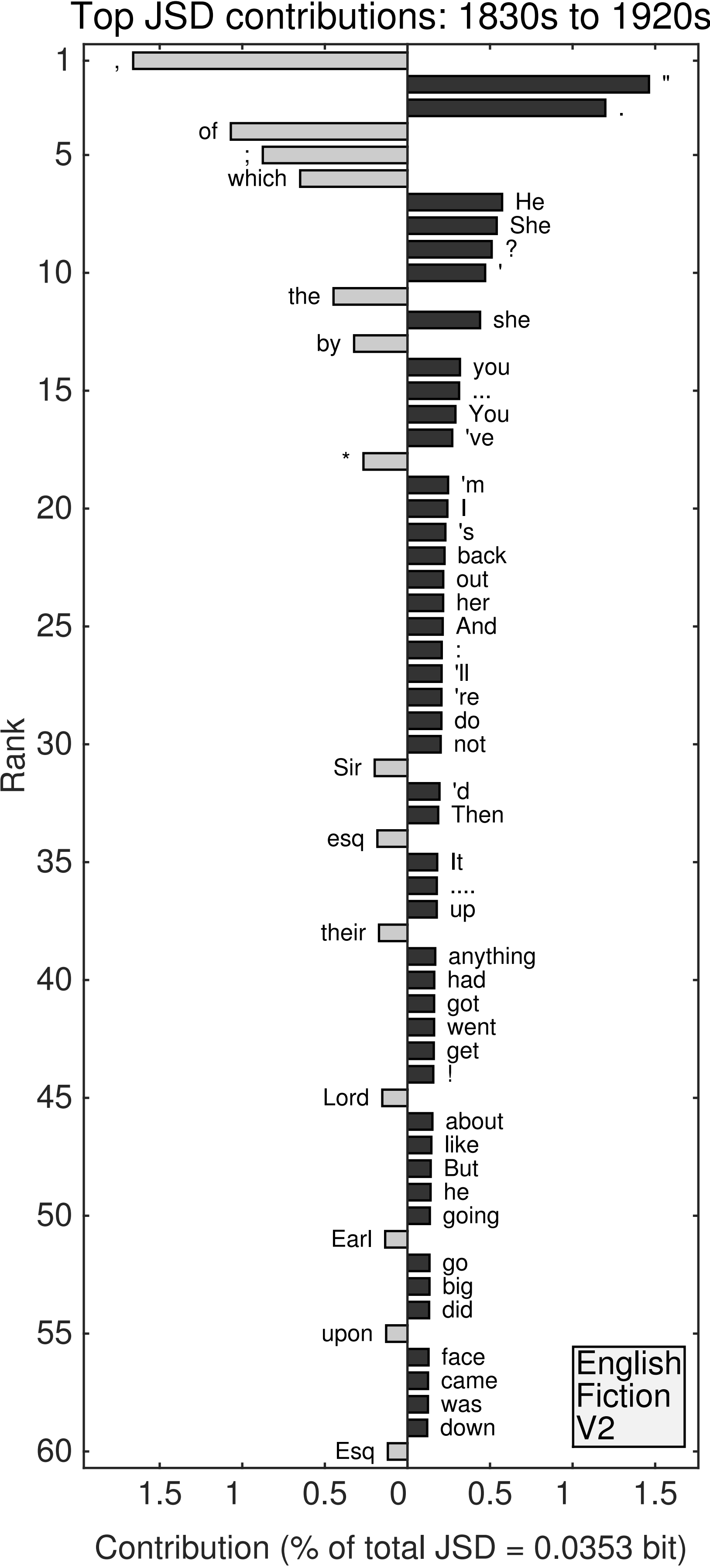

Top JSD contributions: 1830s to 1920s
Rank
Contribution (% of total JSD = 0.0353 bit)
English Fiction V2
1
5
10
15
20
25
30
35
40
45
50
55
60
1.5
1
0.5
0
0.5
1
1.5
,
"
.
of
;
which
He
She
?
'
the
she
by
you
...
You
've
*
'm
I
's
back
out
her
And
:
'll
're
do
not
Sir
'd
Then
esq
It
....
up
their
anything
had
got
went
get
!
Lord
about
like
But
he
going
Earl
go
big
did
upon
face
came
was
down
Esq

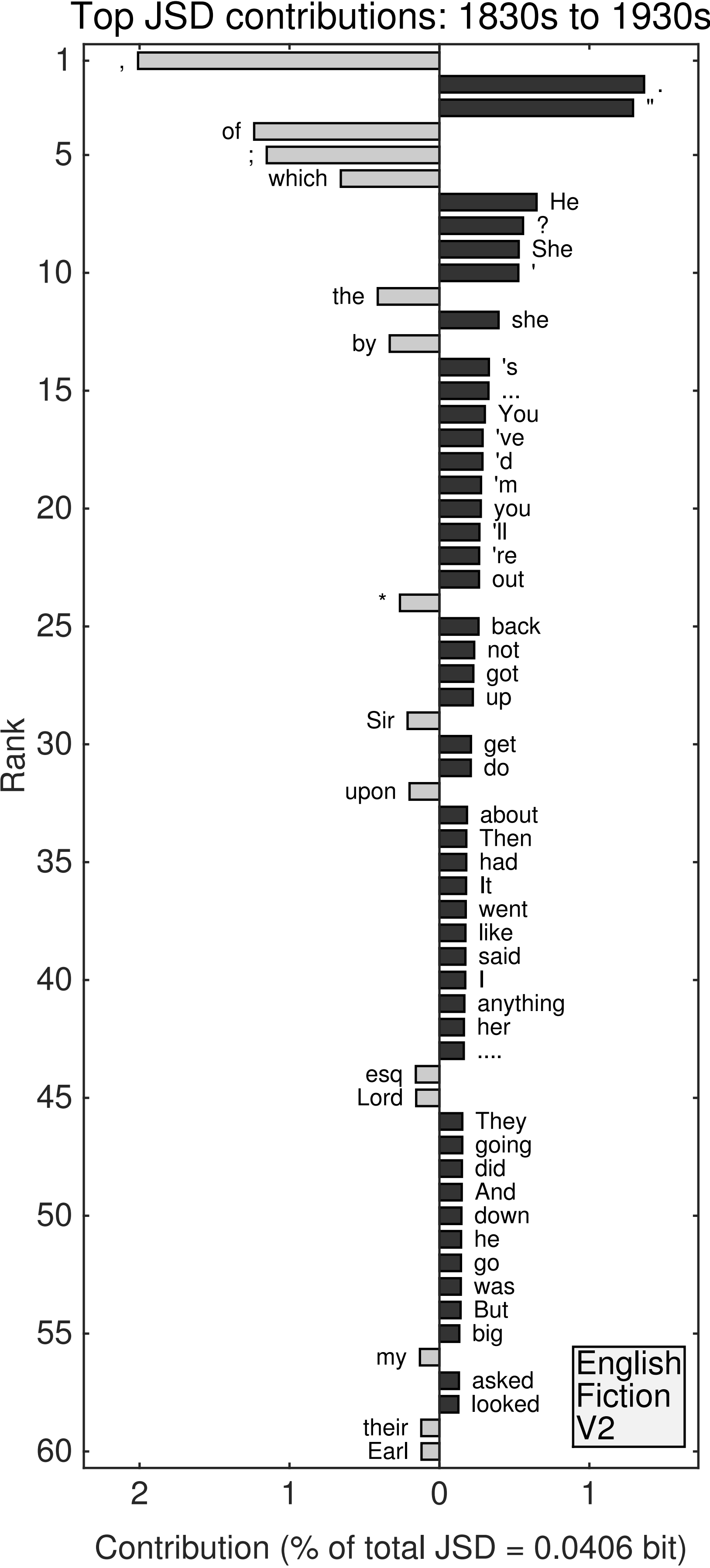
Top JSD contributions: 1830s to 1930s
Rank
Contribution (% of total JSD = 0.0406 bit)
English Fiction V2
,
.
"
of
;
which
He
?
She
'
the
she
by
's
...
You
've
'd
'm
you
'll
're
out
*
back
not
got
up
Sir
get
do
upon
about
Then
had
It
went
like
said
I
anything
her
....
esq
Lord
They
going
did
And
down
he
go
was
But
big
my
asked
looked
their
Earl
1
5
10
15
20
25
30
35
40
45
50
55
60
2
1
0
1

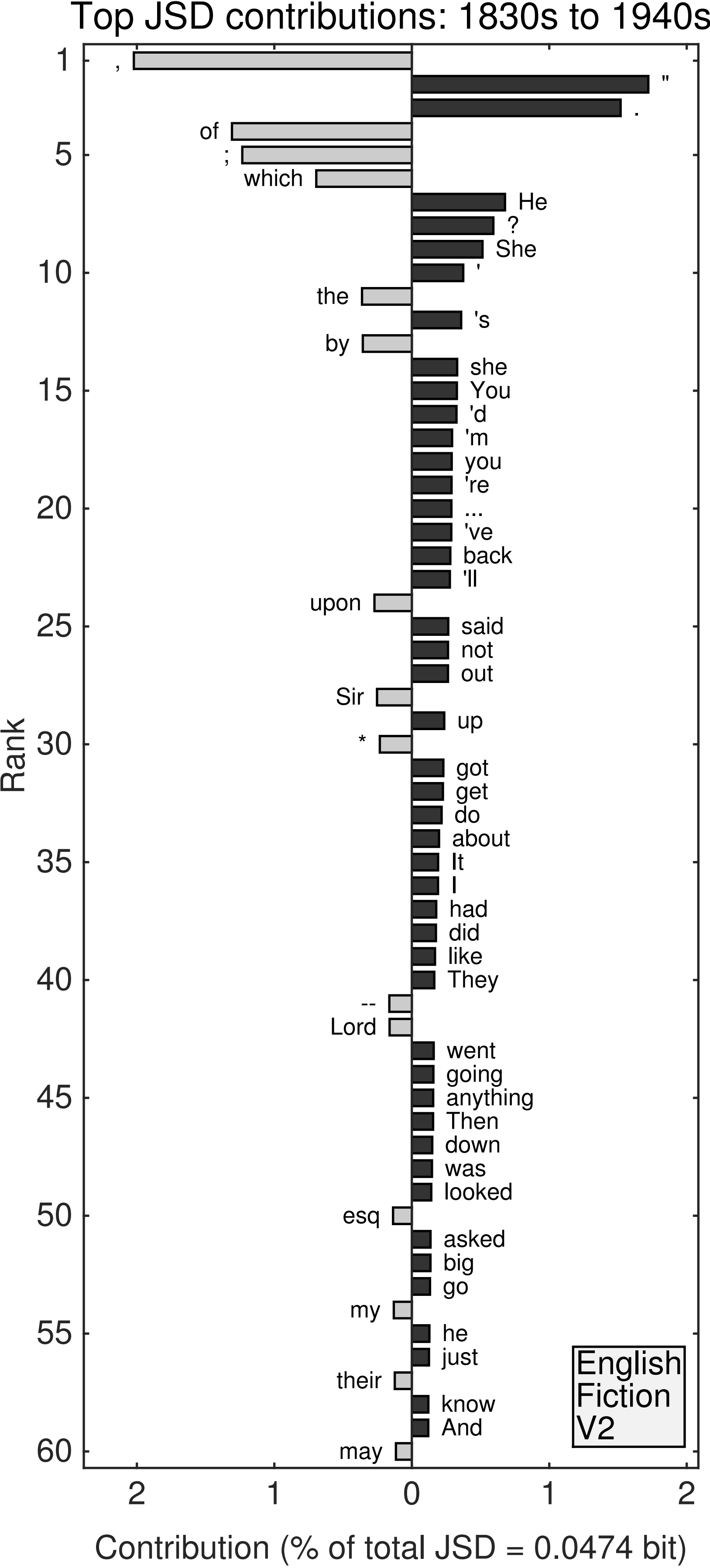

Top JSD contributions: 1830s to 1940s
Rank
Contribution (% of total JSD = 0.0474 bit)
English Fiction V2
,
"
.
of
;
which
He
?
She
'
the
's
by
she
You
'd
'm
you
're
...
've
back
'll
upon
said
not
out
Sir
up
*
got
get
do
about
It
I
had
did
like
They
--
Lord
went
going
anything
Then
down
was
looked
esq
asked
big
go
my
he
just
their
know
And
may

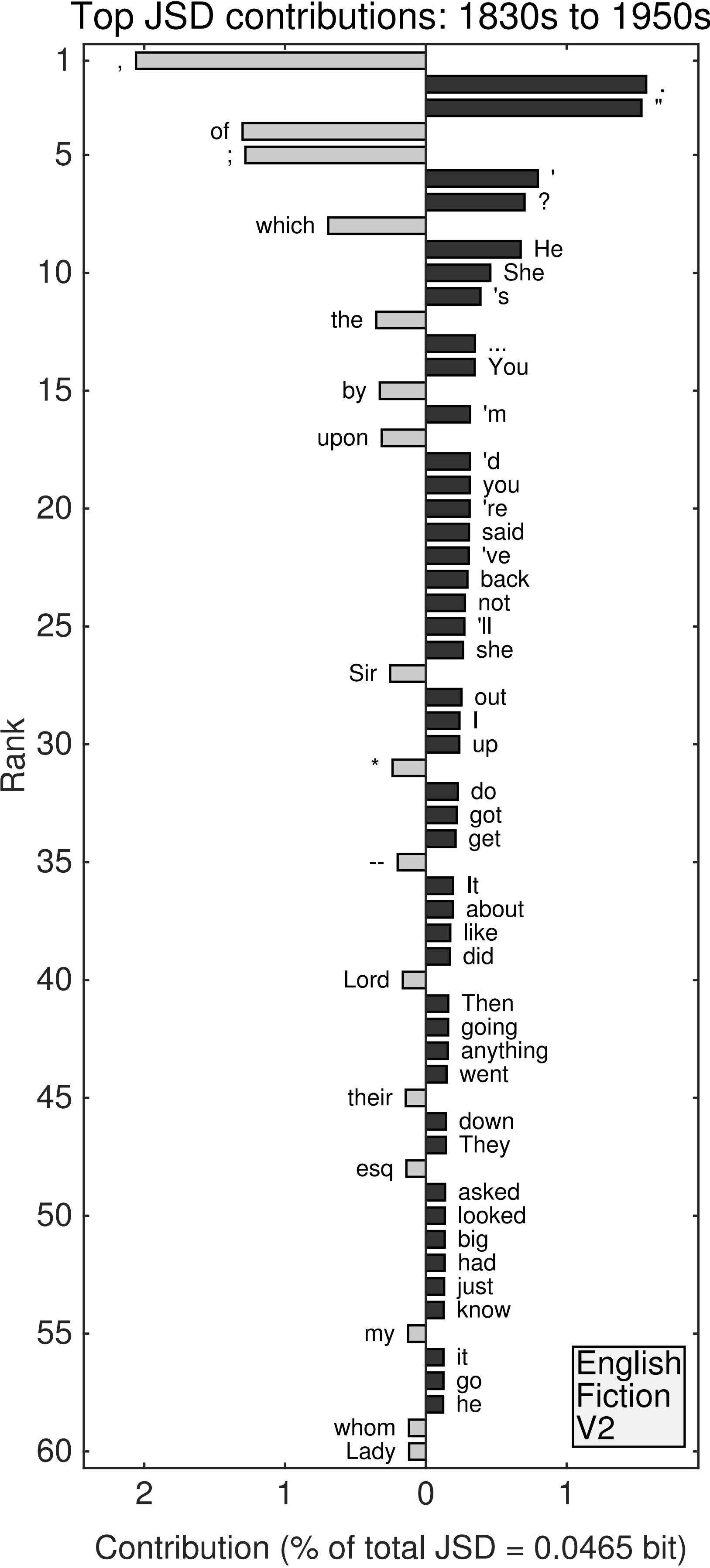

Top JSD contributions: 1830s to 1950s
Rank
Contribution (% of total JSD = 0.0465 bit)
English Fiction V2
1
5
10
15
20
25
30
35
40
45
50
55
60
2
1
0
1
,
.
"
of
;
'
?
which
He
She
's
the
...
You
by
'm
upon
'd
you
're
said
've
back
not
'll
she
Sir
out
I
up
*
do
got
get
--
It
about
like
did
Lord
Then
going
anything
went
their
down
They
esq
asked
looked
big
had
just
know
my
it
go
he
whom
Lady

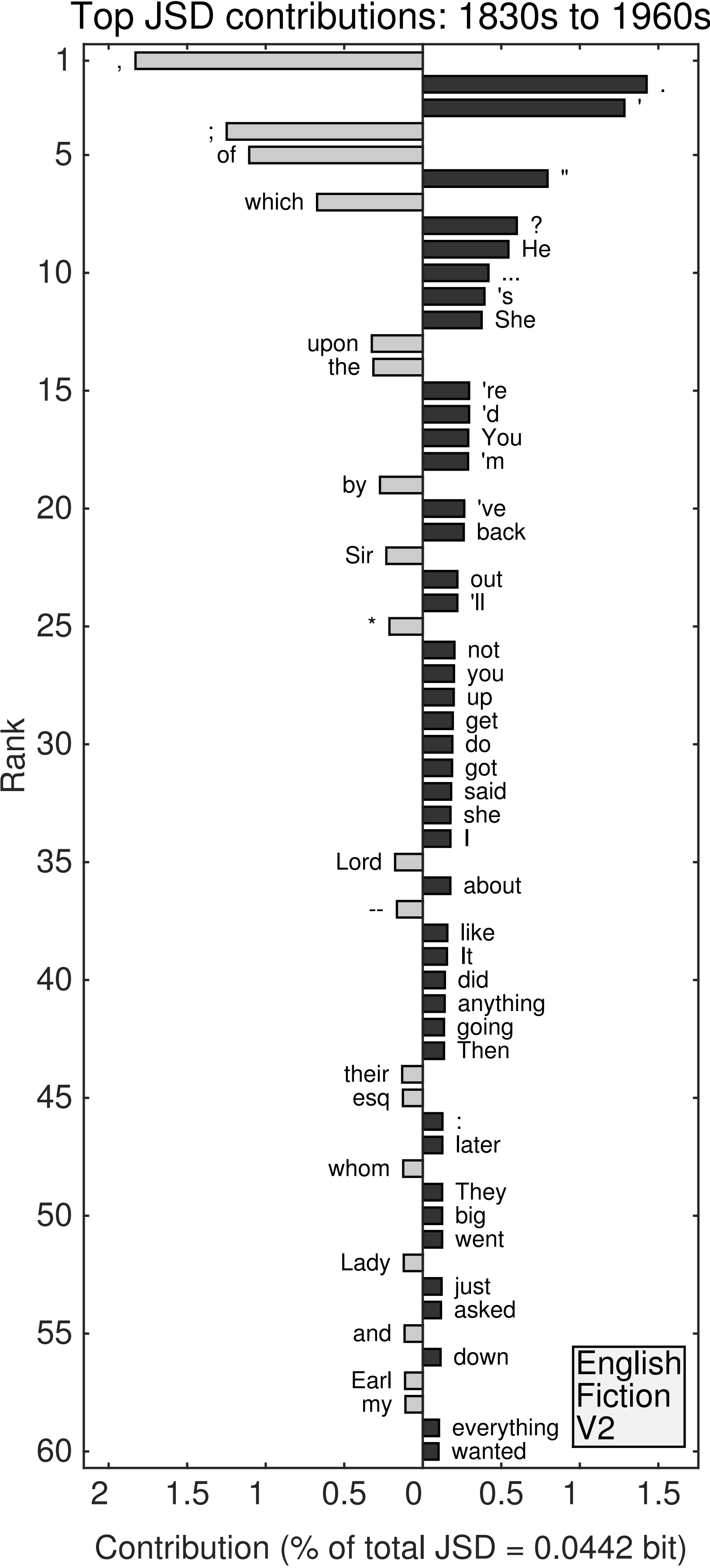
Top JSD contributions: 1830s to 1960s
Rank
Contribution (% of total JSD = 0.0442 bit)
English Fiction V2
,
.
'
;
of
"
which
?
He
...
's
She
upon
the
're
'd
You
'm
by
've
back
Sir
out
'll
*
not
you
up
get
do
got
said
she
I
Lord
about
--
like
It
did
anything
going
Then
their
esq
:
later
whom
They
big
went
Lady
just
asked
and
down
Earl
my
everything
wanted

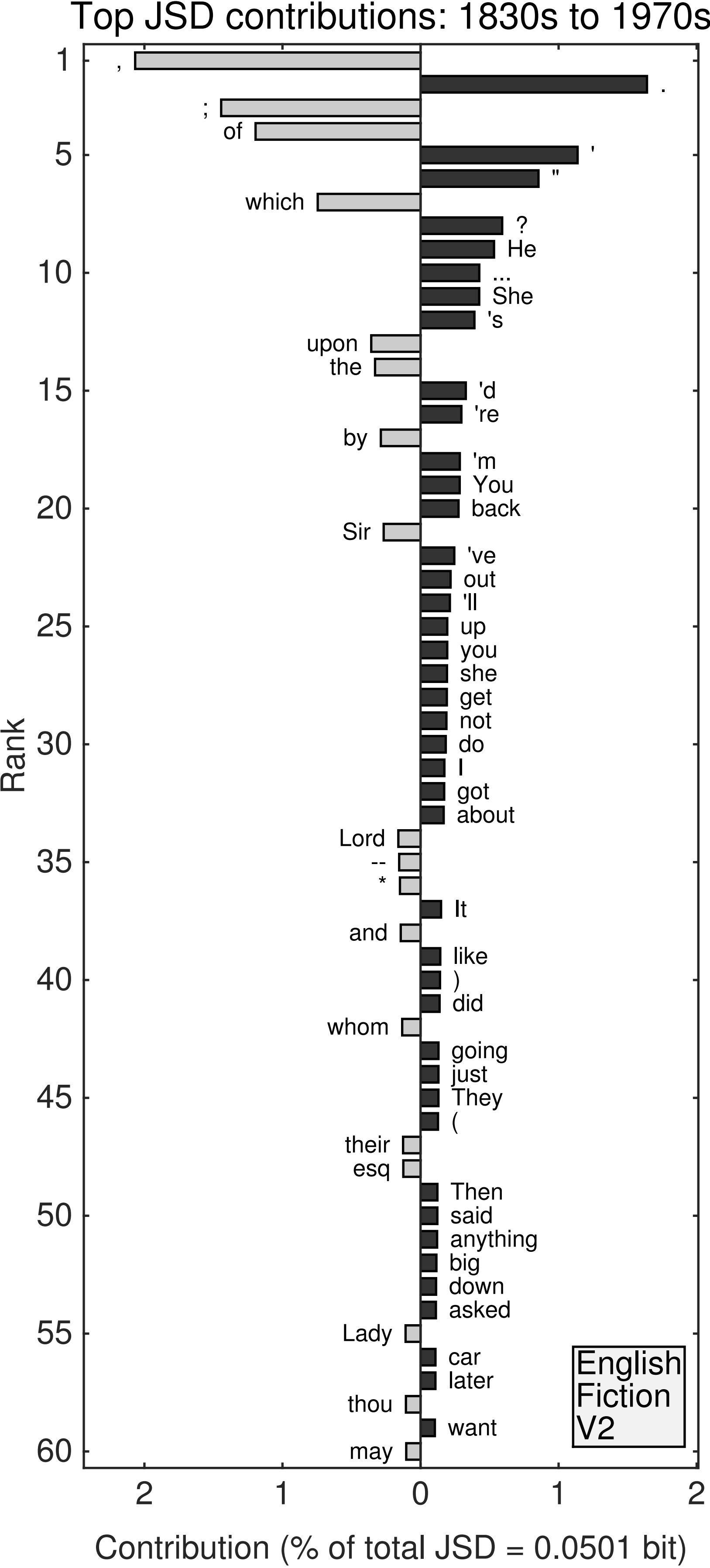

Top JSD contributions: 1830s to 1970s
Rank
Contribution (% of total JSD = 0.0501 bit)
English Fiction V2
,
.
;
of
'
"
which
?
He
...
She
's
upon
the
'd
're
by
'm
You
back
Sir
've
out
'll
up
you
she
get
not
do
I
got
about
Lord
--
*
It
and
like
)
did
whom
going
just
They
(
their
esq
Then
said
anything
big
down
asked
Lady
car
later
thou
want
may

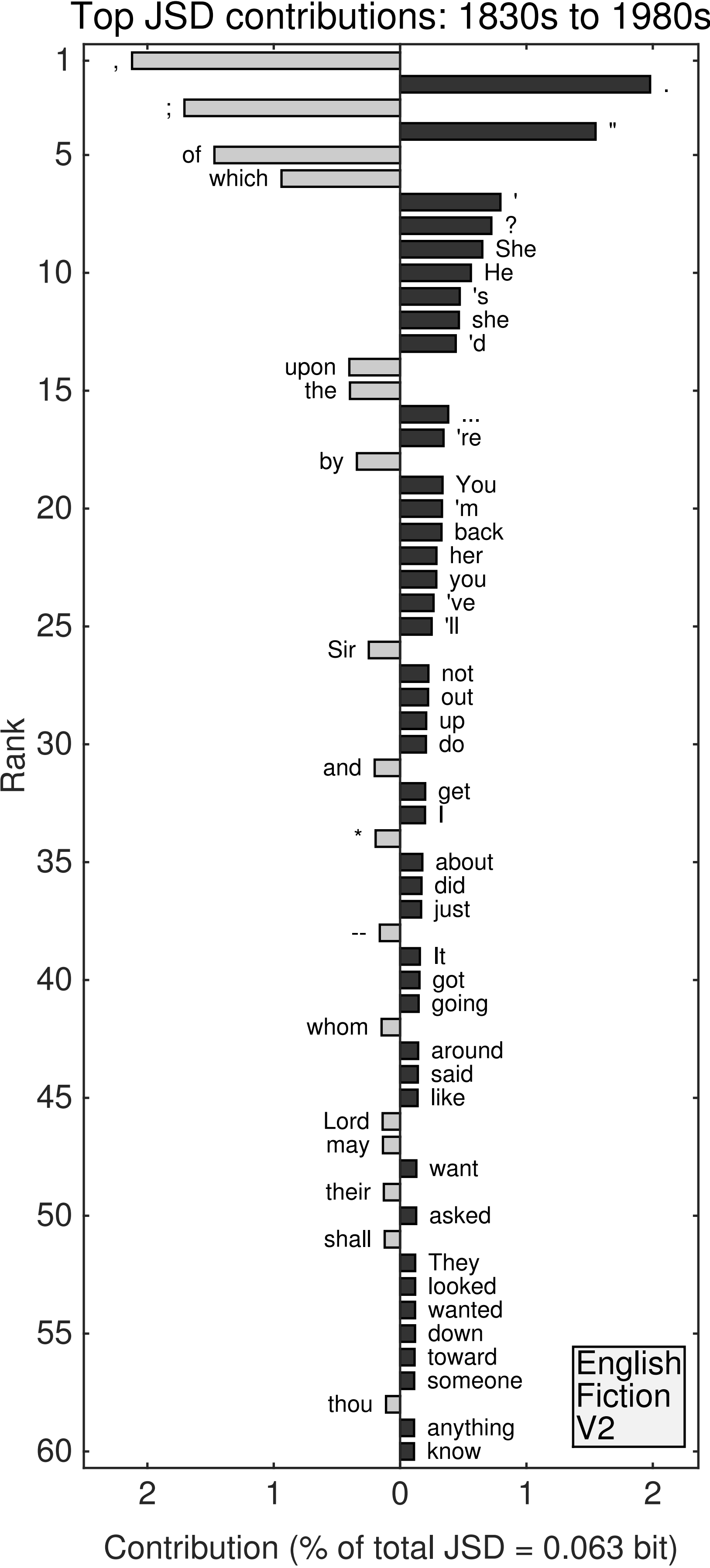
Top JSD contributions: 1830s to 1980s
Rank
Contribution (% of total JSD = 0.063 bit)
English Fiction V2
,
.
;
"
of
which
'
?
She
He
's
she
'd
upon
the
...
're
by
You
'm
back
her
you
've
'll
Sir
not
out
up
do
and
get
I
*
about
did
just
--
It
got
going
whom
around
said
like
Lord
may
want
their
asked
shall
They
looked
wanted
down
toward
someone
thou
anything
know

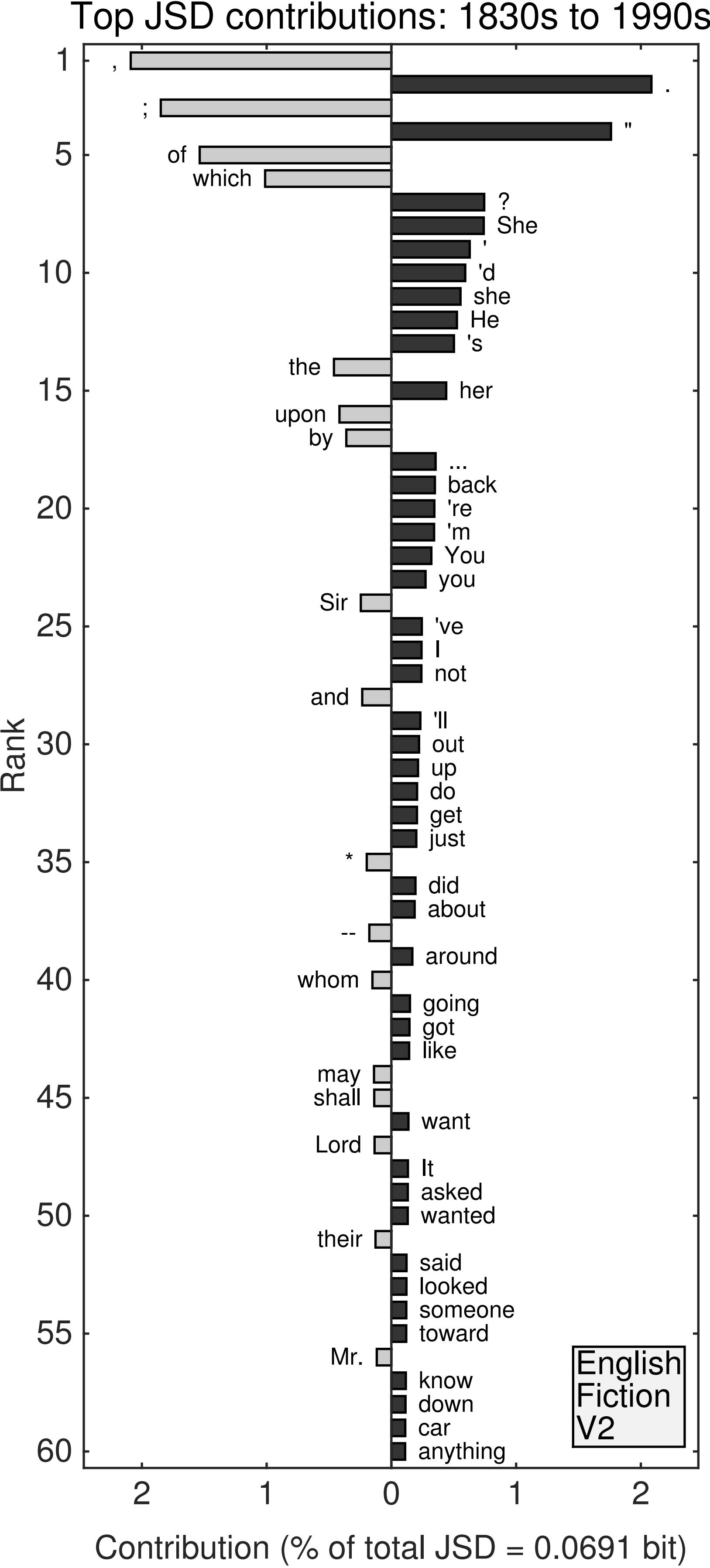
Top JSD contributions: 1830s to 1990s
Rank
Contribution (% of total JSD = 0.0691 bit)
English Fiction V2
1
5
10
15
20
25
30
35
40
45
50
55
60
2
1
0
1
2
,
.
;
"
of
which
?
She
'
'd
she
He
's
the
her
upon
by
...
back
're
'm
You
you
Sir
've
I
not
and
'll
out
up
do
get
just
*
did
about
--
around
whom
going
got
like
may
shall
want
Lord
It
asked
wanted
their
said
looked
someone
toward
Mr.
know
down
car
anything

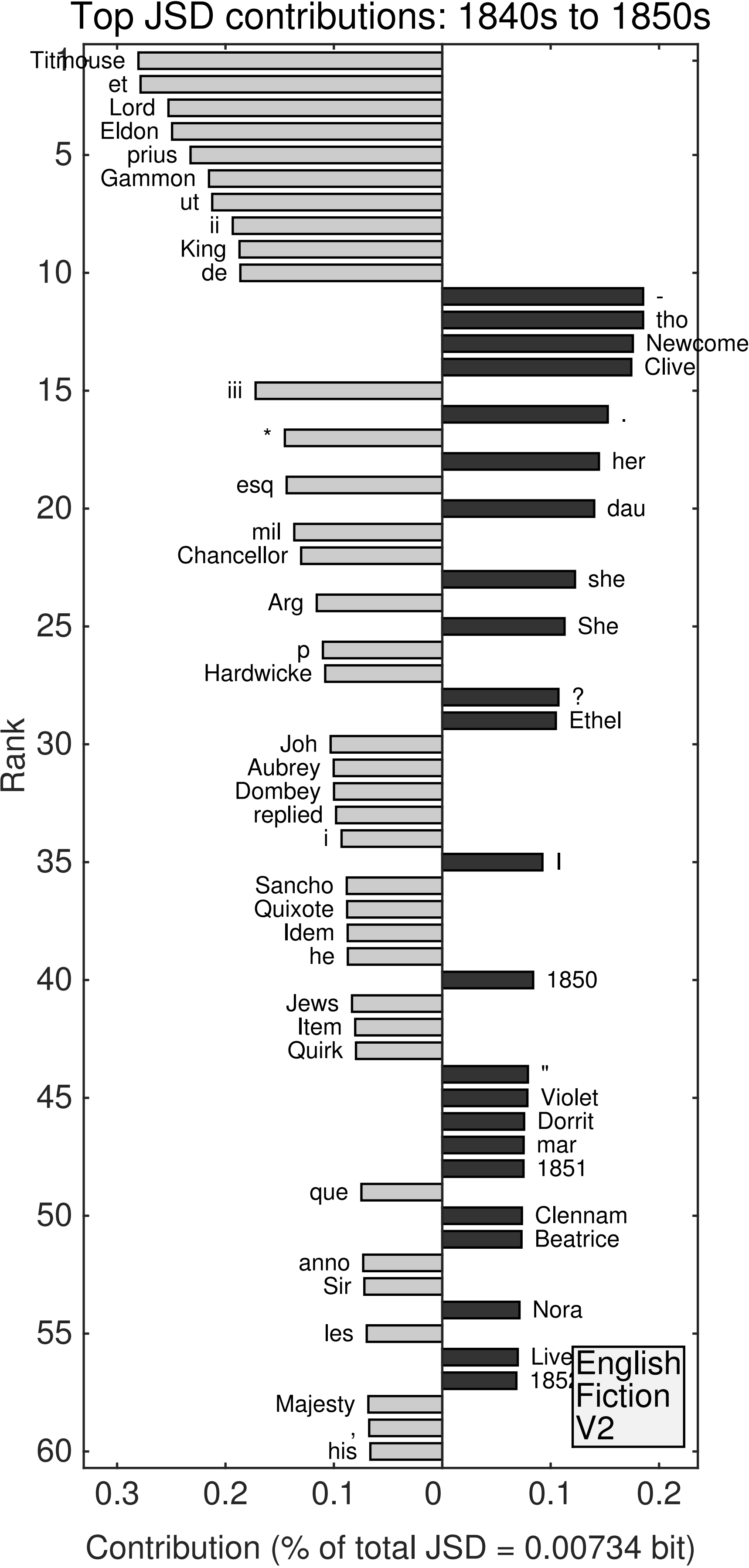
Top JSD contributions: 1840s to 1850s
Rank
Contribution (% of total JSD = 0.00734 bit)
0.3
0.2
0.1
0
0.1
0.2
1
5
10
15
20
25
30
35
40
45
50
55
60
Titmouse
et
Lord
Eldon
prius
Gammon
ut
ii
King
de
-
tho
Newcome
Clive
iii
.
*
her
esq
dau
mil
Chancellor
she
Arg
She
p
Hardwicke
?
Ethel
Joh
Aubrey
Dombey
replied
i
I
Sancho
Quixote
Idem
he
1850
Jews
Item
Quirk
"
Violet
Dorrit
mar
1851
que
Clennam
Beatrice
anno
Sir
Nora
les
Majesty
,
his
English
Fiction
V2

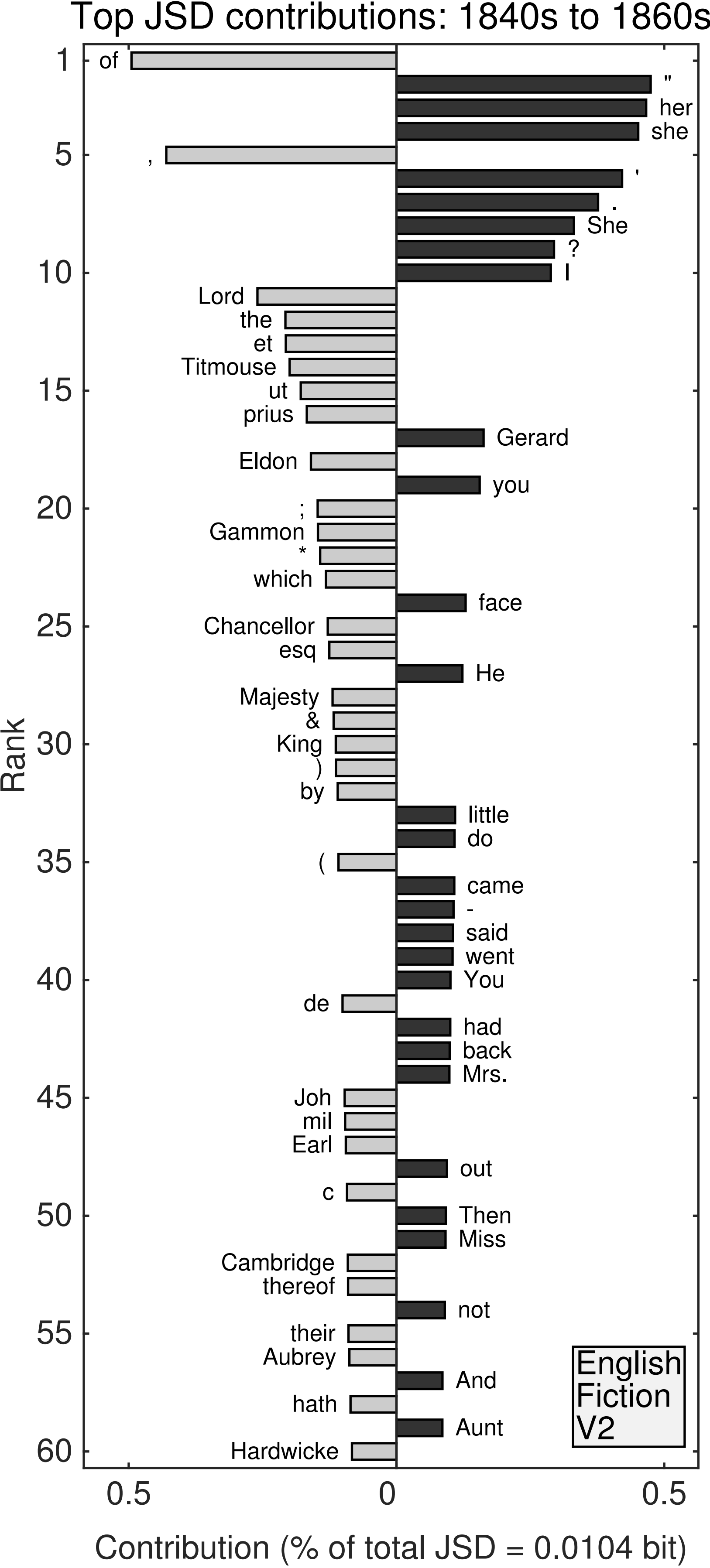
Top JSD contributions: 1840s to 1860s
Rank
Contribution (% of total JSD = 0.0104 bit)
English Fiction V2
of
"
her
she
,
'
.
She
?
I
Lord
the
et
Titmouse
ut
prius
Gerard
Eldon
you
;
Gammon
*
which
face
Chancellor
esq
He
Majesty
&
King
)
by
little
do
(
came
-
said
went
You
de
had
back
Mrs.
Joh
mil
Earl
out
c
Then
Miss
Cambridge
thereof
not
their
Aubrey
And
hath
Aunt
Hardwicke
1
5
10
15
20
25
30
35
40
45
50
55
60
0.5
0
0.5

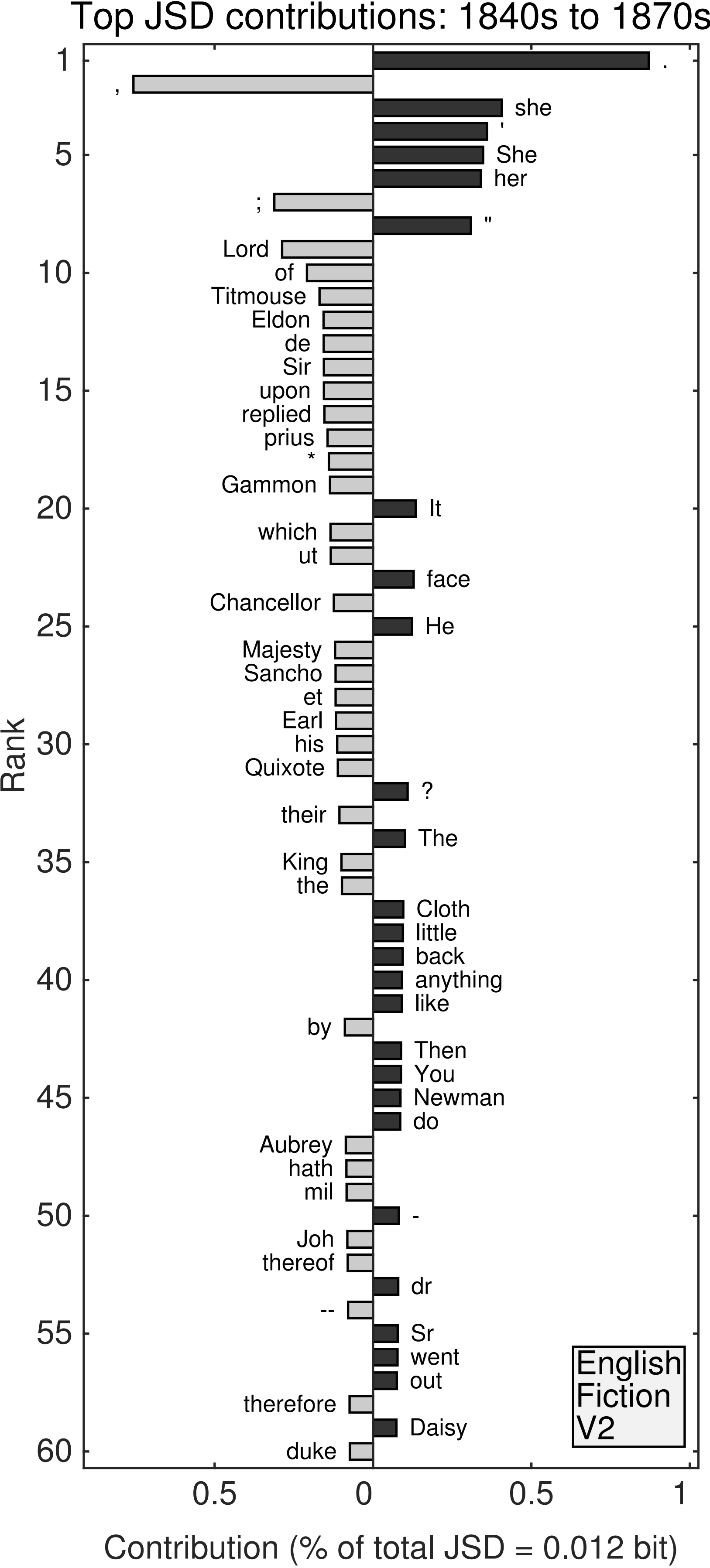
Top JSD contributions: 1840s to 1870s
Rank
Contribution (% of total JSD = 0.012 bit)
English Fiction V2
.
,
she
'
She
her
;
"
Lord
of
Titmouse
Eldon
de
Sir
upon
replied
prius
*
Gammon
It
which
ut
face
Chancellor
He
Majesty
Sancho
et
Earl
his
Quixote
?
their
The
King
the
Cloth
little
back
anything
like
by
Then
You
Newman
do
Aubrey
hath
mil
-
Joh
thereof
dr
--
Sr
went
out
therefore
Daisy
duke

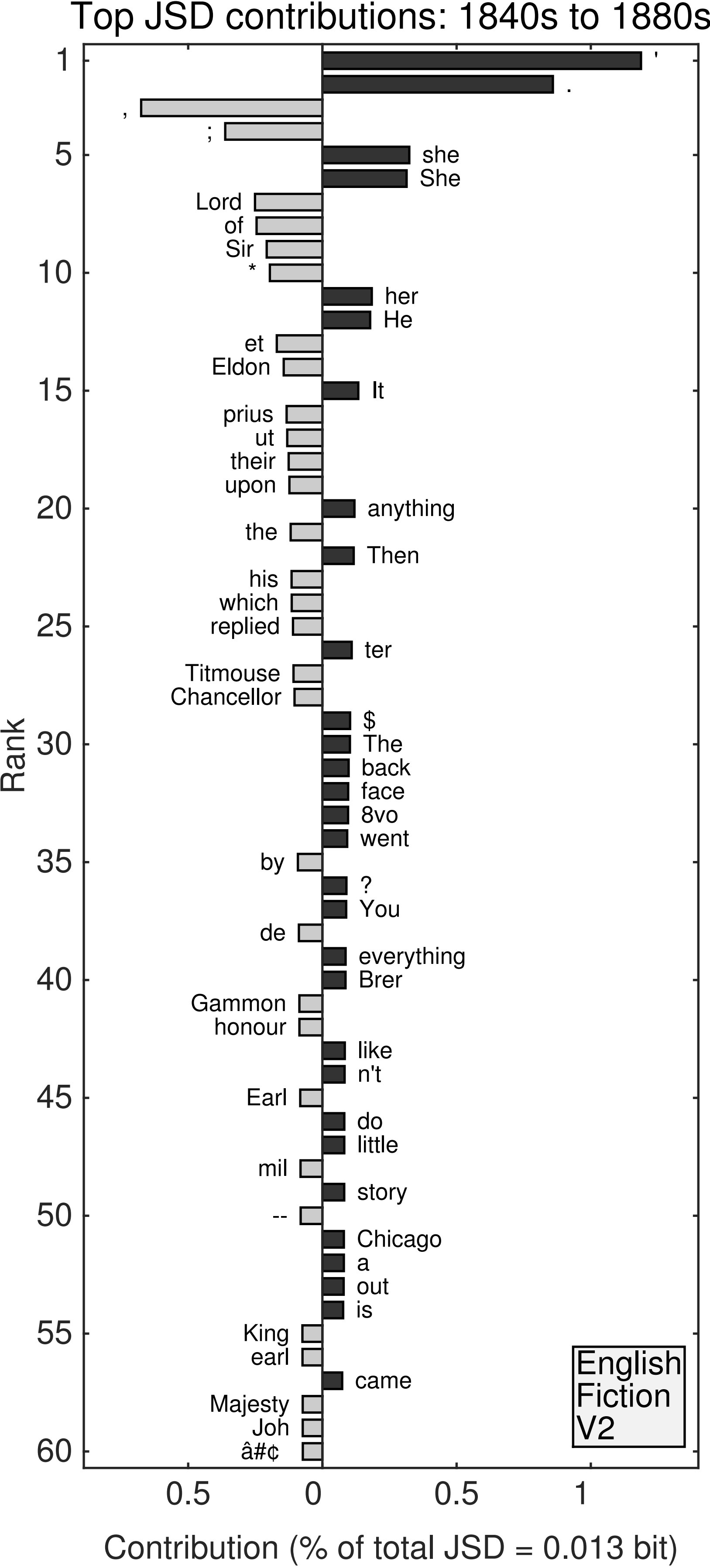
Top JSD contributions: 1840s to 1880s
'
.
,
;
she
She
Lord
of
Sir
*
her
He
et
Eldon
It
prius
ut
their
upon
anything
the
Then
his
which
replied
ter
Titmouse
Chancellor
$
The
back
face
8vo
went
by
?
You
de
everything
Brer
Gammon
honour
like
n't
Earl
do
little
mil
story
--
Chicago
a
out
is
King
earl
came
Majesty
Joh
â#¢
English
Fiction
V2
Rank
1
5
10
15
20
25
30
35
40
45
50
55
60
0.5
0
0.5
1
Contribution (% of total JSD = 0.013 bit)

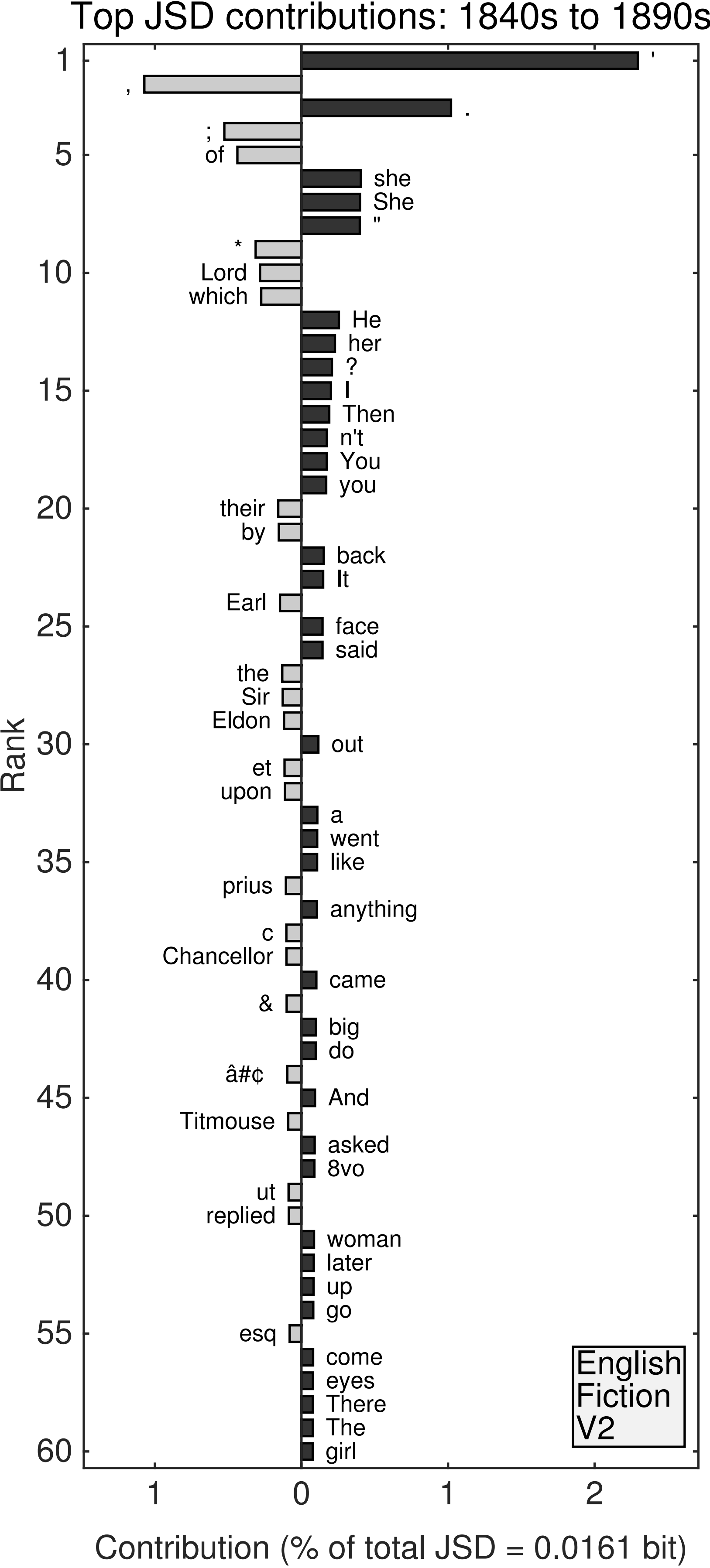

Top JSD contributions: 1840s to 1890s
Rank
Contribution (% of total JSD = 0.0161 bit)
English Fiction V2
'
,
.
;
of
she
She
"
*
Lord
which
He
her
?
I
Then
n't
You
you
their
by
back
It
Earl
face
said
the
Sir
Eldon
out
et
upon
a
went
like
prius
anything
c
Chancellor
came
&
big
do
â#¢
And
Titmouse
asked
8vo
ut
replied
woman
later
up
go
esq
come
eyes
There
The
girl

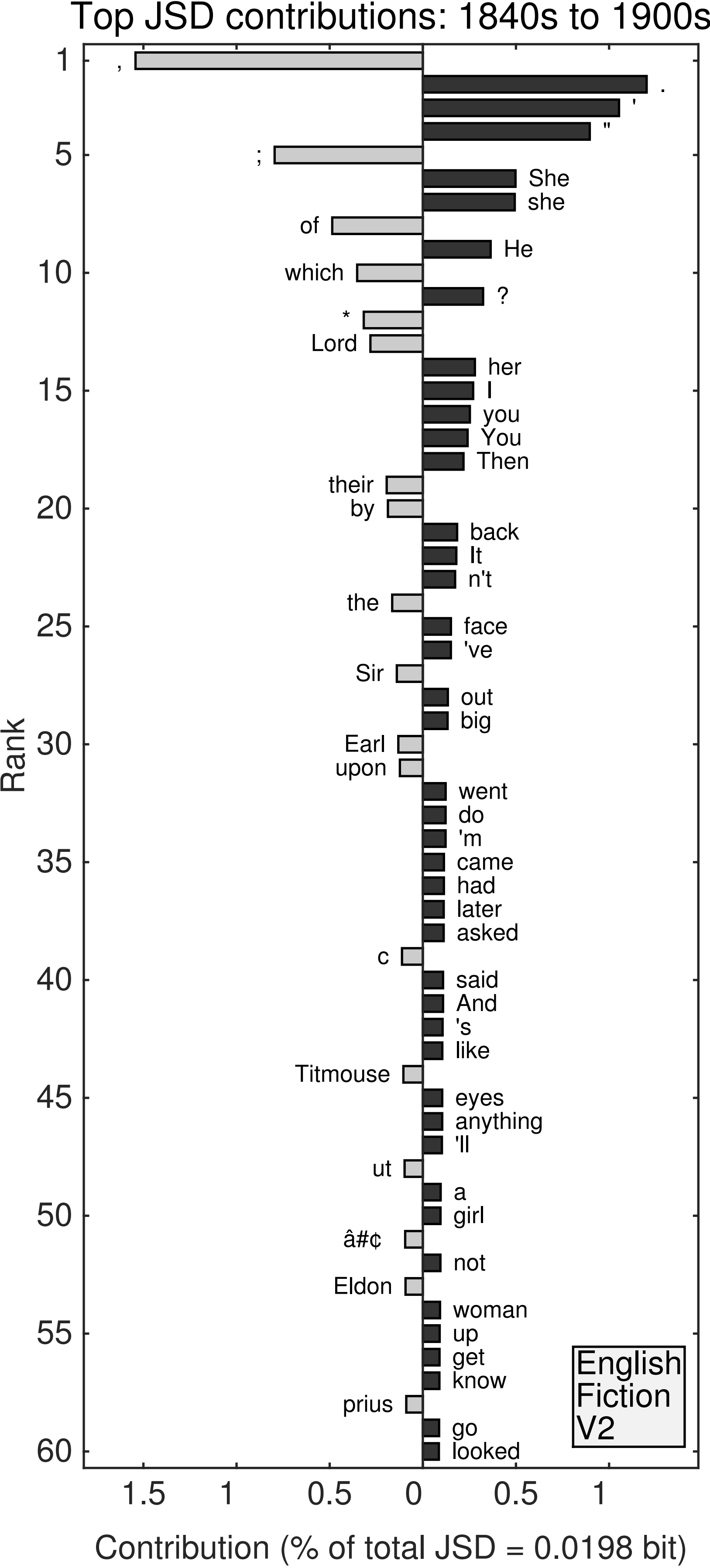
Top JSD contributions: 1840s to 1900s
Rank
Contribution (% of total JSD = 0.0198 bit)
English Fiction V2
,
.
'
"
;
She
she
of
He
which
?
*
Lord
her
I
you
You
Then
their
by
back
It
n't
the
face
've
Sir
out
big
Earl
upon
went
do
'm
came
had
later
asked
c
said
And
's
like
Titmouse
eyes
anything
'll
ut
a
girl
â#¢
not
Eldon
woman
up
get
know
prius
go
looked

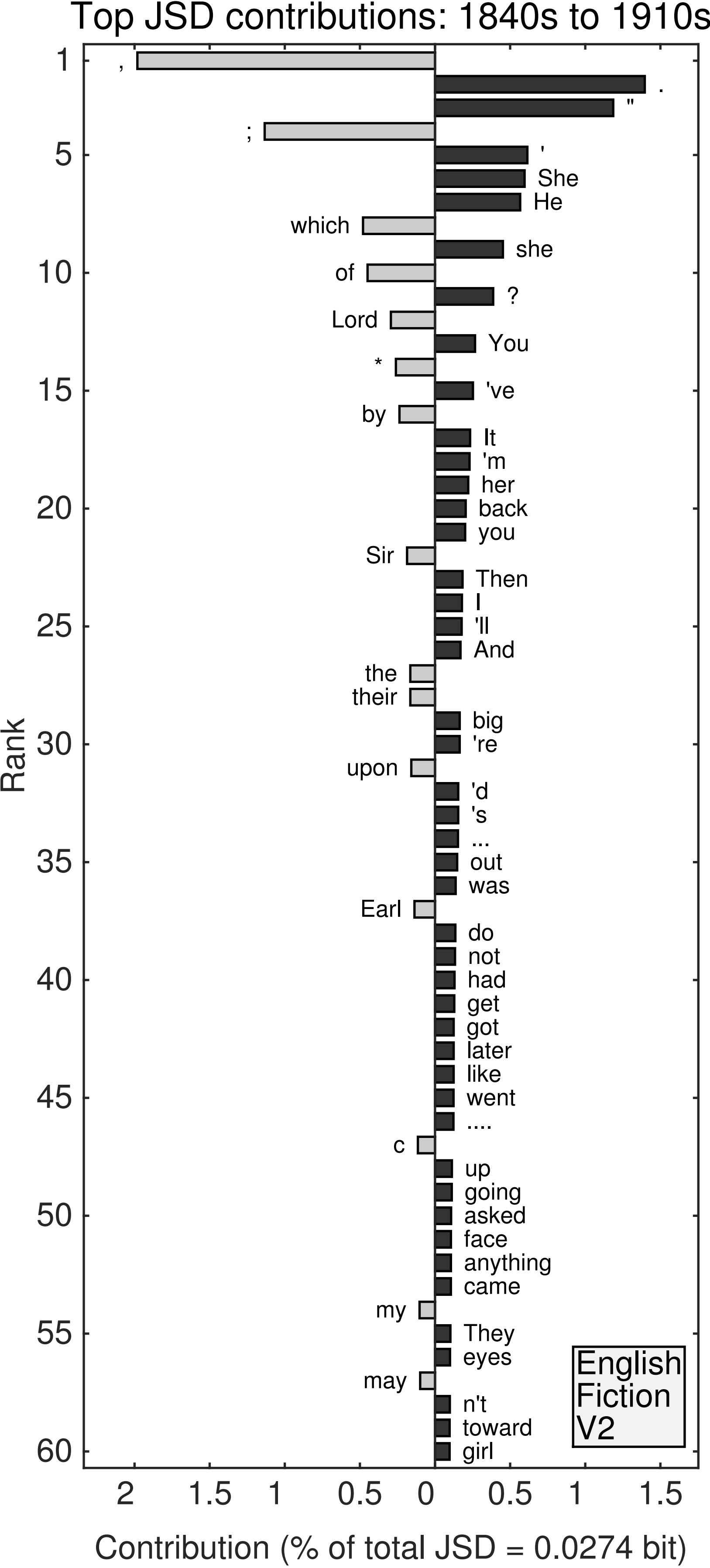

Top JSD contributions: 1840s to 1910s
Rank
Contribution (% of total JSD = 0.0274 bit)
English Fiction V2
,
.
"
;
'
She
He
which
she
of
?
Lord
You
*
've
by
It
'm
her
back
you
Sir
Then
I
'll
And
the
their
big
're
upon
'd
's
...
out
was
Earl
do
not
had
get
got
later
like
went
....
c
up
going
asked
face
anything
came
my
They
eyes
may
n't
toward
girl

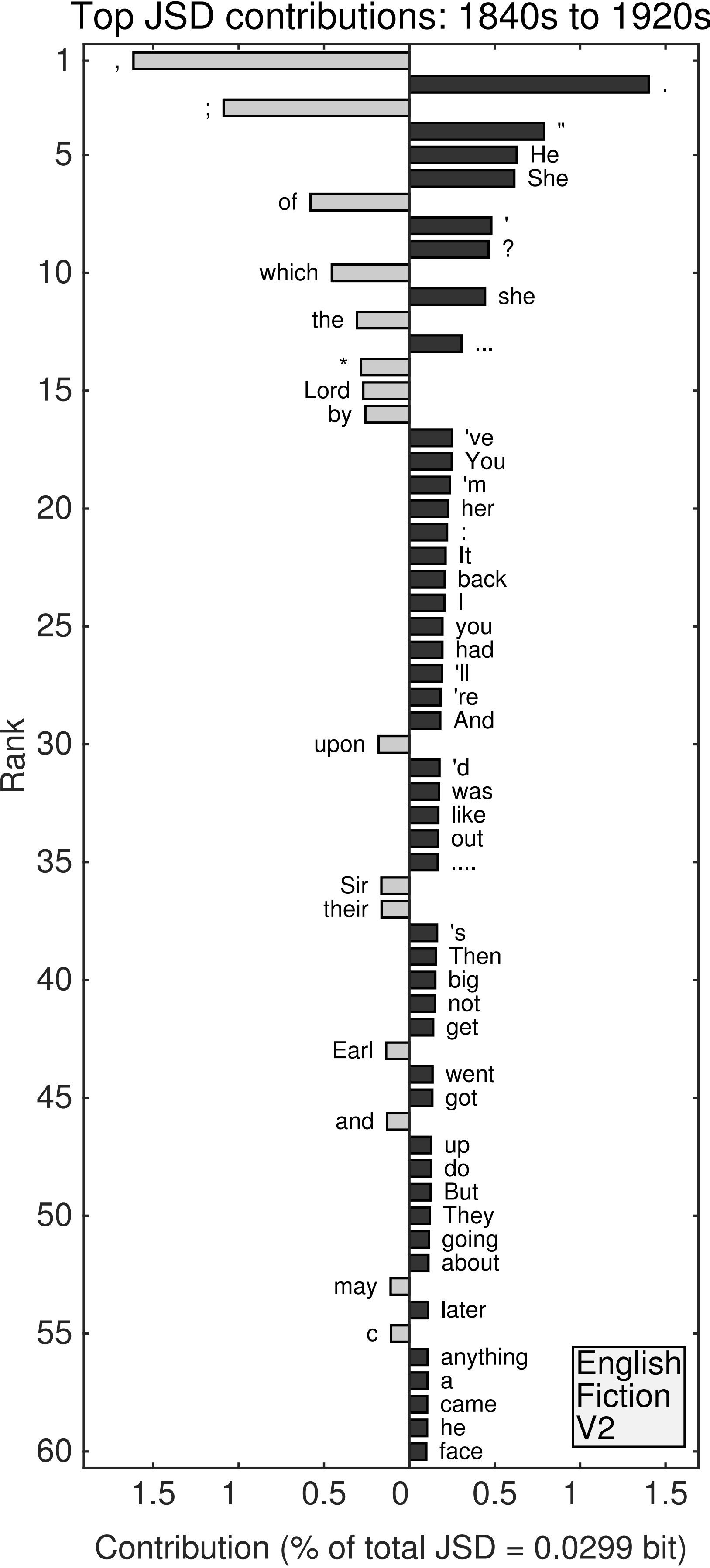

Top JSD contributions: 1840s to 1920s
Rank
Contribution (% of total JSD = 0.0299 bit)
1.5 1 0.5 0 0.5 1 1.5
1 5 10 15 20 25 30 35 40 45 50 55 60
,
.
;
"
He
She
of
'
?
which
she
the
...
*
Lord
by
've
You
'm
her
:
It
back
I
you
had
'll
're
And
upon
'd
was
like
out
....
Sir
their
's
Then
big
not
get
Earl
went
got
and
up
do
But
They
going
about
may
later
c
anything
a
came
he
face
English
Fiction
V2

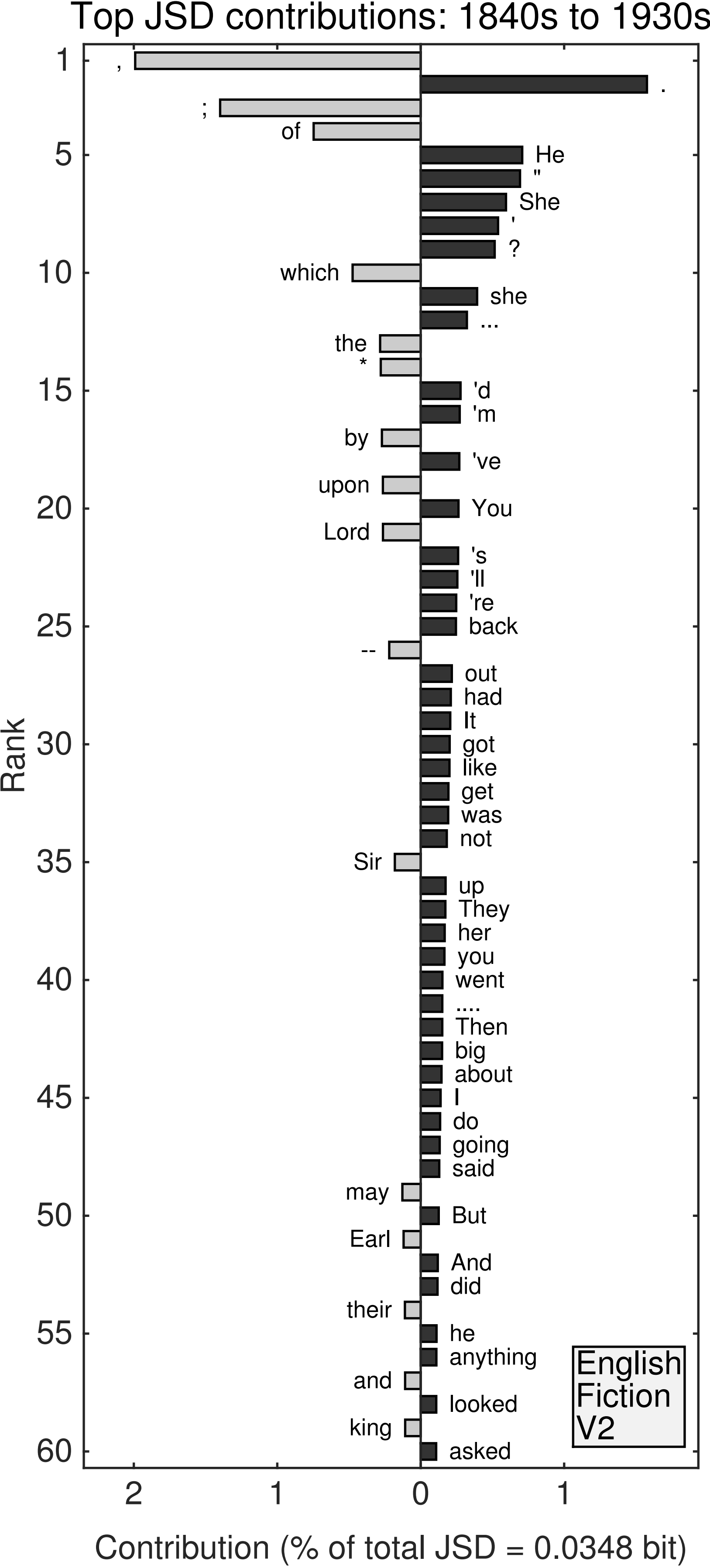

Top JSD contributions: 1840s to 1930s
Rank
Contribution (% of total JSD = 0.0348 bit)
English Fiction V2
, . ; of He " She ' ? which she ... the * 'd 'm by 've upon You Lord 's 'll 're back -- out had It got like get was not Sir up They her you went .... Then big about I do going said may But Earl And did their he anything and looked king asked
1 5 10 15 20 25 30 35 40 45 50 55 60
2 1 0 1

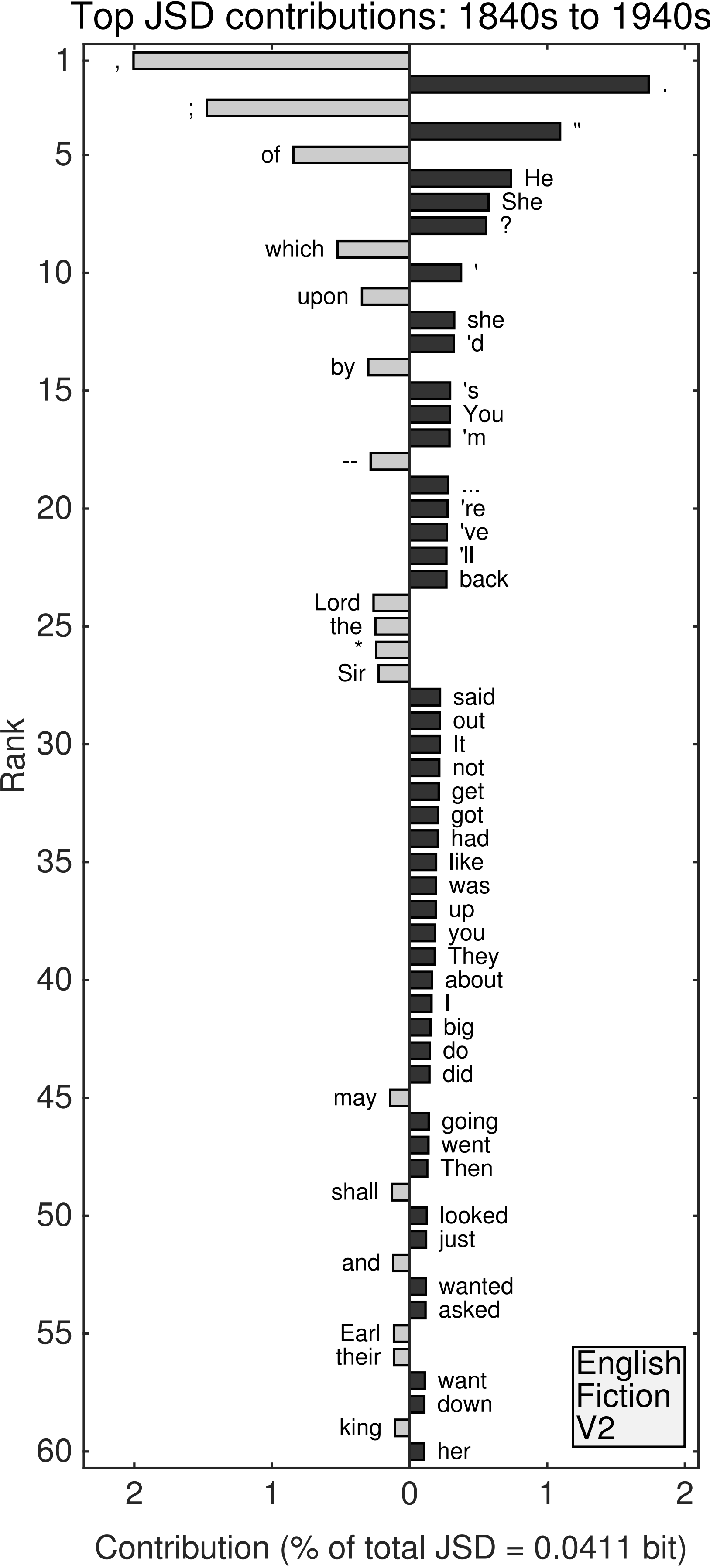

Top JSD contributions: 1840s to 1940s
Rank
Contribution (% of total JSD = 0.0411 bit)
English Fiction V2
,
.
;
"
of
He
She
?
which
'
upon
she
'd
by
's
You
'm
--
...
're
've
'll
back
Lord
the
*
Sir
said
out
It
not
get
got
had
like
was
up
you
They
about
I
big
do
did
may
going
went
Then
shall
looked
just
and
wanted
asked
Earl
their
want
down
king
her

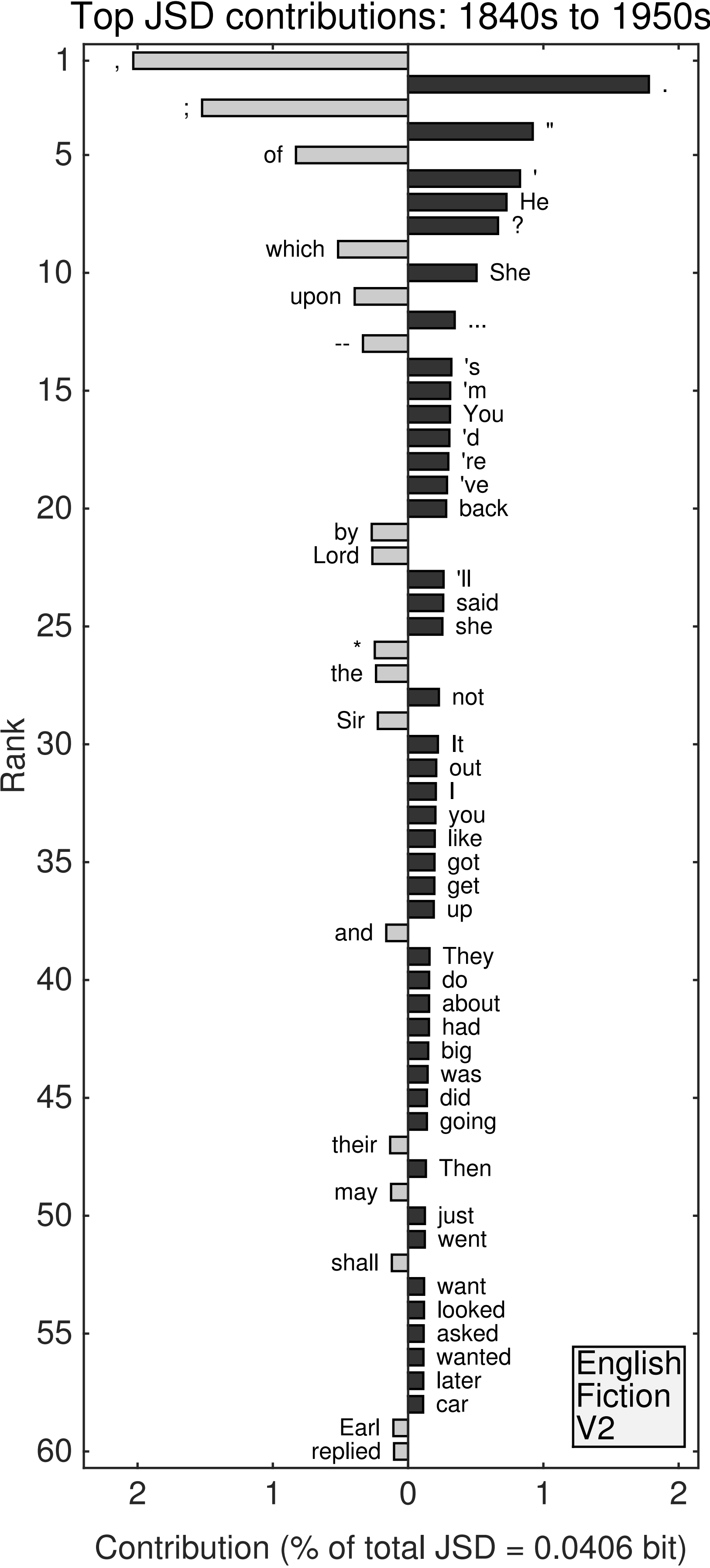
Top JSD contributions: 1840s to 1950s
Rank
Contribution (% of total JSD = 0.0406 bit)
English Fiction V2
1
5
10
15
20
25
30
35
40
45
50
55
60
2
1
0
1
2
,
.
;
"
of
'
He
?
which
She
upon
...
--
's
'm
You
'd
're
've
back
by
Lord
'll
said
she
*
the
not
Sir
It
out
I
you
like
got
get
up
and
They
do
about
had
big
was
did
going
their
Then
may
just
went
shall
want
looked
asked
wanted
later
car
Earl
replied

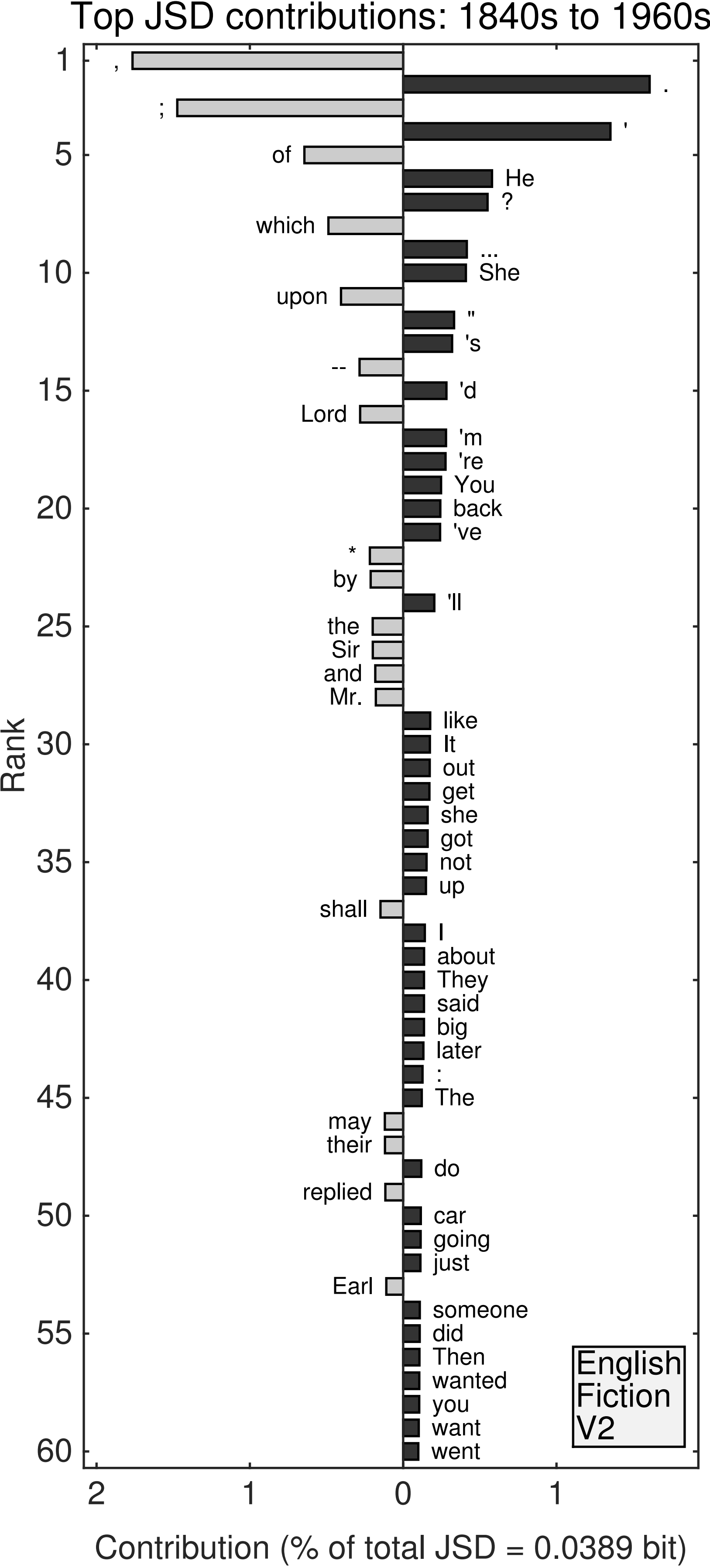

Top JSD contributions: 1840s to 1960s
Rank
Contribution (% of total JSD = 0.0389 bit)
English Fiction V2
,
.
;
'
of
He
?
which
...
She
upon
"
's
--
'd
Lord
'm
're
You
back
've
*
by
'll
the
Sir
and
Mr.
like
It
out
get
she
got
not
up
shall
I
about
They
said
big
later
:
The
may
their
do
replied
car
going
just
Earl
someone
did
Then
wanted
you
want
went

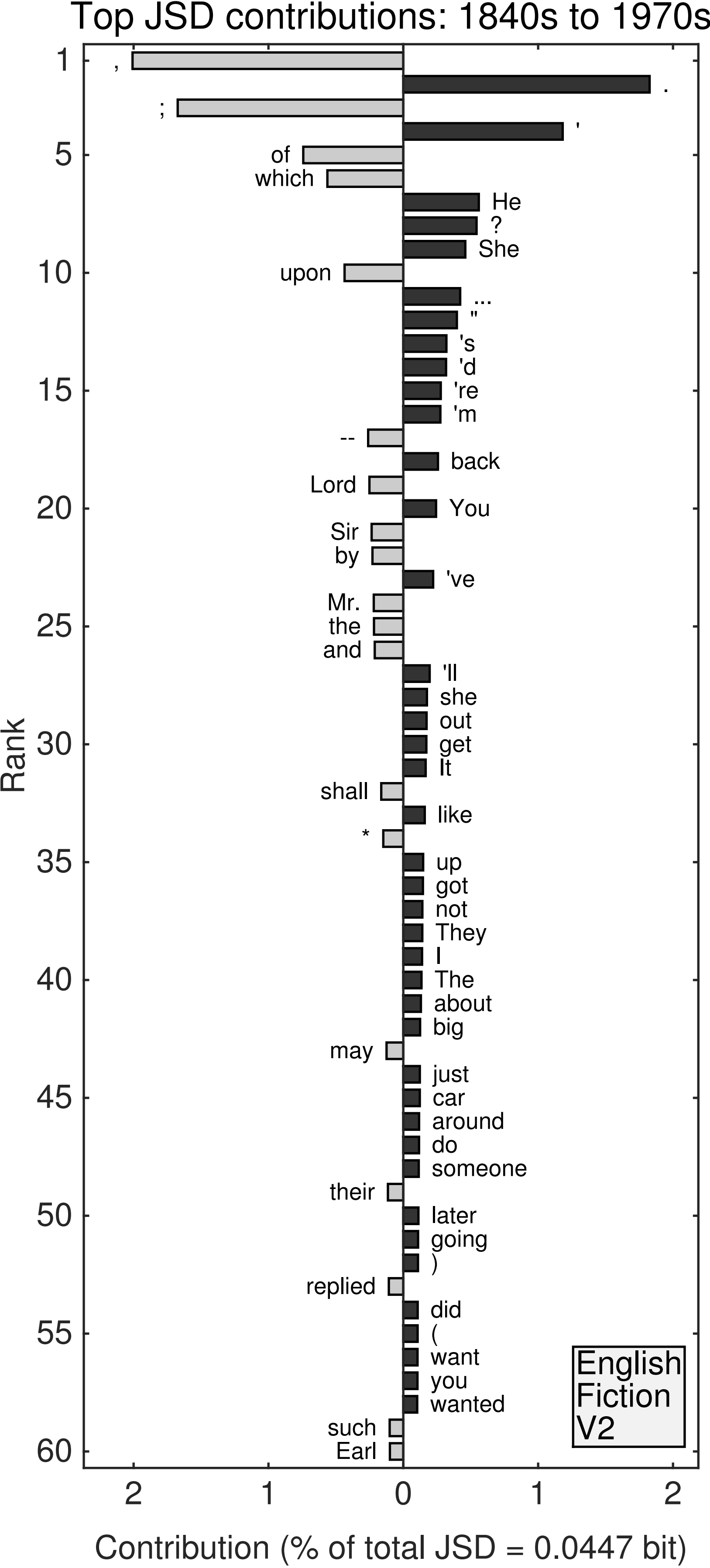
Top JSD contributions: 1840s to 1970s
Rank
Contribution (% of total JSD = 0.0447 bit)
English Fiction V2
1
5
10
15
20
25
30
35
40
45
50
55
60
2
1
0
1
2
,
.
;
'
of
which
He
?
She
upon
...
"
's
'd
're
'm
--
back
Lord
You
Sir
by
've
Mr.
the
and
'll
she
out
get
It
shall
like
*
up
got
not
They
I
The
about
big
may
just
car
around
do
someone
their
later
going
)
replied
did
(
want
you
wanted
such
Earl

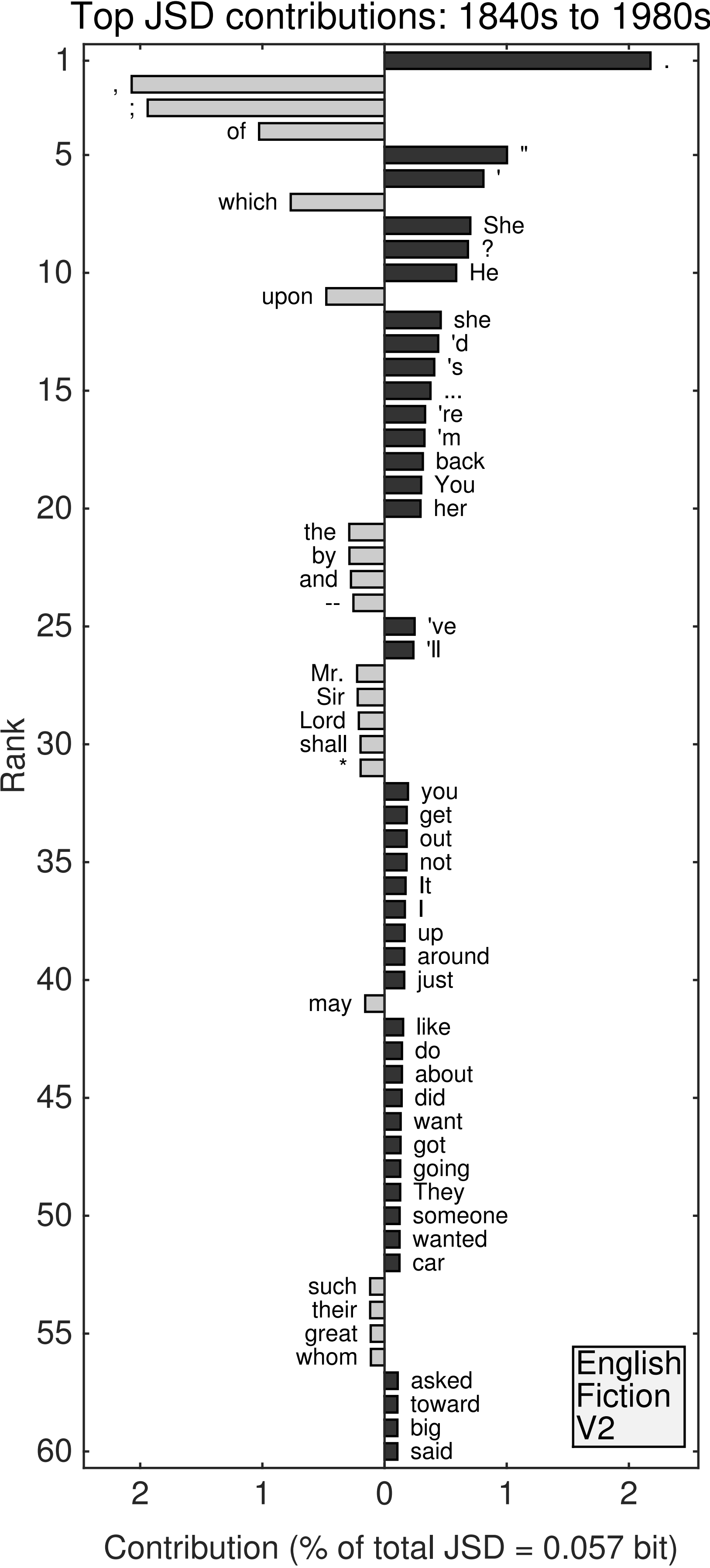

Top JSD contributions: 1840s to 1980s
Rank
Contribution (% of total JSD = 0.057 bit)
.
,
;
of
"
'
which
She
?
He
upon
she
'd
's
...
're
'm
back
You
her
the
by
and
--
've
'll
Mr.
Sir
Lord
shall
*
you
get
out
not
It
I
up
around
just
may
like
do
about
did
want
got
going
They
someone
wanted
car
such
their
great
whom
asked
toward
big
said
English Fiction V2

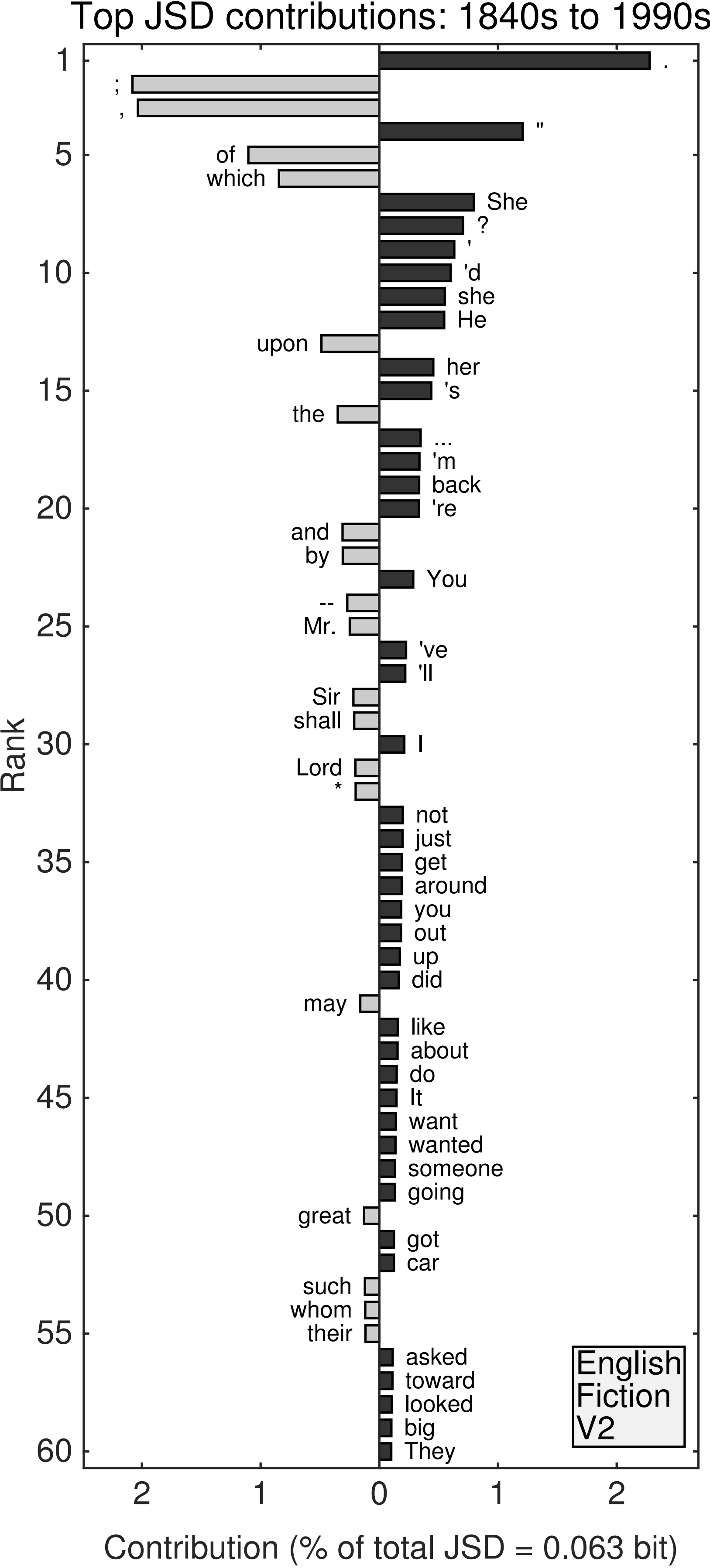

Top JSD contributions: 1840s to 1990s
Rank
Contribution (% of total JSD = 0.063 bit)
English Fiction V2
.
;
,
"
of
which
She
?
'
'd
she
He
upon
her
's
the
...
'm
back
're
and
by
You
--
Mr.
've
'll
Sir
shall
I
Lord
*
not
just
get
around
you
out
up
did
may
like
about
do
It
want
wanted
someone
going
great
got
car
such
whom
their
asked
toward
looked
big
They

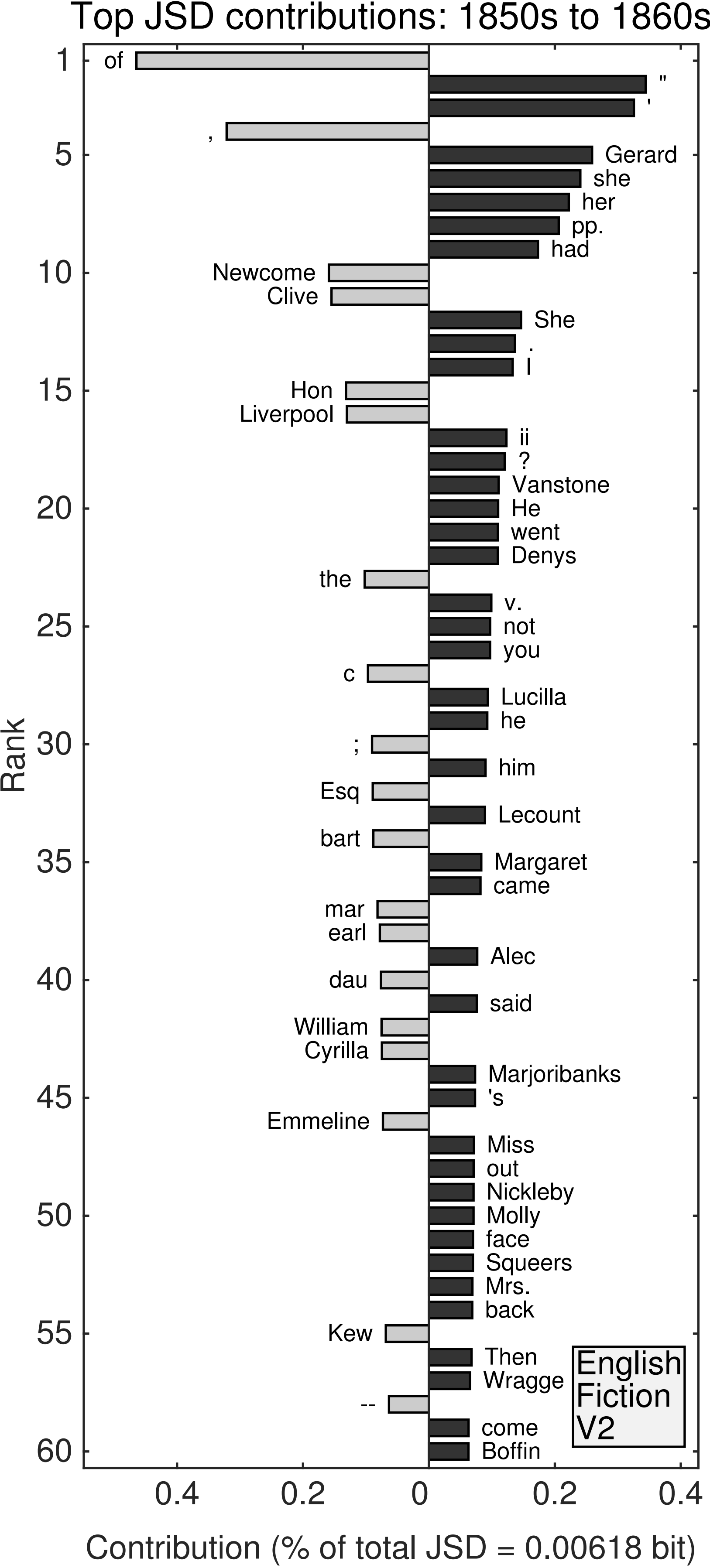
Top JSD contributions: 1850s to 1860s
Rank
Contribution (% of total JSD = 0.00618 bit)
of
"
'
,
Gerard
she
her
pp.
had
Newcome
Clive
She
.
i
Hon
Liverpool
ii
?
Vanstone
He
went
Denys
the
v.
not
you
c
Lucilla
he
;
him
Esq
Lecount
bart
Margaret
came
mar
earl
Alec
dau
said
William
Cyrilla
Marjoribanks
's
Emmeline
Miss
out
Nickleby
Molly
face
Squeers
Mrs.
back
Kew
Then
Wragge
--
come
Boffin
English Fiction V2

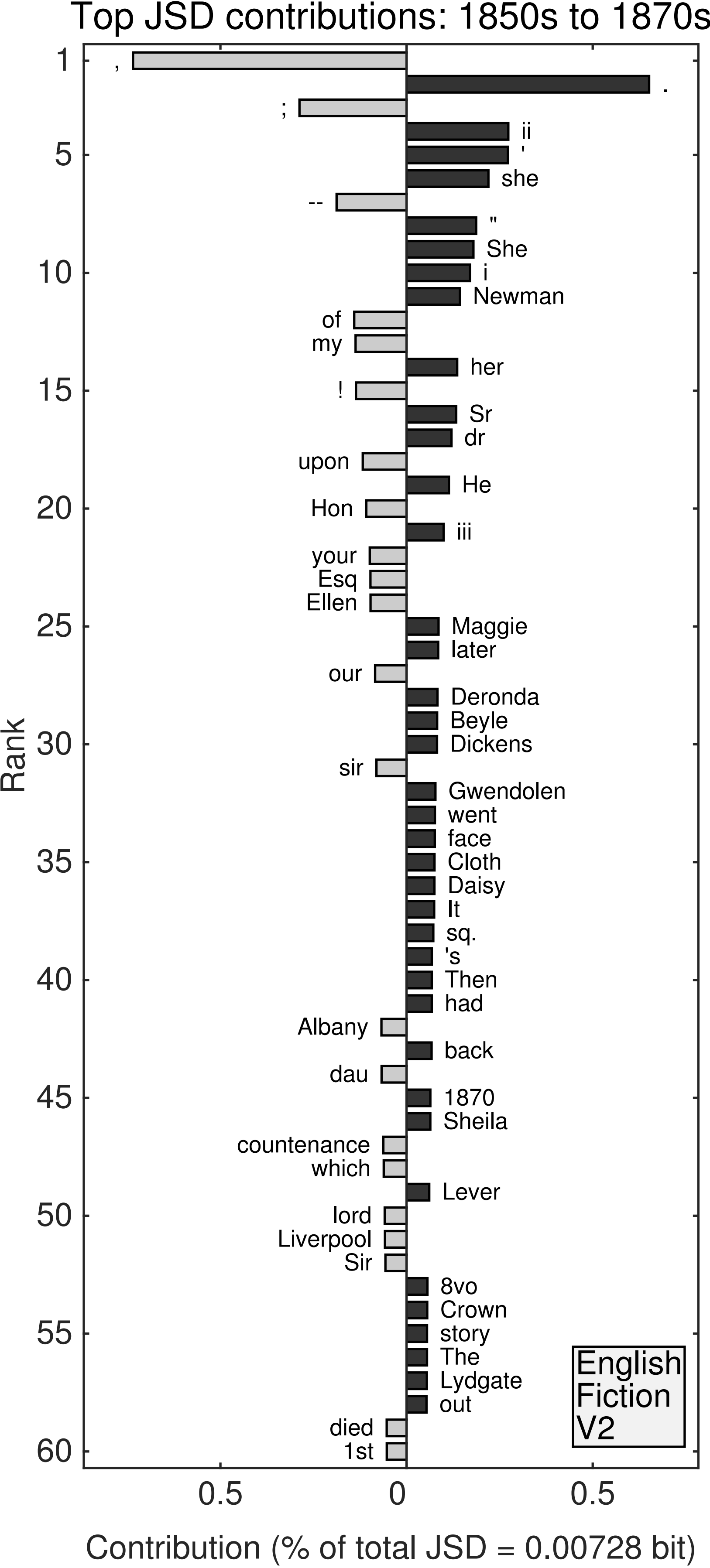
Top JSD contributions: 1850s to 1870s
Rank
Contribution (% of total JSD = 0.00728 bit)
English Fiction V2
,
.
;
ii
'
she
--
"
She
i
Newman
of
my
her
!
Sr
dr
upon
He
Hon
iii
your
Esq
Ellen
Maggie
later
our
Deronda
Beyle
Dickens
sir
Gwendolen
went
face
Cloth
Daisy
It
sq.
's
Then
had
Albany
back
dau
1870
Sheila
countenance
which
Lever
lord
Liverpool
Sir
8vo
Crown
story
The
Lydgate
out
died
1st
1
5
10
15
20
25
30
35
40
45
50
55
60
0.5
0
0.5

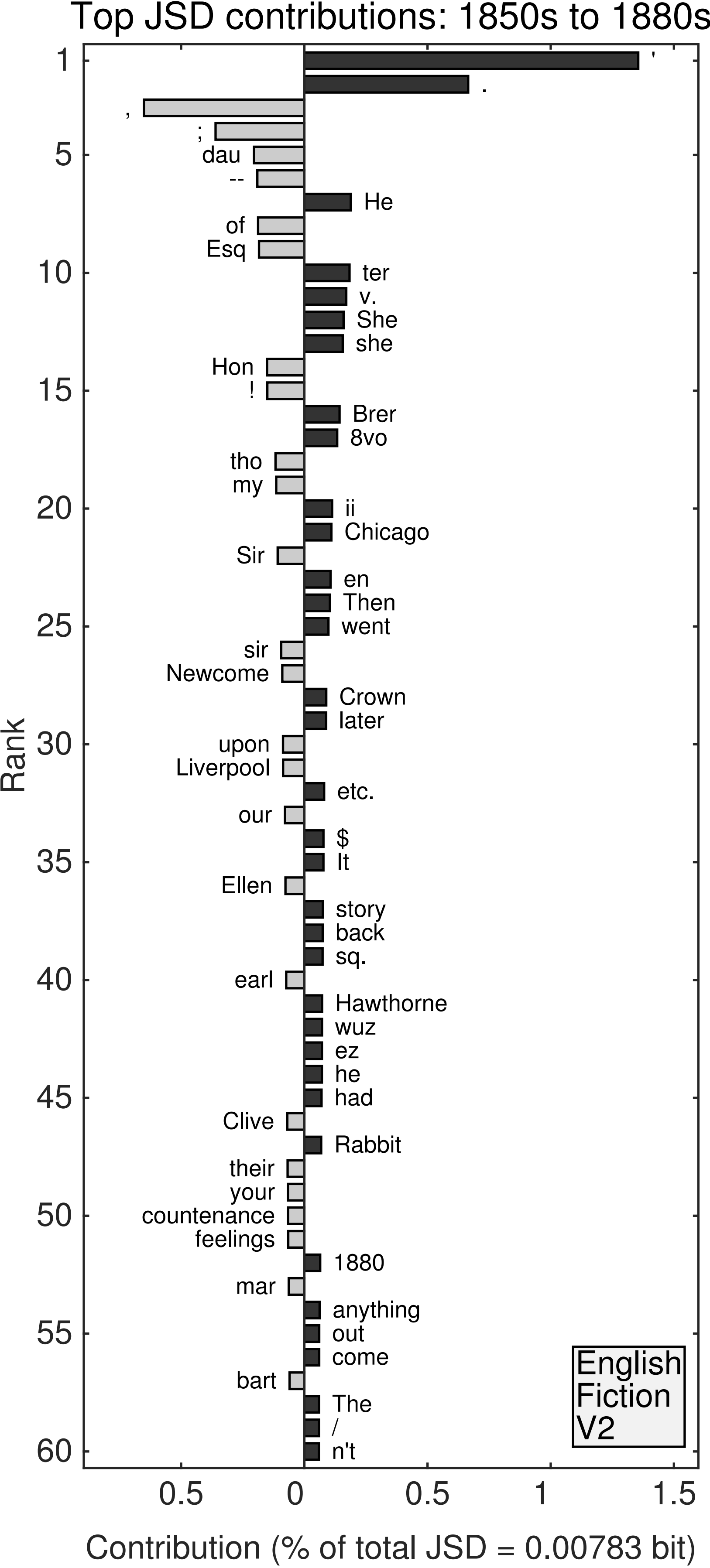

Top JSD contributions: 1850s to 1880s
Rank
Contribution (% of total JSD = 0.00783 bit)
English Fiction V2
'
.
,
;
dau
--
He
of
Esq
ter
v.
She
she
Hon
!
Brer
8vo
tho
my
ii
Chicago
Sir
en
Then
went
sir
Newcome
Crown
later
upon
Liverpool
etc.
our
$
It
Ellen
story
back
sq.
earl
Hawthorne
wuz
ez
he
had
Clive
Rabbit
their
your
countenance
feelings
1880
mar
anything
out
come
bart
The
/
n't

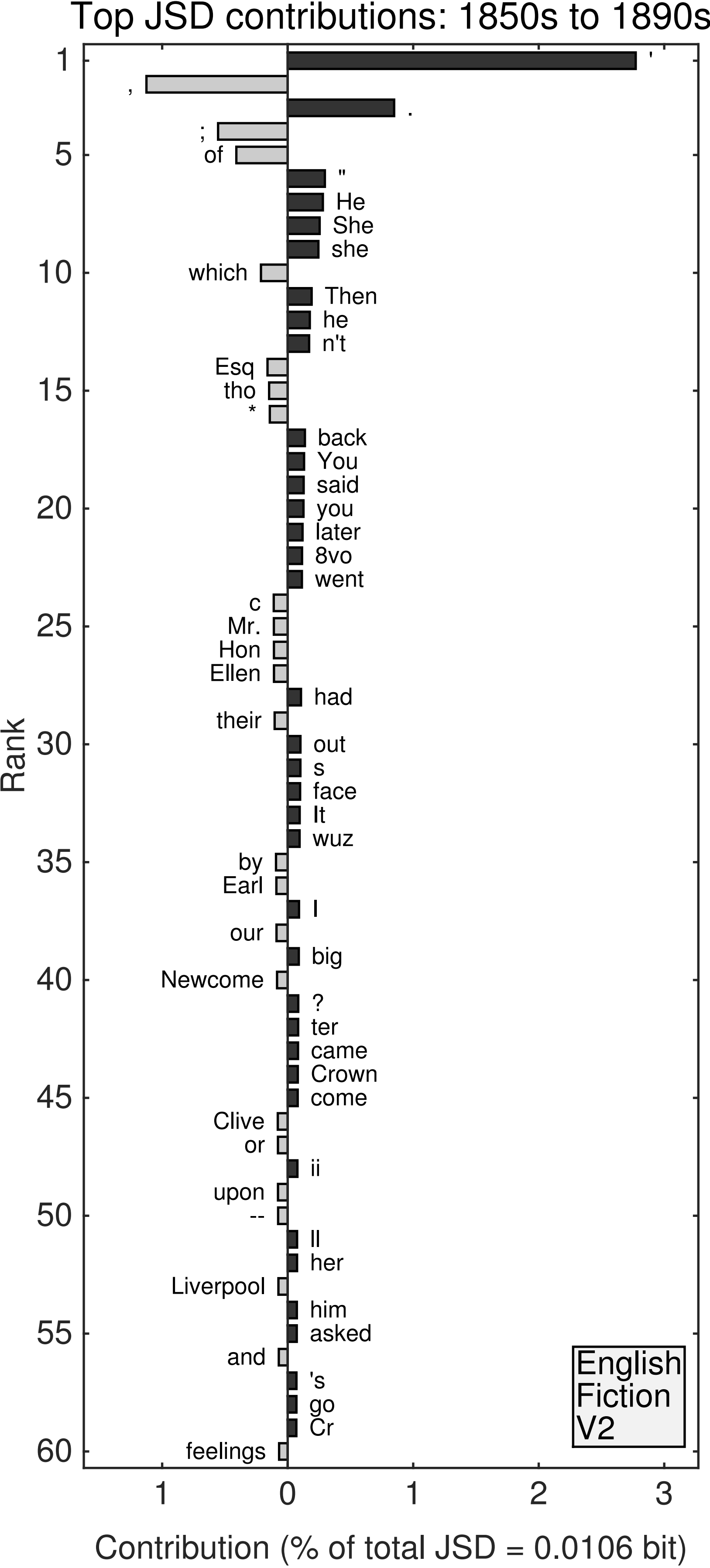

Top JSD contributions: 1850s to 1890s
Rank
Contribution (% of total JSD = 0.0106 bit)
English Fiction V2
'
,
.
;
of
"
He
She
she
which
Then
he
n't
Esq
tho
*
back
You
said
you
later
8vo
went
c
Mr.
Hon
Ellen
had
their
out
s
face
It
wuz
by
Earl
I
our
big
Newcome
?
ter
came
Crown
come
Clive
or
ii
upon
--
ll
her
Liverpool
him
asked
and
's
go
Cr
feelings

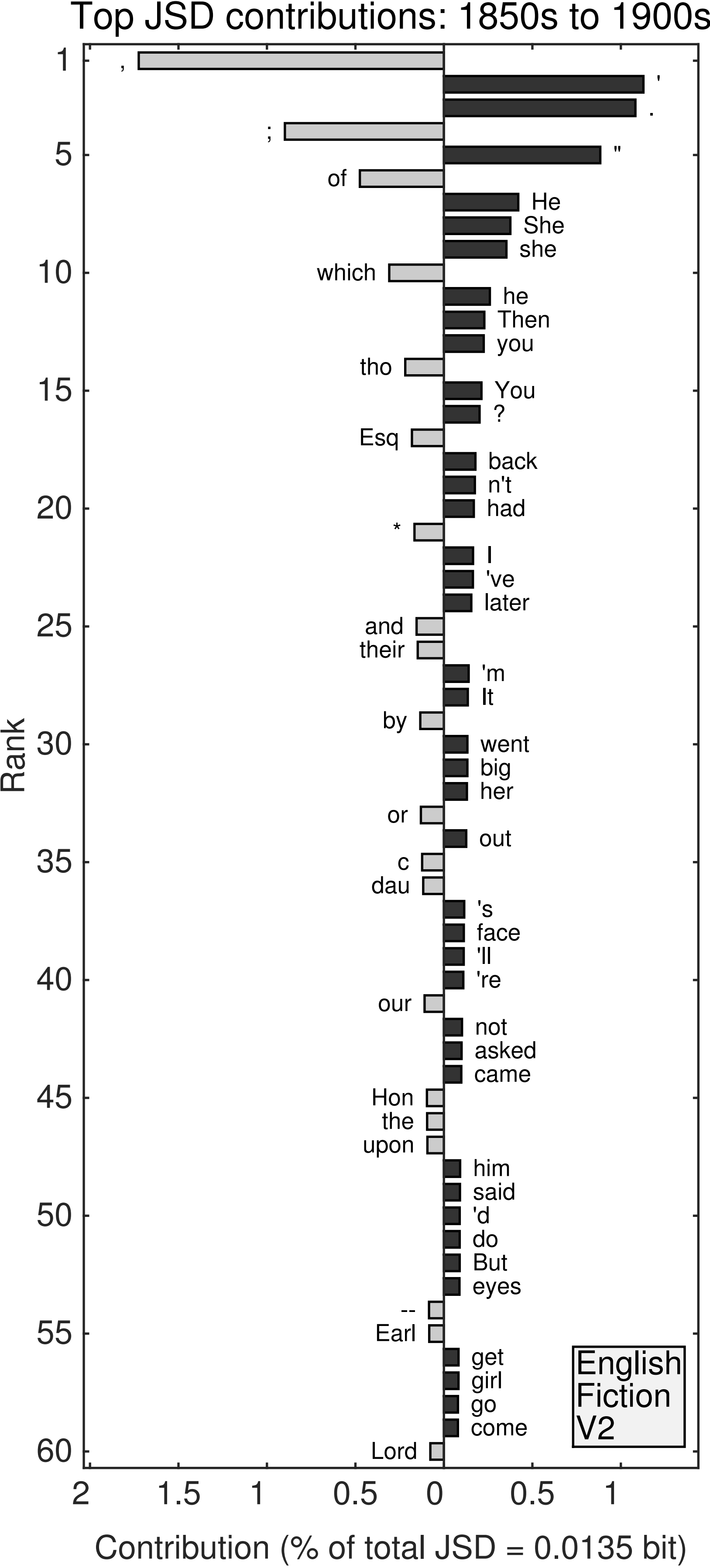

Top JSD contributions: 1850s to 1900s
Rank
Contribution (% of total JSD = 0.0135 bit)
English Fiction V2
,
'
.
;
"
of
He
She
she
which
he
Then
you
tho
You
?
Esq
back
n't
had
*
I
've
later
and
their
'm
It
by
went
big
her
or
out
c
dau
's
face
'll
're
our
not
asked
came
Hon
the
upon
him
said
'd
do
But
eyes
--
Earl
get
girl
go
come
Lord

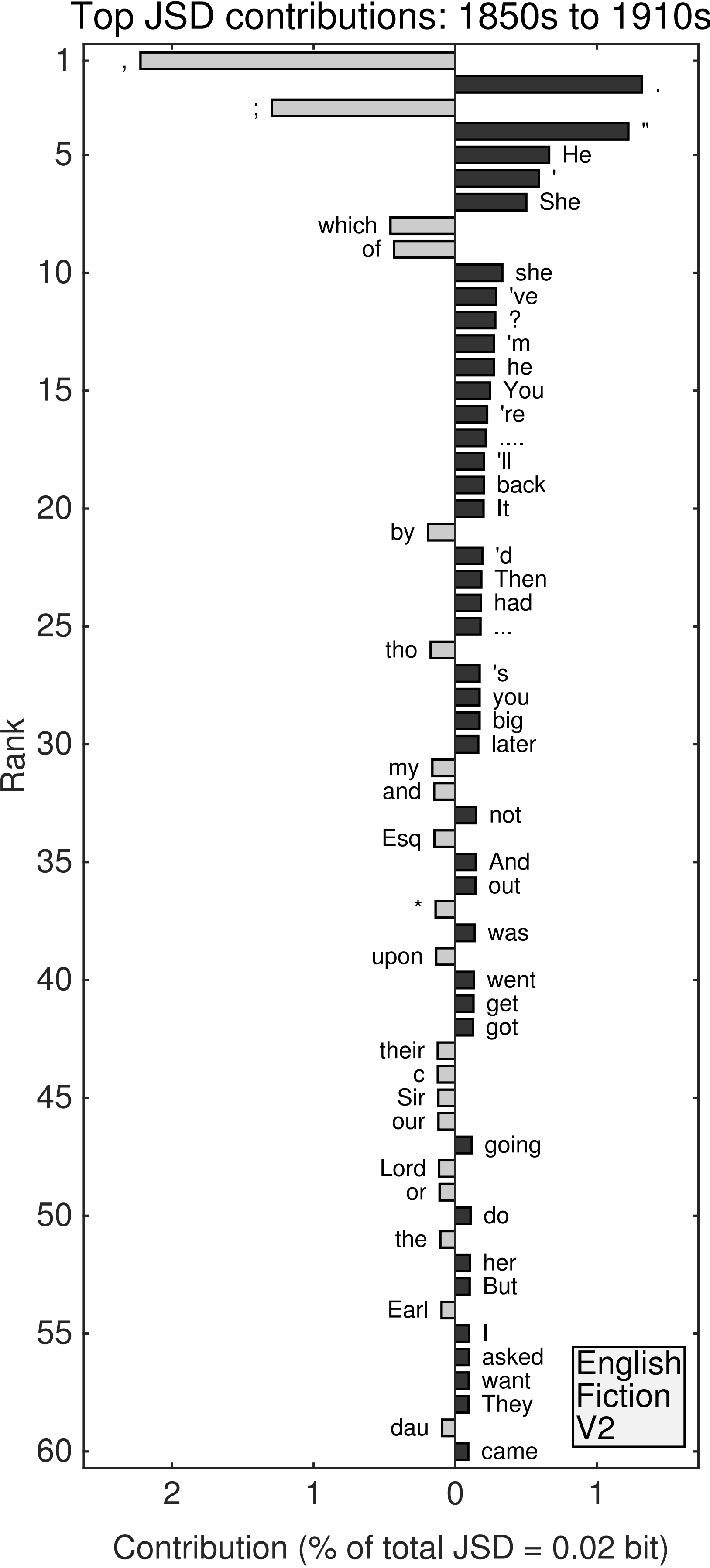
Top JSD contributions: 1850s to 1910s
Rank
Contribution (% of total JSD = 0.02 bit)
English Fiction V2
1
5
10
15
20
25
30
35
40
45
50
55
60
2
1
0
1
,
.
;
"
He
'
She
which
of
she
've
?
'm
he
You
're
....
'll
back
It
by
'd
Then
had
...
tho
's
you
big
later
my
and
not
Esq
And
out
*
was
upon
went
get
got
their
c
Sir
our
going
Lord
or
do
the
her
But
Earl
I
asked
want
They
dau
came

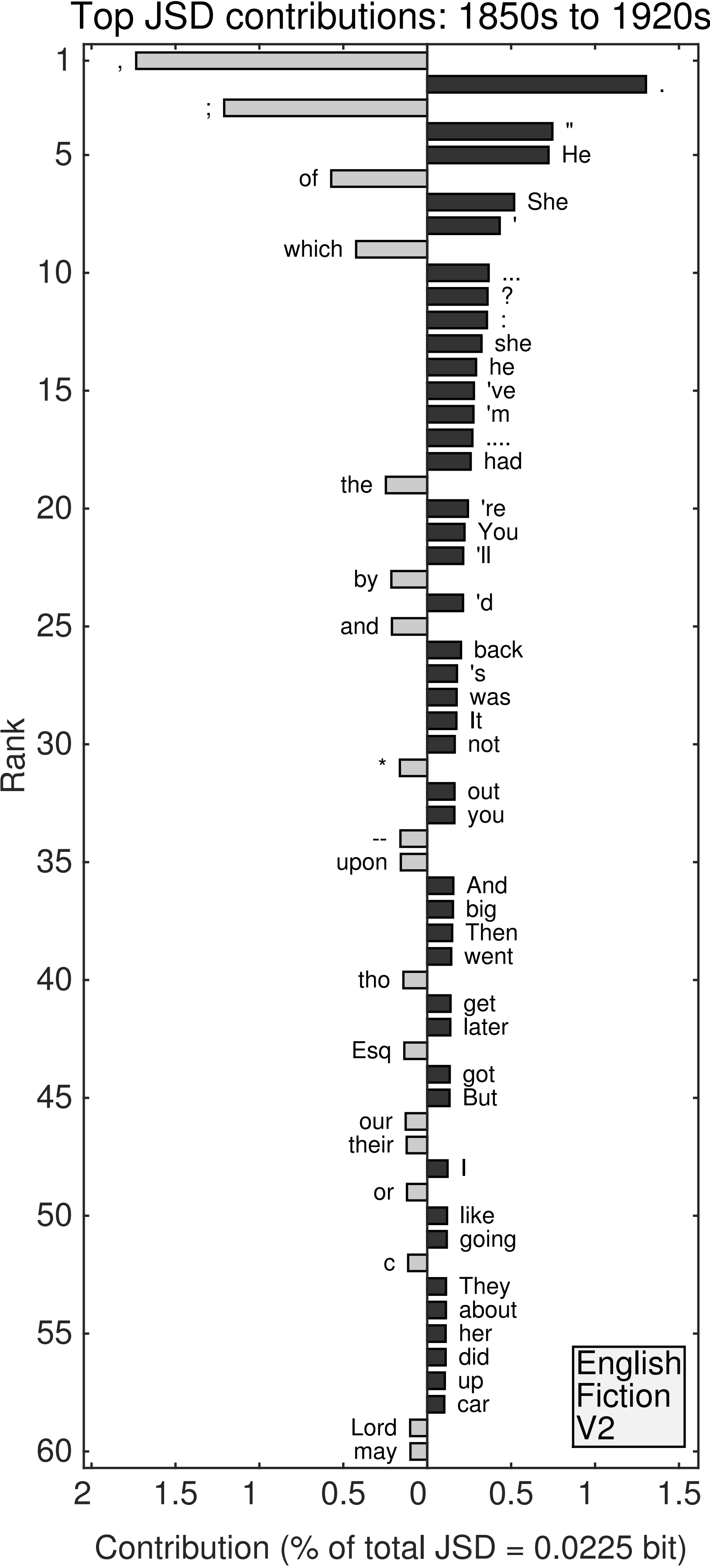
Top JSD contributions: 1850s to 1920s
Rank
Contribution (% of total JSD = 0.0225 bit)
English Fiction V2
,
.
;
"
He
of
She
'
which
...
?
:
she
he
've
'm
....
had
the
're
You
'll
by
'd
and
back
's
was
It
not
*
out
you
--
upon
And
big
Then
went
tho
get
later
Esq
got
But
our
their
I
or
like
going
c
They
about
her
did
up
car
Lord
may

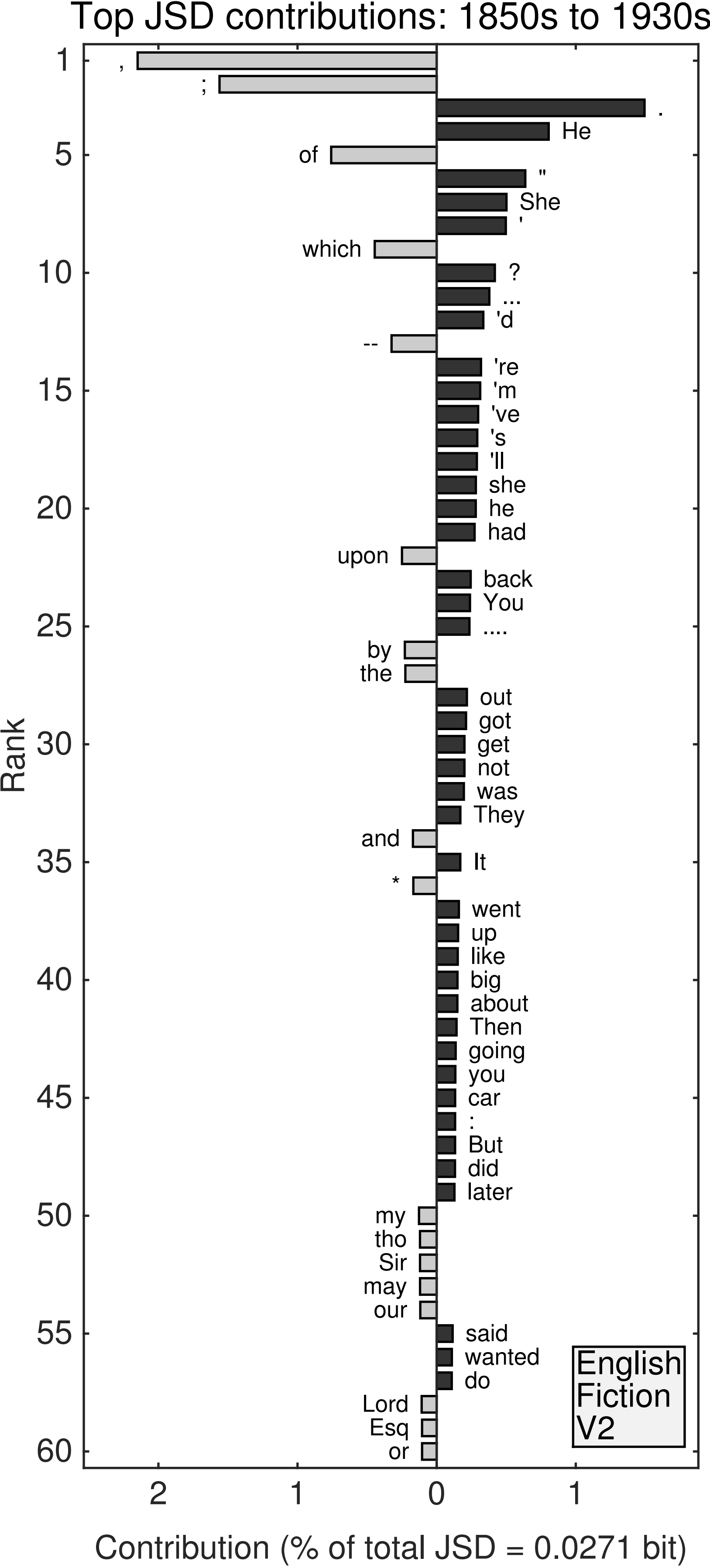

Top JSD contributions: 1850s to 1930s
Rank
Contribution (% of total JSD = 0.0271 bit)
,
;
.
He
of
"
She
'
which
?
...
'd
--
're
'm
've
's
'll
she
he
had
upon
back
You
....
by
the
out
got
get
not
was
They
and
It
*
went
up
like
big
about
Then
going
you
car
:
But
did
later
my
tho
Sir
may
our
said
wanted
do
Lord
Esq
or
English
Fiction
V2

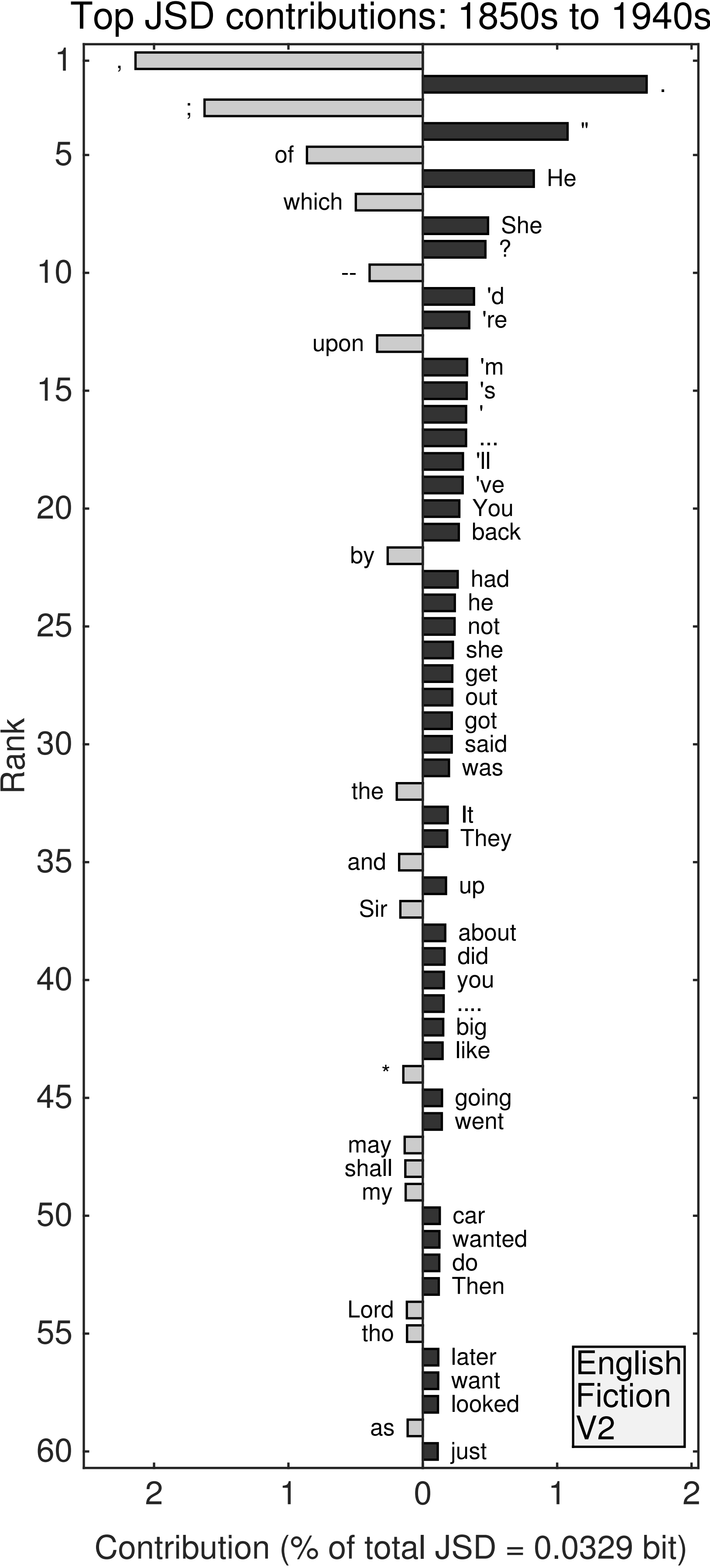
Top JSD contributions: 1850s to 1940s
Rank
Contribution (% of total JSD = 0.0329 bit)
English Fiction V2
, . ; " of He which She ? -- 'd 're upon 'm 's ' ... 'll 've You back by had he not she get out got said was the It They and up Sir about did you .... big like * going went may shall my car wanted do Then Lord tho later want looked as just

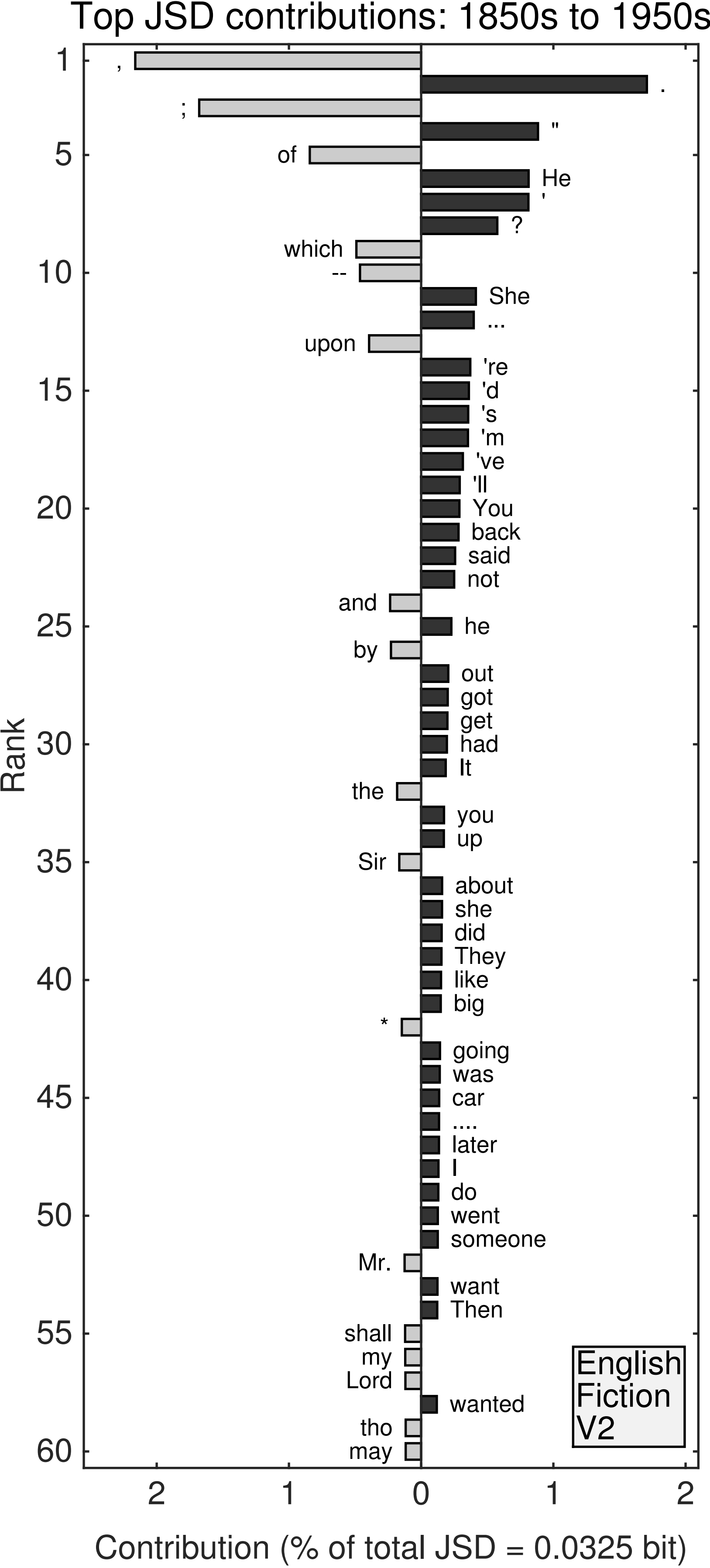

Top JSD contributions: 1850s to 1950s
Rank
Contribution (% of total JSD = 0.0325 bit)
English Fiction V2
,
.
;
"
of
He
'
?
which
--
She
...
upon
're
'd
's
'm
've
'll
You
back
said
not
and
he
by
out
got
get
had
It
the
you
up
Sir
about
she
did
They
like
big
*
going
was
car
....
later
I
do
went
someone
Mr.
want
Then
shall
my
Lord
wanted
tho
may

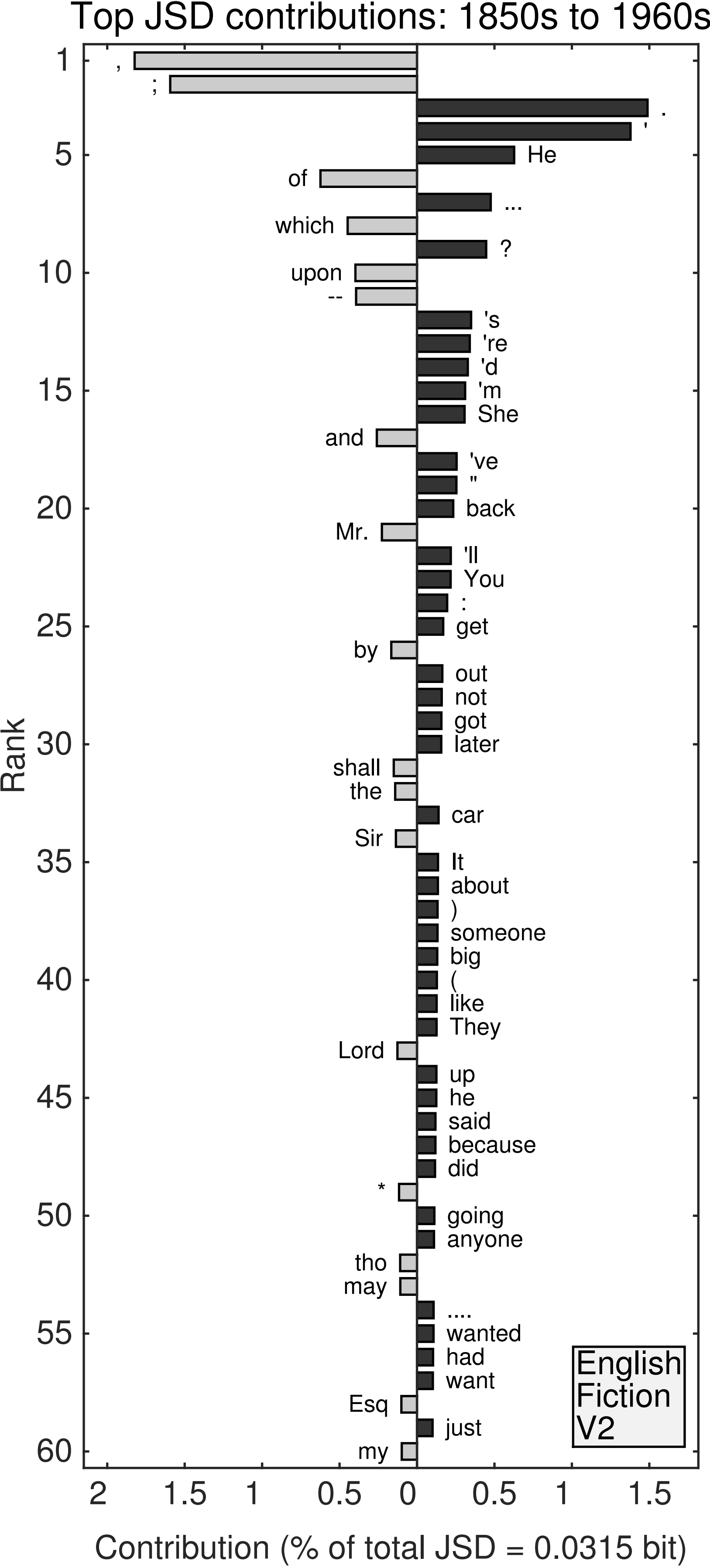
Top JSD contributions: 1850s to 1960s
Rank
Contribution (% of total JSD = 0.0315 bit)
English Fiction V2
,
;
.
'
He
of
...
which
?
upon
--
's
're
'd
'm
She
and
've
"
back
Mr.
'll
You
:
get
by
out
not
got
later
shall
the
car
Sir
It
about
)
someone
big
(
like
They
Lord
up
he
said
because
did
*
going
anyone
tho
may
....
wanted
had
want
Esq
just
my

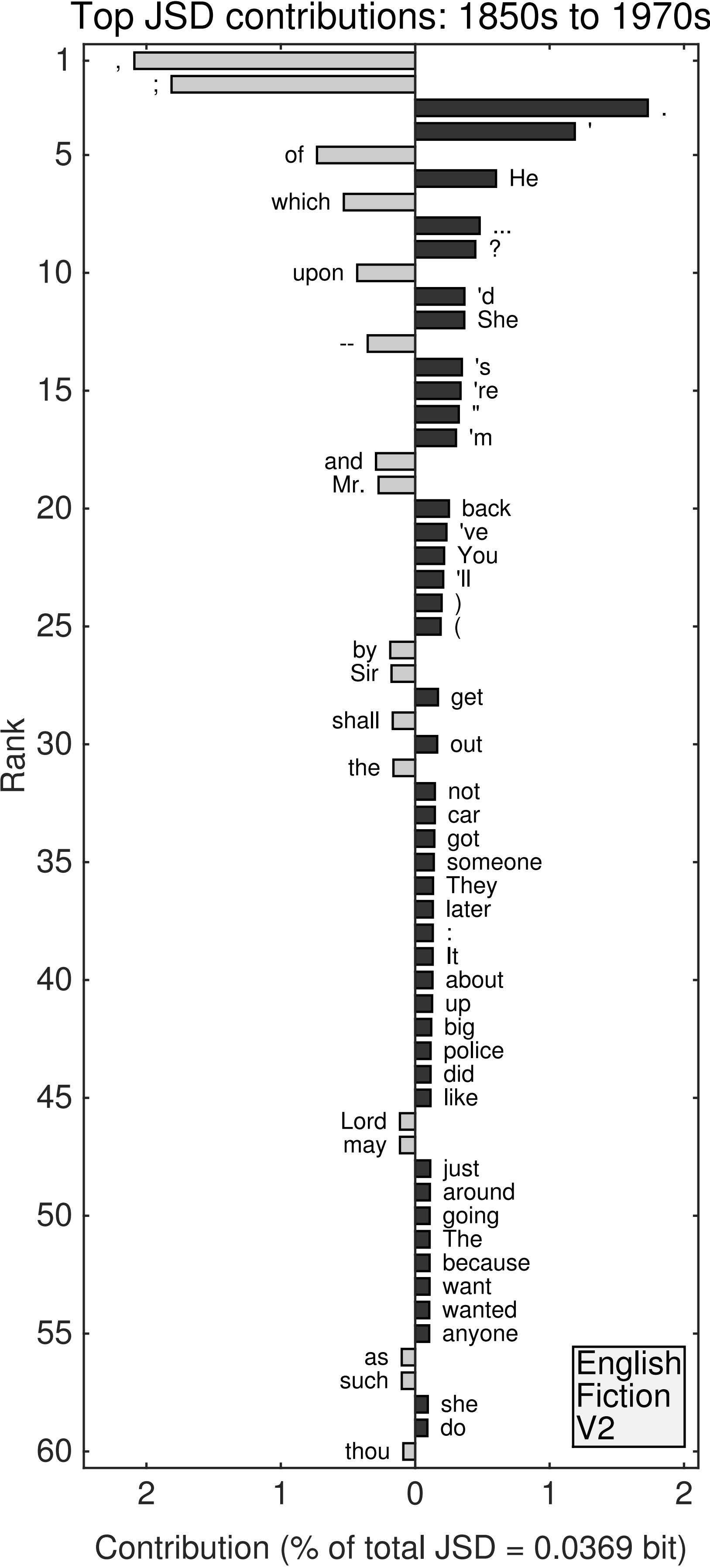
Top JSD contributions: 1850s to 1970s
Rank
Contribution (% of total JSD = 0.0369 bit)
English Fiction V2
,
;
.
'
of
He
which
...
?
upon
'd
She
--
's
're
"
'm
and
Mr.
back
've
You
'll
)
(
by
Sir
get
shall
out
the
not
car
got
someone
They
later
:
It
about
up
big
police
did
like
Lord
may
just
around
going
The
because
want
wanted
anyone
as
such
she
do
thou

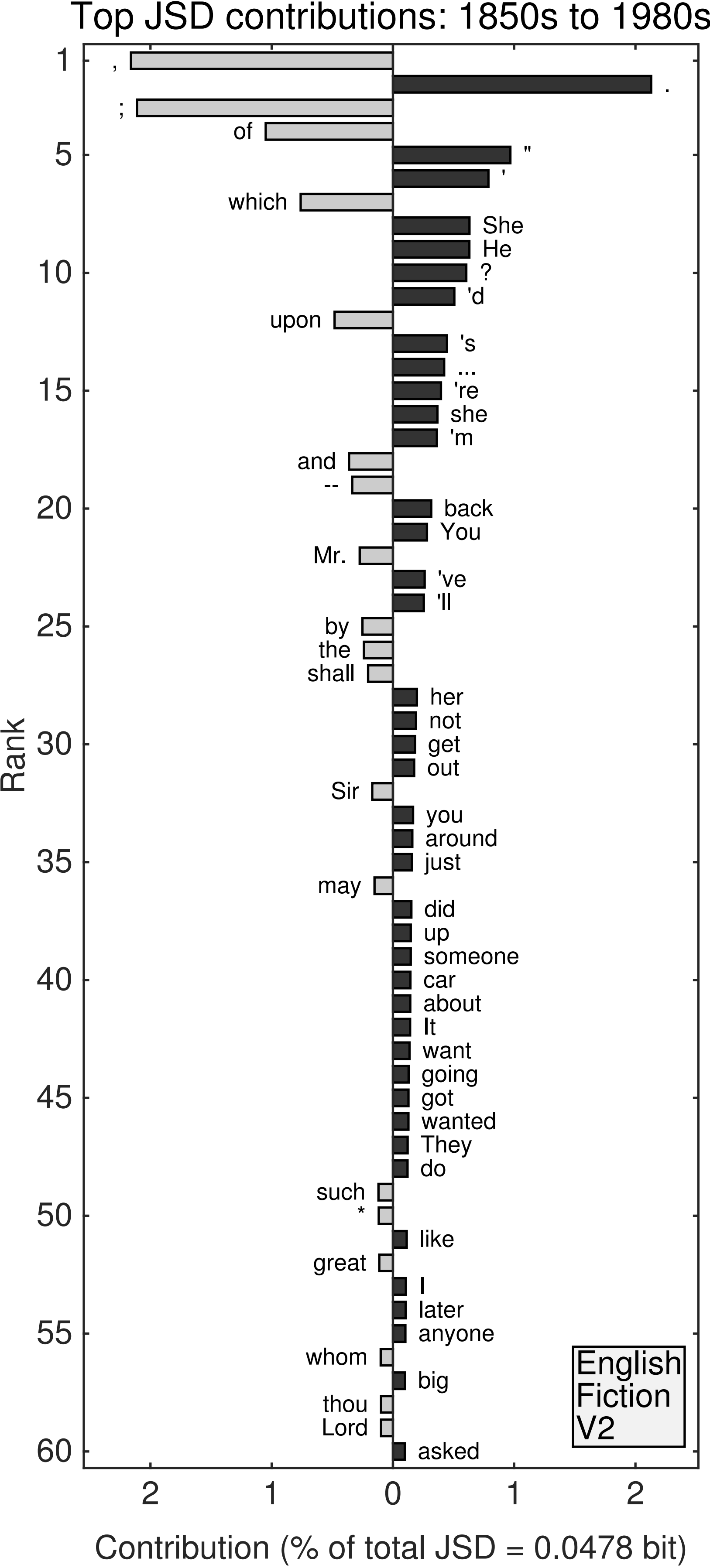

Top JSD contributions: 1850s to 1980s
Rank
Contribution (% of total JSD = 0.0478 bit)
English Fiction V2
,
.
;
of
"
'
which
She
He
?
'd
upon
's
...
're
she
'm
and
--
back
You
Mr.
've
'll
by
the
shall
her
not
get
out
Sir
you
around
just
may
did
up
someone
car
about
It
want
going
got
wanted
They
do
such
*
like
great
I
later
anyone
whom
big
thou
Lord
asked

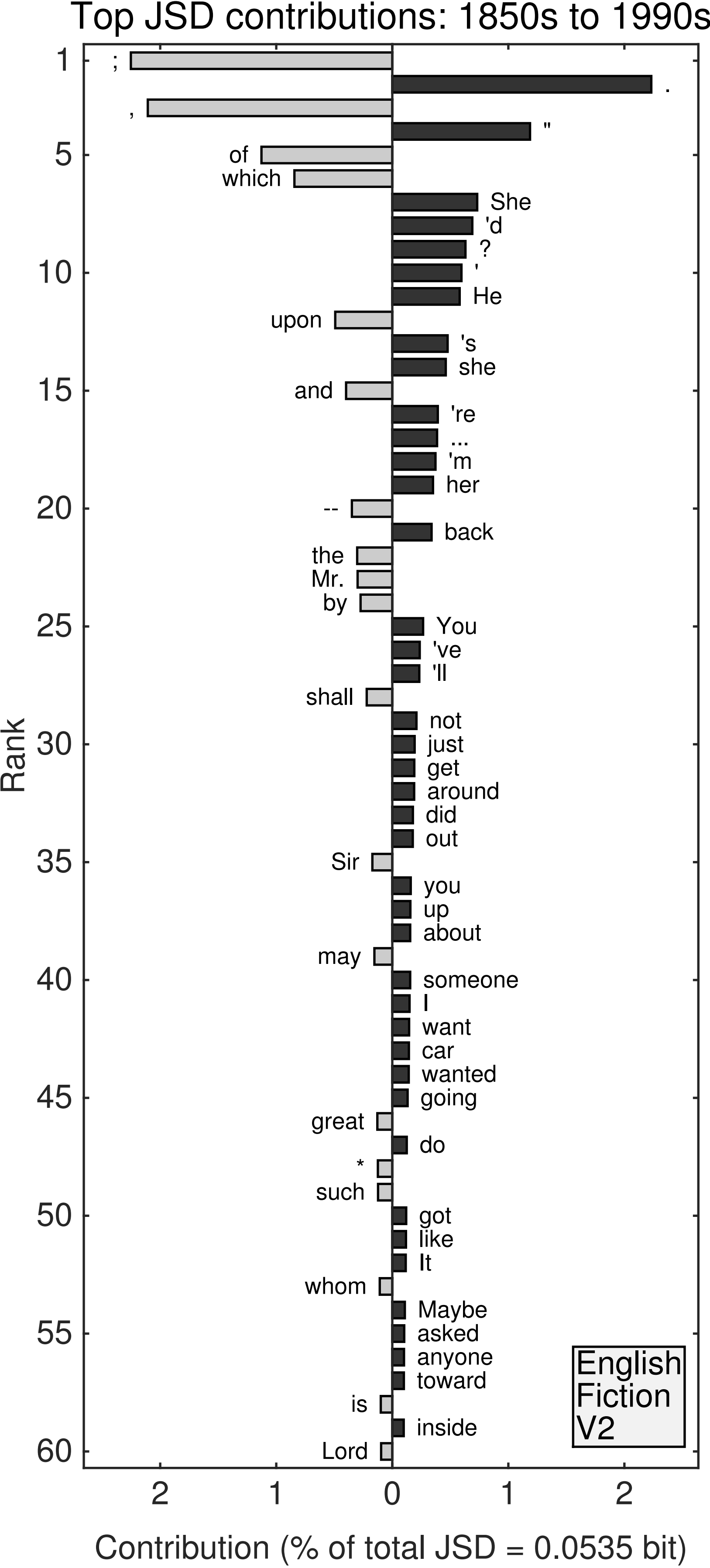

Top JSD contributions: 1850s to 1990s
Rank
1
5
10
15
20
25
30
35
40
45
50
55
60
;
.
,
"
of
which
She
'd
?
'
He
upon
's
she
and
're
...
'm
her
--
back
the
Mr.
by
You
've
'll
shall
not
just
get
around
did
out
Sir
you
up
about
may
someone
I
want
car
wanted
going
great
do
*
such
got
like
It
whom
Maybe
asked
anyone
toward
is
inside
Lord
English
Fiction
V2
2
1
0
1
2
Contribution (% of total JSD = 0.0535 bit)

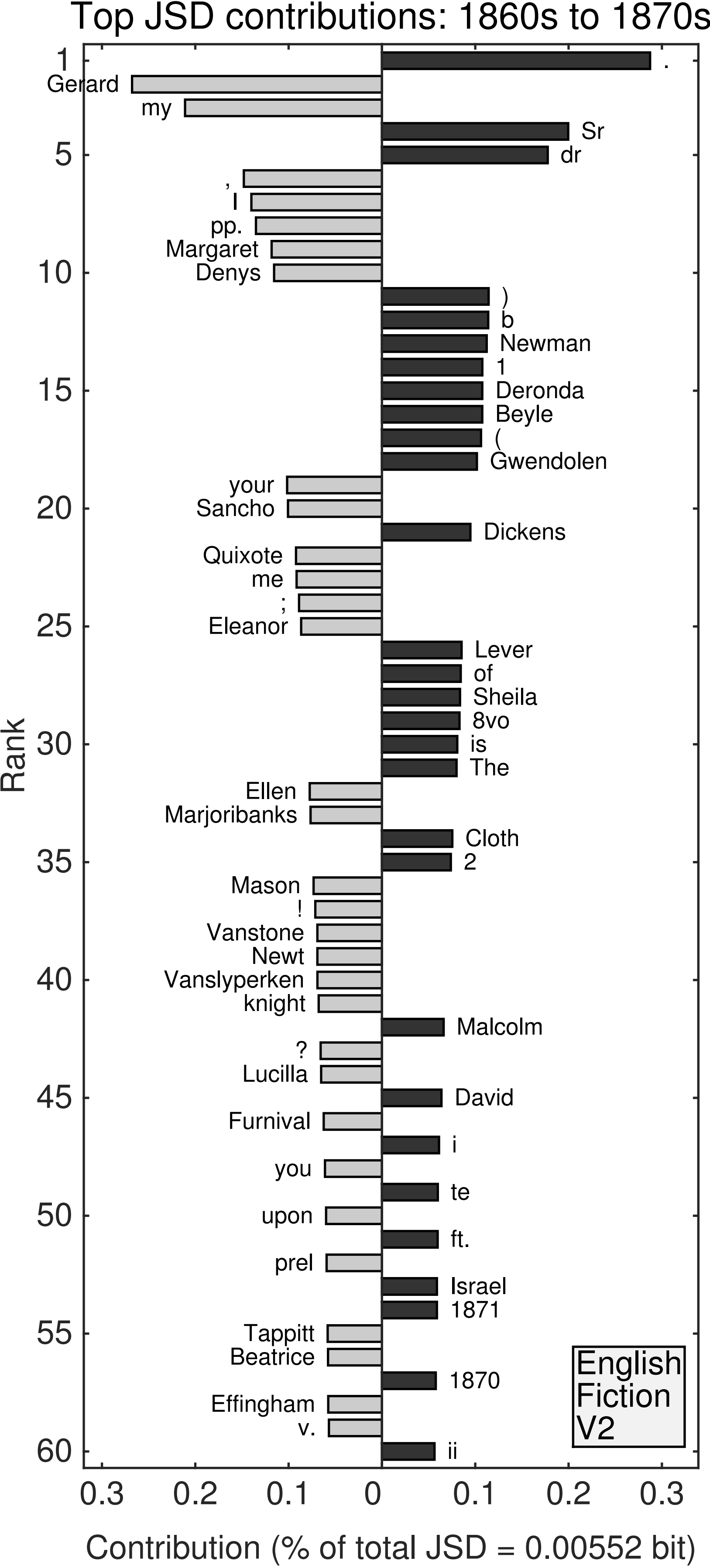
Top JSD contributions: 1860s to 1870s
Rank
Contribution (% of total JSD = 0.00552 bit)
English Fiction V2
.
Gerard
my
Sr
dr
,
I
pp.
Margaret
Denys
)
b
Newman
1
Deronda
Beyle
(
Gwendolen
your
Sancho
Dickens
Quixote
me
;
Eleanor
Lever
of
Sheila
8vo
is
The
Ellen
Marjoribanks
Cloth
2
Mason
!
Vanstone
Newt
Vanslyperken
knight
Malcolm
?
Lucilla
David
Furnival
i
you
te
upon
ft.
prel
Israel
1871
Tappitt
Beatrice
1870
Effingham
v.
ii
1
5
10
15
20
25
30
35
40
45
50
55
60
0.3
0.2
0.1
0
0.1
0.2
0.3

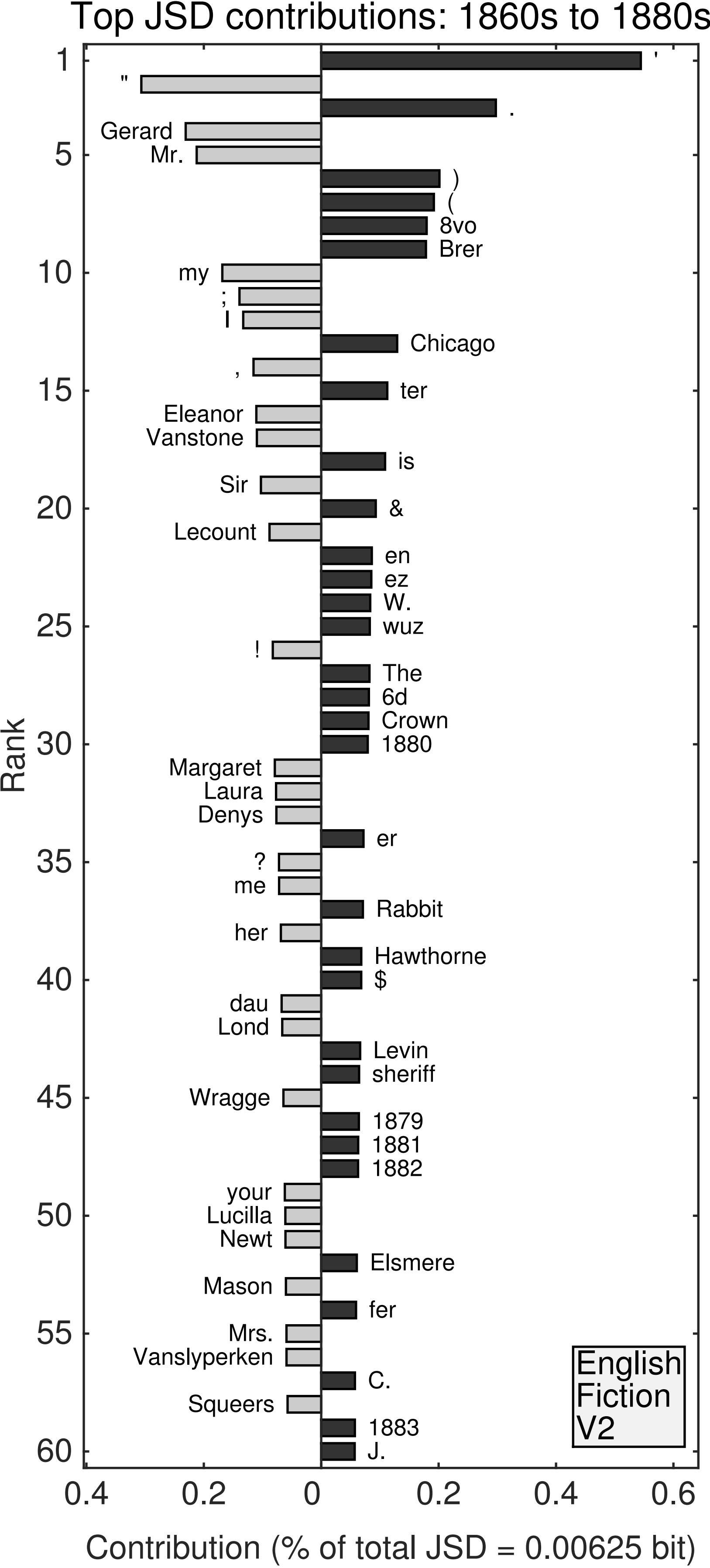
Top JSD contributions: 1860s to 1880s
Rank
Contribution (% of total JSD = 0.00625 bit)
English Fiction V2
'
"
.
Gerard
Mr.
)
(
8vo
Brer
my
;
I
Chicago
,
ter
Eleanor
Vanstone
is
Sir
&
Lecount
en
ez
W.
wuz
!
The
6d
Crown
1880
Margaret
Laura
Denys
er
?
me
Rabbit
her
Hawthorne
$
dau
Lond
Levin
sheriff
Wragge
1879
1881
1882
your
Lucilla
Newt
Elsmere
Mason
fer
Mrs.
Vanslyperken
C.
Squeers
1883
J.

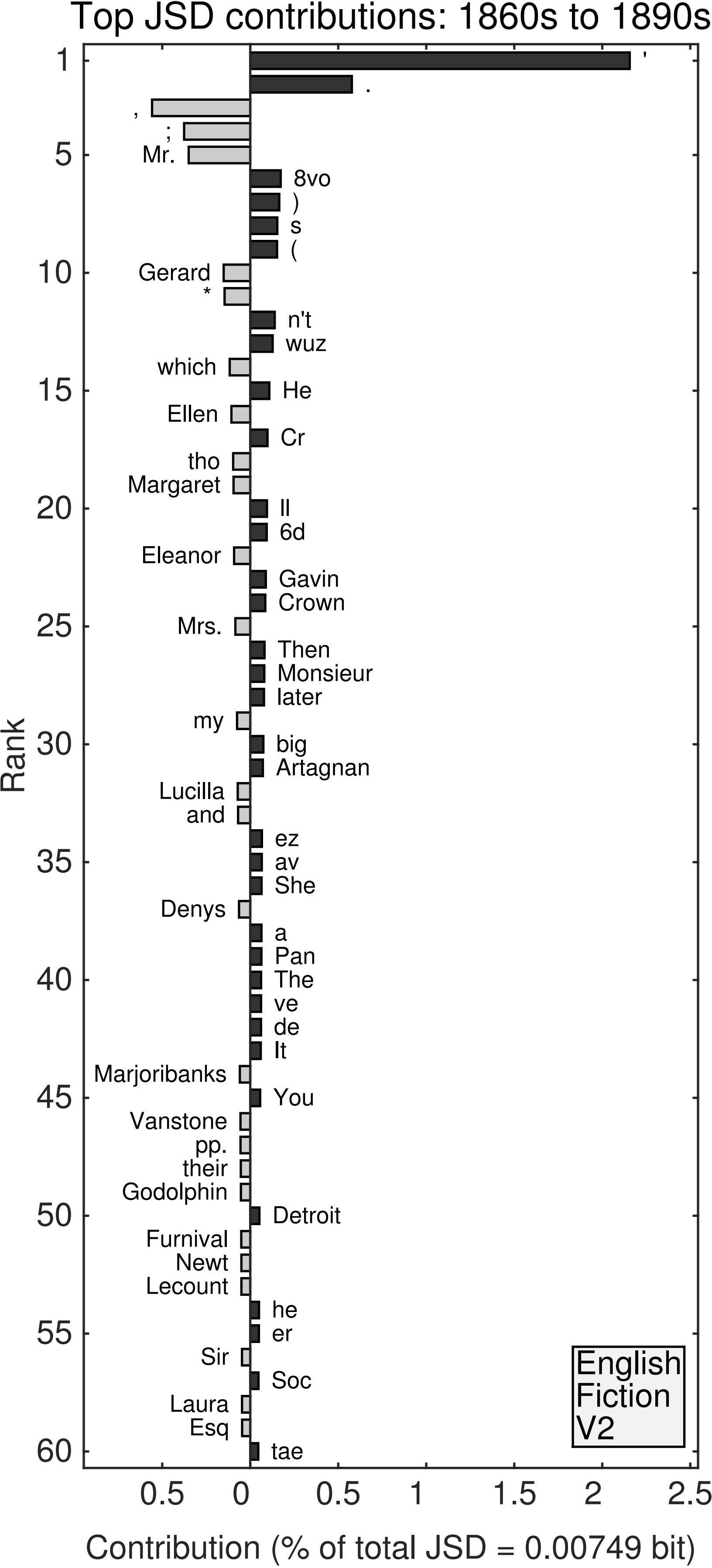

Top JSD contributions: 1860s to 1890s
Rank
Contribution (% of total JSD = 0.00749 bit)
English Fiction V2
'
.
,
;
Mr.
8vo
)
s
(
Gerard
*
n't
wuz
which
He
Ellen
Cr
tho
Margaret
ll
6d
Eleanor
Gavin
Crown
Mrs.
Then
Monsieur
later
my
big
Artagnan
Lucilla
and
ez
av
She
Denys
a
Pan
The
ve
de
It
Marjoribanks
You
Vanstone
pp.
their
Godolphin
Detroit
Furnival
Newt
Lecount
he
er
Sir
Soc
Laura
Esq
tae

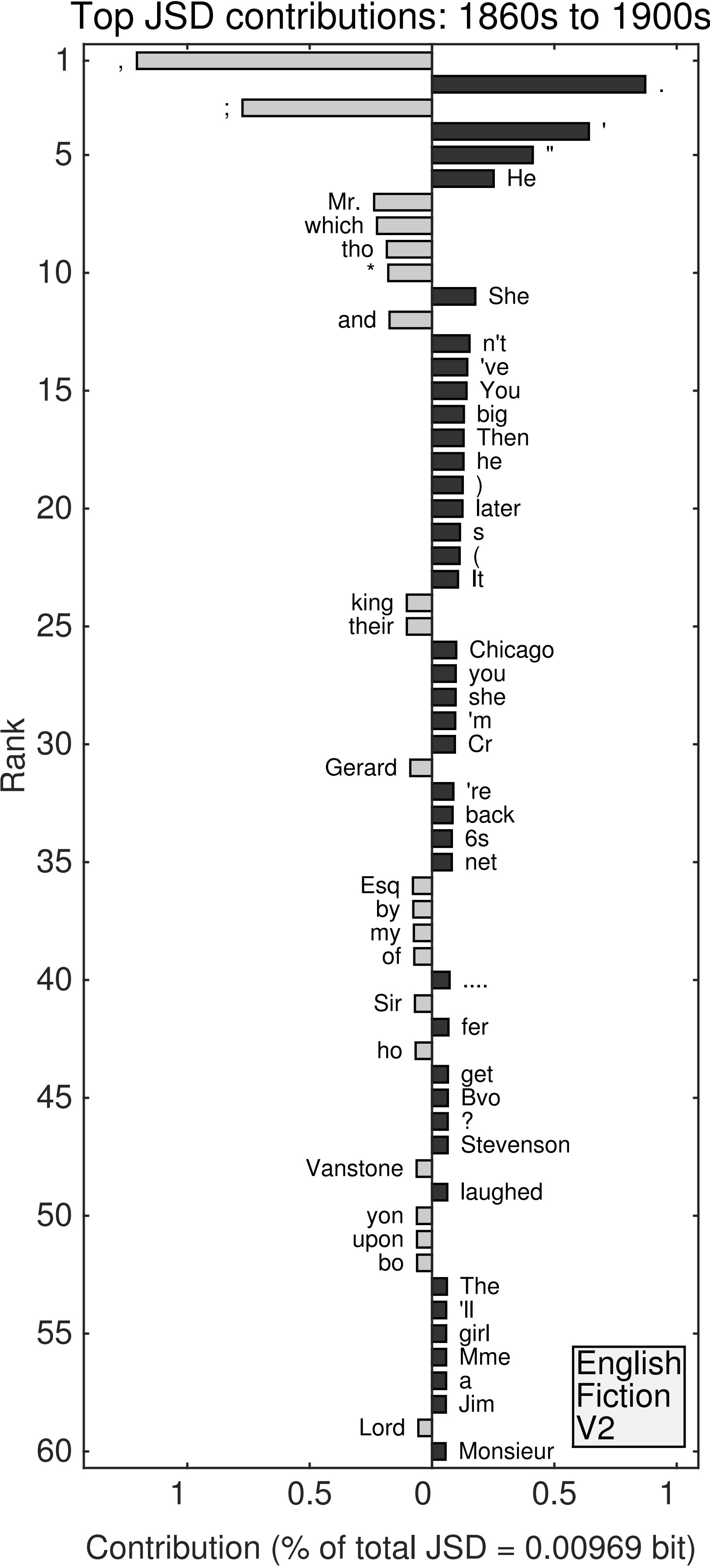
Top JSD contributions: 1860s to 1900s
Rank
Contribution (% of total JSD = 0.00969 bit)
1
0.5
0
0.5
1
1
5
10
15
20
25
30
35
40
45
50
55
60
,
.
;
'
"
He
Mr.
which
tho
*
She
and
n't
've
You
big
Then
he
)
later
s
(
It
king
their
Chicago
you
she
'm
Cr
Gerard
're
back
6s
net
Esq
by
my
of
....
Sir
fer
ho
get
Bvo
?
Stevenson
Vanstone
laughed
yon
upon
bo
The
'll
girl
Mme
a
Jim
Lord
Monsieur
English
Fiction
V2

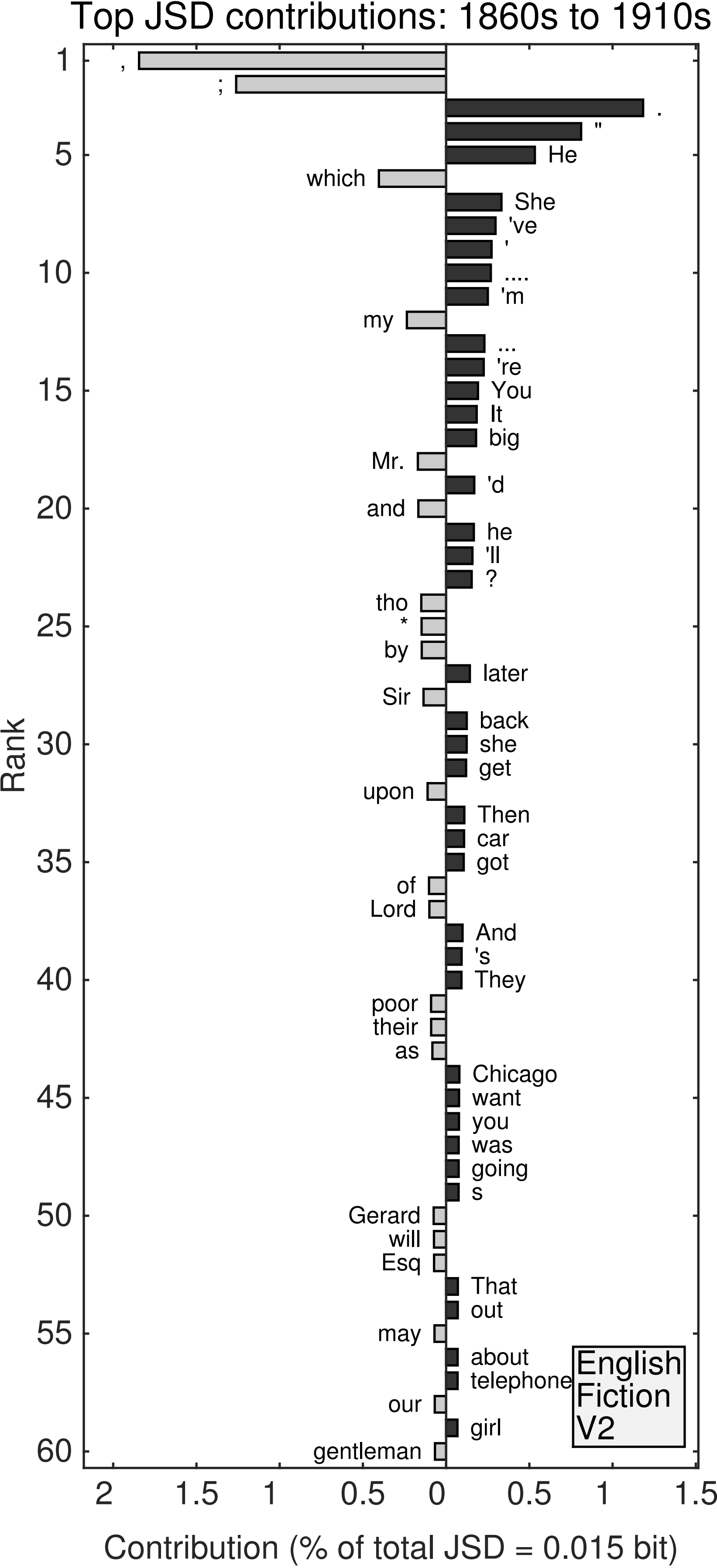

Top JSD contributions: 1860s to 1910s
Rank
Contribution (% of total JSD = 0.015 bit)
English Fiction V2
,
;
.
"
He
which
She
've
'
....
'm
my
...
're
You
It
big
Mr.
'd
and
he
'll
?
tho
*
by
later
Sir
back
she
get
upon
Then
car
got
of
Lord
And
's
They
poor
their
as
Chicago
want
you
was
going
s
Gerard
will
Esq
That
out
may
about
telephone
our
girl
gentleman

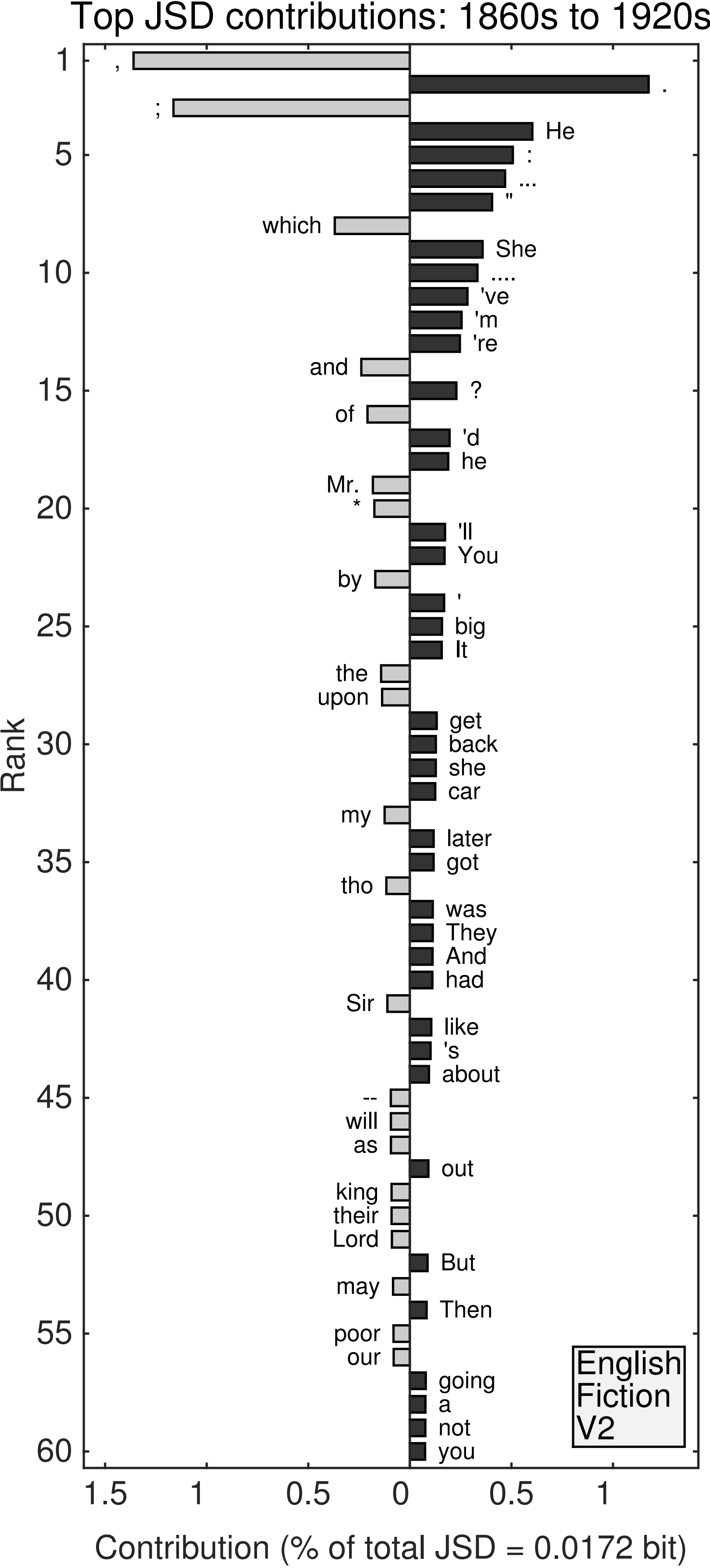

Top JSD contributions: 1860s to 1920s
Rank
Contribution (% of total JSD = 0.0172 bit)
English Fiction V2
1.5
1
0.5
0
0.5
1
1
5
10
15
20
25
30
35
40
45
50
55
60
,
.
;
He
:
...
"
which
She
....
've
'm
're
and
?
of
'd
he
Mr.
*
'll
You
by
'
big
It
the
upon
get
back
she
car
my
later
got
tho
was
They
And
had
Sir
like
's
about
--
will
as
out
king
their
Lord
But
may
Then
poor
our
going
a
not
you

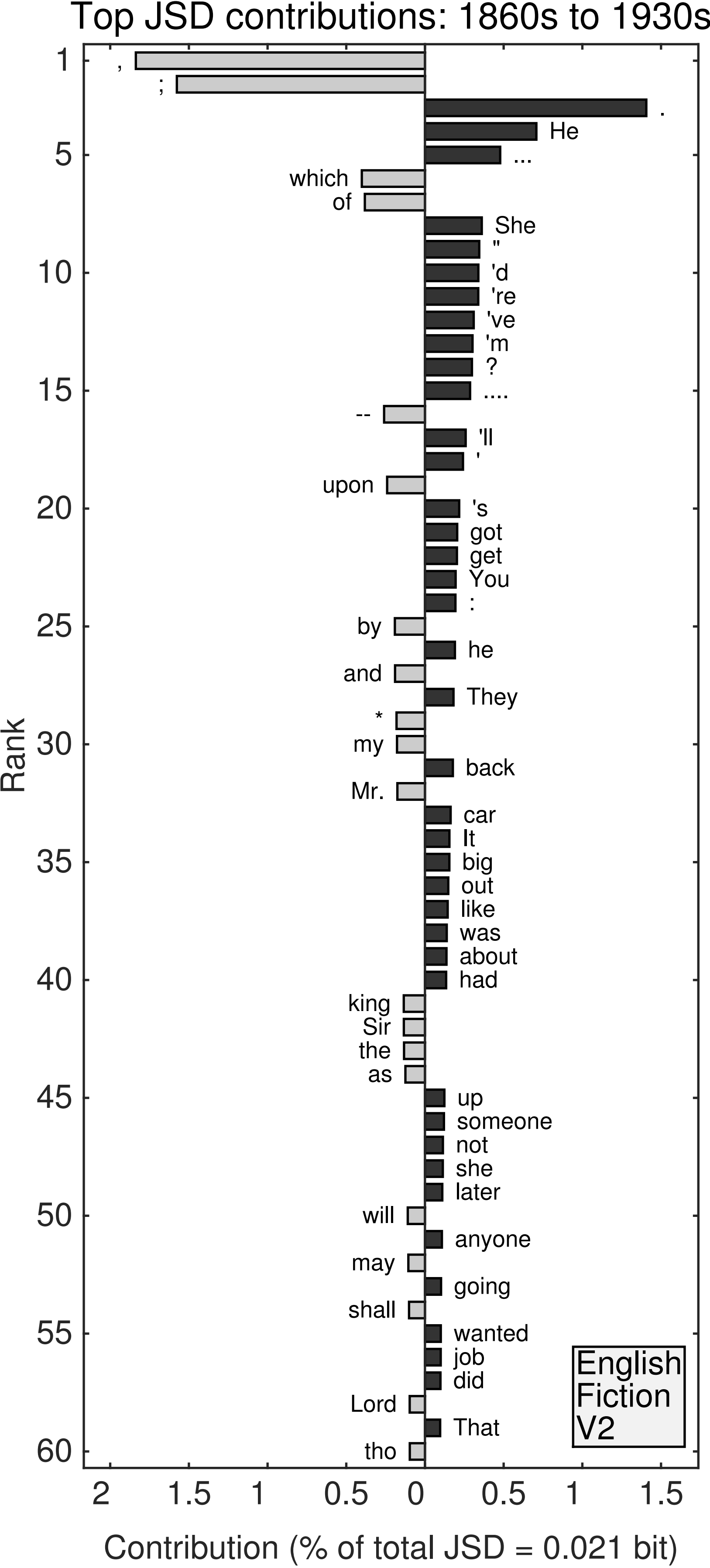

Top JSD contributions: 1860s to 1930s
Rank
Contribution (% of total JSD = 0.021 bit)
English Fiction V2
,
;
.
He
...
which
of
She
"
'd
're
've
'm
?
....
--
'll
'
upon
's
got
get
You
:
by
he
and
They
*
my
back
Mr.
car
It
big
out
like
was
about
had
king
Sir
the
as
up
someone
not
she
later
will
anyone
may
going
shall
wanted
job
did
Lord
That
tho

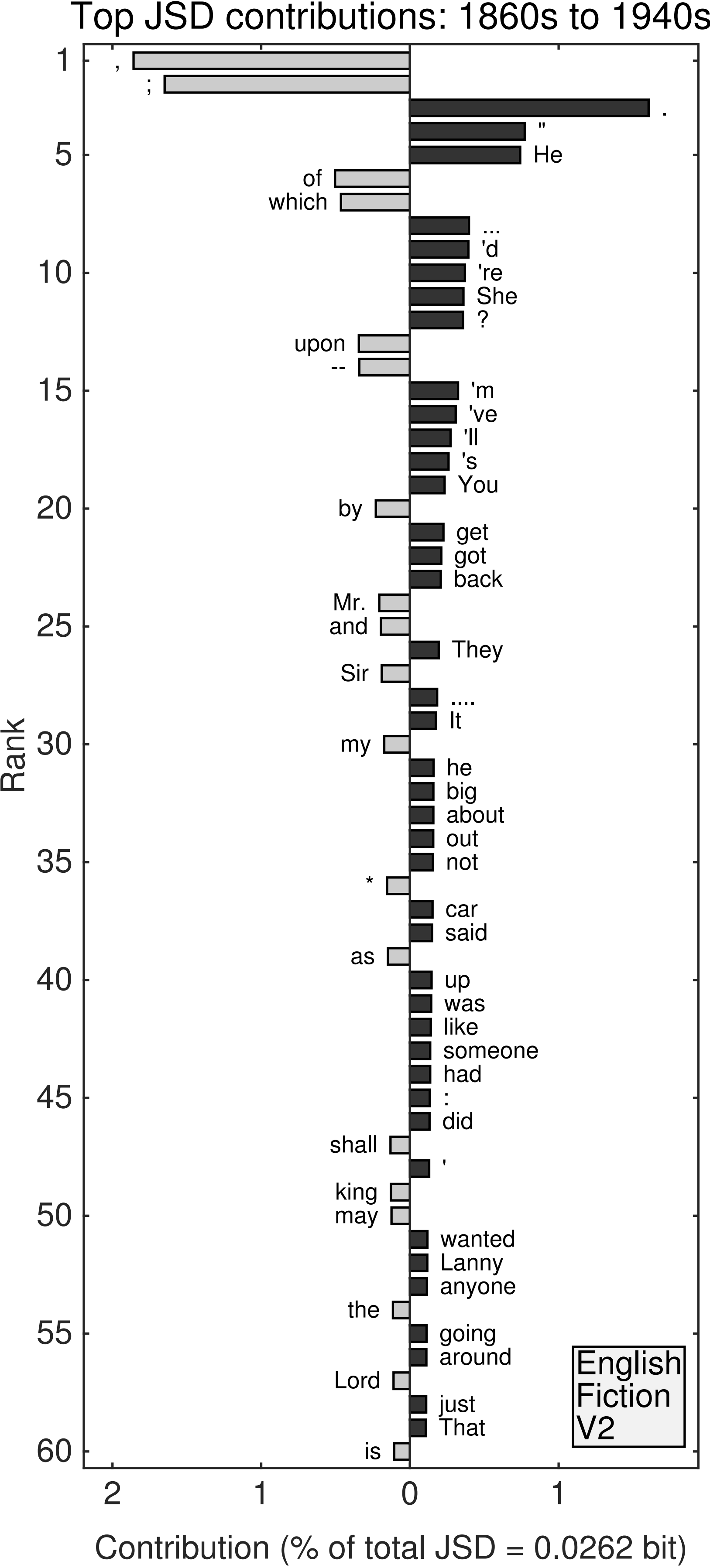
Top JSD contributions: 1860s to 1940s
Rank
Contribution (% of total JSD = 0.0262 bit)
English Fiction V2
1
5
10
15
20
25
30
35
40
45
50
55
60
2
1
0
1
,
;
.
"
He
of
which
...
'd
're
She
?
upon
--
'm
've
'll
's
You
by
get
got
back
Mr.
and
They
Sir
....
It
my
he
big
about
out
not
*
car
said
as
up
was
like
someone
had
:
did
shall
'
king
may
wanted
Lanny
anyone
the
going
around
Lord
just
That
is

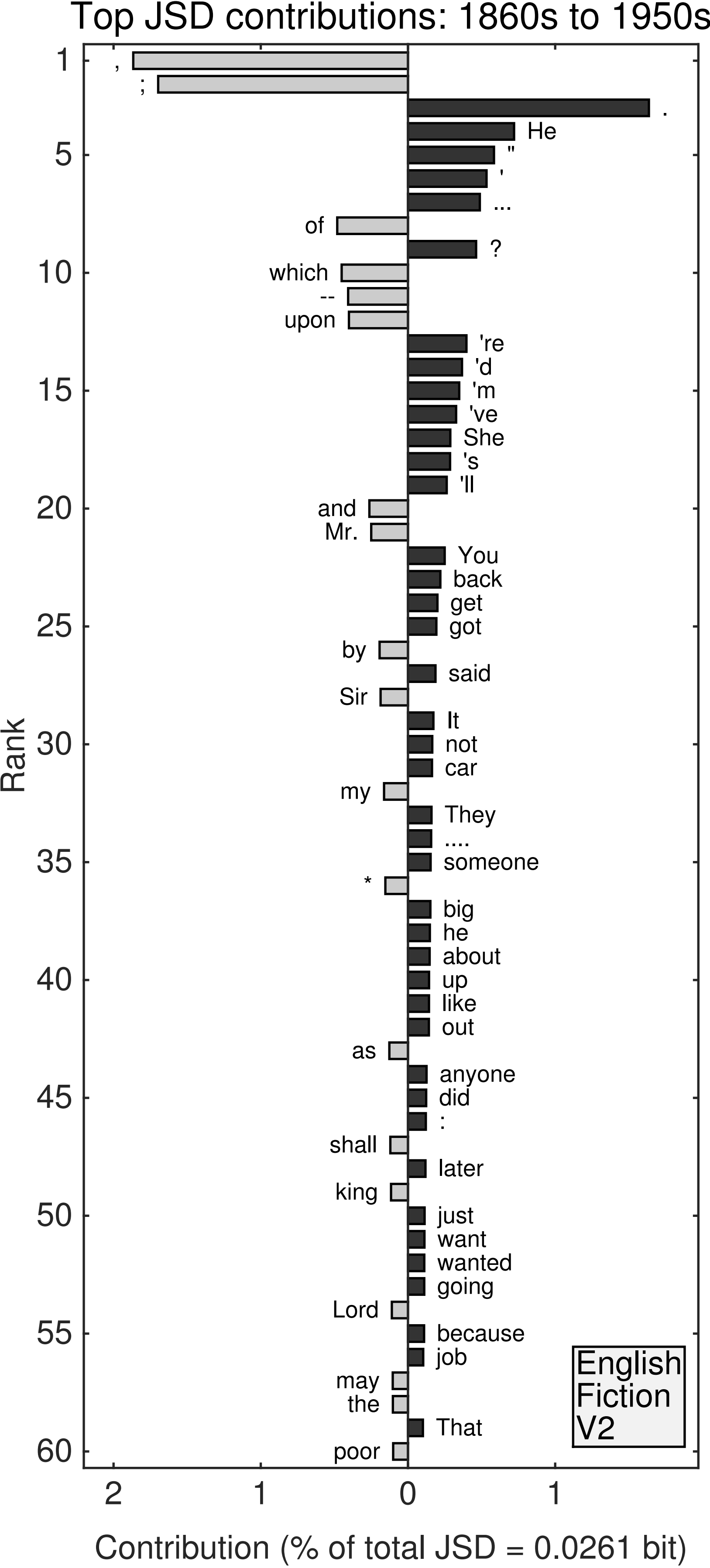

Top JSD contributions: 1860s to 1950s
Rank
Contribution (% of total JSD = 0.0261 bit)
English Fiction V2
1
5
10
15
20
25
30
35
40
45
50
55
60
2
1
0
1
,
;
.
He
"
'
...
of
?
which
--
upon
're
'd
'm
've
She
's
'll
and
Mr.
You
back
get
got
by
said
Sir
It
not
car
my
They
....
someone
*
big
he
about
up
like
out
as
anyone
did
:
shall
later
king
just
want
wanted
going
Lord
because
job
may
the
That
poor

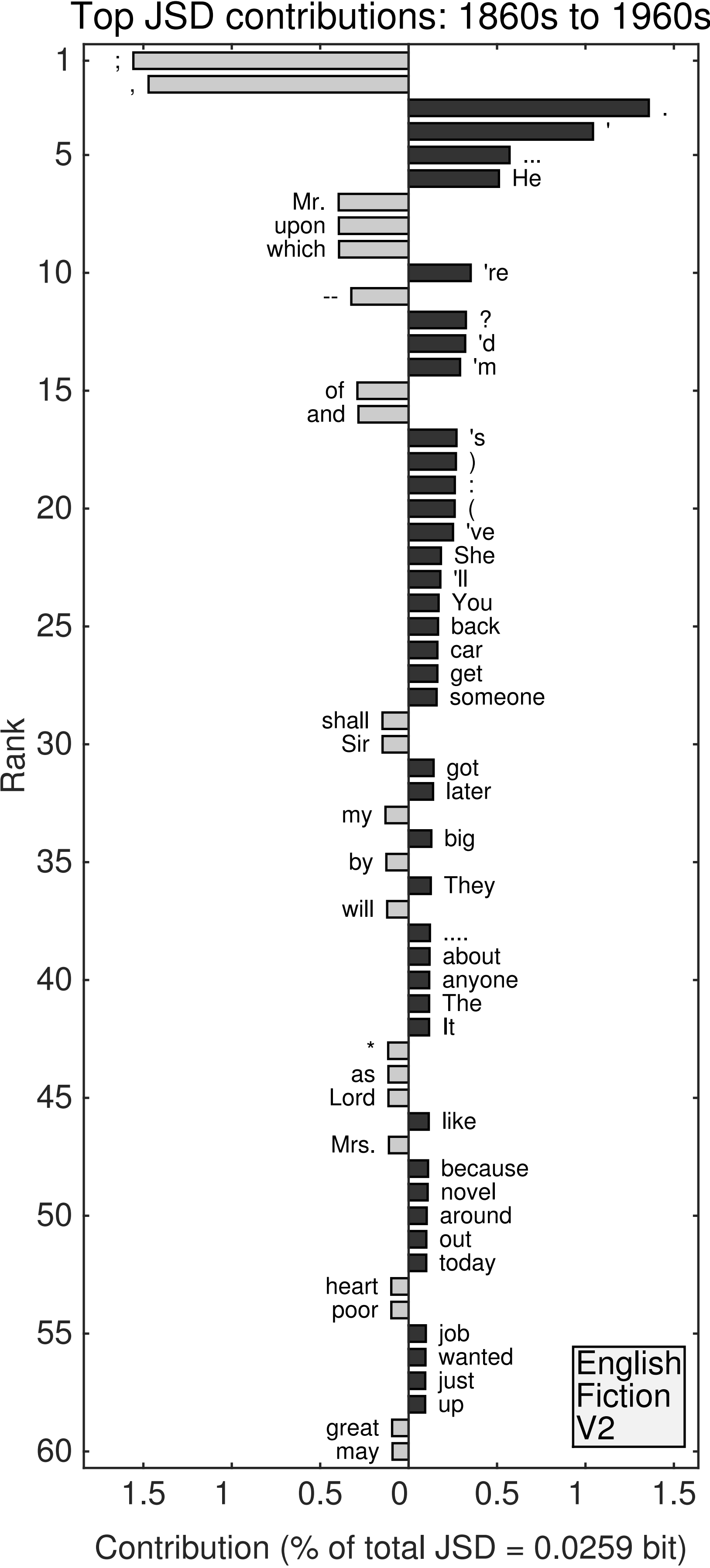

Top JSD contributions: 1860s to 1960s
Rank
Contribution (% of total JSD = 0.0259 bit)
English Fiction V2
;
,
.
'
...
He
Mr.
upon
which
're
--
?
'd
'm
of
and
's
)
:
(
've
She
'll
You
back
car
get
someone
shall
Sir
got
later
my
big
by
They
will
....
about
anyone
The
It
*
as
Lord
like
Mrs.
because
novel
around
out
today
heart
poor
job
wanted
just
up
great
may

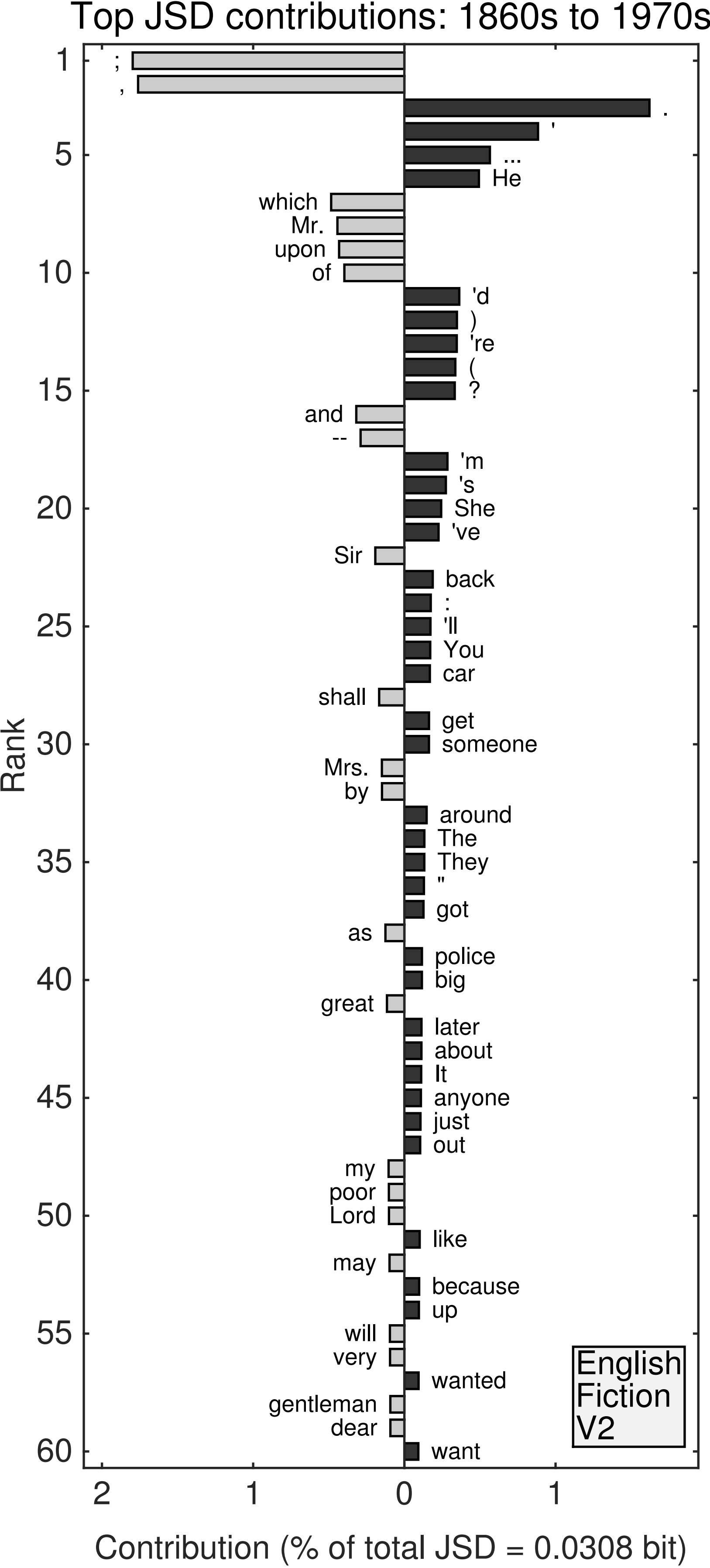

Top JSD contributions: 1860s to 1970s
Rank
Contribution (% of total JSD = 0.0308 bit)
English Fiction V2
;
,
.
'
...
He
which
Mr.
upon
of
'd
)
're
(
?
and
--
'm
's
She
've
Sir
back
:
'll
You
car
shall
get
someone
Mrs.
by
around
The
They
"
got
as
police
big
great
later
about
It
anyone
just
out
my
poor
Lord
like
may
because
up
will
very
wanted
gentleman
dear
want

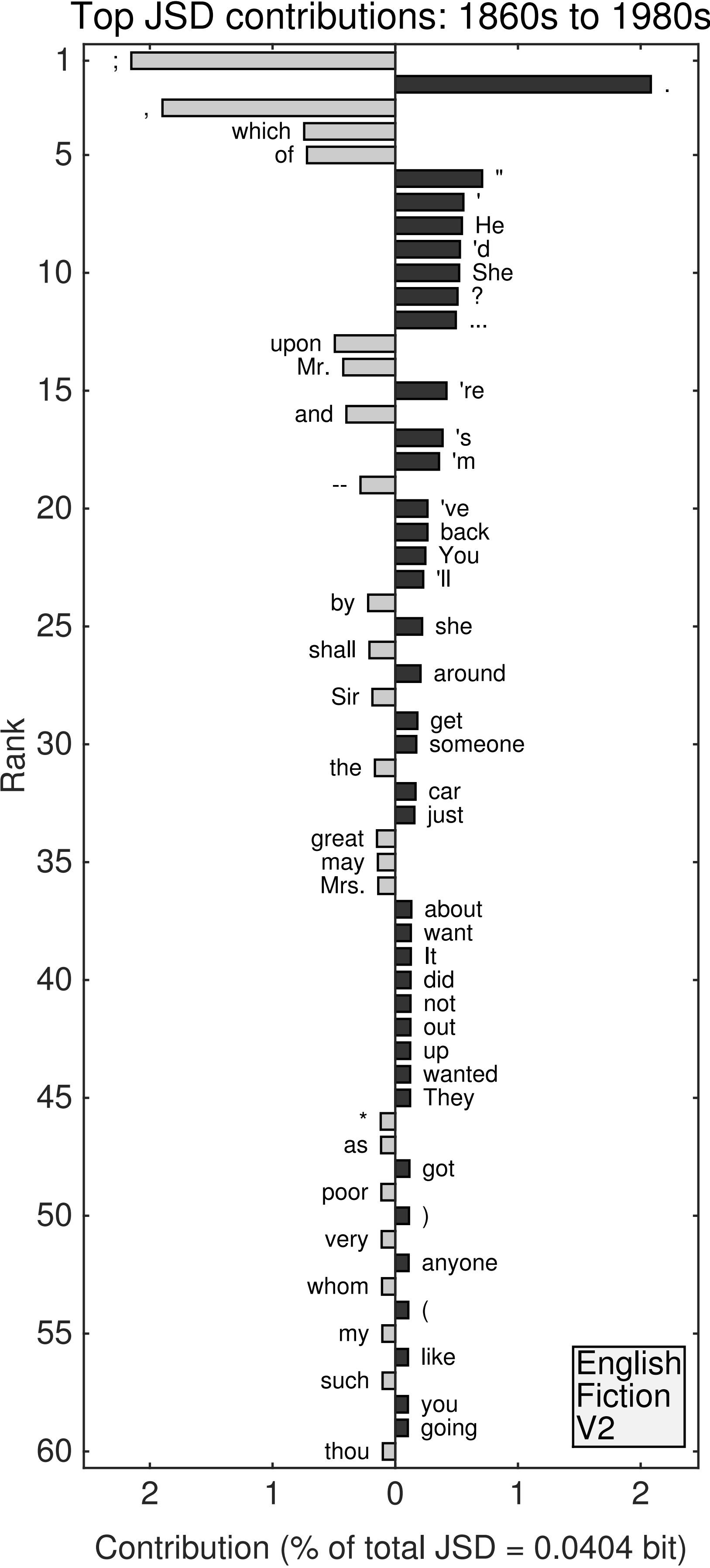
Top JSD contributions: 1860s to 1980s
Rank
Contribution (% of total JSD = 0.0404 bit)
English Fiction V2
;
.
,
which
of
"
'
He
'd
She
?
...
upon
Mr.
're
and
's
'm
--
've
back
You
'll
by
she
shall
around
Sir
get
someone
the
car
just
great
may
Mrs.
about
want
It
did
not
out
up
wanted
They
*
as
got
poor
)
very
anyone
whom
(
my
like
such
you
going
thou

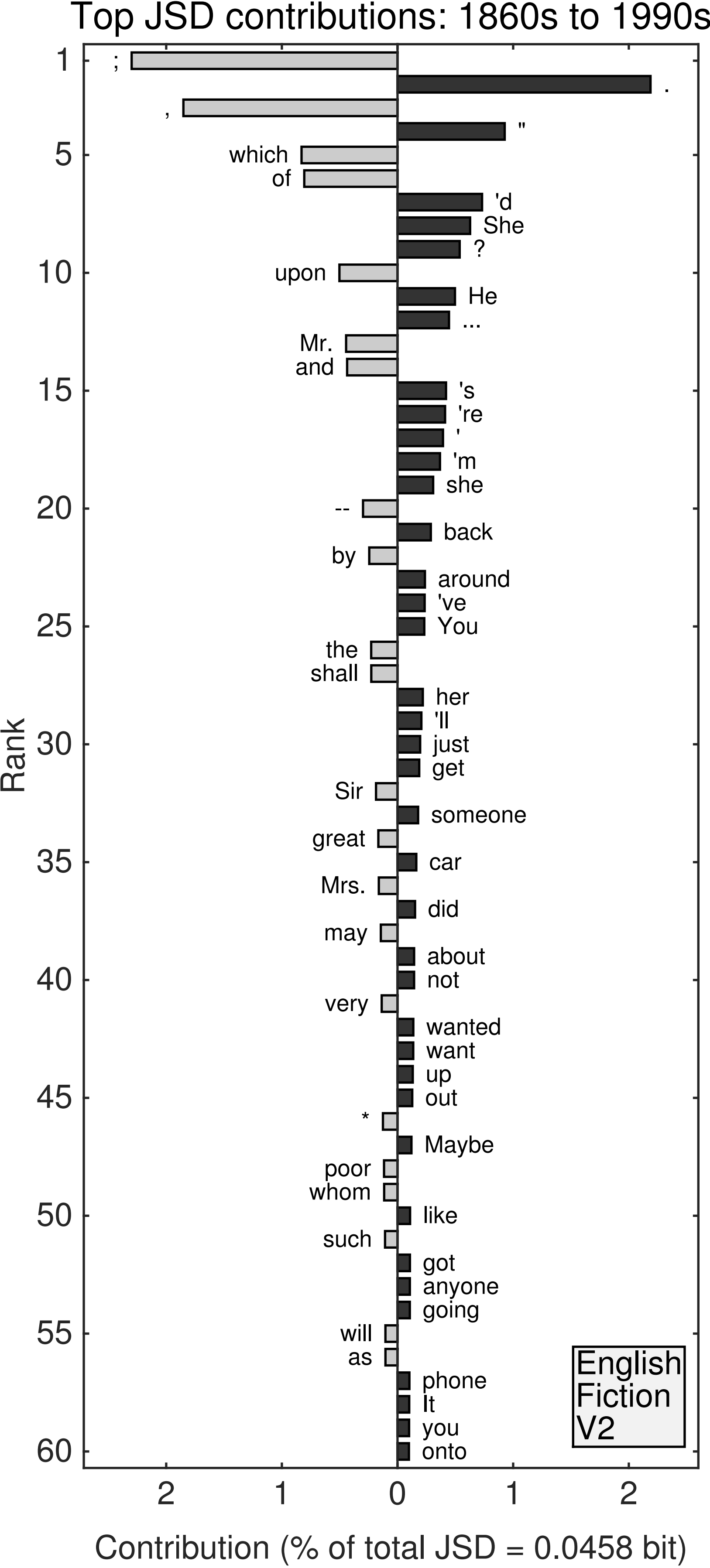

Top JSD contributions: 1860s to 1990s
Rank
1
5
10
15
20
25
30
35
40
45
50
55
60
;
.
,
"
which
of
'd
She
?
upon
He
...
Mr.
and
's
're
'
'm
she
--
back
by
around
've
You
the
shall
her
'll
just
get
Sir
someone
great
car
Mrs.
did
may
about
not
very
wanted
want
up
out
*
Maybe
poor
whom
like
such
got
anyone
going
will
as
phone
It
you
onto
English Fiction V2
2
1
0
1
2
Contribution (% of total JSD = 0.0458 bit)

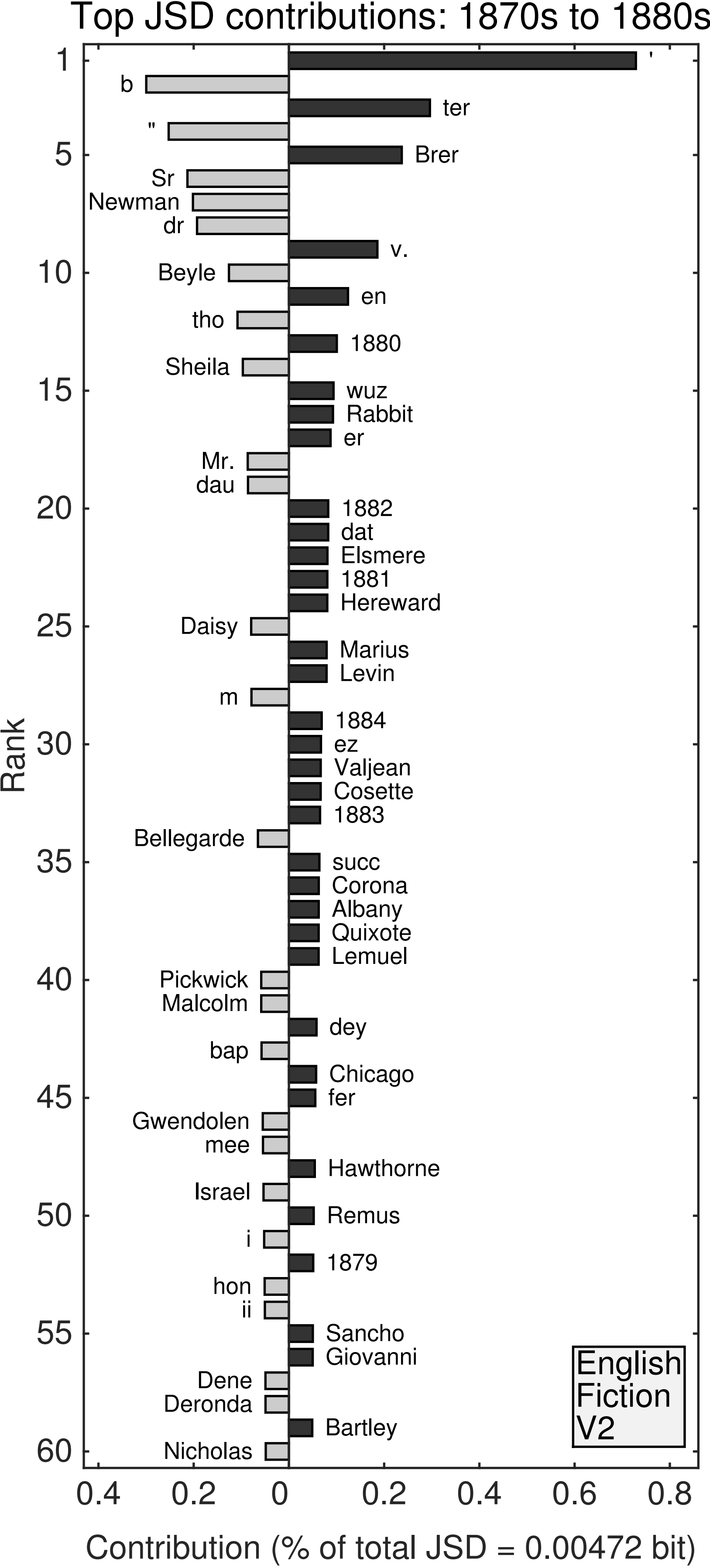
Top JSD contributions: 1870s to 1880s
Rank
Contribution (% of total JSD = 0.00472 bit)
'
b
ter
"
Brer
Sr
Newman
dr
v.
Beyle
en
tho
1880
Sheila
wuz
Rabbit
er
Mr.
dau
1882
dat
Elsmere
1881
Hereward
Daisy
Marius
Levin
m
1884
ez
Valjean
Cosette
1883
Bellegarde
succ
Corona
Albany
Quixote
Lemuel
Pickwick
Malcolm
dey
bap
Chicago
fer
Gwendolen
mee
Hawthorne
Israel
Remus
i
1879
hon
ii
Sancho
Giovanni
Dene
Deronda
Bartley
Nicholas
English
Fiction
V2

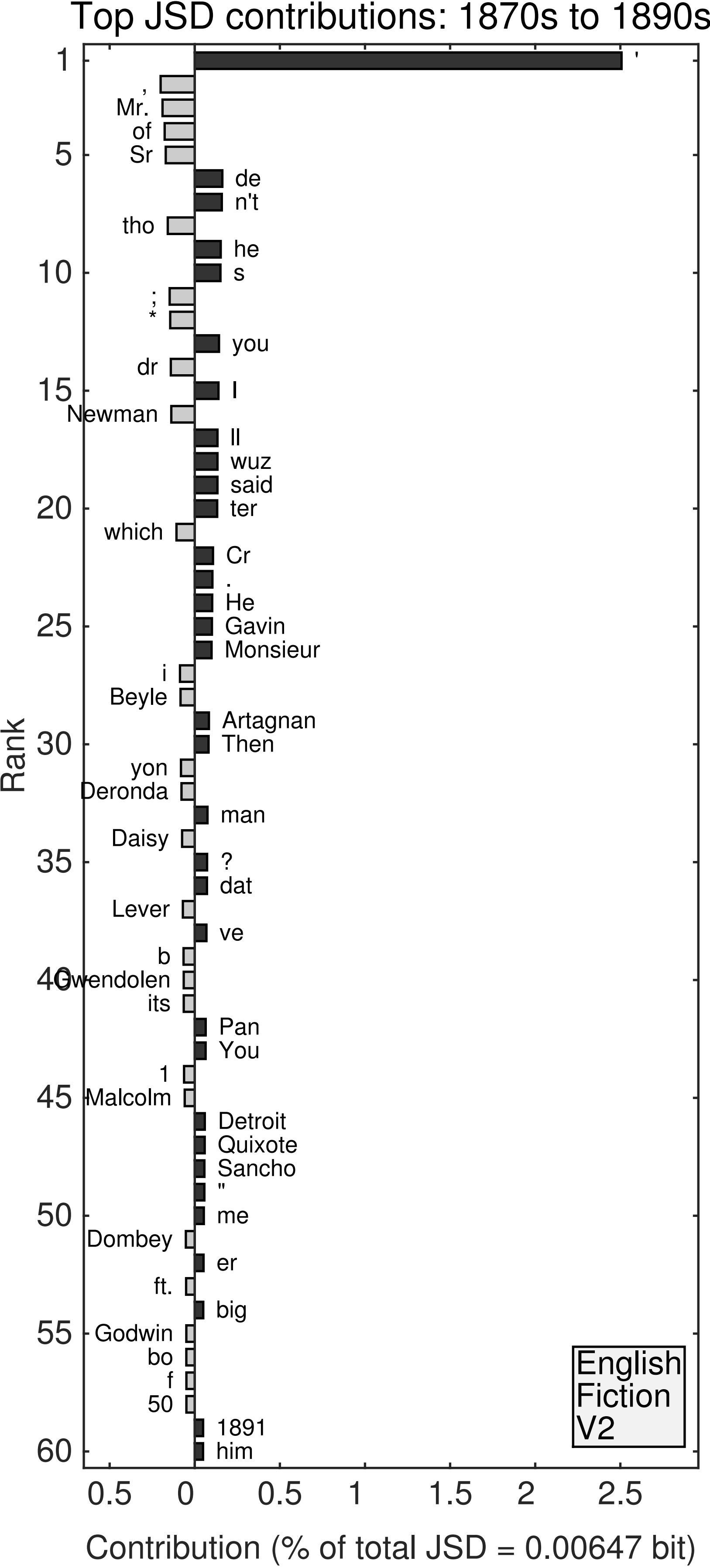

Top JSD contributions: 1870s to 1890s
Rank
Contribution (% of total JSD = 0.00647 bit)
'
,
Mr.
of
Sr
de
n't
tho
he
s
;
*
you
dr
I
Newman
ll
wuz
said
ter
which
Cr
.
He
Gavin
Monsieur
i
Beyle
Artagnan
Then
yon
Deronda
man
Daisy
?
dat
Lever
ve
b
Gwendolen
its
Pan
You
1
Malcolm
Detroit
Quixote
Sancho
"
me
Dombey
er
ft.
big
Godwin
bo
f
50
1891
him
English Fiction V2

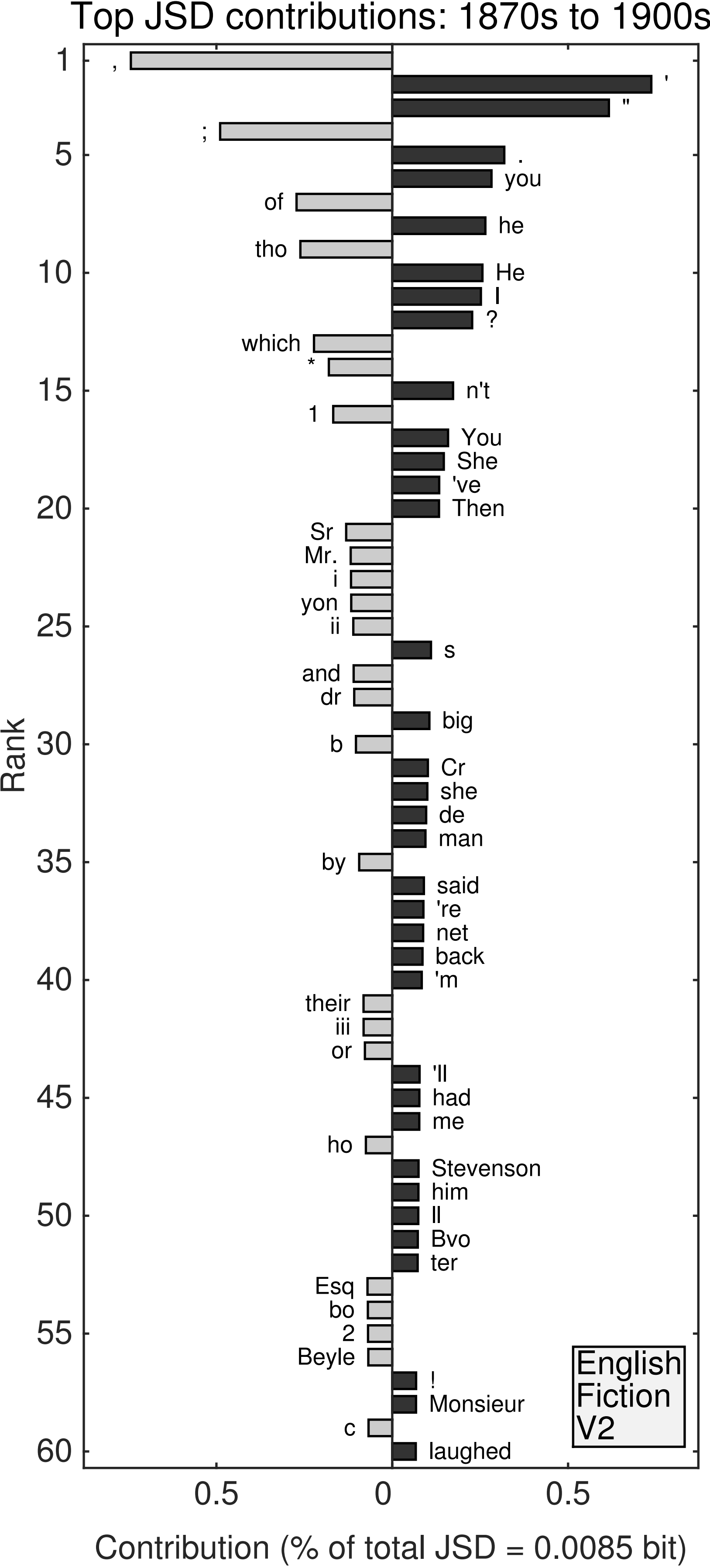

Top JSD contributions: 1870s to 1900s
Rank
Contribution (% of total JSD = 0.0085 bit)
English Fiction V2
,
'
"
;
.
you
of
he
tho
He
I
?
which
*
n't
1
You
She
've
Then
Sr
Mr.
i
yon
ii
s
and
dr
big
b
Cr
she
de
man
by
said
're
net
back
'm
their
iii
or
'll
had
me
ho
Stevenson
him
ll
Bvo
ter
Esq
bo
2
Beyle
!
Monsieur
c
laughed

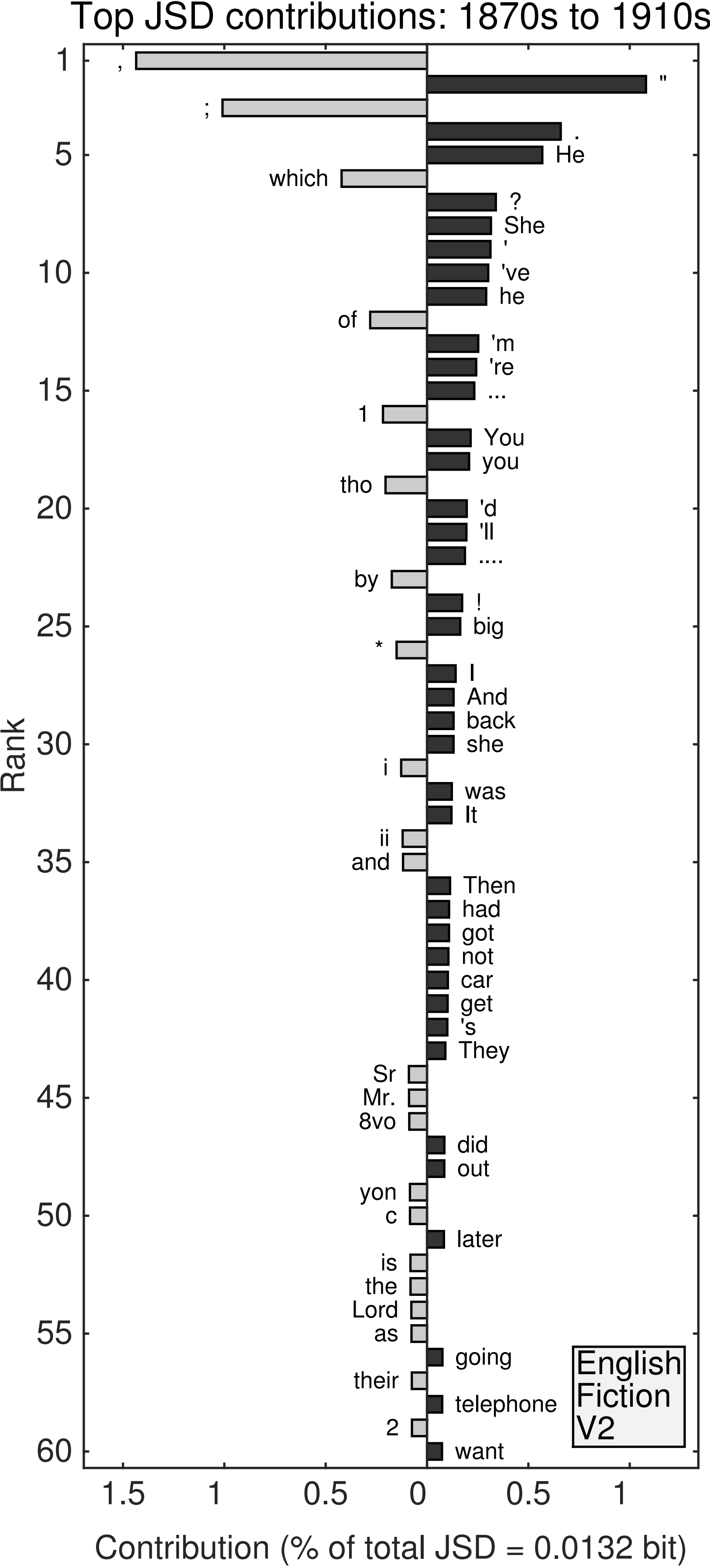

Top JSD contributions: 1870s to 1910s
Rank
Contribution (% of total JSD = 0.0132 bit)
English Fiction V2
1.5
1
0.5
0
0.5
1
1
5
10
15
20
25
30
35
40
45
50
55
60
,
"
;
.
He
which
?
She
'
've
he
of
'm
're
...
1
You
you
tho
'd
'll
....
by
!
big
*
I
And
back
she
i
was
It
ii
and
Then
had
got
not
car
get
's
They
Sr
Mr.
8vo
did
out
yon
c
later
is
the
Lord
as
going
their
telephone
2
want

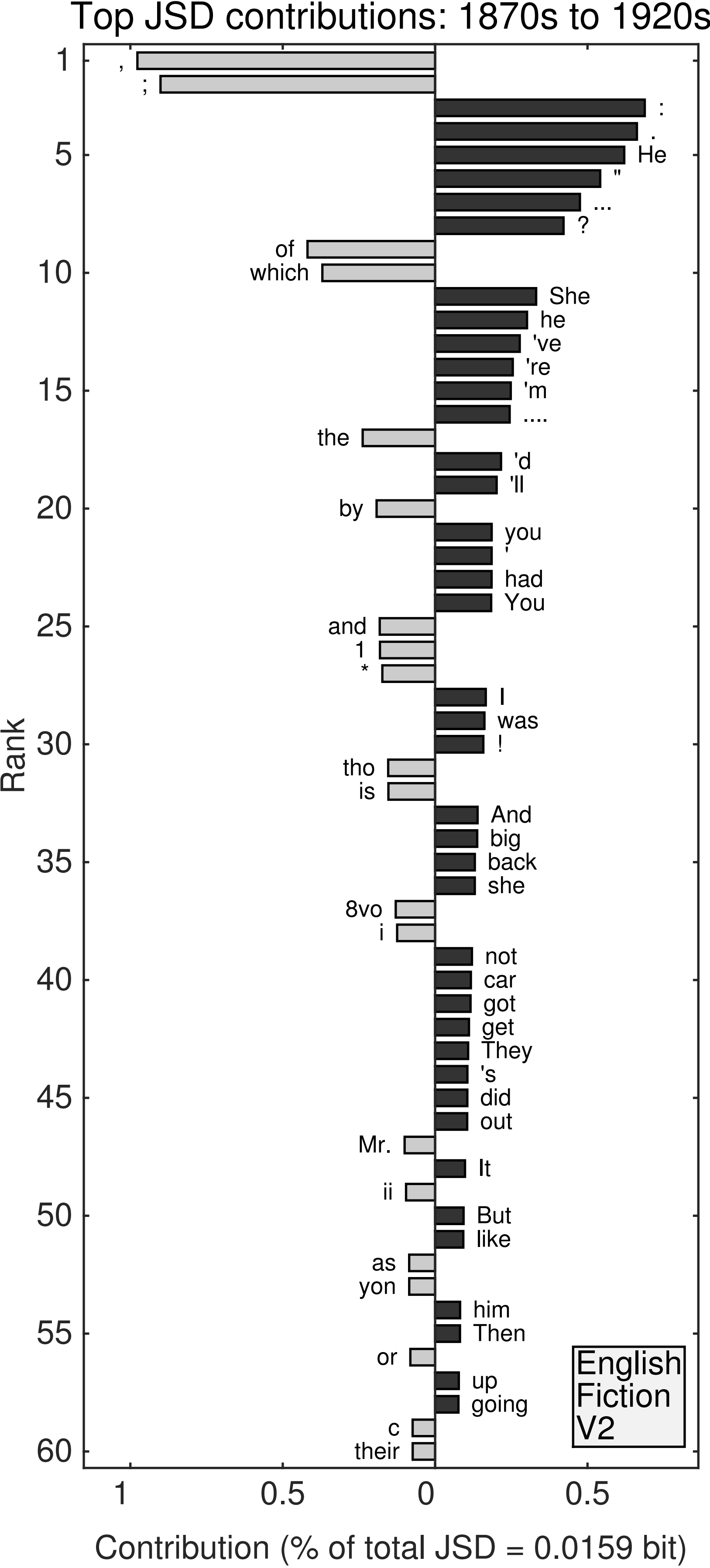
Top JSD contributions: 1870s to 1920s
Rank
Contribution (% of total JSD = 0.0159 bit)
English Fiction V2
1
5
10
15
20
25
30
35
40
45
50
55
60
1
0.5
0
0.5
,
;
:
.
He
"
...
?
of
which
She
he
've
're
'm
....
the
'd
'll
by
you
'
had
You
and
1
*
I
was
!
tho
is
And
big
back
she
8vo
i
not
car
got
get
They
's
did
out
Mr.
It
ii
But
like
as
yon
him
Then
or
up
going
c
their

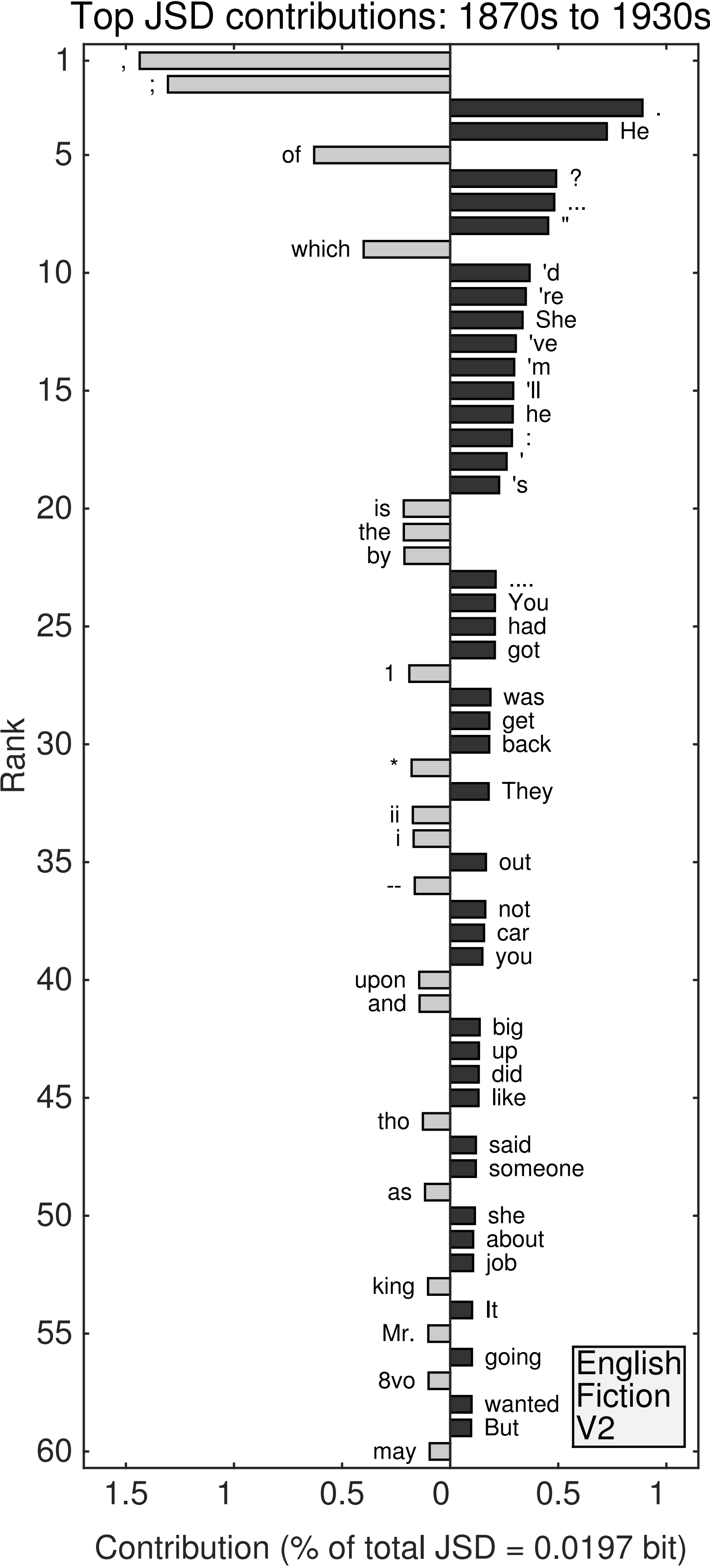
Top JSD contributions: 1870s to 1930s
Rank
Contribution (% of total JSD = 0.0197 bit)
English Fiction V2
1
5
10
15
20
25
30
35
40
45
50
55
60
1.5
1
0.5
0
0.5
1
,
;
.
He
of
?
...
"
which
'd
're
She
've
'm
'll
he
:
'
's
is
the
by
....
You
had
got
1
was
get
back
*
They
ii
i
out
--
not
car
you
upon
and
big
up
did
like
tho
said
someone
as
she
about
job
king
It
Mr.
going
8vo
wanted
But
may

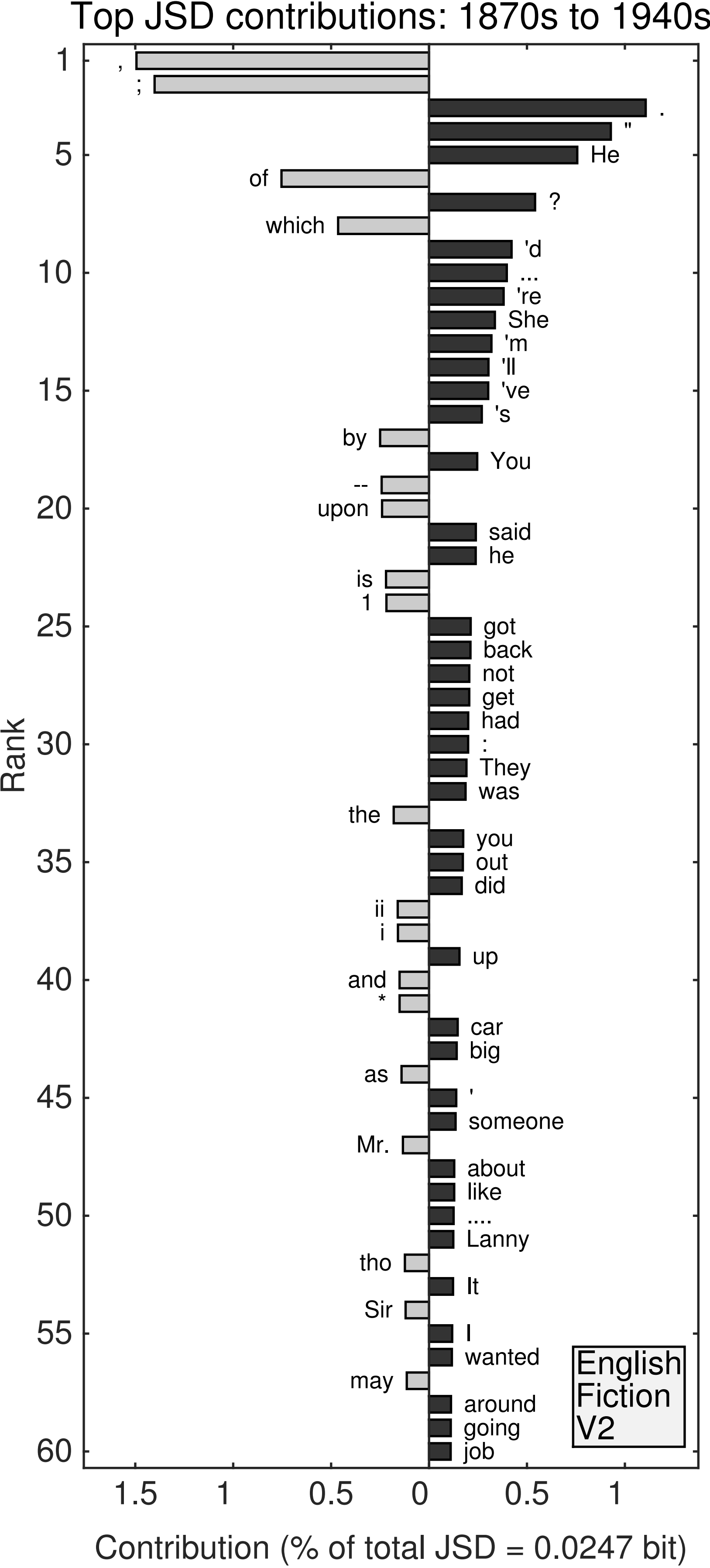

Top JSD contributions: 1870s to 1940s
Rank
Contribution (% of total JSD = 0.0247 bit)
English Fiction V2
, ; . " He of ? which 'd ... 're She 'm 'll 've 's by You -- upon said he is 1 got back not get had : They was the you out did ii i up and * car big as ' someone Mr. about like .... Lanny tho It Sir I wanted may around going job

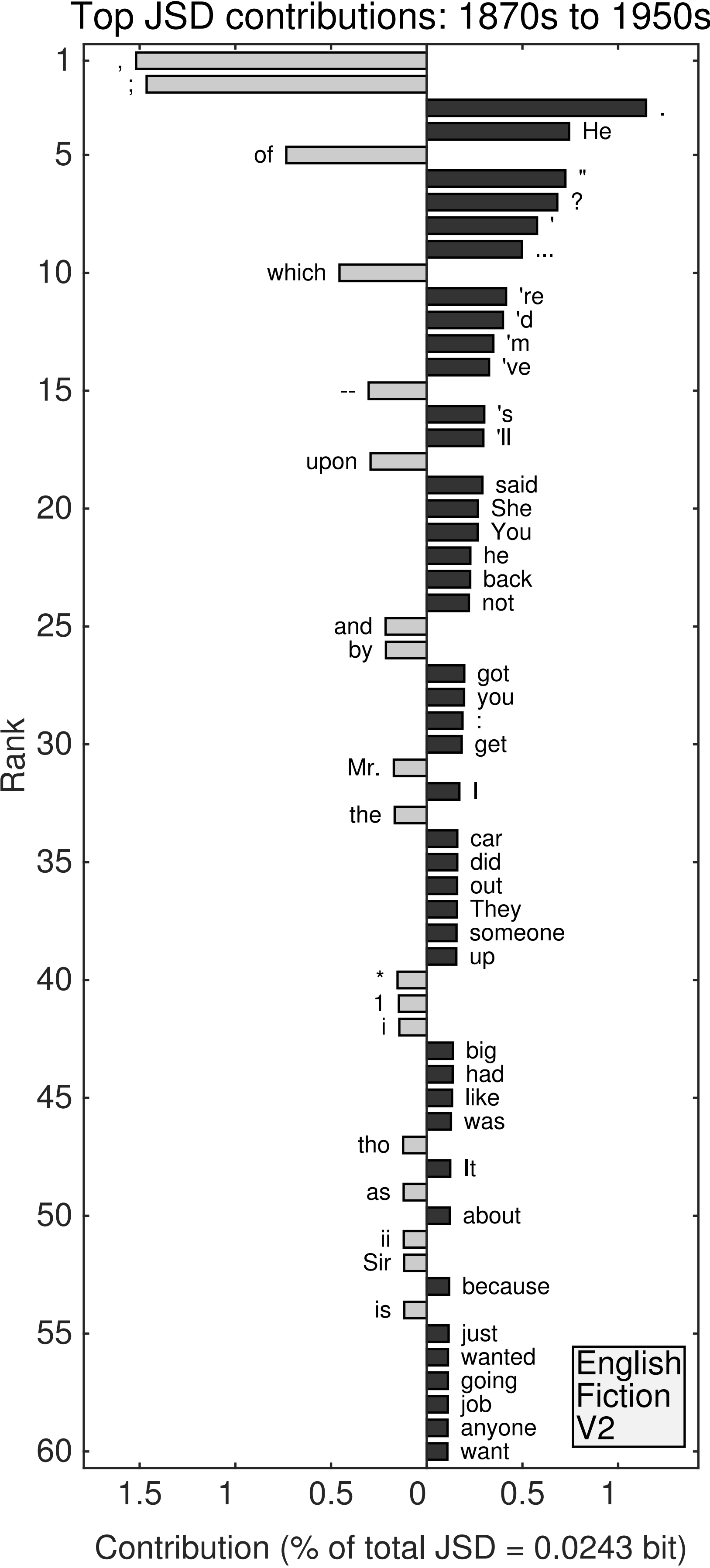
Top JSD contributions: 1870s to 1950s
Rank
Contribution (% of total JSD = 0.0243 bit)
English Fiction V2
,
;
.
He
of
"
?
'
...
which
're
'd
'm
've
--
's
'll
upon
said
She
You
he
back
not
and
by
got
you
:
get
Mr.
I
the
car
did
out
They
someone
up
*
1
i
big
had
like
was
tho
It
as
about
ii
Sir
because
is
just
wanted
going
job
anyone
want

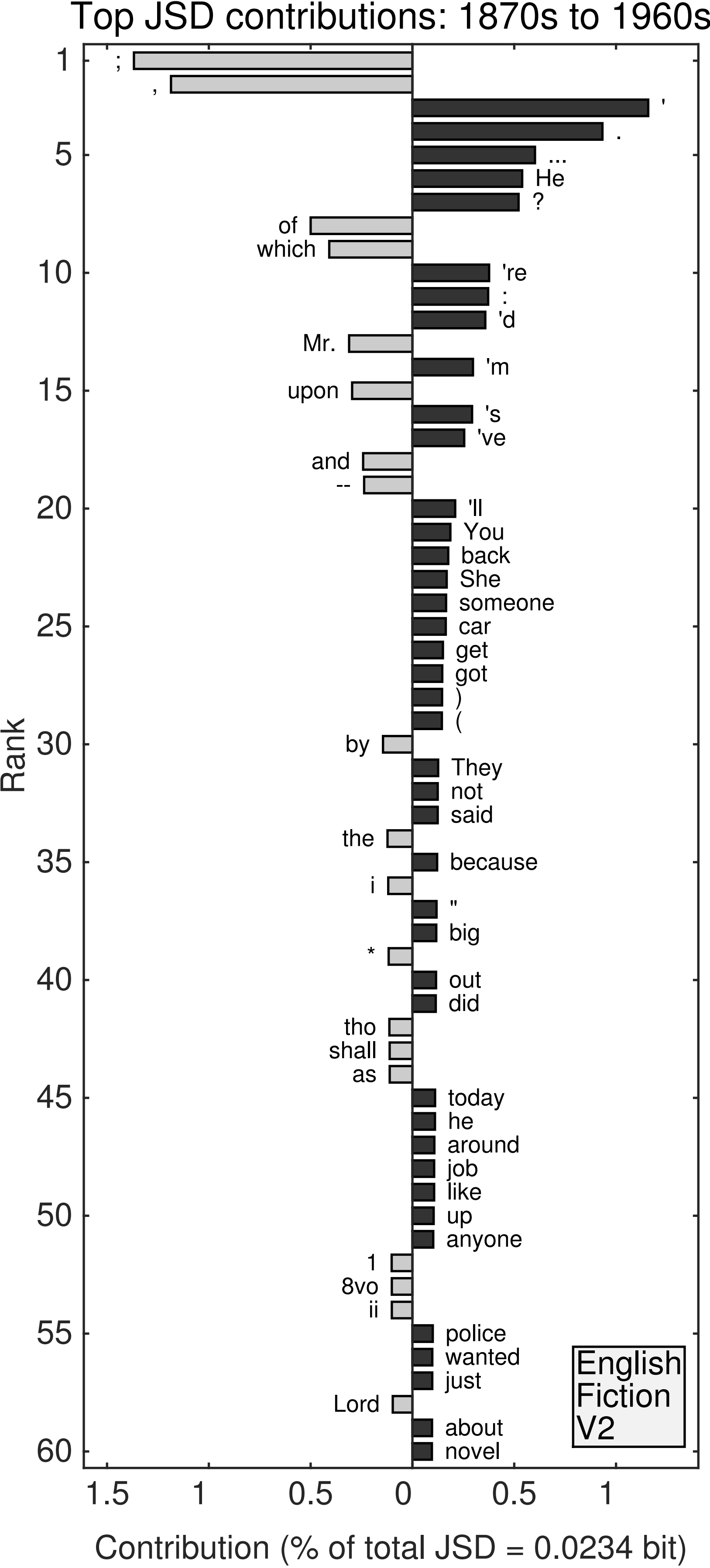
Top JSD contributions: 1870s to 1960s
Rank
Contribution (% of total JSD = 0.0234 bit)
English Fiction V2
;
,
'
.
...
He
?
of
which
're
:
'd
Mr.
'm
upon
's
've
and
--
'll
You
back
She
someone
car
get
got
)
(
by
They
not
said
the
because
i
"
big
*
out
did
tho
shall
as
today
he
around
job
like
up
anyone
1
8vo
ii
police
wanted
just
Lord
about
novel
1
5
10
15
20
25
30
35
40
45
50
55
60
1.5
1
0.5
0
0.5
1

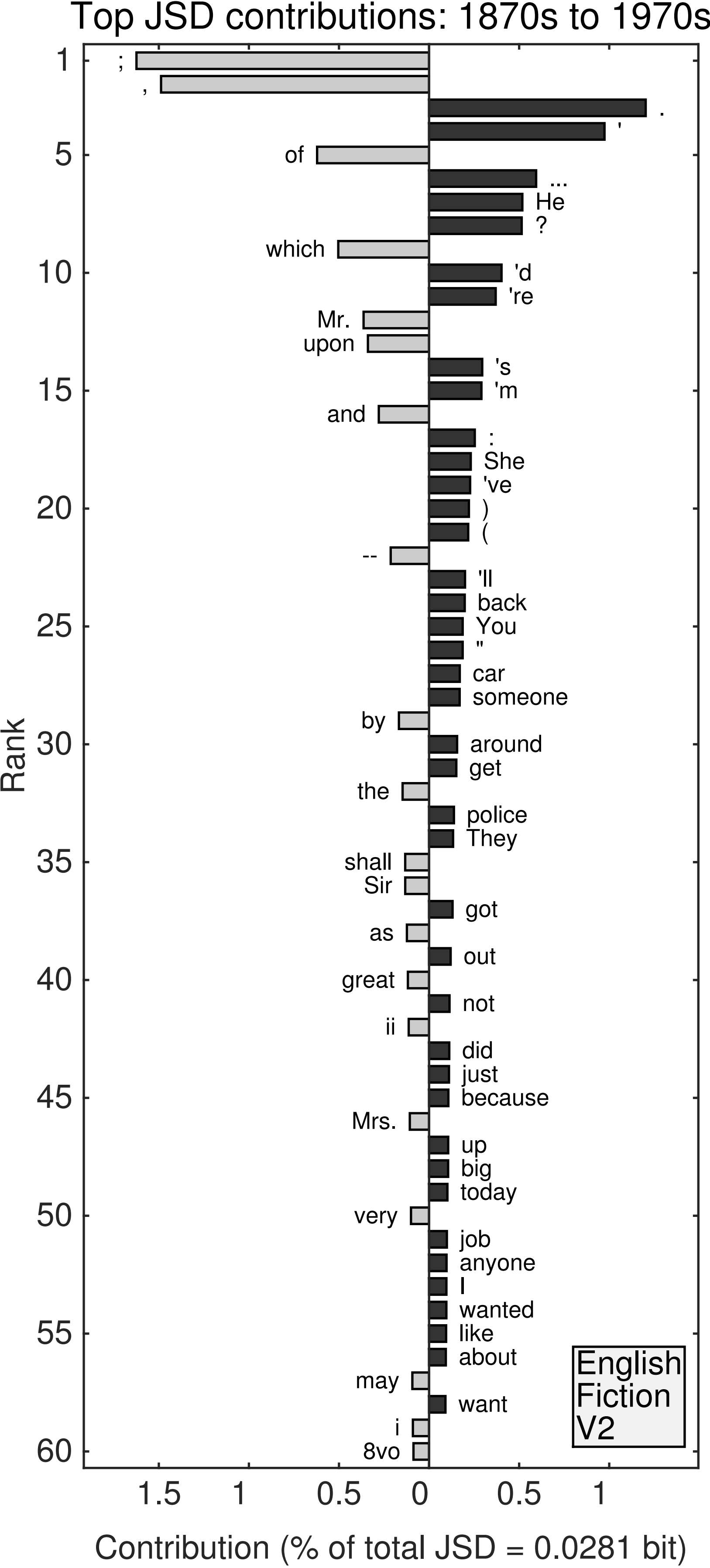

Top JSD contributions: 1870s to 1970s
Rank
Contribution (% of total JSD = 0.0281 bit)
English Fiction V2
;
,
.
'
of
...
He
?
which
'd
're
Mr.
upon
's
'm
and
:
She
've
)
(
--
'll
back
You
"
car
someone
by
around
get
the
police
They
shall
Sir
got
as
out
great
not
ii
did
just
because
Mrs.
up
big
today
very
job
anyone
I
wanted
like
about
may
want
i
8vo

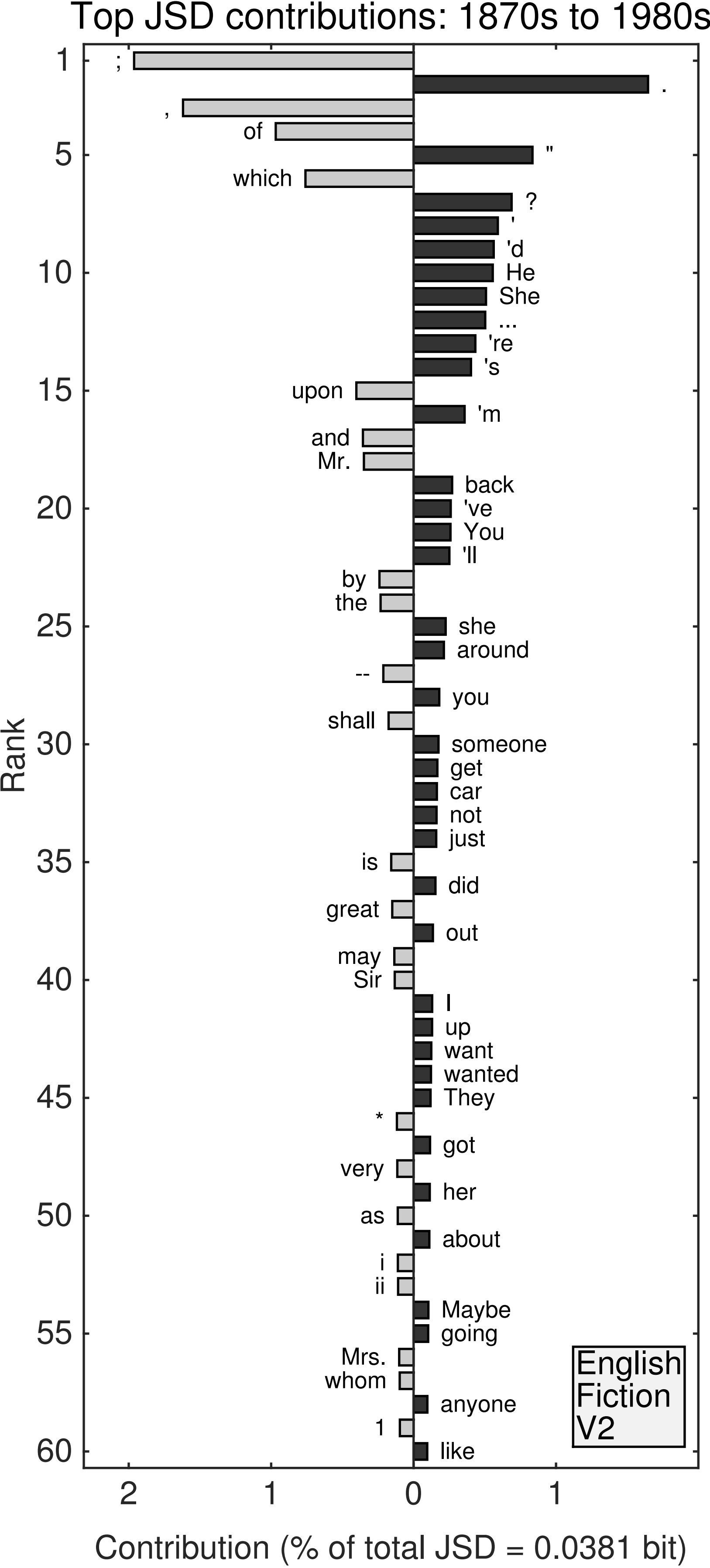
Top JSD contributions: 1870s to 1980s
Rank
Contribution (% of total JSD = 0.0381 bit)
English Fiction V2
;
.
,
of
"
which
?
'
'd
He
She
...
're
's
upon
'm
and
Mr.
back
've
You
'll
by
the
she
around
--
you
shall
someone
get
car
not
just
is
did
great
out
may
Sir
I
up
want
wanted
They
*
got
very
her
as
about
i
ii
Maybe
going
Mrs.
whom
anyone
1
like

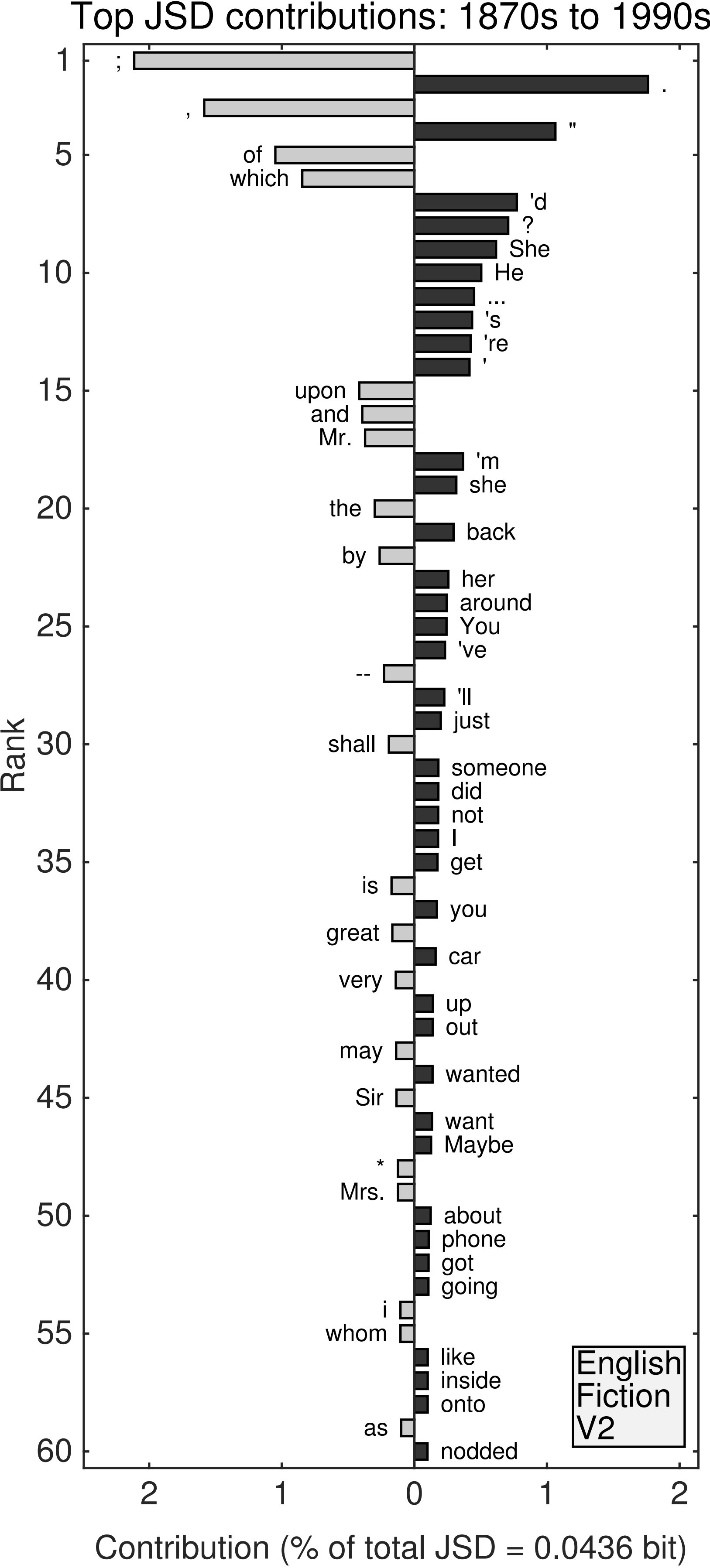
Top JSD contributions: 1870s to 1990s
Rank
Contribution (% of total JSD = 0.0436 bit)
English Fiction V2
;
.
,
"
of
which
'd
?
She
He
...
's
're
'
upon
and
Mr.
'm
she
the
back
by
her
around
You
've
--
'll
just
shall
someone
did
not
I
get
is
you
great
car
very
up
out
may
wanted
Sir
want
Maybe
*
Mrs.
about
phone
got
going
i
whom
like
inside
onto
as
nodded

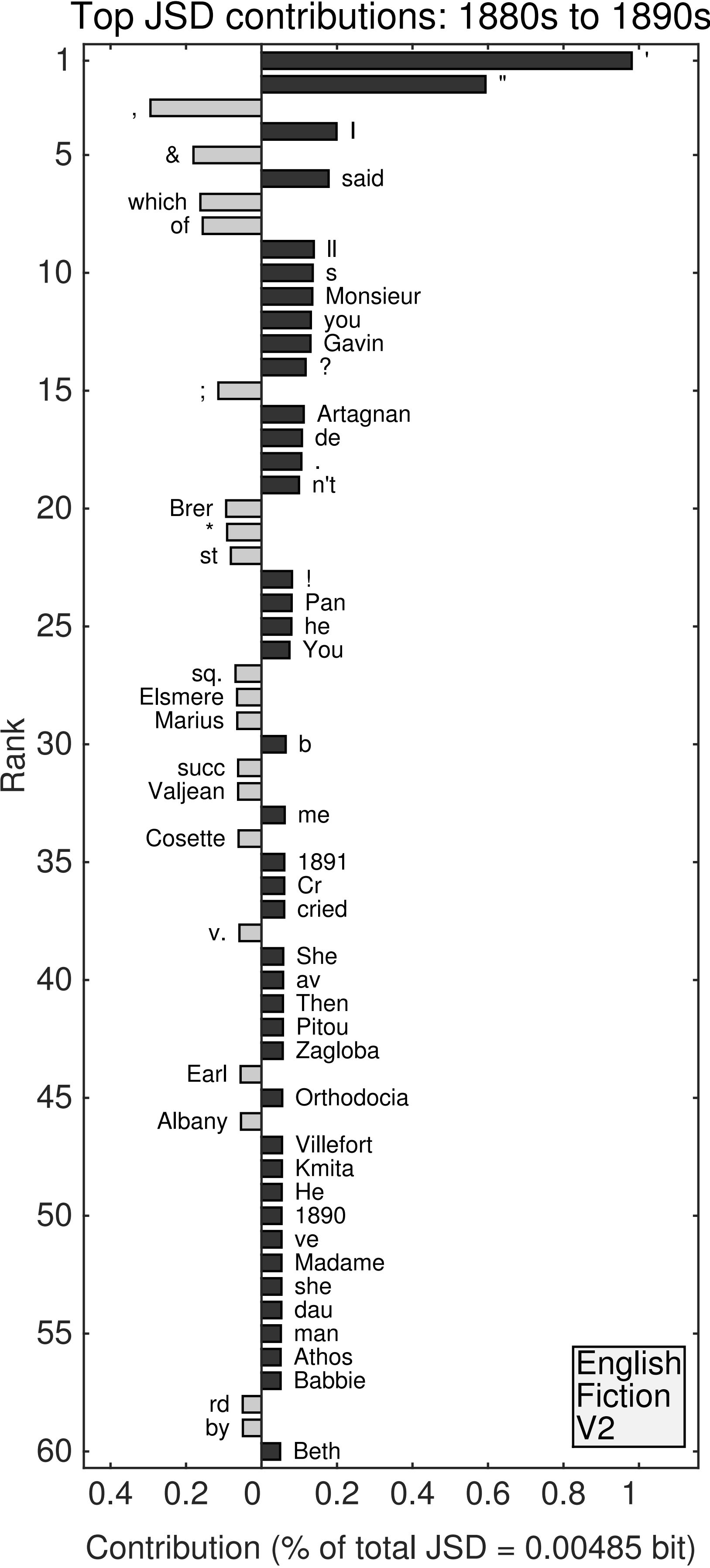
Top JSD contributions: 1880s to 1890s
Rank
Contribution (% of total JSD = 0.00485 bit)
English Fiction V2
'
"
,
I
&
said
which
of
ll
s
Monsieur
you
Gavin
?
;
Artagnan
de
.
n't
Brer
*
st
!
Pan
he
You
sq.
Elsmere
Marius
b
succ
Valjean
me
Cosette
1891
Cr
cried
v.
She
av
Then
Pitou
Zagloba
Earl
Orthodocia
Albany
Villefort
Kmita
He
1890
ve
Madame
she
dau
man
Athos
Babbie
rd
by
Beth

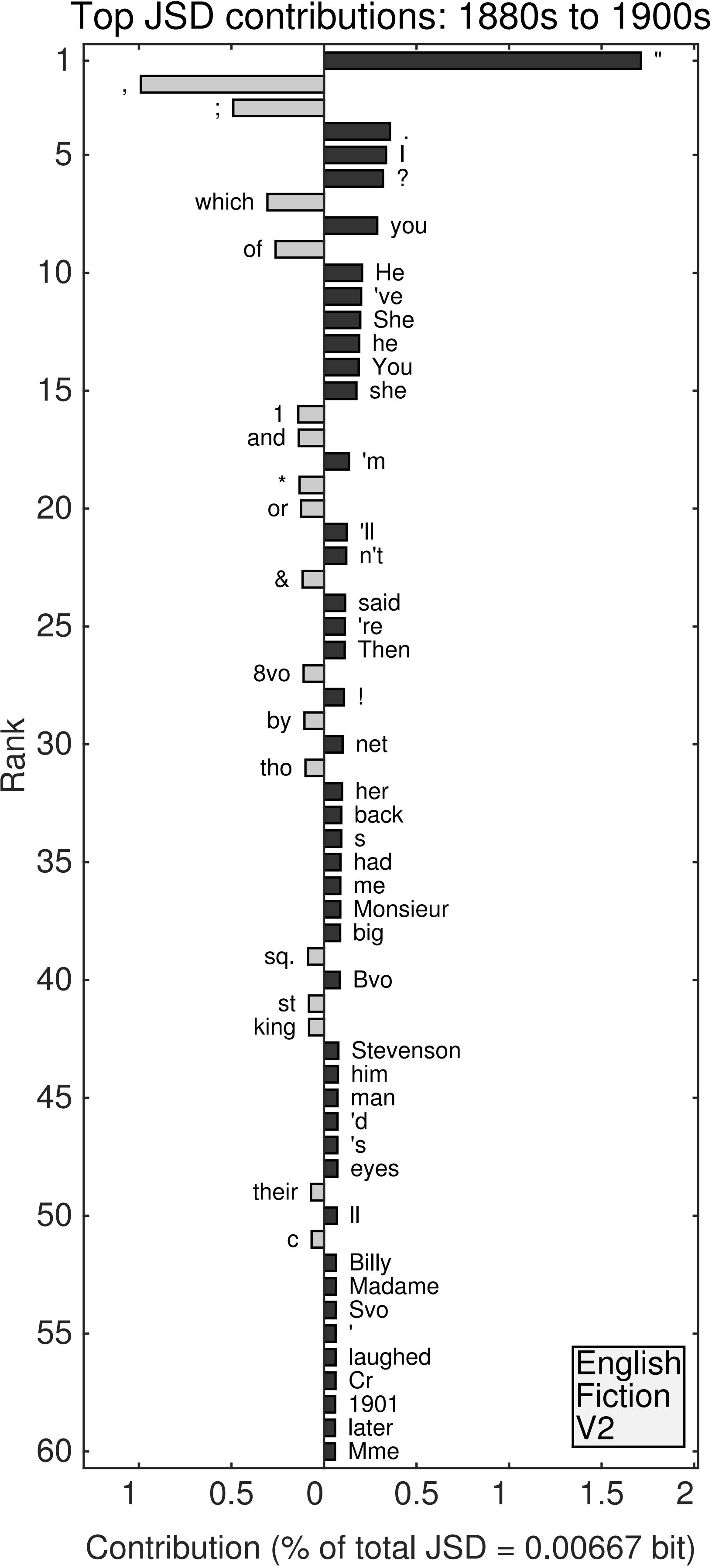
Top JSD contributions: 1880s to 1900s
Rank
Contribution (% of total JSD = 0.00667 bit)
"
,
;
.
I
?
which
you
of
He
've
She
he
You
she
1
and
'm
*
or
'll
n't
&
said
're
Then
8vo
!
by
net
tho
her
back
s
had
me
Monsieur
big
sq.
Bvo
st
king
Stevenson
him
man
'd
's
eyes
their
ll
c
Billy
Madame
Svo
'
laughed
Cr
1901
later
Mme
English
Fiction
V2

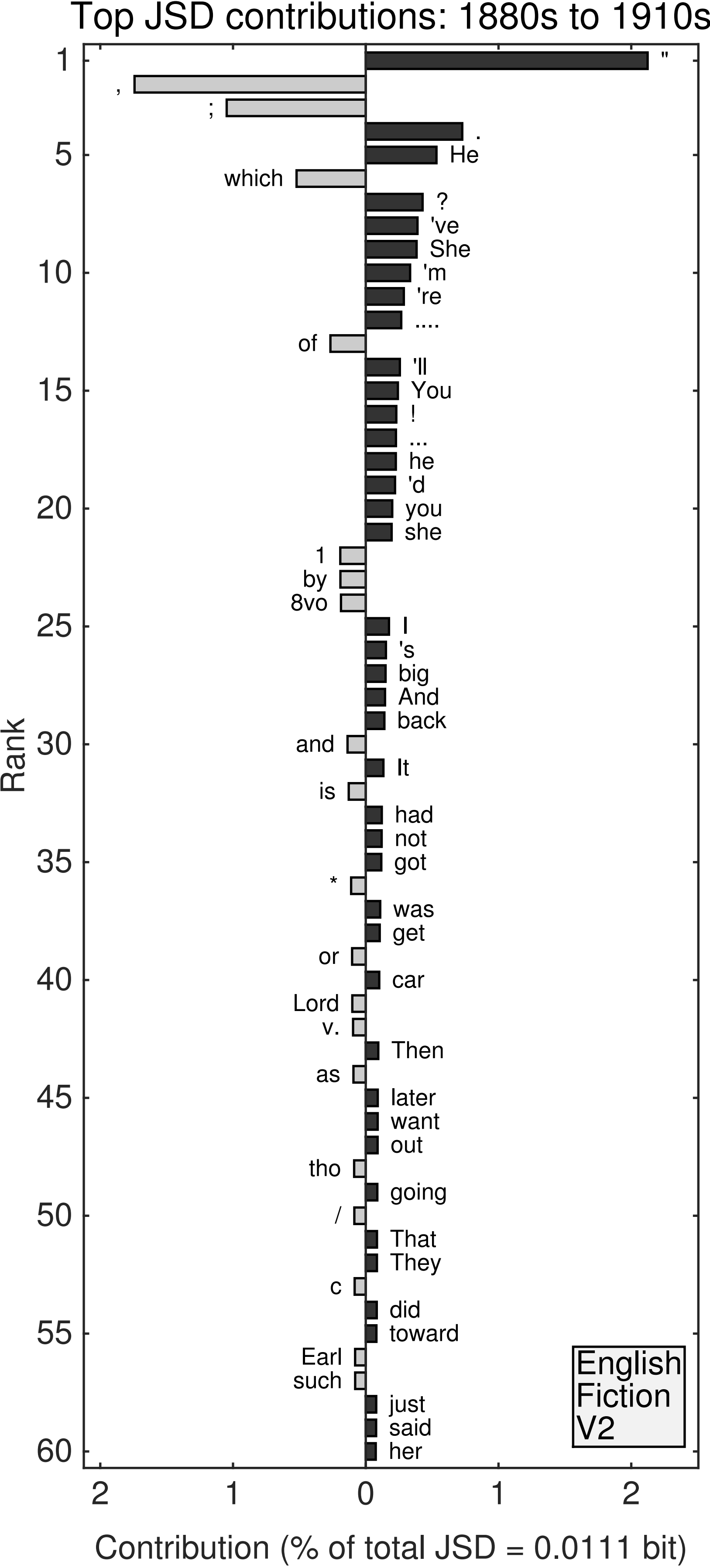
Top JSD contributions: 1880s to 1910s
Rank
Contribution (% of total JSD = 0.0111 bit)
English Fiction V2
"
,
;
.
He
which
?
've
She
'm
're
....
of
'll
You
!
...
he
'd
you
she
1
by
8vo
I
's
big
And
back
and
It
is
had
not
got
*
was
get
or
car
Lord
v.
Then
as
later
want
out
tho
going
/
That
They
c
did
toward
Earl
such
just
said
her
1
5
10
15
20
25
30
35
40
45
50
55
60
2
1
0
1
2

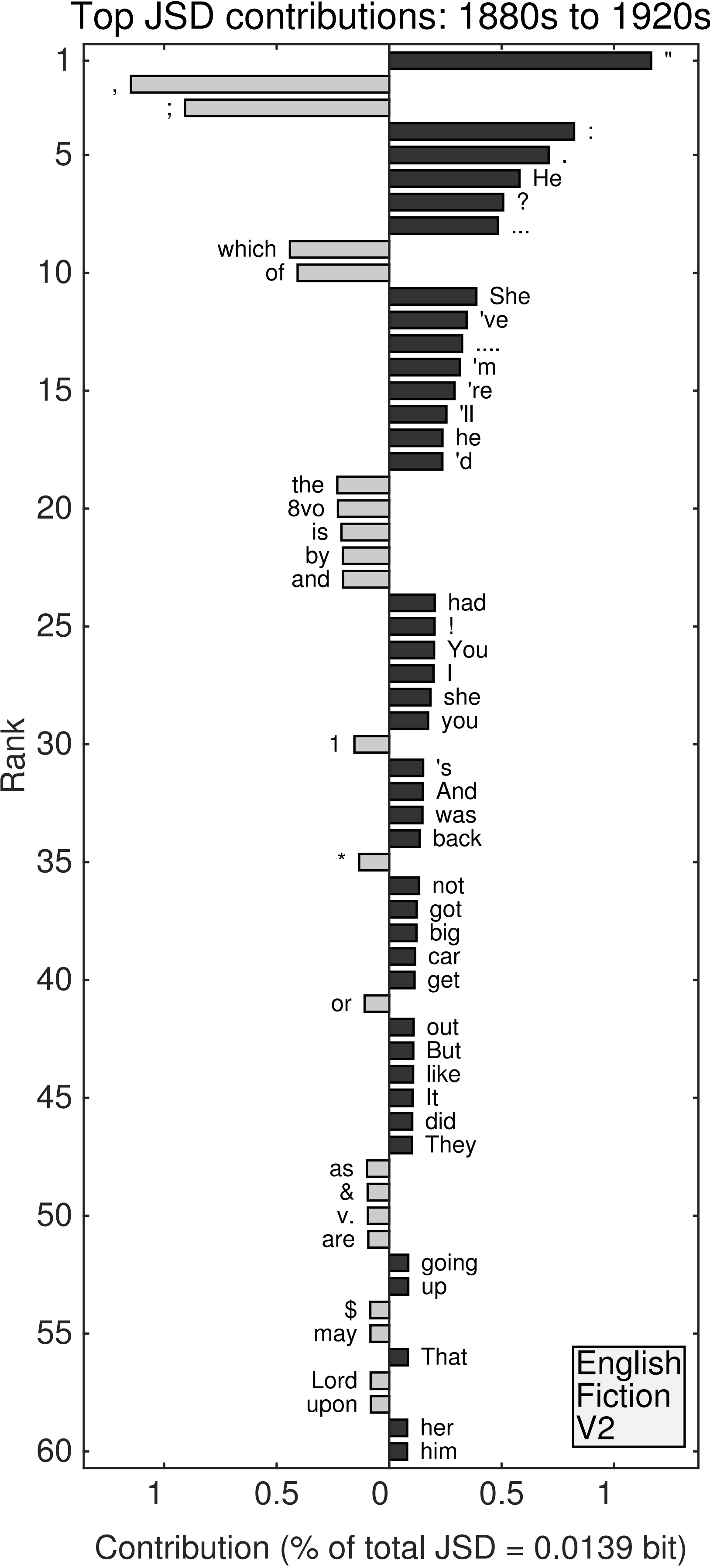
Top JSD contributions: 1880s to 1920s
Rank
Contribution (% of total JSD = 0.0139 bit)
English
Fiction
V2
"
,
;
:
.
He
?
...
which
of
She
've
....
'm
're
'll
he
'd
the
8vo
is
by
and
had
!
You
I
she
you
1
's
And
was
back
*
not
got
big
car
get
or
out
But
like
It
did
They
as
&
v.
are
going
up
$
may
That
Lord
upon
her
him

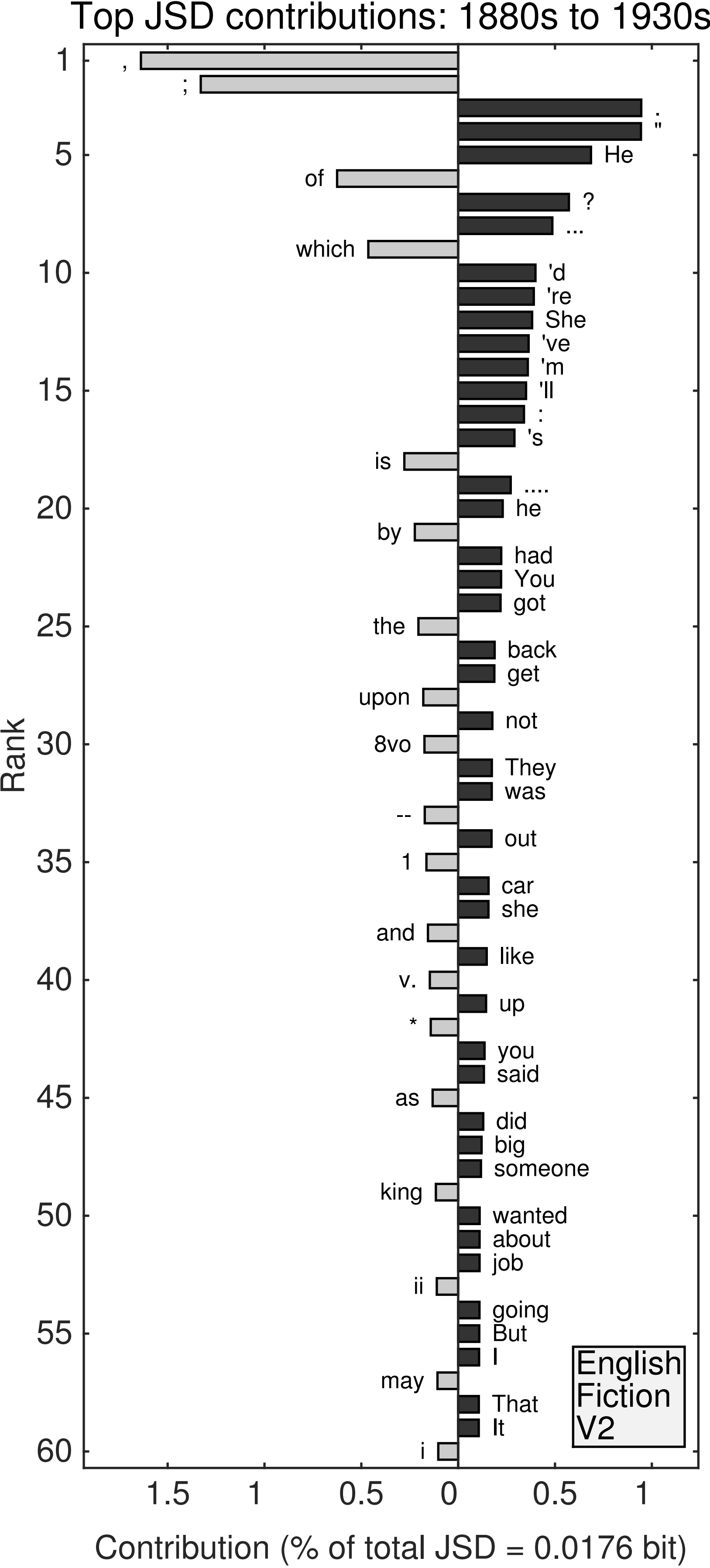
Top JSD contributions: 1880s to 1930s
Rank
Contribution (% of total JSD = 0.0176 bit)
English Fiction V2
,
;
:
"
He
of
?
...
which
'd
're
She
've
'm
'll
:
's
is
....
he
by
had
You
got
the
back
get
upon
not
8vo
They
was
--
out
1
car
she
and
like
v.
up
*
you
said
as
did
big
someone
king
wanted
about
job
ii
going
But
I
may
That
It
i

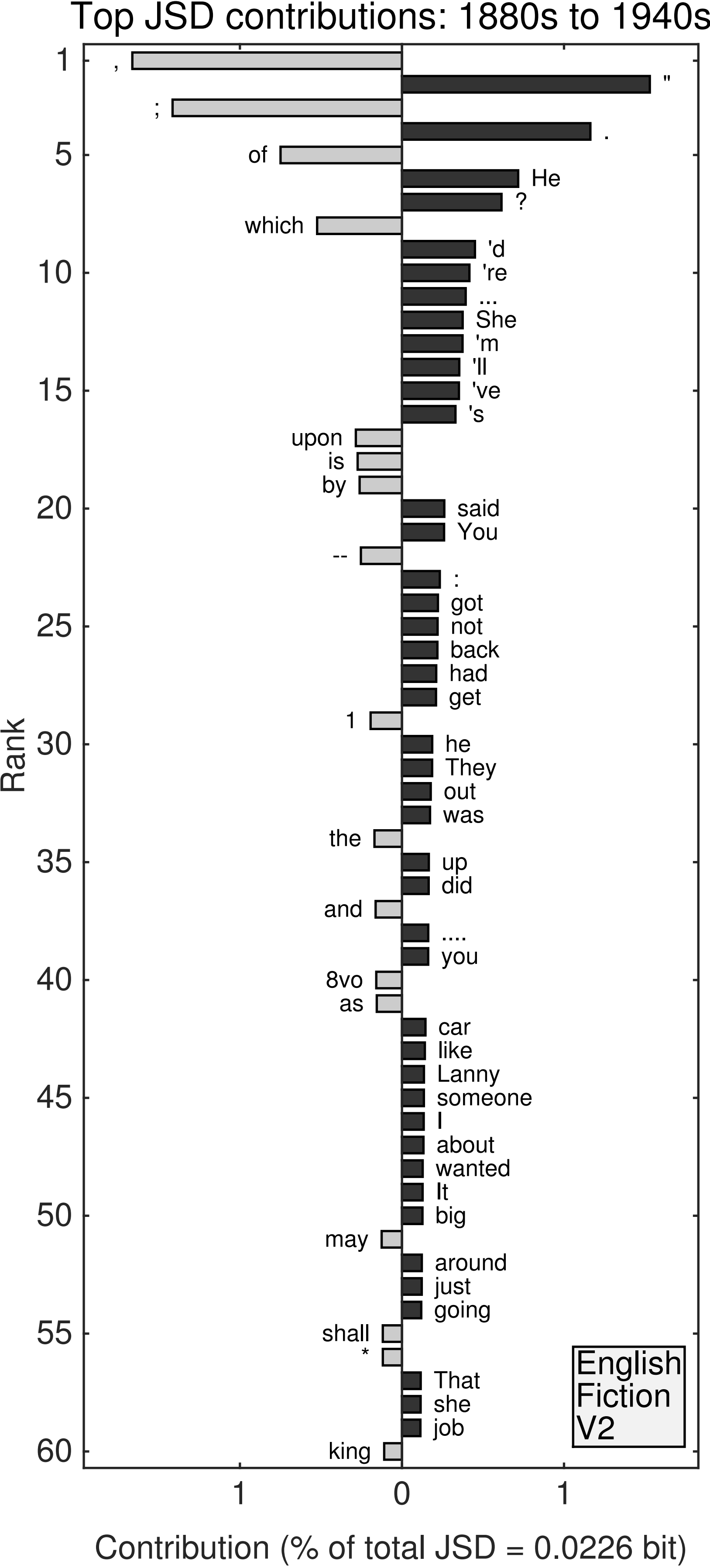

Top JSD contributions: 1880s to 1940s
Rank
Contribution (% of total JSD = 0.0226 bit)
English Fiction V2
,
"
;
.
of
He
?
which
'd
're
...
She
'm
'll
've
's
upon
is
by
said
You
--
:
got
not
back
had
get
1
he
They
out
was
the
up
did
and
....
you
8vo
as
car
like
Lanny
someone
I
about
wanted
It
big
may
around
just
going
shall
*
That
she
job
king

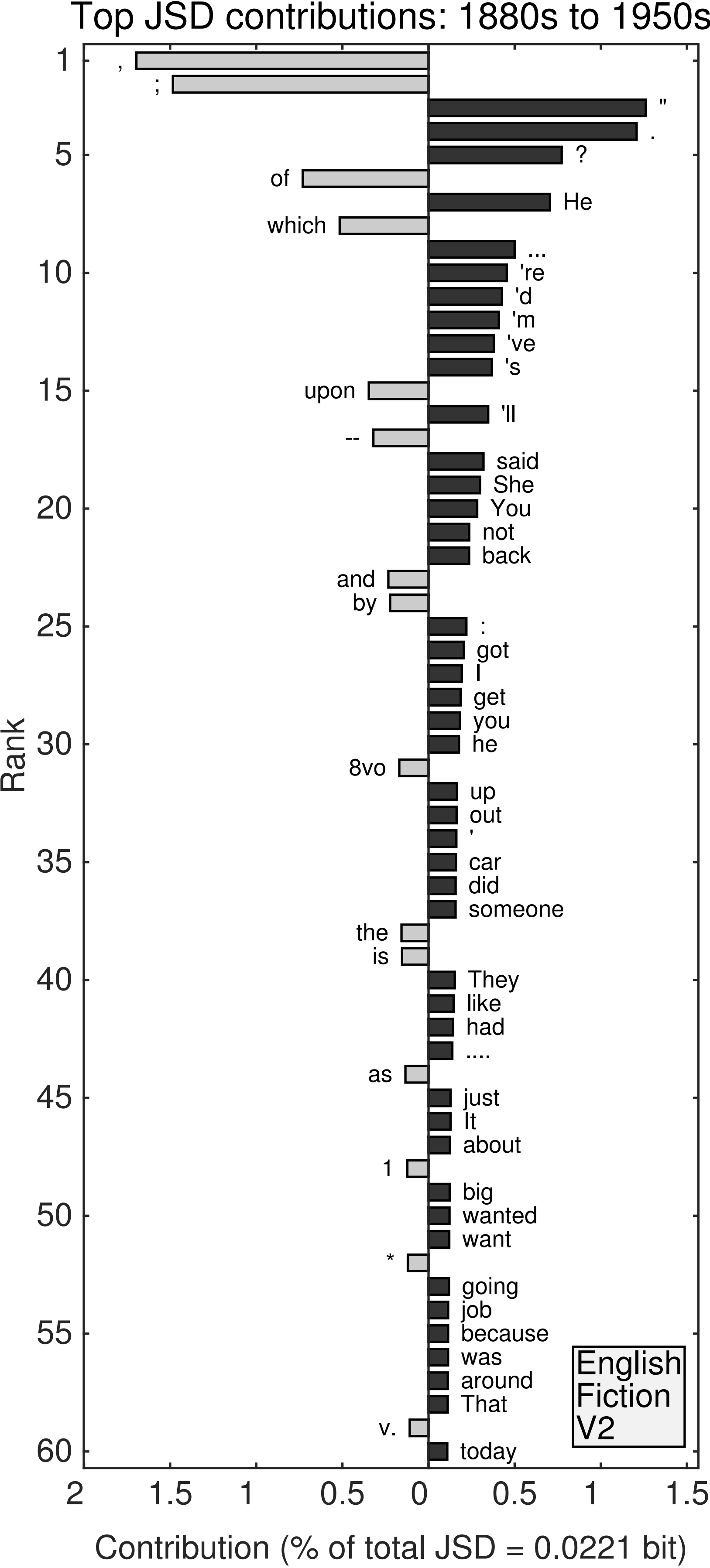
Top JSD contributions: 1880s to 1950s
Rank
Contribution (% of total JSD = 0.0221 bit)
English Fiction V2
,
;
"
.
?
of
He
which
...
're
'd
'm
've
's
upon
'll
--
said
She
You
not
back
and
by
:
got
I
get
you
he
8vo
up
out
'
car
did
someone
the
is
They
like
had
....
as
just
It
about
1
big
wanted
want
*
going
job
because
was
around
That
v.
today
1
5
10
15
20
25
30
35
40
45
50
55
60
2
1.5
1
0.5
0
0.5
1
1.5

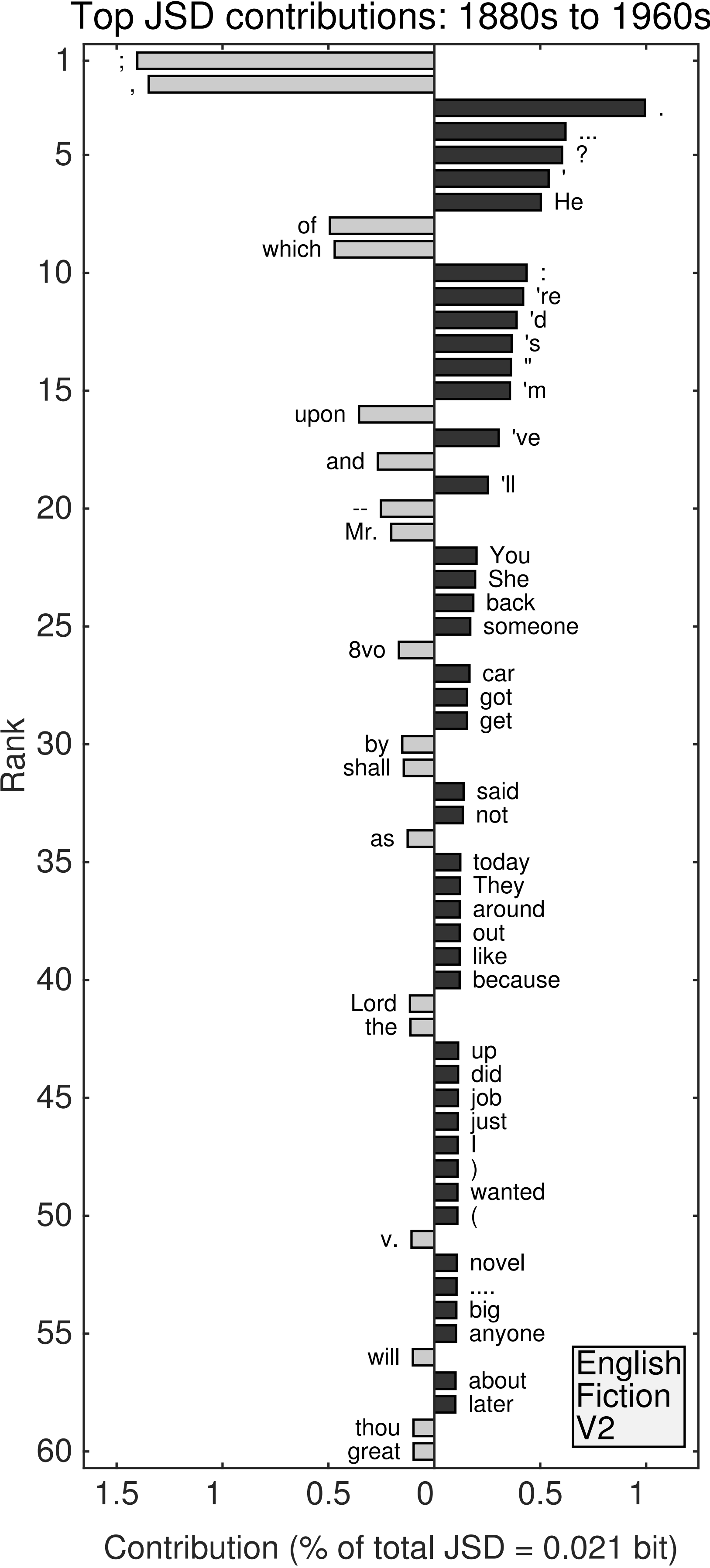

Top JSD contributions: 1880s to 1960s
Rank
Contribution (% of total JSD = 0.021 bit)
1.5
1
0.5
0
0.5
1
1
5
10
15
20
25
30
35
40
45
50
55
60
;
,
.
...
?
'
He
of
which
:
're
'd
's
"
'm
upon
've
and
'll
--
Mr.
You
She
back
someone
8vo
car
got
get
by
shall
said
not
as
today
They
around
out
like
because
Lord
the
up
did
job
just
I
)
wanted
(
v.
novel
....
big
anyone
will
about
later
thou
great
English
Fiction
V2

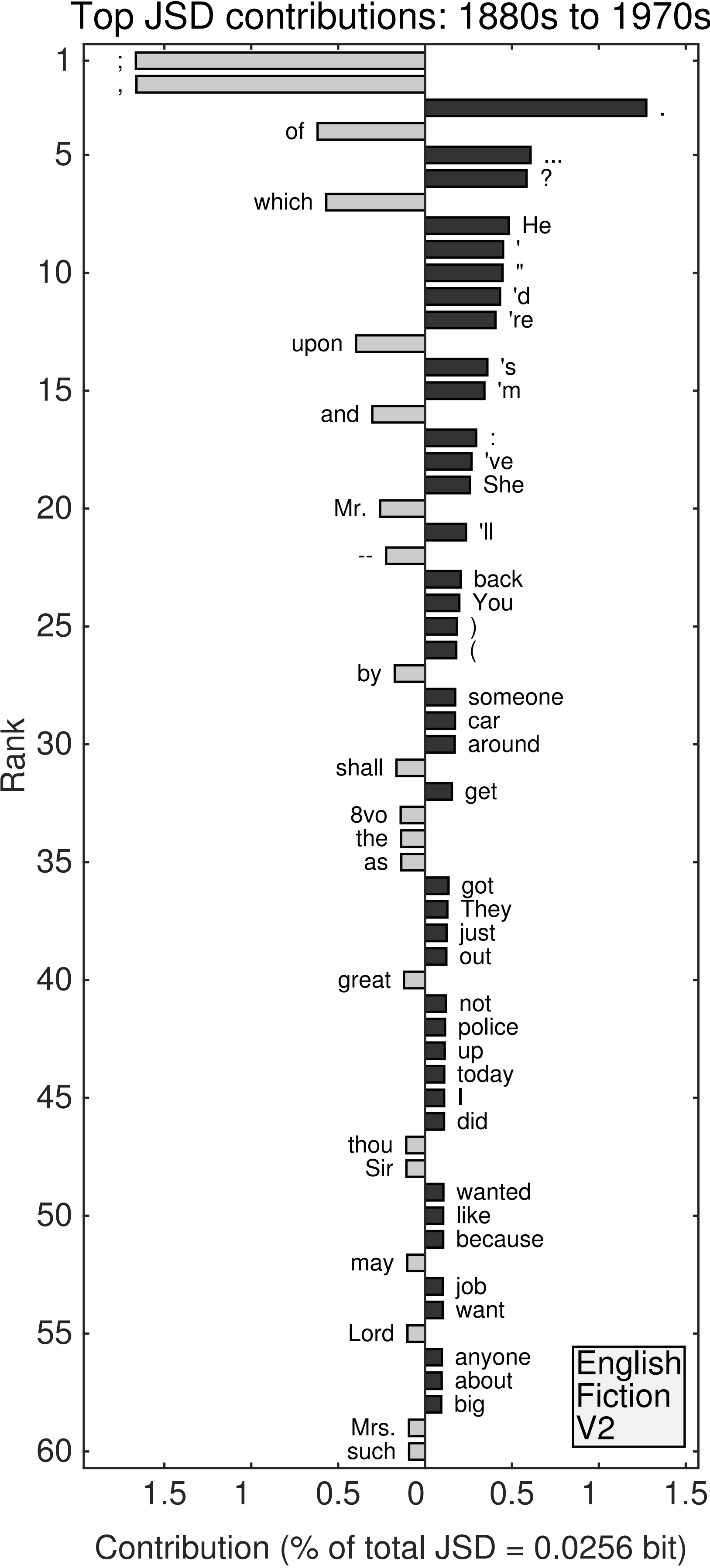
Top JSD contributions: 1880s to 1970s
Rank
Contribution (% of total JSD = 0.0256 bit)
English Fiction V2
;
,
.
of
...
?
which
He
'
"
'd
're
upon
's
'm
and
:
've
She
Mr.
'll
--
back
You
)
(
by
someone
car
around
shall
get
8vo
the
as
got
They
just
out
great
not
police
up
today
I
did
thou
Sir
wanted
like
because
may
job
want
Lord
anyone
about
big
Mrs.
such
1
5
10
15
20
25
30
35
40
45
50
55
60
1.5
1
0.5
0
0.5
1
1.5

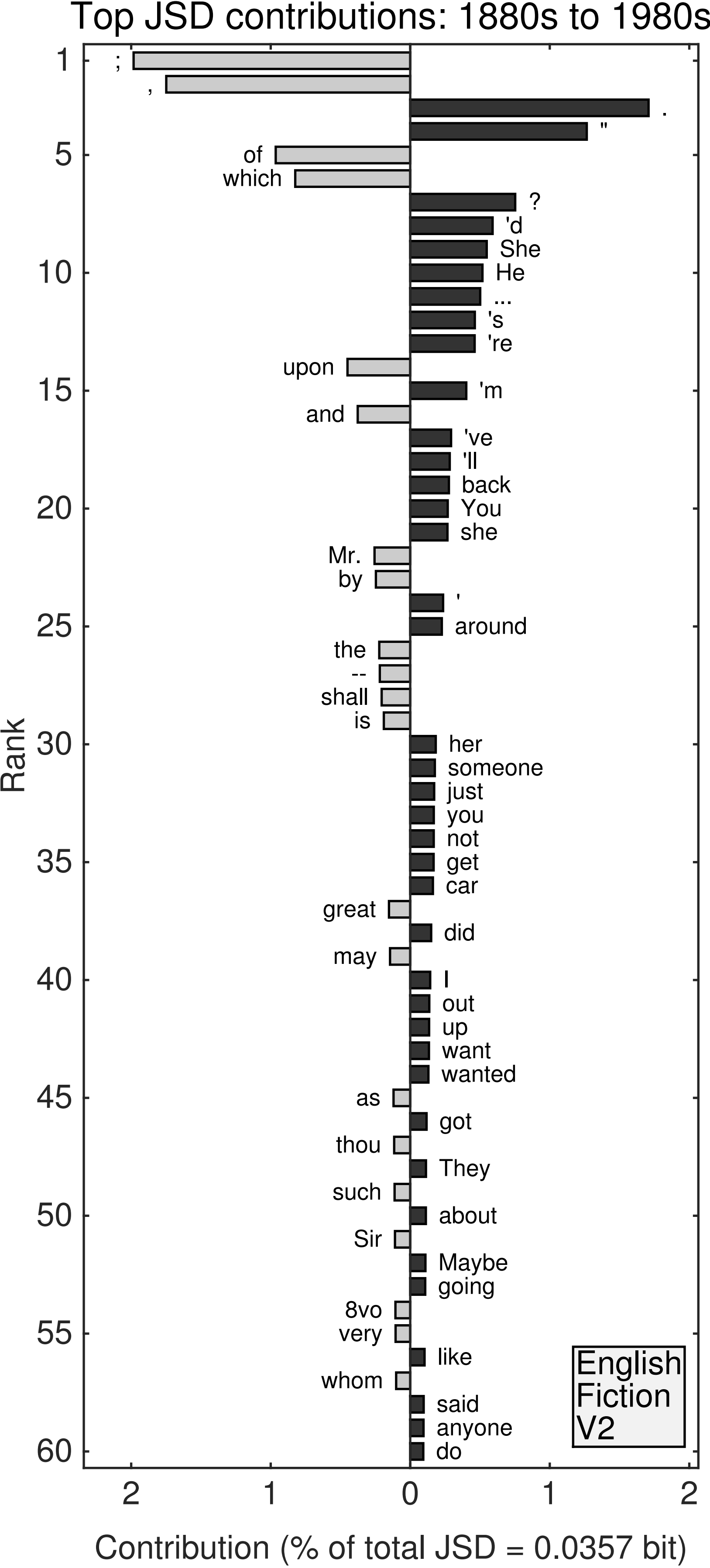
Top JSD contributions: 1880s to 1980s
Rank
1
5
10
15
20
25
30
35
40
45
50
55
60
;
,
.
"
of
which
?
'd
She
He
...
's
're
upon
'm
and
've
'll
back
You
she
Mr.
by
'
around
the
--
shall
is
her
someone
just
you
not
get
car
great
did
may
I
out
up
want
wanted
as
got
thou
They
such
about
Sir
Maybe
going
8vo
very
like
whom
said
anyone
do
English Fiction V2
2
1
0
1
2
Contribution (% of total JSD = 0.0357 bit)

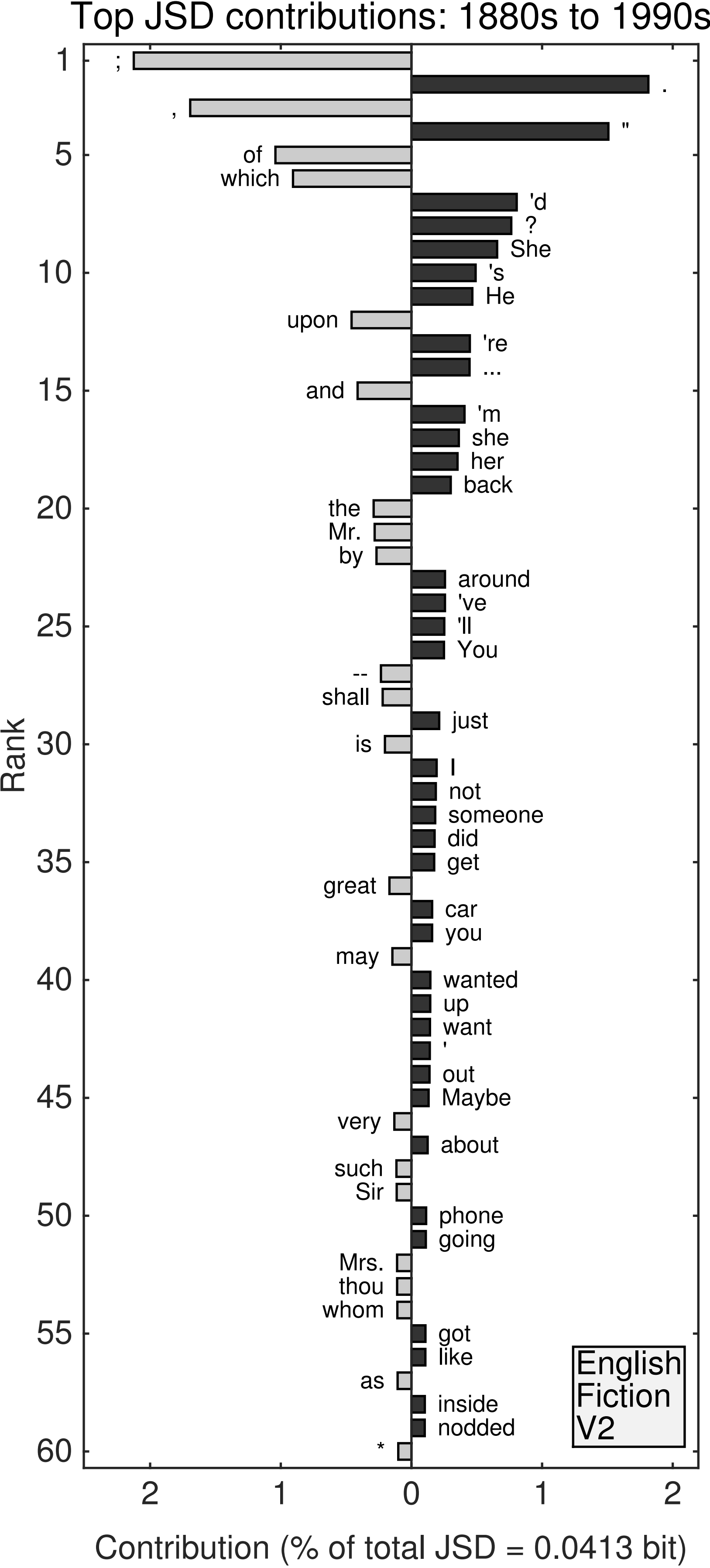
Top JSD contributions: 1880s to 1990s
Rank
Contribution (% of total JSD = 0.0413 bit)
English Fiction V2
;
.
,
"
of
which
'd
?
She
's
He
upon
're
...
and
'm
she
her
back
the
Mr.
by
around
've
'll
You
--
shall
just
is
I
not
someone
did
get
great
car
you
may
wanted
up
want
'
out
Maybe
very
about
such
Sir
phone
going
Mrs.
thou
whom
got
like
as
inside
nodded
*
1
5
10
15
20
25
30
35
40
45
50
55
60
2
1
0
1
2

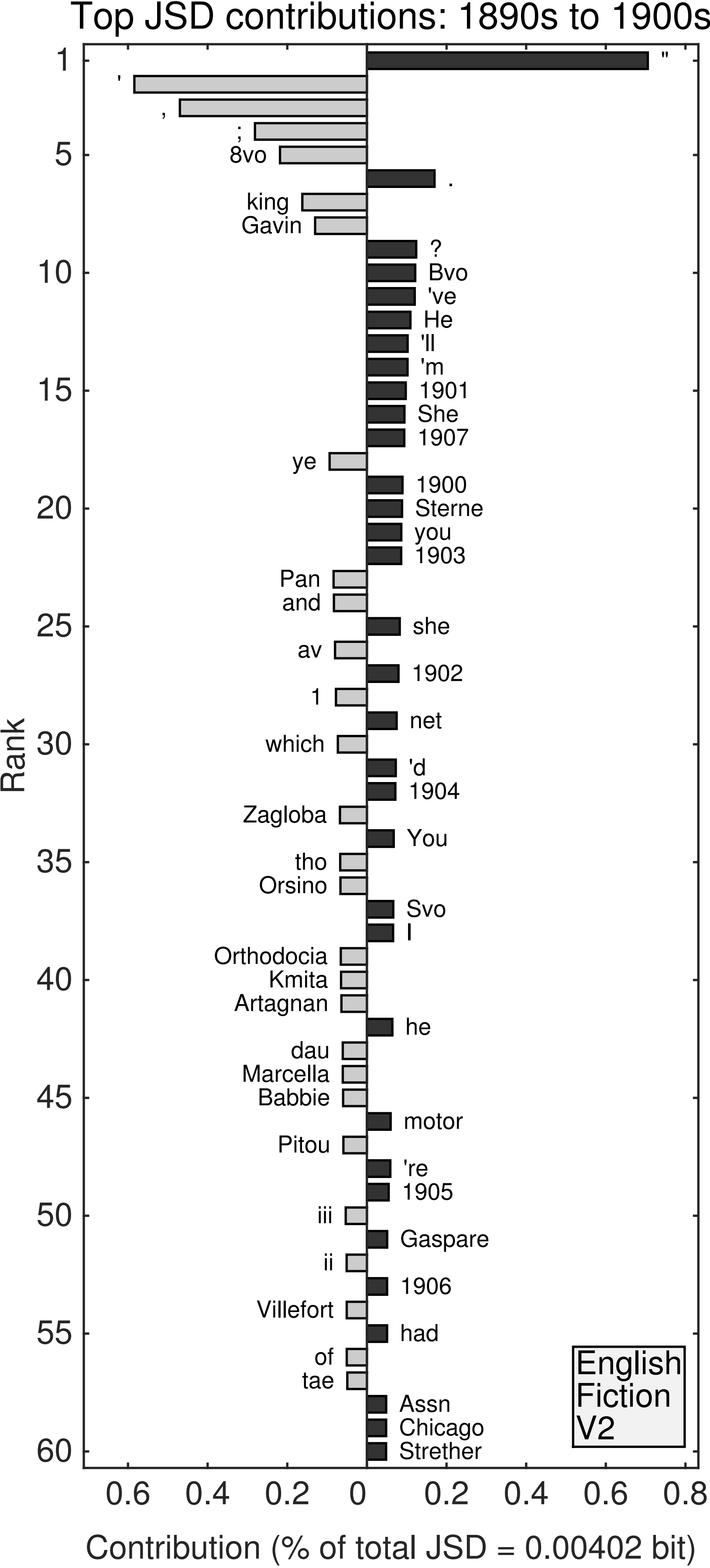

Top JSD contributions: 1890s to 1900s
Rank
Contribution (% of total JSD = 0.00402 bit)
English Fiction V2
"
'
,
;
8vo
.
king
Gavin
?
Bvo
've
He
'll
'm
1901
She
1907
ye
1900
Sterne
you
1903
Pan
and
she
av
1902
1
net
which
'd
1904
Zagloba
You
tho
Orsino
Svo
I
Orthodocia
Kmita
Artagnan
he
dau
Marcella
Babbie
motor
Pitou
're
1905
iii
Gaspare
ii
1906
Villefort
had
of
tae
Assn
Chicago
Strether

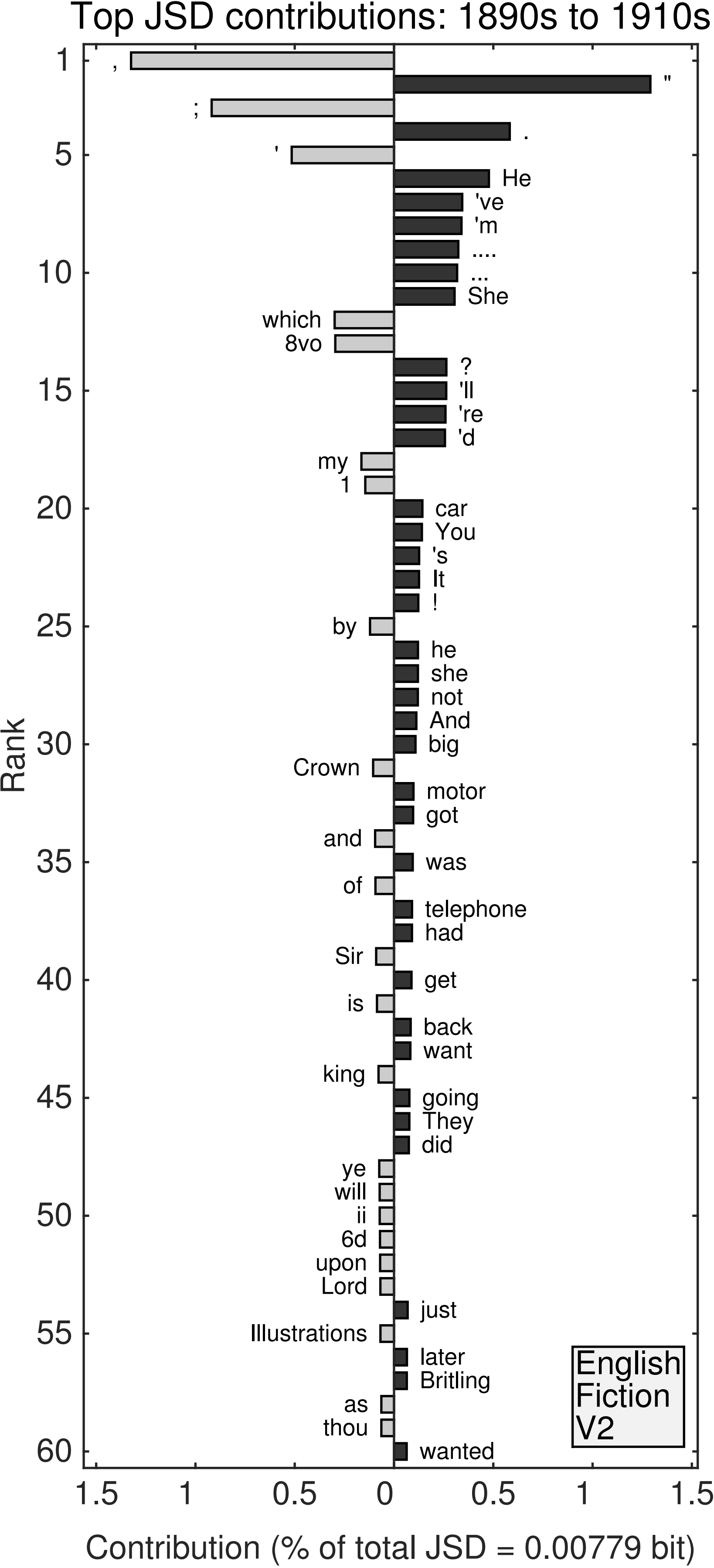

Top JSD contributions: 1890s to 1910s
Rank
Contribution (% of total JSD = 0.00779 bit)
,
"
;
.
'
He
've
'm
....
...
She
which
8vo
?
'll
're
'd
my
1
car
You
's
It
!
by
he
she
not
And
big
Crown
motor
got
and
was
of
telephone
had
Sir
get
is
back
want
king
going
They
did
ye
will
ii
6d
upon
Lord
just
Illustrations
later
Britling
as
thou
wanted
English Fiction V2

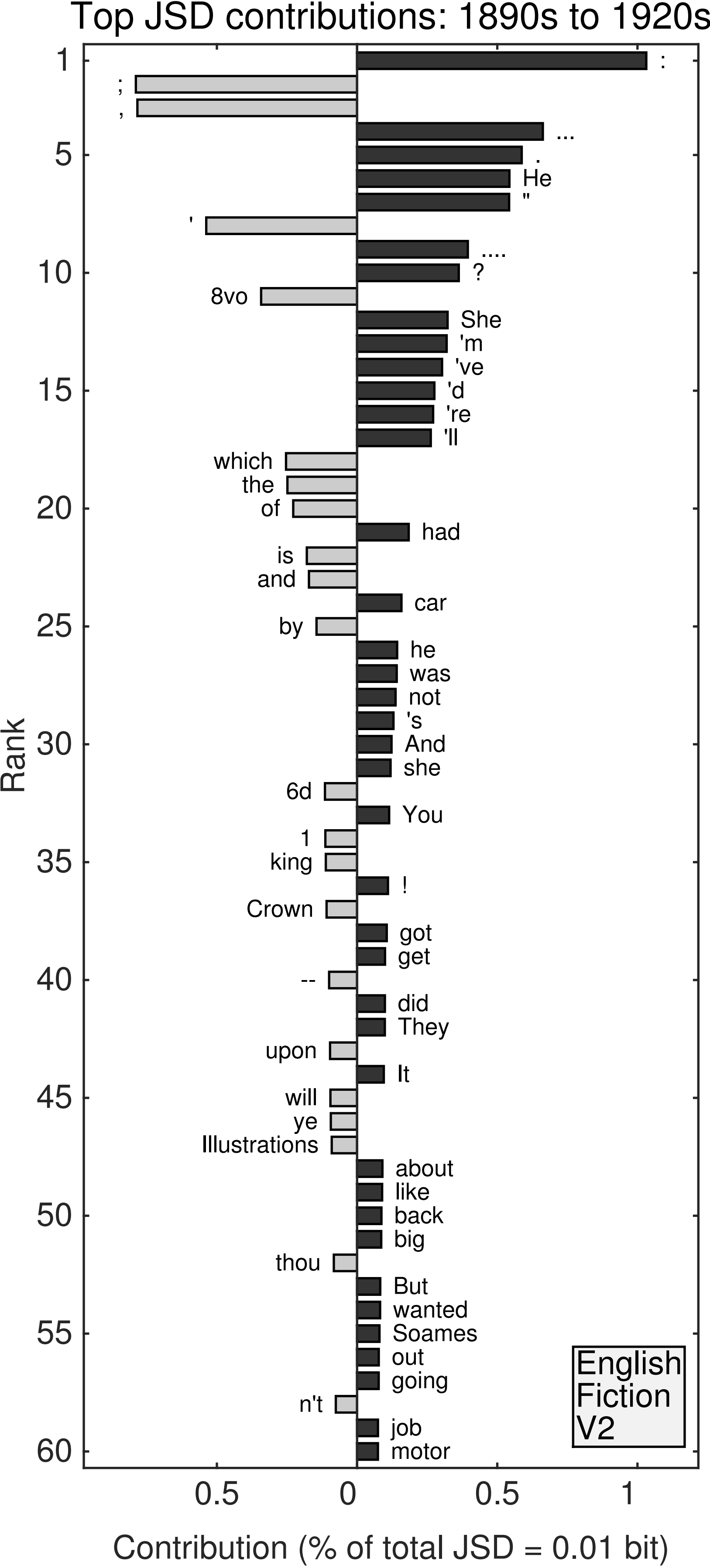

Top JSD contributions: 1890s to 1920s
Rank
Contribution (% of total JSD = 0.01 bit)
English Fiction V2
:
;
,
...
.
He
"
'
....
?
8vo
She
'm
've
'd
're
'll
which
the
of
had
is
and
car
by
he
was
not
's
And
she
6d
You
1
king
!
Crown
got
get
--
did
They
upon
It
will
ye
Illustrations
about
like
back
big
thou
But
wanted
Soames
out
going
n't
job
motor

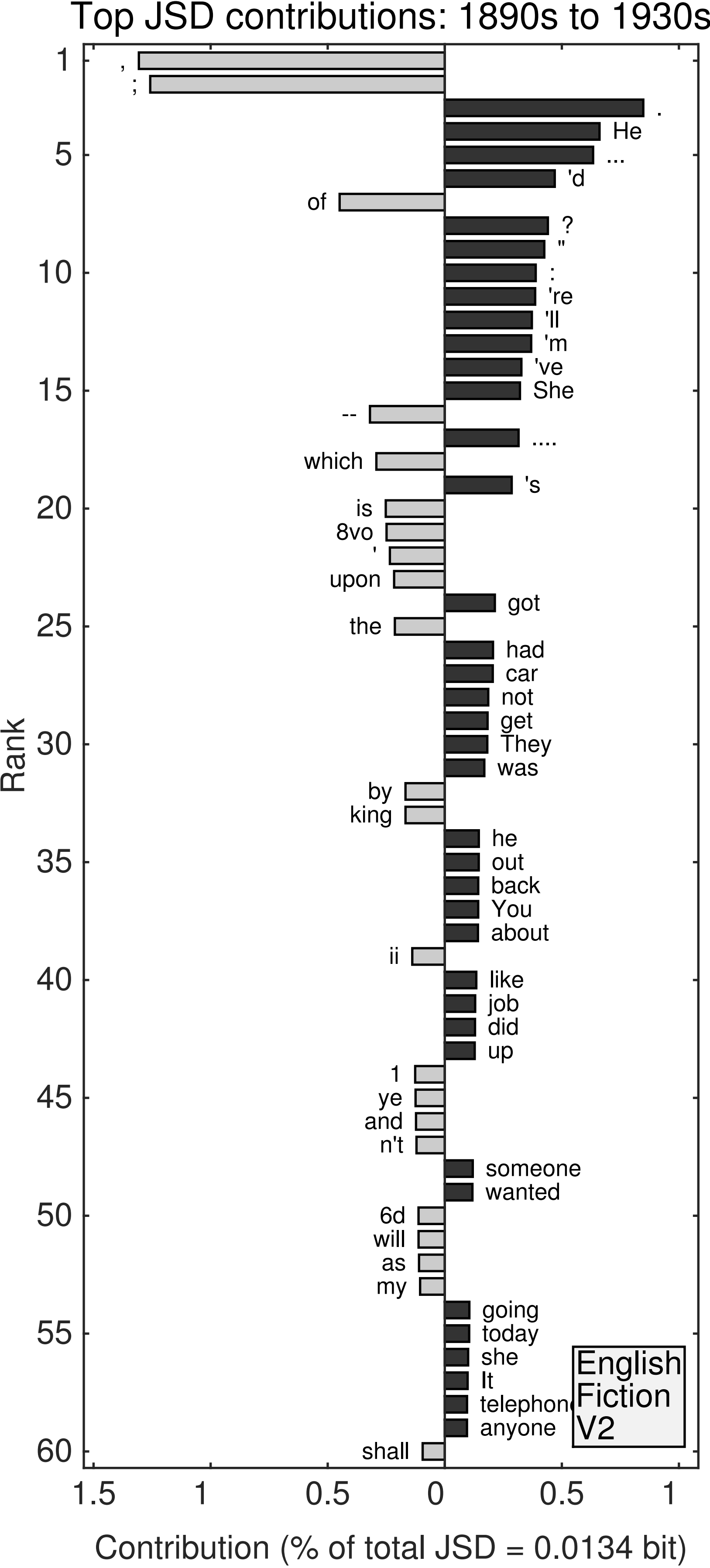
Top JSD contributions: 1890s to 1930s
Rank
Contribution (% of total JSD = 0.0134 bit)
English
Fiction
V2
,
;
.
He
...
'd
of
?
"
:
're
'll
'm
've
She
--
....
which
's
is
8vo
'
upon
got
the
had
car
not
get
They
was
by
king
he
out
back
You
about
ii
like
job
did
up
1
ye
and
n't
someone
wanted
6d
will
as
my
going
today
she
It
telephone
anyone
shall

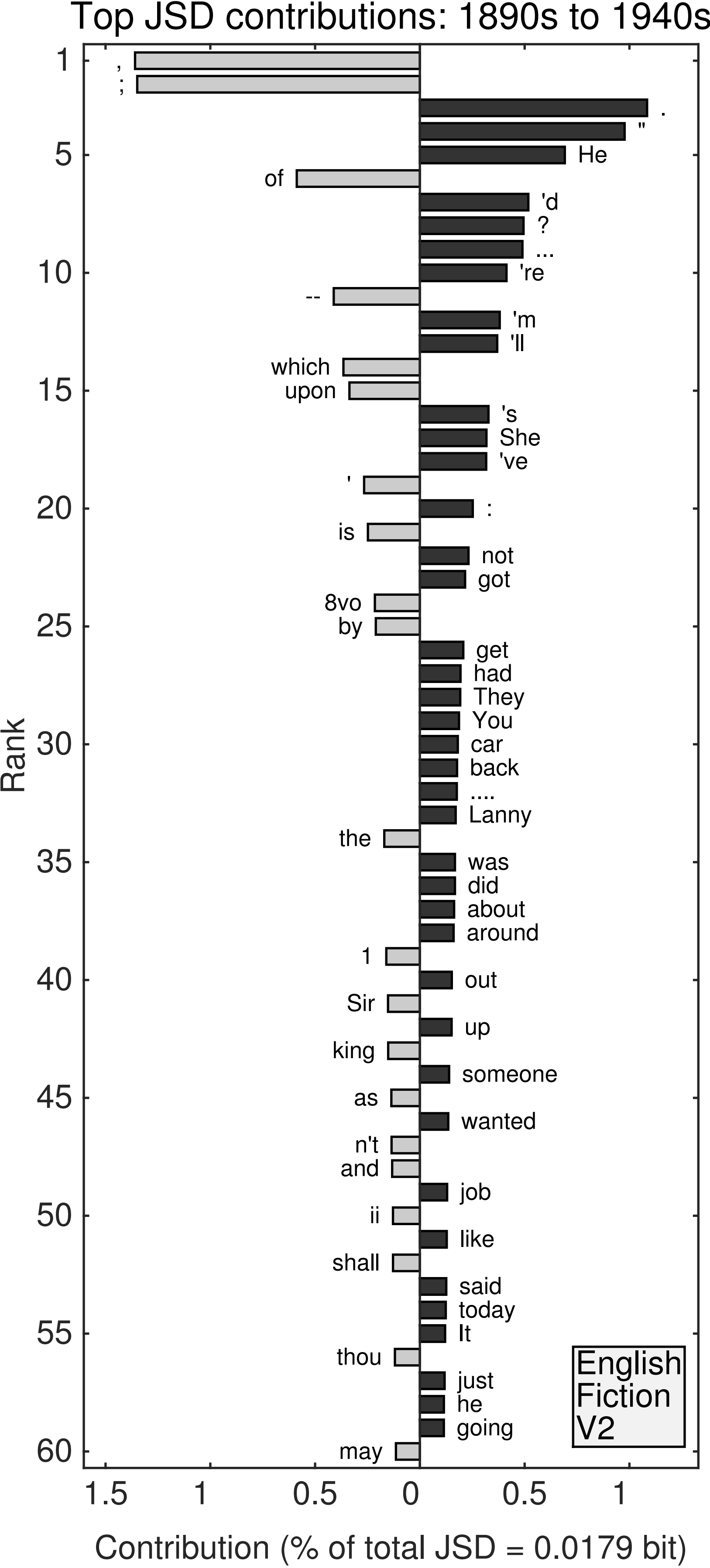

Top JSD contributions: 1890s to 1940s
Rank
1
5
10
15
20
25
30
35
40
45
50
55
60
,
;
.
"
He
of
'd
?
...
're
--
'm
'll
which
upon
's
She
've
'
:
is
not
got
8vo
by
get
had
They
You
car
back
....
Lanny
the
was
did
about
around
1
out
Sir
up
king
someone
as
wanted
n't
and
job
ii
like
shall
said
today
It
thou
just
he
going
may
English Fiction V2
1.5
1
0.5
0
0.5
1
Contribution (% of total JSD = 0.0179 bit)

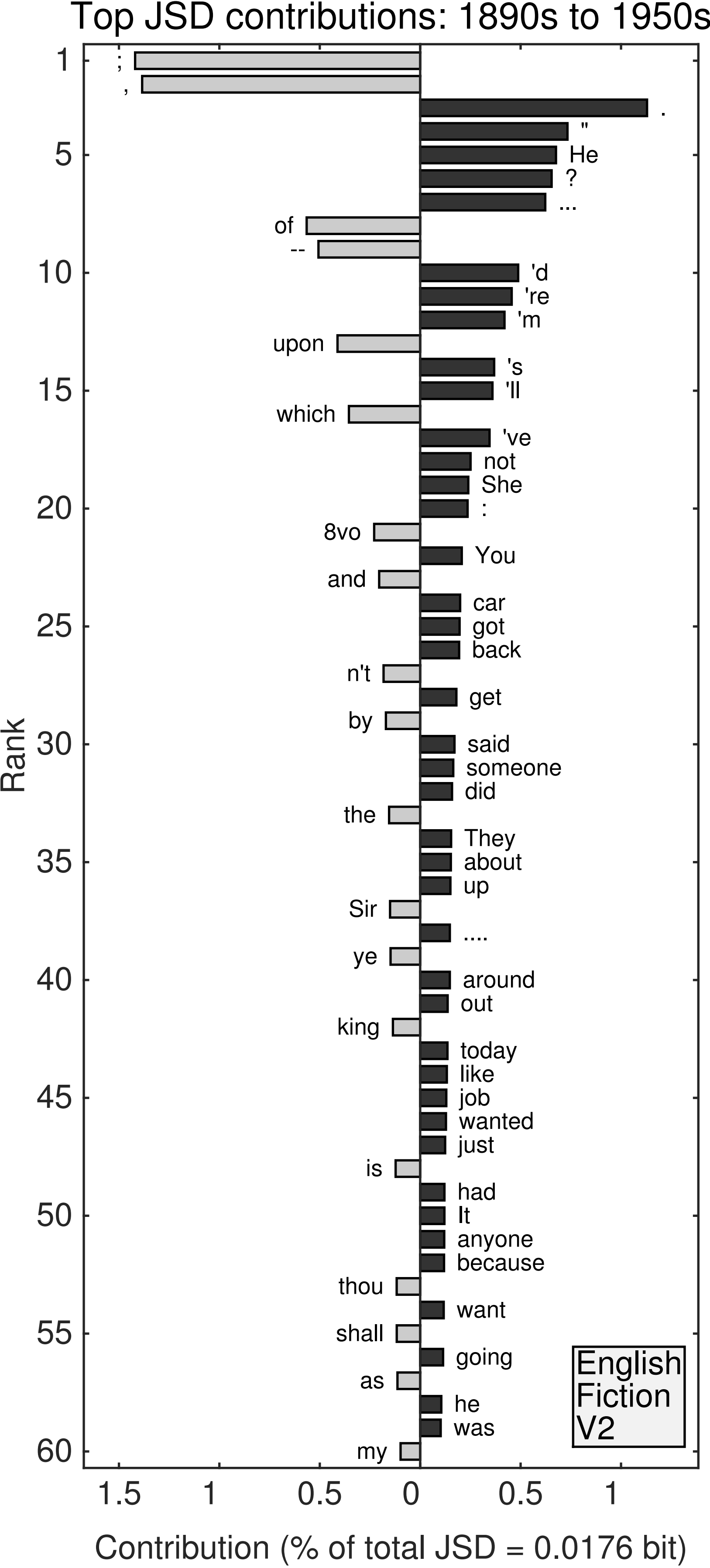

Top JSD contributions: 1890s to 1950s
Rank
Contribution (% of total JSD = 0.0176 bit)
English Fiction V2
;
,
.
"
He
?
...
of
--
'd
're
'm
upon
's
'll
which
've
not
She
:
8vo
You
and
car
got
back
n't
get
by
said
someone
did
the
They
about
up
Sir
....
ye
around
out
king
today
like
job
wanted
just
is
had
It
anyone
because
thou
want
shall
going
as
he
was
my

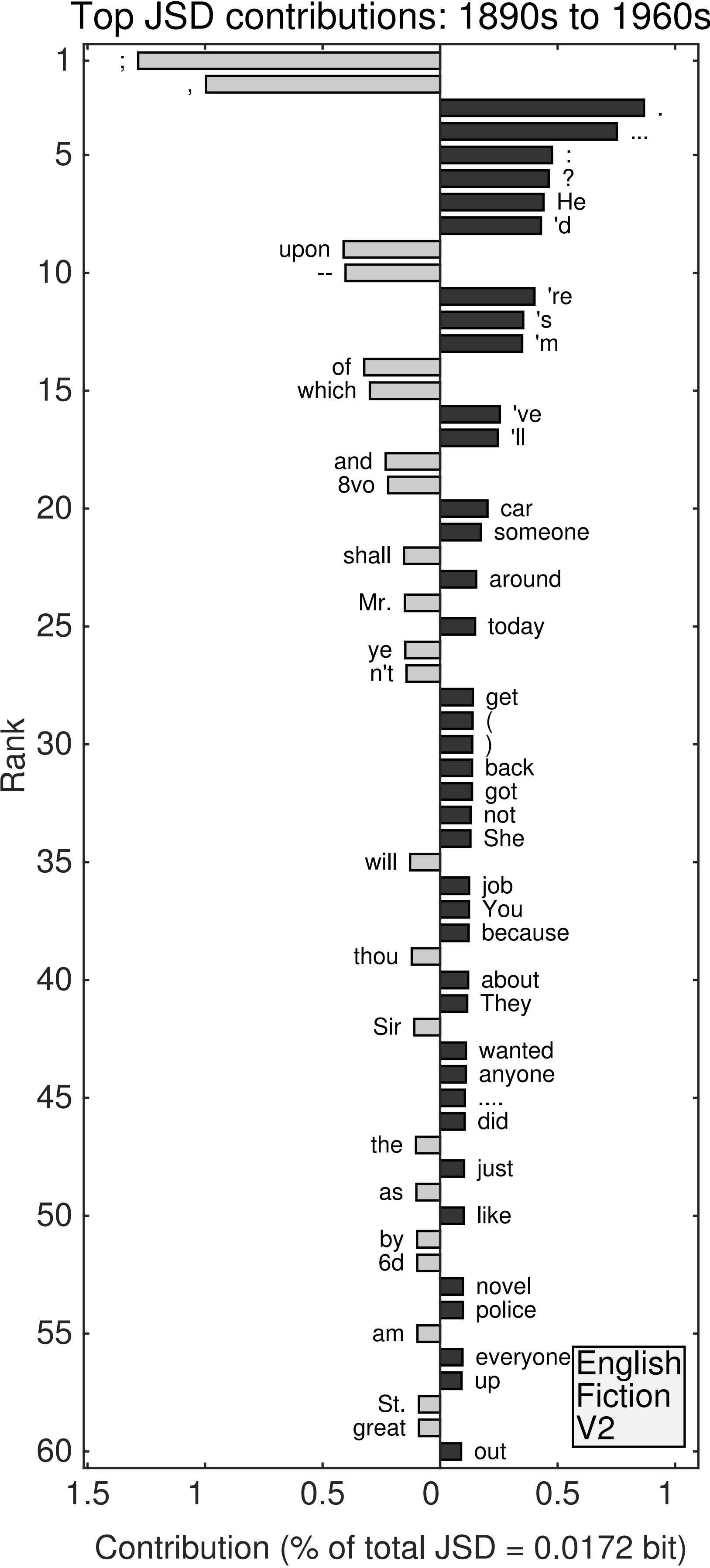
Top JSD contributions: 1890s to 1960s
Rank
Contribution (% of total JSD = 0.0172 bit)
English Fiction V2
;
,
.
...
:
?
He
'd
upon
--
're
's
'm
of
which
've
'll
and
8vo
car
someone
shall
around
Mr.
today
ye
n't
get
(
)
back
got
not
She
will
job
You
because
thou
about
They
Sir
wanted
anyone
....
did
the
just
as
like
by
6d
novel
police
am
everyone
up
St.
great
out

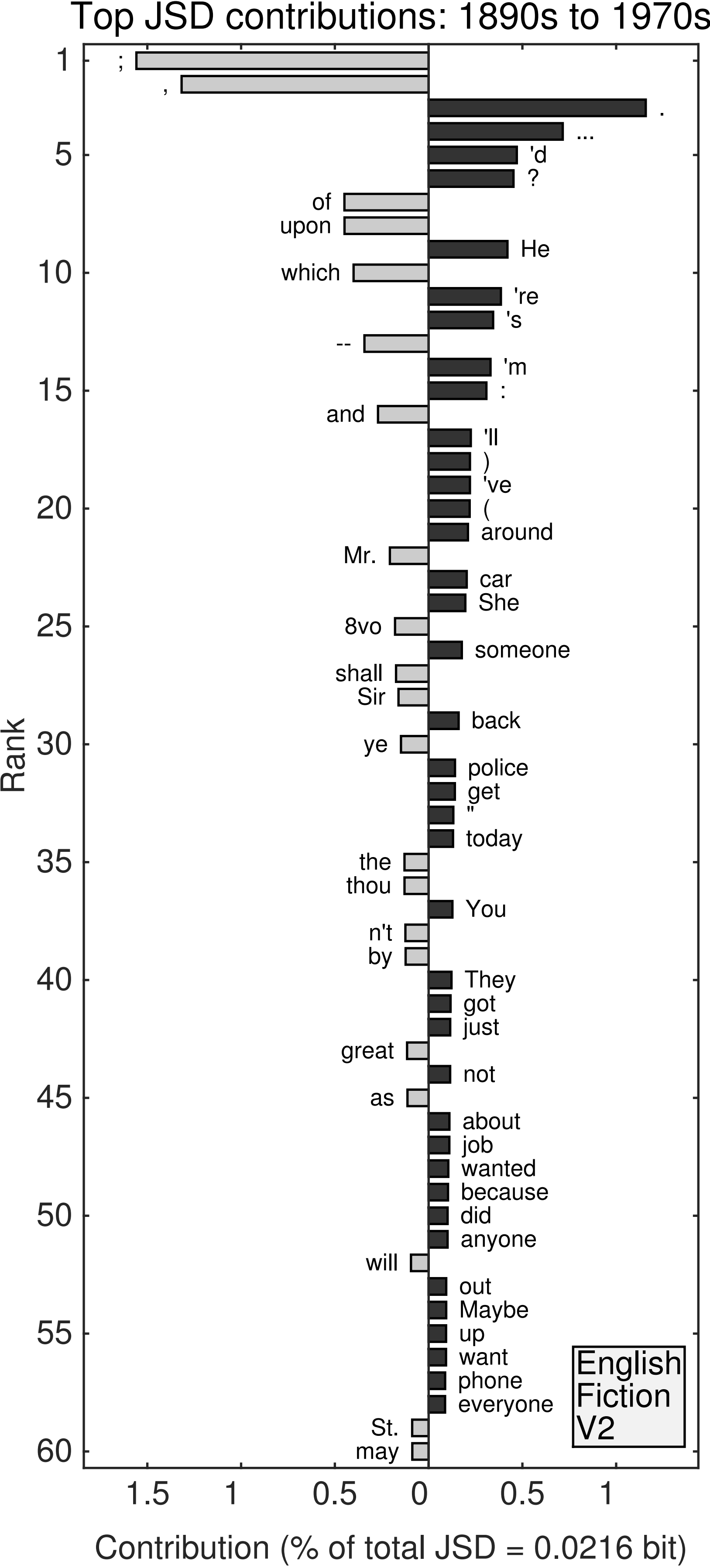

Top JSD contributions: 1890s to 1970s
Rank
Contribution (% of total JSD = 0.0216 bit)
1.5
1
0.5
0
0.5
1
5
10
15
20
25
30
35
40
45
50
55
60
;
,
.
...
'd
?
of
upon
He
which
're
's
--
'm
:
and
'll
)
've
(
around
Mr.
car
She
8vo
someone
shall
Sir
back
ye
police
get
"
today
the
thou
You
n't
by
They
got
just
great
not
as
about
job
wanted
because
did
anyone
will
out
Maybe
up
want
phone
everyone
St.
may
English
Fiction
V2

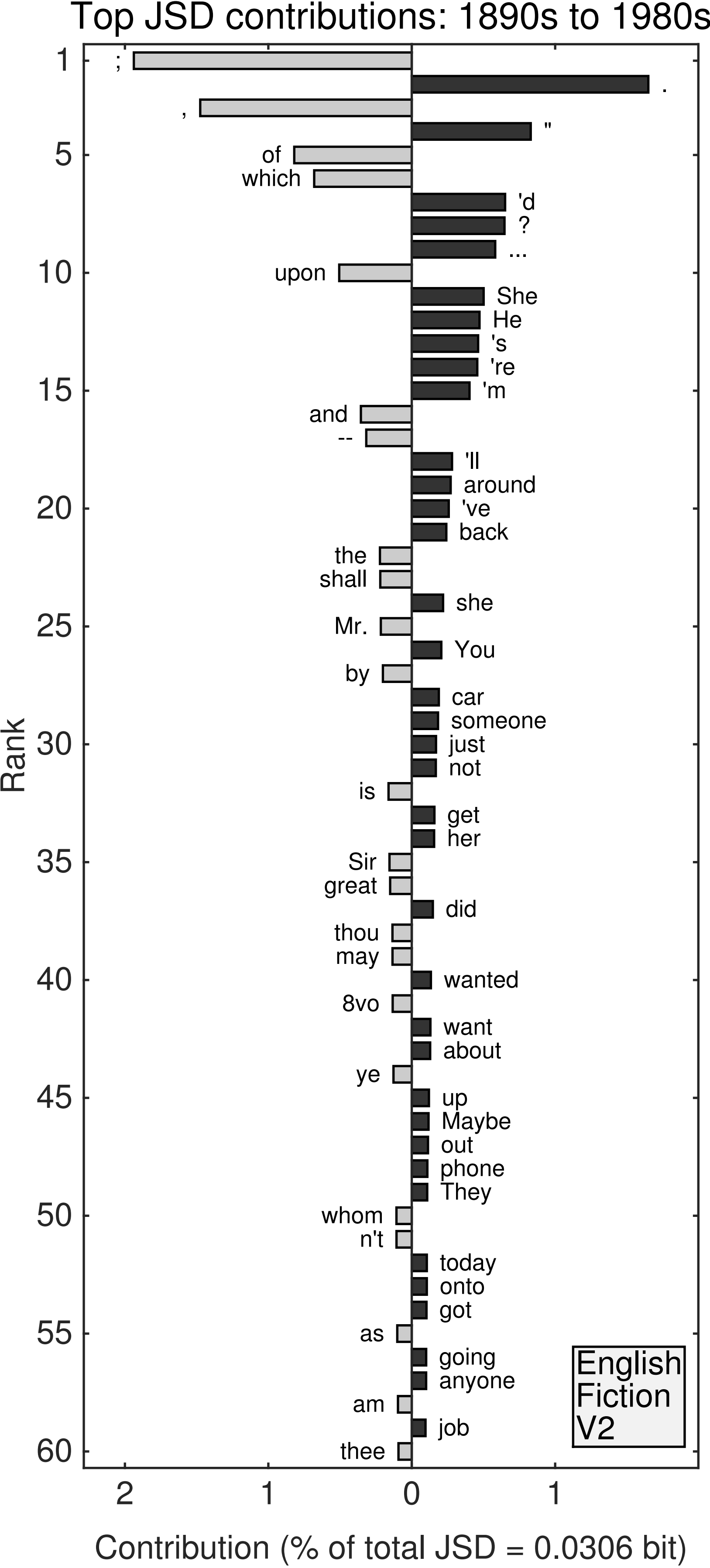

Top JSD contributions: 1890s to 1980s
Rank
Contribution (% of total JSD = 0.0306 bit)
English Fiction V2
;
.
,
"
of
which
'd
?
...
upon
She
He
's
're
'm
and
--
'll
around
've
back
the
shall
she
Mr.
You
by
car
someone
just
not
is
get
her
Sir
great
did
thou
may
wanted
8vo
want
about
ye
up
Maybe
out
phone
They
whom
n't
today
onto
got
as
going
anyone
am
job
thee

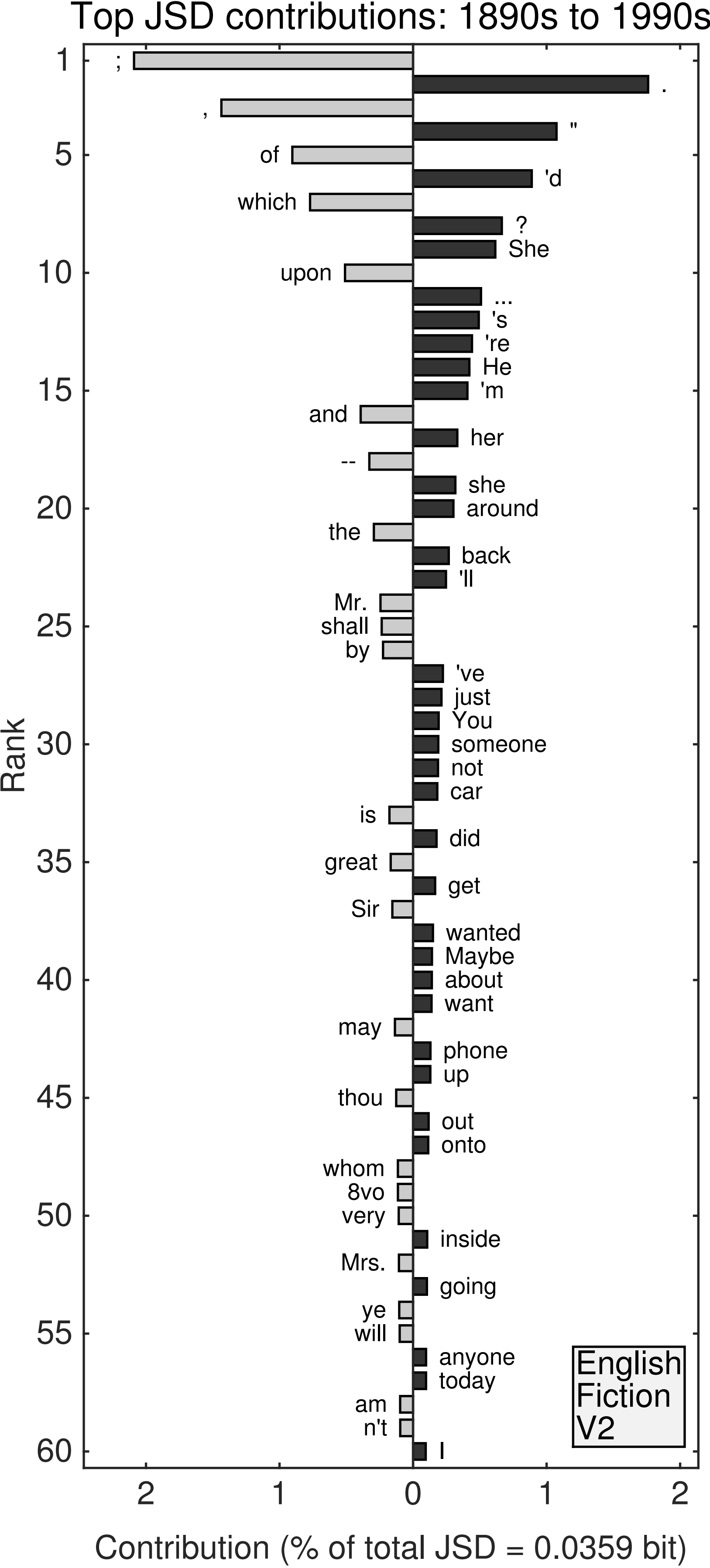
Top JSD contributions: 1890s to 1990s
Rank
Contribution (% of total JSD = 0.0359 bit)
English Fiction V2
;
.
,
"
of
'd
which
?
She
upon
...
's
're
He
'm
and
her
--
she
around
the
back
'll
Mr.
shall
by
've
just
You
someone
not
car
is
did
great
get
Sir
wanted
Maybe
about
want
may
phone
up
thou
out
onto
whom
8vo
very
inside
Mrs.
going
ye
will
anyone
today
am
n't
I

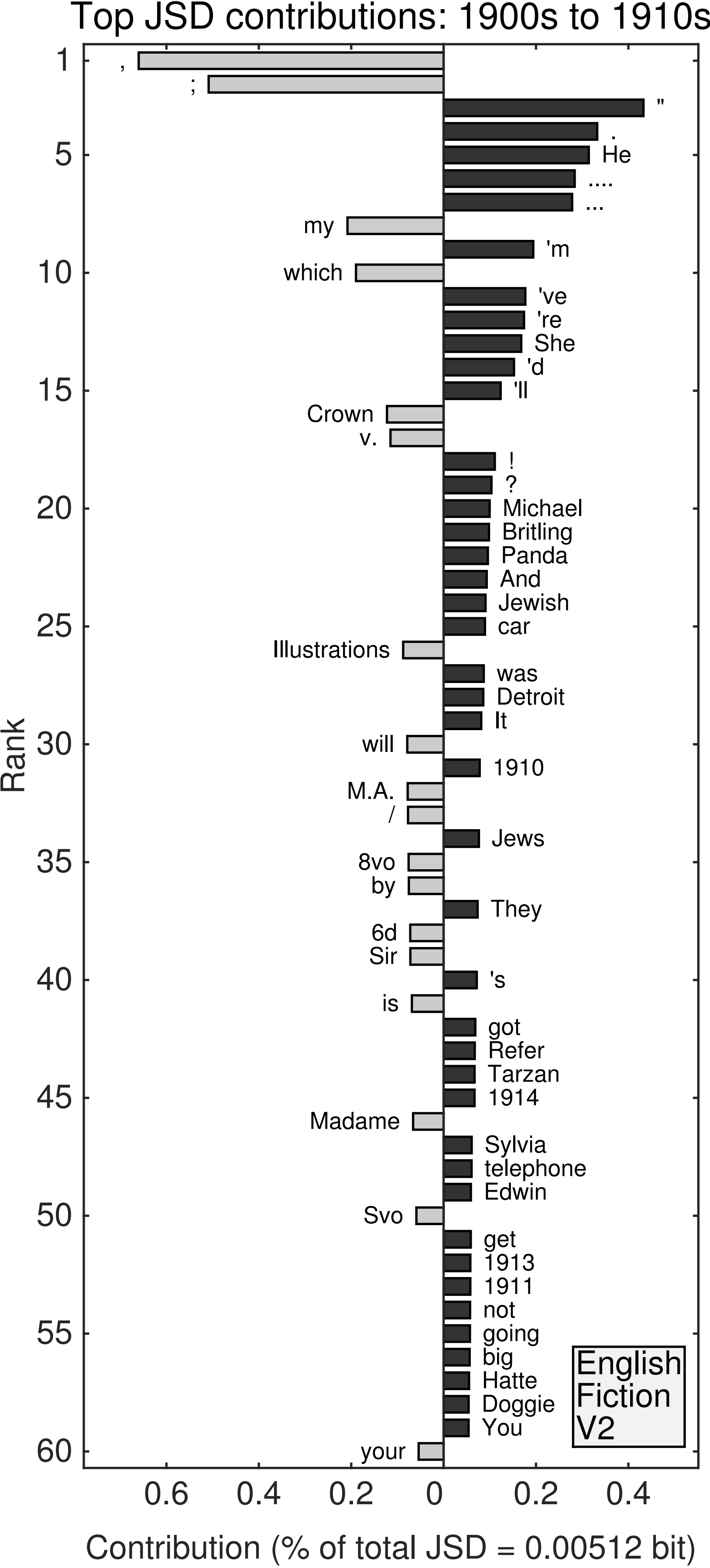
Top JSD contributions: 1900s to 1910s
Rank
Contribution (% of total JSD = 0.00512 bit)
,
;
"
.
He
....
...
my
'm
which
've
're
She
'd
'll
Crown
v.
!
?
Michael
Britling
Panda
And
Jewish
car
Illustrations
was
Detroit
It
will
1910
M.A.
/
Jews
8vo
by
They
6d
Sir
's
is
got
Refer
Tarzan
1914
Madame
Sylvia
telephone
Edwin
Svo
get
1913
1911
not
going
big
Hatte
Doggie
You
your
English Fiction V2

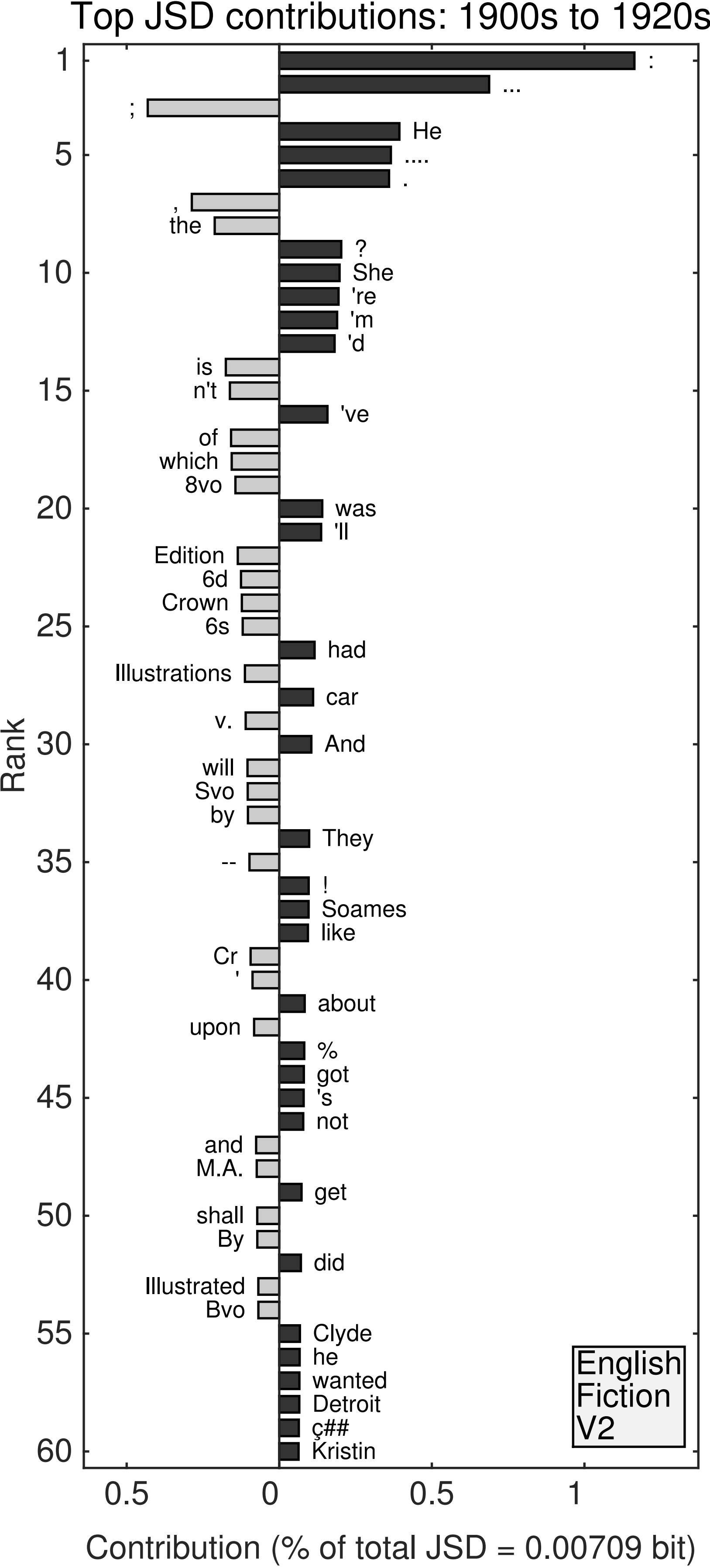

Top JSD contributions: 1900s to 1920s
Rank
Contribution (% of total JSD = 0.00709 bit)
0.5
0
0.5
1
1
5
10
15
20
25
30
35
40
45
50
55
60
:
...
;
He
....
.
,
the
?
She
're
'm
'd
is
n't
've
of
which
8vo
was
'll
Edition
6d
Crown
6s
had
Illustrations
car
v.
And
will
Svo
by
They
--
!
Soames
like
Cr
'
about
upon
%
got
's
not
and
M.A.
get
shall
By
did
Illustrated
Bvo
Clyde
he
wanted
Detroit
ç##
Kristin
English
Fiction
V2

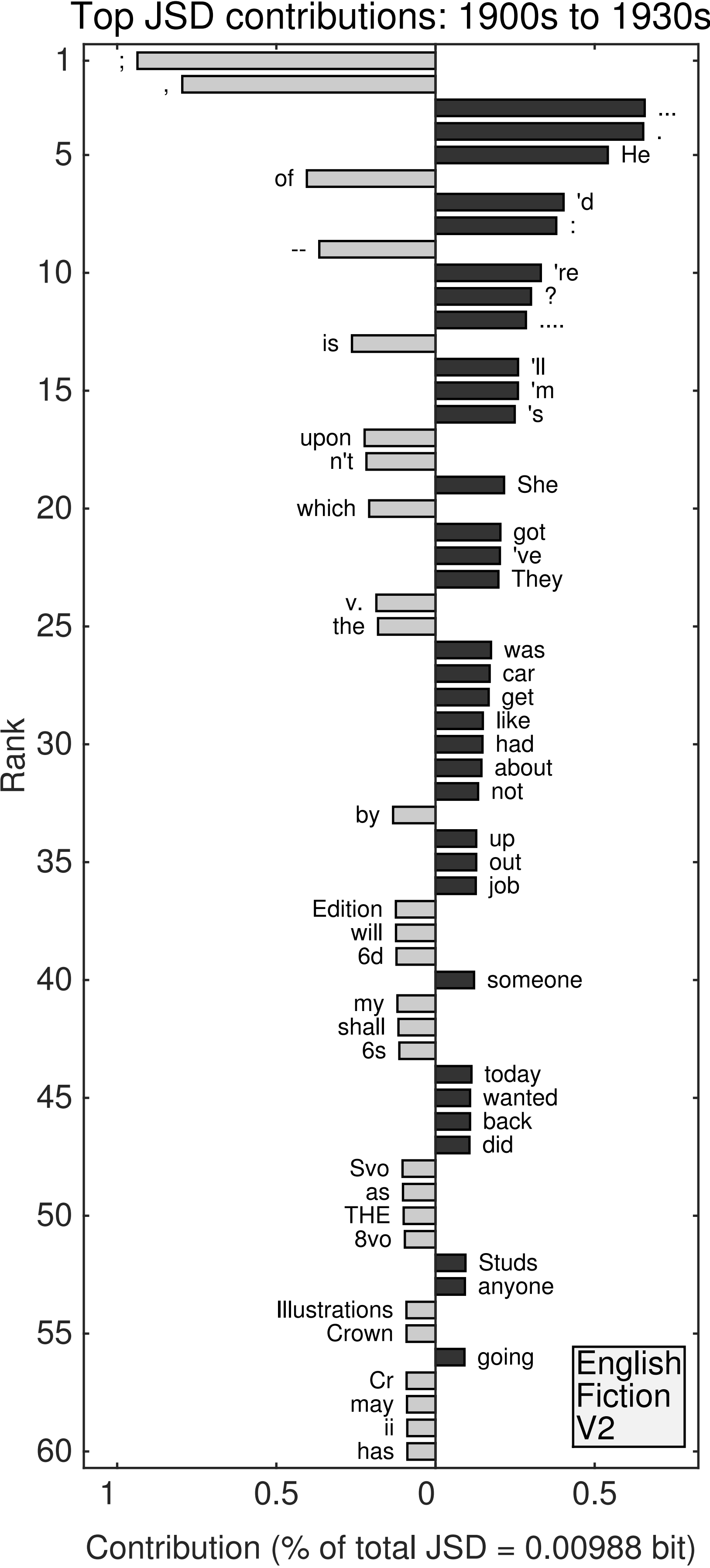

Top JSD contributions: 1900s to 1930s
Rank
Contribution (% of total JSD = 0.00988 bit)
English Fiction V2
;
,
...
.
He
of
'd
:
--
're
?
....
is
'll
'm
's
upon
n't
She
which
got
've
They
v.
the
was
car
get
like
had
about
not
by
up
out
job
Edition
will
6d
someone
my
shall
6s
today
wanted
back
did
Svo
as
THE
8vo
Studs
anyone
Illustrations
Crown
going
Cr
may
ii
has

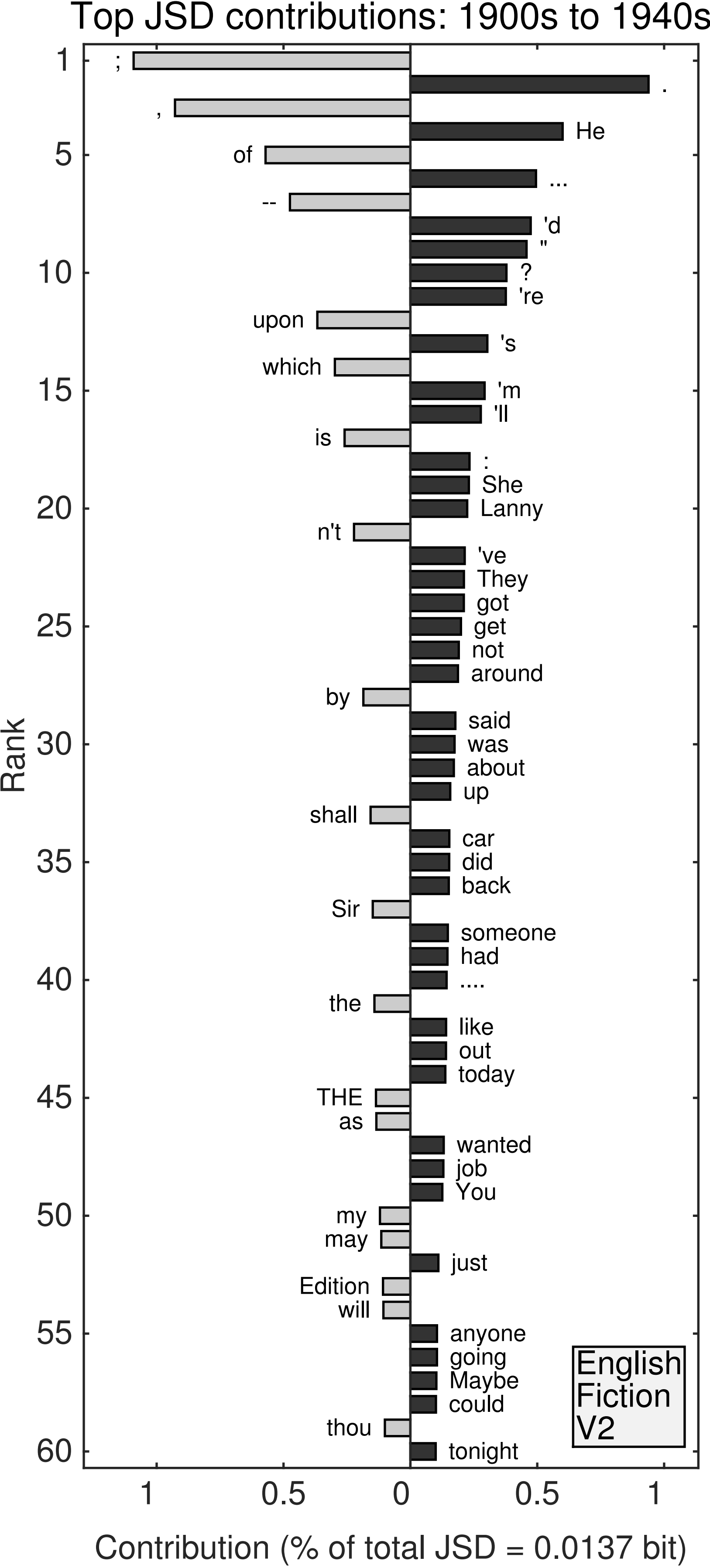
Top JSD contributions: 1900s to 1940s
Rank
Contribution (% of total JSD = 0.0137 bit)
;
.
,
He
of
...
--
'd
"
?
're
upon
's
which
'm
'll
is
:
She
Lanny
n't
've
They
got
get
not
around
by
said
was
about
up
shall
car
did
back
Sir
someone
had
....
the
like
out
today
THE
as
wanted
job
You
my
may
just
Edition
will
anyone
going
Maybe
could
thou
tonight
English
Fiction
V2

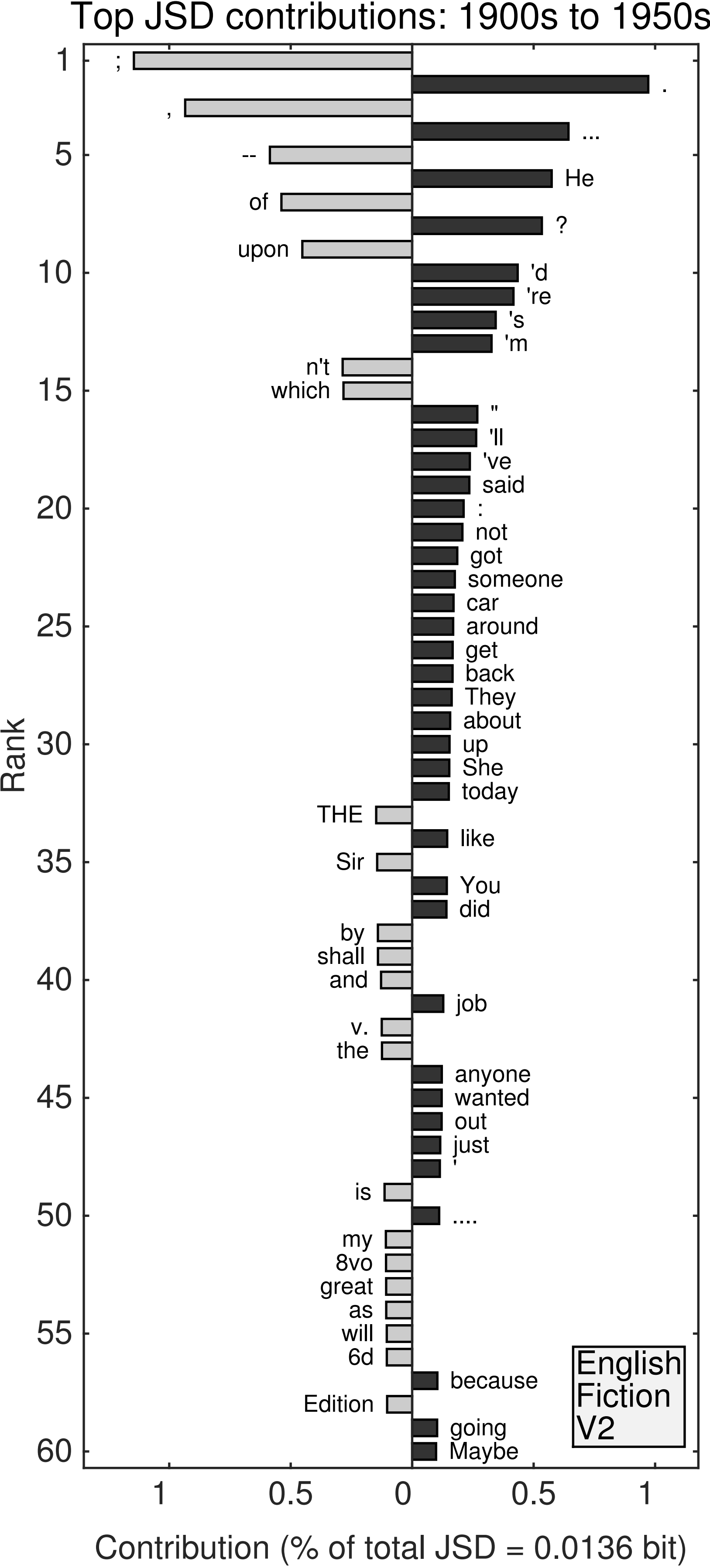
Top JSD contributions: 1900s to 1950s
Rank
Contribution (% of total JSD = 0.0136 bit)
English Fiction V2
;
.
,
...
--
He
of
?
upon
'd
're
's
'm
n't
which
"
'll
've
said
:
not
got
someone
car
around
get
back
They
about
up
She
today
THE
like
Sir
You
did
by
shall
and
job
v.
the
anyone
wanted
out
just
'
is
....
my
8vo
great
as
will
6d
because
Edition
going
Maybe

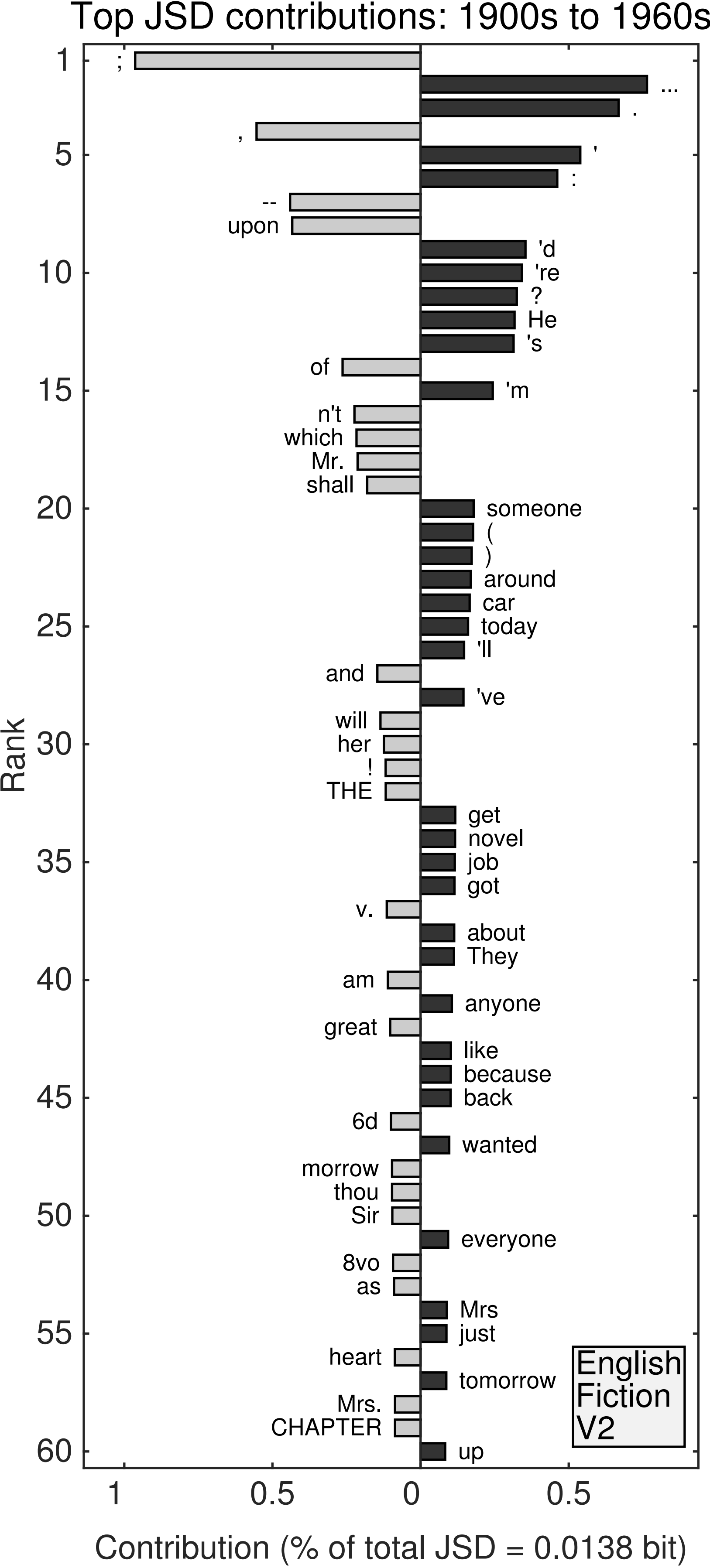
Top JSD contributions: 1900s to 1960s
Rank
Contribution (% of total JSD = 0.0138 bit)
English Fiction V2
;
...
.
,
'
:
--
upon
'd
're
?
He
's
of
'm
n't
which
Mr.
shall
someone
(
)
around
car
today
'll
and
've
will
her
!
THE
get
novel
job
got
v.
about
They
am
anyone
great
like
because
back
6d
wanted
morrow
thou
Sir
everyone
8vo
as
Mrs
just
heart
tomorrow
Mrs.
CHAPTER
up

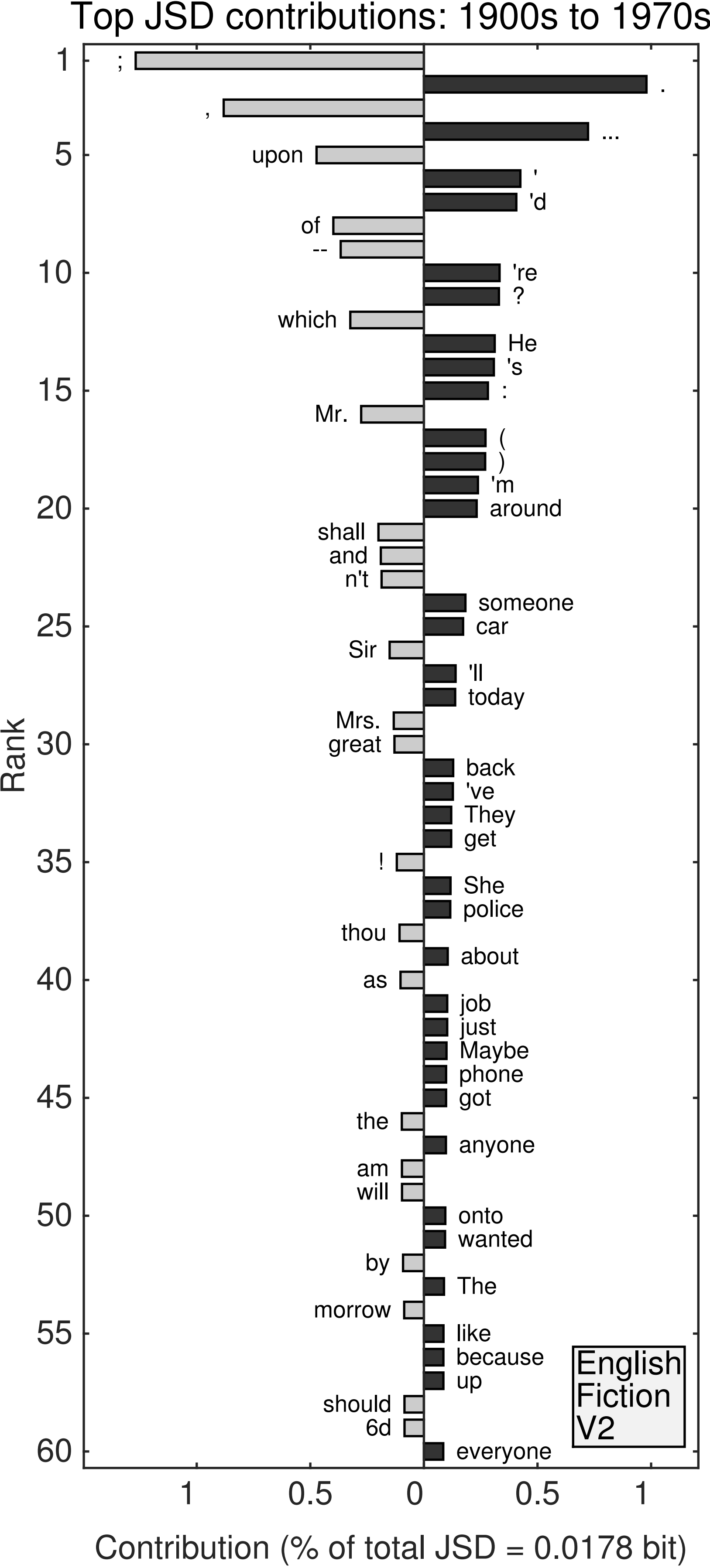
Top JSD contributions: 1900s to 1970s
Rank
Contribution (% of total JSD = 0.0178 bit)
English Fiction V2
;
.
,
...
upon
'
'd
of
--
're
?
which
He
's
:
Mr.
(
)
'm
around
shall
and
n't
someone
car
Sir
'll
today
Mrs.
great
back
've
They
get
!
She
police
thou
about
as
job
just
Maybe
phone
got
the
anyone
am
will
onto
wanted
by
The
morrow
like
because
up
should
6d
everyone

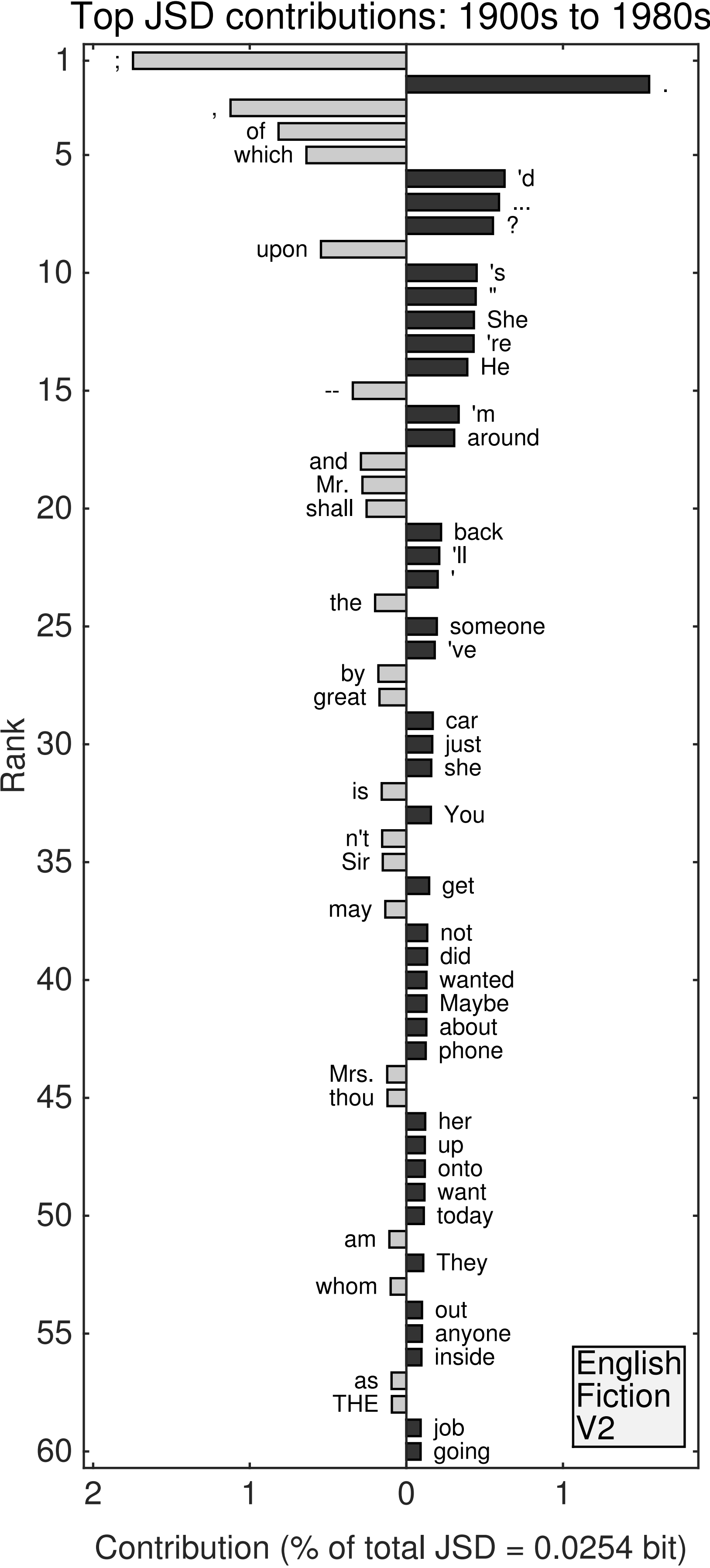

Top JSD contributions: 1900s to 1980s
Rank
Contribution (% of total JSD = 0.0254 bit)
English Fiction V2
;
.
,
of
which
'd
...
?
upon
's
"
She
're
He
--
'm
around
and
Mr.
shall
back
'll
'
the
someone
've
by
great
car
just
she
is
You
n't
Sir
get
may
not
did
wanted
Maybe
about
phone
Mrs.
thou
her
up
onto
want
today
am
They
whom
out
anyone
inside
as
THE
job
going

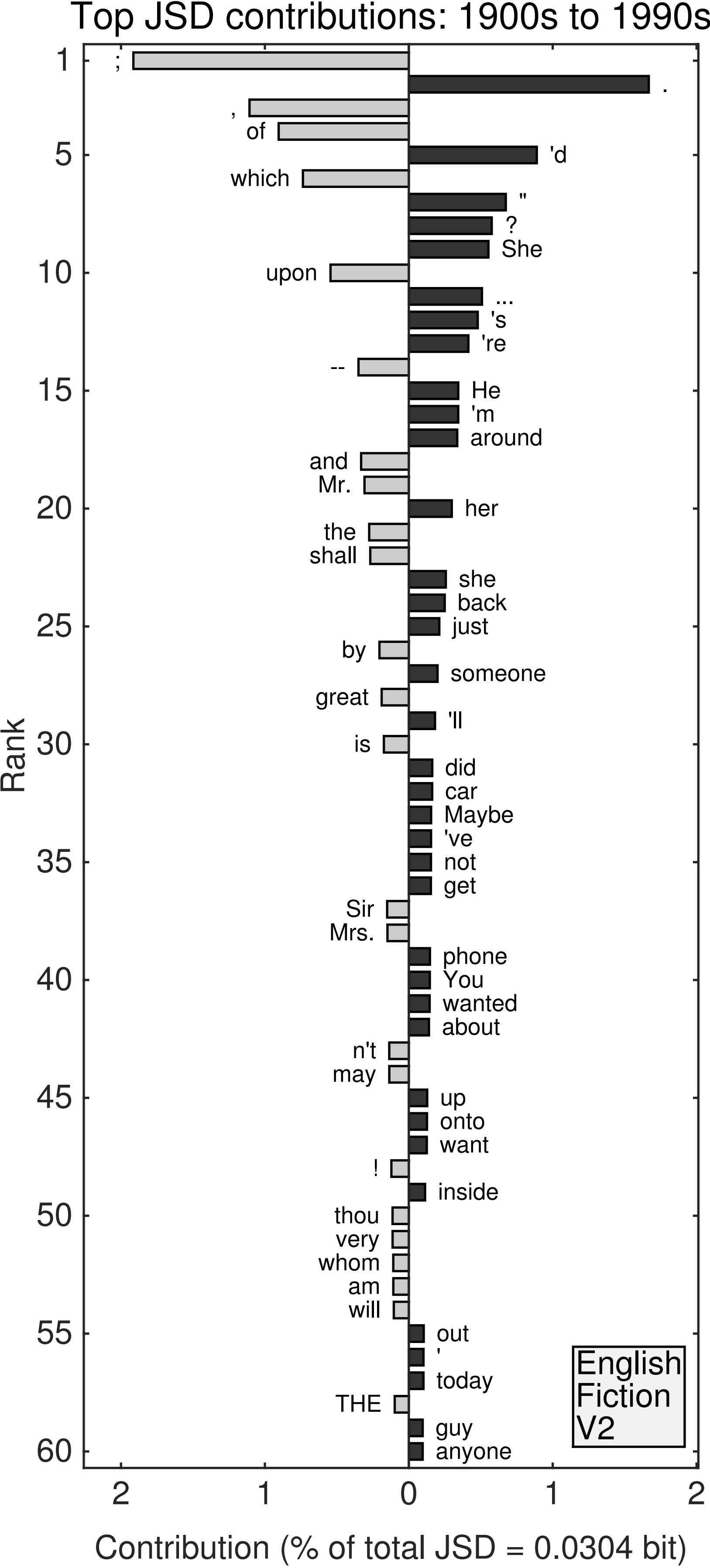

Top JSD contributions: 1900s to 1990s
Rank
Contribution (% of total JSD = 0.0304 bit)
;
.
,
of
'd
which
"
?
She
upon
...
's
're
--
He
'm
around
and
Mr.
her
the
shall
she
back
just
by
someone
great
'll
is
did
car
Maybe
've
not
get
Sir
Mrs.
phone
You
wanted
about
n't
may
up
onto
want
!
inside
thou
very
whom
am
will
out
'
today
THE
guy
anyone
English Fiction V2

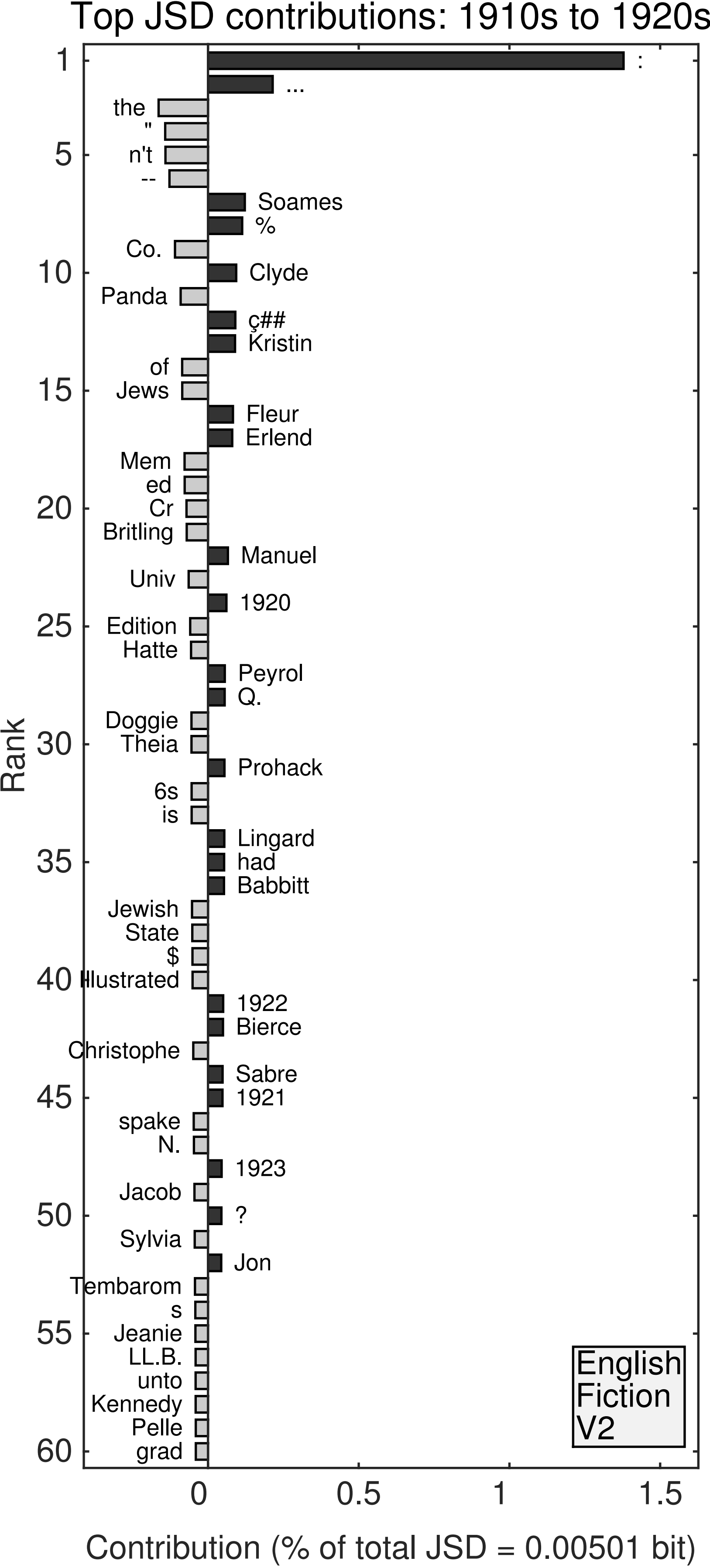

Top JSD contributions: 1910s to 1920s
Rank
Contribution (% of total JSD = 0.00501 bit)
English Fiction V2
:
...
the
"
n't
--
Soames
%
Co.
Clyde
Panda
ç##
Kristin
of
Jews
Fleur
Erlend
Mem
ed
Cr
Britling
Manuel
Univ
1920
Edition
Hatte
Peyrol
Q.
Doggie
Theia
Prohack
6s
is
Lingard
had
Babbitt
Jewish
State
$
Illustrated
1922
Bierce
Christophe
Sabre
1921
spake
N.
1923
Jacob
?
Sylvia
Jon
Tembarom
s
Jeanie
LL.B.
unto
Kennedy
Pelle
grad

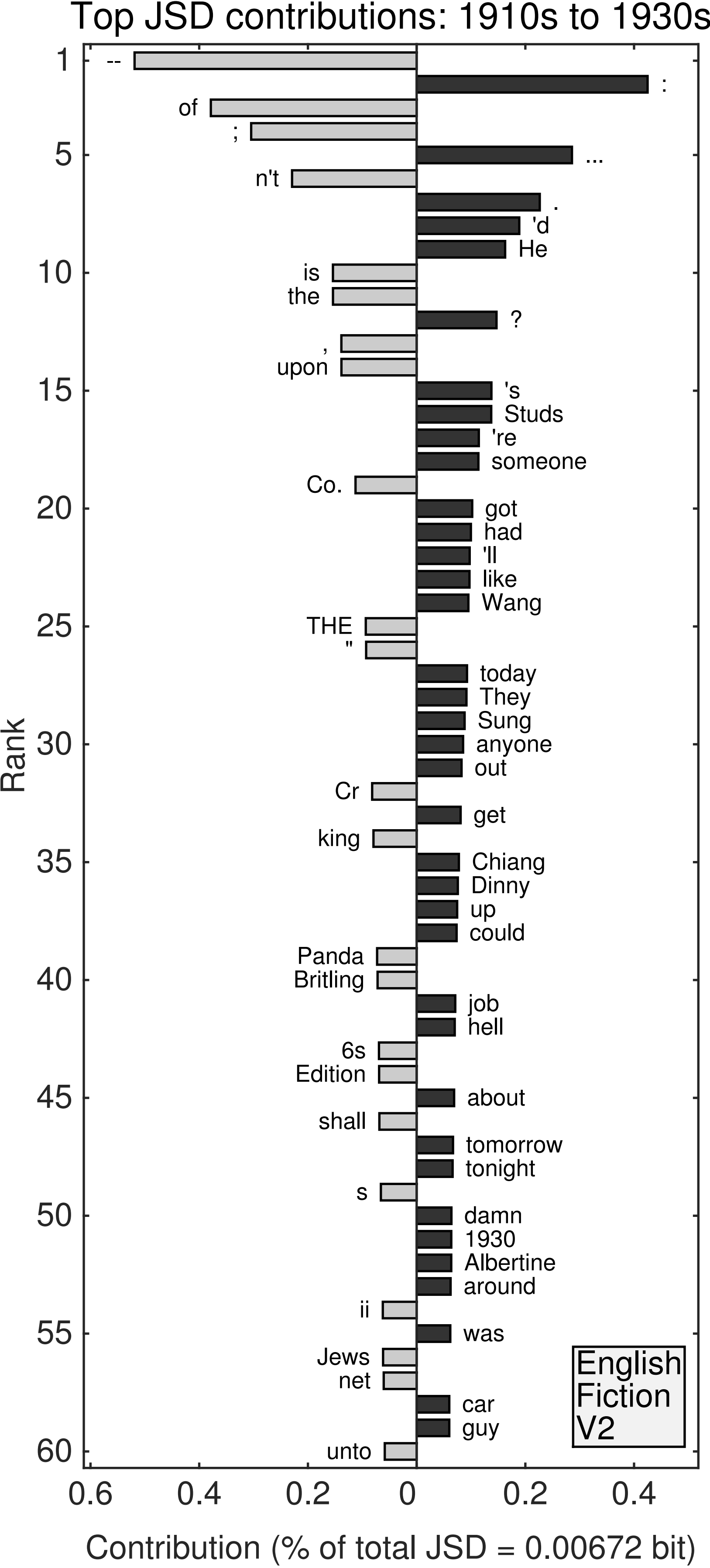

Top JSD contributions: 1910s to 1930s
Rank
Contribution (% of total JSD = 0.00672 bit)
English
Fiction
V2
--
:
of
;
...
n't
.
'd
He
is
the
?
,
upon
's
Studs
're
someone
Co.
got
had
'll
like
Wang
THE
"
today
They
Sung
anyone
out
Cr
get
king
Chiang
Dinny
up
could
Panda
Britling
job
hell
6s
Edition
about
shall
tomorrow
tonight
s
damn
1930
Albertine
around
ii
was
Jews
net
car
guy
unto

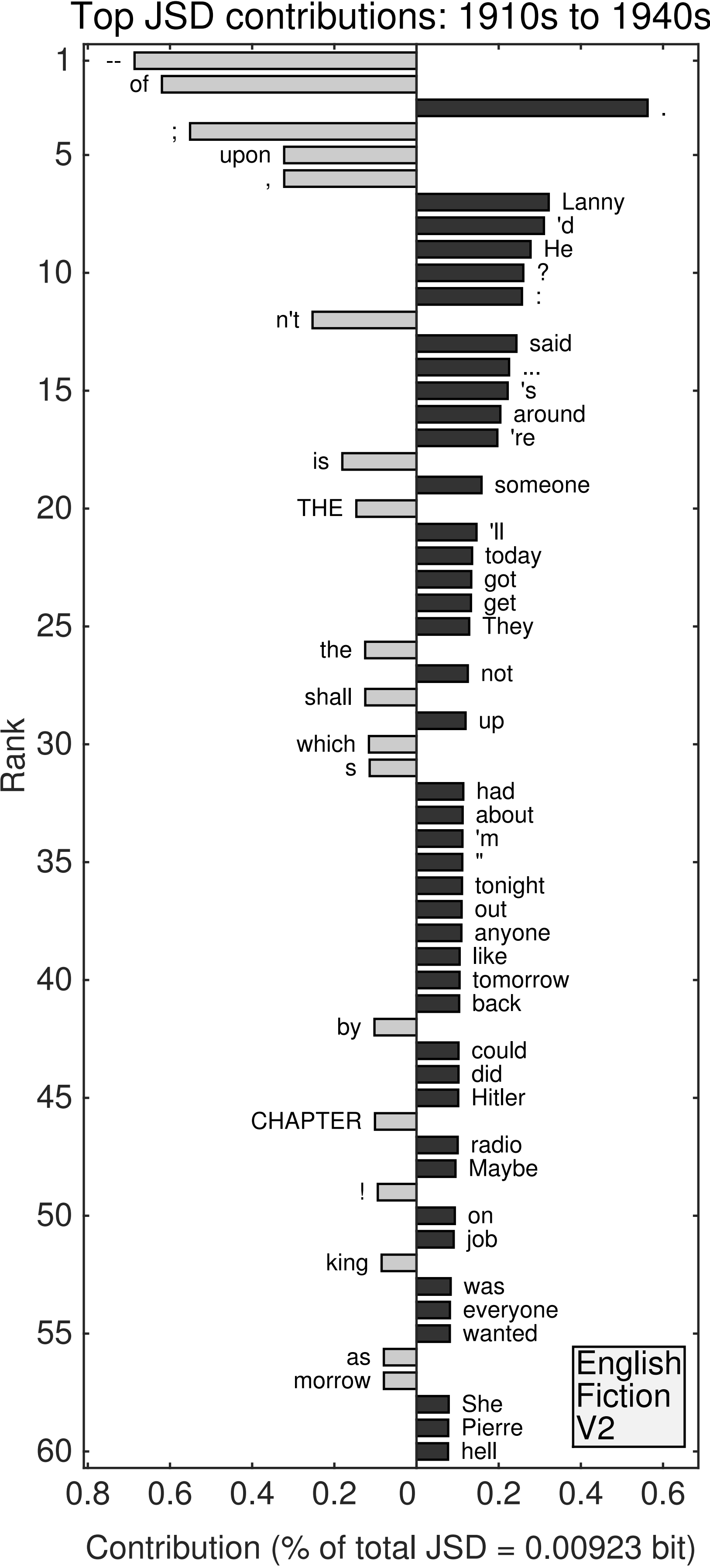
Top JSD contributions: 1910s to 1940s
Rank
Contribution (% of total JSD = 0.00923 bit)
English
Fiction
V2
--
of
.
;
upon
,
Lanny
'd
He
?
:
n't
said
...
's
around
're
is
someone
THE
'll
today
got
get
They
the
not
shall
up
which
s
had
about
'm
"
tonight
out
anyone
like
tomorrow
back
by
could
did
Hitler
CHAPTER
radio
Maybe
!
on
job
king
was
everyone
wanted
as
morrow
She
Pierre
hell
0.8
0.6
0.4
0.2
0
0.2
0.4
0.6
1
5
10
15
20
25
30
35
40
45
50
55
60

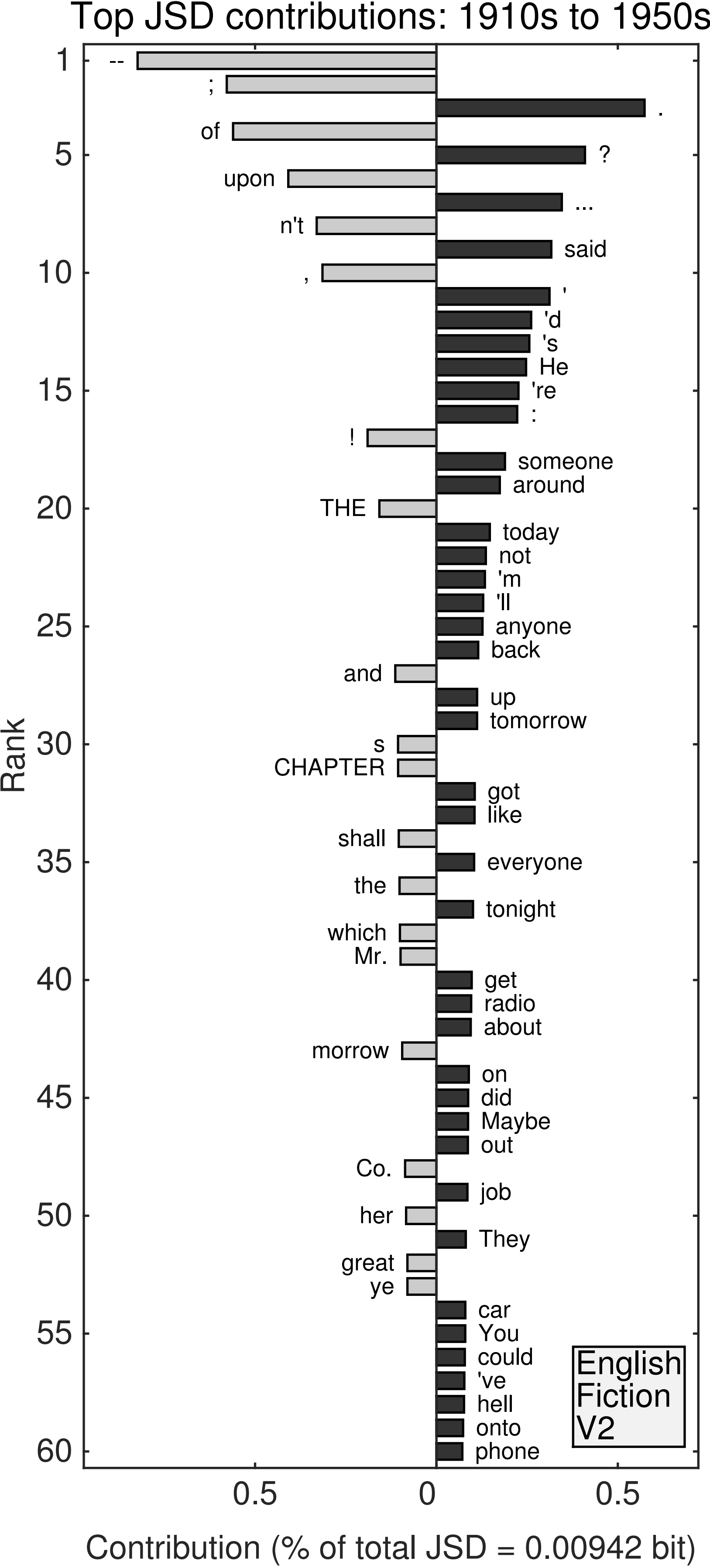
Top JSD contributions: 1910s to 1950s
Rank
1
5
10
15
20
25
30
35
40
45
50
55
60
--
;
.
of
?
upon
...
n't
said
,
'
'd
's
He
're
:
!
someone
around
THE
today
not
'm
'll
anyone
back
and
up
tomorrow
s
CHAPTER
got
like
shall
everyone
the
tonight
which
Mr.
get
radio
about
morrow
on
did
Maybe
out
Co.
job
her
They
great
ye
car
You
could
've
hell
onto
phone
English
Fiction
V2
0.5
0
0.5
Contribution (% of total JSD = 0.00942 bit)

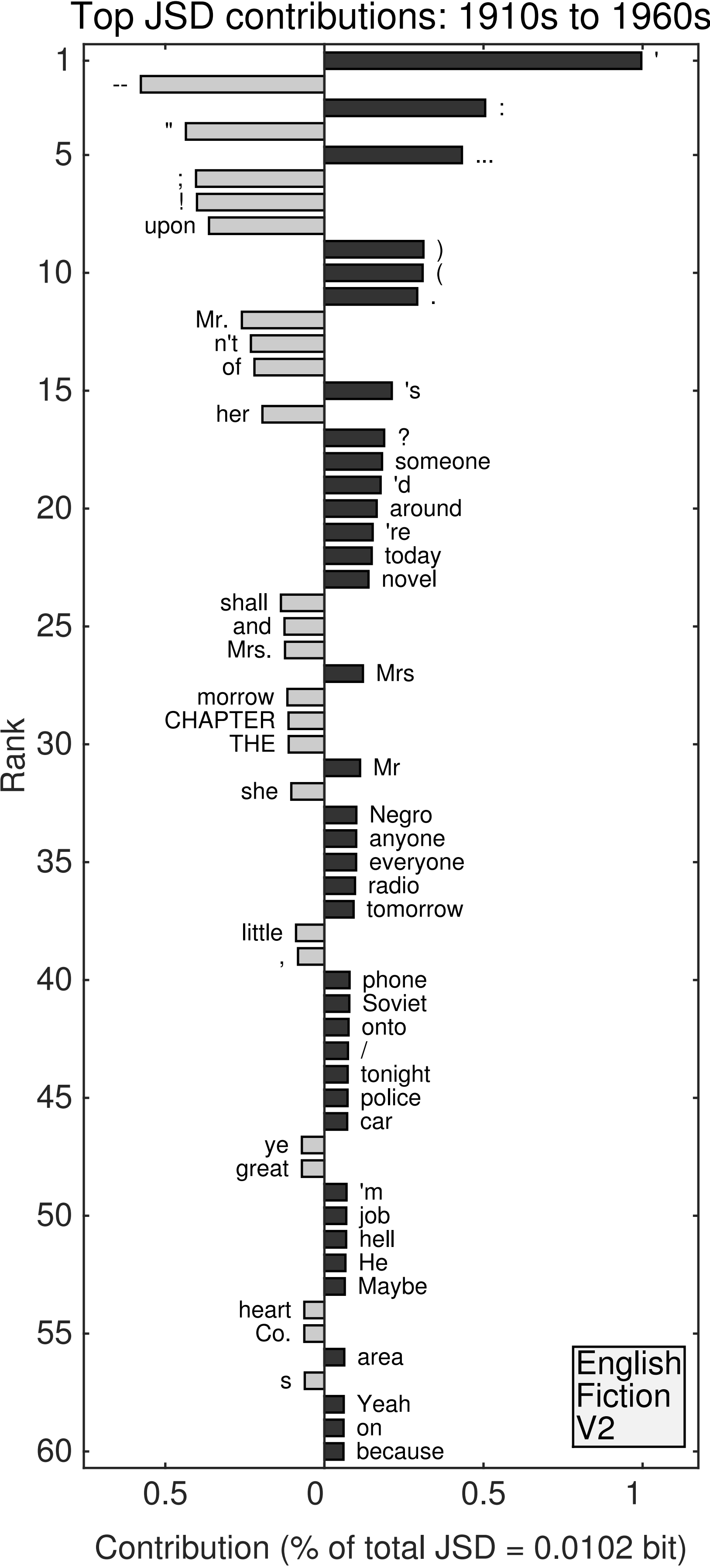
Top JSD contributions: 1910s to 1960s
Rank
Contribution (% of total JSD = 0.0102 bit)
English Fiction V2
1
5
10
15
20
25
30
35
40
45
50
55
60
0.5
0
0.5
1
'
--
:
"
...
;
!
upon
)
(
.
Mr.
n't
of
's
her
?
someone
'd
around
're
today
novel
shall
and
Mrs.
Mrs
morrow
CHAPTER
THE
Mr
she
Negro
anyone
everyone
radio
tomorrow
little
,
phone
Soviet
onto
/
tonight
police
car
ye
great
'm
job
hell
He
Maybe
heart
Co.
area
s
Yeah
on
because

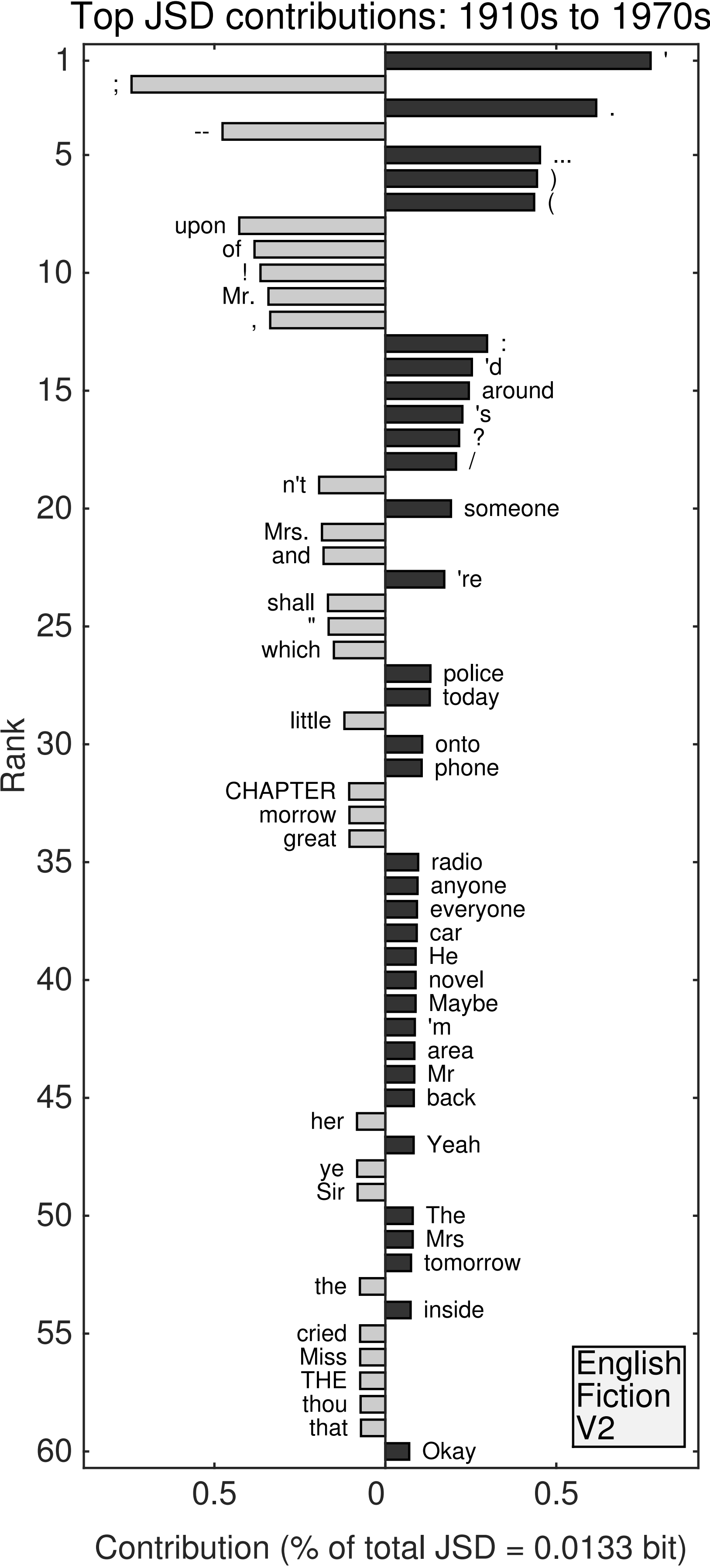
Top JSD contributions: 1910s to 1970s
Rank
Contribution (% of total JSD = 0.0133 bit)
English Fiction V2
'
;
.
--
...
)
(
upon
of
!
Mr.
,
:
'd
around
's
?
/
n't
someone
Mrs.
and
're
shall
"
which
police
today
little
onto
phone
CHAPTER
morrow
great
radio
anyone
everyone
car
He
novel
Maybe
'm
area
Mr
back
her
Yeah
ye
Sir
The
Mrs
tomorrow
the
inside
cried
Miss
THE
thou
that
Okay

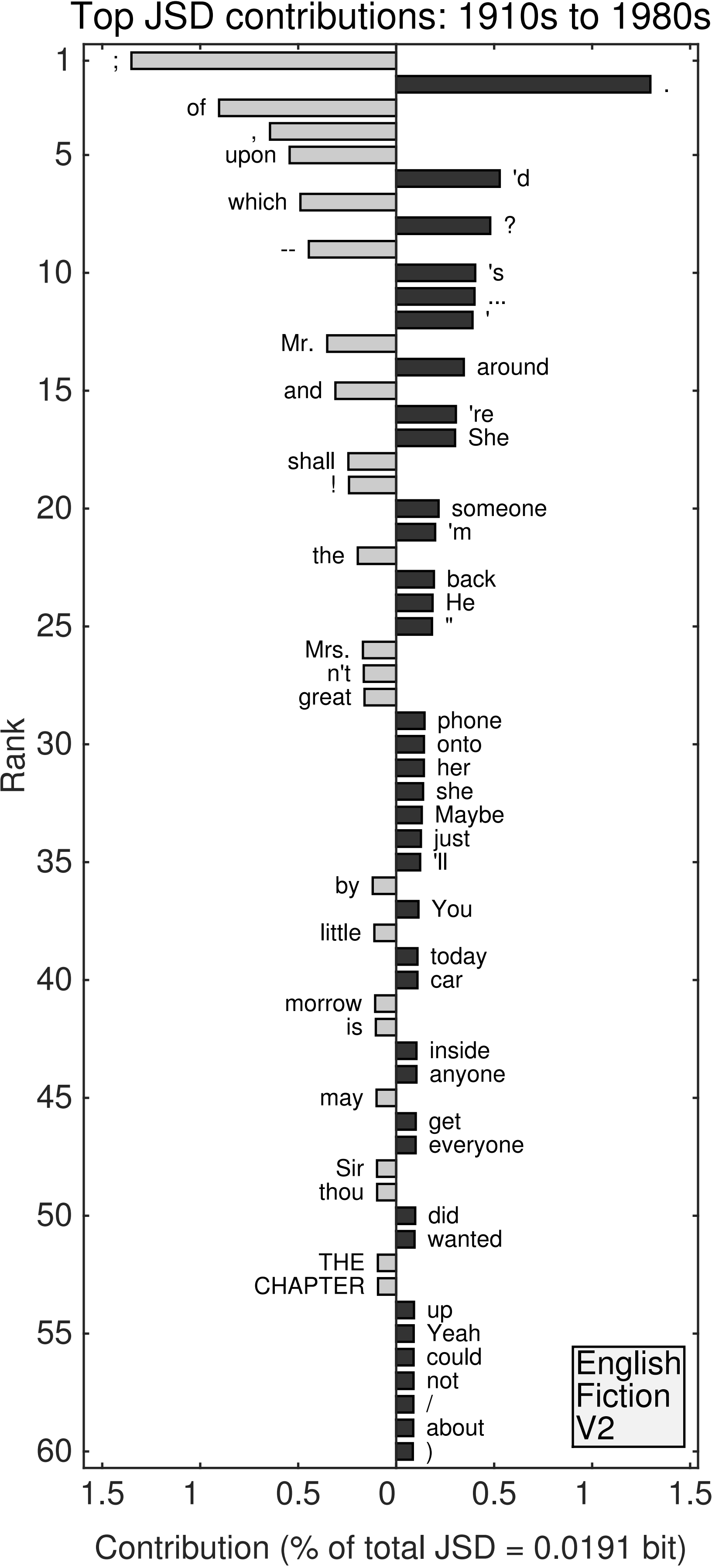
Top JSD contributions: 1910s to 1980s
Rank
Contribution (% of total JSD = 0.0191 bit)
;
.
of
,
upon
'd
which
?
--
's
...
'
Mr.
around
and
're
She
shall
!
someone
'm
the
back
He
"
Mrs.
n't
great
phone
onto
her
she
Maybe
just
'll
by
You
little
today
car
morrow
is
inside
anyone
may
get
everyone
Sir
thou
did
wanted
THE
CHAPTER
up
Yeah
could
not
/
about
)
English Fiction V2

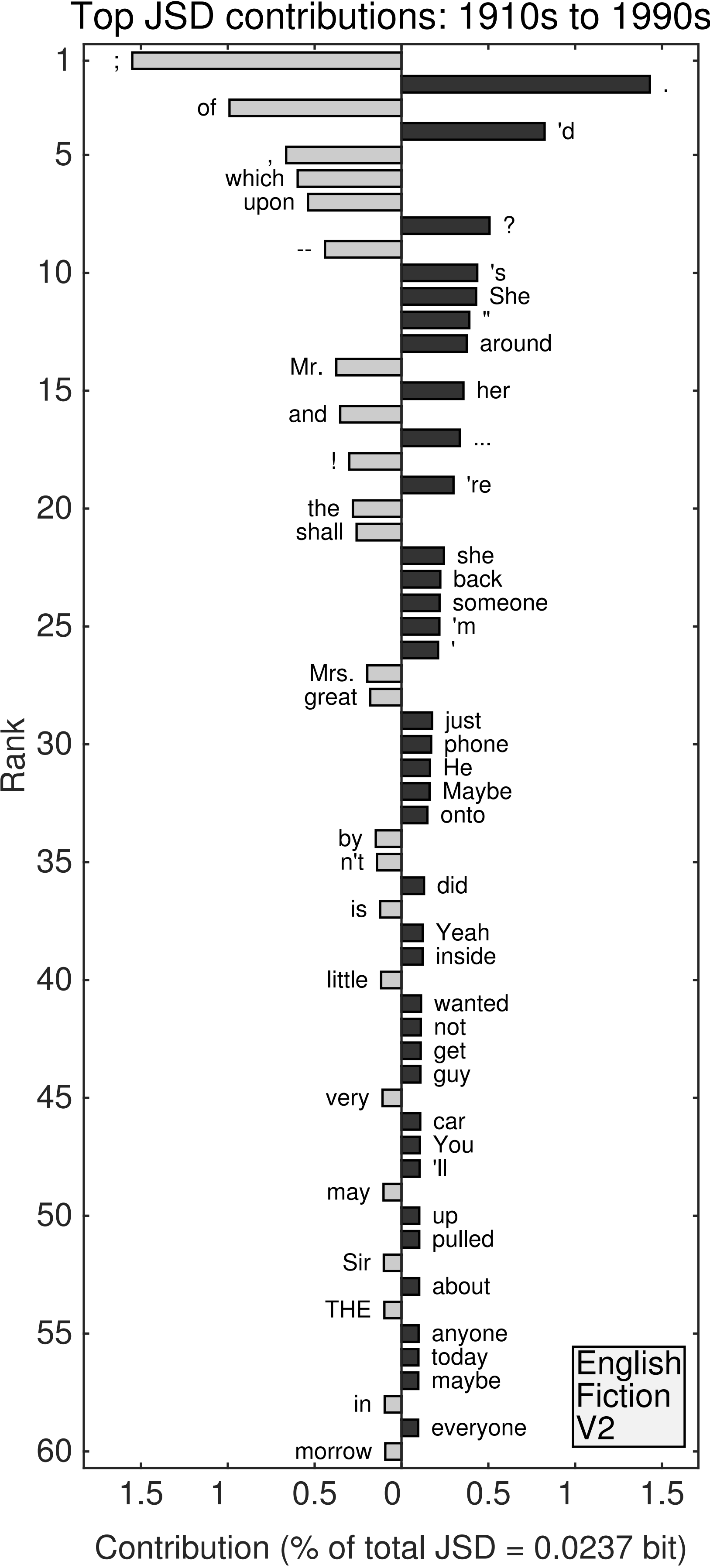

Top JSD contributions: 1910s to 1990s
Rank
Contribution (% of total JSD = 0.0237 bit)
English Fiction V2
;
.
of
'd
,
which
upon
?
--
's
She
"
around
Mr.
her
and
...
!
're
the
shall
she
back
someone
'm
'
Mrs.
great
just
phone
He
Maybe
onto
by
n't
did
is
Yeah
inside
little
wanted
not
get
guy
very
car
You
'll
may
up
pulled
Sir
about
THE
anyone
today
maybe
in
everyone
morrow

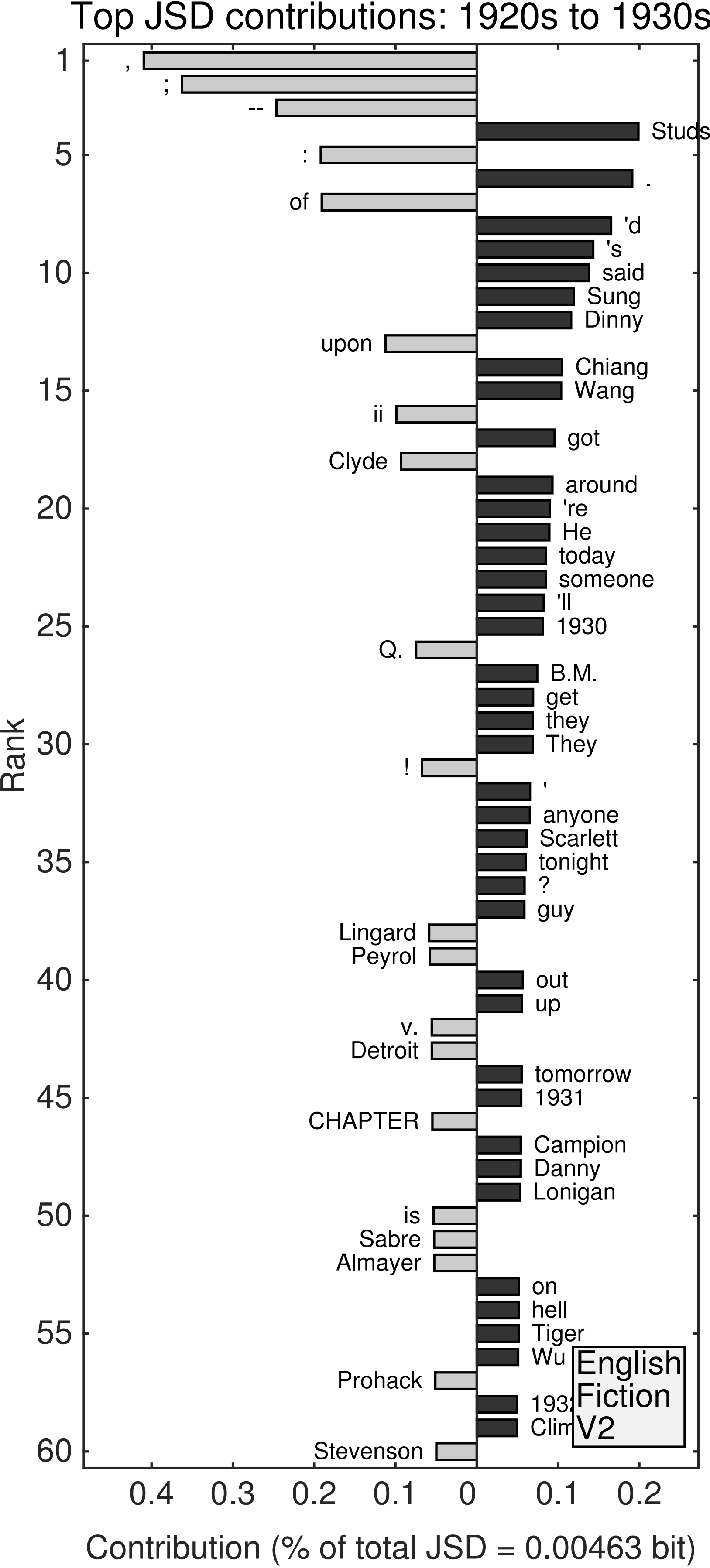
Top JSD contributions: 1920s to 1930s
Rank
Contribution (% of total JSD = 0.00463 bit)
,
;
--
Studs
:
.
of
'd
's
said
Sung
Dinny
upon
Chiang
Wang
ii
got
Clyde
around
're
He
today
someone
'll
1930
Q.
B.M.
get
they
They
!
'
anyone
Scarlett
tonight
?
guy
Lingard
Peyrol
out
up
v.
Detroit
tomorrow
1931
CHAPTER
Campion
Danny
Lonigan
is
Sabre
Almayer
on
hell
Tiger
Wu
Prohack
Stevenson
English Fiction V2

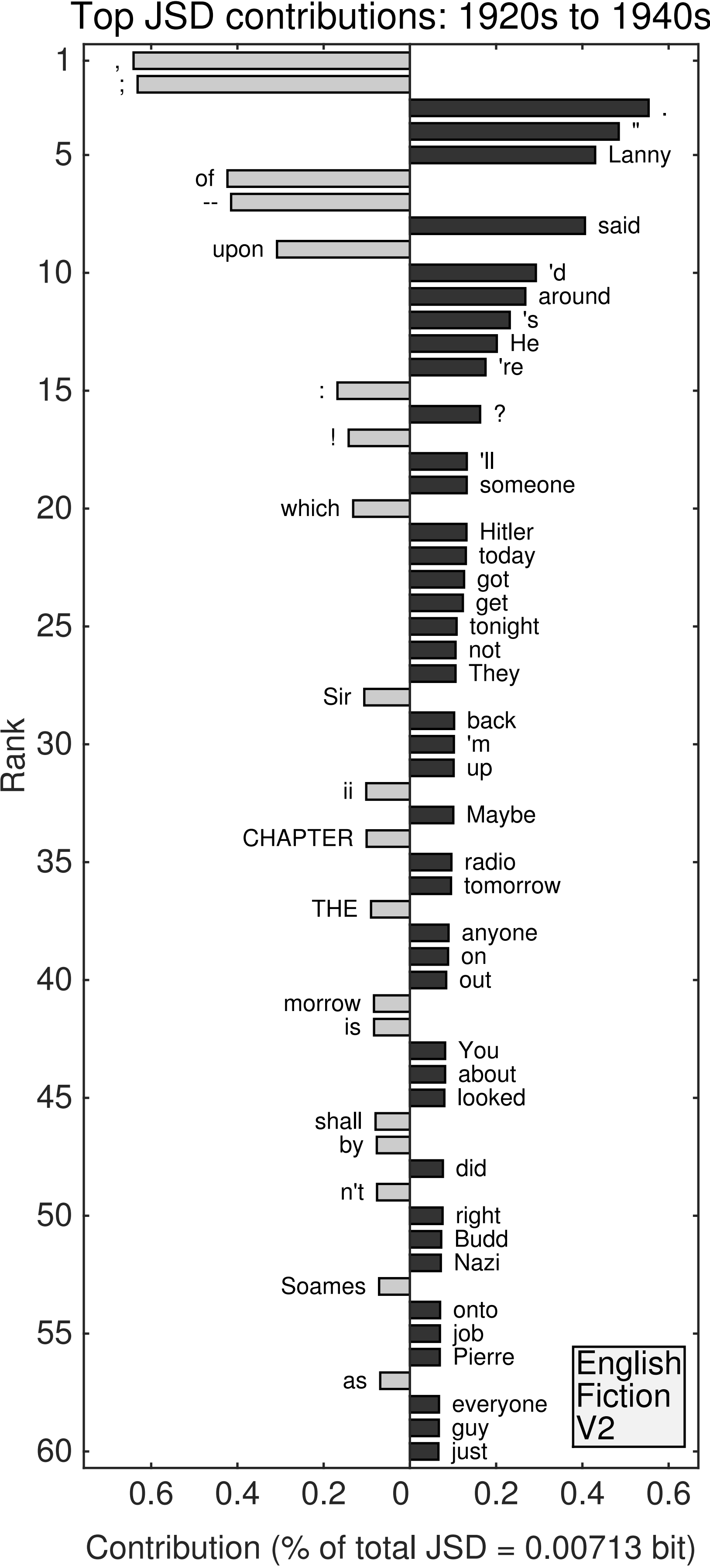
Top JSD contributions: 1920s to 1940s
Rank
Contribution (% of total JSD = 0.00713 bit)
English Fiction V2
,
;
.
"
Lanny
of
--
said
upon
'd
around
's
He
're
:
?
!
'll
someone
which
Hitler
today
got
get
tonight
not
They
Sir
back
'm
up
ii
Maybe
CHAPTER
radio
tomorrow
THE
anyone
on
out
morrow
is
You
about
looked
shall
by
did
n't
right
Budd
Nazi
Soames
onto
job
Pierre
as
everyone
guy
just
1
5
10
15
20
25
30
35
40
45
50
55
60
0.6
0.4
0.2
0
0.2
0.4
0.6

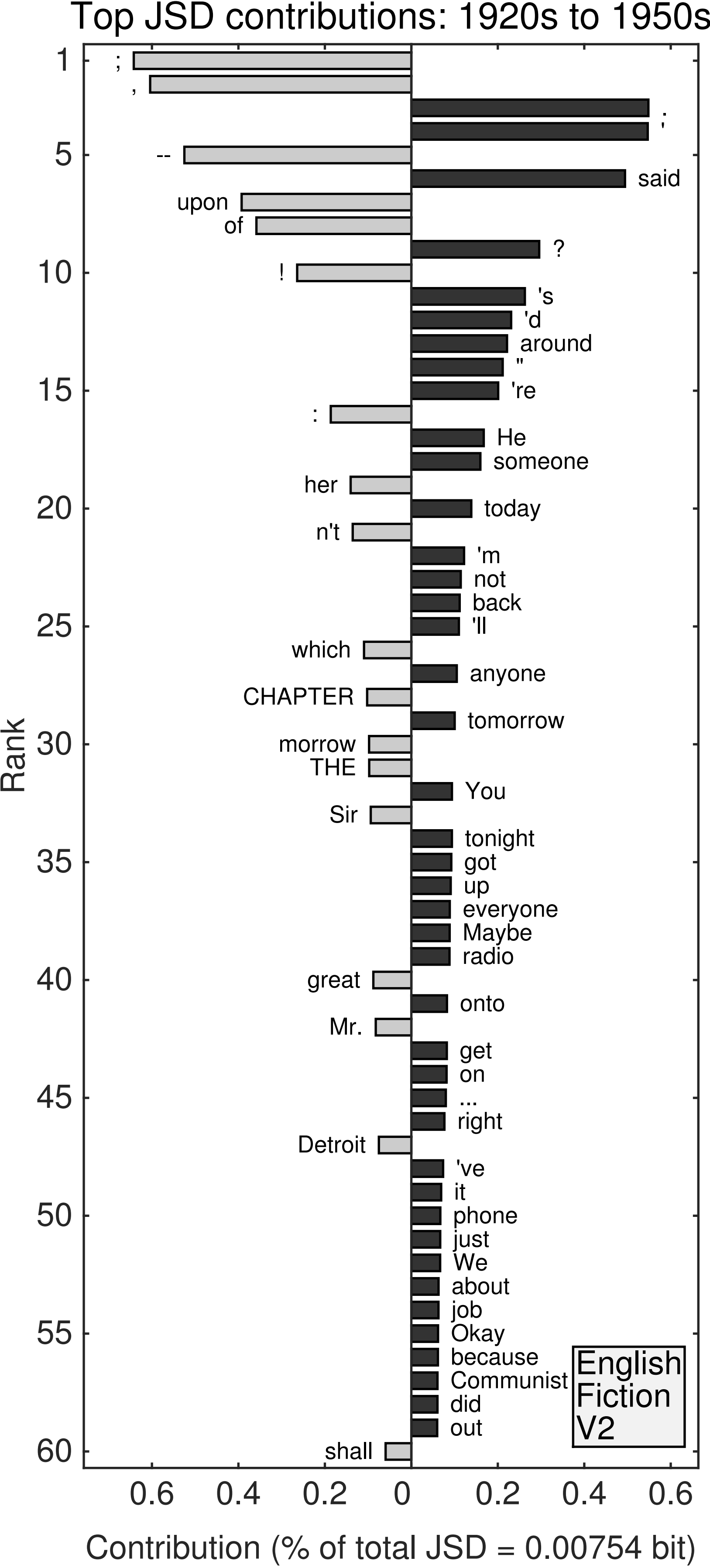
Top JSD contributions: 1920s to 1950s
Rank
Contribution (% of total JSD = 0.00754 bit)
English Fiction V2
;
,
;
'
--
said
upon
of
?
!
's
'd
around
"
're
:
He
someone
her
today
n't
'm
not
back
'll
which
anyone
CHAPTER
tomorrow
morrow
THE
You
Sir
tonight
got
up
everyone
Maybe
radio
great
onto
Mr.
get
on
...
right
Detroit
've
it
phone
just
We
about
job
Okay
because
Communist
did
out
shall

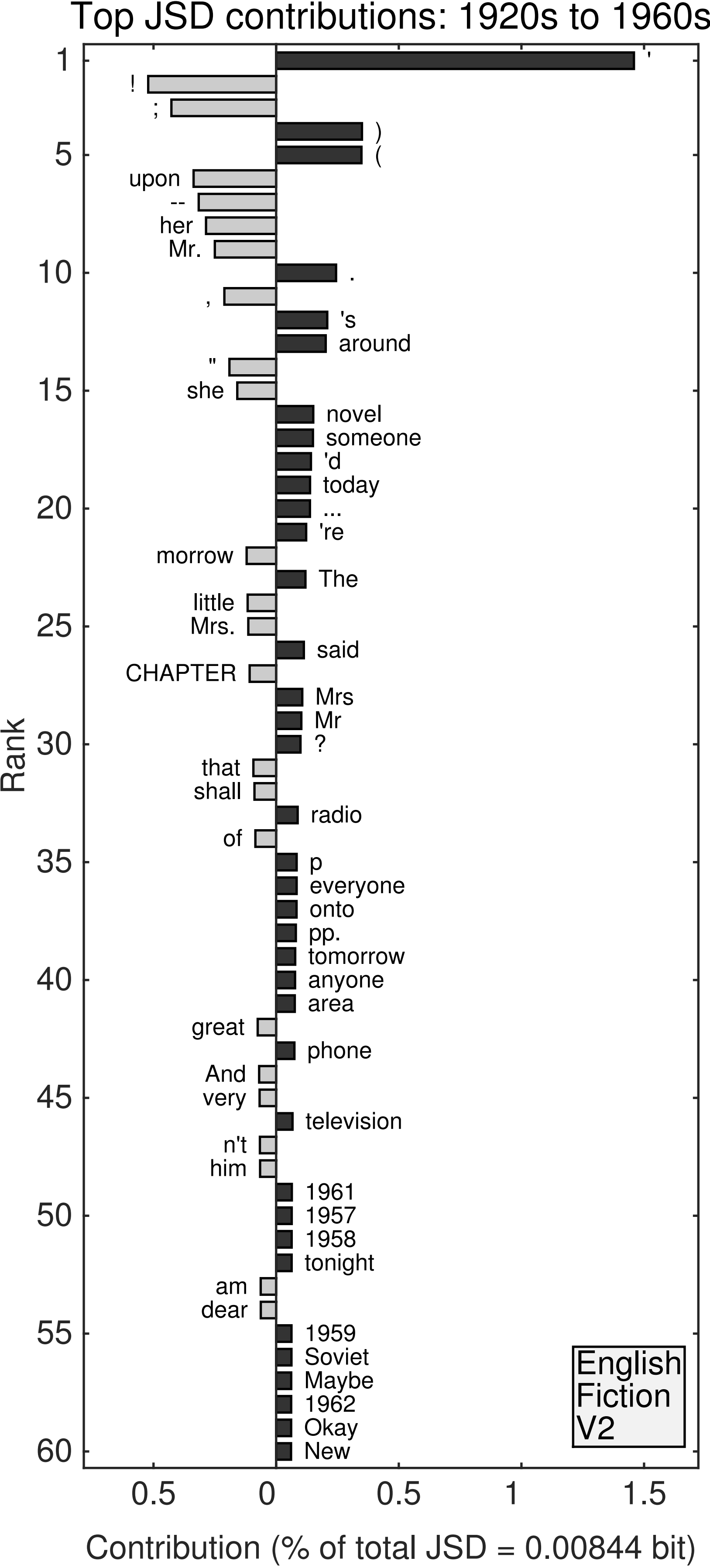

Top JSD contributions: 1920s to 1960s
Rank
Contribution (% of total JSD = 0.00844 bit)
English Fiction V2
'
!
;
)
(
upon
--
her
Mr.
.
,
's
around
"
she
novel
someone
'd
today
...
're
morrow
The
little
Mrs.
said
CHAPTER
Mrs
Mr
?
that
shall
radio
of
p
everyone
onto
pp.
tomorrow
anyone
area
great
phone
And
very
television
n't
him
1961
1957
1958
tonight
am
dear
1959
Soviet
Maybe
1962
Okay
New

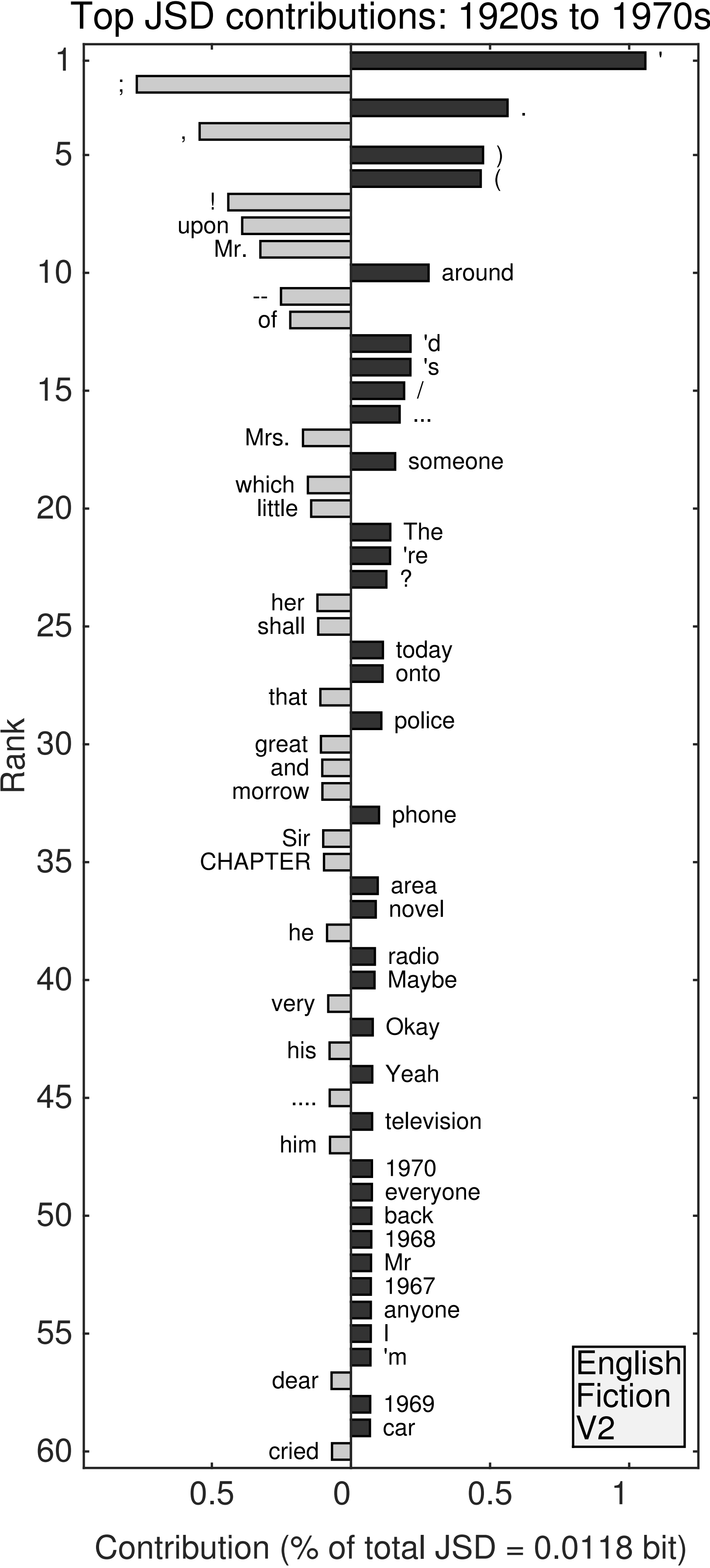
Top JSD contributions: 1920s to 1970s
Rank
Contribution (% of total JSD = 0.0118 bit)
English Fiction V2
'
;
.
,
)
(
!
upon
Mr.
around
--
of
'd
's
/
...
Mrs.
someone
which
little
The
're
?
her
shall
today
onto
that
police
great
and
morrow
phone
Sir
CHAPTER
area
novel
he
radio
Maybe
very
Okay
his
Yeah
....
television
him
1970
everyone
back
1968
Mr
1967
anyone
I
'm
dear
1969
car
cried

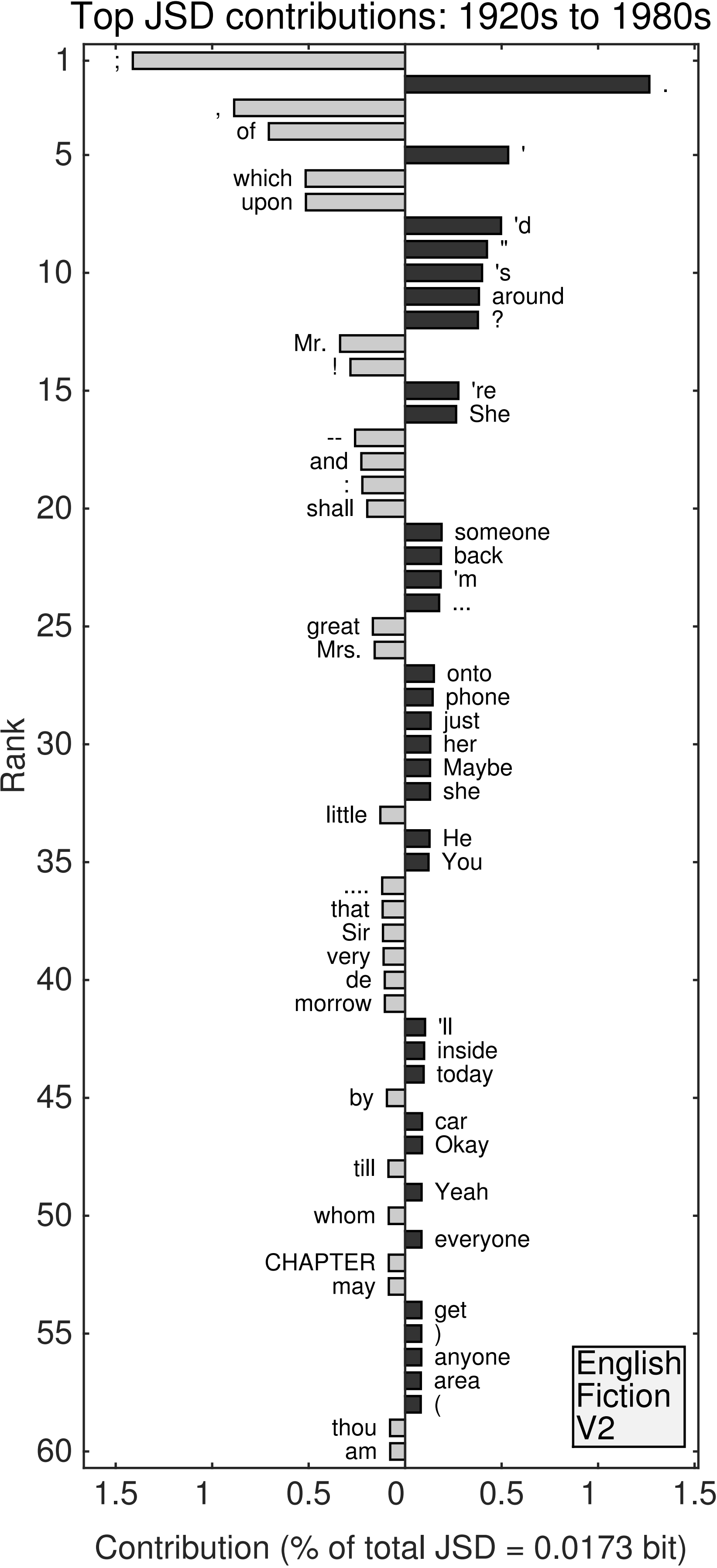

Top JSD contributions: 1920s to 1980s
Rank
Contribution (% of total JSD = 0.0173 bit)
1.5
1
0.5
0
0.5
1
1.5
1
5
10
15
20
25
30
35
40
45
50
55
60
;
.
,
of
'
which
upon
'd
"
's
around
?
Mr.
!
're
She
--
and
:
shall
someone
back
'm
...
great
Mrs.
onto
phone
just
her
Maybe
she
little
He
You
....
that
Sir
very
de
morrow
'll
inside
today
by
car
Okay
till
Yeah
whom
everyone
CHAPTER
may
get
)
anyone
area
(
thou
am
English
Fiction
V2

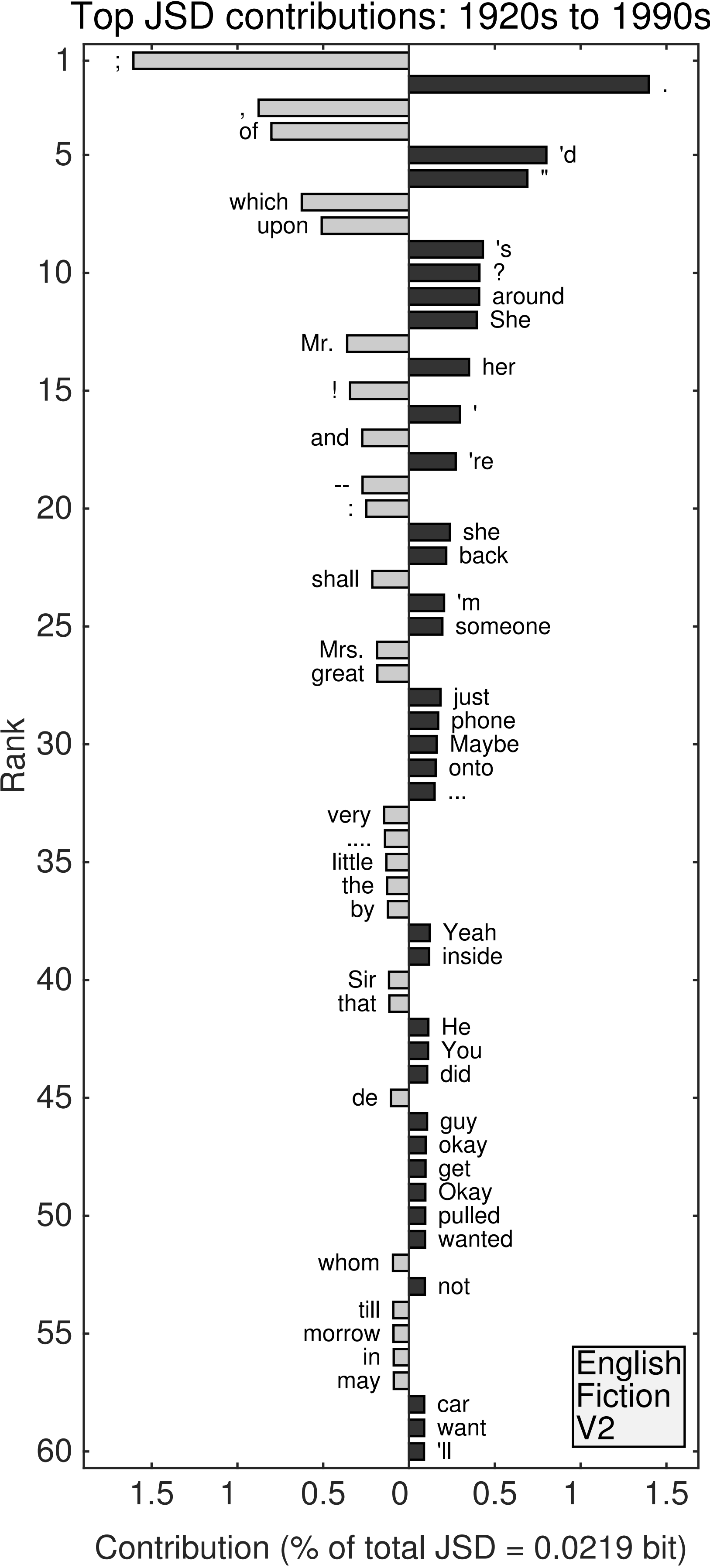
Top JSD contributions: 1920s to 1990s
Rank
Contribution (% of total JSD = 0.0219 bit)
English Fiction V2
;
.
,
of
'd
"
which
upon
's
?
around
She
Mr.
her
!
'
and
're
--
:
she
back
shall
'm
someone
Mrs.
great
just
phone
Maybe
onto
...
very
....
little
the
by
Yeah
inside
Sir
that
He
You
did
de
guy
okay
get
Okay
pulled
wanted
whom
not
till
morrow
in
may
car
want
'll

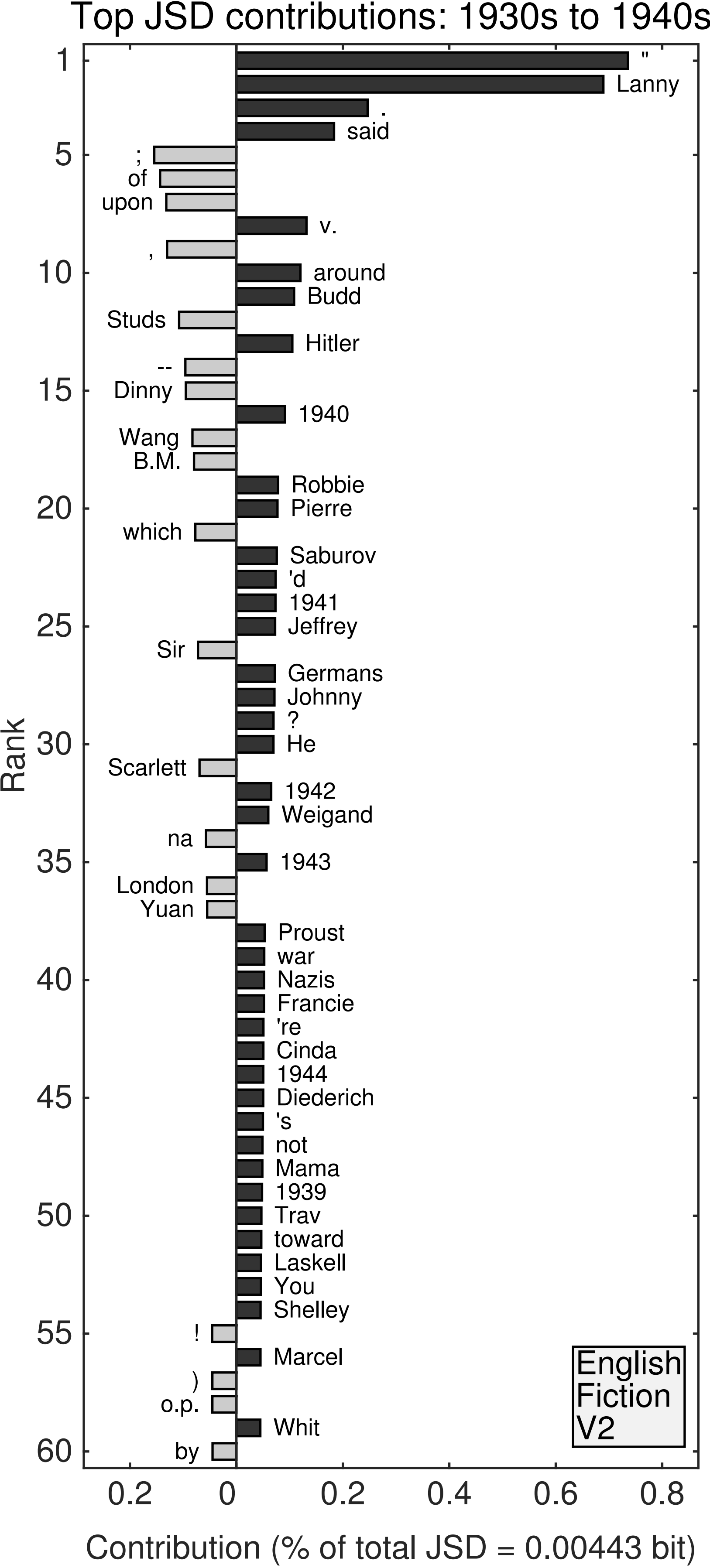

Top JSD contributions: 1930s to 1940s
Rank
Contribution (% of total JSD = 0.00443 bit)
English Fiction V2
"
Lanny
.
said
;
of
upon
v.
,
around
Budd
Studs
Hitler
--
Dinny
1940
Wang
B.M.
Robbie
Pierre
which
Saburov
'd
1941
Jeffrey
Sir
Germans
Johnny
?
He
Scarlett
1942
Weigand
na
1943
London
Yuan
Proust
war
Nazis
Francie
're
Cinda
1944
Diederich
's
not
Mama
1939
Trav
toward
Laskell
You
Shelley
!
Marcel
)
o.p.
Whit
by

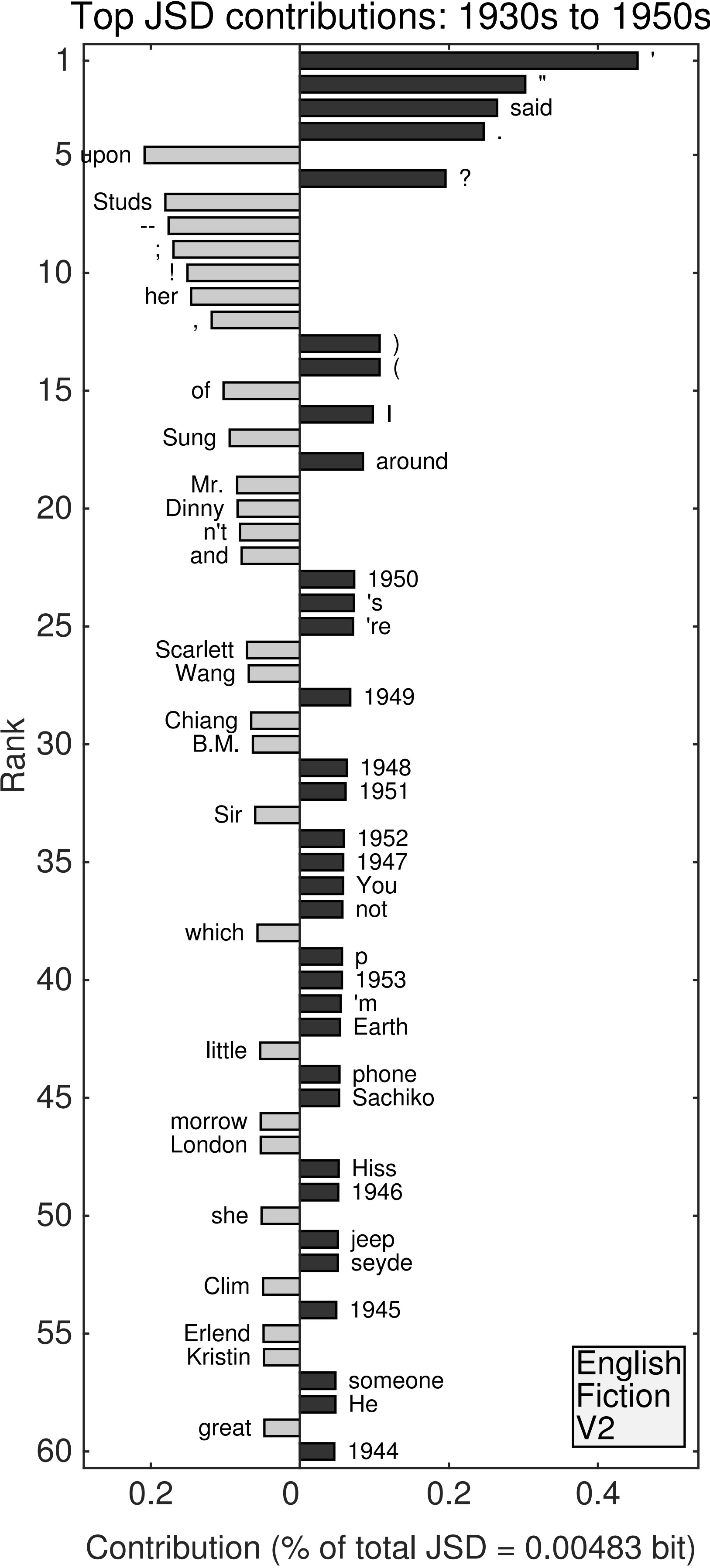
Top JSD contributions: 1930s to 1950s
Rank
Contribution (% of total JSD = 0.00483 bit)
English Fiction V2
'
"
said
.
upon
?
Studs
--
;
!
her
,
)
(
of
I
Sung
around
Mr.
Dinny
n't
and
1950
's
're
Scarlett
Wang
1949
Chiang
B.M.
1948
1951
Sir
1952
1947
You
not
which
p
1953
'm
Earth
little
phone
Sachiko
morrow
London
Hiss
1946
she
jeep
seyde
Clim
1945
Erlend
Kristin
someone
He
great
1944

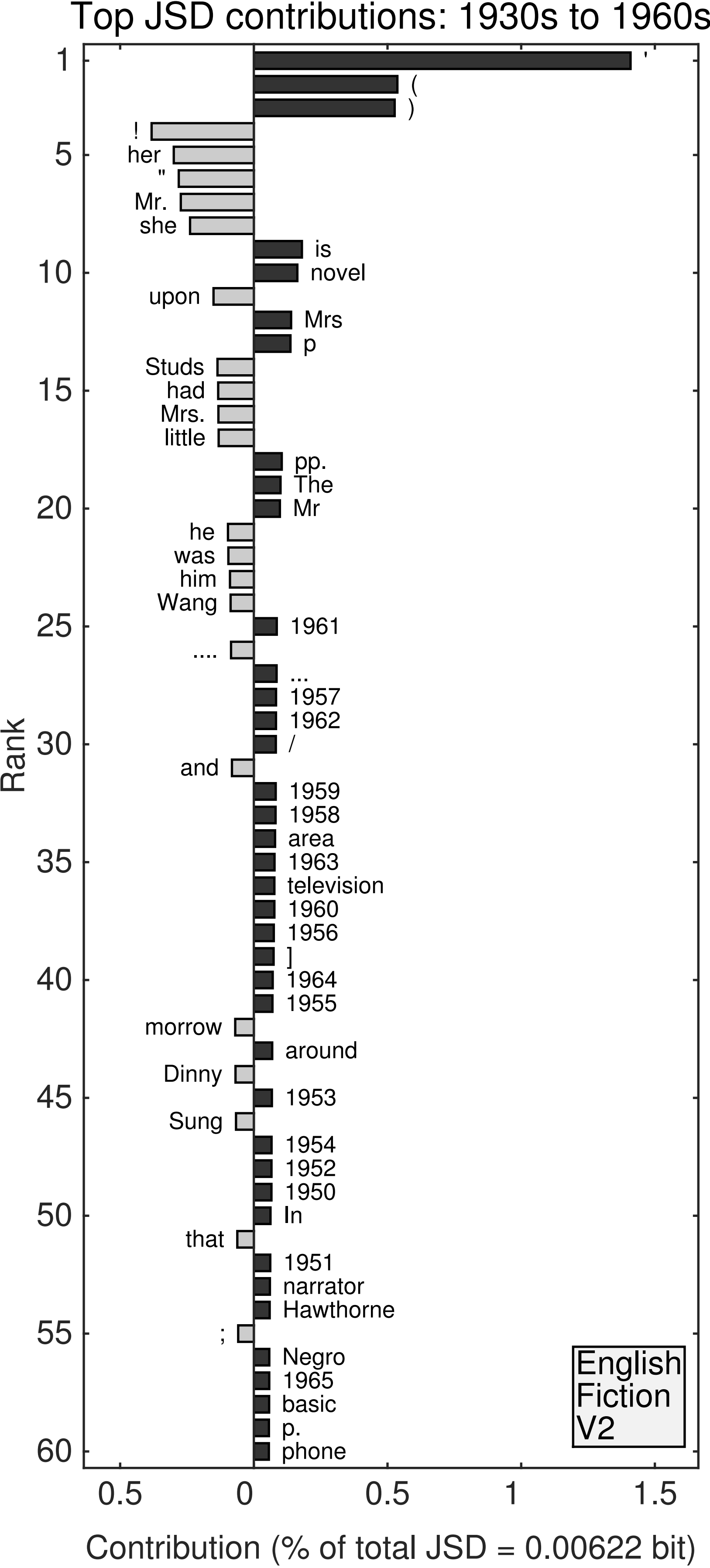
Top JSD contributions: 1930s to 1960s
Rank
Contribution (% of total JSD = 0.00622 bit)
English Fiction V2
'
(
)
!
her
"
Mr.
she
is
novel
upon
Mrs
p
Studs
had
Mrs.
little
pp.
The
Mr
he
was
him
Wang
1961
....
...
1957
1962
/
and
1959
1958
area
1963
television
1960
1956
]
1964
1955
morrow
around
Dinny
1953
Sung
1954
1952
1950
In
that
1951
narrator
Hawthorne
;
Negro
1965
basic
p.
phone

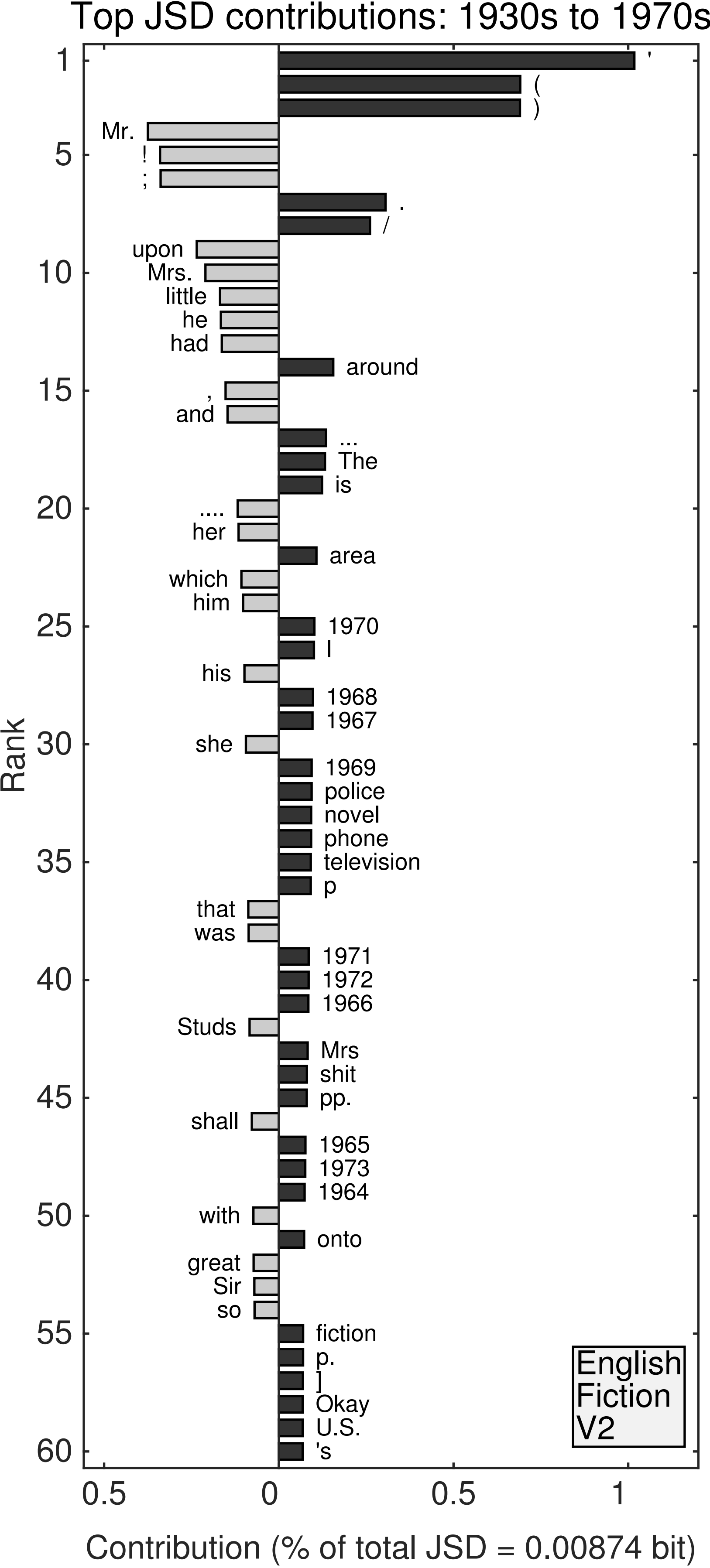

Top JSD contributions: 1930s to 1970s
Rank
'
(
)
Mr.
!
;
.
/
upon
Mrs.
little
he
had
around
,
and
...
The
is
....
her
area
which
him
1970
I
his
1968
1967
she
1969
police
novel
phone
television
p
that
was
1971
1972
1966
Studs
Mrs
shit
pp.
shall
1965
1973
1964
with
onto
great
Sir
so
fiction
p.
]
Okay
U.S.
's
English Fiction V2
Contribution (% of total JSD = 0.00874 bit)

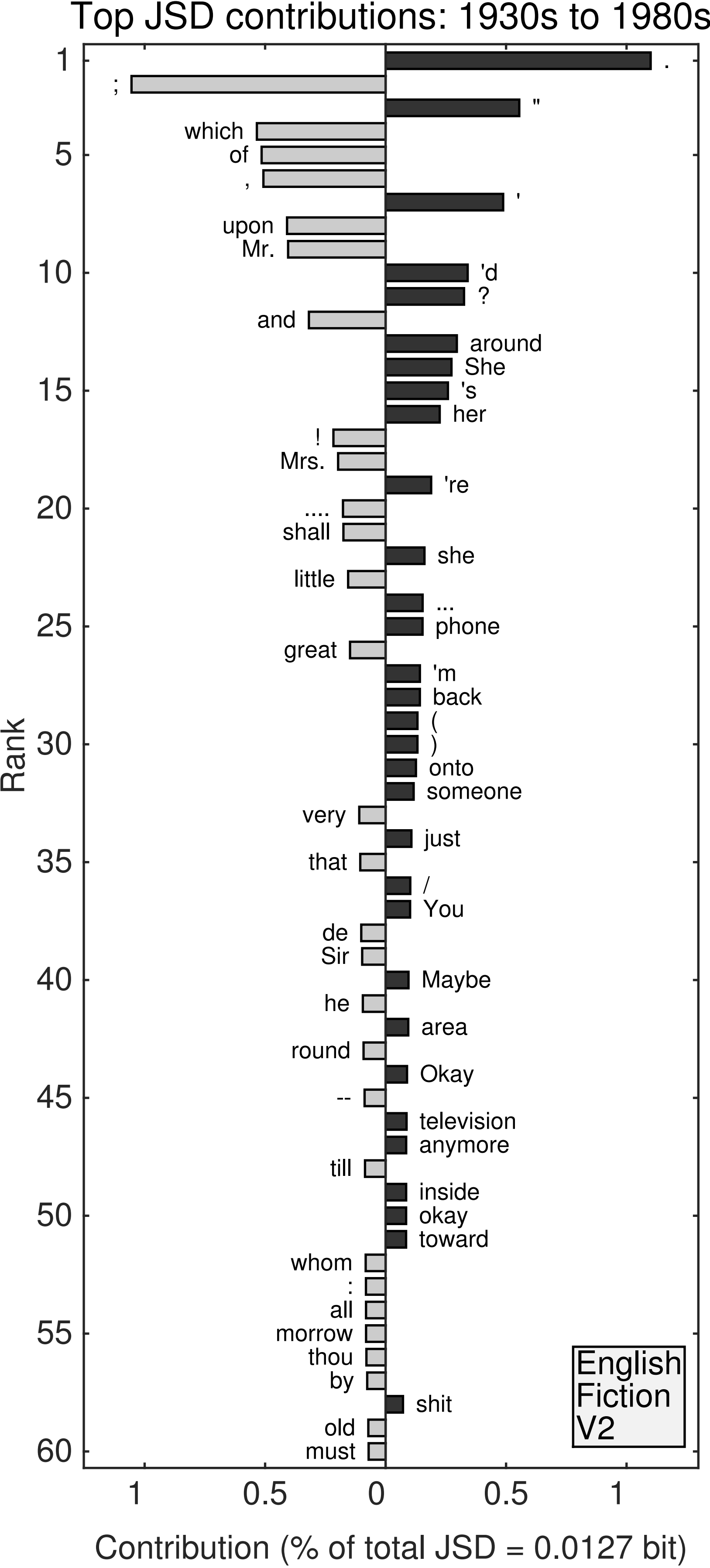

Top JSD contributions: 1930s to 1980s
Rank
Contribution (% of total JSD = 0.0127 bit)
1
5
10
15
20
25
30
35
40
45
50
55
60
1
0.5
0
0.5
1
.
;
"
which
of
,
'
upon
Mr.
'd
?
and
around
She
's
her
!
Mrs.
're
....
shall
she
little
...
phone
great
'm
back
(
)
onto
someone
very
just
that
/
You
de
Sir
Maybe
he
area
round
Okay
--
television
anymore
till
inside
okay
toward
whom
:
all
morrow
thou
by
shit
old
must
English
Fiction
V2

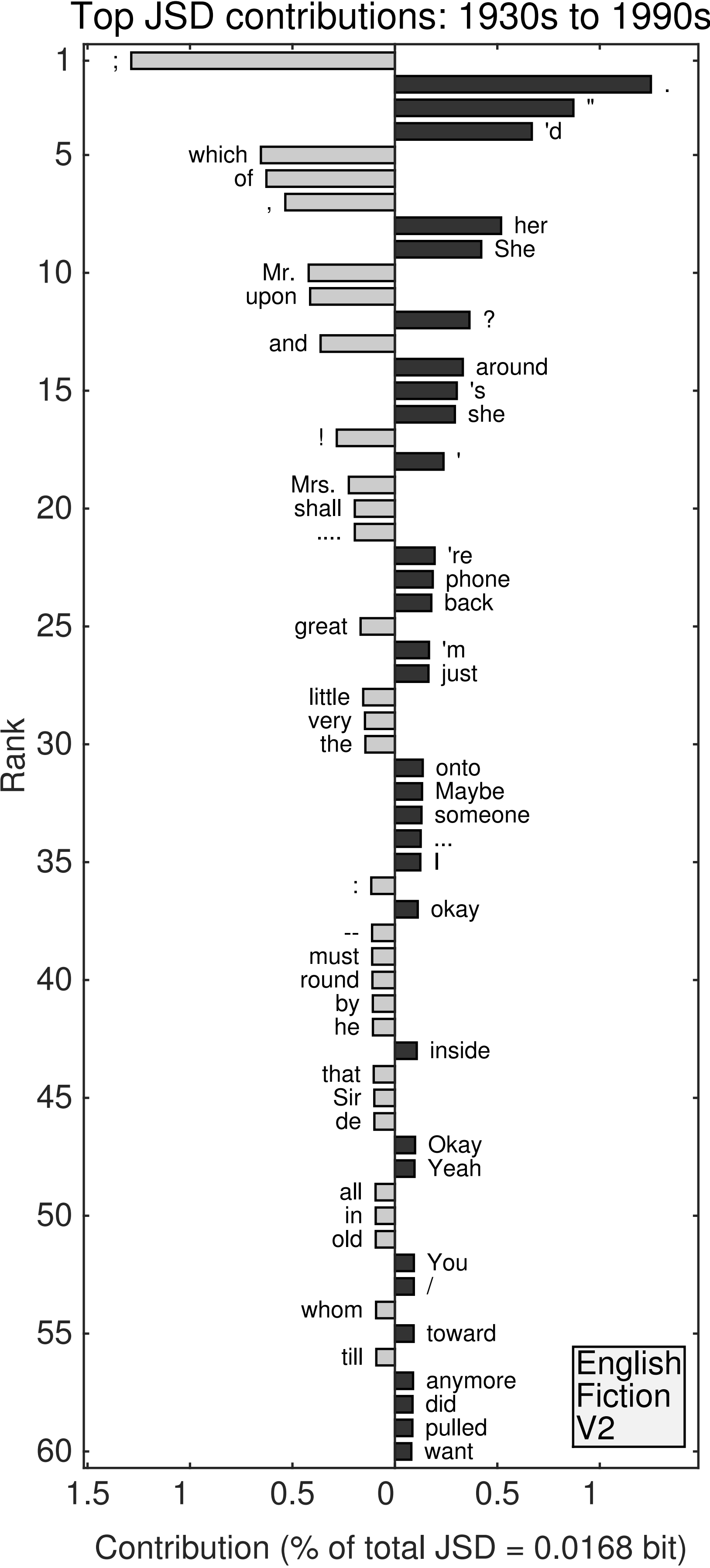

Top JSD contributions: 1930s to 1990s
Rank
Contribution (% of total JSD = 0.0168 bit)
;
.
"
'd
which
of
,
her
She
Mr.
upon
?
and
around
's
she
!
'
Mrs.
shall
....
're
phone
back
great
'm
just
little
very
the
onto
Maybe
someone
...
I
:
okay
--
must
round
by
he
inside
that
Sir
de
Okay
Yeah
all
in
old
You
/
whom
toward
till
anymore
did
pulled
want
English
Fiction
V2

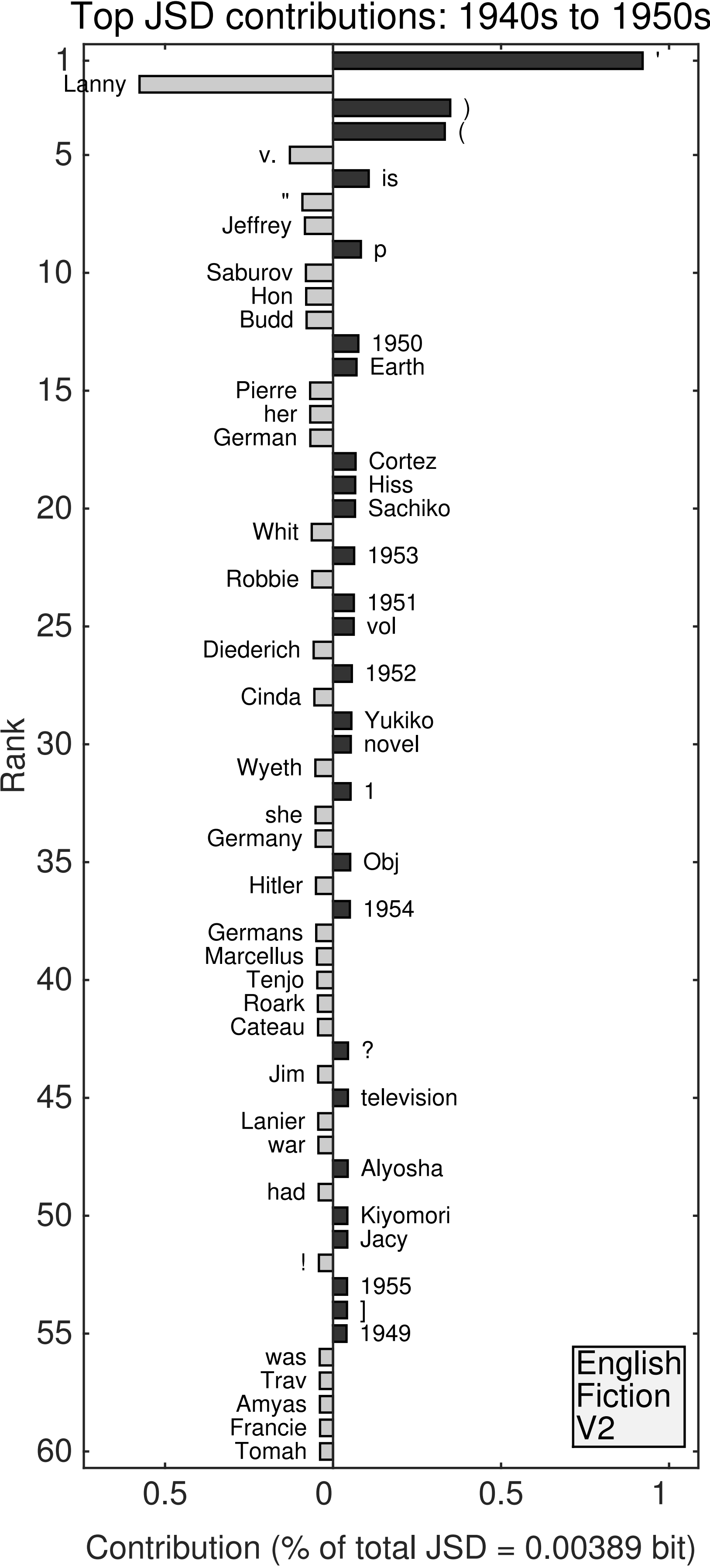
Top JSD contributions: 1940s to 1950s
Rank
Contribution (% of total JSD = 0.00389 bit)
English Fiction V2
'
Lanny
)
(
v.
is
"
Jeffrey
p
Saburov
Hon
Budd
1950
Earth
Pierre
her
German
Cortez
Hiss
Sachiko
Whit
1953
Robbie
1951
vol
Diederich
1952
Cinda
Yukiko
novel
Wyeth
1
she
Germany
Obj
Hitler
1954
Germans
Marcellus
Tenjo
Roark
Cateau
?
Jim
television
Lanier
war
Alyosha
had
Kiyomori
Jacy
!
1955
]
1949
was
Trav
Amyas
Francie
Tomah

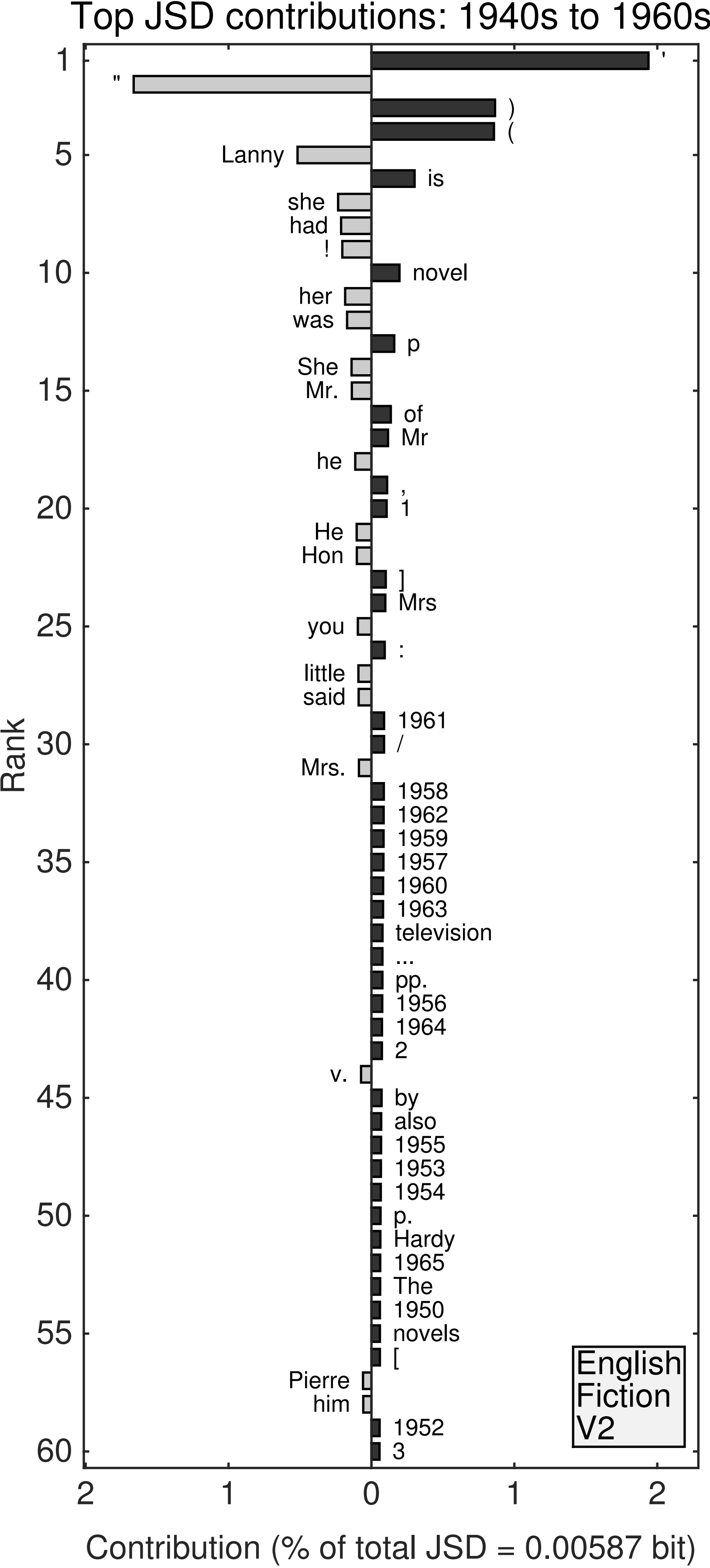

Top JSD contributions: 1940s to 1960s
Rank
Contribution (% of total JSD = 0.00587 bit)
English Fiction V2
'
"
)
(
Lanny
is
she
had
!
novel
her
was
p
She
Mr.
of
Mr
he
,
1
He
Hon
]
Mrs
you
:
little
said
1961
/
Mrs.
1958
1962
1959
1957
1960
1963
television
...
pp.
1956
1964
2
v.
by
also
1955
1953
1954
p.
Hardy
1965
The
1950
novels
[
Pierre
him
1952
3

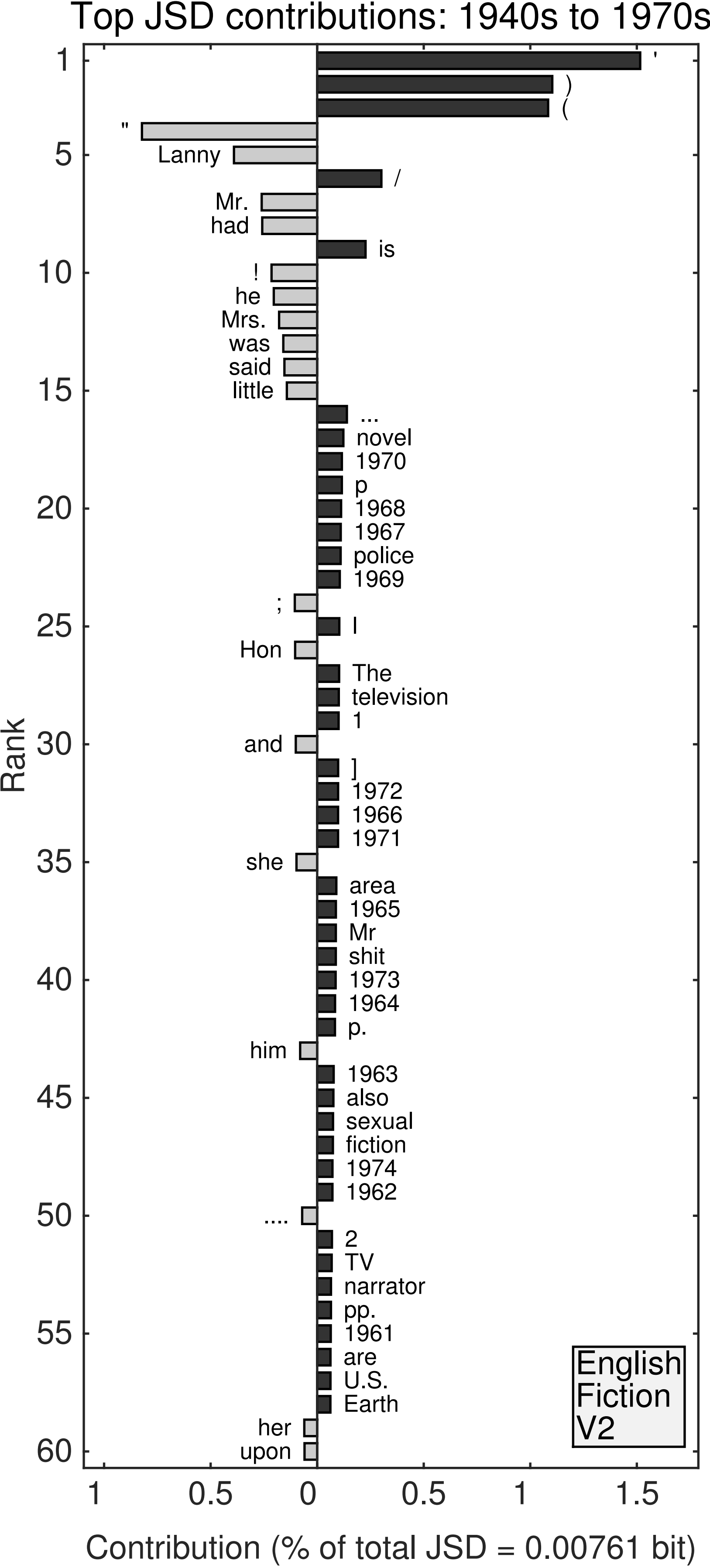

Top JSD contributions: 1940s to 1970s
Rank
Contribution (% of total JSD = 0.00761 bit)
English Fiction V2
'
)
(
"
Lanny
/
Mr.
had
is
!
he
Mrs.
was
said
little
...
novel
1970
p
1968
1967
police
1969
;
I
Hon
The
television
1
and
]
1972
1966
1971
she
area
1965
Mr
shit
1973
1964
p.
him
1963
also
sexual
fiction
1974
1962
....
2
TV
narrator
pp.
1961
are
U.S.
Earth
her
upon

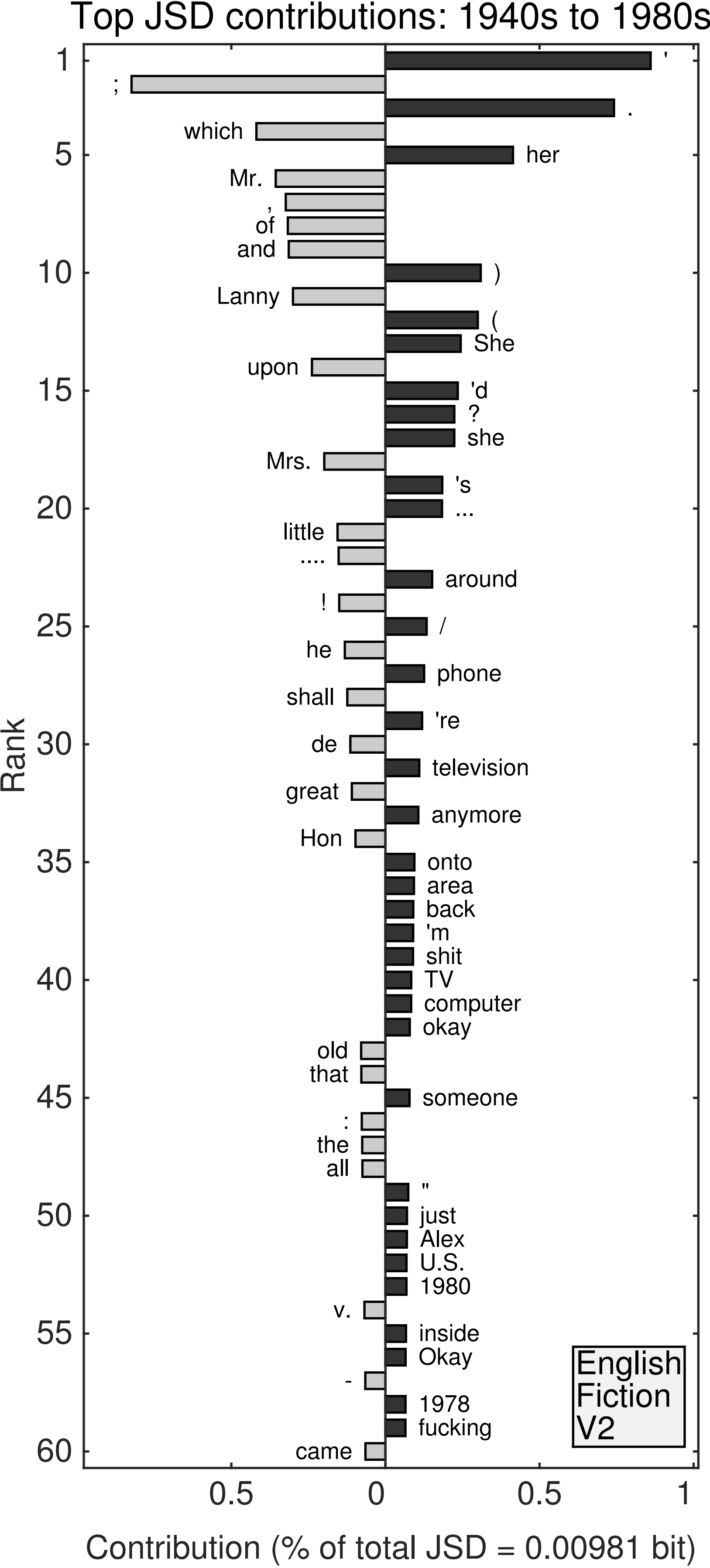

Top JSD contributions: 1940s to 1980s
Rank
Contribution (% of total JSD = 0.00981 bit)
0.5
0
0.5
1
1
5
10
15
20
25
30
35
40
45
50
55
60
'
;
.
which
her
Mr.
,
of
and
)
Lanny
(
She
upon
'd
?
she
Mrs.
's
...
little
....
around
!
/
he
phone
shall
're
de
television
great
anymore
Hon
onto
area
back
'm
shit
TV
computer
okay
old
that
someone
:
the
all
"
just
Alex
U.S.
1980
v.
inside
Okay
-
1978
fucking
came
English
Fiction
V2

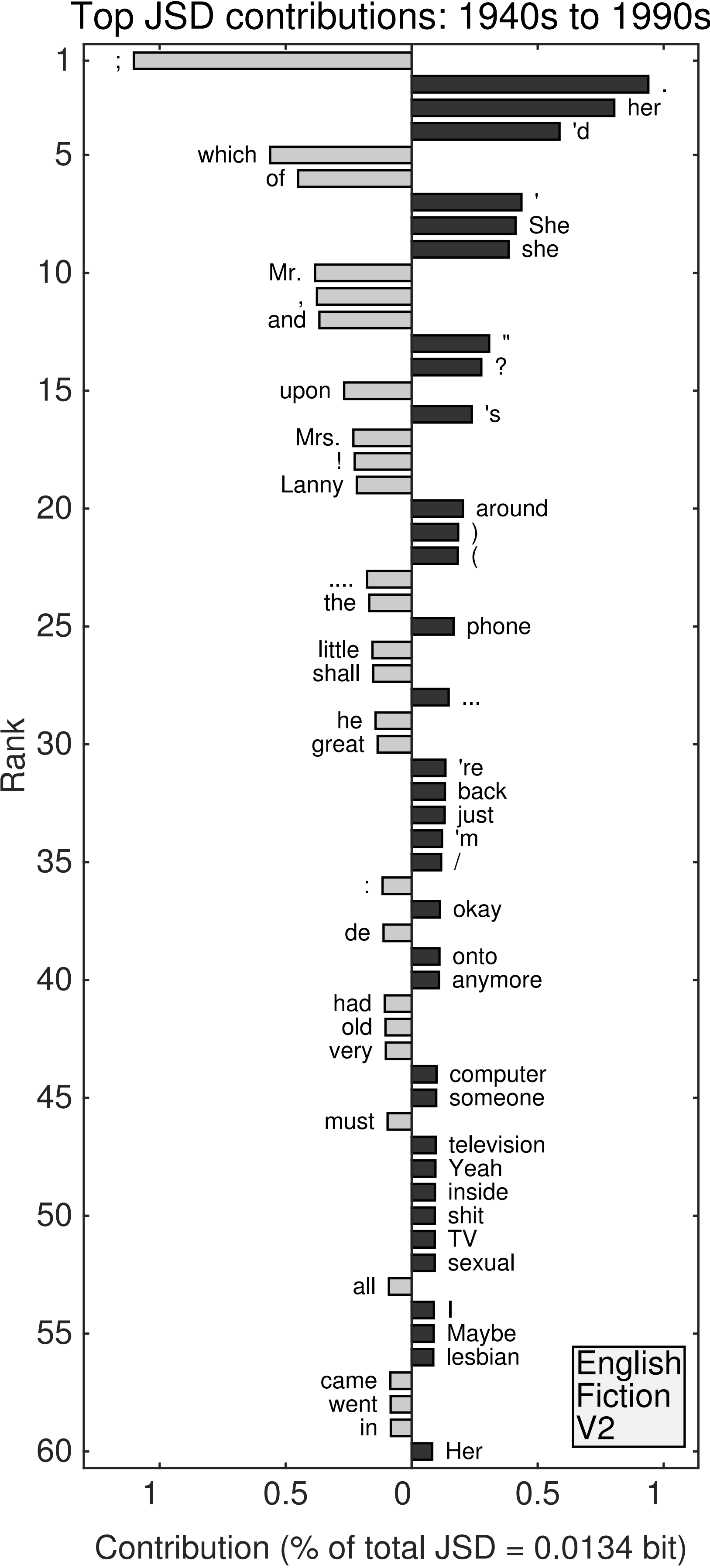
Top JSD contributions: 1940s to 1990s
Rank
Contribution (% of total JSD = 0.0134 bit)
English Fiction V2
;
.
her
'd
which
of
'
She
she
Mr.
,
and
"
?
upon
's
Mrs.
!
Lanny
around
)
(
....
the
phone
little
shall
...
he
great
're
back
just
'm
/
:
okay
de
onto
anymore
had
old
very
computer
someone
must
television
Yeah
inside
shit
TV
sexual
all
I
Maybe
lesbian
came
went
in
Her

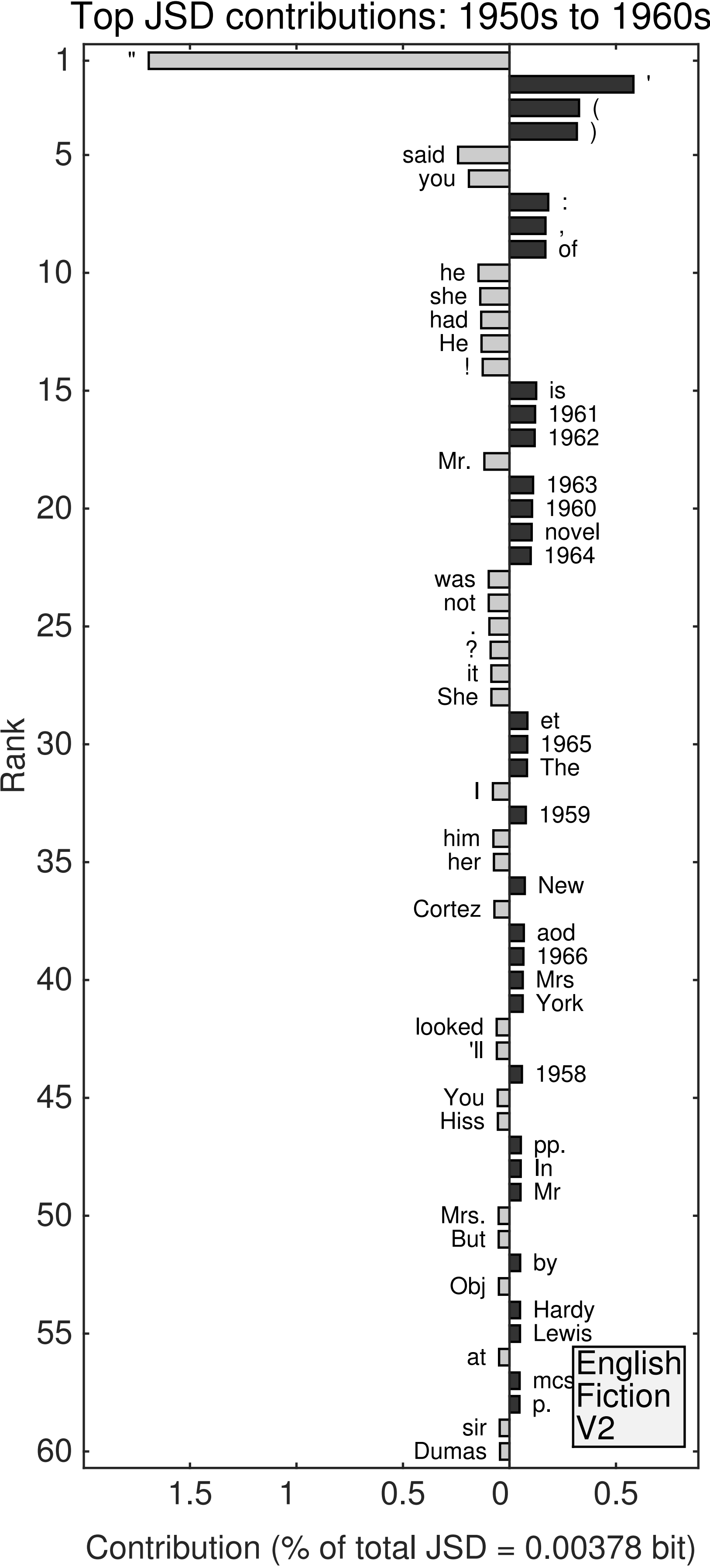

Top JSD contributions: 1950s to 1960s
Rank
Contribution (% of total JSD = 0.00378 bit)
English Fiction V2
" ' ( ) said you : , of he she had He ! is 1961 1962 Mr. 1963 1960 novel 1964 was not . ? it She et 1965 The I 1959 him her New Cortez aod 1966 Mrs York looked 'll 1958 You Hiss pp. In Mr Mrs. But by Obj Hardy Lewis at mcs p. sir Dumas

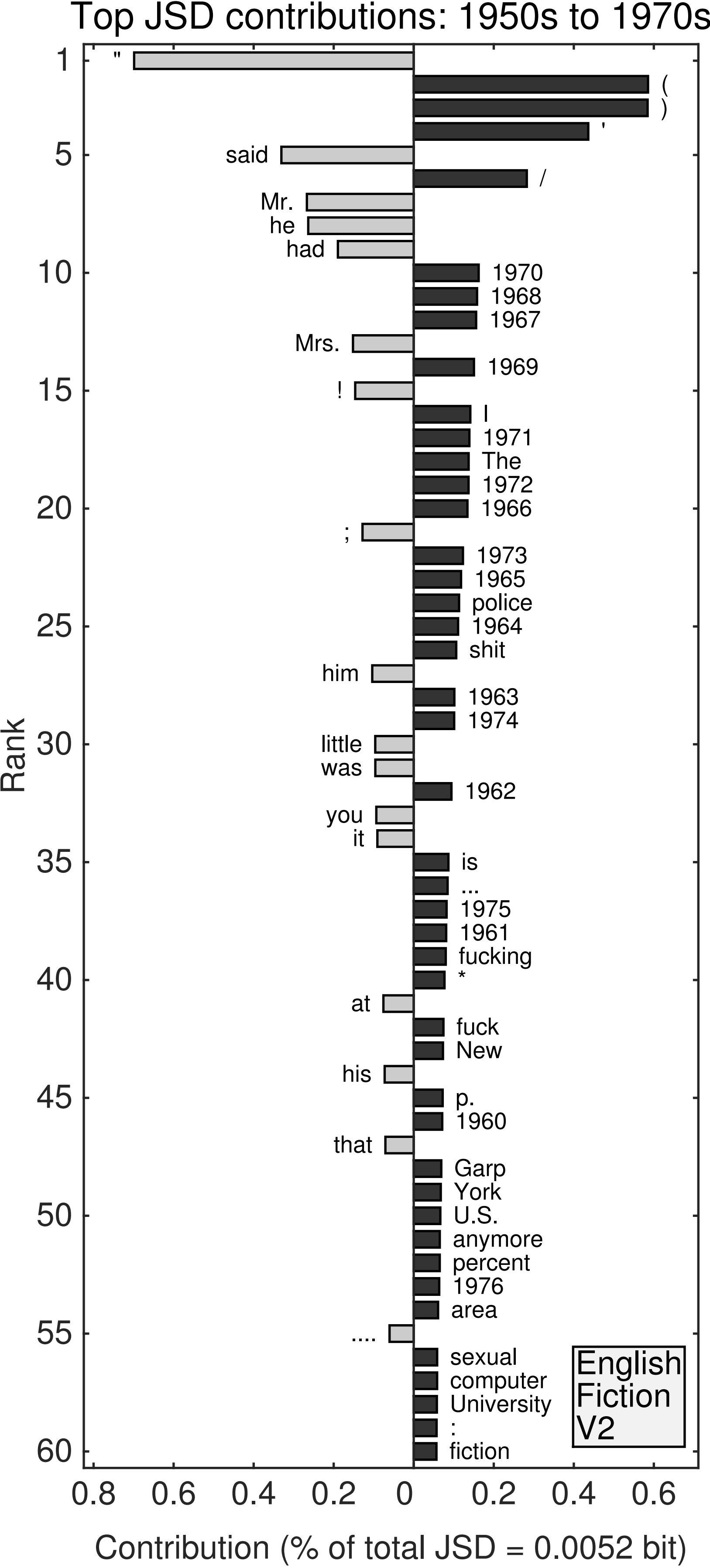
Top JSD contributions: 1950s to 1970s
Rank
Contribution (% of total JSD = 0.0052 bit)
English Fiction V2
"
(
)
'
said
/
Mr.
he
had
1970
1968
1967
Mrs.
1969
!
I
1971
The
1972
1966
;
1973
1965
police
1964
shit
him
1963
1974
little
was
1962
you
it
is
...
1975
1961
fucking
*
at
fuck
New
his
p.
1960
that
Garp
York
U.S.
anymore
percent
1976
area
....
sexual
computer
University
:
fiction

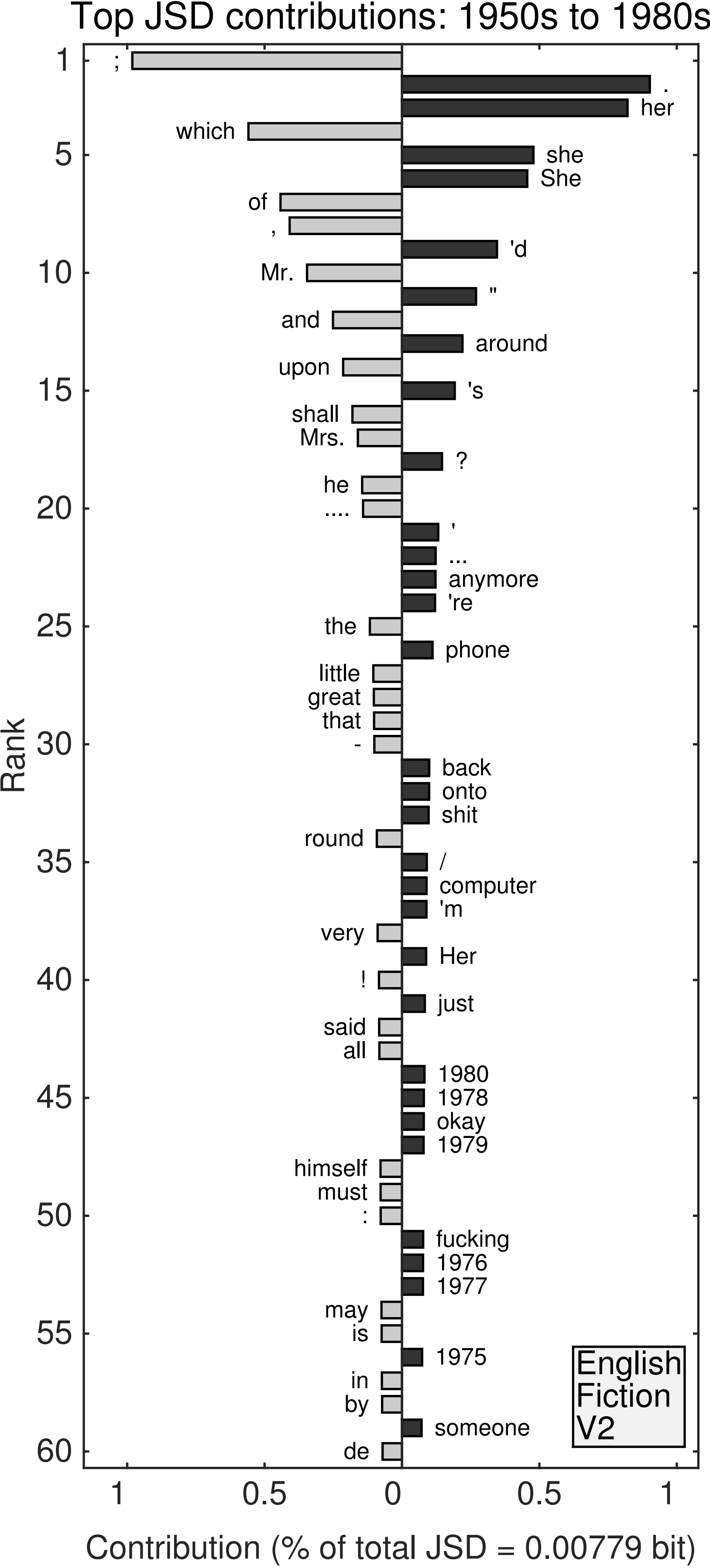
Top JSD contributions: 1950s to 1980s
Rank
Contribution (% of total JSD = 0.00779 bit)
English Fiction V2
;
.
her
which
she
She
of
,
'd
Mr.
"
and
around
upon
's
shall
Mrs.
?
he
....
'
...
anymore
're
the
phone
little
great
that
-
back
onto
shit
round
/
computer
'm
very
Her
!
just
said
all
1980
1978
okay
1979
himself
must
:
fucking
1976
1977
may
is
1975
in
by
someone
de
1
5
10
15
20
25
30
35
40
45
50
55
60
1
0.5
0
0.5
1

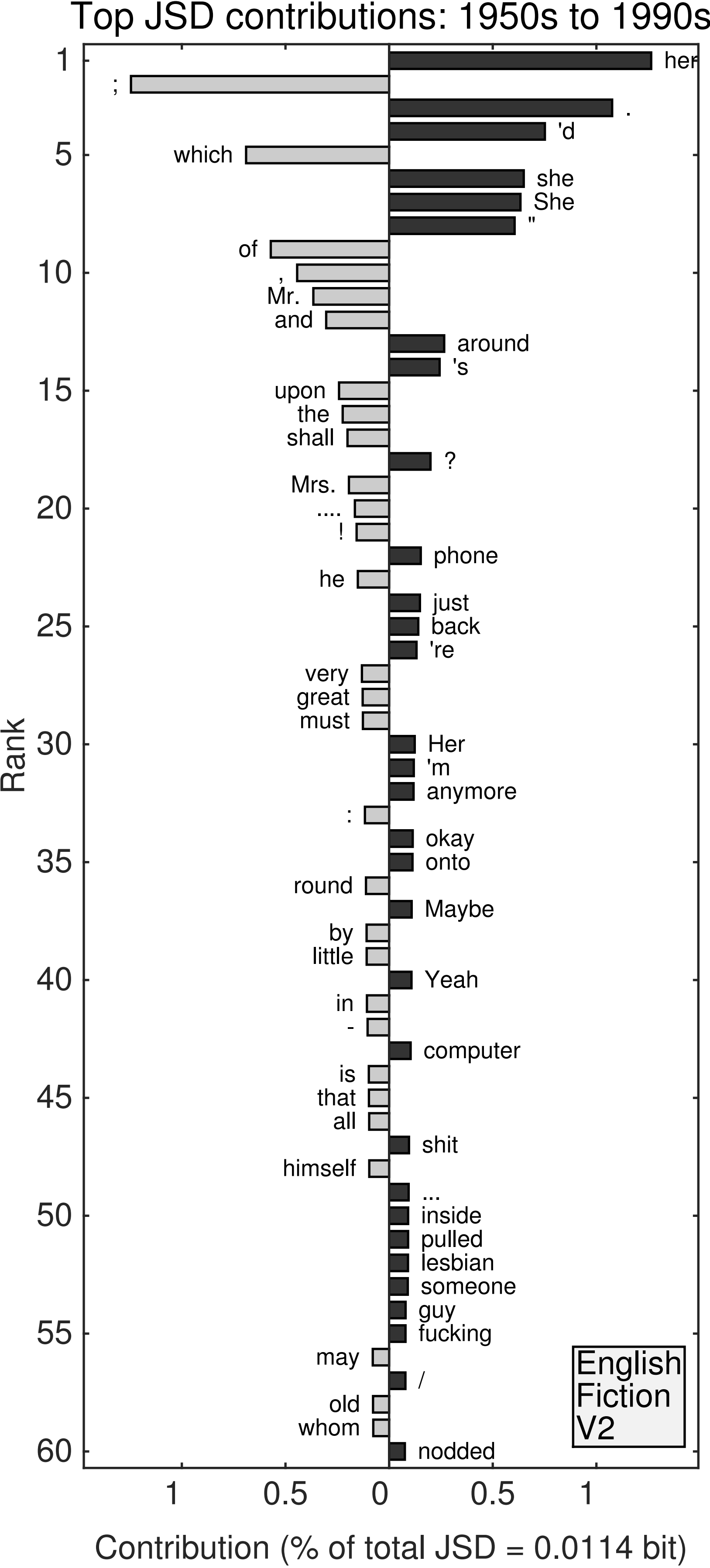

Top JSD contributions: 1950s to 1990s
Rank
Contribution (% of total JSD = 0.0114 bit)
1
5
10
15
20
25
30
35
40
45
50
55
60
1
0.5
0
0.5
1
her
;
.
'd
which
she
She
"
of
,
Mr.
and
around
's
upon
the
shall
?
Mrs.
....
!
phone
he
just
back
're
very
great
must
Her
'm
anymore
:
okay
onto
round
Maybe
by
little
Yeah
in
-
computer
is
that
all
shit
himself
...
inside
pulled
lesbian
someone
guy
fucking
may
/
old
whom
nodded
English
Fiction
V2

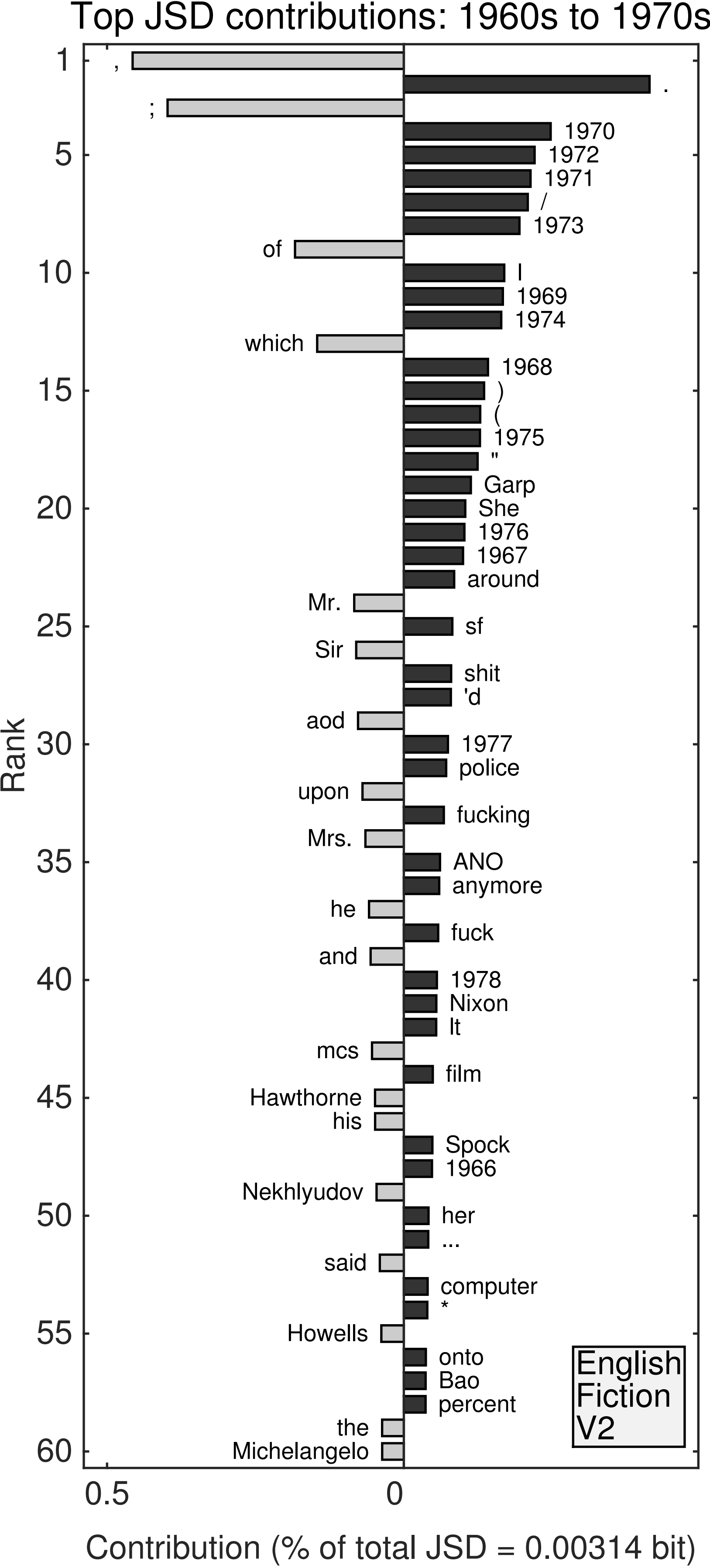

Top JSD contributions: 1960s to 1970s
Rank
Contribution (% of total JSD = 0.00314 bit)
0.5
0
1
5
10
15
20
25
30
35
40
45
50
55
60
,
.
;
1970
1972
1971
/
1973
of
I
1969
1974
which
1968
)
(
1975
"
Garp
She
1976
1967
around
Mr.
sf
Sir
shit
'd
aod
1977
police
upon
fucking
Mrs.
ANO
anymore
he
fuck
and
1978
Nixon
It
mcs
film
Hawthorne
his
Spock
1966
Nekhlyudov
her
...
said
computer
*
Howells
onto
Bao
percent
the
Michelangelo
English Fiction V2

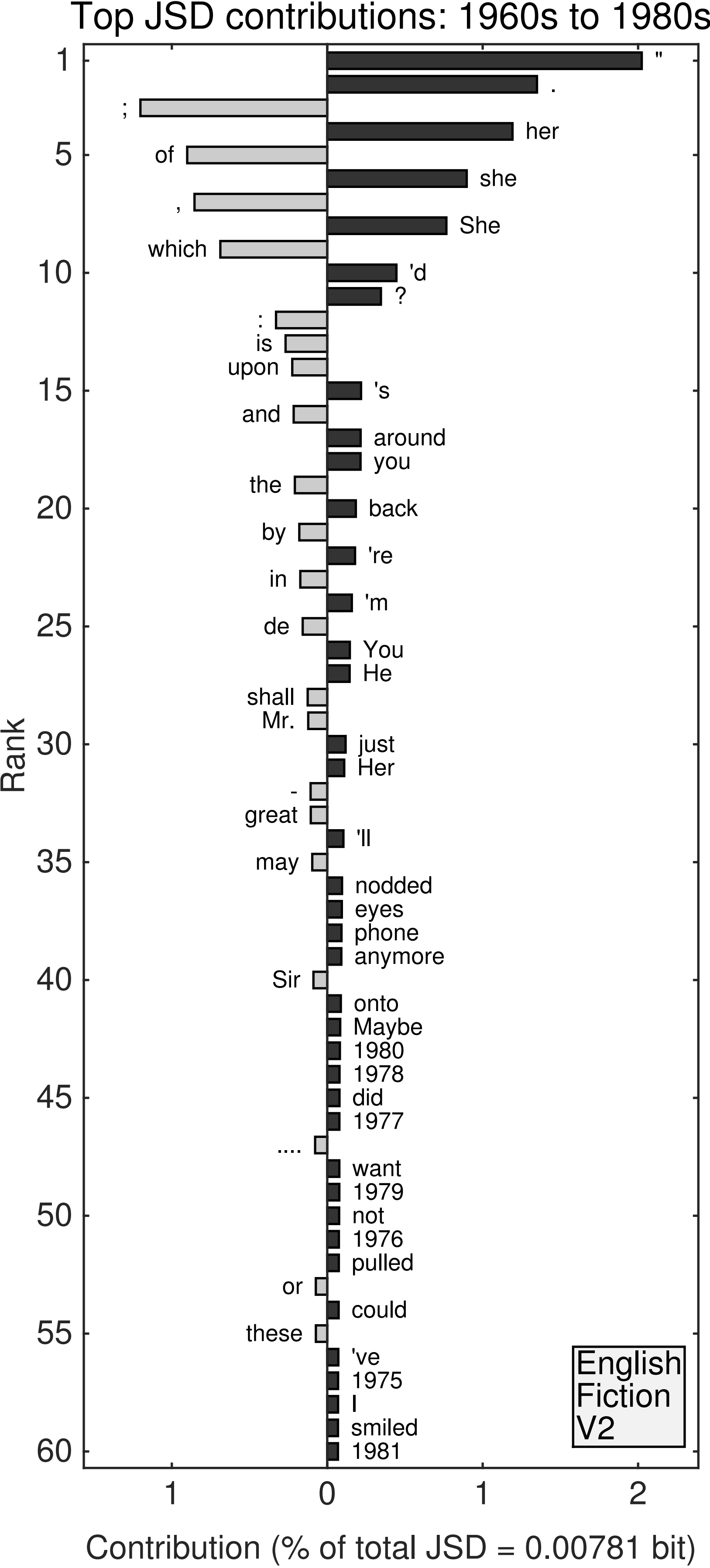

Top JSD contributions: 1960s to 1980s
Rank
Contribution (% of total JSD = 0.00781 bit)
English Fiction V2
"
.
;
her
of
she
,
She
which
'd
?
:
is
upon
's
and
around
you
the
back
by
're
in
'm
de
You
He
shall
Mr.
just
Her
-
great
'll
may
nodded
eyes
phone
anymore
Sir
onto
Maybe
1980
1978
did
1977
....
want
1979
not
1976
pulled
or
could
these
've
1975
I
smiled
1981

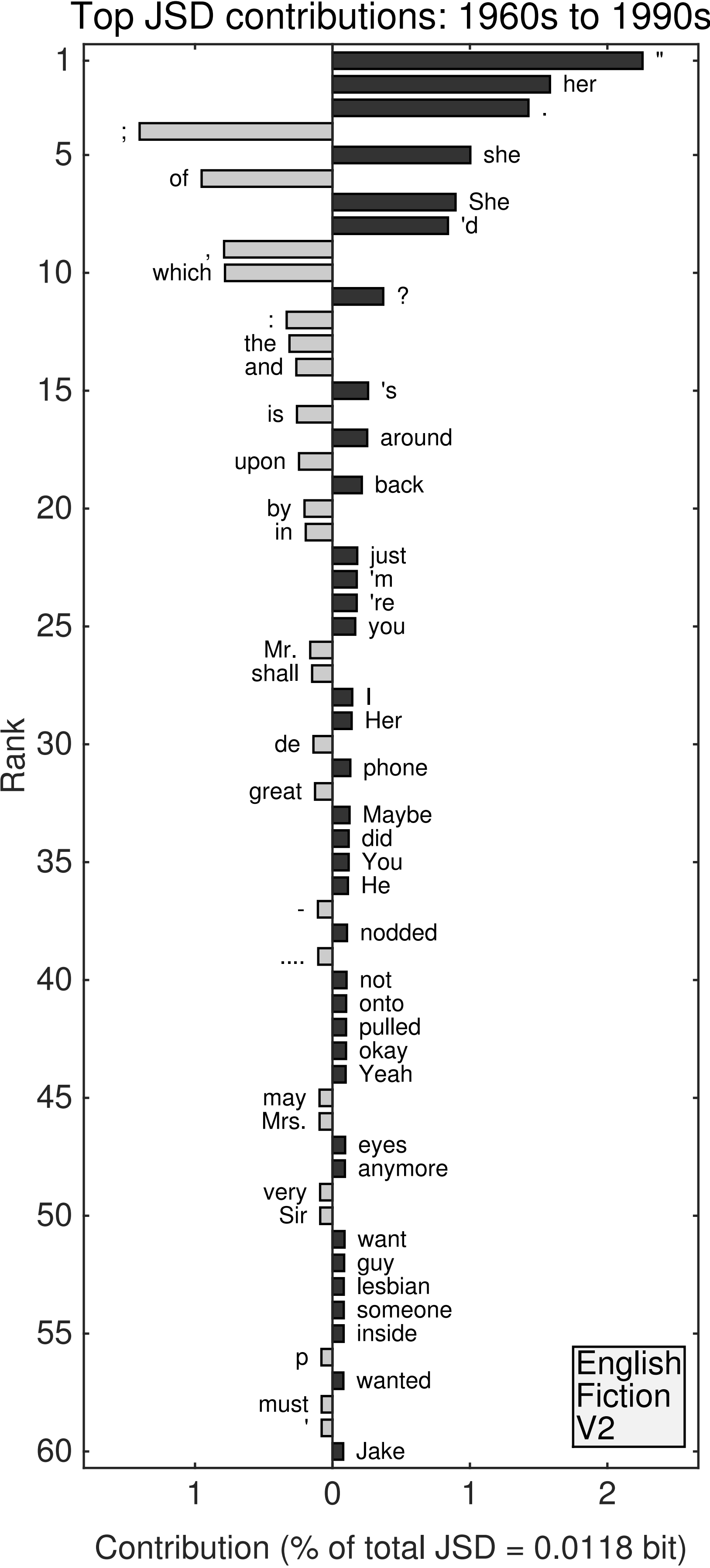

Top JSD contributions: 1960s to 1990s
Rank
Contribution (% of total JSD = 0.0118 bit)
English Fiction V2
"
her
.
;
she
of
She
'd
,
which
?
:
the
and
's
is
around
upon
back
by
in
just
'm
're
you
Mr.
shall
I
Her
de
phone
great
Maybe
did
You
He
-
nodded
....
not
onto
pulled
okay
Yeah
may
Mrs.
eyes
anymore
very
Sir
want
guy
lesbian
someone
inside
p
wanted
must
'
Jake

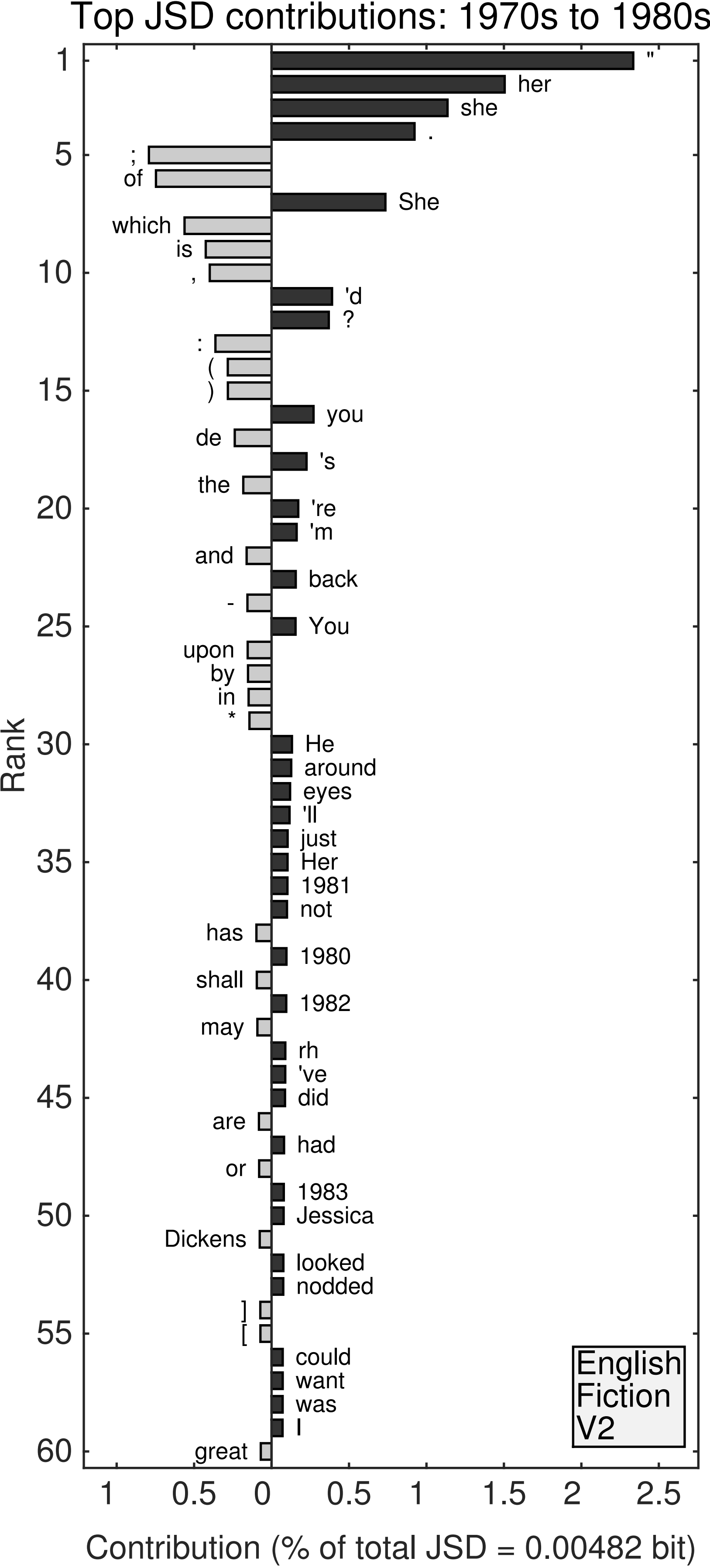

Top JSD contributions: 1970s to 1980s
Rank
Contribution (% of total JSD = 0.00482 bit)
English Fiction V2
"
her
she
.
;
of
She
which
is
,
'd
?
:
(
)
you
de
's
the
're
'm
and
back
-
You
upon
by
in
*
He
around
eyes
'll
just
Her
1981
not
has
1980
shall
1982
may
rh
've
did
are
had
or
1983
Jessica
Dickens
looked
nodded
]
[
could
want
was
I
great

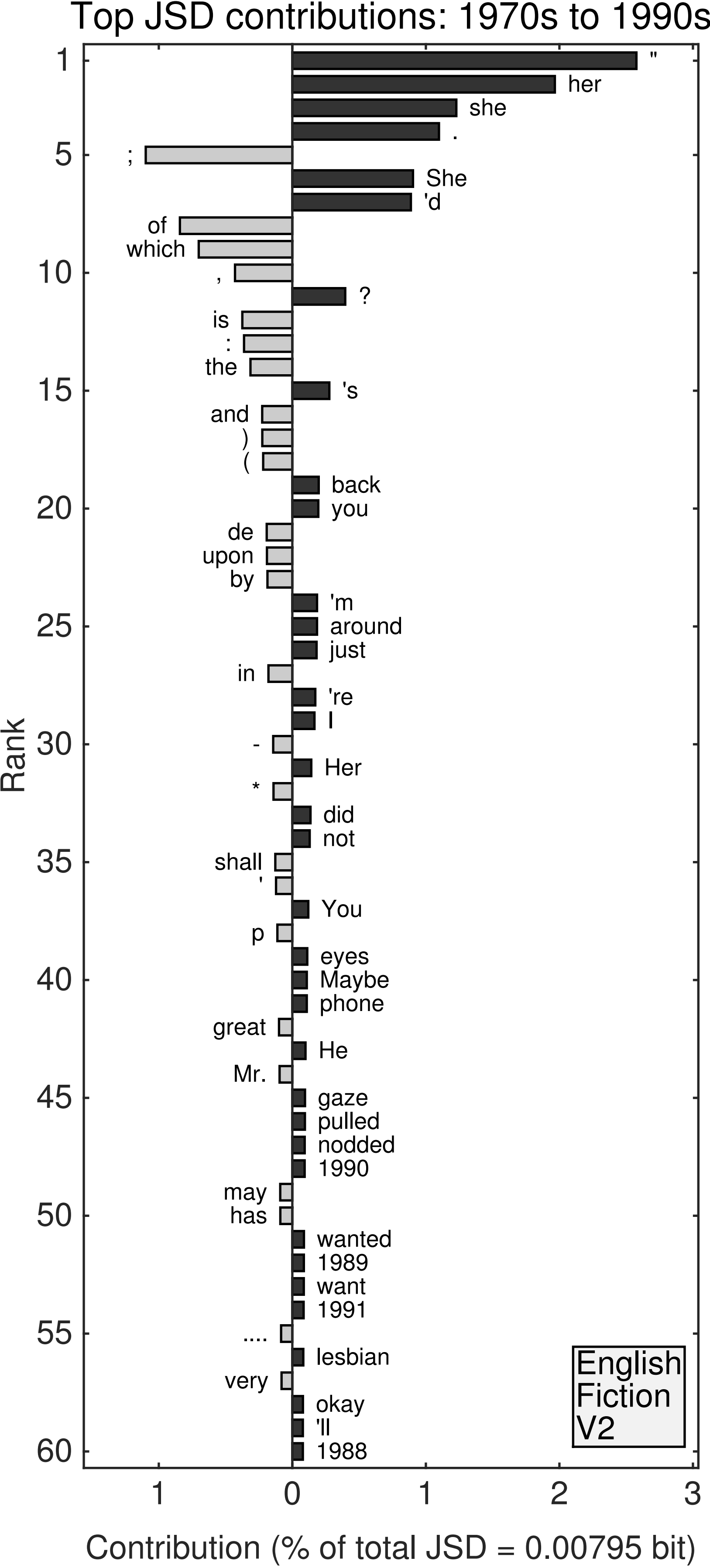
Top JSD contributions: 1970s to 1990s
Rank
Contribution (% of total JSD = 0.00795 bit)
"
her
she
.
;
She
'd
of
which
,
?
is
:
the
's
and
)
(
back
you
de
upon
by
'm
around
just
in
're
I
-
Her
*
did
not
shall
'
You
p
eyes
Maybe
phone
great
He
Mr.
gaze
pulled
nodded
1990
may
has
wanted
1989
want
1991
....
lesbian
very
okay
'll
1988
English
Fiction
V2

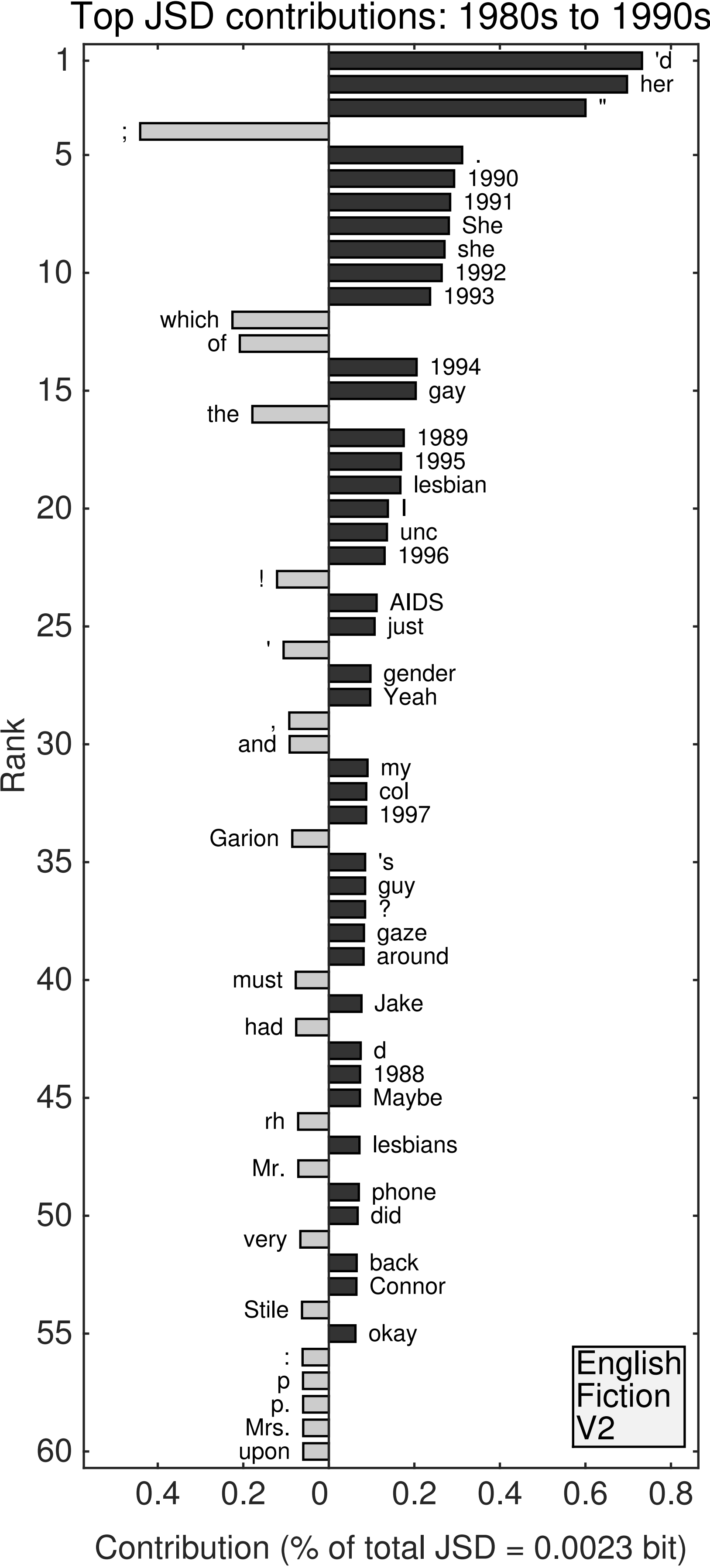
Top JSD contributions: 1980s to 1990s
Rank
'd
her
"
;
.
1990
1991
She
she
1992
1993
which
of
1994
gay
the
1989
1995
lesbian
I
unc
1996
!
AIDS
just
'
gender
Yeah
,
and
my
col
1997
Garion
's
guy
?
gaze
around
must
Jake
had
d
1988
Maybe
rh
lesbians
Mr.
phone
did
very
back
Connor
Stile
okay
:
p
p.
Mrs.
upon
English Fiction V2
Contribution (% of total JSD = 0.0023 bit)
