# Supplementary material for: Characterizing the Google Books Corpus: Strong Limits to Inferences of Socio-Cultural and Linguistic Evolution
Source: PLoS One. 2015 Oct 7;10(10):e0137041. doi: 10.1371/journal.pone.0137041 (PMC4596490; doi:10.1371/journal.pone.0137041)
Supplement: S4 File — (PDF) [file pone.0137041.s004.pdf]

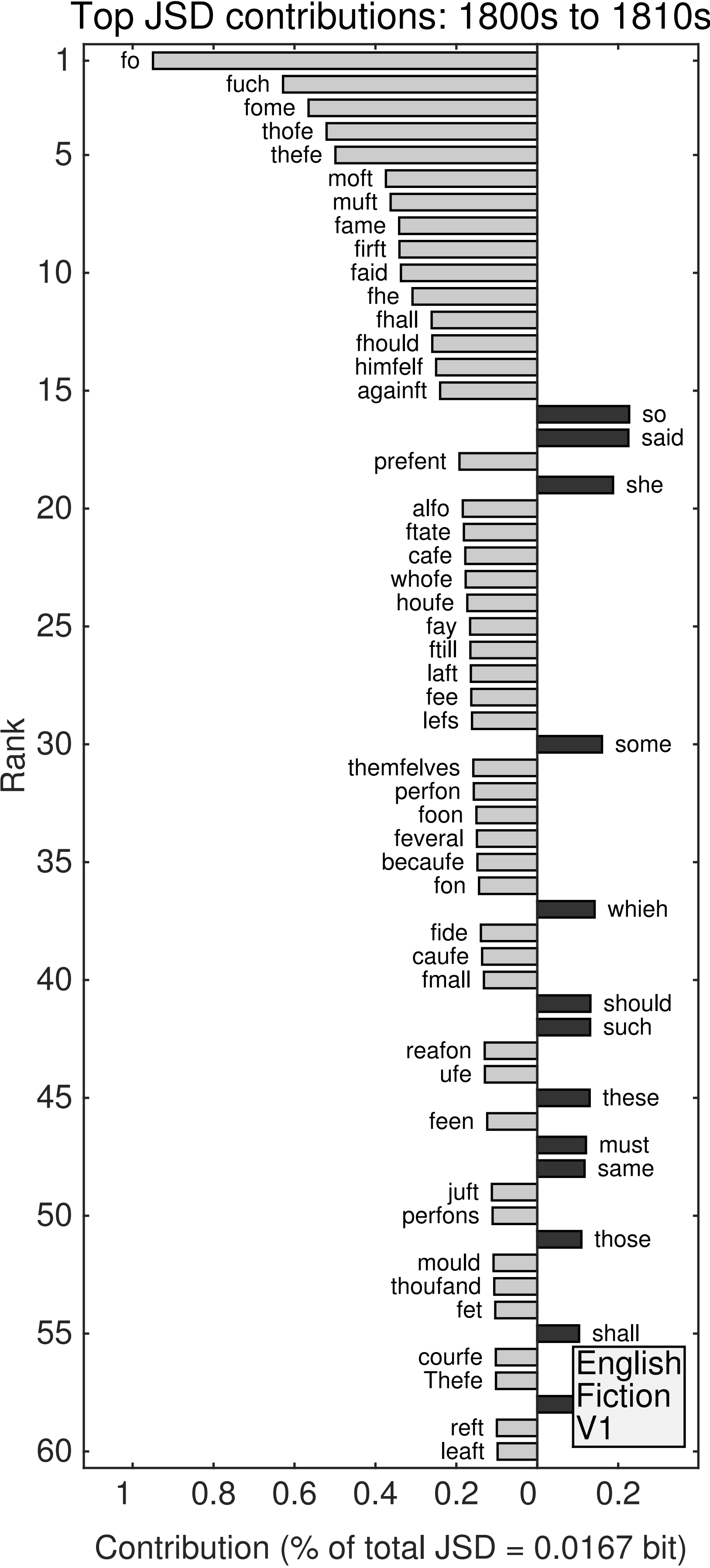
Top JSD contributions: 1800s to 1810s
Rank
Contribution (% of total JSD = 0.0167 bit)
1
5
10
15
20
25
30
35
40
45
50
55
60
1
0.8
0.6
0.4
0.2
0
0.2
fo
fuch
fome
thofe
thefe
moft
muft
fame
firft
faid
fhe
fhall
fhould
himfelf
againft
so
said
prefent
she
alfo
ftate
cafe
whofe
houfe
fay
ftill
laft
fee
lefs
some
themfelves
perfon
foon
feveral
becaufe
fon
whieh
fide
caufe
fmall
should
such
reafon
ufe
these
feen
must
same
juft
perfons
those
mould
thoufand
fet
shall
courfe
Thefe
reft
leaft
English
Fiction
V1

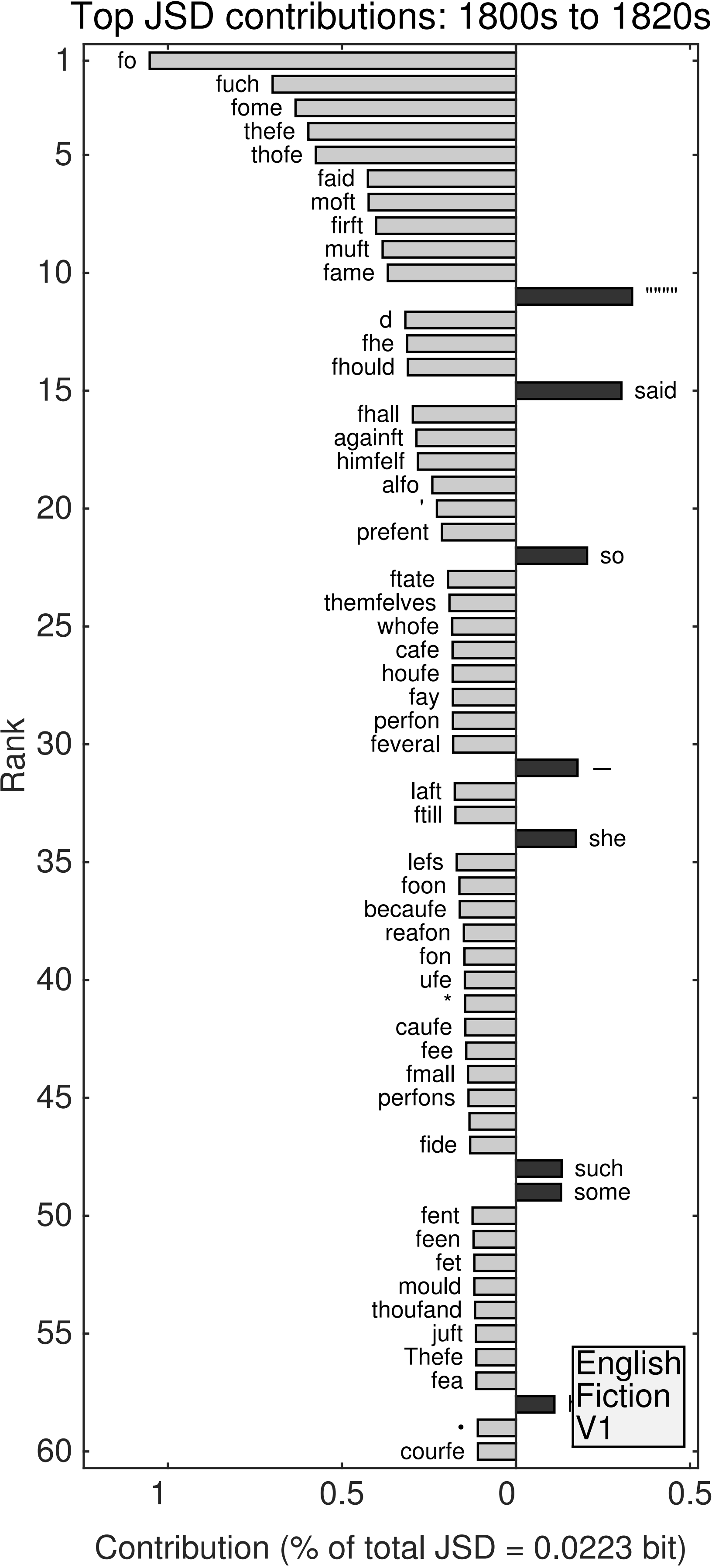

Top JSD contributions: 1800s to 1820s
Rank
Contribution (% of total JSD = 0.0223 bit)
fo
fuch
fome
thefe
thofe
faid
moft
firft
muft
fame
""""
d
fhe
fhould
said
fhall
againft
himfelf
alfo
'
prefent
so
ftate
themfelves
whofe
cafe
houfe
fay
perfon
feveral
—
laft
ftill
she
lefs
foon
becaufe
reafon
fon
ufe
*
caufe
fee
fmall
perfons
fide
such
some
fent
feen
fet
mould
thoufand
juft
Thefe
fea
•
courfe
English Fiction V1

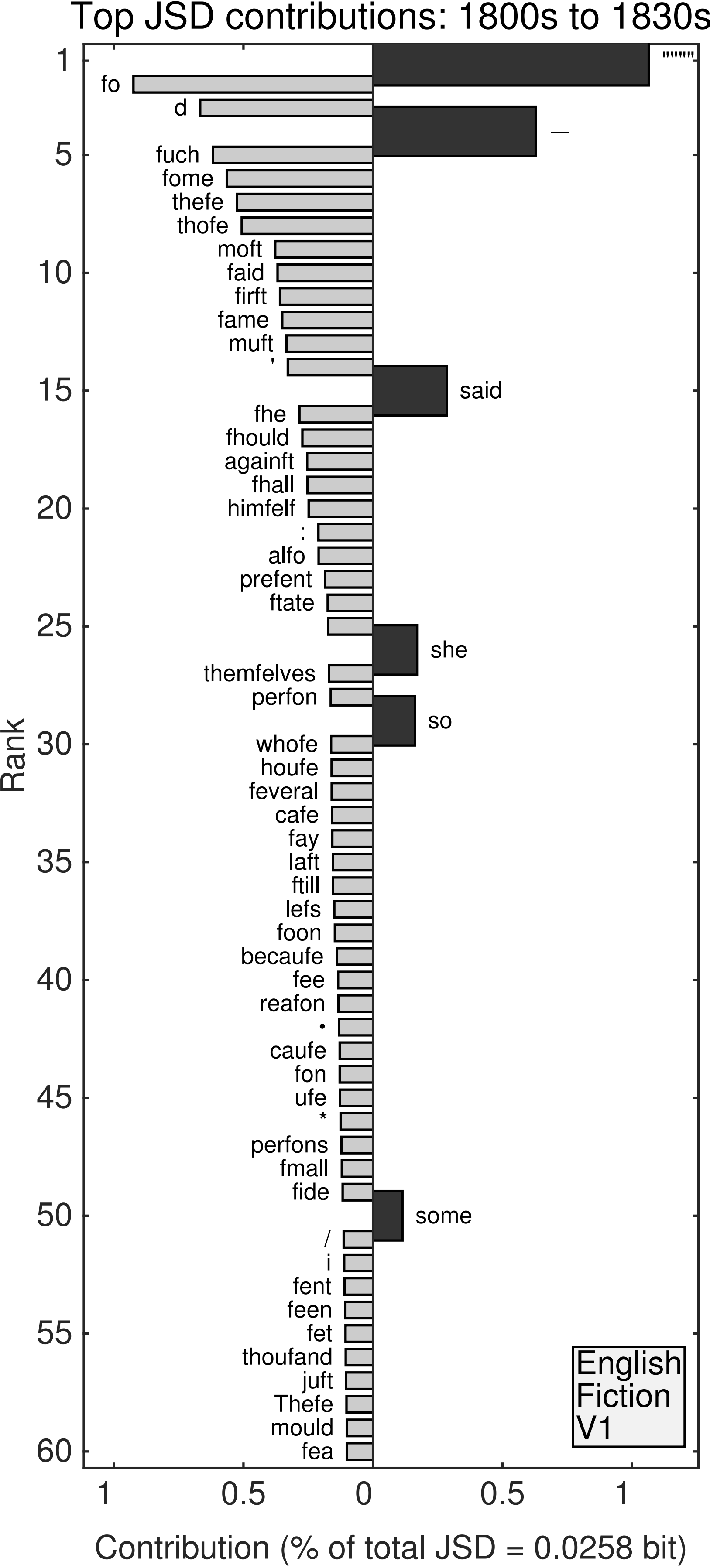
Top JSD contributions: 1800s to 1830s
Rank
Contribution (% of total JSD = 0.0258 bit)
English Fiction V1
"""""
fo
d
—
fuch
fome
thefe
thofe
moft
faid
firft
fame
muft
'
said
fhe
fhould
againft
fhall
himfelf
:
alfo
prefent
ftate
she
themfelves
perfon
so
whofe
houfe
feveral
cafe
fay
laft
ftill
lefs
foon
becaufe
fee
reafon
•
caufe
fon
ufe
*
perfons
fmall
fide
some
/
i
fent
feen
fet
thoufand
juft
Thefe
mould
fea

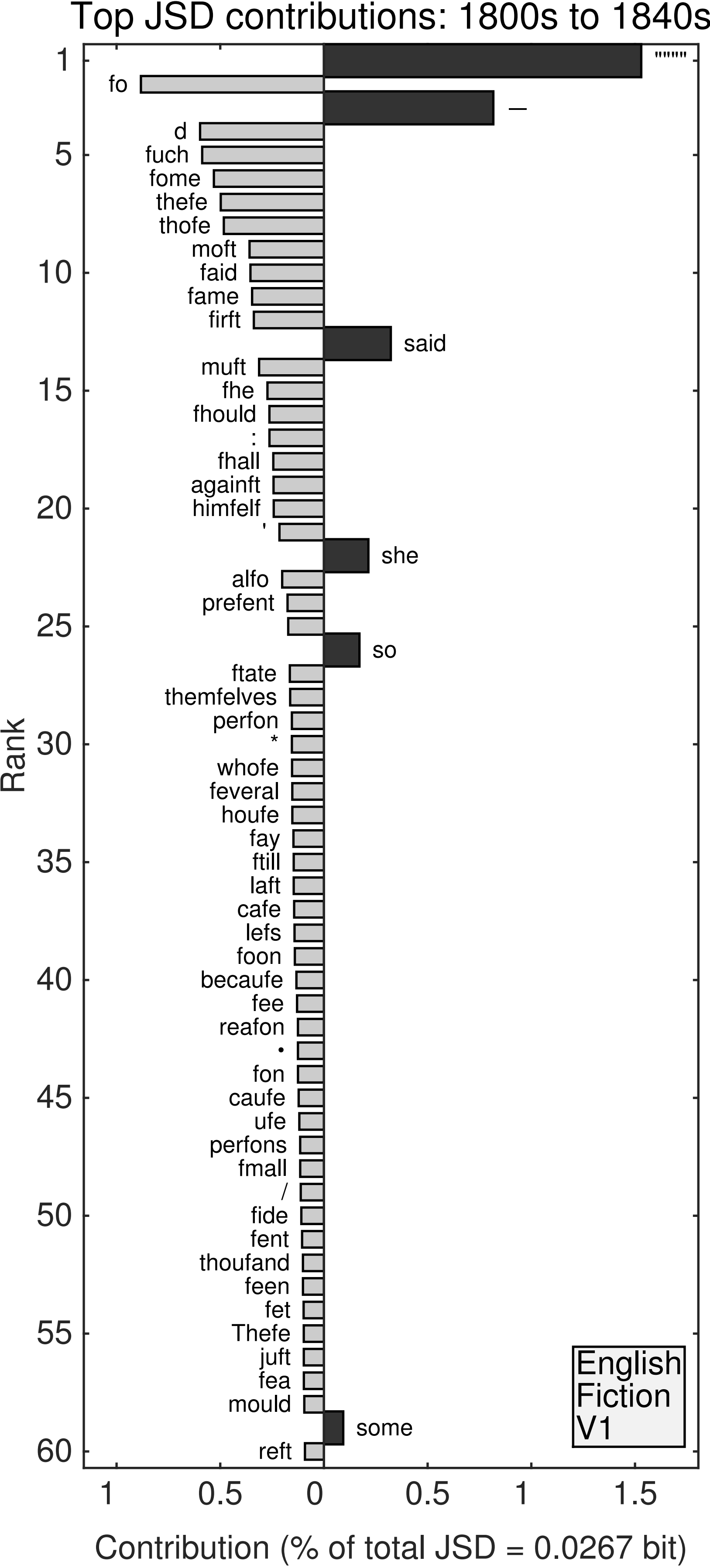

Top JSD contributions: 1800s to 1840s
Rank
Contribution (% of total JSD = 0.0267 bit)
English Fiction V1
"""""
fo
—
d
fuch
fome
thefe
thofe
moft
faid
fame
firft
said
muft
fhe
fhould
:
fhall
againft
himfelf
'
she
alfo
prefent
so
ftate
themfelves
perfon
*
whofe
feveral
houfe
fay
ftill
laft
cafe
lefs
foon
becaufe
fee
reafon
•
fon
caufe
ufe
perfons
fmall
/
fide
fent
thoufand
feen
fet
Thefe
juft
fea
mould
some
reft

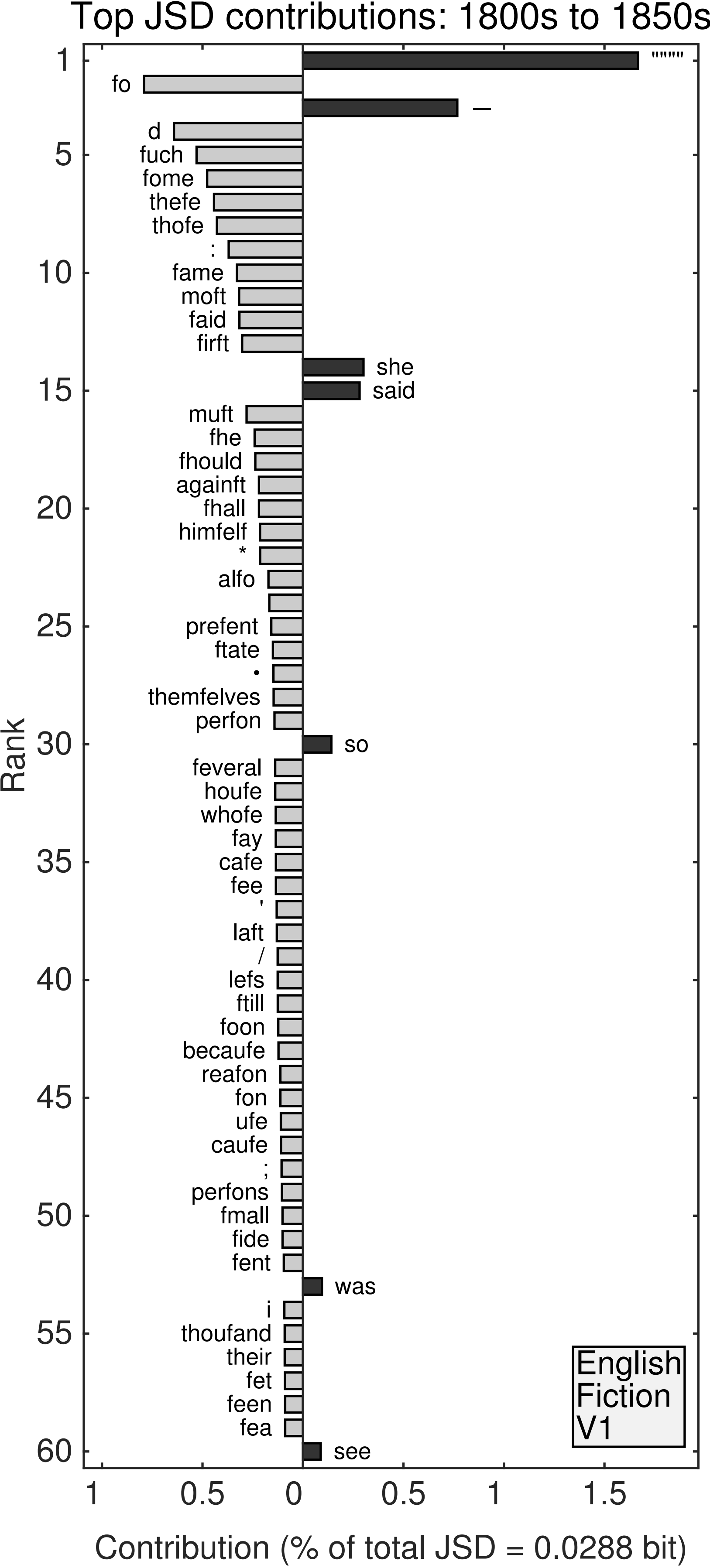
Top JSD contributions: 1800s to 1850s
Rank
1
5
10
15
20
25
30
35
40
45
50
55
60
""""
fo
—
d
fuch
fome
thefe
thofe
:
fame
moft
faid
firft
she
said
muft
fhe
fhould
againft
fhall
himfelf
*
alfo
prefent
ftate
•
themfelves
perfon
so
feveral
houfe
whofe
fay
cafe
fee
'
laft
/
lefs
ftill
foon
becaufe
reafon
fon
ufe
caufe
;
perfons
fmall
fide
fent
was
i
thoufand
their
fet
feen
fea
see
English Fiction V1
1
0.5
0
0.5
1
1.5
Contribution (% of total JSD = 0.0288 bit)

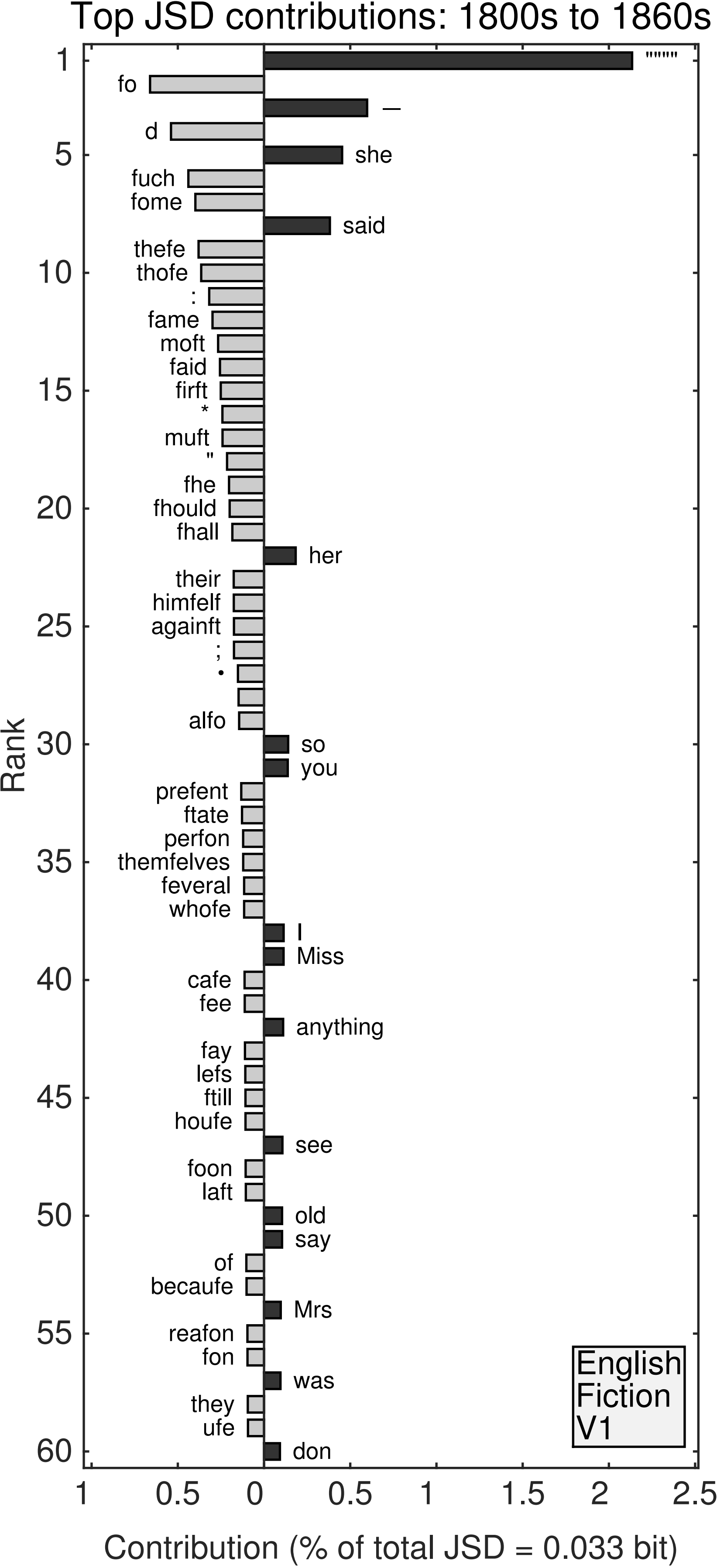
Top JSD contributions: 1800s to 1860s
Rank
"""
fo
—
d
she
fuch
fome
said
thefe
thofe
:
fame
moft
faid
firft
*
muft
"
fhe
fhould
fhall
her
their
himfelf
againft
;
•
alfo
so
you
prefent
ftate
perfon
themfelves
feveral
whofe
I
Miss
cafe
fee
anything
fay
lefs
ftill
houfe
see
foon
laft
old
say
of
becaufe
Mrs
reafon
fon
was
they
ufe
don
English Fiction V1
Contribution (% of total JSD = 0.033 bit)

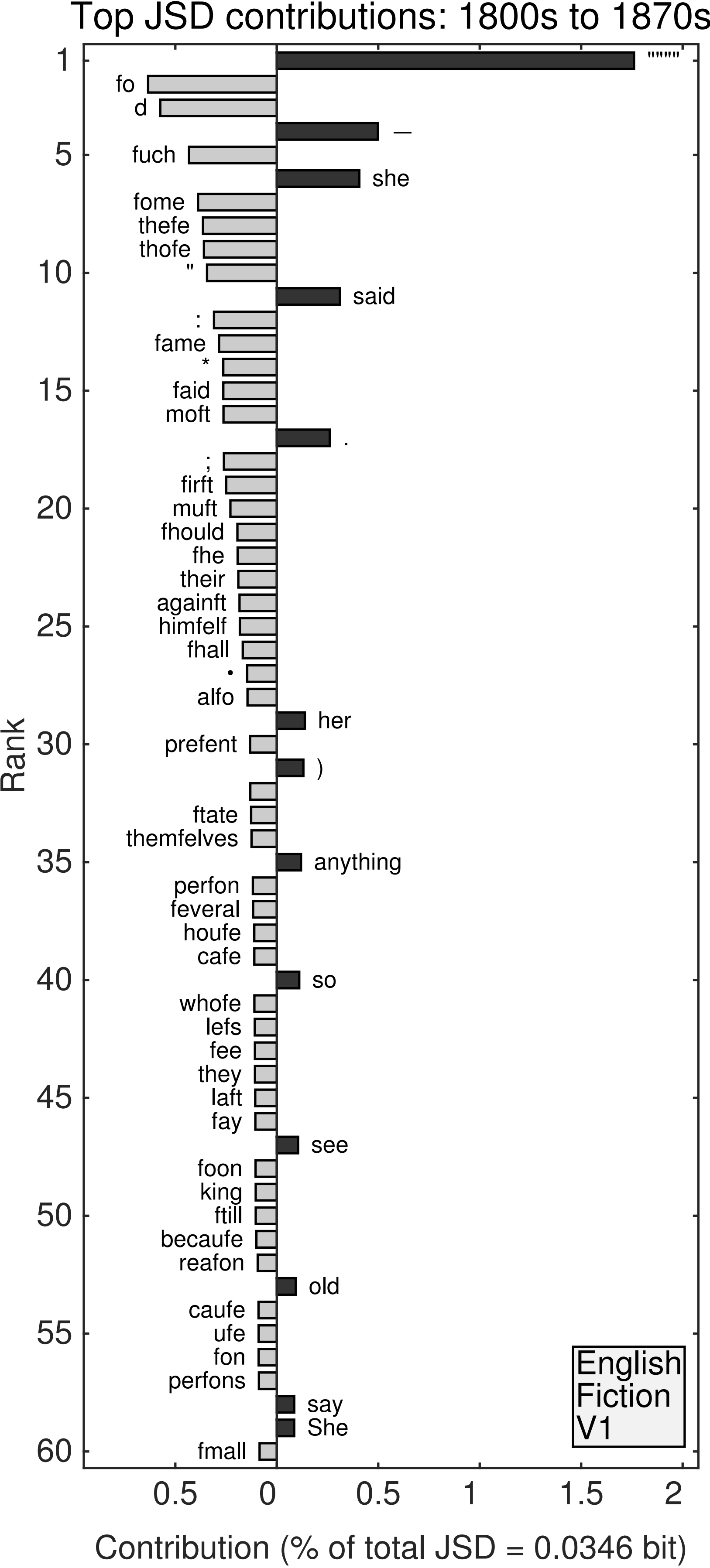

Top JSD contributions: 1800s to 1870s
Rank
Contribution (% of total JSD = 0.0346 bit)
"""""
fo
d
—
fuch
she
fome
thefe
thofe
"
said
:
fame
*
faid
moft
.
;
firft
muft
fhould
fhe
their
againft
himfelf
fhall
•
alfo
her
prefent
)
ftate
themfelves
anything
perfon
feveral
houfe
cafe
so
whofe
lefs
fee
they
laft
fay
see
foon
king
ftill
becaufe
reafon
old
caufe
ufe
fon
perfons
say
She
fmall
English Fiction V1

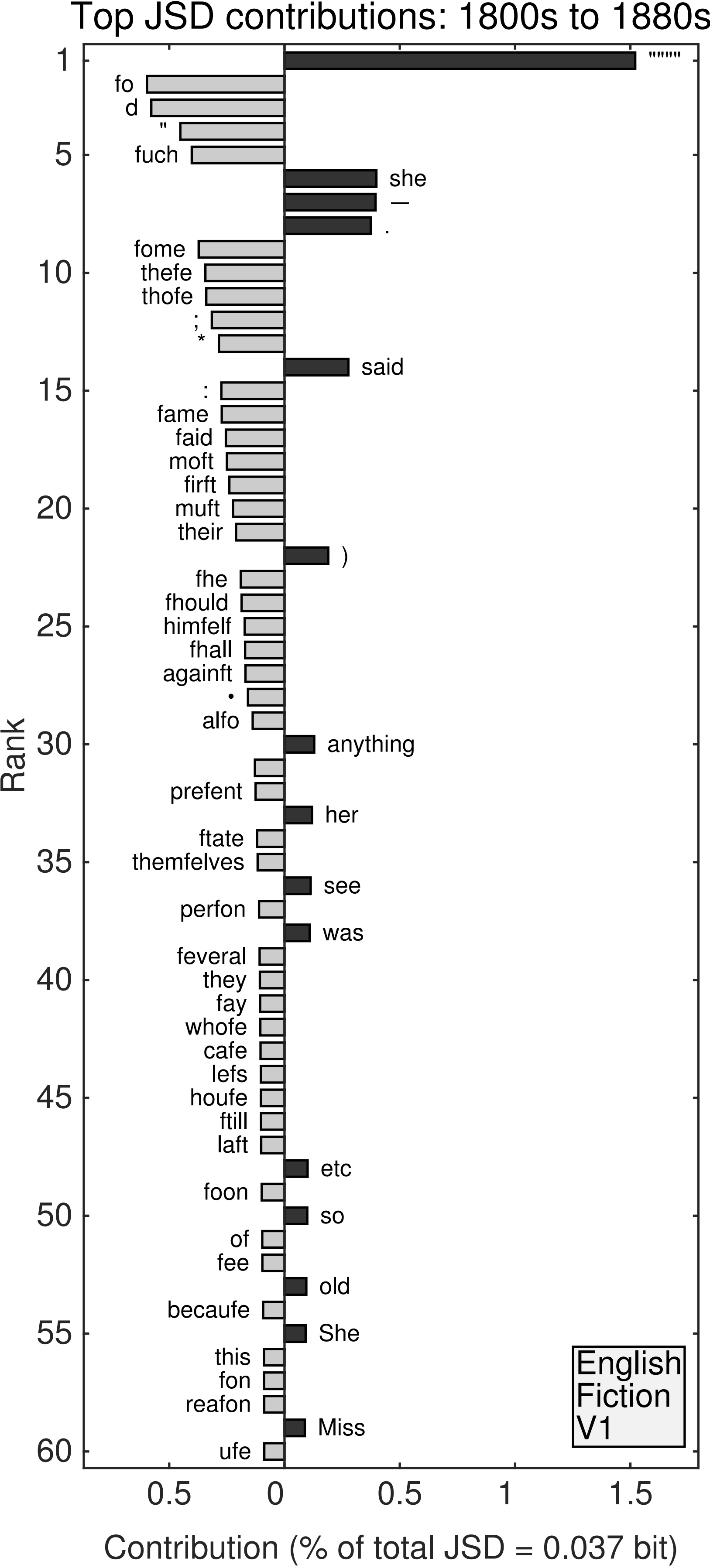

Top JSD contributions: 1800s to 1880s
Rank
Contribution (% of total JSD = 0.037 bit)
""""
fo
d
"
fuch
she
—
.
fome
thefe
thofe
;
*
said
:
fame
faid
moft
firft
muft
their
)
fhe
fhould
himfelf
fhall
againft
•
alfo
anything
prefent
her
ftate
themfelves
see
perfon
was
feveral
they
fay
whofe
cafe
lefs
houfe
ftill
laft
etc
foon
so
of
fee
old
becaufe
She
this
fon
reafon
Miss
ufe
English
Fiction
V1

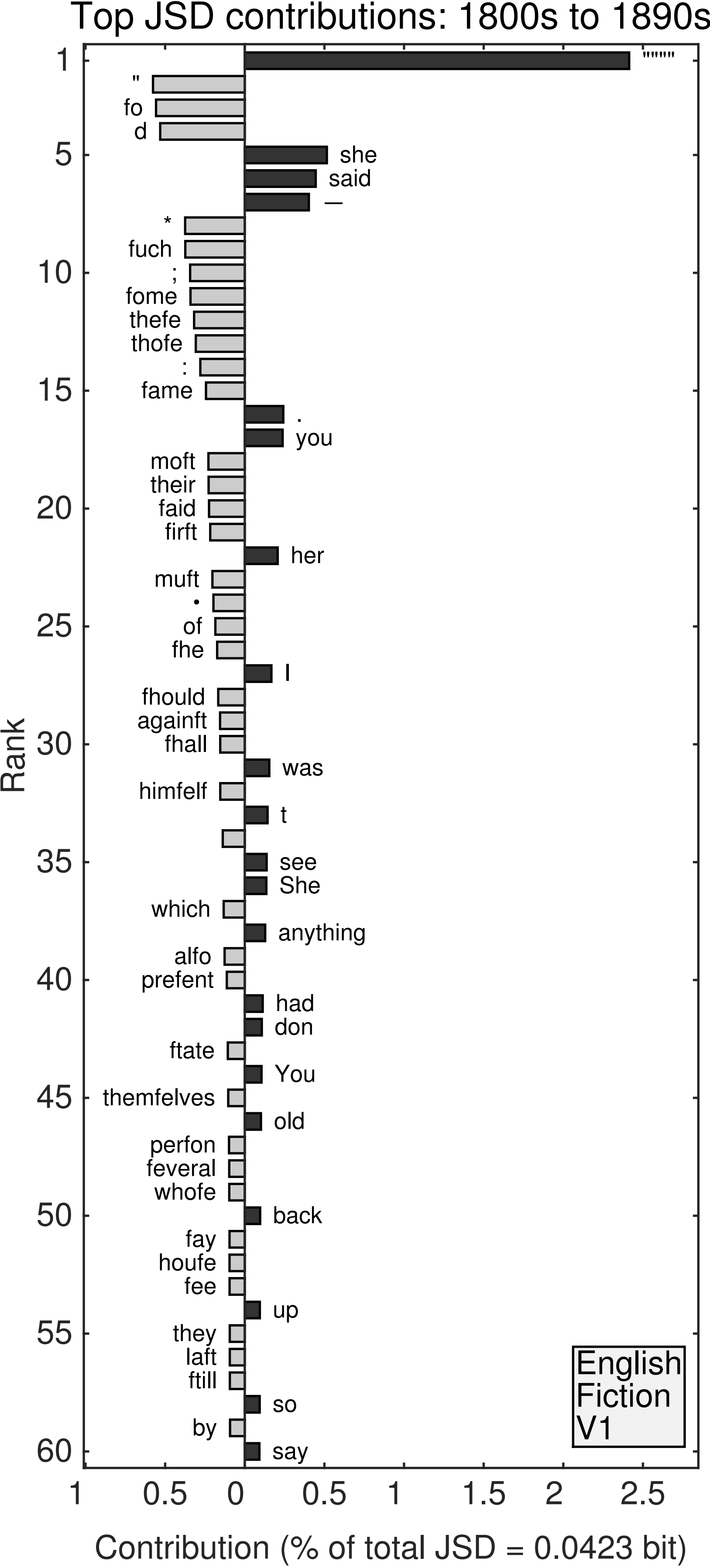

Top JSD contributions: 1800s to 1890s
Rank
Contribution (% of total JSD = 0.0423 bit)
English Fiction V1
"""""
"
fo
d
she
said
—
*
fuch
;
fome
thefe
thofe
:
fame
.
you
moft
their
faid
firft
her
muft
•
of
fhe
I
fhould
againft
fhall
was
himfelf
t
see
She
which
anything
alfo
prefent
had
don
ftate
You
themfelves
old
perfon
feveral
whofe
back
fay
houfe
fee
up
they
laft
ftill
so
by
say

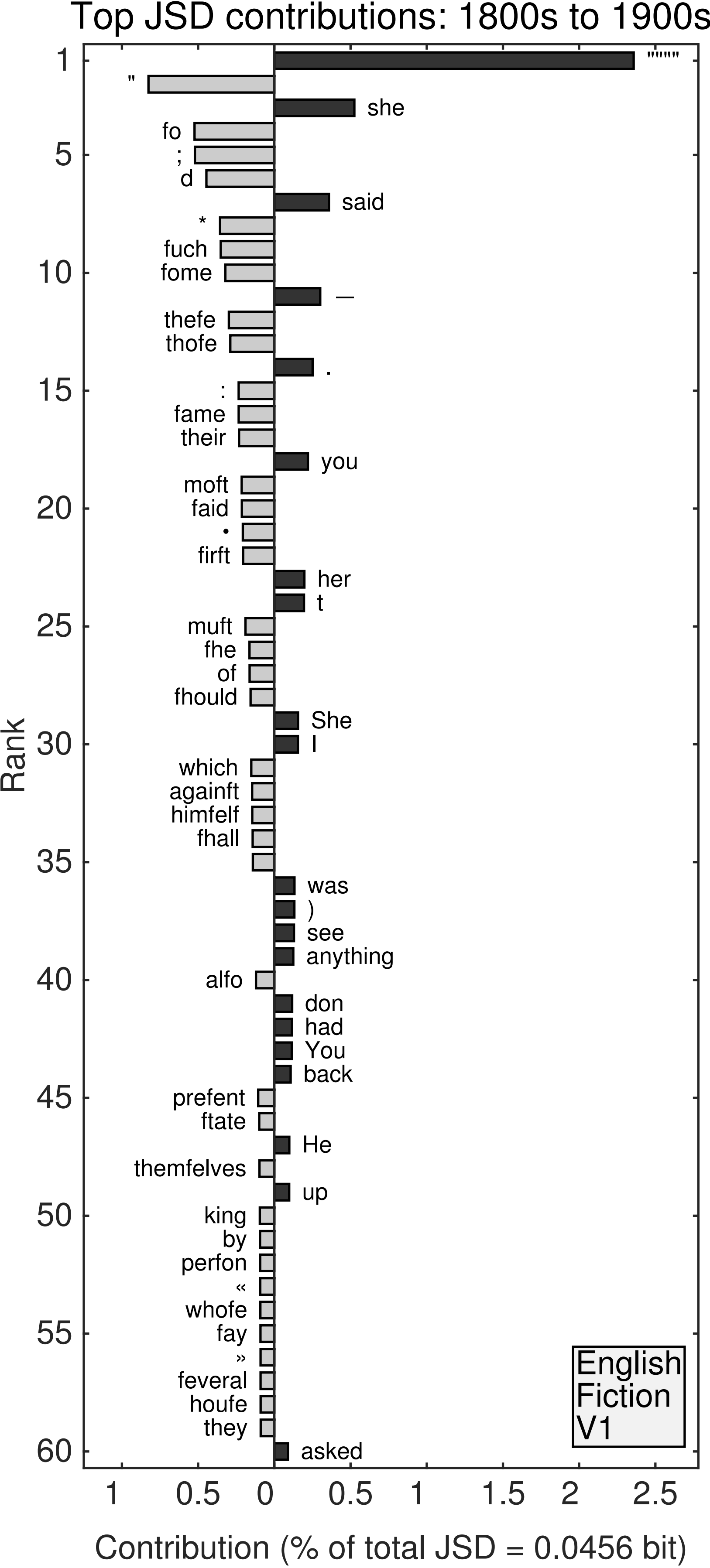

Top JSD contributions: 1800s to 1900s
Rank
Contribution (% of total JSD = 0.0456 bit)
English Fiction V1
"""""
"
she
fo
;
d
said
*
fuch
fome
—
thefe
thofe
.
:
fame
their
you
moft
faid
•
firft
her
t
muft
fhe
of
fhould
She
I
which
againft
himfelf
fhall
was
)
see
anything
alfo
don
had
You
back
prefent
ftate
He
themfelves
up
king
by
perfon
«
whofe
fay
»
feveral
houfe
they
asked

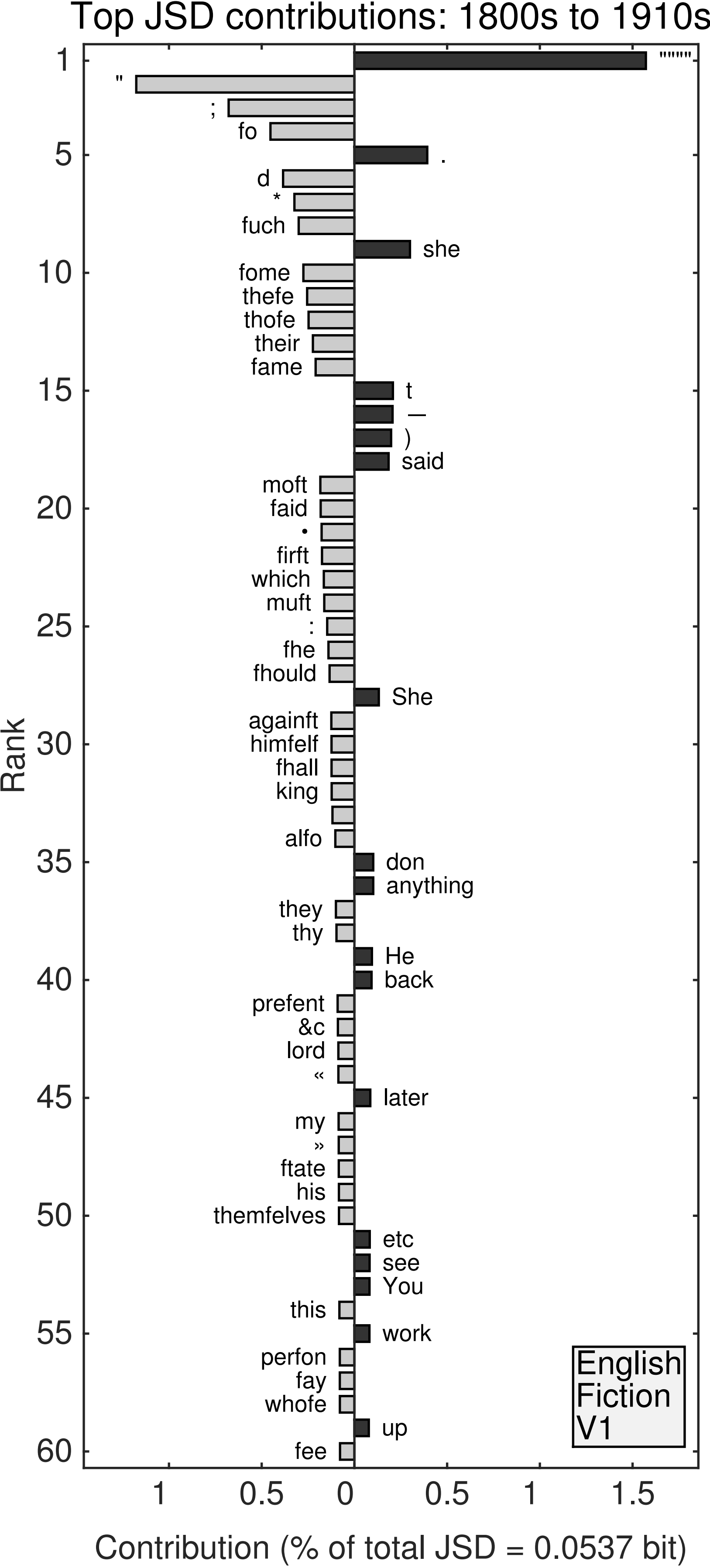

Top JSD contributions: 1800s to 1910s
Rank
Contribution (% of total JSD = 0.0537 bit)
English Fiction V1
"""""
"
;
fo
.
d
*
fuch
she
fome
thefe
thofe
their
fame
t
—
)
said
moft
faid
•
firft
which
muft
:
fhe
fhould
She
againft
himfelf
fhall
king
alfo
don
anything
they
thy
He
back
prefent
&c
lord
«
later
my
»
ftate
his
themfelves
etc
see
You
this
work
perfon
fay
whofe
up
fee

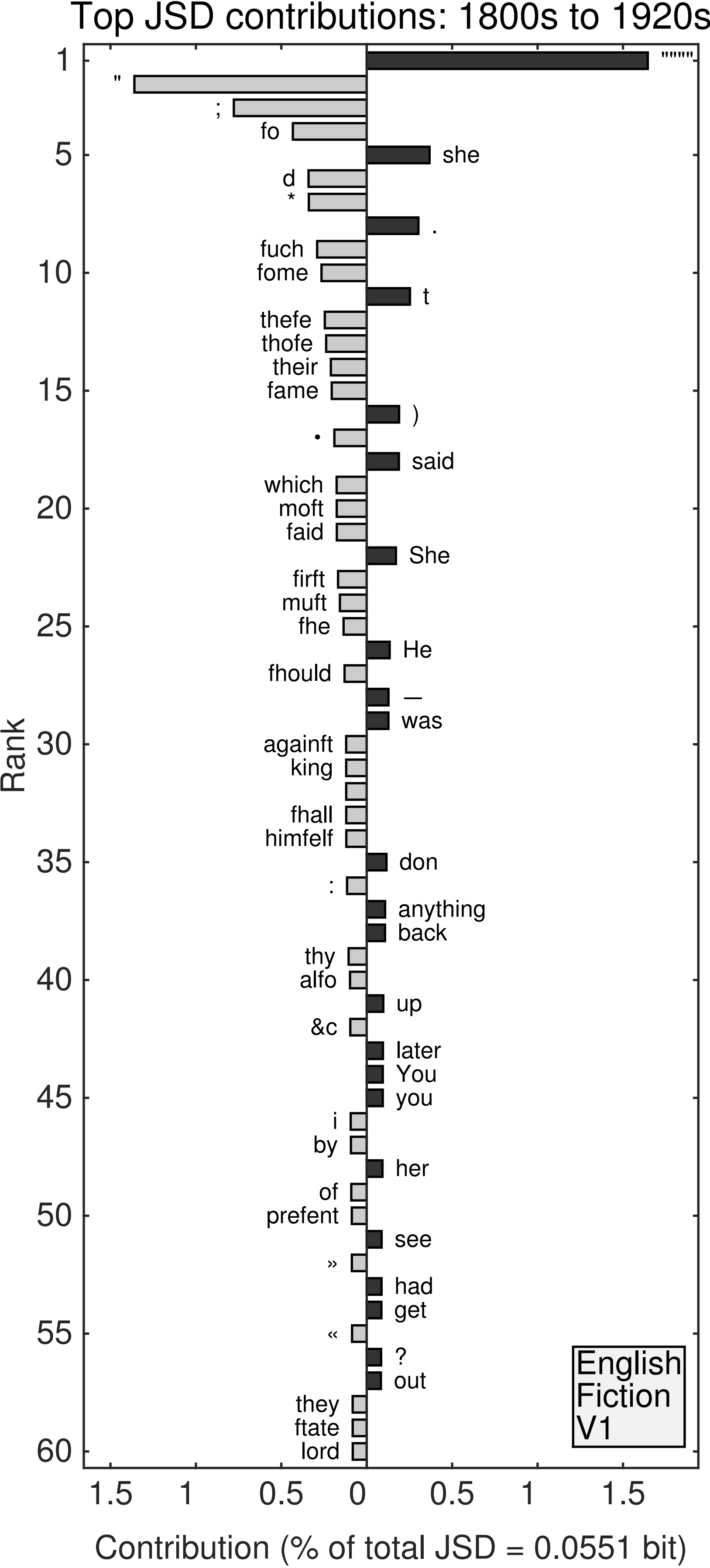
Top JSD contributions: 1800s to 1920s
Rank
Contribution (% of total JSD = 0.0551 bit)
English Fiction V1
""""
"
;
fo
she
d
*
.
fuch
fome
t
thefe
thofe
their
fame
)
•
said
which
moft
faid
She
firft
muft
fhe
He
fhould
—
was
againft
king
fhall
himfelf
don
:
anything
back
thy
alfo
up
&c
later
You
you
i
by
her
of
prefent
see
»
had
get
«
?
out
they
ftate
lord

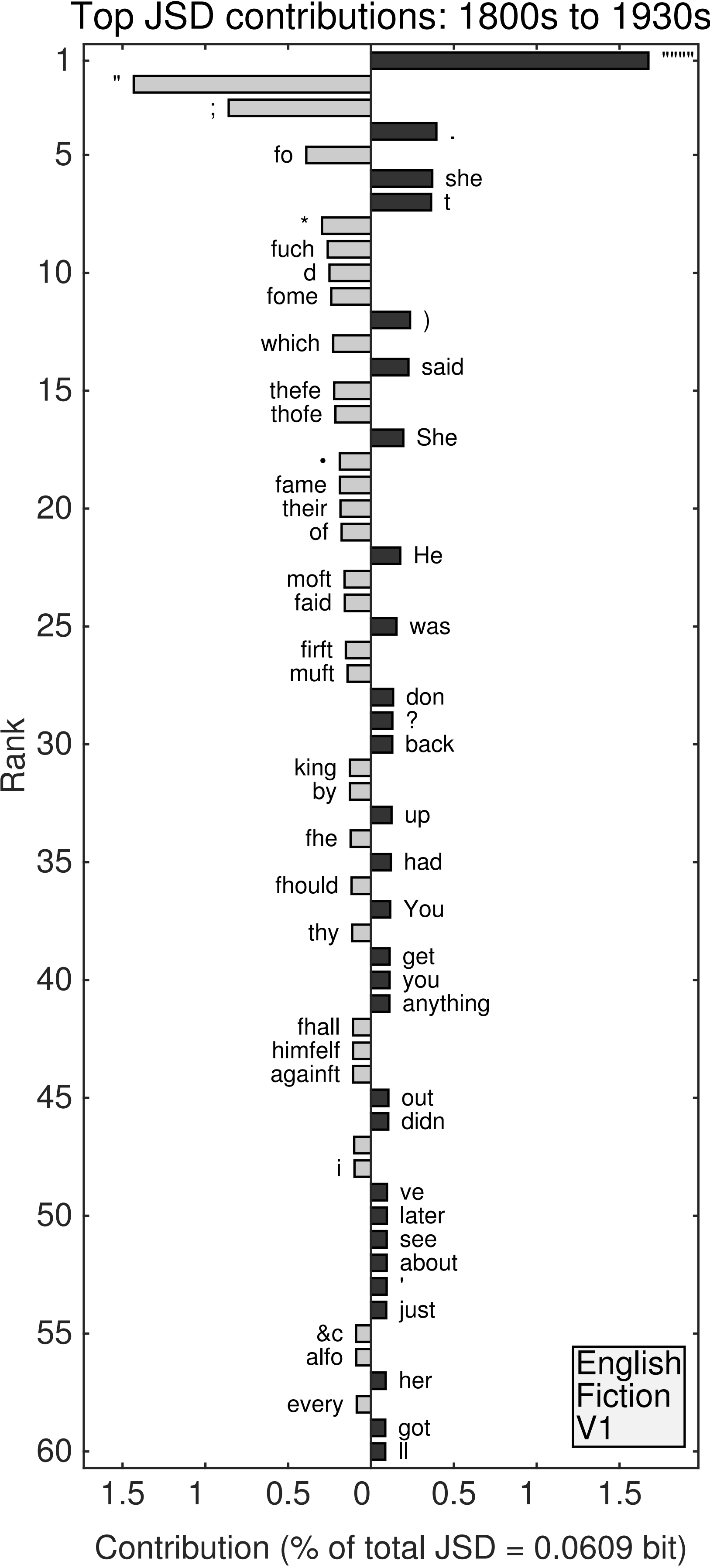

Top JSD contributions: 1800s to 1930s
Rank
Contribution (% of total JSD = 0.0609 bit)
English Fiction V1
1.5
1
0.5
0
0.5
1
1.5
1
5
10
15
20
25
30
35
40
45
50
55
60
"""""
"
;
.
fo
she
t
*
fuch
d
fome
)
which
said
thefe
thofe
She
•
fame
their
of
He
moft
faid
was
firft
muft
don
?
back
king
by
up
fhe
had
fhould
You
thy
get
you
anything
fhall
himfelf
againft
out
didn
i
ve
later
see
about
'
just
&c
alfo
her
every
got
ll

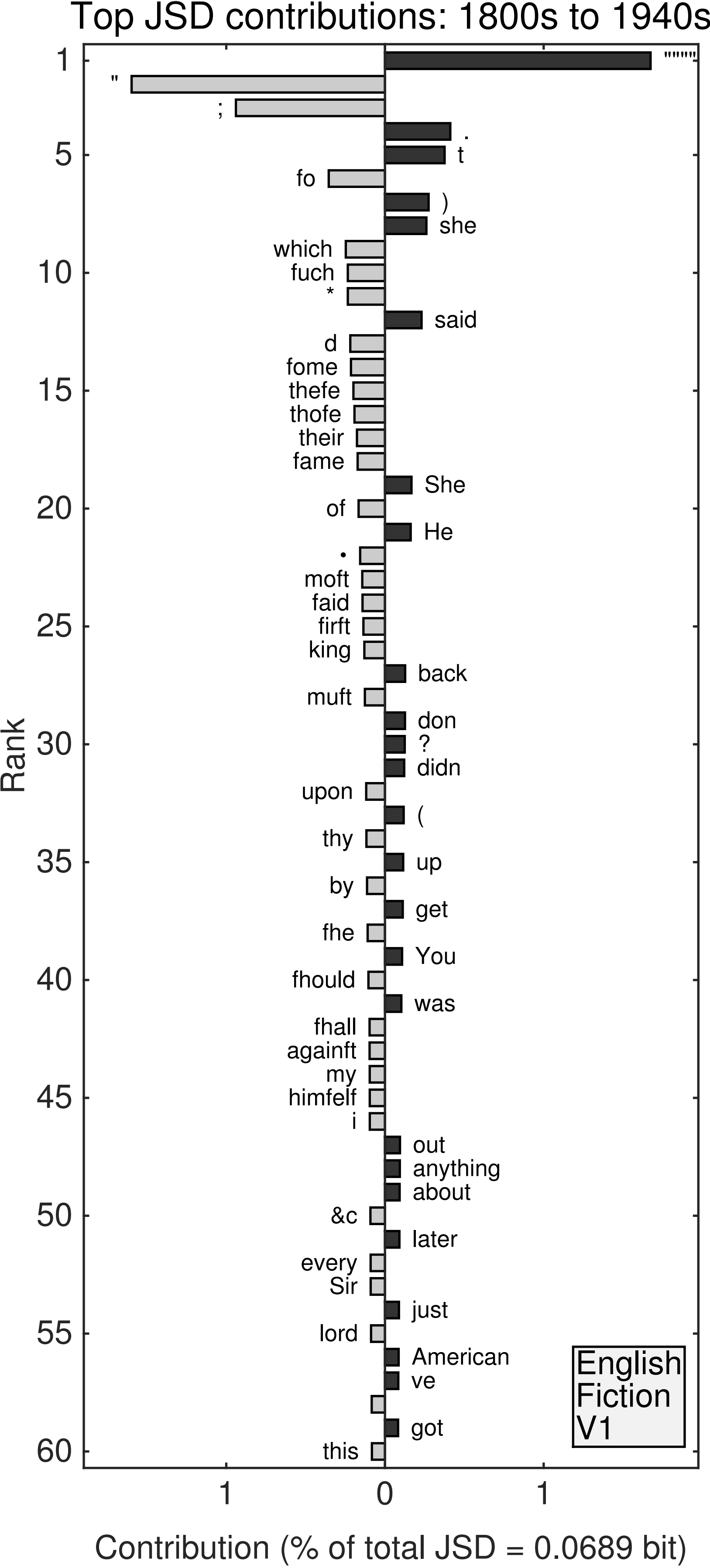

Top JSD contributions: 1800s to 1940s
Rank
Contribution (% of total JSD = 0.0689 bit)
English Fiction V1
1
5
10
15
20
25
30
35
40
45
50
55
60
""""
"
;
.
t
fo
)
she
which
fuch
*
said
d
fome
thefe
thofe
their
fame
She
of
He
•
moft
faid
firft
king
back
muft
don
?
didn
upon
(
thy
up
by
get
fhe
You
fhould
was
fhall
againft
my
himfelf
i
out
anything
about
&c
later
every
Sir
just
lord
American
ve
got
this

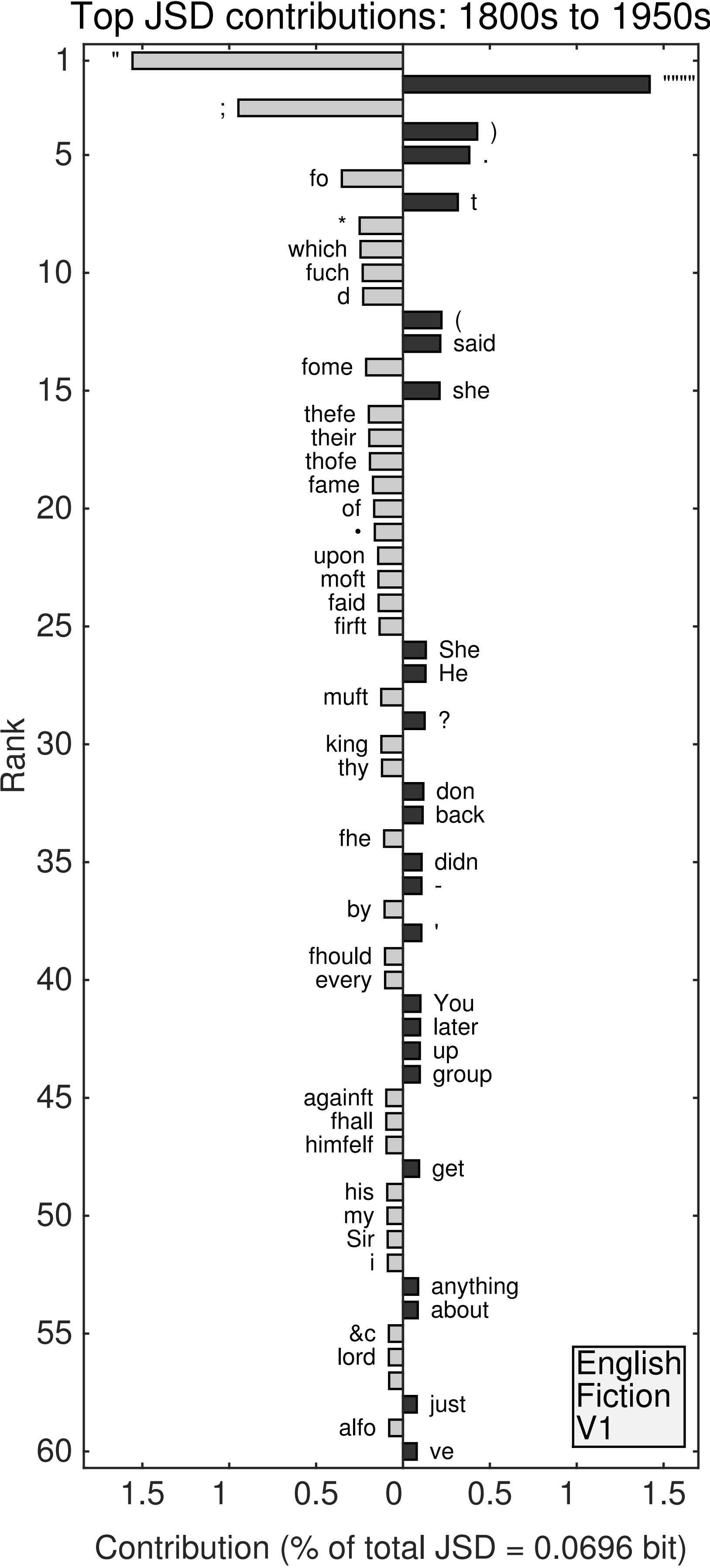
Top JSD contributions: 1800s to 1950s
Rank
"
""""
;
)
.
fo
t
*
which
fuch
d
(
said
fome
she
thefe
their
thofe
fame
of
•
upon
moft
faid
firft
She
He
muft
?
king
thy
don
back
fhe
didn
-
by
'
fhould
every
You
later
up
group
againft
fhall
himfelf
get
his
my
Sir
i
anything
about
&c
lord
just
alfo
ve
1
5
10
15
20
25
30
35
40
45
50
55
60
1.5
1
0.5
0
0.5
1
1.5
Contribution (% of total JSD = 0.0696 bit)
English
Fiction
V1

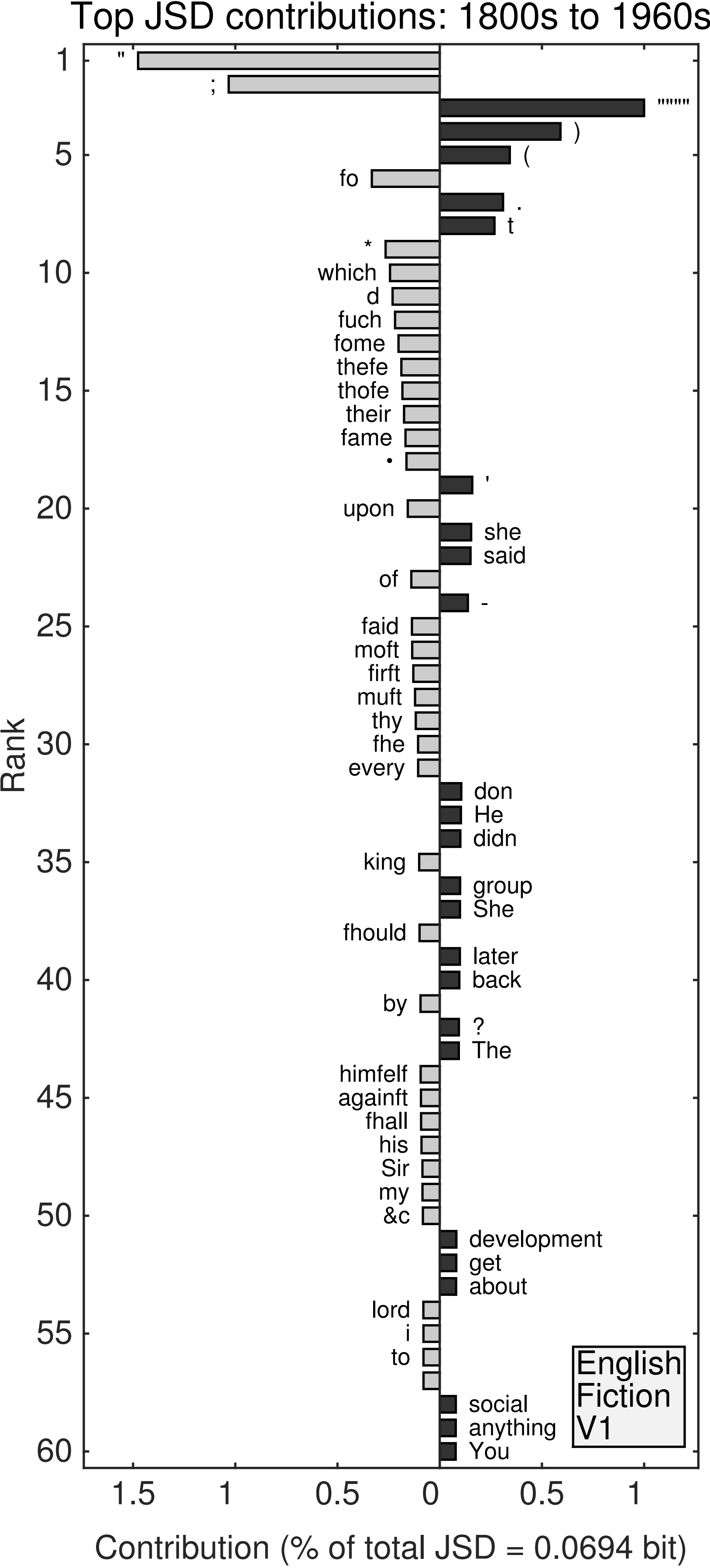
Top JSD contributions: 1800s to 1960s
Rank
Contribution (% of total JSD = 0.0694 bit)
English Fiction V1
1.5
1
0.5
0
0.5
1
5
10
15
20
25
30
35
40
45
50
55
60
"
;
""""
)
(
fo
.
t
*
which
d
fuch
fome
thefe
thofe
their
fame
•
'
upon
she
said
of
-
faid
moft
firft
muft
thy
fhe
every
don
He
didn
king
group
She
fhould
later
back
by
?
The
himfelf
againft
fhall
his
Sir
my
&c
development
get
about
lord
i
to
social
anything
You

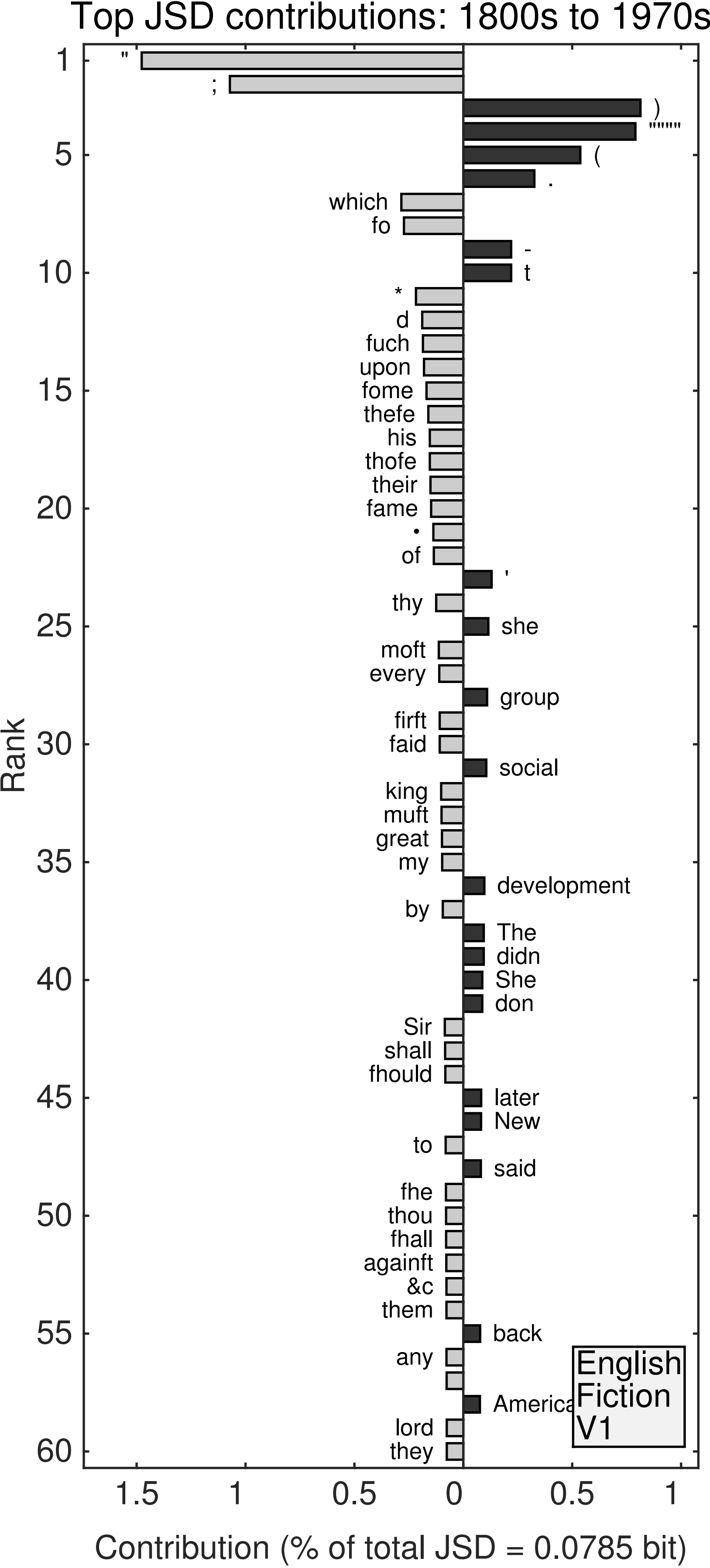

Top JSD contributions: 1800s to 1970s
Rank
Contribution (% of total JSD = 0.0785 bit)
English Fiction V1
"
;
)
""""
(
.
which
fo
-
t
*
d
fuch
upon
fome
thefe
his
thofe
their
fame
•
of
'
thy
she
moft
every
group
firft
faid
social
king
muft
great
my
development
by
The
didn
She
don
Sir
shall
fhould
later
New
to
said
fhe
thou
fhall
againft
&c
them
back
any
America
lord
they

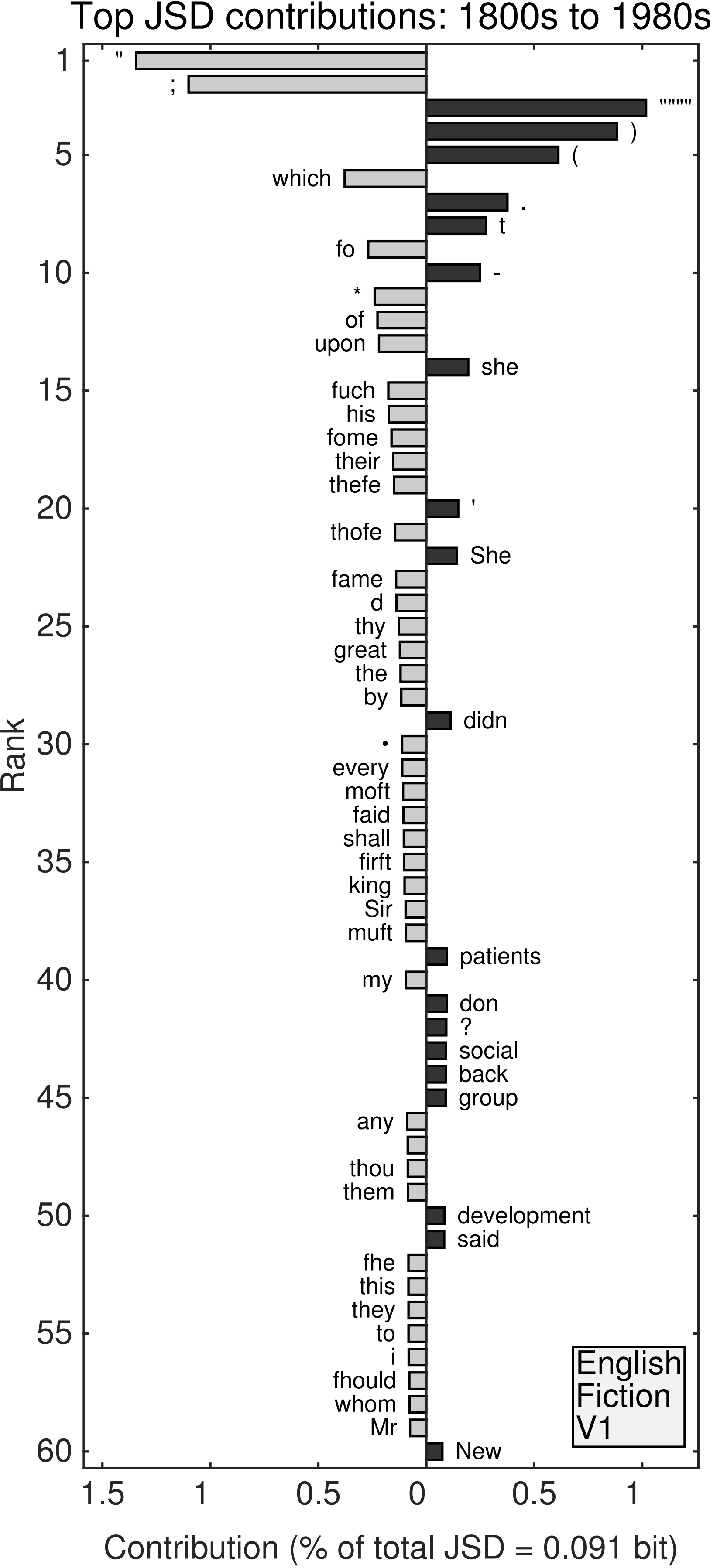
Top JSD contributions: 1800s to 1980s
Rank
Contribution (% of total JSD = 0.091 bit)
1.5
1
0.5
0
0.5
1
1
5
10
15
20
25
30
35
40
45
50
55
60
"
;
""""
)
(
which
.
t
fo
-
*
of
upon
she
fuch
his
fome
their
thefe
'
thofe
She
fame
d
thy
great
the
by
didn
•
every
moft
faid
shall
firft
king
Sir
muft
patients
my
don
?
social
back
group
any
thou
them
development
said
fhe
this
they
to
i
fhould
whom
Mr
New
English
Fiction
V1

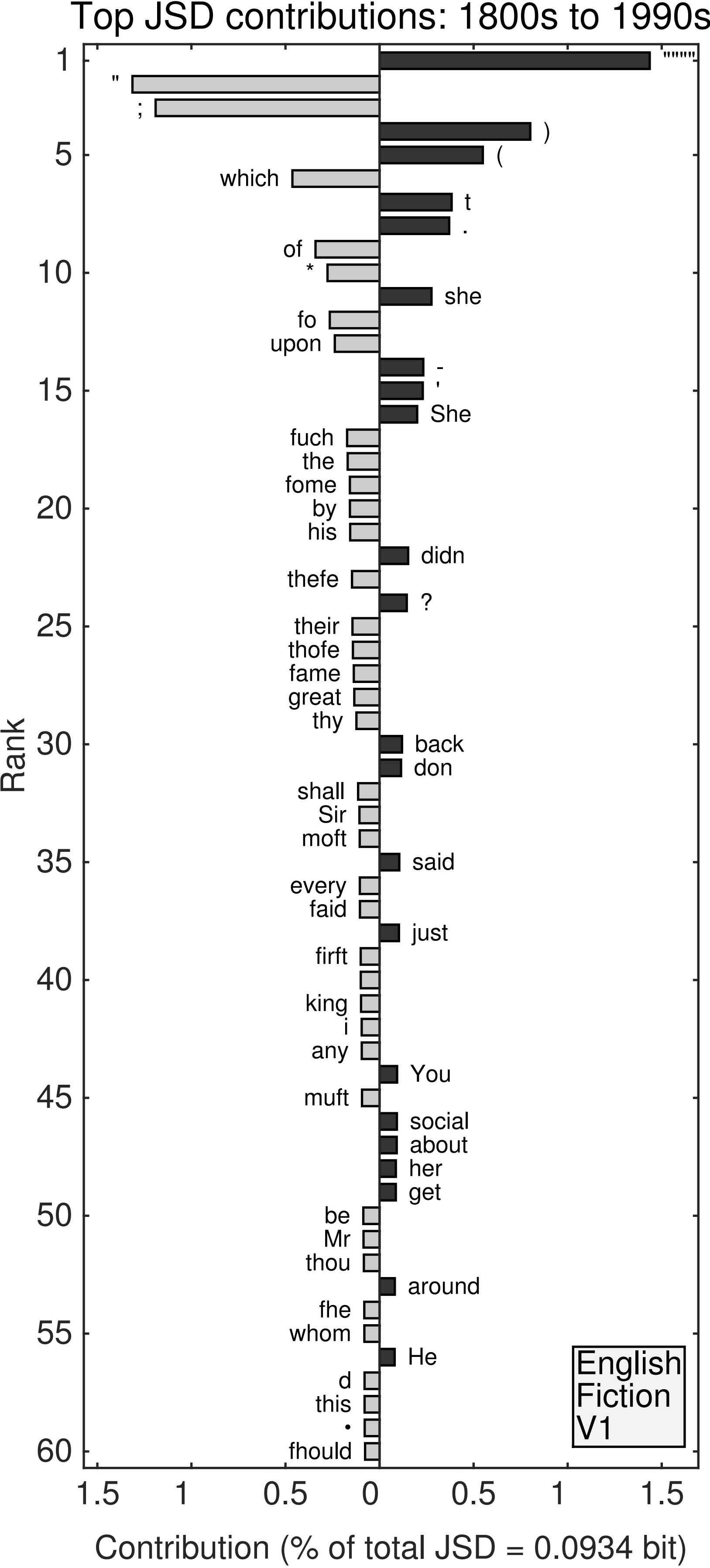

Top JSD contributions: 1800s to 1990s
Rank
Contribution (% of total JSD = 0.0934 bit)
English Fiction V1
"""""
"
;
)
(
which
t
.
of
*
she
fo
upon
-
'
She
fuch
the
fome
by
his
didn
thefe
?
their
thofe
fame
great
thy
back
don
shall
Sir
moft
said
every
faid
just
firft
king
i
any
You
muft
social
about
her
get
be
Mr
thou
around
fhe
whom
He
d
this
•
fhould

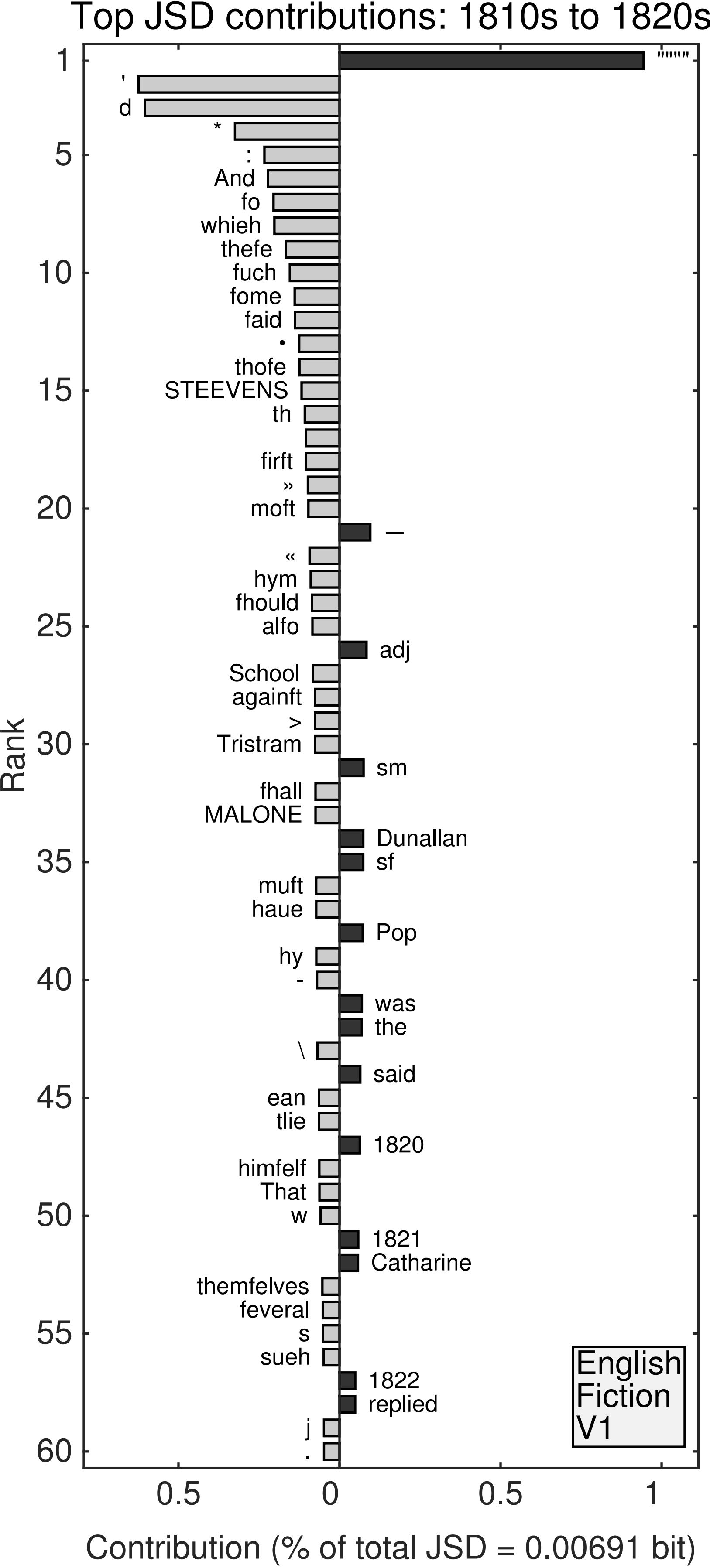
Top JSD contributions: 1810s to 1820s
Rank
Contribution (% of total JSD = 0.00691 bit)
English Fiction V1
"""""
'
d
*
:
And
fo
whieh
thefe
fuch
fome
faid
•
thofe
STEEVENS
th
firft
»
moft
—
«
hym
fhould
alfo
adj
School
againft
>
Tristram
sm
fhall
MALONE
Dunallan
sf
muft
haue
Pop
hy
-
was
the
\
said
ean
tlie
1820
himfelf
That
w
1821
Catharine
themfelves
feveral
s
sueh
1822
replied
j
.
1
5
10
15
20
25
30
35
40
45
50
55
60
0.5
0
0.5
1

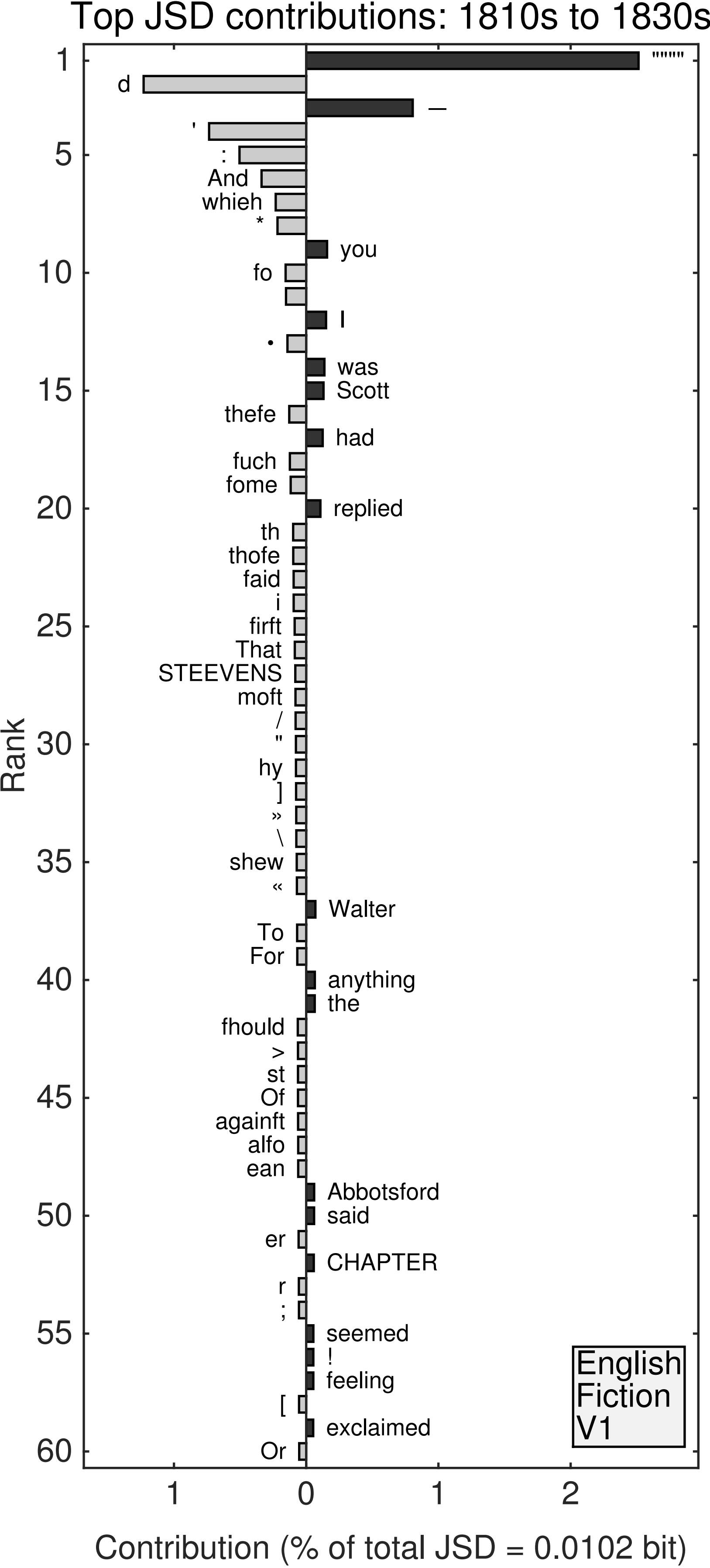

Top JSD contributions: 1810s to 1830s
Rank
Contribution (% of total JSD = 0.0102 bit)
English Fiction V1
""""
d
—
'
:
And
whieh
*
you
fo
I
•
was
Scott
thefe
had
fuch
fome
replied
th
thofe
faid
i
firft
That
STEEVENS
moft
/
"
hy
]
»
\
shew
«
Walter
To
For
anything
the
fhould
>
st
Of
againft
alfo
ean
Abbotsford
said
er
CHAPTER
r
;
seemed
!
feeling
[
exclaimed
Or

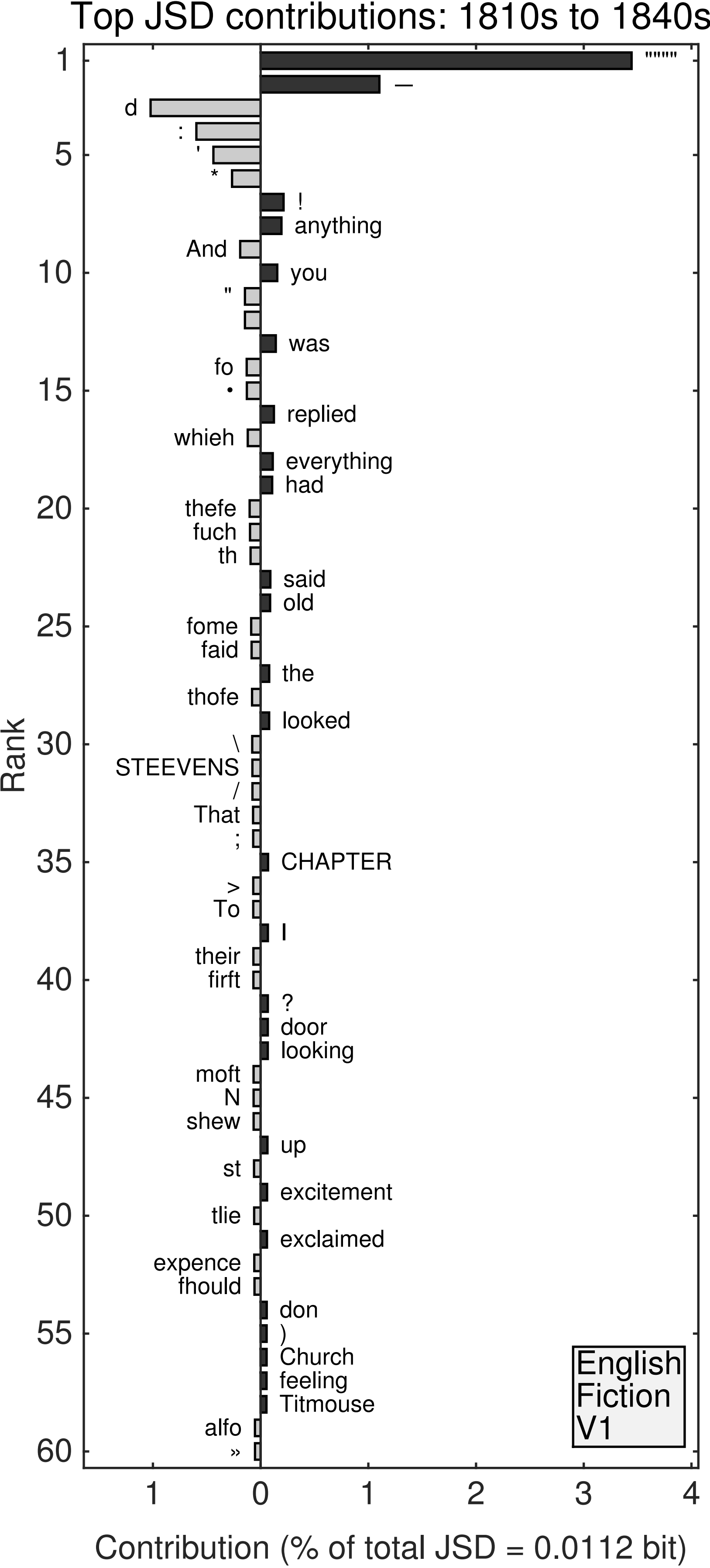

Top JSD contributions: 1810s to 1840s
"""""
—
d
:
'
*
!
anything
And
you
"
was
fo
•
replied
whieh
everything
had
thefe
fuch
th
said
old
fome
faid
the
thofe
looked
\
STEEVENS
/
That
;
CHAPTER
>
To
I
their
firft
?
door
looking
moft
N
shew
up
st
excitement
tlie
exclaimed
expence
fhould
don
)
Church
feeling
Titmouse
alfo
»
English
Fiction
V1
Rank
1
5
10
15
20
25
30
35
40
45
50
55
60
1
0
1
2
3
4
Contribution (% of total JSD = 0.0112 bit)

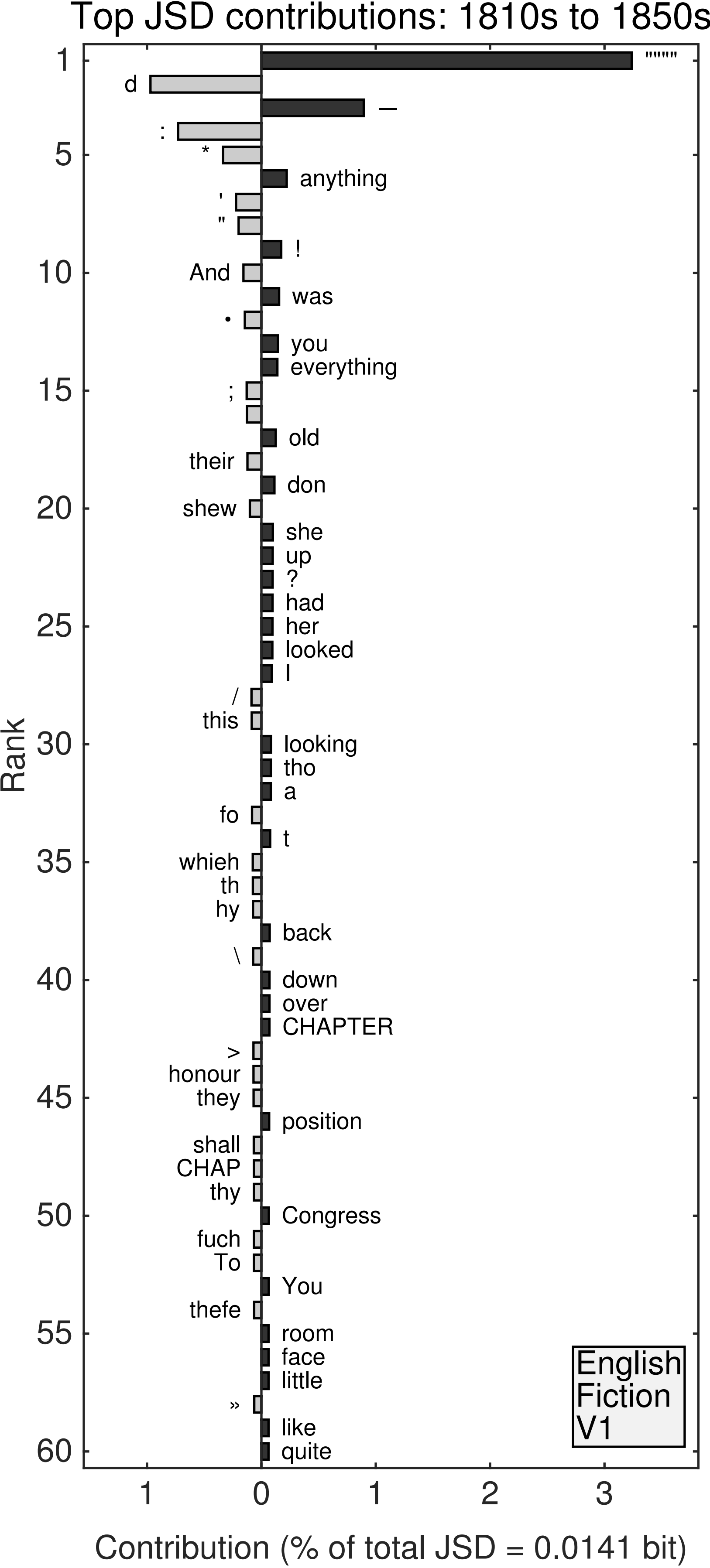

Top JSD contributions: 1810s to 1850s
Rank
Contribution (% of total JSD = 0.0141 bit)
""""
d
—
:
*
anything
'
"
!
And
was
•
you
everything
;
old
their
don
shew
she
up
?
had
her
looked
I
/
this
looking
tho
a
fo
t
whieh
th
hy
back
\
down
over
CHAPTER
>
honour
they
position
shall
CHAP
thy
Congress
fuch
To
You
thefe
room
face
little
»
like
quite
English
Fiction
V1

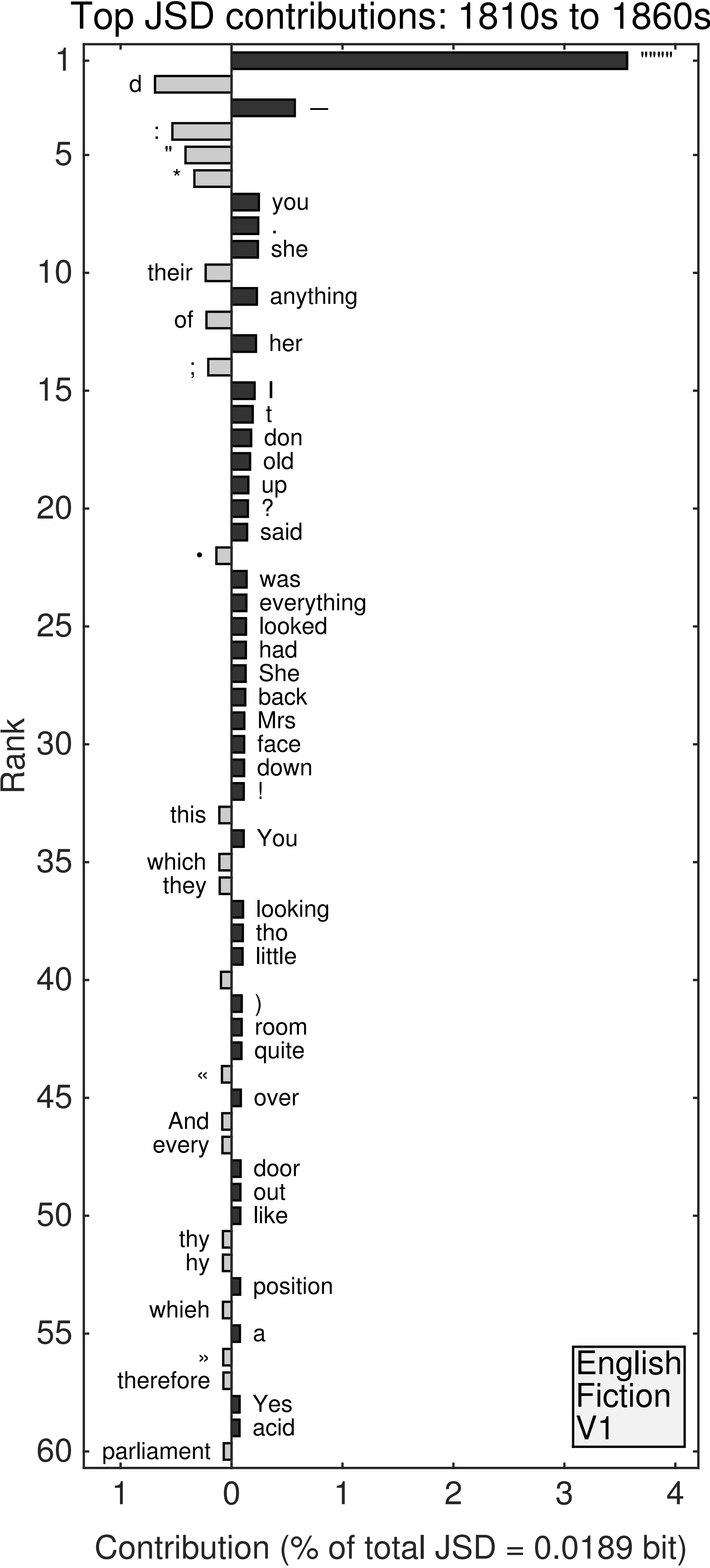

Top JSD contributions: 1810s to 1860s
Rank
Contribution (% of total JSD = 0.0189 bit)
English Fiction V1
"""""
d
—
:
"
*
you
.
she
their
anything
of
her
;
I
t
don
old
up
?
said
•
was
everything
looked
had
She
back
Mrs
face
down
!
this
You
which
they
looking
tho
little
)
room
quite
«
over
And
every
door
out
like
thy
hy
position
whieh
a
»
therefore
Yes
acid
parliament

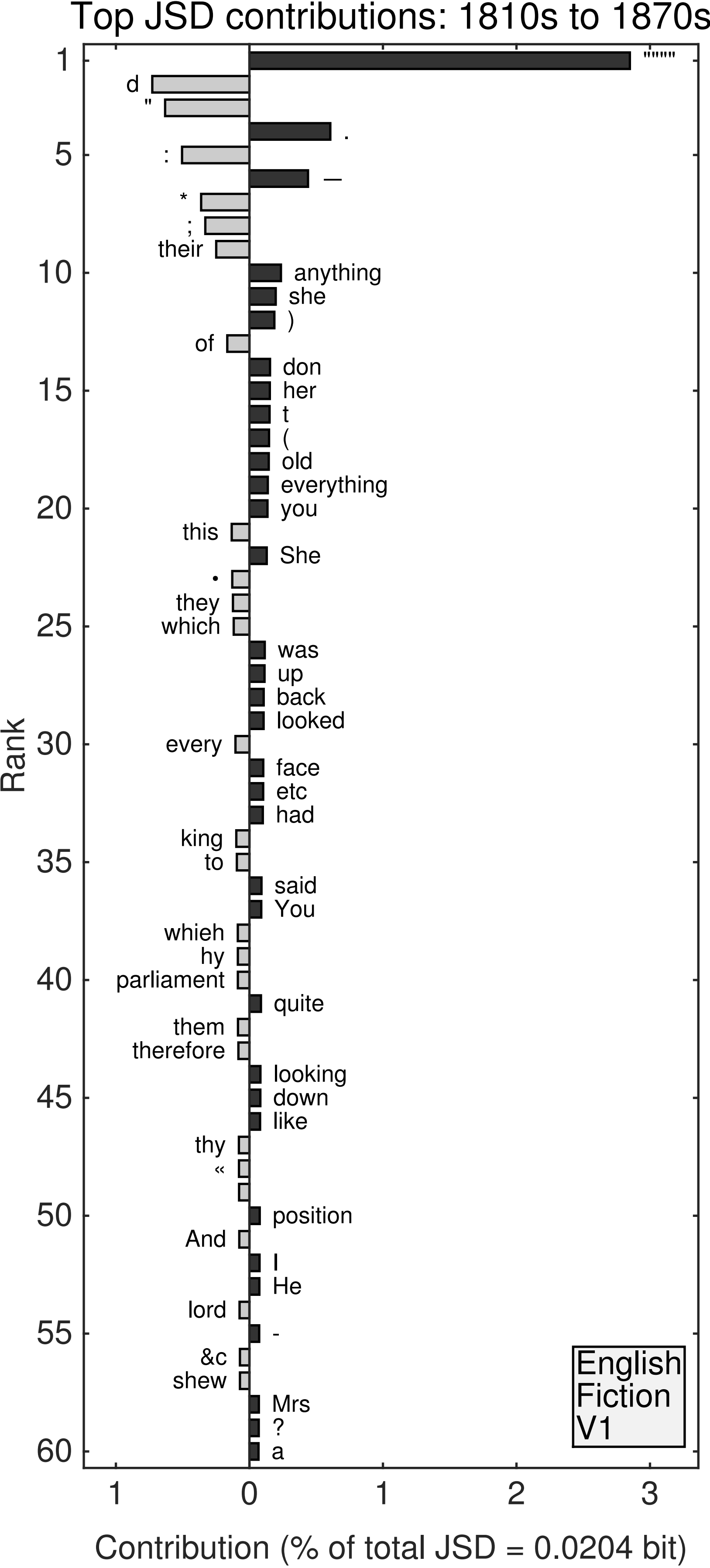

Top JSD contributions: 1810s to 1870s
Rank
Contribution (% of total JSD = 0.0204 bit)
English Fiction V1
""""
d
"
.
:
—
*
;
their
anything
she
)
of
don
her
t
(
old
everything
you
this
She
•
they
which
was
up
back
looked
every
face
etc
had
king
to
said
You
whieh
hy
parliament
quite
them
therefore
looking
down
like
thy
«
position
And
I
He
lord
-
&c
shew
Mrs
?
a

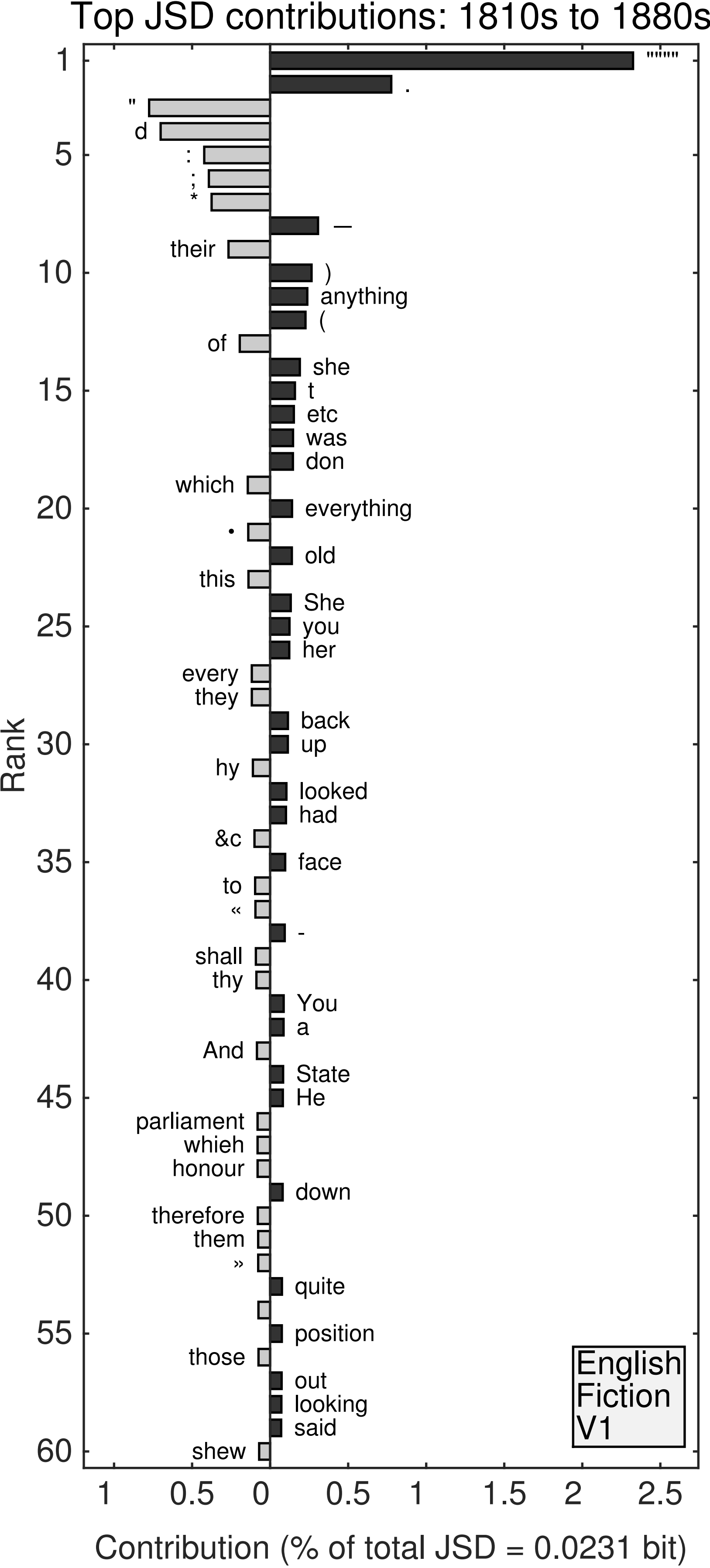

Top JSD contributions: 1810s to 1880s
Rank
1
5
10
15
20
25
30
35
40
45
50
55
60
""""
.
"
d
:
;
*
—
their
)
anything
(
of
she
t
etc
was
don
which
everything
•
old
this
She
you
her
every
they
back
up
hy
looked
had
&c
face
to
«
-
shall
thy
You
a
And
State
He
parliament
whieh
honour
down
therefore
them
»
quite
position
those
out
looking
said
shew
English
Fiction
V1
1
0.5
0
0.5
1
1.5
2
2.5
Contribution (% of total JSD = 0.0231 bit)

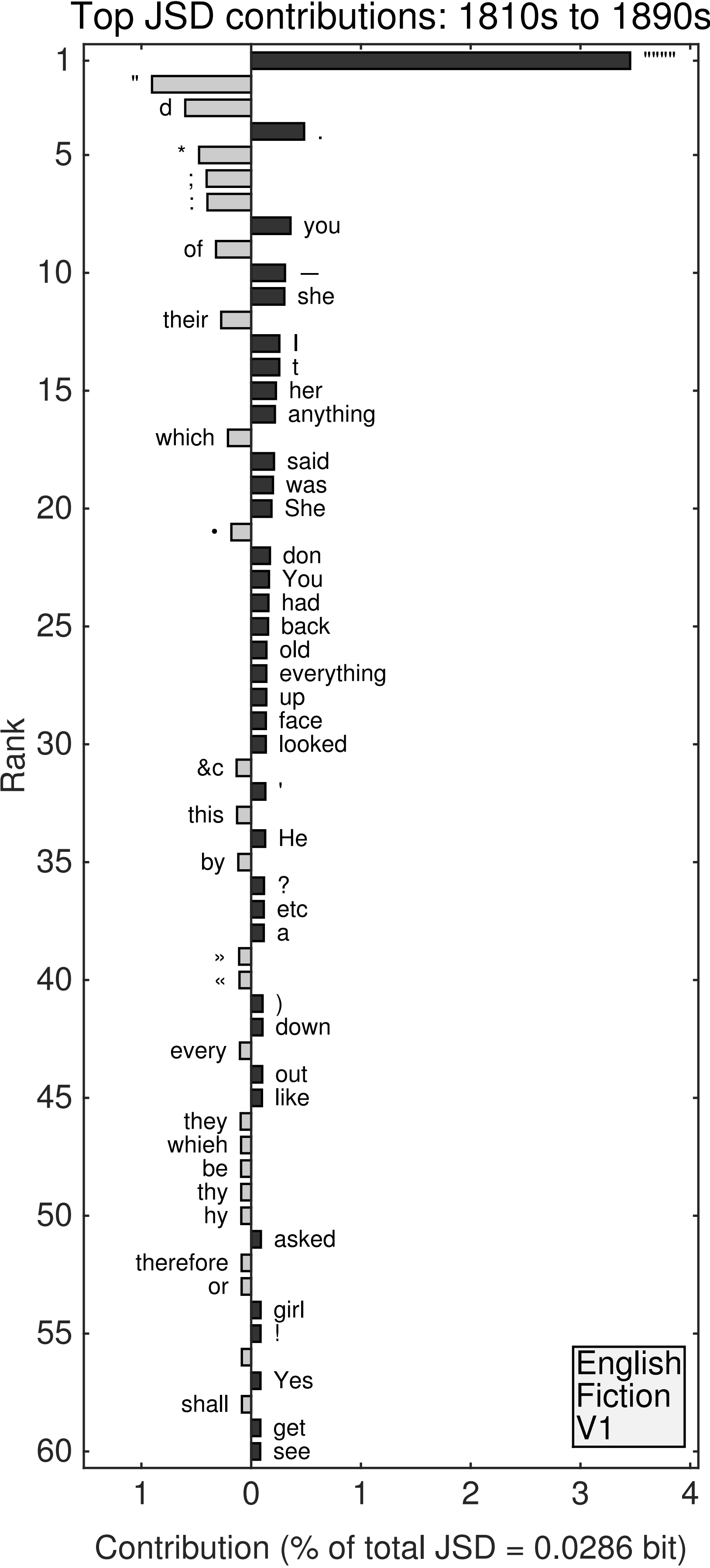

Top JSD contributions: 1810s to 1890s
Rank
Contribution (% of total JSD = 0.0286 bit)
English Fiction V1
"""""
"
d
.
*
;
:
you
of
—
she
their
I
t
her
anything
which
said
was
She
•
don
You
had
back
old
everything
up
face
looked
&c
'
this
He
by
?
etc
a
»
«
)
down
every
out
like
they
whieh
be
thy
hy
asked
therefore
or
girl
!
Yes
shall
get
see

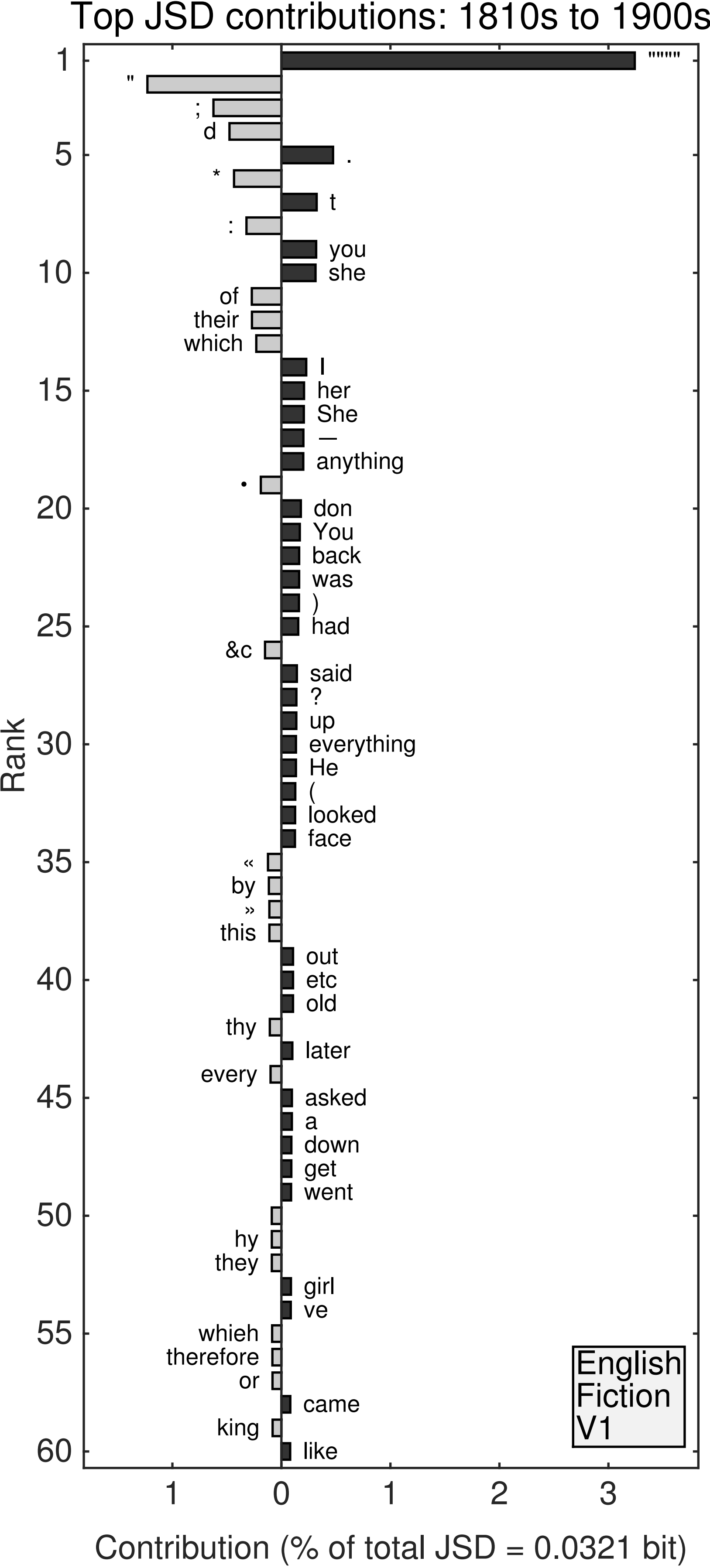

Top JSD contributions: 1810s to 1900s
Rank
Contribution (% of total JSD = 0.0321 bit)
English Fiction V1
""""
"
;
d
.
*
t
:
you
she
of
their
which
I
her
She
—
anything
•
don
You
back
was
)
had
&c
said
?
up
everything
He
(
looked
face
«
by
»
this
out
etc
old
thy
later
every
asked
a
down
get
went
hy
they
girl
ve
whieh
therefore
or
came
king
like

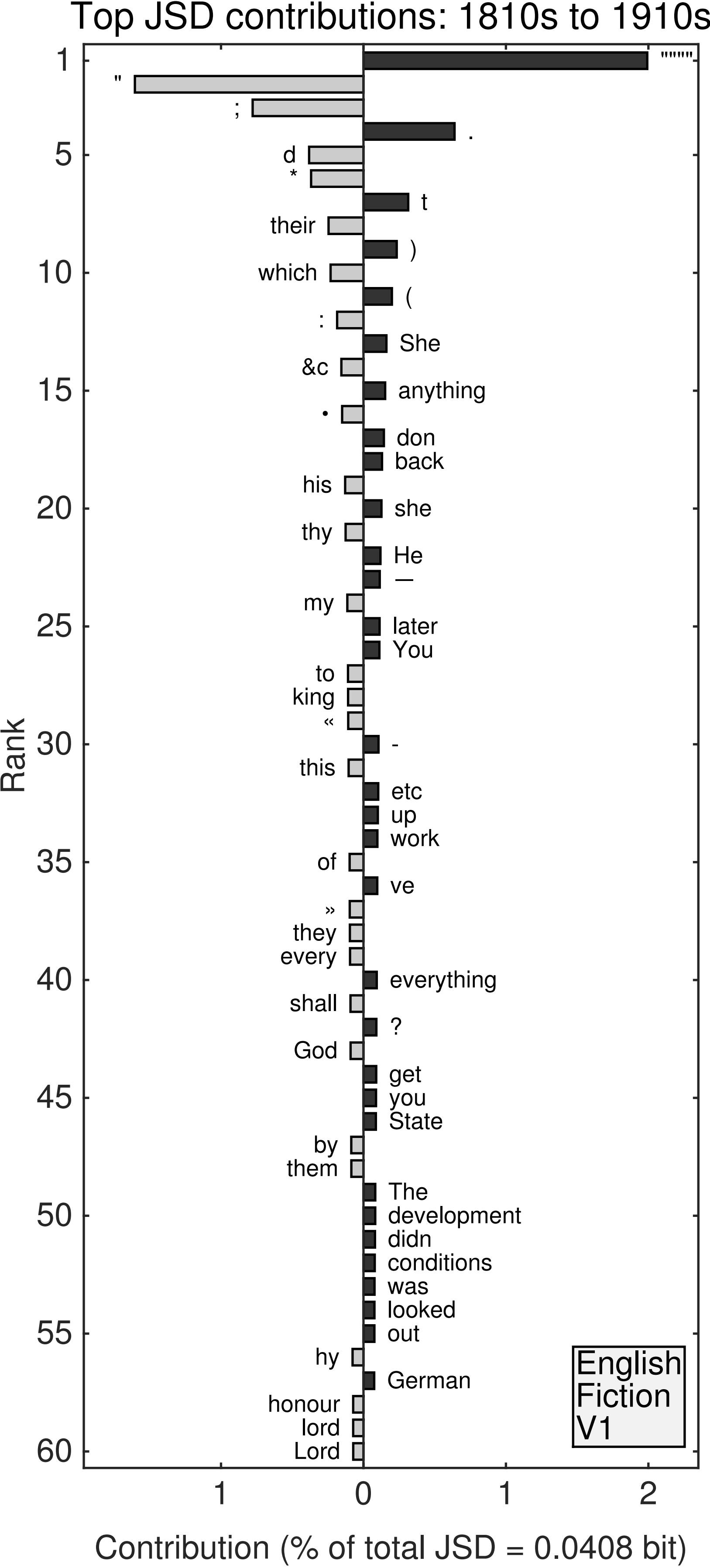
Top JSD contributions: 1810s to 1910s
Rank
Contribution (% of total JSD = 0.0408 bit)
English Fiction V1
"""""
"
;
.
d
*
t
their
)
which
(
:
She
&c
anything
•
don
back
his
she
thy
He
—
my
later
You
to
king
«
-
this
etc
up
work
of
ve
»
they
every
everything
shall
?
God
get
you
State
by
them
The
development
didn
conditions
was
looked
out
hy
German
honour
lord
Lord

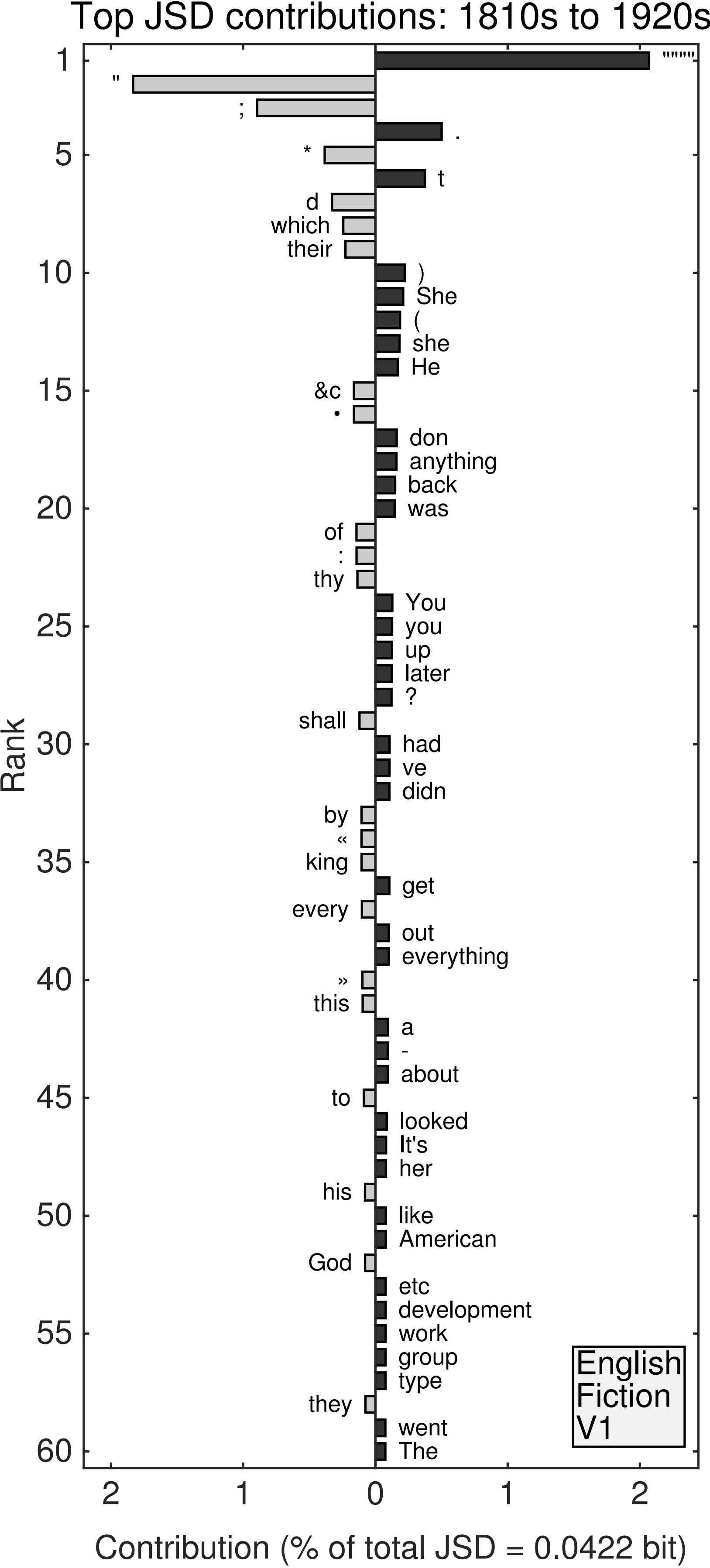
Top JSD contributions: 1810s to 1920s
Rank
Contribution (% of total JSD = 0.0422 bit)
English Fiction V1
"""""
"
;
.
*
t
d
which
their
)
She
(
she
He
&c
•
don
anything
back
was
of
:
thy
You
you
up
later
?
shall
had
ve
didn
by
«
king
get
every
out
everything
»
this
a
-
about
to
looked
It's
her
his
like
American
God
etc
development
work
group
type
they
went
The

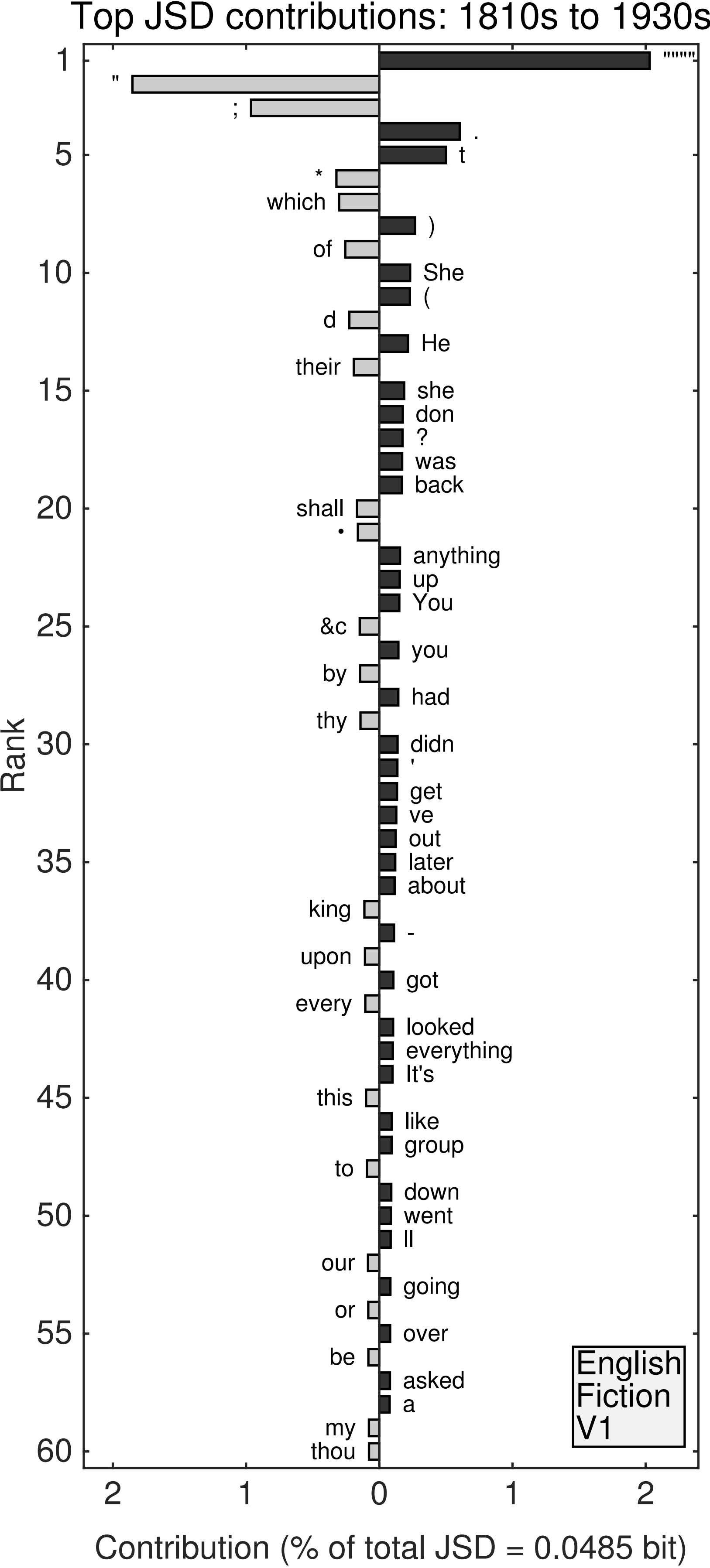

Top JSD contributions: 1810s to 1930s
Rank
Contribution (% of total JSD = 0.0485 bit)
English Fiction V1
"""""
"
;
.
t
*
which
)
of
She
(
d
He
their
she
don
?
was
back
shall
•
anything
up
You
&c
you
by
had
thy
didn
'
get
ve
out
later
about
king
-
upon
got
every
looked
everything
It's
this
like
group
to
down
went
ll
our
going
or
over
be
asked
a
my
thou

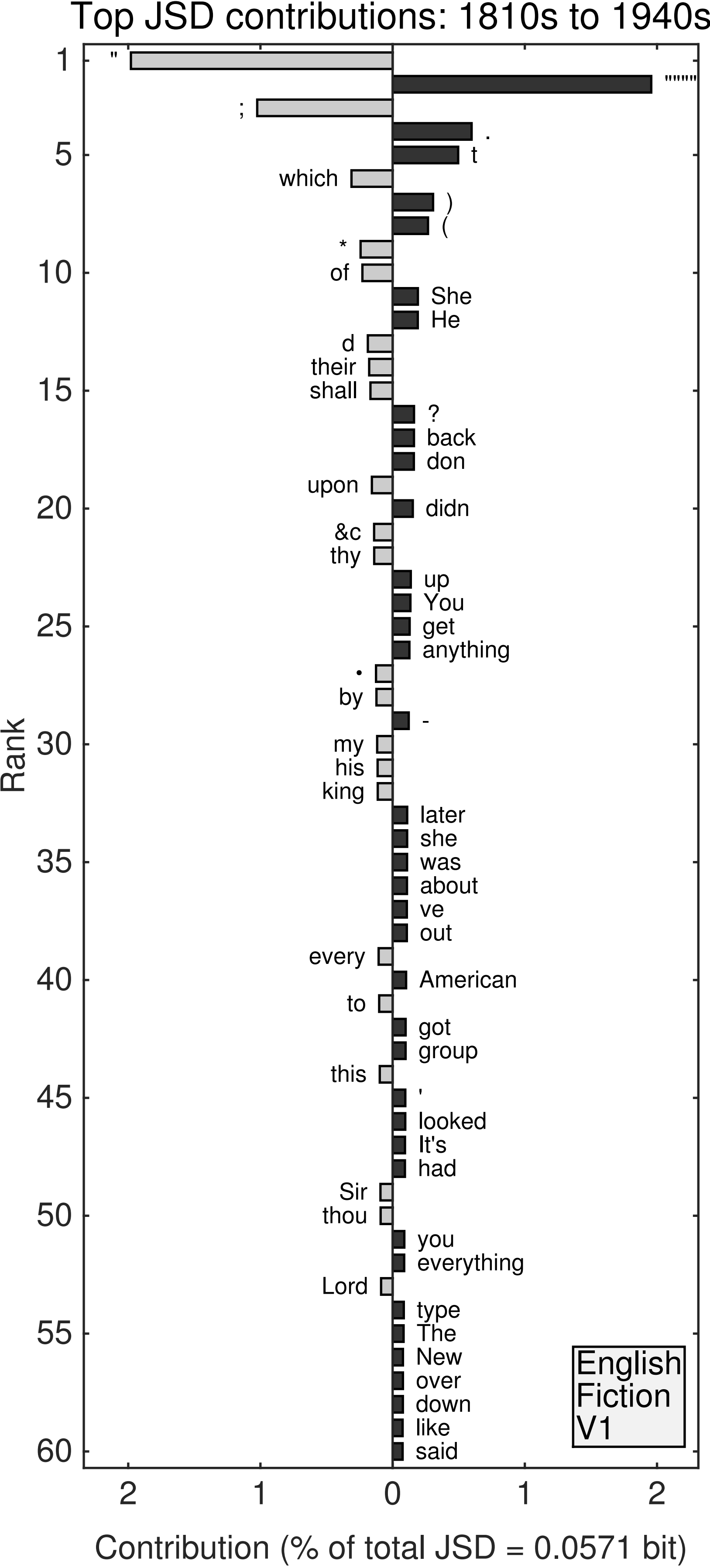

Top JSD contributions: 1810s to 1940s
Rank
Contribution (% of total JSD = 0.0571 bit)
English Fiction V1
"
""""
;
.
t
which
)
(
*
of
She
He
d
their
shall
?
back
don
upon
didn
&c
thy
up
You
get
anything
•
by
-
my
his
king
later
she
was
about
ve
out
every
American
to
got
group
this
'
looked
It's
had
Sir
thou
you
everything
Lord
type
The
New
over
down
like
said

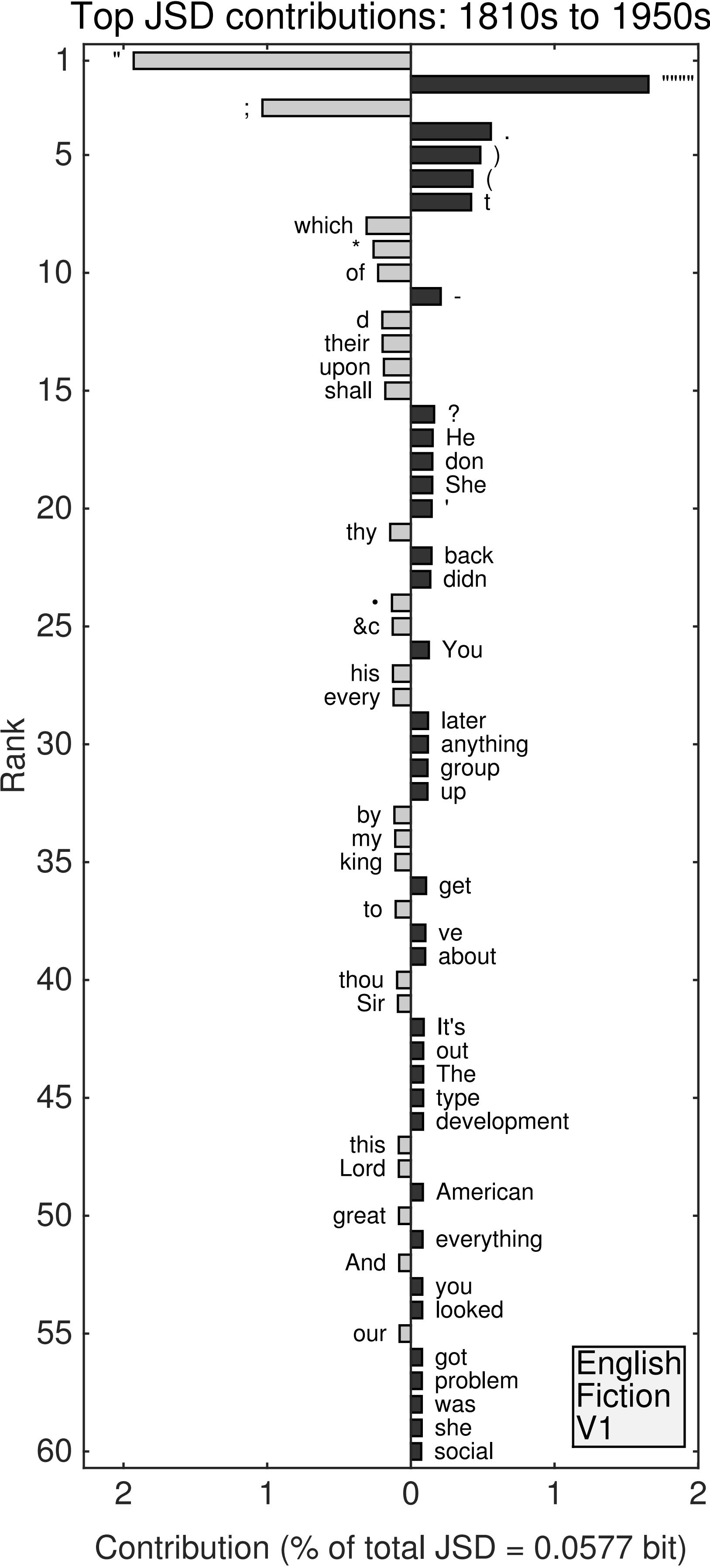
Top JSD contributions: 1810s to 1950s
Rank
Contribution (% of total JSD = 0.0577 bit)
English Fiction V1
"
""""
;
.
)
(
t
which
*
of
-
d
their
upon
shall
?
He
don
She
'
thy
back
didn
•
&c
You
his
every
later
anything
group
up
by
my
king
get
to
ve
about
thou
Sir
It's
out
The
type
development
this
Lord
American
great
everything
And
you
looked
our
got
problem
was
she
social

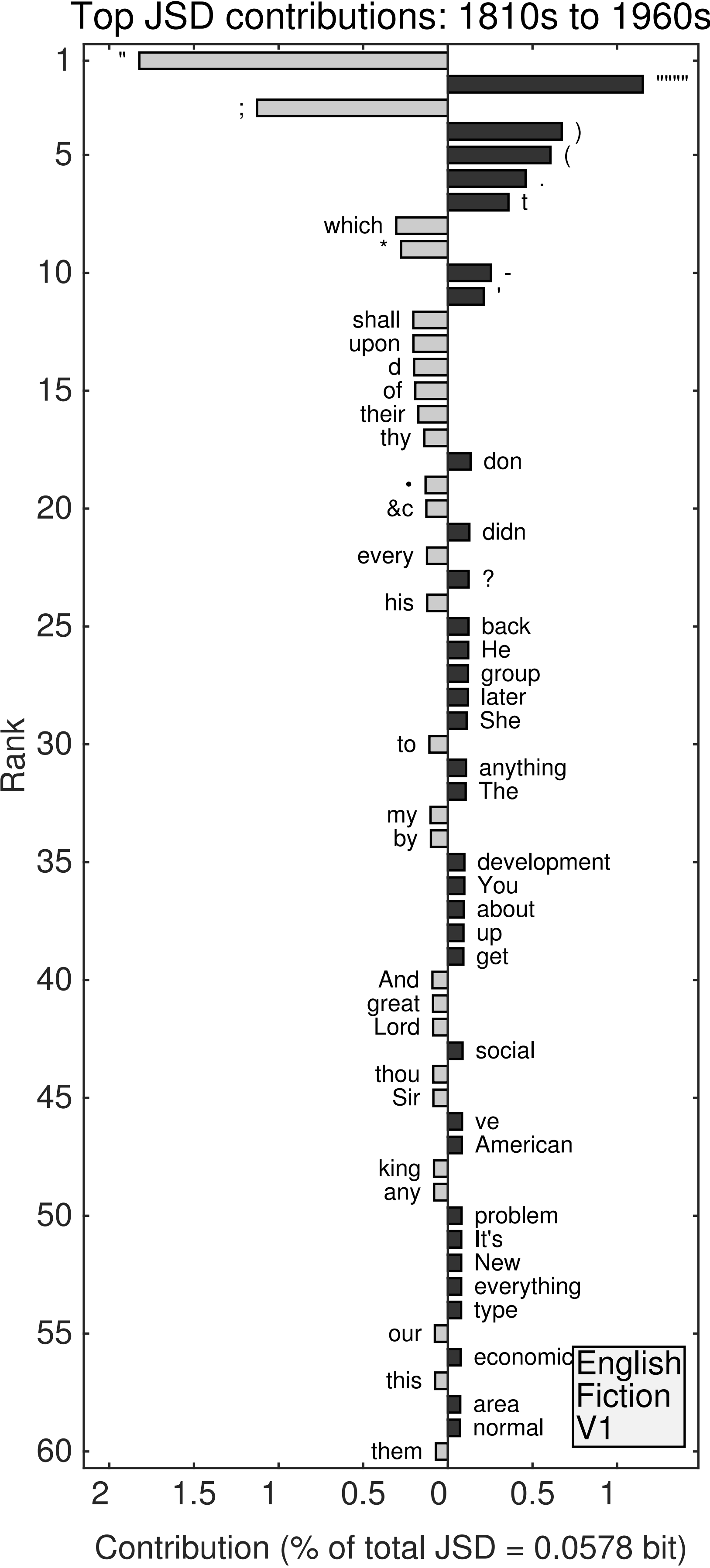

Top JSD contributions: 1810s to 1960s
Rank
Contribution (% of total JSD = 0.0578 bit)
English Fiction V1
"
""""
;
)
(
.
t
which
*
-
'
shall
upon
d
of
their
thy
don
•
&c
didn
every
?
his
back
He
group
later
She
to
anything
The
my
by
development
You
about
up
get
And
great
Lord
social
thou
Sir
ve
American
king
any
problem
It's
New
everything
type
our
economic
this
area
normal
them

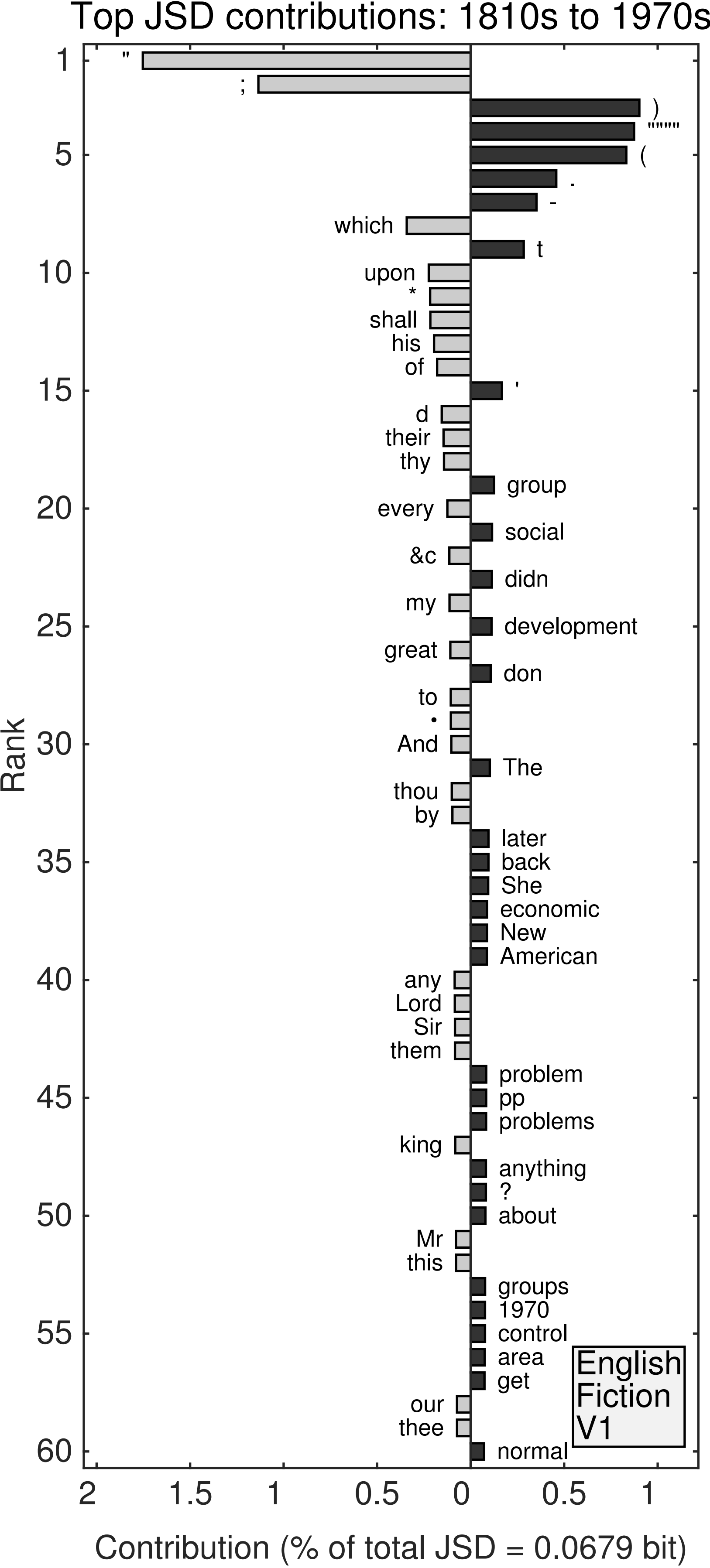

Top JSD contributions: 1810s to 1970s
Rank
Contribution (% of total JSD = 0.0679 bit)
"
;
)
""""
(
.
-
which
t
upon
*
shall
his
of
'
d
their
thy
group
every
social
&c
didn
my
development
great
don
to
•
And
The
thou
by
later
back
She
economic
New
American
any
Lord
Sir
them
problem
pp
problems
king
anything
?
about
Mr
this
groups
1970
control
area
get
our
thee
normal
English Fiction V1

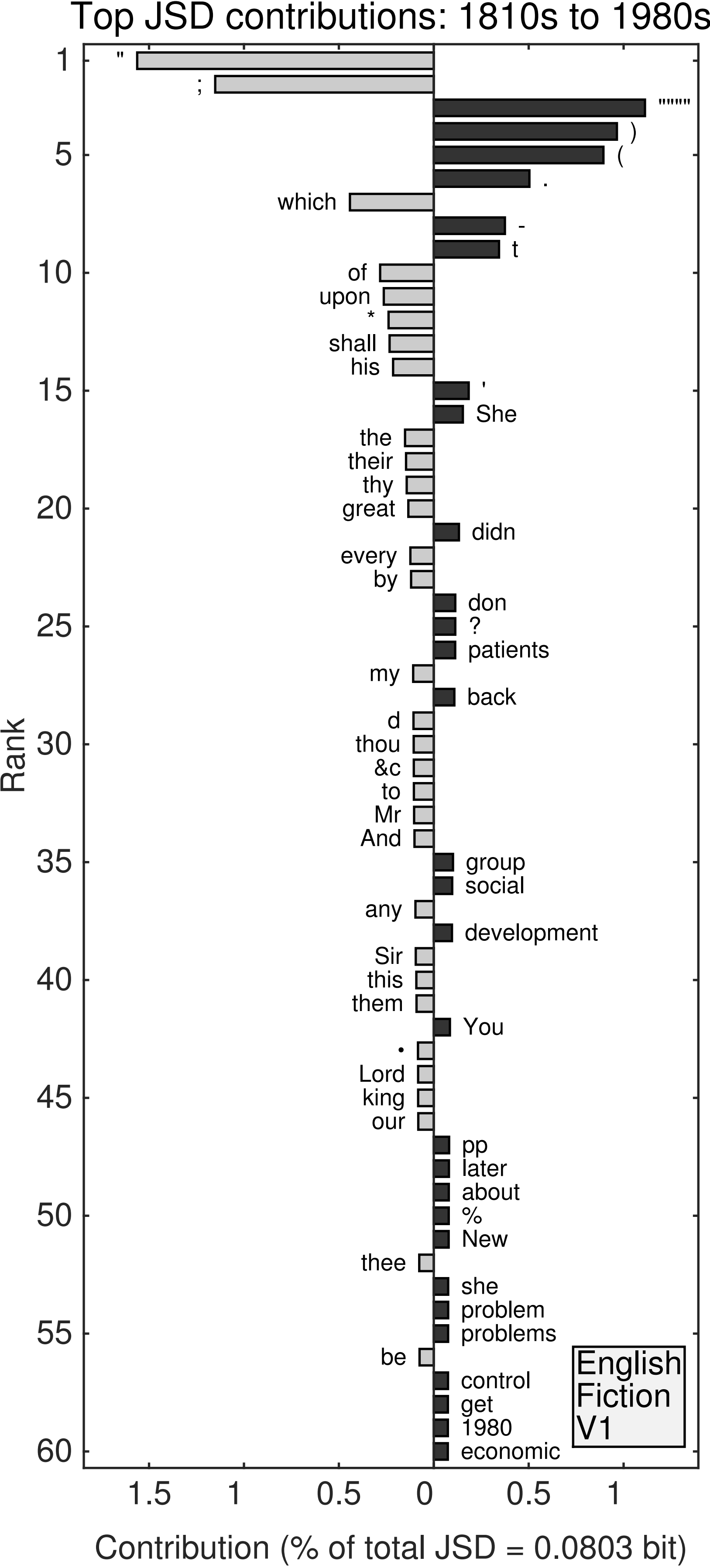

Top JSD contributions: 1810s to 1980s
Rank
Contribution (% of total JSD = 0.0803 bit)
English Fiction V1
"
;
""""
)
(
.
which
-
t
of
upon
*
shall
his
'
She
the
their
thy
great
didn
every
by
don
?
patients
my
back
d
thou
&c
to
Mr
And
group
social
any
development
Sir
this
them
You
•
Lord
king
our
pp
later
about
%
New
thee
she
problem
problems
be
control
get
1980
economic

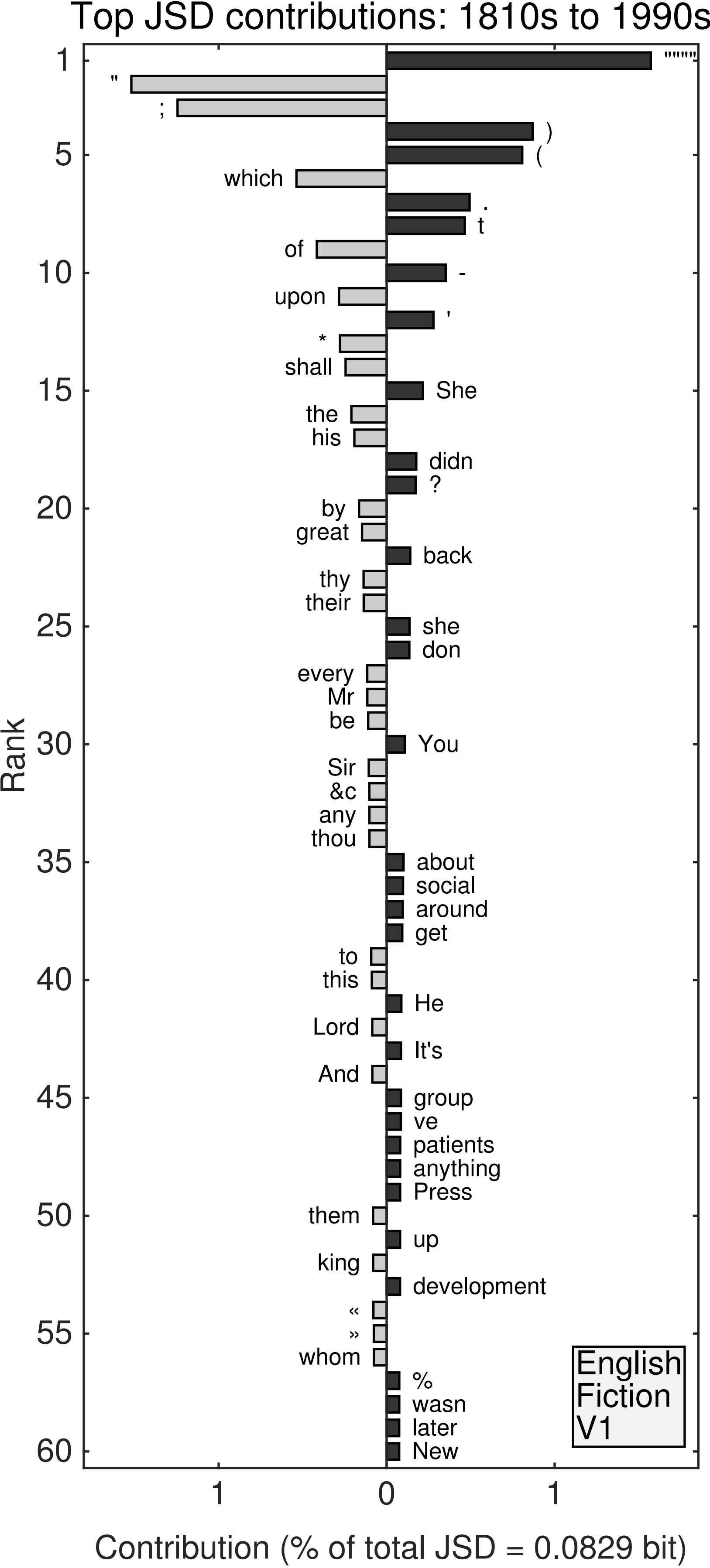

Top JSD contributions: 1810s to 1990s
Rank
1
5
10
15
20
25
30
35
40
45
50
55
60
""""
"
;
)
(
which
.
t
of
-
upon
'
*
shall
She
the
his
didn
?
by
great
back
thy
their
she
don
every
Mr
be
You
Sir
&c
any
thou
about
social
around
get
to
this
He
Lord
It's
And
group
ve
patients
anything
Press
them
up
king
development
«
»
whom
%
wasn
later
New
English Fiction V1
1
0
1
Contribution (% of total JSD = 0.0829 bit)

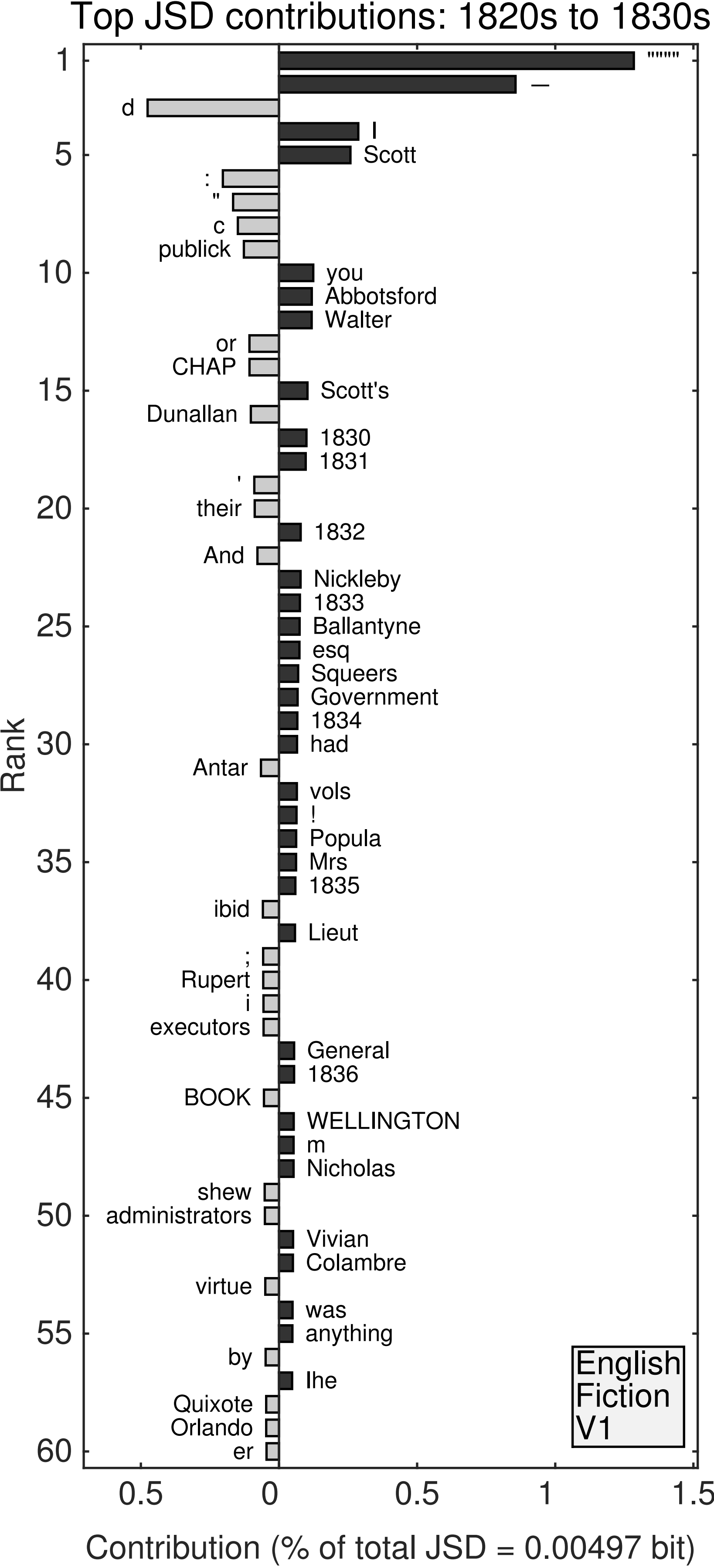

Top JSD contributions: 1820s to 1830s
Rank
Contribution (% of total JSD = 0.00497 bit)
0.5
0
0.5
1
1.5
1
5
10
15
20
25
30
35
40
45
50
55
60
""""
—
d
I
Scott
:
"
c
publick
you
Abbotsford
Walter
or
CHAP
Scott's
Dunallan
1830
1831
'
their
1832
And
Nickleby
1833
Ballantyne
esq
Squeers
Government
1834
had
Antar
vols
!
Popula
Mrs
1835
ibid
Lieut
;
Rupert
i
executors
General
1836
BOOK
WELLINGTON
m
Nicholas
shew
administrators
Vivian
Colambre
virtue
was
anything
by
Ihe
Quixote
Orlando
er
English Fiction V1

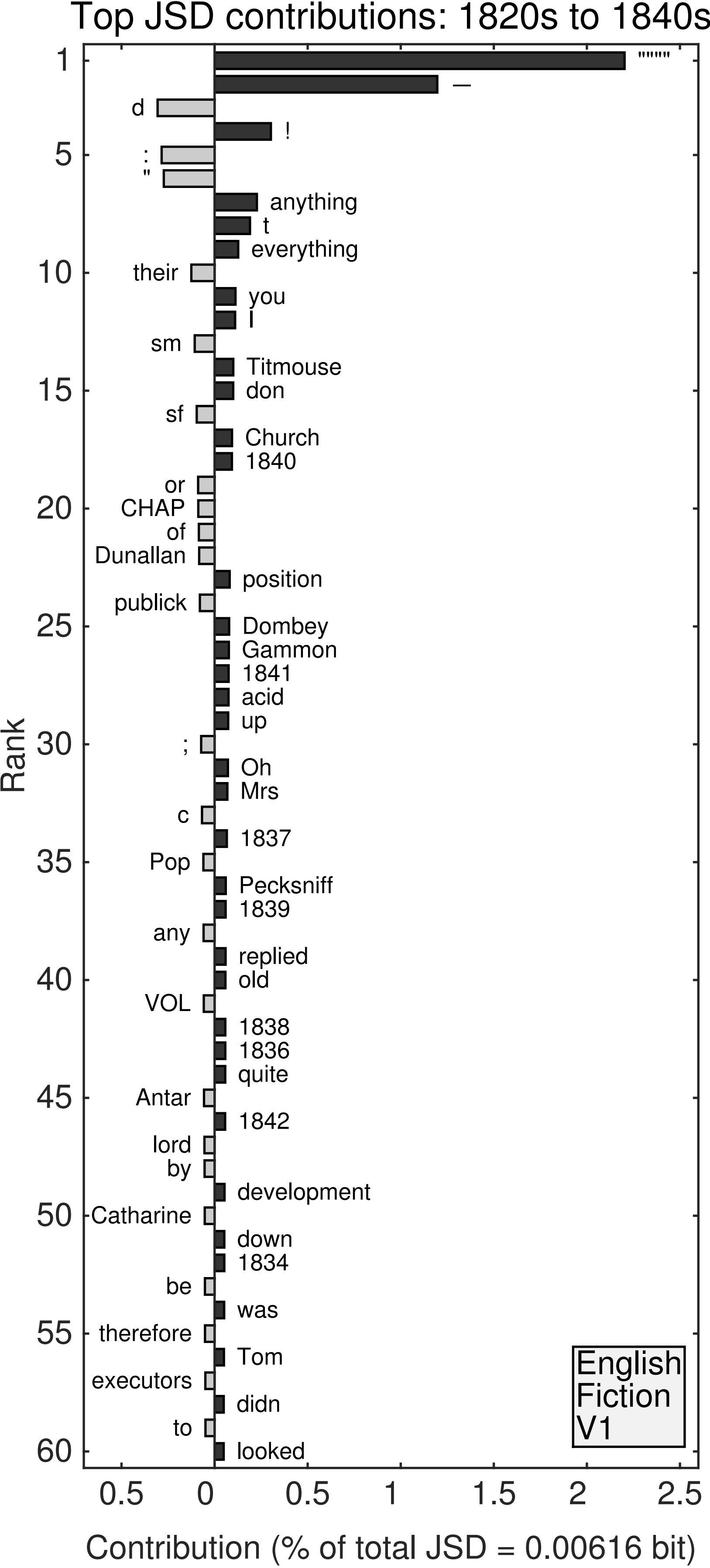

Top JSD contributions: 1820s to 1840s
Rank
Contribution (% of total JSD = 0.00616 bit)
"""""
—
d
!
:
"
anything
t
everything
their
you
I
sm
Titmouse
don
sf
Church
1840
or
CHAP
of
Dunallan
position
publick
Dombey
Gammon
1841
acid
up
;
Oh
Mrs
c
1837
Pop
Pecksniff
1839
any
replied
old
VOL
1838
1836
quite
Antar
1842
lord
by
development
Catharine
down
1834
be
was
therefore
Tom
executors
didn
to
looked
English Fiction V1

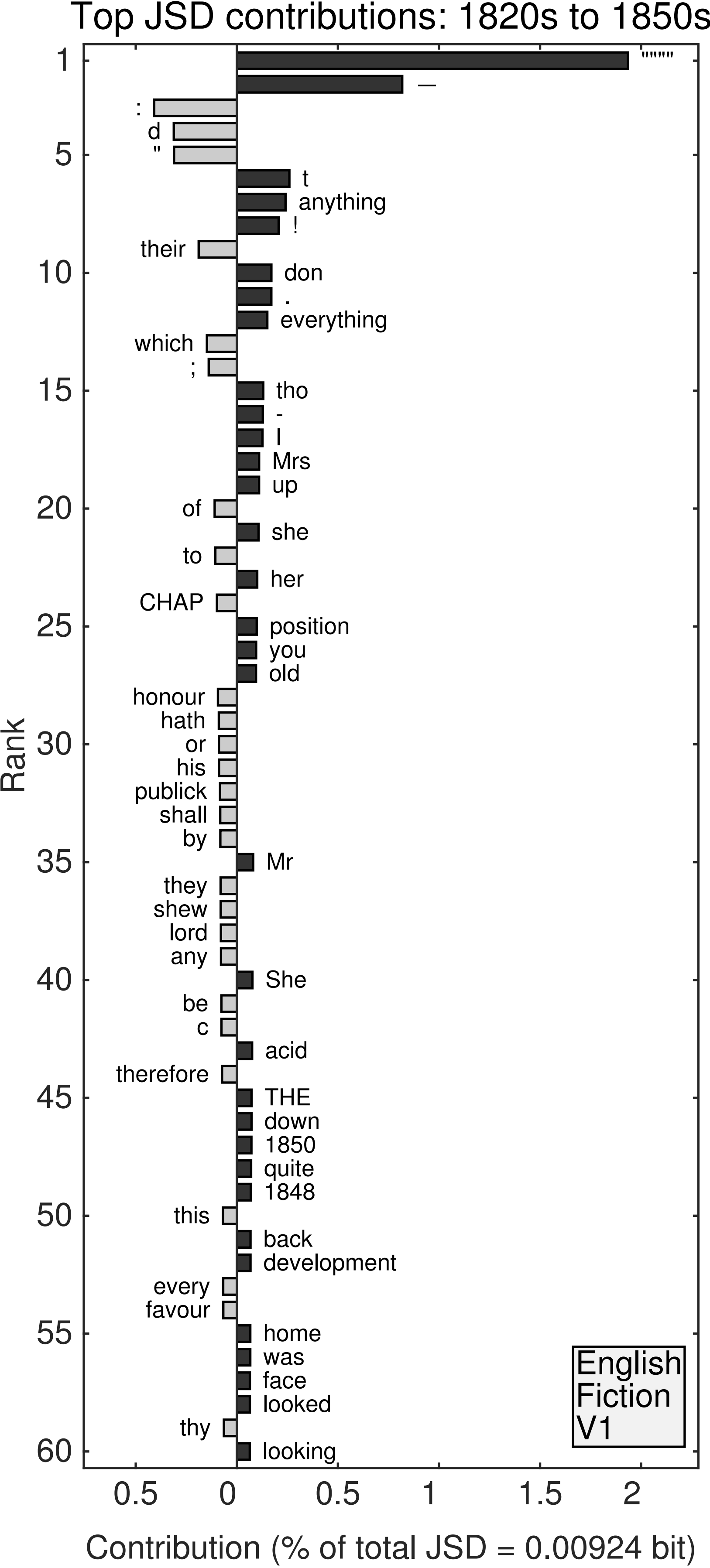

Top JSD contributions: 1820s to 1850s
Rank
Contribution (% of total JSD = 0.00924 bit)
0.5
0
0.5
1
1.5
2
1
5
10
15
20
25
30
35
40
45
50
55
60
""""
—
:
d
"
t
anything
!
their
don
.
everything
which
;
tho
-
I
Mrs
up
of
she
to
her
CHAP
position
you
old
honour
hath
or
his
publick
shall
by
Mr
they
shew
lord
any
She
be
c
acid
therefore
THE
down
1850
quite
1848
this
back
development
every
favour
home
was
face
looked
thy
looking
English
Fiction
V1

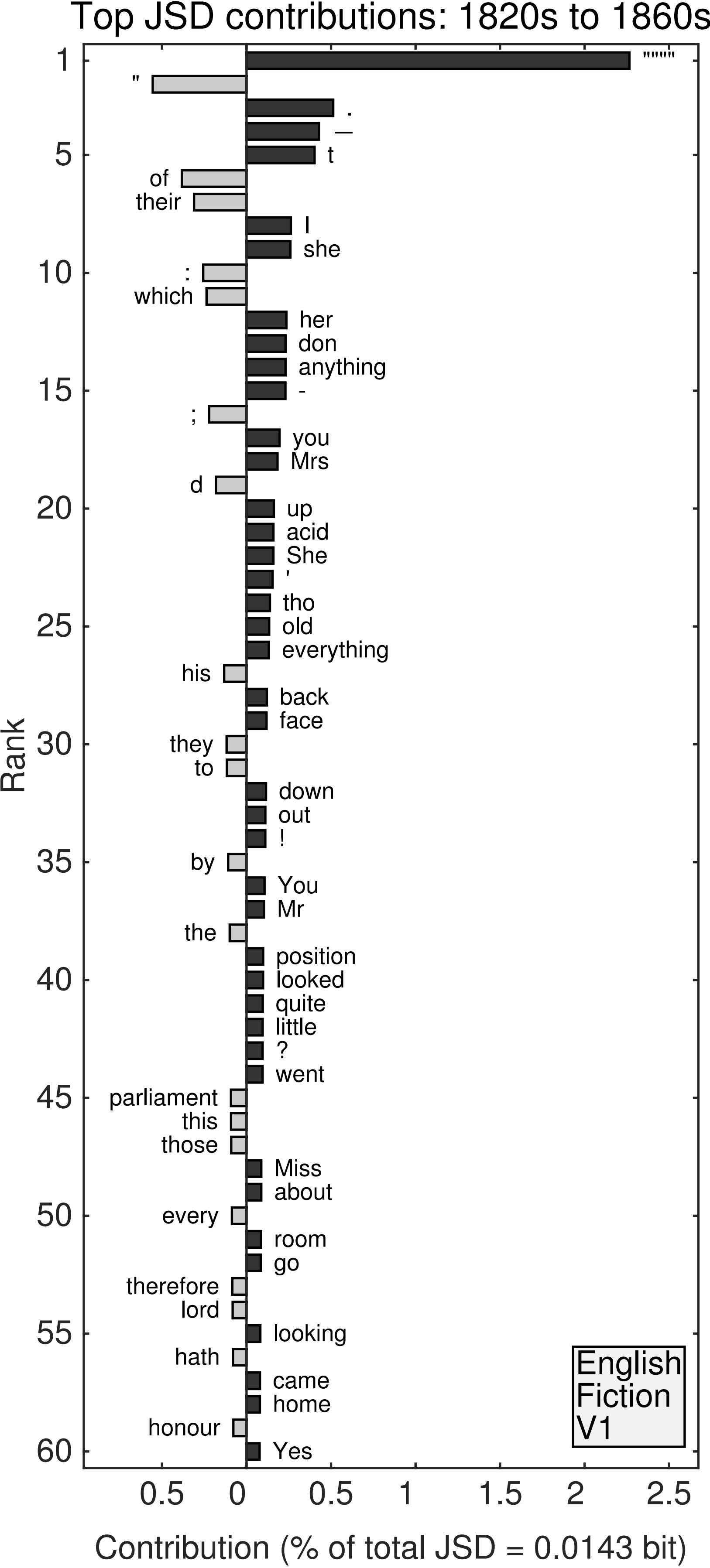
Top JSD contributions: 1820s to 1860s
Rank
Contribution (% of total JSD = 0.0143 bit)
English Fiction V1
1 5 10 15 20 25 30 35 40 45 50 55 60
0.5 0 0.5 1 1.5 2 2.5
""""
"
.
—
t
of
their
I
she
:
which
her
don
anything
-
;
you
Mrs
d
up
acid
She
'
tho
old
everything
his
back
face
they
to
down
out
!
by
You
Mr
the
position
looked
quite
little
?
went
parliament
this
those
Miss
about
every
room
go
therefore
lord
looking
hath
came
home
honour
Yes

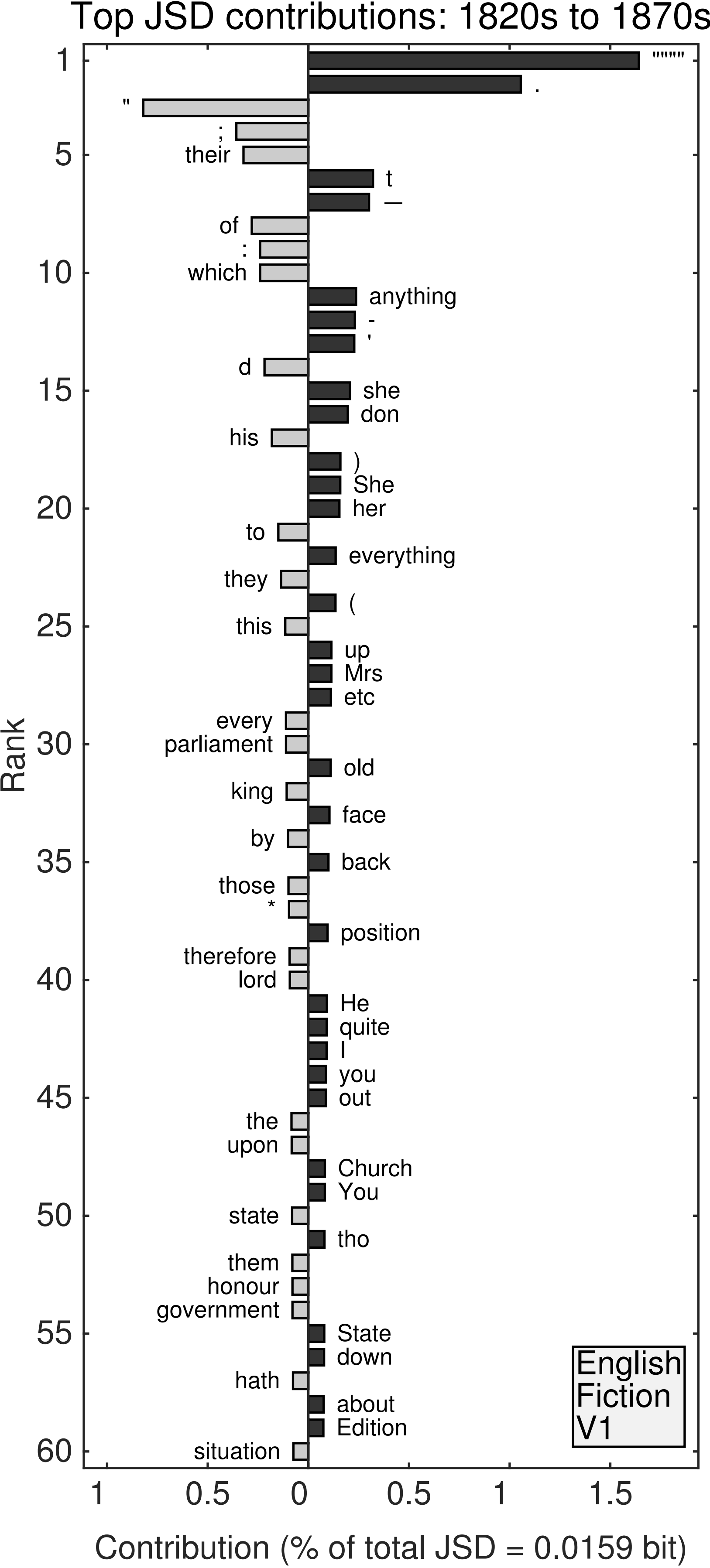
Top JSD contributions: 1820s to 1870s
Rank
Contribution (% of total JSD = 0.0159 bit)
""""
.
"
;
their
t
—
of
:
which
anything
-
'
d
she
don
his
)
She
her
to
everything
they
(
this
up
Mrs
etc
every
parliament
old
king
face
by
back
those
*
position
therefore
lord
He
quite
I
you
out
the
upon
Church
You
state
tho
them
honour
government
State
down
hath
about
Edition
situation
English Fiction V1

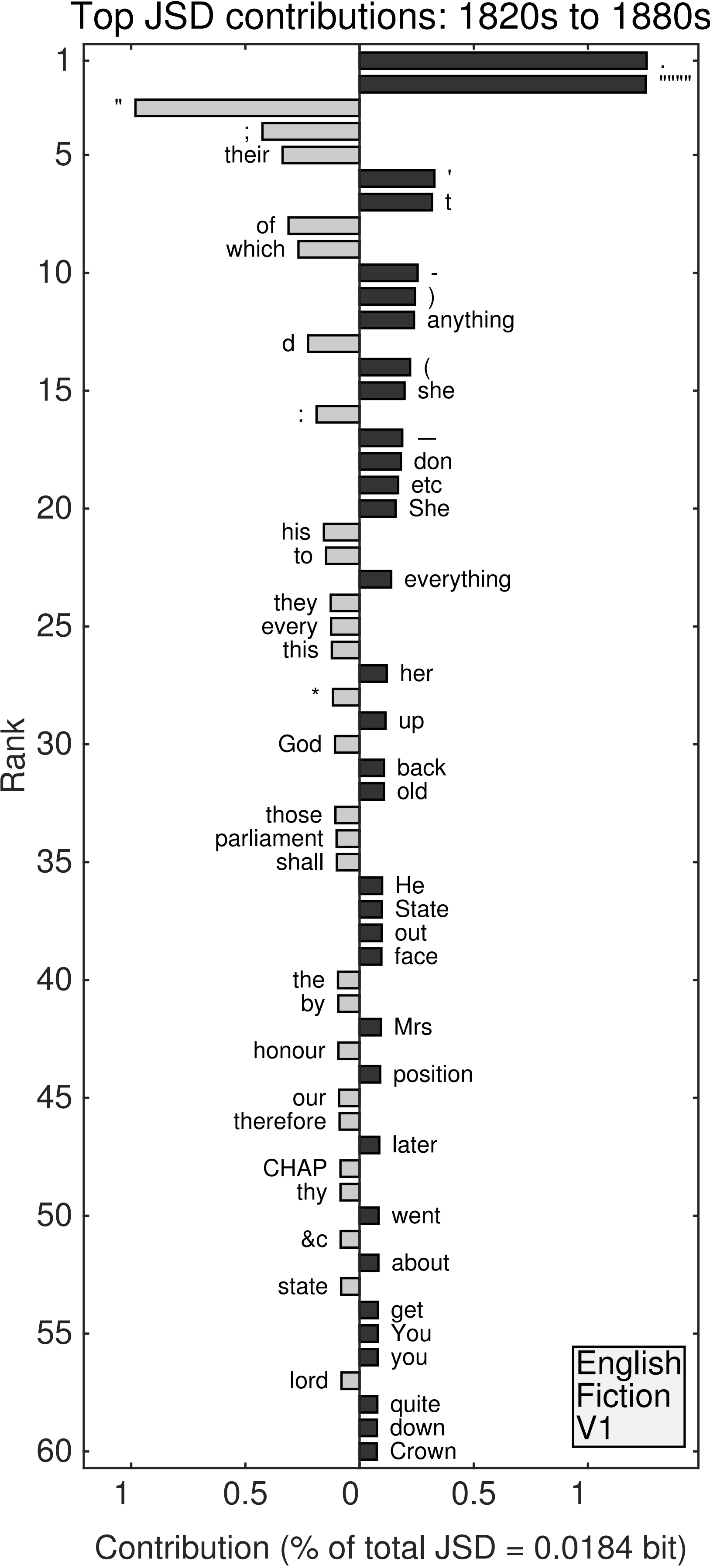

Top JSD contributions: 1820s to 1880s
Rank
Contribution (% of total JSD = 0.0184 bit)
English Fiction V1
.
""""
"
;
their
'
t
of
which
-
)
anything
d
(
she
:
—
don
etc
She
his
to
everything
they
every
this
her
*
up
God
back
old
those
parliament
shall
He
State
out
face
the
by
Mrs
honour
position
our
therefore
later
CHAP
thy
went
&c
about
state
get
You
you
lord
quite
down
Crown

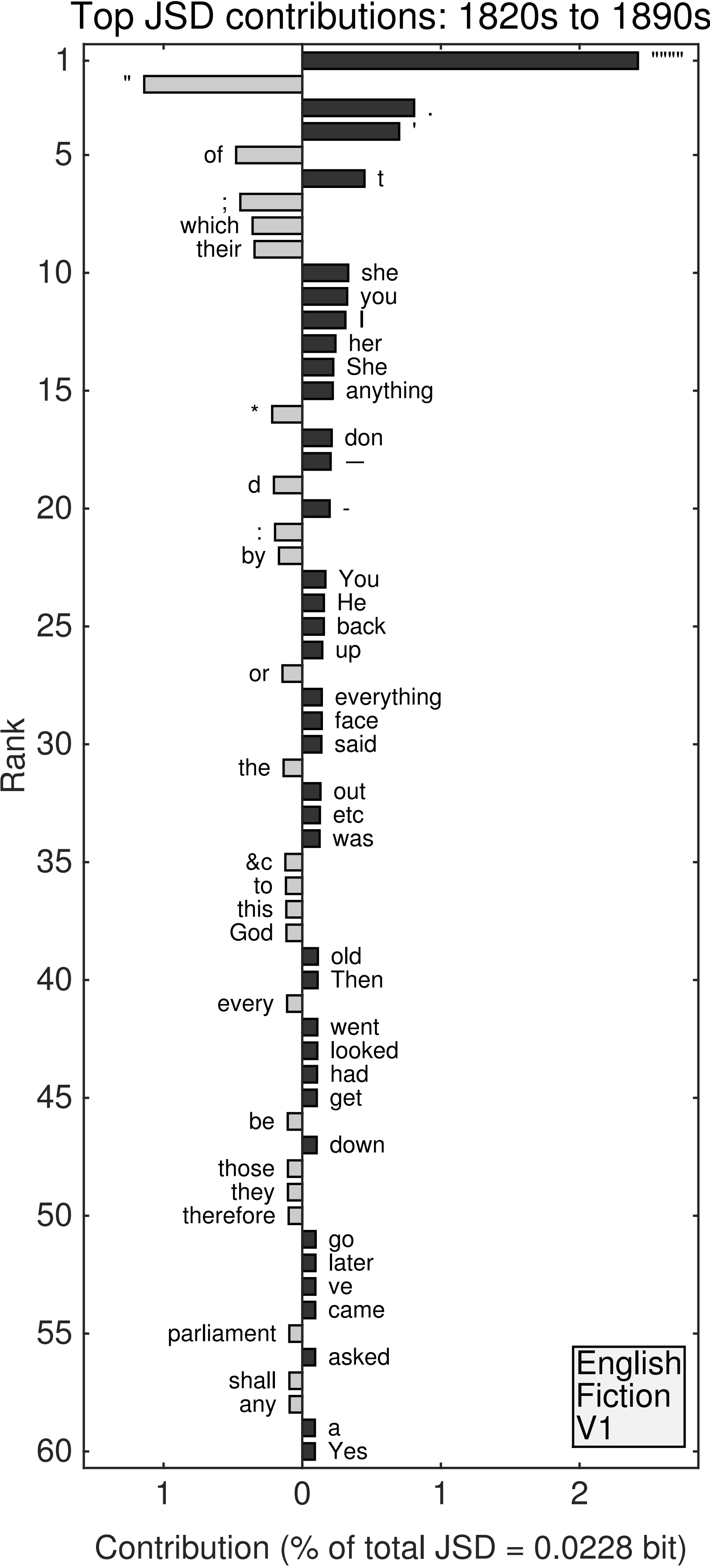
Top JSD contributions: 1820s to 1890s
Rank
Contribution (% of total JSD = 0.0228 bit)
English Fiction V1
"""""
"
.
'
of
t
;
which
their
she
you
I
her
She
anything
*
don
—
d
-
:
by
You
He
back
up
or
everything
face
said
the
out
etc
was
&c
to
this
God
old
Then
every
went
looked
had
get
be
down
those
they
therefore
go
later
ve
came
parliament
asked
shall
any
a
Yes

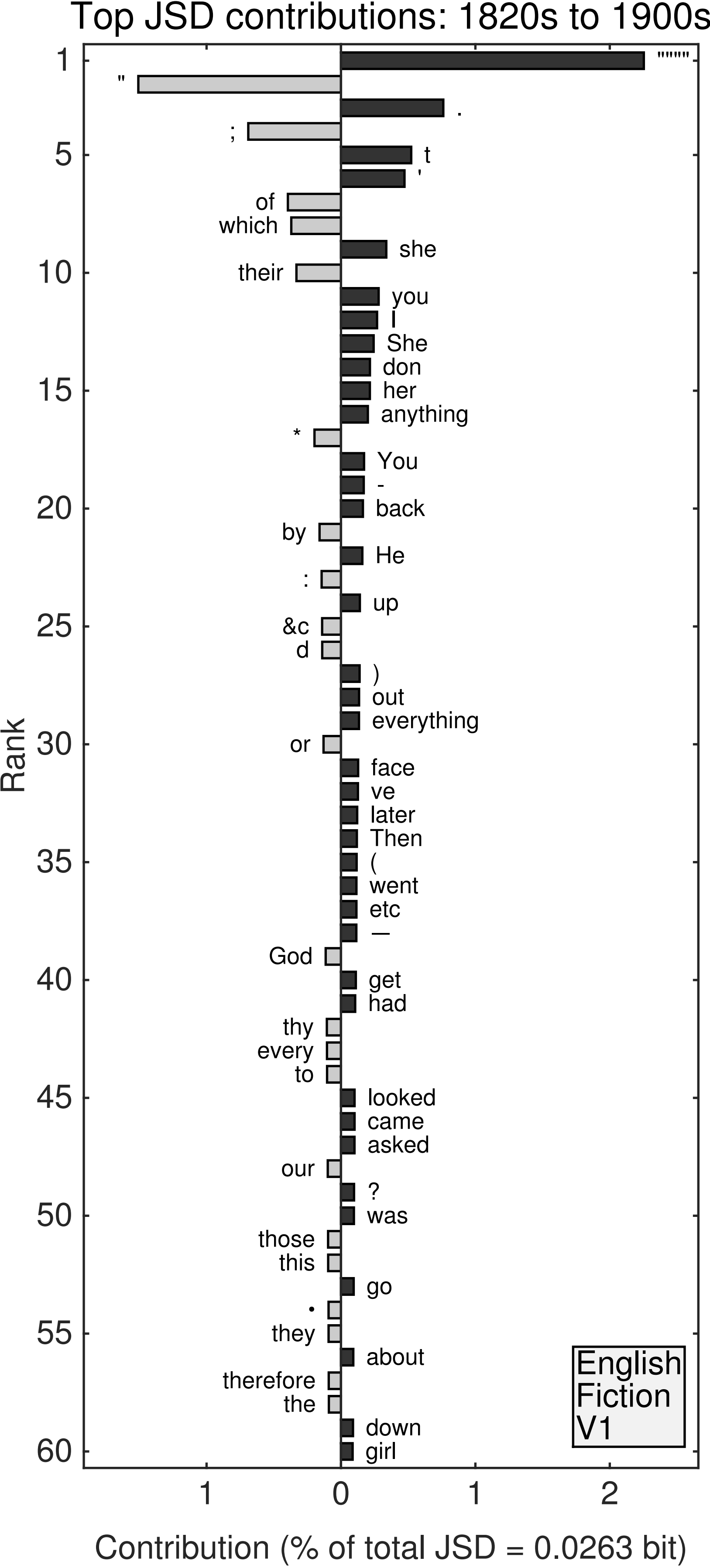
Top JSD contributions: 1820s to 1900s
Rank
1
5
10
15
20
25
30
35
40
45
50
55
60
"""""
"
.
;
t
'
of
which
she
their
you
I
She
don
her
anything
*
You
-
back
by
He
:
up
&c
d
)
out
everything
or
face
ve
later
Then
(
went
etc
—
God
get
had
thy
every
to
looked
came
asked
our
?
was
those
this
go
•
they
about
therefore
the
down
girl
English
Fiction
V1
1
0
1
2
Contribution (% of total JSD = 0.0263 bit)

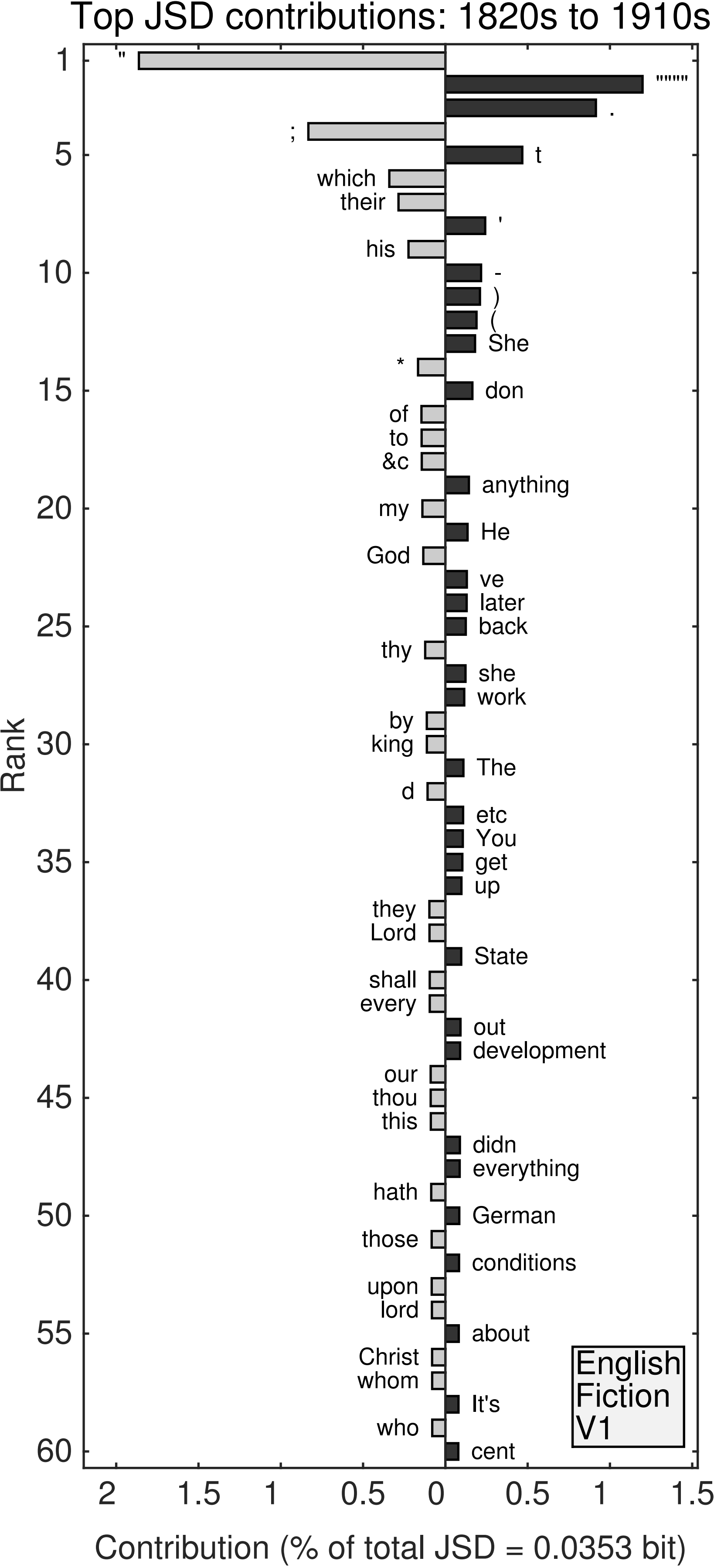

Top JSD contributions: 1820s to 1910s
Rank
Contribution (% of total JSD = 0.0353 bit)
English Fiction V1
"
""""
.
;
t
which
their
'
his
-
)
(
She
*
don
of
to
&c
anything
my
He
God
ve
later
back
thy
she
work
by
king
The
d
etc
You
get
up
they
Lord
State
shall
every
out
development
our
thou
this
didn
everything
hath
German
those
conditions
upon
lord
about
Christ
whom
It's
who
cent

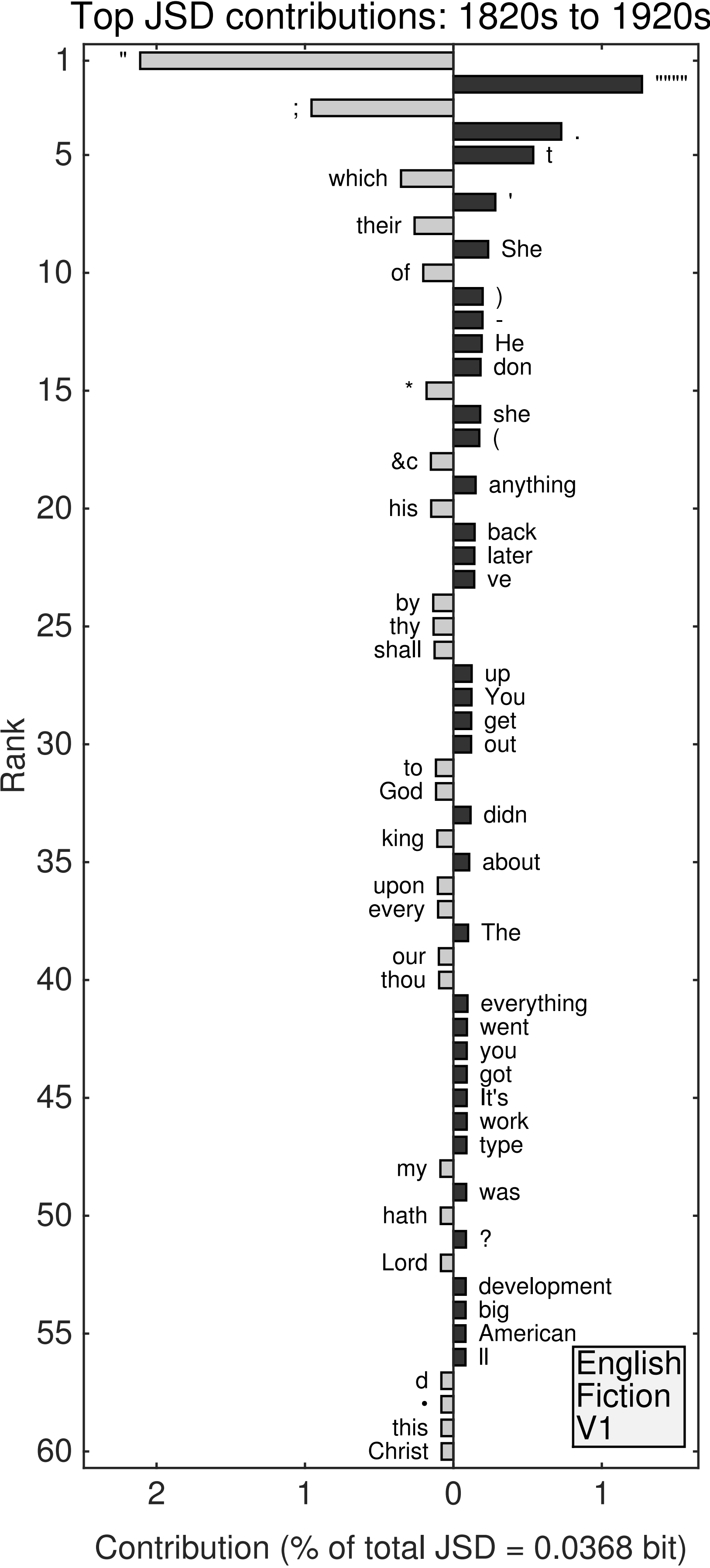

Top JSD contributions: 1820s to 1920s
Rank
1
5
10
15
20
25
30
35
40
45
50
55
60
"
""""
;
.
t
which
'
their
She
of
)
-
He
don
*
she
(
&c
anything
his
back
later
ve
by
thy
shall
up
You
get
out
to
God
didn
king
about
upon
every
The
our
thou
everything
went
you
got
It's
work
type
my
was
hath
?
Lord
development
big
American
ll
d
•
this
Christ
English
Fiction
V1
2
1
0
1
Contribution (% of total JSD = 0.0368 bit)

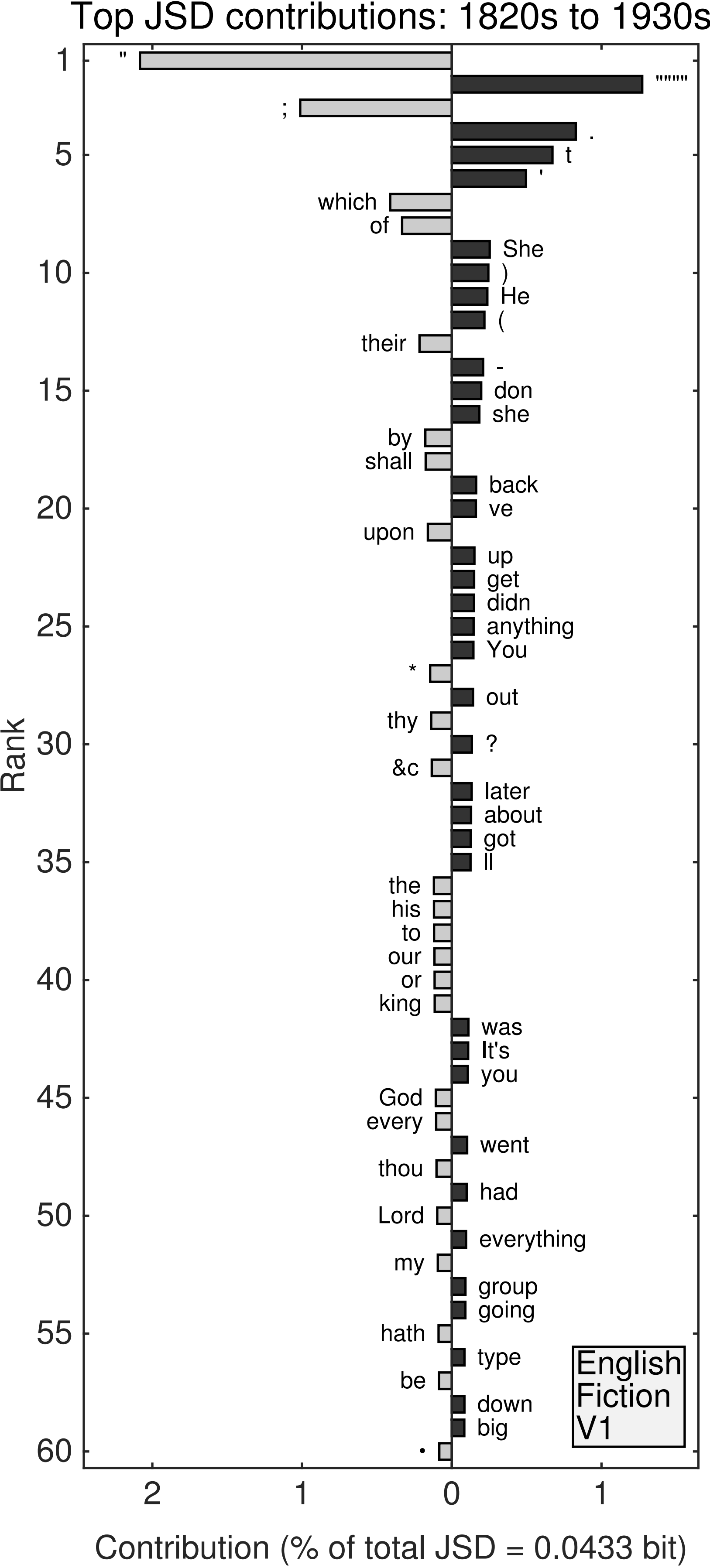

Top JSD contributions: 1820s to 1930s
Rank
Contribution (% of total JSD = 0.0433 bit)
"
""""
;
.
t
'
which
of
She
)
He
(
their
-
don
she
by
shall
back
ve
upon
up
get
didn
anything
You
*
out
thy
?
&c
later
about
got
ll
the
his
to
our
or
king
was
It's
you
God
every
went
thou
had
Lord
everything
my
group
going
hath
type
be
down
big
•
English
Fiction
V1

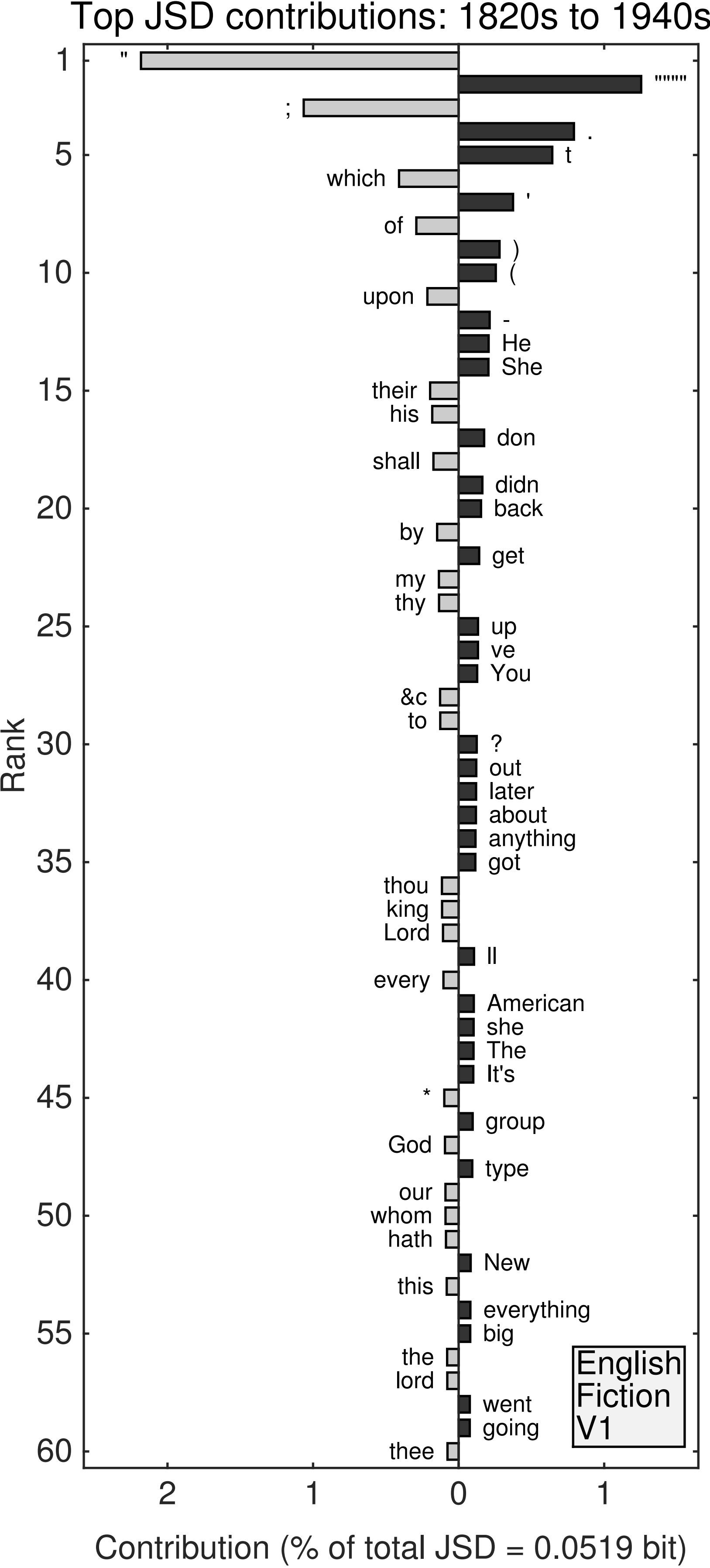

Top JSD contributions: 1820s to 1940s
Rank
1
5
10
15
20
25
30
35
40
45
50
55
60
"
""""
;
.
t
which
'
of
)
(
upon
-
He
She
their
his
don
shall
didn
back
by
get
my
thy
up
ve
You
&c
to
?
out
later
about
anything
got
thou
king
Lord
ll
every
American
she
The
It's
*
group
God
type
our
whom
hath
New
this
everything
big
the
lord
went
going
thee
English
Fiction
V1
2
1
0
1
Contribution (% of total JSD = 0.0519 bit)

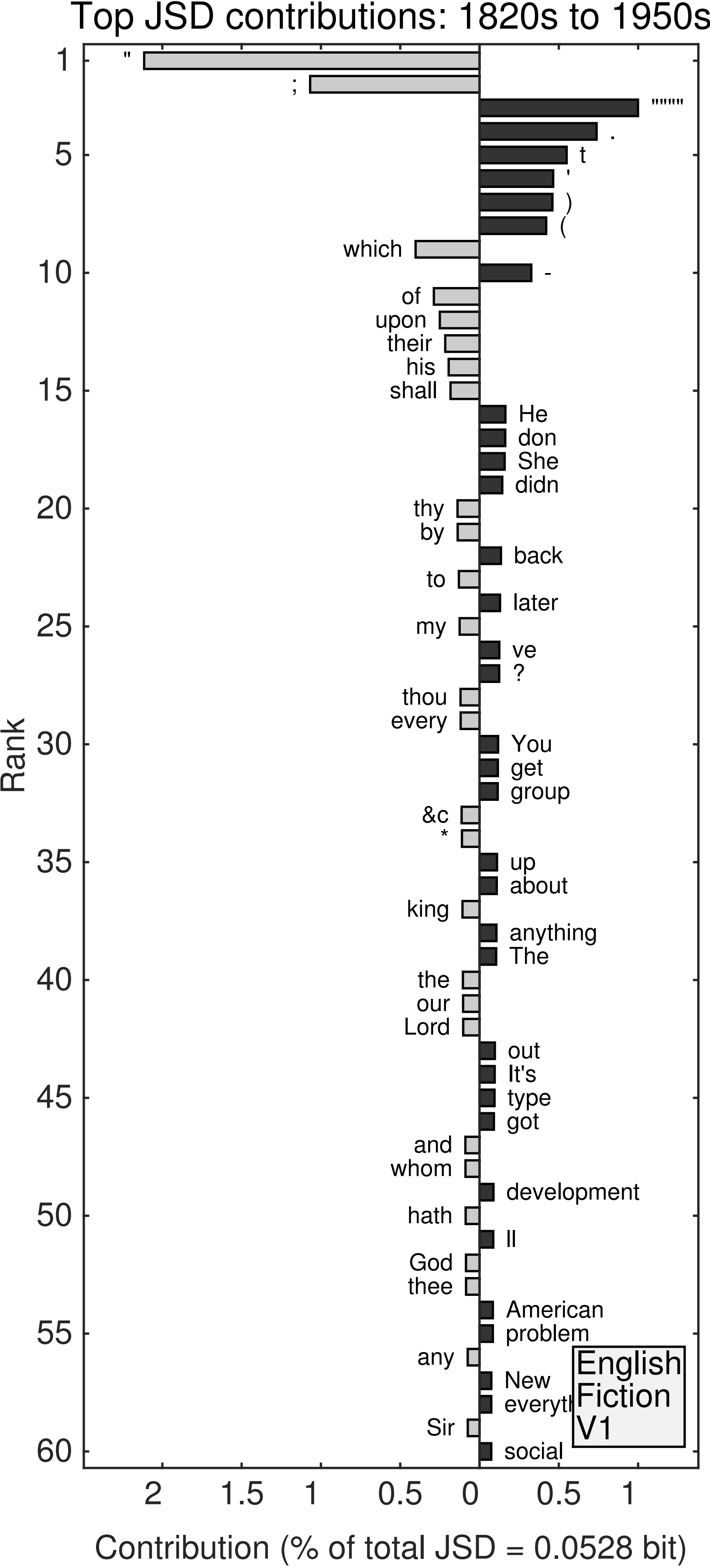

Top JSD contributions: 1820s to 1950s
Rank
Contribution (% of total JSD = 0.0528 bit)
English Fiction V1
"
;
""""
.
t
'
)
(
which
-
of
upon
their
his
shall
He
don
She
didn
thy
by
back
to
later
my
ve
?
thou
every
You
get
group
&c
*
up
about
king
anything
The
the
our
Lord
out
It's
type
got
and
whom
development
hath
ll
God
thee
American
problem
any
New
Sir
social

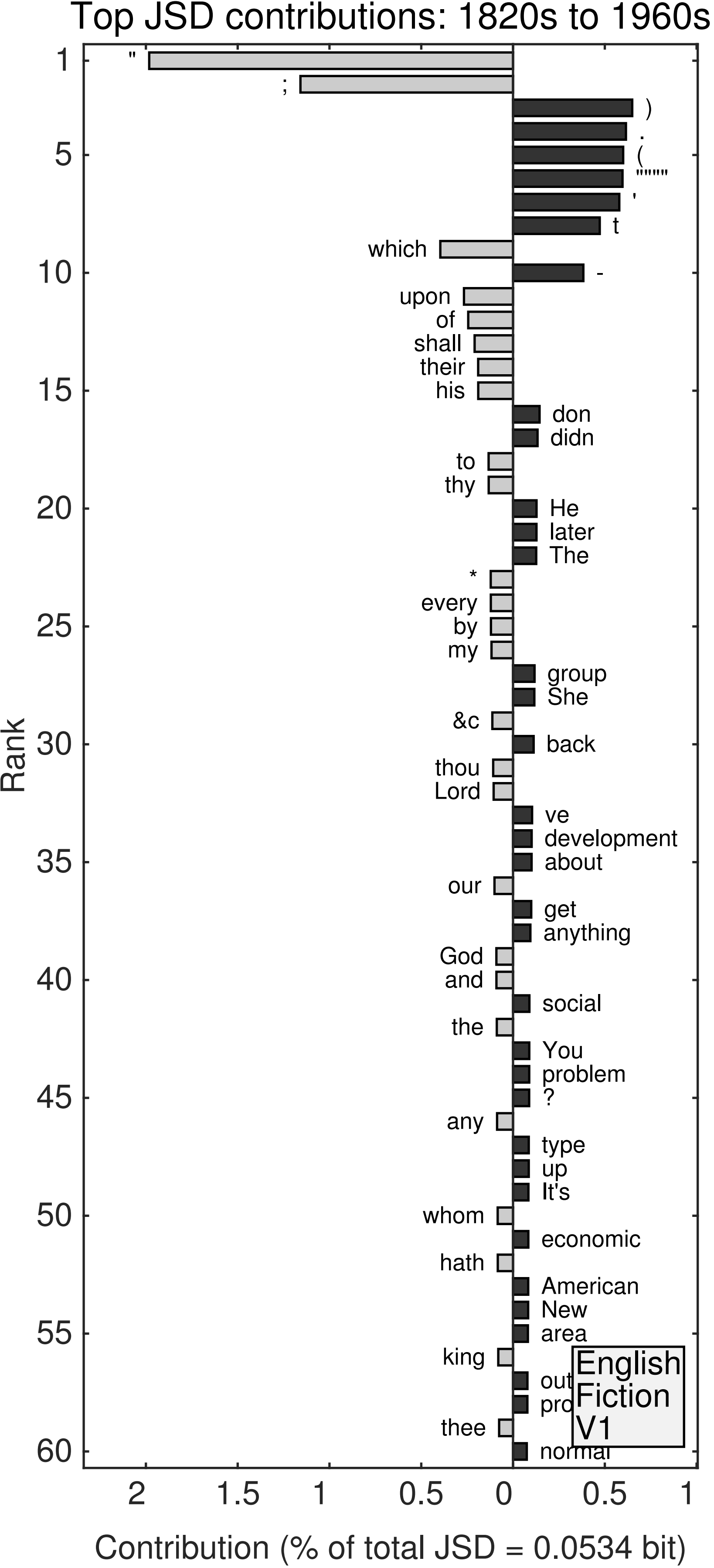
Top JSD contributions: 1820s to 1960s
Rank
Contribution (% of total JSD = 0.0534 bit)
English Fiction V1
"
;
)
.
(
'
t
which
-
upon
of
shall
their
his
don
didn
to
thy
He
later
The
*
every
by
my
group
She
&c
back
thou
Lord
ve
development
about
our
get
anything
God
and
social
the
You
problem
?
any
type
up
It's
whom
economic
hath
American
New
area
king
thee
normal

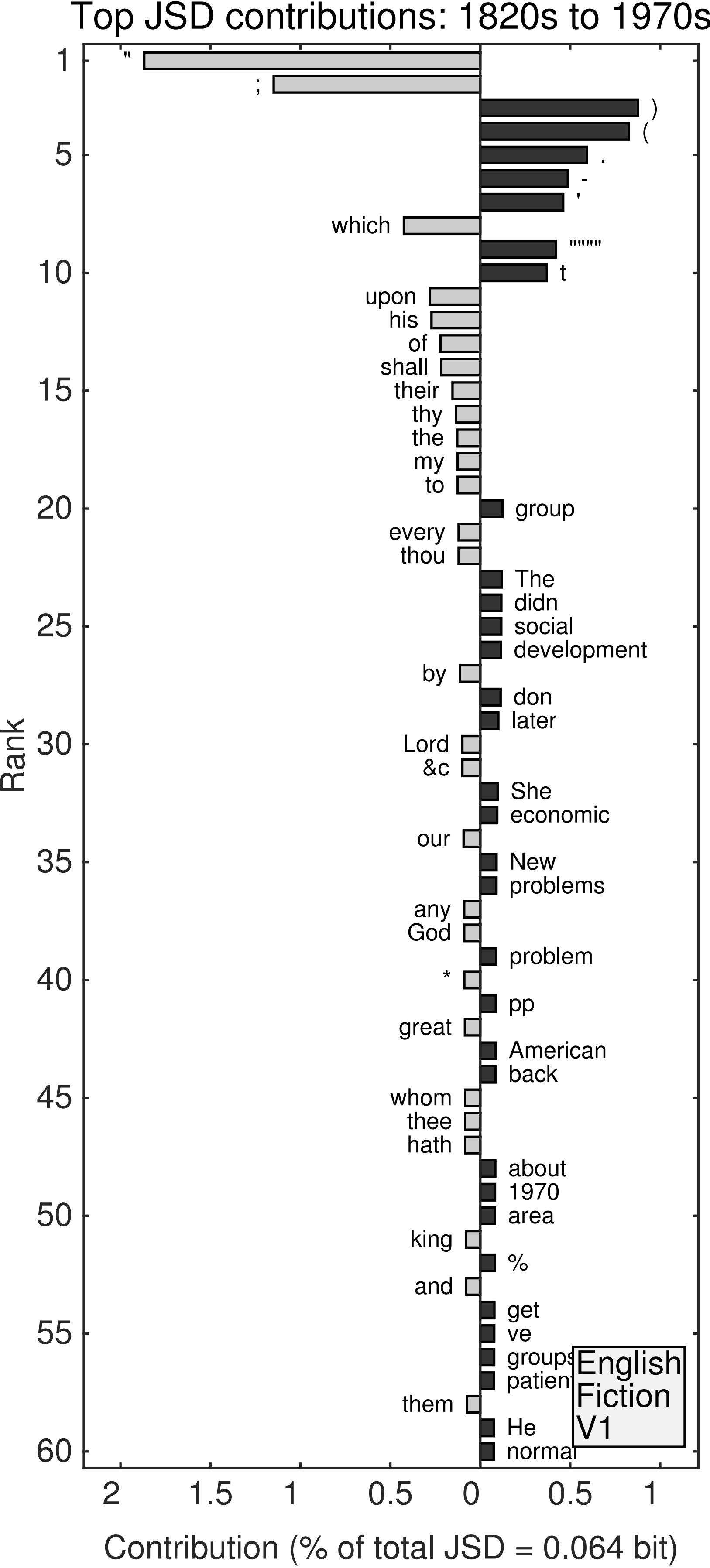

Top JSD contributions: 1820s to 1970s
Rank
Contribution (% of total JSD = 0.064 bit)
English Fiction V1
"
;
)
(
.
-
'
which
""""
t
upon
his
of
shall
their
thy
the
my
to
group
every
thou
The
didn
social
development
by
don
later
Lord
&c
She
economic
our
New
problems
any
God
problem
*
pp
great
American
back
whom
thee
hath
about
1970
area
king
%
and
get
ve
groups
patient
them
He
normal

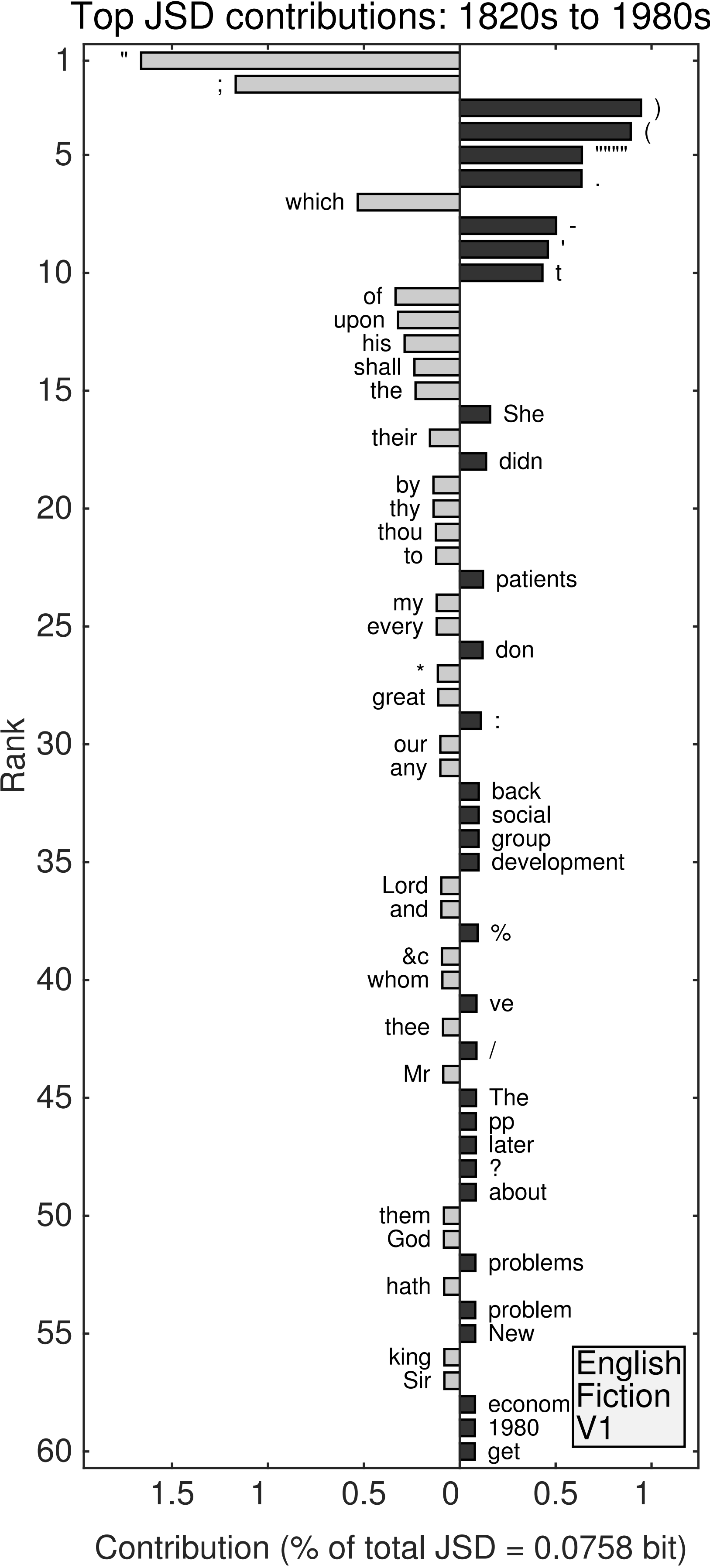

Top JSD contributions: 1820s to 1980s
Rank
Contribution (% of total JSD = 0.0758 bit)
"
;
)
(
""""
.
which
-
'
t
of
upon
his
shall
the
She
their
didn
by
thy
thou
to
patients
my
every
don
*
great
:
our
any
back
social
group
development
Lord
and
%
&c
whom
ve
thee
/
Mr
The
pp
later
?
about
them
God
problems
hath
problem
New
king
Sir
econom
1980
get
English Fiction V1

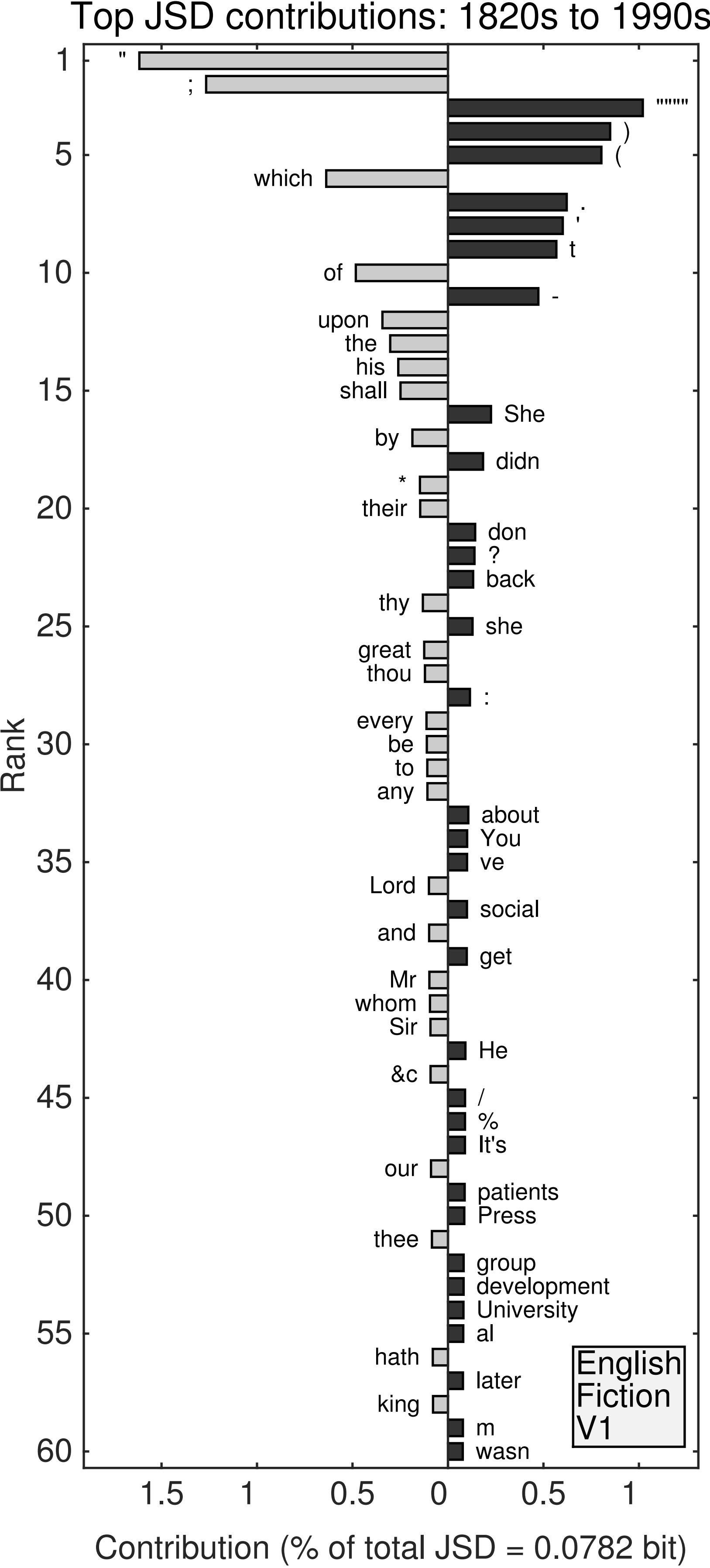

Top JSD contributions: 1820s to 1990s
Rank
Contribution (% of total JSD = 0.0782 bit)
1.5
1
0.5
0
0.5
1
1
5
10
15
20
25
30
35
40
45
50
55
60
"
;
""""
)
(
which
.
'
t
of
-
upon
the
his
shall
She
by
didn
*
their
don
?
back
thy
she
great
thou
:
every
be
to
any
about
You
ve
Lord
social
and
get
Mr
whom
Sir
He
&c
/
%
It's
our
patients
Press
thee
group
development
University
al
hath
later
king
m
wasn
English
Fiction
V1

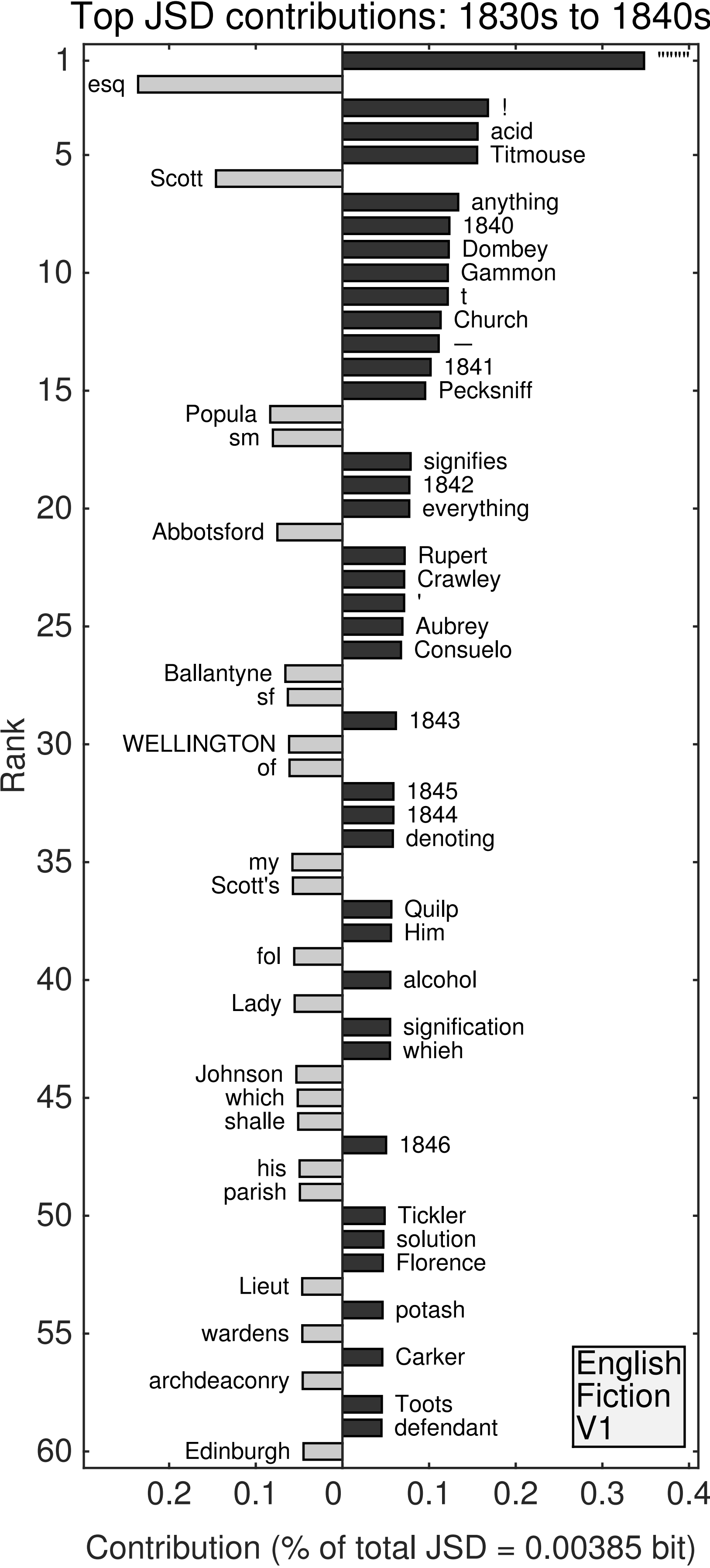

Top JSD contributions: 1830s to 1840s
Rank
""""""
esq
!
acid
Titmouse
Scott
anything
1840
Dombey
Gammon
t
Church
—
1841
Pecksniff
Popula
sm
signifies
1842
everything
Abbotsford
Rupert
Crawley
'
Aubrey
Consuelo
Ballantyne
sf
1843
WELLINGTON
of
1845
1844
denoting
my
Scott's
Quilp
Him
fol
alcohol
Lady
signification
whieh
Johnson
which
shalle
1846
his
parish
Tickler
solution
Florence
Lieut
potash
wardens
Carker
archdeaconry
Toots
defendant
Edinburgh
English Fiction V1
Contribution (% of total JSD = 0.00385 bit)

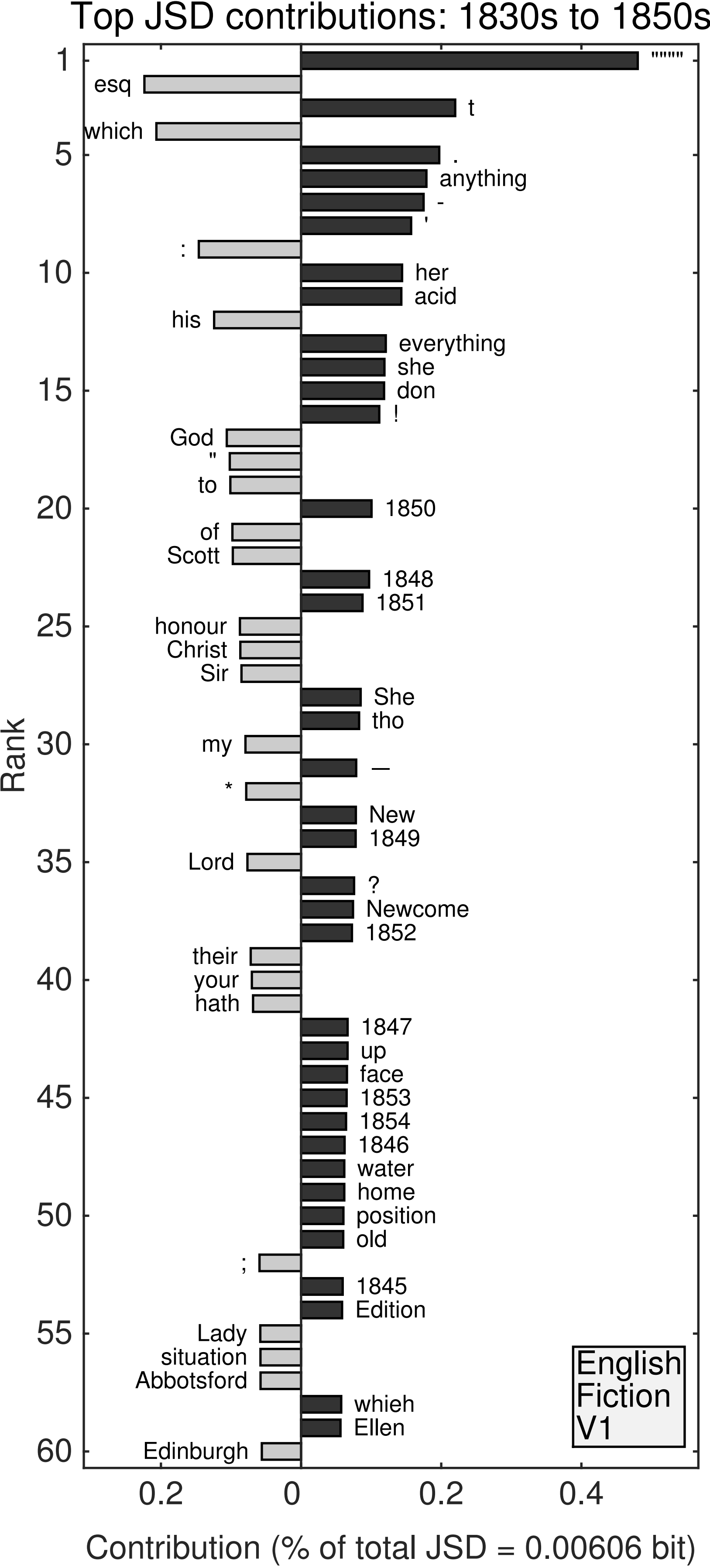
Top JSD contributions: 1830s to 1850s
Rank
Contribution (% of total JSD = 0.00606 bit)
0.2
0
0.2
0.4
""""
esq
t
which
.
anything
-
'
:
her
acid
his
everything
she
don
!
God
"
to
1850
of
Scott
1848
1851
honour
Christ
Sir
She
tho
my
—
*
New
1849
Lord
?
Newcome
1852
their
your
hath
1847
up
face
1853
1854
1846
water
home
position
old
;
1845
Edition
Lady
situation
Abbotsford
whieh
Ellen
Edinburgh
English Fiction V1

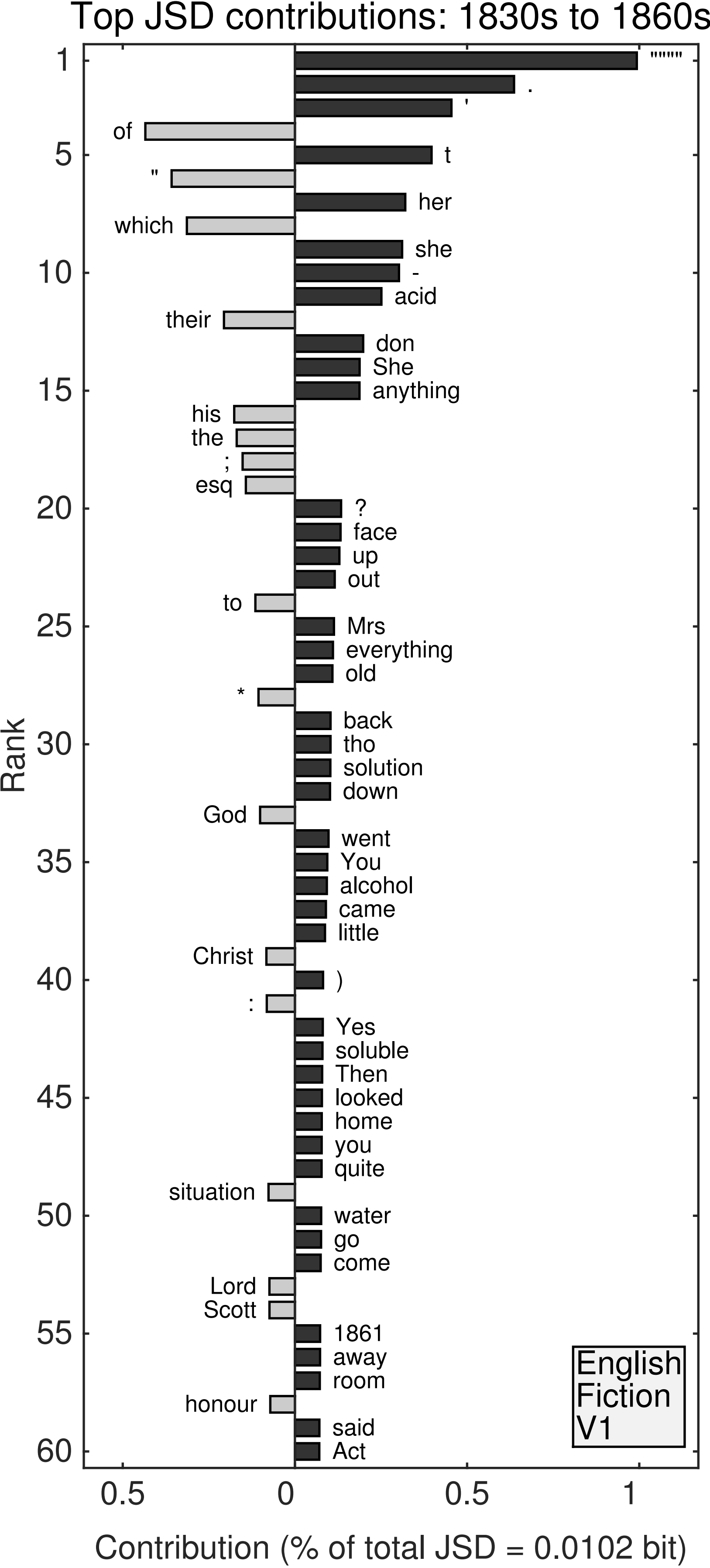

Top JSD contributions: 1830s to 1860s
Rank
Contribution (% of total JSD = 0.0102 bit)
English Fiction V1
""""
.
'
of
t
"
her
which
she
-
acid
their
don
She
anything
his
the
;
esq
?
face
up
out
to
Mrs
everything
old
*
back
tho
solution
down
God
went
You
alcohol
came
little
Christ
)
:
Yes
soluble
Then
looked
home
you
quite
situation
water
go
come
Lord
Scott
1861
away
room
honour
said
Act

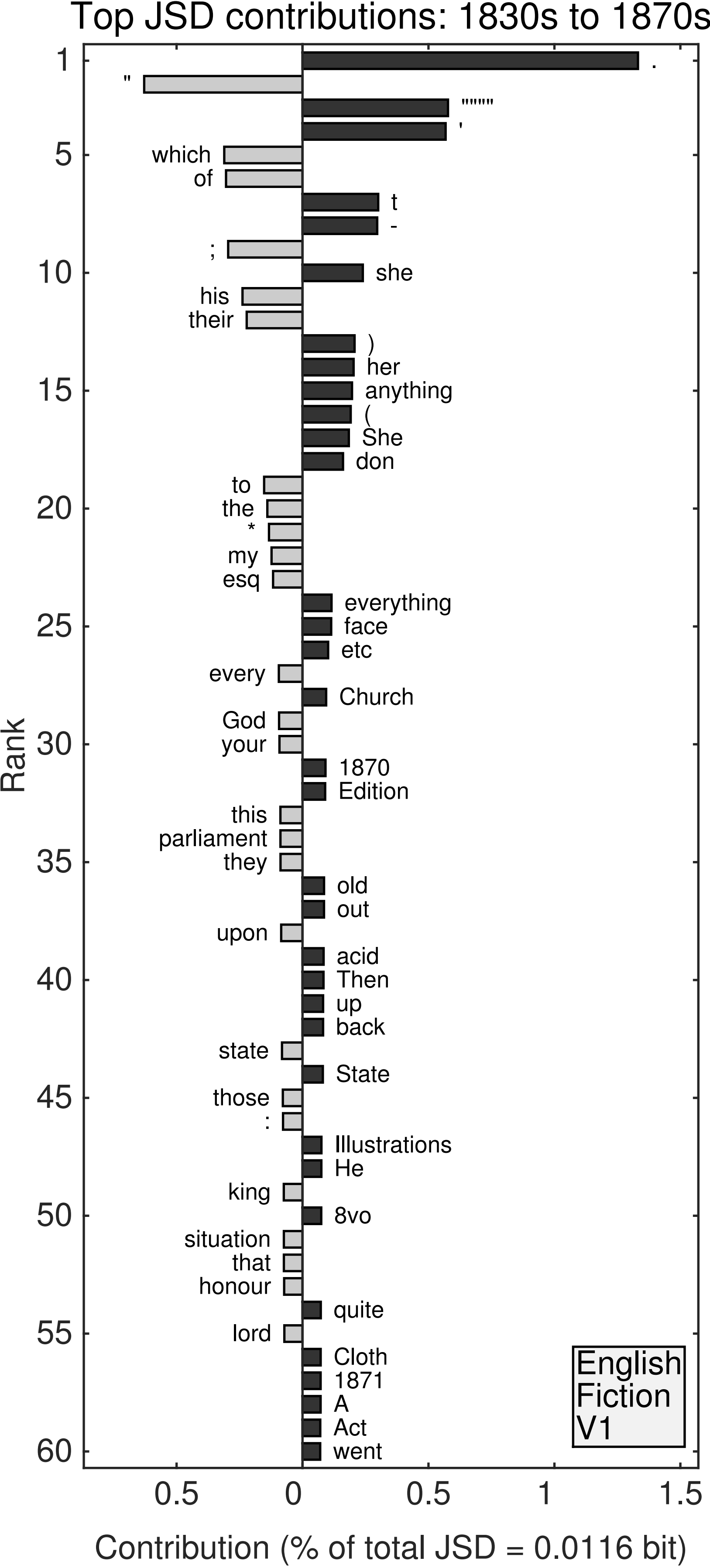

Top JSD contributions: 1830s to 1870s
Rank
Contribution (% of total JSD = 0.0116 bit)
English Fiction V1
1
5
10
15
20
25
30
35
40
45
50
55
60
0.5
0
0.5
1
1.5
.
"
""""
'
which
of
t
-
;
she
his
their
)
her
anything
(
She
don
to
the
*
my
esq
everything
face
etc
every
Church
God
your
1870
Edition
this
parliament
they
old
out
upon
acid
Then
up
back
state
State
those
:
Illustrations
He
king
8vo
situation
that
honour
quite
lord
Cloth
1871
A
Act
went

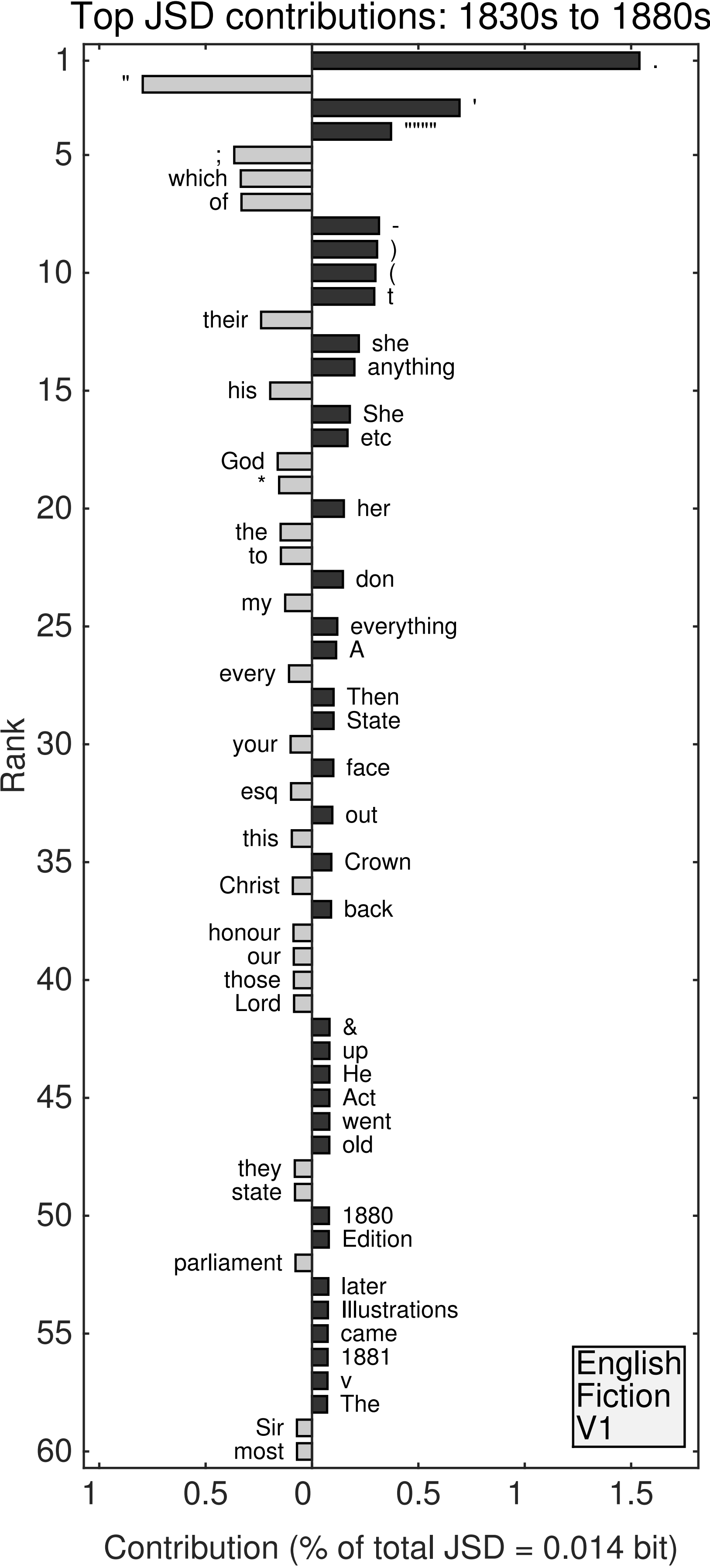
Top JSD contributions: 1830s to 1880s
Rank
Contribution (% of total JSD = 0.014 bit)
English Fiction V1
.
"
'
""""
;
which
of
-
)
(
t
their
she
anything
his
She
etc
God
*
her
the
to
don
my
everything
A
every
Then
State
your
face
esq
out
this
Crown
Christ
back
honour
our
those
Lord
&
up
He
Act
went
old
they
state
1880
Edition
parliament
later
Illustrations
came
1881
v
The
Sir
most

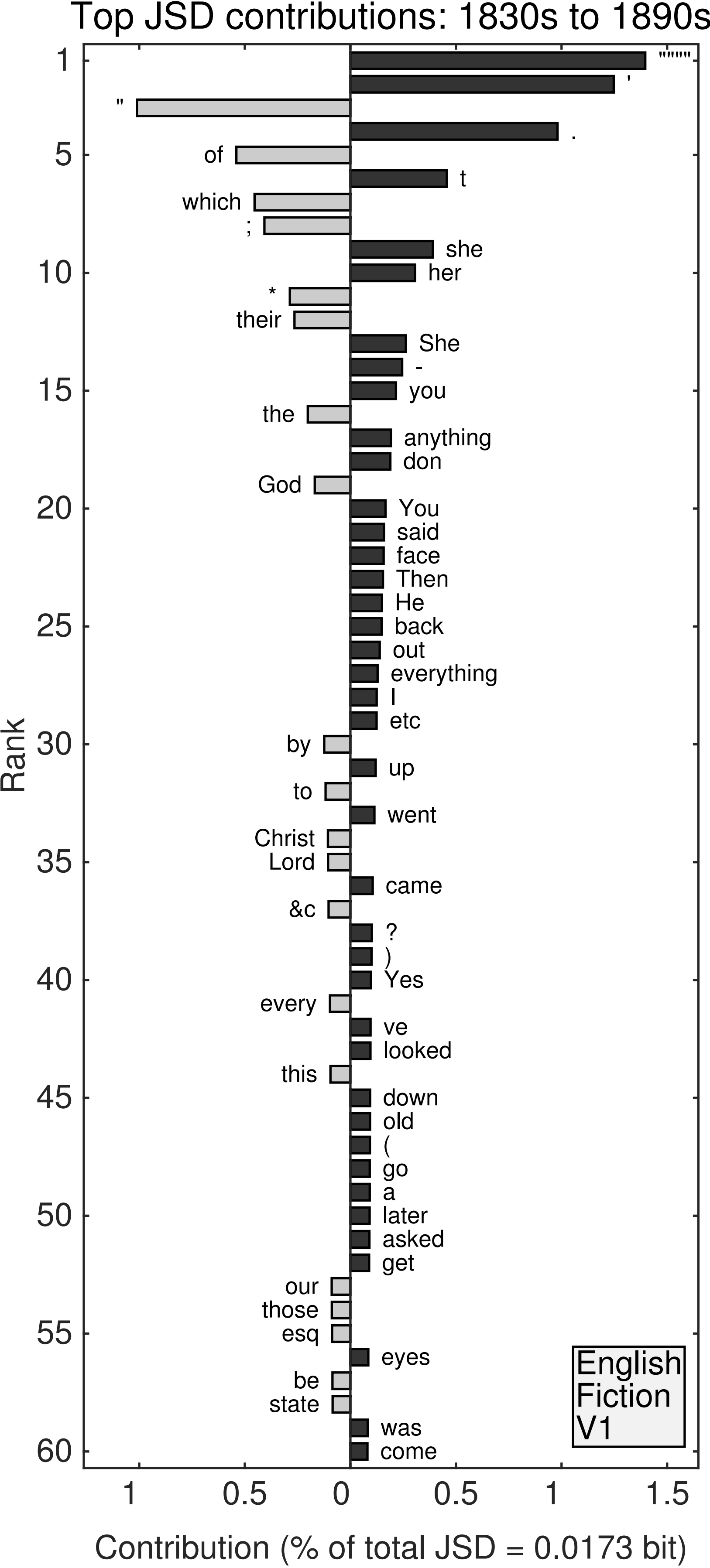

Top JSD contributions: 1830s to 1890s
Rank
Contribution (% of total JSD = 0.0173 bit)
English Fiction V1
"""""
'
"
.
of
t
which
;
she
her
*
their
She
-
you
the
anything
don
God
You
said
face
Then
He
back
out
everything
I
etc
by
up
to
went
Christ
Lord
came
&c
?
)
Yes
every
ve
looked
this
down
old
(
go
a
later
asked
get
our
those
esq
eyes
be
state
was
come
1
5
10
15
20
25
30
35
40
45
50
55
60
1
0.5
0
0.5
1
1.5

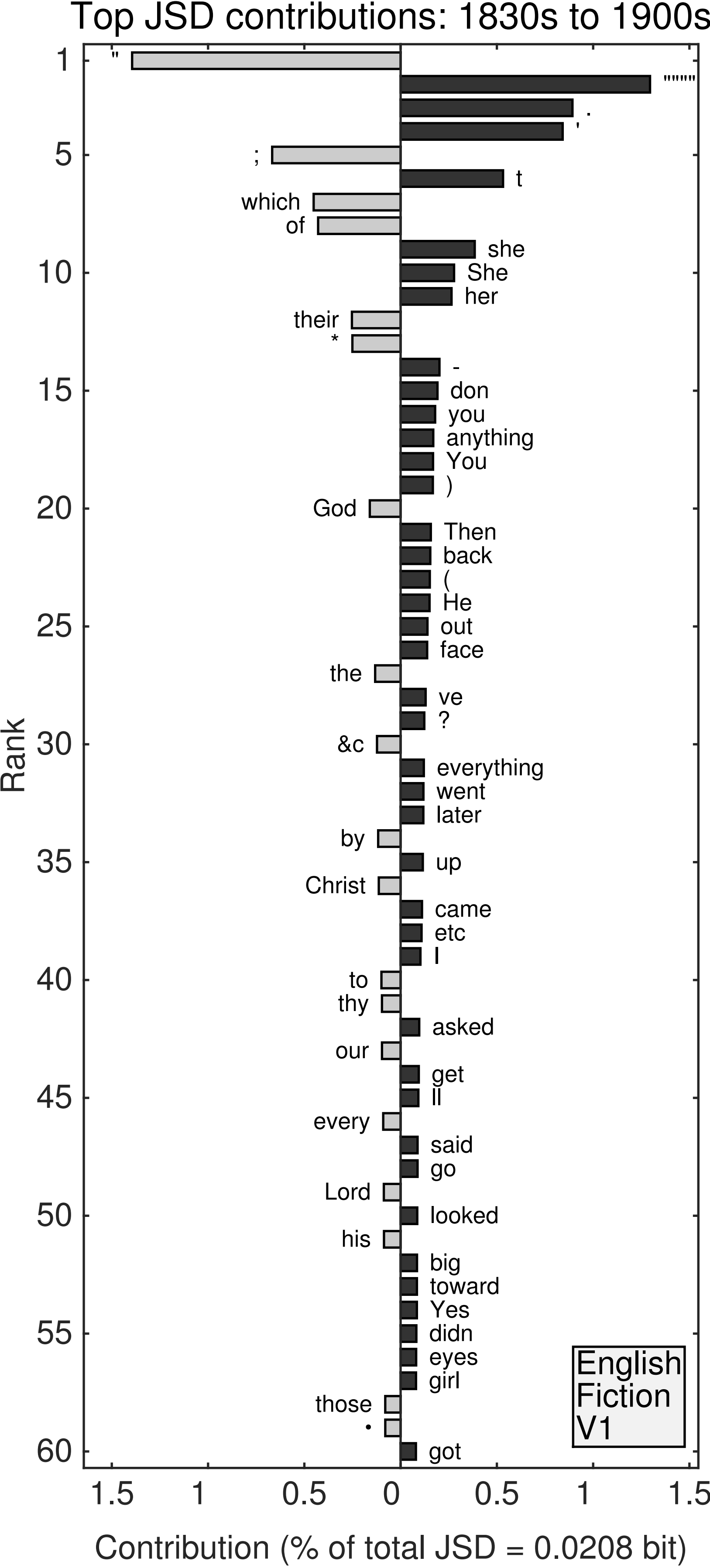

Top JSD contributions: 1830s to 1900s
Rank
Contribution (% of total JSD = 0.0208 bit)
English Fiction V1
1
5
10
15
20
25
30
35
40
45
50
55
60
1.5
1
0.5
0
0.5
1
1.5
"
""""
.
'
;
t
which
of
she
She
her
their
*
-
don
you
anything
You
)
God
Then
back
(
He
out
face
the
ve
?
&c
everything
went
later
by
up
Christ
came
etc
I
to
thy
asked
our
get
ll
every
said
go
Lord
looked
his
big
toward
Yes
didn
eyes
girl
those
•
got

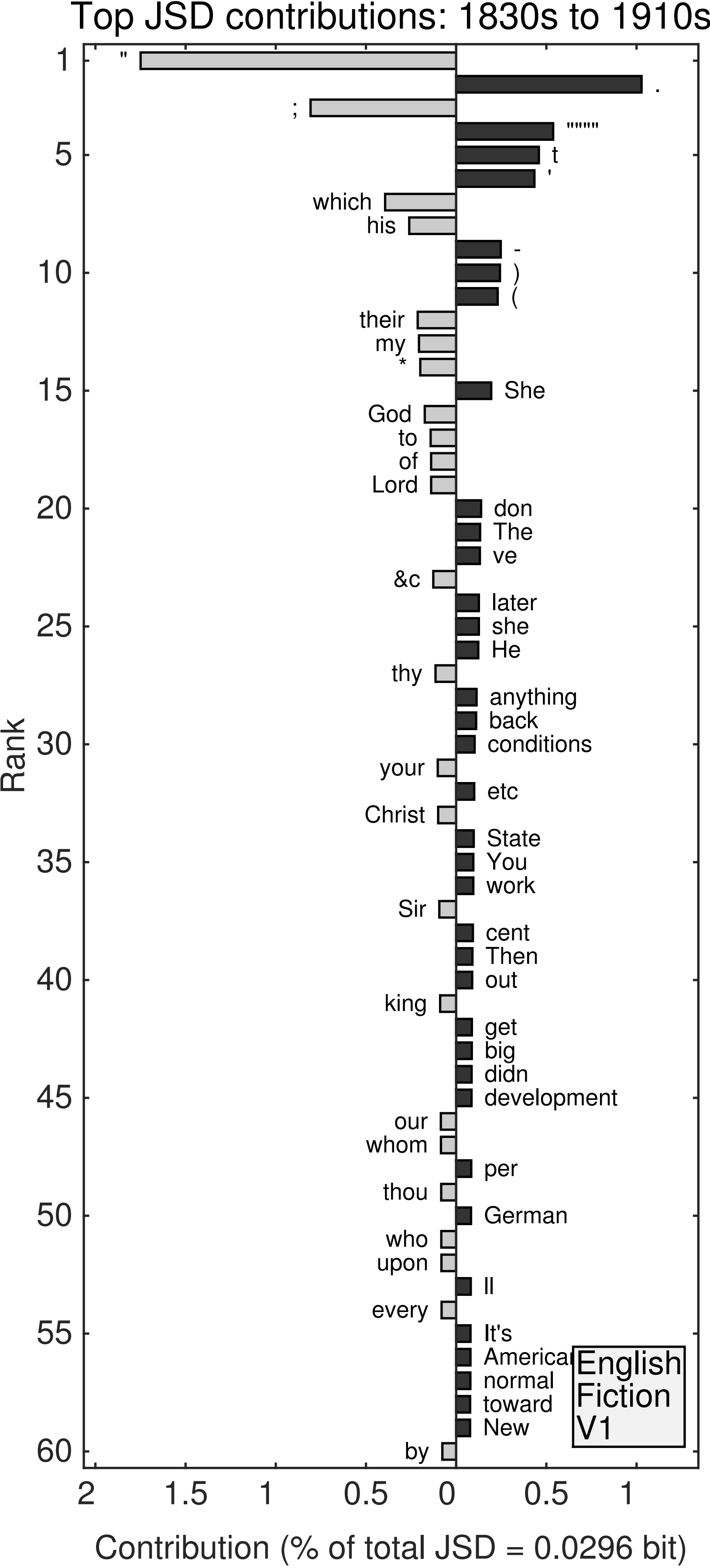
Top JSD contributions: 1830s to 1910s
Rank
Contribution (% of total JSD = 0.0296 bit)
English Fiction V1
"
.
;
"""""
t
'
which
his
-
)
(
their
my
*
She
God
to
of
Lord
don
The
ve
&c
later
she
He
thy
anything
back
conditions
your
etc
Christ
State
You
work
Sir
cent
Then
out
king
get
big
didn
development
our
whom
per
thou
German
who
upon
ll
every
It's
American
normal
toward
New
by

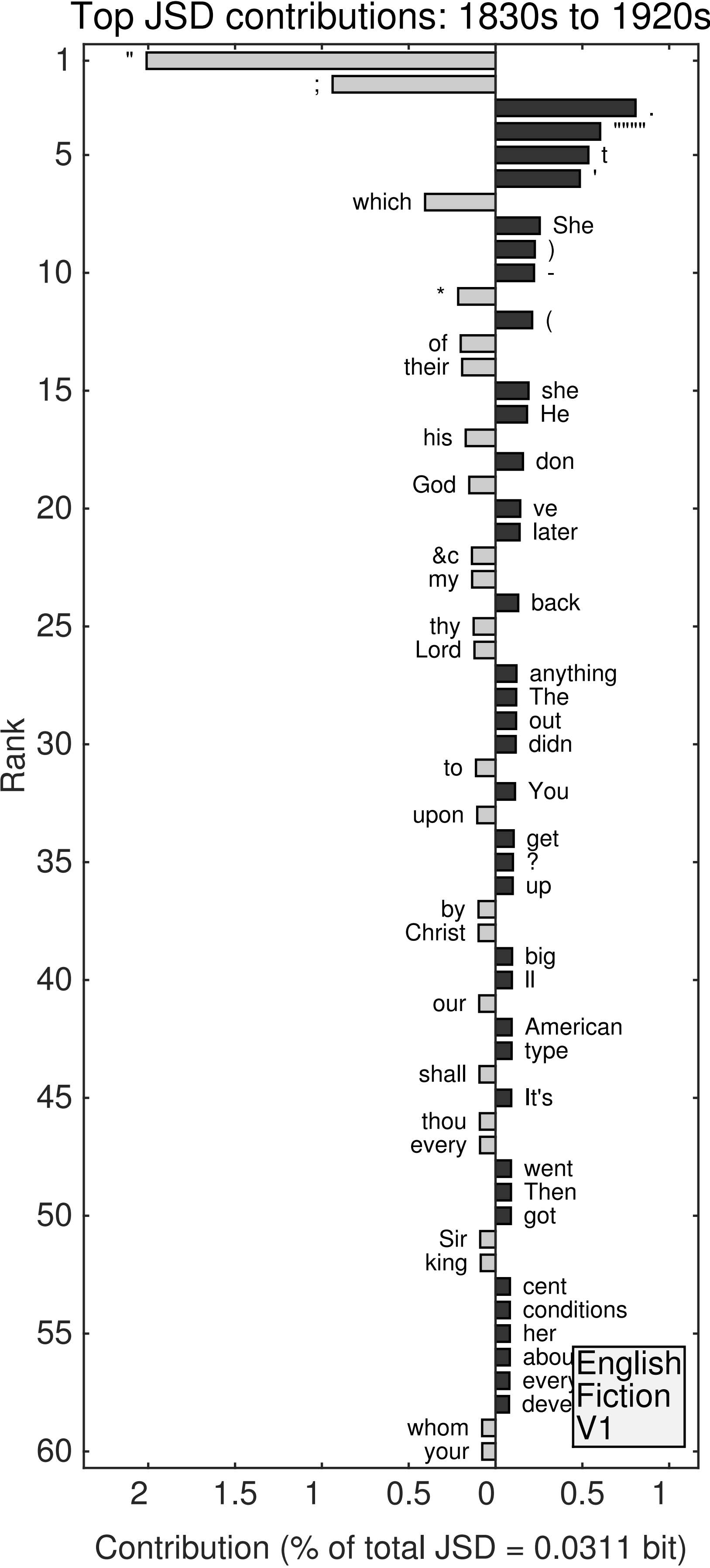

Top JSD contributions: 1830s to 1920s
Rank
"
;
.
""""
t
'
which
She
)
-
*
(
of
their
she
He
his
don
God
ve
later
&c
my
back
thy
Lord
anything
The
out
didn
to
You
upon
get
?
up
by
Christ
big
ll
our
American
type
shall
It's
thou
every
went
Then
got
Sir
king
cent
conditions
her
abou
every
deve
whom
your
English
Fiction
V1
1
5
10
15
20
25
30
35
40
45
50
55
60
2
1.5
1
0.5
0
0.5
1
Contribution (% of total JSD = 0.0311 bit)

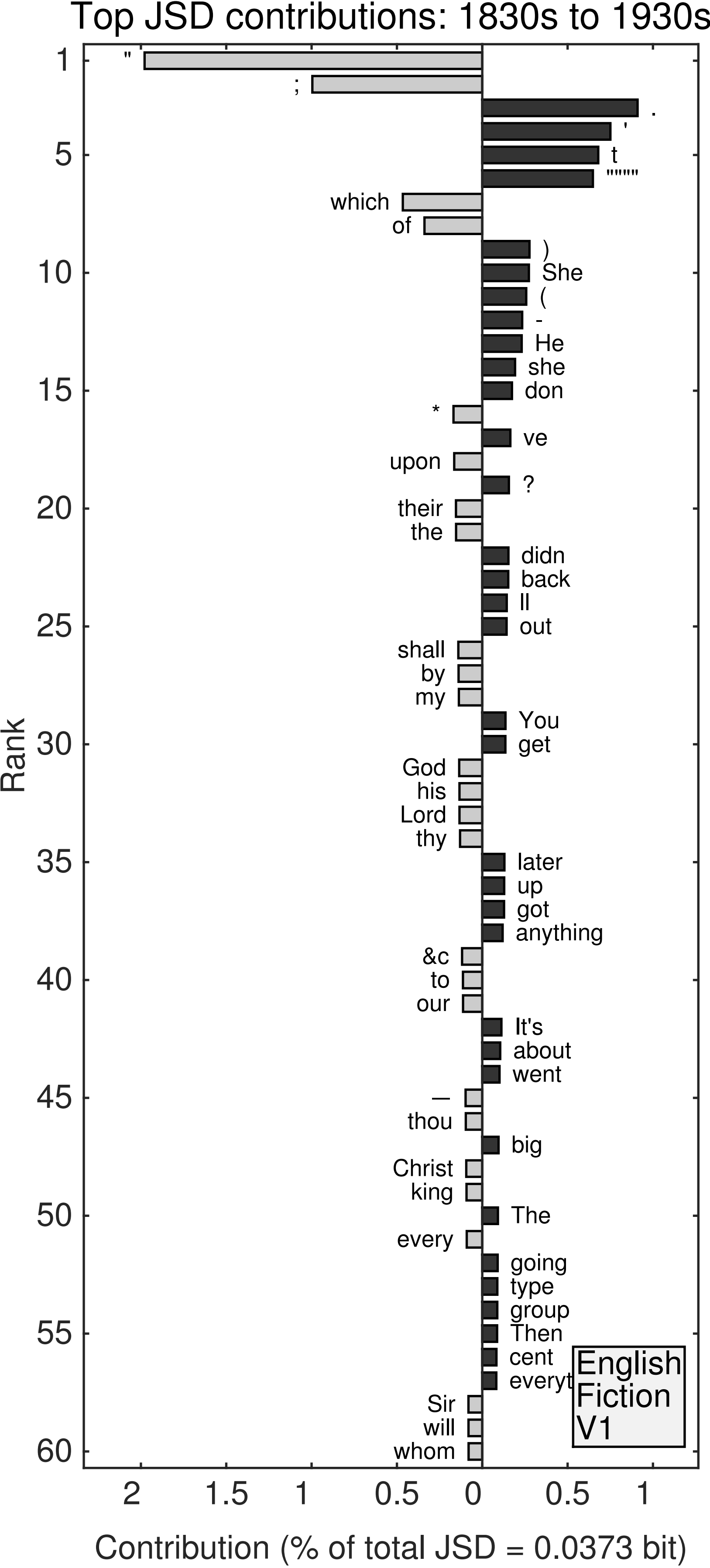

Top JSD contributions: 1830s to 1930s
Rank
Contribution (% of total JSD = 0.0373 bit)
"
;
.
'
t
""""
which
of
)
She
(
-
He
she
don
*
ve
upon
?
their
the
didn
back
ll
out
shall
by
my
You
get
God
his
Lord
thy
later
up
got
anything
&c
to
our
It's
about
went
—
thou
big
Christ
king
The
every
going
type
group
Then
cent
everyt
Sir
will
whom
English
Fiction
V1

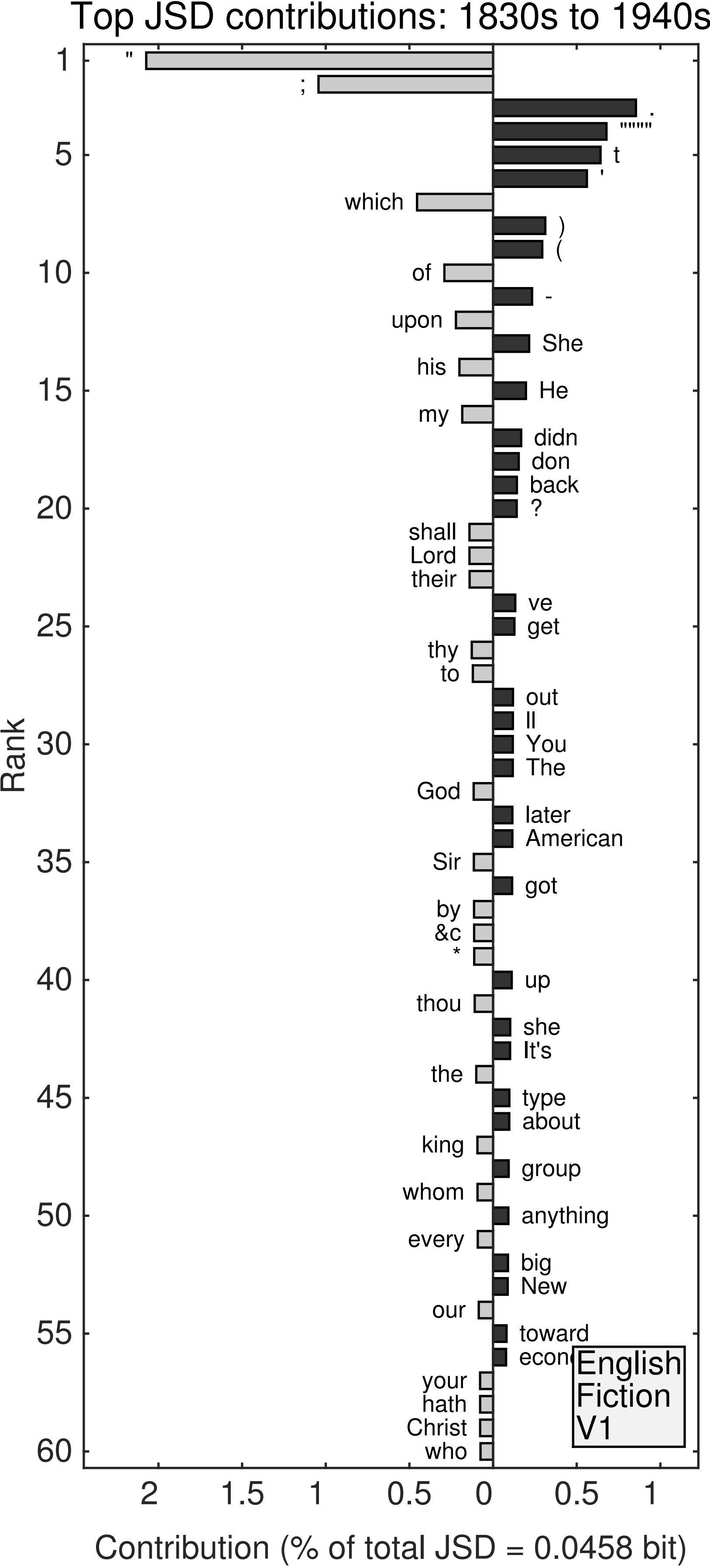
Top JSD contributions: 1830s to 1940s
Rank
Contribution (% of total JSD = 0.0458 bit)
English Fiction V1
"
;
.
""""
t
'
which
)
(
of
-
upon
She
his
He
my
didn
don
back
?
shall
Lord
their
ve
get
thy
to
out
ll
You
The
God
later
American
Sir
got
by
&c
*
up
thou
she
It's
the
type
about
king
group
whom
anything
every
big
New
our
toward
your
hath
Christ
who

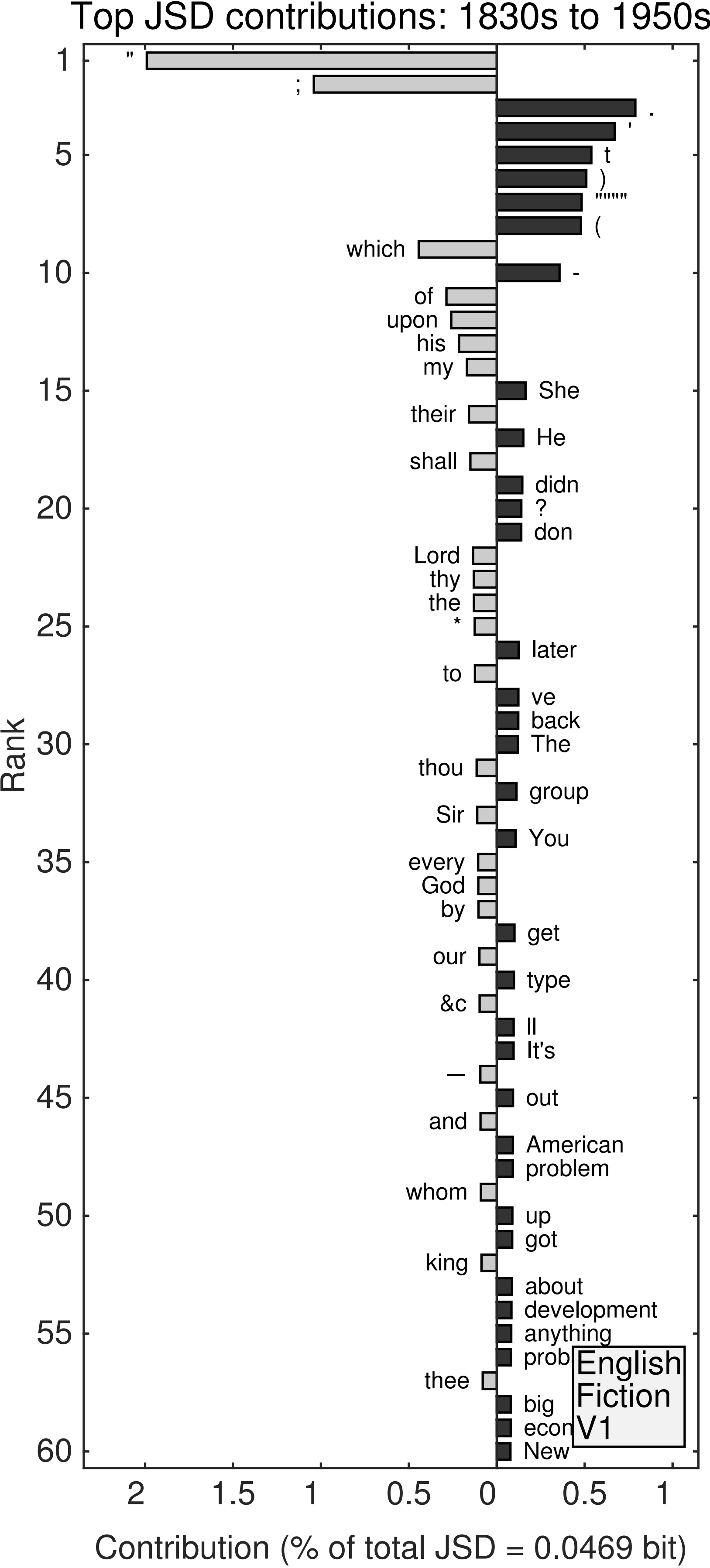

Top JSD contributions: 1830s to 1950s
Rank
Contribution (% of total JSD = 0.0469 bit)
"
;
.
'
t
)
""""
(
which
-
of
upon
his
my
She
their
He
shall
didn
?
don
Lord
thy
the
*
later
to
ve
back
The
thou
group
Sir
You
every
God
by
get
our
type
&c
ll
It's
—
out
and
American
problem
whom
up
got
king
about
development
anything
thee
big
New
English
Fiction
V1

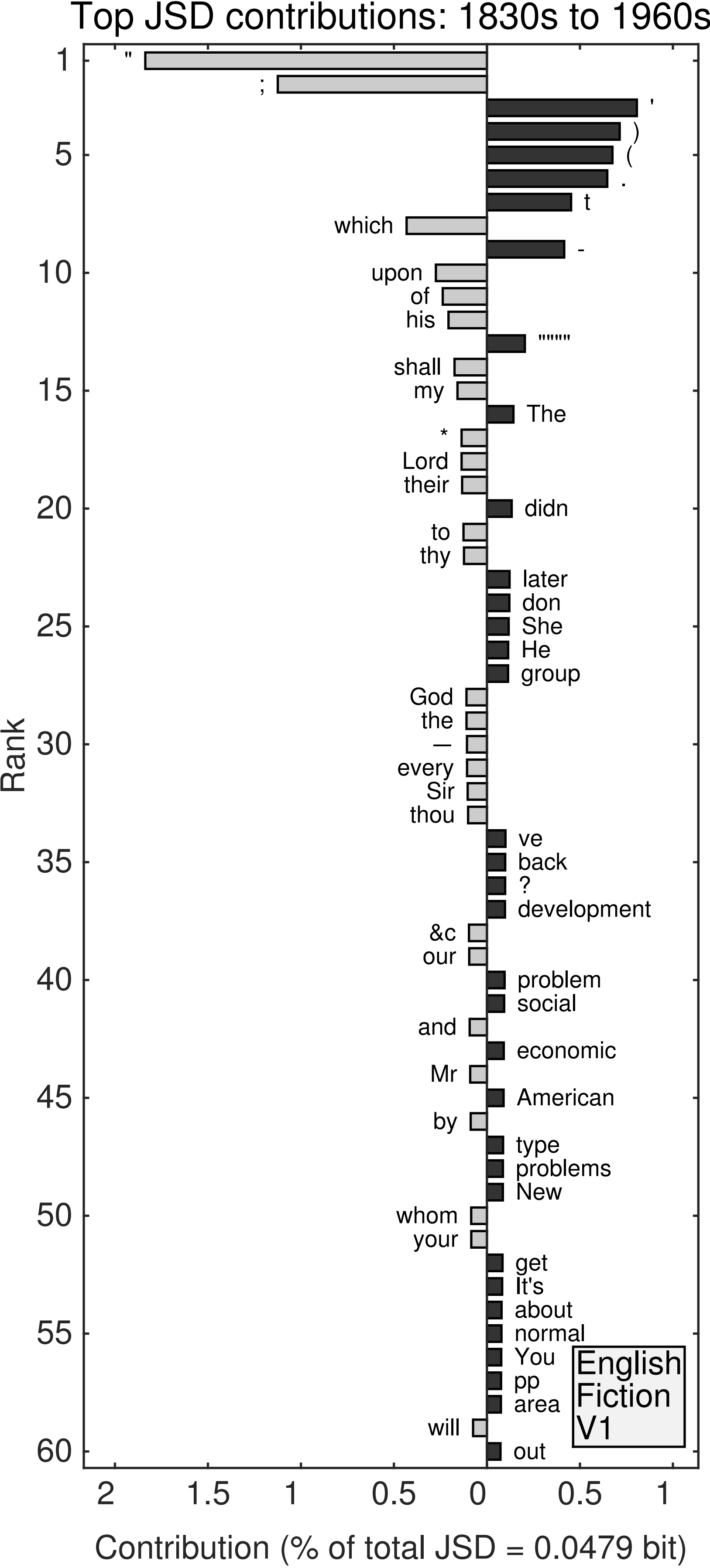

Top JSD contributions: 1830s to 1960s
Rank
Contribution (% of total JSD = 0.0479 bit)
"
;
'
)
(
.
t
which
-
upon
of
his
""""
shall
my
The
*
Lord
their
didn
to
thy
later
don
She
He
group
God
the
—
every
Sir
thou
ve
back
?
development
&c
our
problem
social
and
economic
Mr
American
by
type
problems
New
whom
your
get
It's
about
normal
You
pp
area
will
out
English Fiction V1

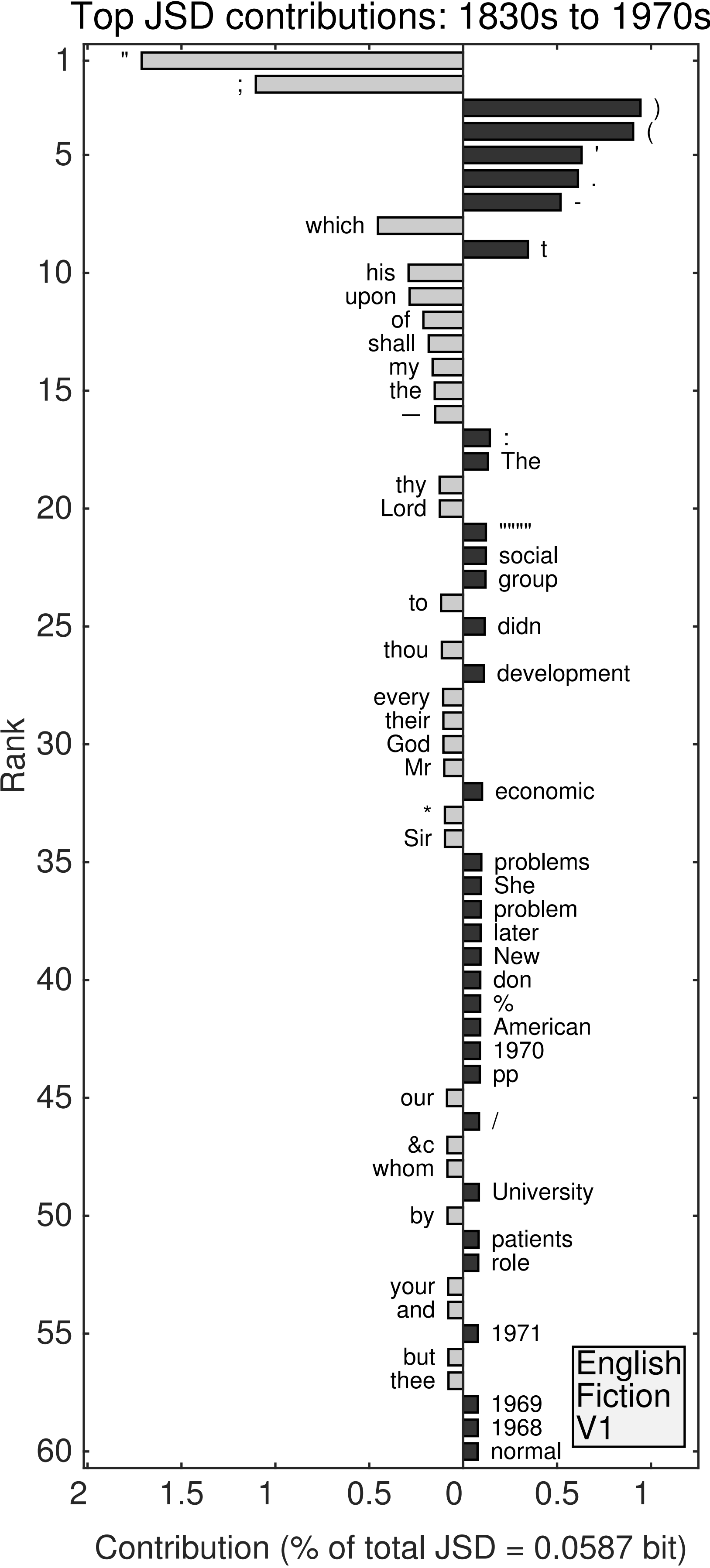

Top JSD contributions: 1830s to 1970s
Rank
Contribution (% of total JSD = 0.0587 bit)
"
;
)
(
'
.
-
which
t
his
upon
of
shall
my
the
—
:
The
thy
Lord
""""
social
group
to
didn
thou
development
every
their
God
Mr
economic
*
Sir
problems
She
problem
later
New
don
%
American
1970
pp
our
/
&c
whom
University
by
patients
role
your
and
1971
but
thee
1969
1968
normal
English Fiction V1

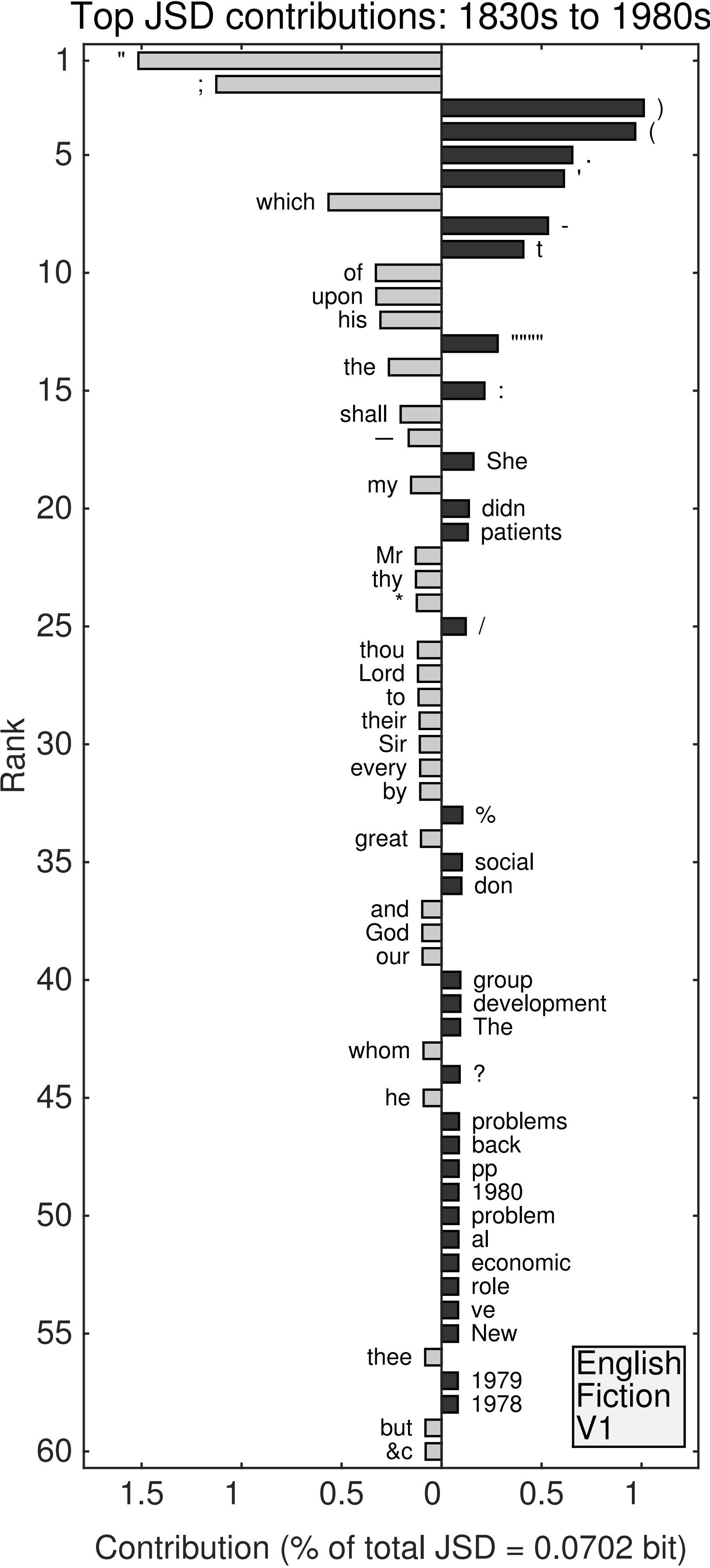
Top JSD contributions: 1830s to 1980s
Rank
Contribution (% of total JSD = 0.0702 bit)
1.5
1
0.5
0
0.5
1
1
5
10
15
20
25
30
35
40
45
50
55
60
"
;
)
(
.
'
which
-
t
of
upon
his
""""
the
:
shall
—
She
my
didn
patients
Mr
thy
*
/
thou
Lord
to
their
Sir
every
by
%
great
social
don
and
God
our
group
development
The
whom
?
he
problems
back
pp
1980
problem
al
economic
role
ve
New
thee
1979
1978
but
&c
English
Fiction
V1

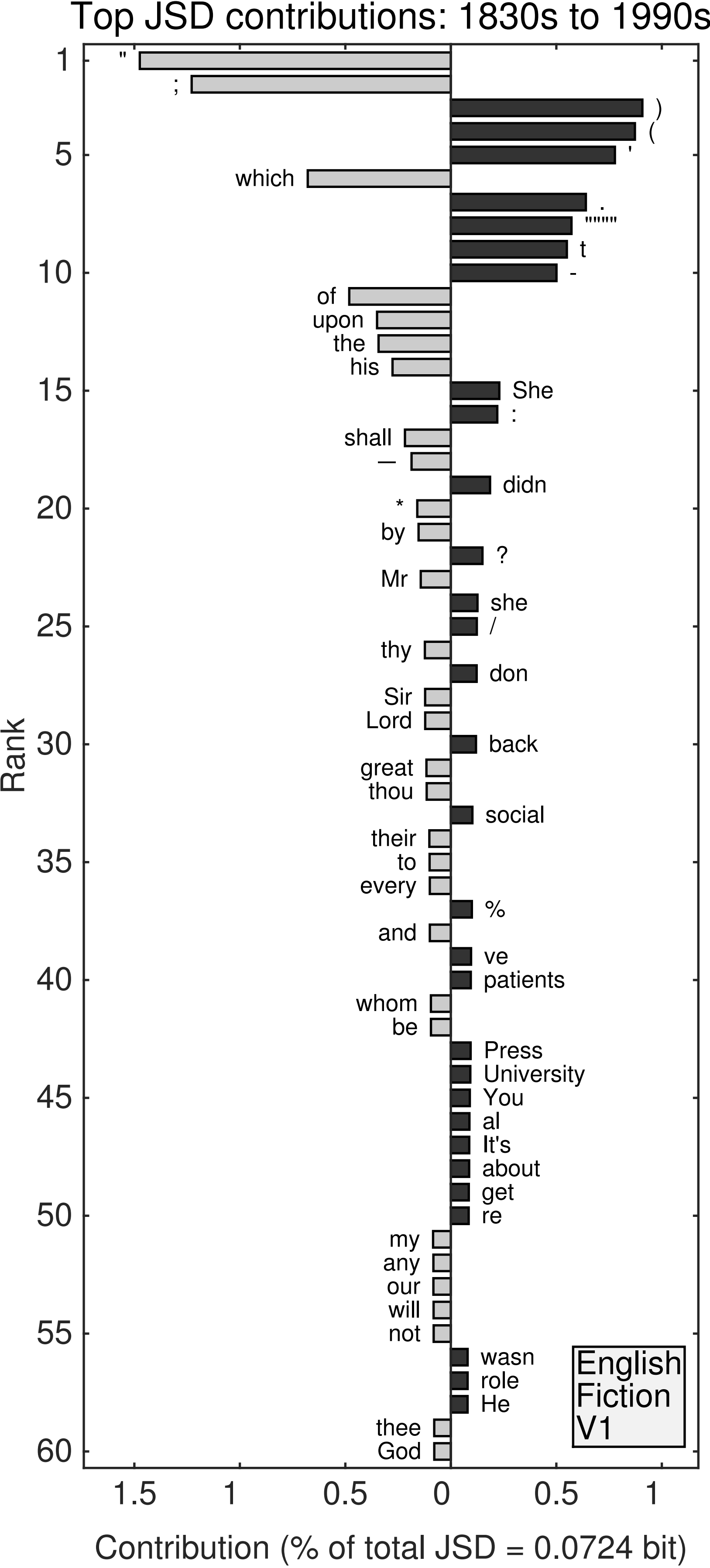

Top JSD contributions: 1830s to 1990s
Rank
" ;) (' which . """" t - of upon the his She : shall — didn * by ? Mr she / thy don Sir Lord back great thou social their to every % and ve patients whom be Press University You al It's about get re my any our will not wasn role He thee God
English Fiction V1
Contribution (% of total JSD = 0.0724 bit)

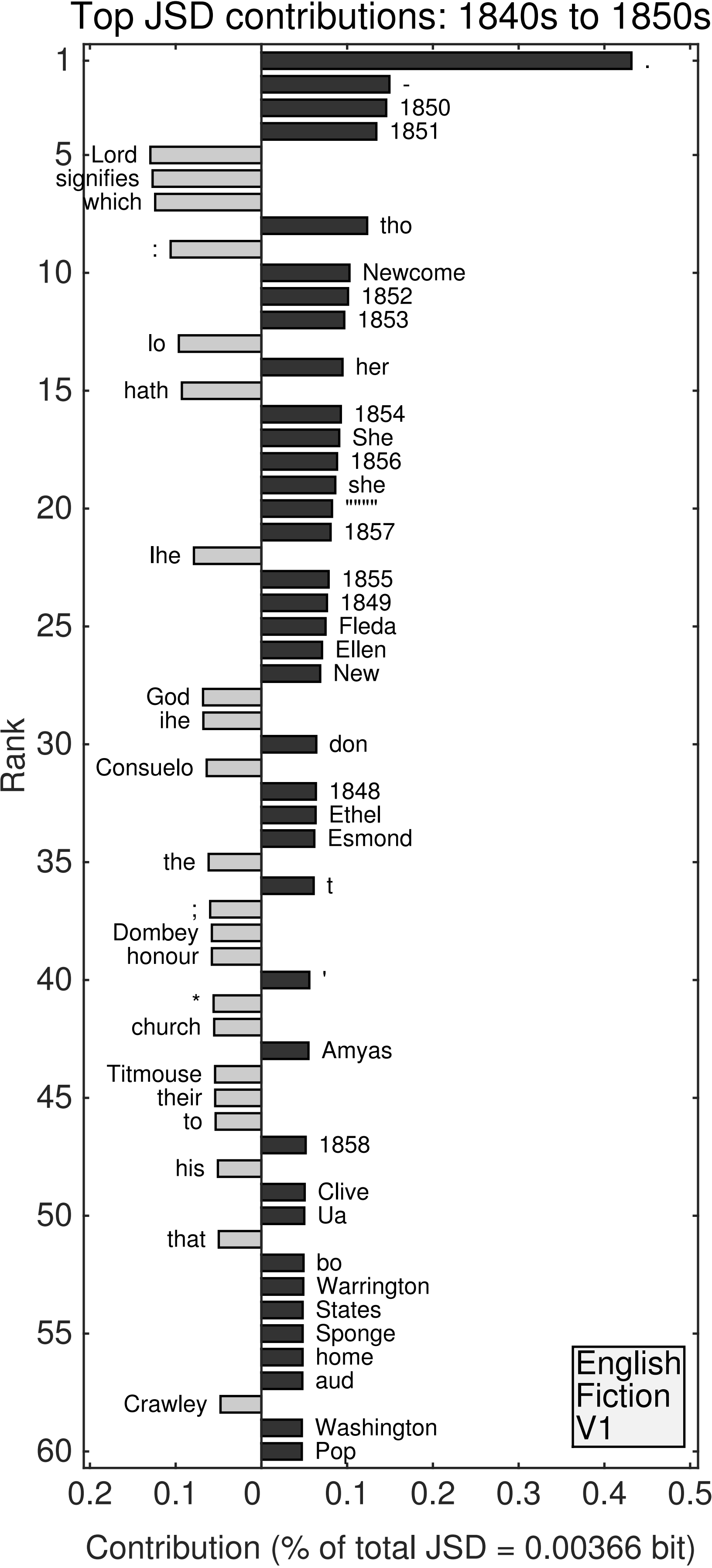
Top JSD contributions: 1840s to 1850s
Rank
Contribution (% of total JSD = 0.00366 bit)
0.2 0.1 0 0.1 0.2 0.3 0.4 0.5
1 5 10 15 20 25 30 35 40 45 50 55 60
.
-
1850
1851
Lord
signifies
which
tho
:
Newcome
1852
1853
lo
her
hath
1854
She
1856
she
""""
1857
Ihe
1855
1849
Fleda
Ellen
New
God
ihe
don
Consuelo
1848
Ethel
Esmond
the
t
;
Dombey
honour
'
*
church
Amyas
Titmouse
their
to
1858
his
Clive
Ua
that
bo
Warrington
States
Sponge
home
aud
Crawley
Washington
Pop
English Fiction V1

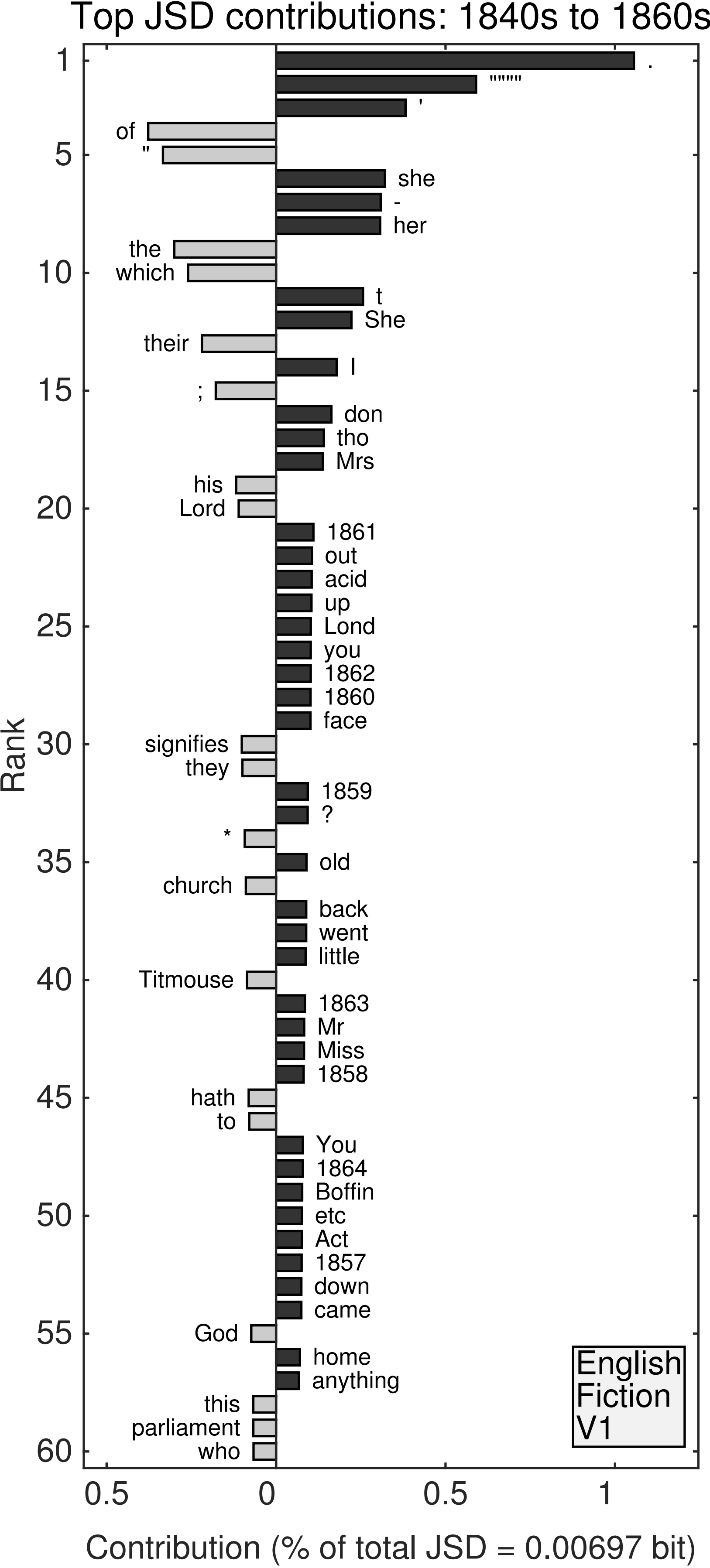

Top JSD contributions: 1840s to 1860s
Rank
Contribution (% of total JSD = 0.00697 bit)
English Fiction V1
.
""""
'
of
"
she
-
her
the
which
t
She
their
I
;
don
tho
Mrs
his
Lord
1861
out
acid
up
Lond
you
1862
1860
face
signifies
they
1859
?
*
old
church
back
went
little
Titmouse
1863
Mr
Miss
1858
hath
to
You
1864
Boffin
etc
Act
1857
down
came
God
home
anything
this
parliament
who

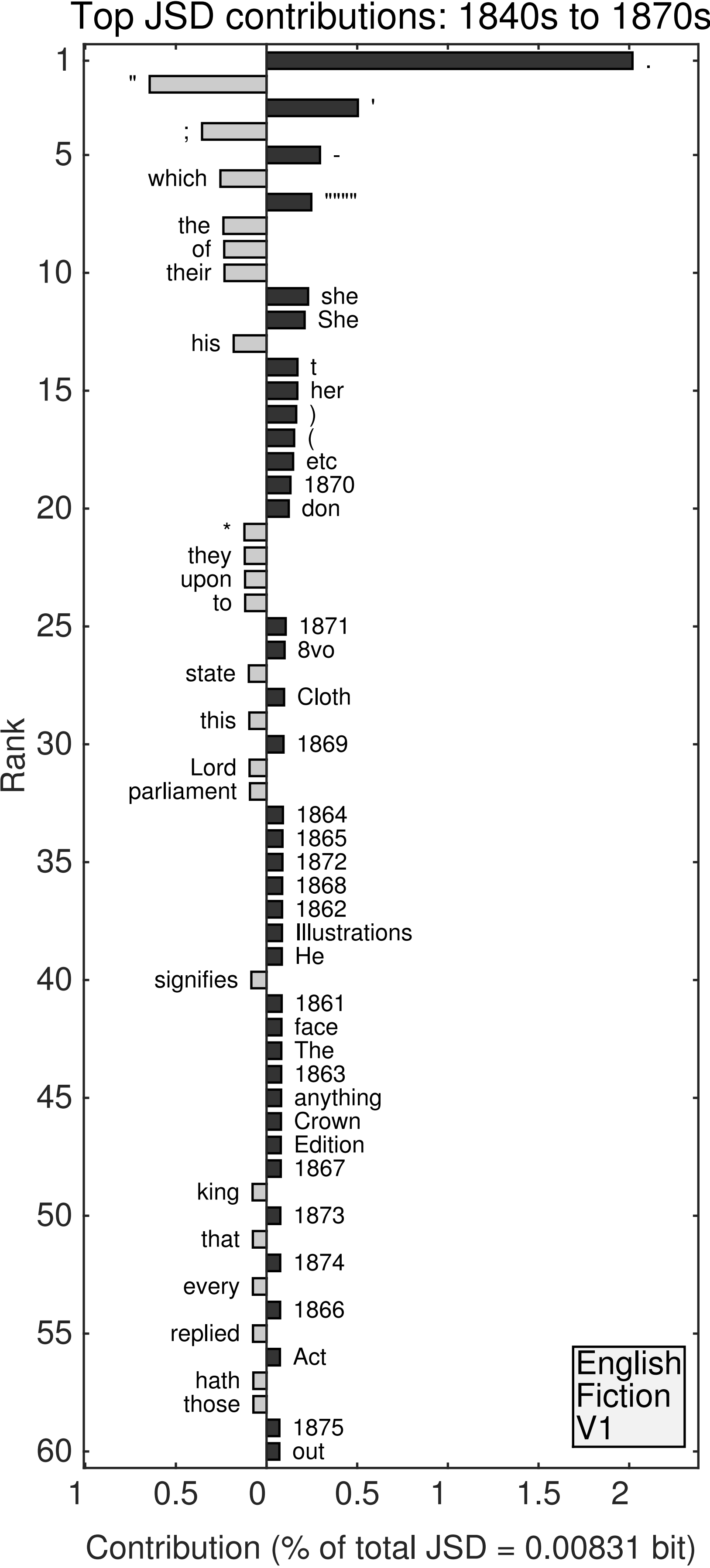

Top JSD contributions: 1840s to 1870s
Rank
1
5
10
15
20
25
30
35
40
45
50
55
60
.
"
'
;
-
which
""""
the
of
their
she
She
his
t
her
)
(
etc
1870
don
*
they
upon
to
1871
8vo
state
Cloth
this
1869
Lord
parliament
1864
1865
1872
1868
1862
Illustrations
He
signifies
1861
face
The
1863
anything
Crown
Edition
1867
king
1873
that
1874
every
1866
replied
Act
hath
those
1875
out
English
Fiction
V1
1
0.5
0
0.5
1
1.5
2
Contribution (% of total JSD = 0.00831 bit)

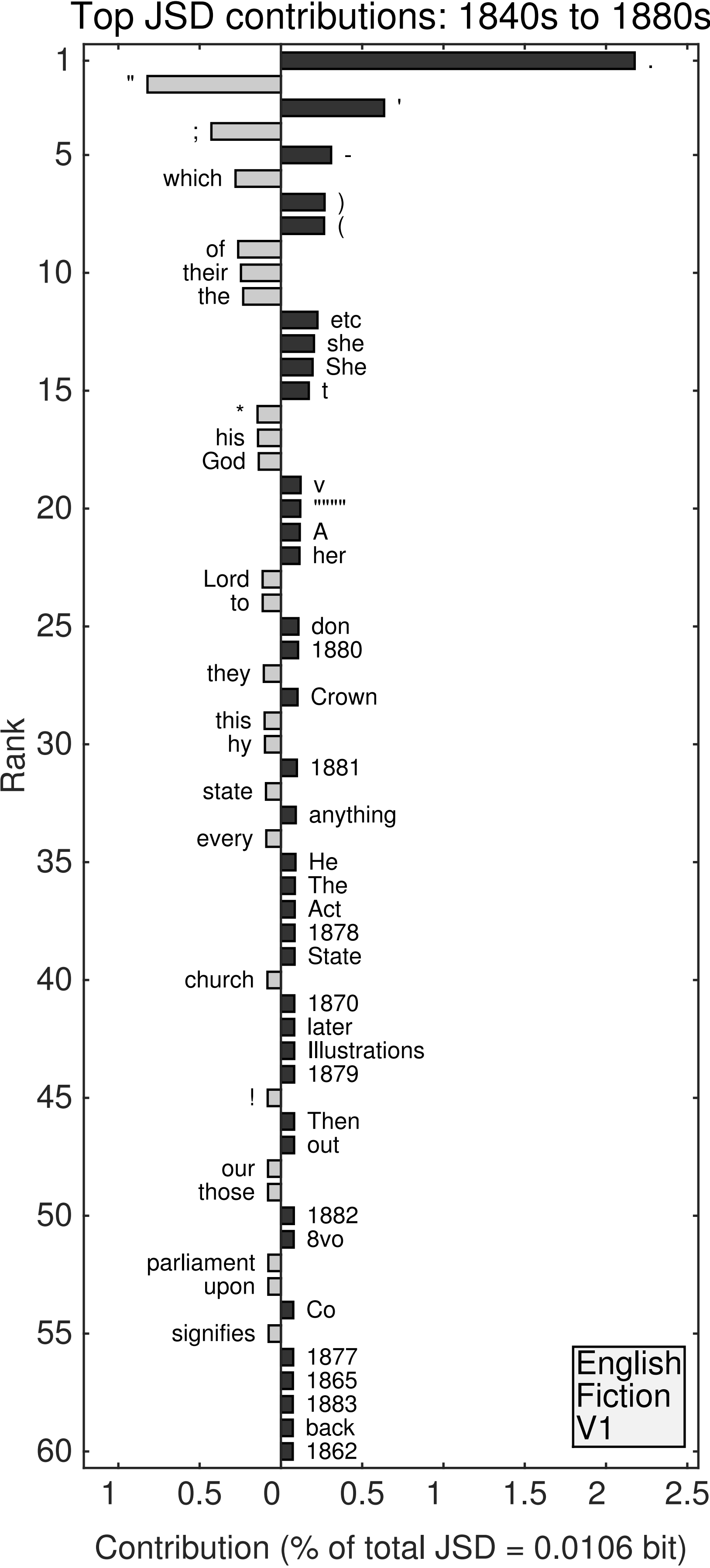
Top JSD contributions: 1840s to 1880s
Rank
1
5
10
15
20
25
30
35
40
45
50
55
60
.
"
'
;
-
which
)
(
of
their
the
etc
she
She
t
*
his
God
v
""""
A
her
Lord
to
don
1880
they
Crown
this
hy
1881
state
anything
every
He
The
Act
1878
State
church
1870
later
Illustrations
1879
!
Then
out
our
those
1882
8vo
parliament
upon
Co
signifies
1877
1865
1883
back
1862
English
Fiction
V1
1
0.5
0
0.5
1
1.5
2
2.5
Contribution (% of total JSD = 0.0106 bit)

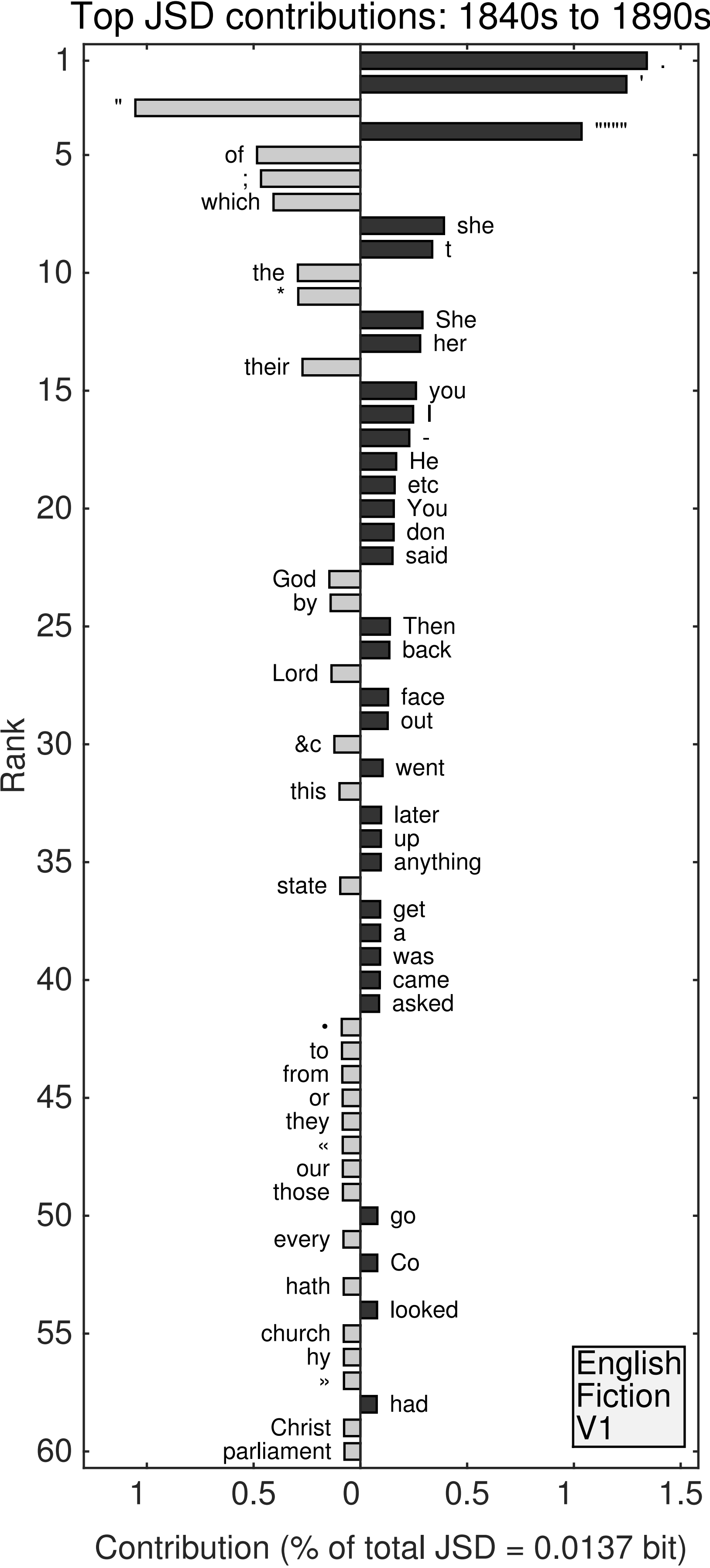

Top JSD contributions: 1840s to 1890s
Rank
Contribution (% of total JSD = 0.0137 bit)
English Fiction V1
.
'
"
""""
of
;
which
she
t
the
*
She
her
their
you
I
-
He
etc
You
don
said
God
by
Then
back
Lord
face
out
&c
went
this
later
up
anything
state
get
a
was
came
asked
•
to
from
or
they
«
our
those
go
every
Co
hath
looked
church
hy
»
had
Christ
parliament

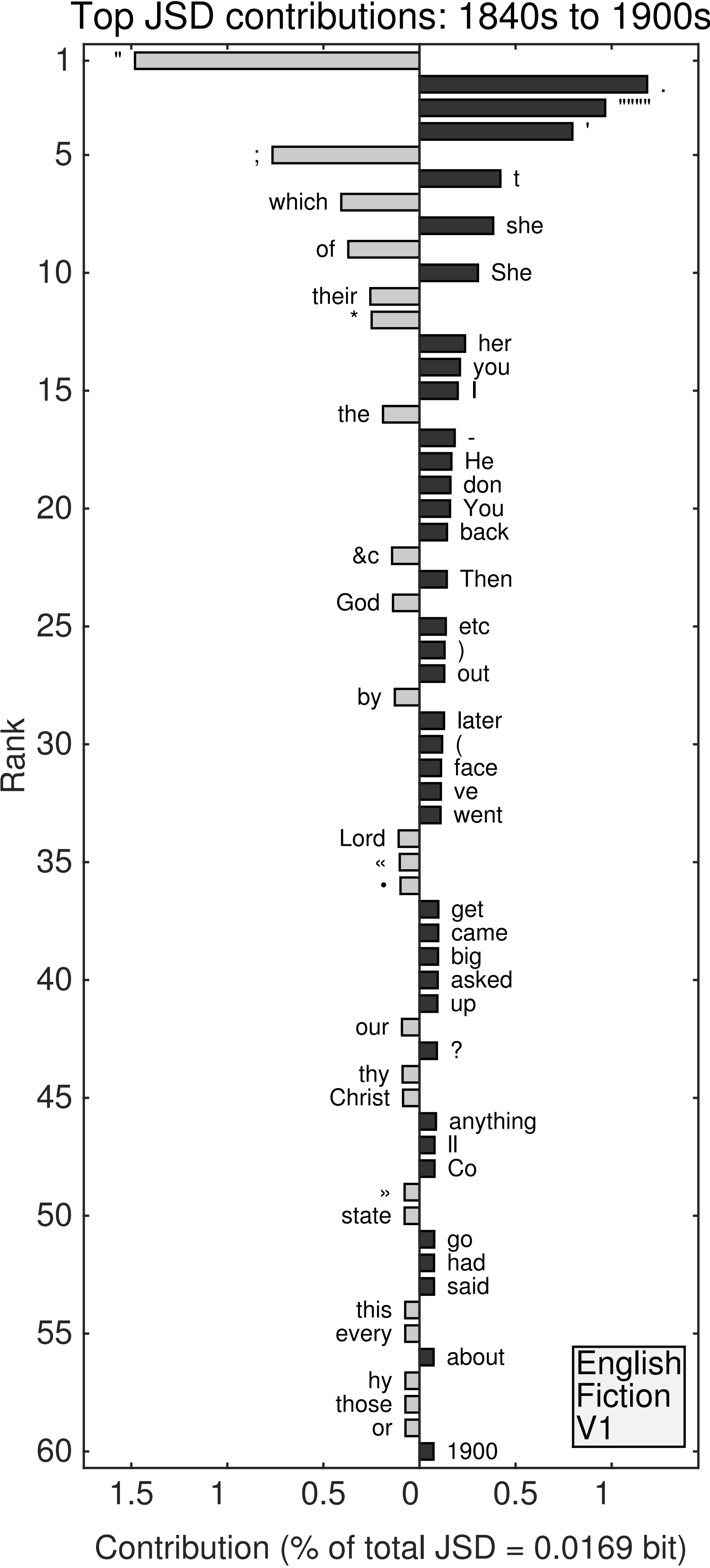

Top JSD contributions: 1840s to 1900s
Rank
Contribution (% of total JSD = 0.0169 bit)
English Fiction V1
"
.
""""
'
;
t
which
she
of
She
their
*
her
you
I
the
-
He
don
You
back
&c
Then
God
etc
)
out
by
later
(
face
ve
went
Lord
«
•
get
came
big
asked
up
our
?
thy
Christ
anything
ll
Co
»
state
go
had
said
this
every
about
hy
those
or
1900

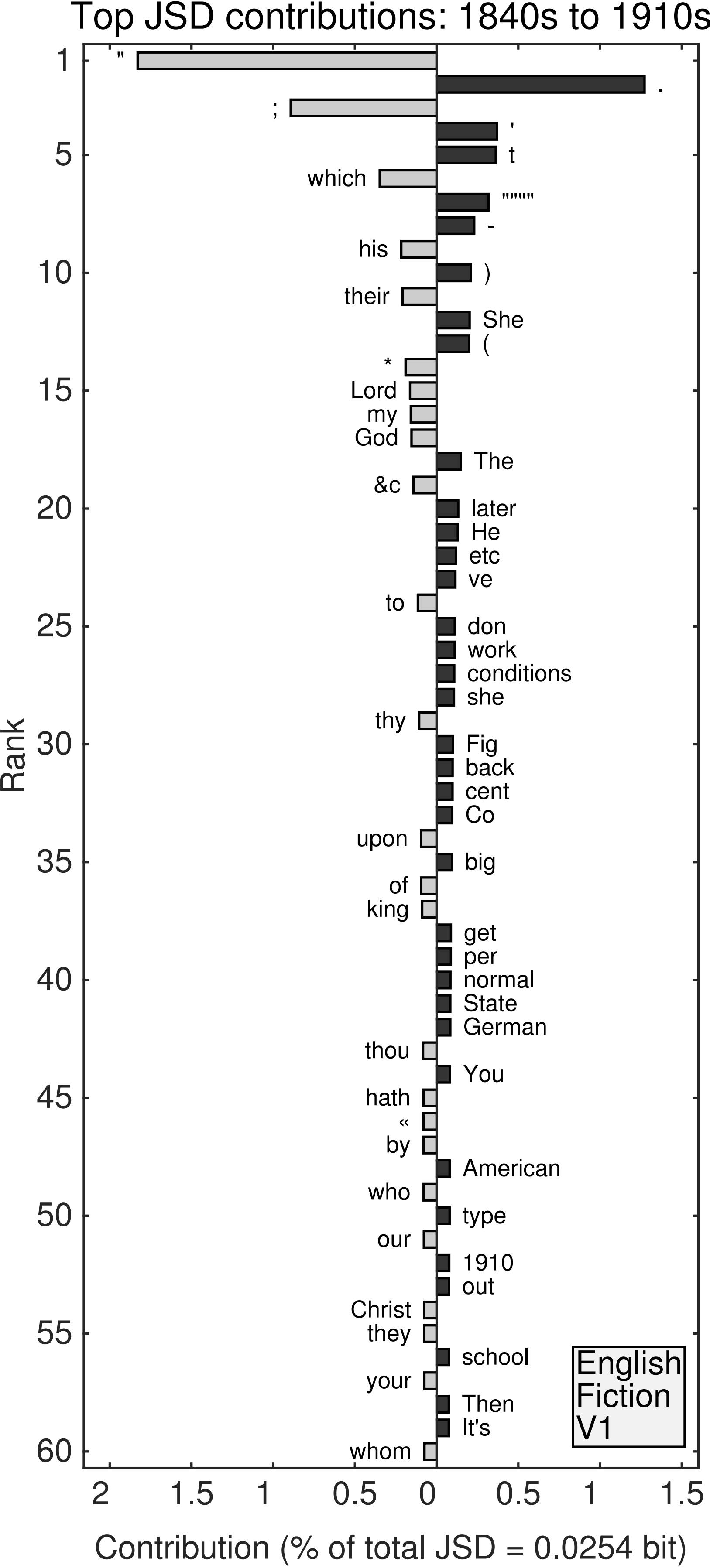

Top JSD contributions: 1840s to 1910s
Rank
Contribution (% of total JSD = 0.0254 bit)
English Fiction V1
"
.
;
'
t
which
""""
-
his
)
their
She
(
*
Lord
my
God
The
&c
later
He
etc
ve
to
don
work
conditions
she
thy
Fig
back
cent
Co
upon
big
of
king
get
per
normal
State
German
thou
You
hath
«
by
American
who
type
our
1910
out
Christ
they
school
your
Then
It's
whom

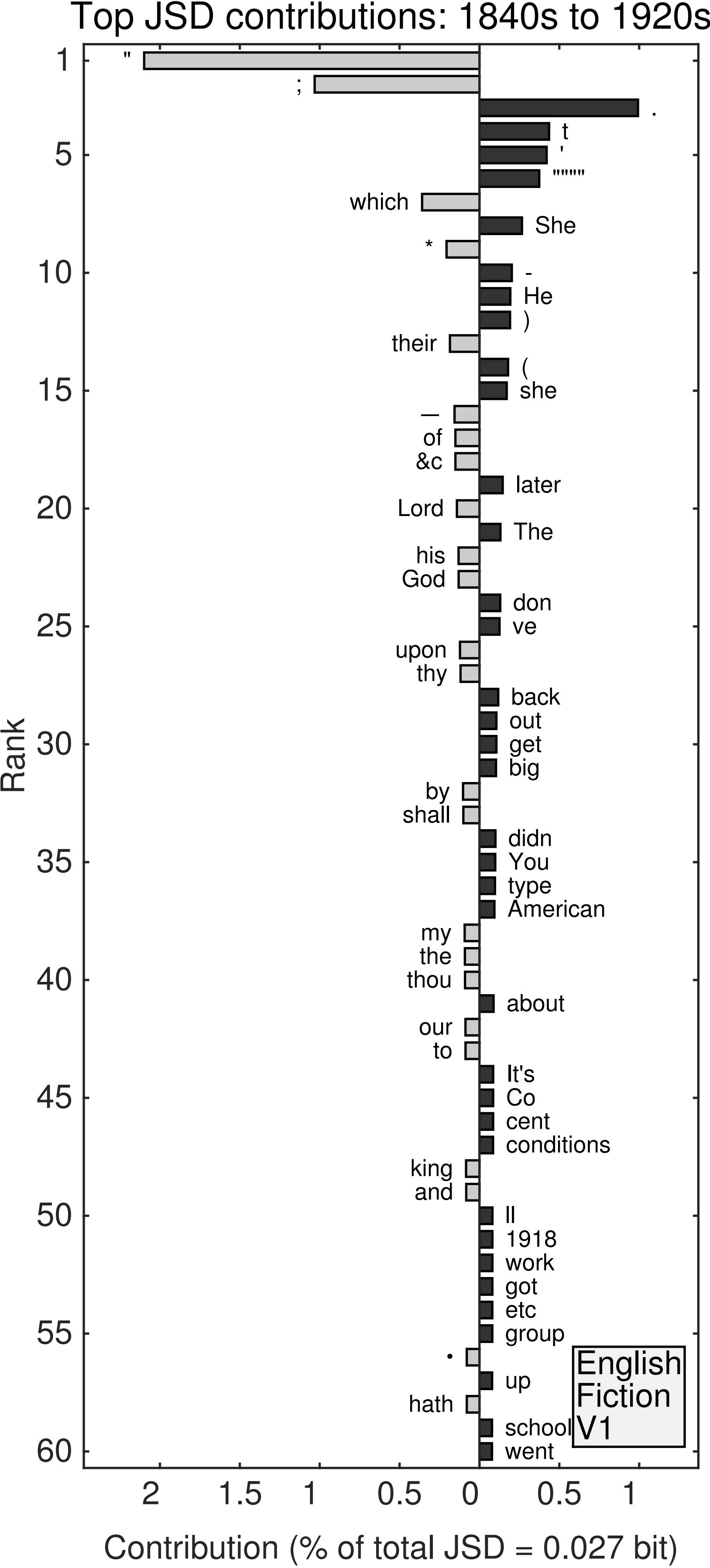

Top JSD contributions: 1840s to 1920s
Rank
Contribution (% of total JSD = 0.027 bit)
2
1.5
1
0.5
0
0.5
1
1
5
10
15
20
25
30
35
40
45
50
55
60
"
;
.
t
'
""""
which
She
*
-
He
)
their
(
she
—
of
&c
later
Lord
The
his
God
don
ve
upon
thy
back
out
get
big
by
shall
didn
You
type
American
my
the
thou
about
our
to
It's
Co
cent
conditions
king
and
ll
1918
work
got
etc
group
•
up
hath
school
went
English
Fiction
V1

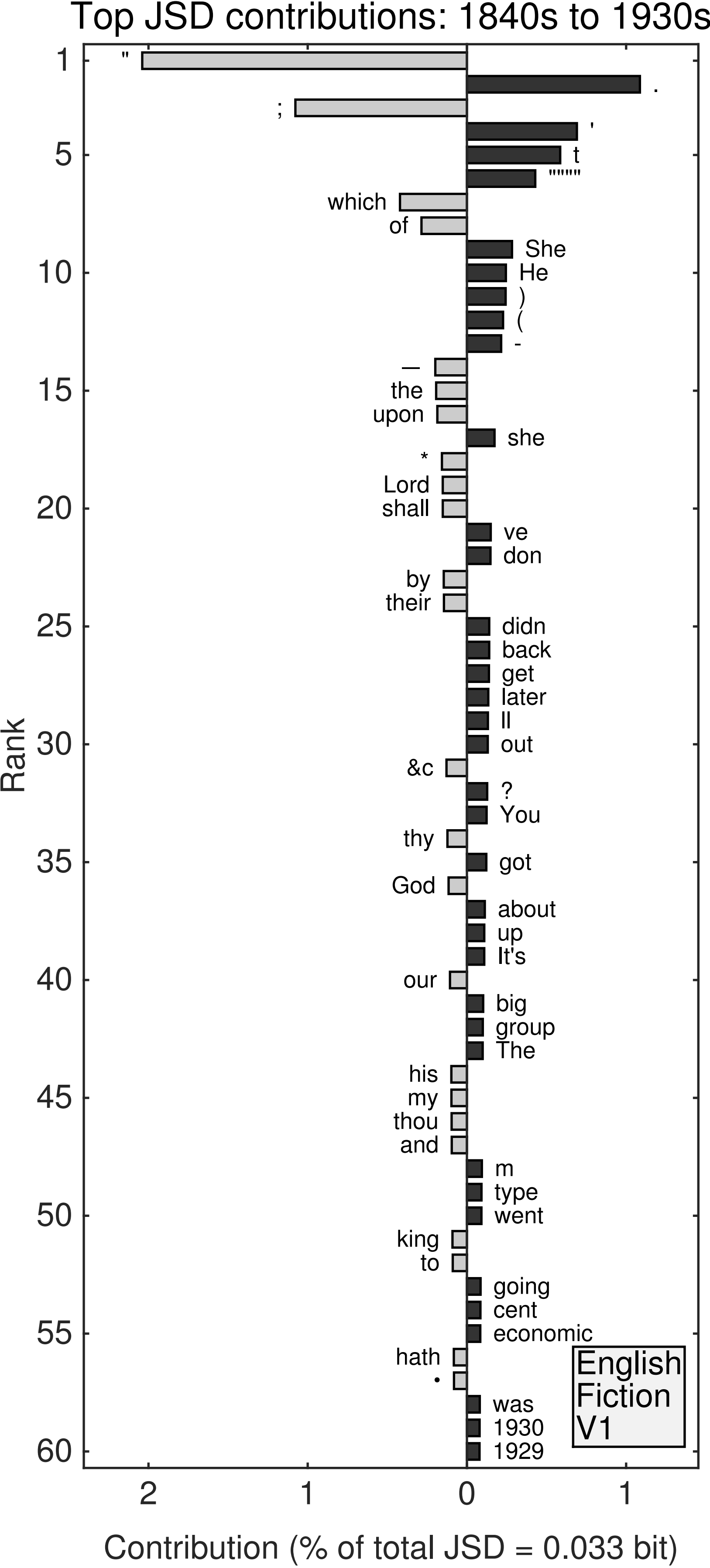
Top JSD contributions: 1840s to 1930s
Rank
Contribution (% of total JSD = 0.033 bit)
"
.
;
'
t
which
of
She
He
)
(
-
—
the
upon
she
*
Lord
shall
ve
don
by
their
didn
back
get
later
ll
out
&c
?
You
thy
got
God
about
up
It's
our
big
group
The
his
my
thou
and
m
type
went
king
to
going
cent
economic
hath
•
was
1930
1929
English Fiction V1

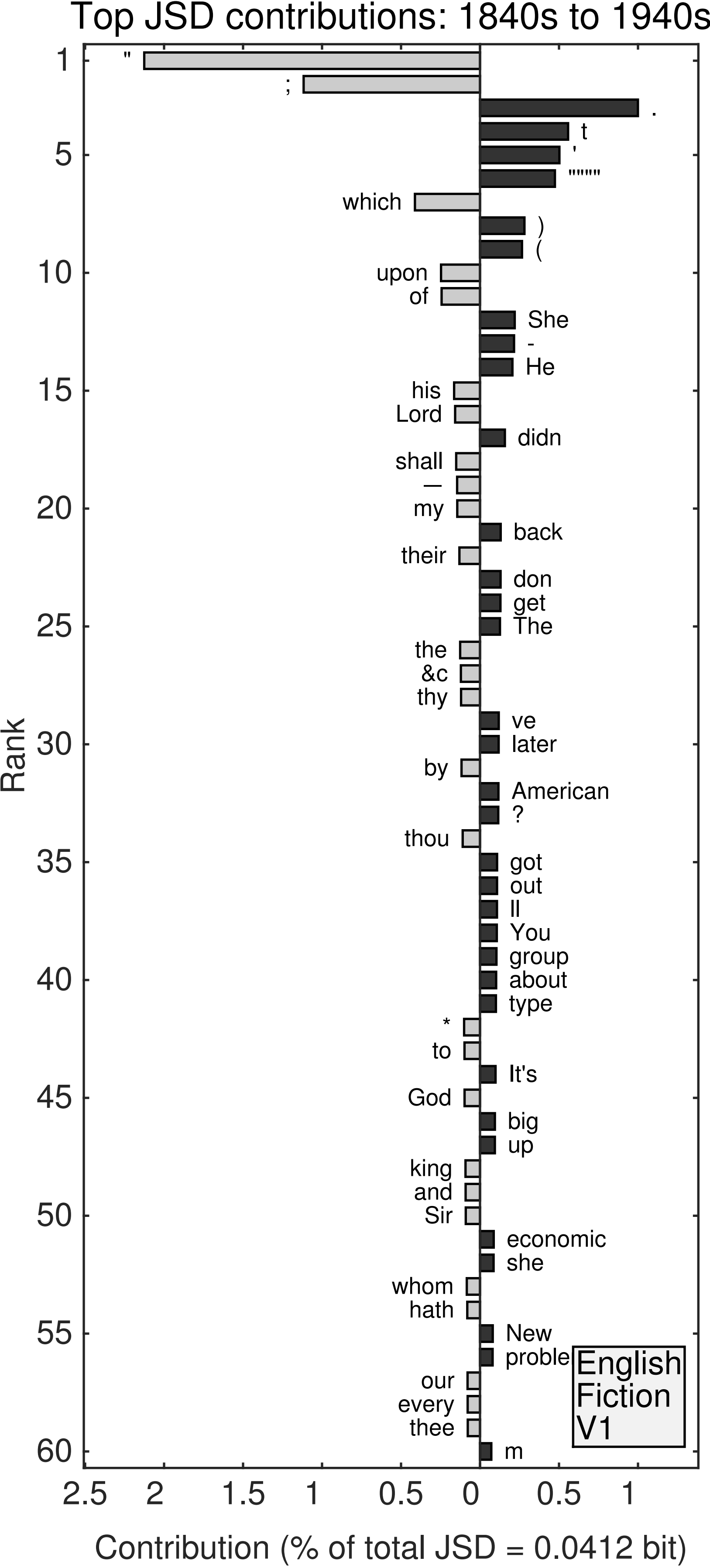

Top JSD contributions: 1840s to 1940s
Rank
Contribution (% of total JSD = 0.0412 bit)
English Fiction V1
"
;
.
t
'
"""""
which
)
(
upon
of
She
-
He
his
Lord
didn
shall
—
my
back
their
don
get
The
the
&c
thy
ve
later
by
American
?
thou
got
out
ll
You
group
about
type
*
to
It's
God
big
up
king
and
Sir
economic
she
whom
hath
New
proble
our
every
thee
m

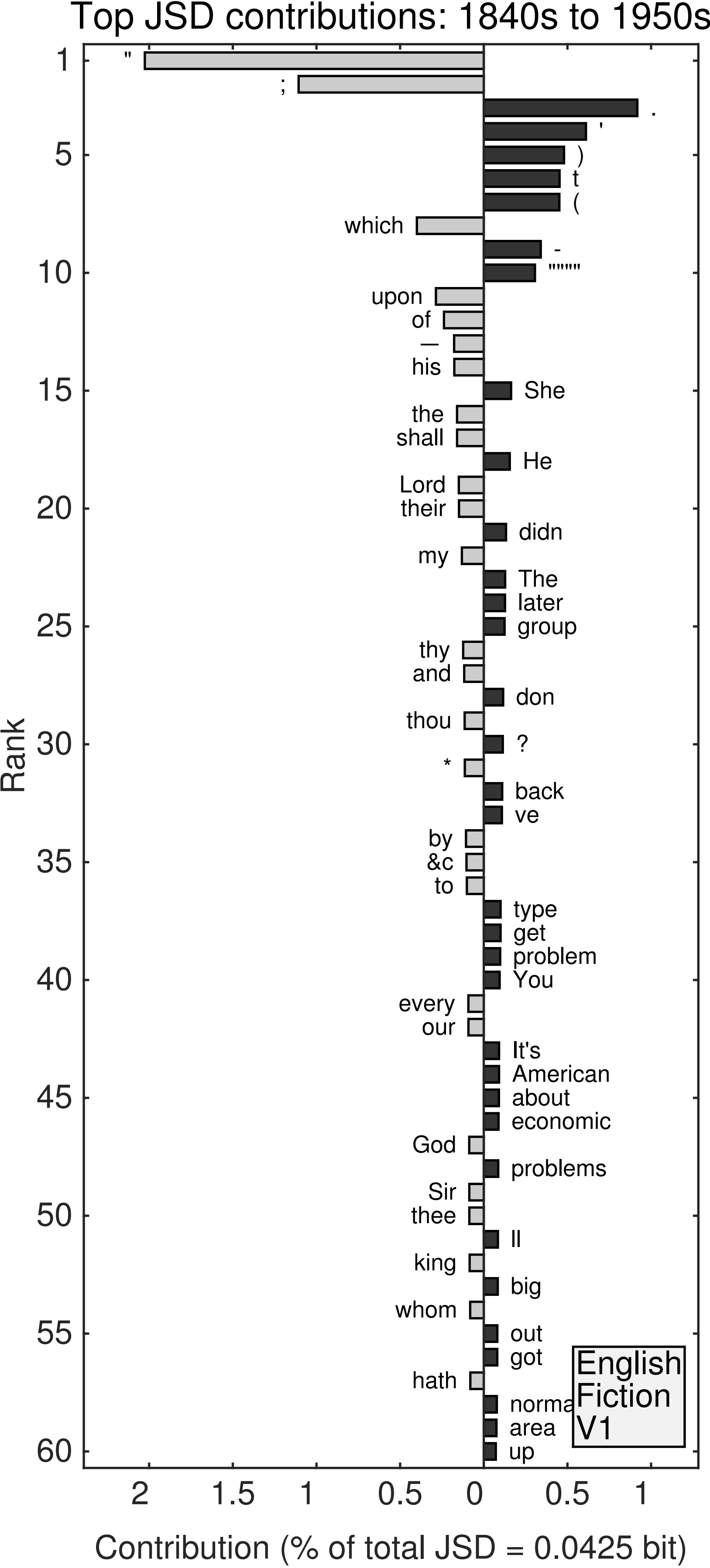

Top JSD contributions: 1840s to 1950s
Rank
Contribution (% of total JSD = 0.0425 bit)
English Fiction V1
1 " ; . ' 5) t (which - 10 """" upon of — his 15 She the shall He Lord 20 their didn my The later 25 group thy and don thou 30 ? * back ve by 35 &c to type get problem 40 You every our It's American 45 about economic God problems Sir 50 thee ll king big whom 55 out got hath norma area 60 up
2 1.5 1 0.5 0 0.5 1

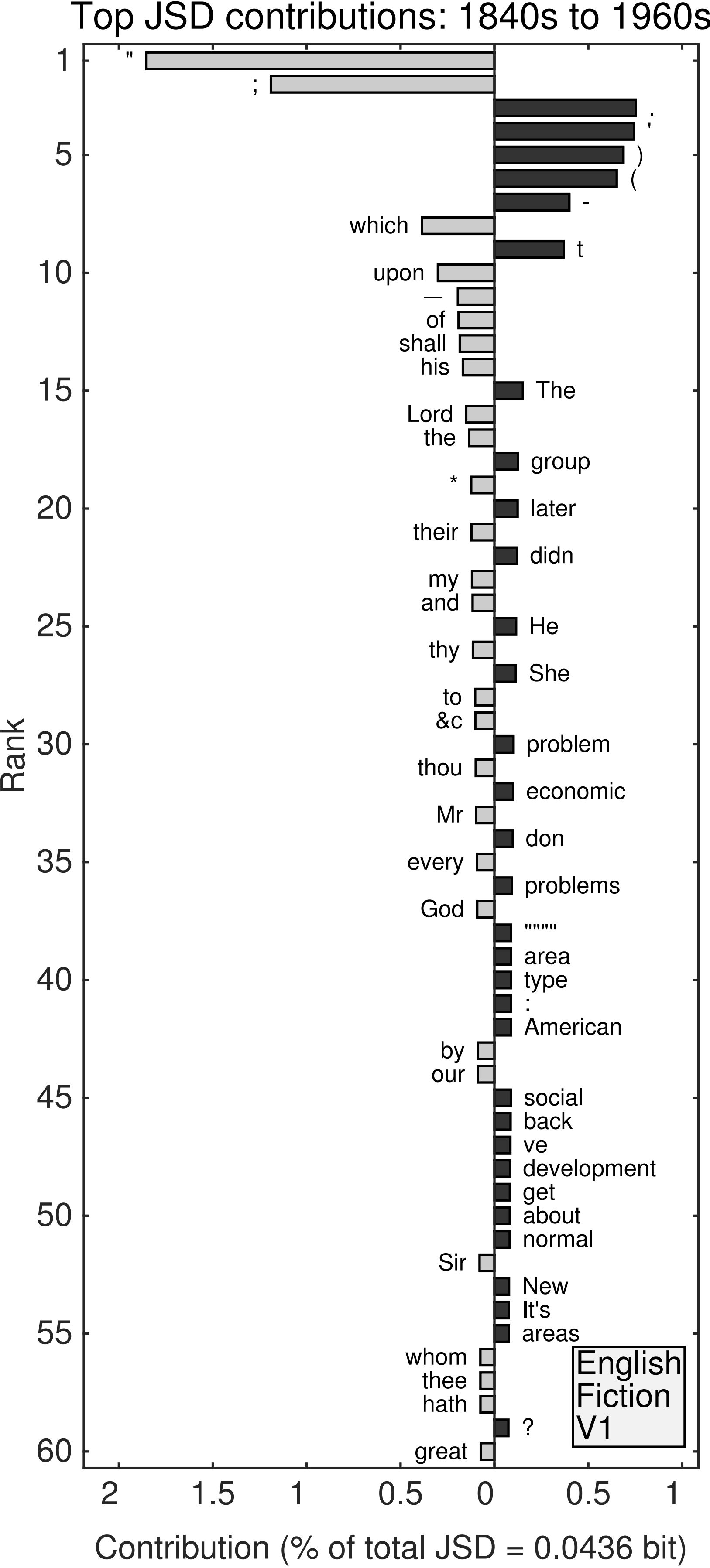
Top JSD contributions: 1840s to 1960s
Rank
Contribution (% of total JSD = 0.0436 bit)
English Fiction V1
"
;
;
'
)
(
-
which
t
upon
—
of
shall
his
The
Lord
the
group
*
later
their
didn
my
and
He
thy
She
to
&c
problem
thou
economic
Mr
don
every
problems
God
""""
area
type
:
American
by
our
social
back
ve
development
get
about
normal
Sir
New
It's
areas
whom
thee
hath
?
great

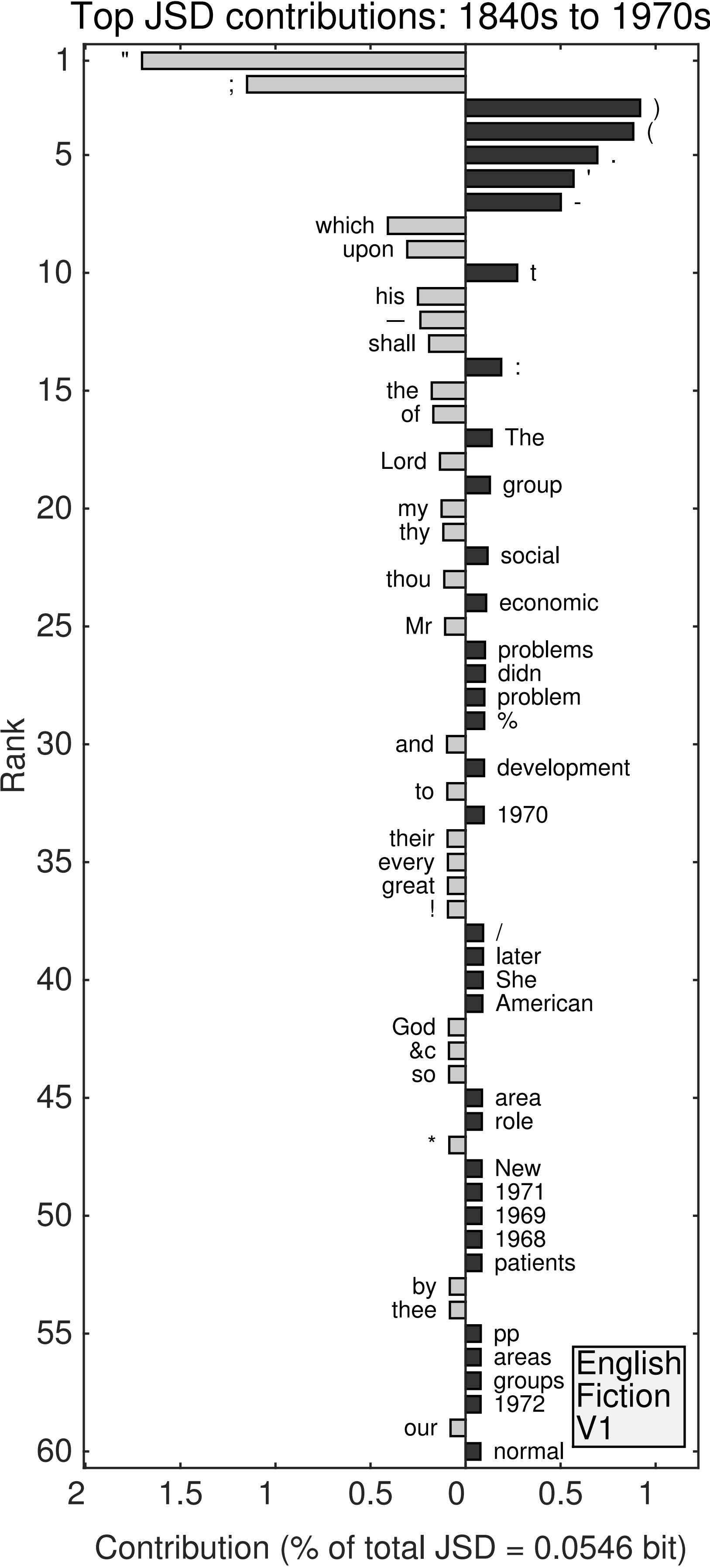
Top JSD contributions: 1840s to 1970s
Rank
Contribution (% of total JSD = 0.0546 bit)
English Fiction V1
"
;
)
(
.
'
-
which
upon
t
his
—
shall
:
the
of
The
Lord
group
my
thy
social
thou
economic
Mr
problems
didn
problem
%
and
development
to
1970
their
every
great
!
/
later
She
American
God
&c
so
area
role
*
New
1971
1969
1968
patients
by
thee
pp
areas
groups
1972
our
normal

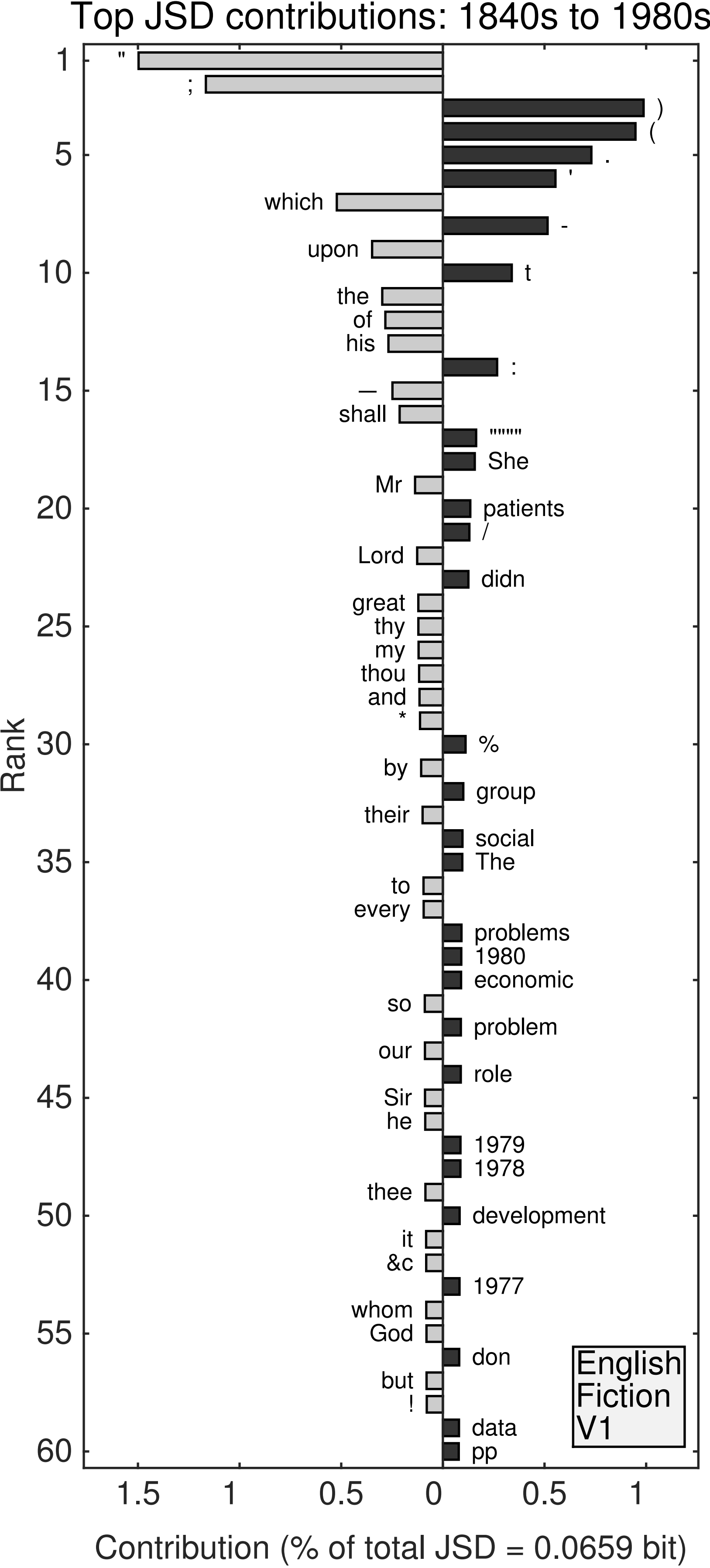

Top JSD contributions: 1840s to 1980s
Rank
Contribution (% of total JSD = 0.0659 bit)
1.5
1
0.5
0
0.5
1
1
5
10
15
20
25
30
35
40
45
50
55
60
"
;
)
(
.
'
which
-
upon
t
the
of
his
:
—
shall
""""
She
Mr
patients
/
Lord
didn
great
thy
my
thou
and
*
%
by
group
their
social
The
to
every
problems
1980
economic
so
problem
our
role
Sir
he
1979
1978
thee
development
it
&c
1977
whom
God
don
but
!
data
pp
English Fiction V1

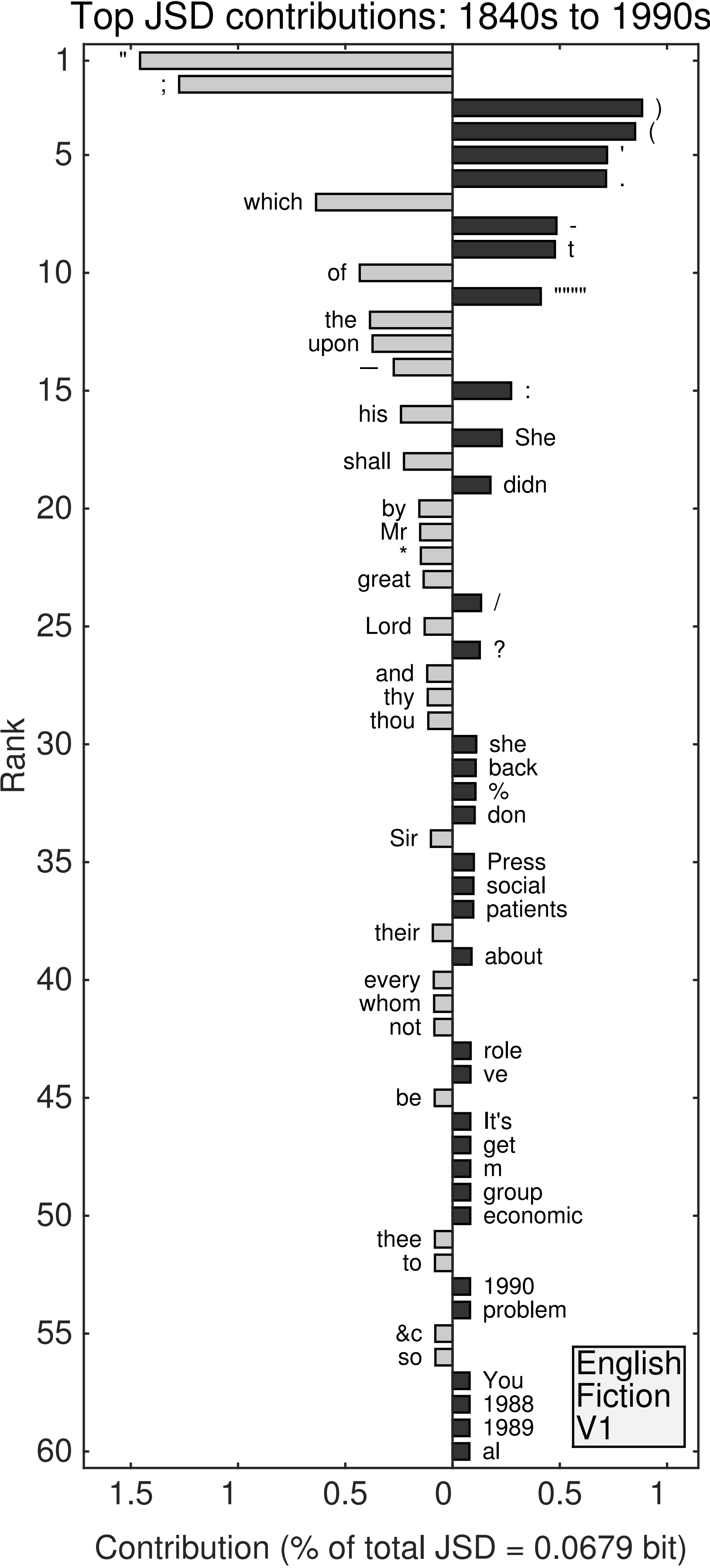

Top JSD contributions: 1840s to 1990s
Rank
Contribution (% of total JSD = 0.0679 bit)
1.5
1
0.5
0
0.5
1
1
5
10
15
20
25
30
35
40
45
50
55
60
"
;
)
(
'
.
which
-
t
of
""""
the
upon
—
:
his
She
shall
didn
by
Mr
*
great
/
Lord
?
and
thy
thou
she
back
%
don
Sir
Press
social
patients
their
about
every
whom
not
role
ve
be
It's
get
m
group
economic
thee
to
1990
problem
&c
so
You
1988
1989
al
English Fiction V1

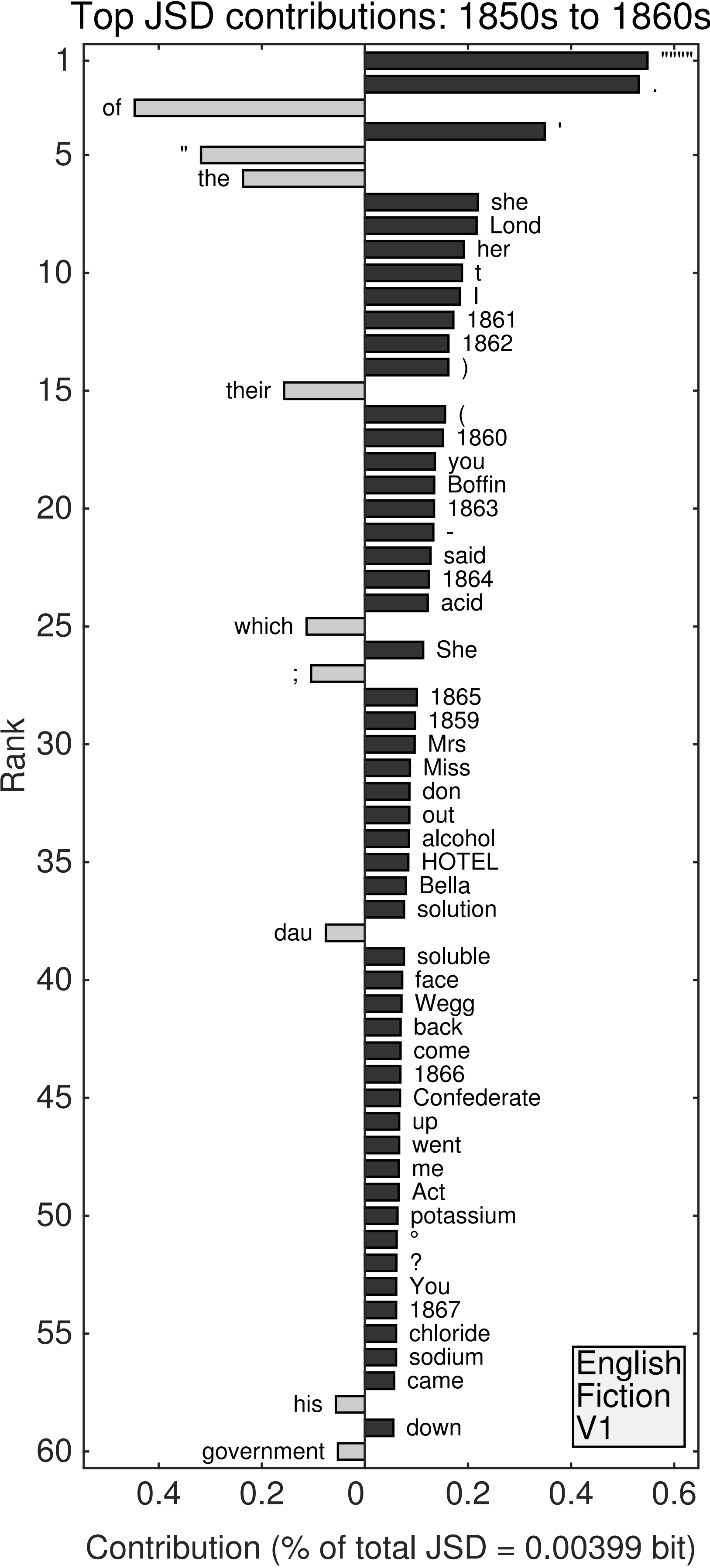

Top JSD contributions: 1850s to 1860s
Rank
Contribution (% of total JSD = 0.00399 bit)
English Fiction V1
"""""
.
of
'
"
the
she
Lond
her
t
I
1861
1862
)
their
(
1860
you
Boffin
1863
-
said
1864
acid
which
She
;
1865
1859
Mrs
Miss
don
out
alcohol
HOTEL
Bella
solution
dau
soluble
face
Wegg
back
come
1866
Confederate
up
went
me
Act
potassium
°
?
You
1867
chloride
sodium
came
his
down
government

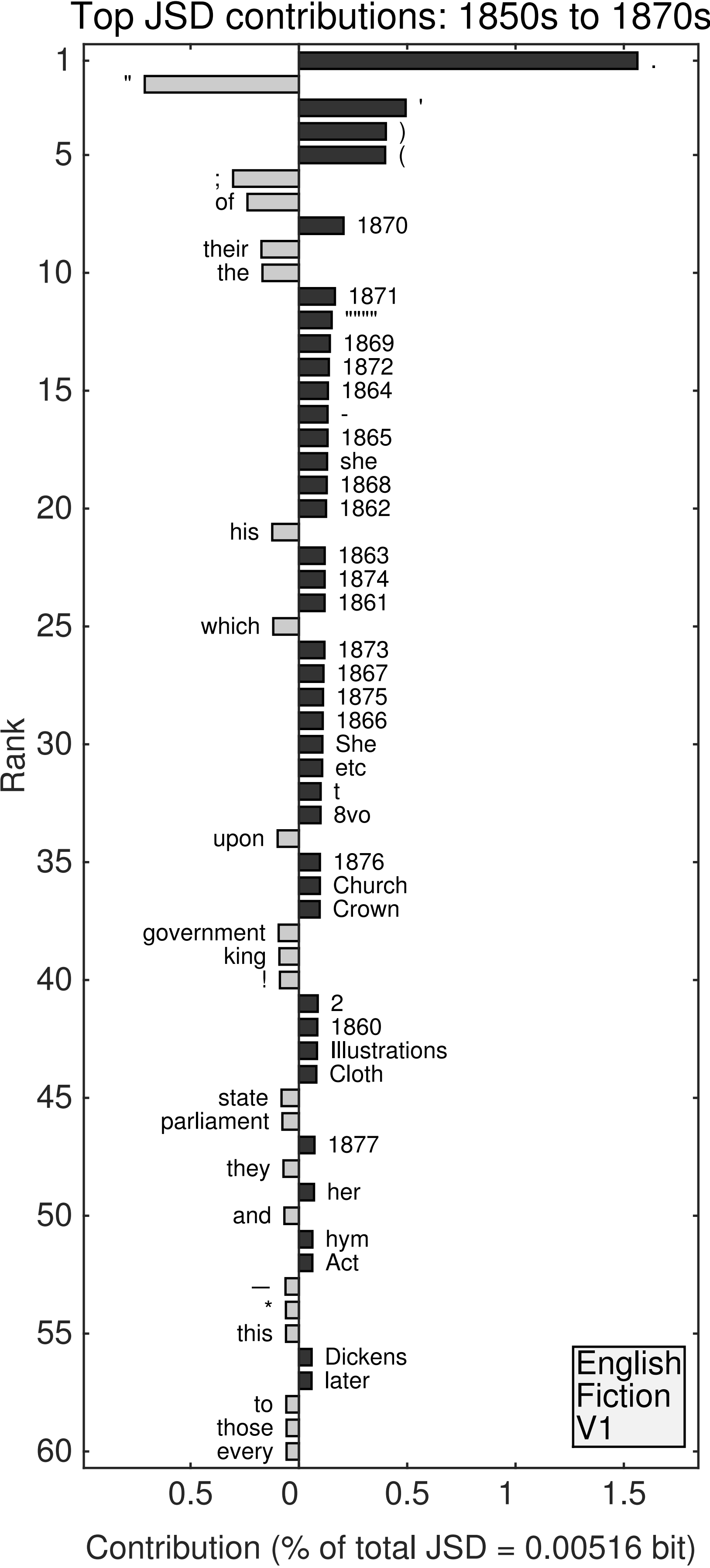

Top JSD contributions: 1850s to 1870s
Rank
Contribution (% of total JSD = 0.00516 bit)
.
"
'
)
(
;
of
1870
their
the
1871
""""
1869
1872
1864
-
1865
she
1868
1862
his
1863
1874
1861
which
1873
1867
1875
1866
She
etc
t
8vo
upon
1876
Church
Crown
government
king
!
2
1860
Illustrations
Cloth
state
parliament
1877
they
her
and
hym
Act
—
*
this
Dickens
later
to
those
every
English
Fiction
V1

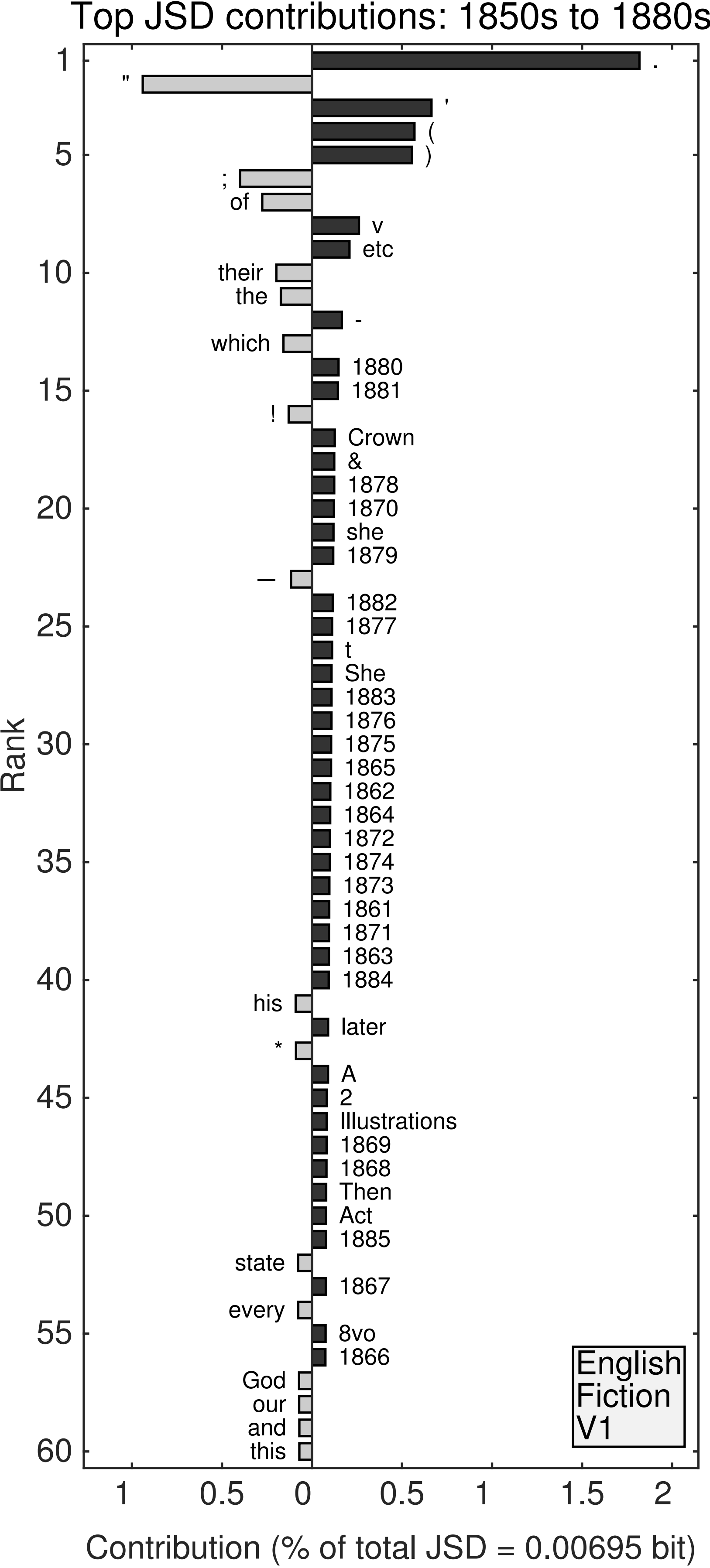
Top JSD contributions: 1850s to 1880s
Rank
Contribution (% of total JSD = 0.00695 bit)
English Fiction V1
.
"
'
(
)
;
of
v
etc
their
the
-
which
1880
1881
!
Crown
&
1878
1870
she
1879
—
1882
1877
t
She
1883
1876
1875
1865
1862
1864
1872
1874
1873
1861
1871
1863
1884
his
later
*
A
2
Illustrations
1869
1868
Then
Act
1885
state
1867
every
8vo
1866
God
our
and
this

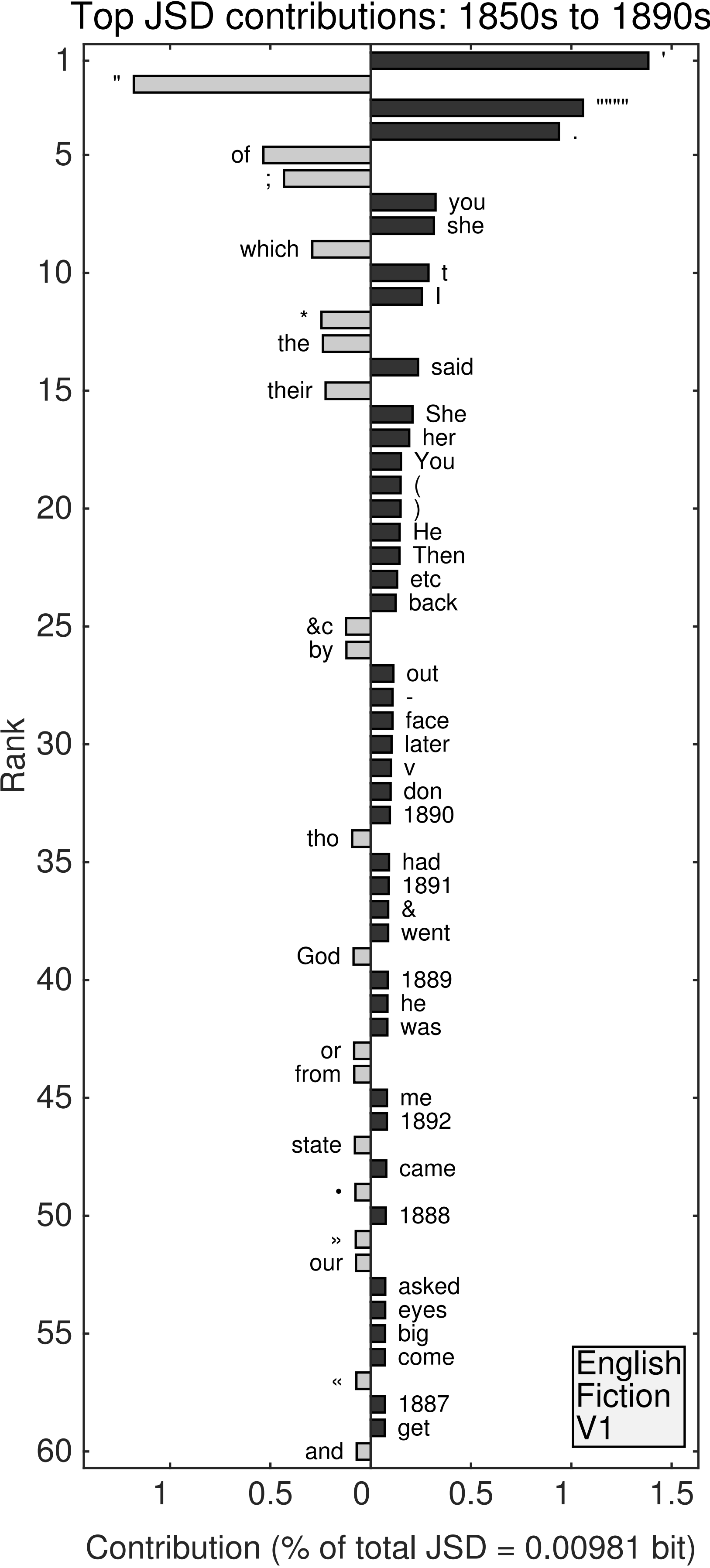

Top JSD contributions: 1850s to 1890s
Rank
Contribution (% of total JSD = 0.00981 bit)
English Fiction V1
1
5
10
15
20
25
30
35
40
45
50
55
60
1
0.5
0
0.5
1
1.5
'
"
"""""
.
of
;
you
she
which
t
I
*
the
said
their
She
her
You
(
)
He
Then
etc
back
&c
by
out
-
face
later
v
don
1890
tho
had
1891
&
went
God
1889
he
was
or
from
me
1892
state
came
•
1888
»
our
asked
eyes
big
come
«
1887
get
and

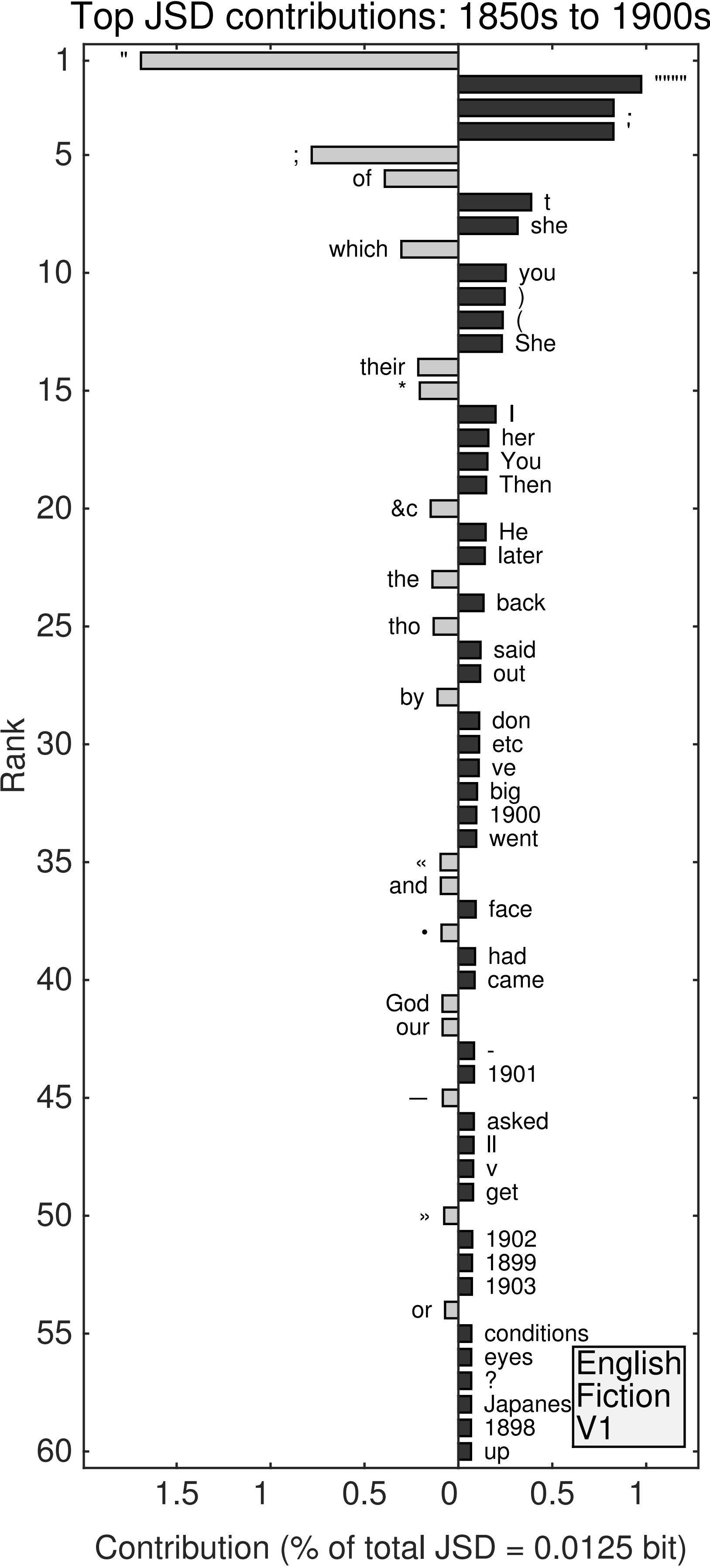
Top JSD contributions: 1850s to 1900s
Rank
Contribution (% of total JSD = 0.0125 bit)
"
""""
;
'
;
of
t
she
which
you
)
(
She
their
*
I
her
You
Then
&c
He
later
the
back
tho
said
out
by
don
etc
ve
big
1900
went
«
and
face
•
had
came
God
our
-
1901
—
asked
ll
v
get
»
1902
1899
1903
or
conditions
eyes
?
Japanes
1898
up
English Fiction V1

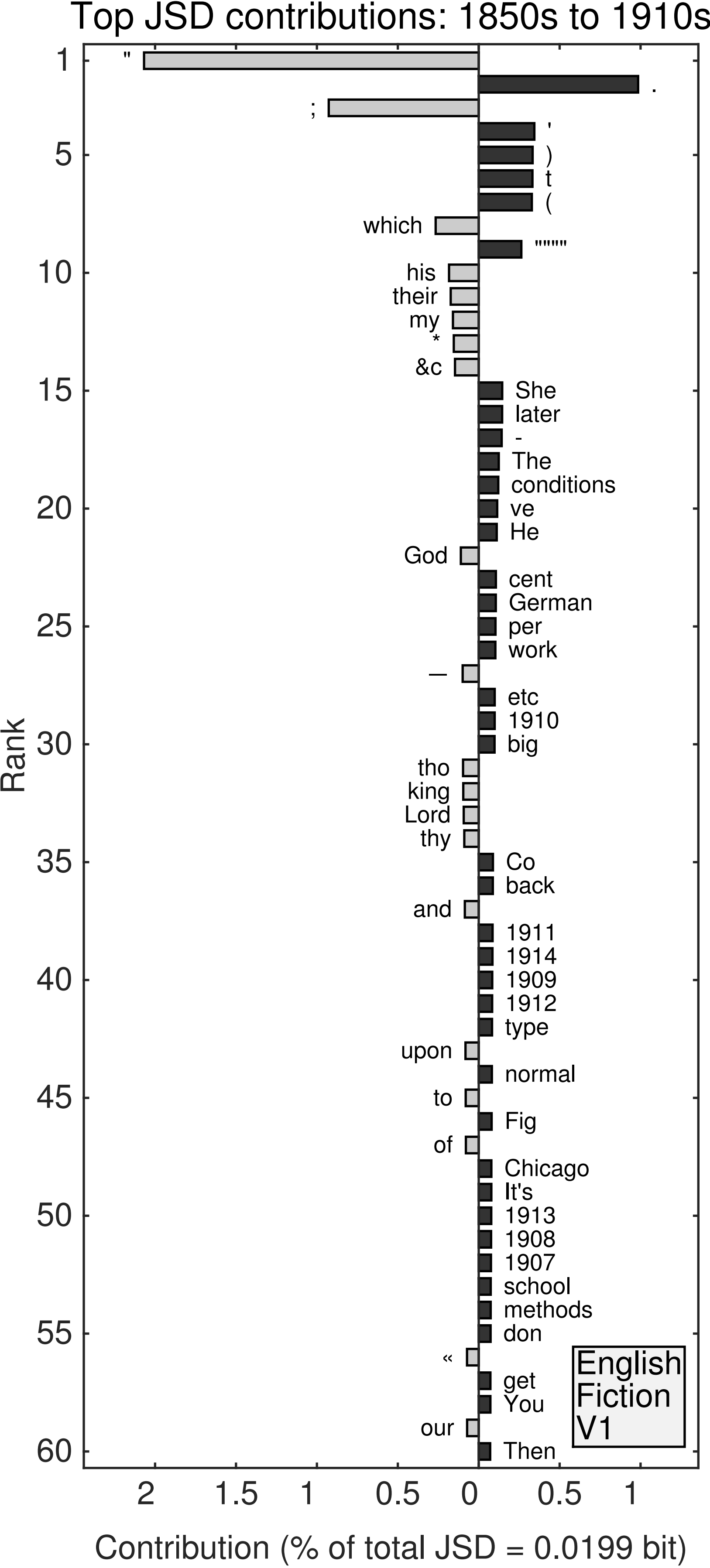

Top JSD contributions: 1850s to 1910s
Rank
Contribution (% of total JSD = 0.0199 bit)
"
.
;
'
)
t
(
which
""""
his
their
my
*
&c
She
later
-
The
conditions
ve
He
God
cent
German
per
work
—
etc
1910
big
tho
king
Lord
thy
Co
back
and
1911
1914
1909
1912
type
upon
normal
to
Fig
of
Chicago
It's
1913
1908
1907
school
methods
don
«
get
You
our
Then
English Fiction V1

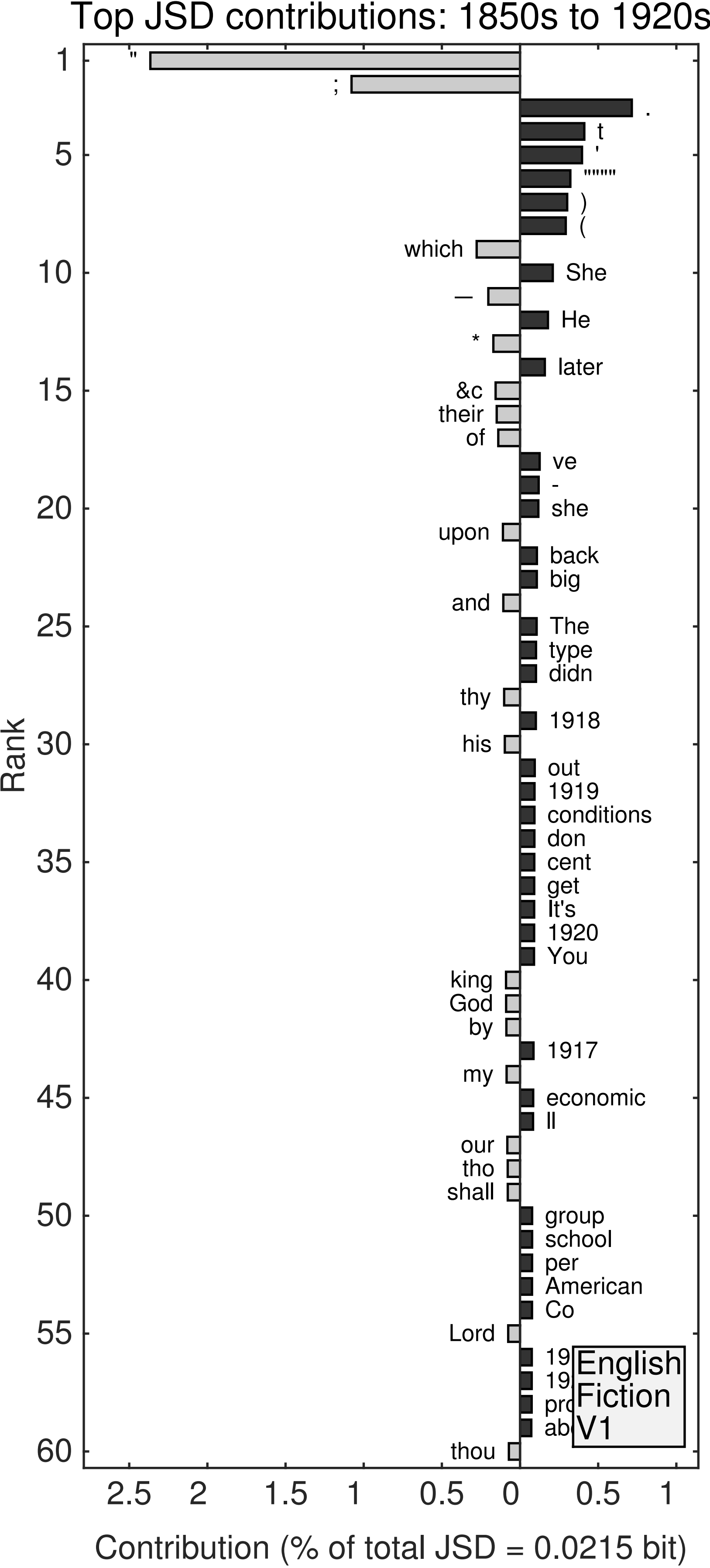
Top JSD contributions: 1850s to 1920s
Rank
Contribution (% of total JSD = 0.0215 bit)
English Fiction V1
"
;
.
t
'
""""
)
(
which
She
—
He
*
later
&c
their
of
ve
-
she
upon
back
big
and
The
type
didn
thy
1918
his
out
1919
conditions
don
cent
get
It's
1920
You
king
God
by
1917
my
economic
ll
our
tho
shall
group
school
per
American
Co
Lord
thou
2.5
2
1.5
1
0.5
0
0.5
1
5
10
15
20
25
30
35
40
45
50
55
60

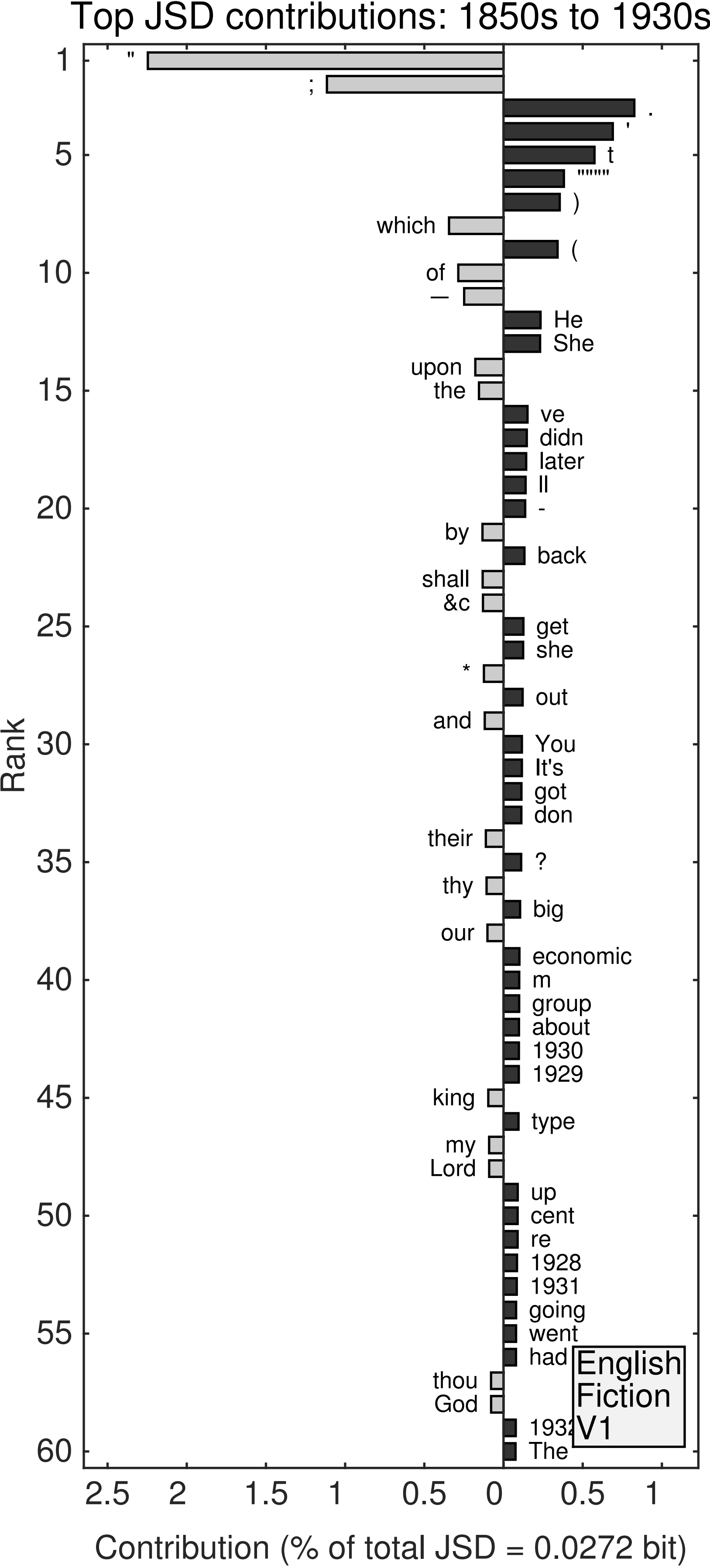
Top JSD contributions: 1850s to 1930s
Rank
Contribution (% of total JSD = 0.0272 bit)
English Fiction V1
"
;
.
'
t
)
which
(
of
—
He
She
upon
the
ve
didn
later
ll
-
by
back
shall
&c
get
she
*
out
and
You
It's
got
don
their
?
thy
big
our
economic
m
group
about
1930
1929
king
type
my
Lord
up
cent
re
1928
1931
going
went
had
thou
God
The

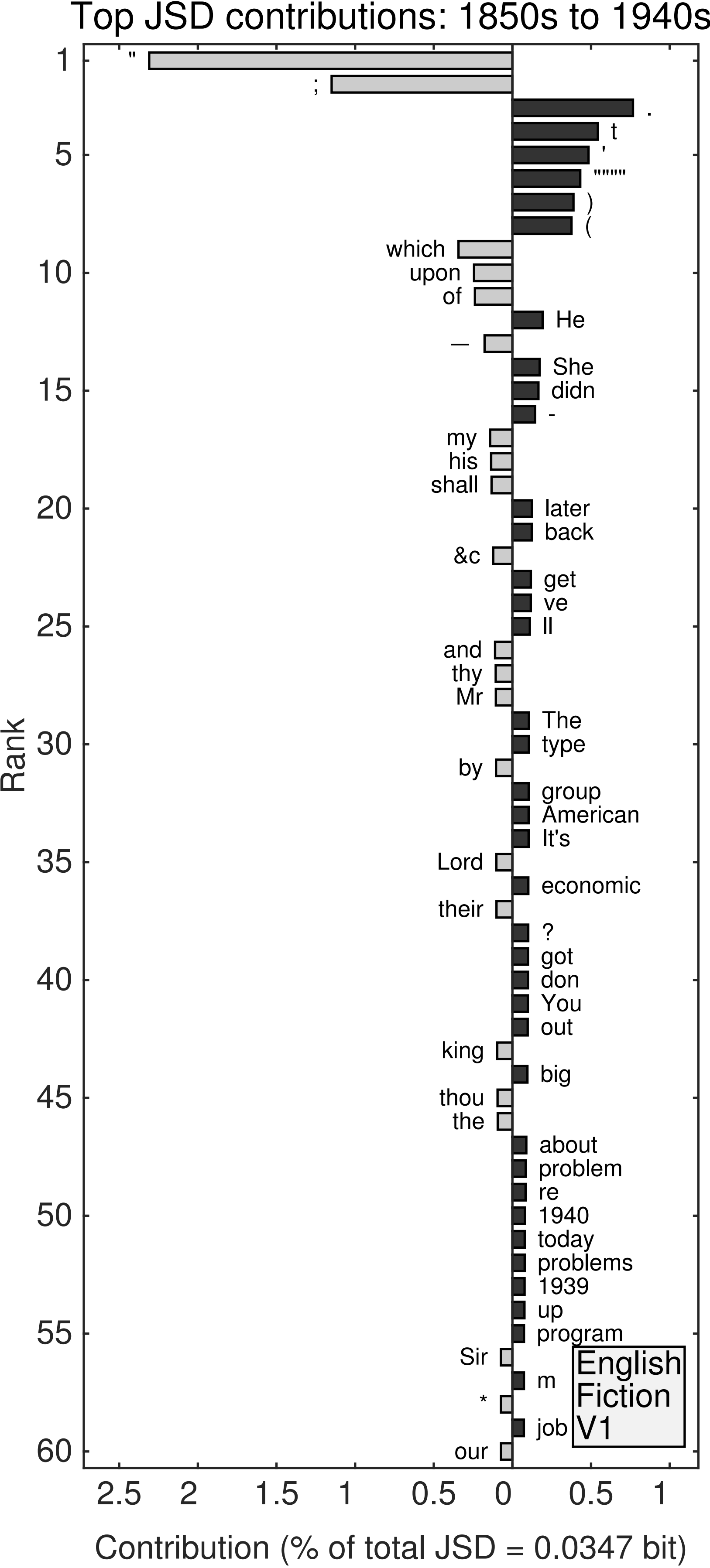

Top JSD contributions: 1850s to 1940s
Rank
Contribution (% of total JSD = 0.0347 bit)
"
;
.
t
'
""""
)
(
which
upon
of
He
—
She
didn
-
my
his
shall
later
back
&c
get
ve
ll
and
thy
Mr
The
type
by
group
American
It's
Lord
economic
their
?
got
don
You
out
king
big
thou
the
about
problem
re
1940
today
problems
1939
up
program
Sir
m
*
job
our
English Fiction V1

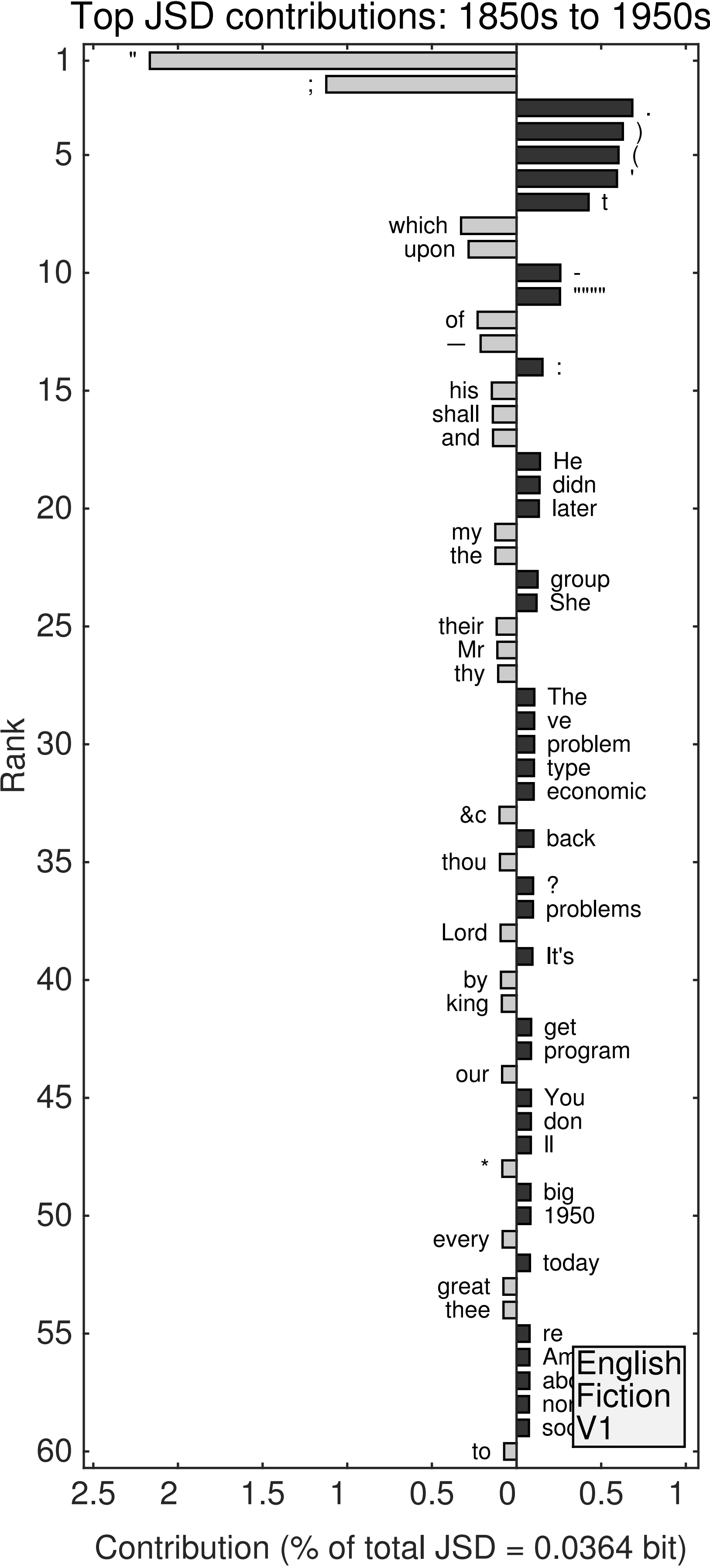

Top JSD contributions: 1850s to 1950s
Rank
Contribution (% of total JSD = 0.0364 bit)
English Fiction V1
"
;
.
)
(
'
t
which
upon
-
""""
of
—
:
his
shall
and
He
didn
later
my
the
group
She
their
Mr
thy
The
ve
problem
type
economic
&c
back
thou
?
problems
Lord
It's
by
king
get
program
our
You
don
ll
*
big
1950
every
today
great
thee
re
to

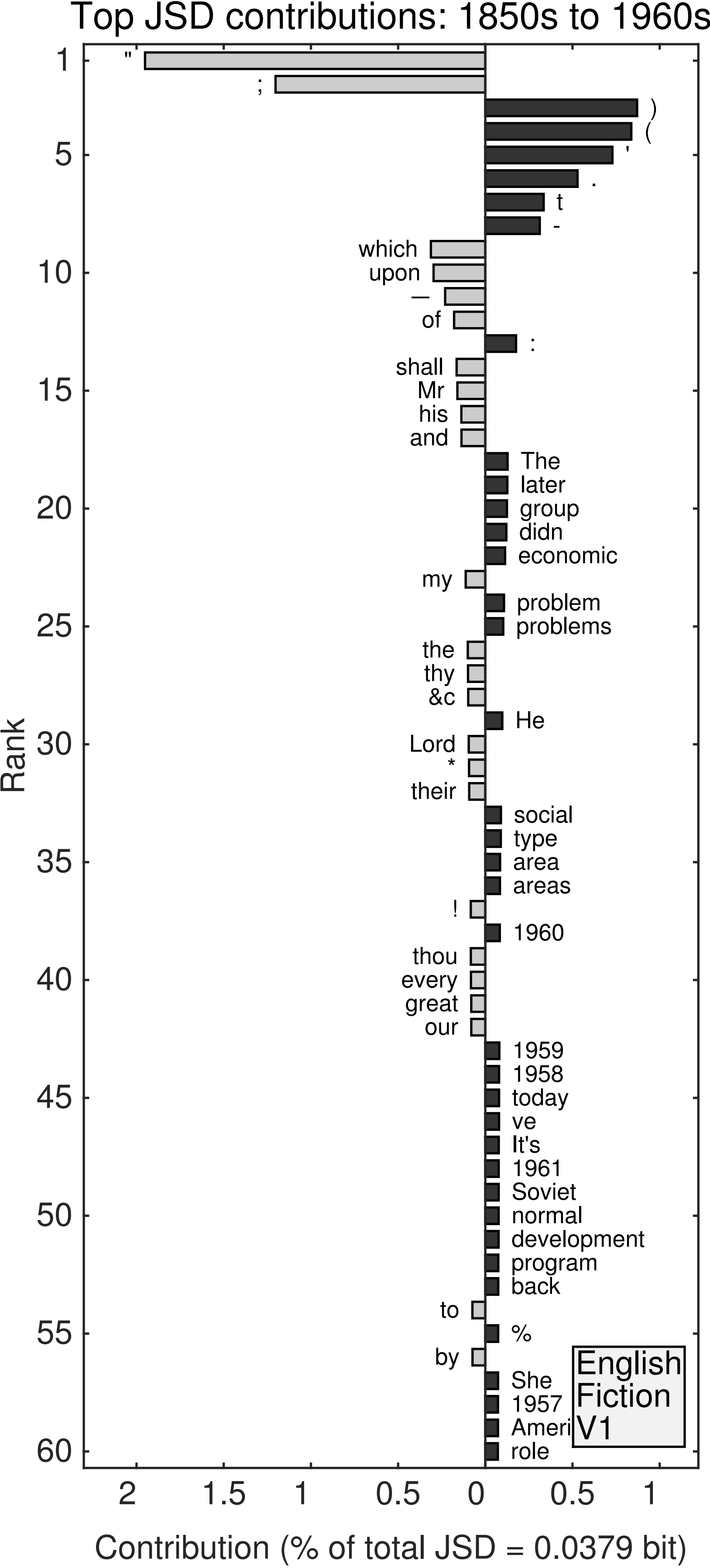

Top JSD contributions: 1850s to 1960s
Rank
Contribution (% of total JSD = 0.0379 bit)
English Fiction V1
"
;
)
(
'
.
t
-
which
upon
—
of
:
shall
Mr
his
and
The
later
group
didn
economic
my
problem
problems
the
thy
&c
He
Lord
*
their
social
type
area
areas
!
1960
thou
every
great
our
1959
1958
today
ve
It's
1961
Soviet
normal
development
program
back
to
%
by
She
1957
Ameri
role

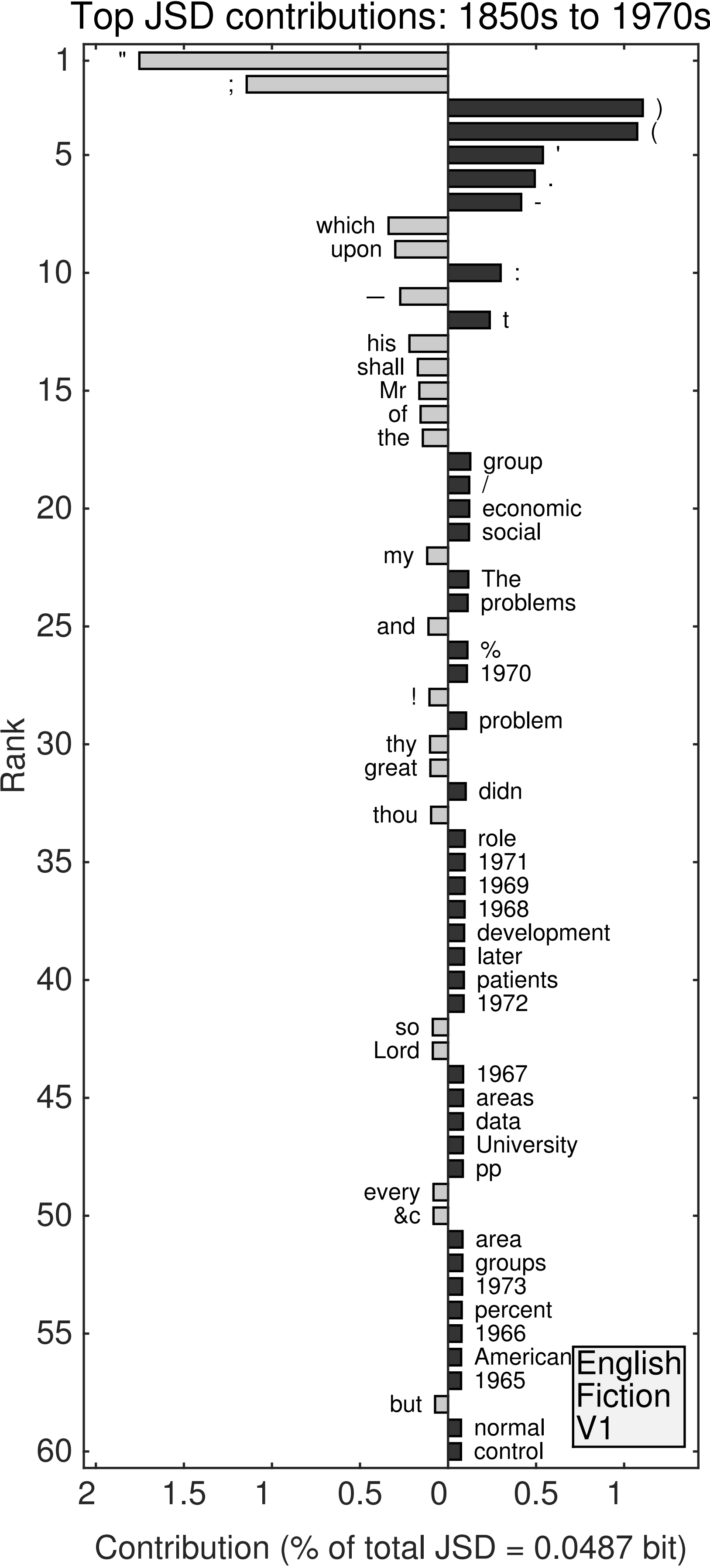

Top JSD contributions: 1850s to 1970s
Rank
Contribution (% of total JSD = 0.0487 bit)
"
;
)
(
'
.
-
which
upon
:
—
t
his
shall
Mr
of
the
group
/
economic
social
my
The
problems
and
%
1970
!
problem
thy
great
didn
thou
role
1971
1969
1968
development
later
patients
1972
so
Lord
1967
areas
data
University
pp
every
&c
area
groups
1973
percent
1966
American
1965
but
normal
control
English
Fiction
V1

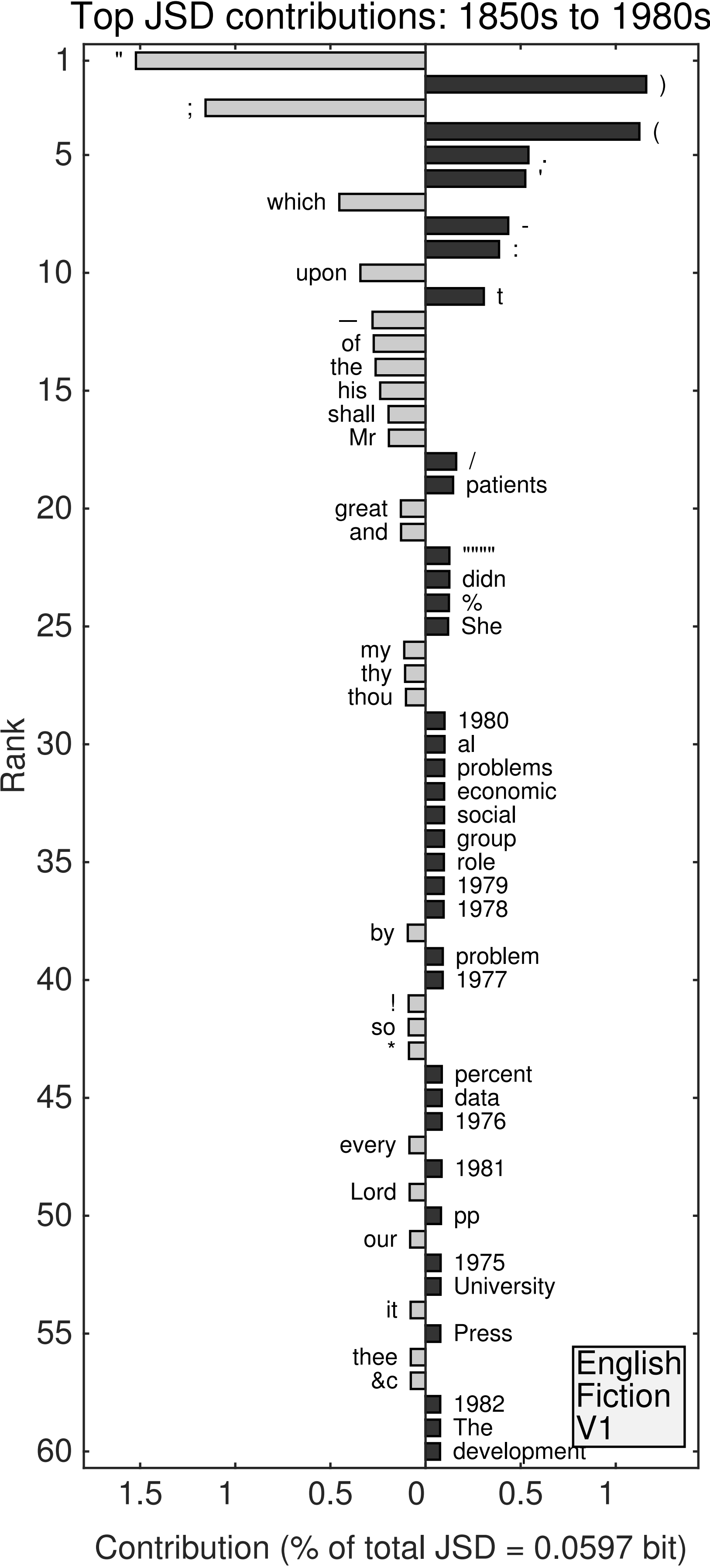

Top JSD contributions: 1850s to 1980s
Rank
Contribution (% of total JSD = 0.0597 bit)
English Fiction V1
"
)
;
(
which
-
:
upon
t
—
of
the
his
shall
Mr
/
patients
great
and
""""
didn
%
She
my
thy
thou
1980
al
problems
economic
social
group
role
1979
1978
by
problem
1977
!
so
*
percent
data
1976
every
1981
Lord
pp
our
1975
University
it
Press
thee
&c
1982
The
development

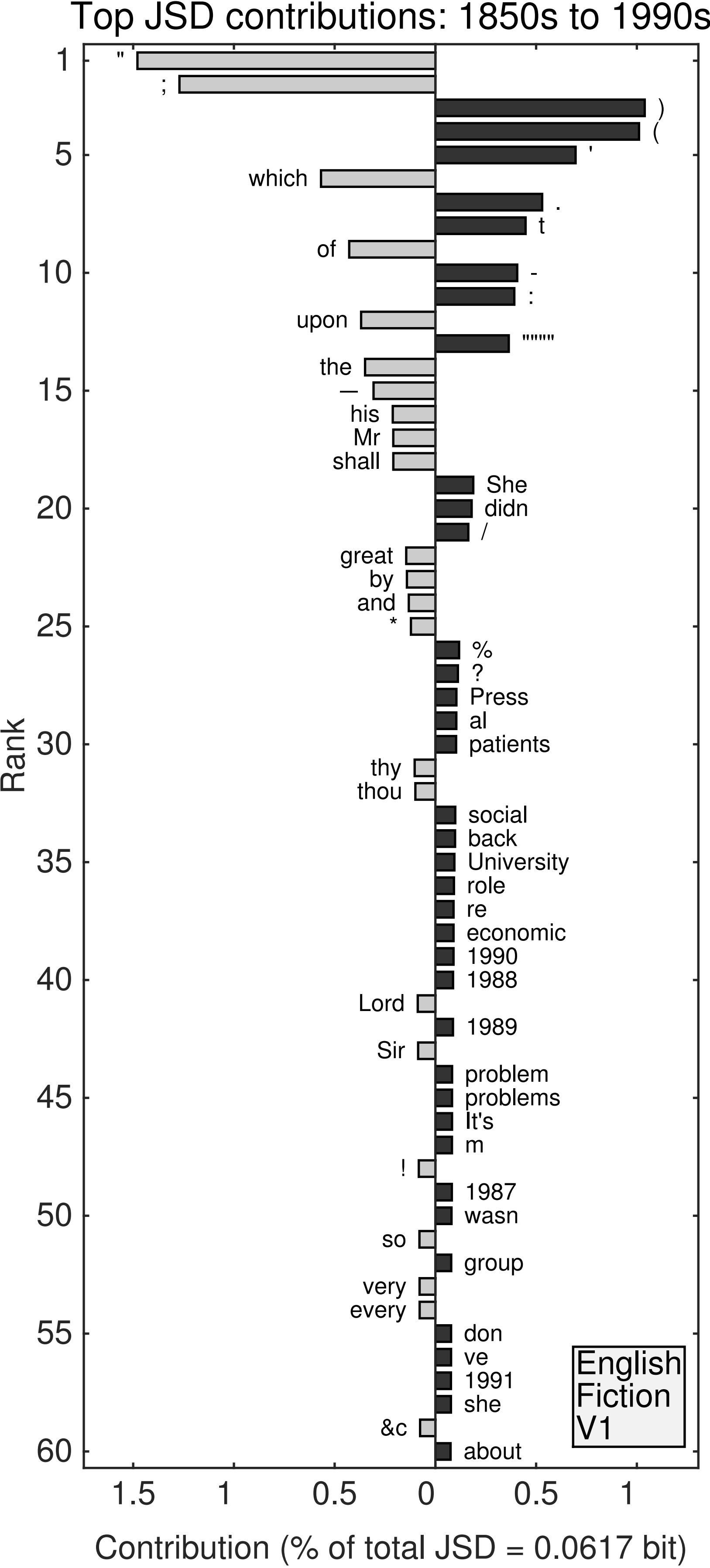

Top JSD contributions: 1850s to 1990s
Rank
Contribution (% of total JSD = 0.0617 bit)
1.5 1 0.5 0 0.5 1
1 5 10 15 20 25 30 35 40 45 50 55 60
"
;
)
(
'
which
.
t
of
-
:
upon
""""
the
—
his
Mr
shall
She
didn
/
great
by
and
*
%
?
Press
al
patients
thy
thou
social
back
University
role
re
economic
1990
1988
Lord
1989
Sir
problem
problems
It's
m
!
1987
wasn
so
group
very
every
don
ve
1991
she
&c
about
English Fiction V1

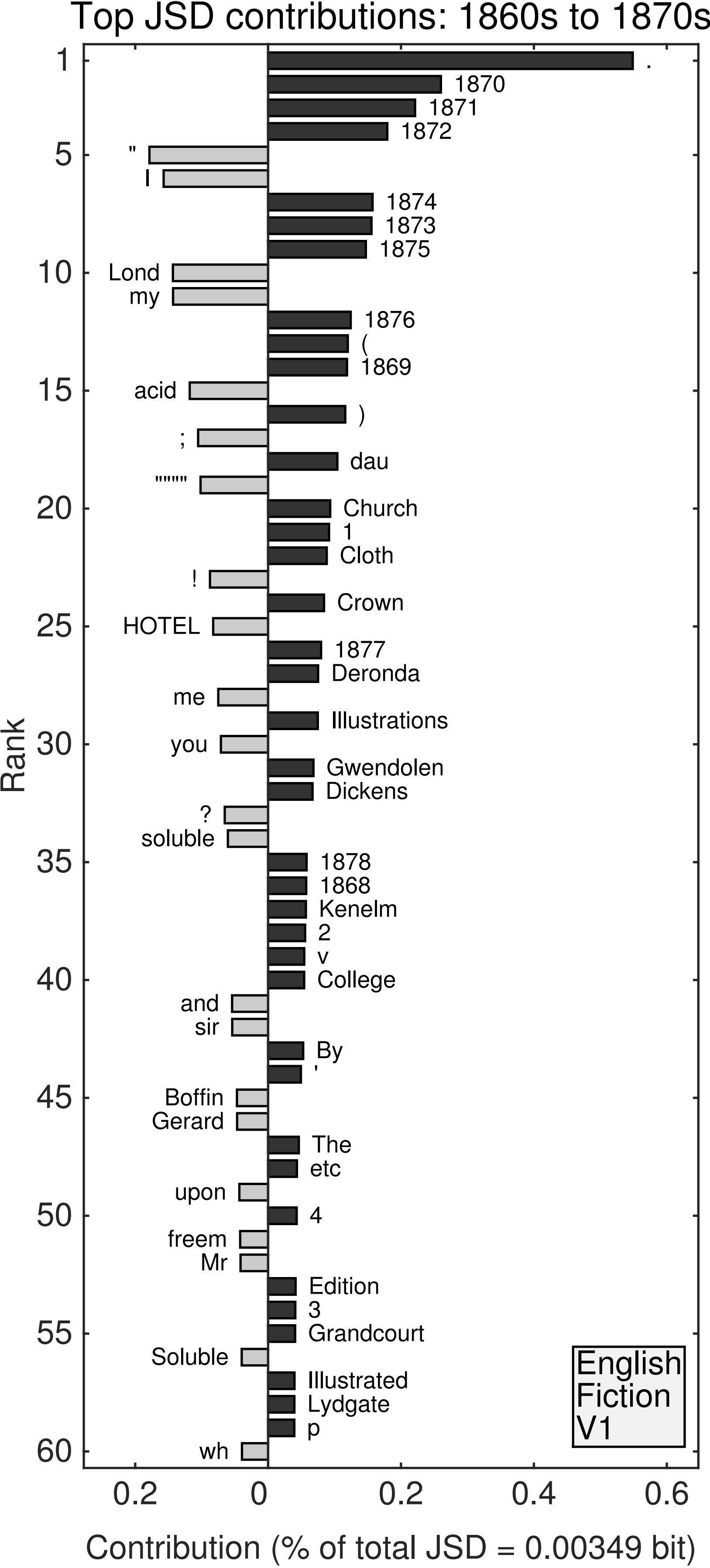
Top JSD contributions: 1860s to 1870s
Rank
Contribution (% of total JSD = 0.00349 bit)
.
1870
1871
1872
"
I
1874
1873
1875
Lond
my
1876
(
1869
acid
)
;
dau
""""
Church
1
Cloth
!
Crown
HOTEL
1877
Deronda
me
Illustrations
you
Gwendolen
Dickens
?
soluble
1878
1868
Kenelm
2
v
College
and
sir
By
'
Boffin
Gerard
The
etc
upon
4
freem
Mr
Edition
3
Grandcourt
Soluble
Illustrated
Lydgate
p
wh
English Fiction V1

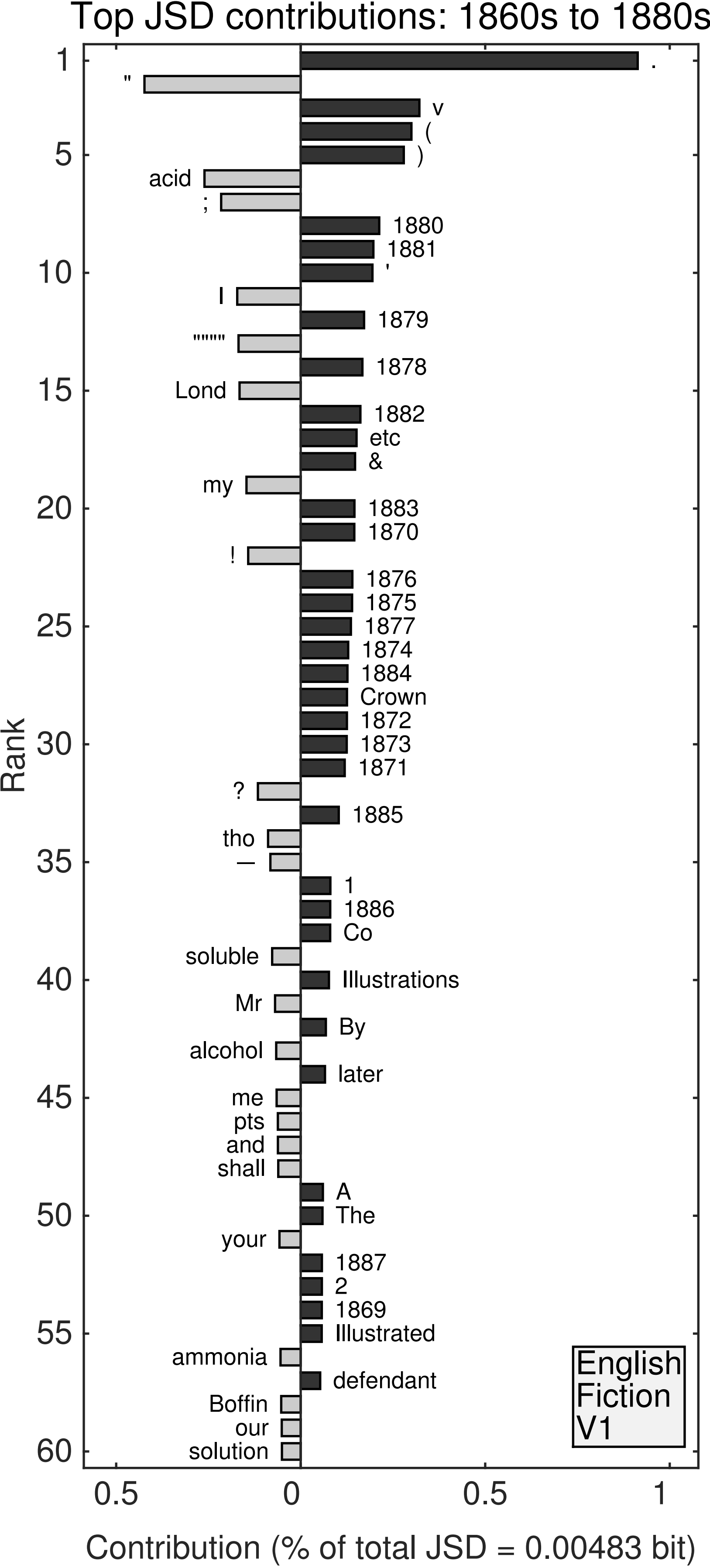

Top JSD contributions: 1860s to 1880s
Rank
Contribution (% of total JSD = 0.00483 bit)
0.5
0
0.5
1
1
5
10
15
20
25
30
35
40
45
50
55
60
.
"
v
(
)
acid
;
1880
1881
'
I
1879
""""
1878
Lond
1882
etc
&
my
1883
1870
!
1876
1875
1877
1874
1884
Crown
1872
1873
1871
?
1885
tho
—
1
1886
Co
soluble
Illustrations
Mr
By
alcohol
later
me
pts
and
shall
A
The
your
1887
2
1869
Illustrated
ammonia
defendant
Boffin
our
solution
English Fiction V1

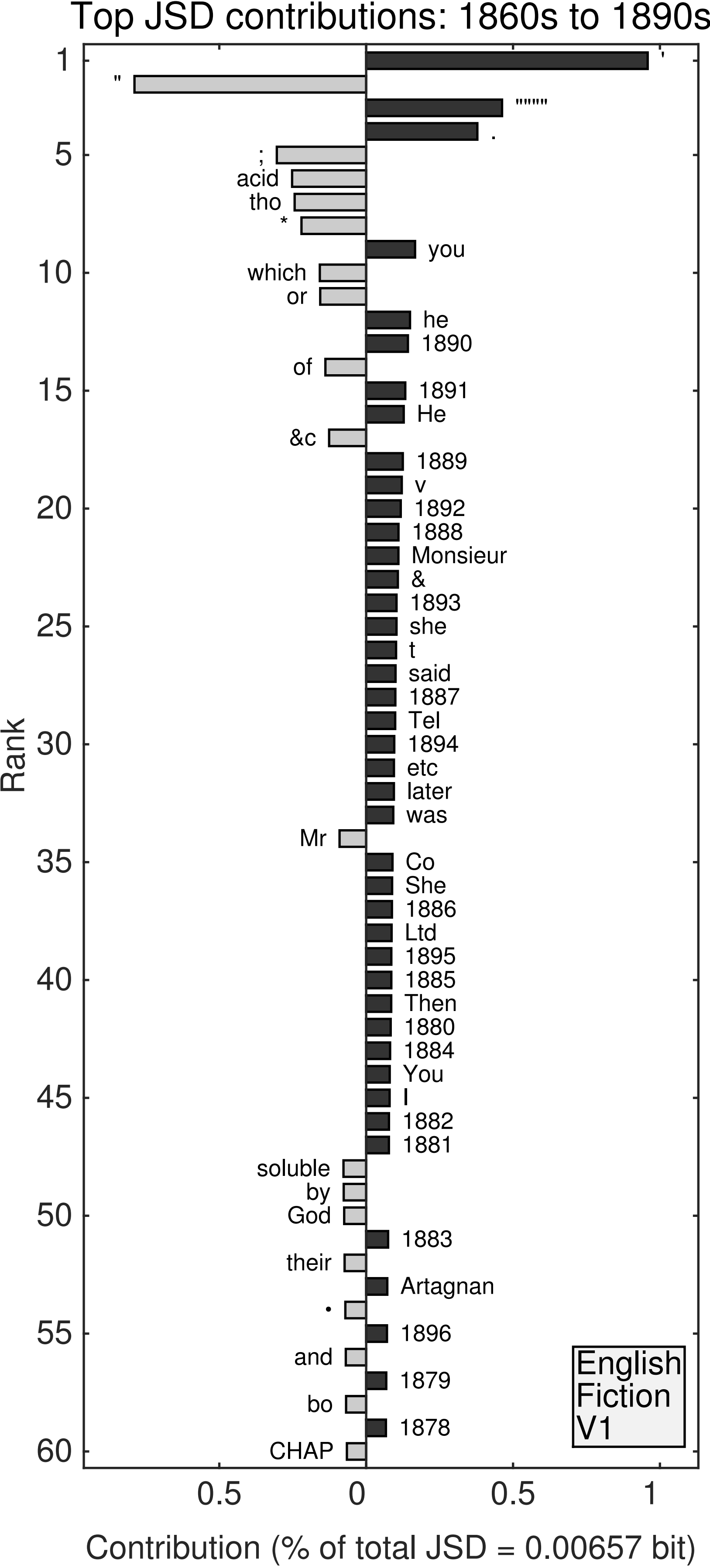

Top JSD contributions: 1860s to 1890s
Rank
Contribution (% of total JSD = 0.00657 bit)
English Fiction V1
'
"
""""
.
;
acid
tho
*
you
which
or
he
1890
of
1891
He
&c
1889
v
1892
1888
Monsieur
&
1893
she
t
said
1887
Tel
1894
etc
later
was
Mr
Co
She
1886
Ltd
1895
1885
Then
1880
1884
You
I
1882
1881
soluble
by
God
1883
their
Artagnan
•
1896
and
1879
bo
1878
CHAP

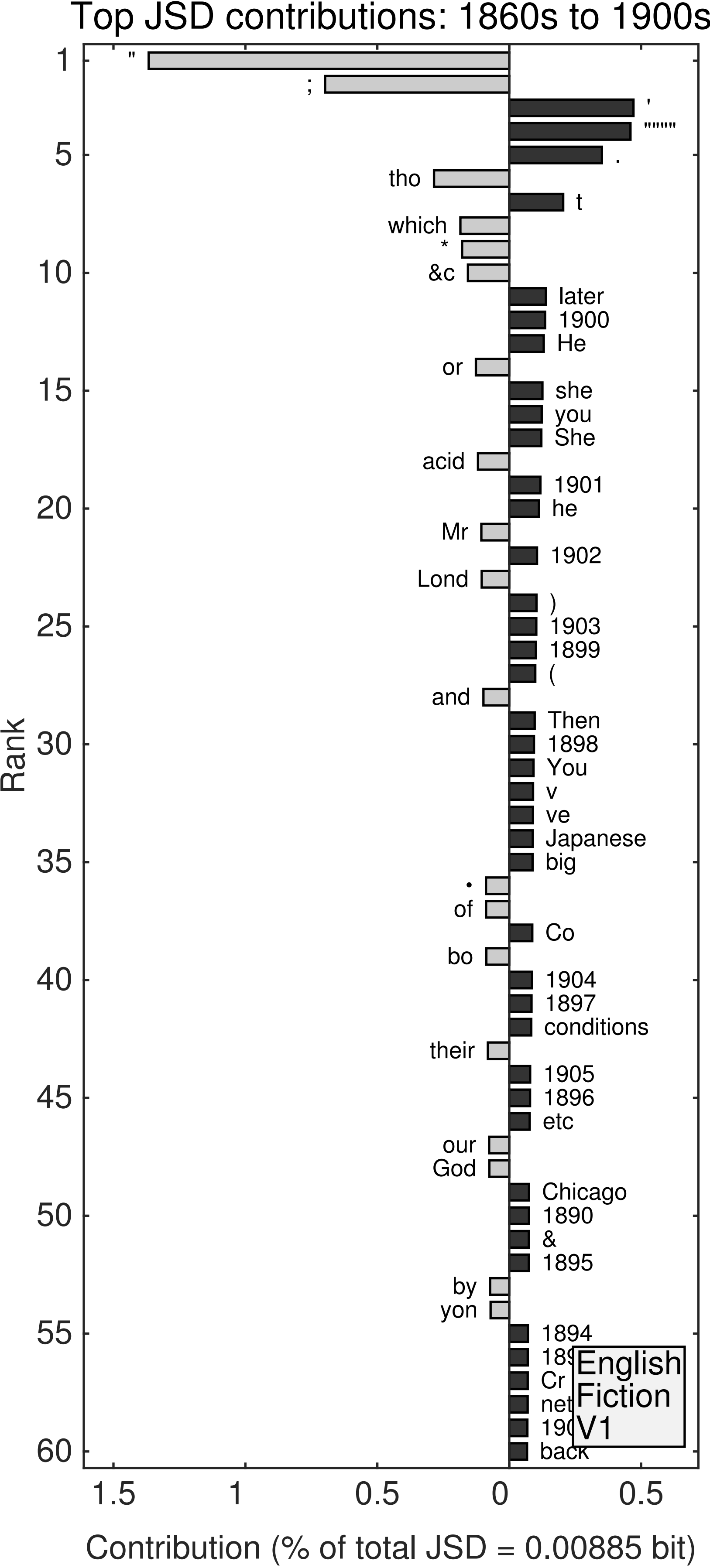
Top JSD contributions: 1860s to 1900s
Rank
Contribution (% of total JSD = 0.00885 bit)
"
;
'
"""""
.
tho
t
which
*
&c
later
1900
He
or
she
you
She
acid
1901
he
Mr
1902
Lond
)
1903
1899
(
and
Then
1898
You
v
ve
Japanese
big
•
of
Co
bo
1904
1897
conditions
their
1905
1896
etc
our
God
Chicago
1890
&
1895
by
yon
1894
Cr
back
English
Fiction
V1

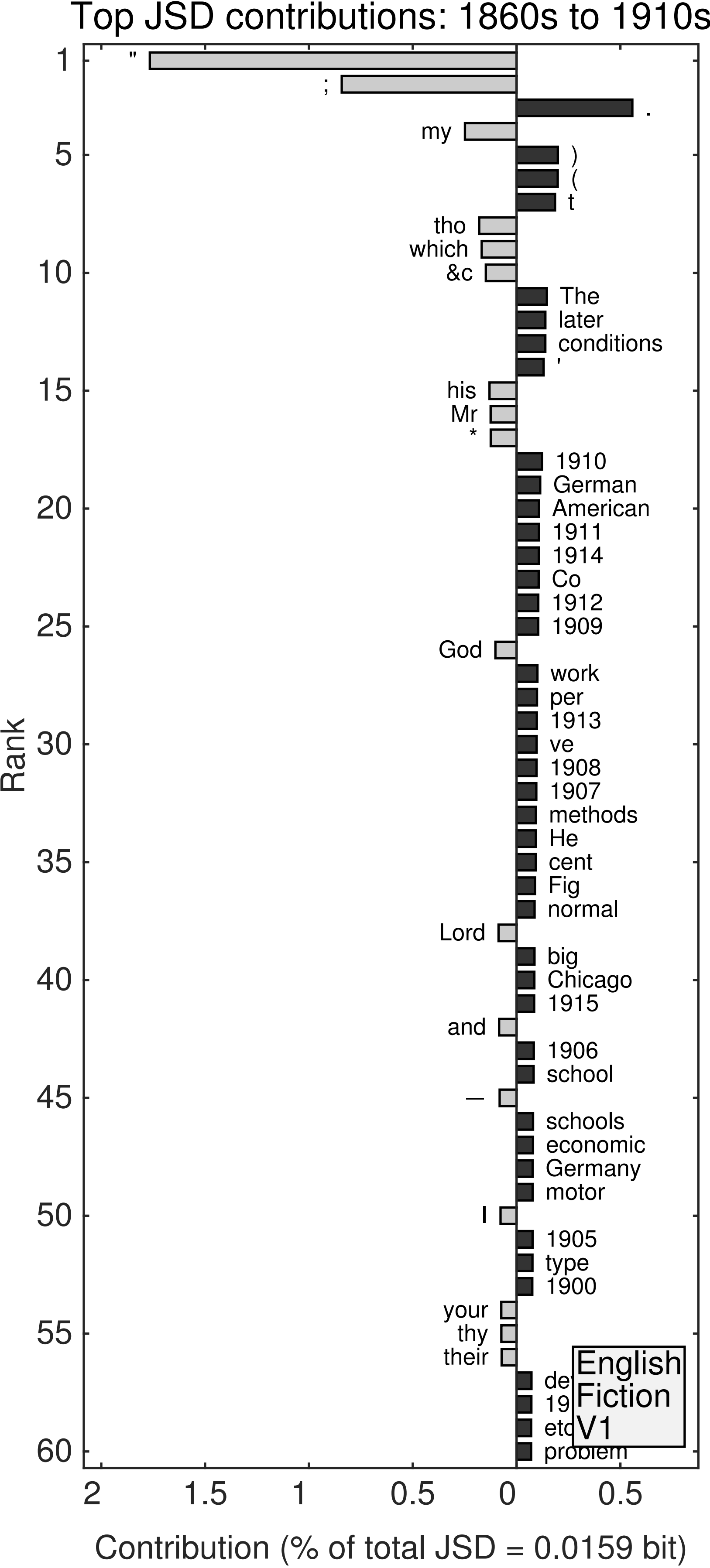

Top JSD contributions: 1860s to 1910s
Rank
Contribution (% of total JSD = 0.0159 bit)
English Fiction V1
1
5
10
15
20
25
30
35
40
45
50
55
60
2
1.5
1
0.5
0
0.5
"
;
.
my
)
(
t
tho
which
&c
The
later
conditions
'
his
Mr
*
1910
German
American
1911
1914
Co
1912
1909
God
work
per
1913
ve
1908
1907
methods
He
cent
Fig
normal
Lord
big
Chicago
1915
and
1906
school
—
schools
economic
Germany
motor
I
1905
type
1900
your
thy
their
problem

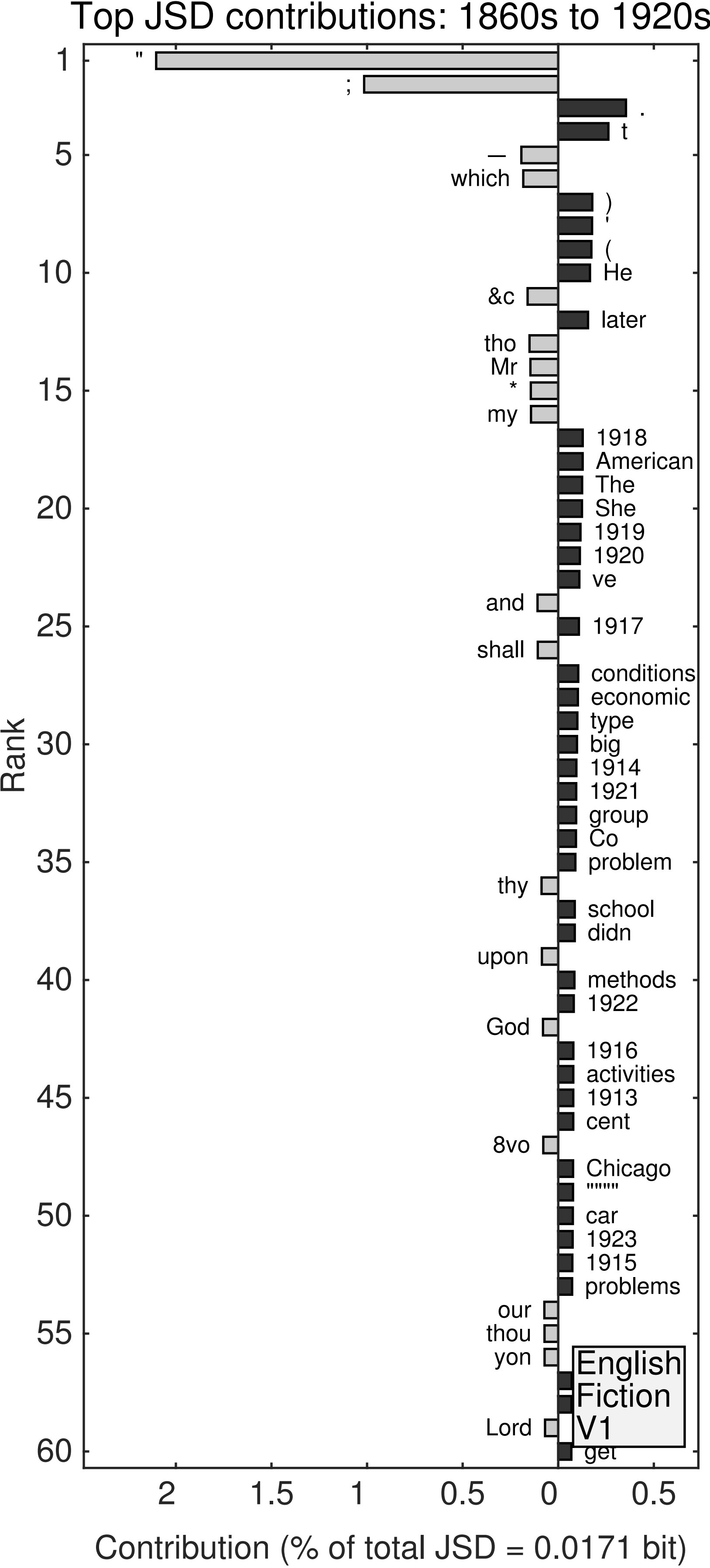
Top JSD contributions: 1860s to 1920s
Rank
Contribution (% of total JSD = 0.0171 bit)
1
5
10
15
20
25
30
35
40
45
50
55
60
2
1.5
1
0.5
0
0.5
"
;
.
t
—
which
)
'
(
He
&c
later
tho
Mr
*
my
1918
American
The
She
1919
1920
ve
and
1917
shall
conditions
economic
type
big
1914
1921
group
Co
problem
thy
school
didn
upon
methods
1922
God
1916
activities
1913
cent
8vo
Chicago
""""
car
1923
1915
problems
our
thou
yon
Lord
get
English
Fiction
V1

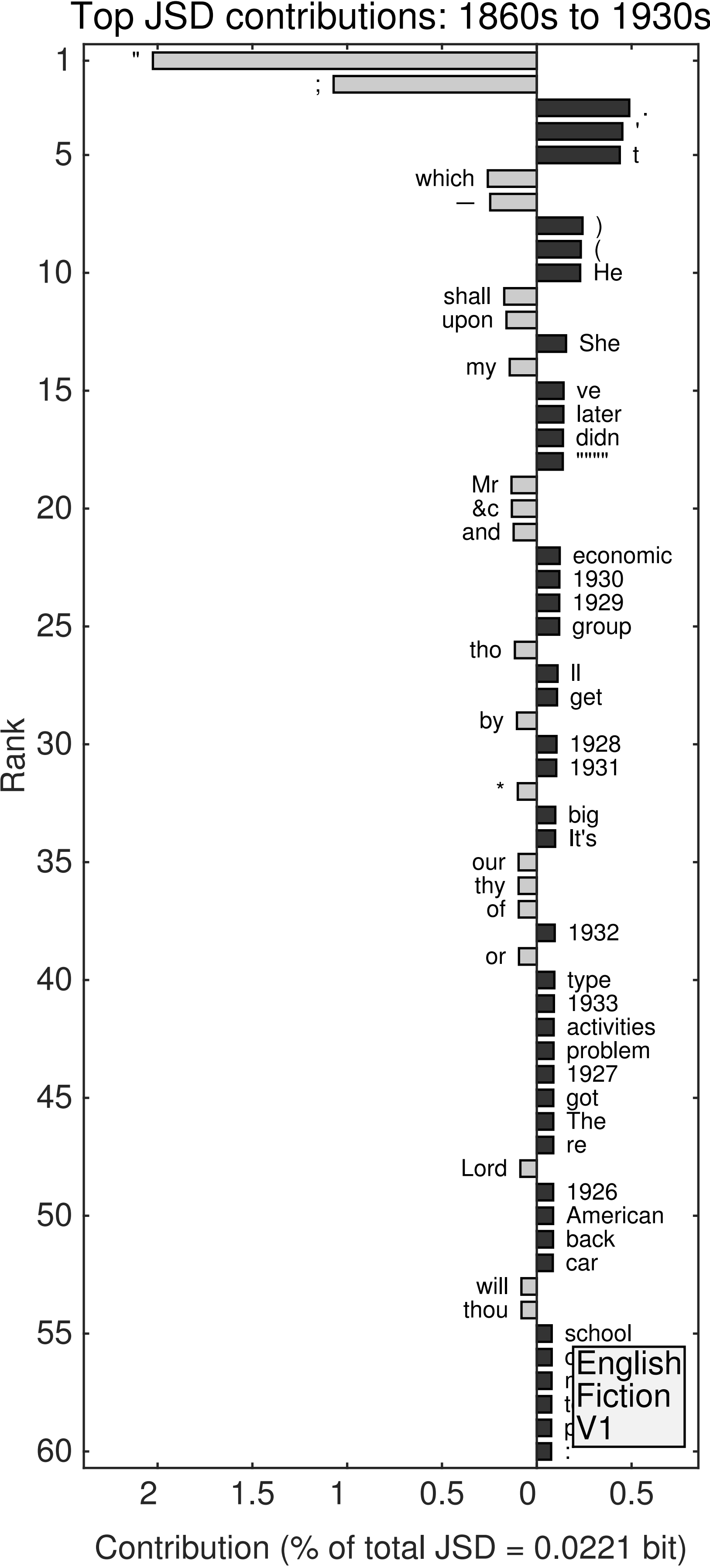

Top JSD contributions: 1860s to 1930s
Rank
Contribution (% of total JSD = 0.0221 bit)
"
;
.
'
t
which
—
)
(
He
shall
upon
She
my
ve
later
didn
""""
Mr
&c
and
economic
1930
1929
group
tho
ll
get
by
1928
1931
*
big
It's
our
thy
of
1932
or
type
1933
activities
problem
1927
got
The
re
Lord
1926
American
back
car
will
thou
school
English
Fiction
V1

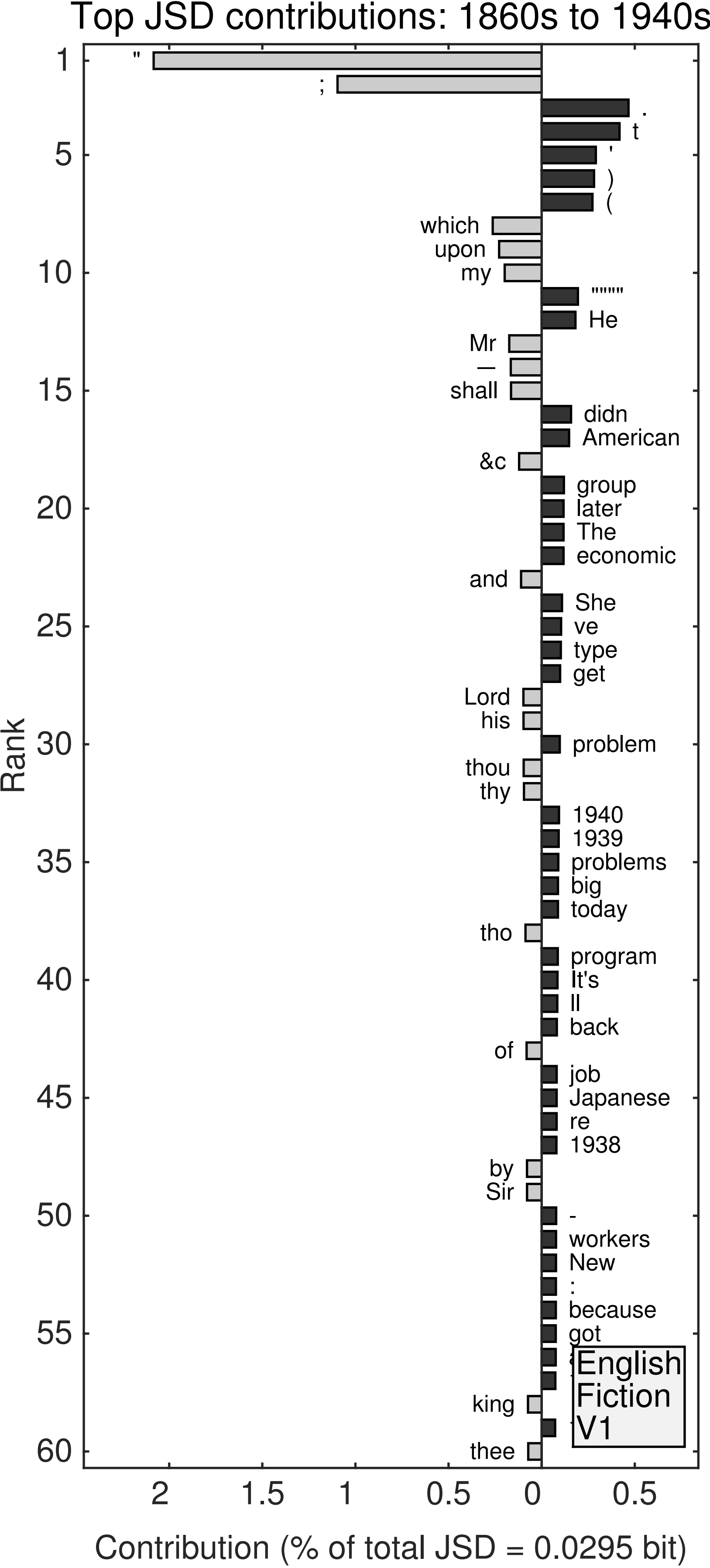

Top JSD contributions: 1860s to 1940s
Rank
Contribution (% of total JSD = 0.0295 bit)
"
;
.
t
'
)
(
which
upon
my
""""
He
Mr
—
shall
didn
American
&c
group
later
The
economic
and
She
ve
type
get
Lord
his
problem
thou
thy
1940
1939
problems
big
today
tho
program
It's
ll
back
of
job
Japanese
re
1938
by
Sir
-
workers
New
:
because
got
king
thee
English Fiction V1

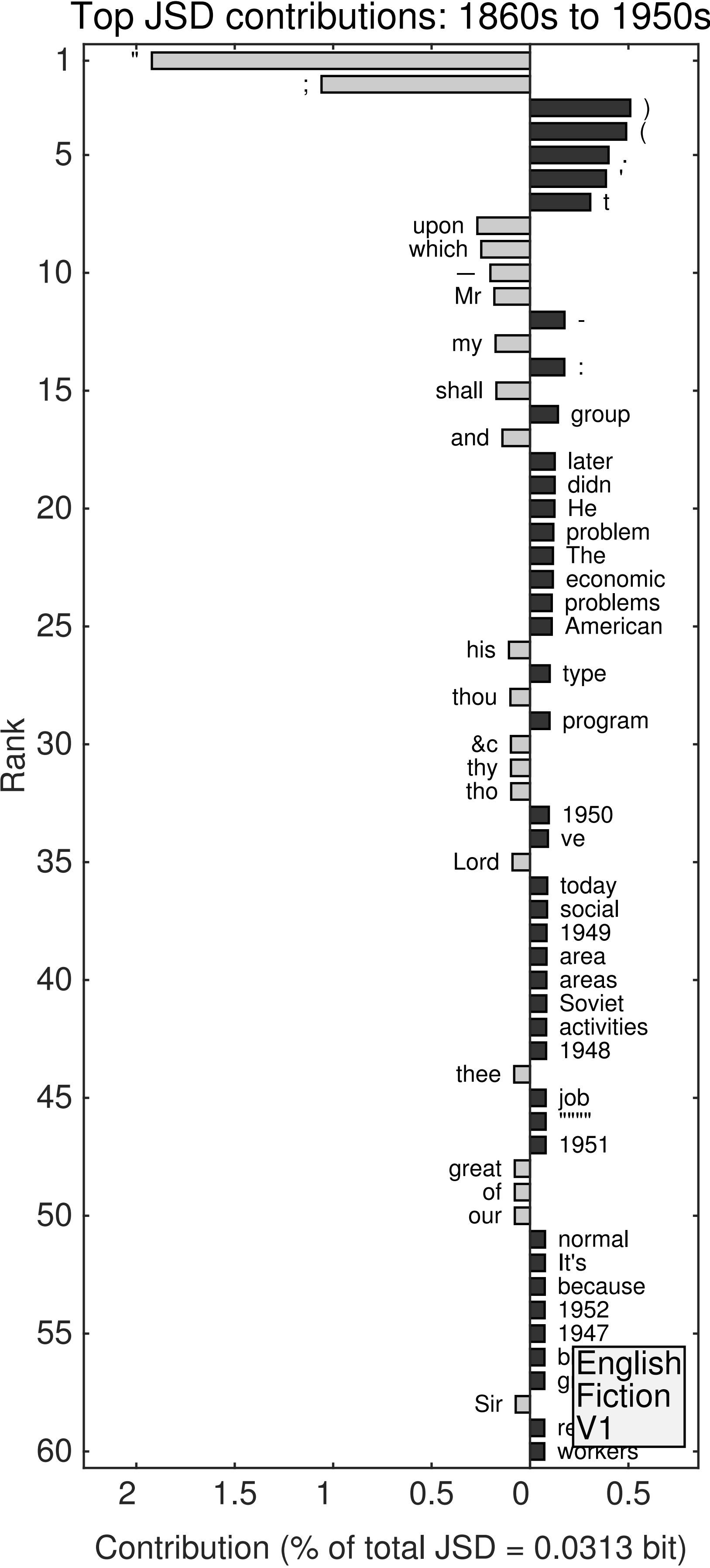
Top JSD contributions: 1860s to 1950s
Rank
Contribution (% of total JSD = 0.0313 bit)
2
1.5
1
0.5
0
0.5
1
5
10
15
20
25
30
35
40
45
50
55
60
"
;
)
(
.
'
t
upon
which
—
Mr
-
my
:
shall
group
and
later
didn
He
problem
The
economic
problems
American
his
type
thou
program
&c
thy
tho
1950
ve
Lord
today
social
1949
area
areas
Soviet
activities
1948
thee
job
1951
great
of
our
normal
It's
because
1952
1947
Sir
workers
English Fiction V1

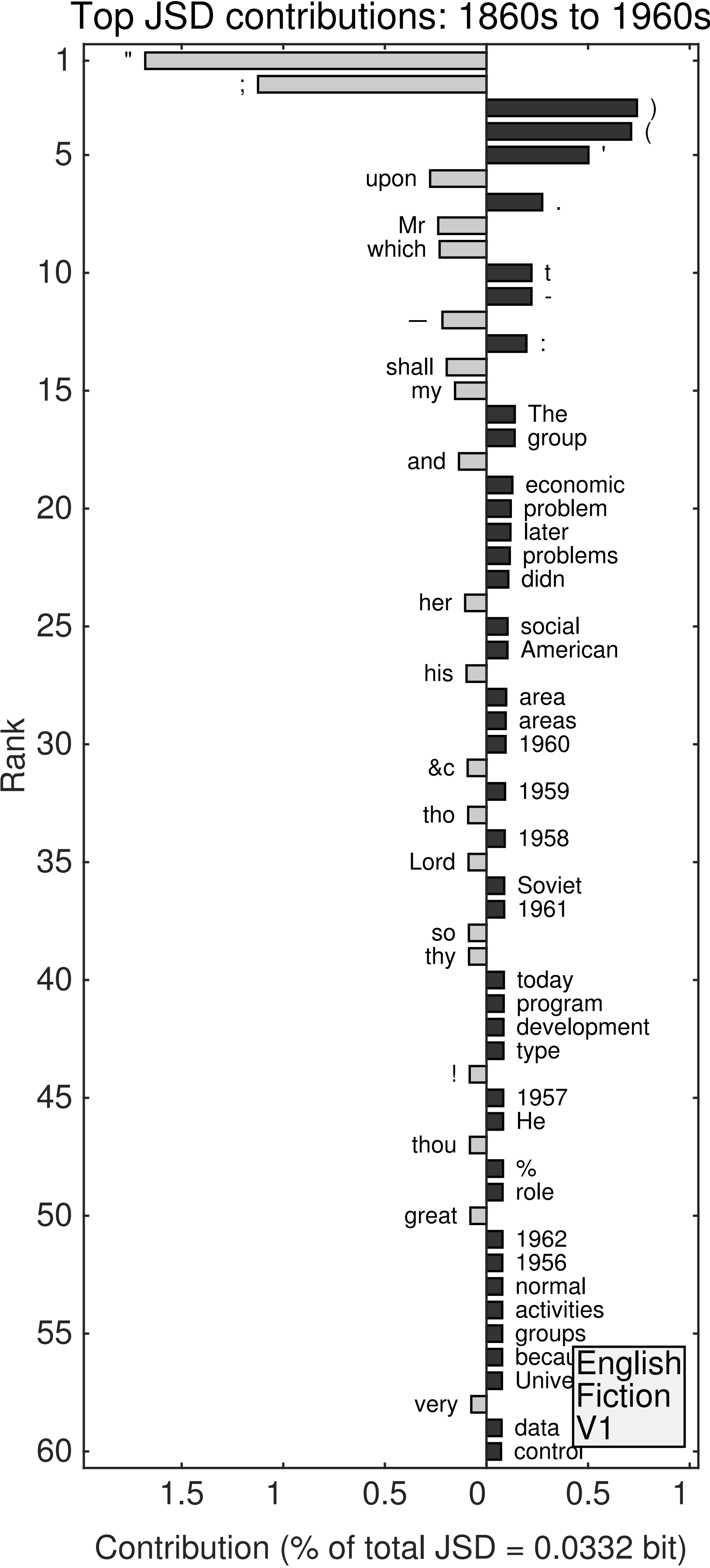
Top JSD contributions: 1860s to 1960s
Rank
Contribution (% of total JSD = 0.0332 bit)
"
;
)
(
'
upon
.
Mr
which
t
-
—
:
shall
my
The
group
and
economic
problem
later
problems
didn
her
social
American
his
area
areas
1960
&c
1959
tho
1958
Lord
Soviet
1961
so
thy
today
program
development
type
!
1957
He
thou
%
role
great
1962
1956
normal
activities
groups
very
data
English
Fiction
V1

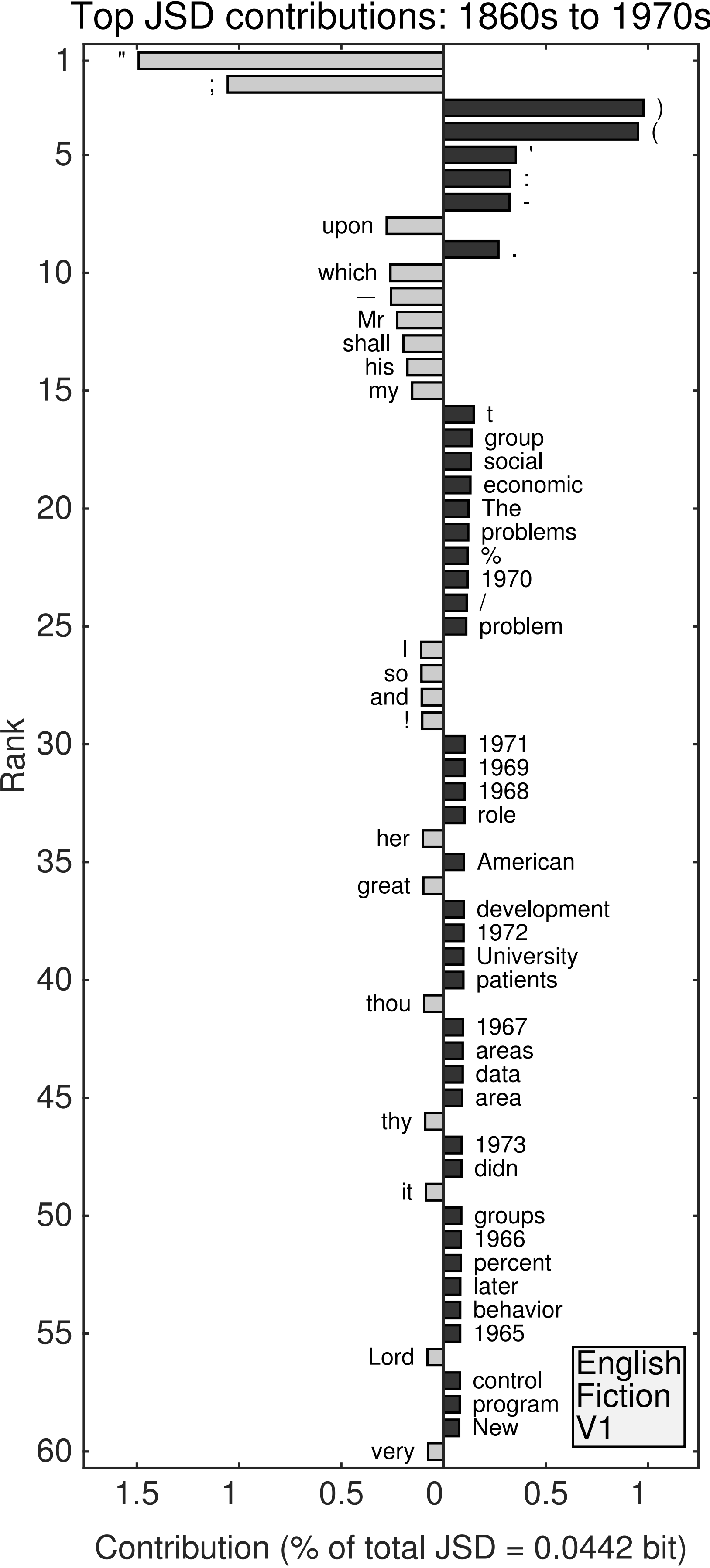
Top JSD contributions: 1860s to 1970s
Rank
Contribution (% of total JSD = 0.0442 bit)
"
;
)
(
'
:
-
upon
.
which
—
Mr
shall
his
my
t
group
social
economic
The
problems
%
1970
/
problem
I
so
and
!
1971
1969
1968
role
her
American
great
development
1972
University
patients
thou
1967
areas
data
area
thy
1973
didn
it
groups
1966
percent
later
behavior
1965
Lord
control
program
New
very
English Fiction V1

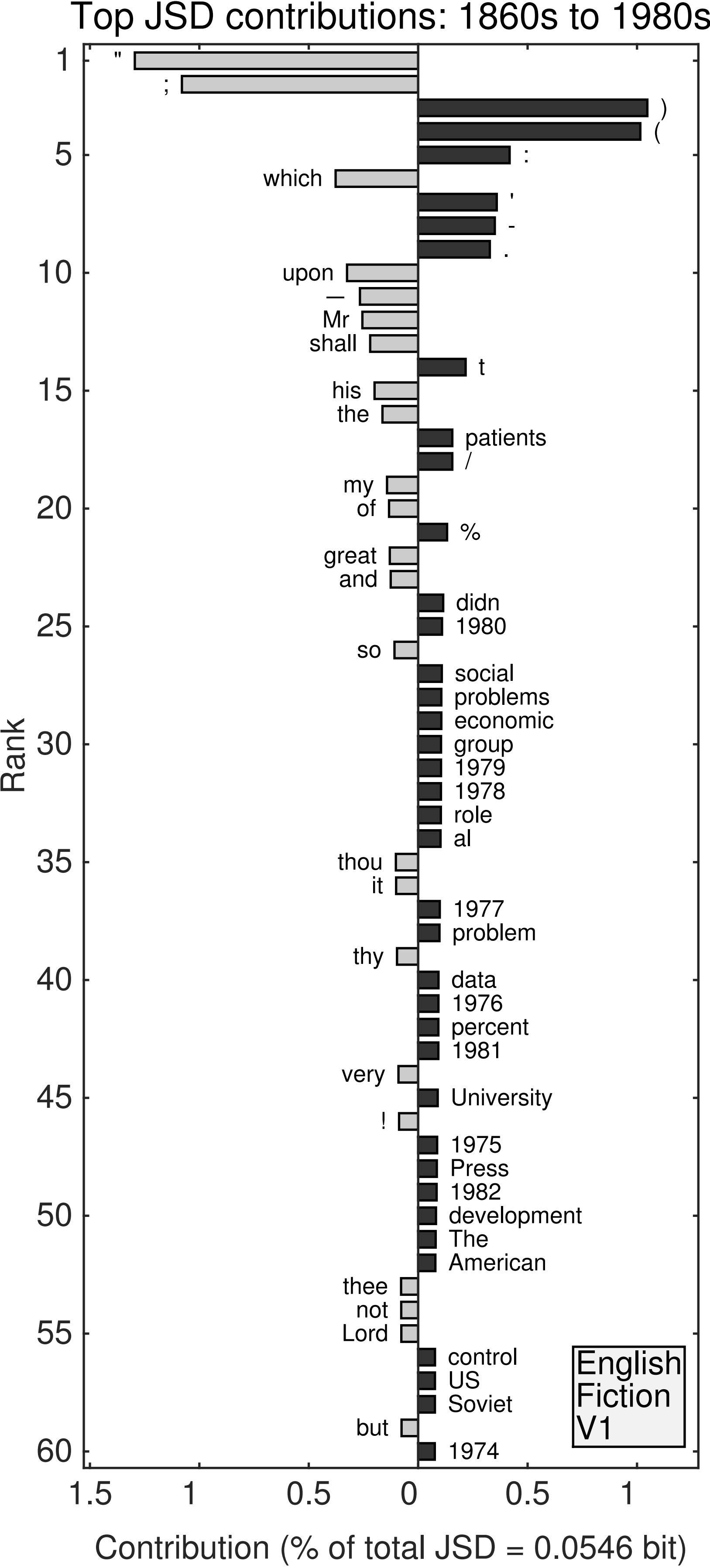

Top JSD contributions: 1860s to 1980s
Rank
Contribution (% of total JSD = 0.0546 bit)
"
;
)
(
:
which
'
-
.
upon
—
Mr
shall
t
his
the
patients
/
my
of
%
great
and
didn
1980
so
social
problems
economic
group
1979
1978
role
al
thou
it
1977
problem
thy
data
1976
percent
1981
very
University
!
1975
Press
1982
development
The
American
thee
not
Lord
control
US
Soviet
but
1974
English Fiction V1

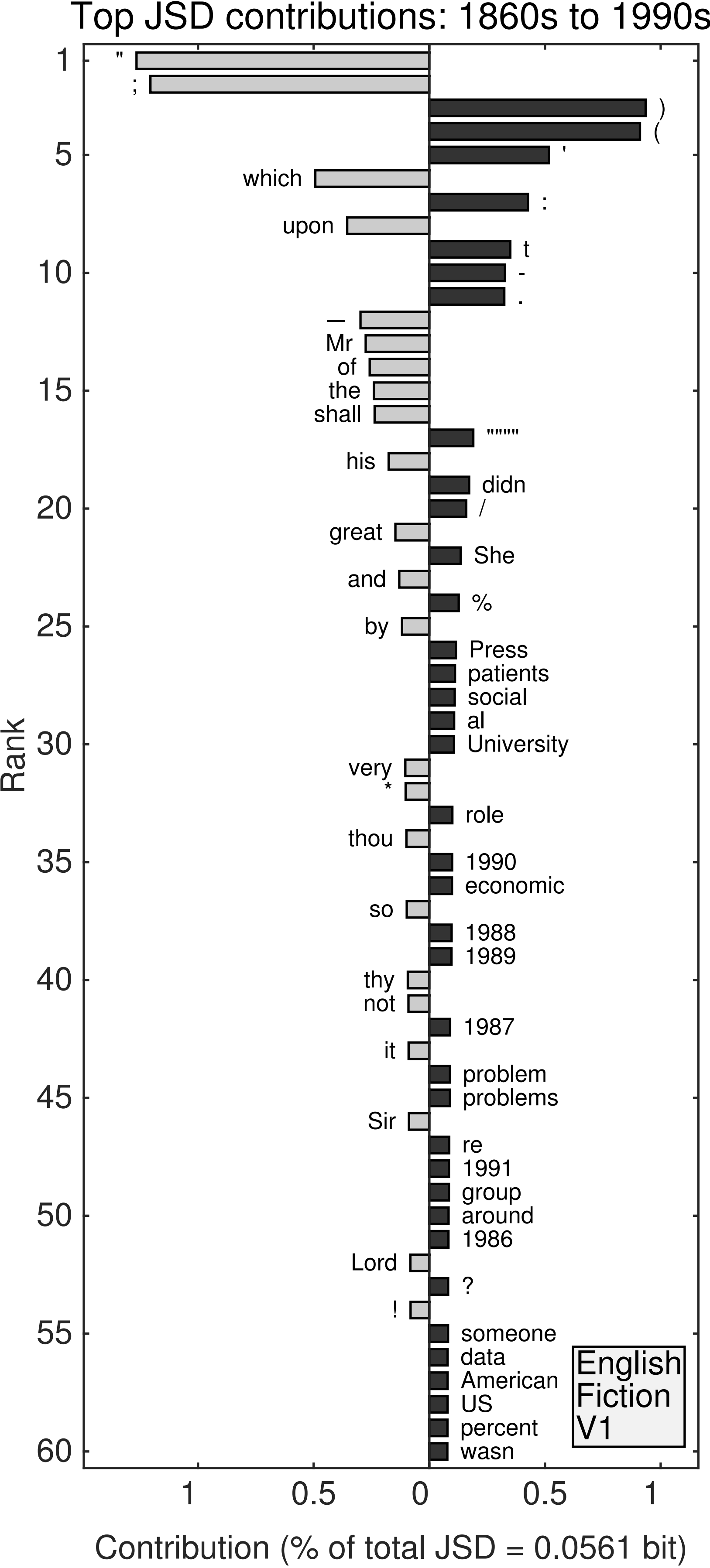
Top JSD contributions: 1860s to 1990s
Rank
Contribution (% of total JSD = 0.0561 bit)
English Fiction V1
1
5
10
15
20
25
30
35
40
45
50
55
60
1
0.5
0
0.5
1
"
;
)
(
'
which
:
upon
t
-
.
—
Mr
of
the
shall
""""
his
didn
/
great
She
and
%
by
Press
patients
social
al
University
very
*
role
thou
1990
economic
so
1988
1989
thy
not
1987
it
problem
problems
Sir
re
1991
group
around
1986
Lord
?
!
someone
data
American
US
percent
wasn

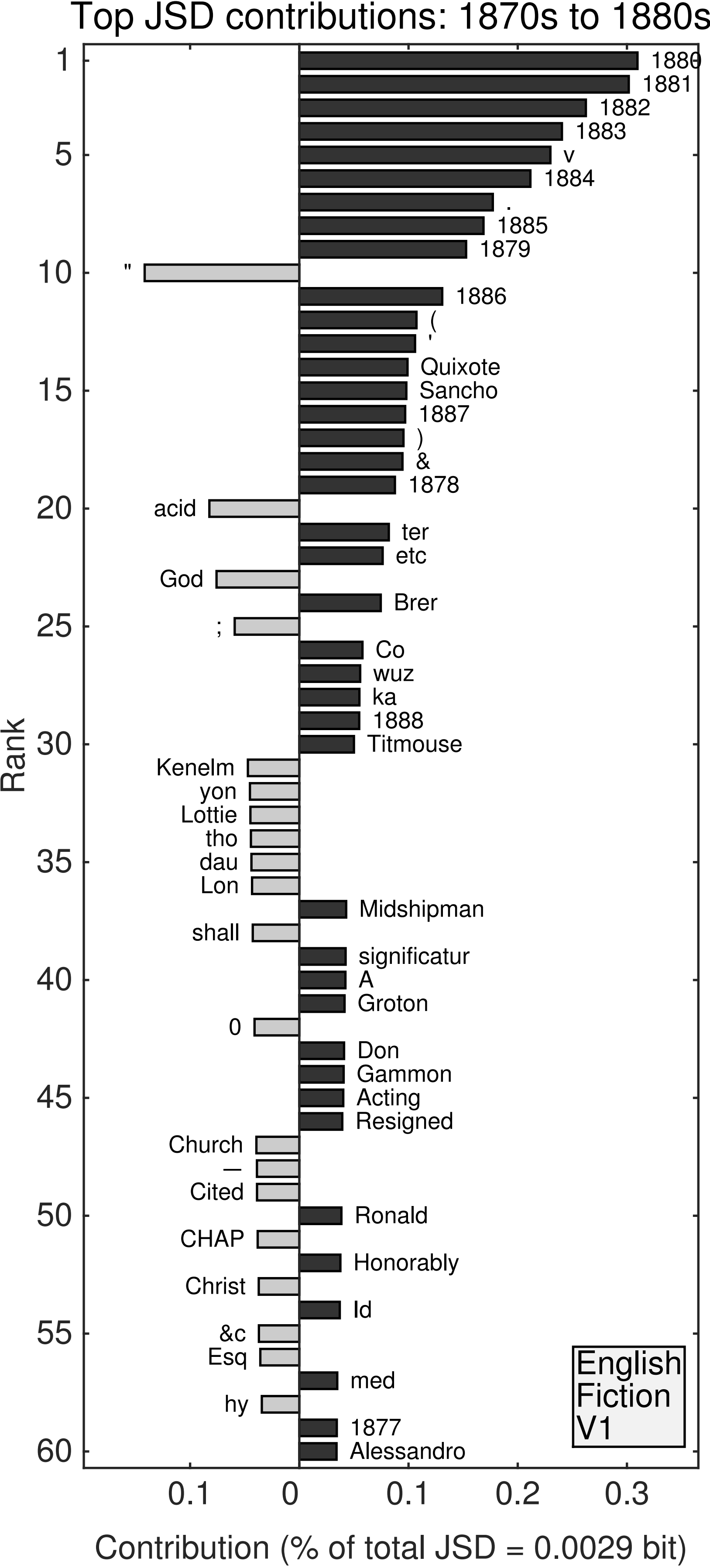

Top JSD contributions: 1870s to 1880s
Rank
Contribution (% of total JSD = 0.0029 bit)
1880
1881
1882
1883
v
1884
.
1885
1879
"
1886
(
'
Quixote
Sancho
1887
)
&
1878
acid
ter
etc
God
Brer
;
Co
wuz
ka
1888
Titmouse
Kenelm
yon
Lottie
tho
dau
Lon
Midshipman
shall
significatur
A
Groton
0
Don
Gammon
Acting
Resigned
Church
—
Cited
Ronald
CHAP
Honorably
Christ
Id
&c
Esq
med
hy
1877
Alessandro
English Fiction V1

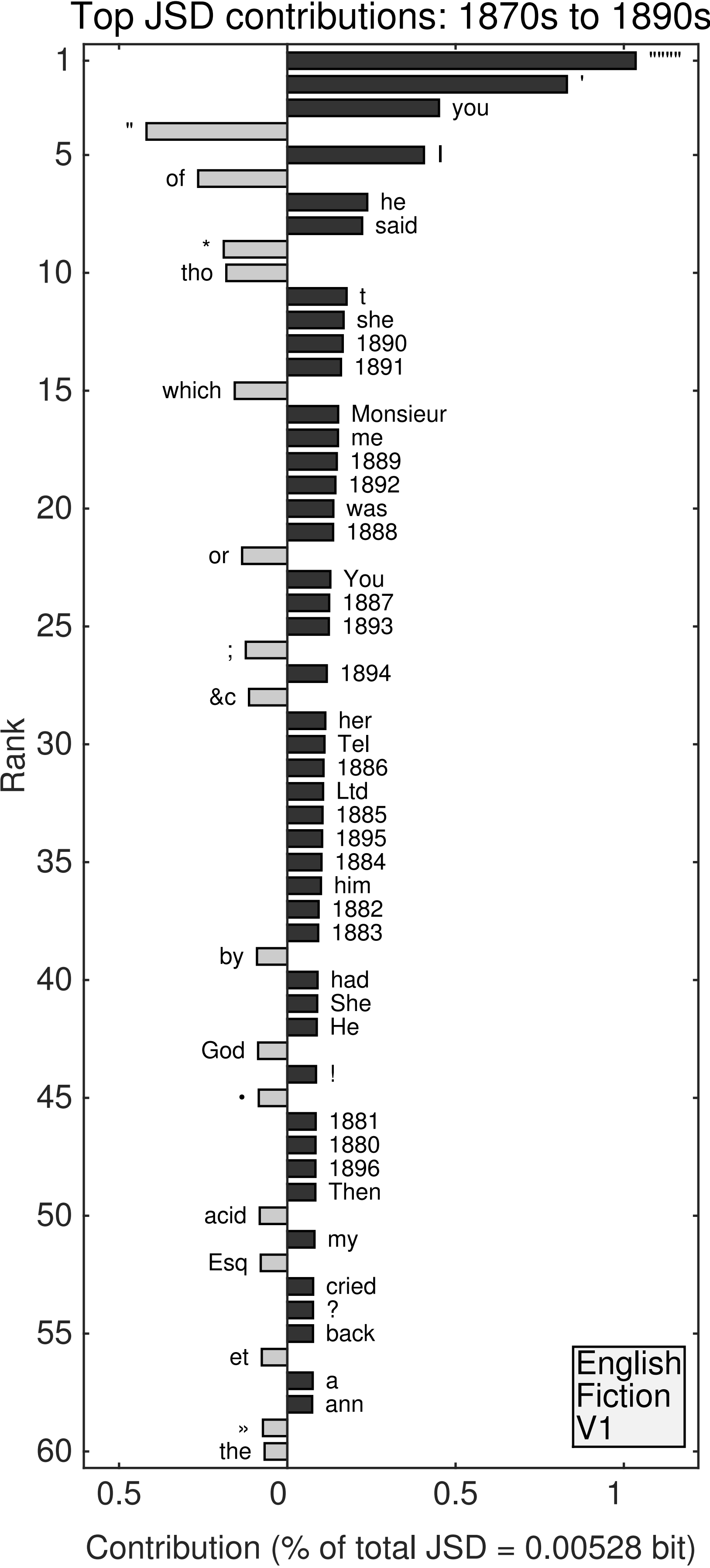
Top JSD contributions: 1870s to 1890s
Rank
Contribution (% of total JSD = 0.00528 bit)
""""
'
you
"
I
of
he
said
*
tho
t
she
1890
1891
which
Monsieur
me
1889
1892
was
1888
or
You
1887
1893
;
1894
&c
her
Tel
1886
Ltd
1885
1895
1884
him
1882
1883
by
had
She
He
God
!
•
1881
1880
1896
Then
acid
my
Esq
cried
?
back
et
a
ann
»
the
English Fiction V1

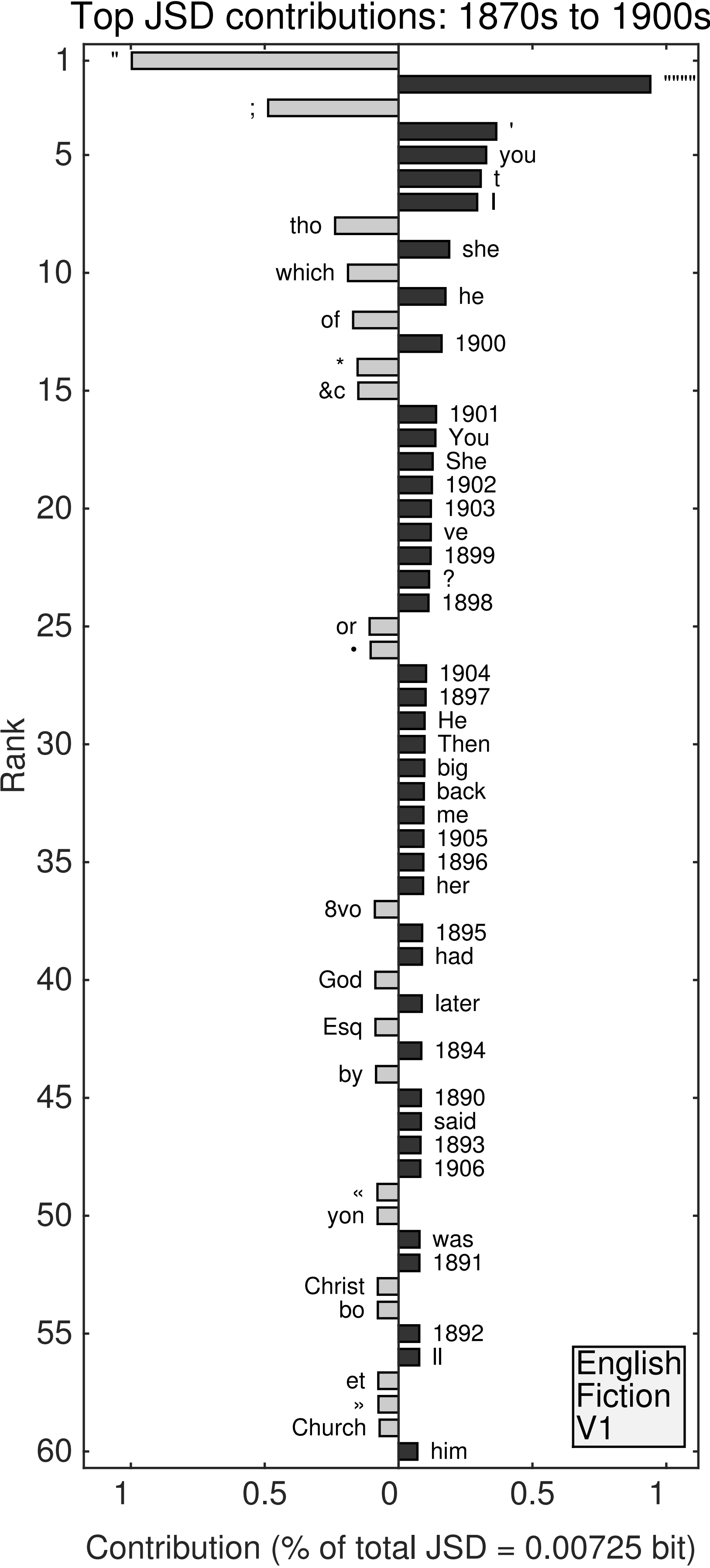

Top JSD contributions: 1870s to 1900s
Rank
Contribution (% of total JSD = 0.00725 bit)
English Fiction V1
"
""""
;
'
you
t
I
tho
she
which
he
of
1900
*
&c
1901
You
She
1902
1903
ve
1899
?
1898
or
•
1904
1897
He
Then
big
back
me
1905
1896
her
8vo
1895
had
God
later
Esq
1894
by
1890
said
1893
1906
«
yon
was
1891
Christ
bo
1892
ll
et
»
Church
him

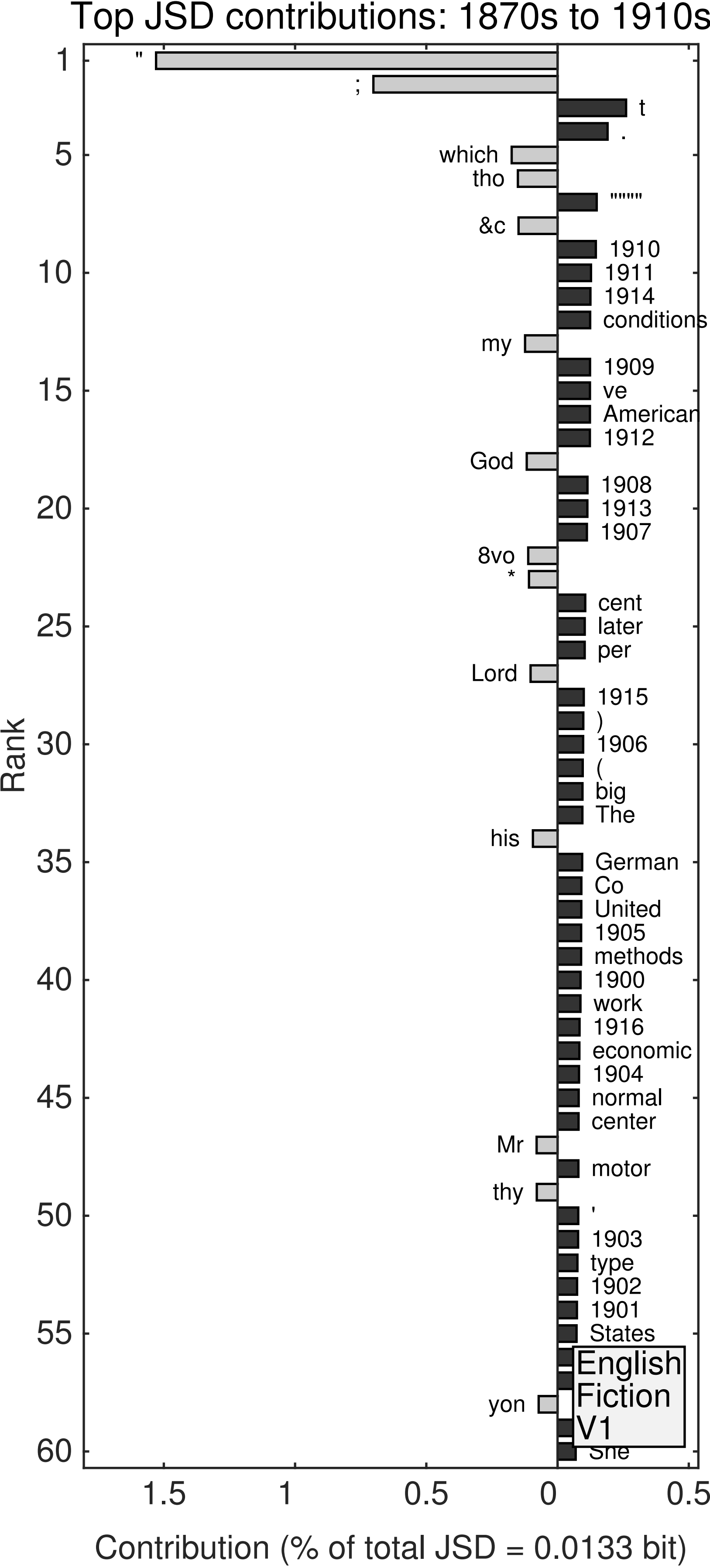

Top JSD contributions: 1870s to 1910s
"
;
t
.
which
tho
""""
&c
1910
1911
1914
conditions
my
1909
ve
American
1912
God
1908
1913
1907
8vo
*
cent
later
per
Lord
1915
)
1906
(
big
The
his
German
Co
United
1905
methods
1900
work
1916
economic
1904
normal
center
Mr
motor
thy
'
1903
type
1902
1901
States
yon
She
English Fiction V1
Rank
Contribution (% of total JSD = 0.0133 bit)

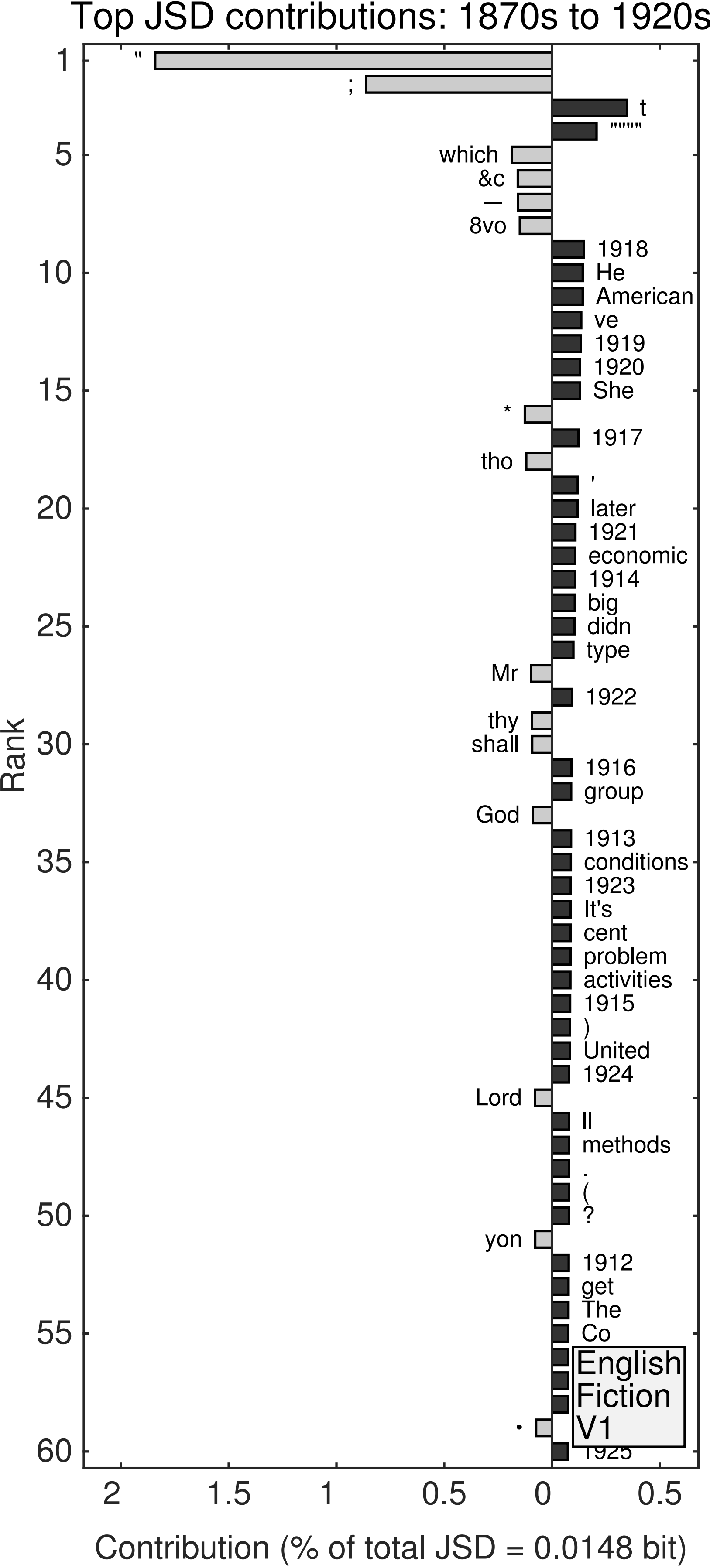

Top JSD contributions: 1870s to 1920s
Rank
"
;
t
""""
which
&c
—
8vo
1918
He
American
ve
1919
1920
She
*
1917
tho
'
later
1921
economic
1914
big
didn
type
Mr
1922
thy
shall
1916
group
God
1913
conditions
1923
It's
cent
problem
activities
1915
)
United
1924
Lord
ll
methods
.
(
?
yon
1912
get
The
Co
•
1925
English Fiction V1
1
5
10
15
20
25
30
35
40
45
50
55
60
2
1.5
1
0.5
0
0.5
Contribution (% of total JSD = 0.0148 bit)

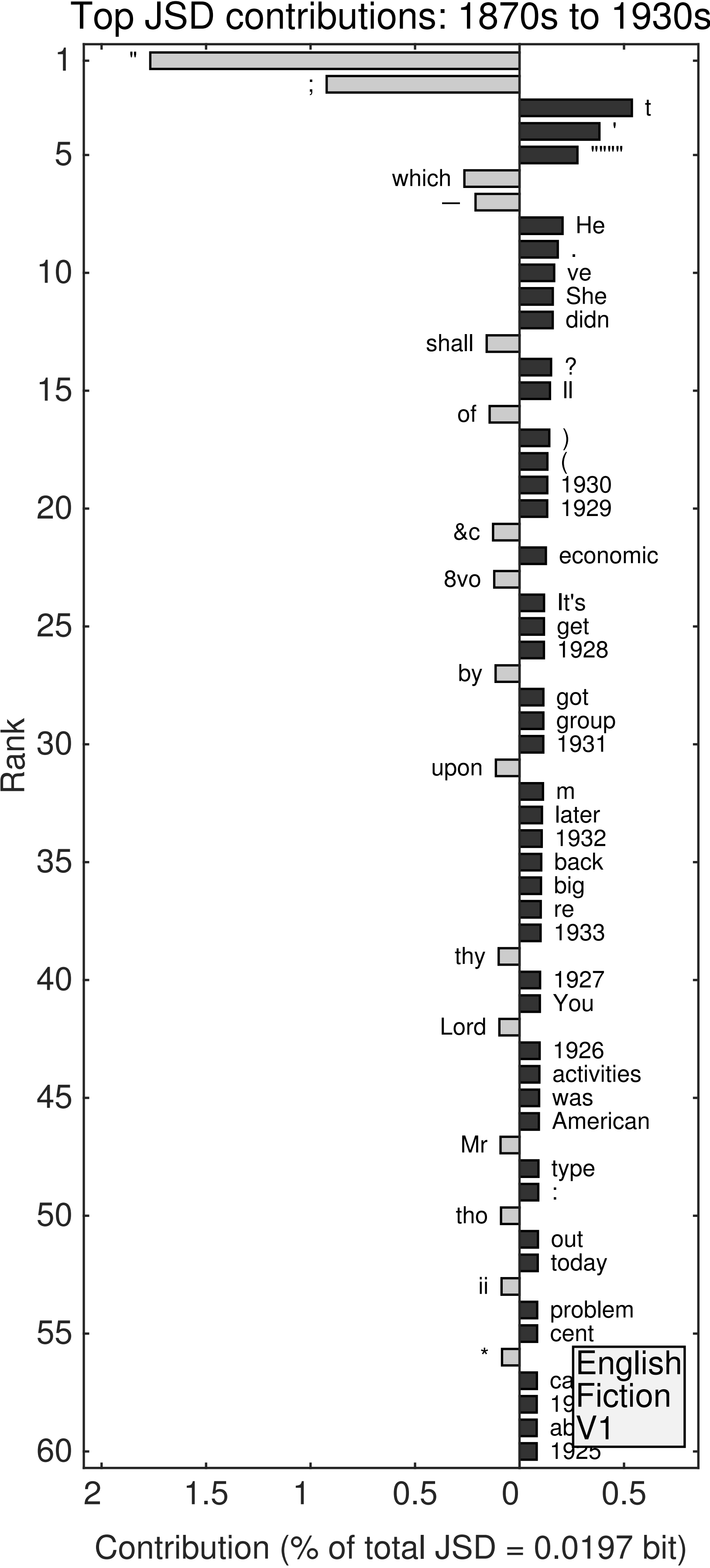

Top JSD contributions: 1870s to 1930s
Rank
Contribution (% of total JSD = 0.0197 bit)
"
;
t
'
""""
which
—
He
.
ve
She
didn
shall
?
ll
of
)
(
1930
1929
&c
economic
8vo
It's
get
1928
by
got
group
1931
upon
m
later
1932
back
big
re
1933
thy
1927
You
Lord
1926
activities
was
American
Mr
type
:
tho
out
today
ii
problem
cent
*
1925
English
Fiction
V1

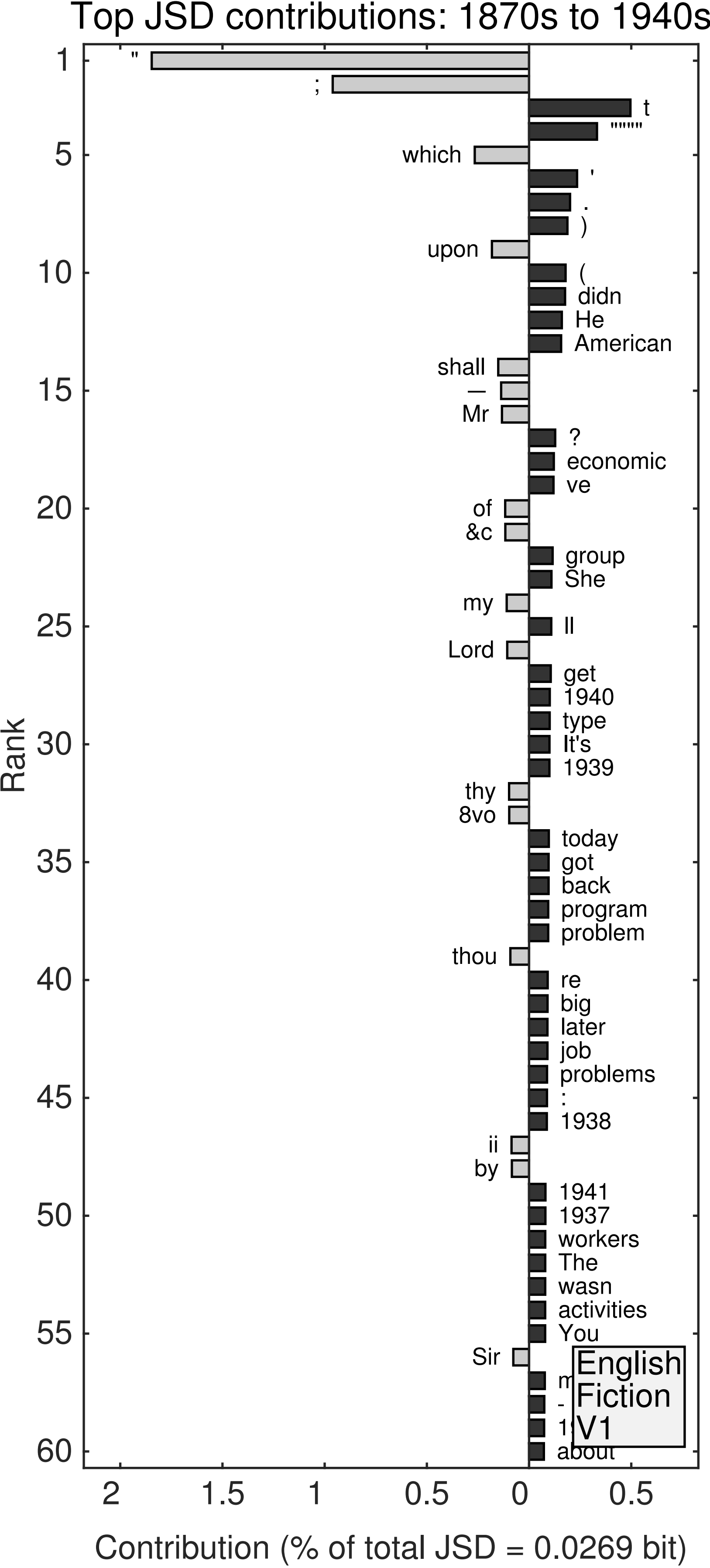
Top JSD contributions: 1870s to 1940s
Rank
Contribution (% of total JSD = 0.0269 bit)
2
1.5
1
0.5
0
0.5
1
5
10
15
20
25
30
35
40
45
50
55
60
"
;
t
""""
which
'
.
)
upon
(
didn
He
American
shall
—
Mr
?
economic
ve
of
&c
group
She
my
ll
Lord
get
1940
type
It's
1939
thy
8vo
today
got
back
program
problem
thou
re
big
later
job
problems
:
1938
ii
by
1941
1937
workers
The
wasn
activities
You
Sir
-
about
English
Fiction
V1

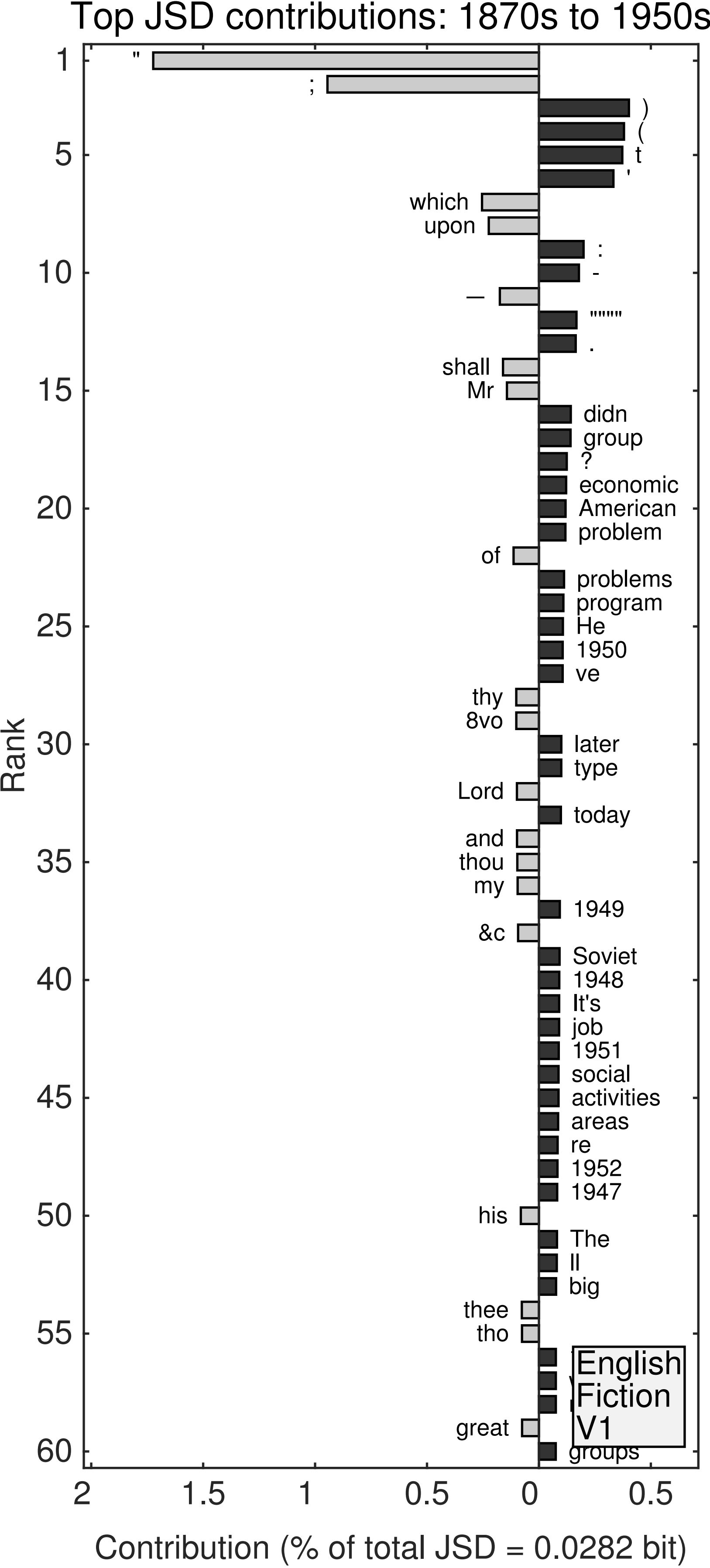

Top JSD contributions: 1870s to 1950s
Rank
Contribution (% of total JSD = 0.0282 bit)
2
1.5
1
0.5
0
0.5
1
5
10
15
20
25
30
35
40
45
50
55
60
"
;
)
(
t
'
which
upon
:
-
—
""""
.
shall
Mr
didn
group
?
economic
American
problem
of
problems
program
He
1950
ve
thy
8vo
later
type
Lord
today
and
thou
my
1949
&c
Soviet
1948
It's
job
1951
social
activities
areas
re
1952
1947
his
The
ll
big
thee
tho
great
groups
English
Fiction
V1

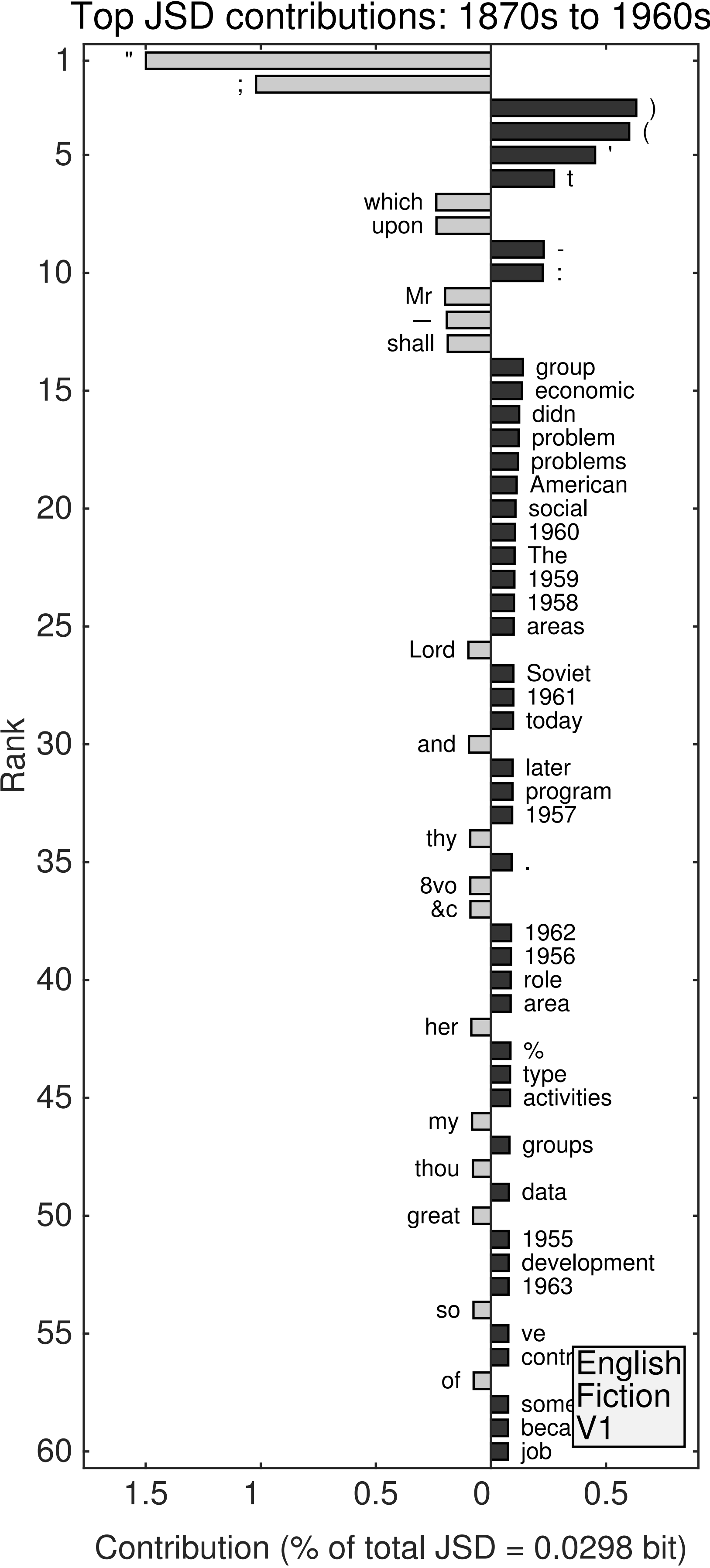
Top JSD contributions: 1870s to 1960s
Rank
"
;
)
(
'
t
which
upon
-
:
Mr
—
shall
group
economic
didn
problem
problems
American
social
1960
The
1959
1958
areas
Lord
Soviet
1961
today
and
later
program
1957
thy
.
8vo
&c
1962
1956
role
area
her
%
type
activities
my
groups
thou
data
great
1955
development
1963
so
ve
of
job
English
Fiction
V1
Contribution (% of total JSD = 0.0298 bit)

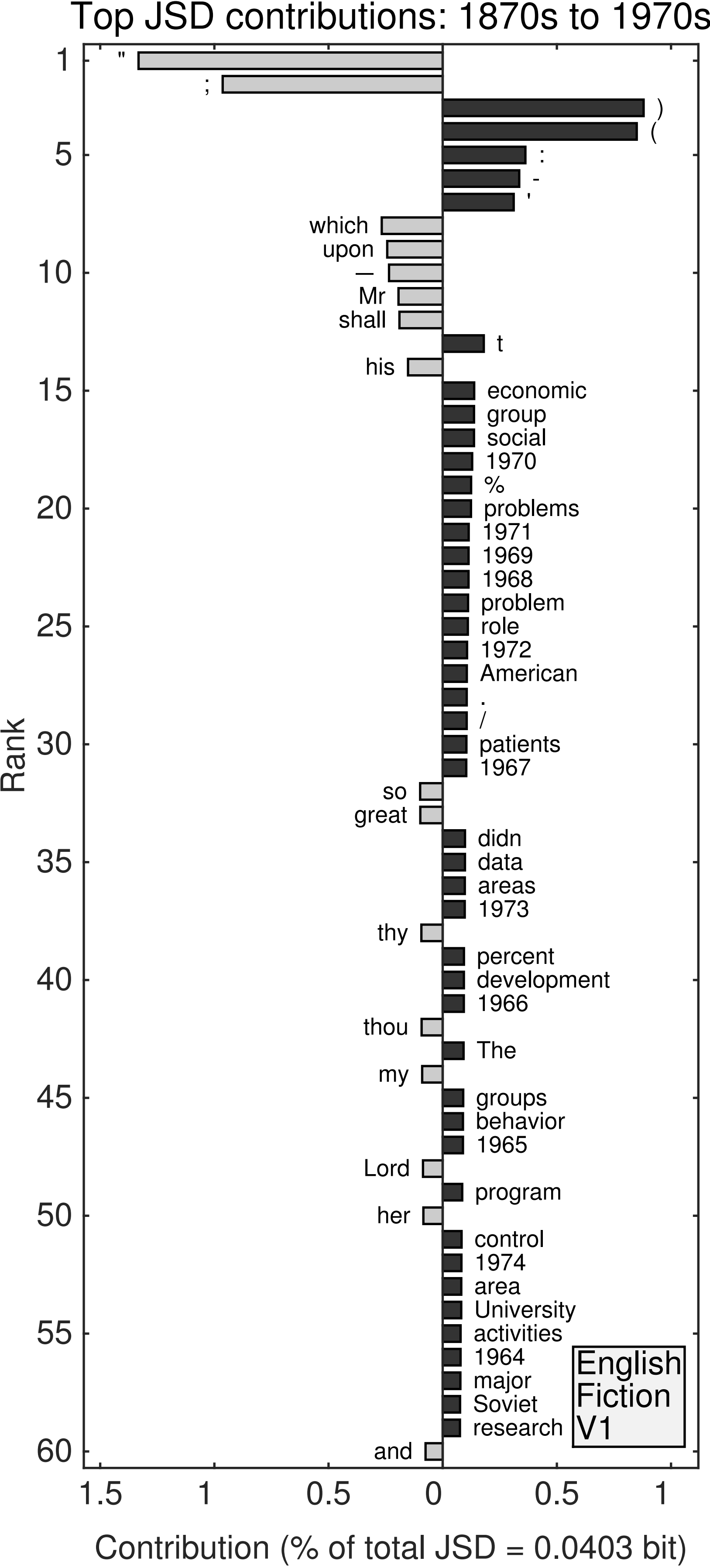

Top JSD contributions: 1870s to 1970s
Rank
"
;
)
(
:
-
'
which
upon
—
Mr
shall
t
his
economic
group
social
1970
%
problems
1971
1969
1968
problem
role
1972
American
.
/
patients
1967
so
great
didn
data
areas
1973
thy
percent
development
1966
thou
The
my
groups
behavior
1965
Lord
program
her
control
1974
area
University
activities
1964
major
Soviet
research
and
English Fiction V1
Contribution (% of total JSD = 0.0403 bit)

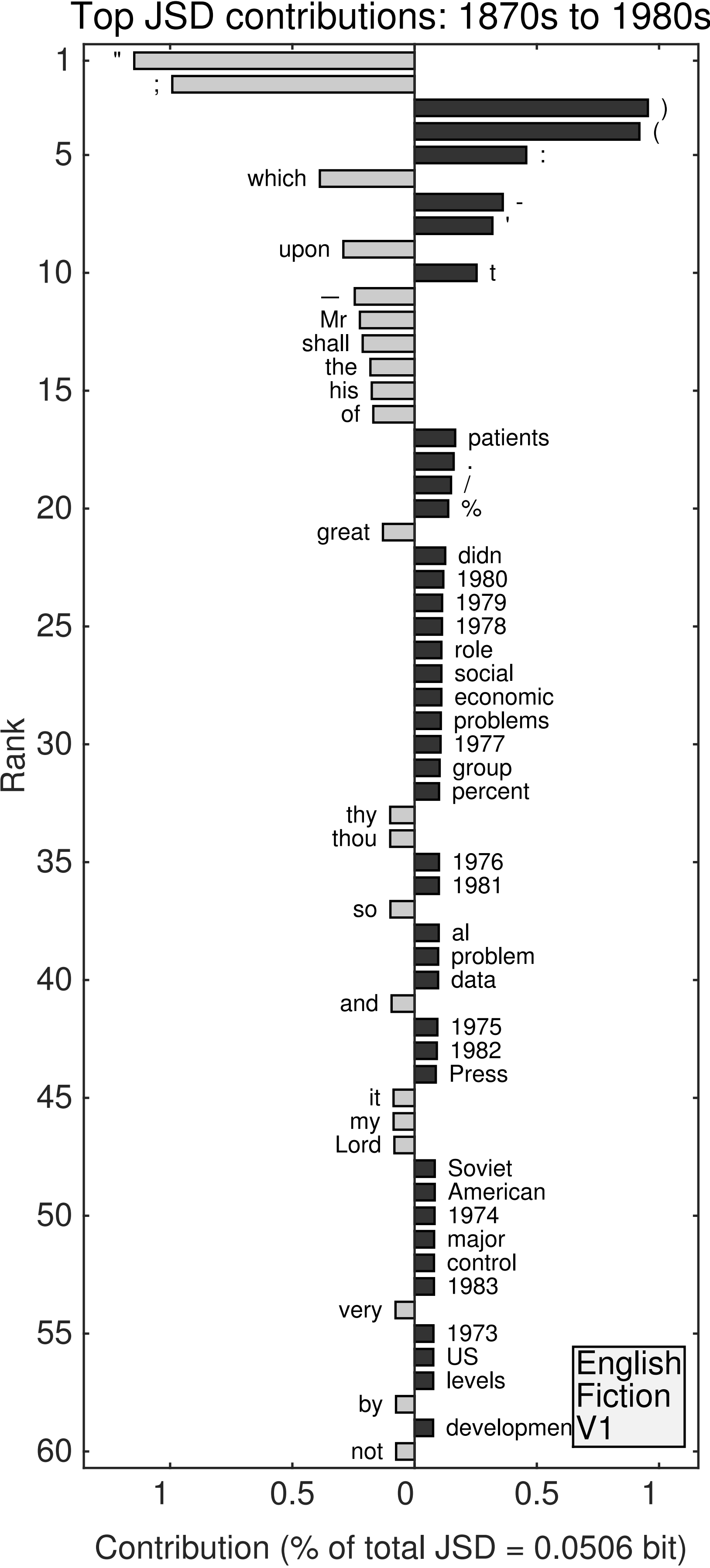
Top JSD contributions: 1870s to 1980s
Rank
Contribution (% of total JSD = 0.0506 bit)
English Fiction V1
"
;
)
(
:
which
-
'
upon
t
—
Mr
shall
the
his
of
patients
.
/
%
great
didn
1980
1979
1978
role
social
economic
problems
1977
group
percent
thy
thou
1976
1981
so
al
problem
data
and
1975
1982
Press
it
my
Lord
Soviet
American
1974
major
control
1983
very
1973
US
levels
by
developmen
not

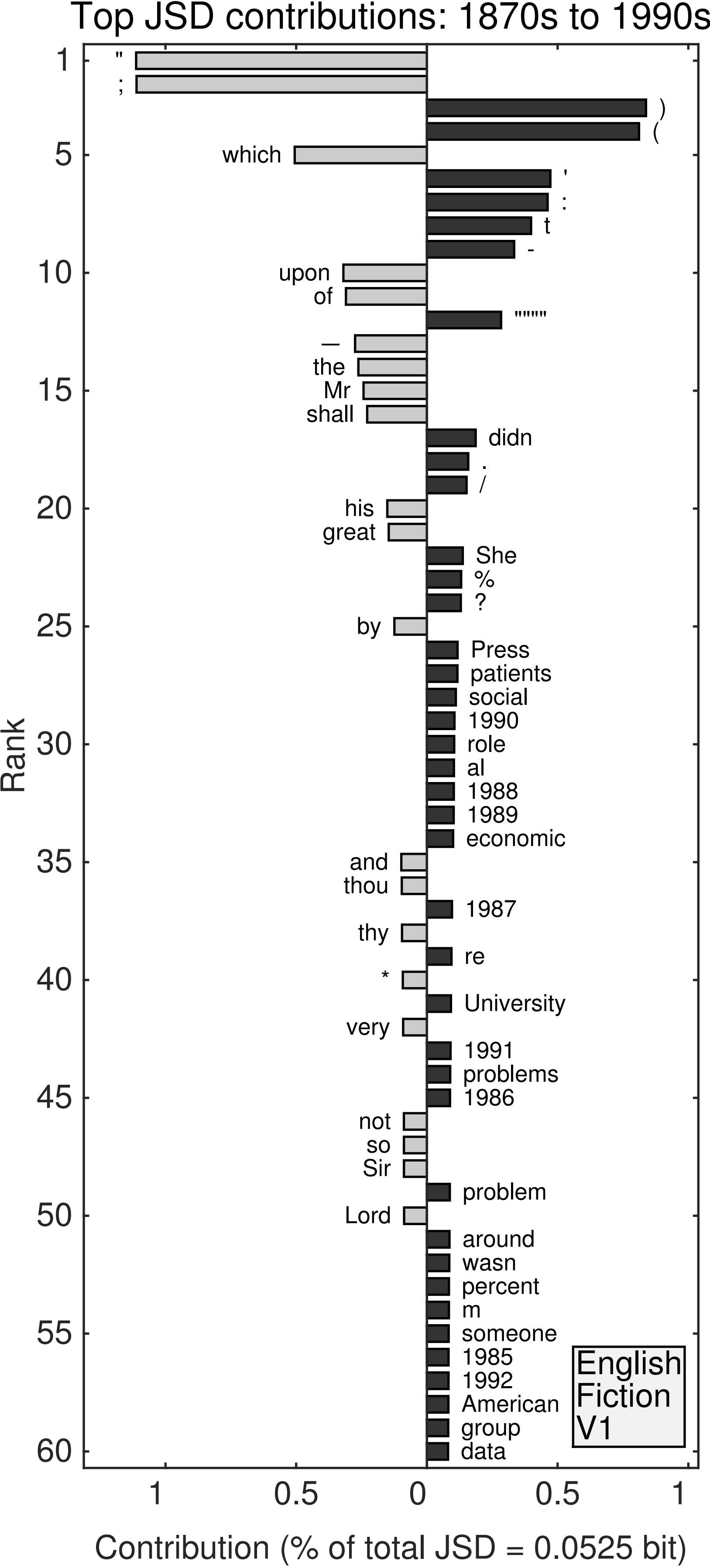

Top JSD contributions: 1870s to 1990s
Rank
Contribution (% of total JSD = 0.0525 bit)
English Fiction V1
"
;
)
(
which
'
:
t
-
upon
of
""""
—
the
Mr
shall
didn
.
/
his
great
She
%
?
by
Press
patients
social
1990
role
al
1988
1989
economic
and
thou
1987
thy
re
*
University
very
1991
problems
1986
not
so
Sir
problem
Lord
around
wasn
percent
m
someone
1985
1992
American
group
data
1
5
10
15
20
25
30
35
40
45
50
55
60
1
0.5
0
0.5
1

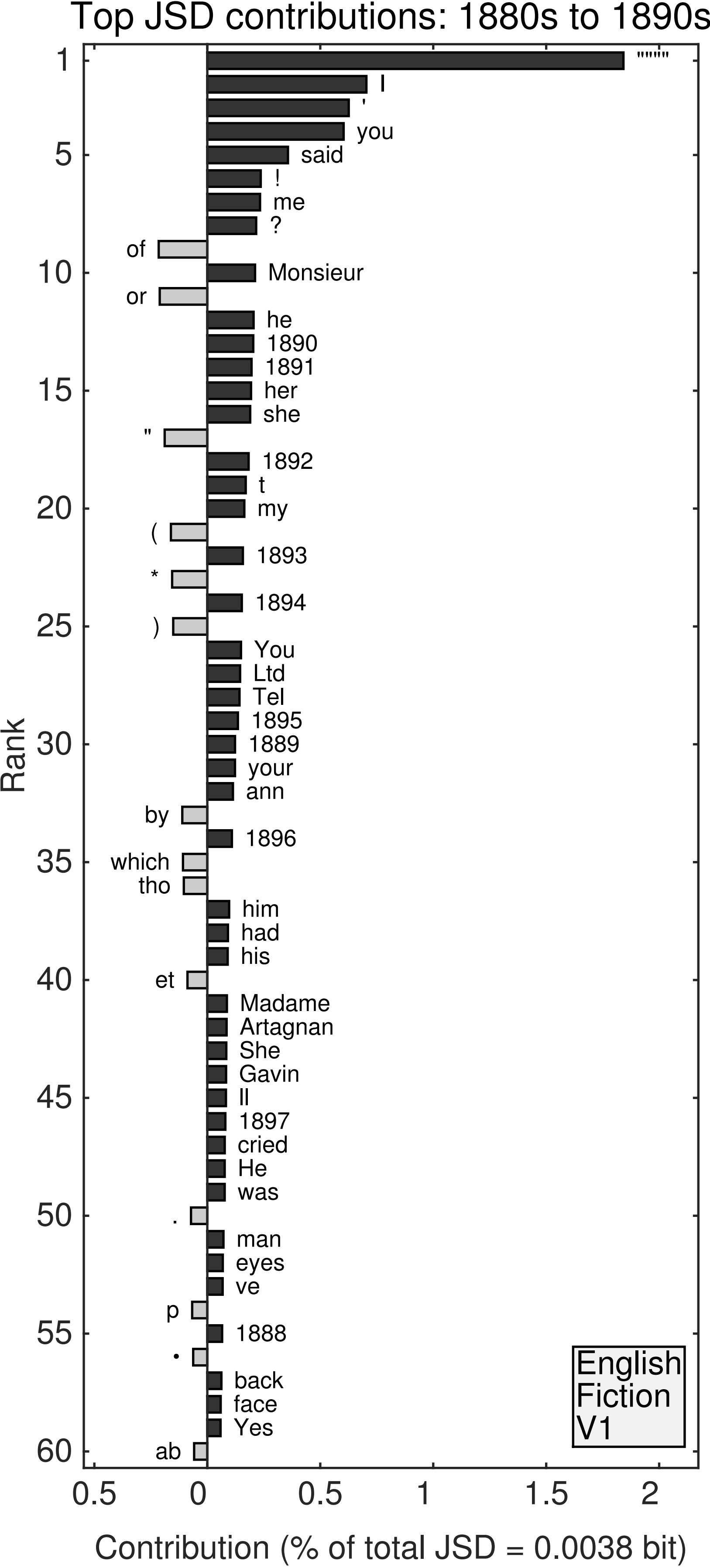

Top JSD contributions: 1880s to 1890s
Rank
Contribution (% of total JSD = 0.0038 bit)
English Fiction V1
"""""
I
'
you
said
!
me
?
of
Monsieur
or
he
1890
1891
her
she
"
1892
t
my
(
1893
*
1894
)
You
Ltd
Tel
1895
1889
your
ann
by
1896
which
tho
him
had
his
et
Madame
Artagnan
She
Gavin
ll
1897
cried
He
was
.
man
eyes
ve
p
1888
•
back
face
Yes
ab

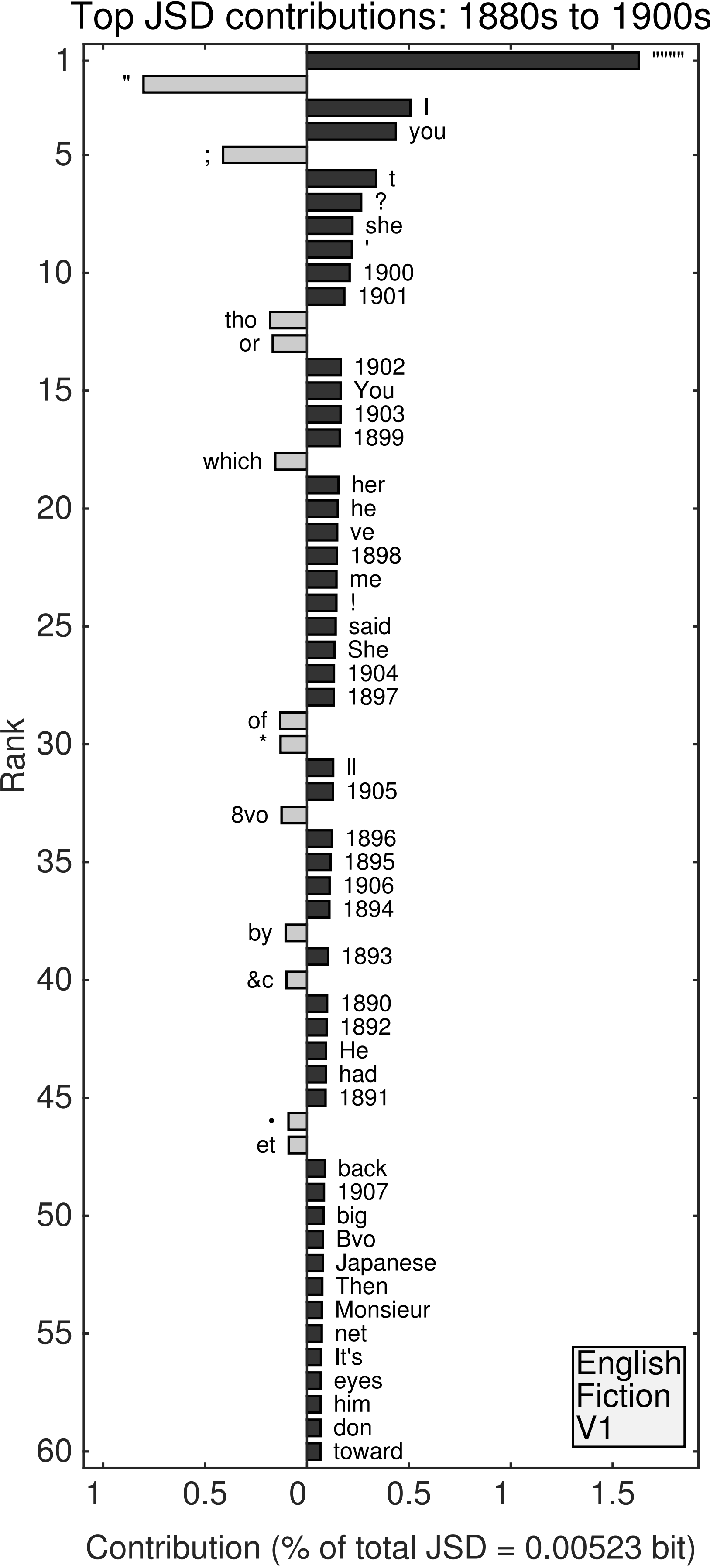

Top JSD contributions: 1880s to 1900s
Rank
Contribution (% of total JSD = 0.00523 bit)
1
5
10
15
20
25
30
35
40
45
50
55
60
1
0.5
0
0.5
1
1.5
""""
"
I
you
;
t
?
she
'
1900
1901
tho
or
1902
You
1903
1899
which
her
he
ve
1898
me
!
said
She
1904
1897
of
*
ll
1905
8vo
1896
1895
1906
1894
by
1893
&c
1890
1892
He
had
1891
•
et
back
1907
big
Bvo
Japanese
Then
Monsieur
net
It's
eyes
him
don
toward
English
Fiction
V1

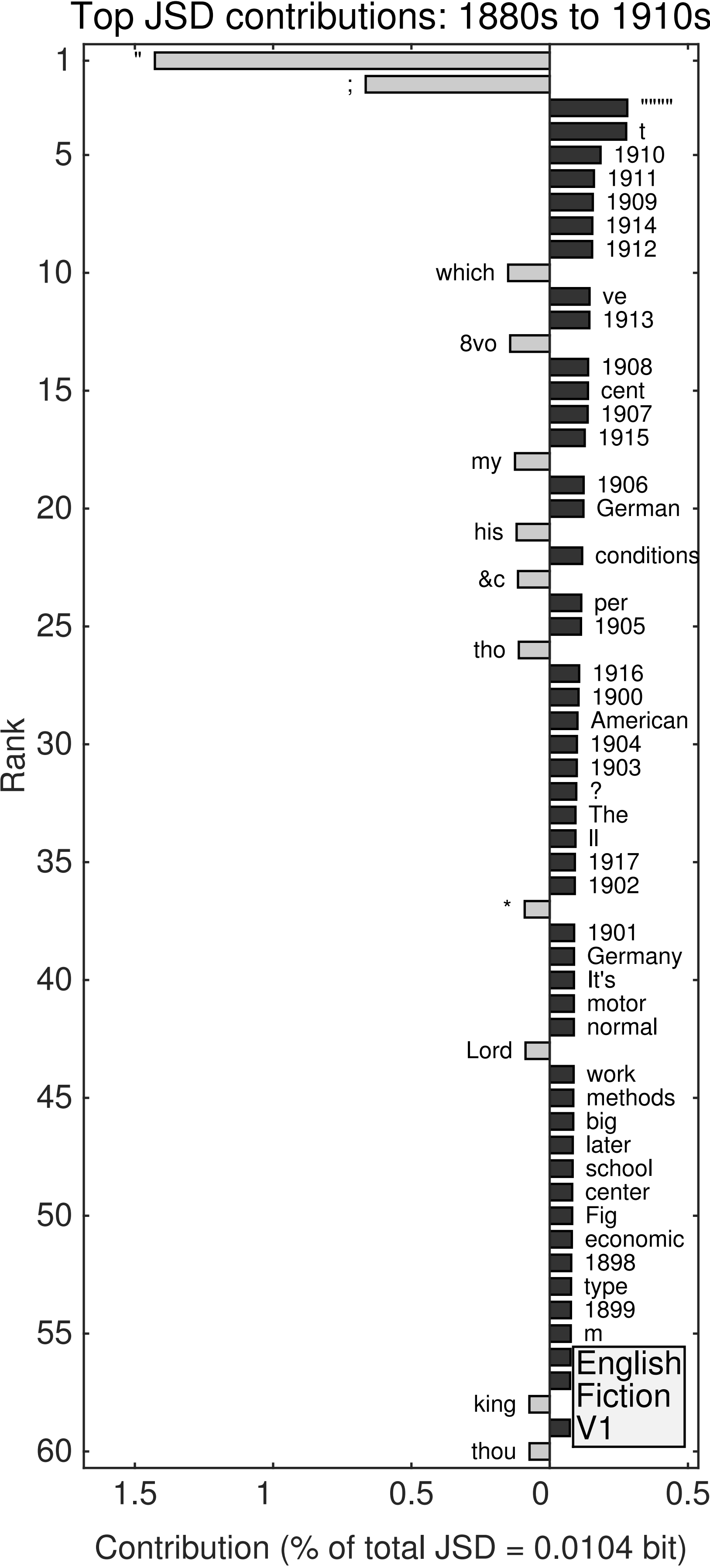

Top JSD contributions: 1880s to 1910s
Rank
Contribution (% of total JSD = 0.0104 bit)
"
;
""""
t
1910
1911
1909
1914
1912
which
ve
1913
8vo
1908
cent
1907
1915
my
1906
German
his
conditions
&c
per
1905
tho
1916
1900
American
1904
1903
?
The
ll
1917
1902
*
1901
Germany
It's
motor
normal
Lord
work
methods
big
later
school
center
Fig
economic
1898
type
1899
m
king
thou
English Fiction V1

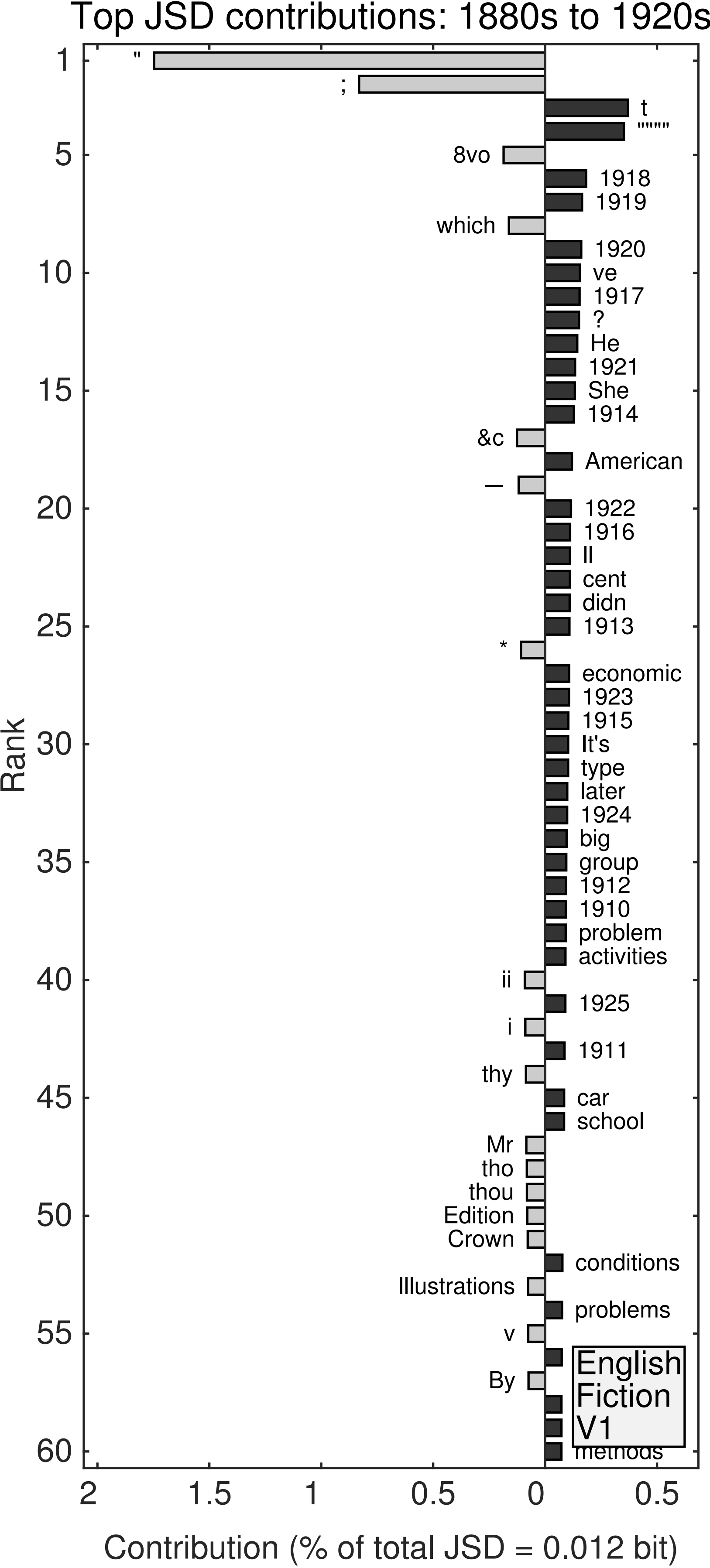

Top JSD contributions: 1880s to 1920s
Rank
Contribution (% of total JSD = 0.012 bit)
2
1.5
1
0.5
0
0.5
1
5
10
15
20
25
30
35
40
45
50
55
60
"
;
t
""""
8vo
1918
1919
which
1920
ve
1917
?
He
1921
She
1914
&c
American
—
1922
1916
ll
cent
didn
1913
*
economic
1923
1915
It's
type
later
1924
big
group
1912
1910
problem
activities
ii
1925
i
1911
thy
car
school
Mr
tho
thou
Edition
Crown
conditions
Illustrations
problems
v
By
methods
English
Fiction
V1

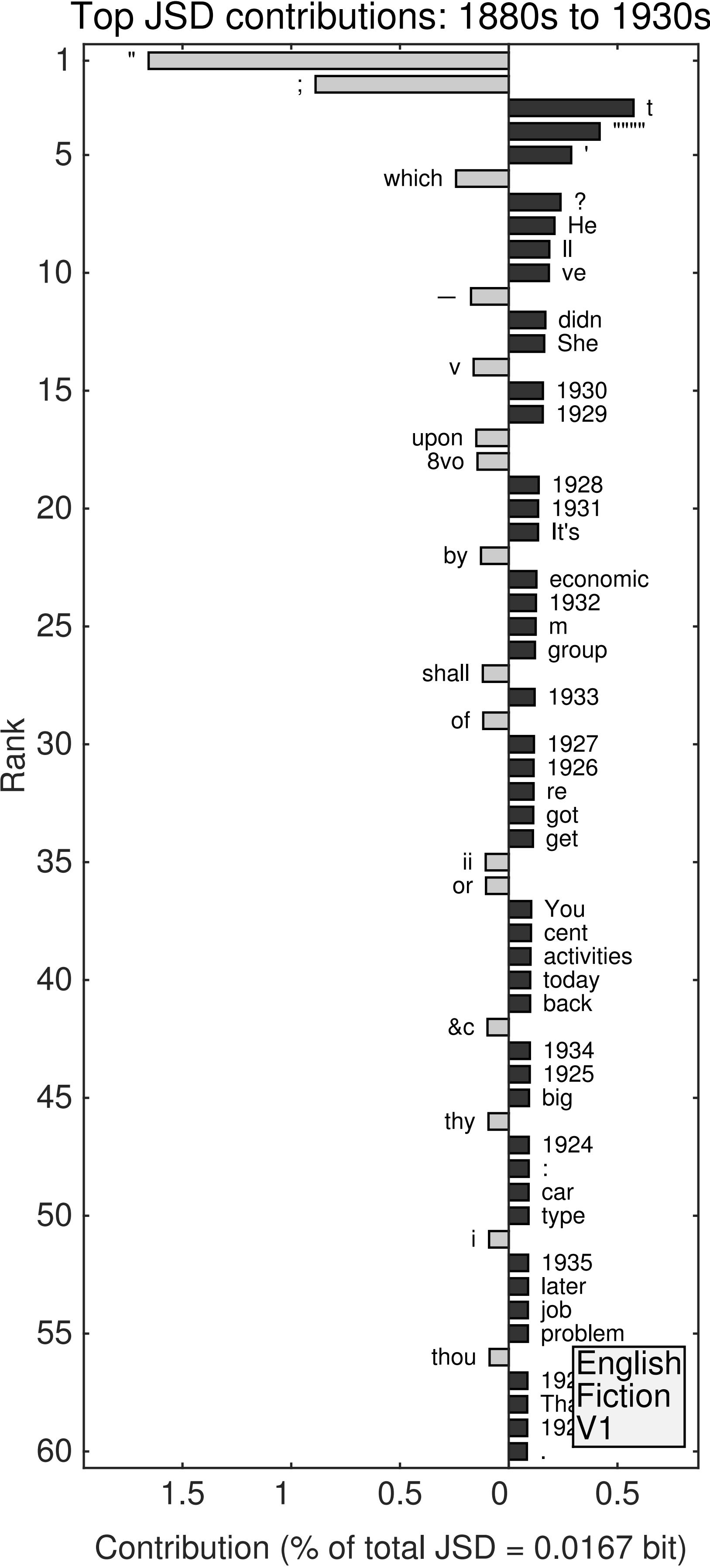
Top JSD contributions: 1880s to 1930s
Rank
"
;
t
"""""
'
which
?
He
ll
ve
—
didn
She
v
1930
1929
upon
8vo
1928
1931
It's
by
economic
1932
m
group
shall
1933
of
1927
1926
re
got
get
ii
or
You
cent
activities
today
back
&c
1934
1925
big
thy
1924
:
car
type
i
1935
later
job
problem
thou
.
English
Fiction
V1
1.5
1
0.5
0
0.5
Contribution (% of total JSD = 0.0167 bit)

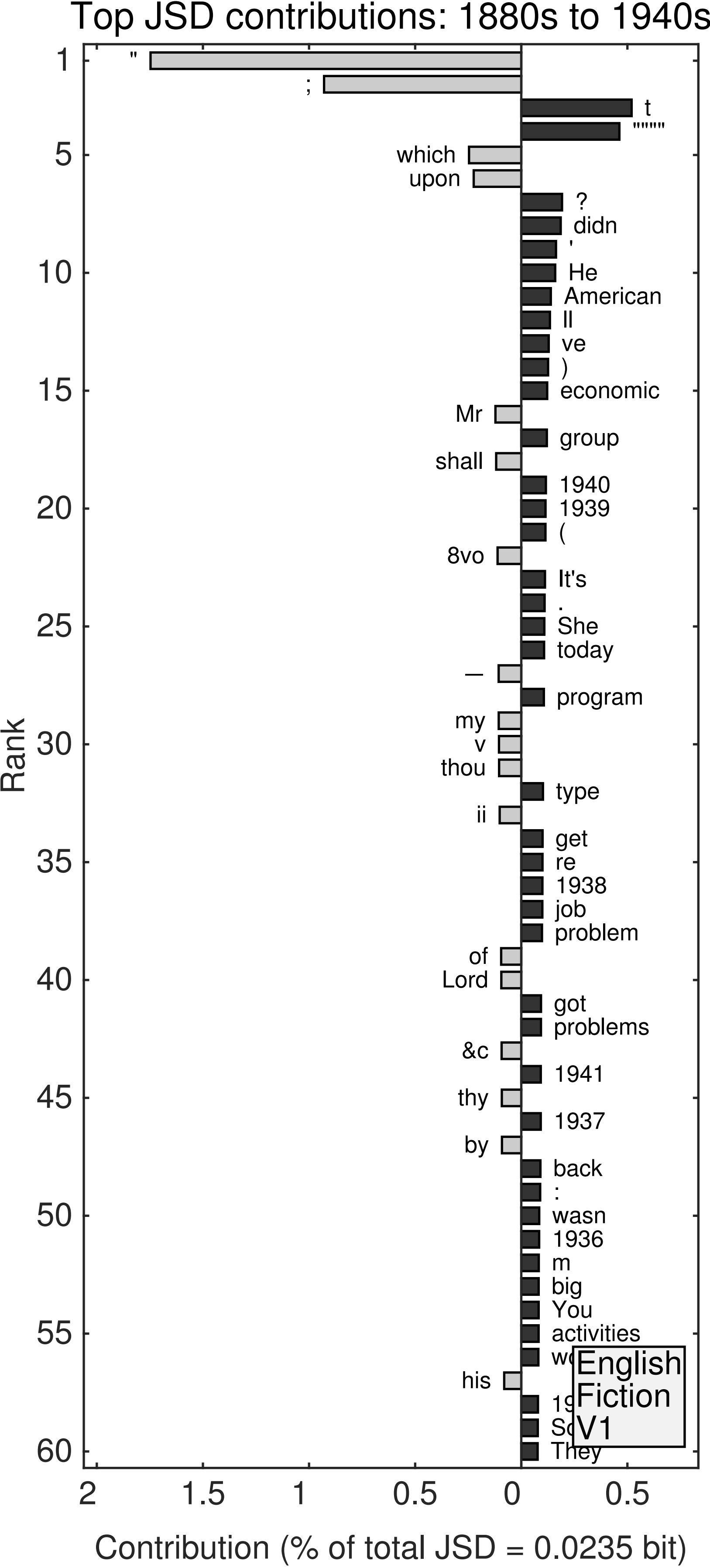

Top JSD contributions: 1880s to 1940s
Rank
Contribution (% of total JSD = 0.0235 bit)
"
;
t
""""
which
upon
?
didn
'
He
American
ll
ve
)
economic
Mr
group
shall
1940
1939
(
8vo
It's
.
She
today
—
program
my
v
thou
type
ii
get
re
1938
job
problem
of
Lord
got
problems
&c
1941
thy
1937
by
back
:
wasn
1936
m
big
You
activities
his
They
English Fiction V1

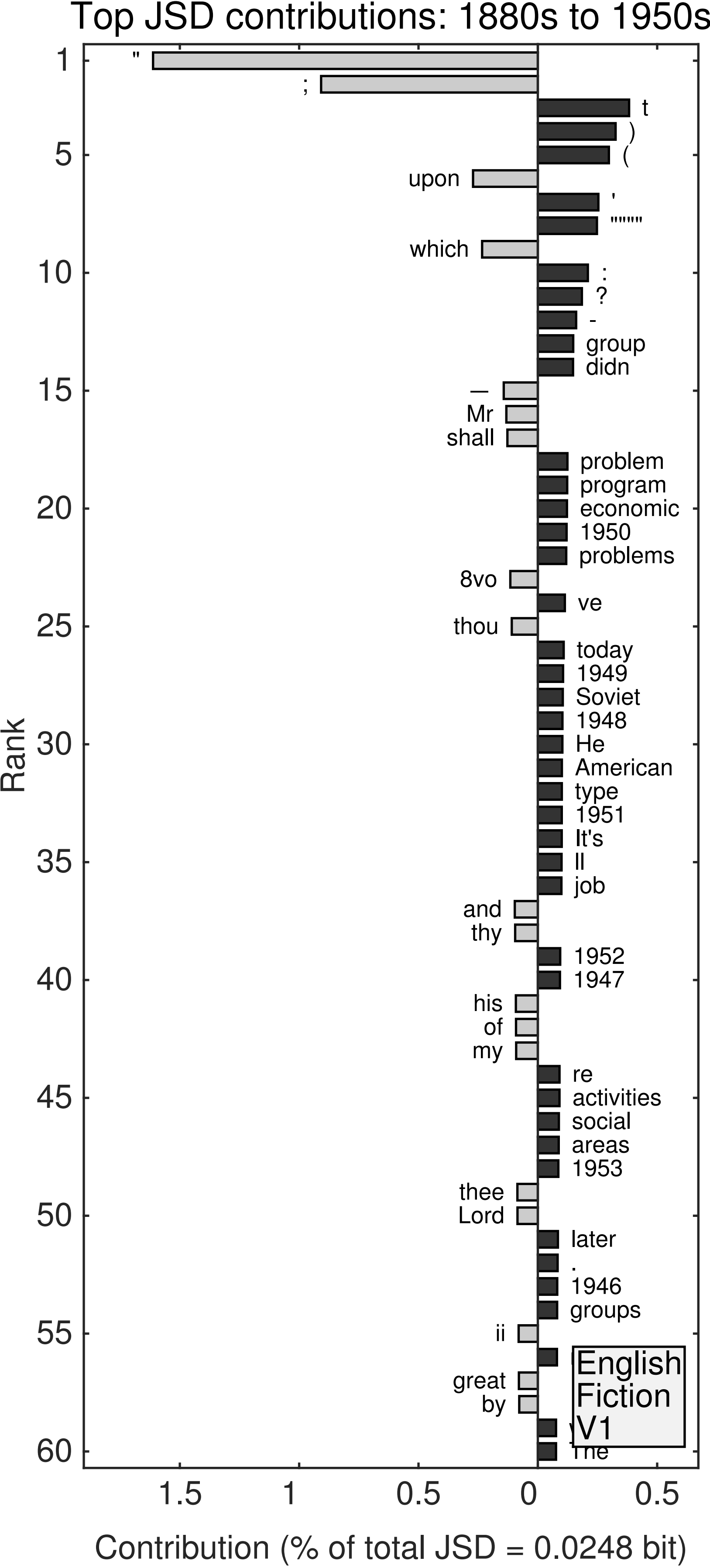
Top JSD contributions: 1880s to 1950s
Rank
Contribution (% of total JSD = 0.0248 bit)
1.5
1
0.5
0
0.5
1
5
10
15
20
25
30
35
40
45
50
55
60
"
;
t
)
(
upon
'
""""
which
:
?
-
group
didn
—
Mr
shall
problem
program
economic
1950
problems
8vo
ve
thou
today
1949
Soviet
1948
He
American
type
1951
It's
ll
job
and
thy
1952
1947
his
of
my
re
activities
social
areas
1953
thee
Lord
later
.
1946
groups
ii
great
by
The
English
Fiction
V1

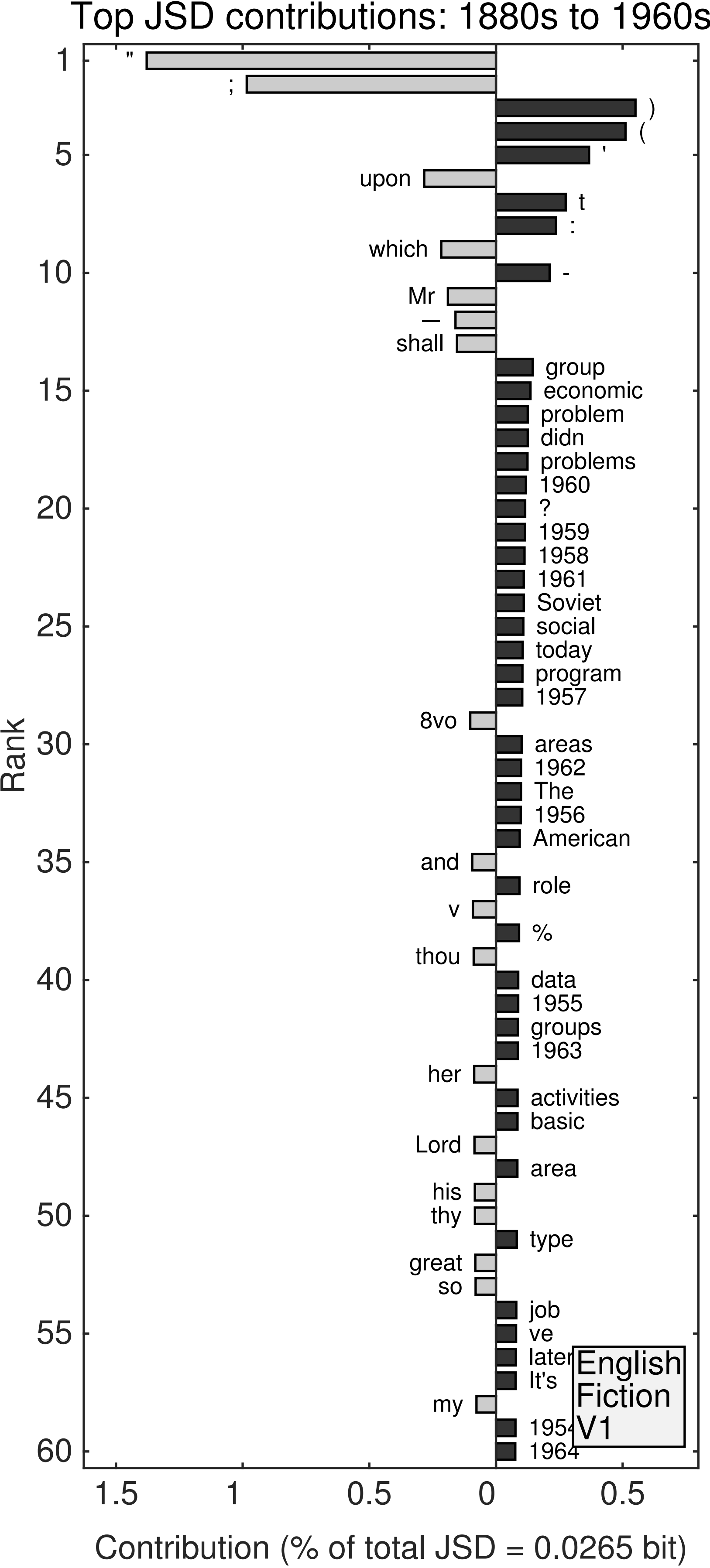
Top JSD contributions: 1880s to 1960s
Rank
Contribution (% of total JSD = 0.0265 bit)
"
;
)
(
'
upon
t
:
which
-
Mr
—
shall
group
economic
problem
didn
problems
1960
?
1959
1958
1961
Soviet
social
today
program
1957
8vo
areas
1962
The
1956
American
and
role
v
%
thou
data
1955
groups
1963
her
activities
basic
Lord
area
his
thy
type
great
so
job
ve
later
It's
my
1954
1964
English Fiction V1

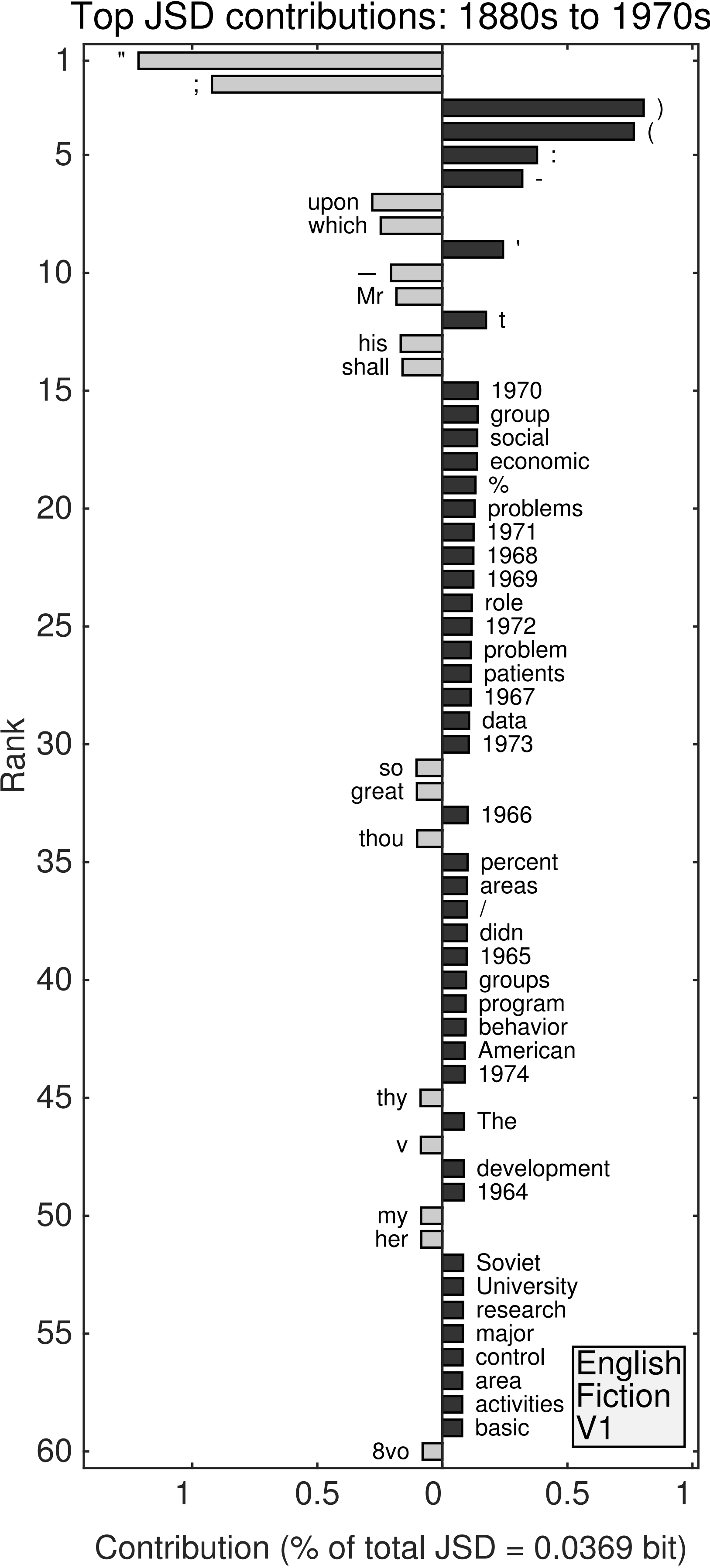

Top JSD contributions: 1880s to 1970s
Rank
"
;
)
(
:
-
upon
which
'
—
Mr
t
his
shall
1970
group
social
economic
%
problems
1971
1968
1969
role
1972
problem
patients
1967
data
1973
so
great
1966
thou
percent
areas
/
didn
1965
groups
program
behavior
American
1974
thy
The
v
development
1964
my
her
Soviet
University
research
major
control
area
activities
basic
8vo
English Fiction V1
Contribution (% of total JSD = 0.0369 bit)

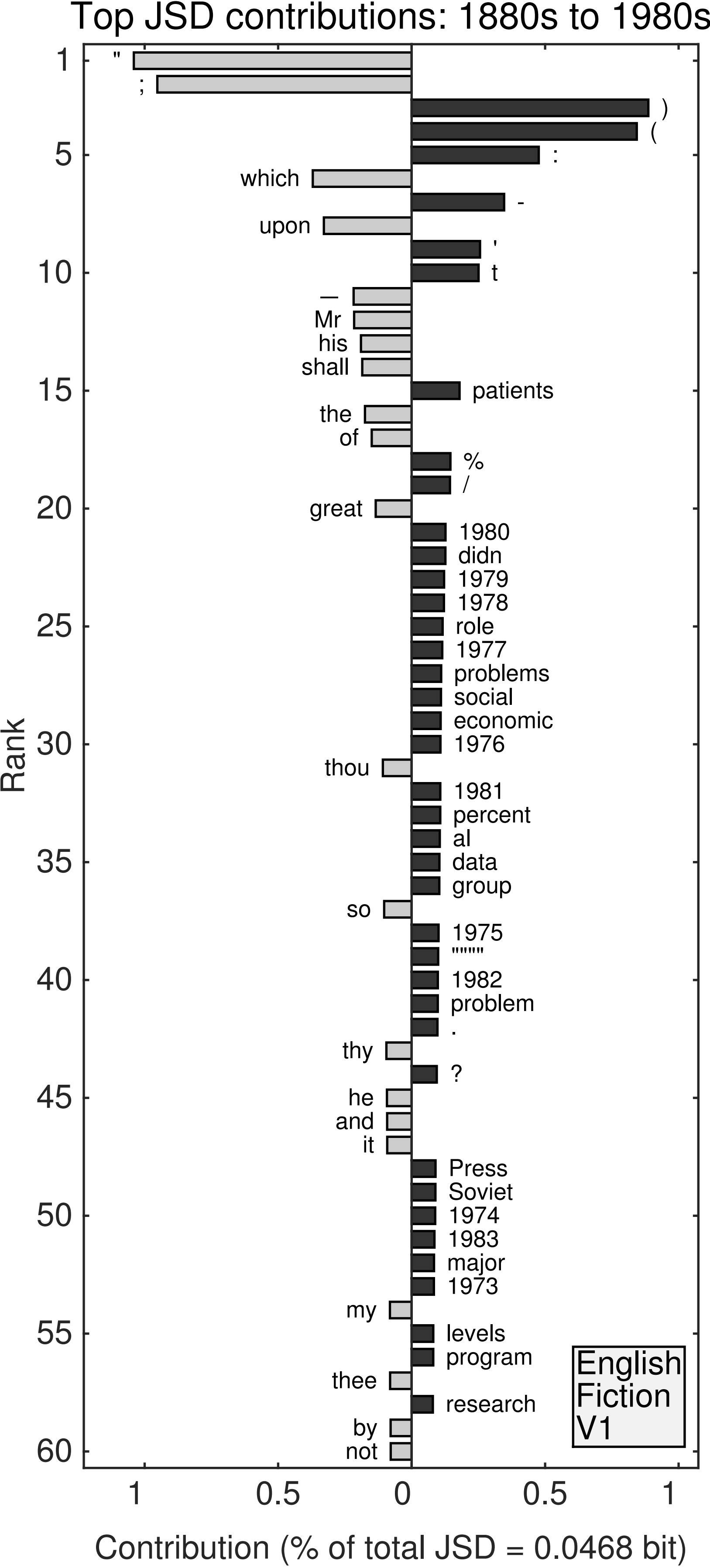

Top JSD contributions: 1880s to 1980s
Rank
Contribution (% of total JSD = 0.0468 bit)
English Fiction V1
"
;
)
(
:
which
-
upon
'
t
—
Mr
his
shall
patients
the
of
%
/
great
1980
didn
1979
1978
role
1977
problems
social
economic
1976
thou
1981
percent
al
data
group
so
1975
""""
1982
problem
.
thy
?
he
and
it
Press
Soviet
1974
1983
major
1973
my
levels
program
thee
research
by
not

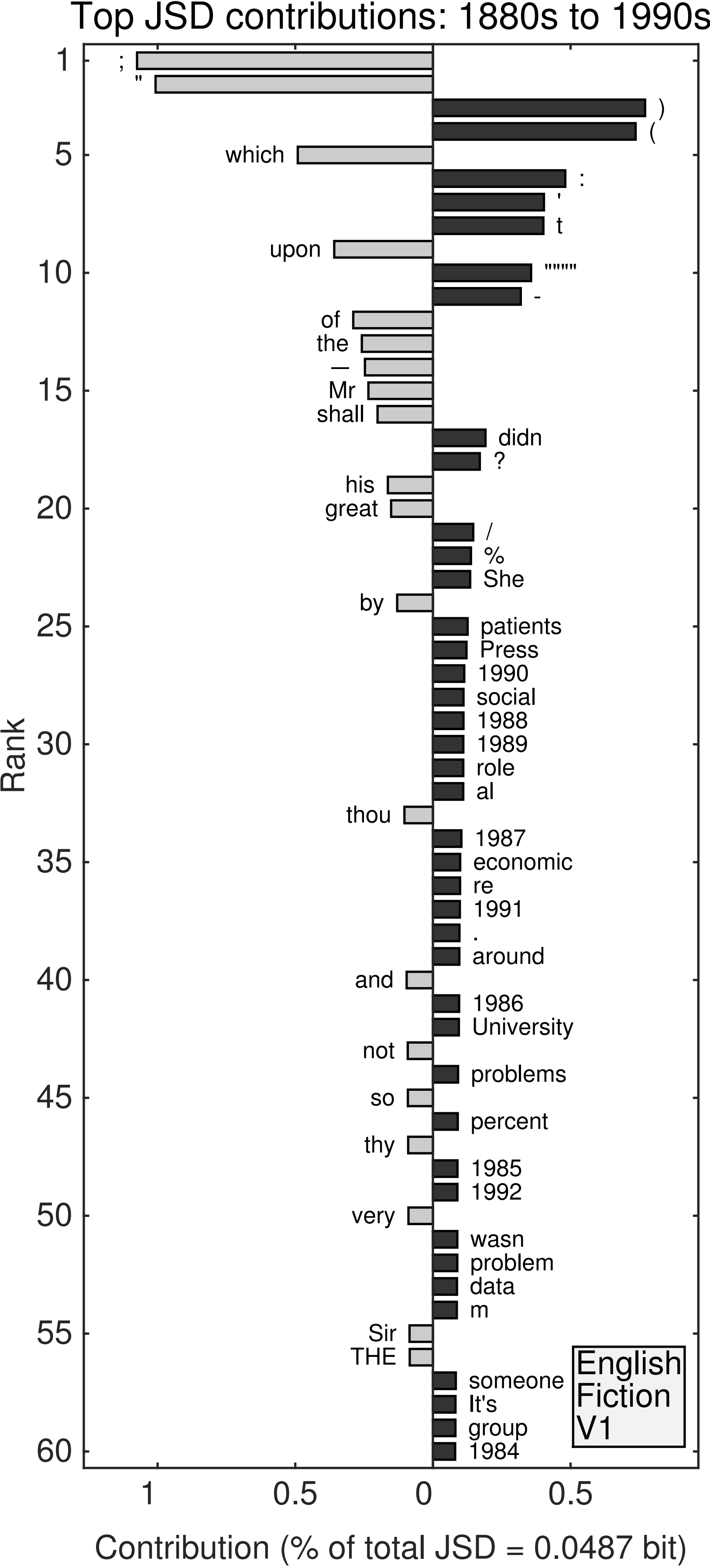

Top JSD contributions: 1880s to 1990s
Rank
Contribution (% of total JSD = 0.0487 bit)
English Fiction V1
;
"
)
(
which
:
'
t
upon
""""
-
of
the
—
Mr
shall
didn
?
his
great
/
%
She
by
patients
Press
1990
social
1988
1989
role
al
thou
1987
economic
re
1991
.
around
and
1986
University
not
problems
so
percent
thy
1985
1992
very
wasn
problem
data
m
Sir
THE
someone
It's
group
1984
1
5
10
15
20
25
30
35
40
45
50
55
60
1
0.5
0
0.5

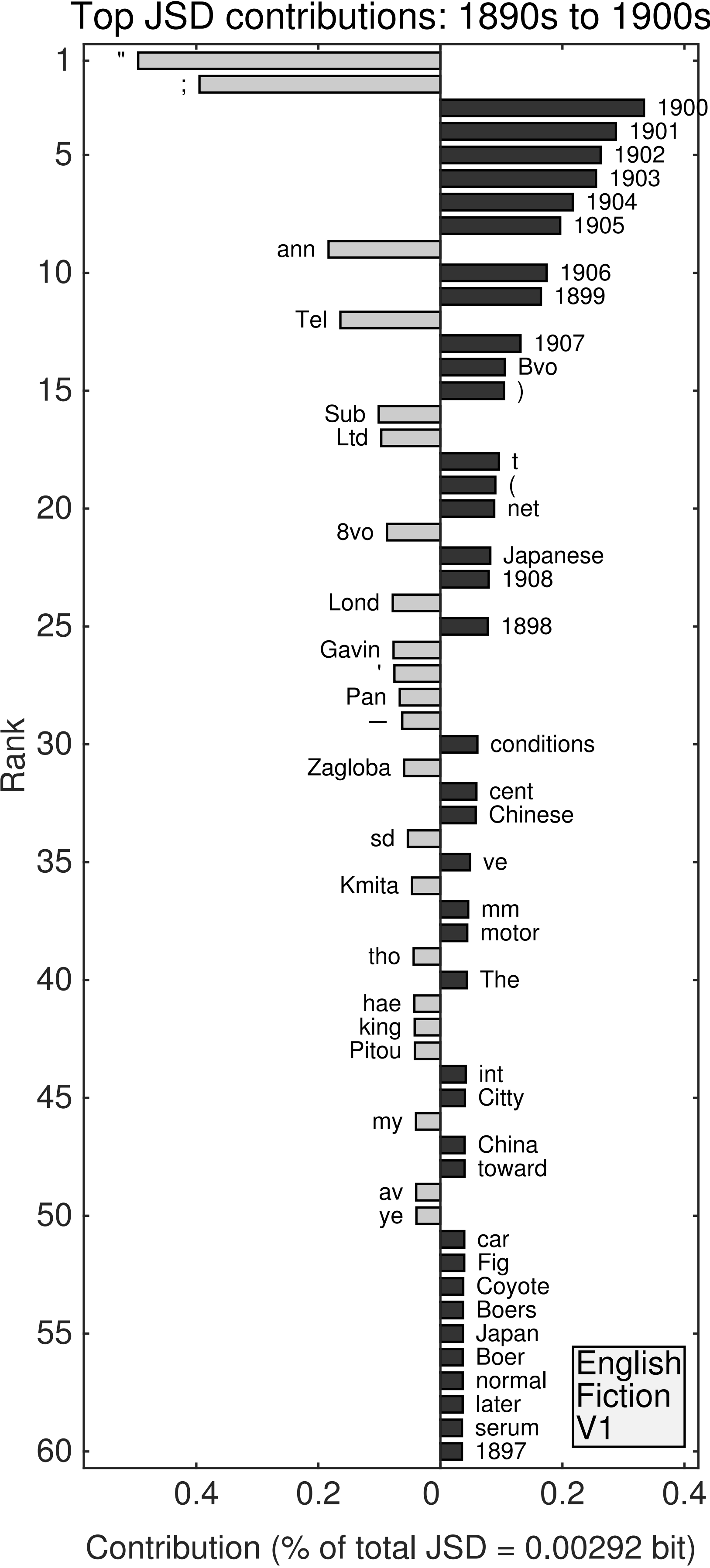

Top JSD contributions: 1890s to 1900s
Rank
Contribution (% of total JSD = 0.00292 bit)
English Fiction V1
"
;
1900
1901
1902
1903
1904
1905
ann
1906
1899
Tel
1907
Bvo
)
Sub
Ltd
t
(
net
8vo
Japanese
1908
Lond
1898
Gavin
'
Pan
—
conditions
Zagloba
cent
Chinese
sd
ve
Kmita
mm
motor
tho
The
hae
king
Pitou
int
Citty
my
China
toward
av
ye
car
Fig
Coyote
Boers
Japan
Boer
normal
later
serum
1897

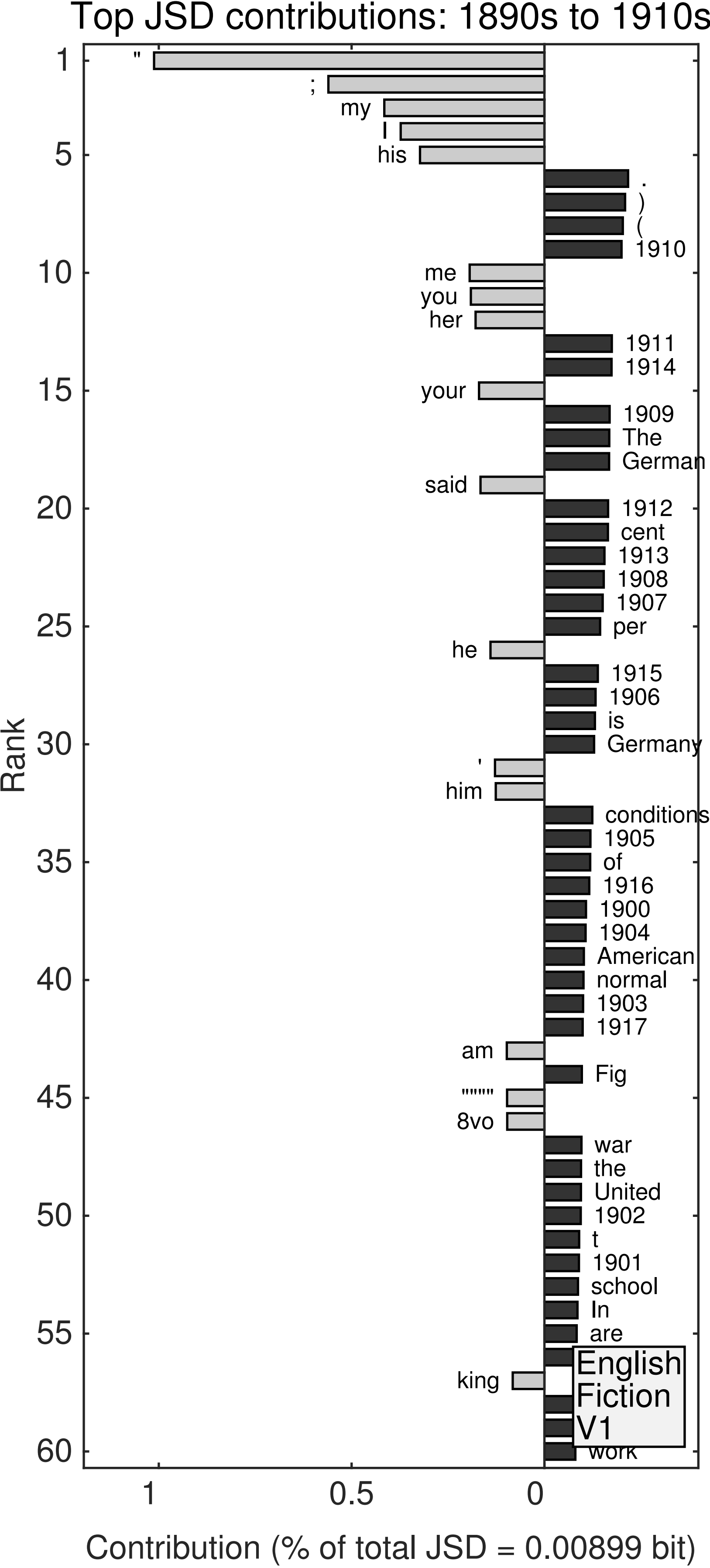
Top JSD contributions: 1890s to 1910s
Rank
Contribution (% of total JSD = 0.00899 bit)
1
5
10
15
20
25
30
35
40
45
50
55
60
1
0.5
0
"
;
my
I
his
.
)
(
1910
me
you
her
1911
1914
your
1909
The
German
said
1912
cent
1913
1908
1907
per
he
1915
1906
is
Germany
'
him
conditions
1905
of
1916
1900
1904
American
normal
1903
1917
am
Fig
""""
8vo
war
the
United
1902
t
1901
school
In
are
king
work
English
Fiction
V1

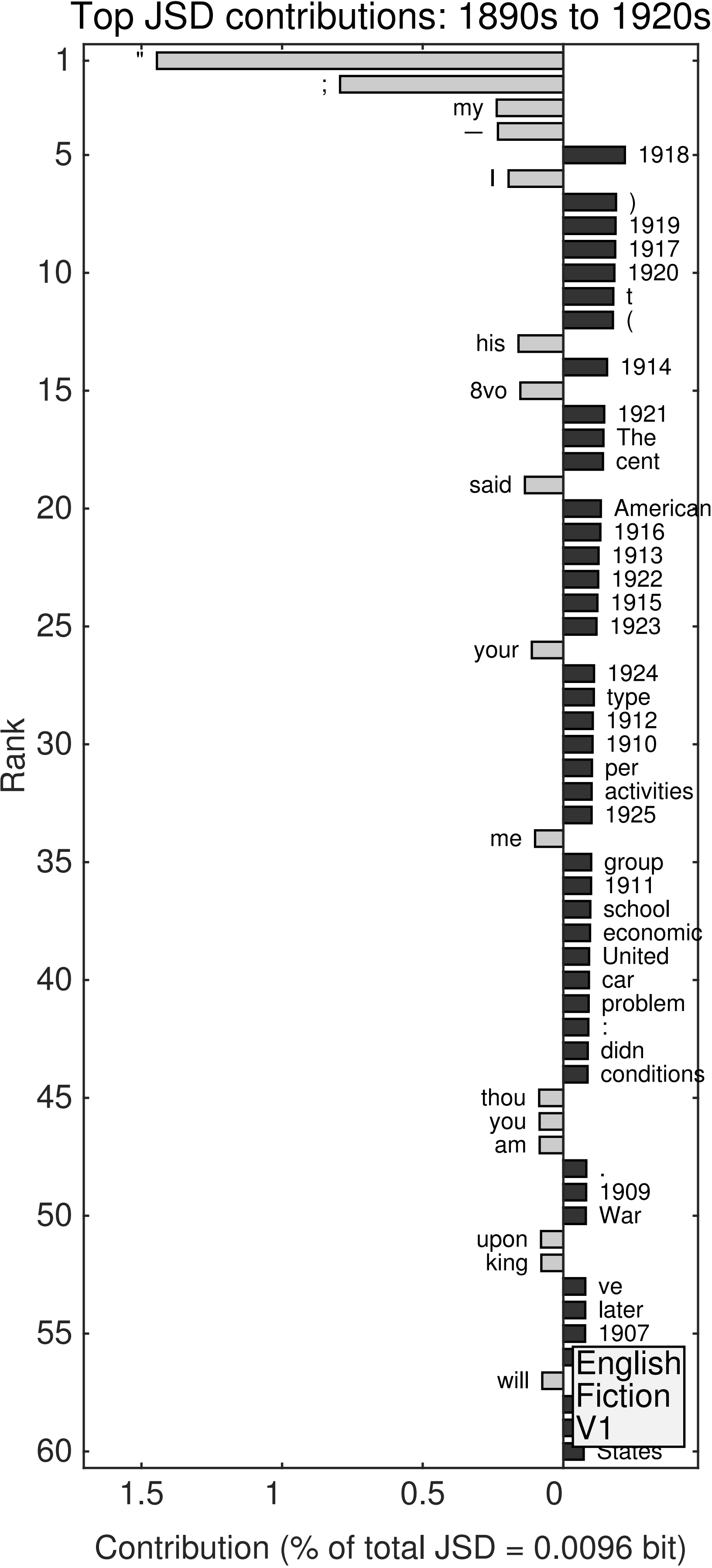

Top JSD contributions: 1890s to 1920s
Rank
Contribution (% of total JSD = 0.0096 bit)
1.5
1
0.5
0
1
5
10
15
20
25
30
35
40
45
50
55
60
"
;
my
—
1918
I
)
1919
1917
1920
t
(
his
1914
8vo
1921
The
cent
said
American
1916
1913
1922
1915
1923
your
1924
type
1912
1910
per
activities
1925
me
group
1911
school
economic
United
car
problem
:
didn
conditions
thou
you
am
.
1909
War
upon
king
ve
later
1907
will
States
English
Fiction
V1

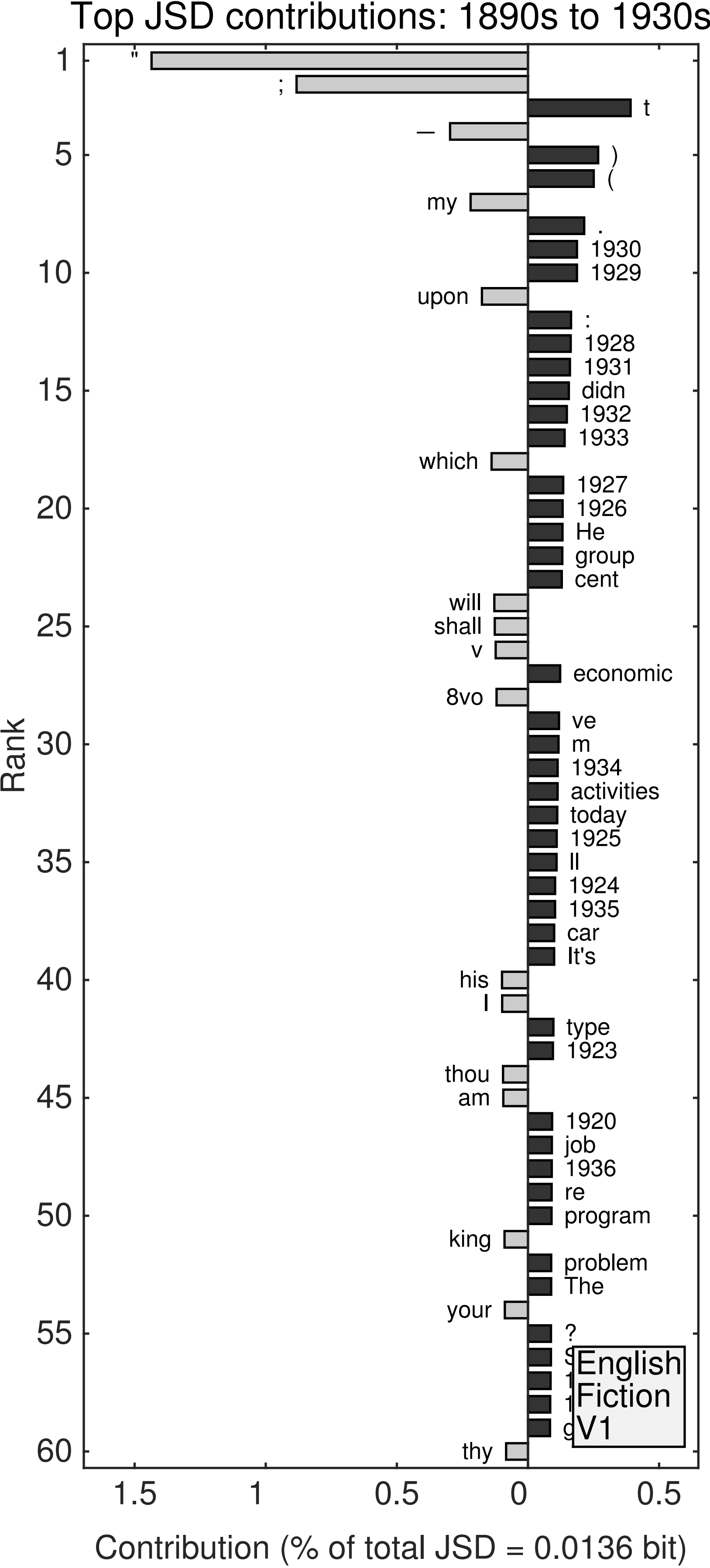
Top JSD contributions: 1890s to 1930s
"
;
t
—
)
(
my
.
1930
1929
upon
:
1928
1931
didn
1932
1933
which
1927
1926
He
group
cent
will
shall
v
economic
8vo
ve
m
1934
activities
today
1925
ll
1924
1935
car
It's
his
I
type
1923
thou
am
1920
job
1936
re
program
king
problem
The
your
?
thy
English
Fiction
V1
Rank
1
5
10
15
20
25
30
35
40
45
50
55
60
1.5
1
0.5
0
0.5
Contribution (% of total JSD = 0.0136 bit)

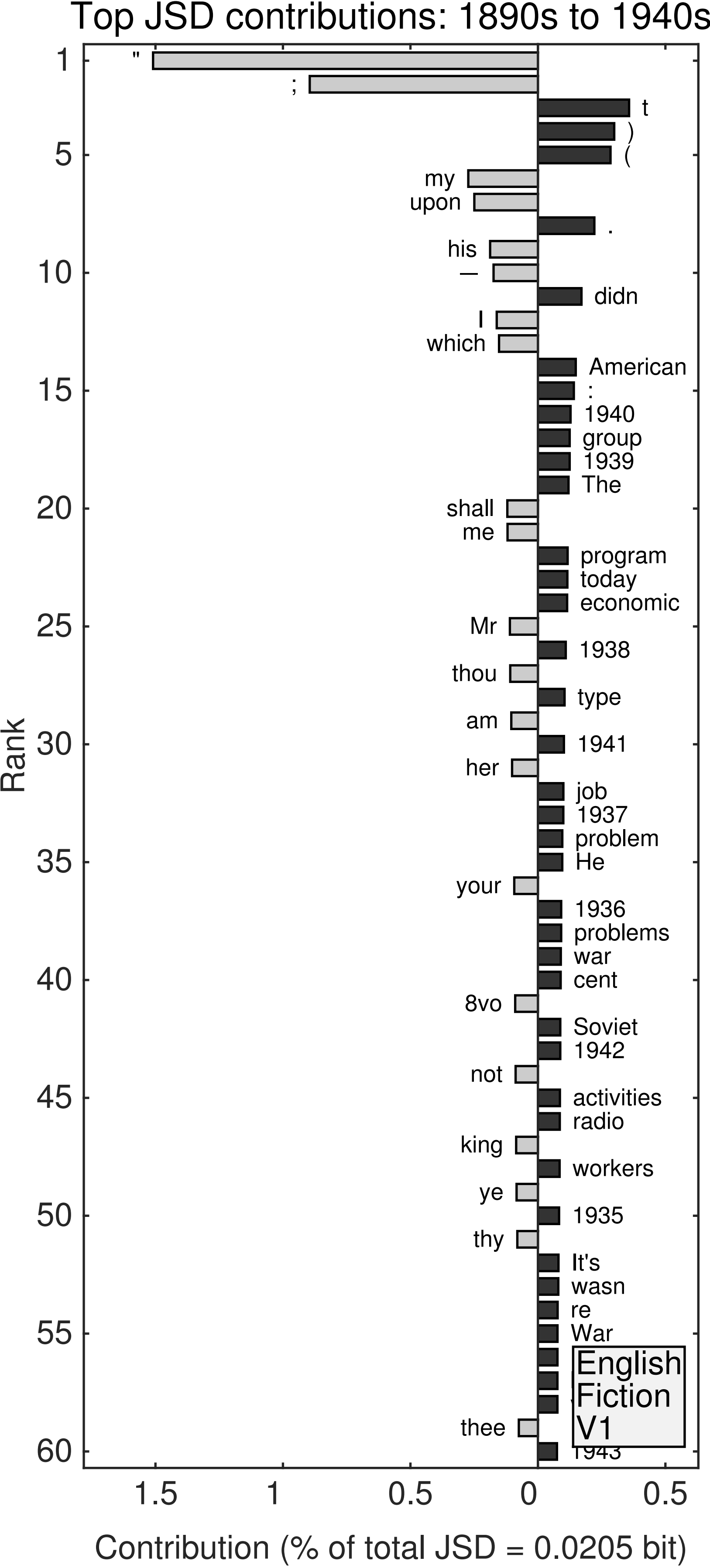

Top JSD contributions: 1890s to 1940s
"
;
t
)
(
my
upon
.
his
—
didn
I
which
American
:
1940
group
1939
The
shall
me
program
today
economic
Mr
1938
thou
type
am
1941
her
job
1937
problem
He
your
1936
problems
war
cent
8vo
Soviet
1942
not
activities
radio
king
workers
ye
1935
thy
It's
wasn
re
War
thee
1943
English Fiction V1
Rank
1
5
10
15
20
25
30
35
40
45
50
55
60
1.5
1
0.5
0
0.5
Contribution (% of total JSD = 0.0205 bit)

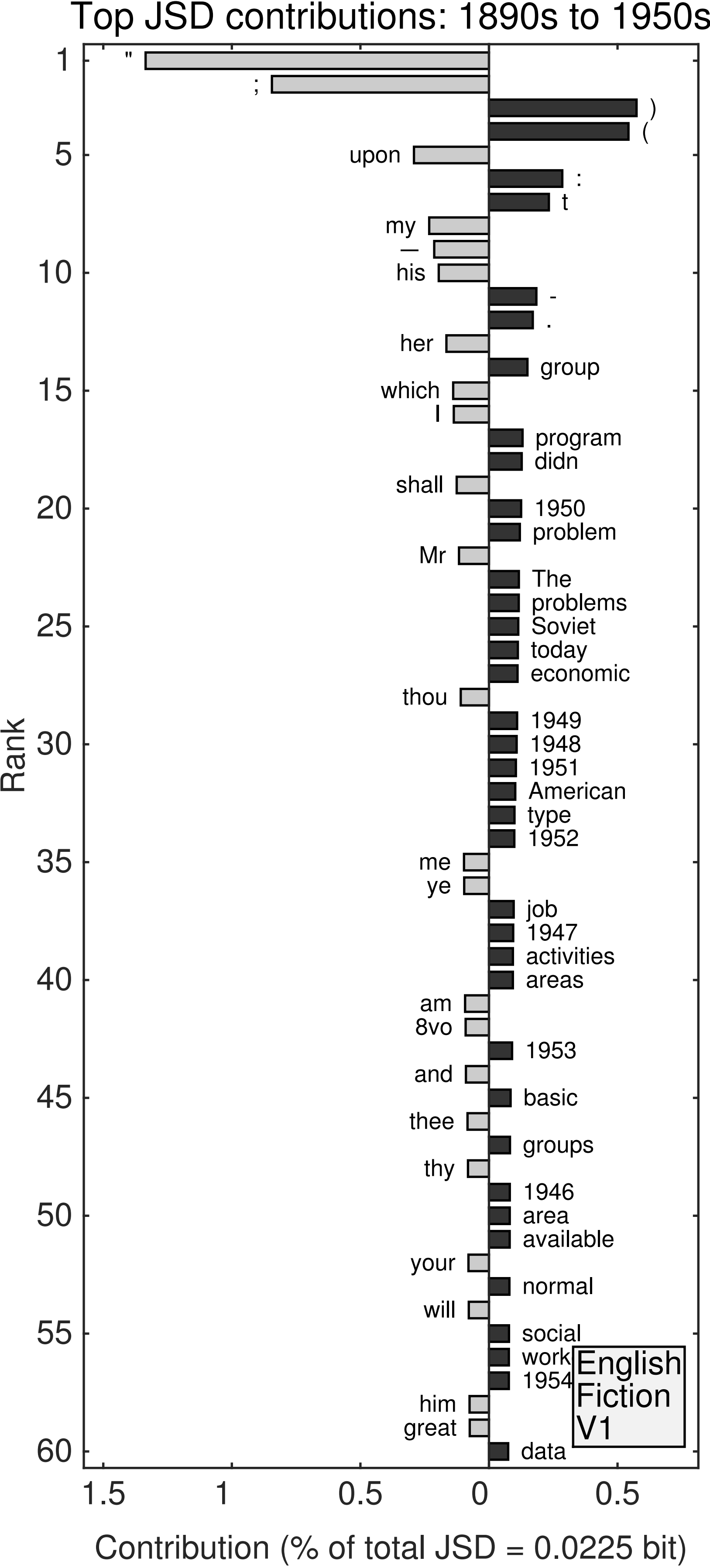

Top JSD contributions: 1890s to 1950s
Rank
Contribution (% of total JSD = 0.0225 bit)
"
;
)
(
upon
:
t
my
—
his
-
.
her
group
which
I
program
didn
shall
1950
problem
Mr
The
problems
Soviet
today
economic
thou
1949
1948
1951
American
type
1952
me
ye
job
1947
activities
areas
am
8vo
1953
and
basic
thee
groups
thy
1946
area
available
your
normal
will
social
work
1954
him
great
data
English Fiction V1

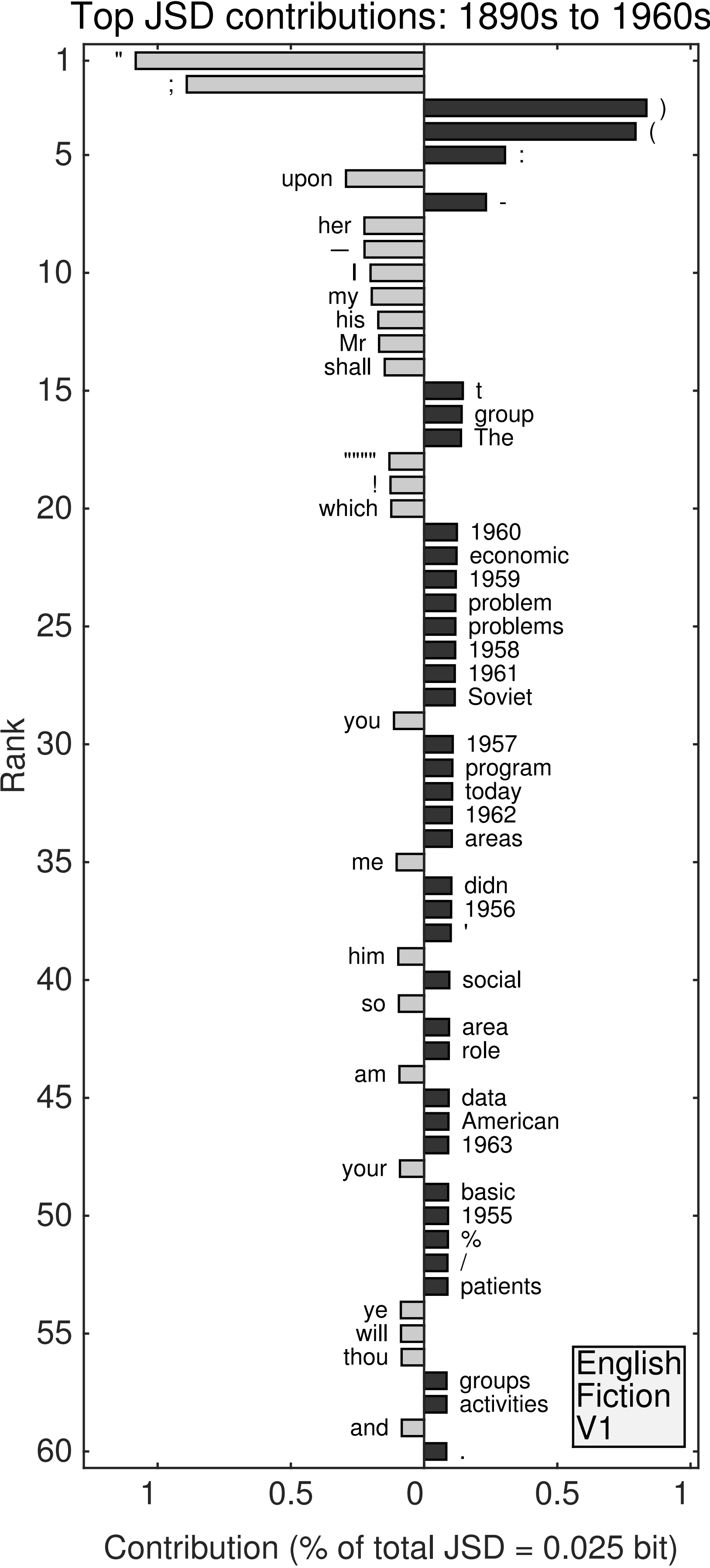

Top JSD contributions: 1890s to 1960s
Rank
Contribution (% of total JSD = 0.025 bit)
English Fiction V1
"
;
)
(
:
upon
-
her
—
I
my
his
Mr
shall
t
group
The
""""
!
which
1960
economic
1959
problem
problems
1958
1961
Soviet
you
1957
program
today
1962
areas
me
didn
1956
'
him
social
so
area
role
am
data
American
1963
your
basic
1955
%
/
patients
ye
will
thou
groups
activities
and
.

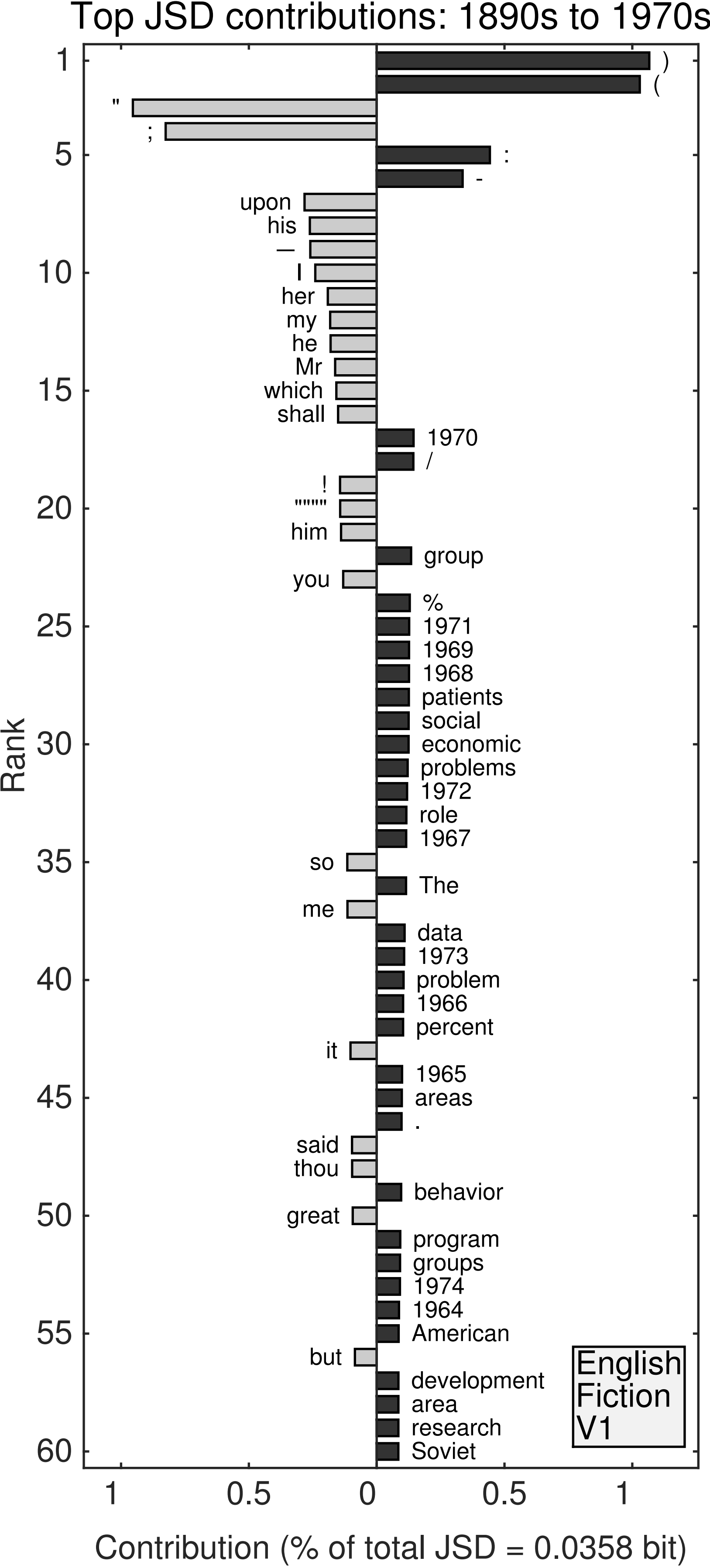

Top JSD contributions: 1890s to 1970s
Rank
Contribution (% of total JSD = 0.0358 bit)
English Fiction V1
)
(
"
;
:
-
upon
his
—
I
her
my
he
Mr
which
shall
1970
/
!
""""
him
group
you
%
1971
1969
1968
patients
social
economic
problems
1972
role
1967
so
The
me
data
1973
problem
1966
percent
it
1965
areas
.
said
thou
behavior
great
program
groups
1974
1964
American
but
development
area
research
Soviet

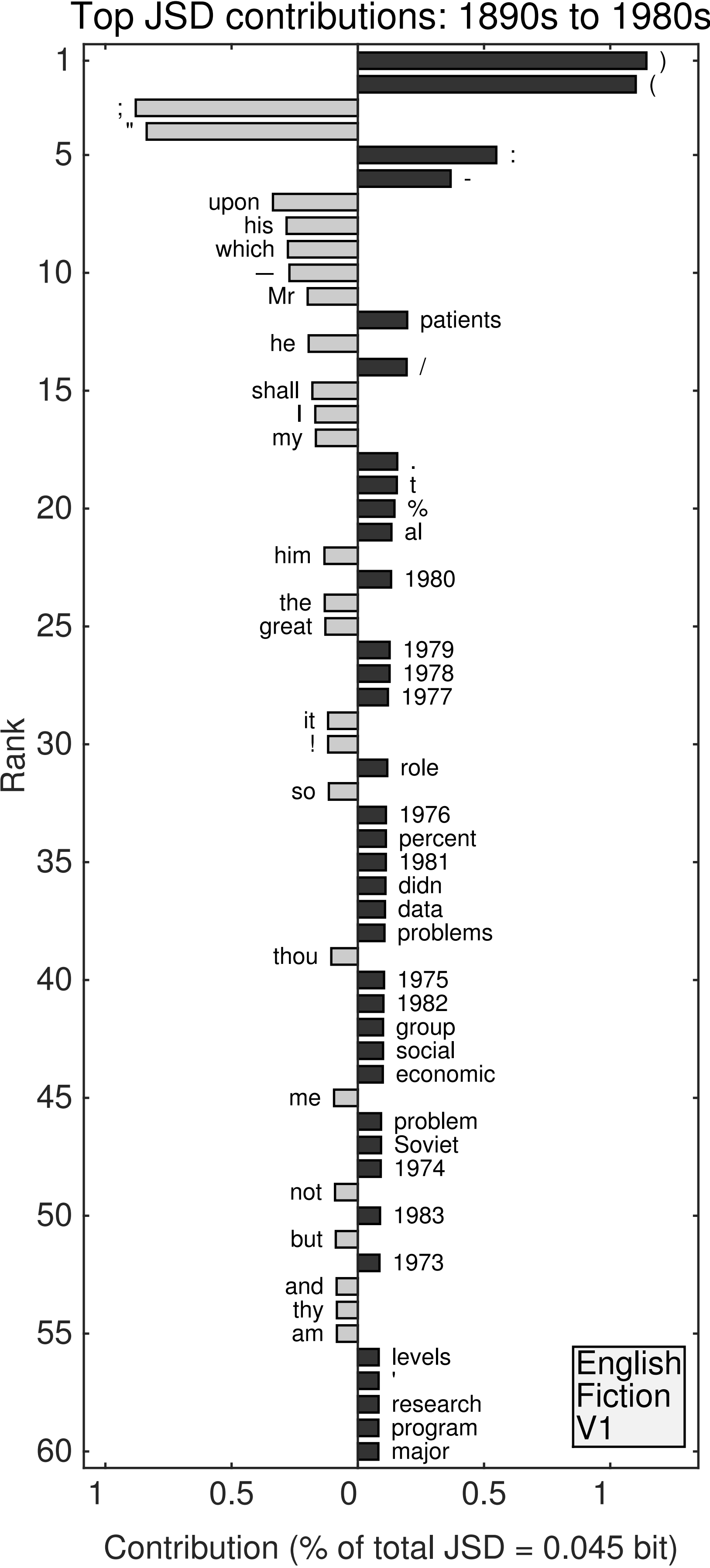

Top JSD contributions: 1890s to 1980s
Rank
Contribution (% of total JSD = 0.045 bit)
)
(
;
"
:
-
upon
his
which
—
Mr
patients
he
/
shall
I
my
.
t
%
al
him
1980
the
great
1979
1978
1977
it
!
role
so
1976
percent
1981
didn
data
problems
thou
1975
1982
group
social
economic
me
problem
Soviet
1974
not
1983
but
1973
and
thy
am
levels
'
research
program
major
English
Fiction
V1

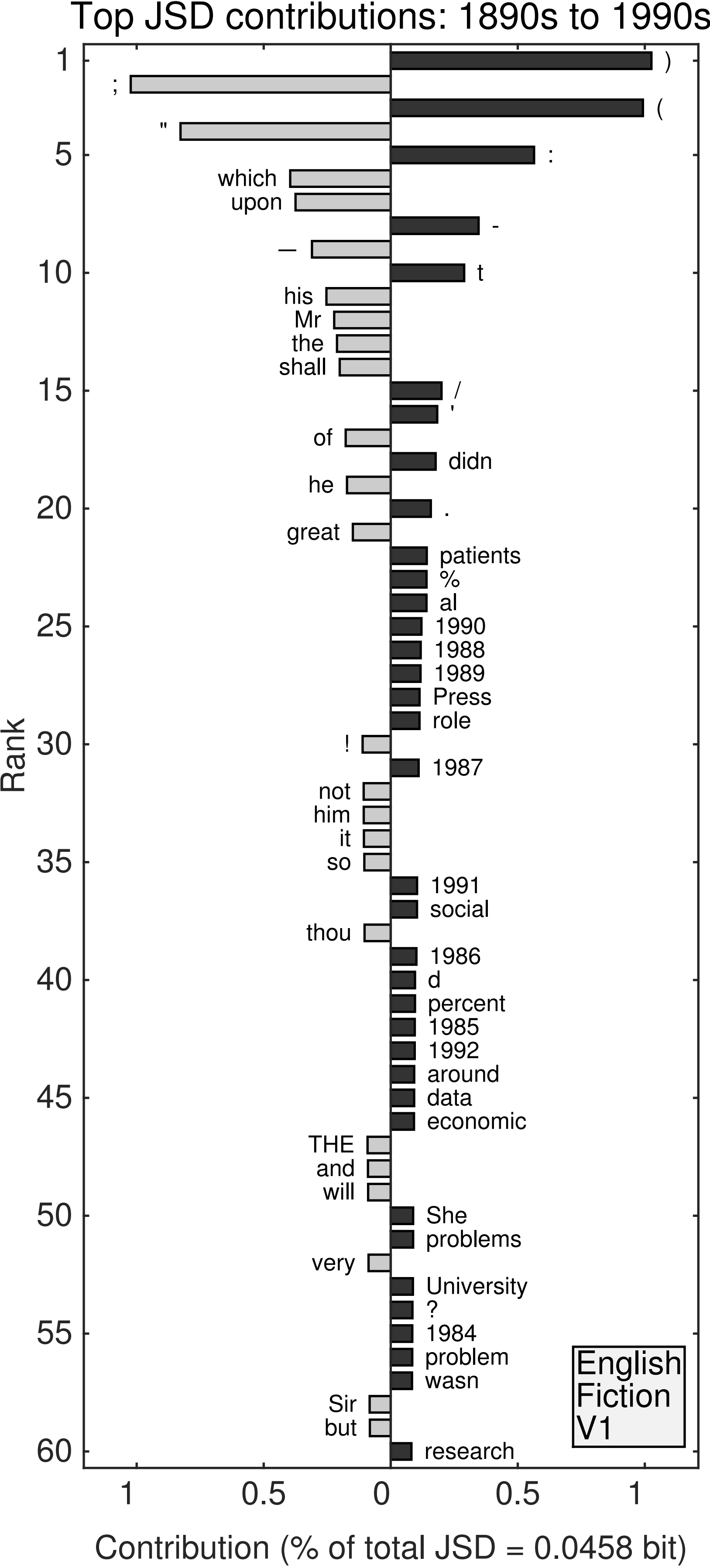

Top JSD contributions: 1890s to 1990s
Rank
Contribution (% of total JSD = 0.0458 bit)
English Fiction V1
)
;
(
"
:
which
upon
-
—
t
his
Mr
the
shall
/
'
of
didn
he
.
great
patients
%
al
1990
1988
1989
Press
role
!
1987
not
him
it
so
1991
social
thou
1986
d
percent
1985
1992
around
data
economic
THE
and
will
She
problems
very
University
?
1984
problem
wasn
Sir
but
research

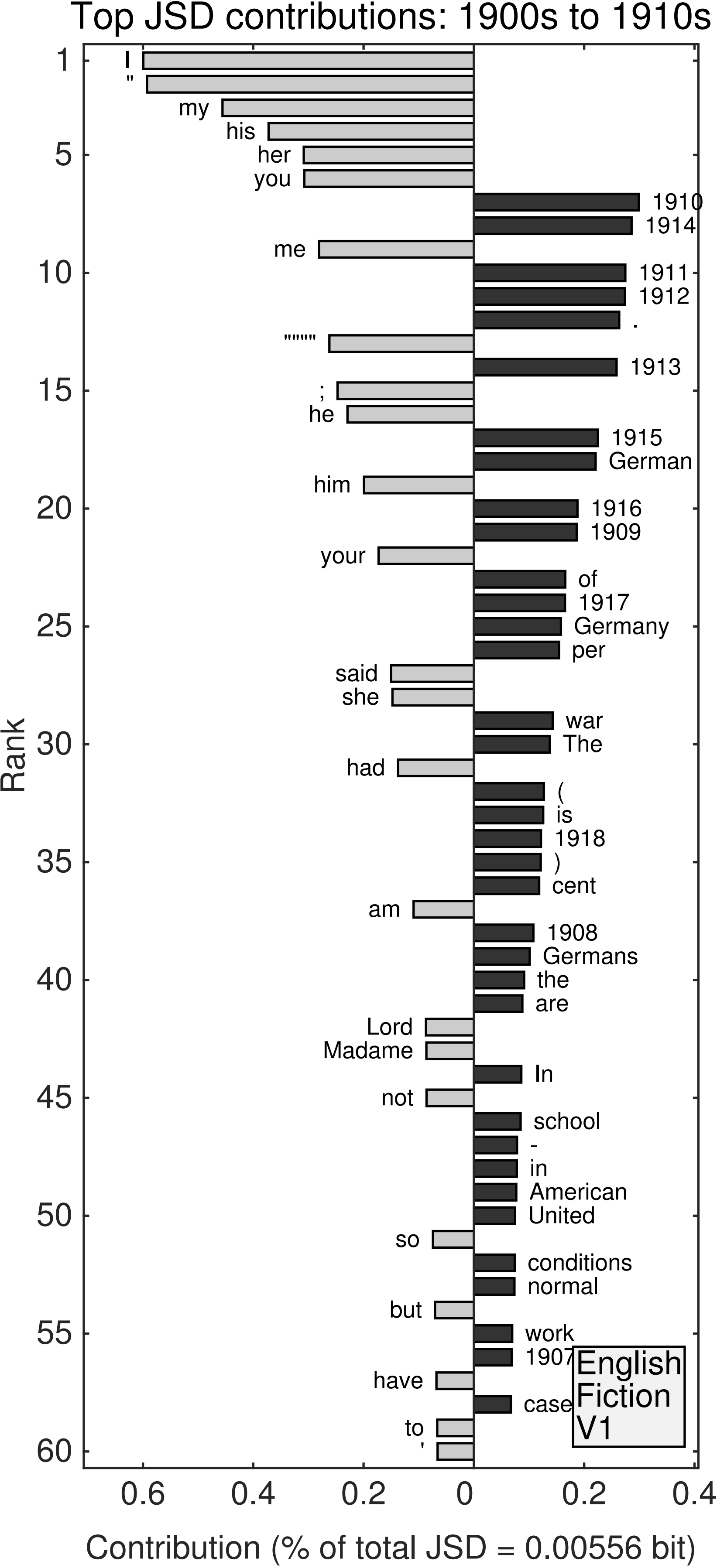

Top JSD contributions: 1900s to 1910s
Rank
Contribution (% of total JSD = 0.00556 bit)
English Fiction V1
I
"
my
his
her
you
1910
1914
me
1911
1912
.
""""
1913
;
he
1915
German
him
1916
1909
your
of
1917
Germany
per
said
she
war
The
had
(
is
1918
)
cent
am
1908
Germans
the
are
Lord
Madame
In
not
school
-
in
American
United
so
conditions
normal
but
work
1907
have
case
to
'

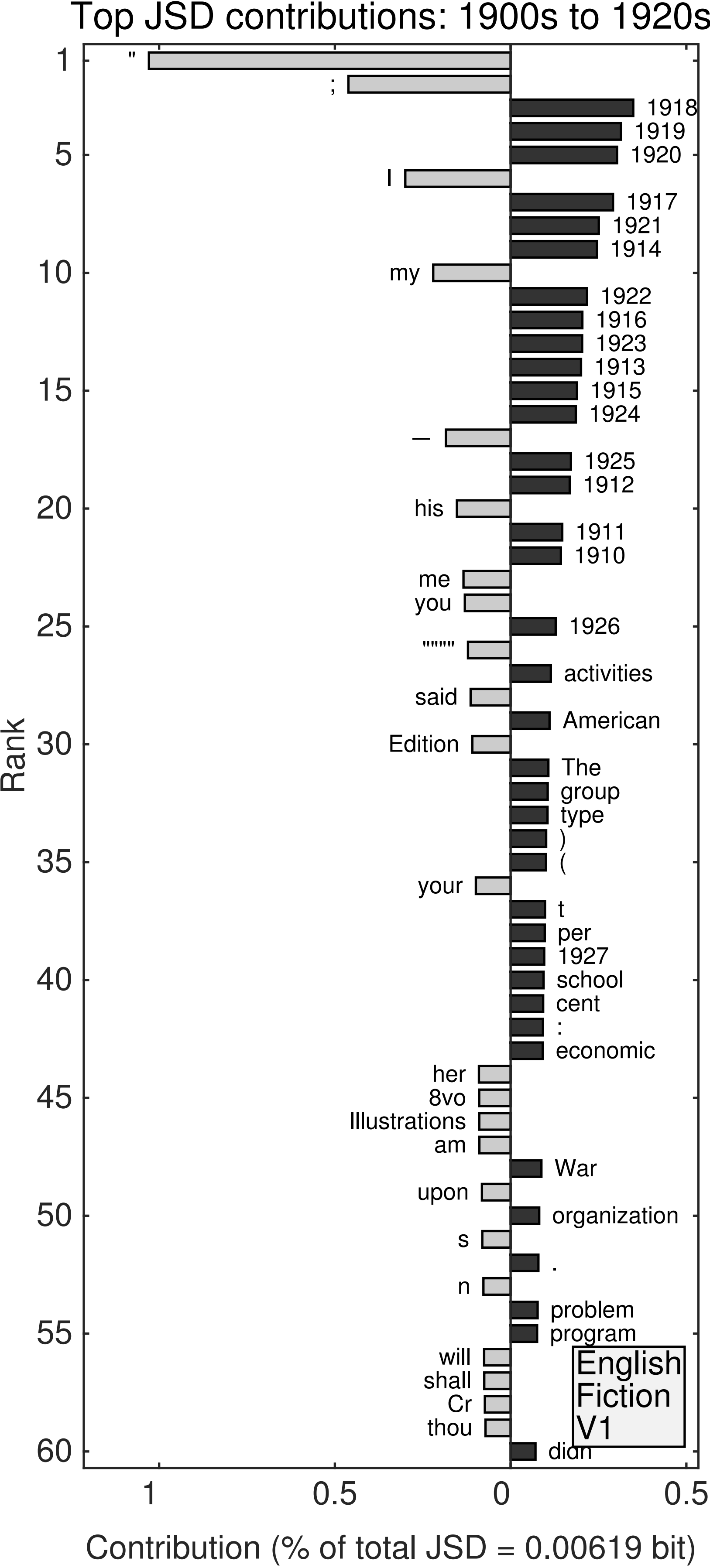
Top JSD contributions: 1900s to 1920s
Rank
Contribution (% of total JSD = 0.00619 bit)
English Fiction V1
"
;
1918
1919
1920
I
1917
1921
1914
my
1922
1916
1923
1913
1915
1924
—
1925
1912
his
1911
1910
me
you
1926
""""
activities
said
American
Edition
The
group
type
)
(
your
t
per
1927
school
cent
:
economic
her
8vo
Illustrations
am
War
upon
organization
s
.
n
problem
program
will
shall
Cr
thou
didn

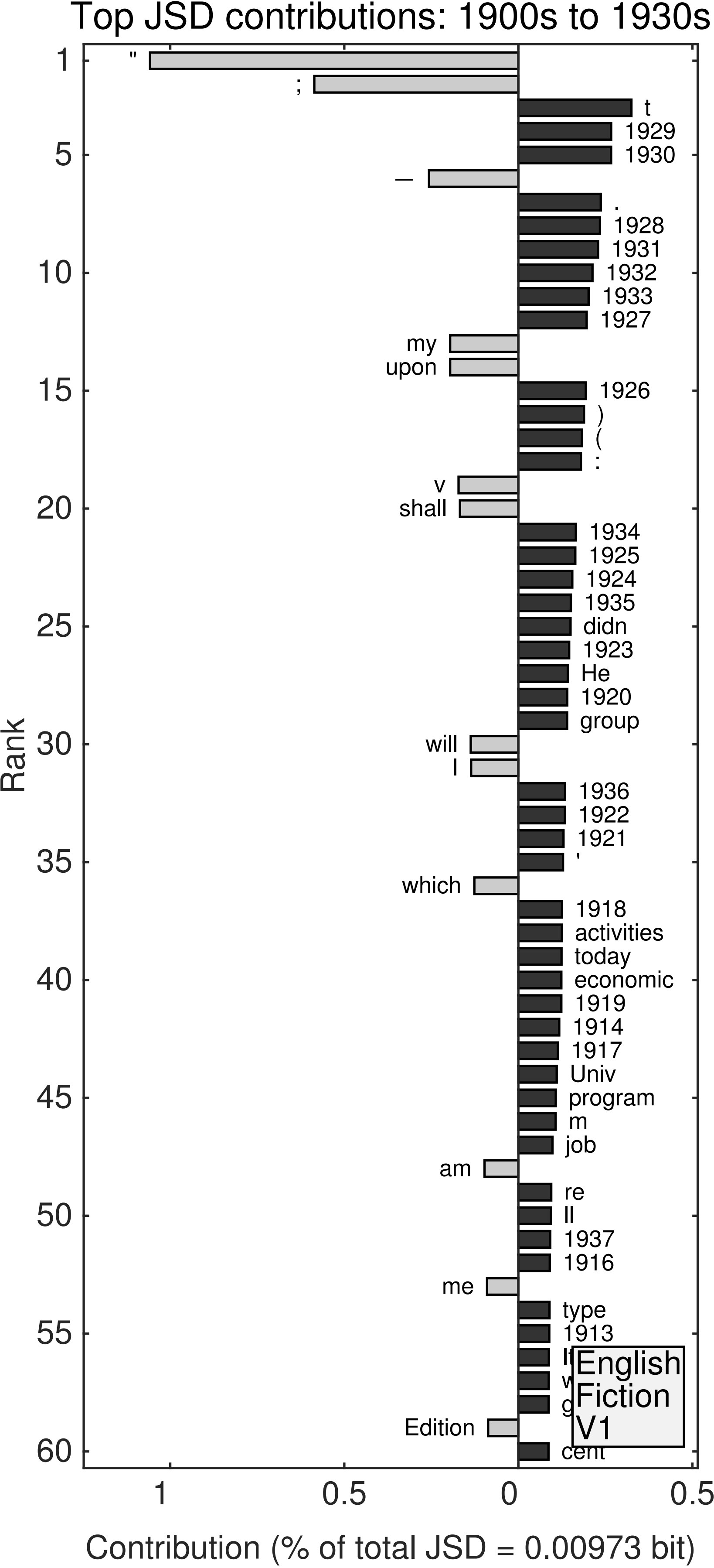
Top JSD contributions: 1900s to 1930s
Rank
Contribution (% of total JSD = 0.00973 bit)
1
5
10
15
20
25
30
35
40
45
50
55
60
1
0.5
0
0.5
"
;
t
1929
1930
—
.
1928
1931
1932
1933
1927
my
upon
1926
)
(
:
v
shall
1934
1925
1924
1935
didn
1923
He
1920
group
will
I
1936
1922
1921
'
which
1918
activities
today
economic
1919
1914
1917
Univ
program
m
job
am
re
ll
1937
1916
me
type
1913
Edition
cent
English
Fiction
V1

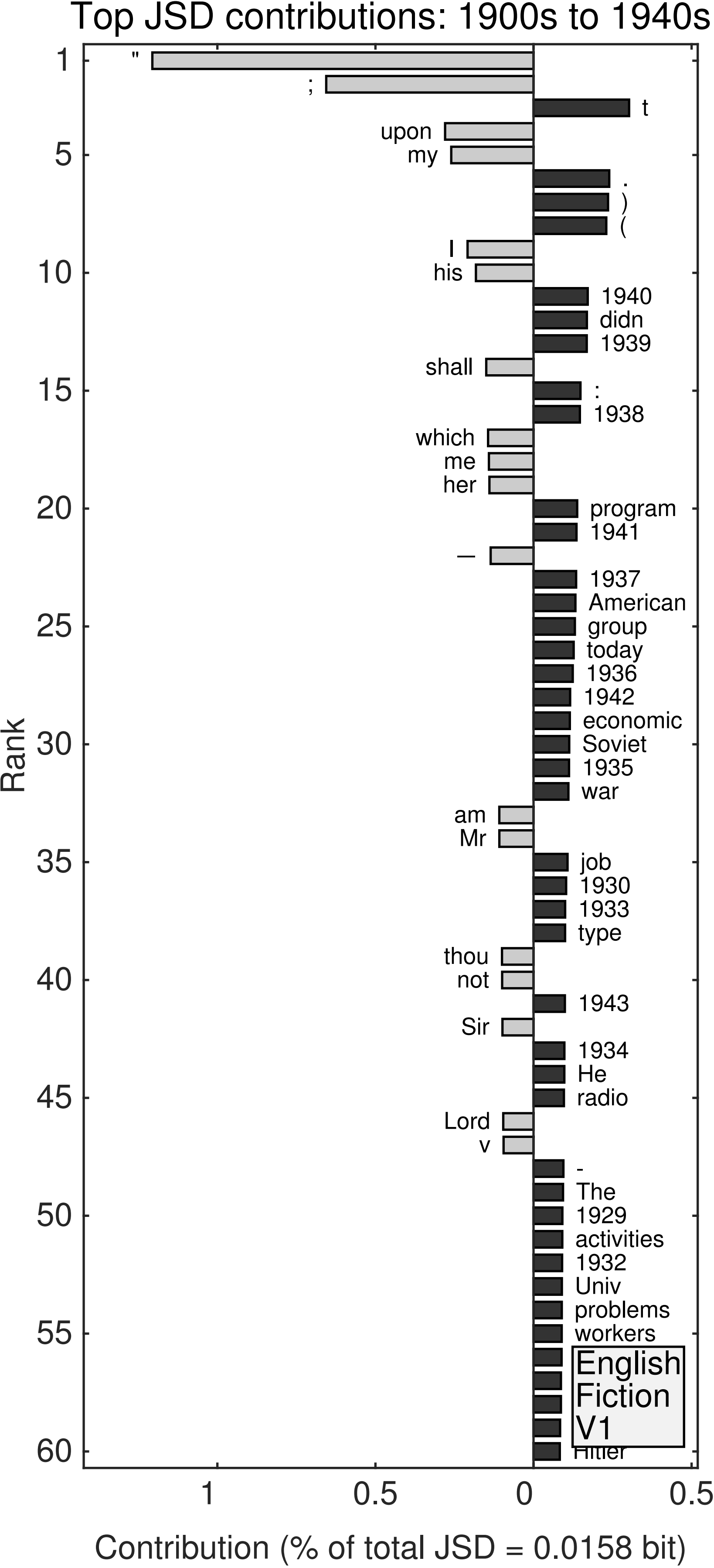

Top JSD contributions: 1900s to 1940s
Rank
Contribution (% of total JSD = 0.0158 bit)
1
0.5
0
0.5
5
10
15
20
25
30
35
40
45
50
55
60
"
;
t
upon
my
.
)
(
I
his
1940
didn
1939
shall
:
1938
which
me
her
program
1941
—
1937
American
group
today
1936
1942
economic
Soviet
1935
war
am
Mr
job
1930
1933
type
thou
not
1943
Sir
1934
He
radio
Lord
v
-
The
1929
activities
1932
Univ
problems
workers
Hitler
English
Fiction
V1

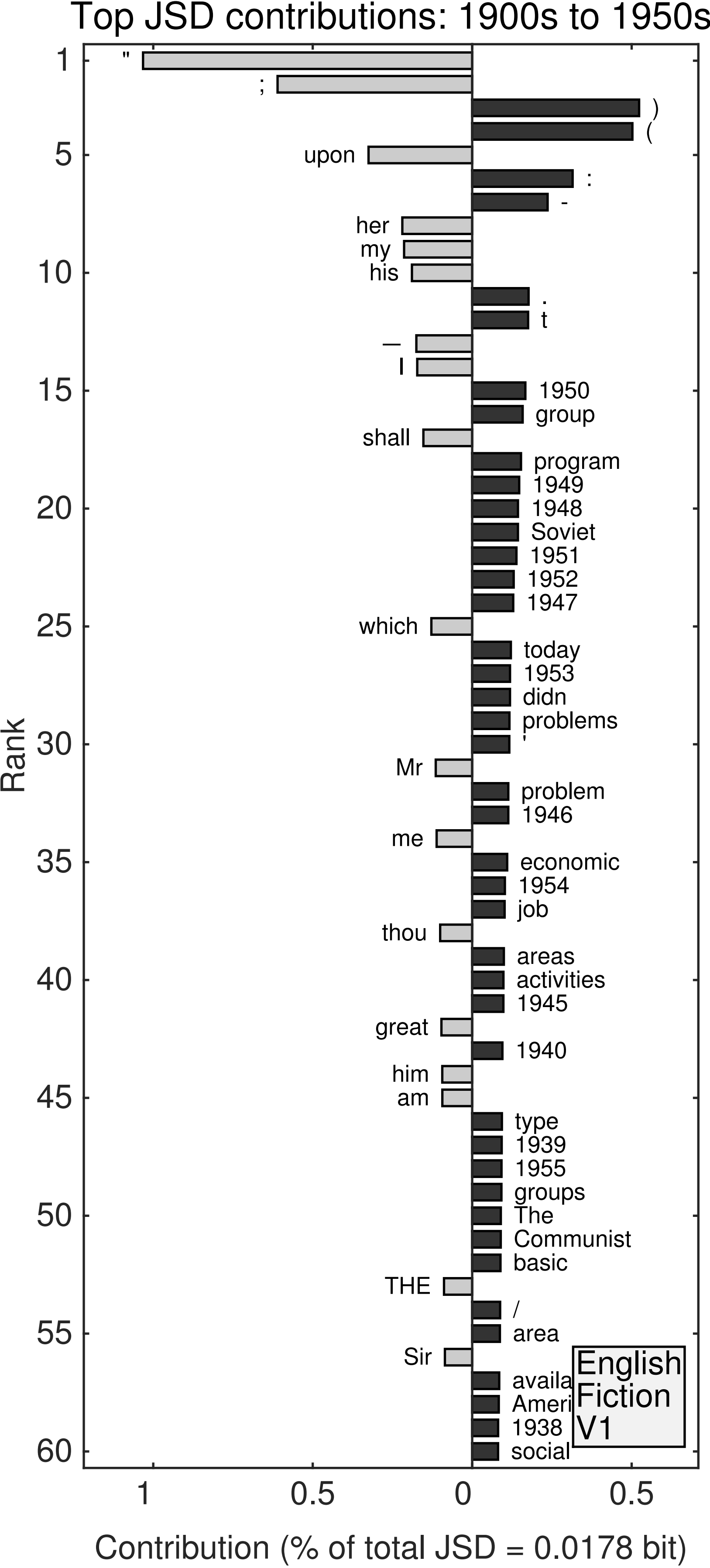

Top JSD contributions: 1900s to 1950s
Rank
Contribution (% of total JSD = 0.0178 bit)
English Fiction V1
"
;
)
(
upon
:
-
her
my
his
.
t
—
I
1950
group
shall
program
1949
1948
Soviet
1951
1952
1947
which
today
1953
didn
problems
'
Mr
problem
1946
me
economic
1954
job
thou
areas
activities
1945
great
1940
him
am
type
1939
1955
groups
The
Communist
basic
THE
/
area
Sir
availa
Ameri
1938
social

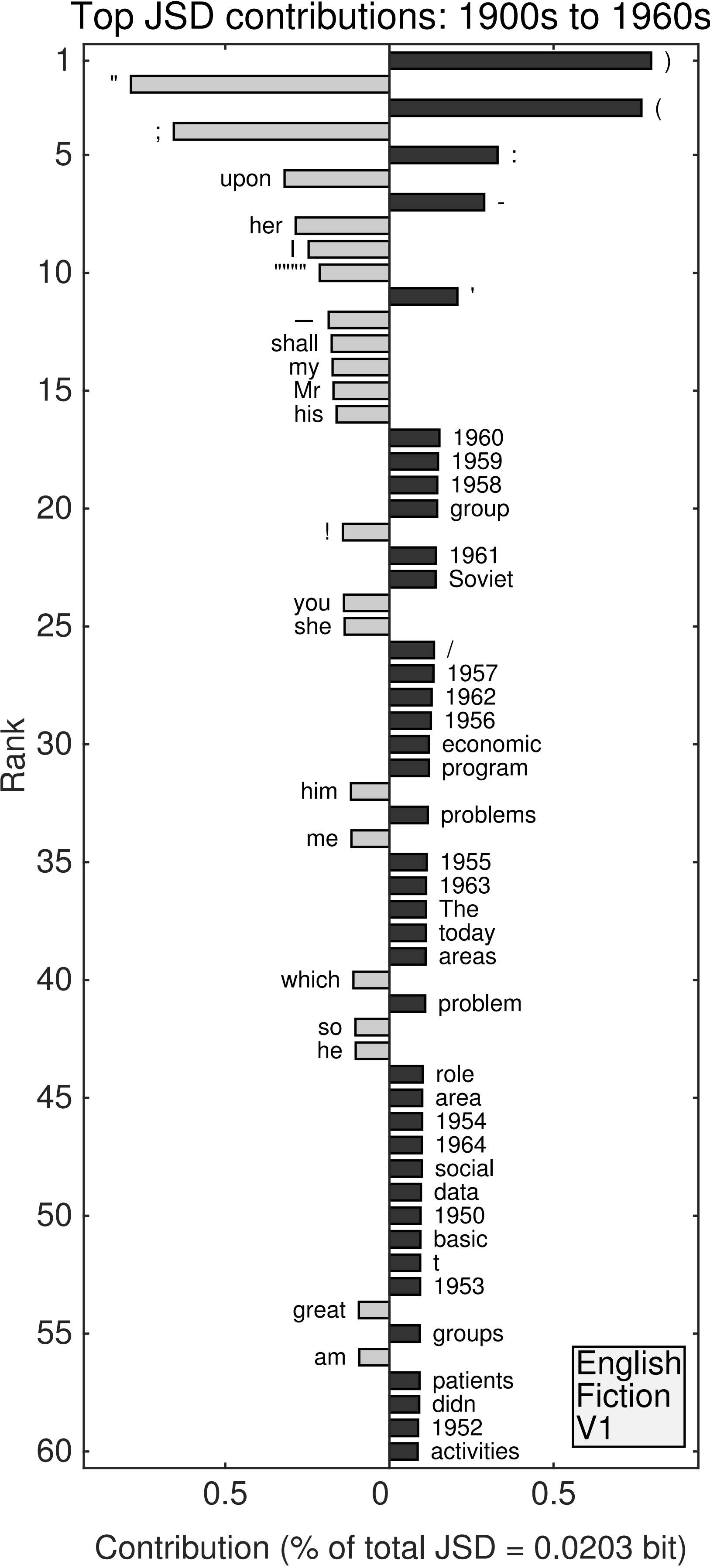

Top JSD contributions: 1900s to 1960s
Rank
Contribution (% of total JSD = 0.0203 bit)
English Fiction V1
)
"
(
;
:
upon
-
her
I
""""
'
—
shall
my
Mr
his
1960
1959
1958
group
!
1961
Soviet
you
she
/
1957
1962
1956
economic
program
him
problems
me
1955
1963
The
today
areas
which
problem
so
he
role
area
1954
1964
social
data
1950
basic
t
1953
great
groups
am
patients
didn
1952
activities

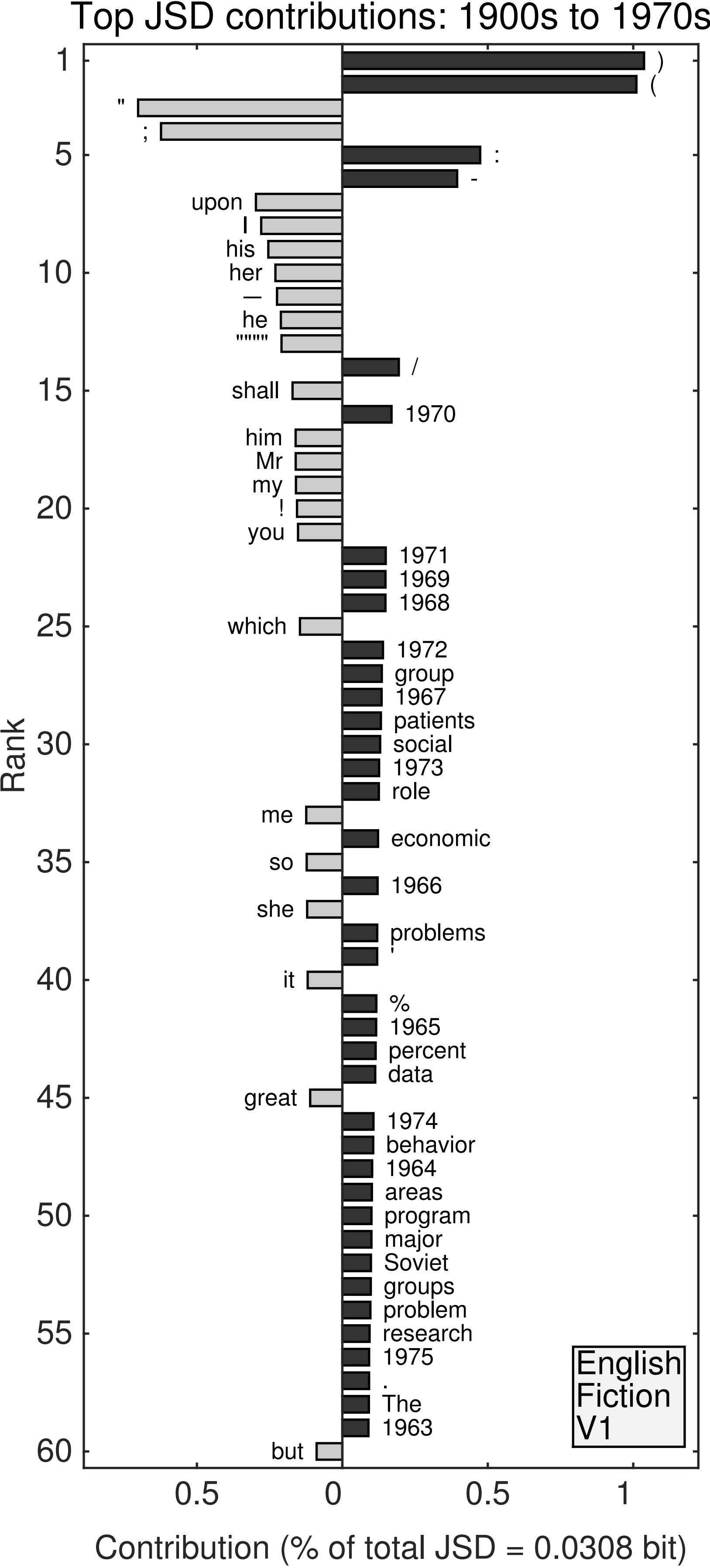

Top JSD contributions: 1900s to 1970s
Rank
Contribution (% of total JSD = 0.0308 bit)
)
(
"
;
:
-
upon
I
his
her
—
he
""""
/
shall
1970
him
Mr
my
!
you
1971
1969
1968
which
1972
group
1967
patients
social
1973
role
me
economic
so
1966
she
problems
'
it
%
1965
percent
data
great
1974
behavior
1964
areas
program
major
Soviet
groups
problem
research
1975
.
The
1963
but
English Fiction V1

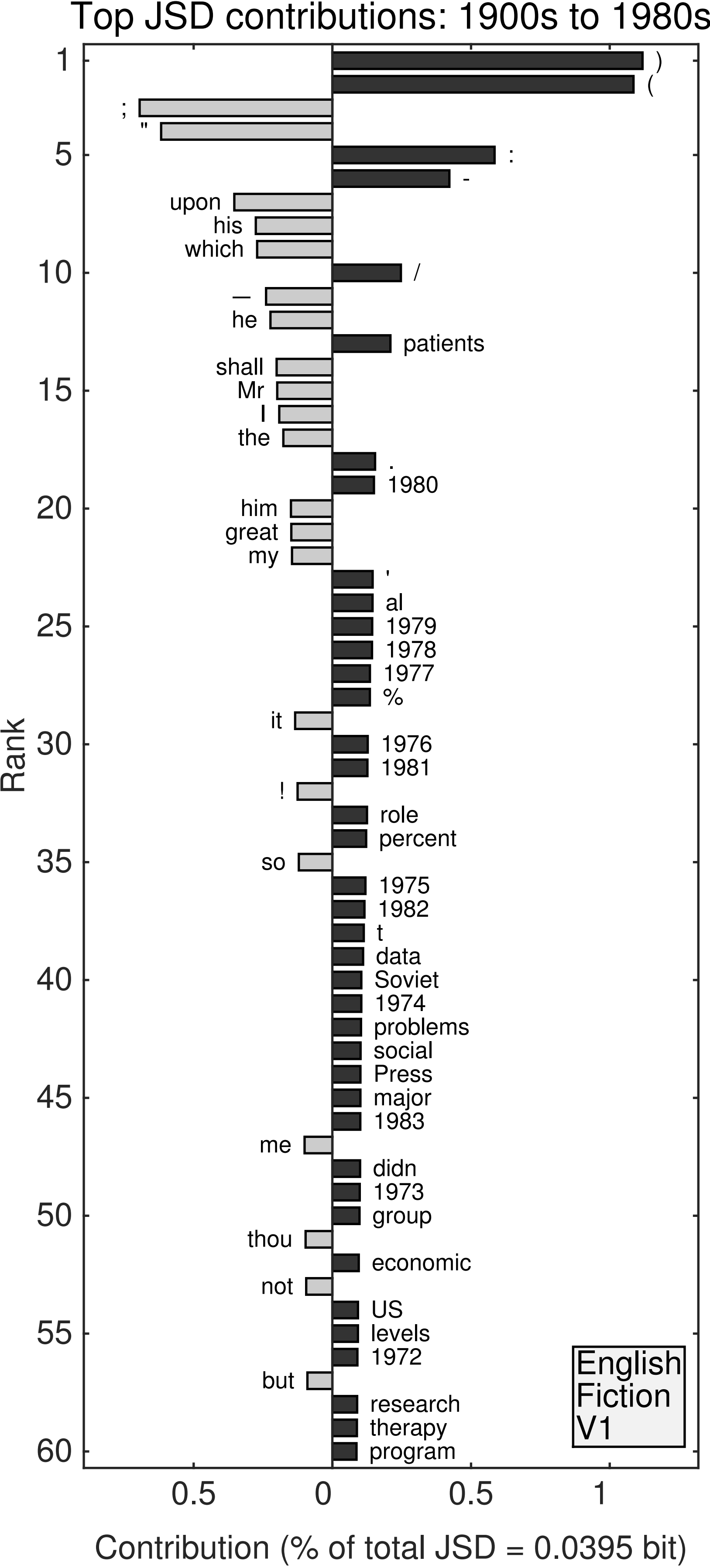

Top JSD contributions: 1900s to 1980s
Rank
Contribution (% of total JSD = 0.0395 bit)
)
(
;
"
:
-
upon
his
which
/
—
he
patients
shall
Mr
I
the
.
1980
him
great
my
'
al
1979
1978
1977
%
it
1976
1981
!
role
percent
so
1975
1982
t
data
Soviet
1974
problems
social
Press
major
1983
me
didn
1973
group
thou
economic
not
US
levels
1972
but
research
therapy
program
English
Fiction
V1

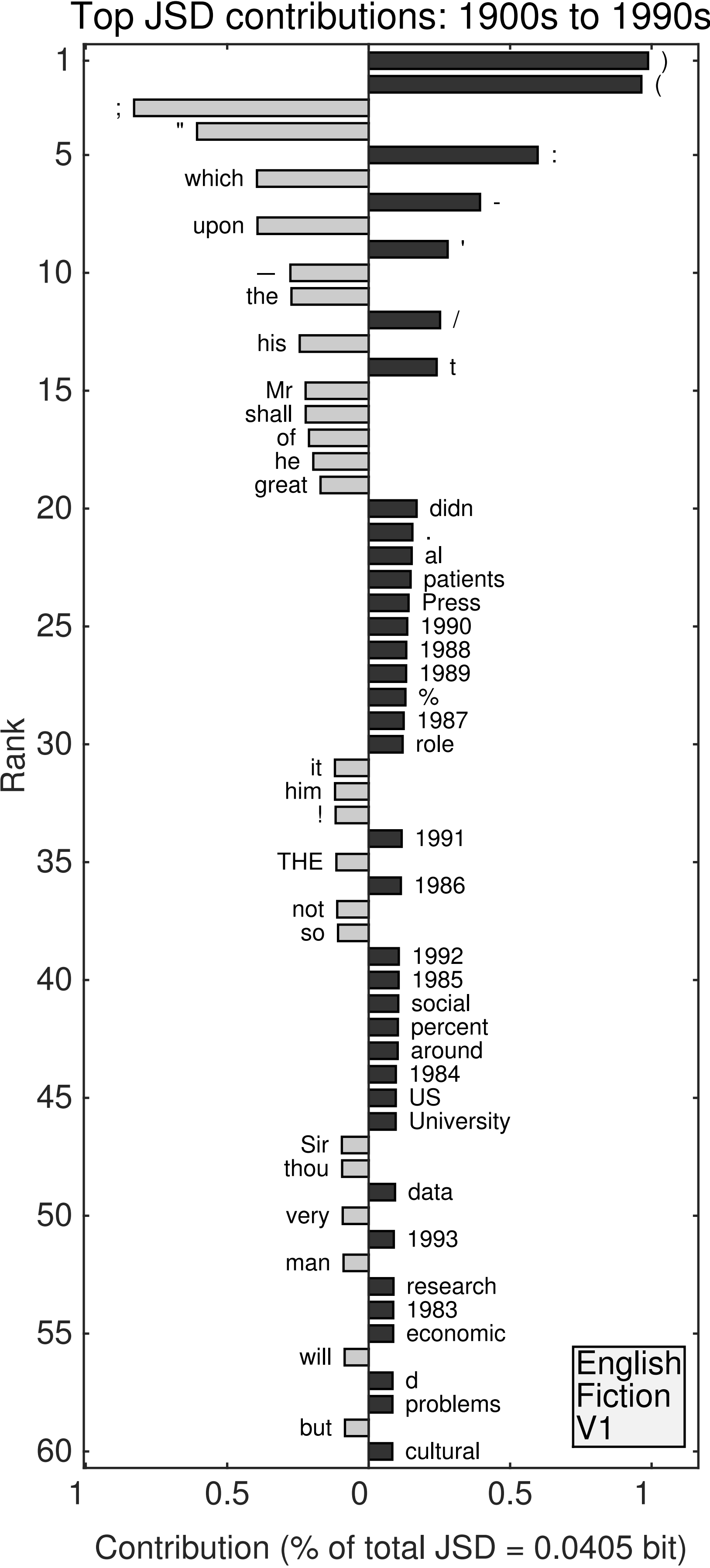

Top JSD contributions: 1900s to 1990s
Rank
Contribution (% of total JSD = 0.0405 bit)
1
5
10
15
20
25
30
35
40
45
50
55
60
1
0.5
0
0.5
1
)
(
;
"
:
which
-
upon
'
—
the
/
his
t
Mr
shall
of
he
great
didn
.
al
patients
Press
1990
1988
1989
%
1987
role
it
him
!
1991
THE
1986
not
so
1992
1985
social
percent
around
1984
US
University
Sir
thou
data
very
1993
man
research
1983
economic
will
d
problems
but
cultural
English Fiction V1

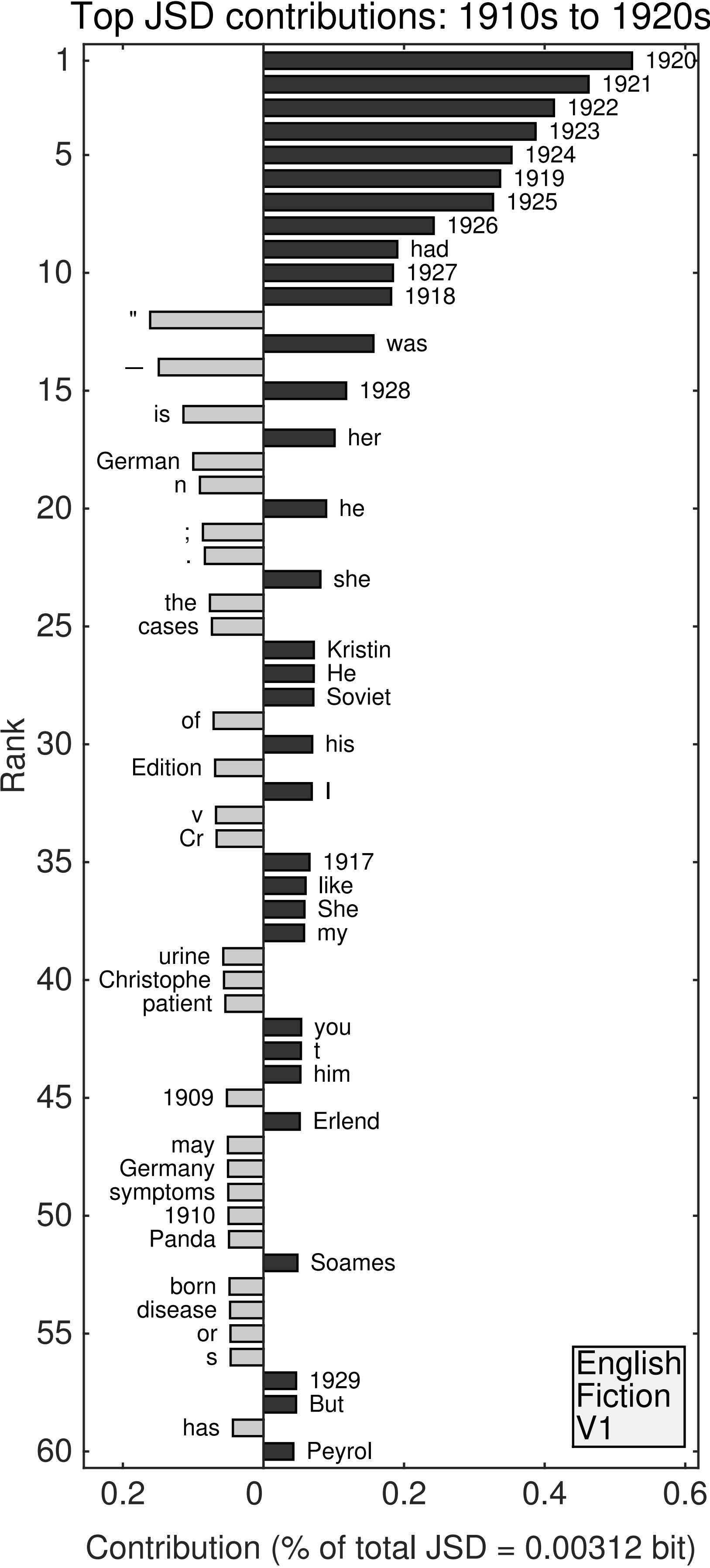

Top JSD contributions: 1910s to 1920s
Rank
Contribution (% of total JSD = 0.00312 bit)
1920
1921
1922
1923
1924
1919
1925
1926
had
1927
1918
"
was
—
1928
is
her
German
n
he
;
.
she
the
cases
Kristin
He
Soviet
of
his
Edition
I
v
Cr
1917
like
She
my
urine
Christophe
patient
you
t
him
1909
Erlend
may
Germany
symptoms
1910
Panda
Soames
born
disease
or
s
1929
But
has
Peyrol
English Fiction V1

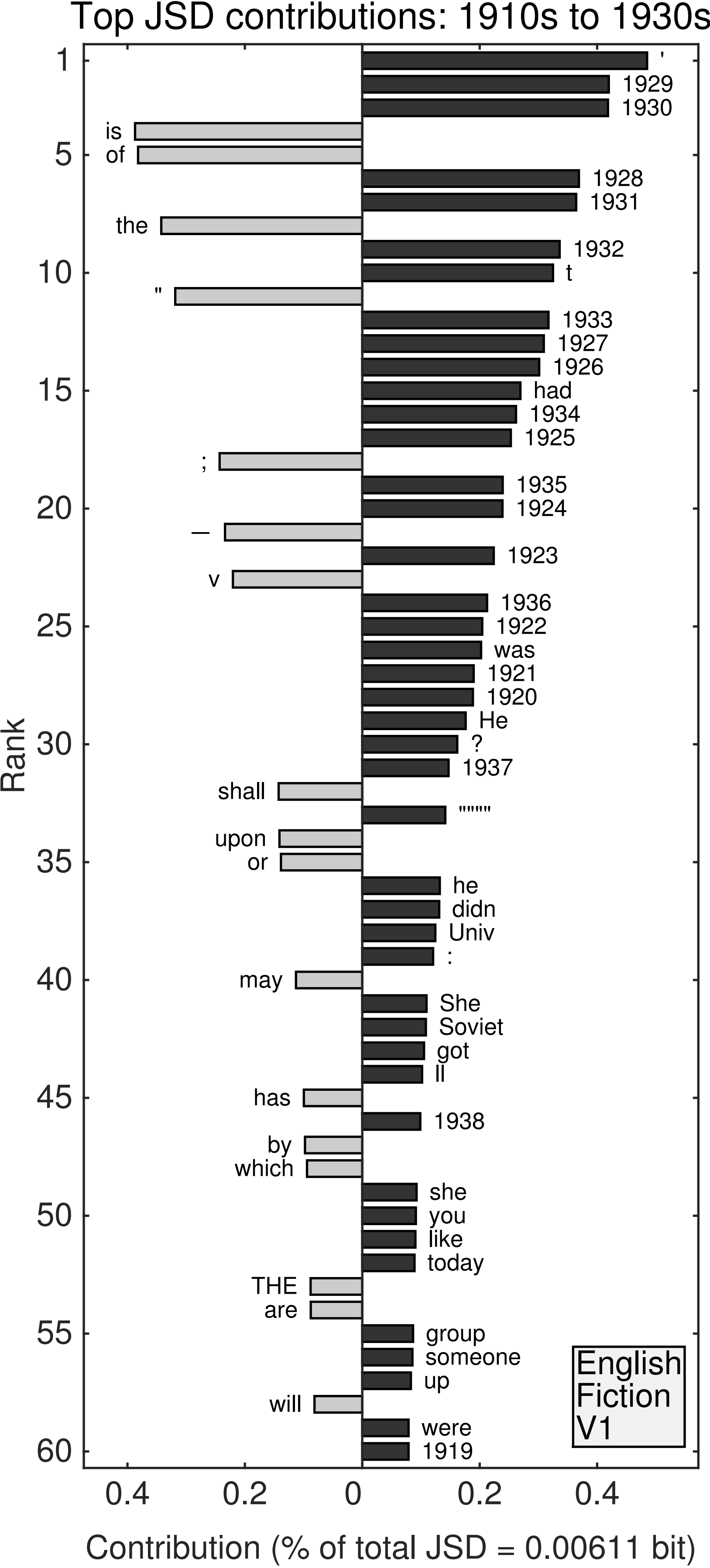

Top JSD contributions: 1910s to 1930s
Rank
Contribution (% of total JSD = 0.00611 bit)
0.4
0.2
0
0.2
0.4
1
5
10
15
20
25
30
35
40
45
50
55
60
'
1929
1930
is
of
1928
1931
the
1932
t
"
1933
1927
1926
had
1934
1925
;
1935
1924
—
1923
v
1936
1922
was
1921
1920
He
?
1937
shall
""""
upon
or
he
didn
Univ
:
may
She
Soviet
got
ll
has
1938
by
which
she
you
like
today
THE
are
group
someone
up
will
were
1919
English Fiction V1

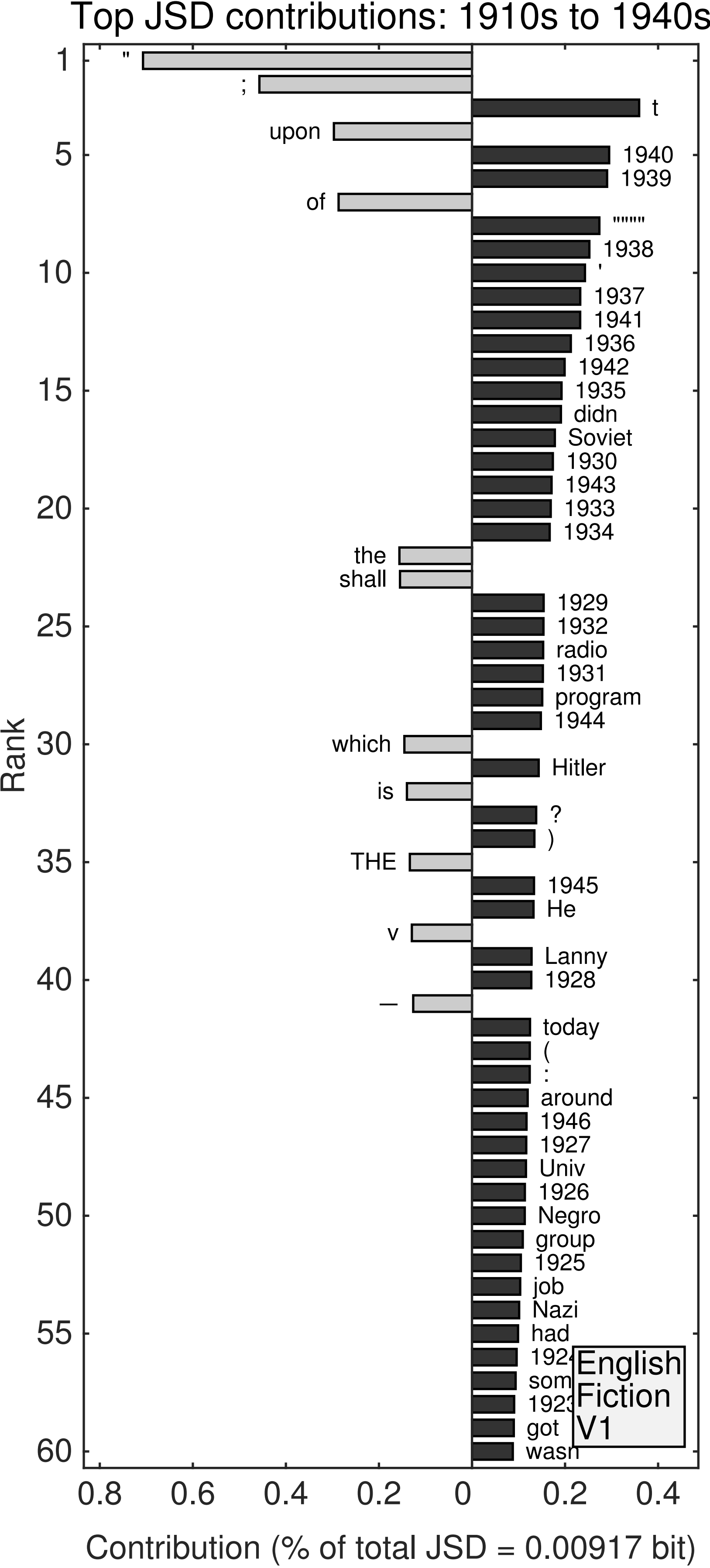

Top JSD contributions: 1910s to 1940s
Rank
Contribution (% of total JSD = 0.00917 bit)
English Fiction V1
"
;
t
upon
1940
1939
of
""""
1938
'
1937
1941
1936
1942
1935
didn
Soviet
1930
1943
1933
1934
the
shall
1929
1932
radio
1931
program
1944
which
Hitler
is
?
)
THE
1945
He
v
Lanny
1928
—
today
(
:
around
1946
1927
Univ
1926
Negro
group
1925
job
Nazi
had
got

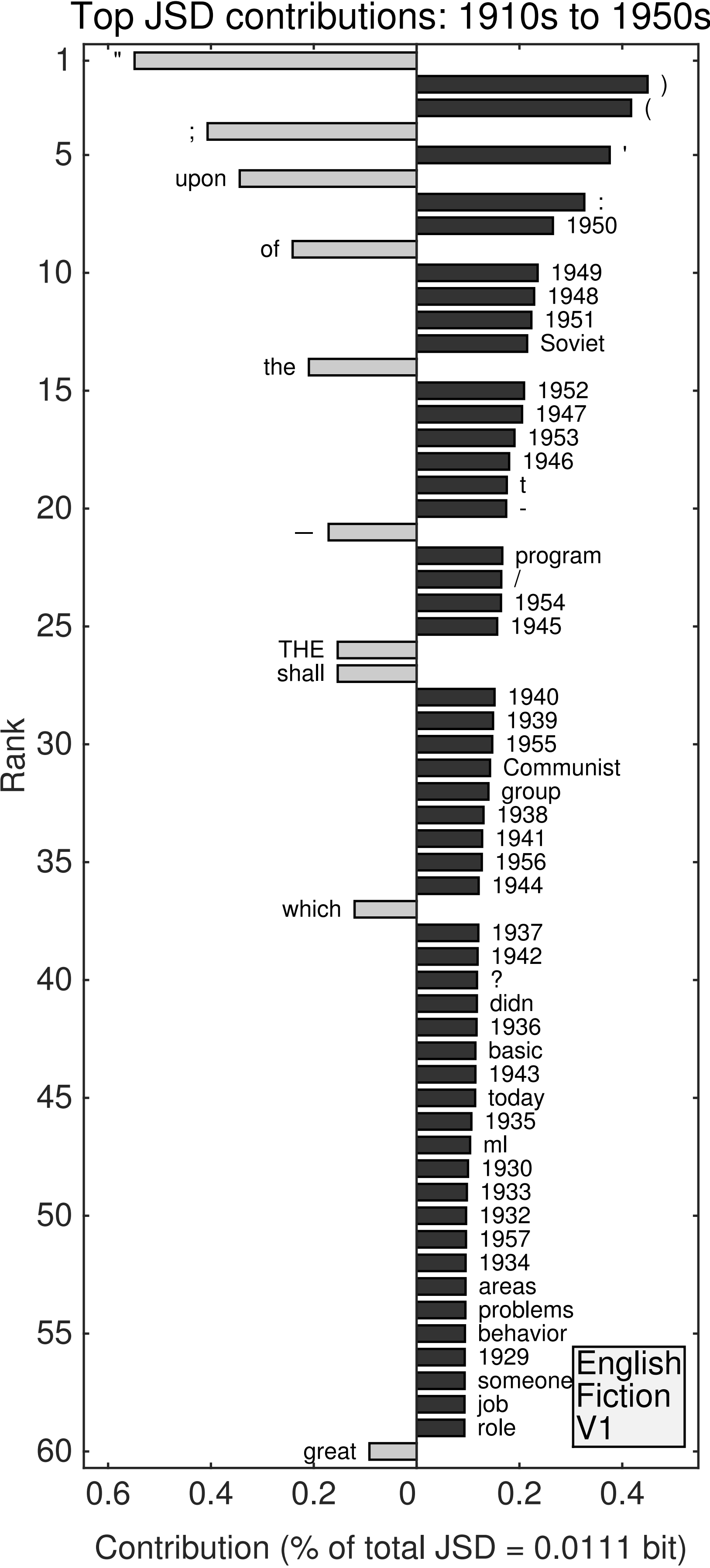

Top JSD contributions: 1910s to 1950s
Rank
Contribution (% of total JSD = 0.0111 bit)
"
)
(
;
'
upon
:
1950
of
1949
1948
1951
Soviet
the
1952
1947
1953
1946
t
-
—
program
/
1954
1945
THE
shall
1940
1939
1955
Communist
group
1938
1941
1956
1944
which
1937
1942
?
didn
1936
basic
1943
today
1935
ml
1930
1933
1932
1957
1934
areas
problems
behavior
1929
someone
job
role
great
English
Fiction
V1

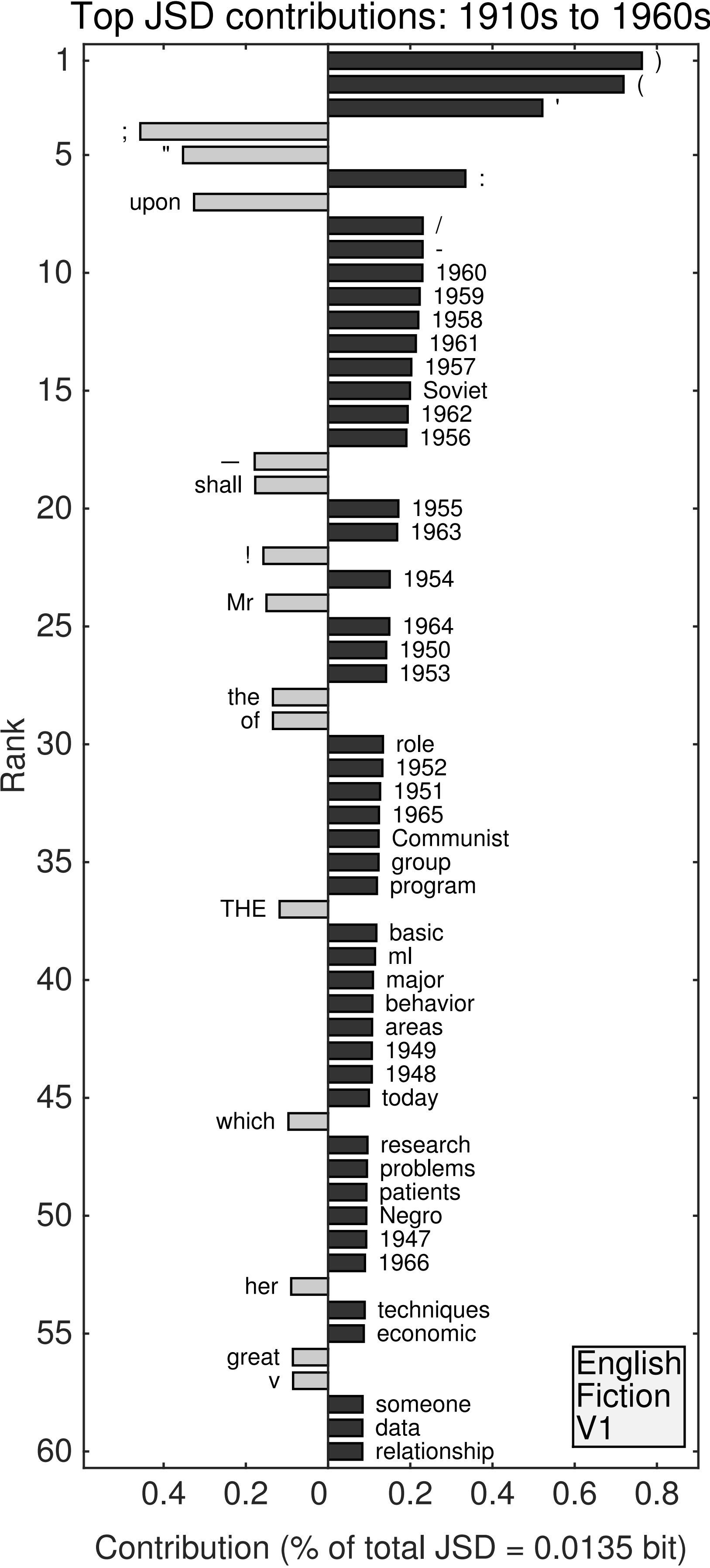
Top JSD contributions: 1910s to 1960s
Rank
Contribution (% of total JSD = 0.0135 bit)
)
(
'
;
"
:
upon
/
-
1960
1959
1958
1961
1957
Soviet
1962
1956
—
shall
1955
1963
!
1954
Mr
1964
1950
1953
the
of
role
1952
1951
1965
Communist
group
program
THE
basic
ml
major
behavior
areas
1949
1948
today
which
research
problems
patients
Negro
1947
1966
her
techniques
economic
great
v
someone
data
relationship
English Fiction V1

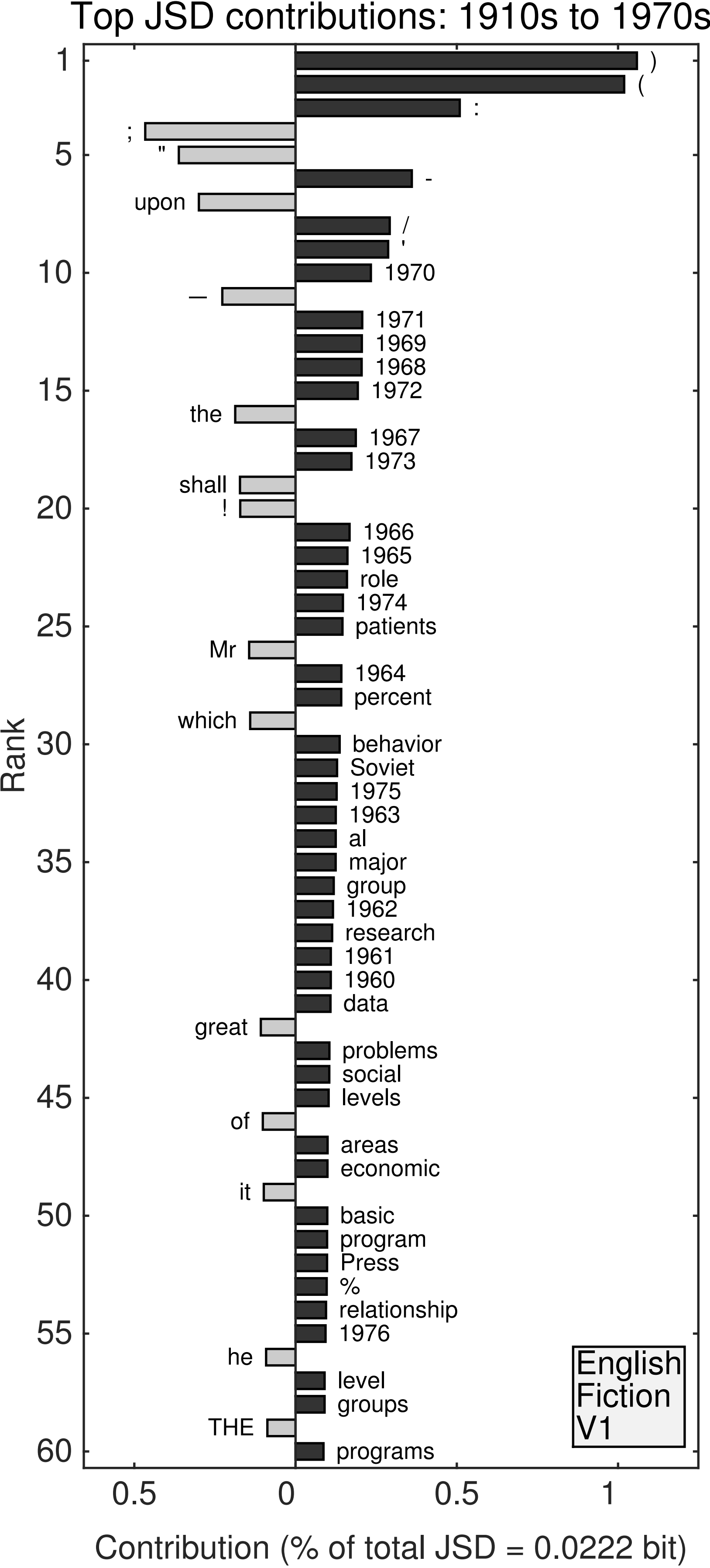

Top JSD contributions: 1910s to 1970s
Rank
Contribution (% of total JSD = 0.0222 bit)
English Fiction V1
)
(
:
;
"
-
upon
/
'
1970
—
1971
1969
1968
1972
the
1967
1973
shall
!
1966
1965
role
1974
patients
Mr
1964
percent
which
behavior
Soviet
1975
1963
al
major
group
1962
research
1961
1960
data
great
problems
social
levels
of
areas
economic
it
basic
program
Press
%
relationship
1976
he
level
groups
THE
programs

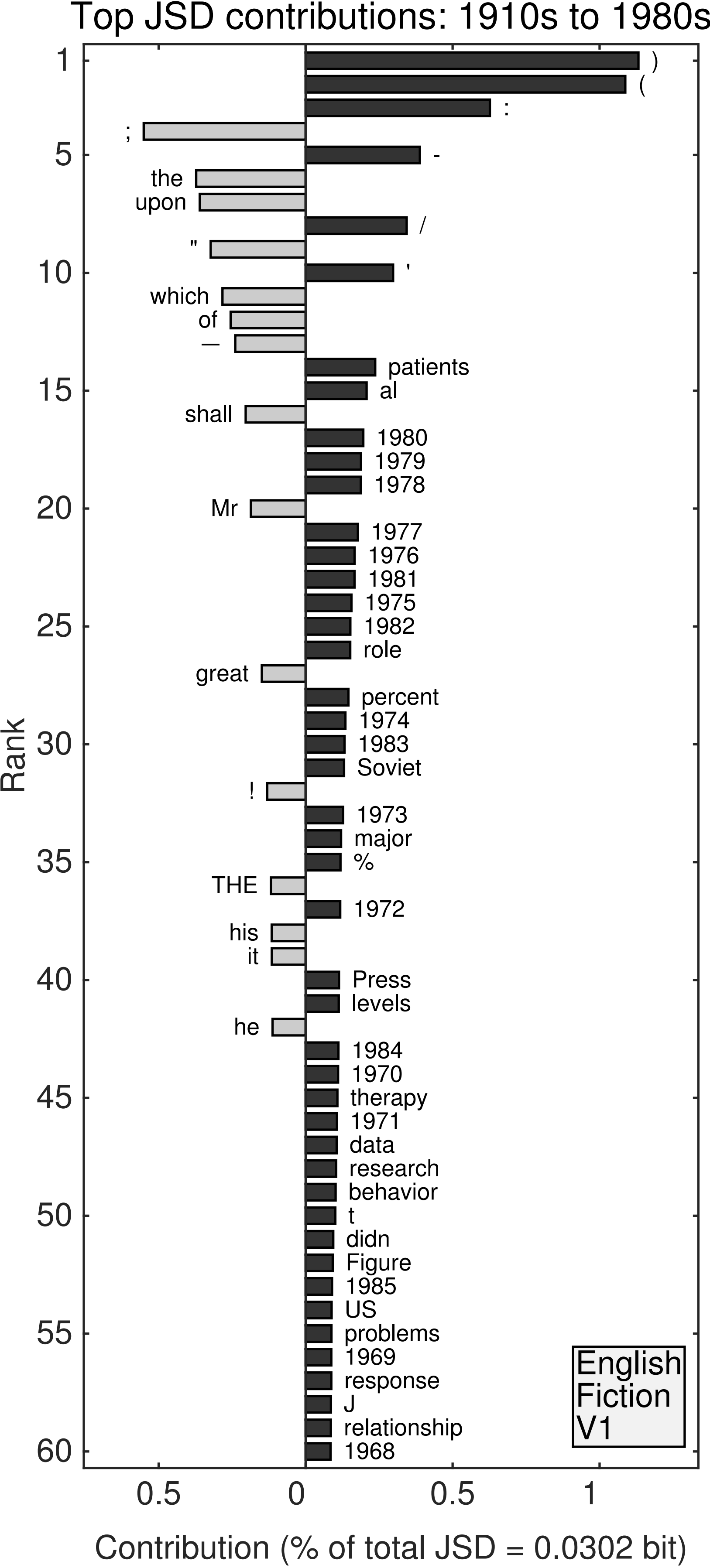
Top JSD contributions: 1910s to 1980s
Rank
Contribution (% of total JSD = 0.0302 bit)
English
Fiction
V1
)
(
:
;
-
the
upon
/
"
'
which
of
—
patients
al
shall
1980
1979
1978
Mr
1977
1976
1981
1975
1982
role
great
percent
1974
1983
Soviet
!
1973
major
%
THE
1972
his
it
Press
levels
he
1984
1970
therapy
1971
data
research
behavior
t
didn
Figure
1985
US
problems
1969
response
J
relationship
1968

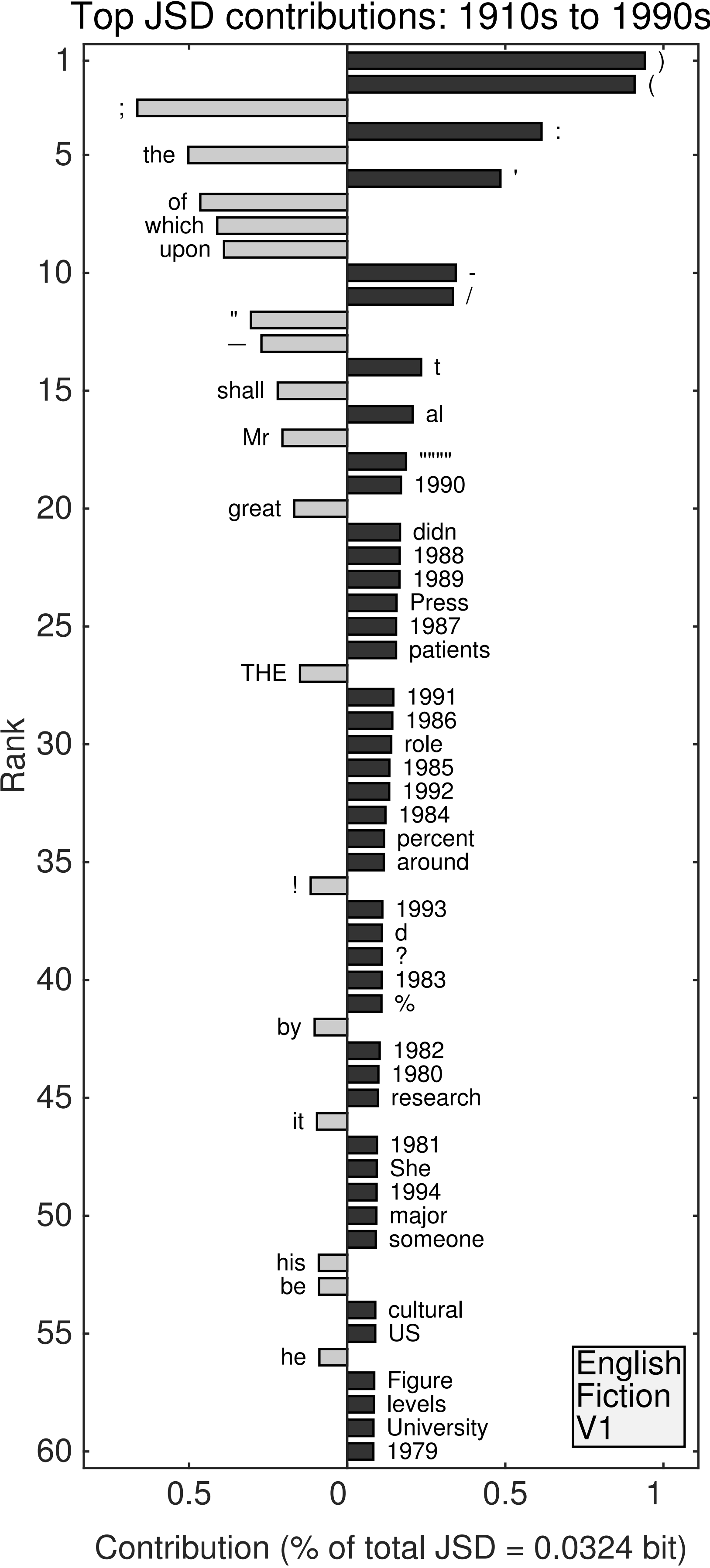

Top JSD contributions: 1910s to 1990s
Rank
Contribution (% of total JSD = 0.0324 bit)
English Fiction V1
)
(
;
:
the
'
of
which
upon
-
/
"
—
t
shall
al
Mr
""""
1990
great
didn
1988
1989
Press
1987
patients
THE
1991
1986
role
1985
1992
1984
percent
around
!
1993
d
?
1983
%
by
1982
1980
research
it
1981
She
1994
major
someone
his
be
cultural
US
he
Figure
levels
University
1979

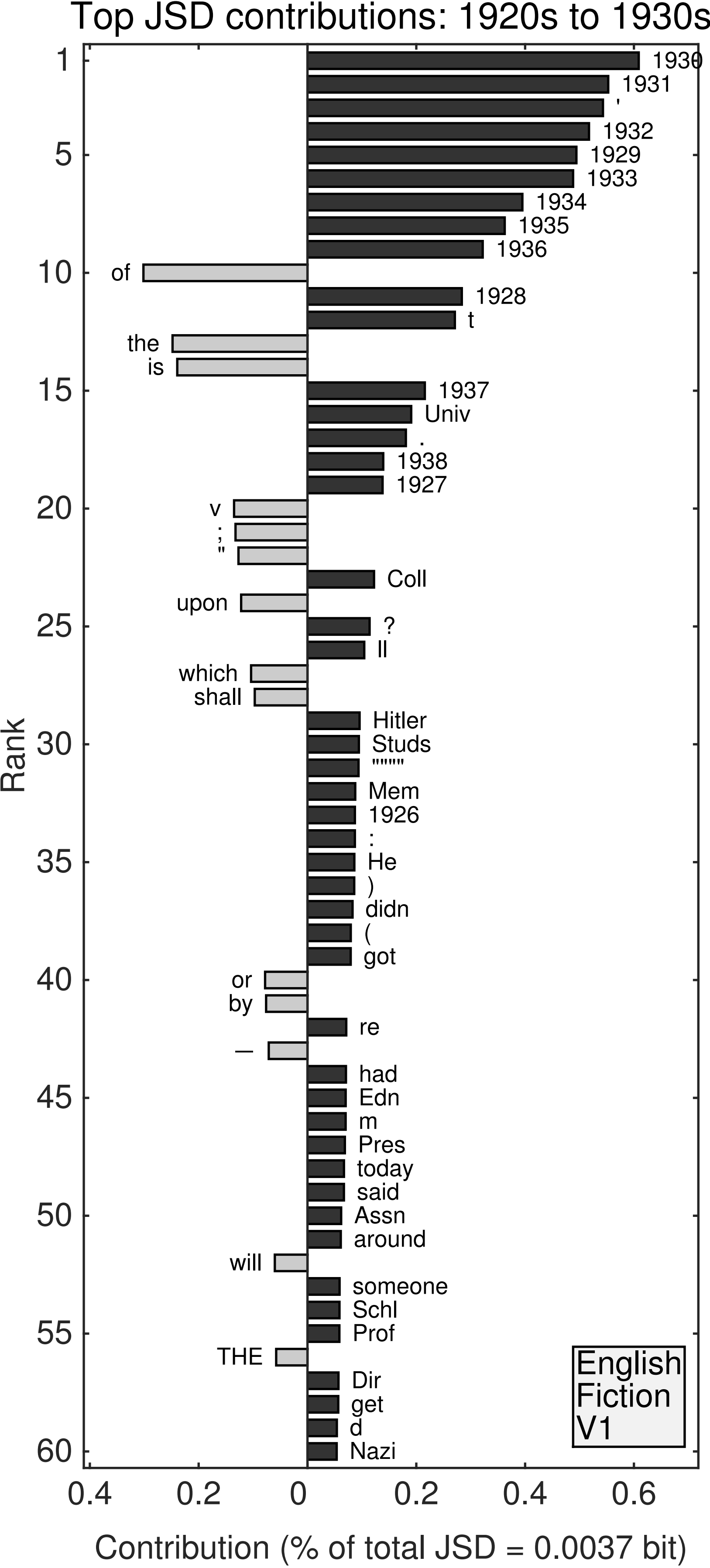

Top JSD contributions: 1920s to 1930s
Rank
Contribution (% of total JSD = 0.0037 bit)
1930
1931
'
1932
1929
1933
1934
1935
1936
of
1928
t
the
is
1937
Univ
.
1938
1927
v
;
"
Coll
upon
?
ll
which
shall
Hitler
Studs
""""
Mem
1926
:
He
)
didn
(
got
or
by
re
—
had
Edn
m
Pres
today
said
Assn
around
will
someone
Schl
Prof
THE
Dir
get
d
Nazi
English Fiction V1

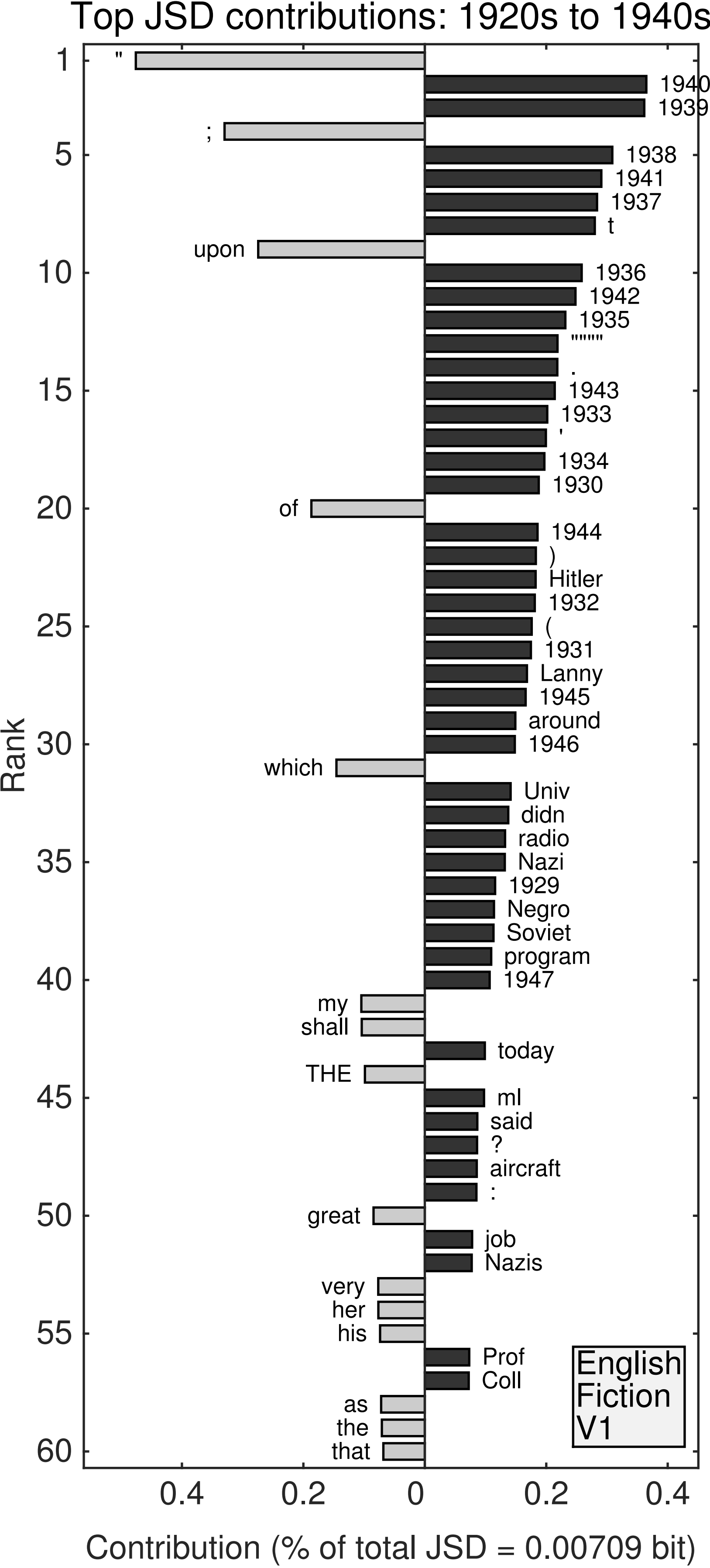

Top JSD contributions: 1920s to 1940s
Rank
Contribution (% of total JSD = 0.00709 bit)
"
1940
1939
;
1938
1941
1937
t
upon
1936
1942
1935
""""
.
1943
1933
'
1934
1930
of
1944
)
Hitler
1932
(
1931
Lanny
1945
around
1946
which
Univ
didn
radio
Nazi
1929
Negro
Soviet
program
1947
my
shall
today
THE
ml
said
?
aircraft
:
great
job
Nazis
very
her
his
Prof
Coll
as
the
that
English Fiction V1

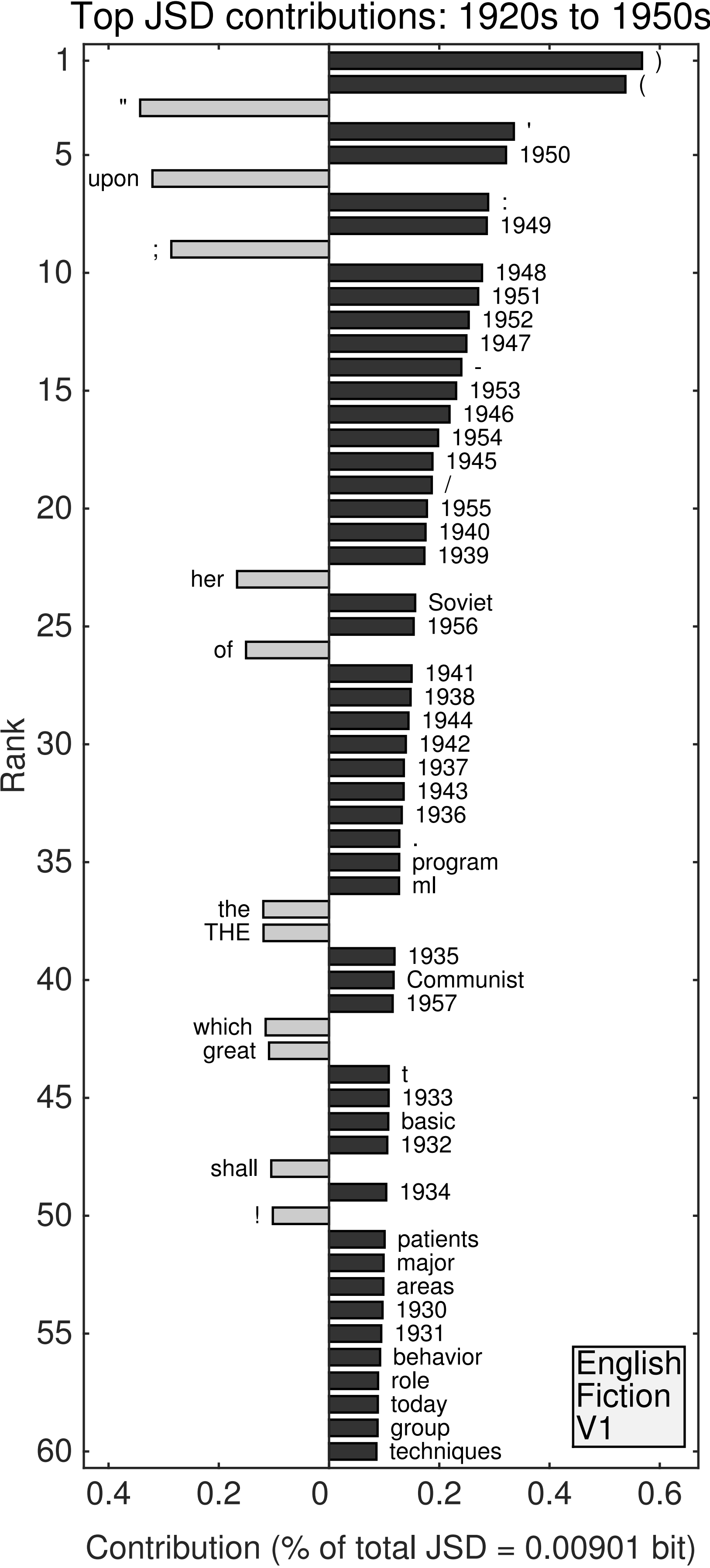

Top JSD contributions: 1920s to 1950s
Rank
Contribution (% of total JSD = 0.00901 bit)
English Fiction V1
)
(
"
'
1950
upon
:
1949
;
1948
1951
1952
1947
-
1953
1946
1954
1945
/
1955
1940
1939
her
Soviet
1956
of
1941
1938
1944
1942
1937
1943
1936
.
program
ml
the
THE
1935
Communist
1957
which
great
t
1933
basic
1932
shall
1934
!
patients
major
areas
1930
1931
behavior
role
today
group
techniques

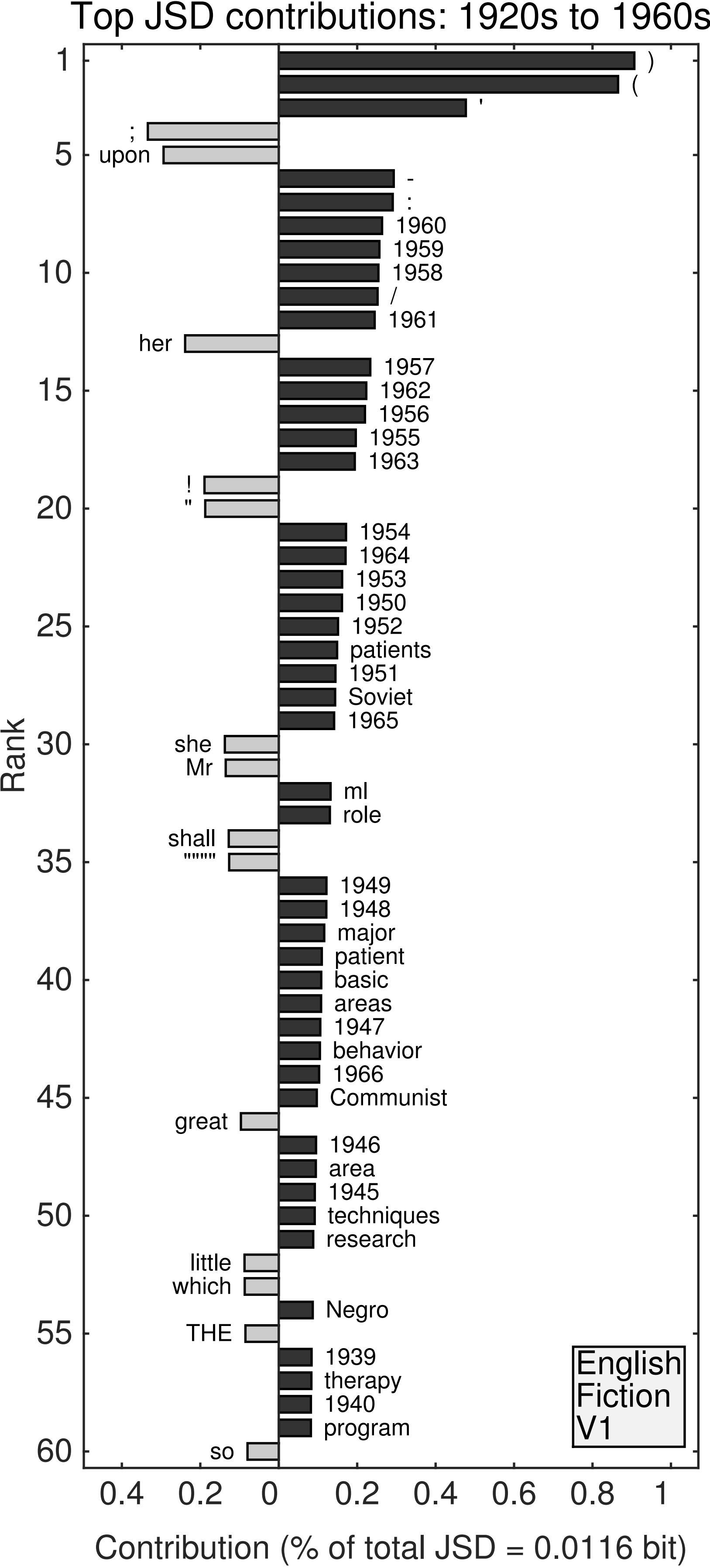

Top JSD contributions: 1920s to 1960s
Rank
1
5
10
15
20
25
30
35
40
45
50
55
60
)
(
'
;
upon
-
:
1960
1959
1958
/
1961
her
1957
1962
1956
1955
1963
!
"
1954
1964
1953
1950
1952
patients
1951
Soviet
1965
she
Mr
ml
role
shall
""""
1949
1948
major
patient
basic
areas
1947
behavior
1966
Communist
great
1946
area
1945
techniques
research
little
which
Negro
THE
1939
therapy
1940
program
so
English Fiction V1
0.4
0.2
0
0.2
0.4
0.6
0.8
1
Contribution (% of total JSD = 0.0116 bit)

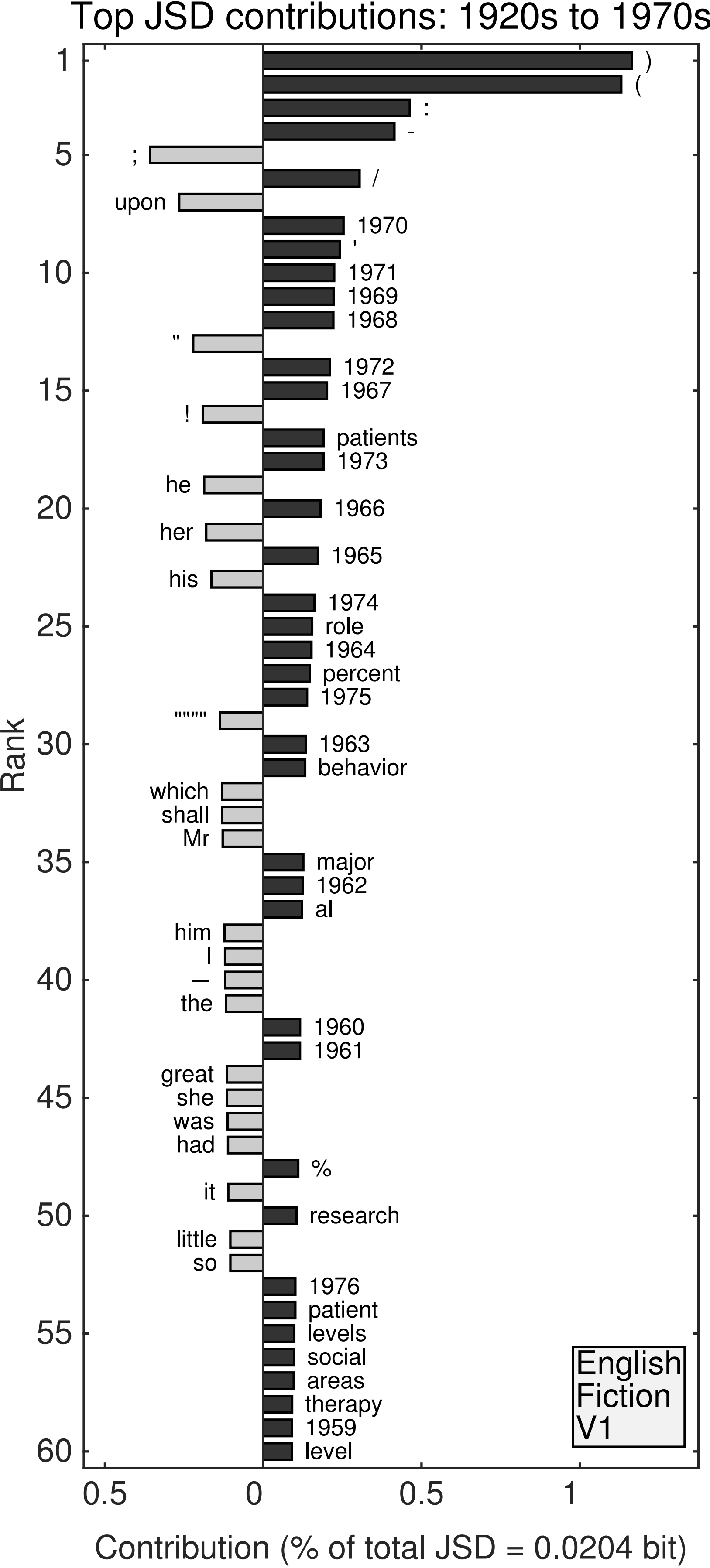

Top JSD contributions: 1920s to 1970s
Rank
Contribution (% of total JSD = 0.0204 bit)
English Fiction V1
)
(
:
-
;
/
upon
1970
'
1971
1969
1968
"
1972
1967
!
patients
1973
he
1966
her
1965
his
1974
role
1964
percent
1975
""""
1963
behavior
which
shall
Mr
major
1962
al
him
I
—
the
1960
1961
great
she
was
had
%
it
research
little
so
1976
patient
levels
social
areas
therapy
1959
level

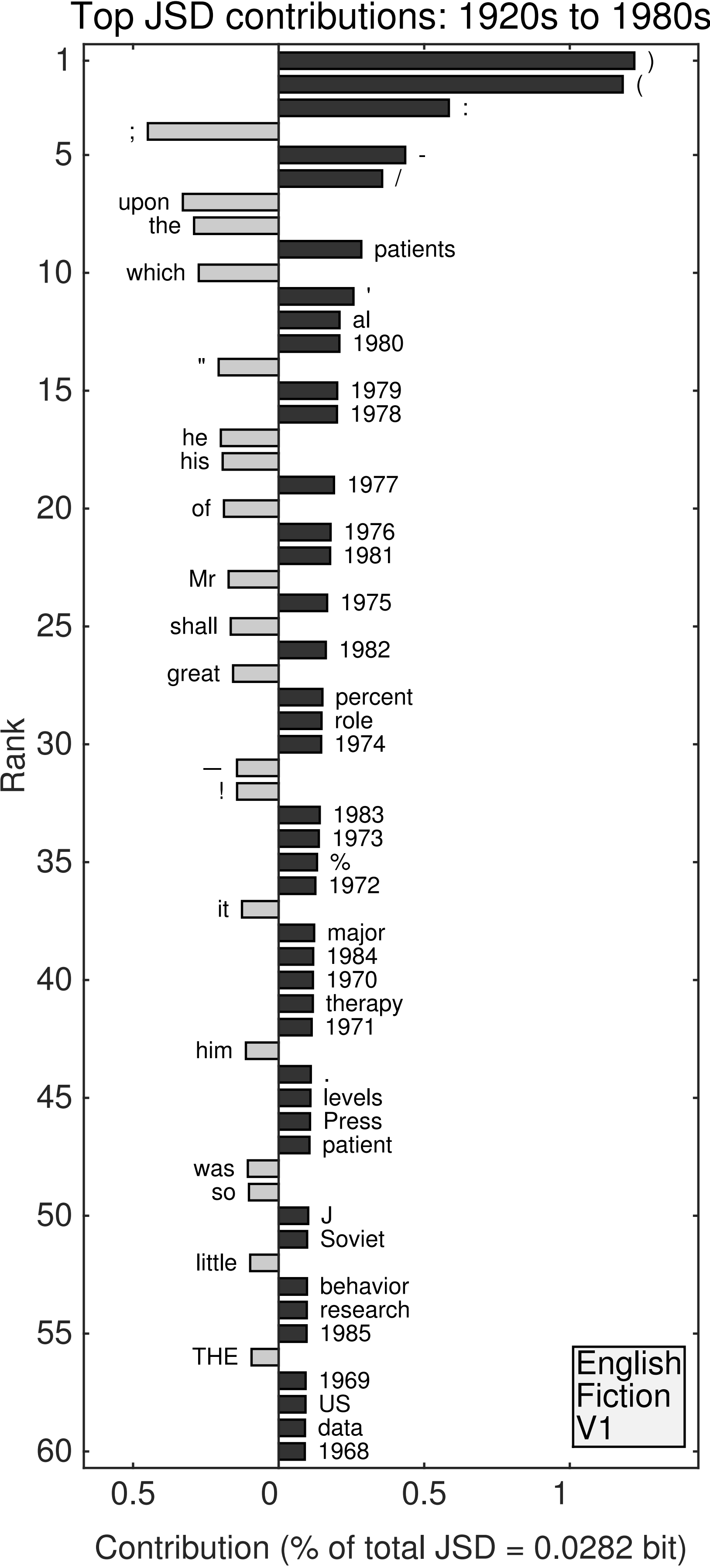
Top JSD contributions: 1920s to 1980s
Rank
Contribution (% of total JSD = 0.0282 bit)
0.5
0
0.5
1
1
5
10
15
20
25
30
35
40
45
50
55
60
)
(
:
;
-
/
upon
the
patients
which
'
al
1980
"
1979
1978
he
his
1977
of
1976
1981
Mr
1975
shall
1982
great
percent
role
1974
—
!
1983
1973
%
1972
it
major
1984
1970
therapy
1971
him
.
levels
Press
patient
was
so
J
Soviet
little
behavior
research
1985
THE
1969
US
data
1968
English
Fiction
V1

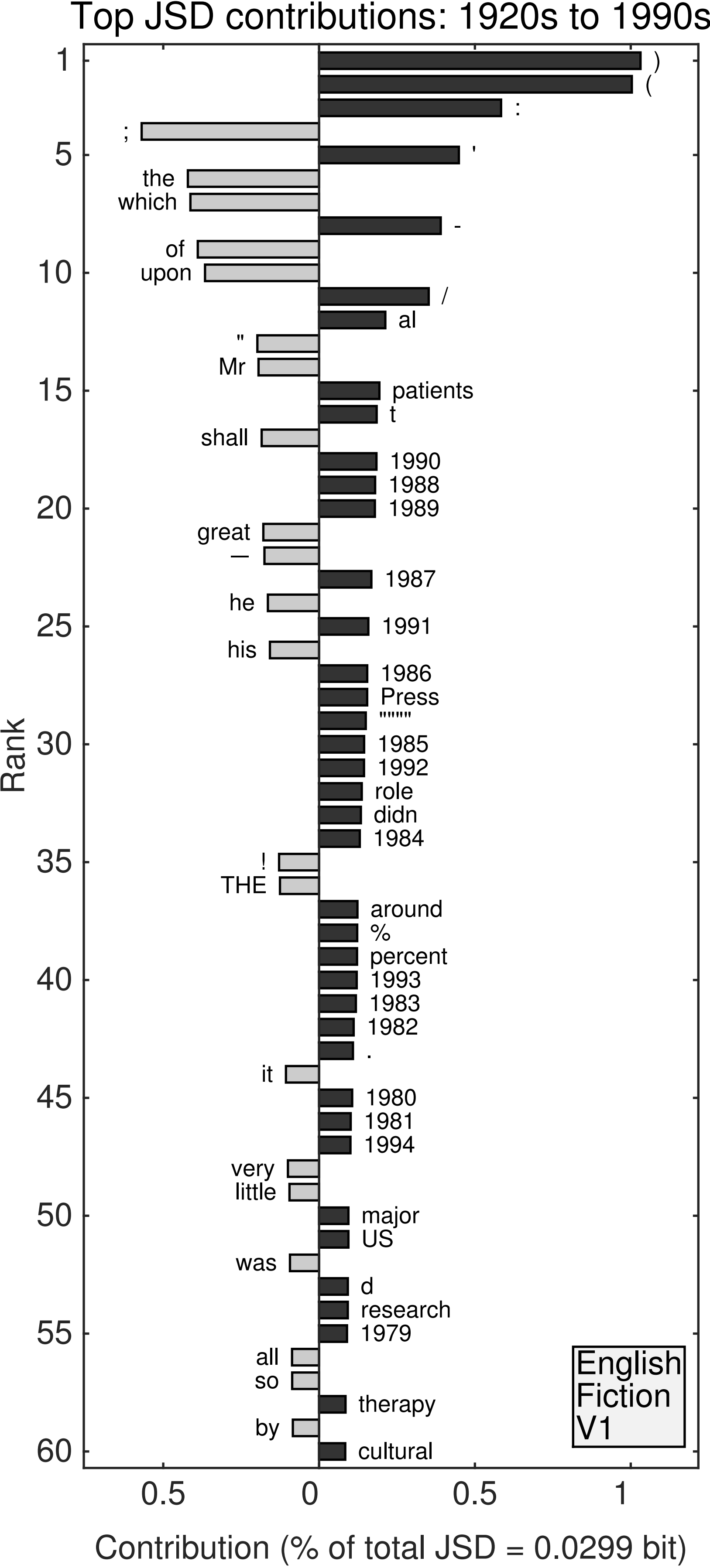
Top JSD contributions: 1920s to 1990s
Rank
Contribution (% of total JSD = 0.0299 bit)
English Fiction V1
)
(
:
;
'
the
which
-
of
upon
/
al
"
Mr
patients
t
shall
1990
1988
1989
great
—
1987
he
1991
his
1986
Press
""""
1985
1992
role
didn
1984
!
THE
around
%
percent
1993
1983
1982
.
it
1980
1981
1994
very
little
major
US
was
d
research
1979
all
so
therapy
by
cultural

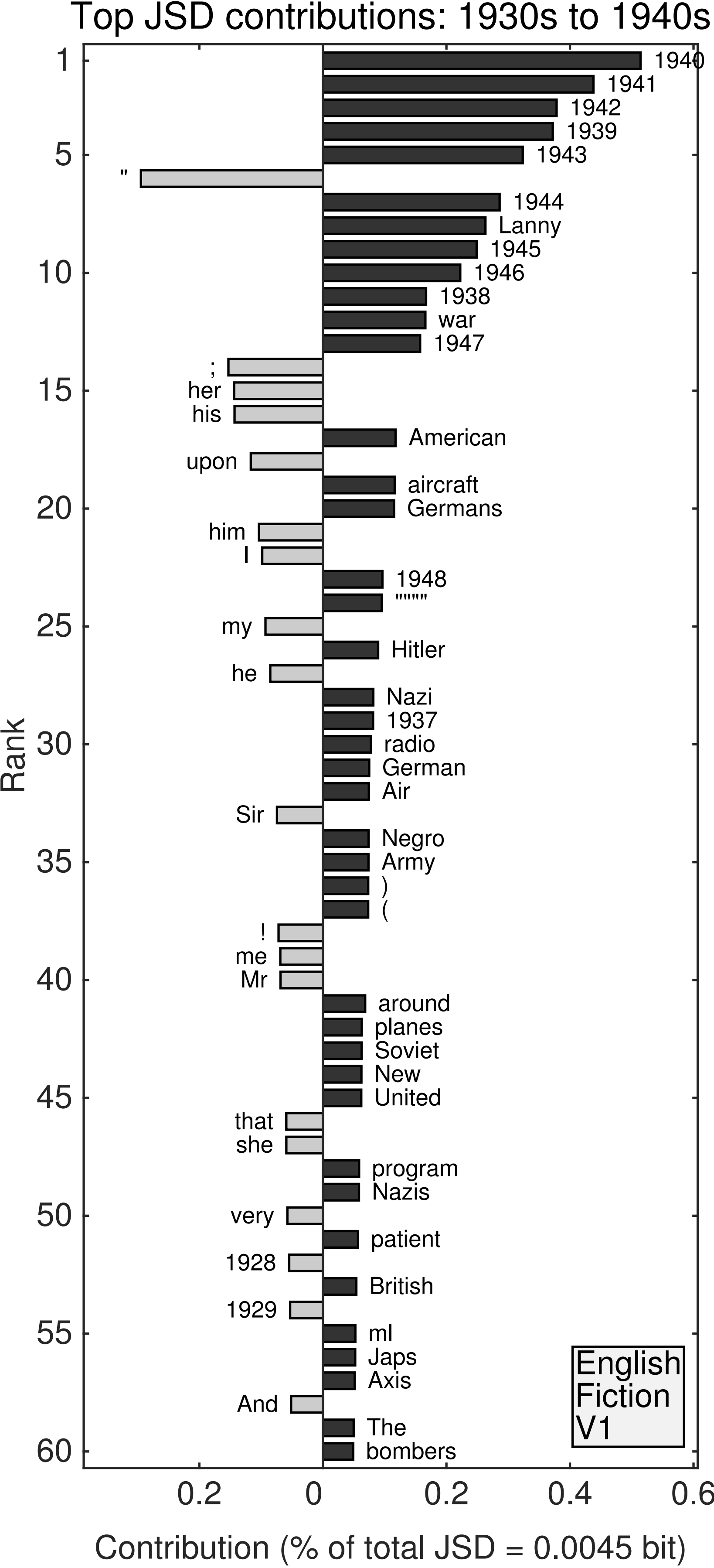
Top JSD contributions: 1930s to 1940s
Rank
Contribution (% of total JSD = 0.0045 bit)
English Fiction V1
1940
1941
1942
1939
1943
"
1944
Lanny
1945
1946
1938
war
1947
;
her
his
American
upon
aircraft
Germans
him
I
1948
""""
my
Hitler
he
Nazi
1937
radio
German
Air
Sir
Negro
Army
)
(
!
me
Mr
around
planes
Soviet
New
United
that
she
program
Nazis
very
patient
1928
British
1929
ml
Japs
Axis
And
The
bombers

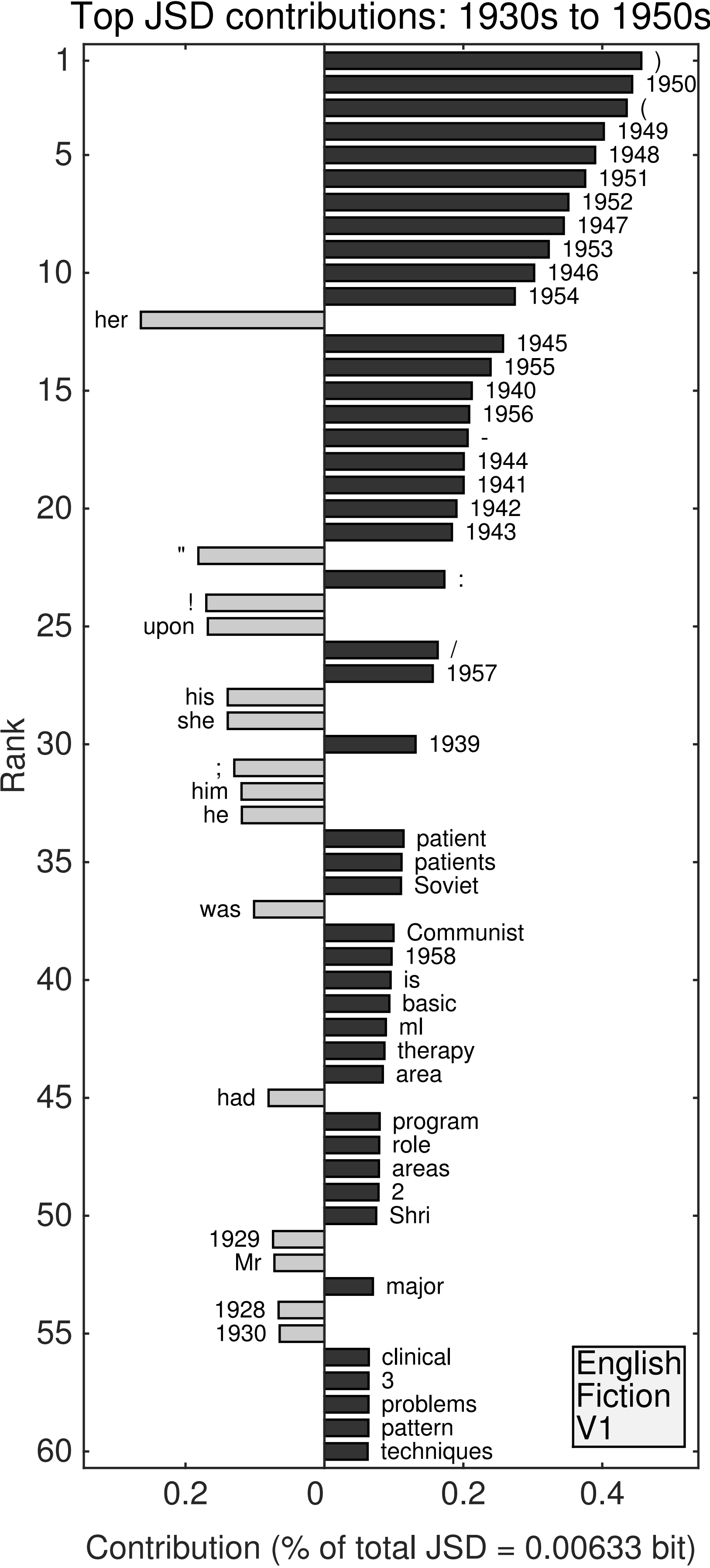

Top JSD contributions: 1930s to 1950s
Rank
)
1950
(
1949
1948
1951
1952
1947
1953
1946
1954
her
1945
1955
1940
1956
-
1944
1941
1942
1943
"
:
!
upon
/
1957
his
she
1939
;
him
he
patient
patients
Soviet
was
Communist
1958
is
basic
ml
therapy
area
had
program
role
areas
2
Shri
1929
Mr
major
1928
1930
clinical
3
problems
pattern
techniques
English Fiction V1
Contribution (% of total JSD = 0.00633 bit)

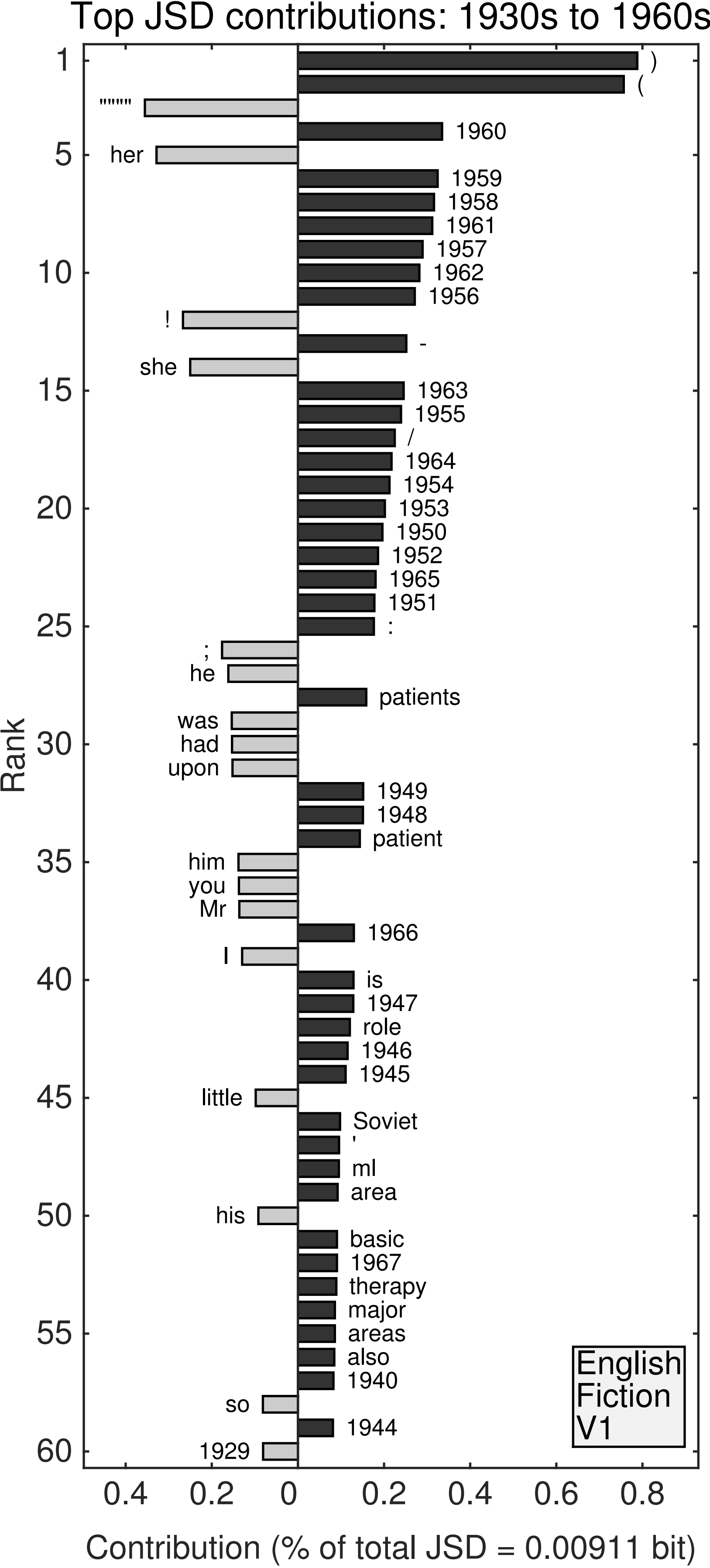
Top JSD contributions: 1930s to 1960s
Rank
1
5
10
15
20
25
30
35
40
45
50
55
60
)
(
""""
1960
her
1959
1958
1961
1957
1962
1956
!
-
she
1963
1955
/
1964
1954
1953
1950
1952
1965
1951
:
;
he
patients
was
had
upon
1949
1948
patient
him
you
Mr
1966
I
is
1947
role
1946
1945
little
Soviet
'
ml
area
his
basic
1967
therapy
major
areas
also
1940
so
1944
1929
English
Fiction
V1
0.4
0.2
0
0.2
0.4
0.6
0.8
Contribution (% of total JSD = 0.00911 bit)

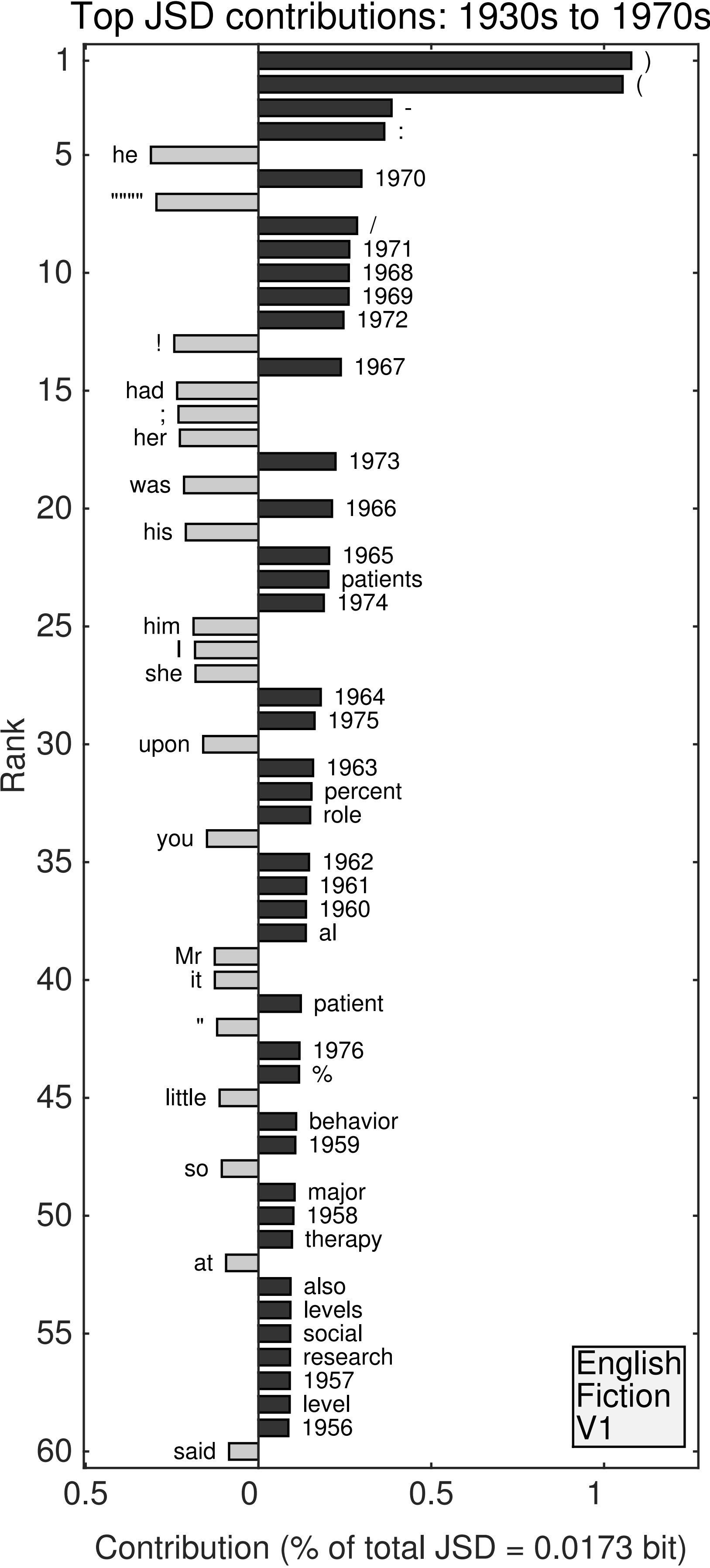

Top JSD contributions: 1930s to 1970s
Rank
Contribution (% of total JSD = 0.0173 bit)
English Fiction V1
1
5
10
15
20
25
30
35
40
45
50
55
60
0.5
0
0.5
1
)
(
-
:
he
1970
""""
/
1971
1968
1969
1972
!
1967
had
;
her
1973
was
1966
his
1965
patients
1974
him
I
she
1964
1975
upon
1963
percent
role
you
1962
1961
1960
al
Mr
it
patient
"
1976
%
little
behavior
1959
so
major
1958
therapy
at
also
levels
social
research
1957
level
1956
said

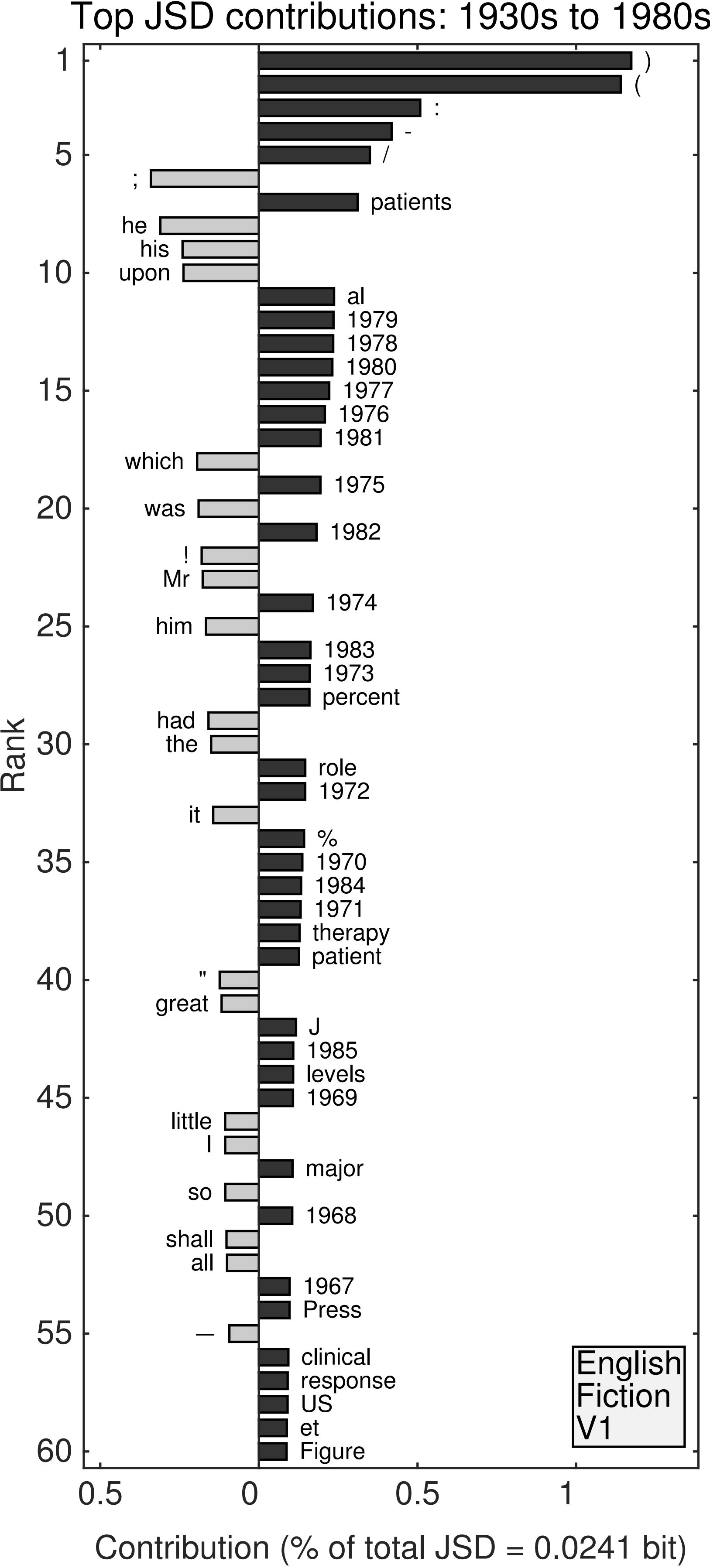

Top JSD contributions: 1930s to 1980s
Rank
)
(
:
-
/
;
patients
he
his
upon
al
1979
1978
1980
1977
1976
1981
which
1975
was
1982
!
Mr
1974
him
1983
1973
percent
had
the
role
1972
it
%
1970
1984
1971
therapy
patient
"
great
J
1985
levels
1969
little
I
major
so
1968
shall
all
1967
Press
—
clinical
response
US
et
Figure
English Fiction V1
Contribution (% of total JSD = 0.0241 bit)

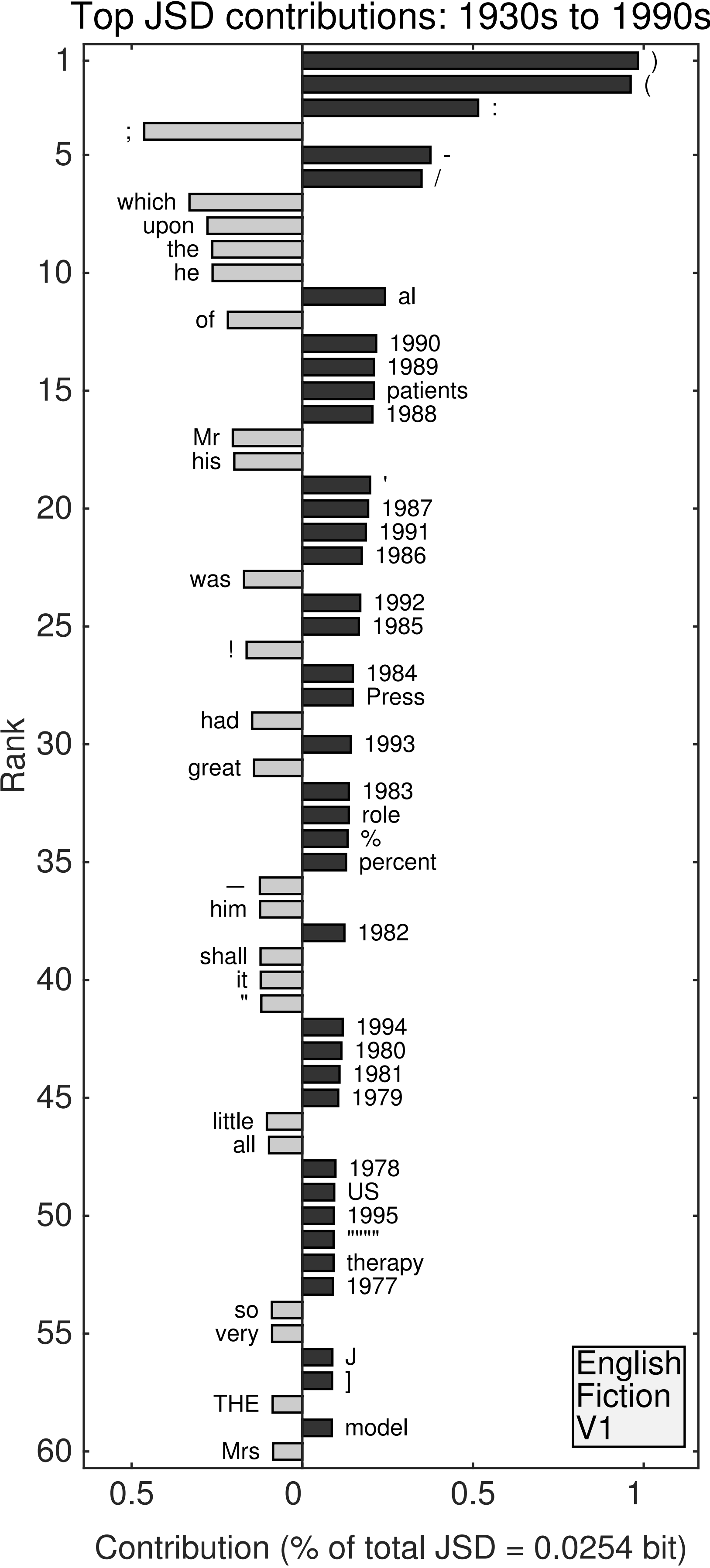

Top JSD contributions: 1930s to 1990s
Rank
Contribution (% of total JSD = 0.0254 bit)
0.5
0
0.5
1
1
5
10
15
20
25
30
35
40
45
50
55
60
)
(
:
;
-
/
which
upon
the
he
al
of
1990
1989
patients
1988
Mr
his
'
1987
1991
1986
was
1992
1985
!
1984
Press
had
1993
great
1983
role
%
percent
—
him
1982
shall
it
"
1994
1980
1981
1979
little
all
1978
US
1995
""""
therapy
1977
so
very
J
]
THE
model
Mrs
English
Fiction
V1

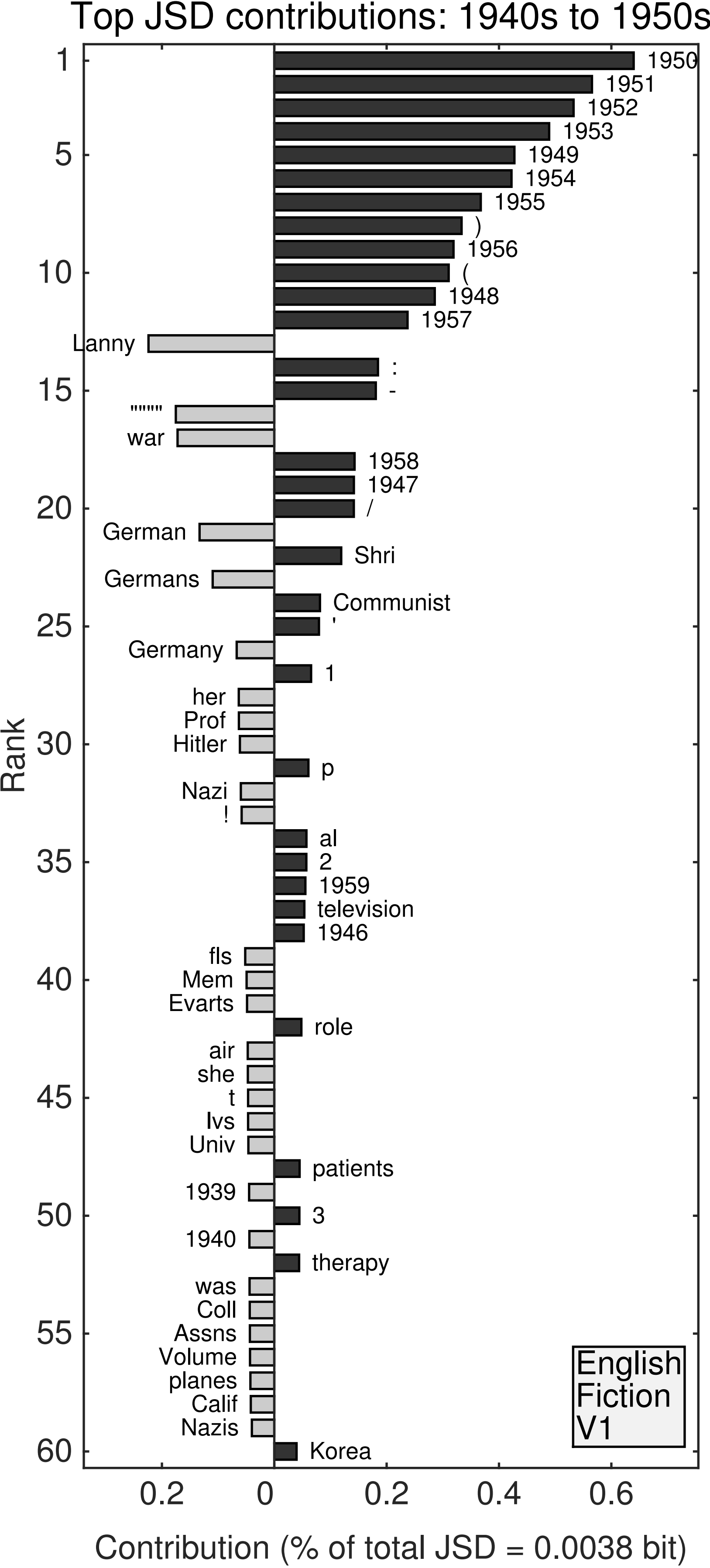

Top JSD contributions: 1940s to 1950s
Rank
Contribution (% of total JSD = 0.0038 bit)
English Fiction V1
1950
1951
1952
1953
1949
1954
1955
)
1956
(
1948
1957
Lanny
:
-
""""
war
1958
1947
/
German
Shri
Germans
Communist
'
Germany
1
her
Prof
Hitler
p
Nazi
!
al
2
1959
television
1946
fls
Mem
Evarts
role
air
she
t
lvs
Univ
patients
1939
3
1940
therapy
was
Coll
Assns
Volume
planes
Calif
Nazis
Korea

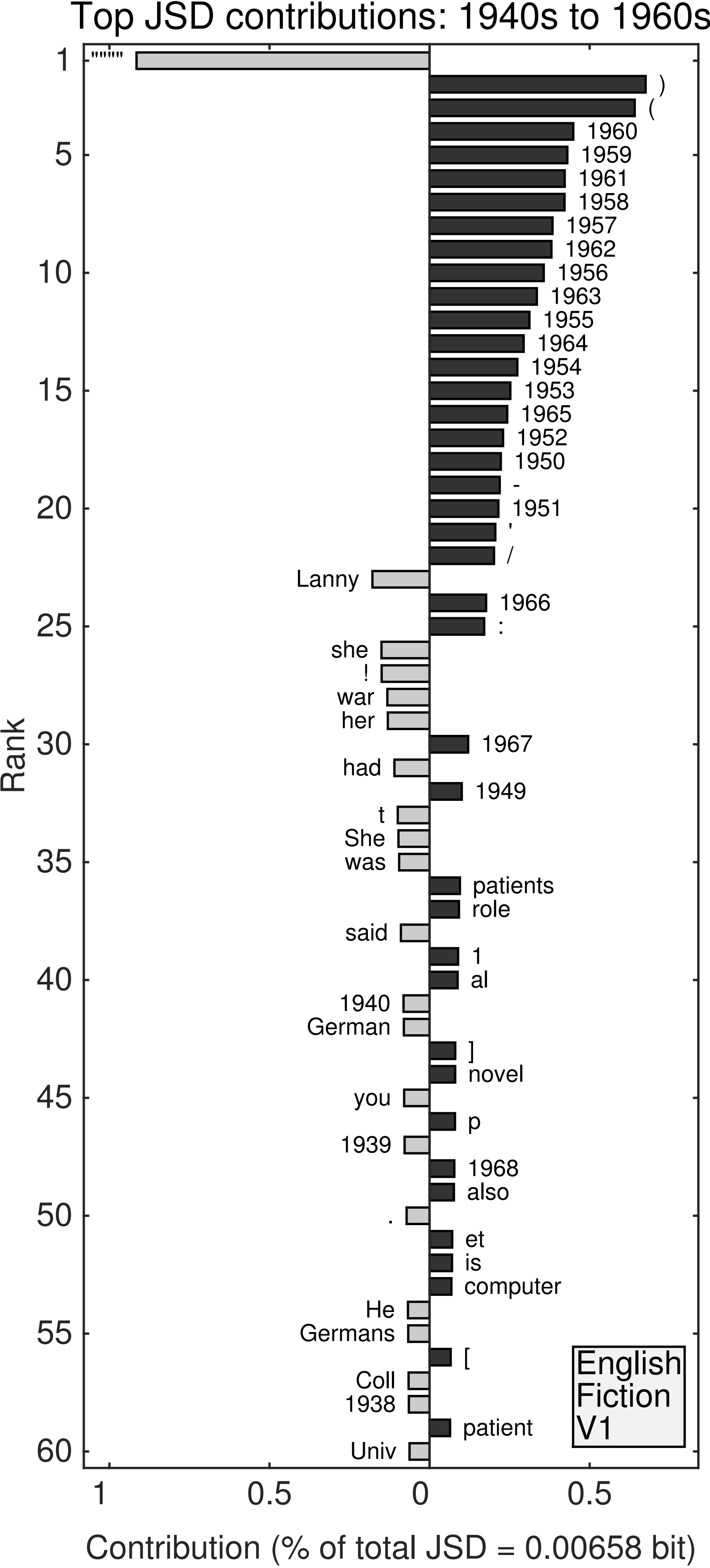

Top JSD contributions: 1940s to 1960s
Rank
Contribution (% of total JSD = 0.00658 bit)
1
0.5
0
0.5
1
5
10
15
20
25
30
35
40
45
50
55
60
""""
)
(
1960
1959
1961
1958
1957
1962
1956
1963
1955
1964
1954
1953
1965
1952
1950
-
1951
'
/
Lanny
1966
:
she
!
war
her
1967
had
1949
t
She
was
patients
role
said
1
al
1940
German
]
novel
you
p
1939
1968
also
.
et
is
computer
He
Germans
[
Coll
1938
patient
Univ
English
Fiction
V1

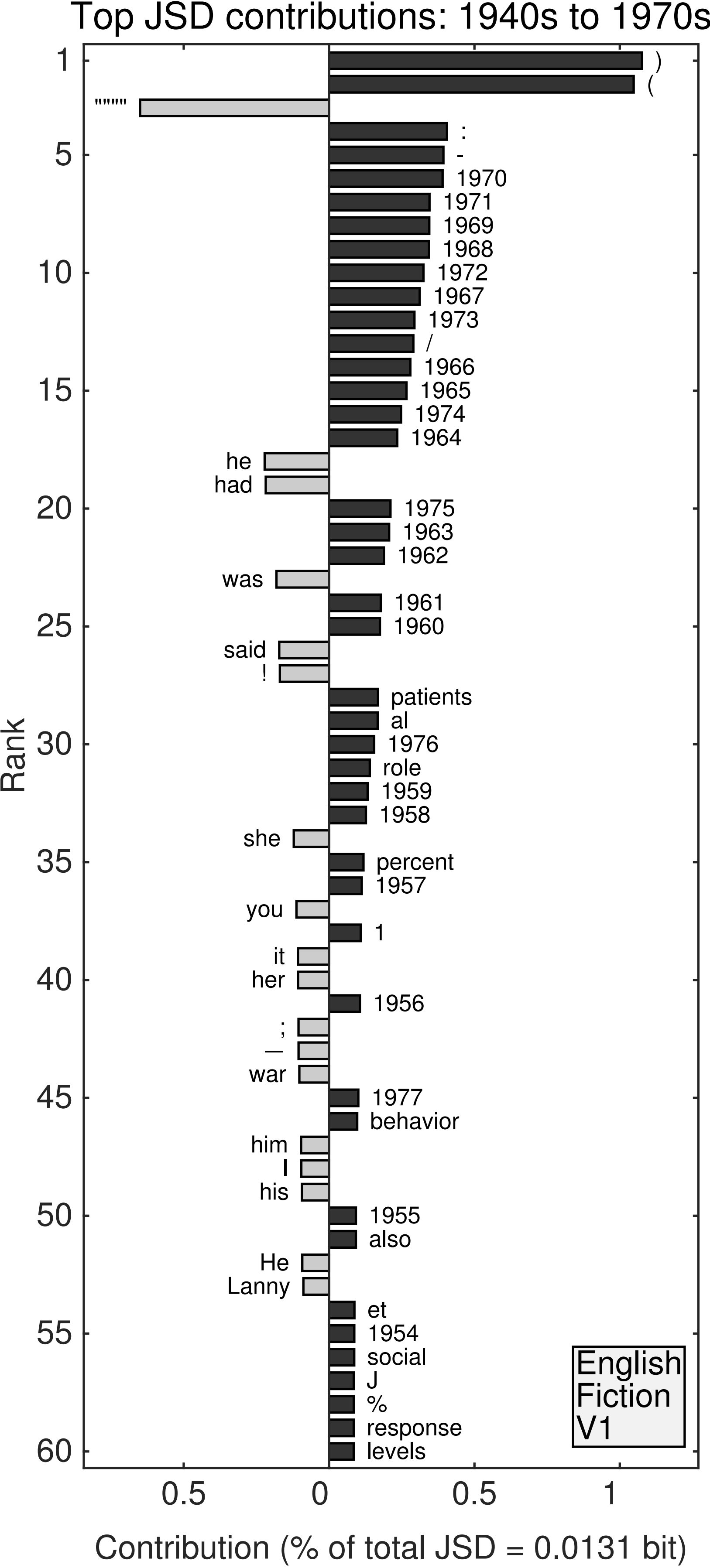

Top JSD contributions: 1940s to 1970s
Rank
Contribution (% of total JSD = 0.0131 bit)
English Fiction V1
)
(
""""
:
-
1970
1971
1969
1968
1972
1967
1973
/
1966
1965
1974
1964
he
had
1975
1963
1962
was
1961
1960
said
!
patients
al
1976
role
1959
1958
she
percent
1957
you
1
it
her
1956
;
—
war
1977
behavior
him
I
his
1955
also
He
Lanny
et
1954
social
J
%
response
levels

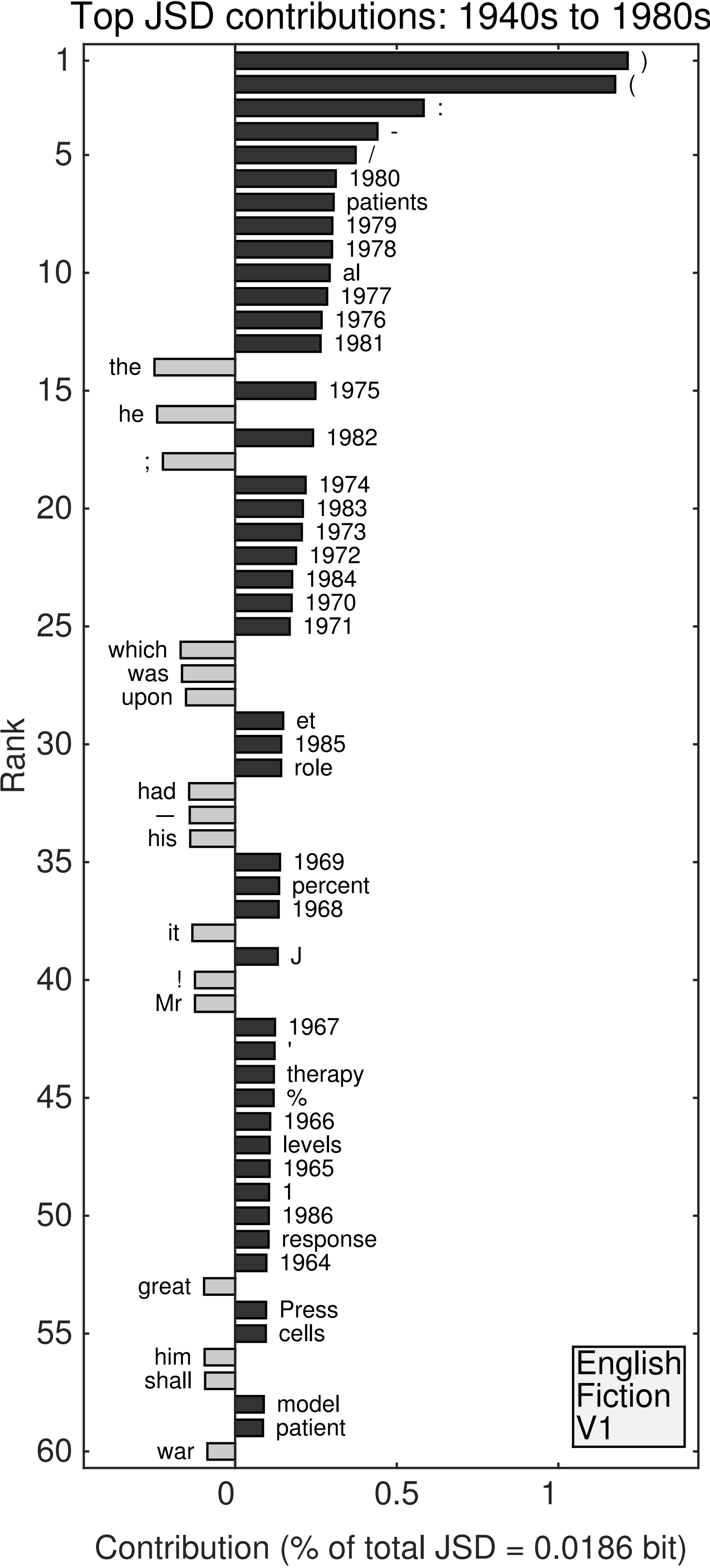

Top JSD contributions: 1940s to 1980s
Rank
Contribution (% of total JSD = 0.0186 bit)
English Fiction V1
)
(
:
-
/
1980
patients
1979
1978
al
1977
1976
1981
the
1975
he
1982
;
1974
1983
1973
1972
1984
1970
1971
which
was
upon
et
1985
role
had
—
his
1969
percent
1968
it
J
!
Mr
1967
'
therapy
%
1966
levels
1965
1
1986
response
1964
great
Press
cells
him
shall
model
patient
war

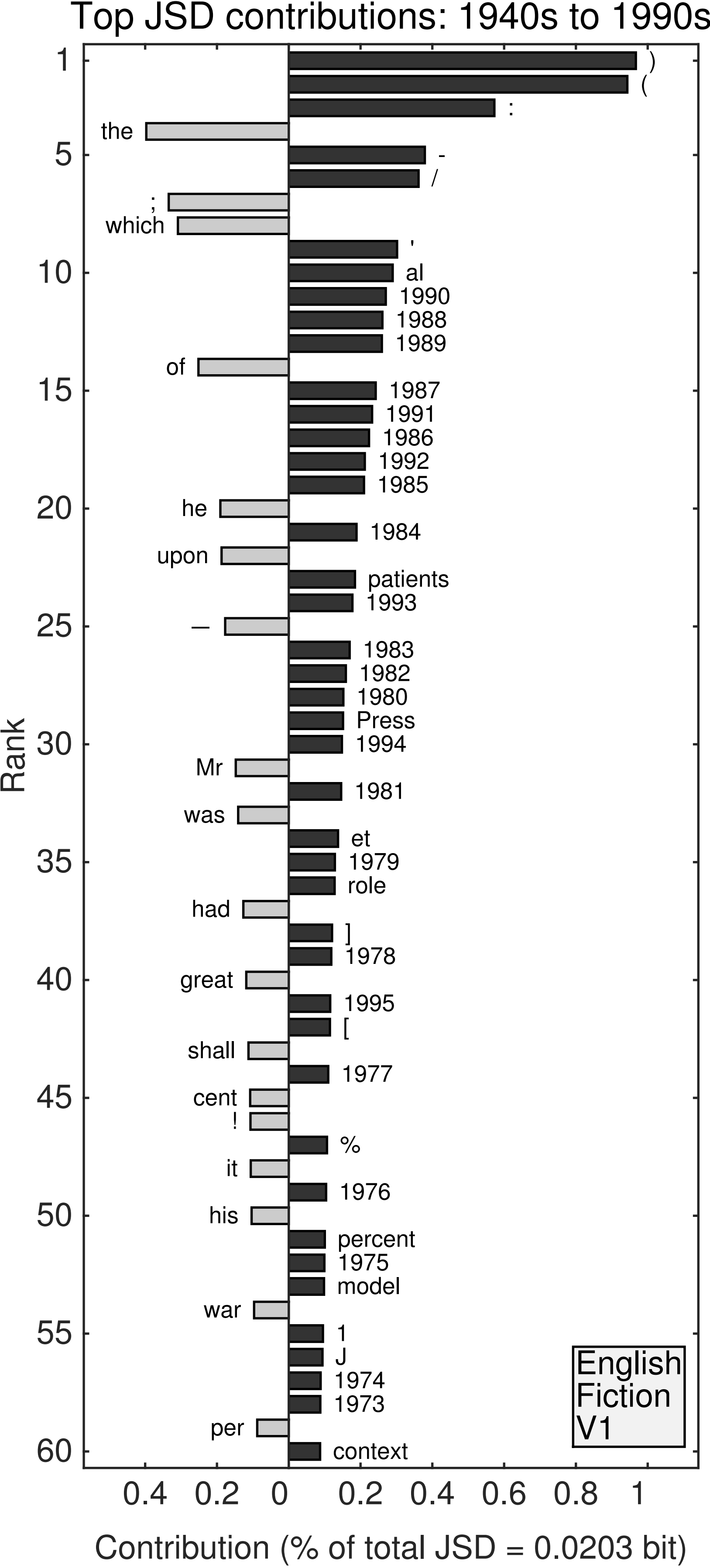

Top JSD contributions: 1940s to 1990s
Rank
Contribution (% of total JSD = 0.0203 bit)
English Fiction V1
)
(
:
the
-
/
;
which
'
al
1990
1988
1989
of
1987
1991
1986
1992
1985
he
1984
upon
patients
1993
—
1983
1982
1980
Press
1994
Mr
1981
was
et
1979
role
had
]
1978
great
1995
[
shall
1977
cent
!
%
it
1976
his
percent
1975
model
war
1
J
1974
1973
per
context

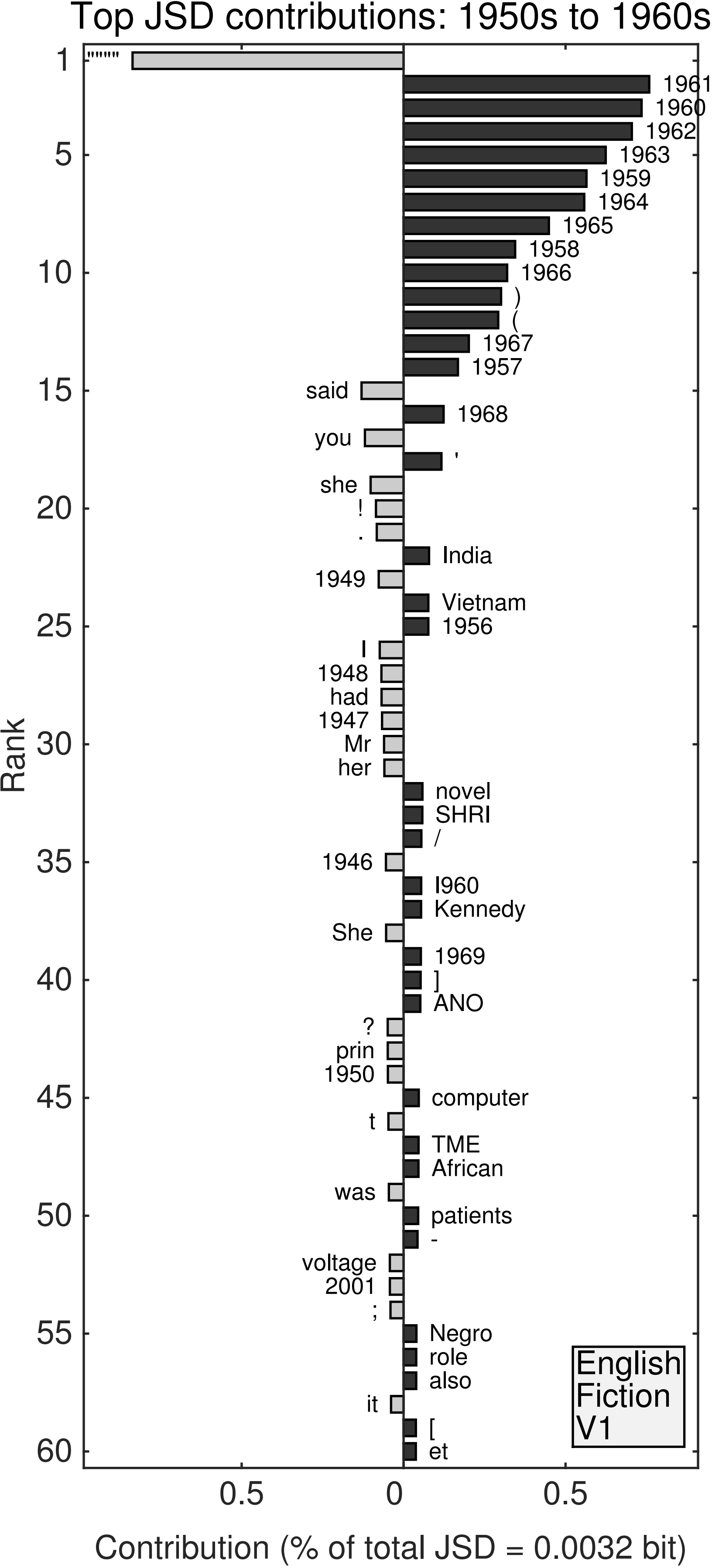
Top JSD contributions: 1950s to 1960s
Rank
Contribution (% of total JSD = 0.0032 bit)
""""
1961
1960
1962
1963
1959
1964
1965
1958
1966
)
(
1967
1957
said
1968
you
'
she
!
.
India
1949
Vietnam
1956
I
1948
had
1947
Mr
her
novel
SHRI
/
1946
I960
Kennedy
She
1969
]
ANO
?
prin
1950
computer
t
TME
African
was
patients
-
voltage
2001
;
Negro
role
also
it
[
et
English Fiction V1

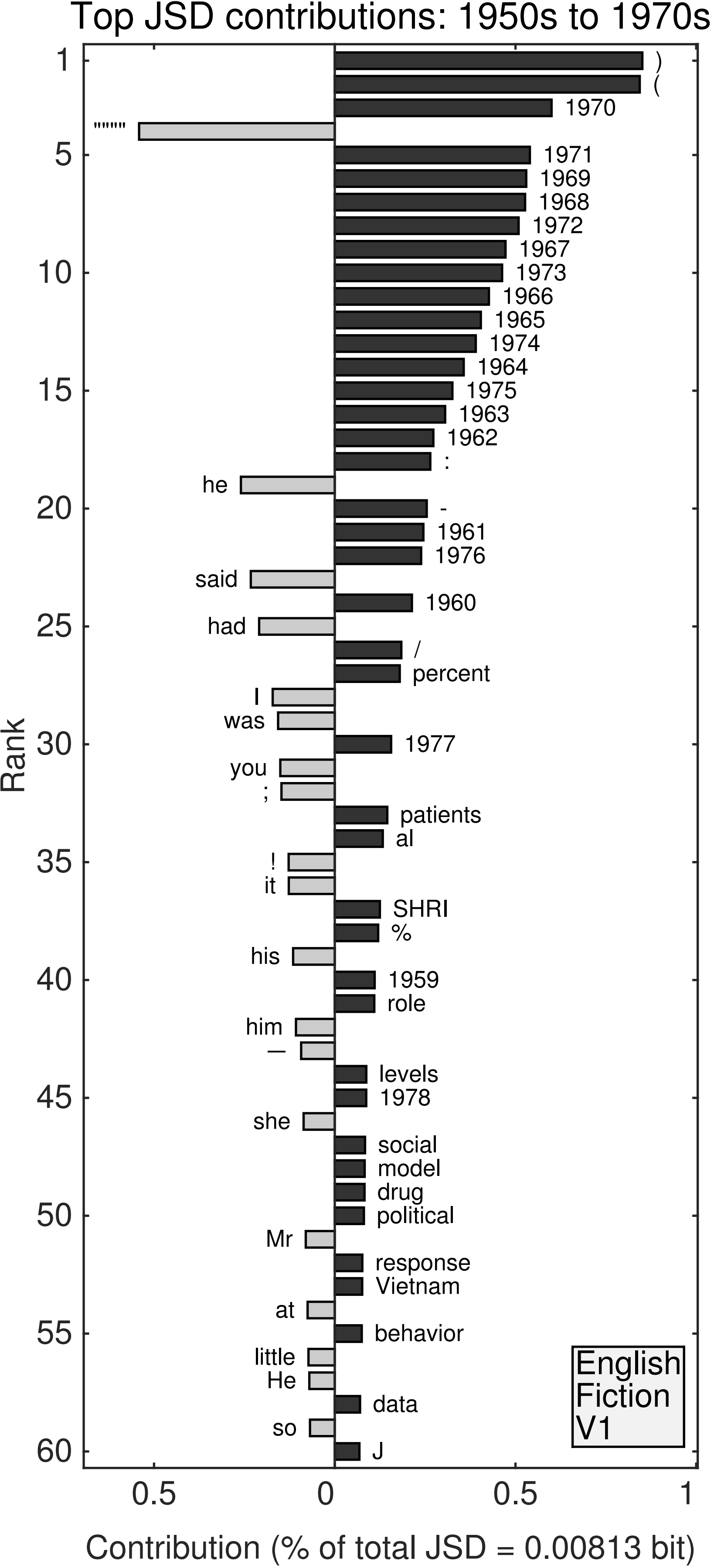
Top JSD contributions: 1950s to 1970s
Rank
Contribution (% of total JSD = 0.00813 bit)
)
(
1970
""""
1971
1969
1968
1972
1967
1973
1966
1965
1974
1964
1975
1963
1962
:
he
-
1961
1976
said
1960
had
/
percent
I
was
1977
you
;
patients
al
!
it
SHRI
%
his
1959
role
him
—
levels
1978
she
social
model
drug
political
Mr
response
Vietnam
at
behavior
little
He
data
so
J
English
Fiction
V1

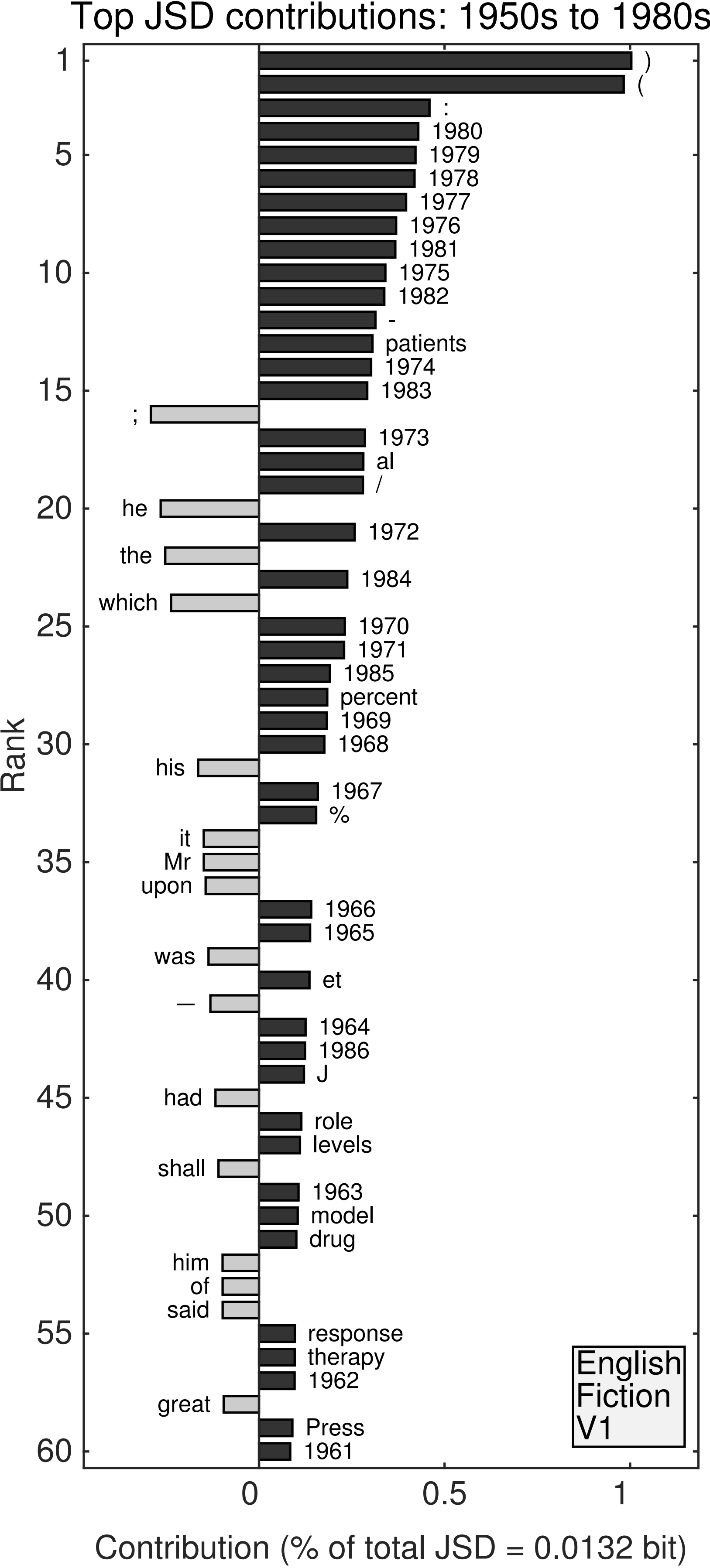

Top JSD contributions: 1950s to 1980s
Rank
Contribution (% of total JSD = 0.0132 bit)
0
0.5
1
1
5
10
15
20
25
30
35
40
45
50
55
60
)
(
:
1980
1979
1978
1977
1976
1981
1975
1982
-
patients
1974
1983
;
1973
al
/
he
1972
the
1984
which
1970
1971
1985
percent
1969
1968
his
1967
%
it
Mr
upon
1966
1965
was
et
—
1964
1986
J
had
role
levels
shall
1963
model
drug
him
of
said
response
therapy
1962
great
Press
1961
English Fiction V1

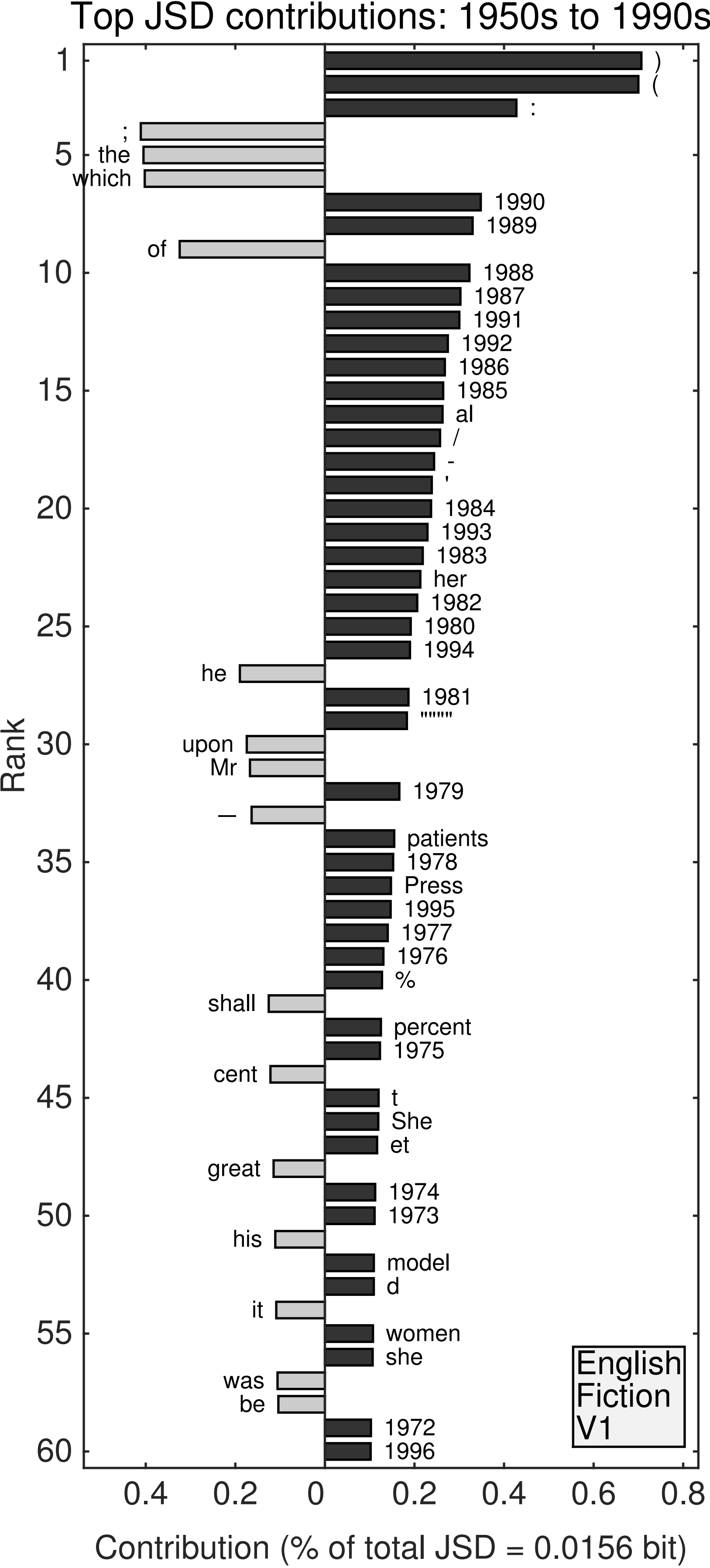
Top JSD contributions: 1950s to 1990s
Rank
1
5
10
15
20
25
30
35
40
45
50
55
60
)
(
:
;
the
which
1990
1989
of
1988
1987
1991
1992
1986
1985
al
/
-
'
1984
1993
1983
her
1982
1980
1994
he
1981
""""
upon
Mr
1979
—
patients
1978
Press
1995
1977
1976
%
shall
percent
1975
cent
t
She
et
great
1974
1973
his
model
d
it
women
she
was
be
1972
1996
English
Fiction
V1
0.4
0.2
0
0.2
0.4
0.6
0.8
Contribution (% of total JSD = 0.0156 bit)

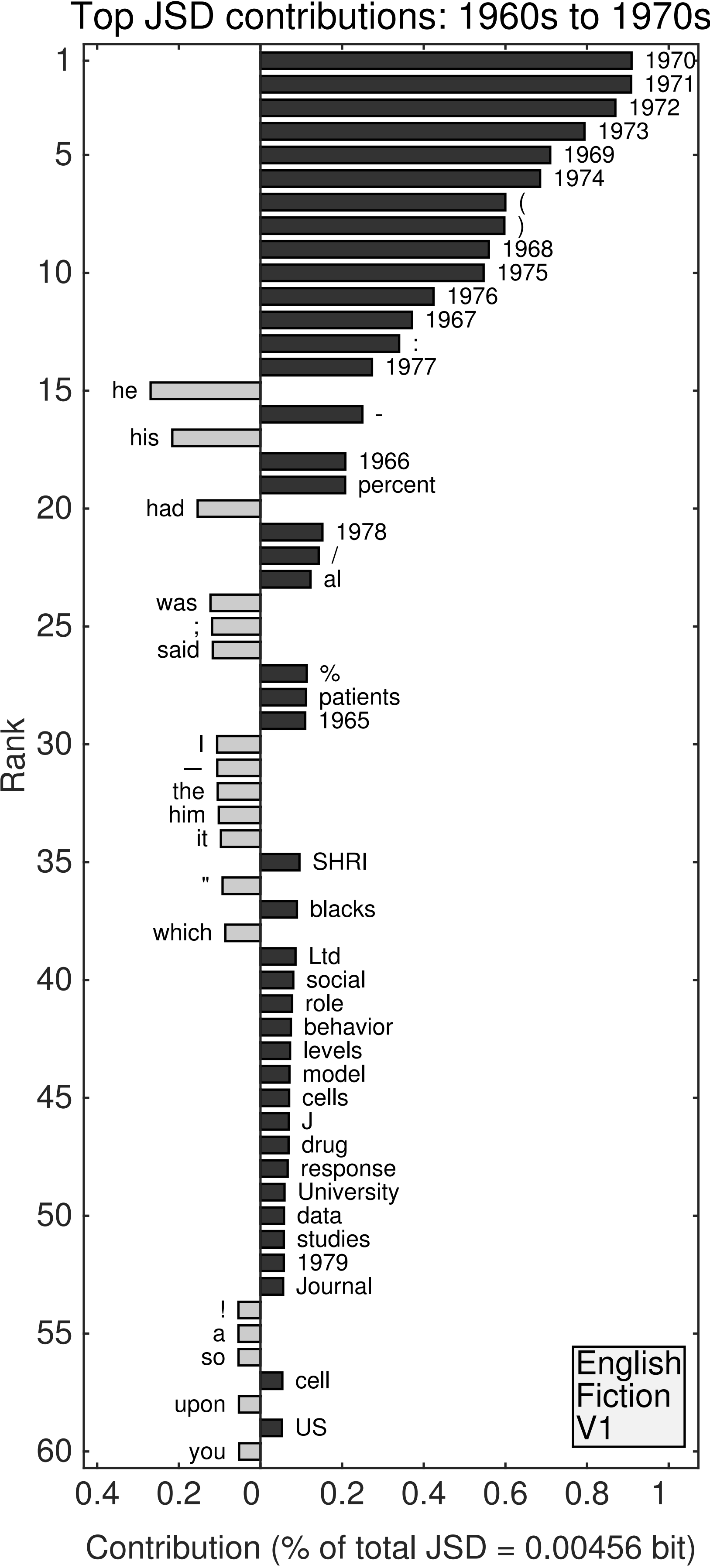
Top JSD contributions: 1960s to 1970s
1970
1971
1972
1973
1969
1974
(
)
1968
1975
1976
1967
:
1977
he
-
his
1966
percent
had
1978
/
al
was
;
said
%
patients
1965
I
—
the
him
it
SHRI
"
blacks
which
Ltd
social
role
behavior
levels
model
cells
J
drug
response
University
data
studies
1979
Journal
!
a
so
cell
upon
US
you
English Fiction V1
Rank
1
5
10
15
20
25
30
35
40
45
50
55
60
0.4
0.2
0
0.2
0.4
0.6
0.8
1
Contribution (% of total JSD = 0.00456 bit)

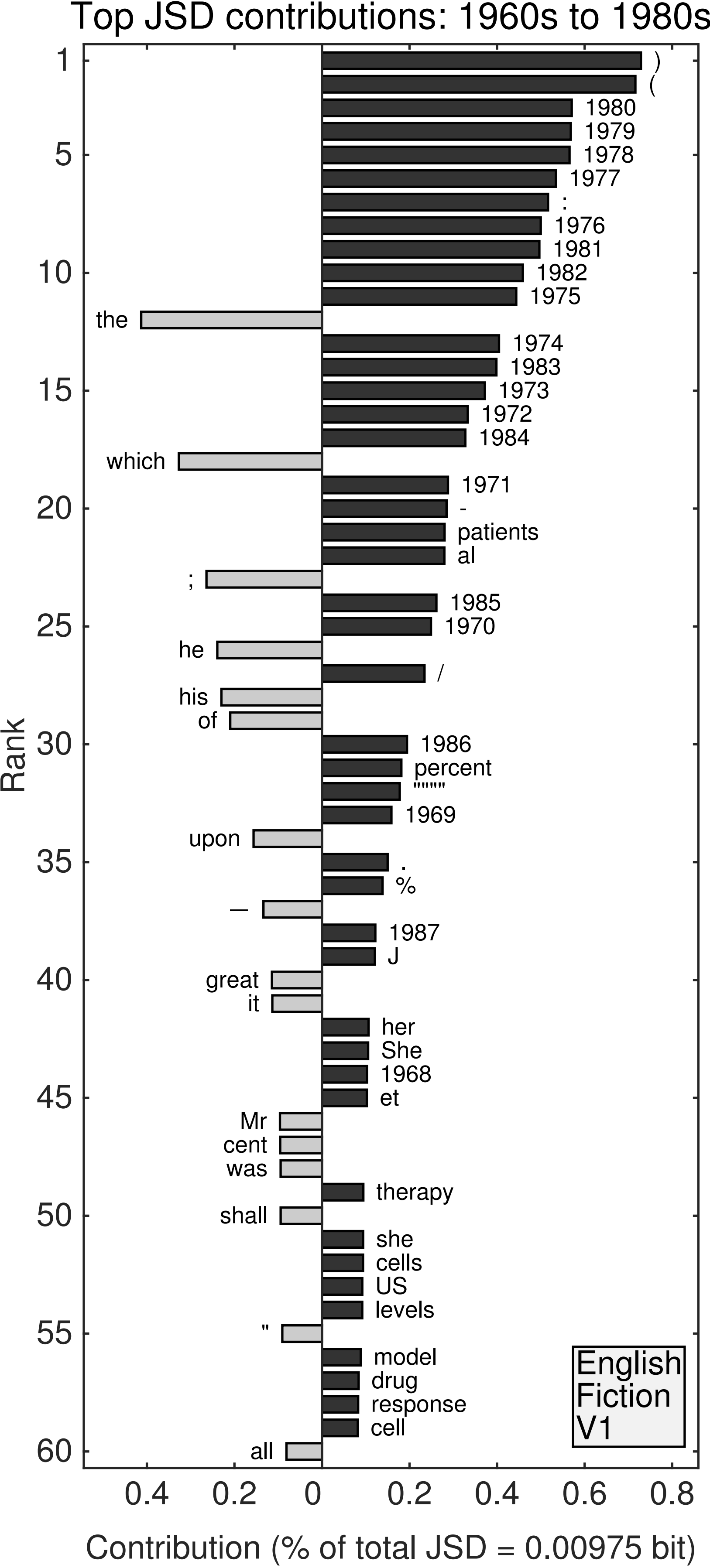

Top JSD contributions: 1960s to 1980s
Rank
Contribution (% of total JSD = 0.00975 bit)
)
(
1980
1979
1978
1977
:
1976
1981
1982
1975
the
1974
1983
1973
1972
1984
which
1971
-
patients
al
;
1985
1970
he
/
his
of
1986
percent
""""
1969
upon
.
%
—
1987
J
great
it
her
She
1968
et
Mr
cent
was
therapy
shall
she
cells
US
levels
"
model
drug
response
cell
all
English
Fiction
V1

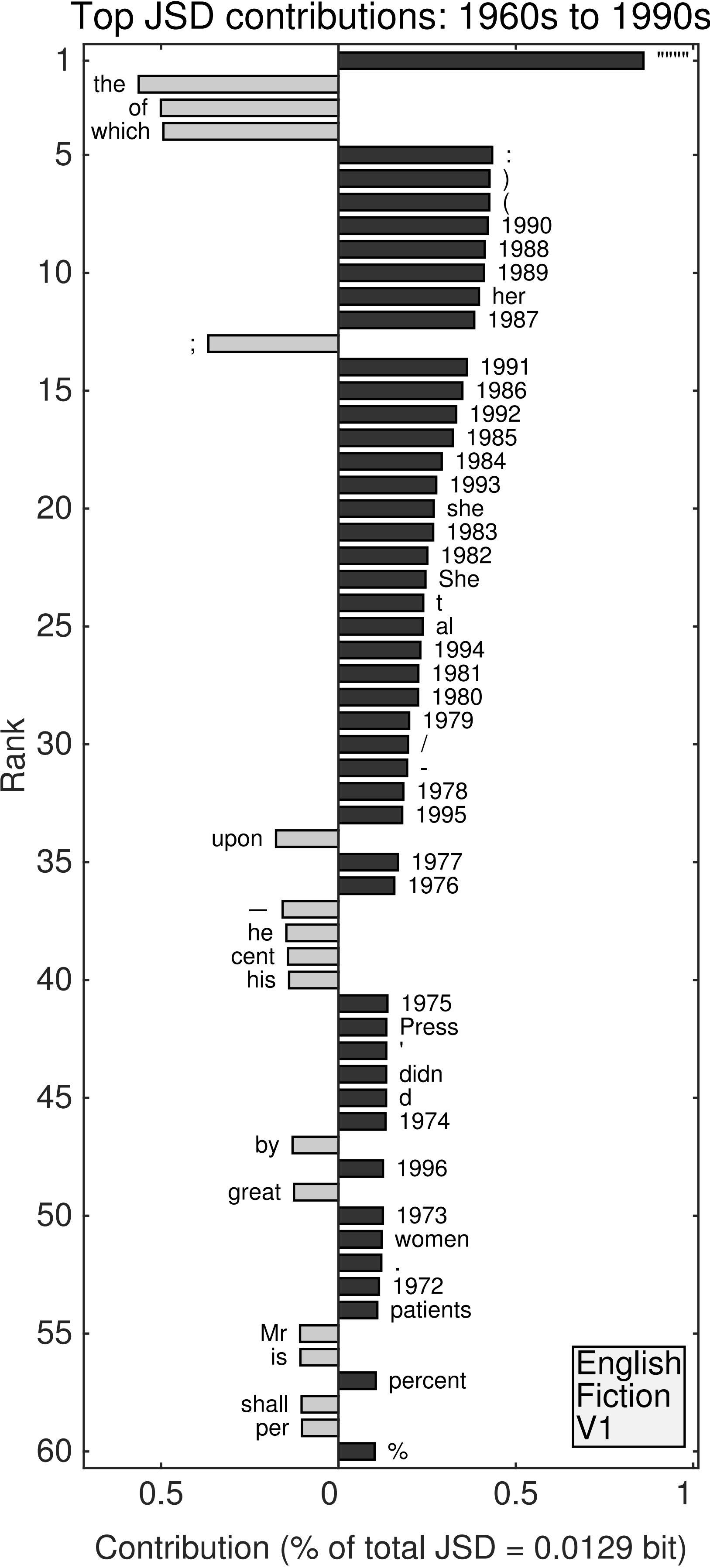
Top JSD contributions: 1960s to 1990s
Rank
Contribution (% of total JSD = 0.0129 bit)
""""
the
of
which
:
)
(
1990
1988
1989
her
1987
;
1991
1986
1992
1985
1984
1993
she
1983
1982
She
t
al
1994
1981
1980
1979
/
-
1978
1995
upon
1977
1976
—
he
cent
his
1975
Press
'
didn
d
1974
by
1996
great
1973
women
.
1972
patients
Mr
is
percent
shall
per
%
English
Fiction
V1

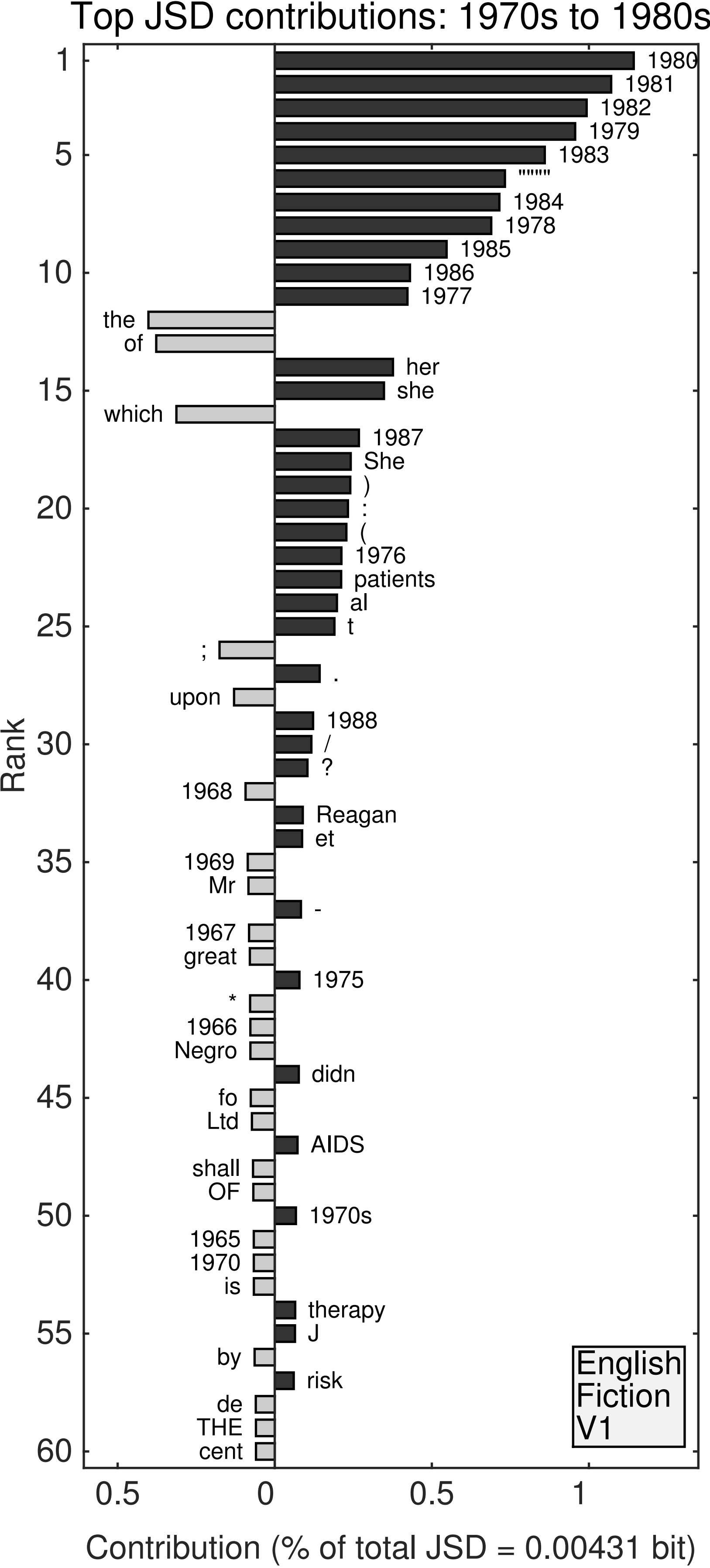

Top JSD contributions: 1970s to 1980s
Rank
1980
1981
1982
1979
1983
""""
1984
1978
1985
1986
1977
the
of
her
she
which
1987
She
)
:
(
1976
patients
al
t
;
.
upon
1988
/
?
1968
Reagan
et
1969
Mr
-
1967
great
1975
*
1966
Negro
didn
fo
Ltd
AIDS
shall
OF
1970s
1965
1970
is
therapy
J
by
risk
de
THE
cent
English
Fiction
V1
Contribution (% of total JSD = 0.00431 bit)

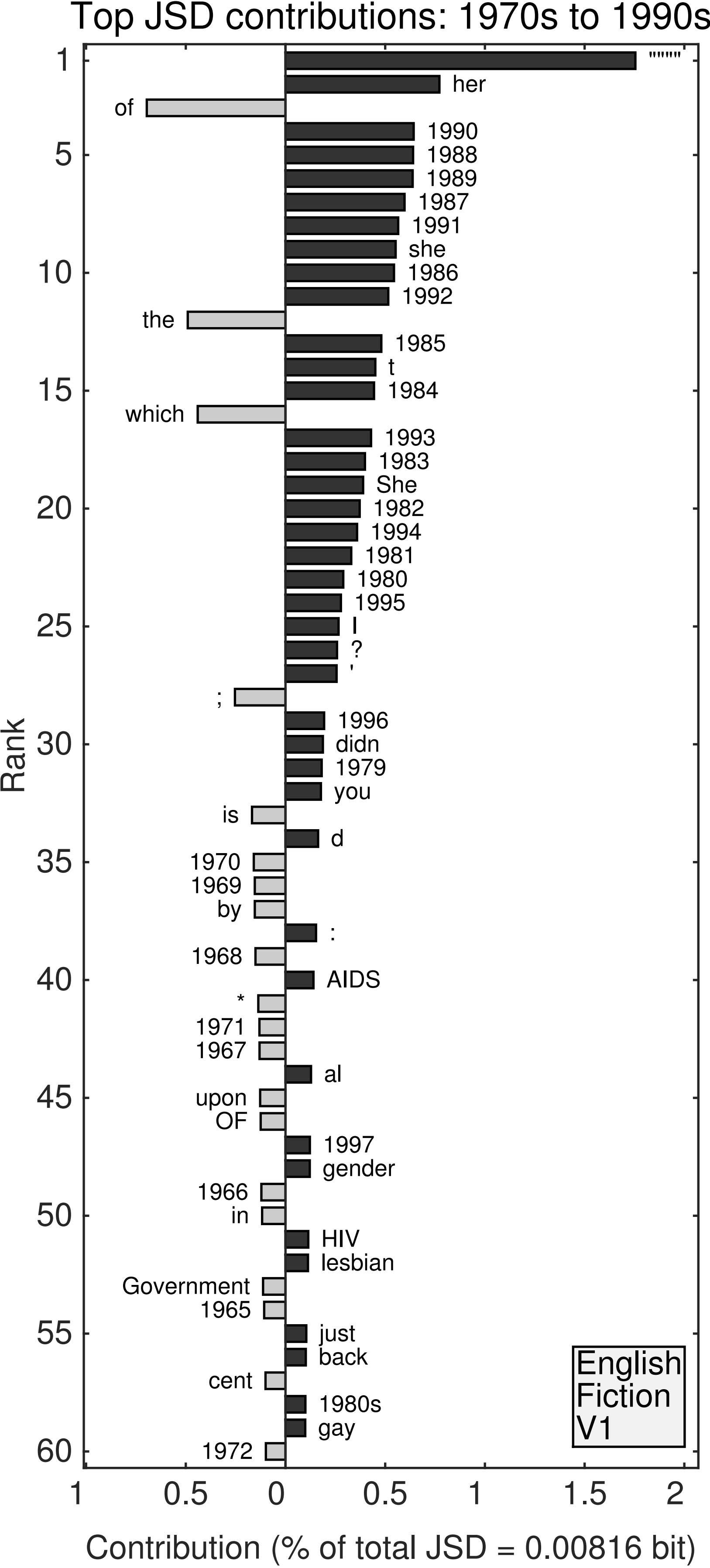

Top JSD contributions: 1970s to 1990s
Rank
Contribution (% of total JSD = 0.00816 bit)
"""""
her
of
1990
1988
1989
1987
1991
she
1986
1992
the
1985
t
1984
which
1993
1983
She
1982
1994
1981
1980
1995
I
?
'
;
1996
didn
1979
you
is
d
1970
1969
by
:
1968
AIDS
*
1971
1967
al
upon
OF
1997
gender
1966
in
HIV
lesbian
Government
1965
just
back
cent
1980s
gay
1972
English Fiction V1

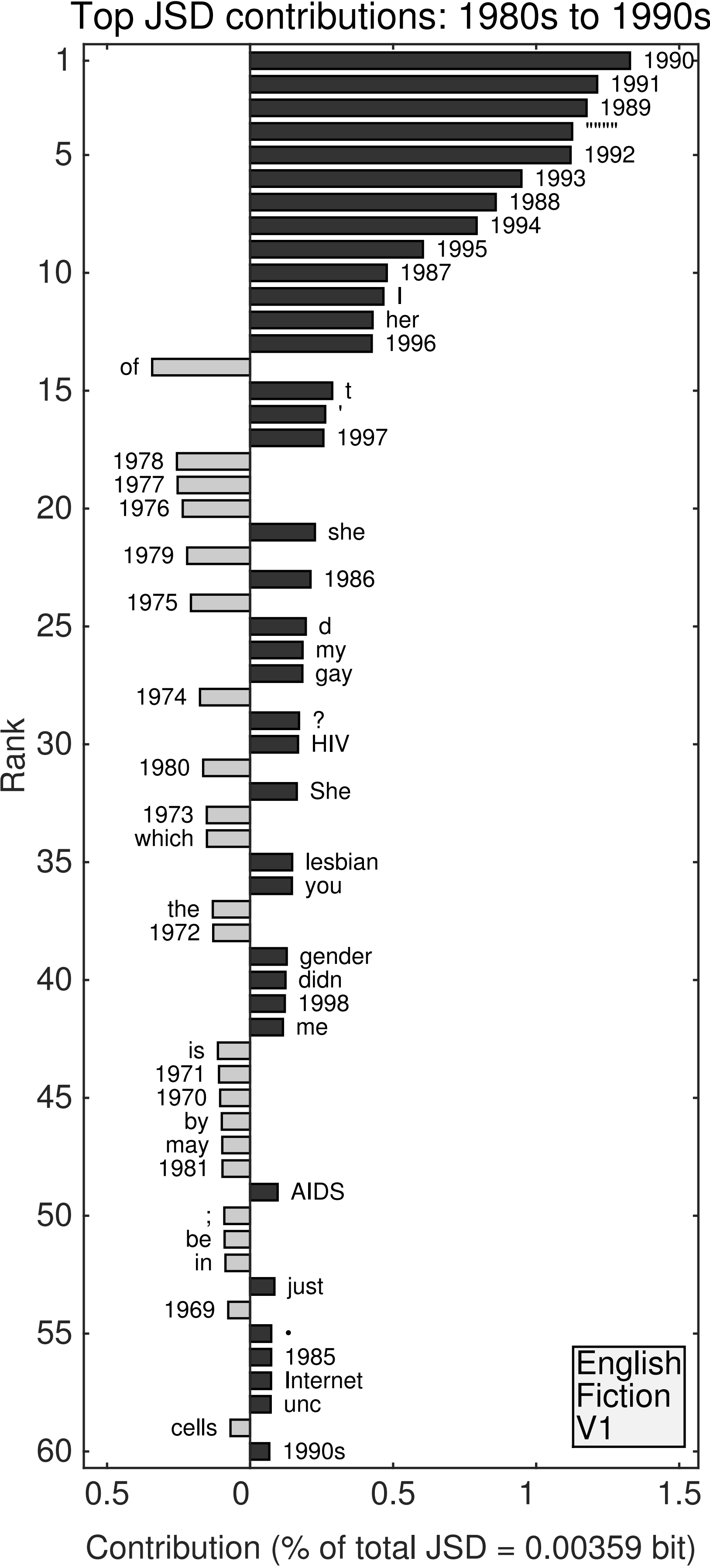

Top JSD contributions: 1980s to 1990s
Rank
Contribution (% of total JSD = 0.00359 bit)
1990
1991
1989
""""
1992
1993
1988
1994
1995
1987
I
her
1996
of
t
'
1997
1978
1977
1976
she
1979
1986
1975
d
my
gay
1974
?
HIV
1980
She
1973
which
lesbian
you
the
1972
gender
didn
1998
me
is
1971
1970
by
may
1981
AIDS
;
be
in
just
1969
•
1985
Internet
unc
cells
1990s
English Fiction V1
